# Biomarkers of Adipose Color: A Multi-Omics Analysis Unravels the Molecular Landscape of White and Yellow Fat in Kazakh Horse

**DOI:** 10.3390/biology15070563

**Published:** 2026-04-01

**Authors:** Xiaokang Chang, Xiangyun Shi, Penghui Luo, Xinkui Yao, Jun Meng, Jianwen Wang, Wanlu Ren, Linling Li, Yaqi Zeng

**Affiliations:** 1 College of Animal Science, Xinjiang Agricultural University, Urumqi 830052, China; changxk312@126.com (X.C.); yxk61@126.com (X.Y.); junm86@sina.com (J.M.); wjw1262022@126.com (J.W.); renwanlu@xjau.edu.cn (W.R.); lilinling@xjau.edu.cn (L.L.); 2 STC (XinJiang) Company Limited, Urumqi 830000, China; shixy612@126.com; 3 Animal Husbandry Station of Xinjiang Uygur Autonomous Regio, Urumqi 830004, China; xmzztxzx1@163.com; 4 Xinjiang Key Laboratory of Equine Breeding and Exercise Physiology, Urumqi 830052, China

**Keywords:** adipose tissue color, Kazakh horse, multi-omics integration, biomarker, lipid metabolism

## Abstract

Fat color is one of the most important economic traits influencing consumer preference and the market value of meat products. To explore the molecular mechanisms underlying abdominal adipose tissue color differentiation in the Kazakh horse, this study employed a multi-omics strategy, integrating targeted metabolomics (fatty acid and amino acid profiling), untargeted metabolomics, and transcriptomic analyses. Two types of adipose tissue, white adipose tissue (WAT, *n* = 8) and yellow adipose tissue (YAT, *n* = 8), were selected for systematic comparative analysis. Fatty acid composition analysis revealed no significant differences between WAT and YAT in total saturated and unsaturated fatty acid content (*p* > 0.05). However, the levels of C15:0 and C17:1 were significantly higher in YAT than in WAT (*p* < 0.05), with C21:0 showing a very significant increase (*p* < 0.01). In contrast, the level of C22:1n9 was significantly lower in YAT (*p* < 0.05). Amino acid analysis indicated that the aspartic acid content in WAT was approximately 3.3 times higher than in YAT (*p* < 0.01). Through analysis of transcriptomic and metabolomic data, a total of 378 differentially expressed genes (DEGs) and 51 differentially expressed metabolites (DEMs) were identified. Further integrative analysis of metabolomic and transcriptomic data identified *LPGAT1*, *AKT2*, and *ADH5* genes, along with metabolites such as stearate and myo-inositol, as potential key regulatory factors and biomarkers associated with fat color differentiation. This study provides a theoretical foundation for understanding the molecular mechanisms governing adipose tissue color variation in horses and their implications for meat quality.

## Simple Summary

Fat color is an important indicator influencing consumer choice and the market value of meat products. To explore the mechanisms underlying the difference in abdominal fat color (white vs. yellow) in Kazakh horses, this study conducted a systematic comparison of the two adipose tissue types by integrating metabolomic and transcriptomic analyses. The findings revealed that certain specific fatty acids (e.g., C21:0) were significantly higher in yellow adipose tissue, while the aspartic acid content in white adipose tissue was approximately 3.3 times that in yellow adipose tissue. Through multi-omics integration analysis, key potential regulatory factors such as the *LPGAT1* and *AKT2* genes, as well as metabolites including stearate and myo-inositol, were identified. These results provide a scientific basis for further understanding the molecular mechanisms of fat color formation in horses and improving meat quality.

## 1. Introduction

As a vital metabolic organ in animals, adipose tissue plays a key role in energy storage and regulation, as well as in modulating various physiological processes via adipocytokine secretion [1,2,3]. Adipocytes can be categorized into white adipose tissue (WAT), brown adipose tissue (BAT) [4,5], and beige adipose tissue, which results from the browning of WAT [6,7]. Adipose color is a crucial economic trait influencing both the sensory quality of meat products and consumer purchasing preferences [8,9]. The colorization of adipose tissue is shaped by multiple factors, including species, age, sex, and feeding regime [10].

A growing body of research in recent years has been dedicated to understanding the nutritional value and physiological roles of adipose tissue. The composition and content of fatty acids and amino acids in adipose tissue not only influence the flavor, tenderness, and juiciness of meat products [11,12] but are also closely associated with organismal health and immune function [13,14]. The advent of RNA-seq has provided a crucial means to investigate the molecular regulatory networks associated with complex traits, particularly those related to adipocyte differentiation and the metabolic composition of fatty acids and amino acids [15,16,17]. Previous studies have shown that differential expression of lipogenic genes such as *SCD*, *FABP4*, and *LPL* in Japanese Black and Holstein cattle significantly influences lipid metabolism [18,19], while the overexpression of *KLF5* enhances subcutaneous preadipocyte proliferation in goats [20]. Additionally, metabolomics has shown unique advantages in explaining the relationships between metabolites and phenotypic traits [21]. Lipidomic analyses have been employed to investigate differences in lipid composition between BAT and WAT [22,23]. Tian et al. [24] identified key metabolites associated with adipose color variation by comparing the metabolic profiles of subcutaneous WAT and YAT in crossbred cattle. However, the limitations of single-omics approaches make it difficult to comprehensively reveal the systemic regulatory mechanisms and complex interactions between biological processes and environmental factors.

Recent advances in omics technologies have positioned multi-omics integration as a promising strategy for systematically dissecting the regulatory mechanisms of quantitative traits in livestock. For example, Liu et al. [25] integrated metabolomic and transcriptomic data and found that *CRTC3* overexpression may influence the metabolism of porcine subcutaneous adipocytes, shedding light on the mechanisms of adipogenic differentiation. Other studies [26] have shown that L-carnitine can activate the AMPK signaling pathway in ovine adipose tissue, thereby promoting BAT cell differentiation and thermogenesis and inducing lipolysis and thermogenesis in WAT cells to reduce lipid accumulation.

During actual production, we observed that horses of the same breed and sex may exhibit pronounced differences in abdominal adipose tissue color at the same anatomical location, ranging from white to bright yellow. To explore the molecular mechanisms underlying this phenomenon, we selected the Kazakh horse as a model for a systematic investigation of the differences between WAT and YAT in terms of fatty acid and amino acid profiles. By integrating transcriptomic and untargeted metabolomic data, we identified key genes and metabolites associated with adipose color differentiation. This study aims to provide a theoretical basis for understanding the molecular underpinnings of adipose tissue color variation in equine animals, thereby establishing a foundation for exploring its influence on meat quality.

## 2. Materials and Methods

### 2.1. Sample Collection

This study was approved by the Animal Welfare and Ethics Committee of Xinjiang Agricultural University (approval number: 2023004). In this study, 16 adult male Kazakh horses aged between 6 and 8 years were selected, and samples from their abdominal adipose tissue were collected. Based on visual color appearance, samples were classified into two groups: WAT, Group W; *n* = 8, and YAT, Group Y; *n* = 8. All samples were collected from Emin County, Tacheng City, Xinjiang. The horses were raised, managed, fattened, and slaughtered under standardized conditions to minimize non-genetic variability. Following slaughter, abdominal adipose tissues were immediately collected from the left half of the carcass, rapidly frozen in liquid nitrogen, and stored at −80 °C until further analysis.

### 2.2. Targeted Metabolomic Sequencing and Analysis

Targeted metabolomic analyses, including quantification of fatty acids and amino acids, were conducted by Beijing Prorevo Biotech Co., Ltd. (Beijing, China).

Fatty Acid Quantification: Approximately 20 mg of adipose tissue was homogenized with 3 mL of n-hexane and heated at 50 °C while shaking for 30 min. Subsequently, 3 mL of methanol-KOH solution (0.4 mol/L) was added and shaken for derivatization for another 30 min at 50 °C. After cooling to room temperature, 1 mL of distilled water was added. After phase separation, the supernatant was diluted 1:5. A 90 μL aliquot of the diluted sample was mixed with 10 μL of internal standard (1n-nonadecanoic acid methyl ester, 125 μg/mL) and analyzed via gas chromatography-mass spectrometry (GC-MS) [27].

Amino Acid Quantification: Samples were hydrolyzed with 2 mL of 6 mol/L hydrochloric acid in a sealed, nitrogen-filled environment at 110 °C for 24 h. A 100 μL aliquot of the hydrolysate was dried at 40 °C using a nitrogen blower and redissolved in 1 mL of water. Then, 50 μL of the sample was mixed with 50 μL of protein precipitant (10% sulfosalicylic acid with NVL) and centrifuged at 13,200 rpm for 4 min. An 8 μL aliquot of the supernatant was mixed with 42 μL of labeling buffer (pH 8.5 borate solution) and briefly centrifuged. Then, 20 μL of derivatization reagent AQC was added, and the mixture was derivatized at 55 °C for 15 min. After cooling, the samples were subjected to instrumental analysis [28].

Quantitative analysis was performed using an external standard method combined with internal standard calibration. For fatty acid detection, ten concentration points were established, with concentration ranges varying by fatty acid (0.15625–240 μg/mL; see Table S1 for details). For amino acid detection, six concentration points were established, with a concentration range of 3.13–800 μmol/L (Table S1). All calibration curves were fitted using weighted least squares regression, and the correlation coefficients (R^2^) were all greater than 0.99, indicating good linearity for each analyte within the corresponding concentration ranges. The accuracy of each calibration point ranged from 85% to 115% (accuracy for fatty acid standards is detailed in File S1, and for amino acid standards in Table S1), meeting the requirements for biological sample analysis. The limits of detection (LOD) and quantification (LOQ) for each metabolite were determined based on signal-to-noise ratios (S/N) ≥3 and ≥10, respectively, and the specific values are summarized in Table S1.

To monitor the stability and data quality of the analytical process, a pooled quality control (QC) sample, prepared by mixing equal aliquots of all test samples, was inserted at intervals of every 10 samples during the fatty acid detection sequence. A total of three QC samples (qc-3, qc-4, qc-5; see File S1) were analyzed. The relative standard deviations (RSDs) for all detected fatty acids in these QC samples were less than 15%, indicating excellent stability and reproducibility of the analytical platform. In the amino acid analysis, instrument performance was monitored by evaluating the accuracy of back-calculated concentrations from standard samples (Table S1, File S2).

### 2.3. Transcriptomic Sequencing and Data Analysis

Total RNA extraction, library construction, and transcriptome sequencing were performed by Guangzhou Gene Denovo Biotechnology Co., Ltd. (Guangzhou, China). Total RNA was extracted from adipose tissue samples using TRIzol reagent (Invitrogen, Carlsbad, CA, USA) according to the manufacturer’s instructions. The purity of the RNA was assessed with a NanoPhotometer^®^ spectrophotometer (Implen, Westlake Village, CA, USA), where samples with OD260/280 ratios between 1.8 and 2.0 and OD260/230 ratios greater than 2.0 were deemed acceptable. RNA concentration was accurately determined using a Qubit^®^ 2.0 Fluorometer (Life Technologies, Carlsbad, CA, USA). RNA integrity was evaluated using an Agilent 2100 Bioanalyzer with the RNA 6000 Nano kit (Agilent Technologies, Santa Clara, CA, USA); only samples with an RNA Integrity Number (RIN) ≥ 7.0 proceeded to library construction. Furthermore, RNA quality was confirmed by 1% agarose gel electrophoresis, which displayed intact 28S and 18S ribosomal RNA bands with a 28S/18S ratio of approximately 2:1, indicating no significant degradation or DNA contamination. Paired-end sequencing was carried out using the Illumina NovaSeq X Plus platform (San Diego, CA, USA). Raw reads were quality controlled using fastp (v0.23.1) [29] to remove low-quality reads, yielding clean reads. Clean reads were aligned to the horse reference genome (Ensembl release 100) using HISAT2 (v2.2.1) [30]. Transcripts were reconstructed using StringTie (v2.2.1) [31], and gene expression levels were estimated using RSEM (v1.3.3), expressed in fragments per kilobase of transcript per million mapped reads (FPKM).

### 2.4. Validation of Differentially Expressed Genes

To validate the transcriptomic data, the selected differentially expressed genes were subjected to RT-qPCR analysis. Total RNA was reverse-transcribed into cDNA using the R123-01 Reverse Transcription Kit (Vazyme Biotech Co., Ltd., Nanjing, China). Quantitative real-time PCR was performed on a QuantStudio™ 5 Real-Time PCR System (Applied Biosystems, Foster City, CA, USA). Actin was used as the reference gene, and primers were designed using Primer Premier 5.0 (Table 1). Equal amounts of RNA from eight individuals per group were pooled to generate one representative sample for each group, and each pooled sample was assayed in triplicate technical replicates. The PCR reaction system consisted of 10 μL, containing 5 μL of 2× qPCR Mix, 0.5 μL each of forward and reverse primers, 2.0 μL of cDNA, and 2.5 μL of nuclease-free water. The amplification protocol included an initial denaturation at 95 °C for 30 s, followed by 40 cycles of denaturation at 95 °C for 10 s and annealing at 60 °C for 30 s, with fluorescence signals collected at 0.5 °C increments. Relative gene expression levels were calculated using the 2^–ΔΔCt^ method and log2-transformed prior to analysis.

### 2.5. Non-Targeted Metabolomic Sequencing and Analysis

Non-targeted metabolomic analysis was also performed by Guangzhou Gene Denovo Biotechnology Co., Ltd. (Guangzhou, China). Adipose tissue samples were ground, sonicated, incubated, centrifuged, dried, and reconstituted for metabolite extraction. Metabolomic profiling was conducted using an Agilent 1290 Infinity III UHPLC system coupled with an AB SCIEX Triple TOF 6600 mass spectrometer (Framingham, MA, USA). The chromatographic column used was an ACQUITY UPLC BEH Amide column (2.1 mm × 100 mm, 1.7 μm; Waters, Drinagh, Ireland), with the column temperature maintained at 25 °C. Mobile phase A consisted of an aqueous solution containing 25 mM ammonium acetate and 25 mM ammonium hydroxide, and mobile phase B was acetonitrile. The gradient elution program was as follows: 0–0.5 min, 95% B; 0.5–7 min, B linearly decreased from 95% to 65%; 7–8 min, B linearly decreased from 65% to 40%; 8-9 min, B maintained at 40%; 9–9.1 min, B linearly increased from 40% to 95%; 9.1–12 min, B maintained at 95%. The flow rate was 0.5 mL/min, the injection volume was 2 μL, and the samples were kept in an autosampler at 4 °C. To avoid the influence of instrument detection signal fluctuations, the samples were analyzed continuously in a random order. Raw data were converted to mzXML format using ProteoWizard (v3.0.6428) software and then imported into XCMS (online 3.7.1) software for peak picking, peak alignment, and retention time correction. To eliminate systematic errors between samples, peak areas were normalized using the total ion current normalization method. To improve data quality, the extracted metabolic features were filtered as follows: (1) Missing value filtering: features with >50% missing values in any group were removed; (2) Quality control sample stability filtering: features with a relative standard deviation (RSD) >50% in QC samples were removed. Missing values were imputed using the K-Nearest Neighbor (KNN) algorithm. Metabolite annotation was performed using public databases (e.g., MassBank, Metlin, MoNA) and an in-house MS/MS spectral library.

### 2.6. Statistical Analysis

Raw data for fatty acids and amino acids were processed using MassHunter Quantitative Software (vB.08.01, Agilent) and Analyst Software (v1.7.4, AB Sciex), respectively. The concentrations of metabolites in each sample were calculated using the internal standard method, with fatty acid and amino acid concentrations reported based on fresh tissue weight. All data were normalized using internal standard correction: C19:0 was used as the internal standard for fatty acids, and norvaline was used as the internal standard for amino acids, to correct for variations during sample preparation and instrument analysis. Analysis of variance was performed using independent sample *t*-tests in SPSS 19.0. Data are expressed as mean ± standard deviation (SD). Significance levels were set at *p* < 0.05, and *p* < 0.01 was considered highly significant.

The gene-level raw read counts obtained from RSEM were used as input, and differential expression genes (DEGs) analysis was performed using the DESeq2 software (v1.20.0) [32]. During the analysis, the default normalization method of DESeq2 was applied to normalize the read counts, and gene expression values were fitted based on a negative binomial distribution model. The design formula for differential expression analysis was design = ~ group, where “group” represents the sample groups (Group-W vs. Group-Y). Significantly differentially expressed genes were identified using the thresholds of |log_2_FoldChange| > log_2_(1.5) and *p* < 0.05. DEGs were further subjected to functional enrichment analysis in the Gene Ontology (GO, http://www.geneontology.org/) and Kyoto Encyclopedia of Genes and Genomes (KEGG, http://www.genome.jp/kegg/, accessed on 3 April 2025) databases. Gene Set Enrichment Analysis (GSEA) and the Molecular Signatures Database (MSigDB) were employed to identify significantly altered GO terms and pathways between the two groups, elucidating their associated biological processes and signaling pathways.

The Principal Component Analysis (PCA) and Orthogonal Projections to Latent Structures Discriminant Analysis (OPLS-DA) were performed on the qualitative and quantitative metabolomics data. Differentially expressed metabolites (DEMs) were identified based on Variable Importance in Projection (VIP) ≥ 1 and *p* < 0.05 (*t*-test).

Functional enrichment of DEMs was conducted using KEGG database annotations following standardized protocols [33].

## 3. Results

### 3.1. Differential Analysis of Fatty Acid and Amino Acid Composition

Targeted metabolomic analysis was conducted to explore the differences in fatty acid and amino acid compositions between the W and Y groups. The results are presented in Table 2. No significant differences were observed in the contents of total saturated fatty acids (SFA), monounsaturated fatty acids (MUFA), and polyunsaturated fatty acids (PUFA) between the two groups (*p* > 0.05). However, several odd-chain fatty acids and individual monounsaturated fatty acids exhibited significant changes (*p* < 0.05). Among the odd-chain fatty acids, compared with WAT, the levels of C15:0 and C17:1 in YAT were significantly increased by 29.4% (*p* = 0.049) and 20.5% (*p* = 0.023), respectively; C21:0 was also highly significantly increased, with an increase of 13.8% (*p* = 0.005). In contrast, among the monounsaturated fatty acids, the content of C22:1n9 in YAT was significantly decreased by 23.9% (*p* = 0.040). These results suggest that although the overall fatty acid composition is relatively stable, the metabolism of specific odd-chain fatty acids and unsaturated fatty acids differ significantly between the two adipose tissue types. Odd-chain fatty acids are typically associated with intestinal microbial metabolism or endogenous synthesis. Considering the consistent dietary conditions of the two groups of animals, these differences are more likely to reflect variations in the adipose tissue’s own metabolic processes.

Regarding amino acid composition (Table 3), a total of 13 amino acids were detected. Essential amino acids, including lysine, phenylalanine, threonine, isoleucine, leucine, and valine, were found to be more abundant in equine adipose tissue. Aspartic acid levels were significantly higher in the W group, with an approximately 3.3-fold increase compared to the Y group (*p* < 0.01), representing a 70.2% decrease in the latter.

### 3.2. Transcriptomic Analysis and Functional Enrichment Analysis of Differentially Expressed Genes

To further investigate the molecular mechanisms underlying the color differences in adipose tissue, transcriptomic sequencing was performed on W and Y groups, generating 16 cDNA libraries. A total of approximately 650 million high-quality reads were obtained, averaging 40,945,991 reads per sample. The GC content ranged from 50.69% to 53.89%, with Q20 and Q30 percentages between 97.51% and 98.20% and 93.90% and 95.45%, respectively. The mapping rate of clean reads to the horse reference genome ranged from 91.81% to 95.81% (Table 4), indicating high-quality sequencing data.

**Figure 1:**
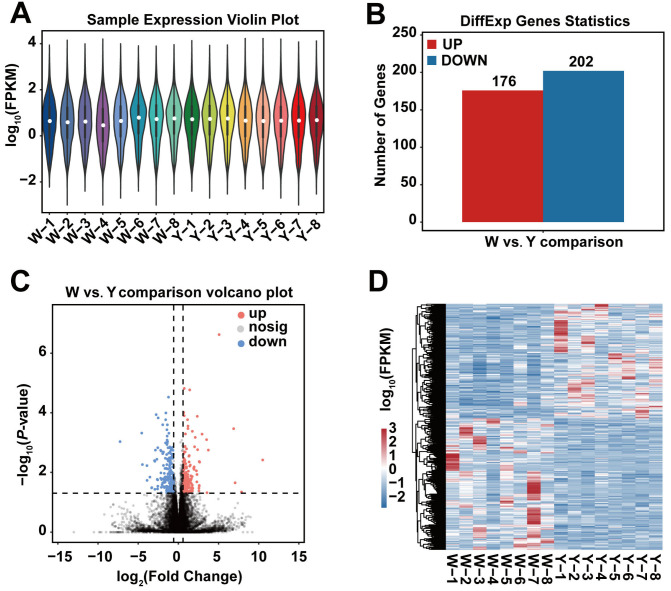
Gene expression analysis of adipose tissue of various colors and DEGs. Note: (**A**) Violin plot of gene expression; (**B**) Bar chart of the number of differentially expressed genes (DEGs); (**C**) Volcano plot of DEGs; (**D**) Clustering heatmap of DEGs.

A total of 21,567 genes were identified (Table S2), showing a consistent distribution of gene expression across samples (Figure 1A). Among these, 378 genes were identified as DEGs (*p* < 0.05), with 176 upregulated (*APOLD1*, *GADD45G*, *NDRG4*, *POPDC3*) and 202 downregulated (*IFI27*, *TMEM98*, *TUFT1*) (Figure 1B,C, Table S3). Clustering heatmap analysis revealed groups of genes with similar expression patterns, implying coordinated participation in specific biological processes or signaling pathways (Figure 1D).

**Figure 2:**
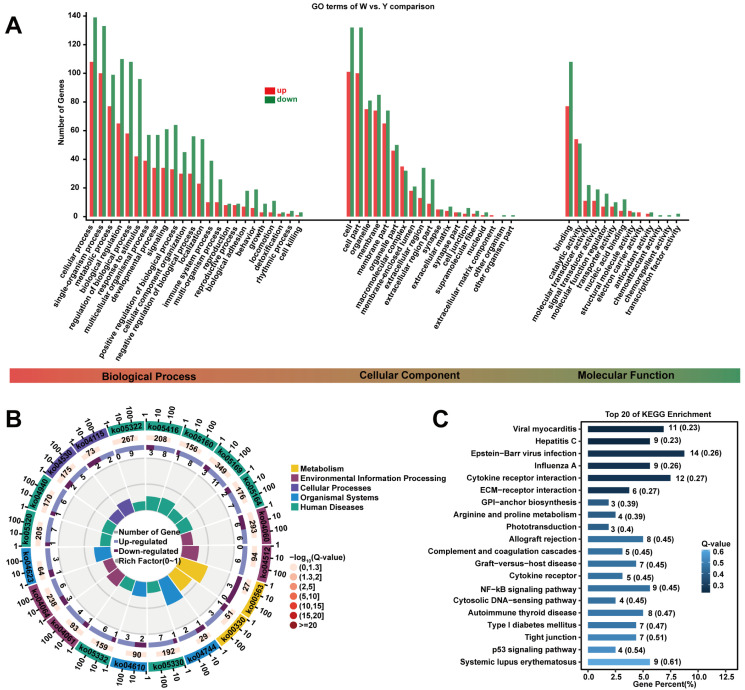
GO and KEGG enrichment analysis. Note: (**A**) GO enrichment analysis of DEGs in W and Y groups; (**B**,**C**) KEGG enrichment analysis of DEGs in W and Y groups.

GO and KEGG enrichment analyses of DEGs revealed significant biological functions. Enriched GO terms included calcium-mediated signaling, lipid metabolic process, cholesterol esterification, cell surface, membrane, external side of the plasma membrane, fatty-acyl-CoA synthase activity, and transferase activity (*p* < 0.05; Figure 2A). KEGG pathway analysis showed that DEGs were significantly involved in glycosylphosphatidylinositol-anchor biosynthesis, arginine, proline metabolism, the cytosolic DNA-sensing pathway, and the p53 signaling pathway (*p* < 0.05; Figure 2B,C, Table S4).

**Figure 3:**
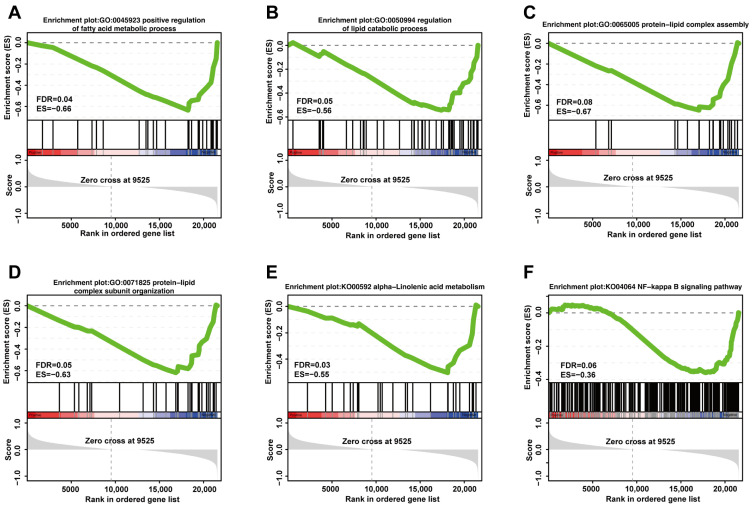
Gene set enrichment analysis (GSEA).

The GSEA further indicated a general downregulation trend in several GO terms in the Y group, particularly in pathways related to positive regulation of fatty acid metabolic process, positive regulation of lipid catabolic process, protein-lipid complex assembly, alpha-linolenic acid metabolism, and NF-kappa B signaling (Figure 3A–F, Table S5). These findings suggest that these pathways may be critically involved in adipose tissue color formation.

### 3.3. Validation of RNA-Seq Results

**Figure 4:**
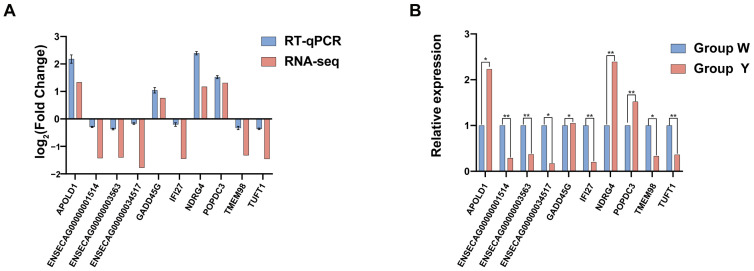
RNA-Seq verification. Note: (**A**) Log2FC obtained by RNA-seq and RT-qPCR of DEmRNAs; (**B**) relative expression of DEGs in RT-qPCR. “*” indicates significant differences (*p* < 0.05), and “**” indicates highly significant differences (*p* < 0.01).

To validate the RNA-Seq results, 10 DEGs involved in the regulation of vascular homeostasis, fat deposition, and adipocyte browning were randomly selected for RT-qPCR analysis, including seven annotated genes (*APOLD1*, *GADD45G*, *IFI27*, *NDRG4*, *POPDC3*, *TMEM98*, and *TUFT1*) and three unannotated genes (*ENSECAG00000001514*, *ENSECAG00000003563*, and *ENSECAG00000034517*). Meanwhile, the Ct values of the reference gene Actin exhibited high stability, ranging from 17.14 to 17.37 in the GroupW samples and from 17.03 to 17.17 in the GroupY samples, demonstrating its suitability as an internal control. The expression trends obtained by RT-qPCR were consistent with RNA-Seq results (Figure 4A), indicating high data reliability. Further analysis revealed that the expression levels of *NDRG4* and *POPDC3* were significantly upregulated in the YAT group (*p* < 0.01), whereas *GADD45G*, *IFI27*, *TMEM98*, *TUFT1*, *ENSECAG00000001514*, and *ENSECAG00000003563* were significantly downregulated (*p* < 0.01) (Figure 4B). These results support the involvement of these genes in the regulation of adipose tissue coloration.

### 3.4. Untargeted Metabolomic Analysis and Functional Enrichment of DEMs

**Figure 5:**
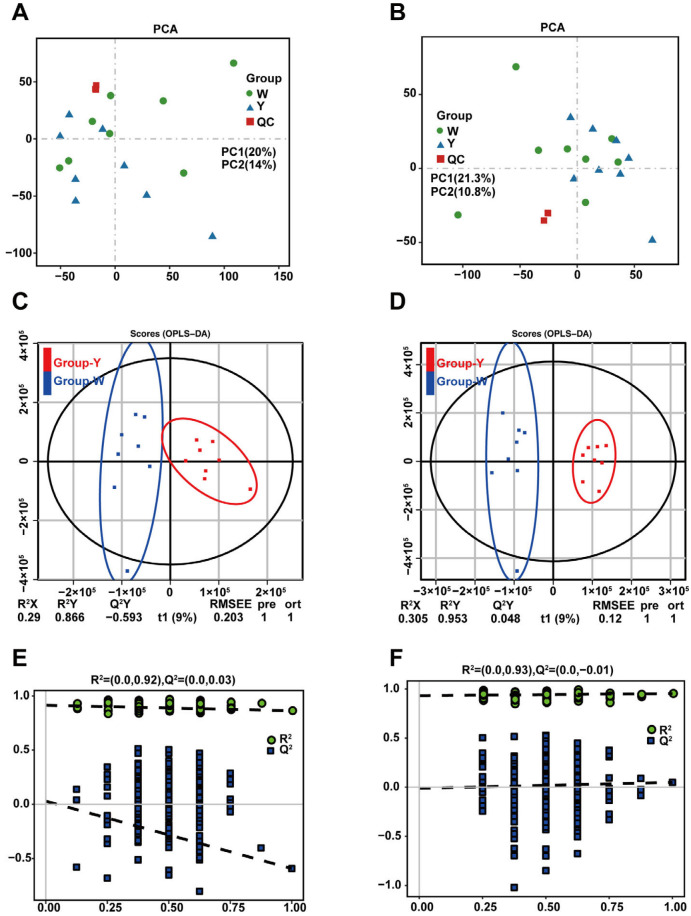
Metabolomic quality control of differently colored adipose tissues. Note: (**A**,**C**,**E**) PCA plots, OPLS-DA plots, and OPLS-DA replacement test plots at POS; (**B**,**D**,**F**) Corresponding plots under NEG mod.

Untargeted metabolomic profiling of 16 samples identified 19,605 metabolites, including 12,009 under POS and 7596 under NEG (Table S6). The PCA showed a distinct separation between the W and Y groups, with high repeatability (Figure 5A,B). OPLS-DA confirmed the clear separation between the two groups. Cross-validation and permutation testing validated the model’s stability and reliability (Figure 5C,F), justifying its use for further differential metabolite screening.

**Figure 6:**
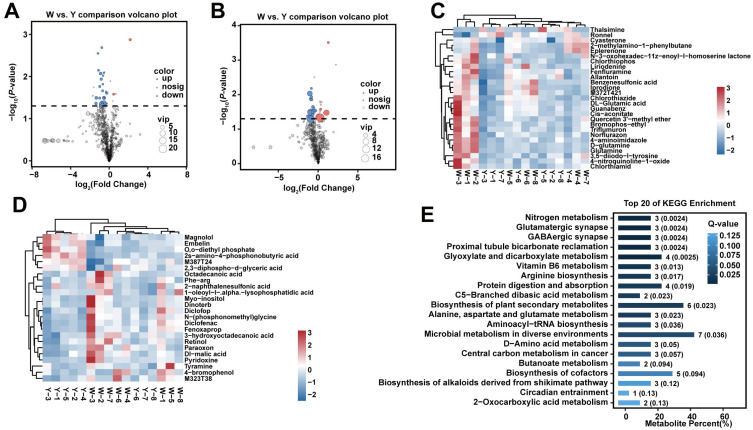
DEM identification and enrichment analyses of different colors of fat. Note: (**A**) Volcano plots of DEMs under POS; (**B**) Volcano plots of DEMs under NEG; (**C**) Heat maps of DEMs under POS; (**D**) Heat maps of DEMs under NEG; (**E**) KEGG enrichment bar chart.

A total of 51 significant DEMs (VIP ≥ 1 and *p* < 0.05) were identified (Table S7). Compared with the W group, two metabolites were significantly upregulated and 25 downregulated in the Y group under POS, while six were upregulated and 18 downregulated under NEG (Figure 6A–D). Notable upregulated metabolites in the Y group included 2,3-diphospho-D-glyceric acid, while significantly downregulated compounds included stearate, glutamine, DL-glutamic acid, pyridoxine, and retinol (Table 5). According to Human Metabolome Database (HMDB) annotations, these DEMs belonged to six major categories: lipids and lipid-like molecules, benzenoids, organic acids and derivatives, organoheterocyclic compounds, phenylpropanoids and polyketides, and organic oxygen compounds (Table S7).

KEGG pathway enrichment analysis indicated that these metabolites were mainly involved in pathways such as vitamin B6 metabolism, arginine biosynthesis, alanine, aspartate, and glutamate metabolism, C5-branched dibasic acid metabolism, and aminoacyl-tRNA biosynthesis (*p* < 0.05; Figure 6E, Table S8).

### 3.5. Integrated Analysis of DEGs and DEMs

**Figure 7:**
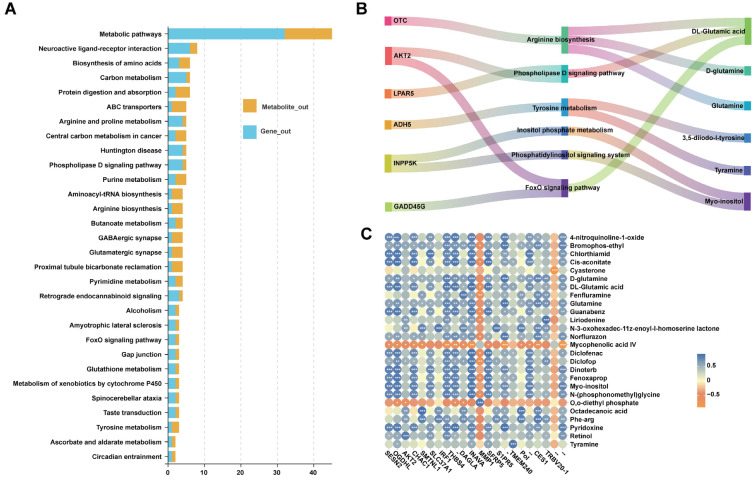
Co-enriched KEGG pathways and Pearson correlation results of DEGs and DEMs. Note: (**A**) Top 30 KEGG co-enrichment pathways between W group and Y group; (**B**) Correlation networks linking DEGs and DEMs; (**C**) Correlation heatmap. “*” indicates significant differences (*p* < 0.05), while “**” and “***” both indicate highly significant differences (*p* < 0.01).

While transcriptomics reveals gene expression changes in tissues, metabolomics provides insight into the organism’s physiological and metabolic states. Integrating both approaches allows for a more comprehensive understanding of the molecular basis underlying phenotypic differences. Co-enrichment analysis of DEGs and DEMs in KEGG pathways, with the top 30 pathways with enrichment significance visualized. The findings revealed six shared pathways: arginine biosynthesis, phospholipase D signaling, tyrosine metabolism, inositol phosphate metabolism, phosphatidylinositol signaling system, and the FoxO signaling pathway (Figure 7A, Table S9).

Eight DEGs (two unannotated) and six DEMs were involved in these pathways. These included genes such as *OTC*, *AKT2*, *LPAR5*, *ADH5*, *INPP5K*, and *GADD45G*, and metabolites such as DL-glutamic acid, D-glutamine, glutamine, 3,5-diiodo-L-tyrosine (3,5-T2), tyramine, and myo-inositol (Figure 7B), which are involved in amino acid metabolism, hormonal signaling, and cellular stress regulation. Notably, the arginine biosynthesis pathway and its related metabolites (DL-glutamic acid, D-glutamine, and glutamine) were consistently downregulated in YAT. Similarly, the inositol phosphate metabolism and phosphatidylinositol signaling pathways, along with the key gene INPP5K and its metabolite myo-inositol, were also significantly downregulated. These coordinated changes across multiple pathways construct a multi-layered regulatory network that contributes to the divergence in adipose tissue color. Pearson correlation coefficient analysis of the expression and abundance between DEGs and DEMs (|r| > 0.5, *p* < 0.05) identified significant gene-metabolite pairs. The top 50 genes and metabolites are visualized in Figure 7C and detailed in Table S10.

## 4. Discussion

### 4.1. Fatty Acid and Amino Acid Composition Differences in Abdominal Adipose Tissue of Various Colors

Adipose tissue functions not only as an energy reserve but also as a source of essential fatty acids and fat-soluble vitamins. Extensive studies have established the importance of fatty acids and amino acids in adipose tissue structure, function, and metabolic homeostasis [34,35]. However, studies investigating compositional differences in fatty acids and amino acids in adipose tissue of varying colors, especially in equids, remain limited.

In this study, we observed that the fatty acid profile of the W group primarily included C16:0, C18:1n9C, C18:2n6C, C18:0, and C16:1, whereas the Y group consisted mainly of C16:0, C18:1n9C, C18:2n6C, C18:3n3, and C18:0. The proportion of unsaturated fatty acids was similar in both groups, approximately 54.5% in W and 53.9% in Y. Among all the detected fatty acids, only C15:0, C17:1, C21:0, and C22:1n9 exhibited statistically significant differences between WAT and YAT, while total SFA, MUFA, and PUFA showed no significant changes between the groups. This result suggests that the two adipose tissue types may be relatively conserved in maintaining basic structural integrity and energy storage functions. Although odd-chain fatty acids such as C15:0, C17:1, and C21:0 were present at low abundances, they all showed a consistent upward trend in YAT. This selective metabolic dysregulation, rather than a global remodeling of the overall lipid pool, may more precisely reflect the metabolic mechanisms underlying fat color differentiation, positioning them as important potential targets for future research. However, current research on C15:0, C17:1, and C21:0 has predominantly focused on the medical field [36,37,38], with no reports yet available on their roles in animal adipose tissue. Therefore, future studies are needed to investigate and validate their specific functions in equine adipose tissue color formation.

Notably, aspartic acid, a metabolite involved in lipid metabolism regulation through AMPK pathway inhibition [39], was significantly elevated in the W group, suggesting its role in suppressing thermogenesis, enhancing WAT energy storage, and contributing to adipose coloration. However, the direct causal relationship between aspartate and equine fat color remains to be elucidated, as the current correlative evidence is primarily derived from rodent models. Therefore, targeted functional studies, such as aspartate supplementation experiments in equine adipocytes, are necessary in the future in order to verify its specific role in determining fat color.

### 4.2. Transcriptional Regulatory Mechanisms of Abdominal Adipose Color Differentiation

Fat deposition is intricately regulated by a network of signaling pathways and transcriptional regulators [40]. In our study, DEGs were significantly enriched in genes associated with lipid metabolic processes, positive regulation of lipid catabolism, and fatty acid metabolism. Key genes identified included *APOA4*, *AKT2*, *SESN2*, and *LPGAT1*. Among these, *APOA4* is involved in lipid transport and metabolism [41]; *AKT2* is central to the insulin signaling pathway and lipid metabolism [42]; and *SESN2* regulates triglyceride (TG) synthesis through lipid synthesis-related pathways [43]. Knockdown of *LPGAT1*, a gene essential for lipid biosynthesis, substantially alters the palmitate/stearate ratio in phosphatidylethanolamine (PE) [44], while excess palmitate has been linked to insulin resistance and increased WAT accumulation [45,46,47,48]. In this context, higher expression of *LPGAT1* in the W group may underlie abdominal adipose color differences in horses. Furthermore, *SOAT2* was significantly enriched in GO terms related to protein-lipid complex assembly and protein-lipid complex subunit organization. Knockdown of this gene, which enzymatically catalyzes the conversion of free cholesterol and fatty acids to cholesteryl esters [49], enhances lipoprotein lipase activity and lipid oxidation [50], with lipoprotein lipase being a key player in TG deposition in adipose tissue [51,52]. These findings suggest that *SOAT2* may also contribute to adipose color regulation via its influence on lipid droplet formation and metabolism. In summary, the upregulation of *LPGAT1* in white adipose tissue may promote a whiter phenotype through palmitate-mediated insulin resistance, while increased *SOAT2* expression in white adipose tissue also contributes to a whiter appearance by enhancing cholesterol esterification and altering lipid droplet dynamics.

### 4.3. Metabolite Characterization in Adipose Color Pattern Differentiation

Non-targeted metabolomic analysis identified significant upregulation of several metabolites in the W group that are closely linked to adipose coloration. Stearate, significantly higher in the W group, is a precursor of oleate through stearoyl-CoA desaturase and plays a role in TG and complex lipid biosynthesis [53]. As TG is the main storage form of lipids in adipose tissue, the stearate level may be a crucial determinant of fat deposition and color [54,55]. Lipids in mature white adipocytes are predominantly stored as TG in lipid droplets, and excessive lipid accumulation increases droplet size [56,57]. Due to the high energy cost of stearate oxidation, it tends to remain in storage form, significantly contributing to distinctive fat color [53]. Additionally, pyridoxine and retinol, both lipid metabolism regulators, were significantly downregulated in the Y group [58,59,60]. Metabolic pathway analysis further showed DEG enrichment in amino acid metabolism pathways, including arginine biosynthesis and glutamate metabolism. Amino acids are critical regulators of lipid metabolism [61]: arginine promotes adipocyte lipid droplet formation in bovine adipocytes [62], alanine exacerbates lipid metabolic disorders in overweight mice [63], and glutamate enhances porcine lean meat percentage and intramuscular fat [64]. Notably, L-arginine supplementation induces rat abdominal WAT browning and promotes lipid oxidation [65]. Thus, amino acid and fatty acid metabolic profiles play a central role in equine fat color formation.

### 4.4. Gene-Metabolite Synergistic Regulation of Adipose Color Differentiation

To systematically analyze the molecular mechanisms underlying adipose color variation, we integrated DEG and DEM pathway annotations, identifying 6 key metabolic pathways potentially involved in regulating equine abdominal fat coloration. In the arginine biosynthesis pathway, DL-glutamic acid, D-glutamine, and glutamine were significantly enriched. Glutamine not only promotes preadipocyte differentiation but also regulates lipolysis in mature adipocytes via the FoxO1 transcription factor [66]. Its levels are also associated with WAT inflammation and metabolic disorders [67]. Notably, *AKT2* was enriched in the phospholipase D signaling pathway, underscoring its central role in insulin signaling, lipid synthesis, adipose tissue growth, and lipid accumulation [68,69,70]. Research has confirmed that the absence of *AKT2* will lead to redistribution of adipose, bringing a significant impact on the nutrient status of BAT [71]. Moreover, the co-enrichment of 3,5-T2, tyramine, and *ADH5* in tyrosine metabolism suggests a role in adipose color formation. 3,5-T2 exhibits anti-adipogenic properties by activating fundamental metabolisms, enhancing fatty acid oxidation and angiogenesis [72], while *ADH5* maintains thermogenesis in BAT through nitroso-redox balance [73]. In the inositol phosphate metabolism and phosphatidylinositol signaling pathways, myo-inositol and *INPP5K* were co-enriched. While INPP5K’s direct role in adipose coloration is unclear, myo-inositol has been demonstrated to upregulate the expression of uncoupling protein 1, a key marker of BAT, in differentiated human adipocytes. This regulation is accompanied by an increase in mitochondrial biogenesis and oxygen consumption rate, thereby promoting the browning of adipocytes [74]. Additionally, *GADD45G*, a gene involved in the FoxO signaling pathway, has been linked to intramuscular fat deposition in pigs [75]. However, its specific role in the formation of adipose tissue color phenotypes remains to be fully explored.

Although this study identified key genes and metabolites associated with adipose tissue color differentiation through multi-omics analysis, providing new clues for deciphering the color phenotype of abdominal adipose tissue in equine animals, the complete regulatory network remains unclear. First, the classification of adipose tissue into WAT and YAT was based on visual observation, and the absence of a quantitative colorimetric assessment is a limitation of this study. Second, a major bottleneck is the lack of suitable cellular models, which prevents direct experimental validation of the functions of potential key markers such as stearate and *LPGAT1*. To overcome this limitation, follow-up research will focus on establishing primary adipocyte culture methods from Kazakh horses, thereby providing an essential experimental foundation for mechanistic investigations.

## 5. Conclusions

By integrating transcriptomic and metabolomic analyses, this study reveals the molecular mechanisms underlying color differentiation in the abdominal adipose tissue of Kazakh horses. It offers a novel perspective on the formation and regulation of adipose tissue color phenotypes in equids. Our findings indicate that key genes such as *LPGAT1*, *AKT2*, and *ADH5* play crucial roles in lipid deposition, lipid droplet formation, and the maintenance of thermogenic homeostasis. Additionally, alterations in the metabolic pathways of stearate, myo-inositol, and tyrosine emerged as important biomarkers and regulatory nodes. The coordinated activity of these genes and metabolic pathways suggests that adipose tissue color is not merely a superficial indicator of energy storage strategies but also reflects the underlying metabolic activity and differentiation status of adipocytes. Future research should prioritize functional validation studies using primary adipocytes to clarify the specific roles of these candidate genes in adipose color formation. Such work will offer valuable molecular targets and theoretical support for the evaluation of adipose quality and the genetic improvement of meat-type horses.

## Figures and Tables

**Table 1:** The primers used for RT-qPCR (F = forward, R = reverse).

Gene	Primer Sequence (5′→3′)	Product Length (bp)
Actin	F: GAGGGAAATCGTGCGTGAC	145
	R: CTCGTTGCCGATGGTGAT	
TUFT1	F: CCGCTCAACCAGTCATTC	94
	R: TCCAGACCTCCCCTTCAA	
ENSECAG00000001514	F: CCCCGACCTATGAGATGC	141
	R: TGAACAGGGACCAGACGAT	
ENSECAG00000003563	F: TGCCCAGACTCGCACATC	147
	R: GGTCACAAGCAGAAACAACAC	
IFI27	F: CCAGGCAGGCATCTTAGTC	208
	R: CGCACCCAGTAGGTTGAA	
TMEM98	F: GCCTTGGTGGTGGTTTGC	190
	R: CGATCCAGTCTTCGTTCTCC	
ENSECAG00000034517	F: ACAGTGATGTTGCGATAAGAA	144
	R: TGTCCAAGGCACGGATGA	
APOLD1	F: CTACTTCATCGTCTTCTTCGG	78
	R: GCCTGGCTAACTTTGGTG	
NDRG4	F: GGGGAACACGACATTGAG	96
	R: CCCTGAGGAAACTGCGAC	
POPDC3	F: CCTCTGGGGACCTCTTTG	164
	R: AGAAACTCGCCATCAACTGT	
GADD45G	F: GTCCGCCAAAGTCCTGAA	81
	R: ATGTCGCCTTCGTCCTCC	

**Table 2:** Differential analysis of fatty acid composition between WAT and YAT (ng/mg).

Fatty Acid	WAT	YAT	*p*-Value
C6:0	2.52 ± 0.82	1.77 ± 0.89	0.099
C8:0	5.37 ± 1.18	3.89 ± 1.60	0.053
C10:0	105.03 ± 11.11	99.76 ± 25.46	0.600
C11:0	2.93 ± 0.70	3.56 ± 0.65	0.085
C12:0	344.84 ± 59.04	396.34 ± 54.41	0.091
C13:0	13.70 ± 5.09	18.06 ± 5.58	0.125
C14:0	13,761.99 ± 2036.73	15,888.78 ± 2432.01	0.079
C14:1	584.91 ± 218.92	644.73 ± 156.19	0.539
C15:0	753.99 ± 150.56	975.61 ± 249.41 *	0.049
C16:0	163,027.20 ± 7537.27	173,702.87 ± 16,769.67	0.123
C16:1	21,349.05 ± 4382.85	22,109.72 ± 4382.85	0.752
C17:0	1537.65 ± 317.05	1807.60 ± 283.44	0.094
C17:1	1490.75 ± 248.98	1796.98 ± 232.36 *	0.023
C18:0	24,171.62 ± 5630.17	22,917.50 ± 3235.59	0.593
C18:1n9T	176.59 ± 21.34	176.45 ± 41.98	0.993
C18:1n9C	151,339.60 ± 10,636.39	151,415.30 ± 26,832.65	0.994
C18:2n6C	45,031.78 ± 19,126.25	42,419.27 ± 13,417.67	0.756
C18:3n6	11.25 ± 31.37	2.55 ± 5.44	0.452
C18:3n3	20,114.19 ± 10,125.98	30,499.46 ± 25,240.40	0.308
C20:0	293.25 ± 62.99	292.45 ± 42.48	0.977
C20:1	2607.75 ± 294.36	2367.02 ± 552.76	0.295
C20:2	749.62 ± 234.97	694.46 ± 190.73	0.614
C21:0	16.88 ± 0.98	19.21 ± 1.73 **	0.005
C20:3n6	98.07 ± 35.02	78.02 ± 14.50	0.157
C20:4n6	97.21 ± 16.00	91.19 ± 18.56	0.498
C20:3n3	656.62 ± 297.29	752.30 ± 473.35	0.636
C22:0	35.92 ± 6.18	39.32 ± 11.91	0.489
C20:5n3	47.41 ± 11.97	45.76 ± 10.22	0.771
C22:1n9	88.17 ± 14.96	67.10 ± 21.71 *	0.040
C22:2n6	36.35 ± 4.10	36.35 ± 4.10	0.811
C24:0	33.22 ± 9.04	34.68 ± 9.04	0.751
C24:1	23.83 ± 7.66	22.10 ± 7.66	0.684
ΣSFA	204,106.13 ± 11,477.47	216,201.38 ± 20,560.12	0.168
ΣMUFA	177,660.65 ± 9393.15	178,599.40 ± 26,254.51	0.935
ΣPUFA	66,842.52 ± 26,254.52	74,618.98 ± 33,286.58	0.612

Note: “*” indicates significant differences (*p* < 0.05), and “**” indicates highly significant differences (*p* < 0.01). “T” denotes trans; “C” denotes cis.

**Table 3:** Differential analysis of amino acid composition in WAT and YAT (ng/mg).

Amino Acid	WAT	YAT	*p*-Value
Glycine	995.58 ± 768.76	563.62 ± 504.90	0.205
Serine	623.71 ± 580.75	337.87 ± 326.49	0.424
Valine	1000.77 ± 528.98	880.57 ± 227.05	0.564
Threonine	918.30 ± 369.29	631.12 ± 243.92	0.586
Cysteine	105.05 ± 27.09	148.59 ± 94.90	0.350
Isoleucine	220.14 ± 100.46	223.19 ± 105.83	0.955
Aspartic acid	355.24 ± 47.73	105.69 ± 41.43 **	0.002
Glutamic acid	1083.50 ± 495.70	1040.12 ± 294.24	0.835
Histidine	523.77 ± 409.26	575.43 ± 390.16	0.800
Phenylalanine	406.54 ± 180.71	327.53 ± 166.28	0.378
Arginine	1656.69 ± 967.44	1315.62 ± 545.45	0.404
Lysine	1174.91 ± 667.22	903.55 ± 241.60	0.298
Leucine	568.82 ± 230.64	577.84 ± 251.33	0.943

Note: “**” indicates highly significant differences (*p* < 0.01).

**Table 4:** Summary of RNA analyses following reference genome alignment.

Sample	RawReads	CleanReads	Q20 (%)	Q30 (%)	N (%)	GC (%)	Total Mapped (%)
W-1	38584664	38520540	97.68	94.27	0.00	52.47	94.89
W-2	39621298	39565644	97.79	94.50	0.00	52.69	93.42
W-3	36884886	36813618	97.82	94.69	0.01	53.03	94.12
W-4	38364646	38305350	97.86	94.67	0.01	53.89	94.58
W-5	38502882	38436410	97.82	94.58	0.01	53.41	94.63
W-6	37259244	37200780	97.89	94.81	0.01	50.69	93.30
W-7	57280626	57189988	98.08	95.45	0.02	51.67	94.59
W-8	37643396	37592420	98.20	95.45	0.00	51.07	95.10
Y-1	44218870	44150118	97.96	95.07	0.00	51.54	95.81
Y-2	38191640	38137338	97.77	94.66	0.00	51.93	94.79
Y-3	37779640	37734974	97.54	93.90	0.00	51.65	95.99
Y-4	38807090	38753826	97.68	94.43	0.01	52.76	91.81
Y-5	40818854	40762928	97.89	94.90	0.00	52.07	94.04
Y-6	42630660	42575150	97.79	94.73	0.00	51.99	92.89
Y-7	47051796	46994188	97.51	93.94	0.01	52.04	95.19
Y-8	42457558	42402586	97.77	94.50	0.00	51.68	92.79

**Table 5:** Selected DEMs and their associated metabolic pathways.

DEMs	log_2_ (FC)	VIP	*p*-Value	Pathway
2,3-diphospho-D-glyceric acid	0.274	1.102	0.027	PI3K-AKT signaling pathway
Stearate	−1.074	13.663	0.049	Fatty acid biosynthesis
Glutamine	−1.409	2.809	0.044	Arginine biosynthesis
DL-glutamic acid	−0.9301	3.008	0.008	FoxO signaling pathway
3,5-diiodo-l-tyrosine	−0.569	1.130	0.031	Tyrosine metabolism
Tyramine	−0.316	1.061	0.029	Tyrosine metabolism
Myo-inositol	−1.07	5.840	0.033	Inositol phosphate metabolism
D-Glutamine	−1.381	3.302	0.032	Phospholipase D signaling pathway
Pyridoxine	−1.351	1.592	0.042	Vitamin B6 metabolism
retinol	−0.758	1.463	0.032	Retinol metabolism

## Data Availability

The transcriptomic data generated in this study have been deposited in the NCBI BioProject database under accession number PRJNA1267749.

## Supplementary Materials

The following supporting information can be downloaded at: https://www.mdpi.com/article/10.3390/biology15070563/s1, File S1: The fatty acid quantitative analysis report and representative chromatographic profiles; File S2: The amino acid quantitative analysis report and representative extracted ion chromatograms; Table S1: Instrument parameters and standards for the determination of fatty acids and amino acids; Table S2: List of all genes identified; Table S3: DEGs; Table S4: Results of GO and KEGG enrichment analyses of DEGs; Table S5: GSEA of DEGs; Table S6: List of all metabolites identified; Table S7: DEMs and their HMDB annotations; Table S8: KEGG enrichment results for DEMs; Table S9: Co-enriched KEGG pathways of DEGs and DEMs; Table S10: Top 50 DEGs and DEMs ranked by relevance.

## Abbreviations

The following abbreviations are used in this manuscript:

WAT	white adipose tissue
YAT	yellow adipose tissue
BAT	brown adipose tissue
DEGs	differentially expressed genes
GO	Gene Ontology
KEGG	Kyoto Encyclopedia of Genes and Genomes
GSEA	gene set enrichment analysis
DEMs	differentially expressed metabolites
POS	positive ion mode
NEG	negative ion mode
OPLS-DA	Orthogonal projections to latent structures discriminant analysis
3,5-T2	3,5-diiodo-L-tyrosine
TG	triglyceride
