# Supplementary material for: Biomarkers of Adipose Color: A Multi-Omics Analysis Unravels the Molecular Landscape of White and Yellow Fat in Kazakh Horse
Source: Biology (Basel). 2026 Apr 1;15(7):563. doi: 10.3390/biology15070563 (PMC13072367; doi:10.3390/biology15070563)
Supplement: Supplementary file 1 [file biology-15-00563-s001.zip › File S1.pdf]

定量分析完成报告

**批处理路径**	G:\GC-MS\HX250430-4-GCMS总脂肪酸靶向检测\HX250430-4\QuantResults\HX250430-4.batch.bin		
**分析时间**	2025/5/14 16:58	**分析员姓名**	DESKTOP-M3A0GPO\omics
**报告时间**	2025/5/16 14:52:54	**报告员姓名**	DESKTOP-M3A0GPO\omics
**最近校正更新**	2025/5/14 16:58	**批处理状态**	已处理
**定量批处理版本**	10.2	**定量报告版本**	10.2

**数据文件**	**样品名称**	**样品类型**	**位置**	**进样量**	**级别**	**采集方法**
blank.D	blank	空白	1	1		脂肪酸
std1.D	std1	校正	2	1	1	脂肪酸
std2.D	std2	校正	3	1	2	脂肪酸
std3.D	std3	校正	4	1	3	脂肪酸
std4.D	std4	校正	5	1	4	脂肪酸
std5.D	std5	校正	6	1	5	脂肪酸
std6.D	std6	校正	7	1	6	脂肪酸
std7.D	std7	校正	8	1	7	脂肪酸
std8.D	std8	校正	9	1	8	脂肪酸
std9.D	std9	校正	10	1	9	脂肪酸
std10.D	std10	校正	11	1	10	脂肪酸
qc-3.D	qc-3	样品	30	1		脂肪酸
sp-17.D	sp-17	样品	31	1		脂肪酸
sp-18.D	sp-18	样品	32	1		脂肪酸
sp-19.D	sp-19	样品	33	1		脂肪酸
sp-20.D	sp-20	样品	34	1		脂肪酸
sp-21.D	sp-21	样品	35	1		脂肪酸
sp-22.D	sp-22	样品	36	1		脂肪酸
sp-23.D	sp-23	样品	37	1		脂肪酸
sp-24.D	sp-24	样品	38	1		脂肪酸
qc-4.D	qc-4	样品	39	1		脂肪酸
sp-25.D	sp-25	样品	40	1		脂肪酸
sp-26.D	sp-26	样品	41	1		脂肪酸
sp-27.D	sp-27	样品	42	1		脂肪酸
sp-28.D	sp-28	样品	43	1		脂肪酸
sp-29.D	sp-29	样品	44	1		脂肪酸
sp-30.D	sp-30	样品	45	1		脂肪酸
sp-31.D	sp-31	样品	46	1		脂肪酸
sp-32.D	sp-32	样品	47	1		脂肪酸
qc-5.D	qc-5	样品	48	1		脂肪酸

定量结果

C4:0

数据文件	化合物	ISTD	样品类型	RT	响应	ISTD 响应	响应比	最终浓度	预期的浓度	准确度
blank.D	C4:0	C19:0	Blank	2.203	0	1614233	0.0000	ND		
std1.D	C4:0	C19:0	Calibration	2.203	6903	1476809	0.0047	ND	0.3125	0.0

C4:0

数据文件	化合物	ISTD	样品类型	RT	响应	ISTD 响应	响应比	最终浓度	预期的浓度	准确度
std2.D	C4:0	C19:0	Calibration	2.203	13000	1765013	0.0074	0.3401	0.6250	54.4
std3.D	C4:0	C19:0	Calibration	2.203	23261	1945904	0.0120	1.0705	1.2500	85.6
std4.D	C4:0	C19:0	Calibration	2.203	47125	2060032	0.0229	2.8298	2.5000	113.2
std5.D	C4:0	C19:0	Calibration	2.203	84256	2159349	0.0390	5.4861	5.0000	109.7
std6.D	C4:0	C19:0	Calibration	2.203	161600	2537627	0.0637	9.6846	10.0000	96.8
std7.D	C4:0	C19:0	Calibration	2.203	331688	2635656	0.1258	21.1776	20.0000	105.9
std8.D	C4:0	C19:0	Calibration	2.203	603804	2927430	0.2063	38.8753	40.0000	97.2
std9.D	C4:0	C19:0	Calibration	2.203	1129200	3505157	0.3222	80.4855	80.0000	100.6
std10.D	C4:0	C19:0	Calibration	2.203	2300709	4076680	0.5644	ND	160.0000	
qc-3.D	C4:0	C19:0	Sample	2.203	535644	2180317	0.2457	49.5185		
sp-17.D	C4:0	C19:0	Sample	2.203	0	1822377	0.0000	ND		
sp-18.D	C4:0	C19:0	Sample	2.198	0	1869407	0.0000	ND		
sp-19.D	C4:0	C19:0	Sample	2.203	232	1809576	0.0001	ND		
sp-20.D	C4:0	C19:0	Sample	2.198	89	1890097	0.0000	ND		
sp-21.D	C4:0	C19:0	Sample	2.199	138	2090151	0.0001	ND		
sp-22.D	C4:0	C19:0	Sample	2.199	203	1884649	0.0001	ND		
sp-23.D	C4:0	C19:0	Sample	2.203	144	1819204	0.0001	ND		
sp-24.D	C4:0	C19:0	Sample	2.203	171	1859181	0.0001	ND		
qc-4.D	C4:0	C19:0	Sample	2.203	467088	2169029	0.2153	41.1755		
sp-25.D	C4:0	C19:0	Sample	2.199	122	1815559	0.0001	ND		
sp-26.D	C4:0	C19:0	Sample	2.198	91	1841988	0.0000	ND		
sp-27.D	C4:0	C19:0	Sample	2.199	27	1938349	0.0000	ND		
sp-28.D	C4:0	C19:0	Sample	2.203	51	1948544	0.0000	ND		
sp-29.D	C4:0	C19:0	Sample	2.203	128	1770121	0.0001	ND		
sp-30.D	C4:0	C19:0	Sample	2.203	186	1720523	0.0001	ND		
sp-31.D	C4:0	C19:0	Sample	2.198	208	1756510	0.0001	ND		
sp-32.D	C4:0	C19:0	Sample	2.198	270	1813710	0.0001	ND		
qc-5.D	C4:0	C19:0	Sample	2.198	470662	2038657	0.2309	45.3041		

C6:0

数据文件	化合物	ISTD	样品类型	RT	响应	ISTD 响应	响应比	最终浓度	预期的浓度	准确度
blank.D	C6:0	C19:0	Blank	2.963	81	1614233	0.0001	0.0003		
std1.D	C6:0	C19:0	Calibration	2.959	16067	1476809	0.0109	0.3627	0.3125	116.1
std2.D	C6:0	C19:0	Calibration	2.959	29610	1765013	0.0168	0.6475	0.6250	103.6
std3.D	C6:0	C19:0	Calibration	2.959	47699	1945904	0.0245	1.0756	1.2500	86.0
std4.D	C6:0	C19:0	Calibration	2.959	99304	2060032	0.0482	2.6587	2.5000	106.3
std5.D	C6:0	C19:0	Calibration	2.959	155929	2159349	0.0722	4.5660	5.0000	91.3
std6.D	C6:0	C19:0	Calibration	2.963	302051	2537627	0.1190	8.9120	10.0000	89.1
std7.D	C6:0	C19:0	Calibration	2.958	629485	2635656	0.2388	22.6303	20.0000	113.2
std8.D	C6:0	C19:0	Calibration	2.959	1098736	2927430	0.3753	41.4364	40.0000	103.6
std9.D	C6:0	C19:0	Calibration	2.963	2093987	3505157	0.5974	77.1790	80.0000	96.5

C6:0

数据文件	化合物	ISTD	样品类型	RT	响应	ISTD 响应	响应比	最终浓度	预期的浓度	准确度
std10.D	C6:0	C19:0	Calibration	2.963	4667340	4076680	1.1449	184.2979	160.0000	115.2
qc-3.D	C6:0	C19:0	Sample	2.959	1198829	2180317	0.5498	69.0697		
sp-17.D	C6:0	C19:0	Sample	2.959	892	1822377	0.0005	0.0057		
sp-18.D	C6:0	C19:0	Sample	2.959	666	1869407	0.0004	0.0037		
sp-19.D	C6:0	C19:0	Sample	2.959	1163	1809576	0.0006	0.0082		
sp-20.D	C6:0	C19:0	Sample	2.959	704	1890097	0.0004	0.0040		
sp-21.D	C6:0	C19:0	Sample	2.959	625	2090151	0.0003	0.0030		
sp-22.D	C6:0	C19:0	Sample	2.959	815	1884649	0.0004	0.0048		
sp-23.D	C6:0	C19:0	Sample	2.959	930	1819204	0.0005	0.0061		
sp-24.D	C6:0	C19:0	Sample	2.959	725	1859181	0.0004	0.0042		
qc-4.D	C6:0	C19:0	Sample	2.959	1045244	2169029	0.4819	57.8937		
sp-25.D	C6:0	C19:0	Sample	2.959	370	1815559	0.0002	0.0018		
sp-26.D	C6:0	C19:0	Sample	2.959	571	1841988	0.0003	0.0031		
sp-27.D	C6:0	C19:0	Sample	2.959	326	1938349	0.0002	0.0014		
sp-28.D	C6:0	C19:0	Sample	2.959	528	1948544	0.0003	0.0026		
sp-29.D	C6:0	C19:0	Sample	2.959	569	1770121	0.0003	0.0033		
sp-30.D	C6:0	C19:0	Sample	2.959	616	1720523	0.0004	0.0038		
sp-31.D	C6:0	C19:0	Sample	2.959	656	1756510	0.0004	0.0040		
sp-32.D	C6:0	C19:0	Sample	2.959	948	1813710	0.0005	0.0062		
qc-5.D	C6:0	C19:0	Sample	2.959	1063853	2038657	0.5218	64.4037		

C8:0

数据文件	化合物	ISTD	样品类型	RT	响应	ISTD 响应	响应比	最终浓度	预期的浓度	准确度
blank.D	C8:0	C19:0	Blank	3.782	0	1614233	0.0000	ND		
std1.D	C8:0	C19:0	Calibration	3.728	24525	1476809	0.0166	0.2956	0.3125	94.6
std2.D	C8:0	C19:0	Calibration	3.728	45885	1765013	0.0260	0.5471	0.6250	87.5
std3.D	C8:0	C19:0	Calibration	3.728	73304	1945904	0.0377	0.9105	1.2500	72.8
std4.D	C8:0	C19:0	Calibration	3.728	155912	2060032	0.0757	2.3736	2.5000	94.9
std5.D	C8:0	C19:0	Calibration	3.728	241330	2159349	0.1118	4.0542	5.0000	81.1
std6.D	C8:0	C19:0	Calibration	3.728	463239	2537627	0.1825	7.9535	10.0000	79.5
std7.D	C8:0	C19:0	Calibration	3.728	1028279	2635656	0.3901	22.5716	20.0000	112.9
std8.D	C8:0	C19:0	Calibration	3.728	1792155	2927430	0.6122	41.9072	40.0000	104.8
std9.D	C8:0	C19:0	Calibration	3.728	3387722	3505157	0.9665	78.4596	80.0000	98.1
std10.D	C8:0	C19:0	Calibration	3.728	7014399	4076680	1.7206	173.2433	160.0000	108.3
qc-3.D	C8:0	C19:0	Sample	3.724	1712973	2180317	0.7857	59.0314		
sp-17.D	C8:0	C19:0	Sample	3.724	2815	1822377	0.0015	0.0113		
sp-18.D	C8:0	C19:0	Sample	3.728	2185	1869407	0.0012	0.0077		
sp-19.D	C8:0	C19:0	Sample	3.724	3315	1809576	0.0018	0.0143		
sp-20.D	C8:0	C19:0	Sample	3.724	2295	1890097	0.0012	0.0081		
sp-21.D	C8:0	C19:0	Sample	3.724	2539	2090151	0.0012	0.0081		
sp-22.D	C8:0	C19:0	Sample	3.724	3423	1884649	0.0018	0.0142		

C8:0

数据文件	化合物	ISTD	样品类型	RT	响应	ISTD 响应	响应比	最终浓度	预期的浓度	准确度
sp-23.D	C8:0	C19:0	Sample	3.724	2628	1819204	0.0014	0.0103		
sp-24.D	C8:0	C19:0	Sample	3.724	2496	1859181	0.0013	0.0093		
qc-4.D	C8:0	C19:0	Sample	3.728	1599633	2169029	0.7375	54.1189		
sp-25.D	C8:0	C19:0	Sample	3.724	1281	1815559	0.0007	0.0039		
sp-26.D	C8:0	C19:0	Sample	3.724	2405	1841988	0.0013	0.0090		
sp-27.D	C8:0	C19:0	Sample	3.724	1331	1938349	0.0007	0.0037		
sp-28.D	C8:0	C19:0	Sample	3.724	1948	1948544	0.0010	0.0062		
sp-29.D	C8:0	C19:0	Sample	3.724	1795	1770121	0.0010	0.0064		
sp-30.D	C8:0	C19:0	Sample	3.724	1939	1720523	0.0011	0.0073		
sp-31.D	C8:0	C19:0	Sample	3.724	2183	1756510	0.0012	0.0084		
sp-32.D	C8:0	C19:0	Sample	3.724	2945	1813710	0.0016	0.0121		
qc-5.D	C8:0	C19:0	Sample	3.724	1588662	2038657	0.7793	58.3737		

C10:0

数据文件	化合物	ISTD	样品类型	RT	响应	ISTD 响应	响应比	最终浓度	预期的浓度	准确度
blank.D	C10:0	C19:0	Blank	4.368	0	1614233	0.0000	ND		
std1.D	C10:0	C19:0	Calibration	4.417	32116	1476809	0.0217	0.3603	0.3125	115.3
std2.D	C10:0	C19:0	Calibration	4.418	62998	1765013	0.0357	0.6650	0.6250	106.4
std3.D	C10:0	C19:0	Calibration	4.417	107795	1945904	0.0554	1.1451	1.2500	91.6
std4.D	C10:0	C19:0	Calibration	4.417	227576	2060032	0.1105	2.6886	2.5000	107.5
std5.D	C10:0	C19:0	Calibration	4.417	376172	2159349	0.1742	4.7220	5.0000	94.4
std6.D	C10:0	C19:0	Calibration	4.417	721537	2537627	0.2843	8.6541	10.0000	86.5
std7.D	C10:0	C19:0	Calibration	4.417	1614554	2635656	0.6126	22.3561	20.0000	111.8
std8.D	C10:0	C19:0	Calibration	4.417	2827697	2927430	0.9659	39.2609	40.0000	98.2
std9.D	C10:0	C19:0	Calibration	4.417	5151378	3505157	1.4697	65.9693	80.0000	82.5
std10.D	C10:0	C19:0	Calibration	4.422	8122381	4076680	1.9924	96.1087	160.0000	60.1
qc-3.D	C10:0	C19:0	Sample	4.417	2119359	2180317	0.9720	39.5682		
sp-17.D	C10:0	C19:0	Sample	4.413	26215	1822377	0.0144	0.2162		
sp-18.D	C10:0	C19:0	Sample	4.417	21002	1869407	0.0112	0.1592		
sp-19.D	C10:0	C19:0	Sample	4.413	27830	1809576	0.0154	0.2348		
sp-20.D	C10:0	C19:0	Sample	4.413	24166	1890097	0.0128	0.1868		
sp-21.D	C10:0	C19:0	Sample	4.413	26034	2090151	0.0125	0.1809		
sp-22.D	C10:0	C19:0	Sample	4.413	29128	1884649	0.0155	0.2362		
sp-23.D	C10:0	C19:0	Sample	4.413	24585	1819204	0.0135	0.2001		
sp-24.D	C10:0	C19:0	Sample	4.413	23587	1859181	0.0127	0.1851		
qc-4.D	C10:0	C19:0	Sample	4.413	2197761	2169029	1.0132	41.6526		
sp-25.D	C10:0	C19:0	Sample	4.413	16557	1815559	0.0091	0.1230		
sp-26.D	C10:0	C19:0	Sample	4.413	26483	1841988	0.0144	0.2160		
sp-27.D	C10:0	C19:0	Sample	4.413	18718	1938349	0.0097	0.1321		
sp-28.D	C10:0	C19:0	Sample	4.413	22503	1948544	0.0115	0.1648		
sp-29.D	C10:0	C19:0	Sample	4.413	20412	1770121	0.0115	0.1644		

C10:0

数据文件	化合物	ISTD	样品类型	RT	响应	ISTD 响应	响应比	最终浓度	预期的浓度	准确度
sp-30.D	C10:0	C19:0	Sample	4.413	20693	1720523	0.0120	0.1732		
sp-31.D	C10:0	C19:0	Sample	4.413	26880	1756510	0.0153	0.2333		
sp-32.D	C10:0	C19:0	Sample	4.413	28357	1813710	0.0156	0.2396		
qc-5.D	C10:0	C19:0	Sample	4.413	2117114	2038657	1.0385	42.9392		

C11:0

数据文件	化合物	ISTD	样品类型	RT	响应	ISTD 响应	响应比	最终浓度	预期的浓度	准确度
blank.D	C11:0	C19:0	Blank	4.733	0	1614233	0.0000	ND		
std1.D	C11:0	C19:0	Calibration	4.733	17645	1476809	0.0119	0.1763	0.1563	112.9
std2.D	C11:0	C19:0	Calibration	4.733	35366	1765013	0.0200	0.3294	0.3125	105.4
std3.D	C11:0	C19:0	Calibration	4.729	62803	1945904	0.0323	0.5859	0.6250	93.7
std4.D	C11:0	C19:0	Calibration	4.733	130127	2060032	0.0632	1.3190	1.2500	105.5
std5.D	C11:0	C19:0	Calibration	4.733	224035	2159349	0.1038	2.4024	2.5000	96.1
std6.D	C11:0	C19:0	Calibration	4.733	435048	2537627	0.1714	4.4077	5.0000	88.2
std7.D	C11:0	C19:0	Calibration	4.733	965645	2635656	0.3664	11.0348	10.0000	110.3
std8.D	C11:0	C19:0	Calibration	4.733	1728180	2927430	0.5903	19.6385	20.0000	98.2
std9.D	C11:0	C19:0	Calibration	4.733	3258125	3505157	0.9295	33.9899	40.0000	85.0
std10.D	C11:0	C19:0	Calibration	4.733	6162737	4076680	1.5117	61.1740	80.0000	76.5
qc-3.D	C11:0	C19:0	Sample	4.729	1137858	2180317	0.5219	16.9207		
sp-17.D	C11:0	C19:0	Sample	4.729	1887	1822377	0.0010	0.0092		
sp-18.D	C11:0	C19:0	Sample	4.729	1024	1869407	0.0005	0.0043		
sp-19.D	C11:0	C19:0	Sample	4.729	1064	1809576	0.0006	0.0046		
sp-20.D	C11:0	C19:0	Sample	4.729	1366	1890097	0.0007	0.0059		
sp-21.D	C11:0	C19:0	Sample	4.729	1288	2090151	0.0006	0.0049		
sp-22.D	C11:0	C19:0	Sample	4.729	1349	1884649	0.0007	0.0059		
sp-23.D	C11:0	C19:0	Sample	4.729	1183	1819204	0.0007	0.0052		
sp-24.D	C11:0	C19:0	Sample	4.729	1092	1859181	0.0006	0.0046		
qc-4.D	C11:0	C19:0	Sample	4.729	1239199	2169029	0.5713	18.8763		
sp-25.D	C11:0	C19:0	Sample	4.729	1522	1815559	0.0008	0.0071		
sp-26.D	C11:0	C19:0	Sample	4.729	1364	1841988	0.0007	0.0061		
sp-27.D	C11:0	C19:0	Sample	4.729	1388	1938349	0.0007	0.0059		
sp-28.D	C11:0	C19:0	Sample	4.729	2044	1948544	0.0010	0.0093		
sp-29.D	C11:0	C19:0	Sample	4.728	1441	1770121	0.0008	0.0069		
sp-30.D	C11:0	C19:0	Sample	4.729	962	1720523	0.0006	0.0044		
sp-31.D	C11:0	C19:0	Sample	4.728	1278	1756510	0.0007	0.0060		
sp-32.D	C11:0	C19:0	Sample	4.729	1326	1813710	0.0007	0.0060		
qc-5.D	C11:0	C19:0	Sample	4.729	1185016	2038657	0.5813	19.2746		

C12:0

数据文件	化合物	ISTD	样品类型	RT	响应	ISTD 响应	响应比	最终浓度	预期的浓度	准确度
blank.D	C12:0	C19:0	Blank	5.240	0	1614233	0.0000	ND		

C12:0

数据文件	化合物	ISTD	样品类型	RT	响应	ISTD 响应	响应比	最终浓度	预期的浓度	准确度
std1.D	C12:0	C19:0	Calibration	5.049	35568	1476809	0.0241	0.3179	0.3125	101.7
std2.D	C12:0	C19:0	Calibration	5.049	74112	1765013	0.0420	0.6198	0.6250	99.2
std3.D	C12:0	C19:0	Calibration	5.044	137700	1945904	0.0708	1.1603	1.2500	92.8
std4.D	C12:0	C19:0	Calibration	5.049	285188	2060032	0.1384	2.5983	2.5000	103.9
std5.D	C12:0	C19:0	Calibration	5.049	516546	2159349	0.2392	5.0125	5.0000	100.3
std6.D	C12:0	C19:0	Calibration	5.049	1012197	2537627	0.3989	9.2644	10.0000	92.6
std7.D	C12:0	C19:0	Calibration	5.048	2170310	2635656	0.8234	22.1310	20.0000	110.7
std8.D	C12:0	C19:0	Calibration	5.049	3824034	2927430	1.3063	38.5262	40.0000	96.3
std9.D	C12:0	C19:0	Calibration	5.049	6792748	3505157	1.9379	61.8803	80.0000	77.4
std10.D	C12:0	C19:0	Calibration	5.053	9395661	4076680	2.3047	76.2069	160.0000	47.6
qc-3.D	C12:0	C19:0	Sample	5.044	2330569	2180317	1.0689	30.2779		
sp-17.D	C12:0	C19:0	Sample	5.044	99907	1822377	0.0548	0.8539		
sp-18.D	C12:0	C19:0	Sample	5.044	61588	1869407	0.0329	0.4631		
sp-19.D	C12:0	C19:0	Sample	5.044	79910	1809576	0.0442	0.6585		
sp-20.D	C12:0	C19:0	Sample	5.044	70897	1890097	0.0375	0.5412		
sp-21.D	C12:0	C19:0	Sample	5.044	104147	2090151	0.0498	0.7613		
sp-22.D	C12:0	C19:0	Sample	5.044	100516	1884649	0.0533	0.8261		
sp-23.D	C12:0	C19:0	Sample	5.044	73759	1819204	0.0405	0.5943		
sp-24.D	C12:0	C19:0	Sample	5.044	68193	1859181	0.0367	0.5269		
qc-4.D	C12:0	C19:0	Sample	5.044	2636005	2169029	1.2153	35.3255		
sp-25.D	C12:0	C19:0	Sample	5.044	95131	1815559	0.0524	0.8087		
sp-26.D	C12:0	C19:0	Sample	5.044	103098	1841988	0.0560	0.8754		
sp-27.D	C12:0	C19:0	Sample	5.044	90389	1938349	0.0466	0.7030		
sp-28.D	C12:0	C19:0	Sample	5.044	101737	1948544	0.0522	0.8053		
sp-29.D	C12:0	C19:0	Sample	5.044	66885	1770121	0.0378	0.5460		
sp-30.D	C12:0	C19:0	Sample	5.044	59832	1720523	0.0348	0.4942		
sp-31.D	C12:0	C19:0	Sample	5.044	84086	1756510	0.0479	0.7255		
sp-32.D	C12:0	C19:0	Sample	5.044	92862	1813710	0.0512	0.7866		
qc-5.D	C12:0	C19:0	Sample	5.044	2492631	2038657	1.2227	35.5837		

C13:0

数据文件	化合物	ISTD	样品类型	RT	响应	ISTD 响应	响应比	最终浓度	预期的浓度	准确度
blank.D	C13:0	C19:0	Blank	5.240	0	1614233	0.0000	ND		
std1.D	C13:0	C19:0	Calibration	5.378	17506	1476809	0.0119	0.1577	0.1563	100.9
std2.D	C13:0	C19:0	Calibration	5.378	37633	1765013	0.0213	0.3119	0.3125	99.8
std3.D	C13:0	C19:0	Calibration	5.378	71662	1945904	0.0368	0.5884	0.6250	94.1
std4.D	C13:0	C19:0	Calibration	5.378	146832	2060032	0.0713	1.2667	1.2500	101.3
std5.D	C13:0	C19:0	Calibration	5.378	279061	2159349	0.1292	2.5281	2.5000	101.1
std6.D	C13:0	C19:0	Calibration	5.378	558053	2537627	0.2199	4.6872	5.0000	93.7
std7.D	C13:0	C19:0	Calibration	5.377	1198590	2635656	0.4548	10.8981	10.0000	109.0
std8.D	C13:0	C19:0	Calibration	5.378	2186179	2927430	0.7468	19.3873	20.0000	96.9

C13:0

数据文件	化合物	ISTD	样品类型	RT	响应	ISTD 响应	响应比	最终浓度	预期的浓度	准确度
std9.D	C13:0	C19:0	Calibration	5.378	4183814	3505157	1.1936	33.4222	40.0000	83.6
std10.D	C13:0	C19:0	Calibration	5.382	7475317	4076680	1.8337	55.0265	80.0000	68.8
qc-3.D	C13:0	C19:0	Sample	5.378	1153928	2180317	0.5292	12.9973		
sp-17.D	C13:0	C19:0	Sample	5.378	8117	1822377	0.0045	0.0506		
sp-18.D	C13:0	C19:0	Sample	5.378	3094	1869407	0.0017	0.0160		
sp-19.D	C13:0	C19:0	Sample	5.373	3312	1809576	0.0018	0.0180		
sp-20.D	C13:0	C19:0	Sample	5.378	4424	1890097	0.0023	0.0240		
sp-21.D	C13:0	C19:0	Sample	5.373	5623	2090151	0.0027	0.0282		
sp-22.D	C13:0	C19:0	Sample	5.378	5369	1884649	0.0028	0.0301		
sp-23.D	C13:0	C19:0	Sample	5.378	3977	1819204	0.0022	0.0221		
sp-24.D	C13:0	C19:0	Sample	5.373	3531	1859181	0.0019	0.0188		
qc-4.D	C13:0	C19:0	Sample	5.378	1361368	2169029	0.6276	15.8435		
sp-25.D	C13:0	C19:0	Sample	5.374	6896	1815559	0.0038	0.0421		
sp-26.D	C13:0	C19:0	Sample	5.378	5817	1841988	0.0032	0.0339		
sp-27.D	C13:0	C19:0	Sample	5.373	6601	1938349	0.0034	0.0371		
sp-28.D	C13:0	C19:0	Sample	5.378	9142	1948544	0.0047	0.0538		
sp-29.D	C13:0	C19:0	Sample	5.373	4370	1770121	0.0025	0.0255		
sp-30.D	C13:0	C19:0	Sample	5.373	3153	1720523	0.0018	0.0180		
sp-31.D	C13:0	C19:0	Sample	5.373	4051	1756510	0.0023	0.0236		
sp-32.D	C13:0	C19:0	Sample	5.378	4826	1813710	0.0027	0.0278		
qc-5.D	C13:0	C19:0	Sample	5.378	1266917	2038657	0.6214	15.6621		

C14:0

数据文件	化合物	ISTD	样品类型	RT	响应	ISTD 响应	响应比	最终浓度	预期的浓度	准确度
blank.D	C14:0	C19:0	Blank	5.783	0	1614233	0.0000	ND		
std1.D	C14:0	C19:0	Calibration	5.747	34247	1476809	0.0232	0.4241	0.3125	135.7
std2.D	C14:0	C19:0	Calibration	5.747	76645	1765013	0.0434	0.7959	0.6250	127.3
std3.D	C14:0	C19:0	Calibration	5.742	152965	1945904	0.0786	1.4463	1.2500	115.7
std4.D	C14:0	C19:0	Calibration	5.742	313068	2060032	0.1520	2.8188	2.5000	112.8
std5.D	C14:0	C19:0	Calibration	5.747	619173	2159349	0.2867	5.4012	5.0000	108.0
std6.D	C14:0	C19:0	Calibration	5.747	1247316	2537627	0.4915	9.4926	10.0000	94.9
std7.D	C14:0	C19:0	Calibration	5.747	2575499	2635656	0.9772	20.2091	20.0000	101.0
std8.D	C14:0	C19:0	Calibration	5.747	4648797	2927430	1.5880	36.9235	40.0000	92.3
std9.D	C14:0	C19:0	Calibration	5.747	8355984	3505157	2.3839	ND	80.0000	
std10.D	C14:0	C19:0	Calibration	5.750	11343421	4076680	2.7825	ND	160.0000	
qc-3.D	C14:0	C19:0	Sample	5.742	3045063	2180317	1.3966	31.1327		
sp-17.D	C14:0	C19:0	Sample	5.742	2490228	1822377	1.3665	30.2759		
sp-18.D	C14:0	C19:0	Sample	5.742	1848245	1869407	0.9887	20.4843		
sp-19.D	C14:0	C19:0	Sample	5.743	2252698	1809576	1.2449	26.9460		
sp-20.D	C14:0	C19:0	Sample	5.742	1931585	1890097	1.0220	21.2868		
sp-21.D	C14:0	C19:0	Sample	5.742	2977132	2090151	1.4244	31.9335		

C14:0

数据文件	化合物	ISTD	样品类型	RT	响应	ISTD 响应	响应比	最终浓度	预期的浓度	准确度
sp-22.D	C14:0	C19:0	Sample	5.743	2717254	1884649	1.4418	32.4425		
sp-23.D	C14:0	C19:0	Sample	5.743	1936363	1819204	1.0644	22.3255		
sp-24.D	C14:0	C19:0	Sample	5.742	1874195	1859181	1.0081	20.9509		
qc-4.D	C14:0	C19:0	Sample	5.742	3614151	2169029	1.6663	39.4991		
sp-25.D	C14:0	C19:0	Sample	5.743	2018957	1815559	1.1120	23.5115		
sp-26.D	C14:0	C19:0	Sample	5.742	2997518	1841988	1.6273	38.2002		
sp-27.D	C14:0	C19:0	Sample	5.743	2640268	1938349	1.3621	30.1533		
sp-28.D	C14:0	C19:0	Sample	5.743	2838545	1948544	1.4568	32.8837		
sp-29.D	C14:0	C19:0	Sample	5.742	1897708	1770121	1.0721	22.5151		
sp-30.D	C14:0	C19:0	Sample	5.742	1749426	1720523	1.0168	21.1619		
sp-31.D	C14:0	C19:0	Sample	5.742	2391768	1756510	1.3617	30.1402		
sp-32.D	C14:0	C19:0	Sample	5.742	2442631	1813710	1.3468	29.7226		
qc-5.D	C14:0	C19:0	Sample	5.742	3333821	2038657	1.6353	38.4634		

C14:1

数据文件	化合物	ISTD	样品类型	RT	响应	ISTD 响应	响应比	最终浓度	预期的浓度	准确度
blank.D	C14:1	C19:0	Blank			1614233		ND		
std1.D	C14:1	C19:0	Calibration	5.916	6864	1476809	0.0046	0.1276	0.1563	81.7
std2.D	C14:1	C19:0	Calibration	5.916	14620	1765013	0.0083	0.3110	0.3125	99.5
std3.D	C14:1	C19:0	Calibration	5.911	28748	1945904	0.0148	0.6384	0.6250	102.1
std4.D	C14:1	C19:0	Calibration	5.911	63277	2060032	0.0307	1.4425	1.2500	115.4
std5.D	C14:1	C19:0	Calibration	5.916	118980	2159349	0.0551	2.6725	2.5000	106.9
std6.D	C14:1	C19:0	Calibration	5.916	246253	2537627	0.0970	4.7880	5.0000	95.8
std7.D	C14:1	C19:0	Calibration	5.911	520980	2635656	0.1977	9.8638	10.0000	98.6
std8.D	C14:1	C19:0	Calibration	5.911	965001	2927430	0.3296	16.5208	20.0000	82.6
std9.D	C14:1	C19:0	Calibration	5.916	1927357	3505157	0.5499	27.6292	40.0000	69.1
std10.D	C14:1	C19:0	Calibration	5.916	3591834	4076680	0.8811	44.3358	80.0000	55.4
qc-3.D	C14:1	C19:0	Sample	5.911	469695	2180317	0.2154	10.7596		
sp-17.D	C14:1	C19:0	Sample	5.911	41131	1822377	0.0226	1.0316		
sp-18.D	C14:1	C19:0	Sample	5.911	28667	1869407	0.0153	0.6667		
sp-19.D	C14:1	C19:0	Sample	5.911	75782	1809576	0.0419	2.0055		
sp-20.D	C14:1	C19:0	Sample	5.911	38598	1890097	0.0204	0.9232		
sp-21.D	C14:1	C19:0	Sample	5.911	64327	2090151	0.0308	1.4456		
sp-22.D	C14:1	C19:0	Sample	5.912	53423	1884649	0.0283	1.3230		
sp-23.D	C14:1	C19:0	Sample	5.912	29309	1819204	0.0161	0.7058		
sp-24.D	C14:1	C19:0	Sample	5.911	31711	1859181	0.0171	0.7535		
qc-4.D	C14:1	C19:0	Sample	5.911	574306	2169029	0.2648	13.2489		
sp-25.D	C14:1	C19:0	Sample	5.912	32613	1815559	0.0180	0.7992		
sp-26.D	C14:1	C19:0	Sample	5.911	69355	1841988	0.0377	1.7924		
sp-27.D	C14:1	C19:0	Sample	5.912	44072	1938349	0.0227	1.0400		
sp-28.D	C14:1	C19:0	Sample	5.912	54850	1948544	0.0281	1.3130		

C14:1

数据文件	化合物	ISTD	样品类型	RT	响应	ISTD 响应	响应比	最终浓度	预期的浓度	准确度
sp-29.D	C14:1	C19:0	Sample	5.911	31441	1770121	0.0178	0.7891		
sp-30.D	C14:1	C19:0	Sample	5.911	34713	1720523	0.0202	0.9108		
sp-31.D	C14:1	C19:0	Sample	5.911	49955	1756510	0.0284	1.3277		
sp-32.D	C14:1	C19:0	Sample	5.911	51265	1813710	0.0283	1.3189		
qc-5.D	C14:1	C19:0	Sample	5.911	533690	2038657	0.2618	13.0980		

C15:0

数据文件	化合物	ISTD	样品类型	RT	响应	ISTD 响应	响应比	最终浓度	预期的浓度	准确度
blank.D	C15:0	C19:0	Blank	6.103	0	1614233	0.0000	ND		
std1.D	C15:0	C19:0	Calibration	6.174	15816	1476809	0.0107	0.1448	0.1563	92.6
std2.D	C15:0	C19:0	Calibration	6.174	36008	1765013	0.0204	0.2970	0.3125	95.0
std3.D	C15:0	C19:0	Calibration	6.169	73702	1945904	0.0379	0.5921	0.6250	94.7
std4.D	C15:0	C19:0	Calibration	6.169	150717	2060032	0.0732	1.2339	1.2500	98.7
std5.D	C15:0	C19:0	Calibration	6.174	310000	2159349	0.1436	2.6165	2.5000	104.7
std6.D	C15:0	C19:0	Calibration	6.174	635348	2537627	0.2504	4.8650	5.0000	97.3
std7.D	C15:0	C19:0	Calibration	6.173	1329210	2635656	0.5043	10.6225	10.0000	106.2
std8.D	C15:0	C19:0	Calibration	6.169	2539594	2927430	0.8675	19.4503	20.0000	97.3
std9.D	C15:0	C19:0	Calibration	6.174	5044623	3505157	1.4392	34.2047	40.0000	85.5
std10.D	C15:0	C19:0	Calibration	6.178	8911026	4076680	2.1859	54.5112	80.0000	68.1
qc-3.D	C15:0	C19:0	Sample	6.169	1139469	2180317	0.5226	11.0532		
sp-17.D	C15:0	C19:0	Sample	6.169	216699	1822377	0.1189	2.1207		
sp-18.D	C15:0	C19:0	Sample	6.169	123166	1869407	0.0659	1.0978		
sp-19.D	C15:0	C19:0	Sample	6.169	123499	1809576	0.0682	1.1418		
sp-20.D	C15:0	C19:0	Sample	6.169	151047	1890097	0.0799	1.3615		
sp-21.D	C15:0	C19:0	Sample	6.169	185903	2090151	0.0889	1.5341		
sp-22.D	C15:0	C19:0	Sample	6.169	182744	1884649	0.0970	1.6892		
sp-23.D	C15:0	C19:0	Sample	6.169	134295	1819204	0.0738	1.2462		
sp-24.D	C15:0	C19:0	Sample	6.169	123174	1859181	0.0663	1.1046		
qc-4.D	C15:0	C19:0	Sample	6.169	1365754	2169029	0.6297	13.6060		
sp-25.D	C15:0	C19:0	Sample	6.169	167090	1815559	0.0920	1.5936		
sp-26.D	C15:0	C19:0	Sample	6.169	197248	1841988	0.1071	1.8869		
sp-27.D	C15:0	C19:0	Sample	6.169	219592	1938349	0.1133	2.0092		
sp-28.D	C15:0	C19:0	Sample	6.169	295228	1948544	0.1515	2.7786		
sp-29.D	C15:0	C19:0	Sample	6.169	146788	1770121	0.0829	1.4188		
sp-30.D	C15:0	C19:0	Sample	6.169	114566	1720523	0.0666	1.1109		
sp-31.D	C15:0	C19:0	Sample	6.169	145188	1756510	0.0827	1.4137		
sp-32.D	C15:0	C19:0	Sample	6.169	183207	1813710	0.1010	1.7680		
qc-5.D	C15:0	C19:0	Sample	6.169	1238365	2038657	0.6074	13.0717		

C15:1

数据文件	化合物	ISTD	样品类型	RT	响应	ISTD 响应	响应比	最终浓度	预期的浓度	准确度
blank.D	C15:1	C19:0	Blank	6.352	0	1614233	0.0000	ND		
std1.D	C15:1	C19:0	Calibration	6.383	7138	1476809	0.0048	ND	0.1563	0.0
std2.D	C15:1	C19:0	Calibration	6.383	16056	1765013	0.0091	0.0953	0.3125	30.5
std3.D	C15:1	C19:0	Calibration	6.378	31509	1945904	0.0162	0.4641	0.6250	74.3
std4.D	C15:1	C19:0	Calibration	6.383	65435	2060032	0.0318	1.2733	1.2500	101.9
std5.D	C15:1	C19:0	Calibration	6.383	134263	2159349	0.0622	2.8539	2.5000	114.2
std6.D	C15:1	C19:0	Calibration	6.383	275315	2537627	0.1085	5.2608	5.0000	105.2
std7.D	C15:1	C19:0	Calibration	6.382	577044	2635656	0.2189	11.0005	10.0000	110.0
std8.D	C15:1	C19:0	Calibration	6.378	1114045	2927430	0.3806	19.3995	20.0000	97.0
std9.D	C15:1	C19:0	Calibration	6.383	2236842	3505157	0.6382	32.7868	40.0000	82.0
std10.D	C15:1	C19:0	Calibration	6.387	4130142	4076680	1.0131	52.2728	80.0000	65.3
qc-3.D	C15:1	C19:0	Sample	6.378	504854	2180317	0.2316	11.6560		
sp-17.D	C15:1	C19:0	Sample	6.432	0	1822377	0.0000	ND		
sp-18.D	C15:1	C19:0	Sample	6.432	0	1869407	0.0000	ND		
sp-19.D	C15:1	C19:0	Sample	6.432	0	1809576	0.0000	ND		
sp-20.D	C15:1	C19:0	Sample	6.432	0	1890097	0.0000	ND		
sp-21.D	C15:1	C19:0	Sample	6.432	0	2090151	0.0000	ND		
sp-22.D	C15:1	C19:0	Sample	6.432	0	1884649	0.0000	ND		
sp-23.D	C15:1	C19:0	Sample	6.432	0	1819204	0.0000	ND		
sp-24.D	C15:1	C19:0	Sample	6.432	0	1859181	0.0000	ND		
qc-4.D	C15:1	C19:0	Sample	6.378	564314	2169029	0.2602	13.1432		
sp-25.D	C15:1	C19:0	Sample	6.432	0	1815559	0.0000	ND		
sp-26.D	C15:1	C19:0	Sample	6.432	0	1841988	0.0000	ND		
sp-27.D	C15:1	C19:0	Sample	6.432	0	1938349	0.0000	ND		
sp-28.D	C15:1	C19:0	Sample	6.423	0	1948544	0.0000	ND		
sp-29.D	C15:1	C19:0	Sample	6.432	0	1770121	0.0000	ND		
sp-30.D	C15:1	C19:0	Sample	6.432	0	1720523	0.0000	ND		
sp-31.D	C15:1	C19:0	Sample	6.432	0	1756510	0.0000	ND		
sp-32.D	C15:1	C19:0	Sample	6.432	0	1813710	0.0000	ND		
qc-5.D	C15:1	C19:0	Sample	6.378	511674	2038657	0.2510	12.6660		

C16:0

数据文件	化合物	ISTD	样品类型	RT	响应	ISTD 响应	响应比	最终浓度	预期的浓度	准确度
blank.D	C16:0	C19:0	Blank	6.690	0	1614233	0.0000	ND		
std1.D	C16:0	C19:0	Calibration	6.690	44744	1476809	0.0303	0.1331	0.4688	28.4
std2.D	C16:0	C19:0	Calibration	6.690	105704	1765013	0.0599	0.3444	0.9375	36.7
std3.D	C16:0	C19:0	Calibration	6.690	226913	1945904	0.1166	0.8724	1.8750	46.5
std4.D	C16:0	C19:0	Calibration	6.690	464812	2060032	0.2256	2.1909	3.7500	58.4
std5.D	C16:0	C19:0	Calibration	6.689	986954	2159349	0.4571	5.8654	7.5000	78.2
std6.D	C16:0	C19:0	Calibration	6.689	2036671	2537627	0.8026	12.8648	15.0000	85.8
std7.D	C16:0	C19:0	Calibration	6.694	4112945	2635656	1.5605	32.5265	30.0000	108.4

C16:0

数据文件	化合物	ISTD	样品类型	RT	响应	ISTD 响应	响应比	最终浓度	预期的浓度	准确度
std8.D	C16:0	C19:0	Calibration	6.694	7743304	2927430	2.6451	67.9105	60.0000	113.2
std9.D	C16:0	C19:0	Calibration	6.698	13522533	3505157	3.8579	114.9721	120.0000	95.8
std10.D	C16:0	C19:0	Calibration	6.702	17904657	4076680	4.3920	137.7664	240.0000	57.4
qc-3.D	C16:0	C19:0	Sample	6.694	9869330	2180317	4.5266	143.6910		
sp-17.D	C16:0	C19:0	Sample	6.699	15121477	1822377	8.2977	334.6394		
sp-18.D	C16:0	C19:0	Sample	6.697	13834625	1869407	7.4005	285.2698		
sp-19.D	C16:0	C19:0	Sample	6.696	13790339	1809576	7.6208	297.1807		
sp-20.D	C16:0	C19:0	Sample	6.697	13864612	1890097	7.3354	281.7729		
sp-21.D	C16:0	C19:0	Sample	6.696	16265198	2090151	7.7818	305.9795		
sp-22.D	C16:0	C19:0	Sample	6.699	15498586	1884649	8.2236	330.4794		
sp-23.D	C16:0	C19:0	Sample	6.694	13500084	1819204	7.4209	286.3638		
sp-24.D	C16:0	C19:0	Sample	6.696	14058751	1859181	7.5618	293.9782		
qc-4.D	C16:0	C19:0	Sample	6.694	11047058	2169029	5.0931	169.3840		
sp-25.D	C16:0	C19:0	Sample	6.699	12357375	1815559	6.8064	253.8346		
sp-26.D	C16:0	C19:0	Sample	6.699	16266664	1841988	8.8310	365.0225		
sp-27.D	C16:0	C19:0	Sample	6.698	15651428	1938349	8.0746	322.1577		
sp-28.D	C16:0	C19:0	Sample	6.698	15768732	1948544	8.0926	323.1574		
sp-29.D	C16:0	C19:0	Sample	6.698	13199847	1770121	7.4570	288.3121		
sp-30.D	C16:0	C19:0	Sample	6.698	12856234	1720523	7.4723	289.1350		
sp-31.D	C16:0	C19:0	Sample	6.696	13954516	1756510	7.9445	314.9363		
sp-32.D	C16:0	C19:0	Sample	6.697	14115491	1813710	7.7827	306.0251		
qc-5.D	C16:0	C19:0	Sample	6.694	10239313	2038657	5.0226	166.1215		

C16:1

数据文件	化合物	ISTD	样品类型	RT	响应	ISTD 响应	响应比	最终浓度	预期的浓度	准确度
blank.D	C16:1	C19:0	Blank			1614233		ND		
std1.D	C16:1	C19:0	Calibration	6.881	5056	1476809	0.0034	0.1209	0.1563	77.3
std2.D	C16:1	C19:0	Calibration	6.881	10789	1765013	0.0061	0.2852	0.3125	91.3
std3.D	C16:1	C19:0	Calibration	6.881	24334	1945904	0.0125	0.6759	0.6250	108.1
std4.D	C16:1	C19:0	Calibration	6.881	48292	2060032	0.0234	1.3442	1.2500	107.5
std5.D	C16:1	C19:0	Calibration	6.881	103309	2159349	0.0478	2.8354	2.5000	113.4
std6.D	C16:1	C19:0	Calibration	6.881	217198	2537627	0.0856	5.1422	5.0000	102.8
std7.D	C16:1	C19:0	Calibration	6.880	454539	2635656	0.1725	10.4507	10.0000	104.5
std8.D	C16:1	C19:0	Calibration	6.885	913884	2927430	0.3122	18.9893	20.0000	94.9
std9.D	C16:1	C19:0	Calibration	6.885	1864800	3505157	0.5320	32.4237	40.0000	81.1
std10.D	C16:1	C19:0	Calibration	6.890	3434330	4076680	0.8424	51.3937	80.0000	64.2
qc-3.D	C16:1	C19:0	Sample	6.881	776605	2180317	0.3562	21.6787		
sp-17.D	C16:1	C19:0	Sample	6.885	1124711	1822377	0.6172	37.6274		
sp-18.D	C16:1	C19:0	Sample	6.881	923349	1869407	0.4939	30.0960		
sp-19.D	C16:1	C19:0	Sample	6.881	1592899	1809576	0.8803	53.7054		
sp-20.D	C16:1	C19:0	Sample	6.881	1084691	1890097	0.5739	34.9822		

C16:1

数据文件	化合物	ISTD	样品类型	RT	响应	ISTD 响应	响应比	最终浓度	预期的浓度	准确度
sp-21.D	C16:1	C19:0	Sample	6.881	1756328	2090151	0.8403	51.2626		
sp-22.D	C16:1	C19:0	Sample	6.885	1531725	1884649	0.8127	49.5790		
sp-23.D	C16:1	C19:0	Sample	6.881	869527	1819204	0.4780	29.1210		
sp-24.D	C16:1	C19:0	Sample	6.881	1001124	1859181	0.5385	32.8185		
qc-4.D	C16:1	C19:0	Sample	6.881	898005	2169029	0.4140	25.2124		
sp-25.D	C16:1	C19:0	Sample	6.881	752466	1815559	0.4145	25.2394		
sp-26.D	C16:1	C19:0	Sample	6.885	1796563	1841988	0.9753	59.5157		
sp-27.D	C16:1	C19:0	Sample	6.881	1349551	1938349	0.6962	42.4595		
sp-28.D	C16:1	C19:0	Sample	6.881	1419469	1948544	0.7285	44.4297		
sp-29.D	C16:1	C19:0	Sample	6.881	830552	1770121	0.4692	28.5854		
sp-30.D	C16:1	C19:0	Sample	6.881	990550	1720523	0.5757	35.0949		
sp-31.D	C16:1	C19:0	Sample	6.881	1075159	1756510	0.6121	37.3177		
sp-32.D	C16:1	C19:0	Sample	6.881	1269982	1813710	0.7002	42.7024		
qc-5.D	C16:1	C19:0	Sample	6.881	817839	2038657	0.4012	24.4273		

C17:0

数据文件	化合物	ISTD	样品类型	RT	响应	ISTD 响应	响应比	最终浓度	预期的浓度	准确度
blank.D	C17:0	C19:0	Blank	7.339	231	1614233	0.0001	0.0030		
std1.D	C17:0	C19:0	Calibration	7.343	12199	1476809	0.0083	0.1723	0.1563	110.3
std2.D	C17:0	C19:0	Calibration	7.343	28406	1765013	0.0161	0.3358	0.3125	107.4
std3.D	C17:0	C19:0	Calibration	7.339	63123	1945904	0.0324	0.6768	0.6250	108.3
std4.D	C17:0	C19:0	Calibration	7.343	129613	2060032	0.0629	1.3127	1.2500	105.0
std5.D	C17:0	C19:0	Calibration	7.343	287436	2159349	0.1331	2.7771	2.5000	111.1
std6.D	C17:0	C19:0	Calibration	7.343	621619	2537627	0.2450	5.1106	5.0000	102.2
std7.D	C17:0	C19:0	Calibration	7.343	1294994	2635656	0.4913	10.2507	10.0000	102.5
std8.D	C17:0	C19:0	Calibration	7.343	2695183	2927430	0.9207	19.2078	20.0000	96.0
std9.D	C17:0	C19:0	Calibration	7.348	5748032	3505157	1.6399	34.2127	40.0000	85.5
std10.D	C17:0	C19:0	Calibration	7.352	10377085	4076680	2.5455	53.1061	80.0000	66.4
qc-3.D	C17:0	C19:0	Sample	7.339	1100187	2180317	0.5046	10.5274		
sp-17.D	C17:0	C19:0	Sample	7.339	350945	1822377	0.1926	4.0177		
sp-18.D	C17:0	C19:0	Sample	7.339	233475	1869407	0.1249	2.6056		
sp-19.D	C17:0	C19:0	Sample	7.334	170876	1809576	0.0944	1.9701		
sp-20.D	C17:0	C19:0	Sample	7.339	310994	1890097	0.1645	3.4328		
sp-21.D	C17:0	C19:0	Sample	7.339	246553	2090151	0.1180	2.4610		
sp-22.D	C17:0	C19:0	Sample	7.339	253343	1884649	0.1344	2.8045		
sp-23.D	C17:0	C19:0	Sample	7.339	251696	1819204	0.1384	2.8865		
sp-24.D	C17:0	C19:0	Sample	7.339	226990	1859181	0.1221	2.5472		
qc-4.D	C17:0	C19:0	Sample	7.339	1215142	2169029	0.5602	11.6879		
sp-25.D	C17:0	C19:0	Sample	7.339	260771	1815559	0.1436	2.9966		
sp-26.D	C17:0	C19:0	Sample	7.339	281191	1841988	0.1527	3.1849		
sp-27.D	C17:0	C19:0	Sample	7.339	326975	1938349	0.1687	3.5193		

C17:0

数据文件	化合物	ISTD	样品类型	RT	响应	ISTD 响应	响应比	最终浓度	预期的浓度	准确度
sp-28.D	C17:0	C19:0	Sample	7.339	414948	1948544	0.2130	4.4428		
sp-29.D	C17:0	C19:0	Sample	7.334	263685	1770121	0.1490	3.1078		
sp-30.D	C17:0	C19:0	Sample	7.334	221281	1720523	0.1286	2.6832		
sp-31.D	C17:0	C19:0	Sample	7.339	230258	1756510	0.1311	2.7349		
sp-32.D	C17:0	C19:0	Sample	7.339	254978	1813710	0.1406	2.9330		
qc-5.D	C17:0	C19:0	Sample	7.339	1104091	2038657	0.5416	11.2989		

C17:1

数据文件	化合物	ISTD	样品类型	RT	响应	ISTD 响应	响应比	最终浓度	预期的浓度	准确度
blank.D	C17:1	C19:0	Blank			1614233		ND		
std1.D	C17:1	C19:0	Calibration	7.592	4563	1476809	0.0031	0.1375	0.1563	88.0
std2.D	C17:1	C19:0	Calibration	7.592	10861	1765013	0.0062	0.3025	0.3125	96.8
std3.D	C17:1	C19:0	Calibration	7.592	23940	1945904	0.0123	0.6337	0.6250	101.4
std4.D	C17:1	C19:0	Calibration	7.592	50280	2060032	0.0244	1.2857	1.2500	102.9
std5.D	C17:1	C19:0	Calibration	7.592	110720	2159349	0.0513	2.7327	2.5000	109.3
std6.D	C17:1	C19:0	Calibration	7.588	240779	2537627	0.0949	5.0814	5.0000	101.6
std7.D	C17:1	C19:0	Calibration	7.592	506735	2635656	0.1923	10.3260	10.0000	103.3
std8.D	C17:1	C19:0	Calibration	7.592	1053004	2927430	0.3597	19.3441	20.0000	96.7
std9.D	C17:1	C19:0	Calibration	7.597	2237317	3505157	0.6383	34.3486	40.0000	85.9
std10.D	C17:1	C19:0	Calibration	7.601	4118789	4076680	1.0103	54.3859	80.0000	68.0
qc-3.D	C17:1	C19:0	Sample	7.588	413093	2180317	0.1895	10.1754		
sp-17.D	C17:1	C19:0	Sample	7.557	121871	1822377	0.0669	3.5729		
sp-18.D	C17:1	C19:0	Sample	7.557	75600	1869407	0.0404	2.1492		
sp-19.D	C17:1	C19:0	Sample	7.552	84598	1809576	0.0468	2.4890		
sp-20.D	C17:1	C19:0	Sample	7.557	123984	1890097	0.0656	3.5040		
sp-21.D	C17:1	C19:0	Sample	7.557	107123	2090151	0.0513	2.7314		
sp-22.D	C17:1	C19:0	Sample	7.557	112296	1884649	0.0596	3.1803		
sp-23.D	C17:1	C19:0	Sample	7.557	83644	1819204	0.0460	2.4474		
sp-24.D	C17:1	C19:0	Sample	7.557	73535	1859181	0.0396	2.1013		
qc-4.D	C17:1	C19:0	Sample	7.588	466644	2169029	0.2151	11.5582		
sp-25.D	C17:1	C19:0	Sample	7.557	98500	1815559	0.0543	2.8931		
sp-26.D	C17:1	C19:0	Sample	7.557	131720	1841988	0.0715	3.8225		
sp-27.D	C17:1	C19:0	Sample	7.557	122779	1938349	0.0633	3.3826		
sp-28.D	C17:1	C19:0	Sample	7.557	147672	1948544	0.0758	4.0528		
sp-29.D	C17:1	C19:0	Sample	7.557	95545	1770121	0.0540	2.8782		
sp-30.D	C17:1	C19:0	Sample	7.552	84984	1720523	0.0494	2.6314		
sp-31.D	C17:1	C19:0	Sample	7.552	87327	1756510	0.0497	2.6488		
sp-32.D	C17:1	C19:0	Sample	7.552	108985	1813710	0.0601	3.2075		
qc-5.D	C17:1	C19:0	Sample	7.583	421295	2038657	0.2067	11.1011		

C18:0

数据文件	化合物	ISTD	样品类型	RT	响应	ISTD 响应	响应比	最终浓度	预期的浓度	准确度
blank.D	C18:0	C19:0	Blank	8.179	209	1614233	0.0001	0.1103		
std1.D	C18:0	C19:0	Calibration	8.179	21989	1476809	0.0149	0.3982	0.3125	127.4
std2.D	C18:0	C19:0	Calibration	8.179	51052	1765013	0.0289	0.6723	0.6250	107.6
std3.D	C18:0	C19:0	Calibration	8.179	118731	1945904	0.0610	1.3004	1.2500	104.0
std4.D	C18:0	C19:0	Calibration	8.179	245607	2060032	0.1192	2.4450	2.5000	97.8
std5.D	C18:0	C19:0	Calibration	8.179	558555	2159349	0.2587	5.2141	5.0000	104.3
std6.D	C18:0	C19:0	Calibration	8.179	1227136	2537627	0.4836	9.7654	10.0000	97.7
std7.D	C18:0	C19:0	Calibration	8.183	2520868	2635656	0.9564	19.7089	20.0000	98.5
std8.D	C18:0	C19:0	Calibration	8.188	5409063	2927430	1.8477	40.1227	40.0000	100.3
std9.D	C18:0	C19:0	Calibration	8.197	11509999	3505157	3.2837	80.1143	80.0000	100.1
std10.D	C18:0	C19:0	Calibration	8.210	20066589	4076680	4.9223	159.9077	160.0000	99.9
qc-3.D	C18:0	C19:0	Sample	8.184	3902534	2180317	1.7899	38.7198		
sp-17.D	C18:0	C19:0	Sample	8.188	4489811	1822377	2.4637	55.9117		
sp-18.D	C18:0	C19:0	Sample	8.184	4231490	1869407	2.2635	50.5980		
sp-19.D	C18:0	C19:0	Sample	8.184	2677429	1809576	1.4796	31.3911		
sp-20.D	C18:0	C19:0	Sample	8.184	4076298	1890097	2.1567	47.8365		
sp-21.D	C18:0	C19:0	Sample	8.184	3344449	2090151	1.6001	34.1989		
sp-22.D	C18:0	C19:0	Sample	8.184	3403211	1884649	1.8058	39.1034		
sp-23.D	C18:0	C19:0	Sample	8.184	4291048	1819204	2.3588	53.1014		
sp-24.D	C18:0	C19:0	Sample	8.184	3550711	1859181	1.9098	41.6432		
qc-4.D	C18:0	C19:0	Sample	8.179	4077123	2169029	1.8797	40.9039		
sp-25.D	C18:0	C19:0	Sample	8.179	2780468	1815559	1.5315	32.5940		
sp-26.D	C18:0	C19:0	Sample	8.188	3682425	1841988	1.9992	43.8563		
sp-27.D	C18:0	C19:0	Sample	8.184	3722941	1938349	1.9207	41.9104		
sp-28.D	C18:0	C19:0	Sample	8.184	3822693	1948544	1.9618	42.9275		
sp-29.D	C18:0	C19:0	Sample	8.179	3384934	1770121	1.9123	41.7032		
sp-30.D	C18:0	C19:0	Sample	8.179	3465857	1720523	2.0144	44.2375		
sp-31.D	C18:0	C19:0	Sample	8.179	3315577	1756510	1.8876	41.0973		
sp-32.D	C18:0	C19:0	Sample	8.179	2872982	1813710	1.5840	33.8219		
qc-5.D	C18:0	C19:0	Sample	8.179	3767970	2038657	1.8483	40.1359		

C18:1n9t

数据文件	化合物	ISTD	样品类型	RT	响应	ISTD 响应	响应比	最终浓度	预期的浓度	准确度
blank.D	C18:1n9t	C19:0	Blank			1614233		ND		
std1.D	C18:1n9t	C19:0	Calibration	8.317	3449	1476809	0.0023	0.1378	0.1563	88.2
std2.D	C18:1n9t	C19:0	Calibration	8.317	8141	1765013	0.0046	0.2721	0.3125	87.1
std3.D	C18:1n9t	C19:0	Calibration	8.317	19084	1945904	0.0098	0.5785	0.6250	92.6
std4.D	C18:1n9t	C19:0	Calibration	8.317	41913	2060032	0.0203	1.2002	1.2500	96.0
std5.D	C18:1n9t	C19:0	Calibration	8.321	95905	2159349	0.0444	2.6200	2.5000	104.8
std6.D	C18:1n9t	C19:0	Calibration	8.317	214075	2537627	0.0844	4.9764	5.0000	99.5
std7.D	C18:1n9t	C19:0	Calibration	8.321	453645	2635656	0.1721	10.1532	10.0000	101.5

C18:1n9t

数据文件	化合物	ISTD	样品类型	RT	响应	ISTD 响应	响应比	最终浓度	预期的浓度	准确度
std8.D	C18:1n9t	C19:0	Calibration	8.326	987844	2927430	0.3374	19.9057	20.0000	99.5
std9.D	C18:1n9t	C19:0	Calibration	8.330	2162177	3505157	0.6169	36.3881	40.0000	91.0
std10.D	C18:1n9t	C19:0	Calibration	8.344	4028333	4076680	0.9881	58.2900	80.0000	72.9
qc-3.D	C18:1n9t	C19:0	Sample	8.321	358828	2180317	0.1646	9.7083		
sp-17.D	C18:1n9t	C19:0	Sample	8.326	9818	1822377	0.0054	0.3178		
sp-18.D	C18:1n9t	C19:0	Sample	8.321	9766	1869407	0.0052	0.3082		
sp-19.D	C18:1n9t	C19:0	Sample	8.322	10968	1809576	0.0061	0.3575		
sp-20.D	C18:1n9t	C19:0	Sample	8.326	13116	1890097	0.0069	0.4093		
sp-21.D	C18:1n9t	C19:0	Sample	8.322	10204	2090151	0.0049	0.2880		
sp-22.D	C18:1n9t	C19:0	Sample	8.322	10498	1884649	0.0056	0.3286		
sp-23.D	C18:1n9t	C19:0	Sample	8.322	9498	1819204	0.0052	0.3080		
sp-24.D	C18:1n9t	C19:0	Sample	8.321	8991	1859181	0.0048	0.2853		
qc-4.D	C18:1n9t	C19:0	Sample	8.321	380372	2169029	0.1754	10.3447		
sp-25.D	C18:1n9t	C19:0	Sample	8.317	6301	1815559	0.0035	0.2047		
sp-26.D	C18:1n9t	C19:0	Sample	8.326	12081	1841988	0.0066	0.3869		
sp-27.D	C18:1n9t	C19:0	Sample	8.322	9964	1938349	0.0051	0.3032		
sp-28.D	C18:1n9t	C19:0	Sample	8.322	8493	1948544	0.0044	0.2571		
sp-29.D	C18:1n9t	C19:0	Sample	8.321	9220	1770121	0.0052	0.3073		
sp-30.D	C18:1n9t	C19:0	Sample	8.321	11567	1720523	0.0067	0.3966		
sp-31.D	C18:1n9t	C19:0	Sample	8.317	9457	1756510	0.0054	0.3176		
sp-32.D	C18:1n9t	C19:0	Sample	8.321	9180	1813710	0.0051	0.2986		
qc-5.D	C18:1n9t	C19:0	Sample	8.317	348694	2038657	0.1710	10.0896		

C18:1n9c

数据文件	化合物	ISTD	样品类型	RT	响应	ISTD 响应	响应比	最终浓度	预期的浓度	准确度
blank.D	C18:1n9c	C19:0	Blank			1614233		ND		
std1.D	C18:1n9c	C19:0	Calibration	8.419	7714	1476809	0.0052	0.2292	0.3125	73.4
std2.D	C18:1n9c	C19:0	Calibration	8.419	18398	1765013	0.0104	0.4785	0.6250	76.6
std3.D	C18:1n9c	C19:0	Calibration	8.419	43086	1945904	0.0221	1.0674	1.2500	85.4
std4.D	C18:1n9c	C19:0	Calibration	8.419	91542	2060032	0.0444	2.2413	2.5000	89.7
std5.D	C18:1n9c	C19:0	Calibration	8.419	211106	2159349	0.0978	5.1902	5.0000	103.8
std6.D	C18:1n9c	C19:0	Calibration	8.419	465395	2537627	0.1834	10.1427	10.0000	101.4
std7.D	C18:1n9c	C19:0	Calibration	8.423	967718	2635656	0.3672	21.2426	20.0000	106.2
std8.D	C18:1n9c	C19:0	Calibration	8.424	2049949	2927430	0.7003	42.2495	40.0000	105.6
std9.D	C18:1n9c	C19:0	Calibration	8.437	4300626	3505157	1.2269	76.7739	80.0000	96.0
std10.D	C18:1n9c	C19:0	Calibration	8.446	7665882	4076680	1.8804	120.9740	160.0000	75.6
qc-3.D	C18:1n9c	C19:0	Sample	8.428	4022699	2180317	1.8450	118.5490		
sp-17.D	C18:1n9c	C19:0	Sample	8.441	7757052	1822377	4.2566	288.7664		
sp-18.D	C18:1n9c	C19:0	Sample	8.437	7122606	1869407	3.8101	256.6235		
sp-19.D	C18:1n9c	C19:0	Sample	8.437	7495252	1809576	4.1420	280.4966		
sp-20.D	C18:1n9c	C19:0	Sample	8.442	8925079	1890097	4.7220	322.5106		

C18:1n9c

数据文件	化合物	ISTD	样品类型	RT	响应	ISTD 响应	响应比	最终浓度	预期的浓度	准确度
sp-21.D	C18:1n9c	C19:0	Sample	8.437	8396776	2090151	4.0173	271.5130		
sp-22.D	C18:1n9c	C19:0	Sample	8.437	7970067	1884649	4.2289	286.7715		
sp-23.D	C18:1n9c	C19:0	Sample	8.437	7195558	1819204	3.9553	267.0546		
sp-24.D	C18:1n9c	C19:0	Sample	8.433	7089862	1859181	3.8134	256.8634		
qc-4.D	C18:1n9c	C19:0	Sample	8.428	4211407	2169029	1.9416	125.1705		
sp-25.D	C18:1n9c	C19:0	Sample	8.428	4844008	1815559	2.6681	175.5911		
sp-26.D	C18:1n9c	C19:0	Sample	8.442	8835371	1841988	4.7966	327.9417		
sp-27.D	C18:1n9c	C19:0	Sample	8.437	8104375	1938349	4.1811	283.3158		
sp-28.D	C18:1n9c	C19:0	Sample	8.437	7518298	1948544	3.8584	260.0917		
sp-29.D	C18:1n9c	C19:0	Sample	8.437	7034089	1770121	3.9738	268.3820		
sp-30.D	C18:1n9c	C19:0	Sample	8.437	7339255	1720523	4.2657	289.4277		
sp-31.D	C18:1n9c	C19:0	Sample	8.433	7021385	1756510	3.9973	270.0769		
sp-32.D	C18:1n9c	C19:0	Sample	8.433	6843617	1813710	3.7733	253.9834		
qc-5.D	C18:1n9c	C19:0	Sample	8.424	3903978	2038657	1.9150	123.3427		

C18:2n6t

数据文件	化合物	ISTD	样品类型	RT	响应	ISTD 响应	响应比	最终浓度	预期的浓度	准确度
blank.D	C18:2n6t	C19:0	Blank			1614233		ND		
std1.D	C18:2n6t	C19:0	Calibration	8.633	3724	1476809	0.0025	0.1569	0.1563	100.4
std2.D	C18:2n6t	C19:0	Calibration	8.633	9023	1765013	0.0051	0.2990	0.3125	95.7
std3.D	C18:2n6t	C19:0	Calibration	8.633	20734	1945904	0.0107	0.6028	0.6250	96.5
std4.D	C18:2n6t	C19:0	Calibration	8.633	44231	2060032	0.0215	1.1957	1.2500	95.7
std5.D	C18:2n6t	C19:0	Calibration	8.633	102750	2159349	0.0476	2.6272	2.5000	105.1
std6.D	C18:2n6t	C19:0	Calibration	8.633	233418	2537627	0.0920	5.0612	5.0000	101.2
std7.D	C18:2n6t	C19:0	Calibration	8.637	503880	2635656	0.1912	10.4991	10.0000	105.0
std8.D	C18:2n6t	C19:0	Calibration	8.637	1109192	2927430	0.3789	20.7899	20.0000	103.9
std9.D	C18:2n6t	C19:0	Calibration	8.642	2467607	3505157	0.7040	38.6118	40.0000	96.5
std10.D	C18:2n6t	C19:0	Calibration	8.650	4616889	4076680	1.1325	62.1033	80.0000	77.6
qc-3.D	C18:2n6t	C19:0	Sample	8.633	400318	2180317	0.1836	10.0840		
sp-17.D	C18:2n6t	C19:0	Sample	8.882	0	1822377	0.0000	ND		
sp-18.D	C18:2n6t	C19:0	Sample	8.877	0	1869407	0.0000	ND		
sp-19.D	C18:2n6t	C19:0	Sample	8.877	0	1809576	0.0000	ND		
sp-20.D	C18:2n6t	C19:0	Sample	8.877	0	1890097	0.0000	ND		
sp-21.D	C18:2n6t	C19:0	Sample	8.882	0	2090151	0.0000	ND		
sp-22.D	C18:2n6t	C19:0	Sample	8.877	0	1884649	0.0000	ND		
sp-23.D	C18:2n6t	C19:0	Sample	8.877	0	1819204	0.0000	ND		
sp-24.D	C18:2n6t	C19:0	Sample	8.882	0	1859181	0.0000	ND		
qc-4.D	C18:2n6t	C19:0	Sample	8.628	420052	2169029	0.1937	10.6351		
sp-25.D	C18:2n6t	C19:0	Sample	8.877	0	1815559	0.0000	ND		
sp-26.D	C18:2n6t	C19:0	Sample	8.877	0	1841988	0.0000	ND		
sp-27.D	C18:2n6t	C19:0	Sample	8.877	0	1938349	0.0000	ND		

C18:2n6t

数据文件	化合物	ISTD	样品类型	RT	响应	ISTD 响应	响应比	最终浓度	预期的浓度	准确度
sp-28.D	C18:2n6t	C19:0	Sample	8.882	0	1948544	0.0000	ND		
sp-29.D	C18:2n6t	C19:0	Sample	8.877	0	1770121	0.0000	ND		
sp-30.D	C18:2n6t	C19:0	Sample	8.873	0	1720523	0.0000	ND		
sp-31.D	C18:2n6t	C19:0	Sample	8.877	0	1756510	0.0000	ND		
sp-32.D	C18:2n6t	C19:0	Sample	8.877	0	1813710	0.0000	ND		
qc-5.D	C18:2n6t	C19:0	Sample	8.628	387449	2038657	0.1901	10.4373		

C18:2n6c

数据文件	化合物	ISTD	样品类型	RT	响应	ISTD 响应	响应比	最终浓度	预期的浓度	准确度
blank.D	C18:2n6c	C19:0	Blank	9.126	0	1614233	0.0000	ND		
std1.D	C18:2n6c	C19:0	Calibration	8.877	4037	1476809	0.0027	0.2219	0.1563	142.0
std2.D	C18:2n6c	C19:0	Calibration	8.877	9822	1765013	0.0056	0.3862	0.3125	123.6
std3.D	C18:2n6c	C19:0	Calibration	8.877	22033	1945904	0.0113	0.6887	0.6250	110.2
std4.D	C18:2n6c	C19:0	Calibration	8.877	45969	2060032	0.0223	1.2289	1.2500	98.3
std5.D	C18:2n6c	C19:0	Calibration	8.877	106206	2159349	0.0492	2.5065	2.5000	100.3
std6.D	C18:2n6c	C19:0	Calibration	8.877	236172	2537627	0.0931	4.6152	5.0000	92.3
std7.D	C18:2n6c	C19:0	Calibration	8.881	506011	2635656	0.1920	9.6682	10.0000	96.7
std8.D	C18:2n6c	C19:0	Calibration	8.882	1101305	2927430	0.3762	20.2925	20.0000	101.5
std9.D	C18:2n6c	C19:0	Calibration	8.891	2429948	3505157	0.6932	42.1630	40.0000	105.4
std10.D	C18:2n6c	C19:0	Calibration	8.895	4531701	4076680	1.1116	77.8080	80.0000	97.3
qc-3.D	C18:2n6c	C19:0	Sample	8.877	1118607	2180317	0.5130	29.1849		
sp-17.D	C18:2n6c	C19:0	Sample	8.882	2790338	1822377	1.5312	121.4369		
sp-18.D	C18:2n6c	C19:0	Sample	8.877	1600751	1869407	0.8563	55.1359		
sp-19.D	C18:2n6c	C19:0	Sample	8.877	1230662	1809576	0.6801	41.1663		
sp-20.D	C18:2n6c	C19:0	Sample	8.877	1408111	1890097	0.7450	46.1537		
sp-21.D	C18:2n6c	C19:0	Sample	8.882	3594647	2090151	1.7198	143.7240		
sp-22.D	C18:2n6c	C19:0	Sample	8.877	2186253	1884649	1.1600	82.4345		
sp-23.D	C18:2n6c	C19:0	Sample	8.877	1969952	1819204	1.0829	75.1104		
sp-24.D	C18:2n6c	C19:0	Sample	8.882	2489368	1859181	1.3390	100.4500		
qc-4.D	C18:2n6c	C19:0	Sample	8.877	1185916	2169029	0.5467	31.5031		
sp-25.D	C18:2n6c	C19:0	Sample	8.877	2272941	1815559	1.2519	91.5053		
sp-26.D	C18:2n6c	C19:0	Sample	8.877	1716583	1841988	0.9319	61.5508		
sp-27.D	C18:2n6c	C19:0	Sample	8.877	1814962	1938349	0.9363	61.9340		
sp-28.D	C18:2n6c	C19:0	Sample	8.882	3012368	1948544	1.5460	123.1250		
sp-29.D	C18:2n6c	C19:0	Sample	8.877	2146466	1770121	1.2126	87.5779		
sp-30.D	C18:2n6c	C19:0	Sample	8.873	1013345	1720523	0.5890	34.4785		
sp-31.D	C18:2n6c	C19:0	Sample	8.877	1922838	1756510	1.0947	76.2156		
sp-32.D	C18:2n6c	C19:0	Sample	8.877	1825150	1813710	1.0063	68.1068		
qc-5.D	C18:2n6c	C19:0	Sample	8.873	1091184	2038657	0.5352	30.7062		

C18:3n6

数据文件	化合物	ISTD	样品类型	RT	响应	ISTD 响应	响应比	最终浓度	预期的浓度	准确度
blank.D	C18:3n6	C19:0	Blank	9.126	16700	1614233	0.0103	ND		
std1.D	C18:3n6	C19:0	Calibration	9.131	20243	1476809	0.0137	0.1220	0.1563	78.1
std2.D	C18:3n6	C19:0	Calibration	9.135	30134	1765013	0.0171	0.3084	0.3125	98.7
std3.D	C18:3n6	C19:0	Calibration	9.140	45753	1945904	0.0235	0.6652	0.6250	106.4
std4.D	C18:3n6	C19:0	Calibration	9.144	72173	2060032	0.0350	1.3035	1.2500	104.3
std5.D	C18:3n6	C19:0	Calibration	9.144	133432	2159349	0.0618	2.7858	2.5000	111.4
std6.D	C18:3n6	C19:0	Calibration	9.144	263826	2537627	0.1040	5.1221	5.0000	102.4
std7.D	C18:3n6	C19:0	Calibration	9.148	515357	2635656	0.1955	10.1947	10.0000	101.9
std8.D	C18:3n6	C19:0	Calibration	9.149	1055796	2927430	0.3607	19.3421	20.0000	96.7
std9.D	C18:3n6	C19:0	Calibration	9.153	2245396	3505157	0.6406	34.8502	40.0000	87.1
std10.D	C18:3n6	C19:0	Calibration	9.162	4193173	4076680	1.0286	56.3433	80.0000	70.4
qc-3.D	C18:3n6	C19:0	Sample	9.144	400931	2180317	0.1839	9.5495		
sp-17.D	C18:3n6	C19:0	Sample	9.131	26802	1822377	0.0147	0.1774		
sp-18.D	C18:3n6	C19:0	Sample	9.122	20450	1869407	0.0109	ND		
sp-19.D	C18:3n6	C19:0	Sample	9.122	19057	1809576	0.0105	ND		
sp-20.D	C18:3n6	C19:0	Sample	9.122	19410	1890097	0.0103	ND		
sp-21.D	C18:3n6	C19:0	Sample	9.122	24131	2090151	0.0115	0.0022		
sp-22.D	C18:3n6	C19:0	Sample	9.122	19969	1884649	0.0106	ND		
sp-23.D	C18:3n6	C19:0	Sample	9.122	19468	1819204	0.0107	ND		
sp-24.D	C18:3n6	C19:0	Sample	9.122	20447	1859181	0.0110	ND		
qc-4.D	C18:3n6	C19:0	Sample	9.144	429355	2169029	0.1979	10.3285		
sp-25.D	C18:3n6	C19:0	Sample	9.126	21077	1815559	0.0116	0.0057		
sp-26.D	C18:3n6	C19:0	Sample	9.122	19043	1841988	0.0103	ND		
sp-27.D	C18:3n6	C19:0	Sample	9.122	22400	1938349	0.0116	0.0028		
sp-28.D	C18:3n6	C19:0	Sample	9.122	23459	1948544	0.0120	0.0296		
sp-29.D	C18:3n6	C19:0	Sample	9.122	19717	1770121	0.0111	ND		
sp-30.D	C18:3n6	C19:0	Sample	9.122	17907	1720523	0.0104	ND		
sp-31.D	C18:3n6	C19:0	Sample	9.122	17986	1756510	0.0102	ND		
sp-32.D	C18:3n6	C19:0	Sample	9.122	18964	1813710	0.0105	ND		
qc-5.D	C18:3n6	C19:0	Sample	9.140	392643	2038657	0.1926	10.0321		

C18:3n3

数据文件	化合物	ISTD	样品类型	RT	响应	ISTD 响应	响应比	最终浓度	预期的浓度	准确度
blank.D	C18:3n3	C19:0	Blank			1614233		ND		
std1.D	C18:3n3	C19:0	Calibration	9.451	4574	1476809	0.0031	0.1477	0.1563	94.5
std2.D	C18:3n3	C19:0	Calibration	9.456	10535	1765013	0.0060	0.2905	0.3125	93.0
std3.D	C18:3n3	C19:0	Calibration	9.451	24443	1945904	0.0126	0.6182	0.6250	98.9
std4.D	C18:3n3	C19:0	Calibration	9.451	50702	2060032	0.0246	1.2174	1.2500	97.4
std5.D	C18:3n3	C19:0	Calibration	9.451	117286	2159349	0.0543	2.6942	2.5000	107.8
std6.D	C18:3n3	C19:0	Calibration	9.451	263848	2537627	0.1040	5.1631	5.0000	103.3
std7.D	C18:3n3	C19:0	Calibration	9.455	558658	2635656	0.2120	10.5321	10.0000	105.3

C18:3n3

数据文件	化合物	ISTD	样品类型	RT	响应	ISTD 响应	响应比	最终浓度	预期的浓度	准确度
std8.D	C18:3n3	C19:0	Calibration	9.455	1222850	2927430	0.4177	20.7621	20.0000	103.8
std9.D	C18:3n3	C19:0	Calibration	9.460	2708947	3505157	0.7728	38.4184	40.0000	96.0
std10.D	C18:3n3	C19:0	Calibration	9.464	5142622	4076680	1.2615	62.7121	80.0000	78.4
qc-3.D	C18:3n3	C19:0	Sample	9.451	833295	2180317	0.3822	18.9956		
sp-17.D	C18:3n3	C19:0	Sample	9.455	3071567	1822377	1.6855	83.7927		
sp-18.D	C18:3n3	C19:0	Sample	9.451	1485309	1869407	0.7945	39.4967		
sp-19.D	C18:3n3	C19:0	Sample	9.451	793437	1809576	0.4385	21.7935		
sp-20.D	C18:3n3	C19:0	Sample	9.451	766186	1890097	0.4054	20.1480		
sp-21.D	C18:3n3	C19:0	Sample	9.451	1809209	2090151	0.8656	43.0293		
sp-22.D	C18:3n3	C19:0	Sample	9.451	1331090	1884649	0.7063	35.1088		
sp-23.D	C18:3n3	C19:0	Sample	9.451	1275508	1819204	0.7011	34.8530		
sp-24.D	C18:3n3	C19:0	Sample	9.446	812261	1859181	0.4369	21.7153		
qc-4.D	C18:3n3	C19:0	Sample	9.446	893811	2169029	0.4121	20.4816		
sp-25.D	C18:3n3	C19:0	Sample	9.460	5202524	1815559	2.8655	142.4627		
sp-26.D	C18:3n3	C19:0	Sample	9.451	1216822	1841988	0.6606	32.8378		
sp-27.D	C18:3n3	C19:0	Sample	9.455	3899922	1938349	2.0120	100.0261		
sp-28.D	C18:3n3	C19:0	Sample	9.451	2844212	1948544	1.4597	72.5656		
sp-29.D	C18:3n3	C19:0	Sample	9.446	1277163	1770121	0.7215	35.8661		
sp-30.D	C18:3n3	C19:0	Sample	9.446	423696	1720523	0.2463	12.2374		
sp-31.D	C18:3n3	C19:0	Sample	9.446	769785	1756510	0.4382	21.7826		
sp-32.D	C18:3n3	C19:0	Sample	9.446	749682	1813710	0.4133	20.5444		
qc-5.D	C18:3n3	C19:0	Sample	9.446	819004	2038657	0.4017	19.9674		

C20:0

数据文件	化合物	ISTD	样品类型	RT	响应	ISTD 响应	响应比	最终浓度	预期的浓度	准确度
blank.D	C20:0	C19:0	Blank	10.060	496	1614233	0.0003	0.0417		
std1.D	C20:0	C19:0	Calibration	10.060	18408	1476809	0.0125	0.3484	0.3125	111.5
std2.D	C20:0	C19:0	Calibration	10.065	39690	1765013	0.0225	0.6012	0.6250	96.2
std3.D	C20:0	C19:0	Calibration	10.060	90943	1945904	0.0467	1.2129	1.2500	97.0
std4.D	C20:0	C19:0	Calibration	10.060	189204	2060032	0.0918	2.3507	2.5000	94.0
std5.D	C20:0	C19:0	Calibration	10.064	437966	2159349	0.2028	5.1500	5.0000	103.0
std6.D	C20:0	C19:0	Calibration	10.060	992176	2537627	0.3910	9.8963	10.0000	99.0
std7.D	C20:0	C19:0	Calibration	10.064	2043689	2635656	0.7754	19.5929	20.0000	98.0
std8.D	C20:0	C19:0	Calibration	10.069	4700404	2927430	1.6056	40.5351	40.0000	101.3
std9.D	C20:0	C19:0	Calibration	10.082	10467244	3505157	2.9862	75.3597	80.0000	94.2
std10.D	C20:0	C19:0	Calibration	10.096	18731212	4076680	4.5947	115.9324	160.0000	72.5
qc-3.D	C20:0	C19:0	Sample	10.060	1902344	2180317	0.8725	22.0423		
sp-17.D	C20:0	C19:0	Sample	10.060	50887	1822377	0.0279	0.7383		
sp-18.D	C20:0	C19:0	Sample	10.056	30985	1869407	0.0166	0.4521		
sp-19.D	C20:0	C19:0	Sample	10.056	23915	1809576	0.0132	0.3673		
sp-20.D	C20:0	C19:0	Sample	10.056	45475	1890097	0.0241	0.6409		

C20:0

数据文件	化合物	ISTD	样品类型	RT	响应	ISTD 响应	响应比	最终浓度	预期的浓度	准确度
sp-21.D	C20:0	C19:0	Sample	10.056	33722	2090151	0.0161	0.4409		
sp-22.D	C20:0	C19:0	Sample	10.056	40714	1884649	0.0216	0.5789		
sp-23.D	C20:0	C19:0	Sample	10.056	39958	1819204	0.0220	0.5880		
sp-24.D	C20:0	C19:0	Sample	10.056	34102	1859181	0.0183	0.4967		
qc-4.D	C20:0	C19:0	Sample	10.056	1835193	2169029	0.8461	21.3760		
sp-25.D	C20:0	C19:0	Sample	10.056	27888	1815559	0.0154	0.4214		
sp-26.D	C20:0	C19:0	Sample	10.056	40645	1841988	0.0221	0.5906		
sp-27.D	C20:0	C19:0	Sample	10.056	33573	1938349	0.0173	0.4709		
sp-28.D	C20:0	C19:0	Sample	10.056	46732	1948544	0.0240	0.6389		
sp-29.D	C20:0	C19:0	Sample	10.056	35069	1770121	0.0198	0.5337		
sp-30.D	C20:0	C19:0	Sample	10.056	34597	1720523	0.0201	0.5412		
sp-31.D	C20:0	C19:0	Sample	10.056	32793	1756510	0.0187	0.5049		
sp-32.D	C20:0	C19:0	Sample	10.056	26946	1813710	0.0149	0.4087		
qc-5.D	C20:0	C19:0	Sample	10.056	1753502	2038657	0.8601	21.7300		

C20:1

数据文件	化合物	ISTD	样品类型	RT	响应	ISTD 响应	响应比	最终浓度	预期的浓度	准确度
blank.D	C20:1	C19:0	Blank			1614233		ND		
std1.D	C20:1	C19:0	Calibration	10.300	3434	1476809	0.0023	0.1832	0.1563	117.3
std2.D	C20:1	C19:0	Calibration	10.300	7385	1765013	0.0042	0.2978	0.3125	95.3
std3.D	C20:1	C19:0	Calibration	10.296	17812	1945904	0.0092	0.6040	0.6250	96.6
std4.D	C20:1	C19:0	Calibration	10.300	37218	2060032	0.0181	1.1533	1.2500	92.3
std5.D	C20:1	C19:0	Calibration	10.300	86339	2159349	0.0400	2.5039	2.5000	100.2
std6.D	C20:1	C19:0	Calibration	10.300	197112	2537627	0.0777	4.8266	5.0000	96.5
std7.D	C20:1	C19:0	Calibration	10.300	421746	2635656	0.1600	9.9008	10.0000	99.0
std8.D	C20:1	C19:0	Calibration	10.300	984696	2927430	0.3364	20.7685	20.0000	103.8
std9.D	C20:1	C19:0	Calibration	10.309	2250474	3505157	0.6420	39.6057	40.0000	99.0
std10.D	C20:1	C19:0	Calibration	10.322	4227381	4076680	1.0370	63.9424	80.0000	79.9
qc-3.D	C20:1	C19:0	Sample	10.296	427042	2180317	0.1959	12.1098		
sp-17.D	C20:1	C19:0	Sample	10.296	173478	1822377	0.0952	5.9062		
sp-18.D	C20:1	C19:0	Sample	10.291	126885	1869407	0.0679	4.2227		
sp-19.D	C20:1	C19:0	Sample	10.291	123141	1809576	0.0680	4.2335		
sp-20.D	C20:1	C19:0	Sample	10.296	161384	1890097	0.0854	5.3017		
sp-21.D	C20:1	C19:0	Sample	10.291	139426	2090151	0.0667	4.1507		
sp-22.D	C20:1	C19:0	Sample	10.296	168579	1884649	0.0894	5.5521		
sp-23.D	C20:1	C19:0	Sample	10.291	129511	1819204	0.0712	4.4270		
sp-24.D	C20:1	C19:0	Sample	10.291	136683	1859181	0.0735	4.5704		
qc-4.D	C20:1	C19:0	Sample	10.296	417234	2169029	0.1924	11.8940		
sp-25.D	C20:1	C19:0	Sample	10.292	65156	1815559	0.0359	2.2515		
sp-26.D	C20:1	C19:0	Sample	10.296	178140	1841988	0.0967	5.9997		
sp-27.D	C20:1	C19:0	Sample	10.291	137013	1938349	0.0707	4.3959		

C20:1

数据文件	化合物	ISTD	样品类型	RT	响应	ISTD 响应	响应比	最终浓度	预期的浓度	准确度
sp-28.D	C20:1	C19:0	Sample	10.292	149551	1948544	0.0768	4.7696		
sp-29.D	C20:1	C19:0	Sample	10.291	101616	1770121	0.0574	3.5776		
sp-30.D	C20:1	C19:0	Sample	10.291	91545	1720523	0.0532	3.3188		
sp-31.D	C20:1	C19:0	Sample	10.291	124683	1756510	0.0710	4.4142		
sp-32.D	C20:1	C19:0	Sample	10.291	136680	1813710	0.0754	4.6839		
qc-5.D	C20:1	C19:0	Sample	10.291	398394	2038657	0.1954	12.0826		

C20:2

数据文件	化合物	ISTD	样品类型	RT	响应	ISTD 响应	响应比	最终浓度	预期的浓度	准确度
blank.D	C20:2	C19:0	Blank			1614233		ND		
std1.D	C20:2	C19:0	Calibration	10.754	3149	1476809	0.0021	0.1852	0.1563	118.5
std2.D	C20:2	C19:0	Calibration	10.758	7375	1765013	0.0042	0.3102	0.3125	99.3
std3.D	C20:2	C19:0	Calibration	10.754	17325	1945904	0.0089	0.5989	0.6250	95.8
std4.D	C20:2	C19:0	Calibration	10.758	36144	2060032	0.0175	1.1270	1.2500	90.2
std5.D	C20:2	C19:0	Calibration	10.758	85641	2159349	0.0397	2.4783	2.5000	99.1
std6.D	C20:2	C19:0	Calibration	10.758	197958	2537627	0.0780	4.8217	5.0000	96.4
std7.D	C20:2	C19:0	Calibration	10.758	419918	2635656	0.1593	9.7903	10.0000	97.9
std8.D	C20:2	C19:0	Calibration	10.758	983181	2927430	0.3359	20.5772	20.0000	102.9
std9.D	C20:2	C19:0	Calibration	10.767	2288759	3505157	0.6530	39.9549	40.0000	99.9
std10.D	C20:2	C19:0	Calibration	10.772	4407678	4076680	1.0812	66.1218	80.0000	82.7
qc-3.D	C20:2	C19:0	Sample	10.754	385225	2180317	0.1767	10.8512		
sp-17.D	C20:2	C19:0	Sample	10.754	55325	1822377	0.0304	1.9100		
sp-18.D	C20:2	C19:0	Sample	10.749	34384	1869407	0.0184	1.1788		
sp-19.D	C20:2	C19:0	Sample	10.749	23491	1809576	0.0130	0.8481		
sp-20.D	C20:2	C19:0	Sample	10.749	24920	1890097	0.0132	0.8605		
sp-21.D	C20:2	C19:0	Sample	10.749	49053	2090151	0.0235	1.4889		
sp-22.D	C20:2	C19:0	Sample	10.754	42261	1884649	0.0224	1.4251		
sp-23.D	C20:2	C19:0	Sample	10.749	37344	1819204	0.0205	1.3092		
sp-24.D	C20:2	C19:0	Sample	10.749	57616	1859181	0.0310	1.9485		
qc-4.D	C20:2	C19:0	Sample	10.749	377337	2169029	0.1740	10.6852		
sp-25.D	C20:2	C19:0	Sample	10.750	36672	1815559	0.0202	1.2891		
sp-26.D	C20:2	C19:0	Sample	10.749	33485	1841988	0.0182	1.1657		
sp-27.D	C20:2	C19:0	Sample	10.749	29862	1938349	0.0154	0.9962		
sp-28.D	C20:2	C19:0	Sample	10.750	60892	1948544	0.0313	1.9644		
sp-29.D	C20:2	C19:0	Sample	10.749	37759	1770121	0.0213	1.3583		
sp-30.D	C20:2	C19:0	Sample	10.749	16295	1720523	0.0095	0.6336		
sp-31.D	C20:2	C19:0	Sample	10.749	31616	1756510	0.0180	1.1547		
sp-32.D	C20:2	C19:0	Sample	10.749	36377	1813710	0.0201	1.2805		
qc-5.D	C20:2	C19:0	Sample	10.749	357368	2038657	0.1753	10.7664		

C21:0

数据文件	化合物	ISTD	样品类型	RT	响应	ISTD 响应	响应比	最终浓度	预期的浓度	准确度
blank.D	C21:0	C19:0	Blank	10.967	1672	1614233	0.0010	0.0287		
std1.D	C21:0	C19:0	Calibration	10.967	10026	1476809	0.0068	0.1870	0.1563	119.6
std2.D	C21:0	C19:0	Calibration	10.967	19624	1765013	0.0111	0.3060	0.3125	97.9
std3.D	C21:0	C19:0	Calibration	10.967	41873	1945904	0.0215	0.5922	0.6250	94.7
std4.D	C21:0	C19:0	Calibration	10.967	86000	2060032	0.0417	1.1486	1.2500	91.9
std5.D	C21:0	C19:0	Calibration	10.967	196118	2159349	0.0908	2.4987	2.5000	99.9
std6.D	C21:0	C19:0	Calibration	10.967	443818	2537627	0.1749	4.8115	5.0000	96.2
std7.D	C21:0	C19:0	Calibration	10.967	929280	2635656	0.3526	9.6996	10.0000	97.0
std8.D	C21:0	C19:0	Calibration	10.972	2175424	2927430	0.7431	20.4432	20.0000	102.2
std9.D	C21:0	C19:0	Calibration	10.981	5116545	3505157	1.4597	40.1569	40.0000	100.4
std10.D	C21:0	C19:0	Calibration	10.994	9619014	4076680	2.3595	64.9103	80.0000	81.1
qc-3.D	C21:0	C19:0	Sample	10.963	928336	2180317	0.4258	11.7133		
sp-17.D	C21:0	C19:0	Sample	10.963	2152	1822377	0.0012	0.0327		
sp-18.D	C21:0	C19:0	Sample	10.958	1849	1869407	0.0010	0.0274		
sp-19.D	C21:0	C19:0	Sample	10.958	2240	1809576	0.0012	0.0342		
sp-20.D	C21:0	C19:0	Sample	10.963	2292	1890097	0.0012	0.0335		
sp-21.D	C21:0	C19:0	Sample	10.963	2222	2090151	0.0011	0.0294		
sp-22.D	C21:0	C19:0	Sample	10.963	2159	1884649	0.0011	0.0317		
sp-23.D	C21:0	C19:0	Sample	10.958	1934	1819204	0.0011	0.0294		
sp-24.D	C21:0	C19:0	Sample	10.958	1953	1859181	0.0011	0.0291		
qc-4.D	C21:0	C19:0	Sample	10.963	890100	2169029	0.4104	11.2893		
sp-25.D	C21:0	C19:0	Sample	10.958	2315	1815559	0.0013	0.0353		
sp-26.D	C21:0	C19:0	Sample	10.963	2159	1841988	0.0012	0.0324		
sp-27.D	C21:0	C19:0	Sample	10.958	2316	1938349	0.0012	0.0331		
sp-28.D	C21:0	C19:0	Sample	10.959	2435	1948544	0.0012	0.0346		
sp-29.D	C21:0	C19:0	Sample	10.958	2254	1770121	0.0013	0.0352		
sp-30.D	C21:0	C19:0	Sample	10.958	2094	1720523	0.0012	0.0337		
sp-31.D	C21:0	C19:0	Sample	10.958	2076	1756510	0.0012	0.0327		
sp-32.D	C21:0	C19:0	Sample	10.958	2060	1813710	0.0011	0.0314		
qc-5.D	C21:0	C19:0	Sample	10.963	855472	2038657	0.4196	11.5440		

C20:3n6

数据文件	化合物	ISTD	样品类型	RT	响应	ISTD 响应	响应比	最终浓度	预期的浓度	准确度
blank.D	C20:3n6	C19:0	Blank			1614233		ND		
std1.D	C20:3n6	C19:0	Calibration	11.007	3263	1476809	0.0022	0.1837	0.1563	117.6
std2.D	C20:3n6	C19:0	Calibration	11.007	7286	1765013	0.0041	0.3049	0.3125	97.6
std3.D	C20:3n6	C19:0	Calibration	11.007	17167	1945904	0.0088	0.6014	0.6250	96.2
std4.D	C20:3n6	C19:0	Calibration	11.007	35762	2060032	0.0174	1.1406	1.2500	91.2
std5.D	C20:3n6	C19:0	Calibration	11.007	83881	2159349	0.0388	2.4975	2.5000	99.9
std6.D	C20:3n6	C19:0	Calibration	11.007	193575	2537627	0.0763	4.8619	5.0000	97.2
std7.D	C20:3n6	C19:0	Calibration	11.011	405416	2635656	0.1538	9.7588	10.0000	97.6

C20:3n6

数据文件	化合物	ISTD	样品类型	RT	响应	ISTD 响应	响应比	最终浓度	预期的浓度	准确度
std8.D	C20:3n6	C19:0	Calibration	11.012	950912	2927430	0.3248	20.5590	20.0000	102.8
std9.D	C20:3n6	C19:0	Calibration	11.021	2213995	3505157	0.6316	39.9359	40.0000	99.8
std10.D	C20:3n6	C19:0	Calibration	11.029	4273562	4076680	1.0483	66.2501	80.0000	82.8
qc-3.D	C20:3n6	C19:0	Sample	11.003	352362	2180317	0.1616	10.2508		
sp-17.D	C20:3n6	C19:0	Sample	11.003	8357	1822377	0.0046	0.3338		
sp-18.D	C20:3n6	C19:0	Sample	11.003	3114	1869407	0.0017	0.1494		
sp-19.D	C20:3n6	C19:0	Sample	11.003	2372	1809576	0.0013	0.1270		
sp-20.D	C20:3n6	C19:0	Sample	11.003	2046	1890097	0.0011	0.1126		
sp-21.D	C20:3n6	C19:0	Sample	11.003	5214	2090151	0.0025	0.2018		
sp-22.D	C20:3n6	C19:0	Sample	11.003	3427	1884649	0.0018	0.1591		
sp-23.D	C20:3n6	C19:0	Sample	11.003	3077	1819204	0.0017	0.1510		
sp-24.D	C20:3n6	C19:0	Sample	11.003	4937	1859181	0.0027	0.2119		
qc-4.D	C20:3n6	C19:0	Sample	11.003	355934	2169029	0.1641	10.4080		
sp-25.D	C20:3n6	C19:0	Sample	10.999	2411	1815559	0.0013	0.1281		
sp-26.D	C20:3n6	C19:0	Sample	11.003	2744	1841988	0.0015	0.1383		
sp-27.D	C20:3n6	C19:0	Sample	11.003	2913	1938349	0.0015	0.1391		
sp-28.D	C20:3n6	C19:0	Sample	10.999	4691	1948544	0.0024	0.1963		
sp-29.D	C20:3n6	C19:0	Sample	11.003	2813	1770121	0.0016	0.1446		
sp-30.D	C20:3n6	C19:0	Sample	11.003	1420	1720523	0.0008	0.0963		
sp-31.D	C20:3n6	C19:0	Sample	10.998	1995	1756510	0.0011	0.1159		
sp-32.D	C20:3n6	C19:0	Sample	10.998	2806	1813710	0.0015	0.1419		
qc-5.D	C20:3n6	C19:0	Sample	11.003	335624	2038657	0.1646	10.4415		

C20:4n6

数据文件	化合物	ISTD	样品类型	RT	响应	ISTD 响应	响应比	最终浓度	预期的浓度	准确度
blank.D	C20:4n6	C19:0	Blank			1614233		ND		
std1.D	C20:4n6	C19:0	Calibration	11.163	3306	1476809	0.0022	0.1748	0.1563	111.9
std2.D	C20:4n6	C19:0	Calibration	11.163	7641	1765013	0.0043	0.3052	0.3125	97.7
std3.D	C20:4n6	C19:0	Calibration	11.163	17658	1945904	0.0091	0.6011	0.6250	96.2
std4.D	C20:4n6	C19:0	Calibration	11.163	37388	2060032	0.0181	1.1670	1.2500	93.4
std5.D	C20:4n6	C19:0	Calibration	11.163	85894	2159349	0.0398	2.5156	2.5000	100.6
std6.D	C20:4n6	C19:0	Calibration	11.163	199708	2537627	0.0787	4.9426	5.0000	98.9
std7.D	C20:4n6	C19:0	Calibration	11.163	416629	2635656	0.1581	9.8921	10.0000	98.9
std8.D	C20:4n6	C19:0	Calibration	11.163	972850	2927430	0.3323	20.7575	20.0000	103.8
std9.D	C20:4n6	C19:0	Calibration	11.172	2217714	3505157	0.6327	39.4878	40.0000	98.7
std10.D	C20:4n6	C19:0	Calibration	11.176	4247049	4076680	1.0418	64.9970	80.0000	81.2
qc-3.D	C20:4n6	C19:0	Sample	11.158	377529	2180317	0.1732	10.8324		
sp-17.D	C20:4n6	C19:0	Sample	11.158	5289	1822377	0.0029	0.2162		
sp-18.D	C20:4n6	C19:0	Sample	11.154	3139	1869407	0.0017	0.1400		
sp-19.D	C20:4n6	C19:0	Sample	11.154	3917	1809576	0.0022	0.1702		
sp-20.D	C20:4n6	C19:0	Sample	11.158	3106	1890097	0.0016	0.1377		

C20:4n6

数据文件	化合物	ISTD	样品类型	RT	响应	ISTD 响应	响应比	最终浓度	预期的浓度	准确度
sp-21.D	C20:4n6	C19:0	Sample	11.154	5563	2090151	0.0027	0.2012		
sp-22.D	C20:4n6	C19:0	Sample	11.159	4404	1884649	0.0023	0.1810		
sp-23.D	C20:4n6	C19:0	Sample	11.154	4881	1819204	0.0027	0.2026		
sp-24.D	C20:4n6	C19:0	Sample	11.154	4258	1859181	0.0023	0.1781		
qc-4.D	C20:4n6	C19:0	Sample	11.154	384809	2169029	0.1774	11.0978		
sp-25.D	C20:4n6	C19:0	Sample	11.154	4028	1815559	0.0022	0.1736		
sp-26.D	C20:4n6	C19:0	Sample	11.154	3610	1841988	0.0020	0.1575		
sp-27.D	C20:4n6	C19:0	Sample	11.154	3602	1938349	0.0019	0.1511		
sp-28.D	C20:4n6	C19:0	Sample	11.154	6793	1948544	0.0035	0.2526		
sp-29.D	C20:4n6	C19:0	Sample	11.154	3335	1770121	0.0019	0.1527		
sp-30.D	C20:4n6	C19:0	Sample	11.154	2501	1720523	0.0015	0.1259		
sp-31.D	C20:4n6	C19:0	Sample	11.154	2934	1756510	0.0017	0.1394		
sp-32.D	C20:4n6	C19:0	Sample	11.154	2977	1813710	0.0016	0.1376		
qc-5.D	C20:4n6	C19:0	Sample	11.154	361306	2038657	0.1772	11.0864		

C20:3n3

数据文件	化合物	ISTD	样品类型	RT	响应	ISTD 响应	响应比	最终浓度	预期的浓度	准确度
blank.D	C20:3n3	C19:0	Blank			1614233		ND		
std1.D	C20:3n3	C19:0	Calibration	11.305	3891	1476809	0.0026	0.1888	0.1563	120.8
std2.D	C20:3n3	C19:0	Calibration	11.305	8777	1765013	0.0050	0.3110	0.3125	99.5
std3.D	C20:3n3	C19:0	Calibration	11.305	20073	1945904	0.0103	0.5904	0.6250	94.5
std4.D	C20:3n3	C19:0	Calibration	11.305	42681	2060032	0.0207	1.1342	1.2500	90.7
std5.D	C20:3n3	C19:0	Calibration	11.305	99056	2159349	0.0459	2.4493	2.5000	98.0
std6.D	C20:3n3	C19:0	Calibration	11.305	231764	2537627	0.0913	4.8260	5.0000	96.5
std7.D	C20:3n3	C19:0	Calibration	11.305	484842	2635656	0.1840	9.6685	10.0000	96.7
std8.D	C20:3n3	C19:0	Calibration	11.305	1152681	2927430	0.3938	20.6370	20.0000	103.2
std9.D	C20:3n3	C19:0	Calibration	11.314	2680917	3505157	0.7648	40.0386	40.0000	100.1
std10.D	C20:3n3	C19:0	Calibration	11.318	5157652	4076680	1.2652	66.1956	80.0000	82.7
qc-3.D	C20:3n3	C19:0	Sample	11.301	437705	2180317	0.2008	10.5467		
sp-17.D	C20:3n3	C19:0	Sample	11.301	87414	1822377	0.0480	2.5588		
sp-18.D	C20:3n3	C19:0	Sample	11.296	49892	1869407	0.0267	1.4464		
sp-19.D	C20:3n3	C19:0	Sample	11.296	24401	1809576	0.0135	0.7560		
sp-20.D	C20:3n3	C19:0	Sample	11.301	25166	1890097	0.0133	0.7471		
sp-21.D	C20:3n3	C19:0	Sample	11.296	37869	2090151	0.0181	0.9983		
sp-22.D	C20:3n3	C19:0	Sample	11.301	39794	1884649	0.0211	1.1550		
sp-23.D	C20:3n3	C19:0	Sample	11.296	40279	1819204	0.0221	1.2086		
sp-24.D	C20:3n3	C19:0	Sample	11.296	27756	1859181	0.0149	0.8316		
qc-4.D	C20:3n3	C19:0	Sample	11.301	433169	2169029	0.1997	10.4920		
sp-25.D	C20:3n3	C19:0	Sample	11.296	93163	1815559	0.0513	2.7338		
sp-26.D	C20:3n3	C19:0	Sample	11.301	35571	1841988	0.0193	1.0606		
sp-27.D	C20:3n3	C19:0	Sample	11.296	88100	1938349	0.0455	2.4273		

C20:3n3

数据文件	化合物	ISTD	样品类型	RT	响应	ISTD 响应	响应比	最终浓度	预期的浓度	准确度
sp-28.D	C20:3n3	C19:0	Sample	11.296	60467	1948544	0.0310	1.6734		
sp-29.D	C20:3n3	C19:0	Sample	11.296	32372	1770121	0.0183	1.0072		
sp-30.D	C20:3n3	C19:0	Sample	11.296	10797	1720523	0.0063	0.3791		
sp-31.D	C20:3n3	C19:0	Sample	11.296	20985	1756510	0.0119	0.6756		
sp-32.D	C20:3n3	C19:0	Sample	11.296	24690	1813710	0.0136	0.7627		
qc-5.D	C20:3n3	C19:0	Sample	11.296	412218	2038657	0.2022	10.6224		

C20:5n3

数据文件	化合物	ISTD	样品类型	RT	响应	ISTD 响应	响应比	最终浓度	预期的浓度	准确度
blank.D	C20:5n3	C19:0	Blank	11.879	0	1614233	0.0000	ND		
std1.D	C20:5n3	C19:0	Calibration	11.705	3469	1476809	0.0023	0.1650	0.1563	105.6
std2.D	C20:5n3	C19:0	Calibration	11.706	8699	1765013	0.0049	0.3076	0.3125	98.4
std3.D	C20:5n3	C19:0	Calibration	11.705	20490	1945904	0.0105	0.6172	0.6250	98.7
std4.D	C20:5n3	C19:0	Calibration	11.701	42763	2060032	0.0208	1.1825	1.2500	94.6
std5.D	C20:5n3	C19:0	Calibration	11.701	98917	2159349	0.0458	2.5669	2.5000	102.7
std6.D	C20:5n3	C19:0	Calibration	11.701	224693	2537627	0.0885	4.9288	5.0000	98.6
std7.D	C20:5n3	C19:0	Calibration	11.705	473008	2635656	0.1795	9.9537	10.0000	99.5
std8.D	C20:5n3	C19:0	Calibration	11.705	1089649	2927430	0.3722	20.6068	20.0000	103.0
std9.D	C20:5n3	C19:0	Calibration	11.710	2503902	3505157	0.7143	39.5152	40.0000	98.8
std10.D	C20:5n3	C19:0	Calibration	11.714	4794968	4076680	1.1762	65.0402	80.0000	81.3
qc-3.D	C20:5n3	C19:0	Sample	11.696	397486	2180317	0.1823	10.1108		
sp-17.D	C20:5n3	C19:0	Sample	11.696	1805	1822377	0.0010	0.0900		
sp-18.D	C20:5n3	C19:0	Sample	11.696	890	1869407	0.0005	0.0615		
sp-19.D	C20:5n3	C19:0	Sample	11.697	807	1809576	0.0004	0.0598		
sp-20.D	C20:5n3	C19:0	Sample	11.696	703	1890097	0.0004	0.0558		
sp-21.D	C20:5n3	C19:0	Sample	11.697	1181	2090151	0.0006	0.0664		
sp-22.D	C20:5n3	C19:0	Sample	11.697	999	1884649	0.0005	0.0645		
sp-23.D	C20:5n3	C19:0	Sample	11.692	1308	1819204	0.0007	0.0749		
sp-24.D	C20:5n3	C19:0	Sample	11.696	587	1859181	0.0003	0.0527		
qc-4.D	C20:5n3	C19:0	Sample	11.696	392915	2169029	0.1811	10.0467		
sp-25.D	C20:5n3	C19:0	Sample	11.697	2511	1815559	0.0014	0.1117		
sp-26.D	C20:5n3	C19:0	Sample	11.696	663	1841988	0.0004	0.0551		
sp-27.D	C20:5n3	C19:0	Sample	11.697	1794	1938349	0.0009	0.0863		
sp-28.D	C20:5n3	C19:0	Sample	11.692	1758	1948544	0.0009	0.0851		
sp-29.D	C20:5n3	C19:0	Sample	11.692	829	1770121	0.0005	0.0611		
sp-30.D	C20:5n3	C19:0	Sample	11.696	433	1720523	0.0003	0.0491		
sp-31.D	C20:5n3	C19:0	Sample	11.696	549	1756510	0.0003	0.0525		
sp-32.D	C20:5n3	C19:0	Sample	11.692	553	1813710	0.0003	0.0521		
qc-5.D	C20:5n3	C19:0	Sample	11.696	369956	2038657	0.1815	10.0646		

C22:0

数据文件	化合物	ISTD	样品类型	RT	响应	ISTD 响应	响应比	最终浓度	预期的浓度	准确度
blank.D	C22:0	C19:0	Blank			1614233		ND		
std1.D	C22:0	C19:0	Calibration	11.825	15503	1476809	0.0105	0.3602	0.3125	115.3
std2.D	C22:0	C19:0	Calibration	11.830	31532	1765013	0.0179	0.5987	0.6250	95.8
std3.D	C22:0	C19:0	Calibration	11.825	69692	1945904	0.0358	1.1799	1.2500	94.4
std4.D	C22:0	C19:0	Calibration	11.825	150469	2060032	0.0730	2.3852	2.5000	95.4
std5.D	C22:0	C19:0	Calibration	11.825	339739	2159349	0.1573	5.1144	5.0000	102.3
std6.D	C22:0	C19:0	Calibration	11.825	757374	2537627	0.2985	9.6836	10.0000	96.8
std7.D	C22:0	C19:0	Calibration	11.829	1597788	2635656	0.6062	19.6482	20.0000	98.2
std8.D	C22:0	C19:0	Calibration	11.830	3679650	2927430	1.2570	40.7174	40.0000	101.8
std9.D	C22:0	C19:0	Calibration	11.839	8406927	3505157	2.3984	77.6760	80.0000	97.1
std10.D	C22:0	C19:0	Calibration	11.856	15354227	4076680	3.7664	121.9655	160.0000	76.2
qc-3.D	C22:0	C19:0	Sample	11.825	1761878	2180317	0.8081	26.1840		
sp-17.D	C22:0	C19:0	Sample	11.821	5073	1822377	0.0028	0.1104		
sp-18.D	C22:0	C19:0	Sample	11.821	2218	1869407	0.0012	0.0587		
sp-19.D	C22:0	C19:0	Sample	11.821	1977	1809576	0.0011	0.0557		
sp-20.D	C22:0	C19:0	Sample	11.821	5879	1890097	0.0031	0.1210		
sp-21.D	C22:0	C19:0	Sample	11.821	3786	2090151	0.0018	0.0789		
sp-22.D	C22:0	C19:0	Sample	11.821	4882	1884649	0.0026	0.1041		
sp-23.D	C22:0	C19:0	Sample	11.817	3793	1819204	0.0021	0.0878		
sp-24.D	C22:0	C19:0	Sample	11.816	3538	1859181	0.0019	0.0819		
qc-4.D	C22:0	C19:0	Sample	11.821	1687522	2169029	0.7780	25.2102		
sp-25.D	C22:0	C19:0	Sample	11.821	2392	1815559	0.0013	0.0629		
sp-26.D	C22:0	C19:0	Sample	11.821	4275	1841988	0.0023	0.0954		
sp-27.D	C22:0	C19:0	Sample	11.817	2133	1938349	0.0011	0.0559		
sp-28.D	C22:0	C19:0	Sample	11.821	3822	1948544	0.0020	0.0838		
sp-29.D	C22:0	C19:0	Sample	11.821	3875	1770121	0.0022	0.0912		
sp-30.D	C22:0	C19:0	Sample	11.816	4086	1720523	0.0024	0.0972		
sp-31.D	C22:0	C19:0	Sample	11.816	3858	1756510	0.0022	0.0914		
sp-32.D	C22:0	C19:0	Sample	11.816	2472	1813710	0.0014	0.0644		
qc-5.D	C22:0	C19:0	Sample	11.821	1627107	2038657	0.7981	25.8617		

C22:1n9

数据文件	化合物	ISTD	样品类型	RT	响应	ISTD 响应	响应比	最终浓度	预期的浓度	准确度
blank.D	C22:1n9	C19:0	Blank	12.132	0	1614233	0.0000	ND		
std1.D	C22:1n9	C19:0	Calibration	12.052	4762	1476809	0.0032	0.2240	0.1563	143.3
std2.D	C22:1n9	C19:0	Calibration	12.052	6620	1765013	0.0038	0.2629	0.3125	84.1
std3.D	C22:1n9	C19:0	Calibration	12.052	14742	1945904	0.0076	0.5461	0.6250	87.4
std4.D	C22:1n9	C19:0	Calibration	12.052	32144	2060032	0.0156	1.1404	1.2500	91.2
std5.D	C22:1n9	C19:0	Calibration	12.057	72217	2159349	0.0334	2.4611	2.5000	98.4
std6.D	C22:1n9	C19:0	Calibration	12.056	162904	2537627	0.0642	4.7377	5.0000	94.8
std7.D	C22:1n9	C19:0	Calibration	12.056	345729	2635656	0.1312	9.6962	10.0000	97.0

C22:1n9

数据文件	化合物	ISTD	样品类型	RT	响应	ISTD 响应	响应比	最终浓度	预期的浓度	准确度
std8.D	C22:1n9	C19:0	Calibration	12.057	820737	2927430	0.2804	20.7408	20.0000	103.7
std9.D	C22:1n9	C19:0	Calibration	12.061	1896201	3505157	0.5410	40.0346	40.0000	100.1
std10.D	C22:1n9	C19:0	Calibration	12.070	3624168	4076680	0.8890	65.7995	80.0000	82.2
qc-3.D	C22:1n9	C19:0	Sample	12.048	377018	2180317	0.1729	12.7867		
sp-17.D	C22:1n9	C19:0	Sample	12.048	4192	1822377	0.0023	0.1555		
sp-18.D	C22:1n9	C19:0	Sample	12.048	3323	1869407	0.0018	0.1168		
sp-19.D	C22:1n9	C19:0	Sample	12.048	5136	1809576	0.0028	0.1953		
sp-20.D	C22:1n9	C19:0	Sample	12.048	5521	1890097	0.0029	0.2015		
sp-21.D	C22:1n9	C19:0	Sample	12.048	4454	2090151	0.0021	0.1430		
sp-22.D	C22:1n9	C19:0	Sample	12.048	4980	1884649	0.0026	0.1808		
sp-23.D	C22:1n9	C19:0	Sample	12.048	3545	1819204	0.0019	0.1295		
sp-24.D	C22:1n9	C19:0	Sample	12.048	4670	1859181	0.0025	0.1712		
qc-4.D	C22:1n9	C19:0	Sample	12.048	353032	2169029	0.1628	12.0347		
sp-25.D	C22:1n9	C19:0	Sample	12.043	1475	1815559	0.0008	0.0454		
sp-26.D	C22:1n9	C19:0	Sample	12.048	4960	1841988	0.0027	0.1846		
sp-27.D	C22:1n9	C19:0	Sample	12.043	2747	1938349	0.0014	0.0901		
sp-28.D	C22:1n9	C19:0	Sample	12.048	3902	1948544	0.0020	0.1335		
sp-29.D	C22:1n9	C19:0	Sample	12.043	3320	1770121	0.0019	0.1241		
sp-30.D	C22:1n9	C19:0	Sample	12.048	2339	1720523	0.0014	0.0859		
sp-31.D	C22:1n9	C19:0	Sample	12.043	3908	1756510	0.0022	0.1499		
sp-32.D	C22:1n9	C19:0	Sample	12.048	3618	1813710	0.0020	0.1329		
qc-5.D	C22:1n9	C19:0	Sample	12.048	343969	2038657	0.1687	12.4761		

C22:2n6

数据文件	化合物	ISTD	样品类型	RT	响应	ISTD 响应	响应比	最终浓度	预期的浓度	准确度
blank.D	C22:2n6	C19:0	Blank	12.808	0	1614233	0.0000	ND		
std1.D	C22:2n6	C19:0	Calibration	12.479	2958	1476809	0.0020	0.1953	0.1563	125.0
std2.D	C22:2n6	C19:0	Calibration	12.479	6142	1765013	0.0035	0.3119	0.3125	99.8
std3.D	C22:2n6	C19:0	Calibration	12.479	13309	1945904	0.0068	0.5774	0.6250	92.4
std4.D	C22:2n6	C19:0	Calibration	12.479	28462	2060032	0.0138	1.1288	1.2500	90.3
std5.D	C22:2n6	C19:0	Calibration	12.479	64810	2159349	0.0300	2.4088	2.5000	96.4
std6.D	C22:2n6	C19:0	Calibration	12.479	151778	2537627	0.0598	4.7636	5.0000	95.3
std7.D	C22:2n6	C19:0	Calibration	12.479	322970	2635656	0.1225	9.7208	10.0000	97.2
std8.D	C22:2n6	C19:0	Calibration	12.479	766807	2927430	0.2619	20.7371	20.0000	103.7
std9.D	C22:2n6	C19:0	Calibration	12.484	1830434	3505157	0.5222	41.3056	40.0000	103.3
std10.D	C22:2n6	C19:0	Calibration	12.492	3557120	4076680	0.8726	68.9920	80.0000	86.2
qc-3.D	C22:2n6	C19:0	Sample	12.475	342315	2180317	0.1570	12.4443		
sp-17.D	C22:2n6	C19:0	Sample	12.470	820	1822377	0.0004	0.0725		
sp-18.D	C22:2n6	C19:0	Sample	12.475	615	1869407	0.0003	0.0629		
sp-19.D	C22:2n6	C19:0	Sample	12.475	532	1809576	0.0003	0.0602		
sp-20.D	C22:2n6	C19:0	Sample	12.475	650	1890097	0.0003	0.0641		

C22:2n6

数据文件	化合物	ISTD	样品类型	RT	响应	ISTD 响应	响应比	最终浓度	预期的浓度	准确度
sp-21.D	C22:2n6	C19:0	Sample	12.475	726	2090151	0.0003	0.0644		
sp-22.D	C22:2n6	C19:0	Sample	12.470	703	1884649	0.0004	0.0664		
sp-23.D	C22:2n6	C19:0	Sample	12.470	610	1819204	0.0003	0.0634		
sp-24.D	C22:2n6	C19:0	Sample	12.470	925	1859181	0.0005	0.0763		
qc-4.D	C22:2n6	C19:0	Sample	12.475	324202	2169029	0.1495	11.8490		
sp-25.D	C22:2n6	C19:0	Sample	12.470	726	1815559	0.0004	0.0685		
sp-26.D	C22:2n6	C19:0	Sample	12.470	697	1841988	0.0004	0.0669		
sp-27.D	C22:2n6	C19:0	Sample	12.470	599	1938349	0.0003	0.0614		
sp-28.D	C22:2n6	C19:0	Sample	12.470	750	1948544	0.0004	0.0674		
sp-29.D	C22:2n6	C19:0	Sample	12.474	606	1770121	0.0003	0.0640		
sp-30.D	C22:2n6	C19:0	Sample	12.479	318	1720523	0.0002	0.0516		
sp-31.D	C22:2n6	C19:0	Sample	12.470	549	1756510	0.0003	0.0617		
sp-32.D	C22:2n6	C19:0	Sample	12.470	594	1813710	0.0003	0.0628		
qc-5.D	C22:2n6	C19:0	Sample	12.470	315898	2038657	0.1550	12.2824		

C23:0

数据文件	化合物	ISTD	样品类型	RT	响应	ISTD 响应	响应比	最终浓度	预期的浓度	准确度
blank.D	C23:0	C19:0	Blank	12.639	0	1614233	0.0000	ND		
std1.D	C23:0	C19:0	Calibration	12.639	7416	1476809	0.0050	0.1736	0.1563	111.1
std2.D	C23:0	C19:0	Calibration	12.639	14948	1765013	0.0085	0.3068	0.3125	98.2
std3.D	C23:0	C19:0	Calibration	12.639	30777	1945904	0.0158	0.5905	0.6250	94.5
std4.D	C23:0	C19:0	Calibration	12.639	65099	2060032	0.0316	1.2001	1.2500	96.0
std5.D	C23:0	C19:0	Calibration	12.639	143829	2159349	0.0666	2.5521	2.5000	102.1
std6.D	C23:0	C19:0	Calibration	12.639	317103	2537627	0.1250	4.8057	5.0000	96.1
std7.D	C23:0	C19:0	Calibration	12.639	685843	2635656	0.2602	10.0294	10.0000	100.3
std8.D	C23:0	C19:0	Calibration	12.639	1556318	2927430	0.5316	20.5117	20.0000	102.6
std9.D	C23:0	C19:0	Calibration	12.644	3602567	3505157	1.0278	39.6737	40.0000	99.2
std10.D	C23:0	C19:0	Calibration	12.652	6976080	4076680	1.7112	66.0681	80.0000	82.6
qc-3.D	C23:0	C19:0	Sample	12.635	798063	2180317	0.3660	14.1161		
sp-17.D	C23:0	C19:0	Sample	12.755	0	1822377	0.0000	ND		
sp-18.D	C23:0	C19:0	Sample	12.786	0	1869407	0.0000	ND		
sp-19.D	C23:0	C19:0	Sample	12.768	0	1809576	0.0000	ND		
sp-20.D	C23:0	C19:0	Sample	12.630	0	1890097	0.0000	ND		
sp-21.D	C23:0	C19:0	Sample	12.786	0	2090151	0.0000	ND		
sp-22.D	C23:0	C19:0	Sample	12.782	0	1884649	0.0000	ND		
sp-23.D	C23:0	C19:0	Sample	12.621	0	1819204	0.0000	ND		
sp-24.D	C23:0	C19:0	Sample	12.626	0	1859181	0.0000	ND		
qc-4.D	C23:0	C19:0	Sample	12.635	769809	2169029	0.3549	13.6865		
sp-25.D	C23:0	C19:0	Sample	12.755	0	1815559	0.0000	ND		
sp-26.D	C23:0	C19:0	Sample	12.786	0	1841988	0.0000	ND		
sp-27.D	C23:0	C19:0	Sample	12.746	0	1938349	0.0000	ND		

C23:0

数据文件	化合物	ISTD	样品类型	RT	响应	ISTD 响应	响应比	最终浓度	预期的浓度	准确度
sp-28.D	C23:0	C19:0	Sample	12.751	0	1948544	0.0000	ND		
sp-29.D	C23:0	C19:0	Sample	12.772	0	1770121	0.0000	ND		
sp-30.D	C23:0	C19:0	Sample	12.755	0	1720523	0.0000	ND		
sp-31.D	C23:0	C19:0	Sample	12.777	0	1756510	0.0000	ND		
sp-32.D	C23:0	C19:0	Sample	12.781	0	1813710	0.0000	ND		
qc-5.D	C23:0	C19:0	Sample	12.630	751553	2038657	0.3687	14.2173		

C24:0

数据文件	化合物	ISTD	样品类型	RT	响应	ISTD 响应	响应比	最终浓度	预期的浓度	准确度
blank.D	C24:0	C19:0	Blank	13.417	0	1614233	0.0000	ND		
std1.D	C24:0	C19:0	Calibration	13.404	10067	1476809	0.0068	0.3137	0.3125	100.4
std2.D	C24:0	C19:0	Calibration	13.404	24127	1765013	0.0137	0.6227	0.6250	99.6
std3.D	C24:0	C19:0	Calibration	13.404	49962	1945904	0.0257	1.1641	1.2500	93.1
std4.D	C24:0	C19:0	Calibration	13.404	112375	2060032	0.0546	2.4661	2.5000	98.6
std5.D	C24:0	C19:0	Calibration	13.404	250237	2159349	0.1159	5.2319	5.0000	104.6
std6.D	C24:0	C19:0	Calibration	13.404	553595	2537627	0.2182	9.8434	10.0000	98.4
std7.D	C24:0	C19:0	Calibration	13.404	1216420	2635656	0.4615	20.8174	20.0000	104.1
std8.D	C24:0	C19:0	Calibration	13.408	2700382	2927430	0.9224	41.6011	40.0000	104.0
std9.D	C24:0	C19:0	Calibration	13.417	6033710	3505157	1.7214	77.6270	80.0000	97.0
std10.D	C24:0	C19:0	Calibration	13.426	11459938	4076680	2.8111	126.7645	160.0000	79.2
qc-3.D	C24:0	C19:0	Sample	13.400	1463832	2180317	0.6714	30.2805		
sp-17.D	C24:0	C19:0	Sample	13.399	3030	1822377	0.0017	0.0813		
sp-18.D	C24:0	C19:0	Sample	13.395	1035	1869407	0.0006	0.0313		
sp-19.D	C24:0	C19:0	Sample	13.395	1585	1809576	0.0009	0.0458		
sp-20.D	C24:0	C19:0	Sample	13.400	3028	1890097	0.0016	0.0786		
sp-21.D	C24:0	C19:0	Sample	13.400	2466	2090151	0.0012	0.0595		
sp-22.D	C24:0	C19:0	Sample	13.400	3286	1884649	0.0017	0.0850		
sp-23.D	C24:0	C19:0	Sample	13.400	1840	1819204	0.0010	0.0519		
sp-24.D	C24:0	C19:0	Sample	13.395	2028	1859181	0.0011	0.0555		
qc-4.D	C24:0	C19:0	Sample	13.399	1415396	2169029	0.6525	29.4311		
sp-25.D	C24:0	C19:0	Sample	13.395	2115	1815559	0.0012	0.0589		
sp-26.D	C24:0	C19:0	Sample	13.395	3298	1841988	0.0018	0.0871		
sp-27.D	C24:0	C19:0	Sample	13.395	1745	1938349	0.0009	0.0469		
sp-28.D	C24:0	C19:0	Sample	13.395	2850	1948544	0.0015	0.0723		
sp-29.D	C24:0	C19:0	Sample	13.399	3004	1770121	0.0017	0.0829		
sp-30.D	C24:0	C19:0	Sample	13.395	1121	1720523	0.0007	0.0357		
sp-31.D	C24:0	C19:0	Sample	13.395	2118	1756510	0.0012	0.0607		
sp-32.D	C24:0	C19:0	Sample	13.395	1575	1813710	0.0009	0.0455		
qc-5.D	C24:0	C19:0	Sample	13.400	1368092	2038657	0.6711	30.2665		

C22:6

数据文件	化合物	ISTD	样品类型	RT	响应	ISTD 响应	响应比	最终浓度	预期的浓度	准确度
blank.D	C22:6	C19:0	Blank	13.426	0	1614233	0.0000	ND		
std1.D	C22:6	C19:0	Calibration	13.524	3129	1476809	0.0021	0.1617	0.1563	103.5
std2.D	C22:6	C19:0	Calibration	13.524	7170	1765013	0.0041	0.3053	0.3125	97.7
std3.D	C22:6	C19:0	Calibration	13.520	14842	1945904	0.0076	0.5686	0.6250	91.0
std4.D	C22:6	C19:0	Calibration	13.524	33231	2060032	0.0161	1.1968	1.2500	95.7
std5.D	C22:6	C19:0	Calibration	13.524	76310	2159349	0.0353	2.6157	2.5000	104.6
std6.D	C22:6	C19:0	Calibration	13.524	175344	2537627	0.0691	5.1095	5.0000	102.2
std7.D	C22:6	C19:0	Calibration	13.524	375373	2635656	0.1424	10.5259	10.0000	105.3
std8.D	C22:6	C19:0	Calibration	13.524	896402	2927430	0.3062	22.6248	20.0000	113.1
std9.D	C22:6	C19:0	Calibration	13.529	2149727	3505157	0.6133	45.3100	40.0000	113.3
std10.D	C22:6	C19:0	Calibration	13.533	4220443	4076680	1.0353	76.4803	80.0000	95.6
qc-3.D	C22:6	C19:0	Sample	13.520	376923	2180317	0.1729	12.7755		
sp-17.D	C22:6	C19:0	Sample	13.511	1336	1822377	0.0007	0.0594		
sp-18.D	C22:6	C19:0	Sample	13.515	838	1869407	0.0004	0.0383		
sp-19.D	C22:6	C19:0	Sample	13.511	603	1809576	0.0003	0.0298		
sp-20.D	C22:6	C19:0	Sample	13.515	489	1890097	0.0003	0.0243		
sp-21.D	C22:6	C19:0	Sample	13.515	1424	2090151	0.0007	0.0555		
sp-22.D	C22:6	C19:0	Sample	13.515	1175	1884649	0.0006	0.0513		
sp-23.D	C22:6	C19:0	Sample	13.515	1288	1819204	0.0007	0.0575		
sp-24.D	C22:6	C19:0	Sample	13.515	662	1859181	0.0004	0.0315		
qc-4.D	C22:6	C19:0	Sample	13.515	359436	2169029	0.1657	12.2464		
sp-25.D	C22:6	C19:0	Sample	13.511	1351	1815559	0.0007	0.0602		
sp-26.D	C22:6	C19:0	Sample	13.515	687	1841988	0.0004	0.0328		
sp-27.D	C22:6	C19:0	Sample	13.511	826	1938349	0.0004	0.0367		
sp-28.D	C22:6	C19:0	Sample	13.515	1730	1948544	0.0009	0.0708		
sp-29.D	C22:6	C19:0	Sample	13.515	789	1770121	0.0004	0.0381		
sp-30.D	C22:6	C19:0	Sample	13.506	403	1720523	0.0002	0.0225		
sp-31.D	C22:6	C19:0	Sample	13.511	529	1756510	0.0003	0.0275		
sp-32.D	C22:6	C19:0	Sample	13.511	465	1813710	0.0003	0.0242		
qc-5.D	C22:6	C19:0	Sample	13.515	346340	2038657	0.1699	12.5547		

C24:1

数据文件	化合物	ISTD	样品类型	RT	响应	ISTD 响应	响应比	最终浓度	预期的浓度	准确度
blank.D	C24:1	C19:0	Blank	13.671	0	1614233	0.0000	ND		
std1.D	C24:1	C19:0	Calibration	13.640	3307	1476809	0.0022	0.1923	0.1563	123.1
std2.D	C24:1	C19:0	Calibration	13.635	7332	1765013	0.0042	0.3612	0.3125	115.6
std3.D	C24:1	C19:0	Calibration	13.635	12172	1945904	0.0063	0.5465	0.6250	87.4
std4.D	C24:1	C19:0	Calibration	13.640	26091	2060032	0.0127	1.1119	1.2500	88.9
std5.D	C24:1	C19:0	Calibration	13.640	56528	2159349	0.0262	2.3037	2.5000	92.1
std6.D	C24:1	C19:0	Calibration	13.635	124624	2537627	0.0491	4.3263	5.0000	86.5
std7.D	C24:1	C19:0	Calibration	13.639	306721	2635656	0.1164	10.2590	10.0000	102.6

C24:1

数据文件	化合物	ISTD	样品类型	RT	响应	ISTD 响应	响应比	最终浓度	预期的浓度	准确度
std8.D	C24:1	C19:0	Calibration	13.640	688642	2927430	0.2352	20.7429	20.0000	103.7
std9.D	C24:1	C19:0	Calibration	13.644	1537559	3505157	0.4387	38.6845	40.0000	96.7
std10.D	C24:1	C19:0	Calibration	13.653	2974941	4076680	0.7297	64.3589	80.0000	80.4
qc-3.D	C24:1	C19:0	Sample	13.635	367375	2180317	0.1685	14.8562		
sp-17.D	C24:1	C19:0	Sample	13.675	0	1822377	0.0000	ND		
sp-18.D	C24:1	C19:0	Sample	13.671	0	1869407	0.0000	ND		
sp-19.D	C24:1	C19:0	Sample	13.675	0	1809576	0.0000	ND		
sp-20.D	C24:1	C19:0	Sample	13.680	0	1890097	0.0000	ND		
sp-21.D	C24:1	C19:0	Sample	13.675	0	2090151	0.0000	ND		
sp-22.D	C24:1	C19:0	Sample	13.675	0	1884649	0.0000	ND		
sp-23.D	C24:1	C19:0	Sample	13.671	0	1819204	0.0000	ND		
sp-24.D	C24:1	C19:0	Sample	13.671	0	1859181	0.0000	ND		
qc-4.D	C24:1	C19:0	Sample	13.631	358876	2169029	0.1655	14.5880		
sp-25.D	C24:1	C19:0	Sample	13.640	0	1815559	0.0000	ND		
sp-26.D	C24:1	C19:0	Sample	13.631	0	1841988	0.0000	ND		
sp-27.D	C24:1	C19:0	Sample	13.666	0	1938349	0.0000	ND		
sp-28.D	C24:1	C19:0	Sample	13.667	0	1948544	0.0000	ND		
sp-29.D	C24:1	C19:0	Sample	13.671	0	1770121	0.0000	ND		
sp-30.D	C24:1	C19:0	Sample	13.671	0	1720523	0.0000	ND		
sp-31.D	C24:1	C19:0	Sample	13.635	0	1756510	0.0000	ND		
sp-32.D	C24:1	C19:0	Sample	13.675	0	1813710	0.0000	ND		
qc-5.D	C24:1	C19:0	Sample	13.631	344696	2038657	0.1691	14.9078		

**批处理路径**	G:\GC-MS\HX250430-4-GCMS总脂肪酸靶向检测\HX250430-4\QuantResults\HX250430-4.batch.bin		
**分析时间**	2025/5/14 16:58	**分析员姓名**	DESKTOP-M3A0GPO\omics
**报告时间**	2025/5/16 14:52:56	**报告员姓名**	DESKTOP-M3A0GPO\omics
**最近校正更新**	2025/5/14 16:58	**批处理状态**	已处理
**定量批处理版本**	10.2	**定量报告版本**	10.2

C4:0

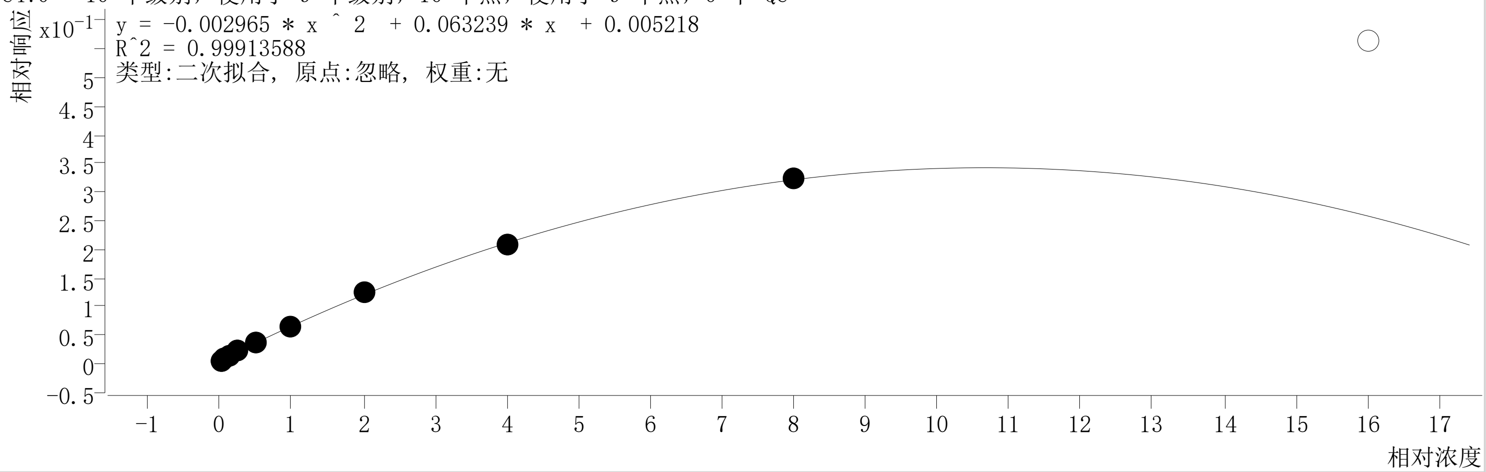

校正 STD 路径	校正类型	级别	已启用	响应	预期的浓度	响应因子
F:\D\GC-MS\脂肪酸\20250508-zfs\std1.D	校正	1	x	6903	0.3125	0.1496
F:\D\GC-MS\脂肪酸\20250508-zfs\std2.D	校正	2	x	13000	0.6250	0.1178
F:\D\GC-MS\脂肪酸\20250508-zfs\std3.D	校正	3	x	23261	1.2500	0.0956
F:\D\GC-MS\脂肪酸\20250508-zfs\std4.D	校正	4	x	47125	2.5000	0.0915
F:\D\GC-MS\脂肪酸\20250508-zfs\std5.D	校正	5	x	84256	5.0000	0.0780
F:\D\GC-MS\脂肪酸\20250508-zfs\std6.D	校正	6	x	161600	10.0000	0.0637
F:\D\GC-MS\脂肪酸\20250508-zfs\std7.D	校正	7	x	331688	20.0000	0.0629
F:\D\GC-MS\脂肪酸\20250508-zfs\std8.D	校正	8	x	603804	40.0000	0.0516
F:\D\GC-MS\脂肪酸\20250508-zfs\std9.D	校正	9	x	1129200	80.0000	0.0403
F:\D\GC-MS\脂肪酸\20250508-zfs\std10.D	校正	10		2300709	160.0000	0.0353

**批处理路径**	G:\GC-MS\HX250430-4-GCMS总脂肪酸靶向检测\HX250430-4\QuantResults\HX250430-4.batch.bin		
**分析时间**	2025/5/14 16:58	**分析员姓名**	DESKTOP-M3A0GPO\omics
**报告时间**	2025/5/16 14:52:56	**报告员姓名**	DESKTOP-M3A0GPO\omics
**最近校正更新**	2025/5/14 16:58	**批处理状态**	已处理
**定量批处理版本**	10.2	**定量报告版本**	10.2

C6:0

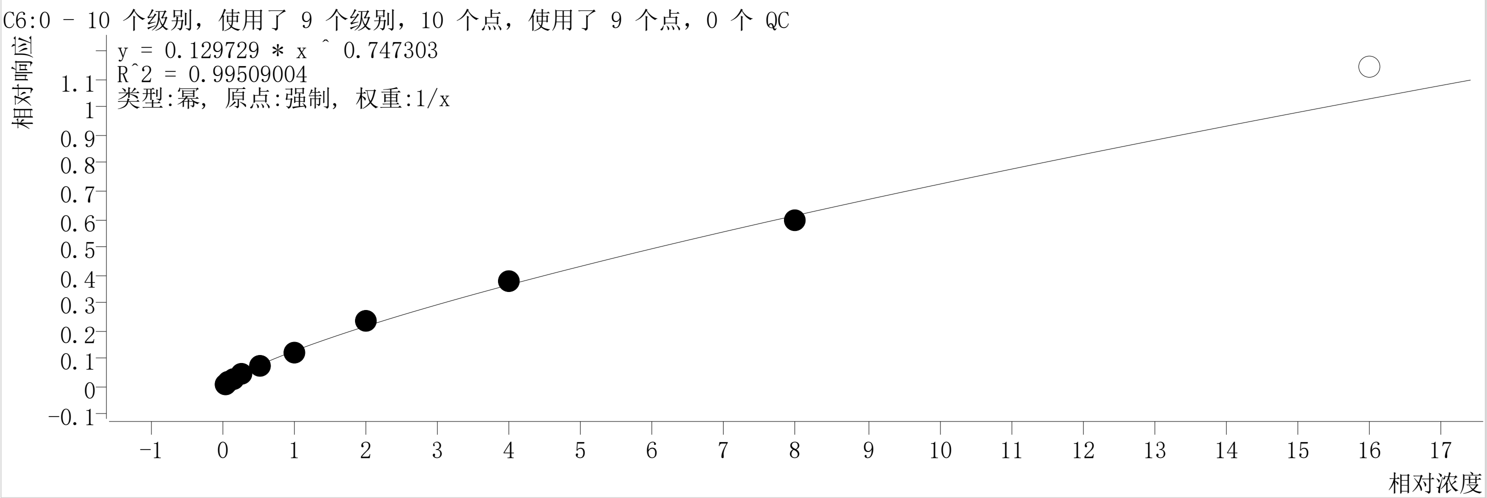

校正 STD 路径	校正类型	级别	已启用	响应	预期的浓度	响应因子
F:\D\GC-MS\脂肪酸\20250508-zfs\std1.D	校正	1	x	16067	0.3125	0.3481
F:\D\GC-MS\脂肪酸\20250508-zfs\std2.D	校正	2	x	29610	0.6250	0.2684
F:\D\GC-MS\脂肪酸\20250508-zfs\std3.D	校正	3	x	47699	1.2500	0.1961
F:\D\GC-MS\脂肪酸\20250508-zfs\std4.D	校正	4	x	99304	2.5000	0.1928
F:\D\GC-MS\脂肪酸\20250508-zfs\std5.D	校正	5	x	155929	5.0000	0.1444
F:\D\GC-MS\脂肪酸\20250508-zfs\std6.D	校正	6	x	302051	10.0000	0.1190
F:\D\GC-MS\脂肪酸\20250508-zfs\std7.D	校正	7	x	629485	20.0000	0.1194
F:\D\GC-MS\脂肪酸\20250508-zfs\std8.D	校正	8	x	1098736	40.0000	0.0938
F:\D\GC-MS\脂肪酸\20250508-zfs\std9.D	校正	9	x	2093987	80.0000	0.0747
F:\D\GC-MS\脂肪酸\20250508-zfs\std10.D	校正	10		4667340	160.0000	0.0716

**批处理路径**	G:\GC-MS\HX250430-4-GCMS总脂肪酸靶向检测\HX250430-4\QuantResults\HX250430-4.batch.bin		
**分析时间**	2025/5/14 16:58	**分析员姓名**	DESKTOP-M3A0GPO\omics
**报告时间**	2025/5/16 14:52:57	**报告员姓名**	DESKTOP-M3A0GPO\omics
**最近校正更新**	2025/5/14 16:58	**批处理状态**	已处理
**定量批处理版本**	10.2	**定量报告版本**	10.2

C8:0

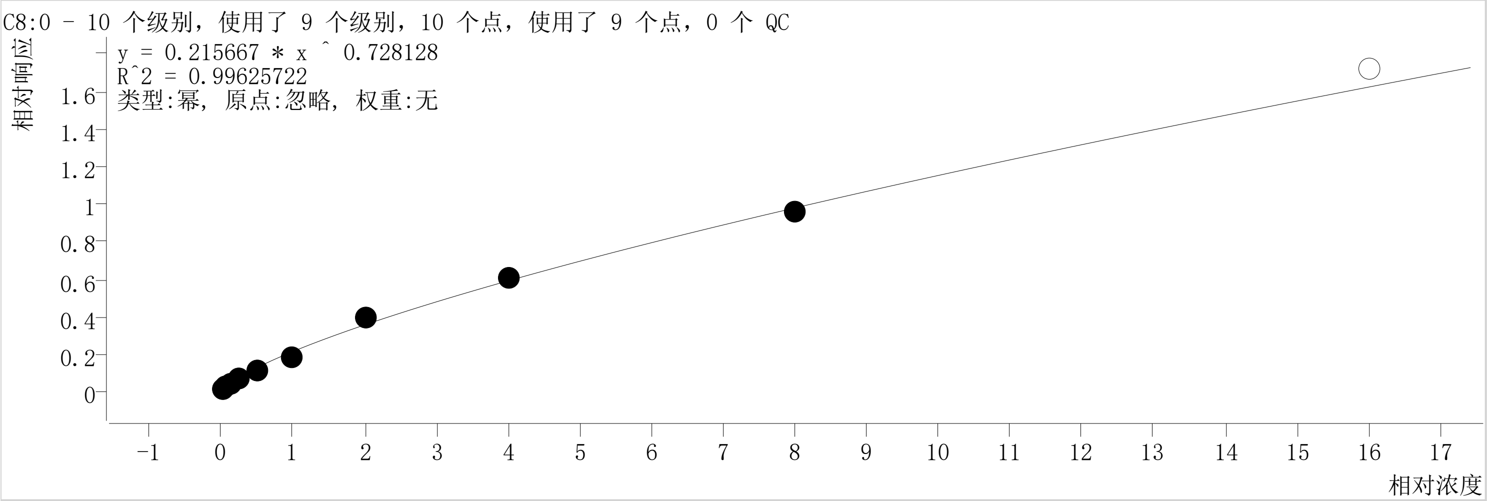

校正 STD 路径	校正类型	级别	已启用	响应	预期的浓度	响应因子
F:\D\GC-MS\脂肪酸\20250508-zfs\std1.D	校正	1	x	24525	0.3125	0.5314
F:\D\GC-MS\脂肪酸\20250508-zfs\std2.D	校正	2	x	45885	0.6250	0.4159
F:\D\GC-MS\脂肪酸\20250508-zfs\std3.D	校正	3	x	73304	1.2500	0.3014
F:\D\GC-MS\脂肪酸\20250508-zfs\std4.D	校正	4	x	155912	2.5000	0.3027
F:\D\GC-MS\脂肪酸\20250508-zfs\std5.D	校正	5	x	241330	5.0000	0.2235
F:\D\GC-MS\脂肪酸\20250508-zfs\std6.D	校正	6	x	463239	10.0000	0.1825
F:\D\GC-MS\脂肪酸\20250508-zfs\std7.D	校正	7	x	1028279	20.0000	0.1951
F:\D\GC-MS\脂肪酸\20250508-zfs\std8.D	校正	8	x	1792155	40.0000	0.1530
F:\D\GC-MS\脂肪酸\20250508-zfs\std9.D	校正	9	x	3387722	80.0000	0.1208
F:\D\GC-MS\脂肪酸\20250508-zfs\std10.D	校正	10		7014399	160.0000	0.1075

**批处理路径**	G:\GC-MS\HX250430-4-GCMS总脂肪酸靶向检测\HX250430-4\QuantResults\HX250430-4.batch.bin		
**分析时间**	2025/5/14 16:58	**分析员姓名**	DESKTOP-M3A0GPO\omics
**报告时间**	2025/5/16 14:52:57	**报告员姓名**	DESKTOP-M3A0GPO\omics
**最近校正更新**	2025/5/14 16:58	**批处理状态**	已处理
**定量批处理版本**	10.2	**定量报告版本**	10.2

C10:0

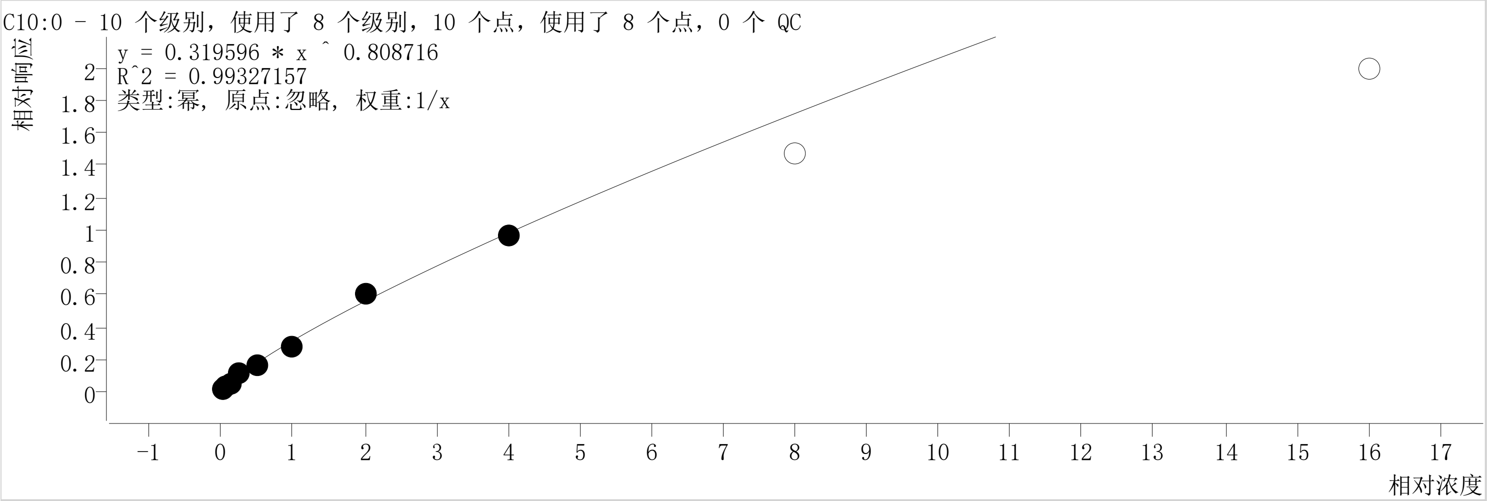

校正 STD 路径	校正类型	级别	已启用	响应	预期的浓度	响应因子
F:\D\GC-MS\脂肪酸\20250508-zfs\std1.D	校正	1	x	32116	0.3125	0.6959
F:\D\GC-MS\脂肪酸\20250508-zfs\std2.D	校正	2	x	62998	0.6250	0.5711
F:\D\GC-MS\脂肪酸\20250508-zfs\std3.D	校正	3	x	107795	1.2500	0.4432
F:\D\GC-MS\脂肪酸\20250508-zfs\std4.D	校正	4	x	227576	2.5000	0.4419
F:\D\GC-MS\脂肪酸\20250508-zfs\std5.D	校正	5	x	376172	5.0000	0.3484
F:\D\GC-MS\脂肪酸\20250508-zfs\std6.D	校正	6	x	721537	10.0000	0.2843
F:\D\GC-MS\脂肪酸\20250508-zfs\std7.D	校正	7	x	1614554	20.0000	0.3063
F:\D\GC-MS\脂肪酸\20250508-zfs\std8.D	校正	8	x	2827697	40.0000	0.2415
F:\D\GC-MS\脂肪酸\20250508-zfs\std9.D	校正	9		5151378	80.0000	0.1837
F:\D\GC-MS\脂肪酸\20250508-zfs\std10.D	校正	10		8122381	160.0000	0.1245

**批处理路径**	G:\GC-MS\HX250430-4-GCMS总脂肪酸靶向检测\HX250430-4\QuantResults\HX250430-4.batch.bin		
**分析时间**	2025/5/14 16:58	**分析员姓名**	DESKTOP-M3A0GPO\omics
**报告时间**	2025/5/16 14:52:57	**报告员姓名**	DESKTOP-M3A0GPO\omics
**最近校正更新**	2025/5/14 16:58	**批处理状态**	已处理
**定量批处理版本**	10.2	**定量报告版本**	10.2

C11:0

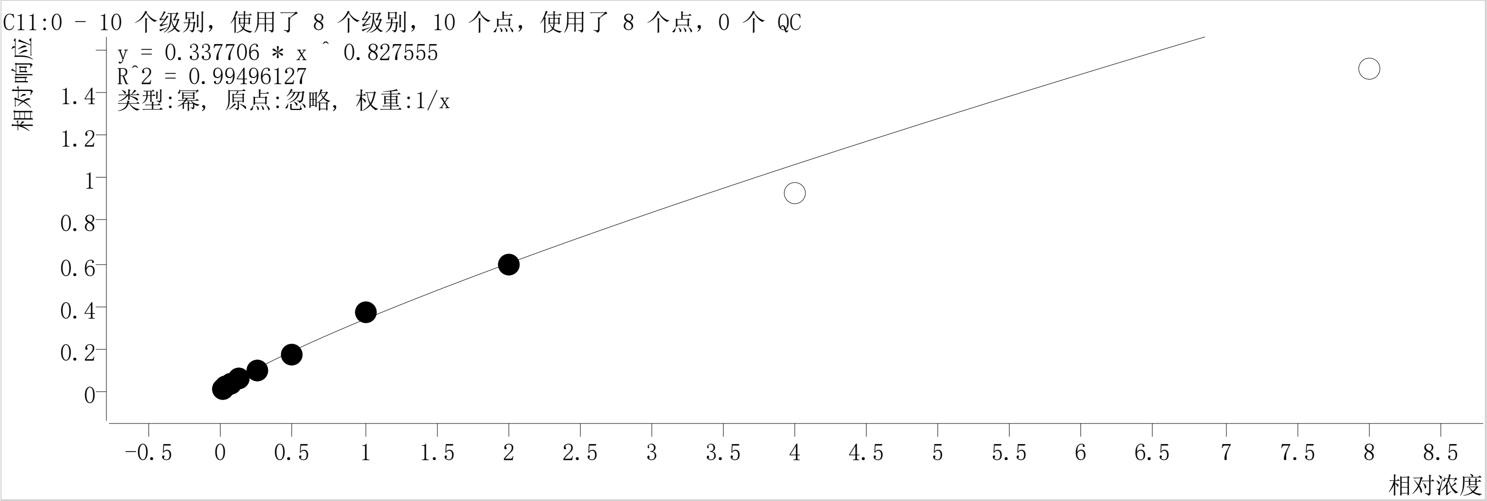

校正 STD 路径	校正类型	级别	已启用	响应	预期的浓度	响应因子
F:\D\GC-MS\脂肪酸\20250508-zfs\std1.D	校正	1	x	17645	0.1563	0.7647
F:\D\GC-MS\脂肪酸\20250508-zfs\std2.D	校正	2	x	35366	0.3125	0.6412
F:\D\GC-MS\脂肪酸\20250508-zfs\std3.D	校正	3	x	62803	0.6250	0.5164
F:\D\GC-MS\脂肪酸\20250508-zfs\std4.D	校正	4	x	130127	1.2500	0.5053
F:\D\GC-MS\脂肪酸\20250508-zfs\std5.D	校正	5	x	224035	2.5000	0.4150
F:\D\GC-MS\脂肪酸\20250508-zfs\std6.D	校正	6	x	435048	5.0000	0.3429
F:\D\GC-MS\脂肪酸\20250508-zfs\std7.D	校正	7	x	965645	10.0000	0.3664
F:\D\GC-MS\脂肪酸\20250508-zfs\std8.D	校正	8	x	1728180	20.0000	0.2952
F:\D\GC-MS\脂肪酸\20250508-zfs\std9.D	校正	9		3258125	40.0000	0.2324
F:\D\GC-MS\脂肪酸\20250508-zfs\std10.D	校正	10		6162737	80.0000	0.1890

**批处理路径**	G:\GC-MS\HX250430-4-GCMS总脂肪酸靶向检测\HX250430-4\QuantResults\HX250430-4.batch.bin		
**分析时间**	2025/5/14 16:58	**分析员姓名**	DESKTOP-M3A0GPO\omics
**报告时间**	2025/5/16 14:52:57	**报告员姓名**	DESKTOP-M3A0GPO\omics
**最近校正更新**	2025/5/14 16:58	**批处理状态**	已处理
**定量批处理版本**	10.2	**定量报告版本**	10.2

C12:0

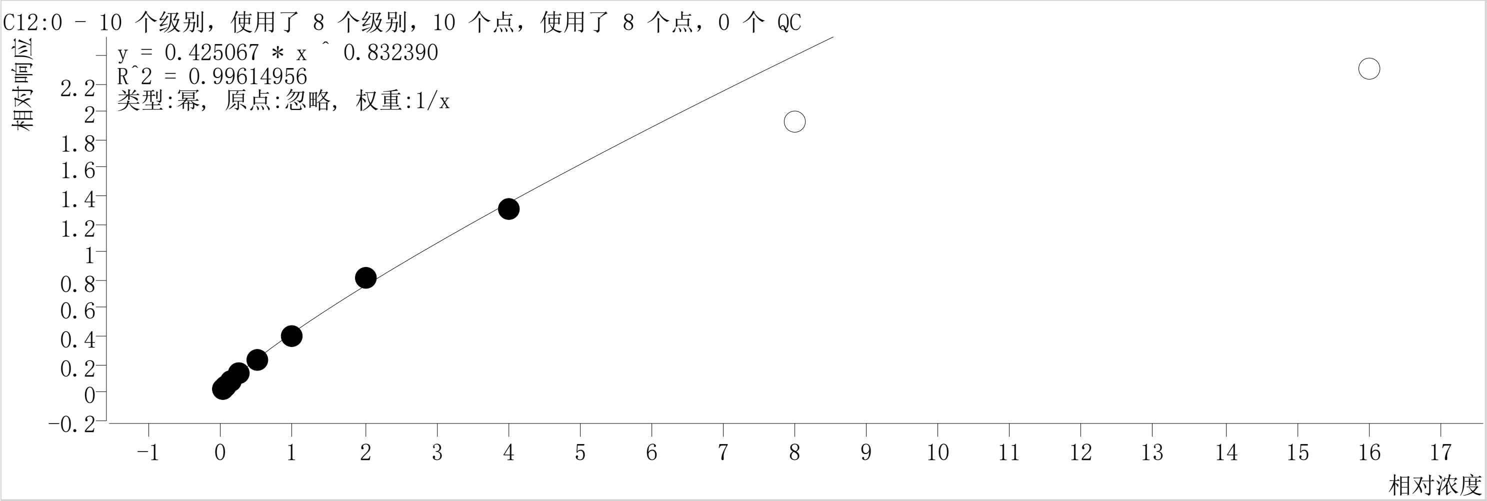

校正 STD 路径	校正类型	级别	已启用	响应	预期的浓度	响应因子
F:\D\GC-MS\脂肪酸\20250508-zfs\std1.D	校正	1	x	35568	0.3125	0.7707
F:\D\GC-MS\脂肪酸\20250508-zfs\std2.D	校正	2	x	74112	0.6250	0.6718
F:\D\GC-MS\脂肪酸\20250508-zfs\std3.D	校正	3	x	137700	1.2500	0.5661
F:\D\GC-MS\脂肪酸\20250508-zfs\std4.D	校正	4	x	285188	2.5000	0.5538
F:\D\GC-MS\脂肪酸\20250508-zfs\std5.D	校正	5	x	516546	5.0000	0.4784
F:\D\GC-MS\脂肪酸\20250508-zfs\std6.D	校正	6	x	1012197	10.0000	0.3989
F:\D\GC-MS\脂肪酸\20250508-zfs\std7.D	校正	7	x	2170310	20.0000	0.4117
F:\D\GC-MS\脂肪酸\20250508-zfs\std8.D	校正	8	x	3824034	40.0000	0.3266
F:\D\GC-MS\脂肪酸\20250508-zfs\std9.D	校正	9		6792748	80.0000	0.2422
F:\D\GC-MS\脂肪酸\20250508-zfs\std10.D	校正	10		9395661	160.0000	0.1440

**批处理路径**	G:\GC-MS\HX250430-4-GCMS总脂肪酸靶向检测\HX250430-4\QuantResults\HX250430-4.batch.bin		
**分析时间**	2025/5/14 16:58	**分析员姓名**	DESKTOP-M3A0GPO\omics
**报告时间**	2025/5/16 14:52:57	**报告员姓名**	DESKTOP-M3A0GPO\omics
**最近校正更新**	2025/5/14 16:58	**批处理状态**	已处理
**定量批处理版本**	10.2	**定量报告版本**	10.2

C13:0

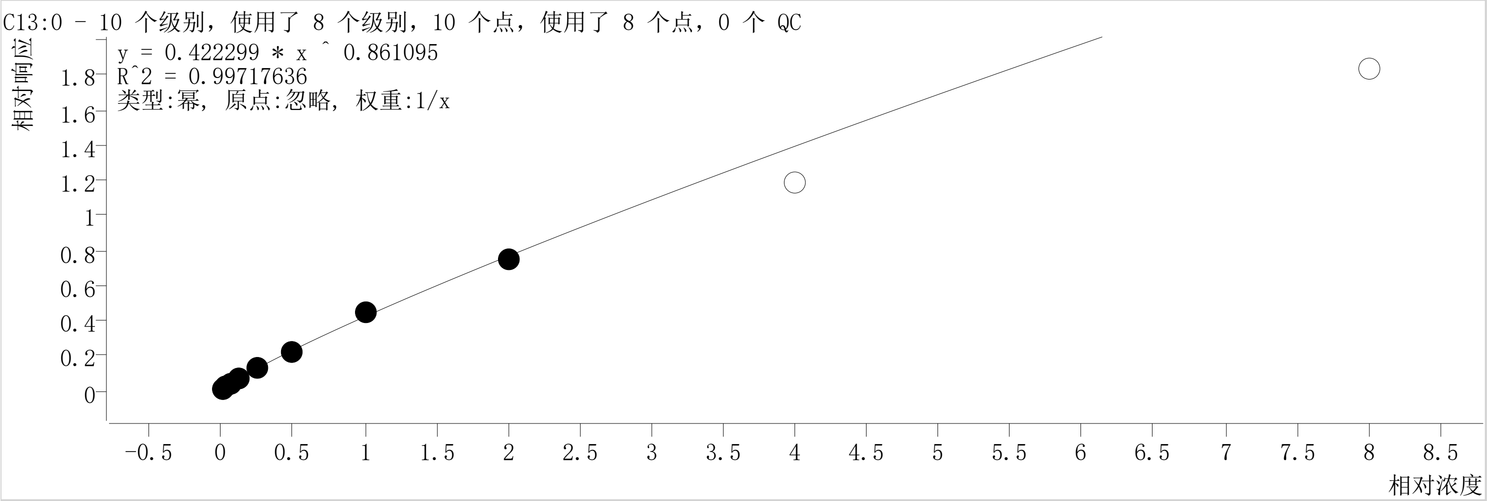

校正 STD 路径	校正类型	级别	已启用	响应	预期的浓度	响应因子
F:\D\GC-MS\脂肪酸\20250508-zfs\std1.D	校正	1	x	17506	0.1563	0.7586
F:\D\GC-MS\脂肪酸\20250508-zfs\std2.D	校正	2	x	37633	0.3125	0.6823
F:\D\GC-MS\脂肪酸\20250508-zfs\std3.D	校正	3	x	71662	0.6250	0.5892
F:\D\GC-MS\脂肪酸\20250508-zfs\std4.D	校正	4	x	146832	1.2500	0.5702
F:\D\GC-MS\脂肪酸\20250508-zfs\std5.D	校正	5	x	279061	2.5000	0.5169
F:\D\GC-MS\脂肪酸\20250508-zfs\std6.D	校正	6	x	558053	5.0000	0.4398
F:\D\GC-MS\脂肪酸\20250508-zfs\std7.D	校正	7	x	1198590	10.0000	0.4548
F:\D\GC-MS\脂肪酸\20250508-zfs\std8.D	校正	8	x	2186179	20.0000	0.3734
F:\D\GC-MS\脂肪酸\20250508-zfs\std9.D	校正	9		4183814	40.0000	0.2984
F:\D\GC-MS\脂肪酸\20250508-zfs\std10.D	校正	10		7475317	80.0000	0.2292

**批处理路径**	G:\GC-MS\HX250430-4-GCMS总脂肪酸靶向检测\HX250430-4\QuantResults\HX250430-4.batch.bin		
**分析时间**	2025/5/14 16:58	**分析员姓名**	DESKTOP-M3A0GPO\omics
**报告时间**	2025/5/16 14:52:57	**报告员姓名**	DESKTOP-M3A0GPO\omics
**最近校正更新**	2025/5/14 16:58	**批处理状态**	已处理
**定量批处理版本**	10.2	**定量报告版本**	10.2

C14:0

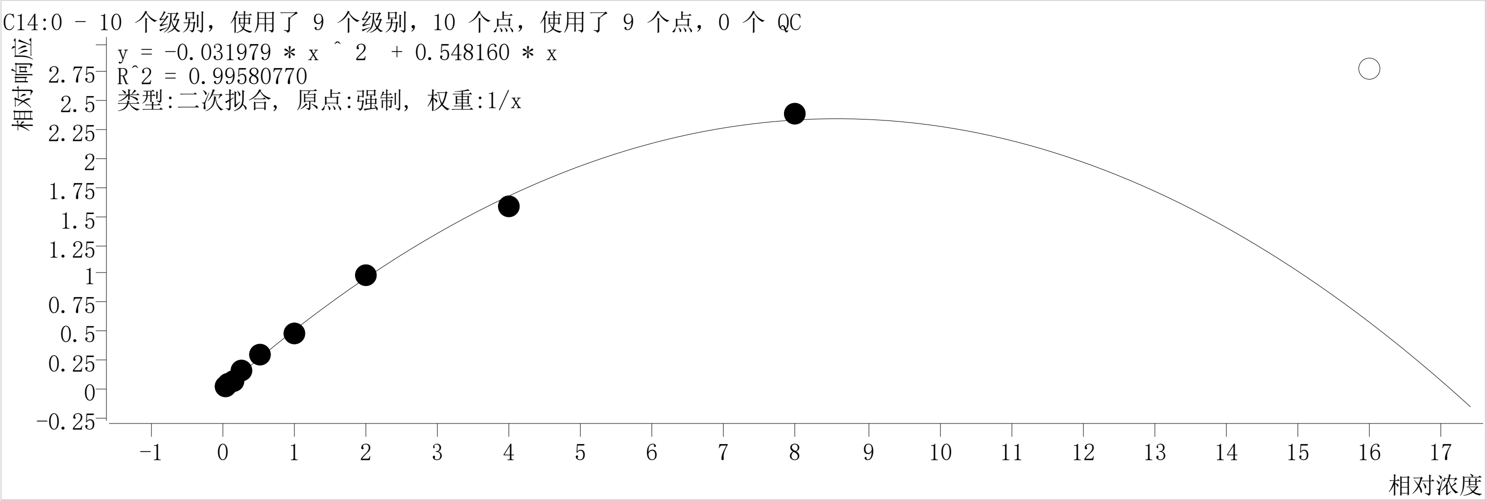

校正 STD 路径	校正类型	级别	已启用	响应	预期的浓度	响应因子
F:\D\GC-MS\脂肪酸\20250508-zfs\std1.D	校正	1	x	34247	0.3125	0.7421
F:\D\GC-MS\脂肪酸\20250508-zfs\std2.D	校正	2	x	76645	0.6250	0.6948
F:\D\GC-MS\脂肪酸\20250508-zfs\std3.D	校正	3	x	152965	1.2500	0.6289
F:\D\GC-MS\脂肪酸\20250508-zfs\std4.D	校正	4	x	313068	2.5000	0.6079
F:\D\GC-MS\脂肪酸\20250508-zfs\std5.D	校正	5	x	619173	5.0000	0.5735
F:\D\GC-MS\脂肪酸\20250508-zfs\std6.D	校正	6	x	1247316	10.0000	0.4915
F:\D\GC-MS\脂肪酸\20250508-zfs\std7.D	校正	7	x	2575499	20.0000	0.4886
F:\D\GC-MS\脂肪酸\20250508-zfs\std8.D	校正	8	x	4648797	40.0000	0.3970
F:\D\GC-MS\脂肪酸\20250508-zfs\std9.D	校正	9	x	8355984	80.0000	0.2980
F:\D\GC-MS\脂肪酸\20250508-zfs\std10.D	校正	10		11343421	160.0000	0.1739

**批处理路径**	G:\GC-MS\HX250430-4-GCMS总脂肪酸靶向检测\HX250430-4\QuantResults\HX250430-4.batch.bin		
**分析时间**	2025/5/14 16:58	**分析员姓名**	DESKTOP-M3A0GPO\omics
**报告时间**	2025/5/16 14:52:57	**报告员姓名**	DESKTOP-M3A0GPO\omics
**最近校正更新**	2025/5/14 16:58	**批处理状态**	已处理
**定量批处理版本**	10.2	**定量报告版本**	10.2

C14:1

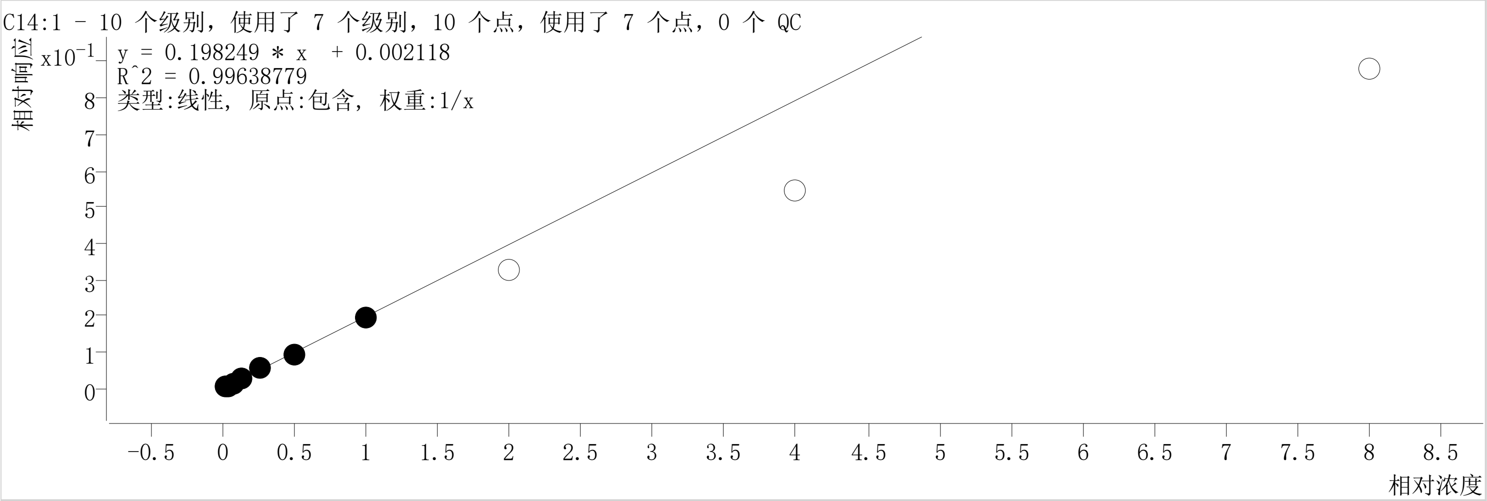

校正 STD 路径	校正类型	级别	已启用	响应	预期的浓度	响应因子
F:\D\GC-MS\脂肪酸\20250508-zfs\std1.D	校正	1	x	6864	0.1563	0.2974
F:\D\GC-MS\脂肪酸\20250508-zfs\std2.D	校正	2	x	14620	0.3125	0.2651
F:\D\GC-MS\脂肪酸\20250508-zfs\std3.D	校正	3	x	28748	0.6250	0.2364
F:\D\GC-MS\脂肪酸\20250508-zfs\std4.D	校正	4	x	63277	1.2500	0.2457
F:\D\GC-MS\脂肪酸\20250508-zfs\std5.D	校正	5	x	118980	2.5000	0.2204
F:\D\GC-MS\脂肪酸\20250508-zfs\std6.D	校正	6	x	246253	5.0000	0.1941
F:\D\GC-MS\脂肪酸\20250508-zfs\std7.D	校正	7	x	520980	10.0000	0.1977
F:\D\GC-MS\脂肪酸\20250508-zfs\std8.D	校正	8		965001	20.0000	0.1648
F:\D\GC-MS\脂肪酸\20250508-zfs\std9.D	校正	9		1927357	40.0000	0.1375
F:\D\GC-MS\脂肪酸\20250508-zfs\std10.D	校正	10		3591834	80.0000	0.1101

**批处理路径**	G:\GC-MS\HX250430-4-GCMS总脂肪酸靶向检测\HX250430-4\QuantResults\HX250430-4.batch.bin		
**分析时间**	2025/5/14 16:58	**分析员姓名**	DESKTOP-M3A0GPO\omics
**报告时间**	2025/5/16 14:52:57	**报告员姓名**	DESKTOP-M3A0GPO\omics
**最近校正更新**	2025/5/14 16:58	**批处理状态**	已处理
**定量批处理版本**	10.2	**定量报告版本**	10.2

C15:0

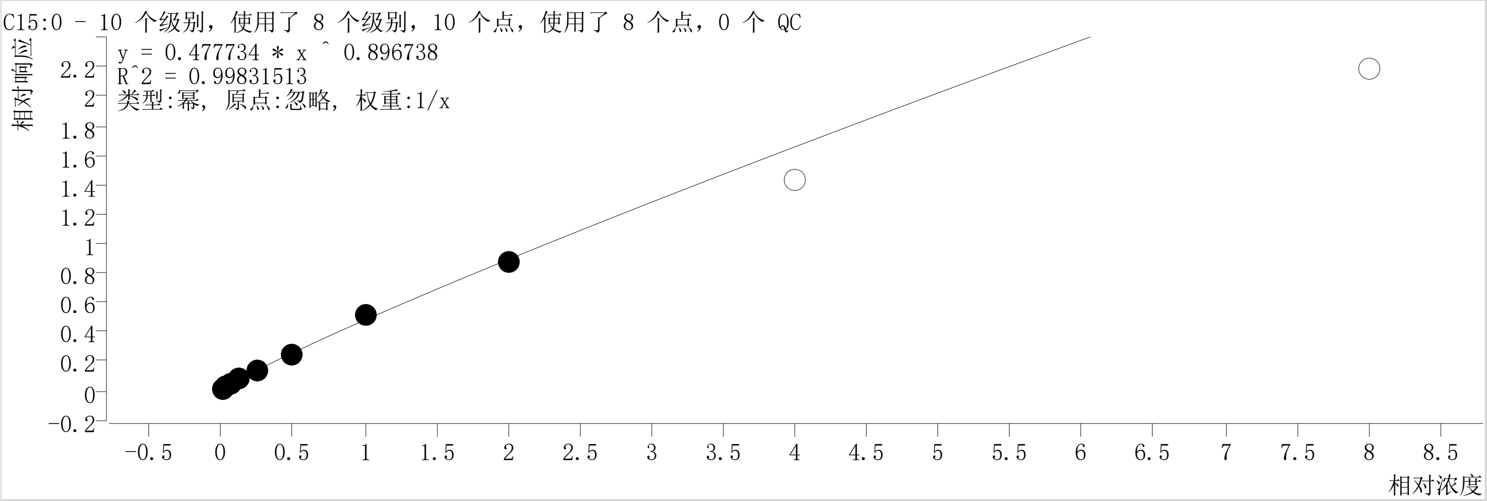

校正 STD 路径	校正类型	级别	已启用	响应	预期的浓度	响应因子
F:\D\GC-MS\脂肪酸\20250508-zfs\std1.D	校正	1	x	15816	0.1563	0.6854
F:\D\GC-MS\脂肪酸\20250508-zfs\std2.D	校正	2	x	36008	0.3125	0.6528
F:\D\GC-MS\脂肪酸\20250508-zfs\std3.D	校正	3	x	73702	0.6250	0.6060
F:\D\GC-MS\脂肪酸\20250508-zfs\std4.D	校正	4	x	150717	1.2500	0.5853
F:\D\GC-MS\脂肪酸\20250508-zfs\std5.D	校正	5	x	310000	2.5000	0.5742
F:\D\GC-MS\脂肪酸\20250508-zfs\std6.D	校正	6	x	635348	5.0000	0.5007
F:\D\GC-MS\脂肪酸\20250508-zfs\std7.D	校正	7	x	1329210	10.0000	0.5043
F:\D\GC-MS\脂肪酸\20250508-zfs\std8.D	校正	8	x	2539594	20.0000	0.4338
F:\D\GC-MS\脂肪酸\20250508-zfs\std9.D	校正	9		5044623	40.0000	0.3598
F:\D\GC-MS\脂肪酸\20250508-zfs\std10.D	校正	10		8911026	80.0000	0.2732

**批处理路径**	G:\GC-MS\HX250430-4-GCMS总脂肪酸靶向检测\HX250430-4\QuantResults\HX250430-4.batch.bin		
**分析时间**	2025/5/14 16:58	**分析员姓名**	DESKTOP-M3A0GPO\omics
**报告时间**	2025/5/16 14:52:57	**报告员姓名**	DESKTOP-M3A0GPO\omics
**最近校正更新**	2025/5/14 16:58	**批处理状态**	已处理
**定量批处理版本**	10.2	**定量报告版本**	10.2

C15:1

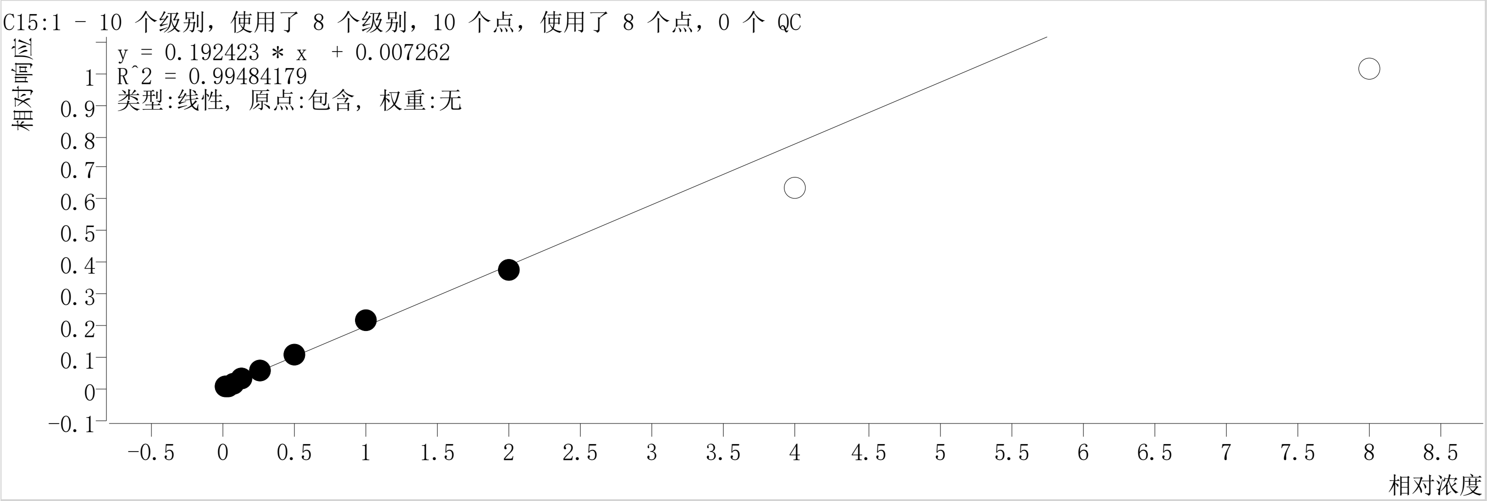

校正 STD 路径	校正类型	级别	已启用	响应	预期的浓度	响应因子
F:\D\GC-MS\脂肪酸\20250508-zfs\std1.D	校正	1	x	7138	0.1563	0.3094
F:\D\GC-MS\脂肪酸\20250508-zfs\std2.D	校正	2	x	16056	0.3125	0.2911
F:\D\GC-MS\脂肪酸\20250508-zfs\std3.D	校正	3	x	31509	0.6250	0.2591
F:\D\GC-MS\脂肪酸\20250508-zfs\std4.D	校正	4	x	65435	1.2500	0.2541
F:\D\GC-MS\脂肪酸\20250508-zfs\std5.D	校正	5	x	134263	2.5000	0.2487
F:\D\GC-MS\脂肪酸\20250508-zfs\std6.D	校正	6	x	275315	5.0000	0.2170
F:\D\GC-MS\脂肪酸\20250508-zfs\std7.D	校正	7	x	577044	10.0000	0.2189
F:\D\GC-MS\脂肪酸\20250508-zfs\std8.D	校正	8	x	1114045	20.0000	0.1903
F:\D\GC-MS\脂肪酸\20250508-zfs\std9.D	校正	9		2236842	40.0000	0.1595
F:\D\GC-MS\脂肪酸\20250508-zfs\std10.D	校正	10		4130142	80.0000	0.1266

**批处理路径**	G:\GC-MS\HX250430-4-GCMS总脂肪酸靶向检测\HX250430-4\QuantResults\HX250430-4.batch.bin		
**分析时间**	2025/5/14 16:58	**分析员姓名**	DESKTOP-M3A0GPO\omics
**报告时间**	2025/5/16 14:52:57	**报告员姓名**	DESKTOP-M3A0GPO\omics
**最近校正更新**	2025/5/14 16:58	**批处理状态**	已处理
**定量批处理版本**	10.2	**定量报告版本**	10.2

C16:0

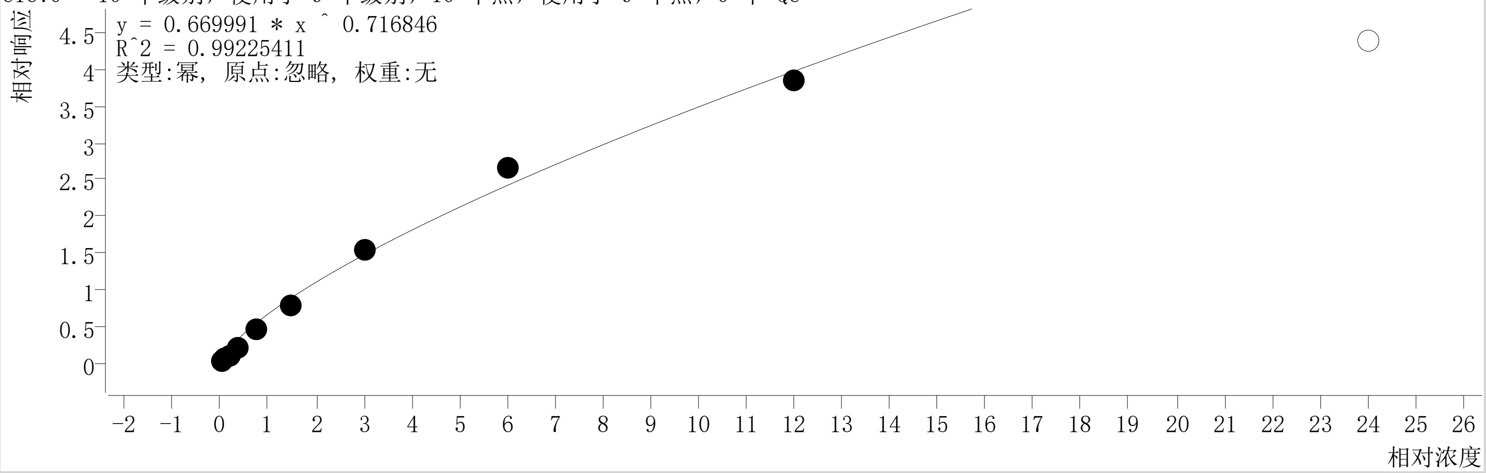

校正 STD 路径	校正类型	级别	已启用	响应	预期的浓度	响应因子
F:\D\GC-MS\脂肪酸\20250508-zfs\std1.D	校正	1	x	44744	0.4688	0.6463
F:\D\GC-MS\脂肪酸\20250508-zfs\std2.D	校正	2	x	105704	0.9375	0.6388
F:\D\GC-MS\脂肪酸\20250508-zfs\std3.D	校正	3	x	226913	1.8750	0.6219
F:\D\GC-MS\脂肪酸\20250508-zfs\std4.D	校正	4	x	464812	3.7500	0.6017
F:\D\GC-MS\脂肪酸\20250508-zfs\std5.D	校正	5	x	986954	7.5000	0.6094
F:\D\GC-MS\脂肪酸\20250508-zfs\std6.D	校正	6	x	2036671	15.0000	0.5351
F:\D\GC-MS\脂肪酸\20250508-zfs\std7.D	校正	7	x	4112945	30.0000	0.5202
F:\D\GC-MS\脂肪酸\20250508-zfs\std8.D	校正	8	x	7743304	60.0000	0.4408
F:\D\GC-MS\脂肪酸\20250508-zfs\std9.D	校正	9	x	13522533	120.0000	0.3215
F:\D\GC-MS\脂肪酸\20250508-zfs\std10.D	校正	10		17904657	240.0000	0.1830

**批处理路径**	G:\GC-MS\HX250430-4-GCMS总脂肪酸靶向检测\HX250430-4\QuantResults\HX250430-4.batch.bin		
**分析时间**	2025/5/14 16:58	**分析员姓名**	DESKTOP-M3AOGPO\omics
**报告时间**	2025/5/16 14:52:57	**报告员姓名**	DESKTOP-M3AOGPO\omics
**最近校正更新**	2025/5/14 16:58	**批处理状态**	已处理
**定量批处理版本**	10.2	**定量报告版本**	10.2

C16:1

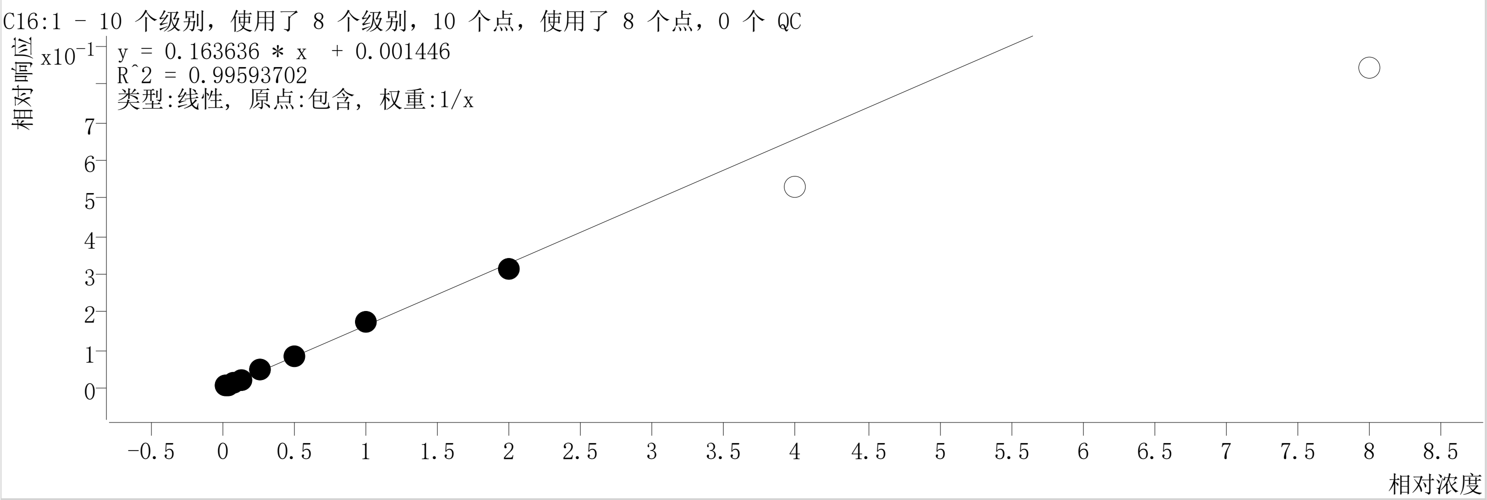

校正 STD 路径	校正类型	级别	已启用	响应	预期的浓度	响应因子
F:\D\GC-MS\脂肪酸\20250508-zfs\std1.D	校正	1	x	5056	0.1563	0.2191
F:\D\GC-MS\脂肪酸\20250508-zfs\std2.D	校正	2	x	10789	0.3125	0.1956
F:\D\GC-MS\脂肪酸\20250508-zfs\std3.D	校正	3	x	24334	0.6250	0.2001
F:\D\GC-MS\脂肪酸\20250508-zfs\std4.D	校正	4	x	48292	1.2500	0.1875
F:\D\GC-MS\脂肪酸\20250508-zfs\std5.D	校正	5	x	103309	2.5000	0.1914
F:\D\GC-MS\脂肪酸\20250508-zfs\std6.D	校正	6	x	217198	5.0000	0.1712
F:\D\GC-MS\脂肪酸\20250508-zfs\std7.D	校正	7	x	454539	10.0000	0.1725
F:\D\GC-MS\脂肪酸\20250508-zfs\std8.D	校正	8	x	913884	20.0000	0.1561
F:\D\GC-MS\脂肪酸\20250508-zfs\std9.D	校正	9		1864800	40.0000	0.1330
F:\D\GC-MS\脂肪酸\20250508-zfs\std10.D	校正	10		3434330	80.0000	0.1053

**批处理路径**	G:\GC-MS\HX250430-4-GCMS总脂肪酸靶向检测\HX250430-4\QuantResults\HX250430-4.batch.bin		
**分析时间**	2025/5/14 16:58	**分析员姓名**	DESKTOP-M3AOGPO\omics
**报告时间**	2025/5/16 14:52:57	**报告员姓名**	DESKTOP-M3AOGPO\omics
**最近校正更新**	2025/5/14 16:58	**批处理状态**	已处理
**定量批处理版本**	10.2	**定量报告版本**	10.2

C17:0

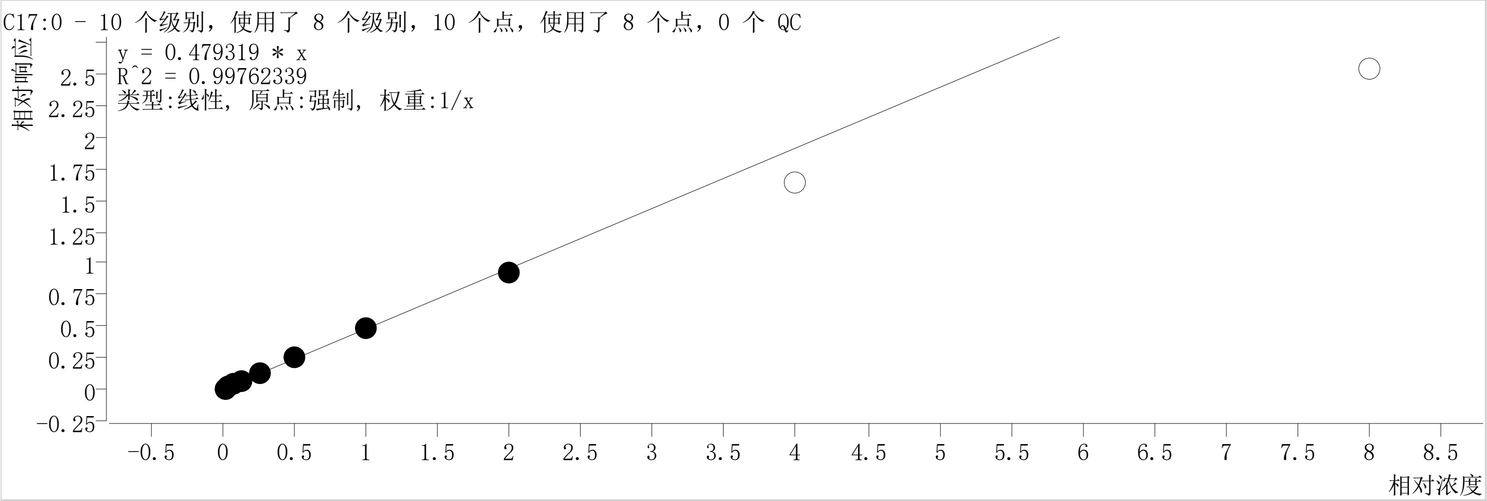

校正 STD 路径	校正类型	级别	已启用	响应	预期的浓度	响应因子
F:\D\GC-MS\脂肪酸\20250508-zfs\std1.D	校正	1	x	12199	0.1563	0.5287
F:\D\GC-MS\脂肪酸\20250508-zfs\std2.D	校正	2	x	28406	0.3125	0.5150
F:\D\GC-MS\脂肪酸\20250508-zfs\std3.D	校正	3	x	63123	0.6250	0.5190
F:\D\GC-MS\脂肪酸\20250508-zfs\std4.D	校正	4	x	129613	1.2500	0.5033
F:\D\GC-MS\脂肪酸\20250508-zfs\std5.D	校正	5	x	287436	2.5000	0.5324
F:\D\GC-MS\脂肪酸\20250508-zfs\std6.D	校正	6	x	621619	5.0000	0.4899
F:\D\GC-MS\脂肪酸\20250508-zfs\std7.D	校正	7	x	1294994	10.0000	0.4913
F:\D\GC-MS\脂肪酸\20250508-zfs\std8.D	校正	8	x	2695183	20.0000	0.4603
F:\D\GC-MS\脂肪酸\20250508-zfs\std9.D	校正	9		5748032	40.0000	0.4100
F:\D\GC-MS\脂肪酸\20250508-zfs\std10.D	校正	10		10377085	80.0000	0.3182

**批处理路径**	G:\GC-MS\HX250430-4-GCMS总脂肪酸靶向检测\HX250430-4\QuantResults\HX250430-4.batch.bin		
**分析时间**	2025/5/14 16:58	**分析员姓名**	DESKTOP-M3A0GPO\omics
**报告时间**	2025/5/16 14:52:57	**报告员姓名**	DESKTOP-M3A0GPO\omics
**最近校正更新**	2025/5/14 16:58	**批处理状态**	已处理
**定量批处理版本**	10.2	**定量报告版本**	10.2

C17:1

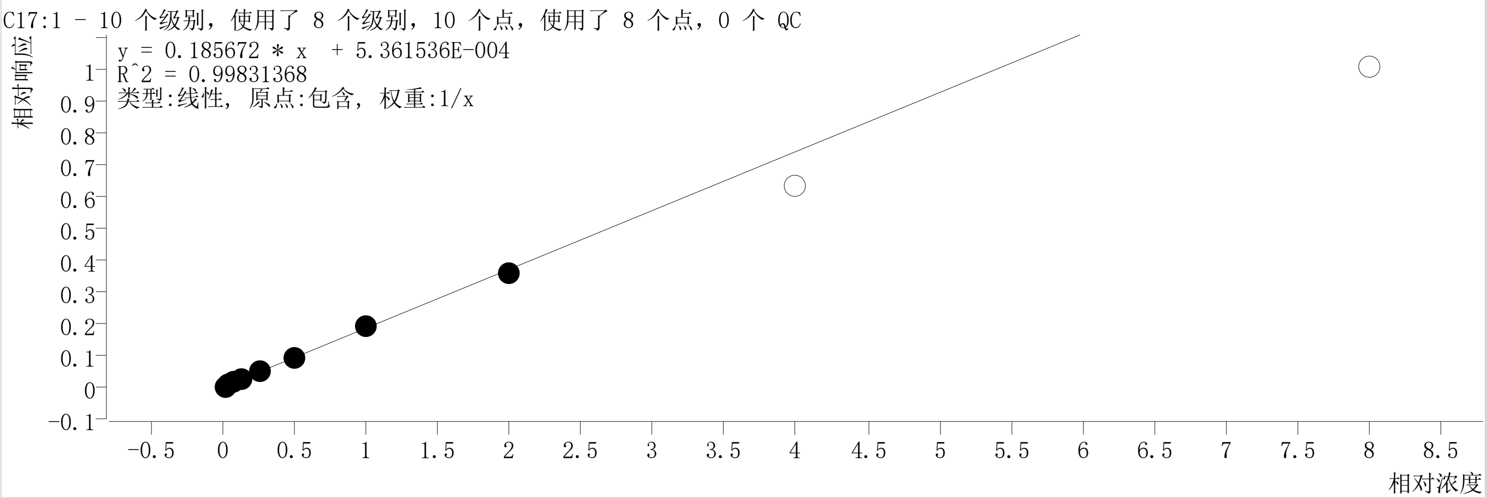

校正 STD 路径	校正类型	级别	已启用	响应	预期的浓度	响应因子
F:\D\GC-MS\脂肪酸\20250508-zfs\std1.D	校正	1	x	4563	0.1563	0.1977
F:\D\GC-MS\脂肪酸\20250508-zfs\std2.D	校正	2	x	10861	0.3125	0.1969
F:\D\GC-MS\脂肪酸\20250508-zfs\std3.D	校正	3	x	23940	0.6250	0.1968
F:\D\GC-MS\脂肪酸\20250508-zfs\std4.D	校正	4	x	50280	1.2500	0.1953
F:\D\GC-MS\脂肪酸\20250508-zfs\std5.D	校正	5	x	110720	2.5000	0.2051
F:\D\GC-MS\脂肪酸\20250508-zfs\std6.D	校正	6	x	240779	5.0000	0.1898
F:\D\GC-MS\脂肪酸\20250508-zfs\std7.D	校正	7	x	506735	10.0000	0.1923
F:\D\GC-MS\脂肪酸\20250508-zfs\std8.D	校正	8	x	1053004	20.0000	0.1799
F:\D\GC-MS\脂肪酸\20250508-zfs\std9.D	校正	9		2237317	40.0000	0.1596
F:\D\GC-MS\脂肪酸\20250508-zfs\std10.D	校正	10		4118789	80.0000	0.1263

**批处理路径**	G:\GC-MS\HX250430-4-GCMS总脂肪酸靶向检测\HX250430-4\QuantResults\HX250430-4.batch.bin		
**分析时间**	2025/5/14 16:58	**分析员姓名**	DESKTOP-M3A0GPO\omics
**报告时间**	2025/5/16 14:52:57	**报告员姓名**	DESKTOP-M3A0GPO\omics
**最近校正更新**	2025/5/14 16:58	**批处理状态**	已处理
**定量批处理版本**	10.2	**定量报告版本**	10.2

C18:0

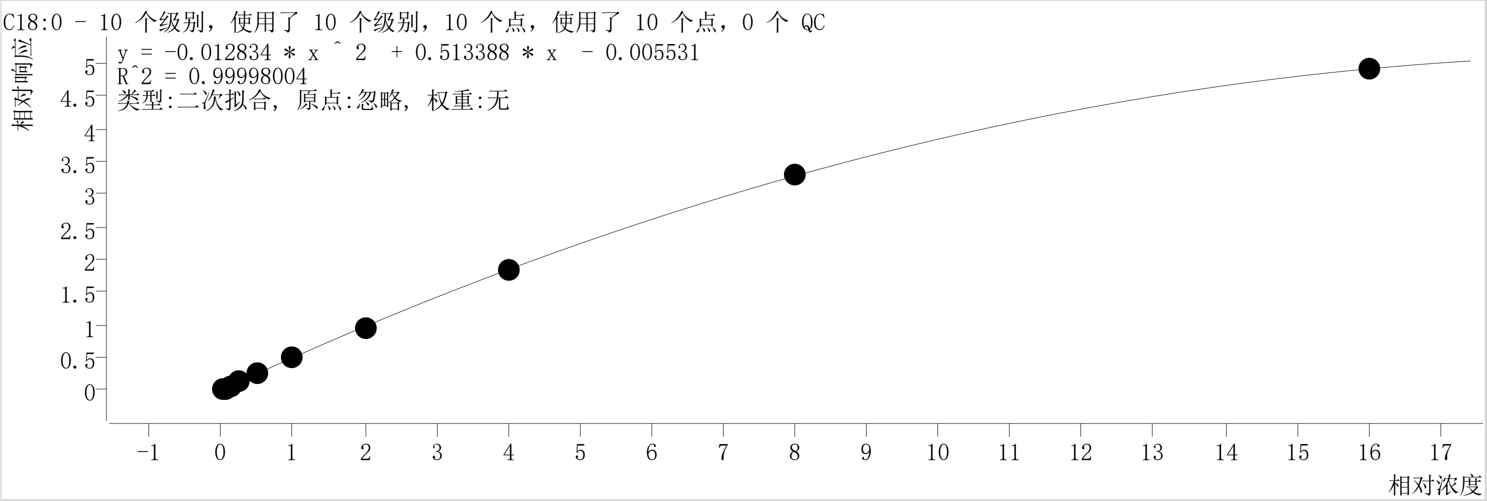

校正 STD 路径	校正类型	级别	已启用	响应	预期的浓度	响应因子
F:\D\GC-MS\脂肪酸\20250508-zfs\std1.D	校正	1	x	21989	0.3125	0.4765
F:\D\GC-MS\脂肪酸\20250508-zfs\std2.D	校正	2	x	51052	0.6250	0.4628
F:\D\GC-MS\脂肪酸\20250508-zfs\std3.D	校正	3	x	118731	1.2500	0.4881
F:\D\GC-MS\脂肪酸\20250508-zfs\std4.D	校正	4	x	245607	2.5000	0.4769
F:\D\GC-MS\脂肪酸\20250508-zfs\std5.D	校正	5	x	558555	5.0000	0.5173
F:\D\GC-MS\脂肪酸\20250508-zfs\std6.D	校正	6	x	1227136	10.0000	0.4836
F:\D\GC-MS\脂肪酸\20250508-zfs\std7.D	校正	7	x	2520868	20.0000	0.4782
F:\D\GC-MS\脂肪酸\20250508-zfs\std8.D	校正	8	x	5409063	40.0000	0.4619
F:\D\GC-MS\脂肪酸\20250508-zfs\std9.D	校正	9	x	11509999	80.0000	0.4105
F:\D\GC-MS\脂肪酸\20250508-zfs\std10.D	校正	10	x	20066589	160.0000	0.3076

**批处理路径**	G:\GC-MS\HX250430-4-GCMS总脂肪酸靶向检测\HX250430-4\QuantResults\HX250430-4.batch.bin		
**分析时间**	2025/5/14 16:58	**分析员姓名**	DESKTOP-M3A0GPO\omics
**报告时间**	2025/5/16 14:52:57	**报告员姓名**	DESKTOP-M3A0GPO\omics
**最近校正更新**	2025/5/14 16:58	**批处理状态**	已处理
**定量批处理版本**	10.2	**定量报告版本**	10.2

C18:1n9t

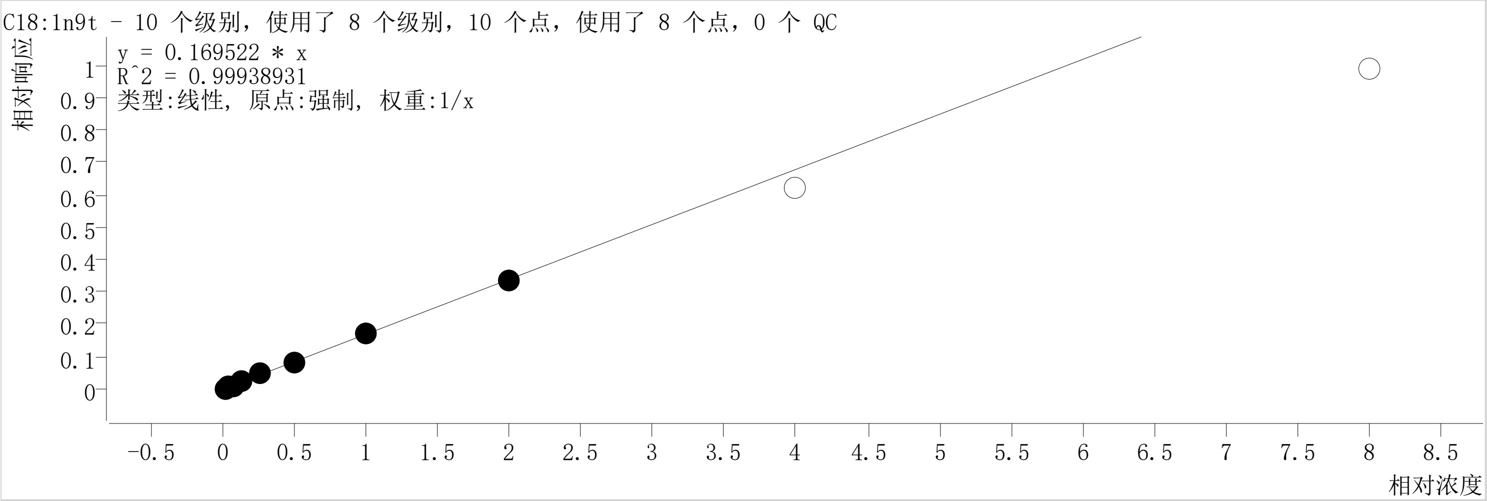

校正 STD 路径	校正类型	级别	已启用	响应	预期的浓度	响应因子
F:\D\GC-MS\脂肪酸\20250508-zfs\std1.D	校正	1	x	3449	0.1563	0.1495
F:\D\GC-MS\脂肪酸\20250508-zfs\std2.D	校正	2	x	8141	0.3125	0.1476
F:\D\GC-MS\脂肪酸\20250508-zfs\std3.D	校正	3	x	19084	0.6250	0.1569
F:\D\GC-MS\脂肪酸\20250508-zfs\std4.D	校正	4	x	41913	1.2500	0.1628
F:\D\GC-MS\脂肪酸\20250508-zfs\std5.D	校正	5	x	95905	2.5000	0.1777
F:\D\GC-MS\脂肪酸\20250508-zfs\std6.D	校正	6	x	214075	5.0000	0.1687
F:\D\GC-MS\脂肪酸\20250508-zfs\std7.D	校正	7	x	453645	10.0000	0.1721
F:\D\GC-MS\脂肪酸\20250508-zfs\std8.D	校正	8	x	987844	20.0000	0.1687
F:\D\GC-MS\脂肪酸\20250508-zfs\std9.D	校正	9		2162177	40.0000	0.1542
F:\D\GC-MS\脂肪酸\20250508-zfs\std10.D	校正	10		4028333	80.0000	0.1235

**批处理路径**	G:\GC-MS\HX250430-4-GCMS总脂肪酸靶向检测\HX250430-4\QuantResults\HX250430-4.batch.bin		
**分析时间**	2025/5/14 16:58	**分析员姓名**	DESKTOP-M3A0GPO\omics
**报告时间**	2025/5/16 14:52:57	**报告员姓名**	DESKTOP-M3A0GPO\omics
**最近校正更新**	2025/5/14 16:58	**批处理状态**	已处理
**定量批处理版本**	10.2	**定量报告版本**	10.2

C18:1n9c

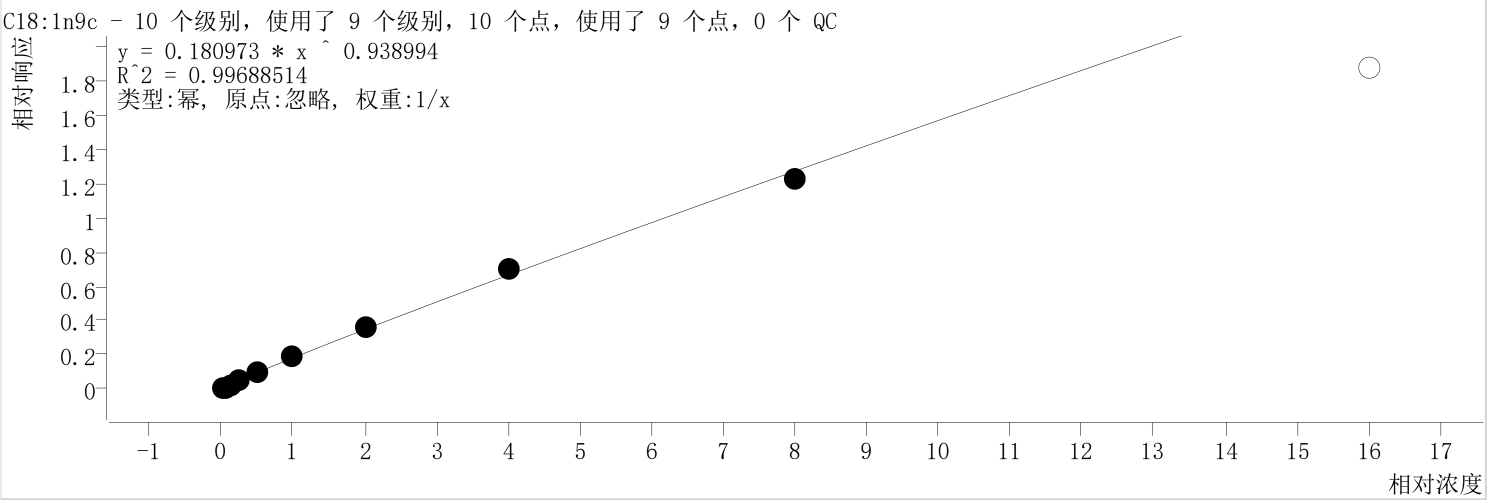

校正 STD 路径	校正类型	级别	已启用	响应	预期的浓度	响应因子
F:\D\GC-MS\脂肪酸\20250508-zfs\std1.D	校正	1	x	7714	0.3125	0.1671
F:\D\GC-MS\脂肪酸\20250508-zfs\std2.D	校正	2	x	18398	0.6250	0.1668
F:\D\GC-MS\脂肪酸\20250508-zfs\std3.D	校正	3	x	43086	1.2500	0.1771
F:\D\GC-MS\脂肪酸\20250508-zfs\std4.D	校正	4	x	91542	2.5000	0.1777
F:\D\GC-MS\脂肪酸\20250508-zfs\std5.D	校正	5	x	211106	5.0000	0.1955
F:\D\GC-MS\脂肪酸\20250508-zfs\std6.D	校正	6	x	465395	10.0000	0.1834
F:\D\GC-MS\脂肪酸\20250508-zfs\std7.D	校正	7	x	967718	20.0000	0.1836
F:\D\GC-MS\脂肪酸\20250508-zfs\std8.D	校正	8	x	2049949	40.0000	0.1751
F:\D\GC-MS\脂肪酸\20250508-zfs\std9.D	校正	9	x	4300626	80.0000	0.1534
F:\D\GC-MS\脂肪酸\20250508-zfs\std10.D	校正	10		7665882	160.0000	0.1175

**批处理路径**	G:\GC-MS\HX250430-4-GCMS总脂肪酸靶向检测\HX250430-4\QuantResults\HX250430-4.batch.bin		
**分析时间**	2025/5/14 16:58	**分析员姓名**	DESKTOP-M3A0GPO\omics
**报告时间**	2025/5/16 14:52:57	**报告员姓名**	DESKTOP-M3A0GPO\omics
**最近校正更新**	2025/5/14 16:58	**批处理状态**	已处理
**定量批处理版本**	10.2	**定量报告版本**	10.2

C18:2n6t

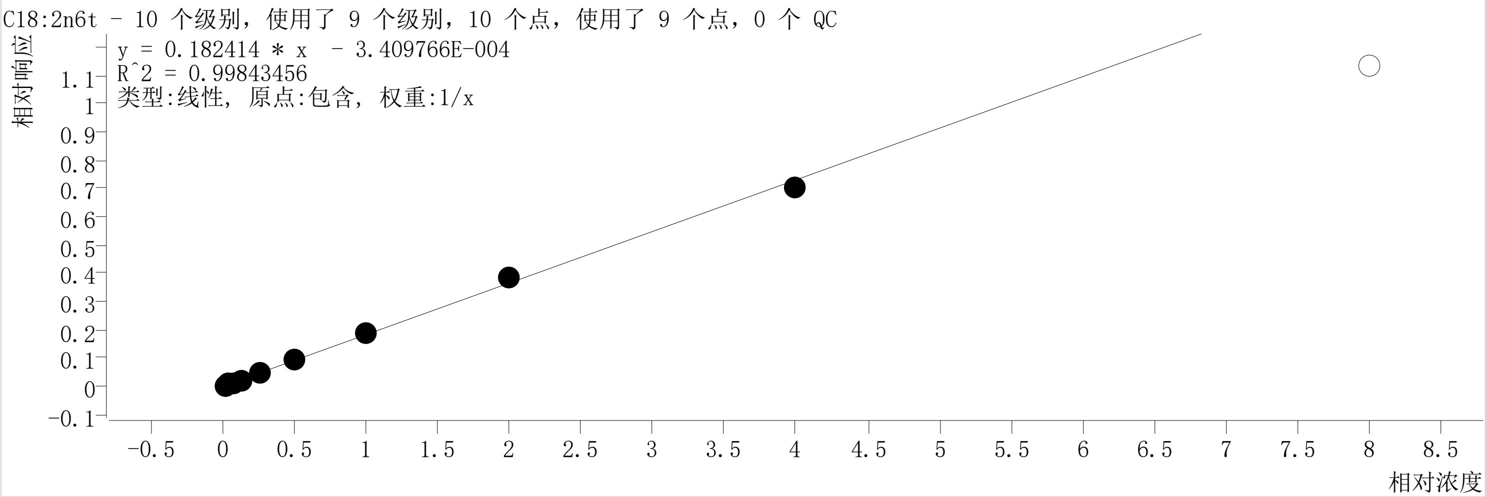

校正 STD 路径	校正类型	级别	已启用	响应	预期的浓度	响应因子
F:\D\GC-MS\脂肪酸\20250508-zfs\std1.D	校正	1	x	3724	0.1563	0.1614
F:\D\GC-MS\脂肪酸\20250508-zfs\std2.D	校正	2	x	9023	0.3125	0.1636
F:\D\GC-MS\脂肪酸\20250508-zfs\std3.D	校正	3	x	20734	0.6250	0.1705
F:\D\GC-MS\脂肪酸\20250508-zfs\std4.D	校正	4	x	44231	1.2500	0.1718
F:\D\GC-MS\脂肪酸\20250508-zfs\std5.D	校正	5	x	102750	2.5000	0.1903
F:\D\GC-MS\脂肪酸\20250508-zfs\std6.D	校正	6	x	233418	5.0000	0.1840
F:\D\GC-MS\脂肪酸\20250508-zfs\std7.D	校正	7	x	503880	10.0000	0.1912
F:\D\GC-MS\脂肪酸\20250508-zfs\std8.D	校正	8	x	1109192	20.0000	0.1894
F:\D\GC-MS\脂肪酸\20250508-zfs\std9.D	校正	9	x	2467607	40.0000	0.1760
F:\D\GC-MS\脂肪酸\20250508-zfs\std10.D	校正	10		4616889	80.0000	0.1416

**批处理路径**	G:\GC-MS\HX250430-4-GCMS总脂肪酸靶向检测\HX250430-4\QuantResults\HX250430-4.batch.bin		
**分析时间**	2025/5/14 16:58	**分析员姓名**	DESKTOP-M3A0GPO\omics
**报告时间**	2025/5/16 14:52:57	**报告员姓名**	DESKTOP-M3A0GPO\omics
**最近校正更新**	2025/5/14 16:58	**批处理状态**	已处理
**定量批处理版本**	10.2	**定量报告版本**	10.2

C18:2n6c

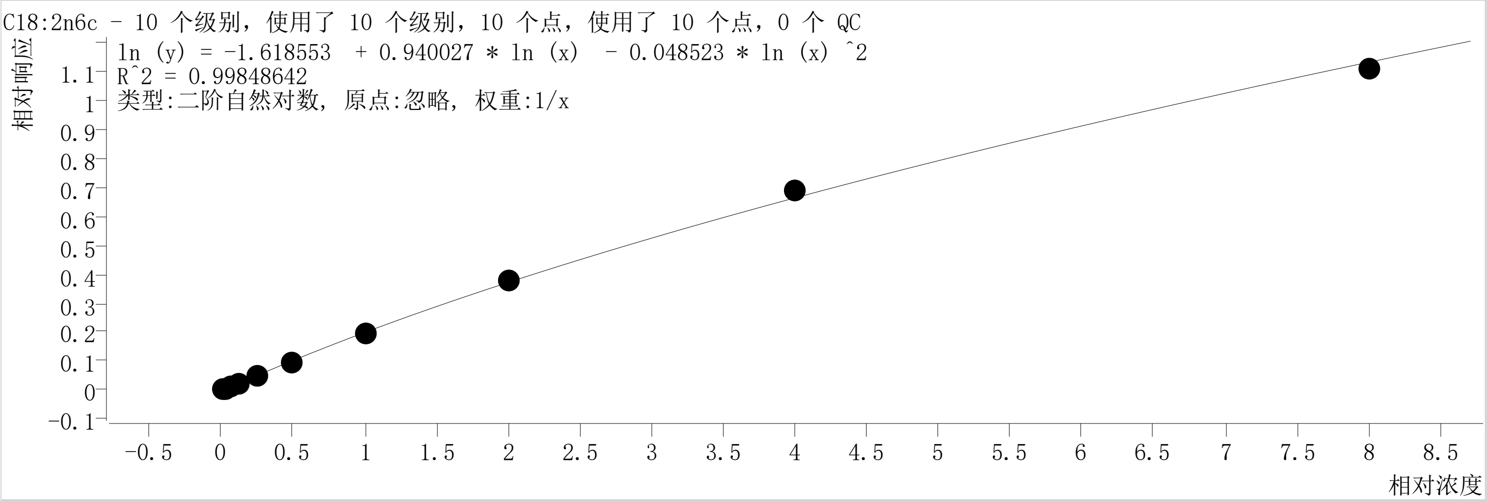

校正 STD 路径	校正类型	级别	已启用	响应	预期的浓度	响应因子
F:\D\GC-MS\脂肪酸\20250508-zfs\std1.D	校正	1	x	4037	0.1563	0.1750
F:\D\GC-MS\脂肪酸\20250508-zfs\std2.D	校正	2	x	9822	0.3125	0.1781
F:\D\GC-MS\脂肪酸\20250508-zfs\std3.D	校正	3	x	22033	0.6250	0.1812
F:\D\GC-MS\脂肪酸\20250508-zfs\std4.D	校正	4	x	45969	1.2500	0.1785
F:\D\GC-MS\脂肪酸\20250508-zfs\std5.D	校正	5	x	106206	2.5000	0.1967
F:\D\GC-MS\脂肪酸\20250508-zfs\std6.D	校正	6	x	236172	5.0000	0.1861
F:\D\GC-MS\脂肪酸\20250508-zfs\std7.D	校正	7	x	506011	10.0000	0.1920
F:\D\GC-MS\脂肪酸\20250508-zfs\std8.D	校正	8	x	1101305	20.0000	0.1881
F:\D\GC-MS\脂肪酸\20250508-zfs\std9.D	校正	9	x	2429948	40.0000	0.1733
F:\D\GC-MS\脂肪酸\20250508-zfs\std10.D	校正	10	x	4531701	80.0000	0.1390

**批处理路径**	G:\GC-MS\HX250430-4-GCMS总脂肪酸靶向检测\HX250430-4\QuantResults\HX250430-4.batch.bin		
**分析时间**	2025/5/14 16:58	**分析员姓名**	DESKTOP-M3A0GPO\omics
**报告时间**	2025/5/16 14:52:57	**报告员姓名**	DESKTOP-M3A0GPO\omics
**最近校正更新**	2025/5/14 16:58	**批处理状态**	已处理
**定量批处理版本**	10.2	**定量报告版本**	10.2

C18:3n6

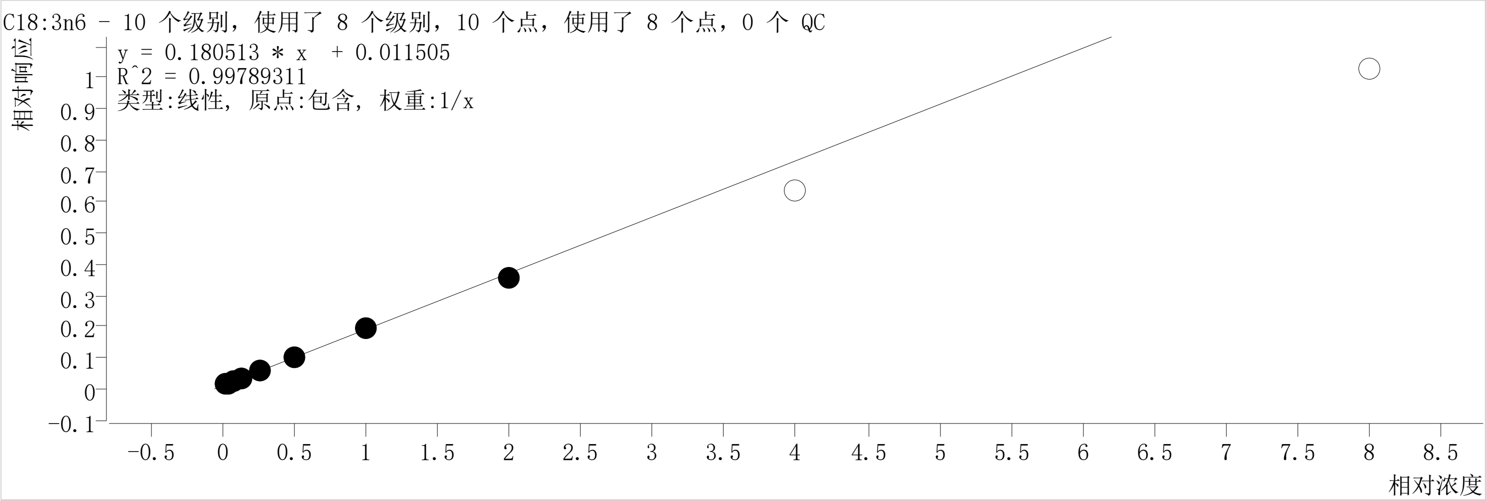

校正 STD 路径	校正类型	级别	已启用	响应	预期的浓度	响应因子
F:\D\GC-MS\脂肪酸\20250508-zfs\std1.D	校正	1	x	20243	0.1563	0.8773
F:\D\GC-MS\脂肪酸\20250508-zfs\std2.D	校正	2	x	30134	0.3125	0.5463
F:\D\GC-MS\脂肪酸\20250508-zfs\std3.D	校正	3	x	45753	0.6250	0.3762
F:\D\GC-MS\脂肪酸\20250508-zfs\std4.D	校正	4	x	72173	1.2500	0.2803
F:\D\GC-MS\脂肪酸\20250508-zfs\std5.D	校正	5	x	133432	2.5000	0.2472
F:\D\GC-MS\脂肪酸\20250508-zfs\std6.D	校正	6	x	263826	5.0000	0.2079
F:\D\GC-MS\脂肪酸\20250508-zfs\std7.D	校正	7	x	515357	10.0000	0.1955
F:\D\GC-MS\脂肪酸\20250508-zfs\std8.D	校正	8	x	1055796	20.0000	0.1803
F:\D\GC-MS\脂肪酸\20250508-zfs\std9.D	校正	9		2245396	40.0000	0.1601
F:\D\GC-MS\脂肪酸\20250508-zfs\std10.D	校正	10		4193173	80.0000	0.1286

**批处理路径**	G:\GC-MS\HX250430-4-GCMS总脂肪酸靶向检测\HX250430-4\QuantResults\HX250430-4.batch.bin		
**分析时间**	2025/5/14 16:58	**分析员姓名**	DESKTOP-M3A0GPO\omics
**报告时间**	2025/5/16 14:52:57	**报告员姓名**	DESKTOP-M3A0GPO\omics
**最近校正更新**	2025/5/14 16:58	**批处理状态**	已处理
**定量批处理版本**	10.2	**定量报告版本**	10.2

C18:3n3

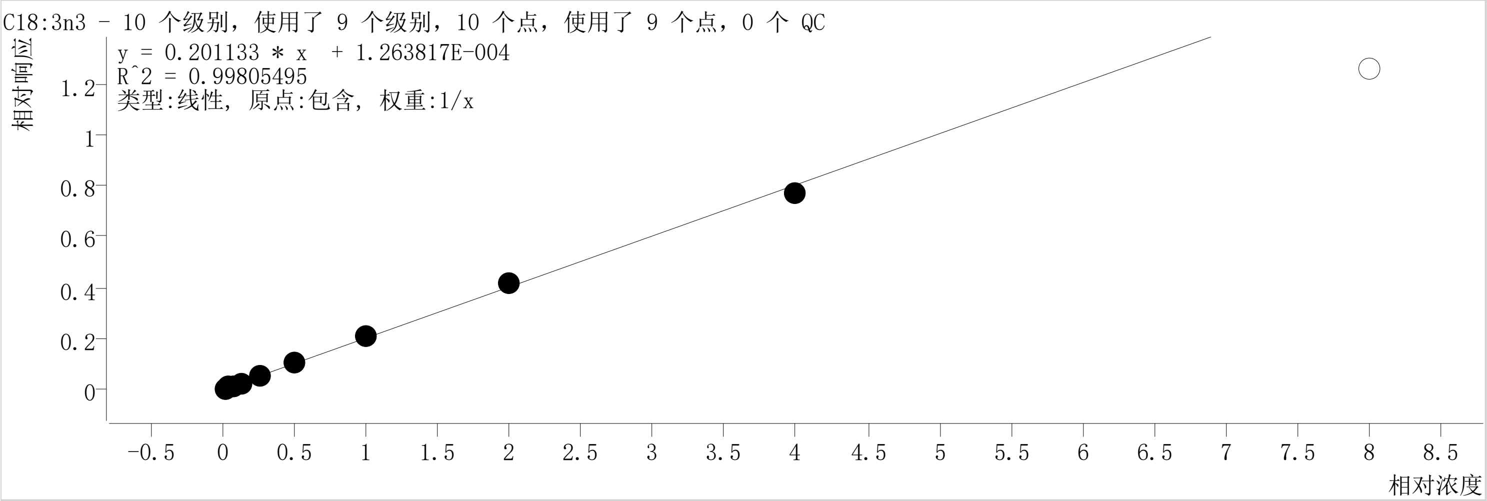

校正 STD 路径	校正类型	级别	已启用	响应	预期的浓度	响应因子
F:\D\GC-MS\脂肪酸\20250508-zfs\std1.D	校正	1	x	4574	0.1563	0.1982
F:\D\GC-MS\脂肪酸\20250508-zfs\std2.D	校正	2	x	10535	0.3125	0.1910
F:\D\GC-MS\脂肪酸\20250508-zfs\std3.D	校正	3	x	24443	0.6250	0.2010
F:\D\GC-MS\脂肪酸\20250508-zfs\std4.D	校正	4	x	50702	1.2500	0.1969
F:\D\GC-MS\脂肪酸\20250508-zfs\std5.D	校正	5	x	117286	2.5000	0.2173
F:\D\GC-MS\脂肪酸\20250508-zfs\std6.D	校正	6	x	263848	5.0000	0.2079
F:\D\GC-MS\脂肪酸\20250508-zfs\std7.D	校正	7	x	558658	10.0000	0.2120
F:\D\GC-MS\脂肪酸\20250508-zfs\std8.D	校正	8	x	1222850	20.0000	0.2089
F:\D\GC-MS\脂肪酸\20250508-zfs\std9.D	校正	9	x	2708947	40.0000	0.1932
F:\D\GC-MS\脂肪酸\20250508-zfs\std10.D	校正	10		5142622	80.0000	0.1577

**批处理路径**	G:\GC-MS\HX250430-4-GCMS总脂肪酸靶向检测\HX250430-4\QuantResults\HX250430-4.batch.bin		
**分析时间**	2025/5/14 16:58	**分析员姓名**	DESKTOP-M3A0GPO\omics
**报告时间**	2025/5/16 14:52:57	**报告员姓名**	DESKTOP-M3A0GPO\omics
**最近校正更新**	2025/5/14 16:58	**批处理状态**	已处理
**定量批处理版本**	10.2	**定量报告版本**	10.2

C20:0

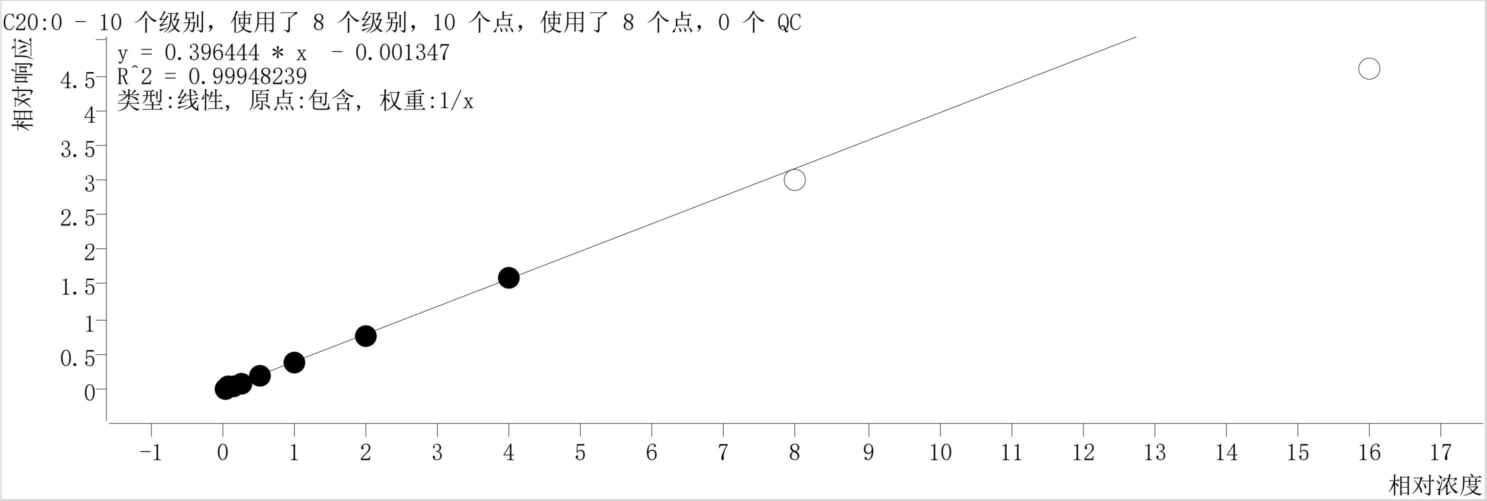

校正 STD 路径	校正类型	级别	已启用	响应	预期的浓度	响应因子
F:\D\GC-MS\脂肪酸\20250508-zfs\std1.D	校正	1	x	18408	0.3125	0.3989
F:\D\GC-MS\脂肪酸\20250508-zfs\std2.D	校正	2	x	39690	0.6250	0.3598
F:\D\GC-MS\脂肪酸\20250508-zfs\std3.D	校正	3	x	90943	1.2500	0.3739
F:\D\GC-MS\脂肪酸\20250508-zfs\std4.D	校正	4	x	189204	2.5000	0.3674
F:\D\GC-MS\脂肪酸\20250508-zfs\std5.D	校正	5	x	437966	5.0000	0.4056
F:\D\GC-MS\脂肪酸\20250508-zfs\std6.D	校正	6	x	992176	10.0000	0.3910
F:\D\GC-MS\脂肪酸\20250508-zfs\std7.D	校正	7	x	2043689	20.0000	0.3877
F:\D\GC-MS\脂肪酸\20250508-zfs\std8.D	校正	8	x	4700404	40.0000	0.4014
F:\D\GC-MS\脂肪酸\20250508-zfs\std9.D	校正	9		10467244	80.0000	0.3733
F:\D\GC-MS\脂肪酸\20250508-zfs\std10.D	校正	10		18731212	160.0000	0.2872

**批处理路径**	G:\GC-MS\HX250430-4-GCMS总脂肪酸靶向检测\HX250430-4\QuantResults\HX250430-4.batch.bin		
**分析时间**	2025/5/14 16:58	**分析员姓名**	DESKTOP-M3A0GPO\omics
**报告时间**	2025/5/16 14:52:57	**报告员姓名**	DESKTOP-M3A0GPO\omics
**最近校正更新**	2025/5/14 16:58	**批处理状态**	已处理
**定量批处理版本**	10.2	**定量报告版本**	10.2

C20:1

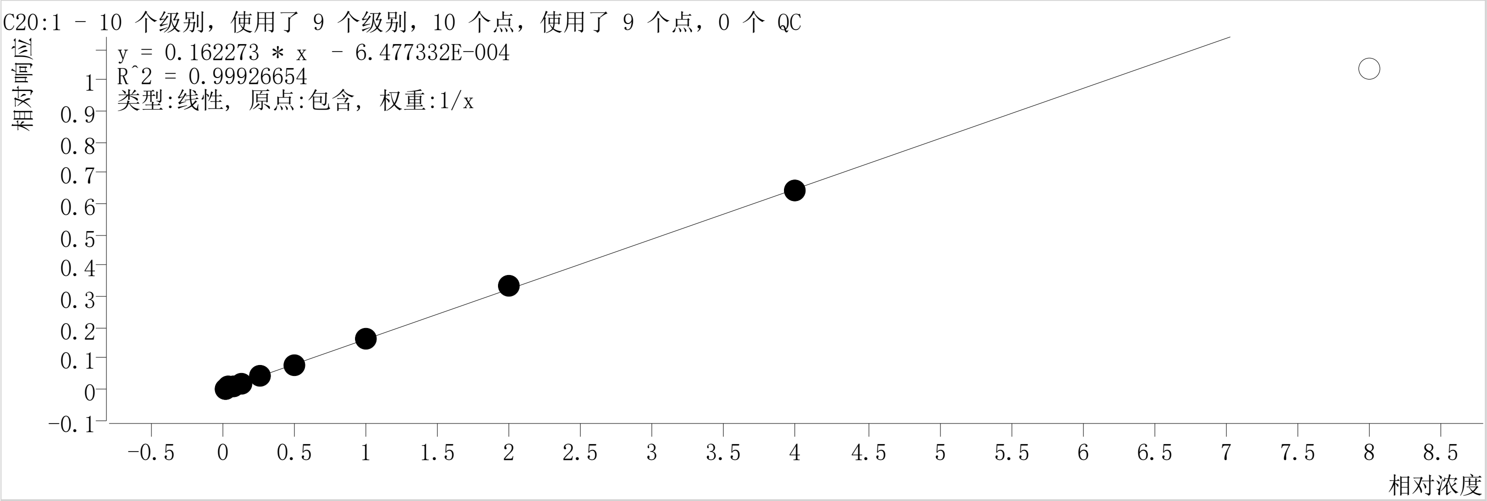

校正 STD 路径	校正类型	级别	已启用	响应	预期的浓度	响应因子
F:\D\GC-MS\脂肪酸\20250508-zfs\std1.D	校正	1	x	3434	0.1563	0.1488
F:\D\GC-MS\脂肪酸\20250508-zfs\std2.D	校正	2	x	7385	0.3125	0.1339
F:\D\GC-MS\脂肪酸\20250508-zfs\std3.D	校正	3	x	17812	0.6250	0.1465
F:\D\GC-MS\脂肪酸\20250508-zfs\std4.D	校正	4	x	37218	1.2500	0.1445
F:\D\GC-MS\脂肪酸\20250508-zfs\std5.D	校正	5	x	86339	2.5000	0.1599
F:\D\GC-MS\脂肪酸\20250508-zfs\std6.D	校正	6	x	197112	5.0000	0.1554
F:\D\GC-MS\脂肪酸\20250508-zfs\std7.D	校正	7	x	421746	10.0000	0.1600
F:\D\GC-MS\脂肪酸\20250508-zfs\std8.D	校正	8	x	984696	20.0000	0.1682
F:\D\GC-MS\脂肪酸\20250508-zfs\std9.D	校正	9	x	2250474	40.0000	0.1605
F:\D\GC-MS\脂肪酸\20250508-zfs\std10.D	校正	10		4227381	80.0000	0.1296

**批处理路径**	G:\GC-MS\HX250430-4-GCMS总脂肪酸靶向检测\HX250430-4\QuantResults\HX250430-4.batch.bin		
**分析时间**	2025/5/14 16:58	**分析员姓名**	DESKTOP-M3A0GPO\omics
**报告时间**	2025/5/16 14:52:57	**报告员姓名**	DESKTOP-M3A0GPO\omics
**最近校正更新**	2025/5/14 16:58	**批处理状态**	已处理
**定量批处理版本**	10.2	**定量报告版本**	10.2

C20:2

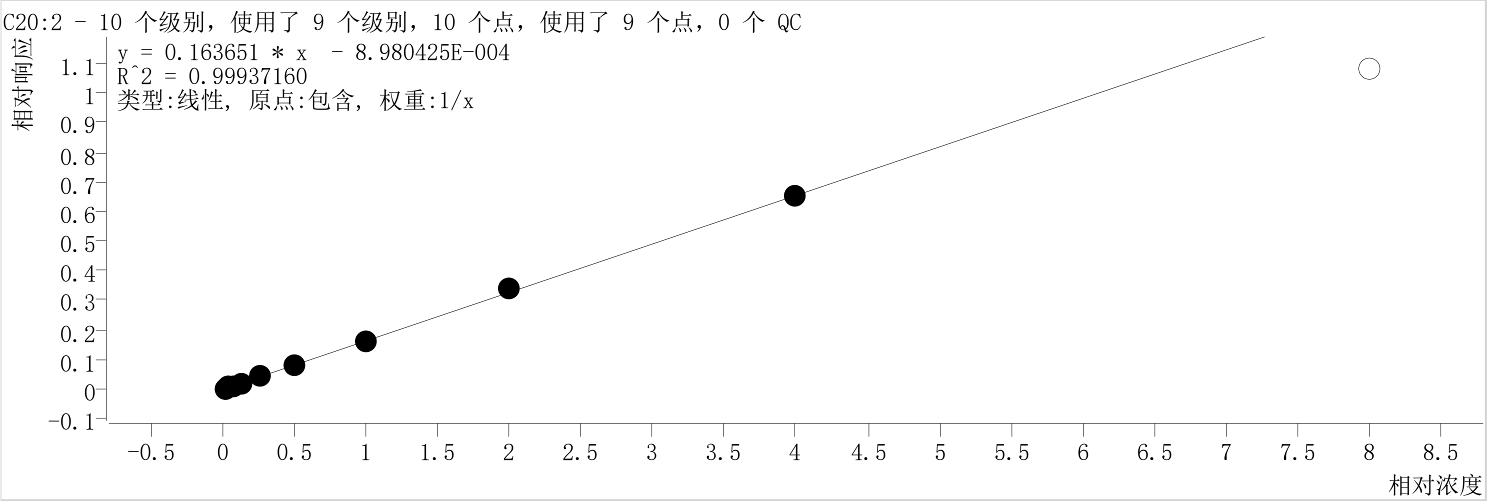

校正 STD 路径	校正类型	级别	已启用	响应	预期的浓度	响应因子
F:\D\GC-MS\脂肪酸\20250508-zfs\std1.D	校正	1	x	3149	0.1563	0.1365
F:\D\GC-MS\脂肪酸\20250508-zfs\std2.D	校正	2	x	7375	0.3125	0.1337
F:\D\GC-MS\脂肪酸\20250508-zfs\std3.D	校正	3	x	17325	0.6250	0.1425
F:\D\GC-MS\脂肪酸\20250508-zfs\std4.D	校正	4	x	36144	1.2500	0.1404
F:\D\GC-MS\脂肪酸\20250508-zfs\std5.D	校正	5	x	85641	2.5000	0.1586
F:\D\GC-MS\脂肪酸\20250508-zfs\std6.D	校正	6	x	197958	5.0000	0.1560
F:\D\GC-MS\脂肪酸\20250508-zfs\std7.D	校正	7	x	419918	10.0000	0.1593
F:\D\GC-MS\脂肪酸\20250508-zfs\std8.D	校正	8	x	983181	20.0000	0.1679
F:\D\GC-MS\脂肪酸\20250508-zfs\std9.D	校正	9	x	2288759	40.0000	0.1632
F:\D\GC-MS\脂肪酸\20250508-zfs\std10.D	校正	10		4407678	80.0000	0.1351

**批处理路径**	G:\GC-MS\HX250430-4-GCMS总脂肪酸靶向检测\HX250430-4\QuantResults\HX250430-4.batch.bin		
**分析时间**	2025/5/14 16:58	**分析员姓名**	DESKTOP-M3A0GPO\omics
**报告时间**	2025/5/16 14:52:57	**报告员姓名**	DESKTOP-M3A0GPO\omics
**最近校正更新**	2025/5/14 16:58	**批处理状态**	已处理
**定量批处理版本**	10.2	**定量报告版本**	10.2

C21:0

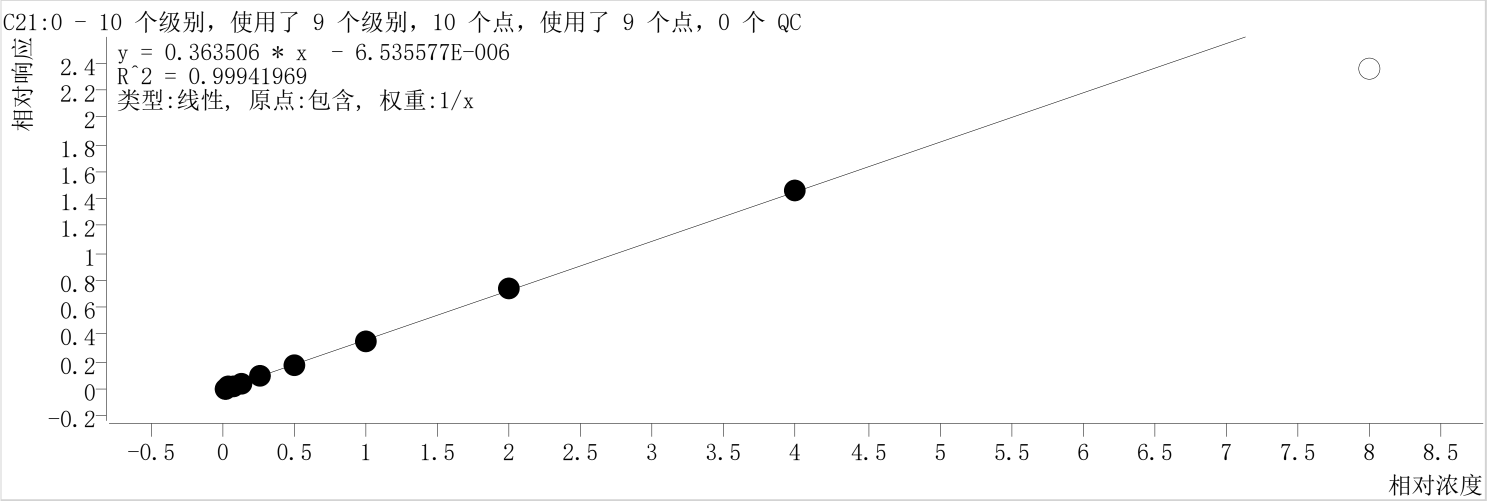

校正 STD 路径	校正类型	级别	已启用	响应	预期的浓度	响应因子
F:\D\GC-MS\脂肪酸\20250508-zfs\std1.D	校正	1	x	10026	0.1563	0.4345
F:\D\GC-MS\脂肪酸\20250508-zfs\std2.D	校正	2	x	19624	0.3125	0.3558
F:\D\GC-MS\脂肪酸\20250508-zfs\std3.D	校正	3	x	41873	0.6250	0.3443
F:\D\GC-MS\脂肪酸\20250508-zfs\std4.D	校正	4	x	86000	1.2500	0.3340
F:\D\GC-MS\脂肪酸\20250508-zfs\std5.D	校正	5	x	196118	2.5000	0.3633
F:\D\GC-MS\脂肪酸\20250508-zfs\std6.D	校正	6	x	443818	5.0000	0.3498
F:\D\GC-MS\脂肪酸\20250508-zfs\std7.D	校正	7	x	929280	10.0000	0.3526
F:\D\GC-MS\脂肪酸\20250508-zfs\std8.D	校正	8	x	2175424	20.0000	0.3716
F:\D\GC-MS\脂肪酸\20250508-zfs\std9.D	校正	9	x	5116545	40.0000	0.3649
F:\D\GC-MS\脂肪酸\20250508-zfs\std10.D	校正	10		9619014	80.0000	0.2949

**批处理路径**	G:\GC-MS\HX250430-4-GCMS总脂肪酸靶向检测\HX250430-4\QuantResults\HX250430-4.batch.bin		
**分析时间**	2025/5/14 16:58	**分析员姓名**	DESKTOP-M3A0GPO\omics
**报告时间**	2025/5/16 14:52:57	**报告员姓名**	DESKTOP-M3A0GPO\omics
**最近校正更新**	2025/5/14 16:58	**批处理状态**	已处理
**定量批处理版本**	10.2	**定量报告版本**	10.2

C20:3n6

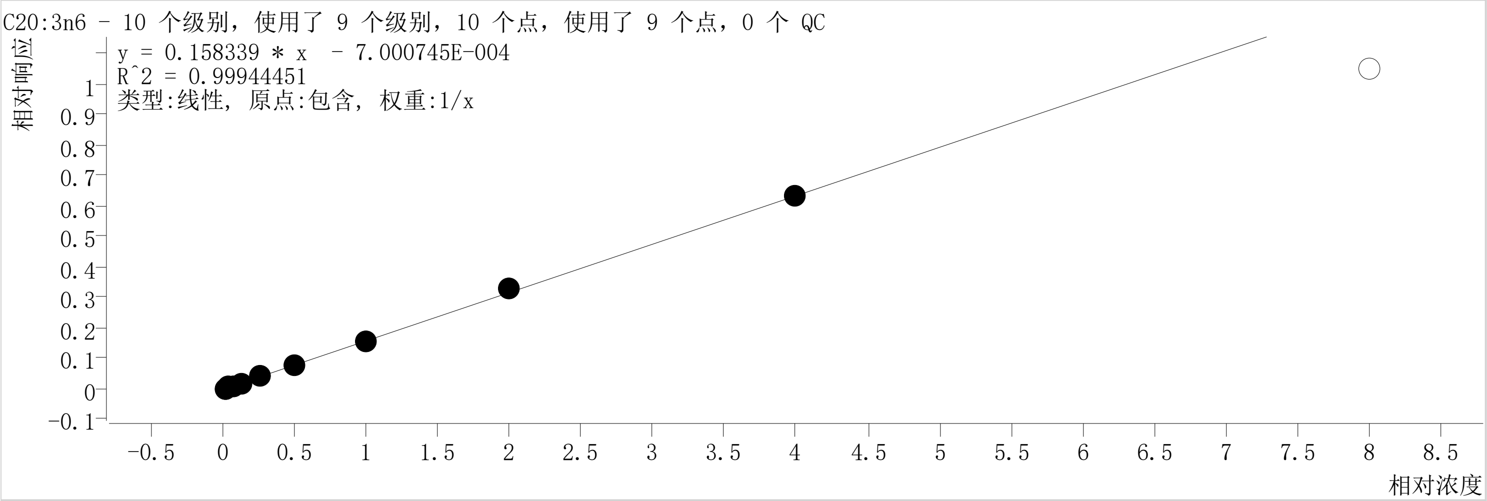

校正 STD 路径	校正类型	级别	已启用	响应	预期的浓度	响应因子
F:\D\GC-MS\脂肪酸\20250508-zfs\std1.D	校正	1	x	3263	0.1563	0.1414
F:\D\GC-MS\脂肪酸\20250508-zfs\std2.D	校正	2	x	7286	0.3125	0.1321
F:\D\GC-MS\脂肪酸\20250508-zfs\std3.D	校正	3	x	17167	0.6250	0.1412
F:\D\GC-MS\脂肪酸\20250508-zfs\std4.D	校正	4	x	35762	1.2500	0.1389
F:\D\GC-MS\脂肪酸\20250508-zfs\std5.D	校正	5	x	83881	2.5000	0.1554
F:\D\GC-MS\脂肪酸\20250508-zfs\std6.D	校正	6	x	193575	5.0000	0.1526
F:\D\GC-MS\脂肪酸\20250508-zfs\std7.D	校正	7	x	405416	10.0000	0.1538
F:\D\GC-MS\脂肪酸\20250508-zfs\std8.D	校正	8	x	950912	20.0000	0.1624
F:\D\GC-MS\脂肪酸\20250508-zfs\std9.D	校正	9	x	2213995	40.0000	0.1579
F:\D\GC-MS\脂肪酸\20250508-zfs\std10.D	校正	10		4273562	80.0000	0.1310

**批处理路径**	G:\GC-MS\HX250430-4-GCMS总脂肪酸靶向检测\HX250430-4\QuantResults\HX250430-4.batch.bin		
**分析时间**	2025/5/14 16:58	**分析员姓名**	DESKTOP-M3A0GPO\omics
**报告时间**	2025/5/16 14:52:57	**报告员姓名**	DESKTOP-M3A0GPO\omics
**最近校正更新**	2025/5/14 16:58	**批处理状态**	已处理
**定量批处理版本**	10.2	**定量报告版本**	10.2

C20:4n6

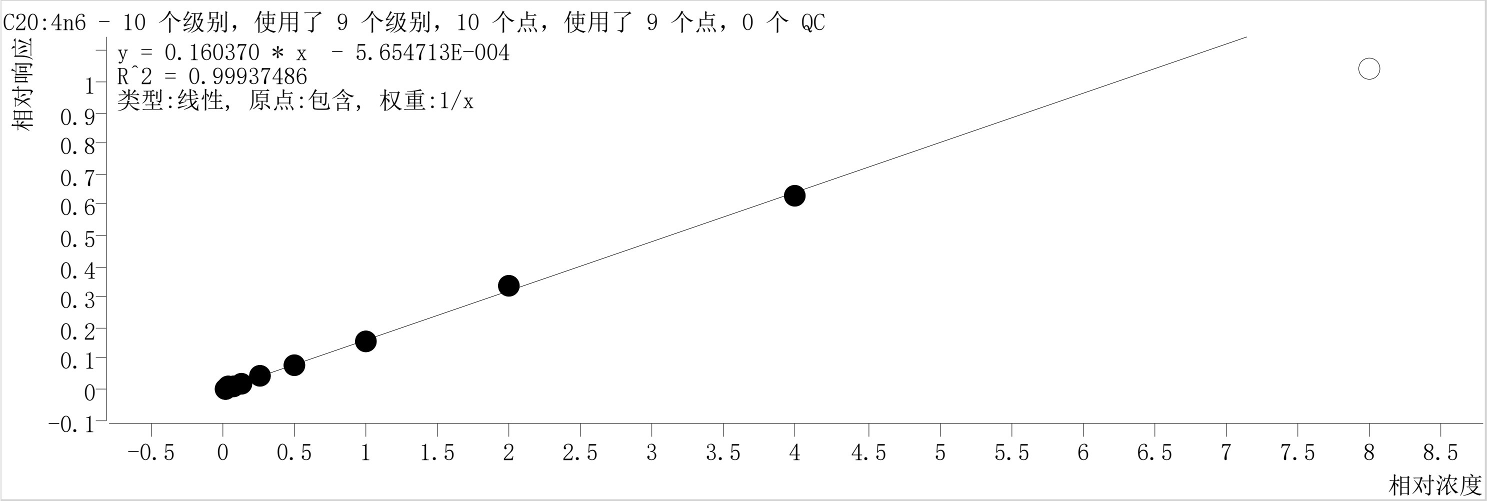

校正 STD 路径	校正类型	级别	已启用	响应	预期的浓度	响应因子
F:\D\GC-MS\脂肪酸\20250508-zfs\std1.D	校正	1	x	3306	0.1563	0.1433
F:\D\GC-MS\脂肪酸\20250508-zfs\std2.D	校正	2	x	7641	0.3125	0.1385
F:\D\GC-MS\脂肪酸\20250508-zfs\std3.D	校正	3	x	17658	0.6250	0.1452
F:\D\GC-MS\脂肪酸\20250508-zfs\std4.D	校正	4	x	37388	1.2500	0.1452
F:\D\GC-MS\脂肪酸\20250508-zfs\std5.D	校正	5	x	85894	2.5000	0.1591
F:\D\GC-MS\脂肪酸\20250508-zfs\std6.D	校正	6	x	199708	5.0000	0.1574
F:\D\GC-MS\脂肪酸\20250508-zfs\std7.D	校正	7	x	416629	10.0000	0.1581
F:\D\GC-MS\脂肪酸\20250508-zfs\std8.D	校正	8	x	972850	20.0000	0.1662
F:\D\GC-MS\脂肪酸\20250508-zfs\std9.D	校正	9	x	2217714	40.0000	0.1582
F:\D\GC-MS\脂肪酸\20250508-zfs\std10.D	校正	10		4247049	80.0000	0.1302

**批处理路径**	G:\GC-MS\HX250430-4-GCMS总脂肪酸靶向检测\HX250430-4\QuantResults\HX250430-4.batch.bin		
**分析时间**	2025/5/14 16:58	**分析员姓名**	DESKTOP-M3A0GPO\omics
**报告时间**	2025/5/16 14:52:57	**报告员姓名**	DESKTOP-M3A0GPO\omics
**最近校正更新**	2025/5/14 16:58	**批处理状态**	已处理
**定量批处理版本**	10.2	**定量报告版本**	10.2

C20:3n3

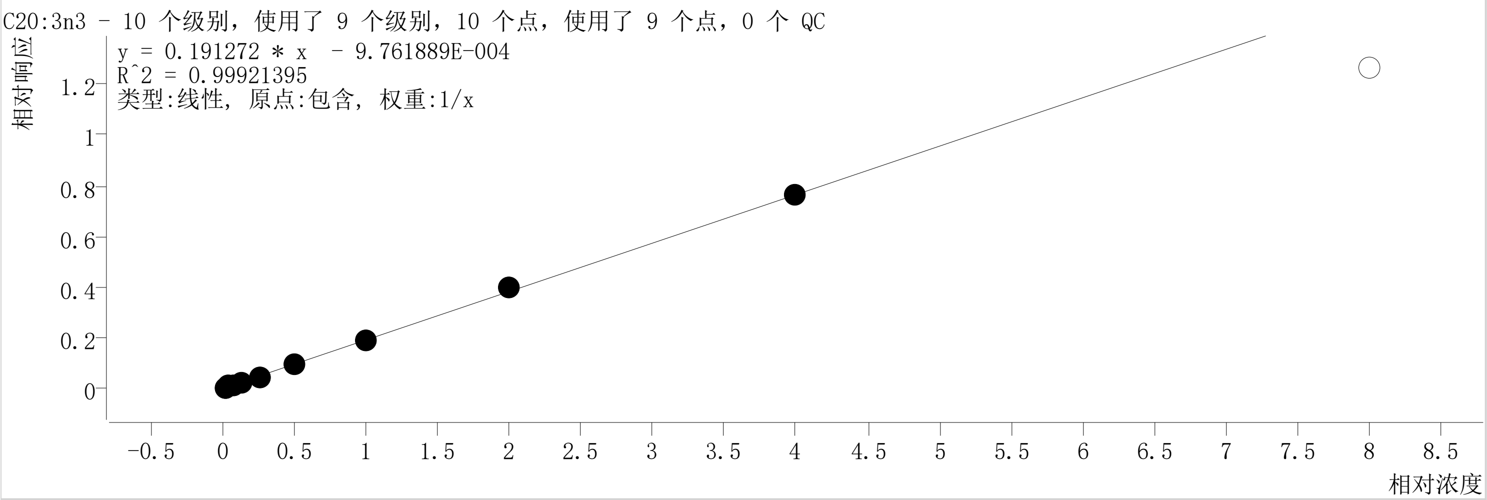

校正 STD 路径	校正类型	级别	已启用	响应	预期的浓度	响应因子
F:\D\GC-MS\脂肪酸\20250508-zfs\std1.D	校正	1	x	3891	0.1563	0.1686
F:\D\GC-MS\脂肪酸\20250508-zfs\std2.D	校正	2	x	8777	0.3125	0.1591
F:\D\GC-MS\脂肪酸\20250508-zfs\std3.D	校正	3	x	20073	0.6250	0.1651
F:\D\GC-MS\脂肪酸\20250508-zfs\std4.D	校正	4	x	42681	1.2500	0.1657
F:\D\GC-MS\脂肪酸\20250508-zfs\std5.D	校正	5	x	99056	2.5000	0.1835
F:\D\GC-MS\脂肪酸\20250508-zfs\std6.D	校正	6	x	231764	5.0000	0.1827
F:\D\GC-MS\脂肪酸\20250508-zfs\std7.D	校正	7	x	484842	10.0000	0.1840
F:\D\GC-MS\脂肪酸\20250508-zfs\std8.D	校正	8	x	1152681	20.0000	0.1969
F:\D\GC-MS\脂肪酸\20250508-zfs\std9.D	校正	9	x	2680917	40.0000	0.1912
F:\D\GC-MS\脂肪酸\20250508-zfs\std10.D	校正	10		5157652	80.0000	0.1581

批处理路径	G:\GC-MS\HX250430-4-GCMS总脂肪酸靶向检测\HX250430-4\QuantResults\HX250430-4.batch.bin		
分析时间	2025/5/14 16:58	分析员姓名	DESKTOP-M3A0GPO\omics
报告时间	2025/5/16 14:52:57	报告员姓名	DESKTOP-M3A0GPO\omics
最近校正更新	2025/5/14 16:58	批处理状态	已处理
定量批处理版本	10.2	定量报告版本	10.2

C20:5n3

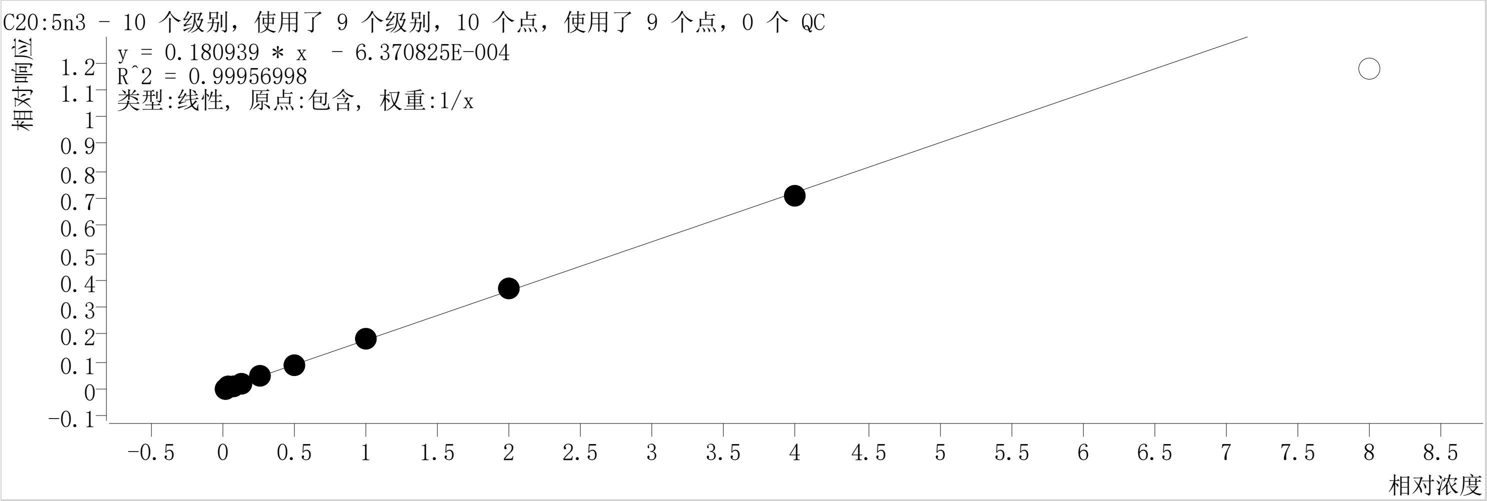

校正 STD 路径	校正类型	级别	已启用	响应	预期的浓度	响应因子
F:\D\GC-MS\脂肪酸\20250508-zfs\std1.D	校正	1	x	3469	0.1563	0.1503
F:\D\GC-MS\脂肪酸\20250508-zfs\std2.D	校正	2	x	8699	0.3125	0.1577
F:\D\GC-MS\脂肪酸\20250508-zfs\std3.D	校正	3	x	20490	0.6250	0.1685
F:\D\GC-MS\脂肪酸\20250508-zfs\std4.D	校正	4	x	42763	1.2500	0.1661
F:\D\GC-MS\脂肪酸\20250508-zfs\std5.D	校正	5	x	98917	2.5000	0.1832
F:\D\GC-MS\脂肪酸\20250508-zfs\std6.D	校正	6	x	224693	5.0000	0.1771
F:\D\GC-MS\脂肪酸\20250508-zfs\std7.D	校正	7	x	473008	10.0000	0.1795
F:\D\GC-MS\脂肪酸\20250508-zfs\std8.D	校正	8	x	1089649	20.0000	0.1861
F:\D\GC-MS\脂肪酸\20250508-zfs\std9.D	校正	9	x	2503902	40.0000	0.1786
F:\D\GC-MS\脂肪酸\20250508-zfs\std10.D	校正	10		4794968	80.0000	0.1470

**批处理路径**	G:\GC-MS\HX250430-4-GCMS总脂肪酸靶向检测\HX250430-4\QuantResults\HX250430-4.batch.bin		
**分析时间**	2025/5/14 16:58	**分析员姓名**	DESKTOP-M3A0GPO\omics
**报告时间**	2025/5/16 14:52:57	**报告员姓名**	DESKTOP-M3A0GPO\omics
**最近校正更新**	2025/5/14 16:58	**批处理状态**	已处理
**定量批处理版本**	10.2	**定量报告版本**	10.2

C22:0

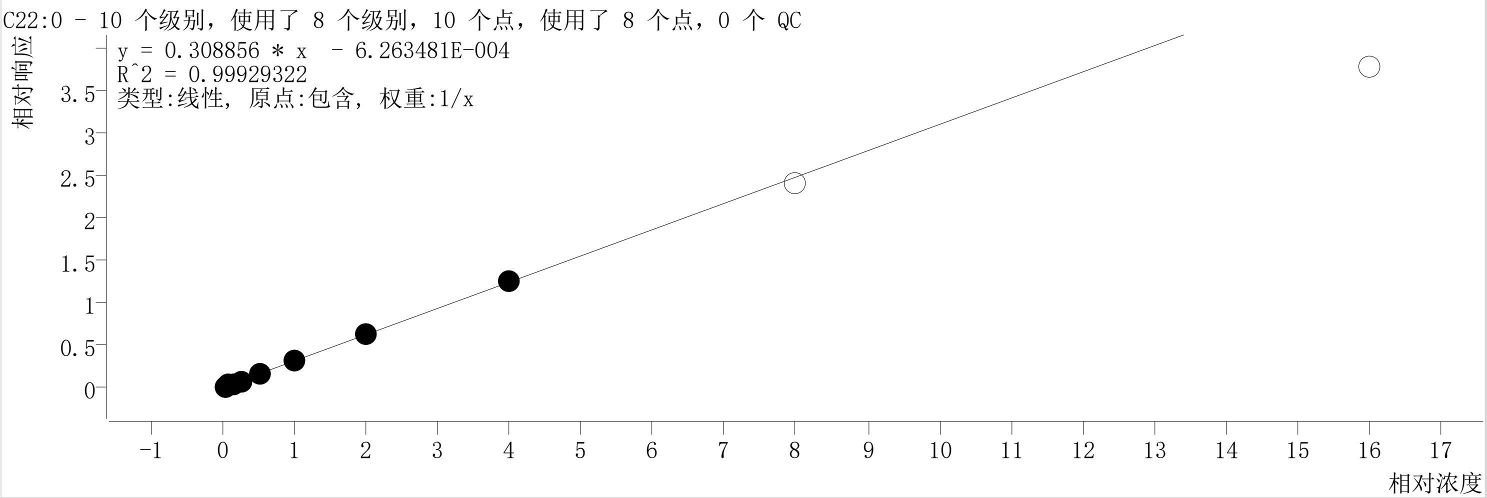

校正 STD 路径	校正类型	级别	已启用	响应	预期的浓度	响应因子
F:\D\GC-MS\脂肪酸\20250508-zfs\std1.D	校正	1	x	15503	0.3125	0.3359
F:\D\GC-MS\脂肪酸\20250508-zfs\std2.D	校正	2	x	31532	0.6250	0.2858
F:\D\GC-MS\脂肪酸\20250508-zfs\std3.D	校正	3	x	69692	1.2500	0.2865
F:\D\GC-MS\脂肪酸\20250508-zfs\std4.D	校正	4	x	150469	2.5000	0.2922
F:\D\GC-MS\脂肪酸\20250508-zfs\std5.D	校正	5	x	339739	5.0000	0.3147
F:\D\GC-MS\脂肪酸\20250508-zfs\std6.D	校正	6	x	757374	10.0000	0.2985
F:\D\GC-MS\脂肪酸\20250508-zfs\std7.D	校正	7	x	1597788	20.0000	0.3031
F:\D\GC-MS\脂肪酸\20250508-zfs\std8.D	校正	8	x	3679650	40.0000	0.3142
F:\D\GC-MS\脂肪酸\20250508-zfs\std9.D	校正	9		8406927	80.0000	0.2998
F:\D\GC-MS\脂肪酸\20250508-zfs\std10.D	校正	10		15354227	160.0000	0.2354

**批处理路径**	G:\GC-MS\HX250430-4-GCMS总脂肪酸靶向检测\HX250430-4\QuantResults\HX250430-4.batch.bin		
**分析时间**	2025/5/14 16:58	**分析员姓名**	DESKTOP-M3A0GPO\omics
**报告时间**	2025/5/16 14:52:57	**报告员姓名**	DESKTOP-M3A0GPO\omics
**最近校正更新**	2025/5/14 16:58	**批处理状态**	已处理
**定量批处理版本**	10.2	**定量报告版本**	10.2

C22:1n9

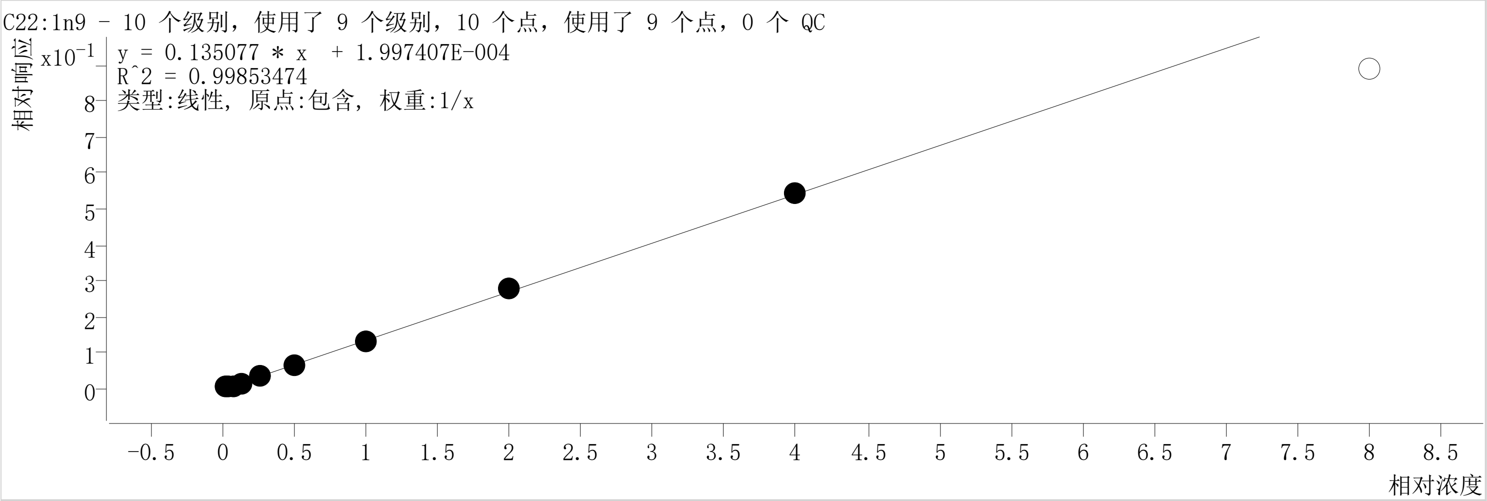

校正 STD 路径	校正类型	级别	已启用	响应	预期的浓度	响应因子
F:\D\GC-MS\脂肪酸\20250508-zfs\std1.D	校正	1	x	4762	0.1563	0.2064
F:\D\GC-MS\脂肪酸\20250508-zfs\std2.D	校正	2	x	6620	0.3125	0.1200
F:\D\GC-MS\脂肪酸\20250508-zfs\std3.D	校正	3	x	14742	0.6250	0.1212
F:\D\GC-MS\脂肪酸\20250508-zfs\std4.D	校正	4	x	32144	1.2500	0.1248
F:\D\GC-MS\脂肪酸\20250508-zfs\std5.D	校正	5	x	72217	2.5000	0.1338
F:\D\GC-MS\脂肪酸\20250508-zfs\std6.D	校正	6	x	162904	5.0000	0.1284
F:\D\GC-MS\脂肪酸\20250508-zfs\std7.D	校正	7	x	345729	10.0000	0.1312
F:\D\GC-MS\脂肪酸\20250508-zfs\std8.D	校正	8	x	820737	20.0000	0.1402
F:\D\GC-MS\脂肪酸\20250508-zfs\std9.D	校正	9	x	1896201	40.0000	0.1352
F:\D\GC-MS\脂肪酸\20250508-zfs\std10.D	校正	10		3624168	80.0000	0.1111

**批处理路径**	G:\GC-MS\HX250430-4-GCMS总脂肪酸靶向检测\HX250430-4\QuantResults\HX250430-4.batch.bin		
**分析时间**	2025/5/14 16:58	**分析员姓名**	DESKTOP-M3A0GPO\omics
**报告时间**	2025/5/16 14:52:57	**报告员姓名**	DESKTOP-M3A0GPO\omics
**最近校正更新**	2025/5/14 16:58	**批处理状态**	已处理
**定量批处理版本**	10.2	**定量报告版本**	10.2

C22:2n6

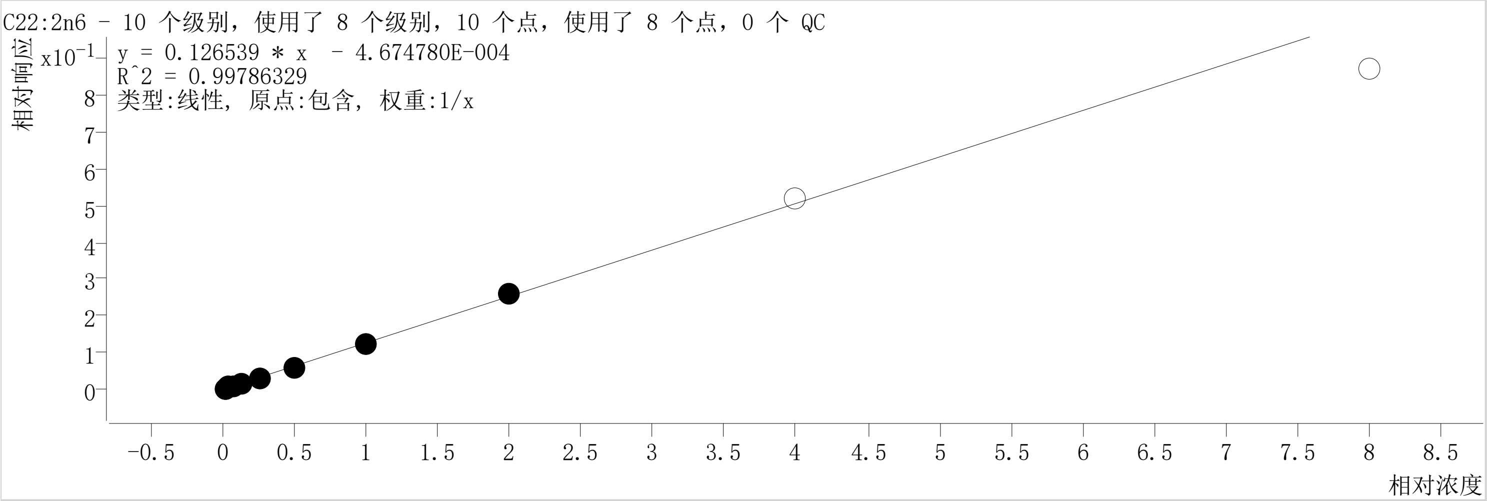

校正 STD 路径	校正类型	级别	已启用	响应	预期的浓度	响应因子
F:\D\GC-MS\脂肪酸\20250508-zfs\std1.D	校正	1	x	2958	0.1563	0.1282
F:\D\GC-MS\脂肪酸\20250508-zfs\std2.D	校正	2	x	6142	0.3125	0.1114
F:\D\GC-MS\脂肪酸\20250508-zfs\std3.D	校正	3	x	13309	0.6250	0.1094
F:\D\GC-MS\脂肪酸\20250508-zfs\std4.D	校正	4	x	28462	1.2500	0.1105
F:\D\GC-MS\脂肪酸\20250508-zfs\std5.D	校正	5	x	64810	2.5000	0.1201
F:\D\GC-MS\脂肪酸\20250508-zfs\std6.D	校正	6	x	151778	5.0000	0.1196
F:\D\GC-MS\脂肪酸\20250508-zfs\std7.D	校正	7	x	322970	10.0000	0.1225
F:\D\GC-MS\脂肪酸\20250508-zfs\std8.D	校正	8	x	766807	20.0000	0.1310
F:\D\GC-MS\脂肪酸\20250508-zfs\std9.D	校正	9		1830434	40.0000	0.1306
F:\D\GC-MS\脂肪酸\20250508-zfs\std10.D	校正	10		3557120	80.0000	0.1091

定量分析完成报告

**批处理路径**	G:\GC-MS\HX250430-4-GCMS总脂肪酸靶向检测\HX250430-4\QuantResults\HX250430-4.batch.bin		
**分析时间**	2025/5/14 16:58	**分析员姓名**	DESKTOP-M3A0GPO\omics
**报告时间**	2025/5/16 14:52:57	**报告员姓名**	DESKTOP-M3A0GPO\omics
**最近校正更新**	2025/5/14 16:58	**批处理状态**	已处理
**定量批处理版本**	10.2	**定量报告版本**	10.2

C23:0

C23:0 - 10 个级别，使用了 9 个级别，10 个点，使用了 9 个点，0 个 QC

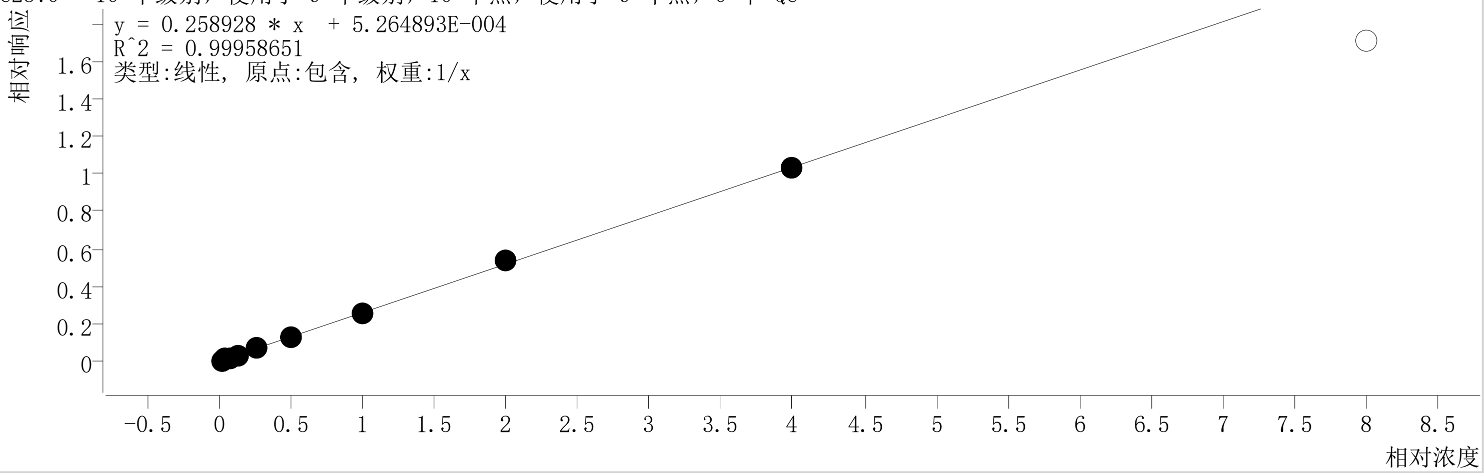

校正 STD 路径	校正类型	级别	已启用	响应	预期的浓度	响应因子
F:\D\GC-MS\脂肪酸\20250508-zfs\std1.D	校正	1	x	7416	0.1563	0.3214
F:\D\GC-MS\脂肪酸\20250508-zfs\std2.D	校正	2	x	14948	0.3125	0.2710
F:\D\GC-MS\脂肪酸\20250508-zfs\std3.D	校正	3	x	30777	0.6250	0.2531
F:\D\GC-MS\脂肪酸\20250508-zfs\std4.D	校正	4	x	65099	1.2500	0.2528
F:\D\GC-MS\脂肪酸\20250508-zfs\std5.D	校正	5	x	143829	2.5000	0.2664
F:\D\GC-MS\脂肪酸\20250508-zfs\std6.D	校正	6	x	317103	5.0000	0.2499
F:\D\GC-MS\脂肪酸\20250508-zfs\std7.D	校正	7	x	685843	10.0000	0.2602
F:\D\GC-MS\脂肪酸\20250508-zfs\std8.D	校正	8	x	1556318	20.0000	0.2658
F:\D\GC-MS\脂肪酸\20250508-zfs\std9.D	校正	9	x	3602567	40.0000	0.2569
F:\D\GC-MS\脂肪酸\20250508-zfs\std10.D	校正	10		6976080	80.0000	0.2139

**批处理路径**	G:\GC-MS\HX250430-4-GCMS总脂肪酸靶向检测\HX250430-4\QuantResults\HX250430-4.batch.bin		
**分析时间**	2025/5/14 16:58	**分析员姓名**	DESKTOP-M3A0GPO\omics
**报告时间**	2025/5/16 14:52:57	**报告员姓名**	DESKTOP-M3A0GPO\omics
**最近校正更新**	2025/5/14 16:58	**批处理状态**	已处理
**定量批处理版本**	10.2	**定量报告版本**	10.2

C24:0

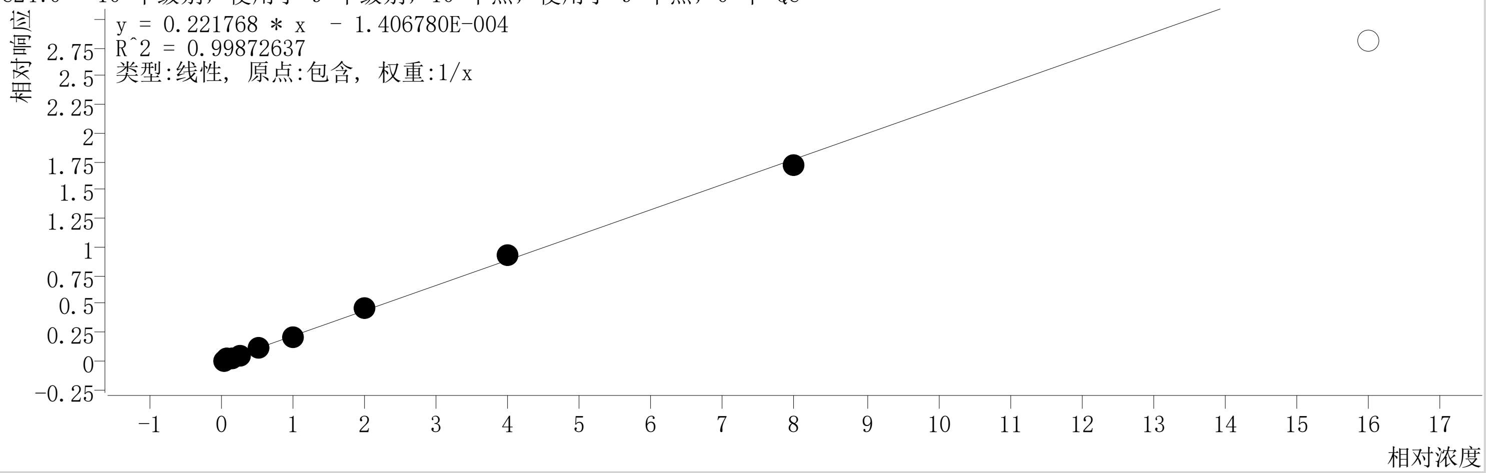

校正 STD 路径	校正类型	级别	已启用	响应	预期的浓度	响应因子
F:\D\GC-MS\脂肪酸\20250508-zfs\std1.D	校正	1	x	10067	0.3125	0.2181
F:\D\GC-MS\脂肪酸\20250508-zfs\std2.D	校正	2	x	24127	0.6250	0.2187
F:\D\GC-MS\脂肪酸\20250508-zfs\std3.D	校正	3	x	49962	1.2500	0.2054
F:\D\GC-MS\脂肪酸\20250508-zfs\std4.D	校正	4	x	112375	2.5000	0.2182
F:\D\GC-MS\脂肪酸\20250508-zfs\std5.D	校正	5	x	250237	5.0000	0.2318
F:\D\GC-MS\脂肪酸\20250508-zfs\std6.D	校正	6	x	553595	10.0000	0.2182
F:\D\GC-MS\脂肪酸\20250508-zfs\std7.D	校正	7	x	1216420	20.0000	0.2308
F:\D\GC-MS\脂肪酸\20250508-zfs\std8.D	校正	8	x	2700382	40.0000	0.2306
F:\D\GC-MS\脂肪酸\20250508-zfs\std9.D	校正	9	x	6033710	80.0000	0.2152
F:\D\GC-MS\脂肪酸\20250508-zfs\std10.D	校正	10		11459938	160.0000	0.1757

**批处理路径**	G:\GC-MS\HX250430-4-GCMS总脂肪酸靶向检测\HX250430-4\QuantResults\HX250430-4.batch.bin		
**分析时间**	2025/5/14 16:58	**分析员姓名**	DESKTOP-M3A0GPO\omics
**报告时间**	2025/5/16 14:52:57	**报告员姓名**	DESKTOP-M3A0GPO\omics
**最近校正更新**	2025/5/14 16:58	**批处理状态**	已处理
**定量批处理版本**	10.2	**定量报告版本**	10.2

C22:6

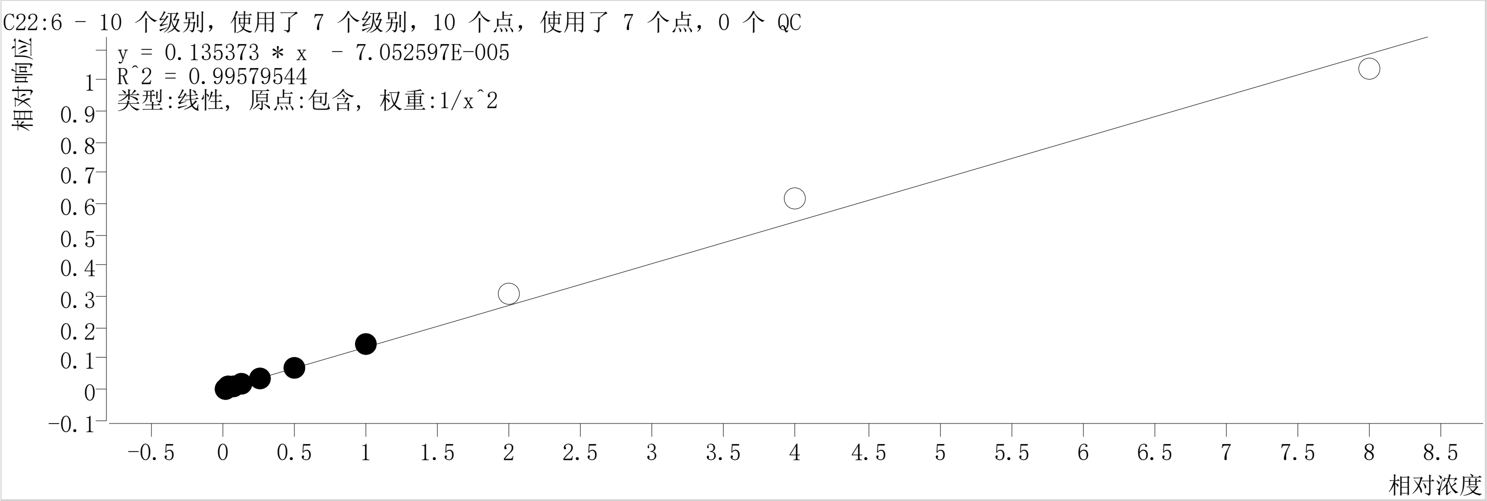

校正 STD 路径	校正类型	级别	已启用	响应	预期的浓度	响应因子
F:\D\GC-MS\脂肪酸\20250508-zfs\std1.D	校正	1	x	3129	0.1563	0.1356
F:\D\GC-MS\脂肪酸\20250508-zfs\std2.D	校正	2	x	7170	0.3125	0.1300
F:\D\GC-MS\脂肪酸\20250508-zfs\std3.D	校正	3	x	14842	0.6250	0.1220
F:\D\GC-MS\脂肪酸\20250508-zfs\std4.D	校正	4	x	33231	1.2500	0.1291
F:\D\GC-MS\脂肪酸\20250508-zfs\std5.D	校正	5	x	76310	2.5000	0.1414
F:\D\GC-MS\脂肪酸\20250508-zfs\std6.D	校正	6	x	175344	5.0000	0.1382
F:\D\GC-MS\脂肪酸\20250508-zfs\std7.D	校正	7	x	375373	10.0000	0.1424
F:\D\GC-MS\脂肪酸\20250508-zfs\std8.D	校正	8		896402	20.0000	0.1531
F:\D\GC-MS\脂肪酸\20250508-zfs\std9.D	校正	9		2149727	40.0000	0.1533
F:\D\GC-MS\脂肪酸\20250508-zfs\std10.D	校正	10		4220443	80.0000	0.1294

**批处理路径**	G:\GC-MS\HX250430-4-GCMS总脂肪酸靶向检测\HX250430-4\QuantResults\HX250430-4.batch.bin		
**分析时间**	2025/5/14 16:58	**分析员姓名**	DESKTOP-M3A0GPO\omics
**报告时间**	2025/5/16 14:52:57	**报告员姓名**	DESKTOP-M3A0GPO\omics
**最近校正更新**	2025/5/14 16:58	**批处理状态**	已处理
**定量批处理版本**	10.2	**定量报告版本**	10.2

C24:1

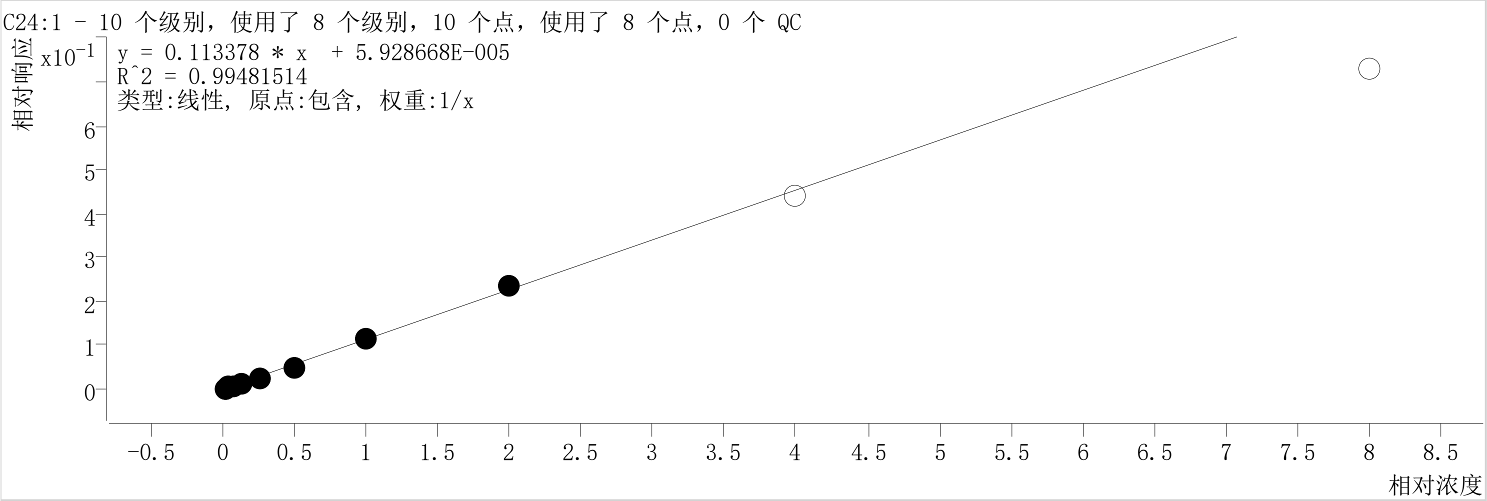

校正 STD 路径	校正类型	级别	已启用	响应	预期的浓度	响应因子
F:\D\GC-MS\脂肪酸\20250508-zfs\std1.D	校正	1	x	3307	0.1563	0.1433
F:\D\GC-MS\脂肪酸\20250508-zfs\std2.D	校正	2	x	7332	0.3125	0.1329
F:\D\GC-MS\脂肪酸\20250508-zfs\std3.D	校正	3	x	12172	0.6250	0.1001
F:\D\GC-MS\脂肪酸\20250508-zfs\std4.D	校正	4	x	26091	1.2500	0.1013
F:\D\GC-MS\脂肪酸\20250508-zfs\std5.D	校正	5	x	56528	2.5000	0.1047
F:\D\GC-MS\脂肪酸\20250508-zfs\std6.D	校正	6	x	124624	5.0000	0.0982
F:\D\GC-MS\脂肪酸\20250508-zfs\std7.D	校正	7	x	306721	10.0000	0.1164
F:\D\GC-MS\脂肪酸\20250508-zfs\std8.D	校正	8	x	688642	20.0000	0.1176
F:\D\GC-MS\脂肪酸\20250508-zfs\std9.D	校正	9		1537559	40.0000	0.1097
F:\D\GC-MS\脂肪酸\20250508-zfs\std10.D	校正	10		2974941	80.0000	0.0912

**批处理路径**	G:\GC-MS\HX250430-4-GCMS总脂肪酸靶向检测\HX250430-4\QuantResults\HX250430-4.batch.bin		
**分析时间**	2025/5/14 16:58	**分析员姓名**	DESKTOP-M3A0GPO\omics
**报告时间**	2025/5/16 14:52:57	**报告员姓名**	DESKTOP-M3A0GPO\omics
**最近校正更新**	2025/5/14 16:58	**批处理状态**	已处理
**定量批处理版本**	10.2	**定量报告版本**	10.2

**采集时间**	2025/5/8 17:08	**数据文件**	blank.D
**样品类型**	空白	**样品名称**	blank
**稀释**	1	**采集方法**	脂肪酸

样品色谱图

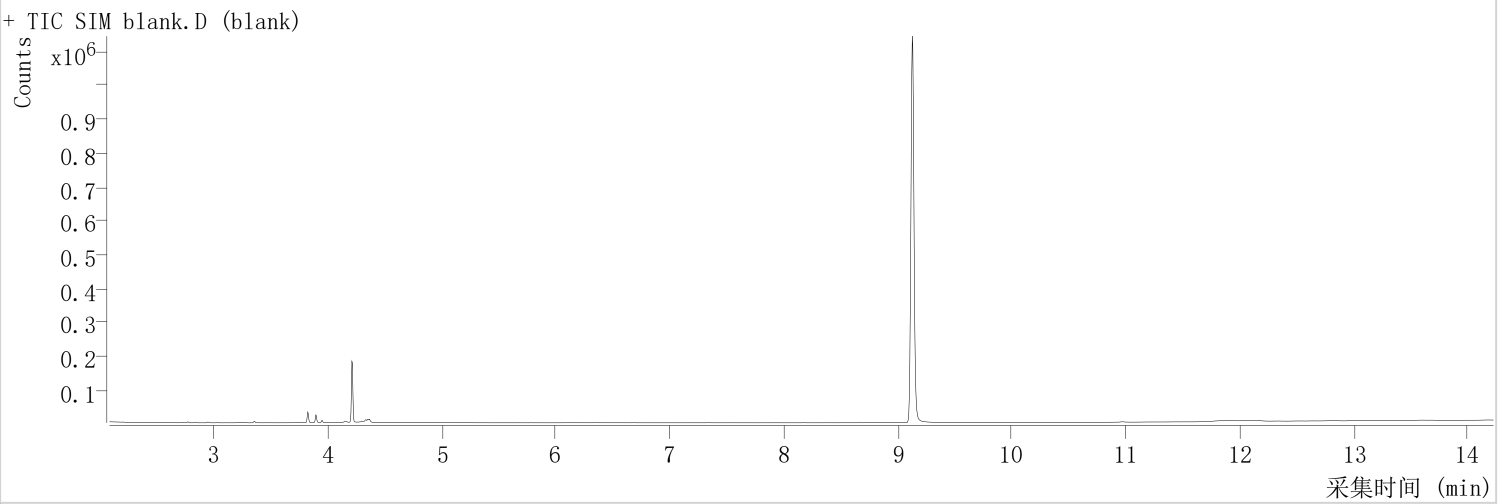

化合物	ISTD	RT	响应	ISTD 响应	响应比	最终浓度	单位
C4:0	C19:0	2.203	0	1614233	0.0000	ND	ug/ml
C6:0	C19:0	2.963	81	1614233	0.0001	0.0003	ug/ml
C8:0	C19:0	3.782	0	1614233	0.0000	ND	ug/ml
C10:0	C19:0	4.368	0	1614233	0.0000	ND	ug/ml
C11:0	C19:0	4.733	0	1614233	0.0000	ND	ug/ml
C12:0	C19:0	5.240	0	1614233	0.0000	ND	ug/ml
C13:0	C19:0	5.240	0	1614233	0.0000	ND	ug/ml
C14:0	C19:0	5.783	0	1614233	0.0000	ND	ug/ml
C14:1	C19:0			1614233		ND	ug/ml
C15:0	C19:0	6.103	0	1614233	0.0000	ND	ug/ml
C15:1	C19:0	6.352	0	1614233	0.0000	ND	ug/ml
C16:0	C19:0	6.690	0	1614233	0.0000	ND	ug/ml
C16:1	C19:0			1614233		ND	ug/ml
C17:0	C19:0	7.339	231	1614233	0.0001	0.0030	ug/ml
C17:1	C19:0			1614233		ND	ug/ml
C18:0	C19:0	8.179	209	1614233	0.0001	0.1103	ug/ml
C18:1n9t	C19:0			1614233		ND	ug/ml
C18:1n9c	C19:0			1614233		ND	ug/ml
C18:2n6t	C19:0			1614233		ND	ug/ml
C18:2n6c	C19:0	9.126	0	1614233	0.0000	ND	ug/ml
C18:3n6	C19:0	9.126	16700	1614233	0.0103	ND	ug/ml
C18:3n3	C19:0			1614233		ND	ug/ml
C20:0	C19:0	10.060	496	1614233	0.0003	0.0417	ug/ml
C20:1	C19:0			1614233		ND	ug/ml
C20:2	C19:0			1614233		ND	ug/ml
C21:0	C19:0	10.967	1672	1614233	0.0010	0.0287	ug/ml
C20:3n6	C19:0			1614233		ND	ug/ml
C20:4n6	C19:0			1614233		ND	ug/ml
C20:3n3	C19:0			1614233		ND	ug/ml
C20:5n3	C19:0	11.879	0	1614233	0.0000	ND	ug/ml

化合物	ISTD	RT	响应	ISTD 响应	响应比	最终浓度	单位
C22:0	C19:0			1614233		ND	ug/ml
C22:1n9	C19:0	12.132	0	1614233	0.0000	ND	ug/ml
C22:2n6	C19:0	12.808	0	1614233	0.0000	ND	ug/ml
C23:0	C19:0	12.639	0	1614233	0.0000	ND	ug/ml
C24:0	C19:0	13.417	0	1614233	0.0000	ND	ug/ml
C22:6	C19:0	13.426	0	1614233	0.0000	ND	ug/ml
C24:1	C19:0	13.671	0	1614233	0.0000	ND	ug/ml

C4:0

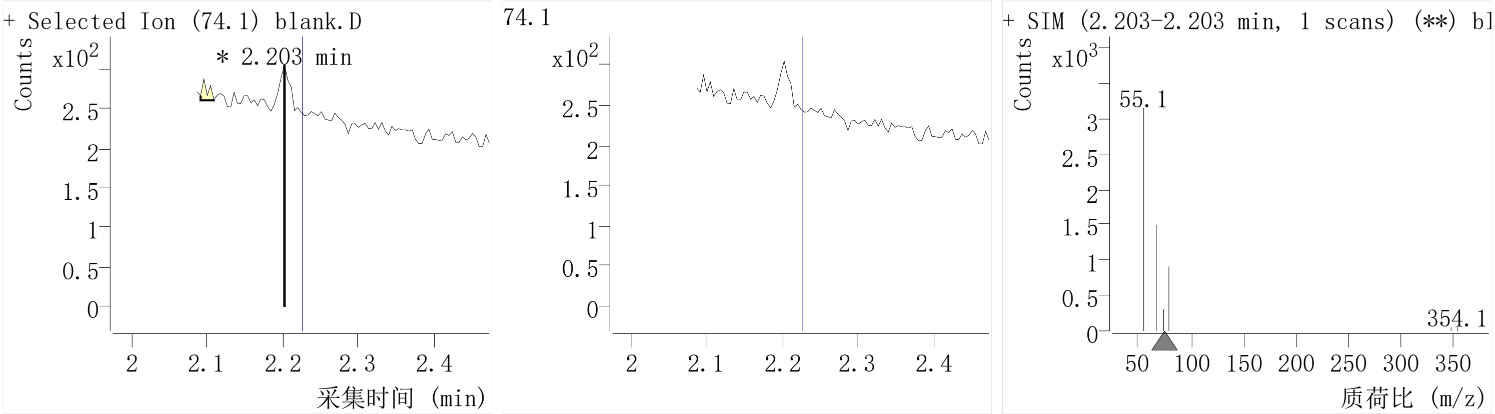

C6:0

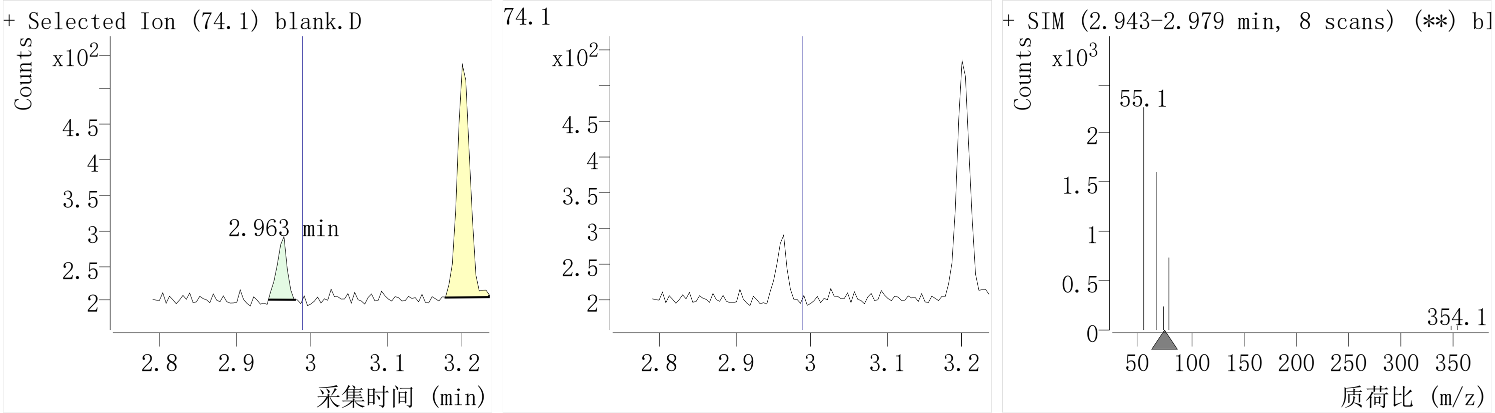

C8:0

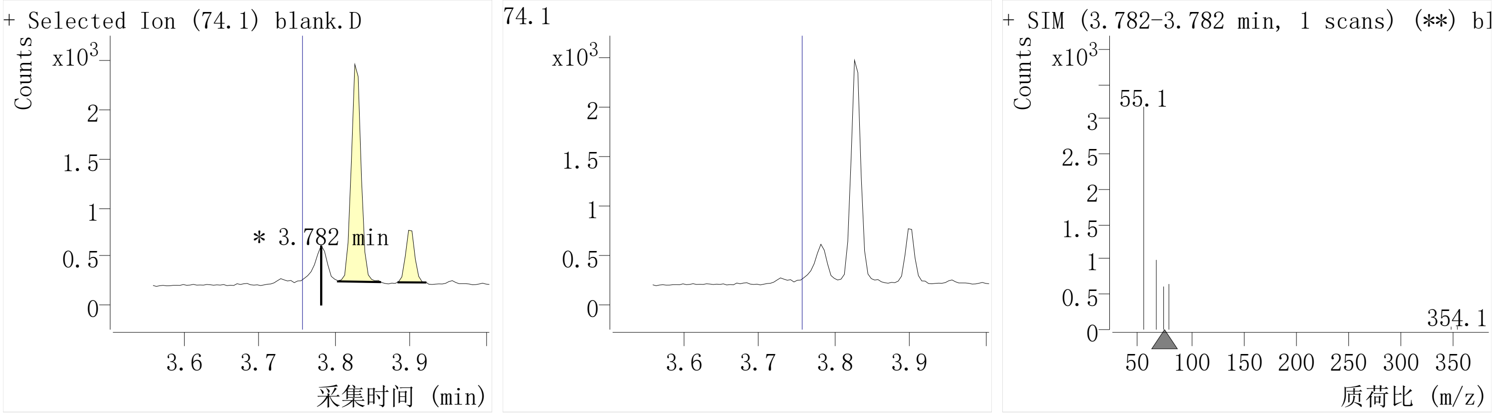

C10:0

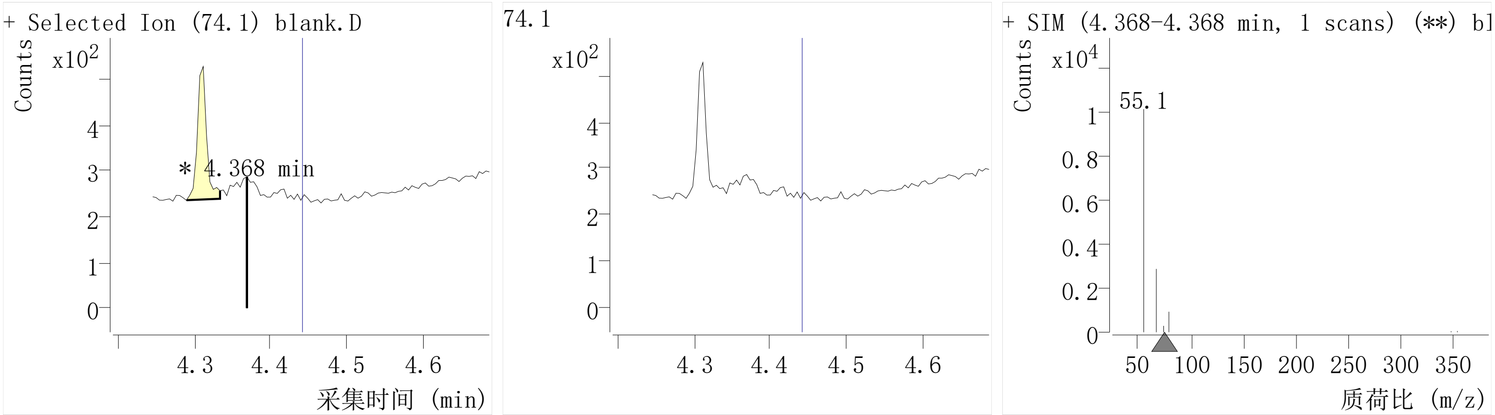

C11:0

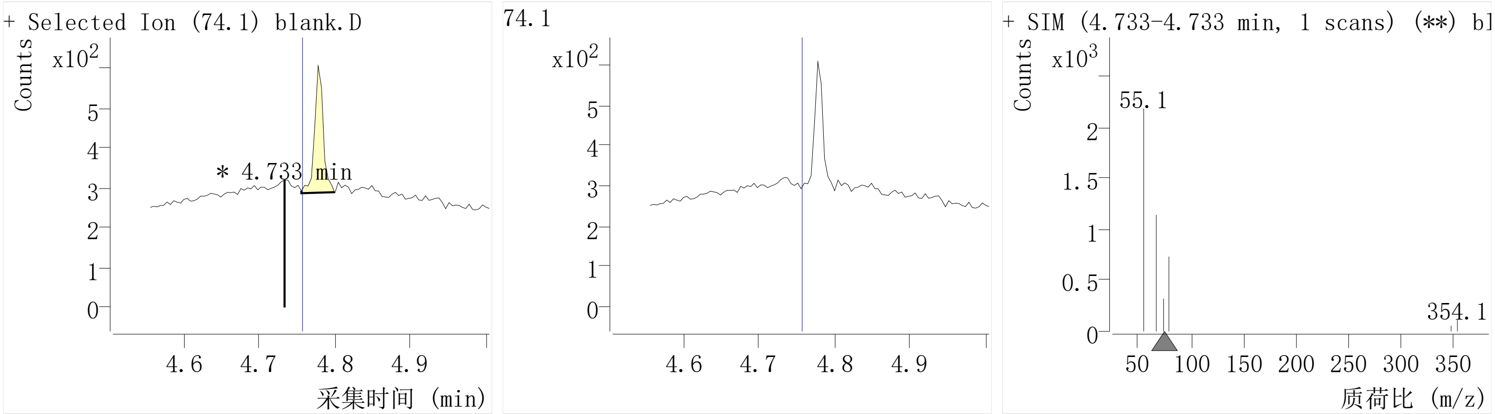

C12:0

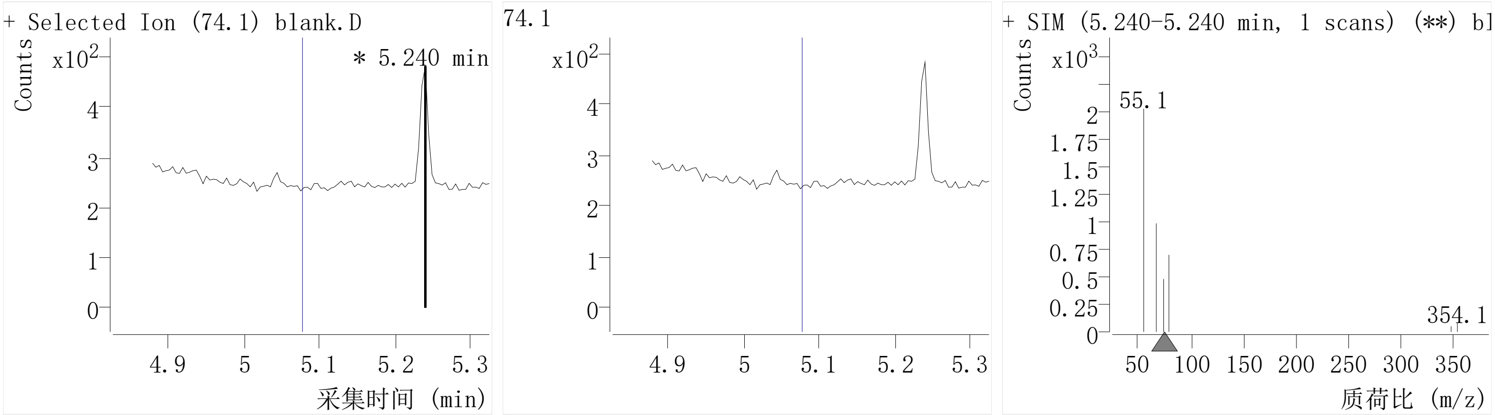

C13:0

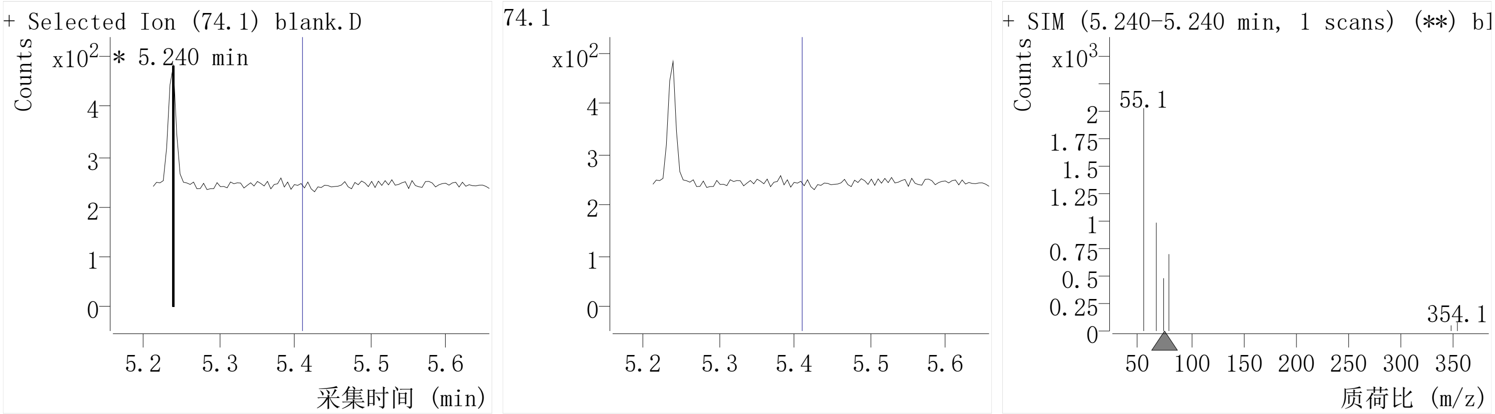

C14:0

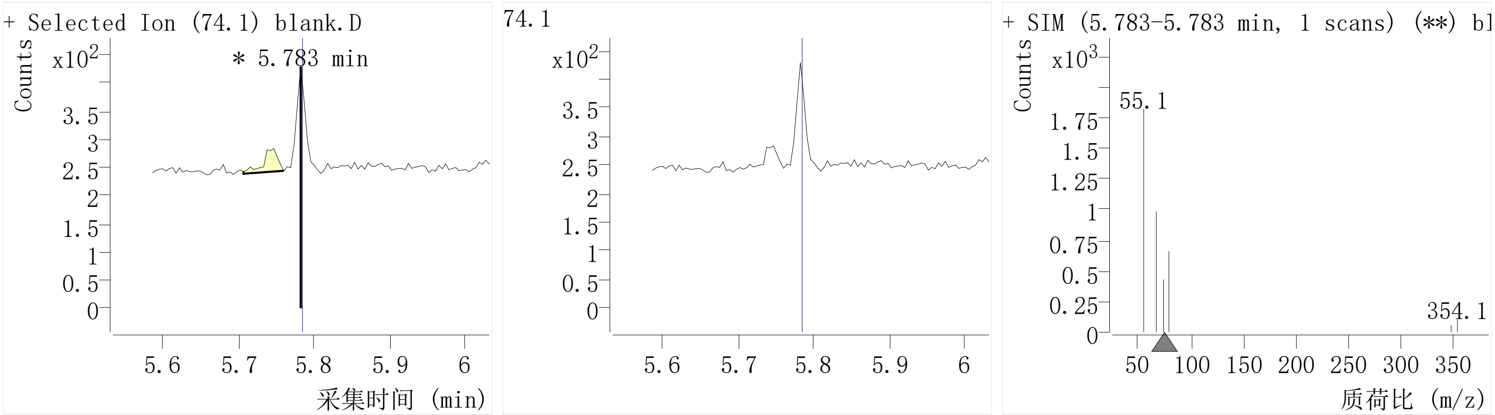

C14:1

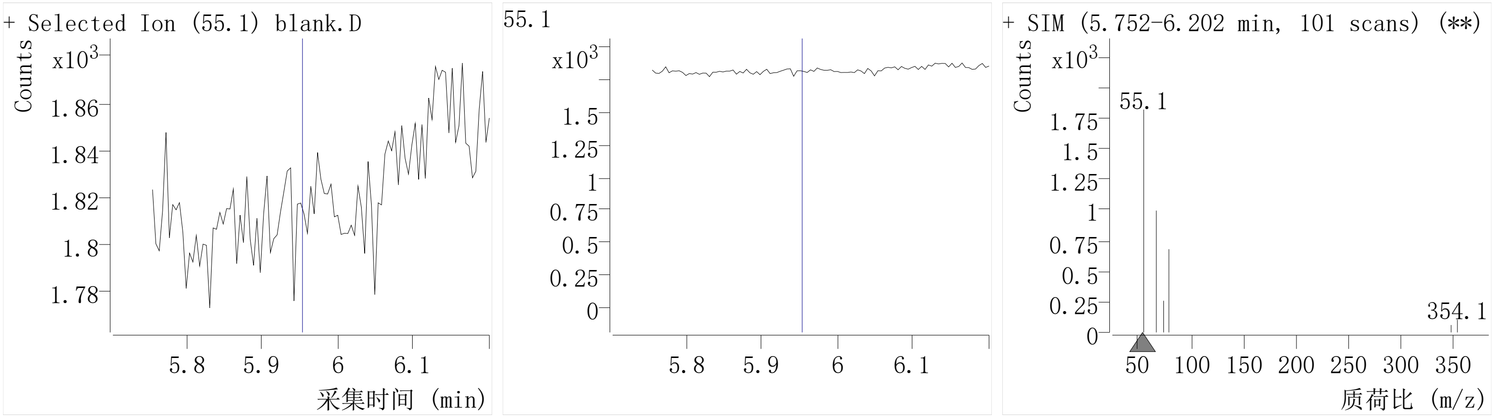

C15:0

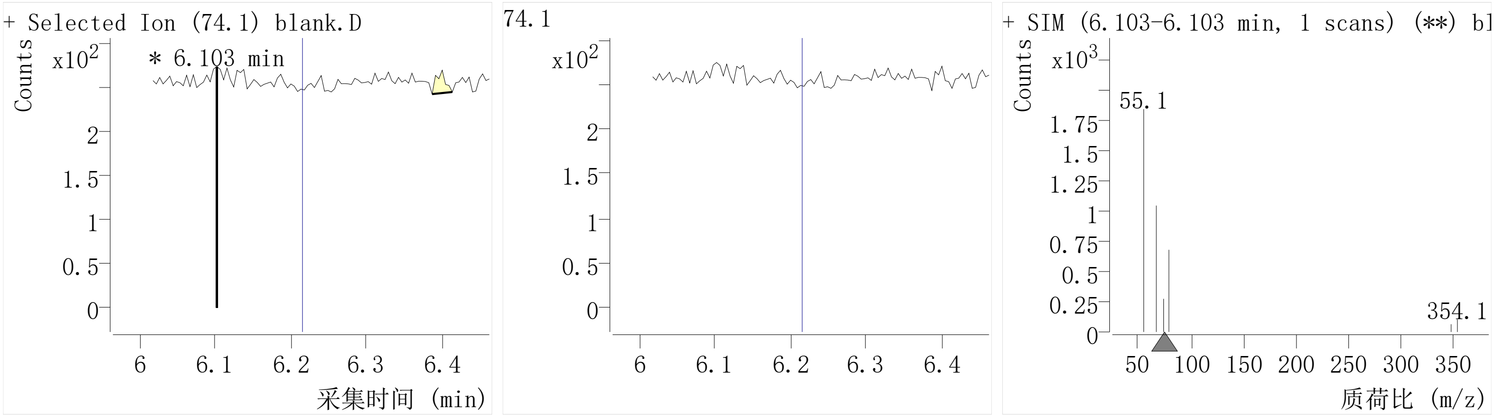

C15:1

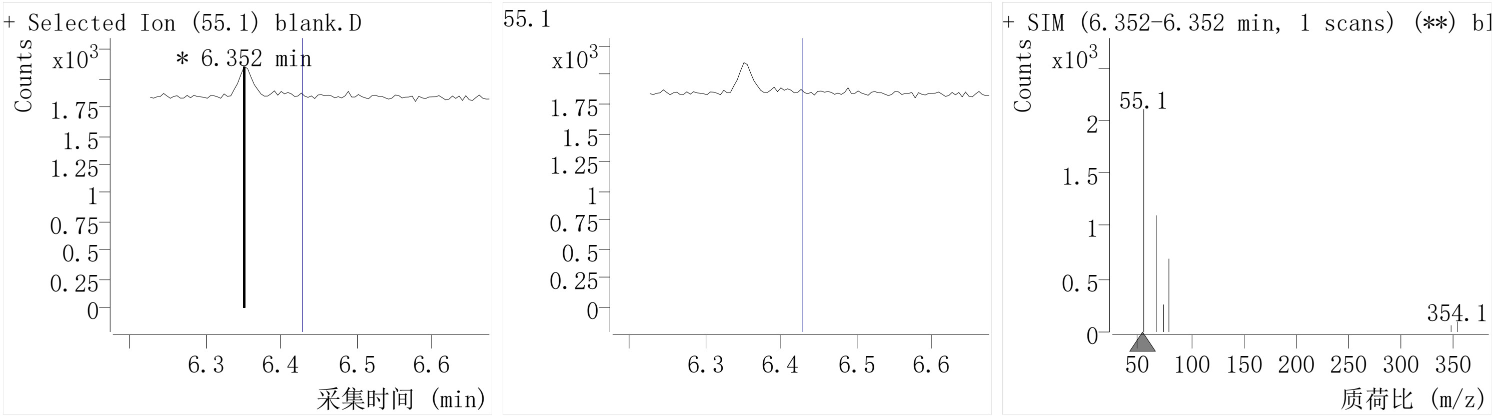

C16:0

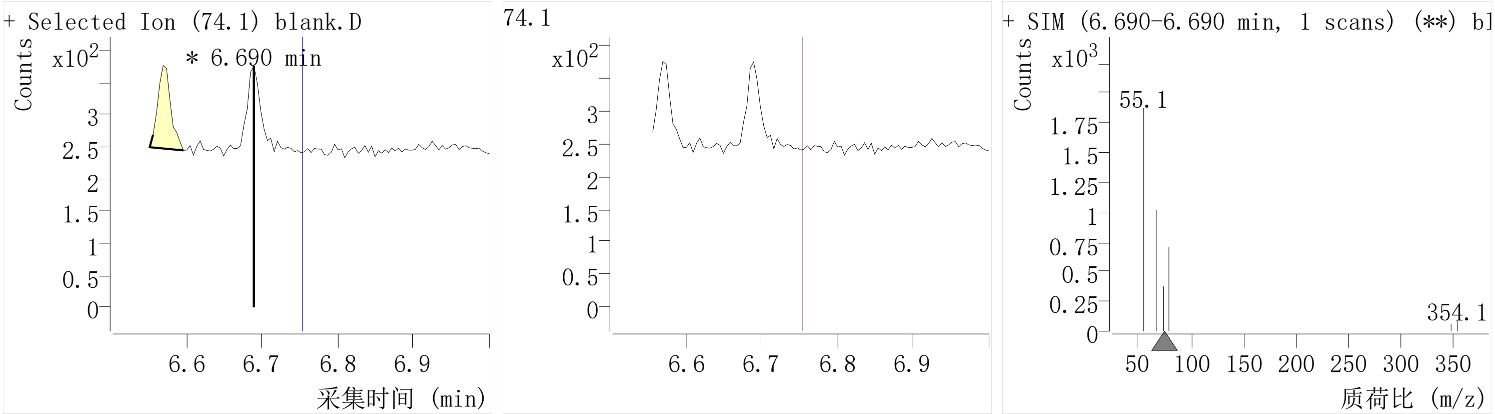

C16:1

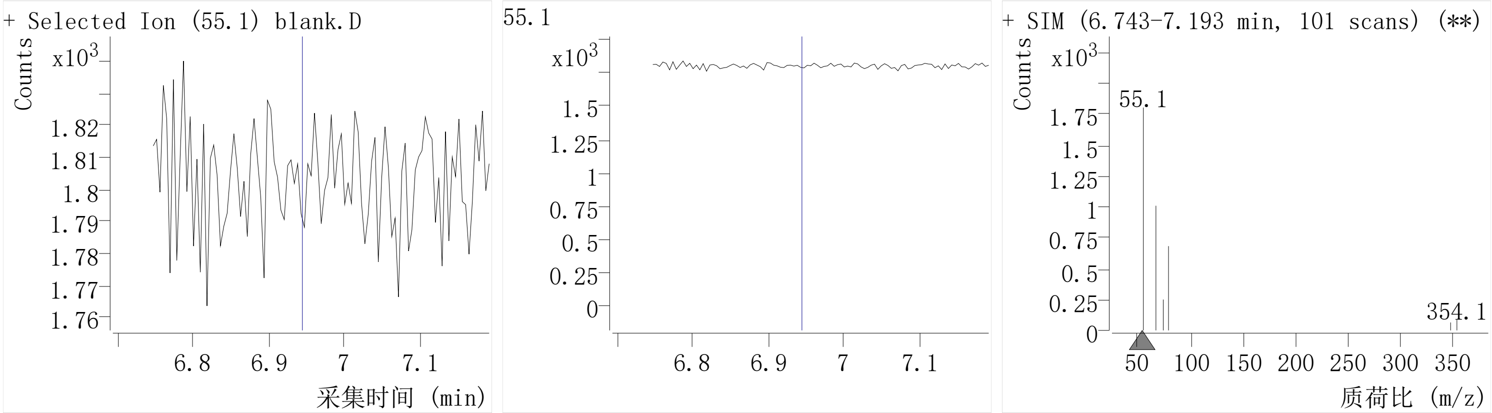

C17:0

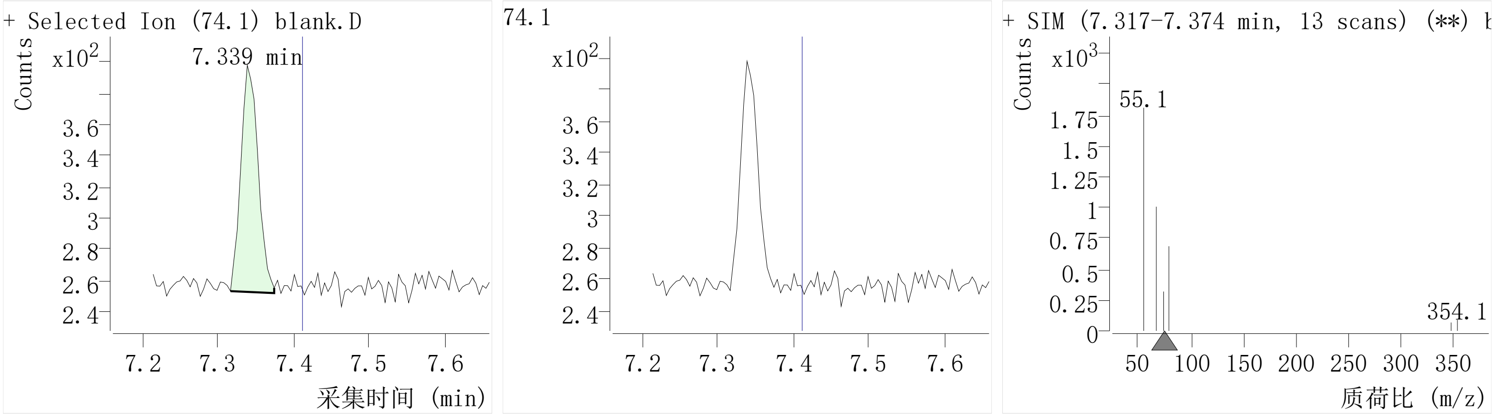

C17:1

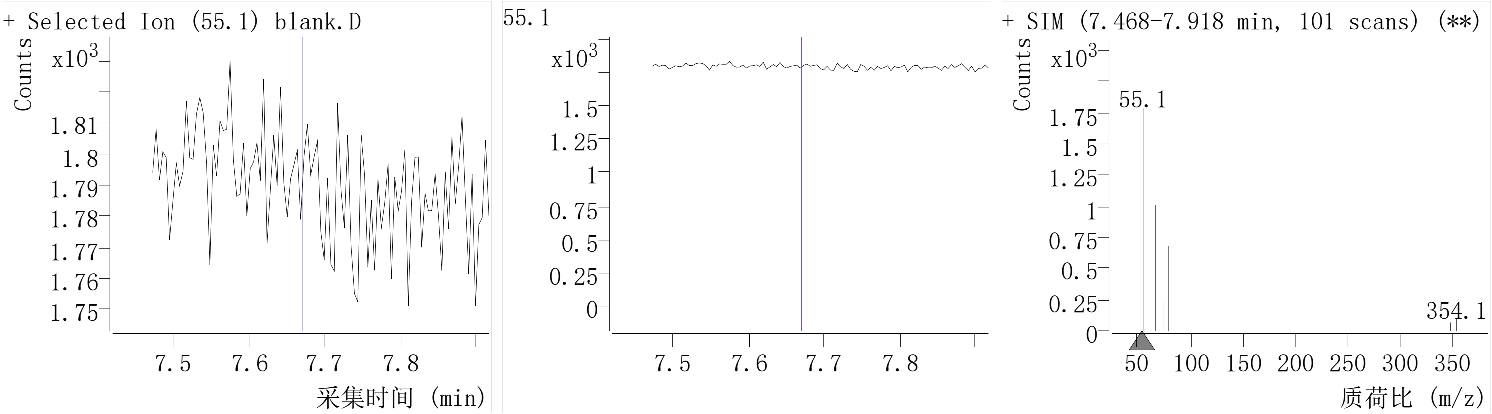

C18:0

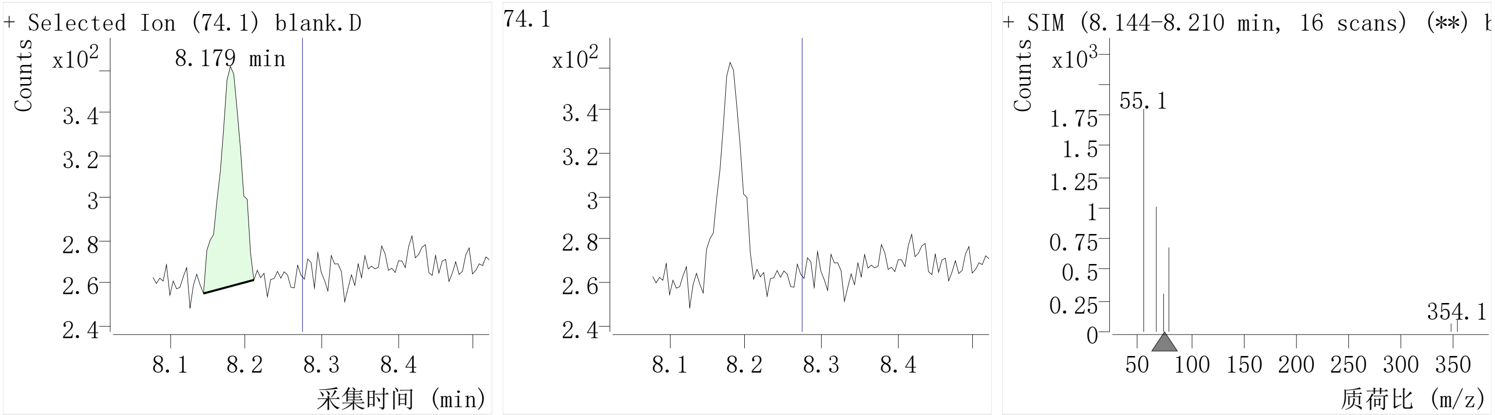

C18:1n9t

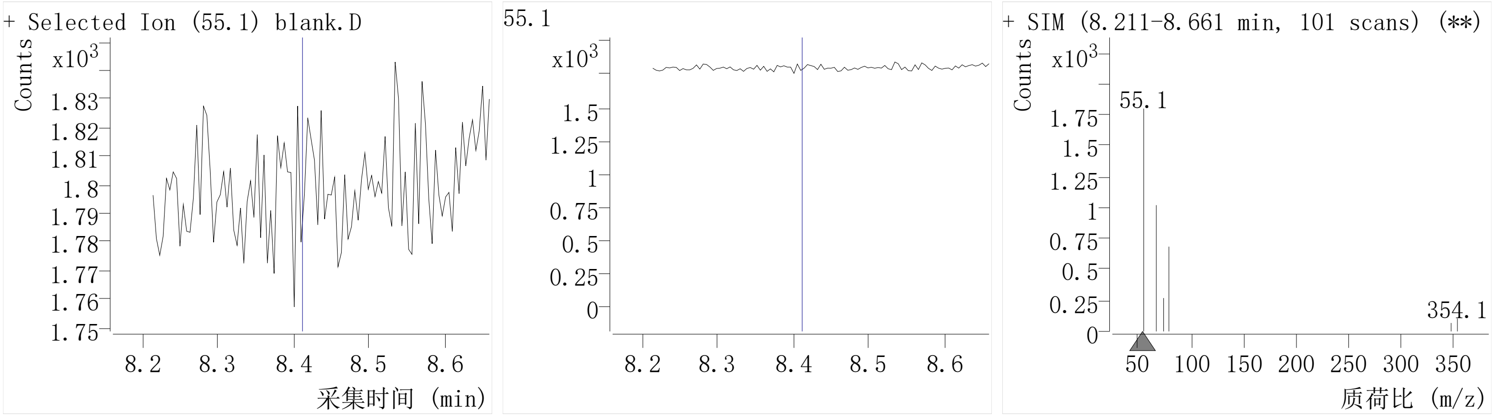

C18:1n9c

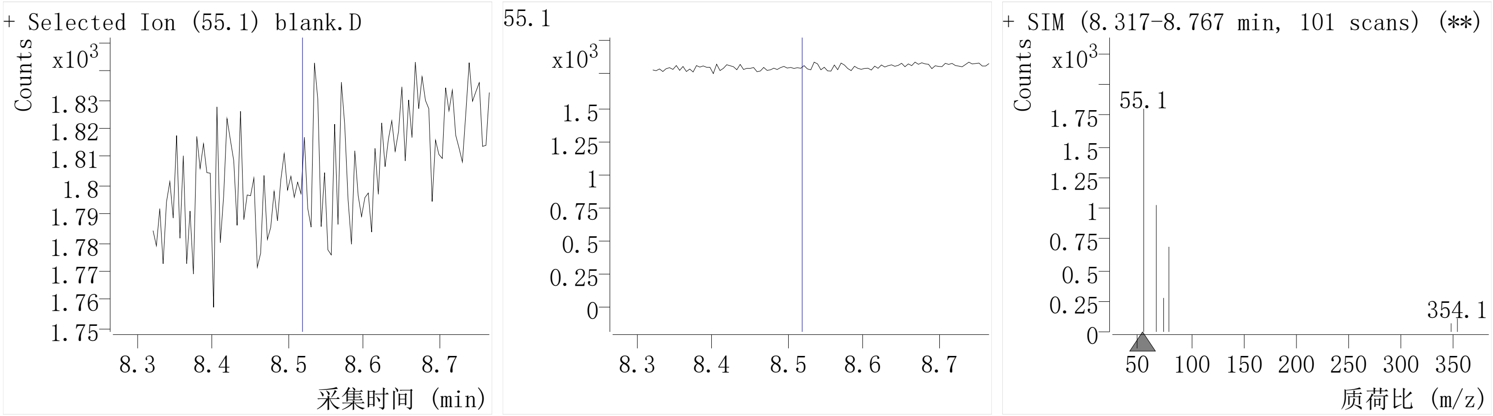

C18:2n6t

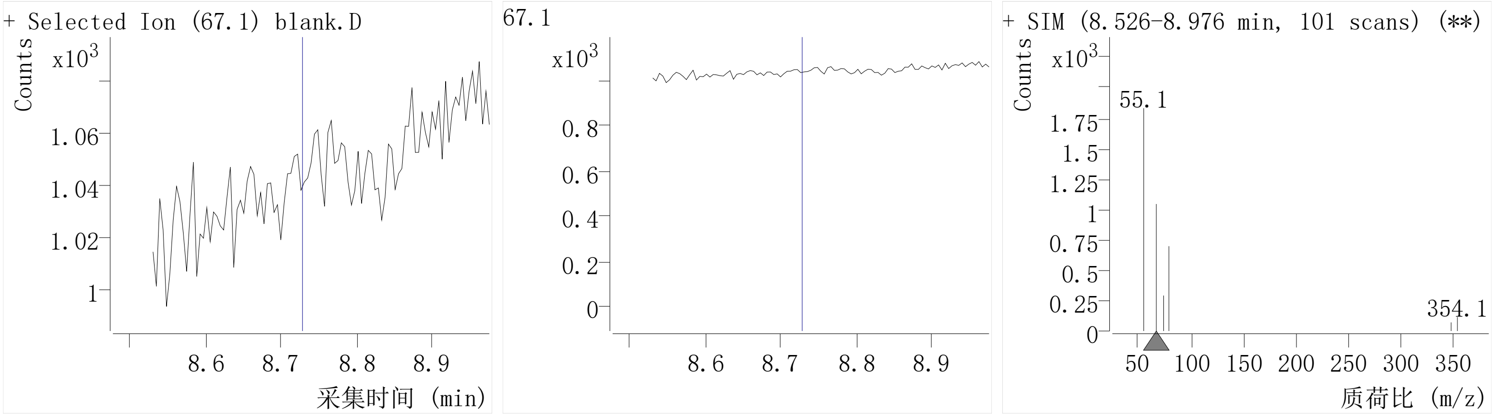

C18:2n6c

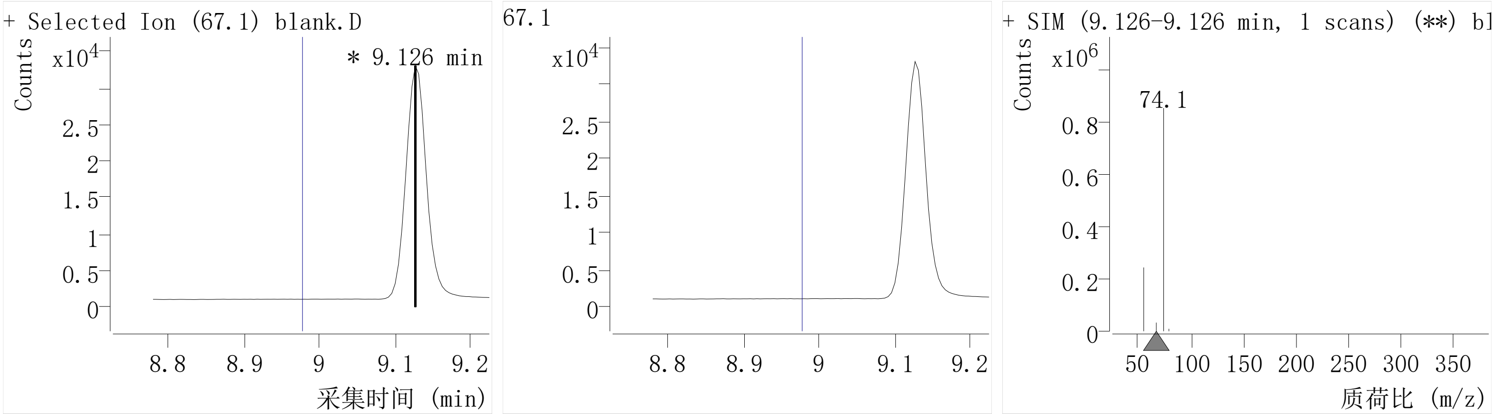

C18:3n6

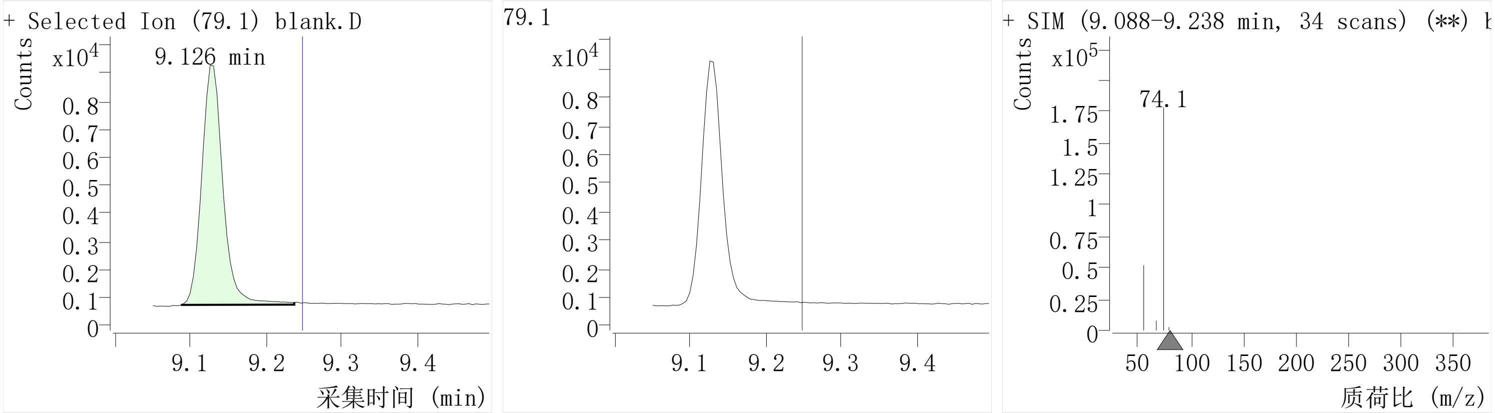

C18:3n3

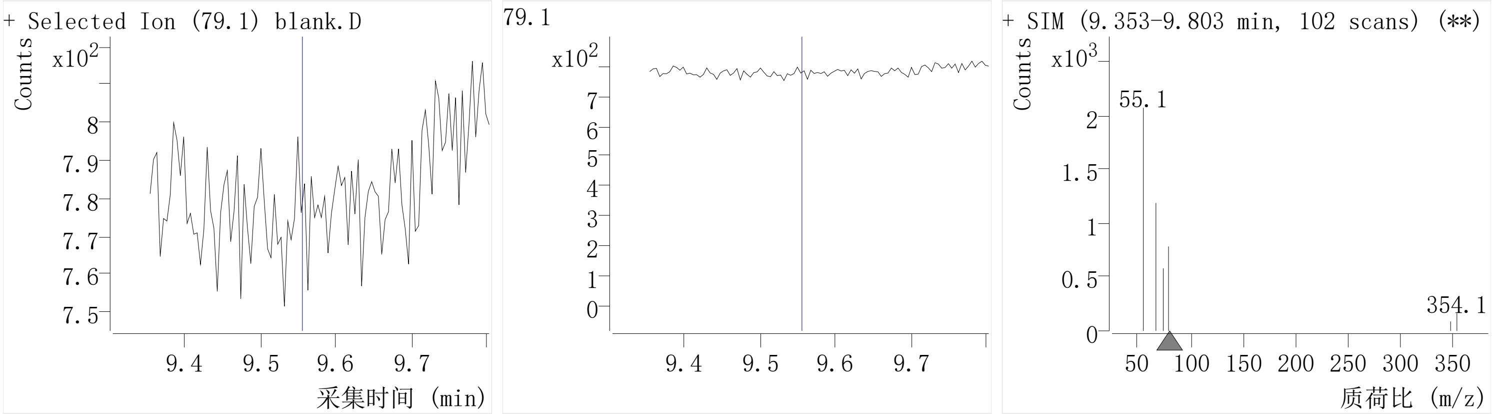

C20:0

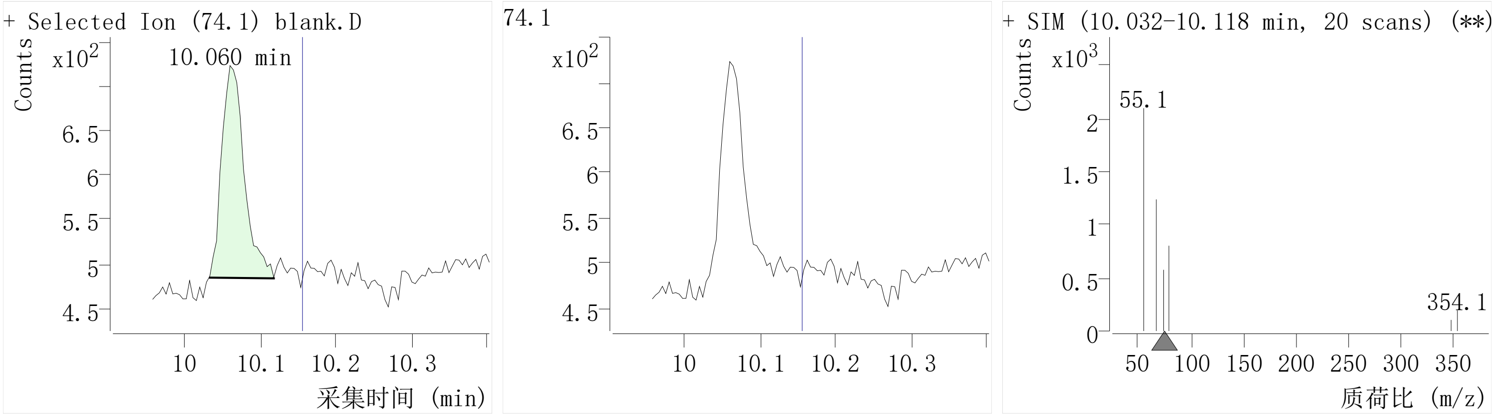

C20:1

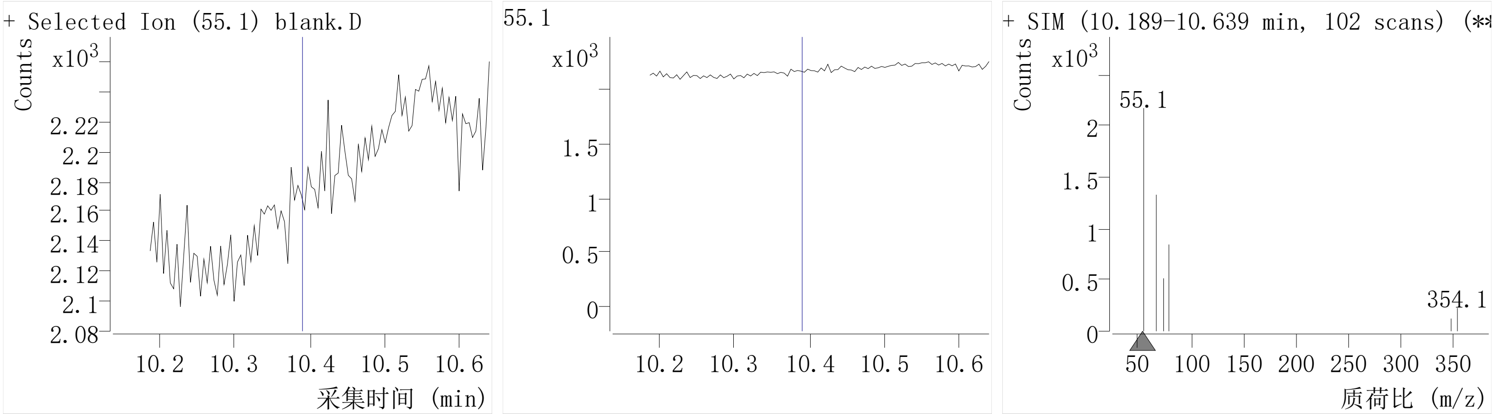

C20:2

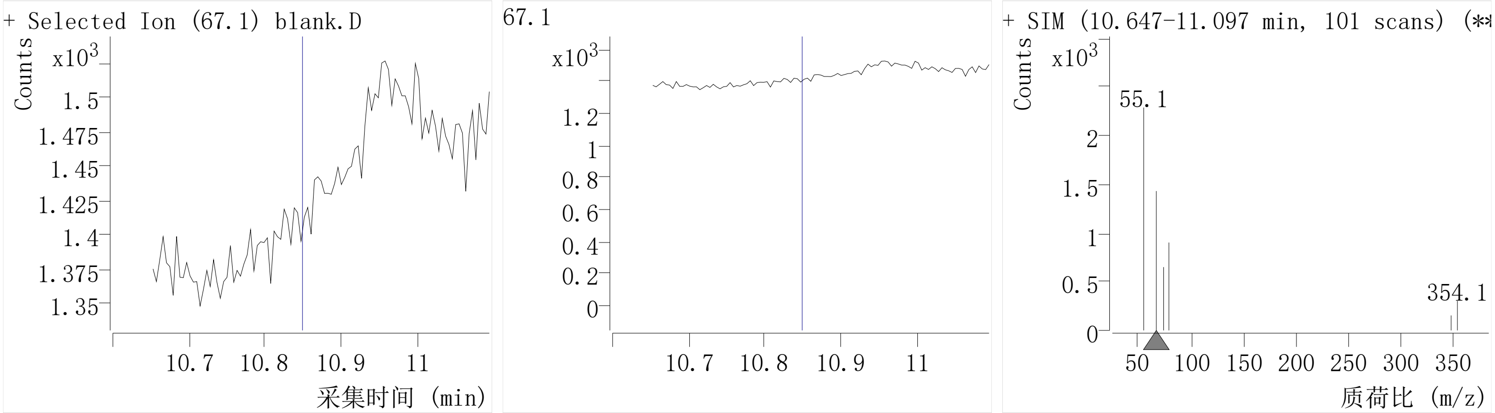

C21:0

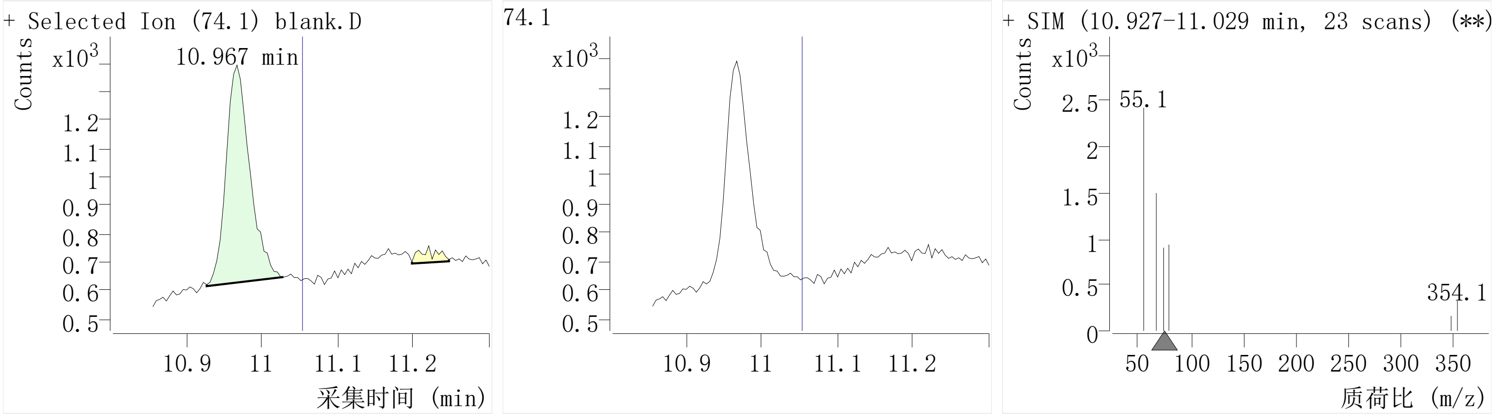

C20:3n6

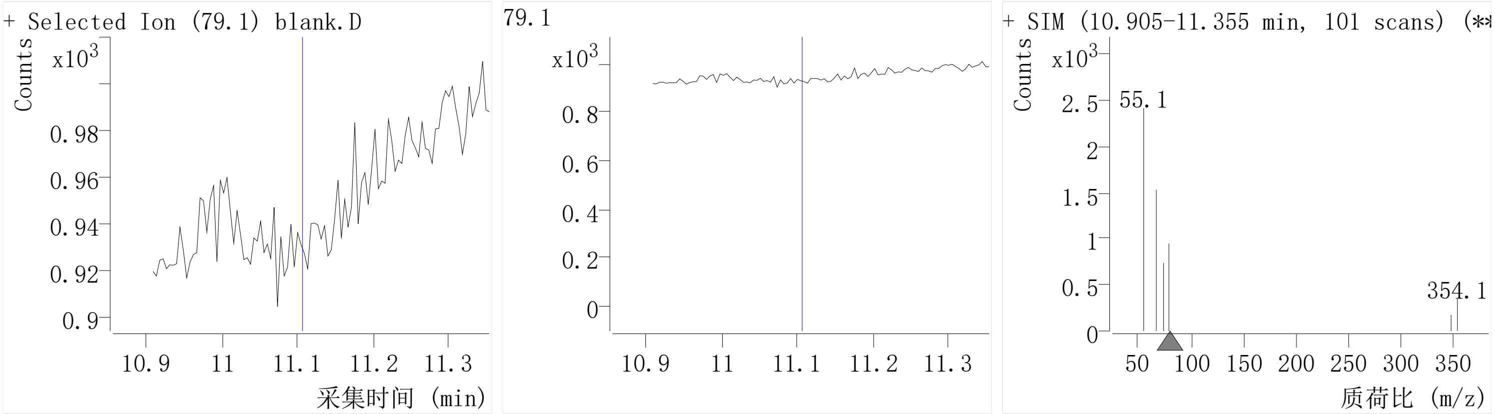

C20:4n6

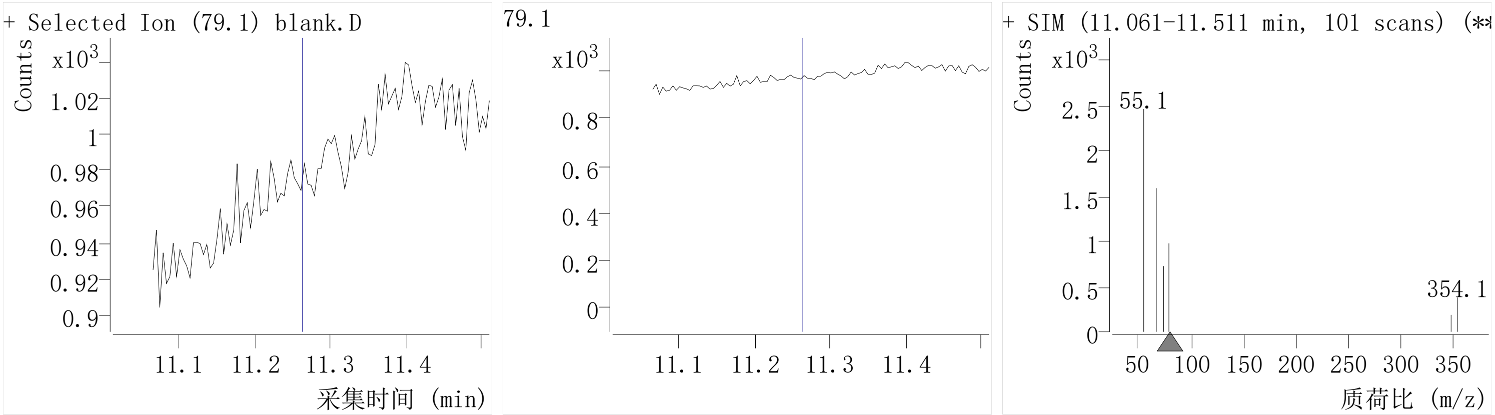

C20:3n3

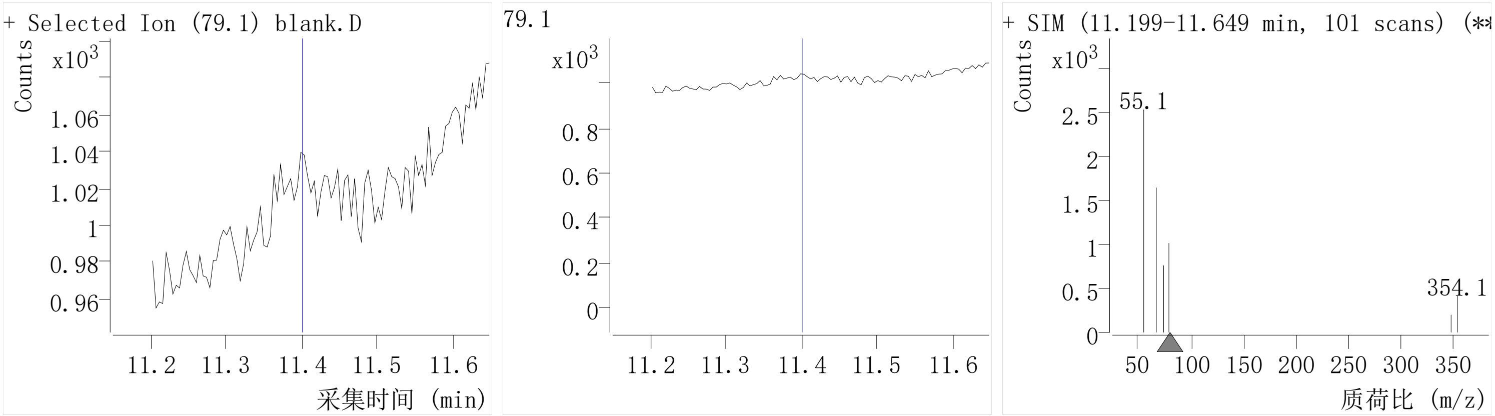

C20:5n3

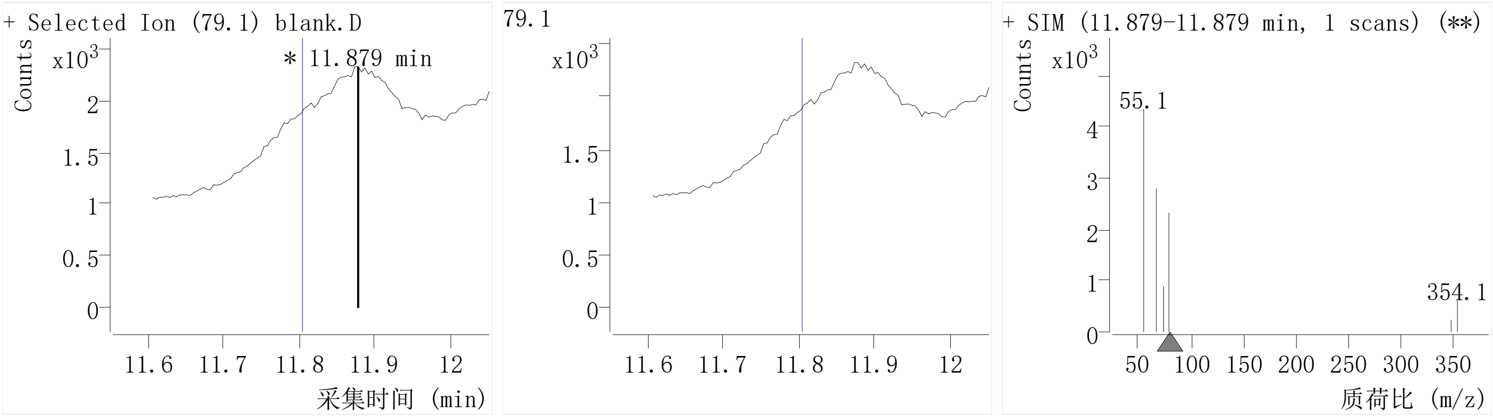

C22:0

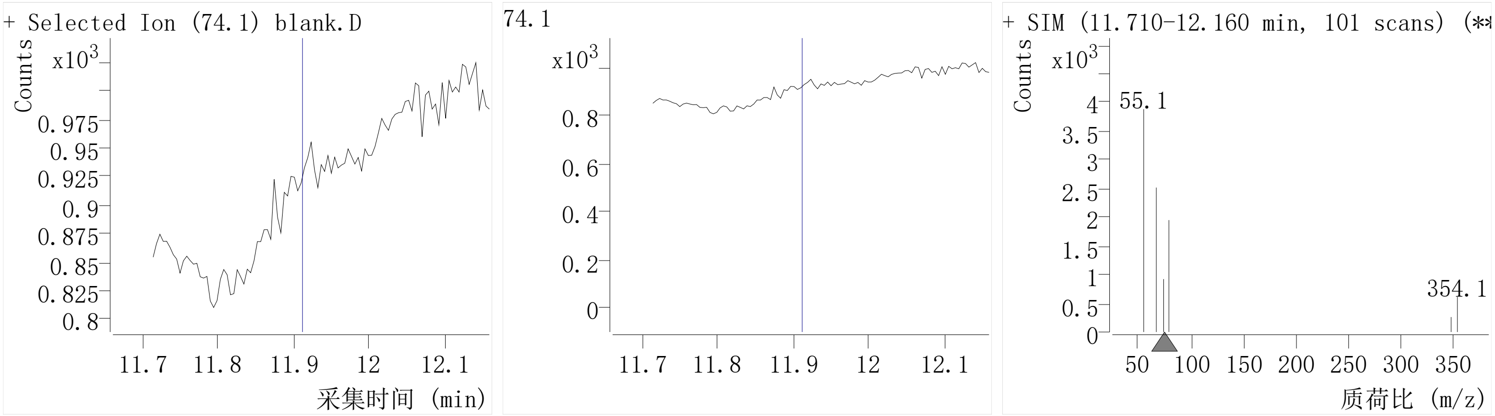

C22:1n9

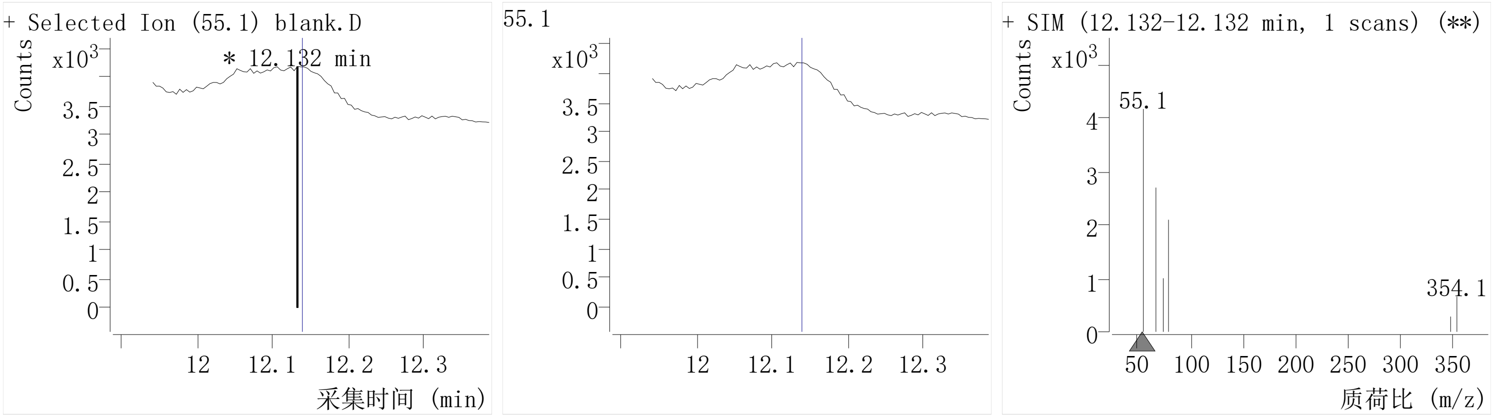

C22:2n6

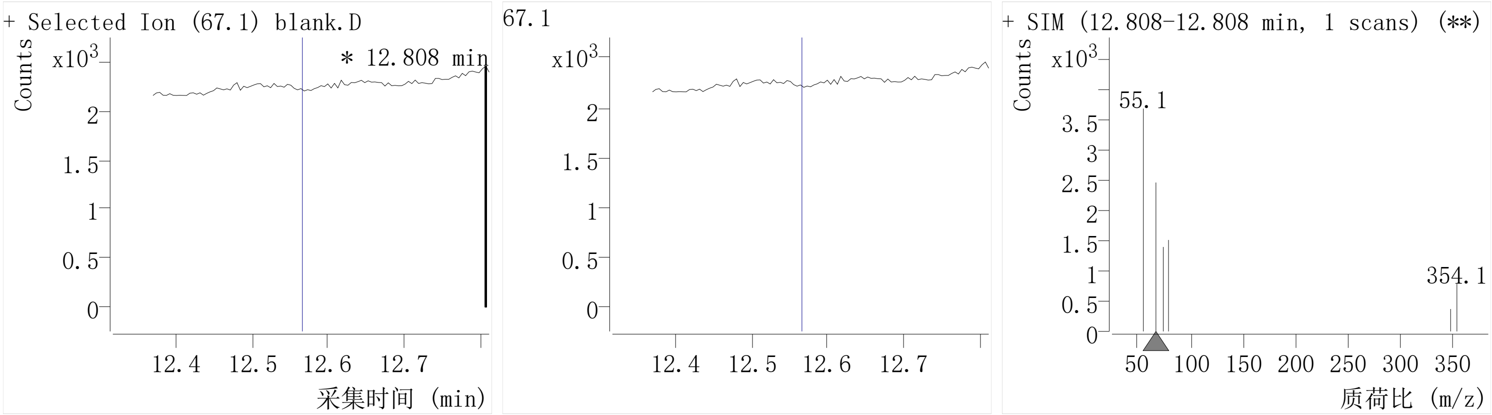

C23:0

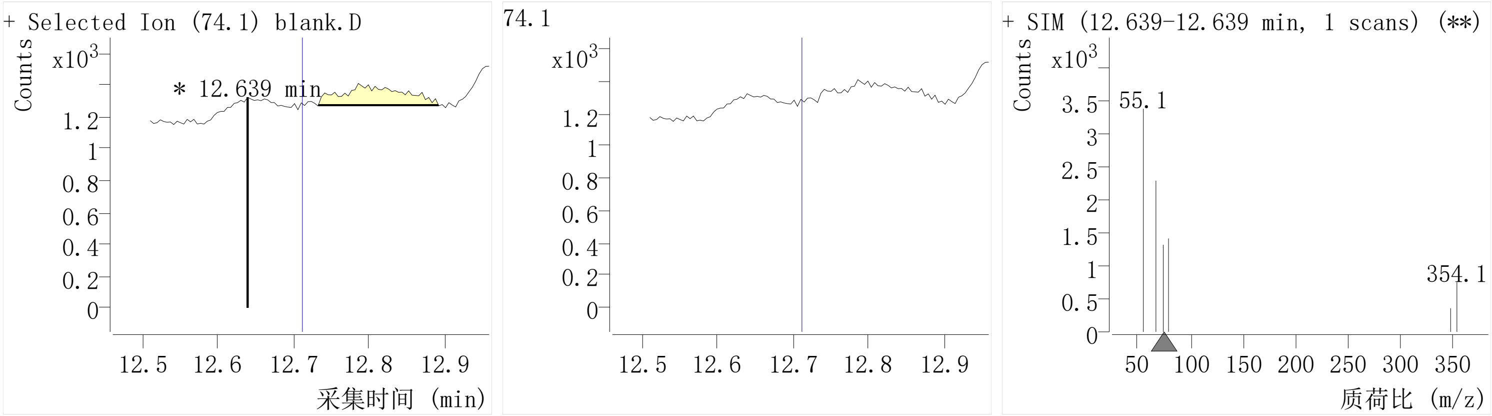

C24:0

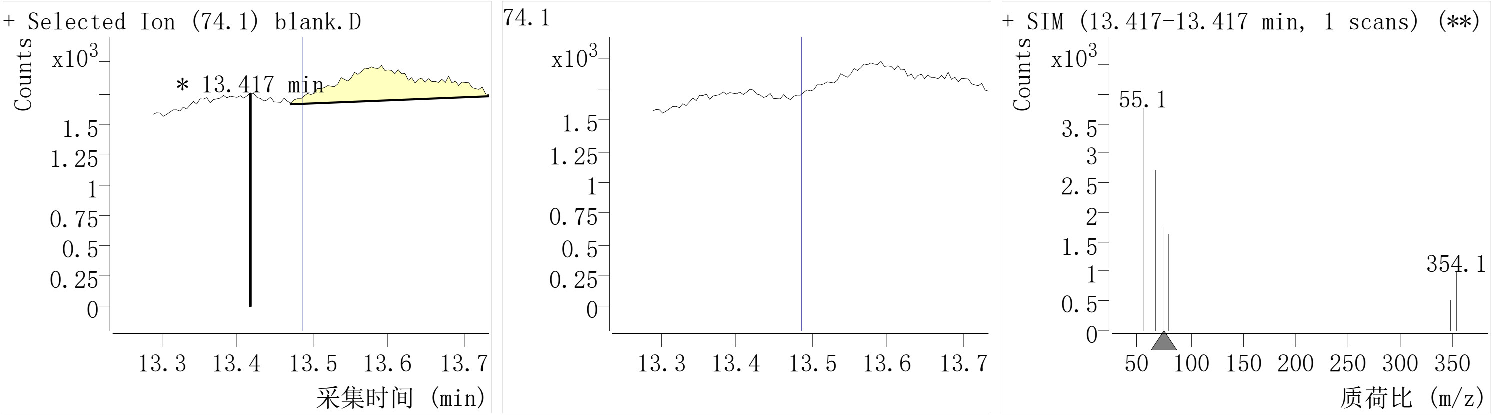

C22:6

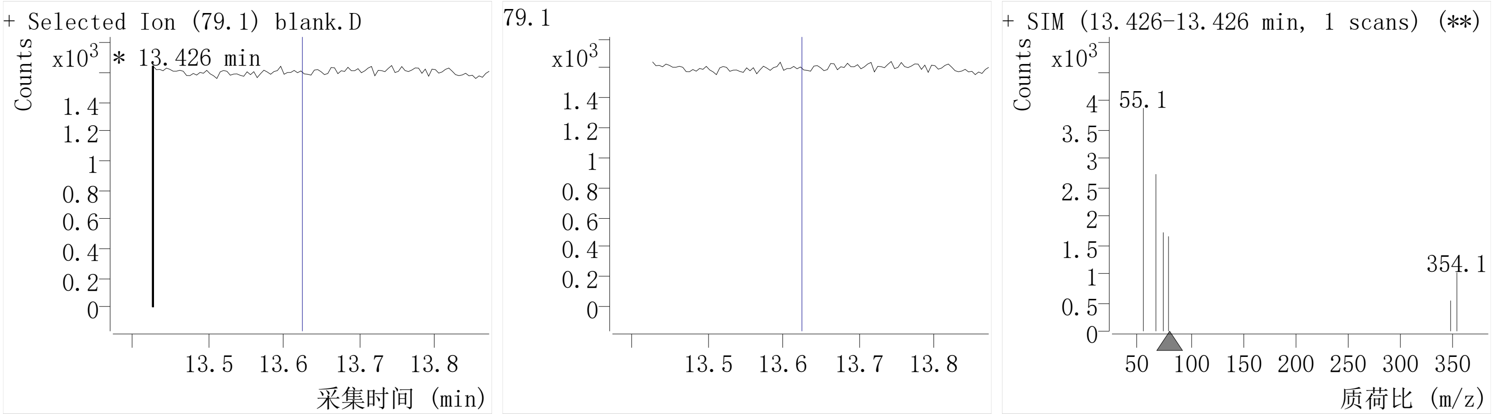
+ Selected Ion (79.1) blank.D
Counts
x10^3
* 13.426 min
1.4
1.2
1
0.8
0.6
0.4
0.2
0
13.5 13.6 13.7 13.8
采集时间 (min)
79.1
x10^3
1.4
1.2
1
0.8
0.6
0.4
0.2
0
13.5 13.6 13.7 13.8
+ SIM (13.426-13.426 min, 1 scans) (**)
Counts
x10^3
4
3.5
3
2.5
2
1.5
1
0.5
0
55.1
354.1
50 100 150 200 250 300 350
质荷比 (m/z)

C24:1

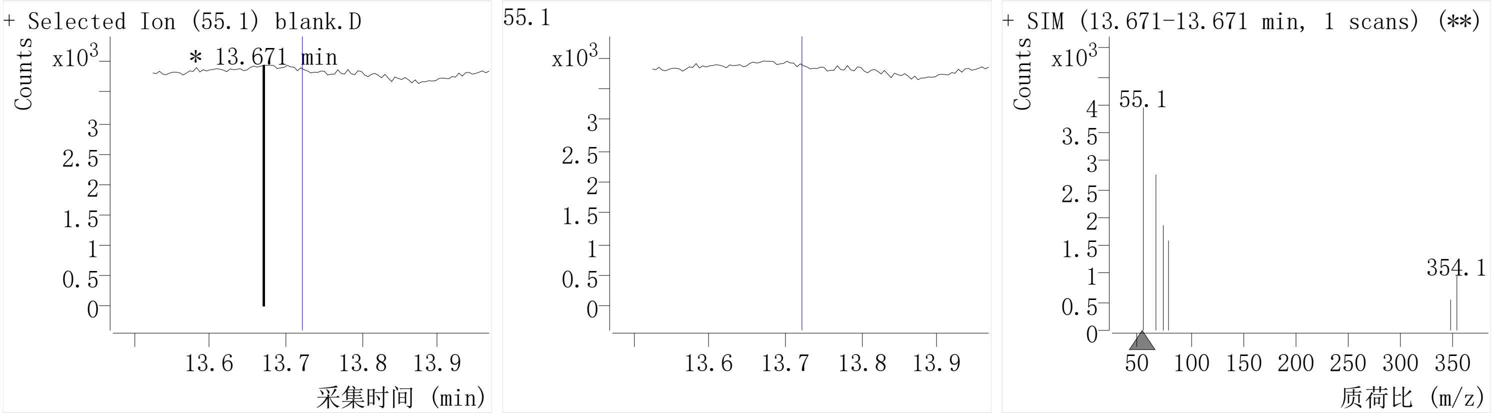
+ Selected Ion (55.1) blank.D
Counts
x10^3
* 13.671 min
3
2.5
2
1.5
1
0.5
0
13.6 13.7 13.8 13.9
采集时间 (min)
55.1
x10^3
3
2.5
2
1.5
1
0.5
0
13.6 13.7 13.8 13.9
+ SIM (13.671-13.671 min, 1 scans) (**)
Counts
x10^3
4
3.5
3
2.5
2
1.5
1
0.5
0
55.1
354.1
50 100 150 200 250 300 350
质荷比 (m/z)

**批处理路径**	G:\GC-MS\HX250430-4-GCMS总脂肪酸靶向检测\HX250430-4\QuantResults\HX250430-4.batch.bin		
**分析时间**	2025/5/14 16:58	**分析员姓名**	DESKTOP-M3A0GPO\omics
**报告时间**	2025/5/16 14:52:59	**报告员姓名**	DESKTOP-M3A0GPO\omics
**最近校正更新**	2025/5/14 16:58	**批处理状态**	已处理
**定量批处理版本**	10.2	**定量报告版本**	10.2

**采集时间**	2025/5/8 17:28	**数据文件**	std1.D
**样品类型**	校正	**样品名称**	std1
**稀释**	1	**采集方法**	脂肪酸

样品色谱图

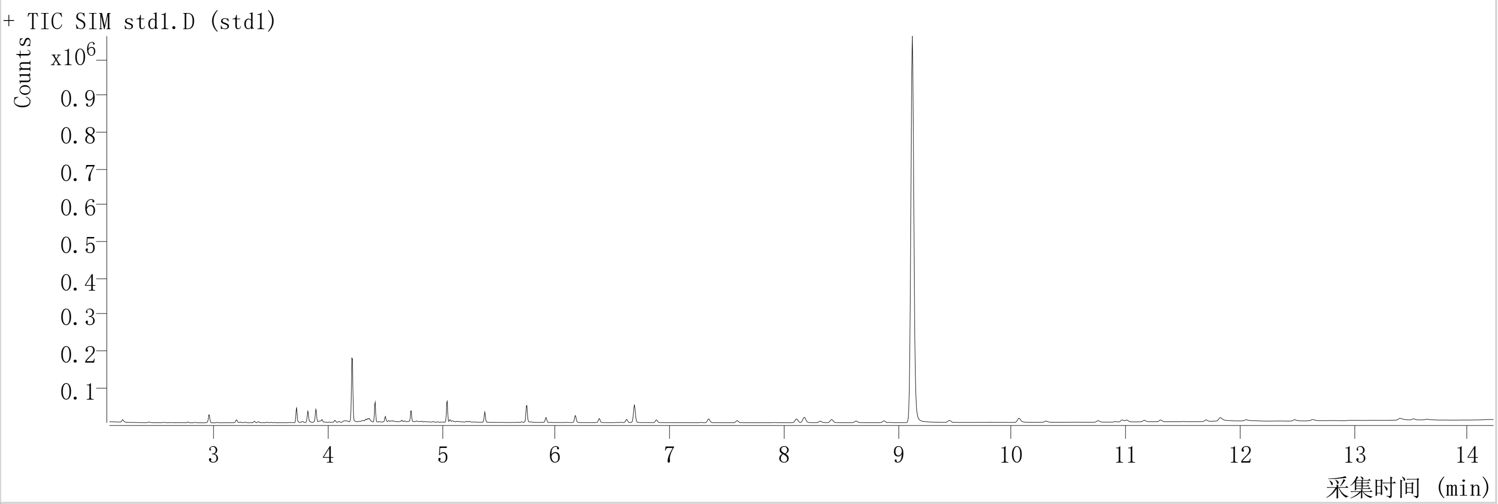

化合物	ISTD	RT	响应	ISTD 响应	响应比	最终浓度	单位
C4:0	C19:0	2.203	6903	1476809	0.0047	ND	ug/ml
C6:0	C19:0	2.959	16067	1476809	0.0109	0.3627	ug/ml
C8:0	C19:0	3.728	24525	1476809	0.0166	0.2956	ug/ml
C10:0	C19:0	4.417	32116	1476809	0.0217	0.3603	ug/ml
C11:0	C19:0	4.733	17645	1476809	0.0119	0.1763	ug/ml
C12:0	C19:0	5.049	35568	1476809	0.0241	0.3179	ug/ml
C13:0	C19:0	5.378	17506	1476809	0.0119	0.1577	ug/ml
C14:0	C19:0	5.747	34247	1476809	0.0232	0.4241	ug/ml
C14:1	C19:0	5.916	6864	1476809	0.0046	0.1276	ug/ml
C15:0	C19:0	6.174	15816	1476809	0.0107	0.1448	ug/ml
C15:1	C19:0	6.383	7138	1476809	0.0048	ND	ug/ml
C16:0	C19:0	6.690	44744	1476809	0.0303	0.1331	ug/ml
C16:1	C19:0	6.881	5056	1476809	0.0034	0.1209	ug/ml
C17:0	C19:0	7.343	12199	1476809	0.0083	0.1723	ug/ml
C17:1	C19:0	7.592	4563	1476809	0.0031	0.1375	ug/ml
C18:0	C19:0	8.179	21989	1476809	0.0149	0.3982	ug/ml
C18:1n9t	C19:0	8.317	3449	1476809	0.0023	0.1378	ug/ml
C18:1n9c	C19:0	8.419	7714	1476809	0.0052	0.2292	ug/ml
C18:2n6t	C19:0	8.633	3724	1476809	0.0025	0.1569	ug/ml
C18:2n6c	C19:0	8.877	4037	1476809	0.0027	0.2219	ug/ml
C18:3n6	C19:0	9.131	20243	1476809	0.0137	0.1220	ug/ml
C18:3n3	C19:0	9.451	4574	1476809	0.0031	0.1477	ug/ml
C20:0	C19:0	10.060	18408	1476809	0.0125	0.3484	ug/ml
C20:1	C19:0	10.300	3434	1476809	0.0023	0.1832	ug/ml
C20:2	C19:0	10.754	3149	1476809	0.0021	0.1852	ug/ml
C21:0	C19:0	10.967	10026	1476809	0.0068	0.1870	ug/ml
C20:3n6	C19:0	11.007	3263	1476809	0.0022	0.1837	ug/ml
C20:4n6	C19:0	11.163	3306	1476809	0.0022	0.1748	ug/ml
C20:3n3	C19:0	11.305	3891	1476809	0.0026	0.1888	ug/ml
C20:5n3	C19:0	11.705	3469	1476809	0.0023	0.1650	ug/ml

化合物	ISTD	RT	响应	ISTD 响应	响应比	最终浓度	单位
C22:0	C19:0	11.825	15503	1476809	0.0105	0.3602	ug/ml
C22:1n9	C19:0	12.052	4762	1476809	0.0032	0.2240	ug/ml
C22:2n6	C19:0	12.479	2958	1476809	0.0020	0.1953	ug/ml
C23:0	C19:0	12.639	7416	1476809	0.0050	0.1736	ug/ml
C24:0	C19:0	13.404	10067	1476809	0.0068	0.3137	ug/ml
C22:6	C19:0	13.524	3129	1476809	0.0021	0.1617	ug/ml
C24:1	C19:0	13.640	3307	1476809	0.0022	0.1923	ug/ml

C4:0

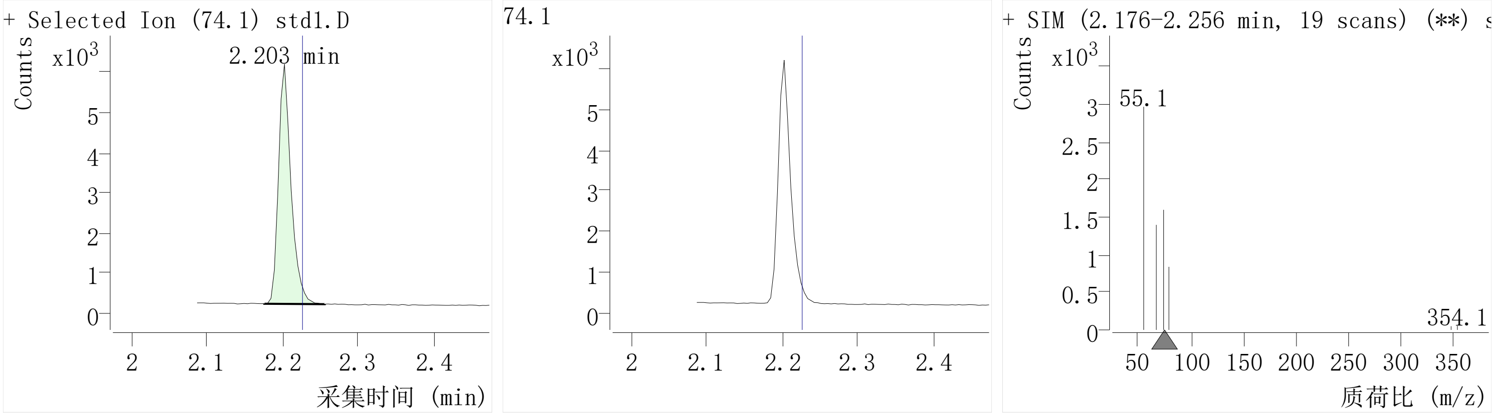

C6:0

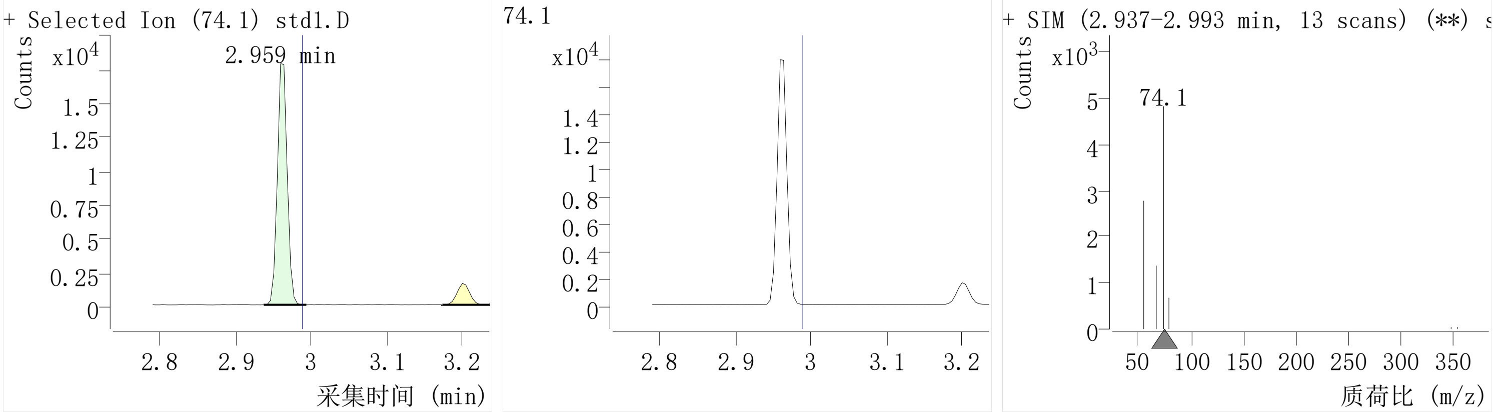

C8:0

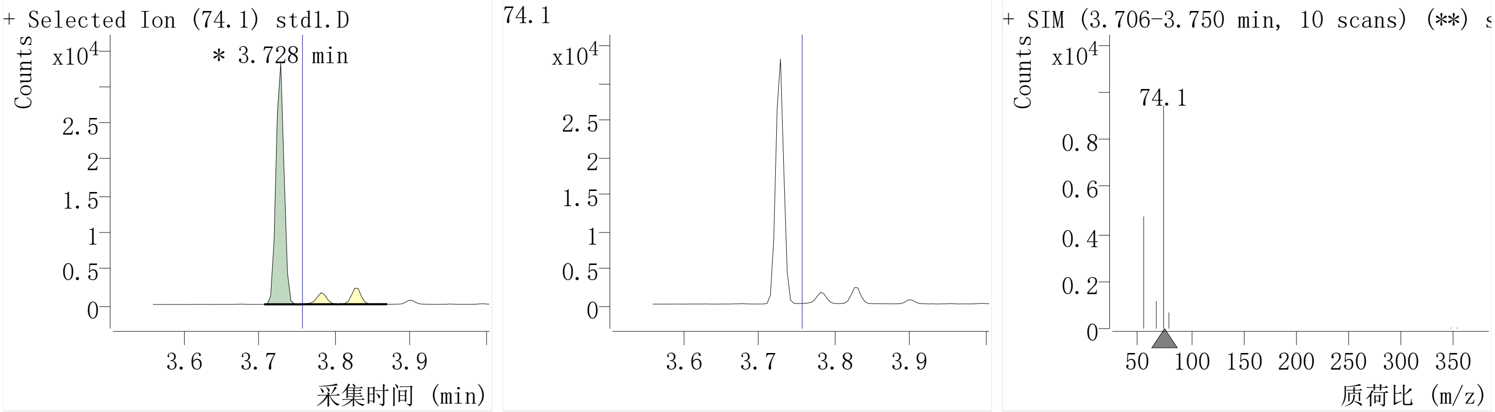

C10:0

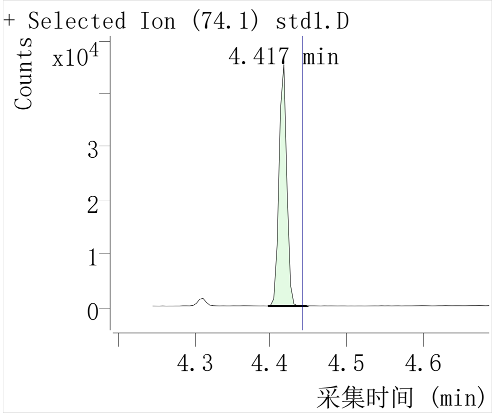

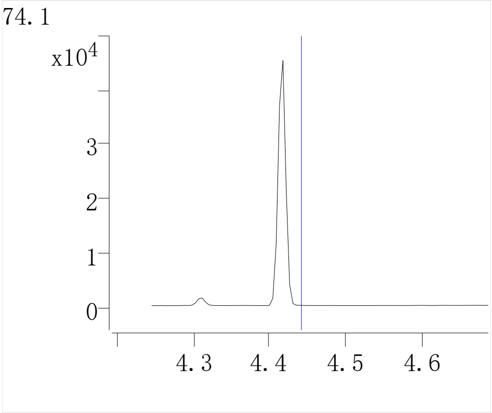

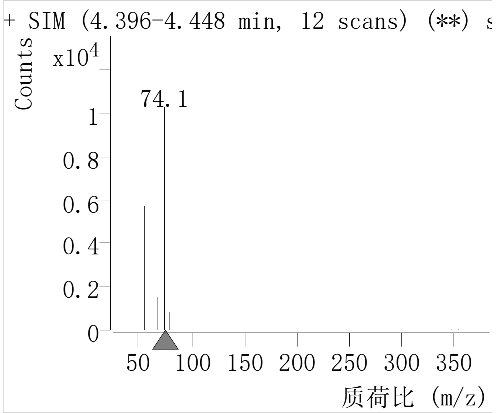

C11:0

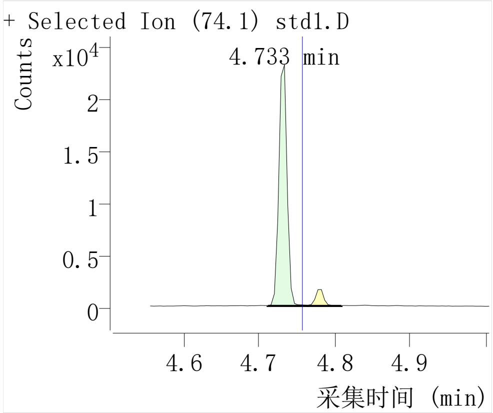

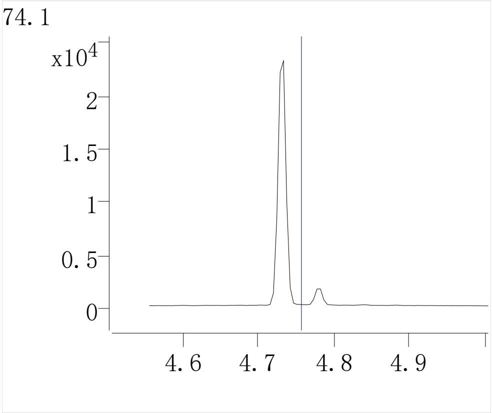

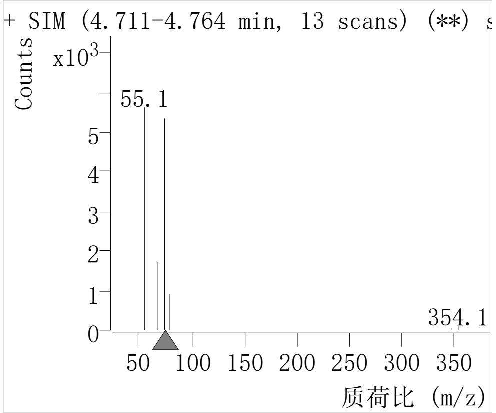

C12:0

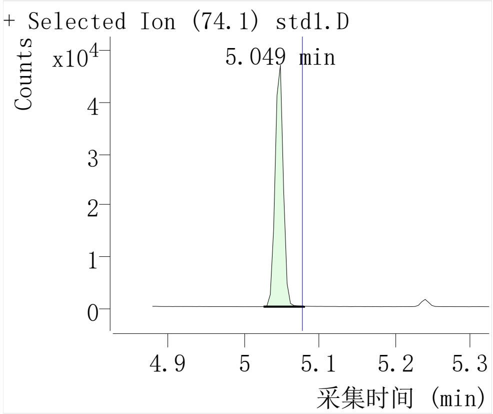

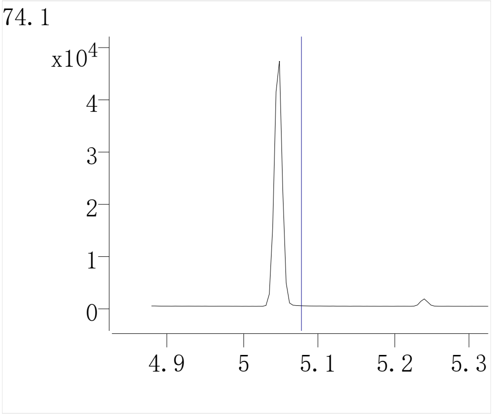

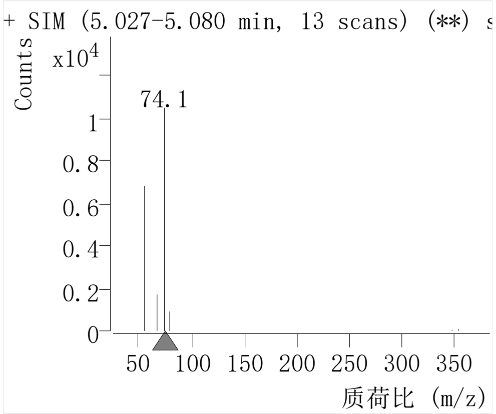

C13:0

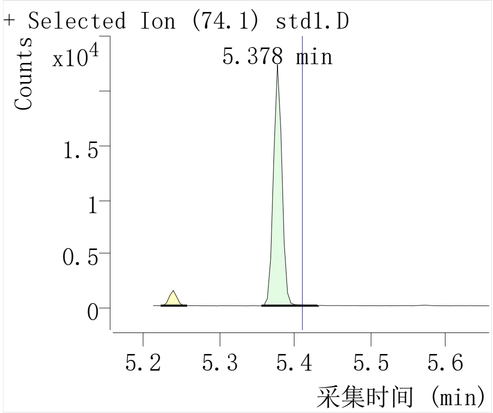

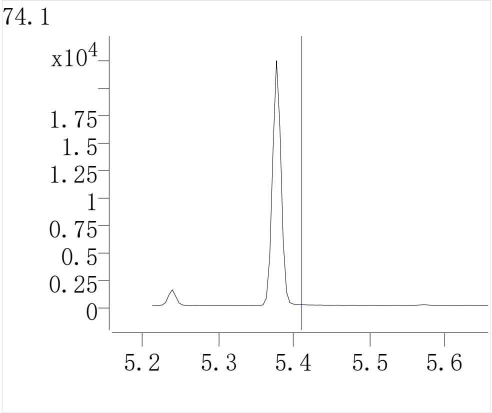

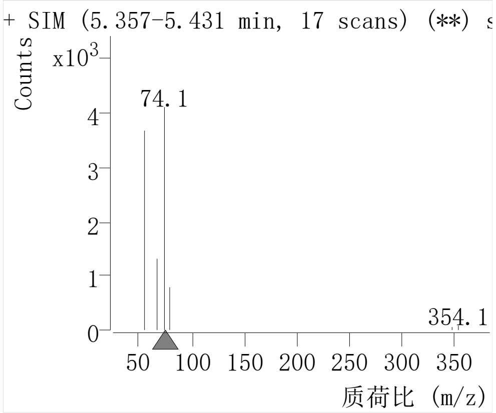

C14:0

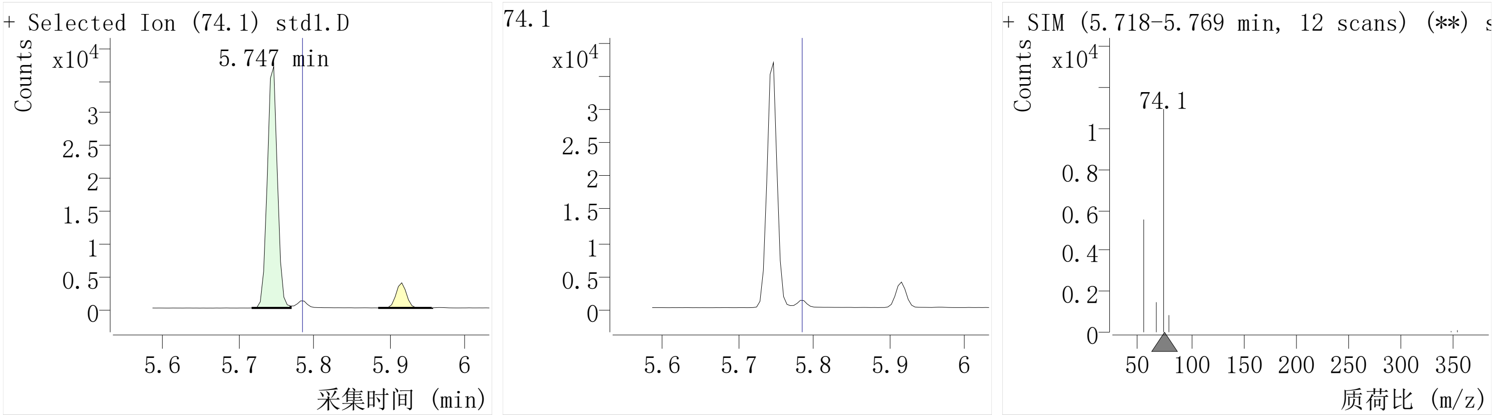

C14:1

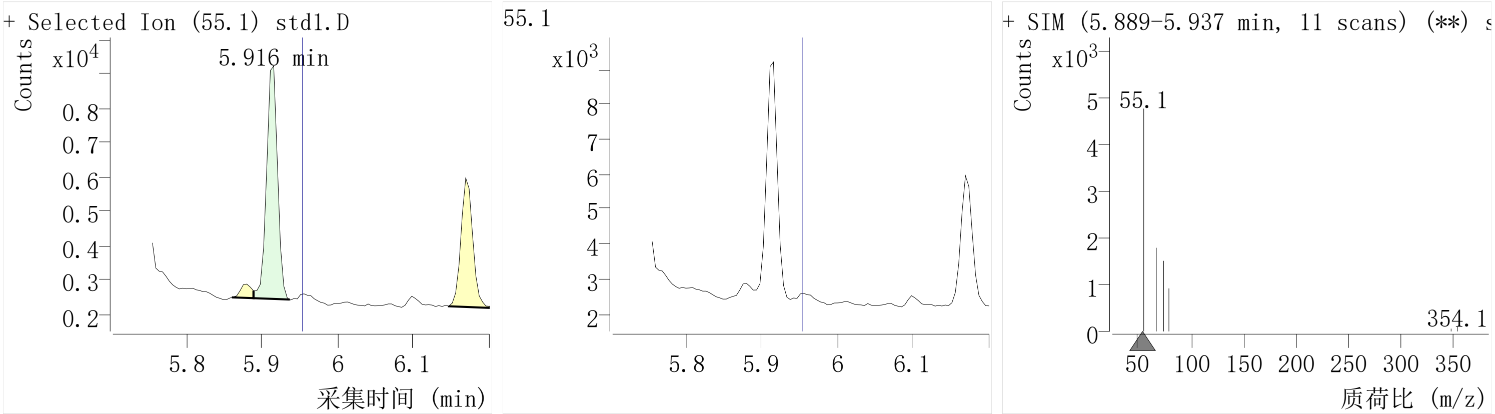

C15:0

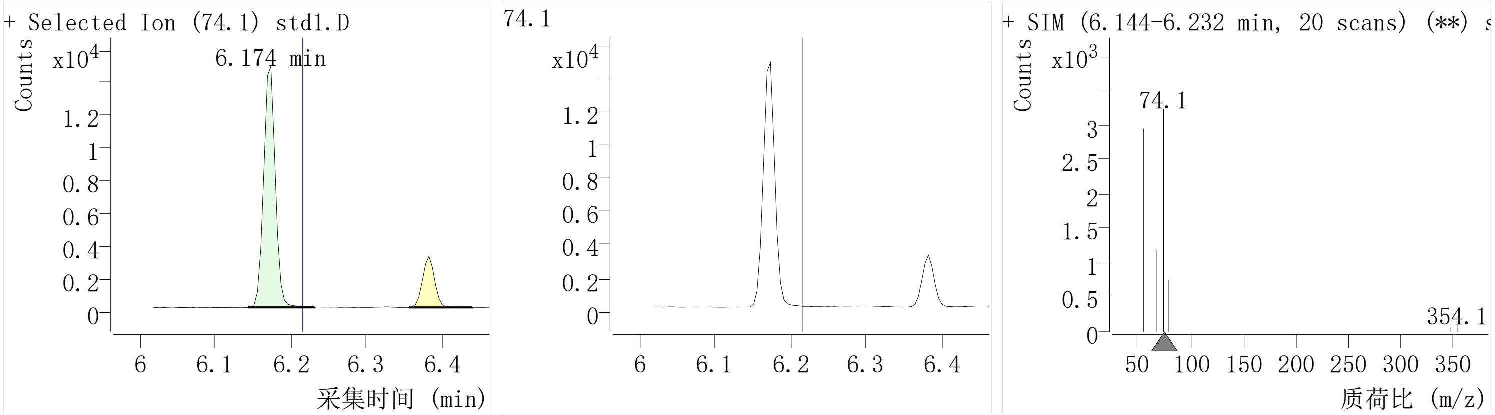

C15:1

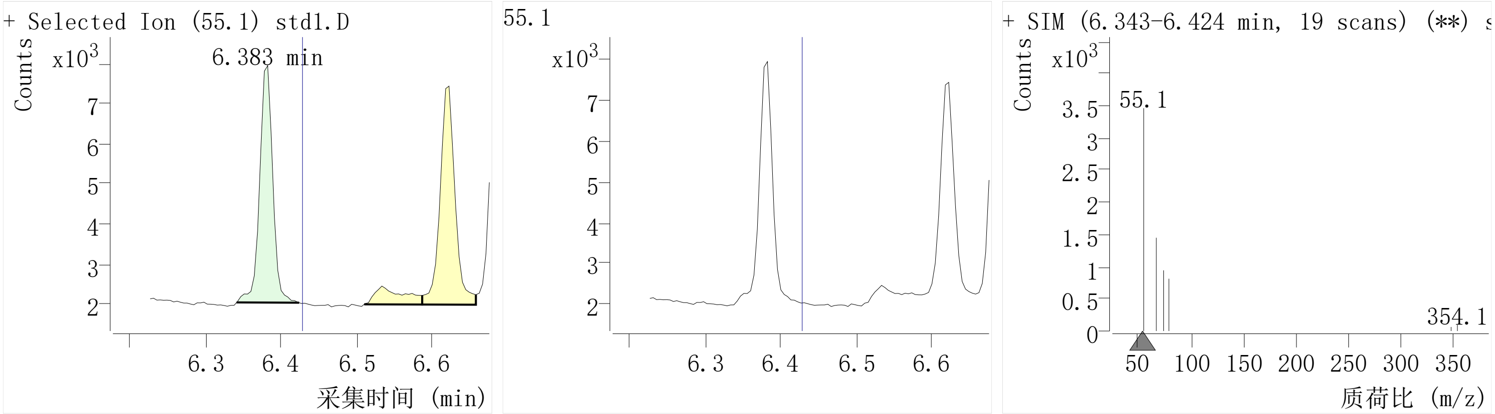

C16:0

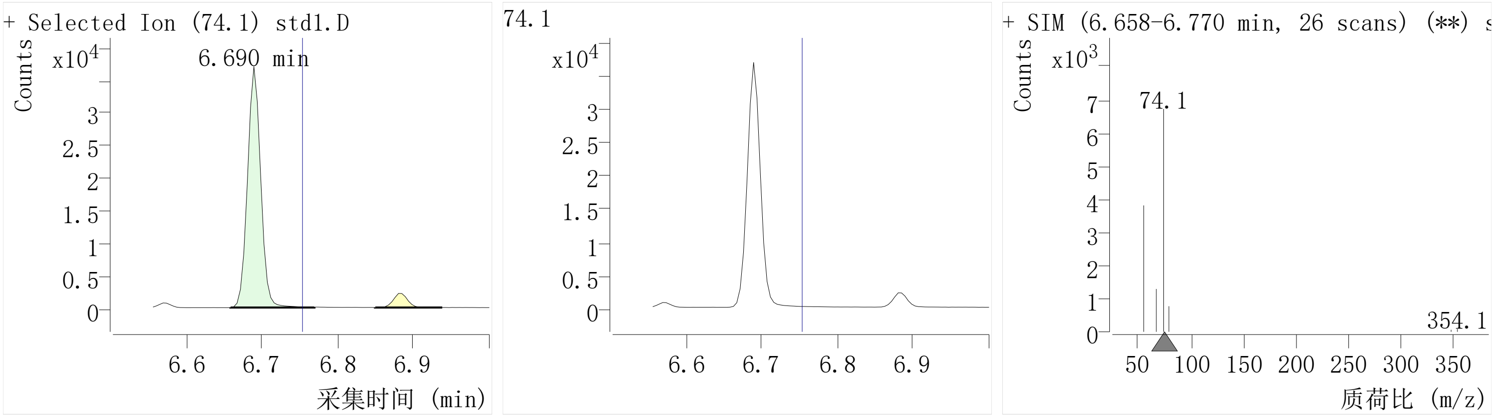

C16:1

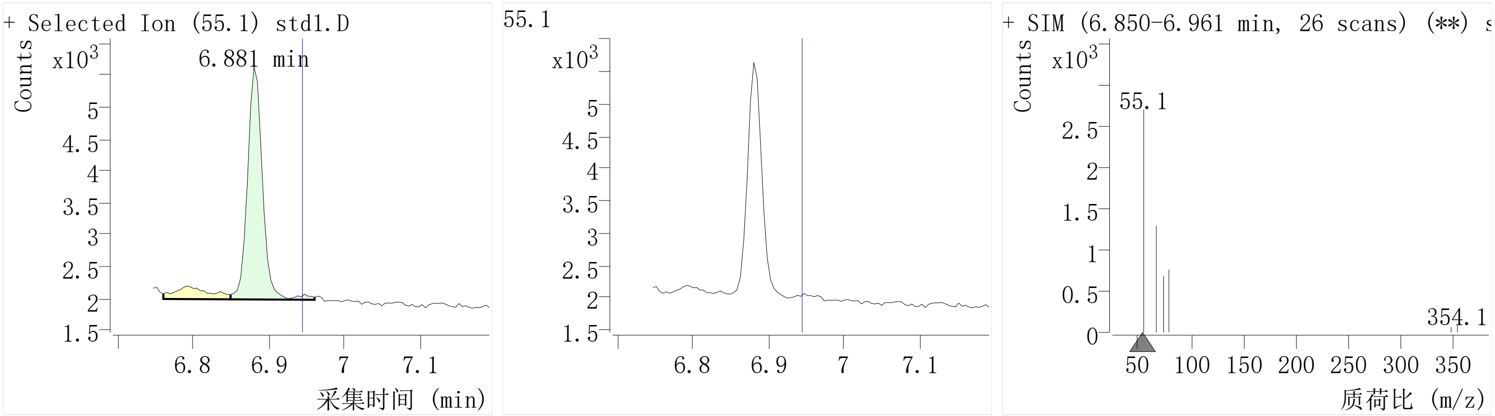

C17:0

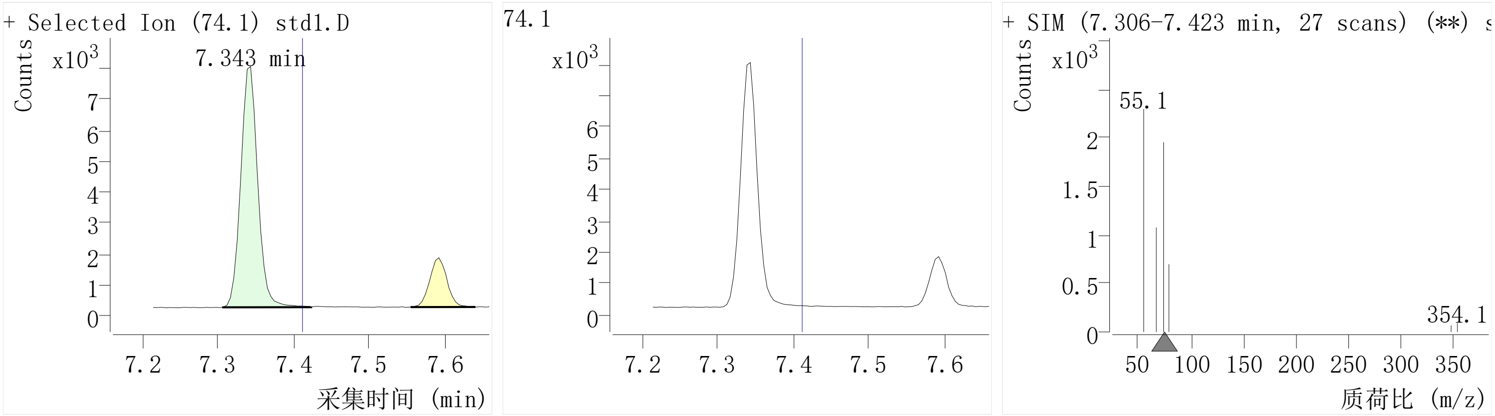

C17:1

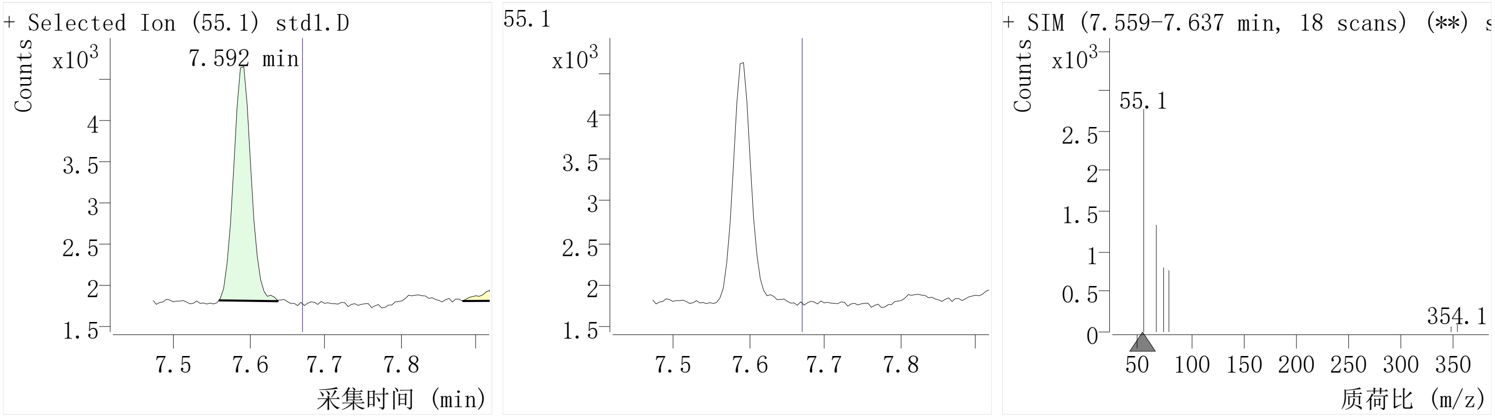

C18:0

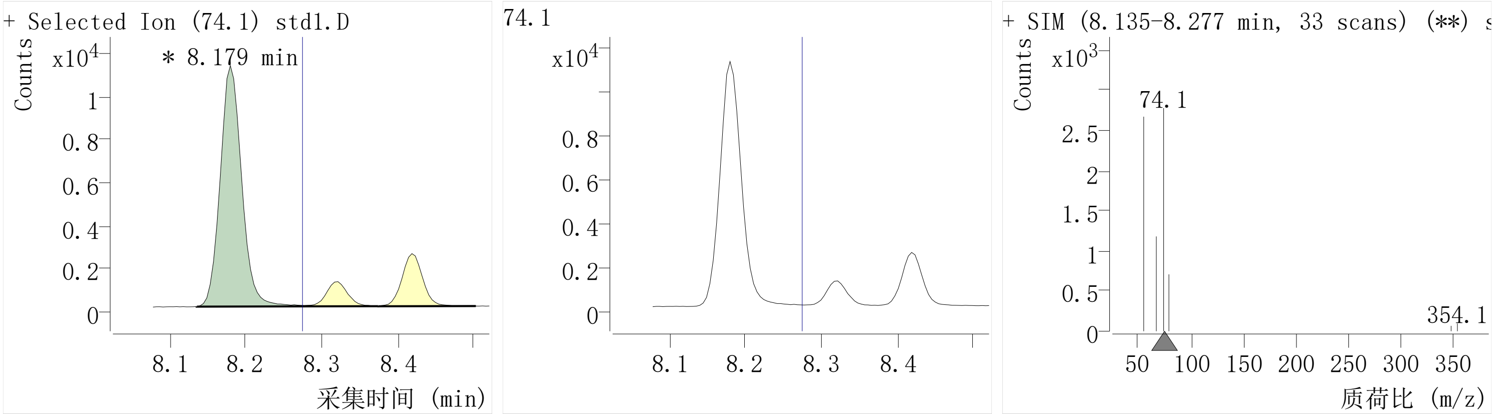

C18:1n9t

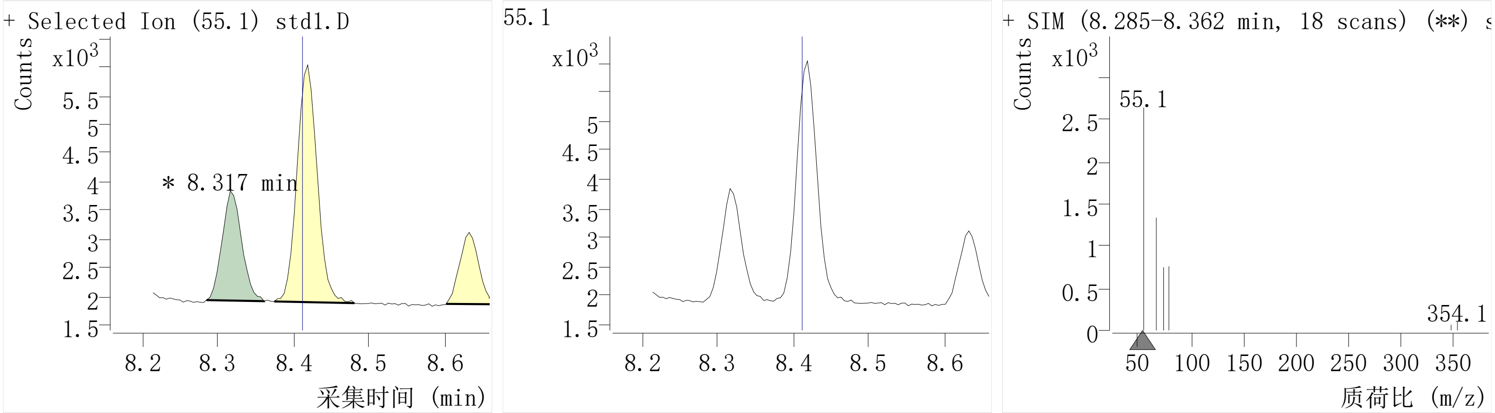

C18:1n9c

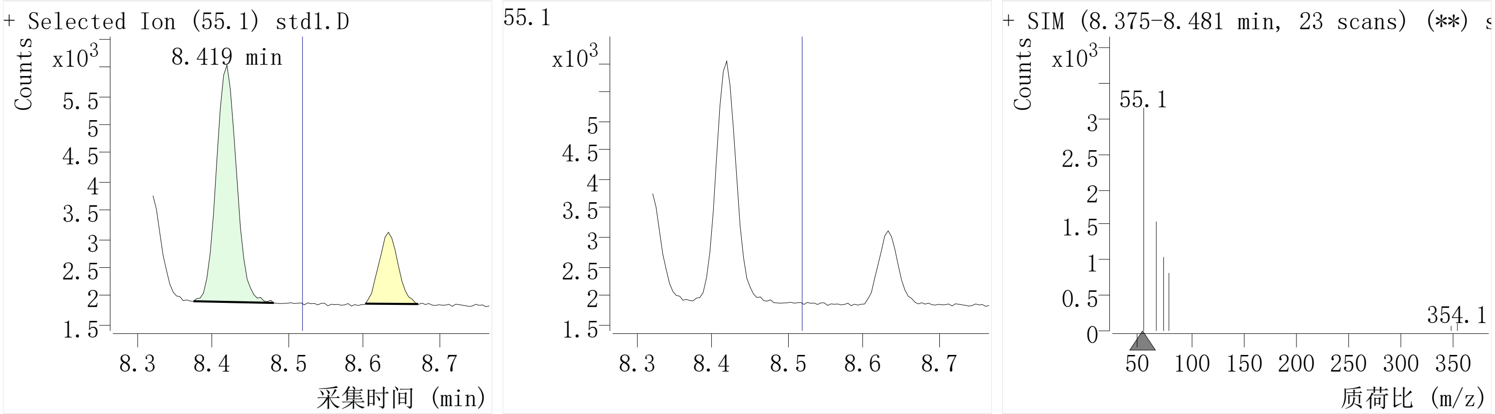

C18:2n6t

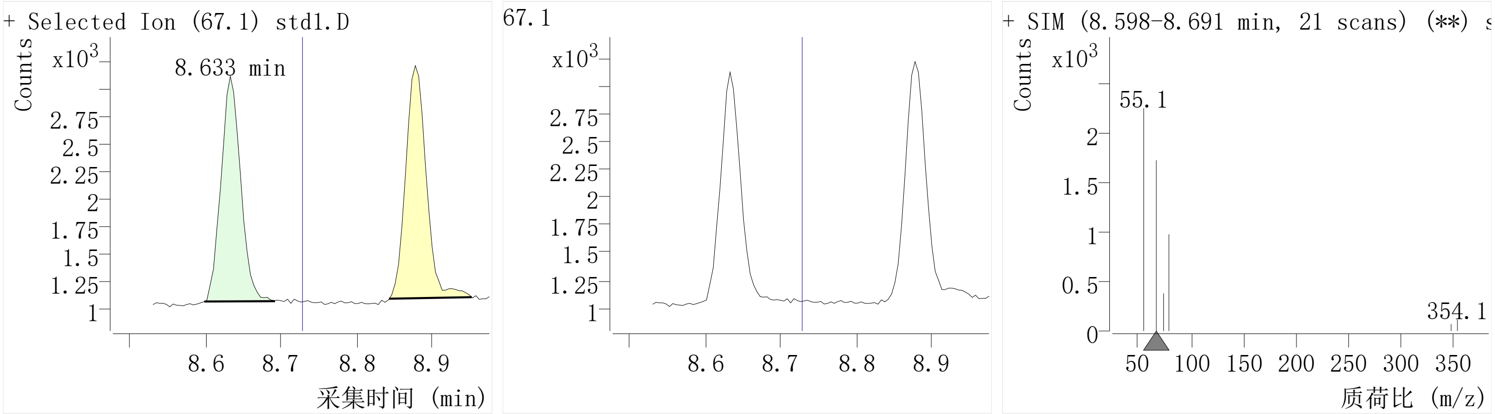

C18:2n6c

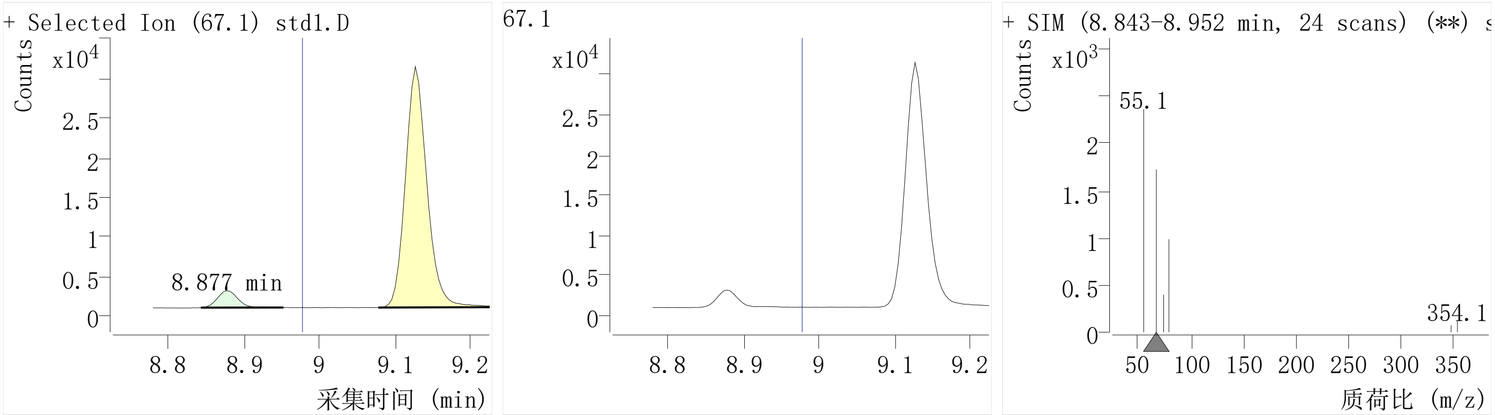

C18:3n6

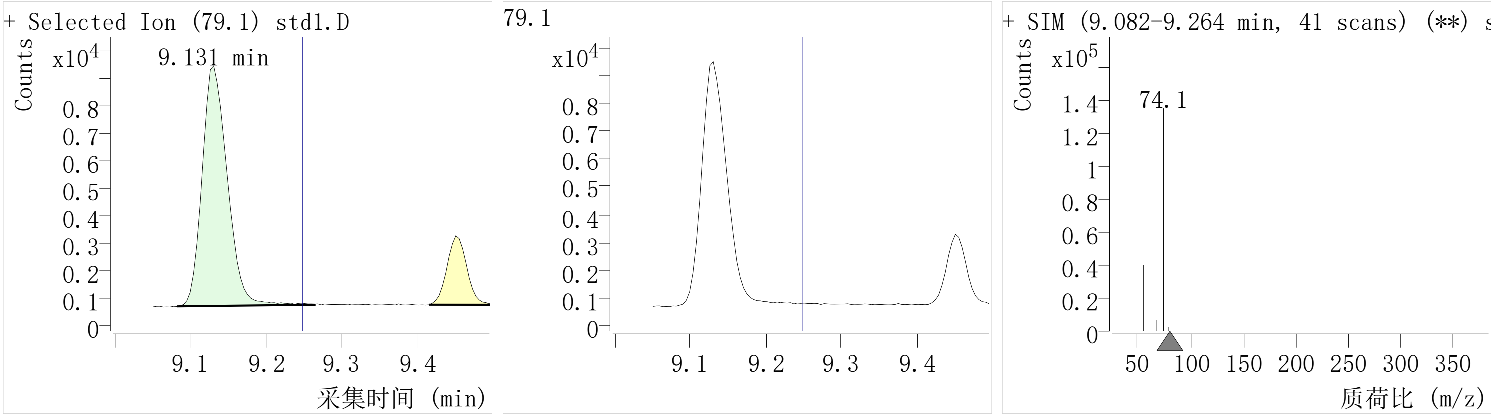

C18:3n3

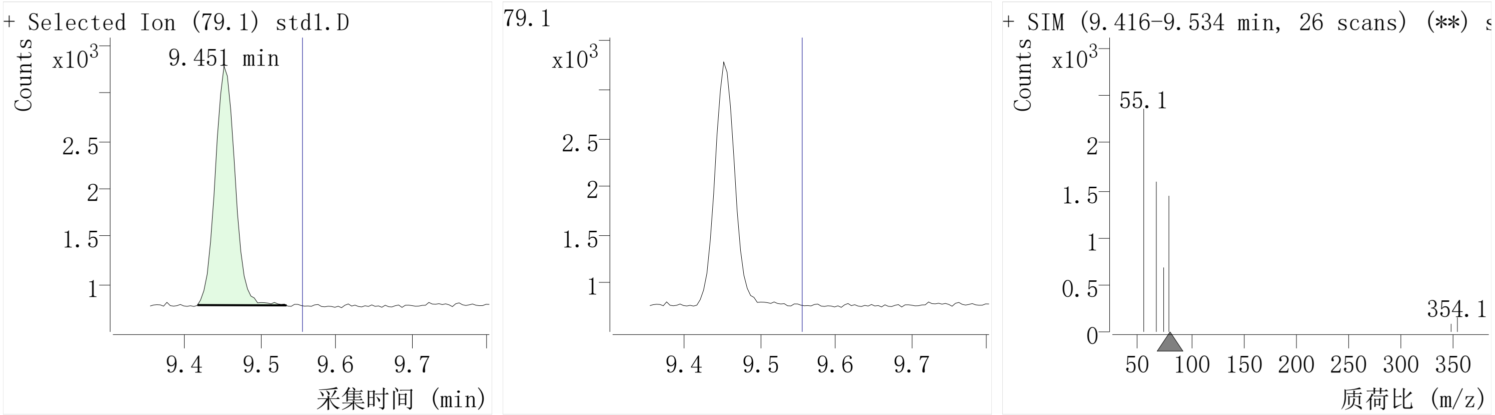

C20:0

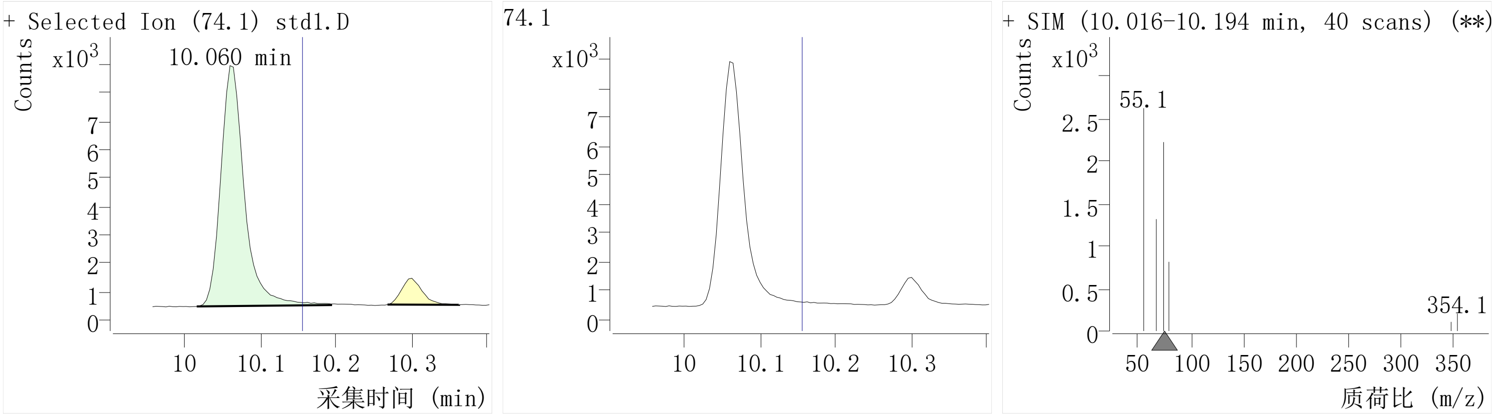

C20:1

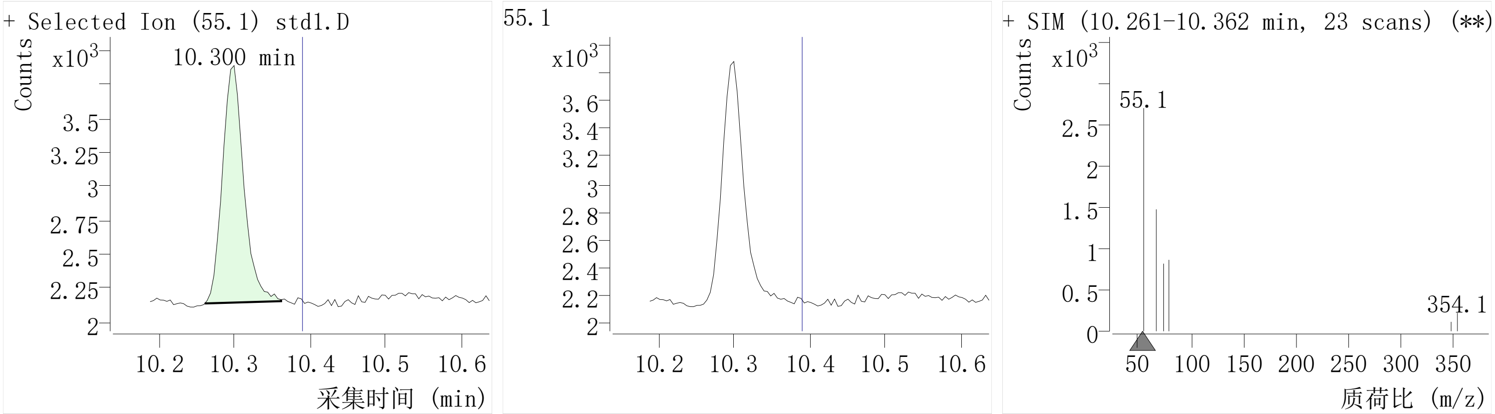

C20:2

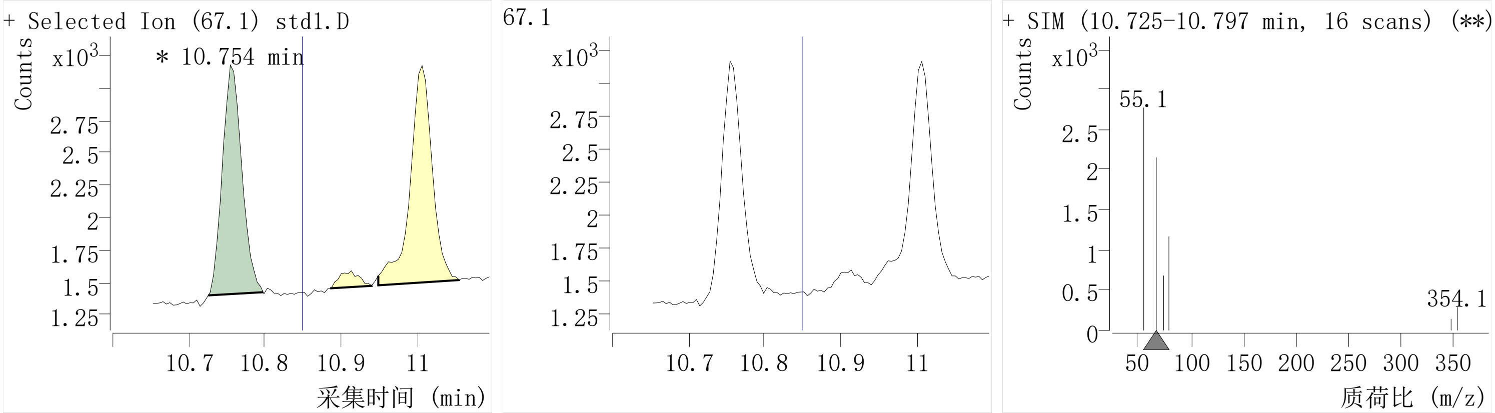

C21:0

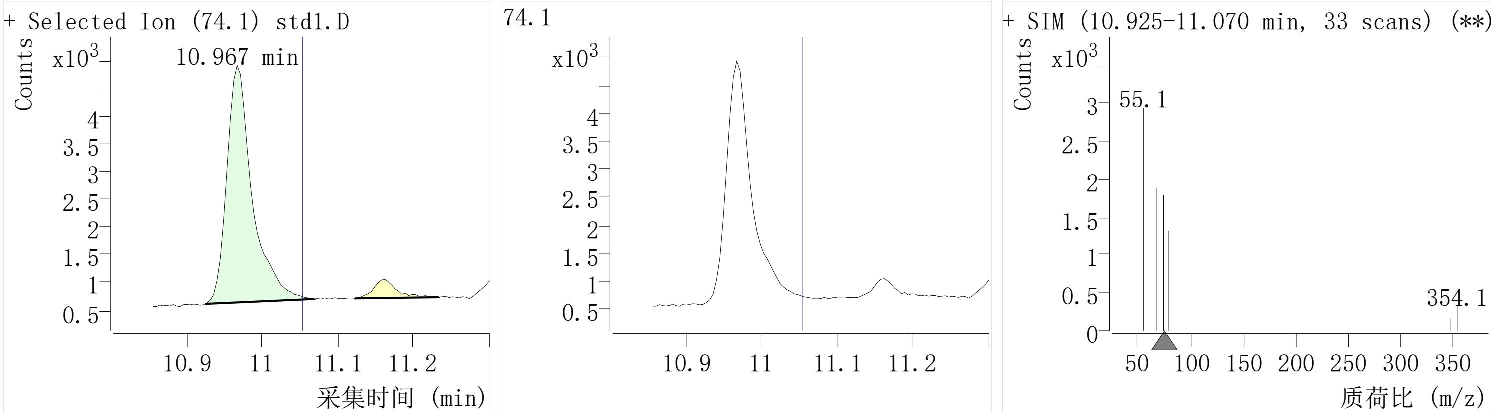

C20:3n6

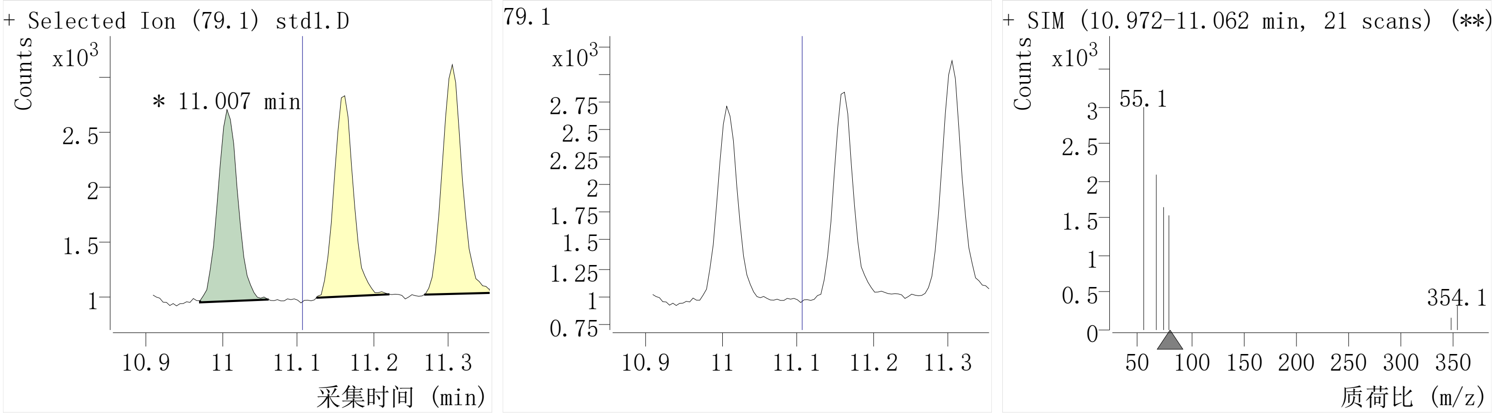

C20:4n6

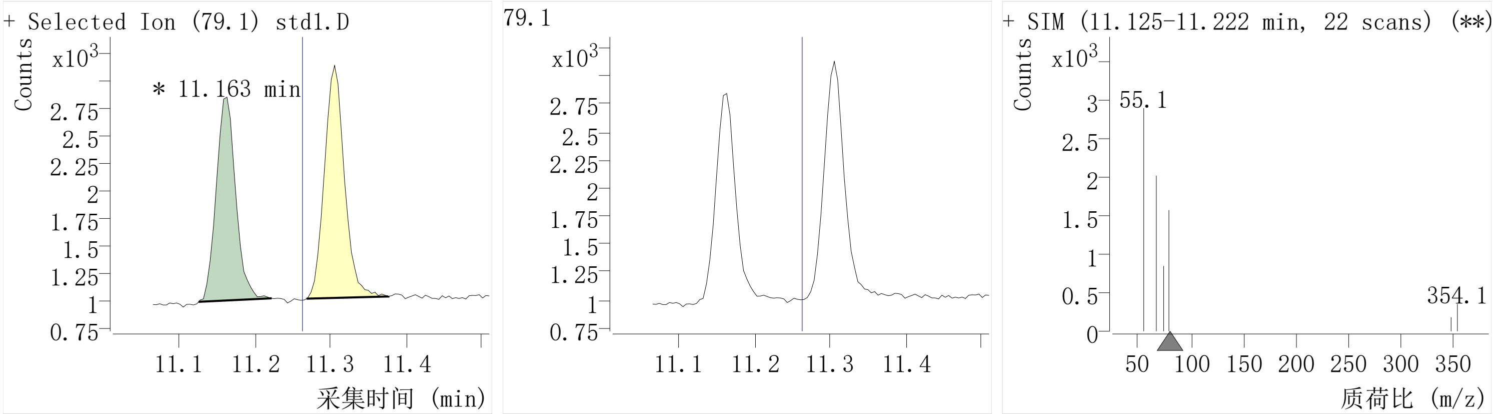

C20:3n3

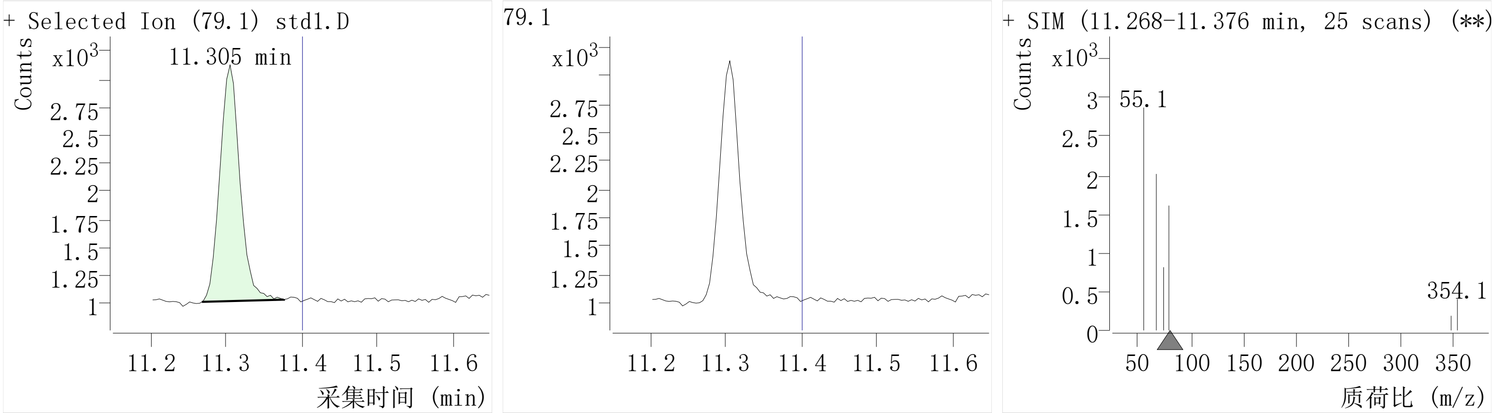

C20:5n3

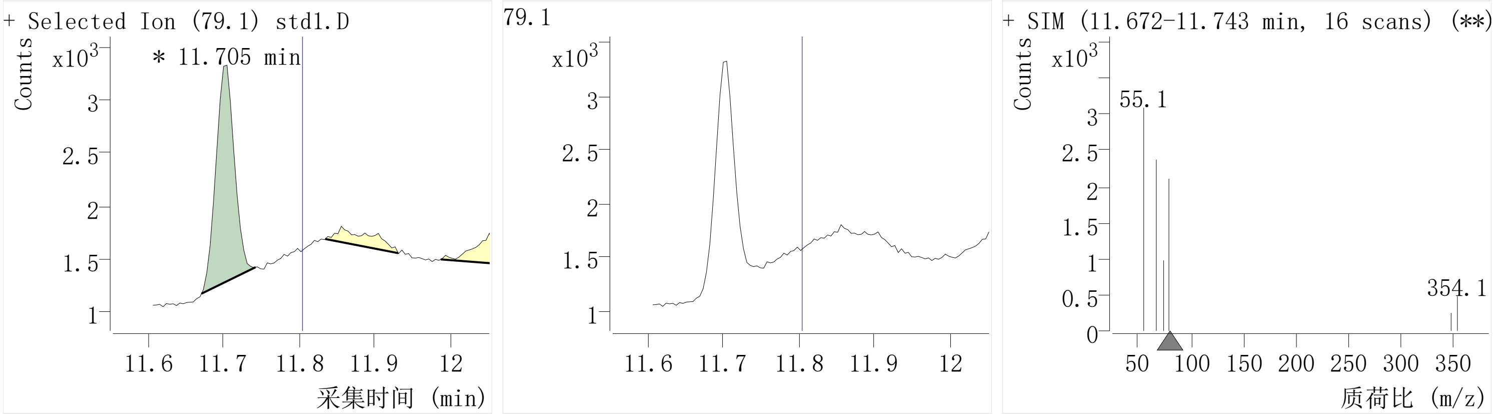

C22:0

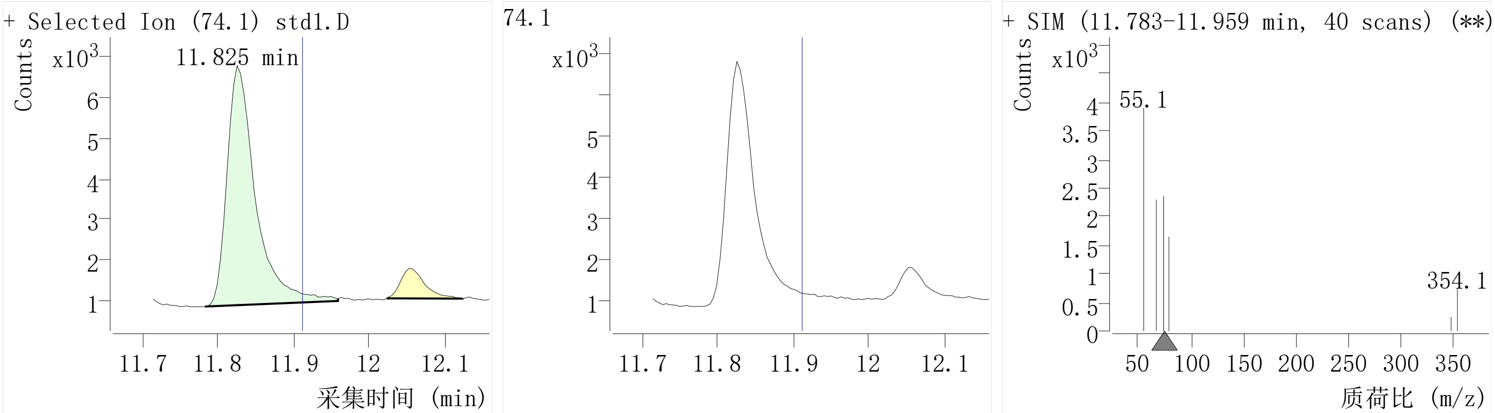

C22:1n9

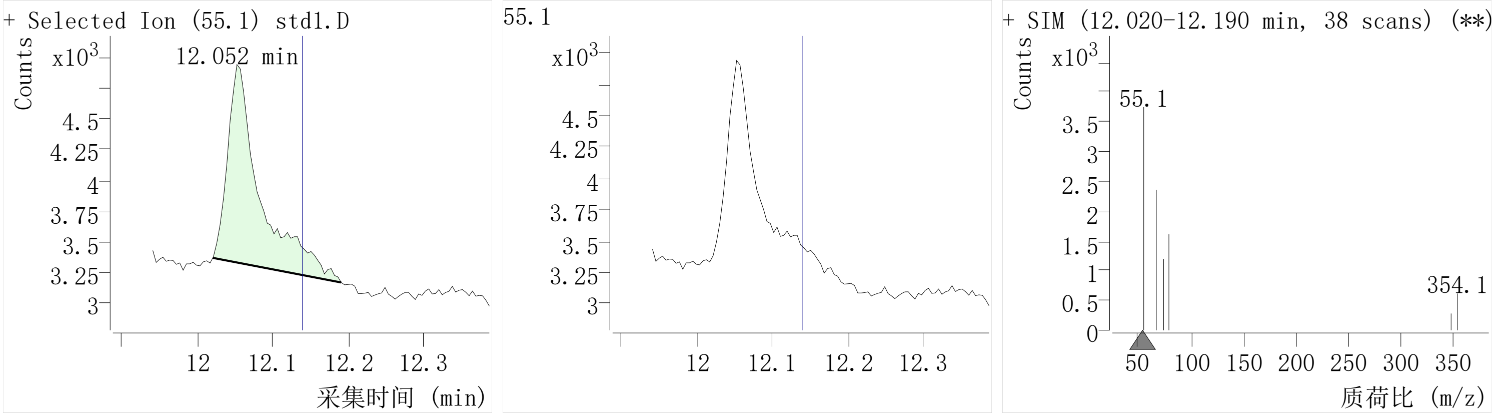

C22:2n6

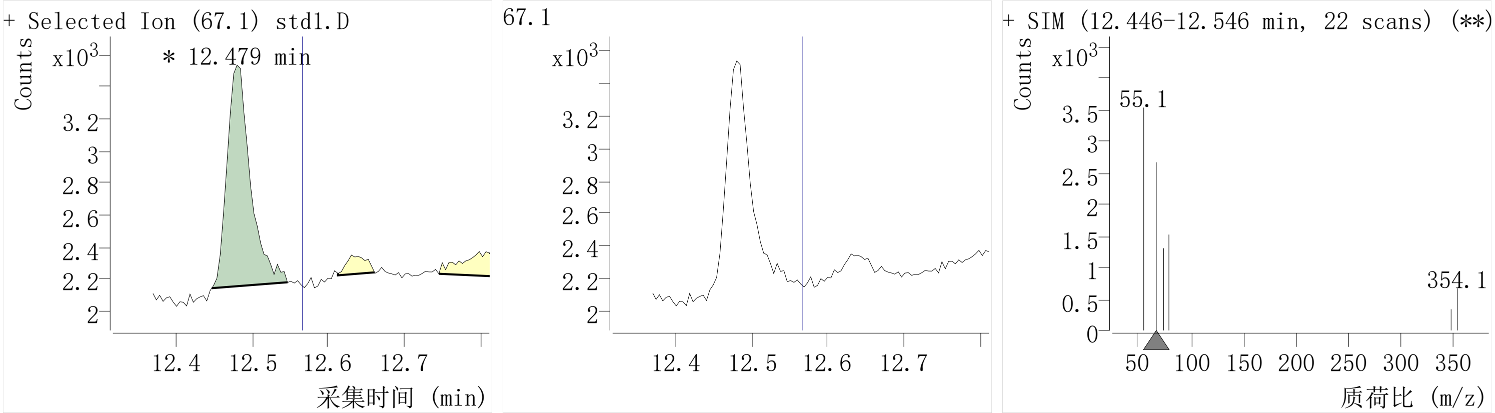

C23:0

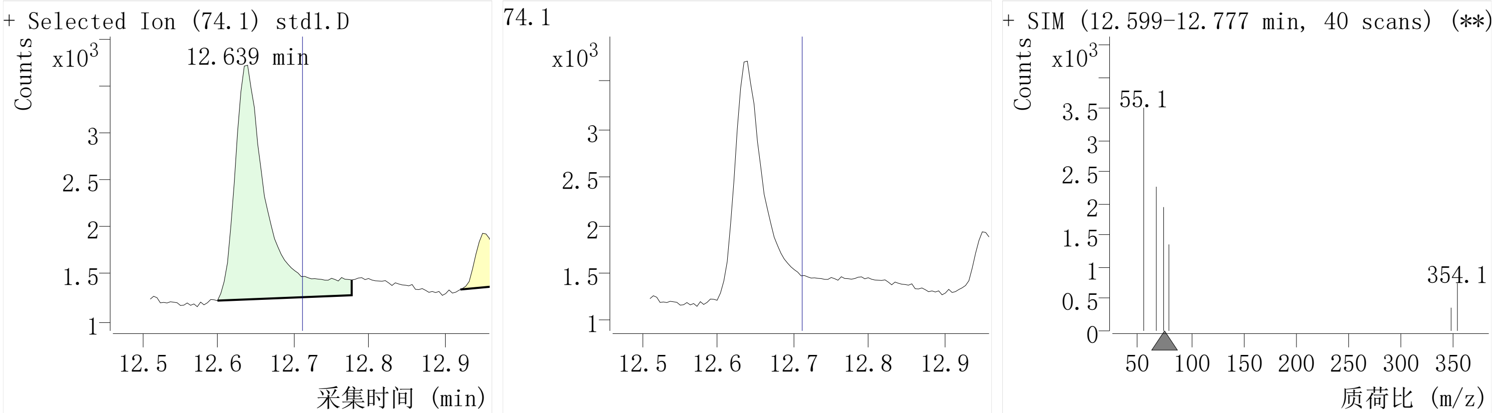

C24:0

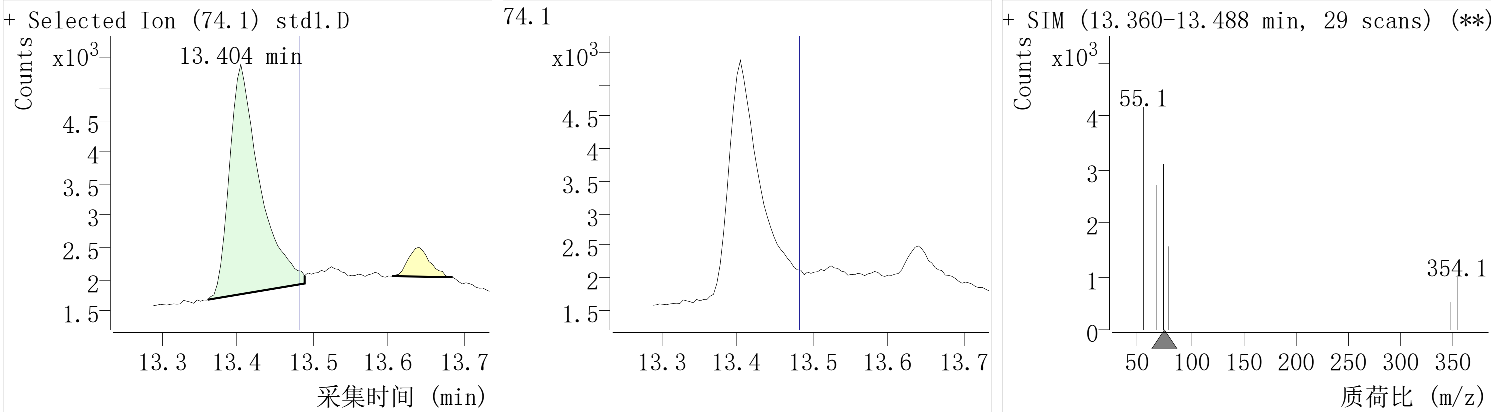

C22:6

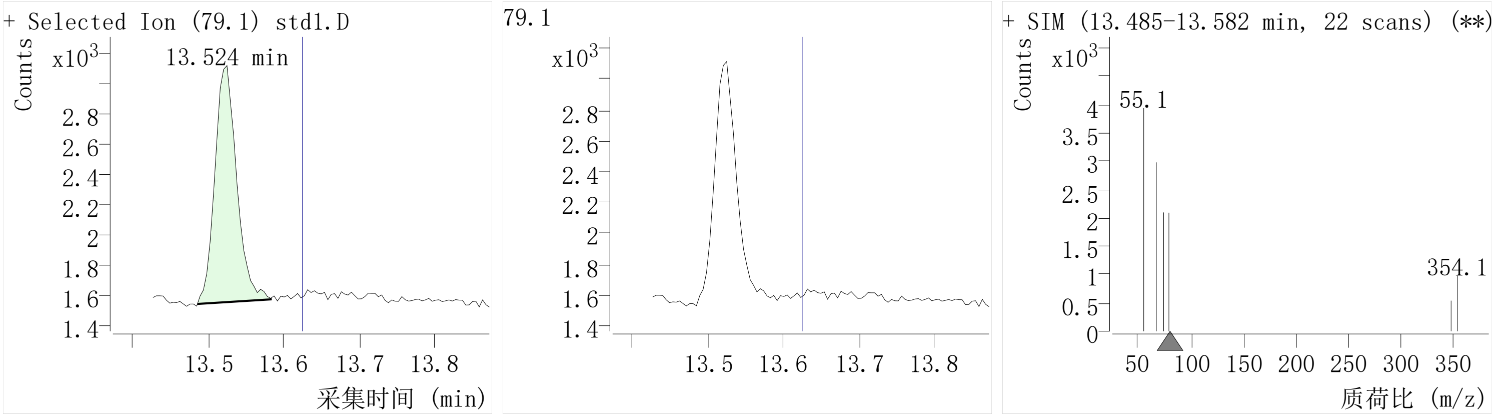
+ Selected Ion (79.1) std1.D
Counts
x10^3
13.524 min
2.8
2.6
2.4
2.2
2
1.8
1.6
1.4
13.5
13.6
13.7
13.8
采集时间 (min)
79.1
+ SIM (13.485-13.582 min, 22 scans) (**)
55.1
354.1
4
3.5
3
2.5
2
1.5
1
0.5
0
50
100
150
200
250
300
350
质荷比 (m/z)

C24:1

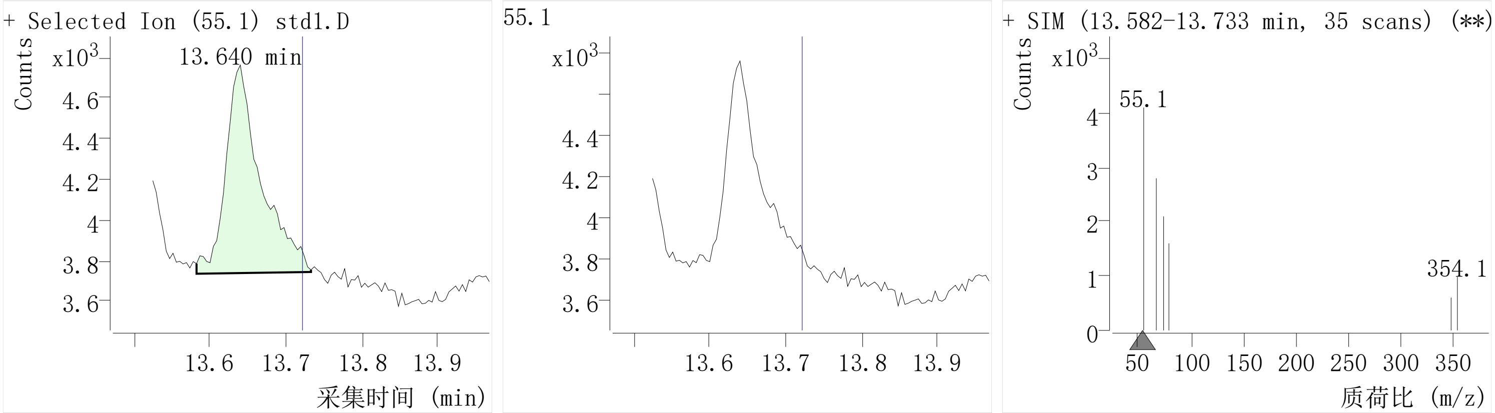
+ Selected Ion (55.1) std1.D
Counts
x10^3
13.640 min
4.6
4.4
4.2
4
3.8
3.6
13.6
13.7
13.8
13.9
采集时间 (min)
55.1
+ SIM (13.582-13.733 min, 35 scans) (**)
55.1
354.1
4
3
2
1
0
50
100
150
200
250
300
350
质荷比 (m/z)

**批处理路径**	G:\GC-MS\HX250430-4-GCMS总脂肪酸靶向检测\HX250430-4\QuantResults\HX250430-4.batch.bin		
**分析时间**	2025/5/14 16:58	**分析员姓名**	DESKTOP-M3A0GPO\omics
**报告时间**	2025/5/16 14:53:00	**报告员姓名**	DESKTOP-M3A0GPO\omics
**最近校正更新**	2025/5/14 16:58	**批处理状态**	已处理
**定量批处理版本**	10.2	**定量报告版本**	10.2

**采集时间**	2025/5/8 17:49	**数据文件**	std2.D
**样品类型**	校正	**样品名称**	std2
**稀释**	1	**采集方法**	脂肪酸

样品色谱图

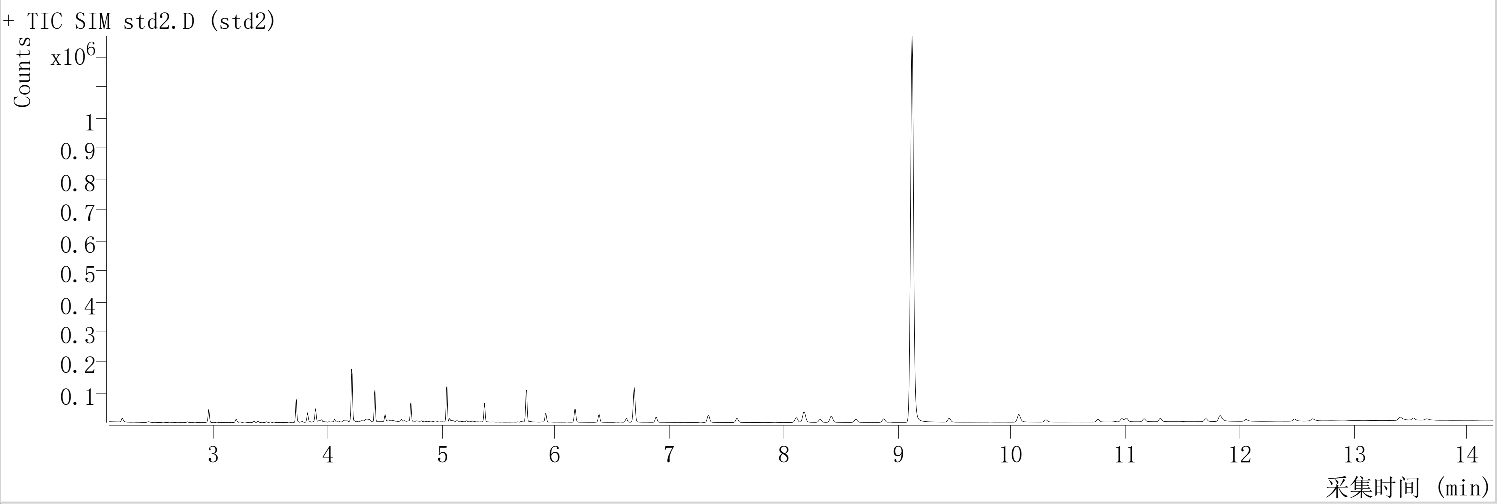

化合物	ISTD	RT	响应	ISTD 响应	响应比	最终浓度	单位
C4:0	C19:0	2.203	13000	1765013	0.0074	0.3401	ug/ml
C6:0	C19:0	2.959	29610	1765013	0.0168	0.6475	ug/ml
C8:0	C19:0	3.728	45885	1765013	0.0260	0.5471	ug/ml
C10:0	C19:0	4.418	62998	1765013	0.0357	0.6650	ug/ml
C11:0	C19:0	4.733	35366	1765013	0.0200	0.3294	ug/ml
C12:0	C19:0	5.049	74112	1765013	0.0420	0.6198	ug/ml
C13:0	C19:0	5.378	37633	1765013	0.0213	0.3119	ug/ml
C14:0	C19:0	5.747	76645	1765013	0.0434	0.7959	ug/ml
C14:1	C19:0	5.916	14620	1765013	0.0083	0.3110	ug/ml
C15:0	C19:0	6.174	36008	1765013	0.0204	0.2970	ug/ml
C15:1	C19:0	6.383	16056	1765013	0.0091	0.0953	ug/ml
C16:0	C19:0	6.690	105704	1765013	0.0599	0.3444	ug/ml
C16:1	C19:0	6.881	10789	1765013	0.0061	0.2852	ug/ml
C17:0	C19:0	7.343	28406	1765013	0.0161	0.3358	ug/ml
C17:1	C19:0	7.592	10861	1765013	0.0062	0.3025	ug/ml
C18:0	C19:0	8.179	51052	1765013	0.0289	0.6723	ug/ml
C18:1n9t	C19:0	8.317	8141	1765013	0.0046	0.2721	ug/ml
C18:1n9c	C19:0	8.419	18398	1765013	0.0104	0.4785	ug/ml
C18:2n6t	C19:0	8.633	9023	1765013	0.0051	0.2990	ug/ml
C18:2n6c	C19:0	8.877	9822	1765013	0.0056	0.3862	ug/ml
C18:3n6	C19:0	9.135	30134	1765013	0.0171	0.3084	ug/ml
C18:3n3	C19:0	9.456	10535	1765013	0.0060	0.2905	ug/ml
C20:0	C19:0	10.065	39690	1765013	0.0225	0.6012	ug/ml
C20:1	C19:0	10.300	7385	1765013	0.0042	0.2978	ug/ml
C20:2	C19:0	10.758	7375	1765013	0.0042	0.3102	ug/ml
C21:0	C19:0	10.967	19624	1765013	0.0111	0.3060	ug/ml
C20:3n6	C19:0	11.007	7286	1765013	0.0041	0.3049	ug/ml
C20:4n6	C19:0	11.163	7641	1765013	0.0043	0.3052	ug/ml
C20:3n3	C19:0	11.305	8777	1765013	0.0050	0.3110	ug/ml
C20:5n3	C19:0	11.706	8699	1765013	0.0049	0.3076	ug/ml

化合物	ISTD	RT	响应	ISTD 响应	响应比	最终浓度	单位
C22:0	C19:0	11.830	31532	1765013	0.0179	0.5987	ug/ml
C22:1n9	C19:0	12.052	6620	1765013	0.0038	0.2629	ug/ml
C22:2n6	C19:0	12.479	6142	1765013	0.0035	0.3119	ug/ml
C23:0	C19:0	12.639	14948	1765013	0.0085	0.3068	ug/ml
C24:0	C19:0	13.404	24127	1765013	0.0137	0.6227	ug/ml
C22:6	C19:0	13.524	7170	1765013	0.0041	0.3053	ug/ml
C24:1	C19:0	13.635	7332	1765013	0.0042	0.3612	ug/ml

C4:0

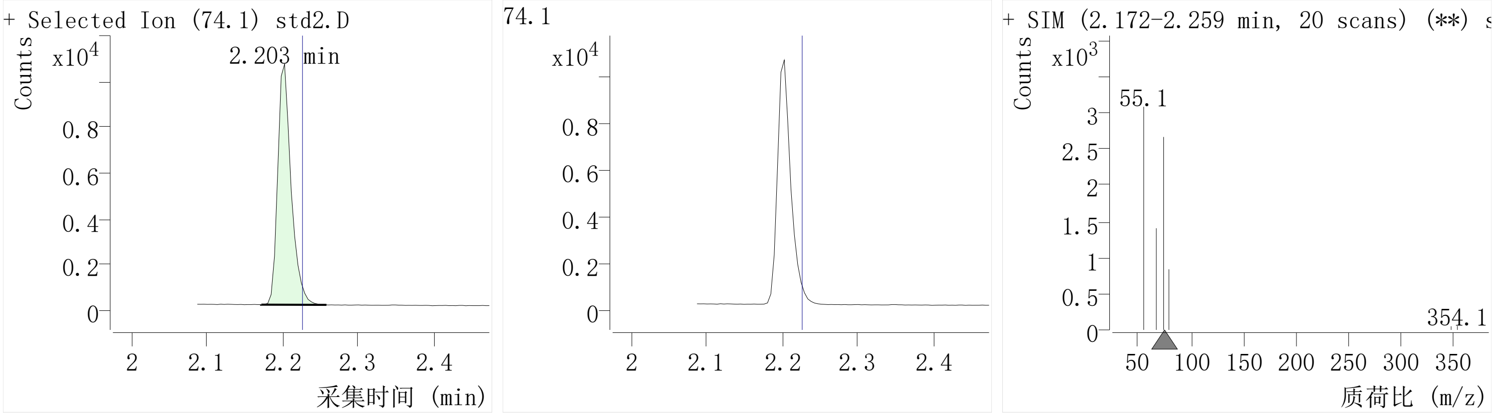

C6:0

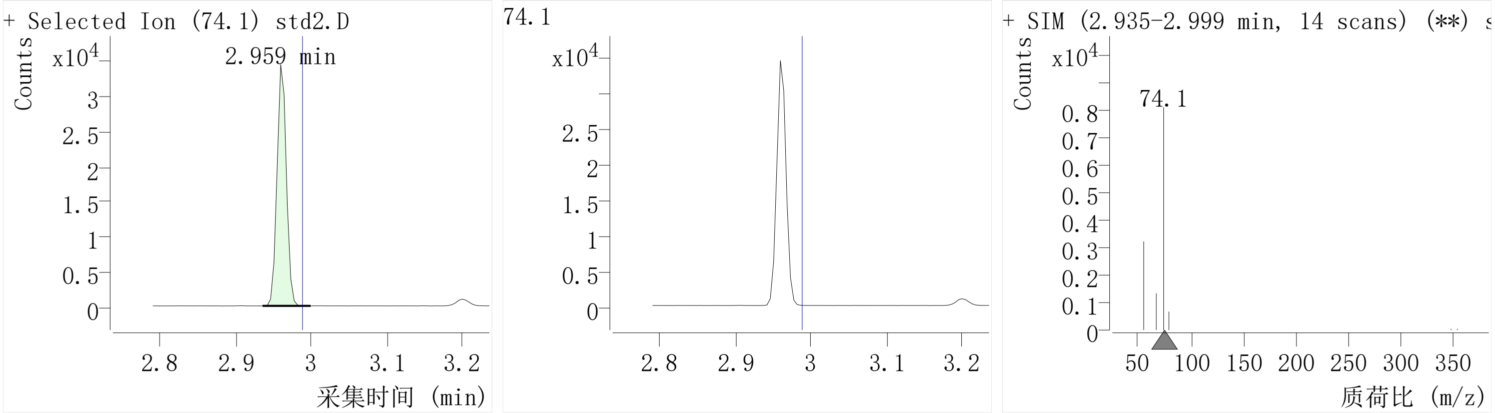

C8:0

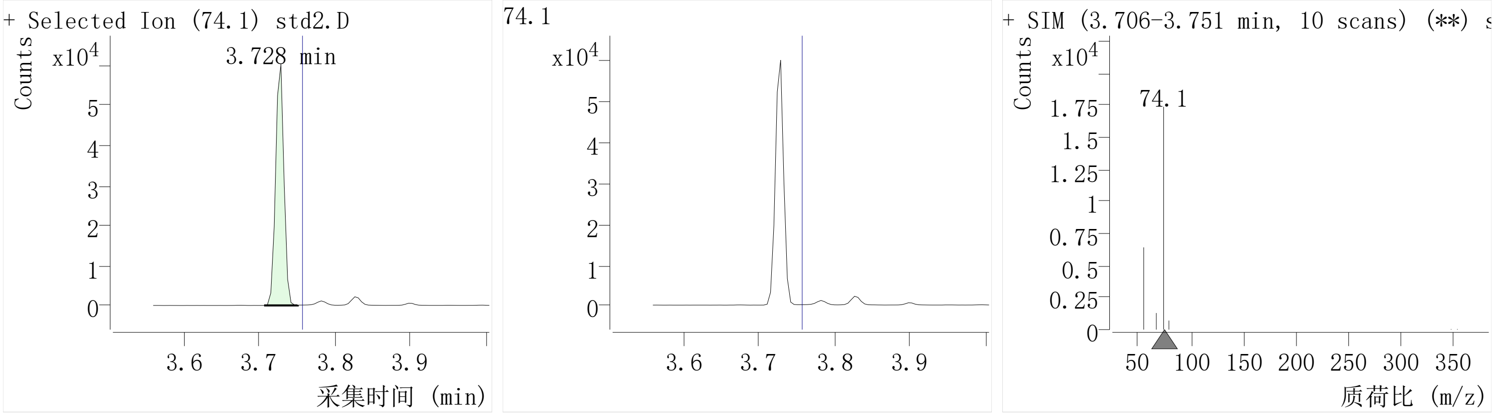

C10:0

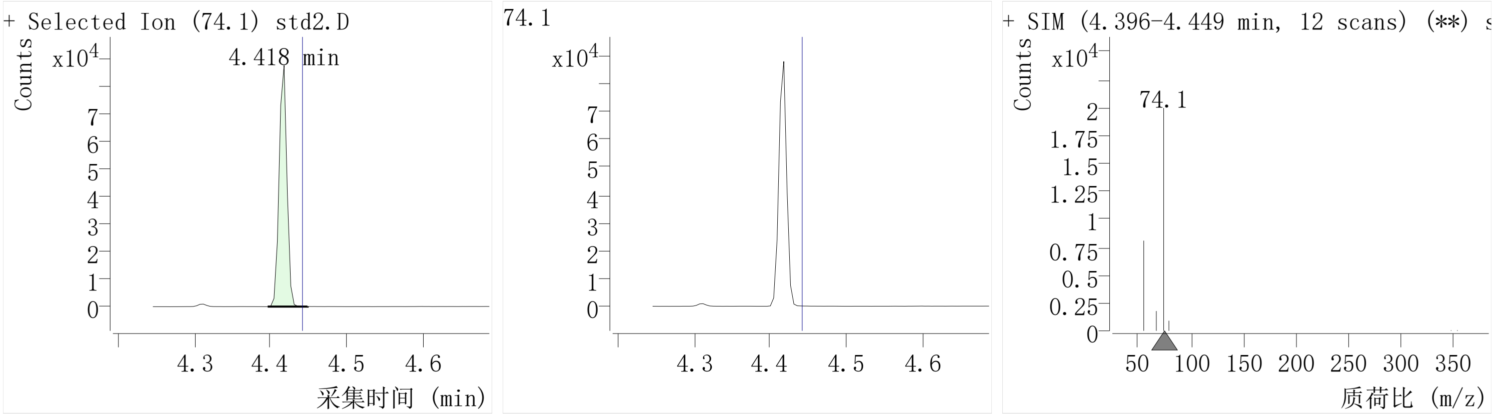

C11:0

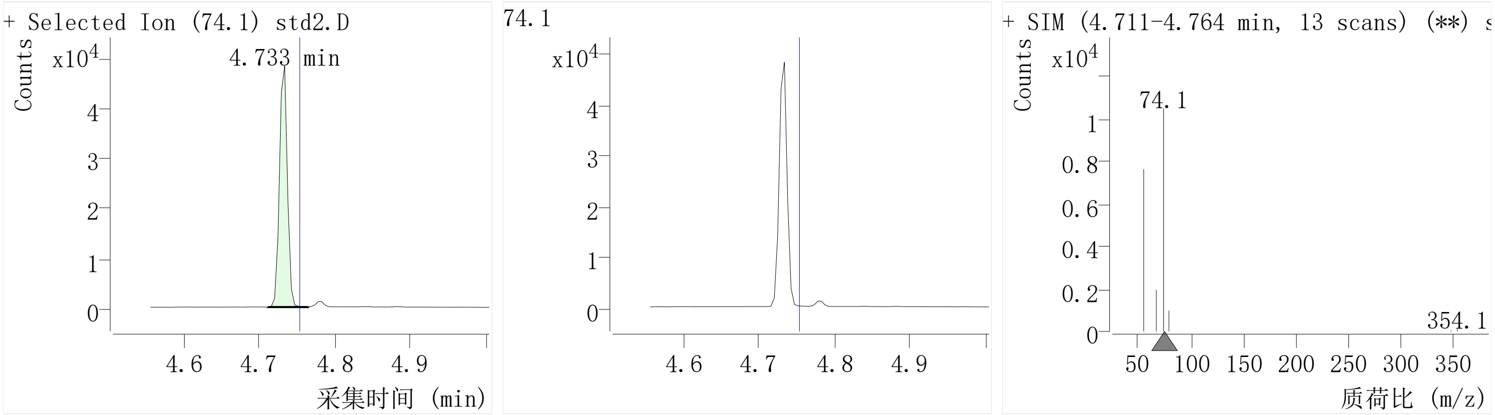

C12:0

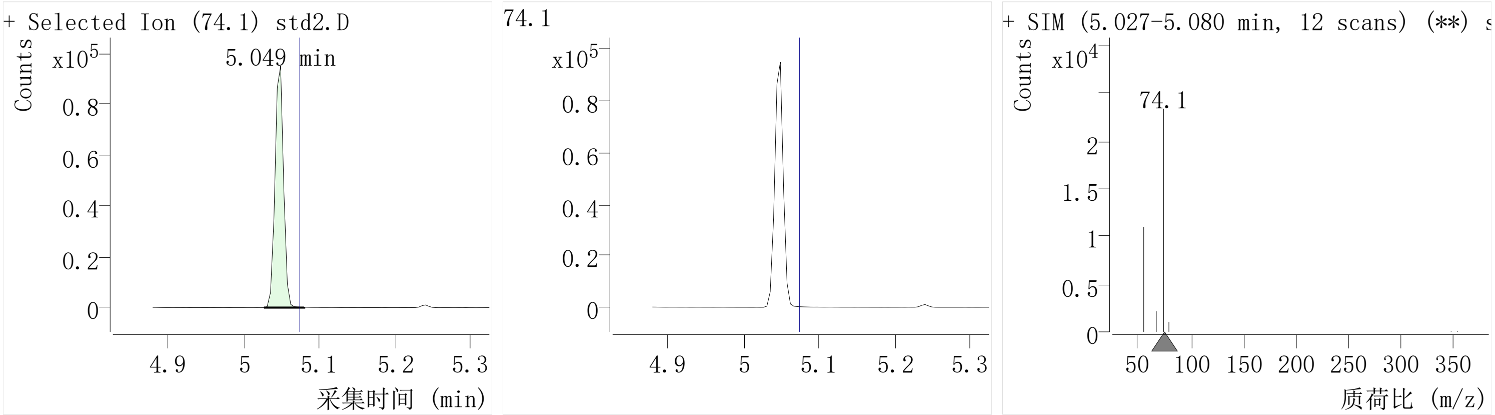

C13:0

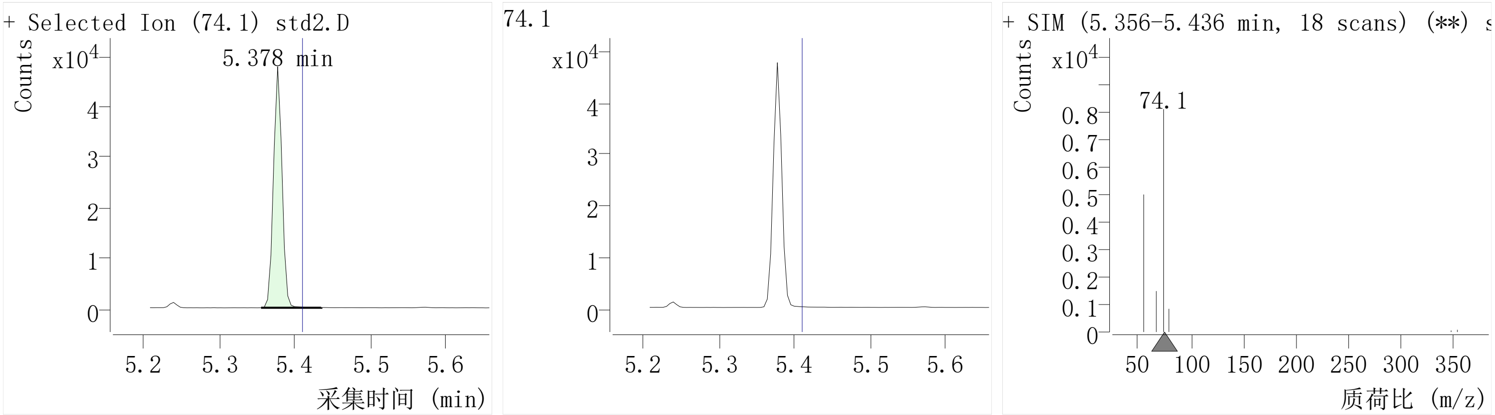

C14:0

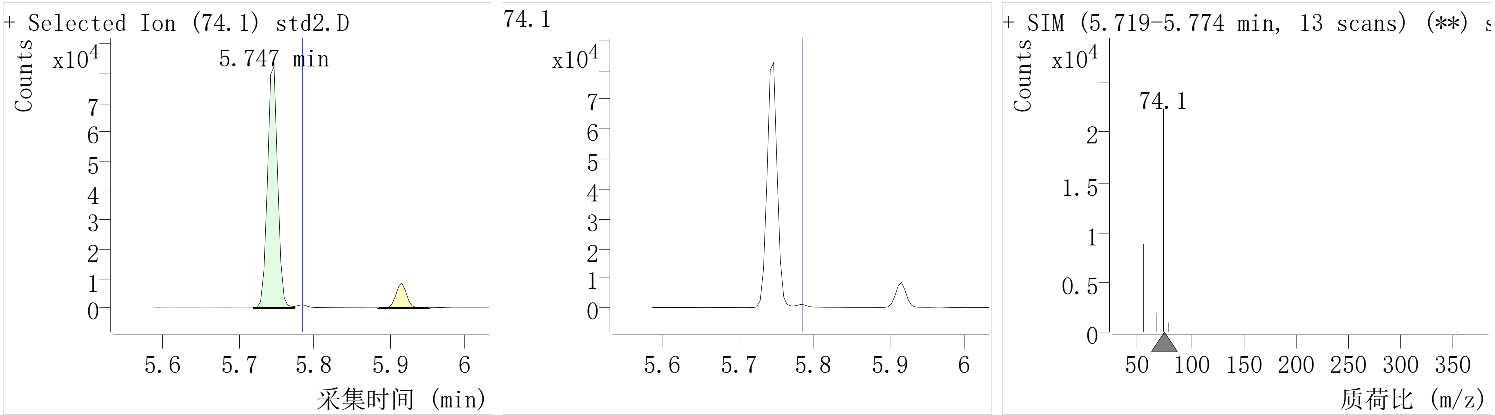

C14:1

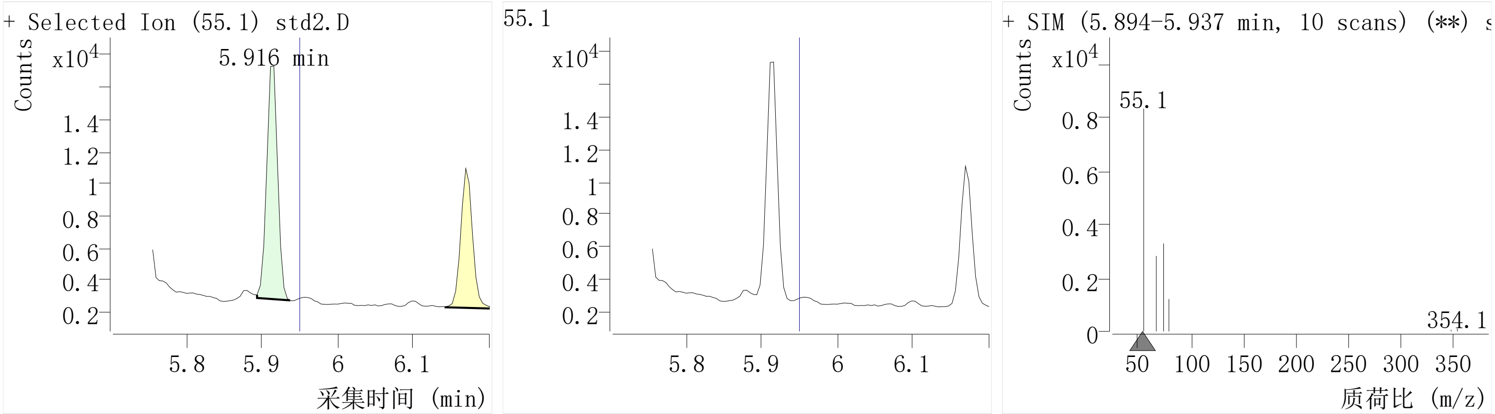

C15:0

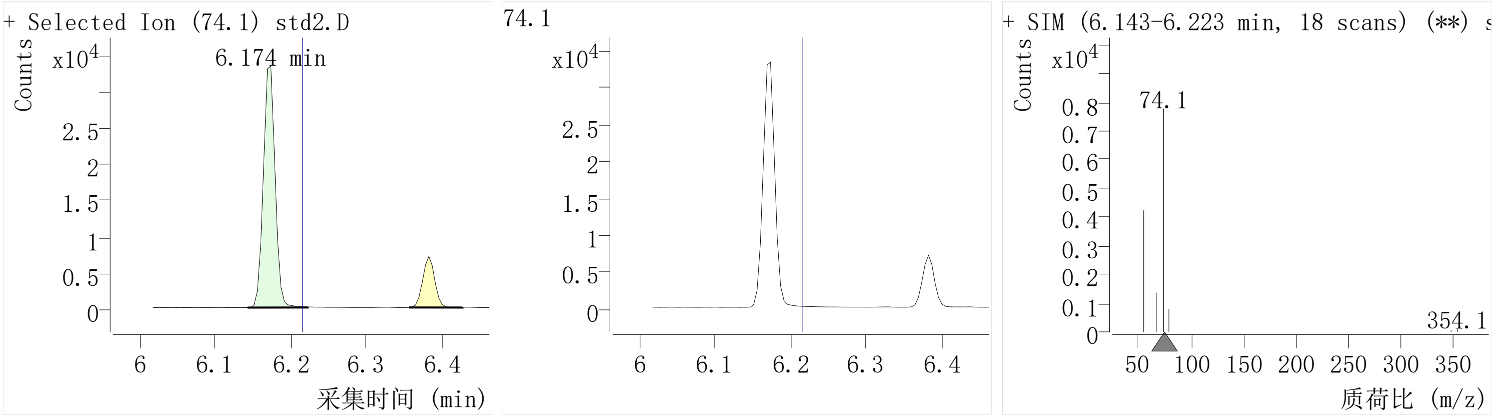

C15:1

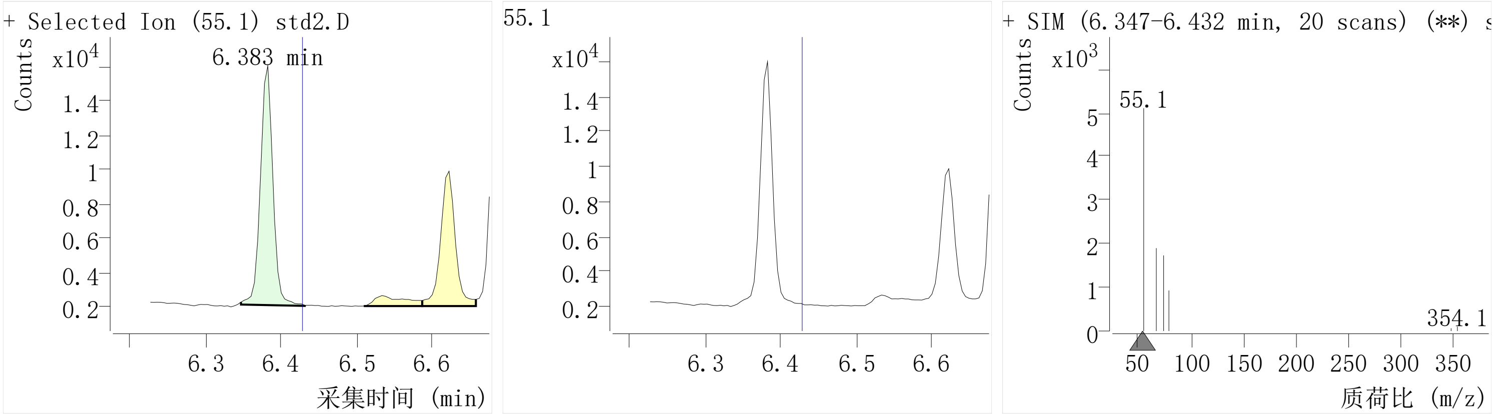

C16:0

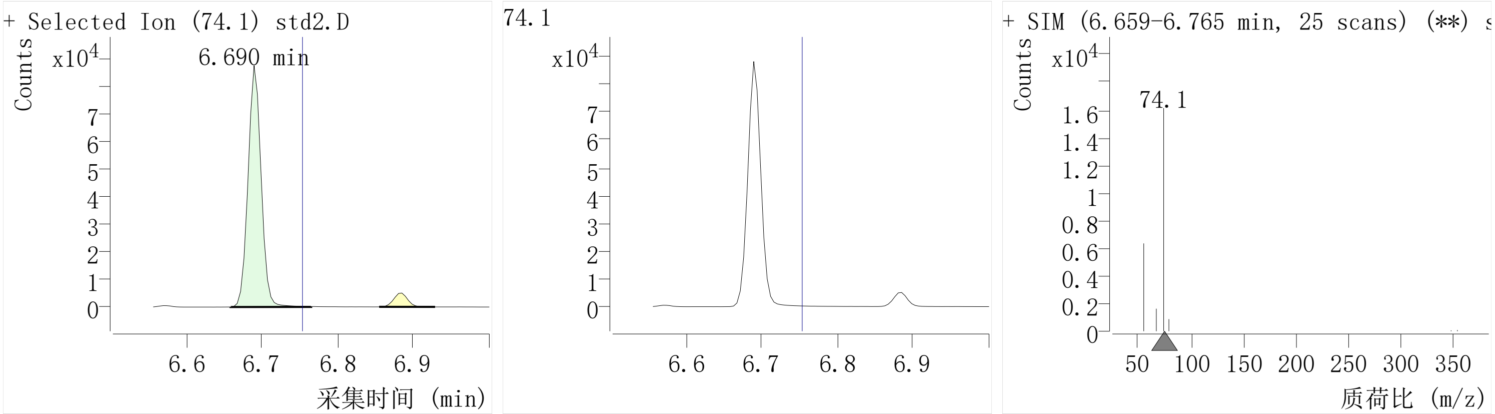

C16:1

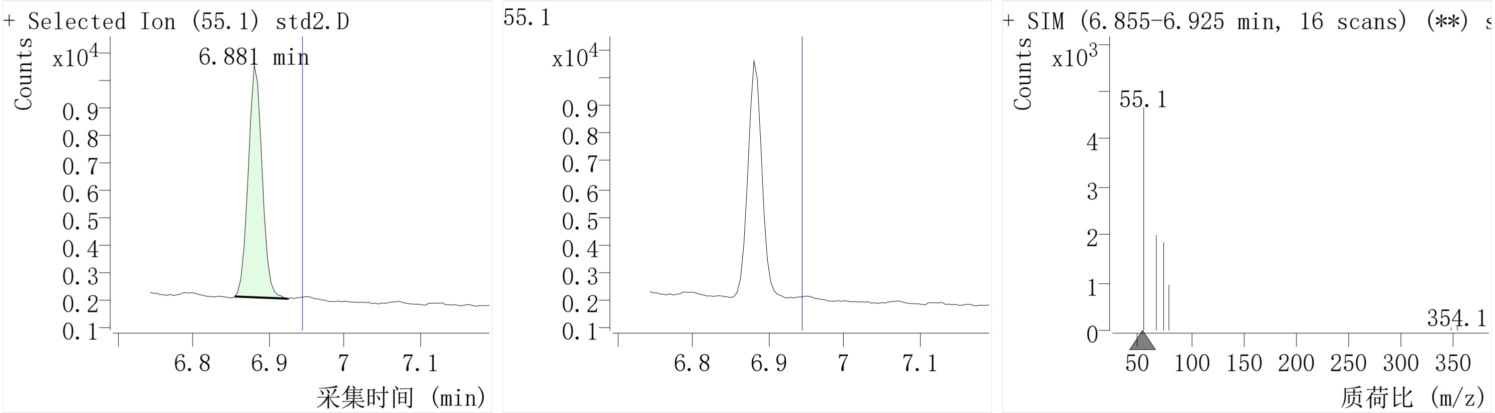

C17:0

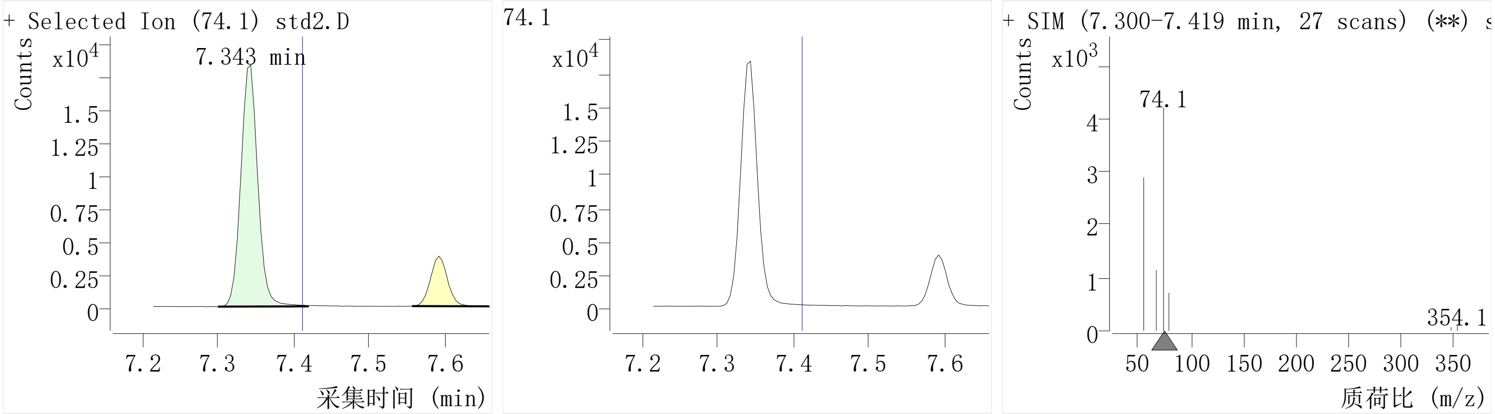

C17:1

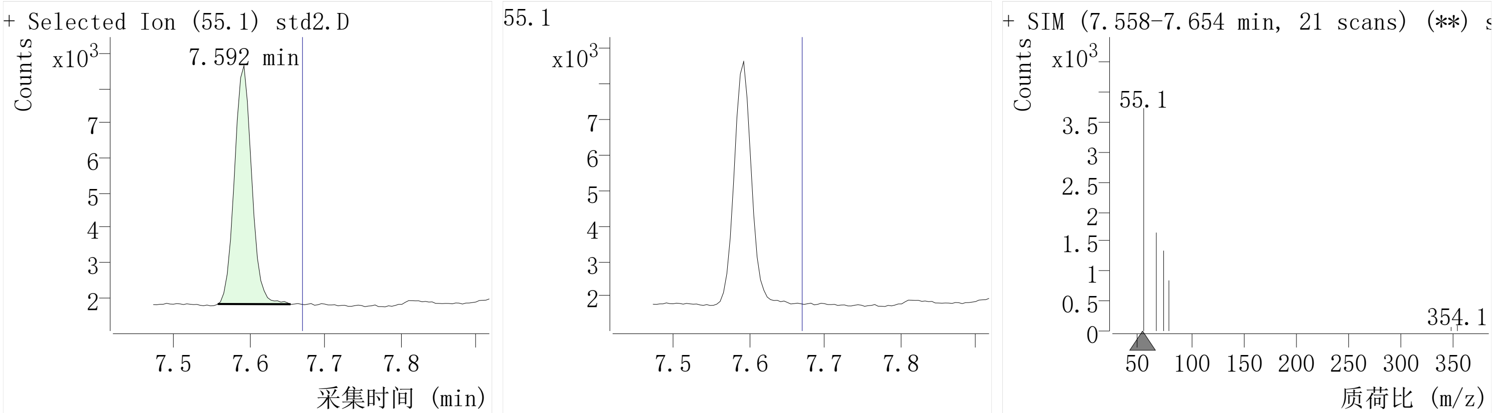

C18:0

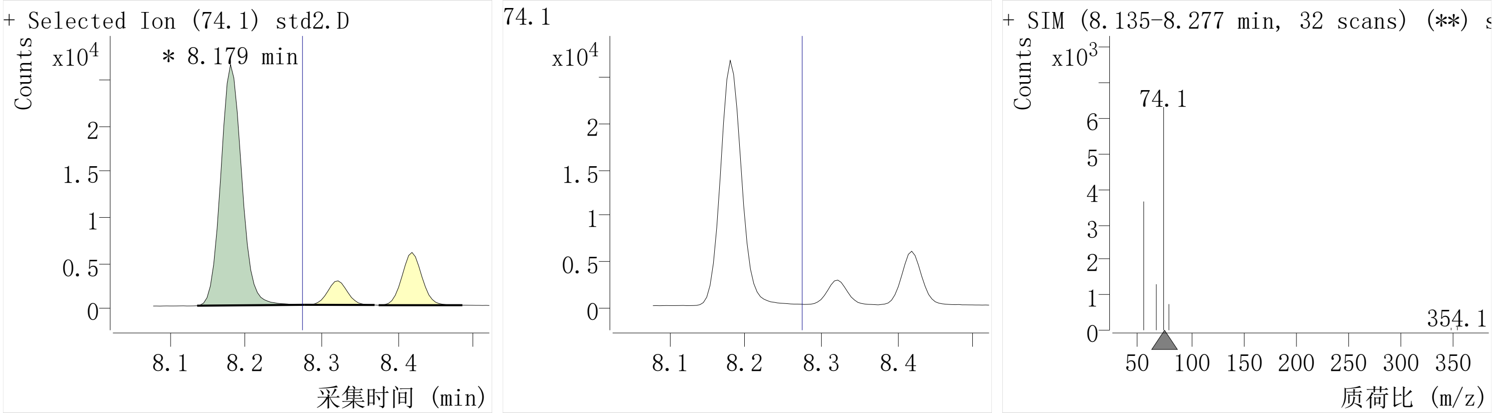

C18:1n9t

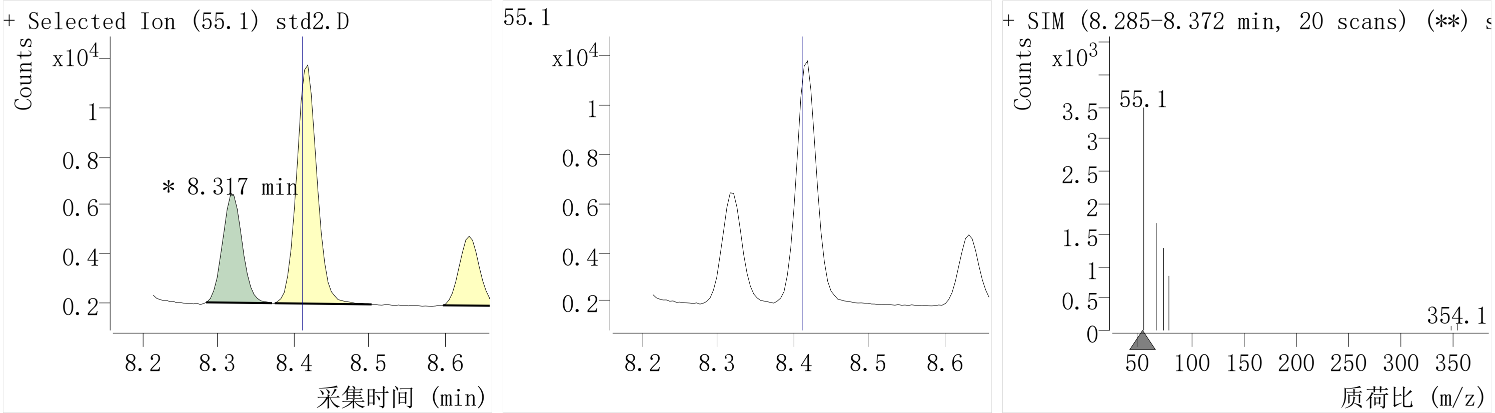

C18:1n9c

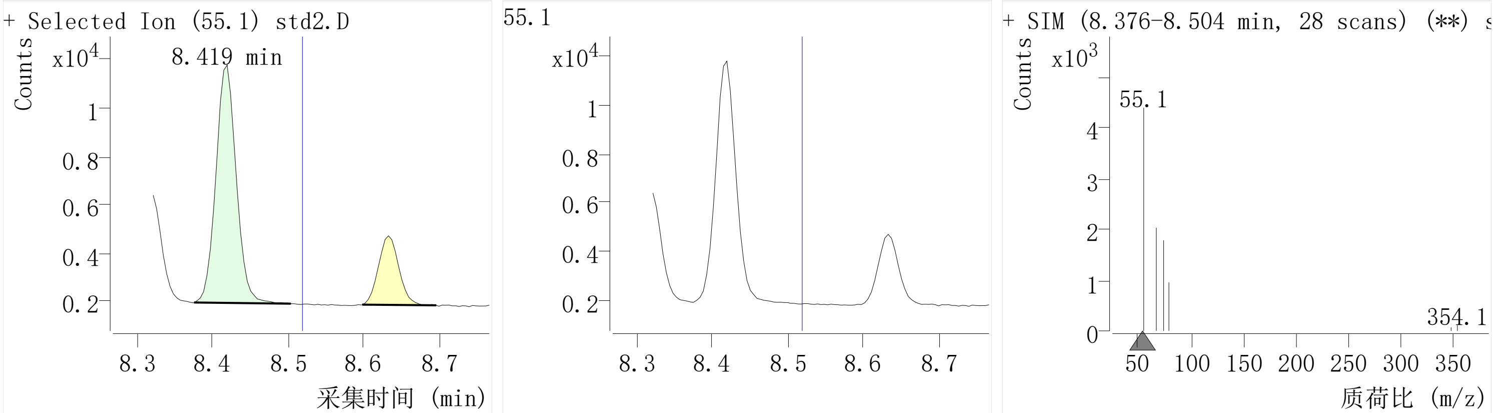

C18:2n6t

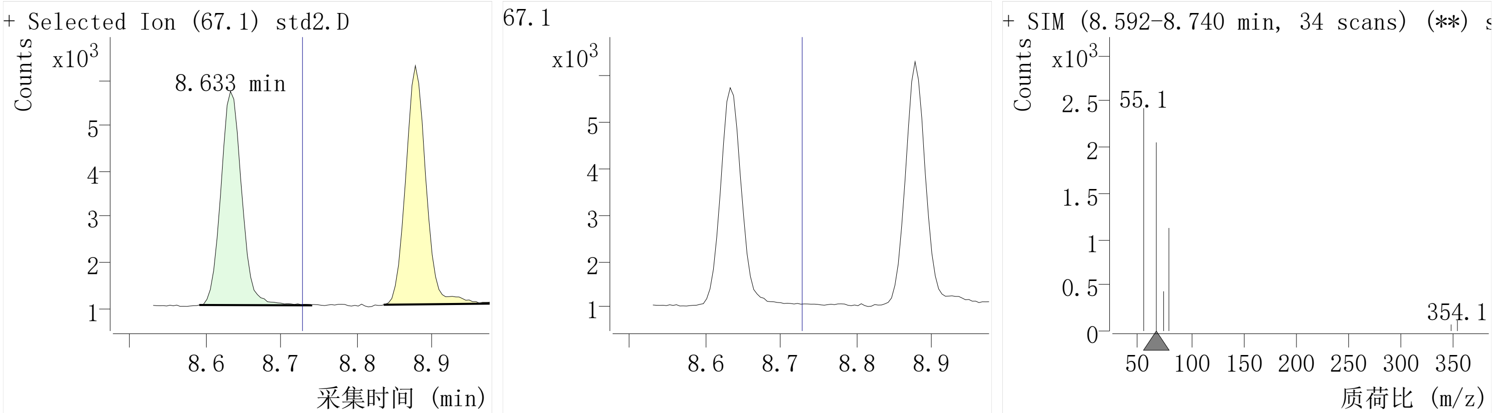

C18:2n6c

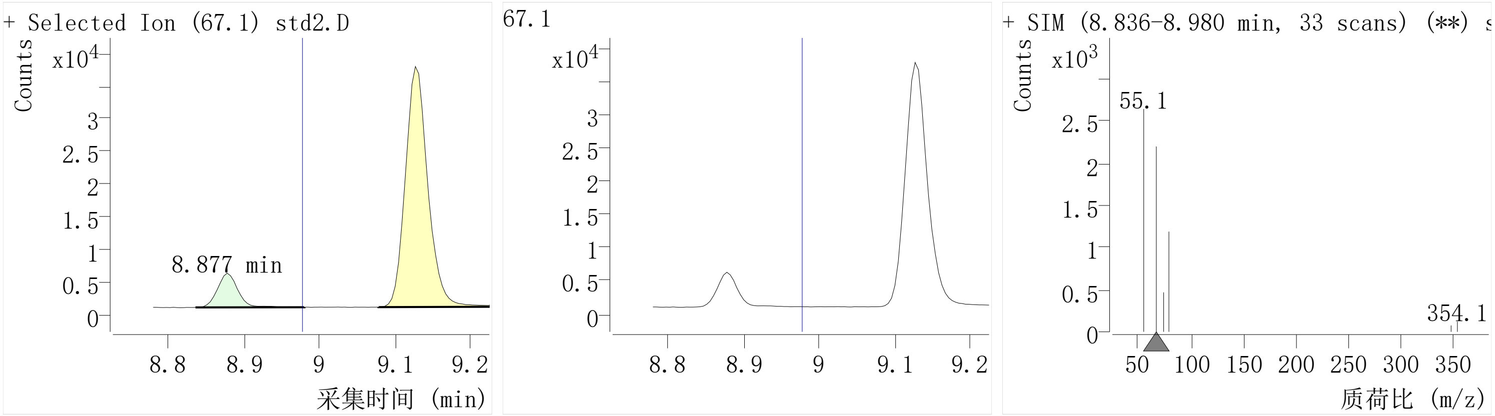

C18:3n6

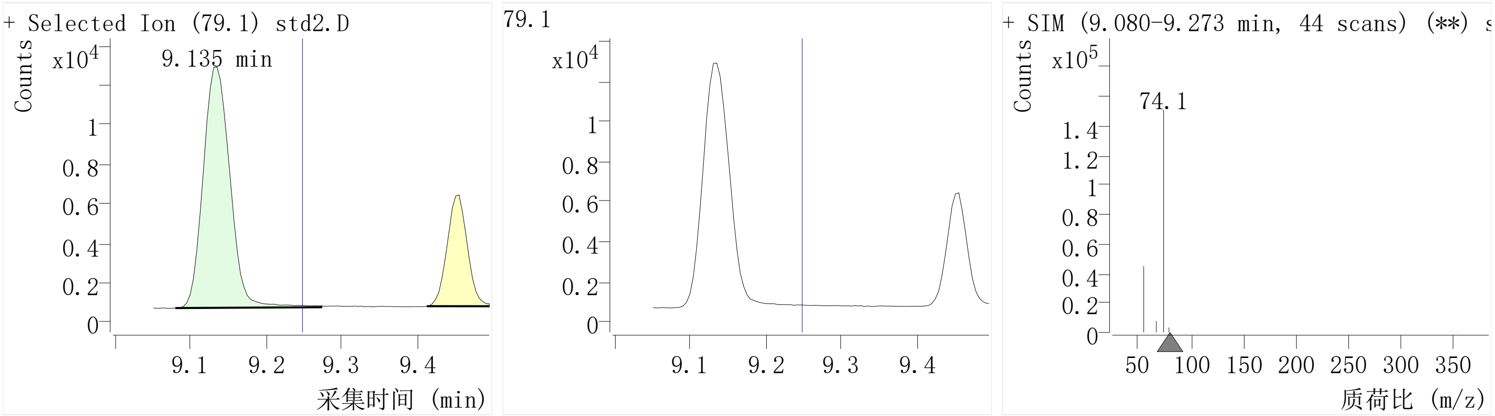

C18:3n3

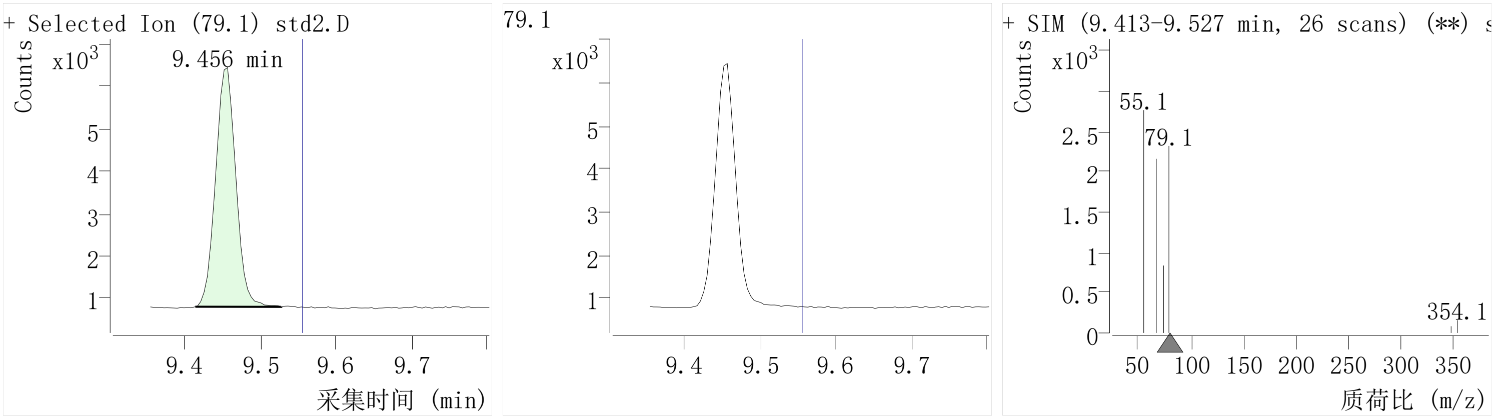

C20:0

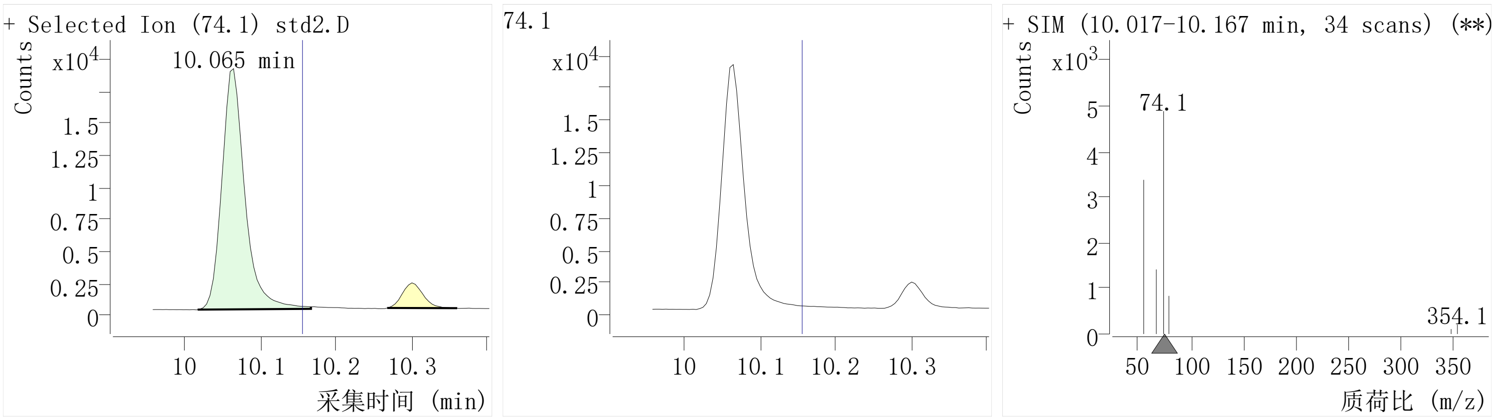

C20:1

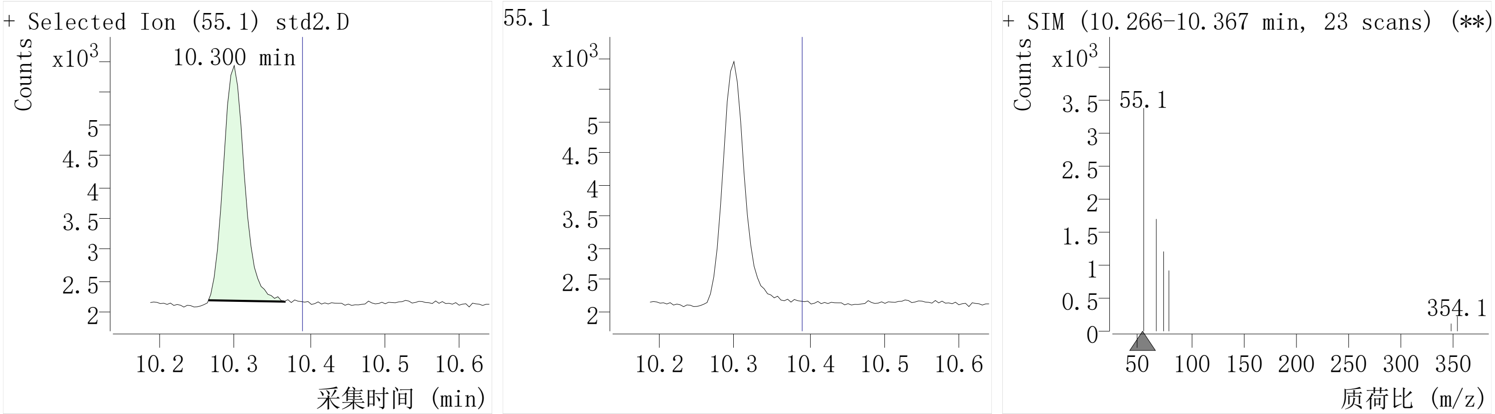

C20:2

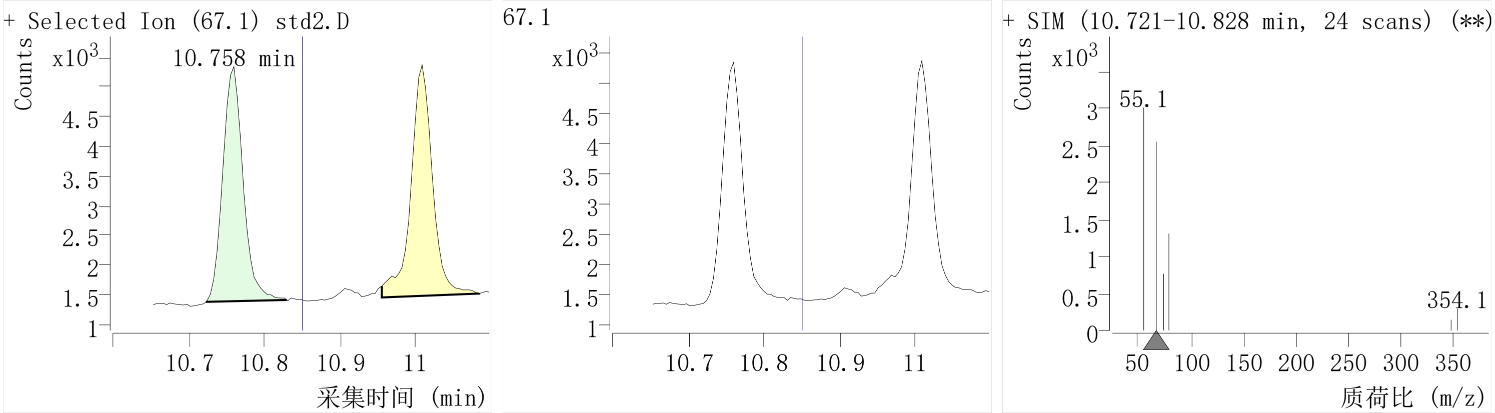

C21:0

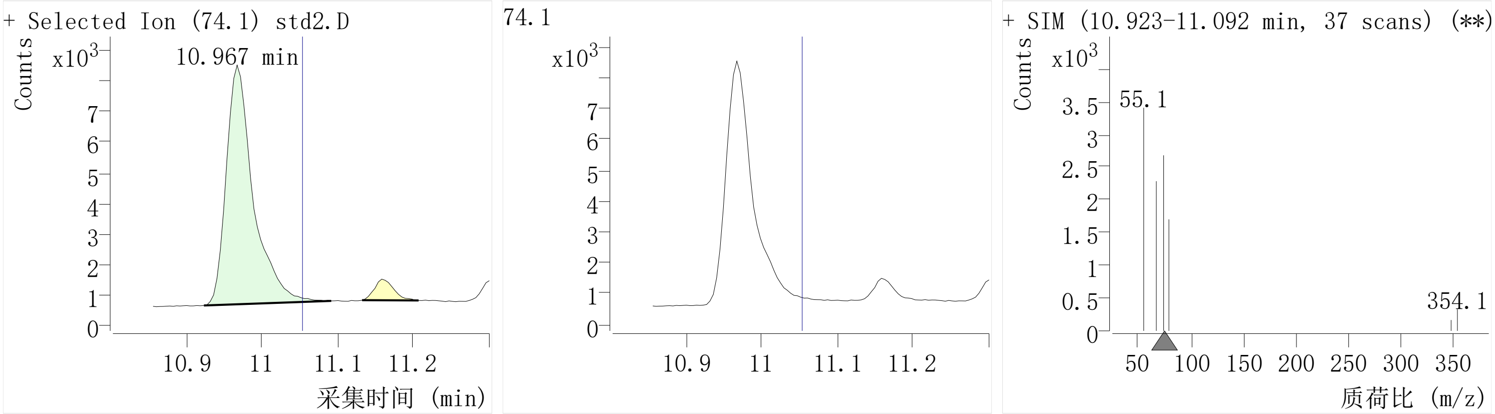

C20:3n6

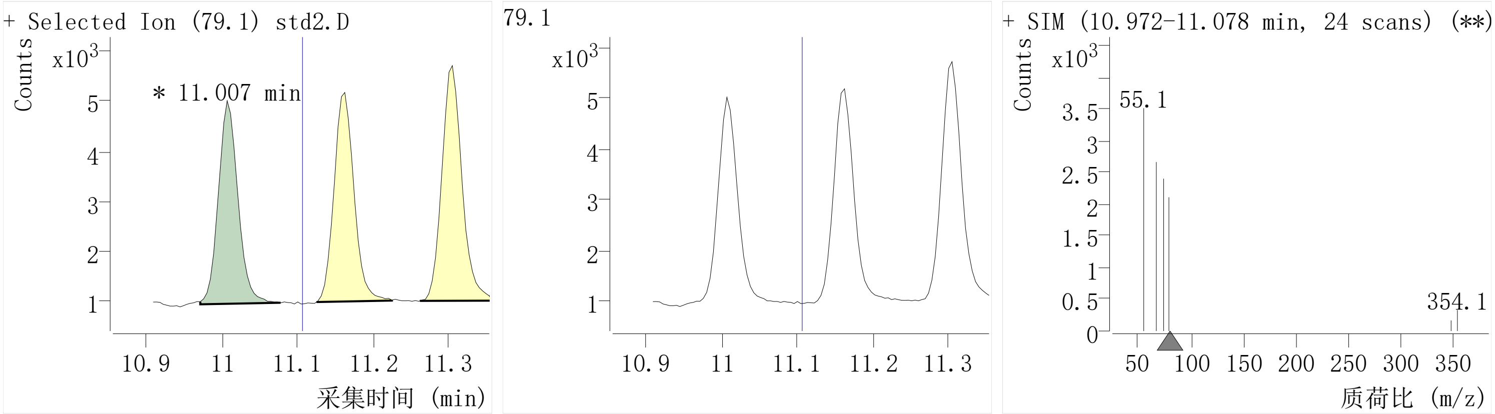

C20:4n6

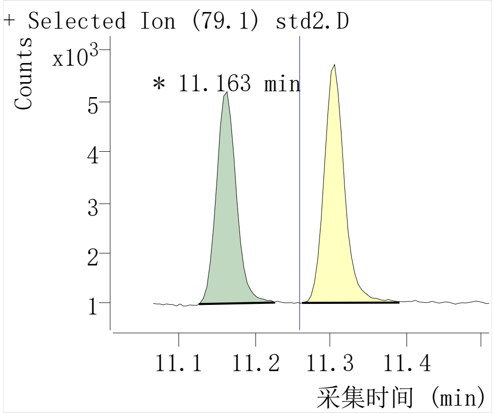

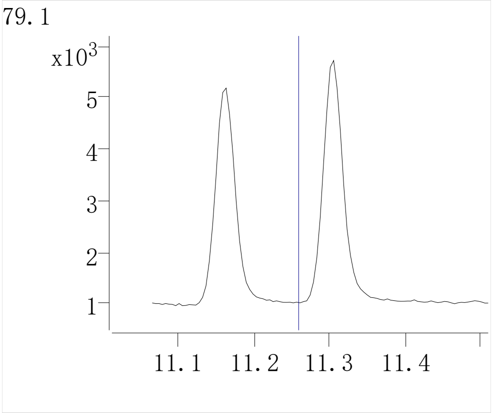

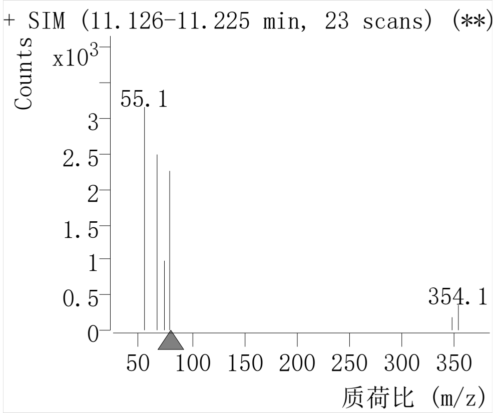

C20:3n3

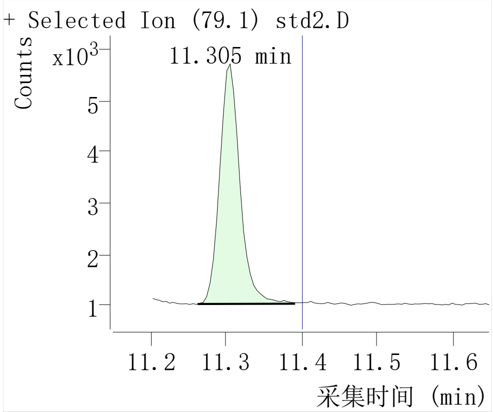

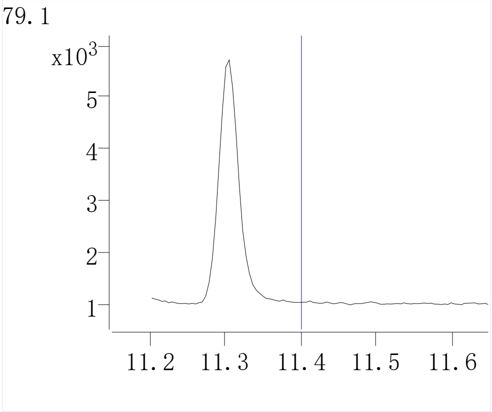

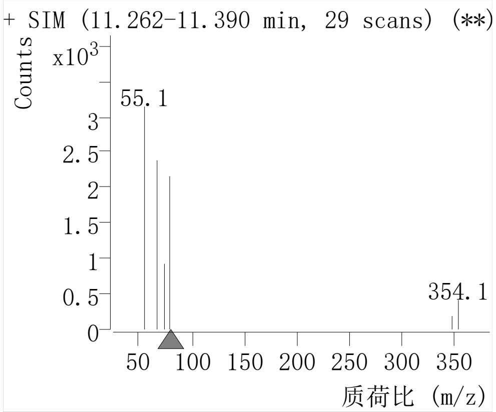

C20:5n3

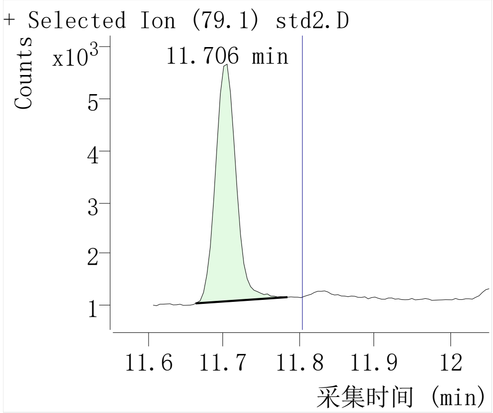

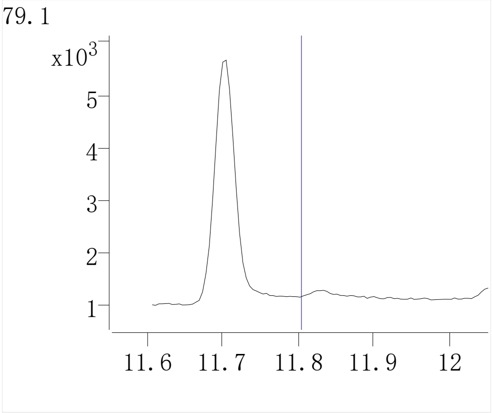

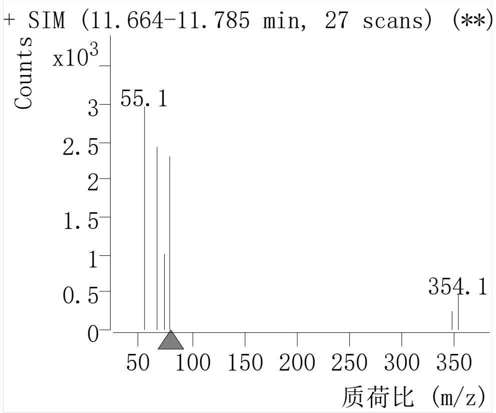

C22:0

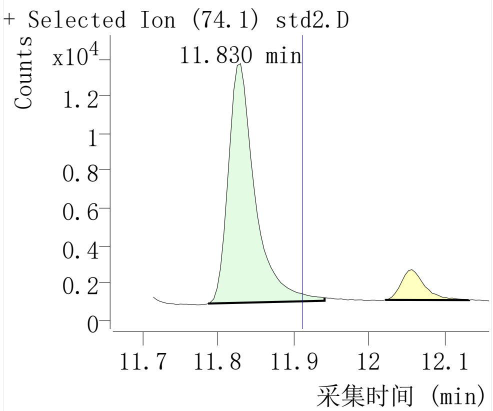

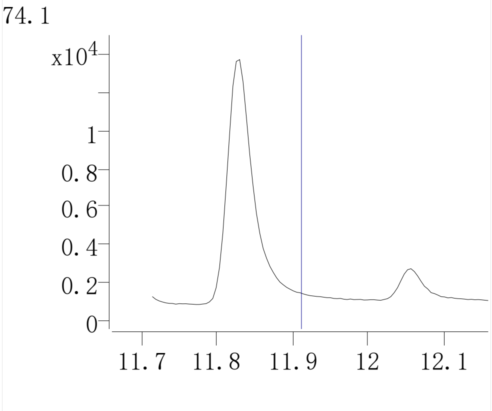

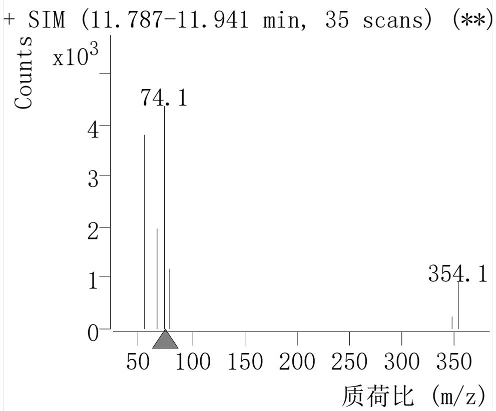

C22:1n9

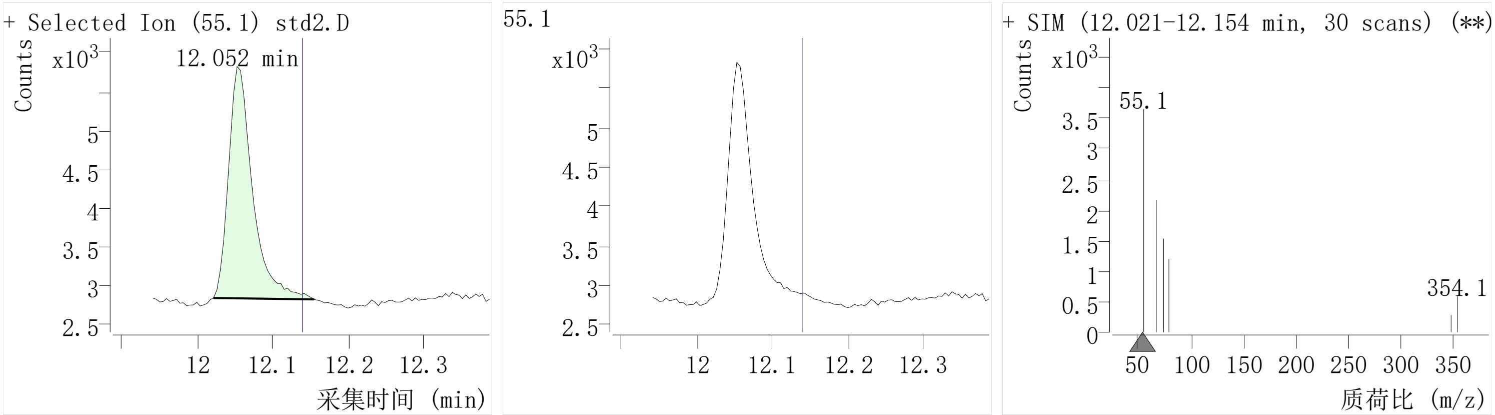

C22:2n6

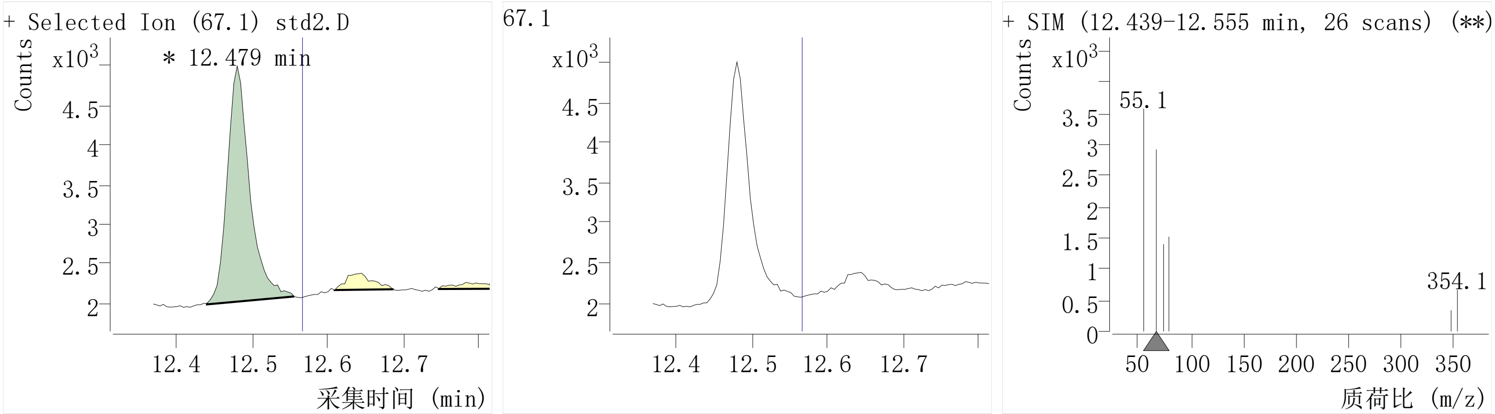

C23:0

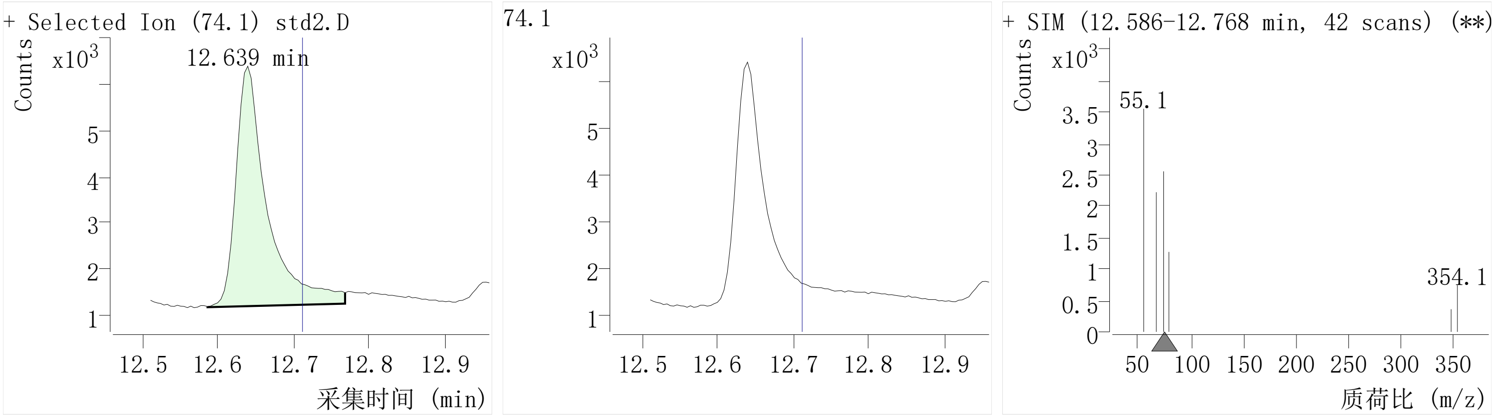

C24:0

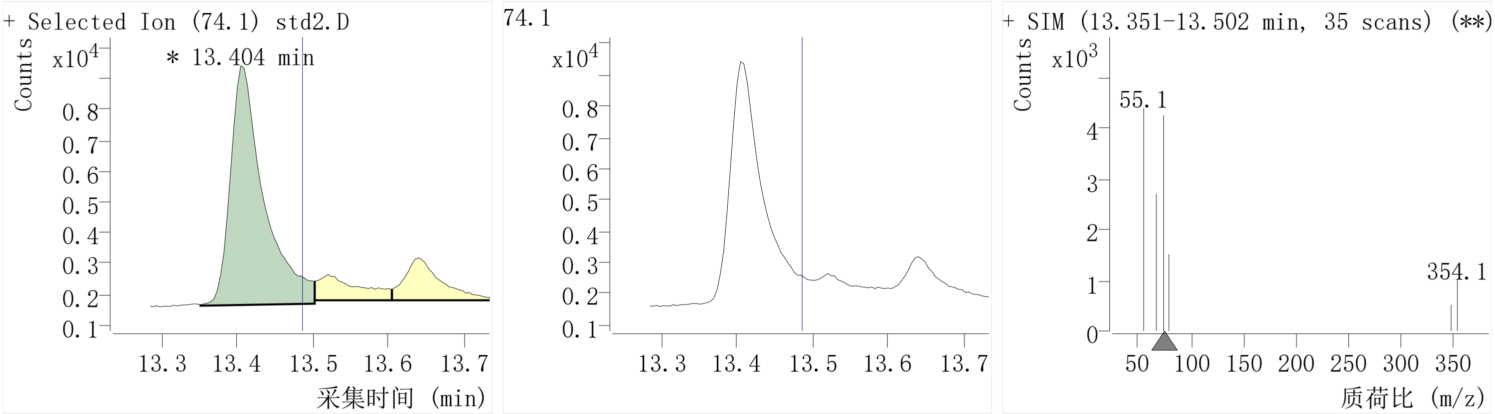

C22:6

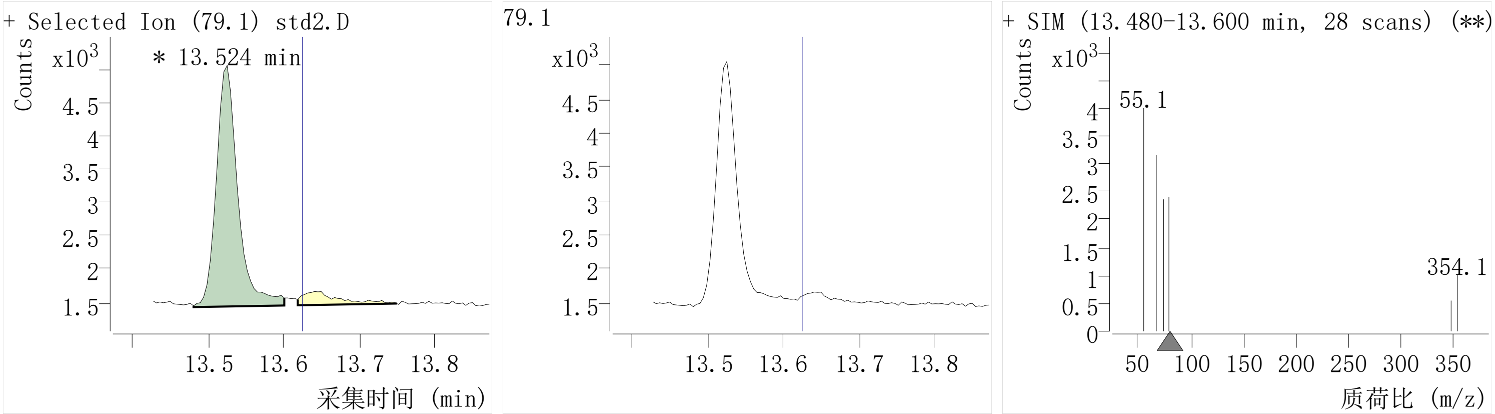
+ Selected Ion (79.1) std2.D
Counts
x10^3
* 13.524 min
4.5
4
3.5
3
2.5
2
1.5
13.5 13.6 13.7 13.8
采集时间 (min)
79.1
+ SIM (13.480-13.600 min, 28 scans) (**)
55.1
354.1
0.5
0
50 100 150 200 250 300 350
质荷比 (m/z)

C24:1

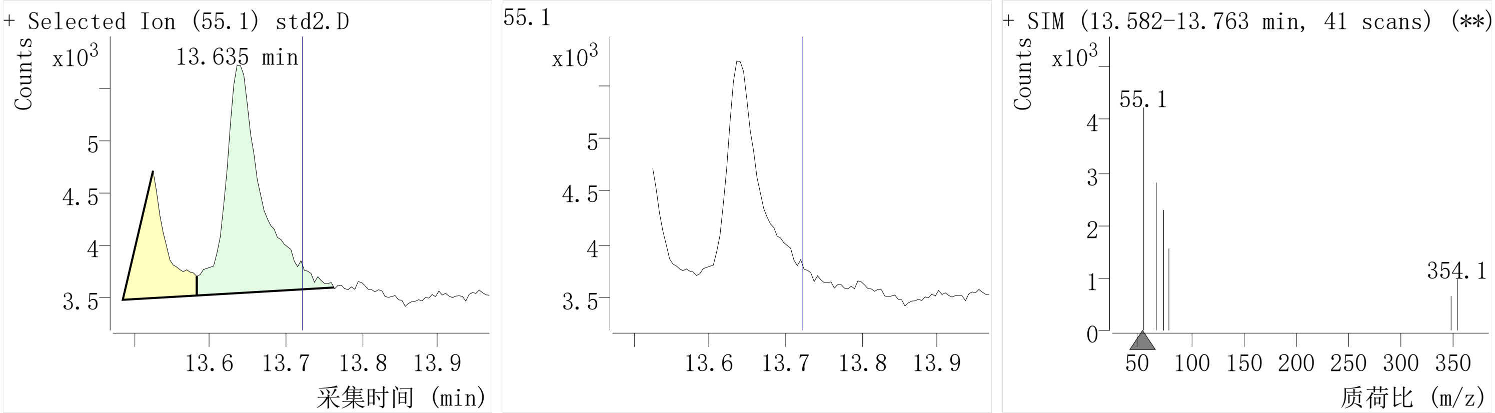
+ Selected Ion (55.1) std2.D
Counts
x10^3
13.635 min
5
4.5
4
3.5
13.6 13.7 13.8 13.9
采集时间 (min)
55.1
+ SIM (13.582-13.763 min, 41 scans) (**)
55.1
354.1
3
2
1
0
50 100 150 200 250 300 350
质荷比 (m/z)

定量分析完成报告

**批处理路径**	G:\GC-MS\HX250430-4-GCMS总脂肪酸靶向检测\HX250430-4\QuantResults\HX250430-4.batch.bin		
**分析时间**	2025/5/14 16:58	**分析员姓名**	DESKTOP-M3A0GPO\omics
**报告时间**	2025/5/16 14:53:02	**报告员姓名**	DESKTOP-M3A0GPO\omics
**最近校正更新**	2025/5/14 16:58	**批处理状态**	已处理
**定量批处理版本**	10.2	**定量报告版本**	10.2

**采集时间**	2025/5/8 18:09	**数据文件**	std3.D
**样品类型**	校正	**样品名称**	std3
**稀释**	1	**采集方法**	脂肪酸

样品色谱图

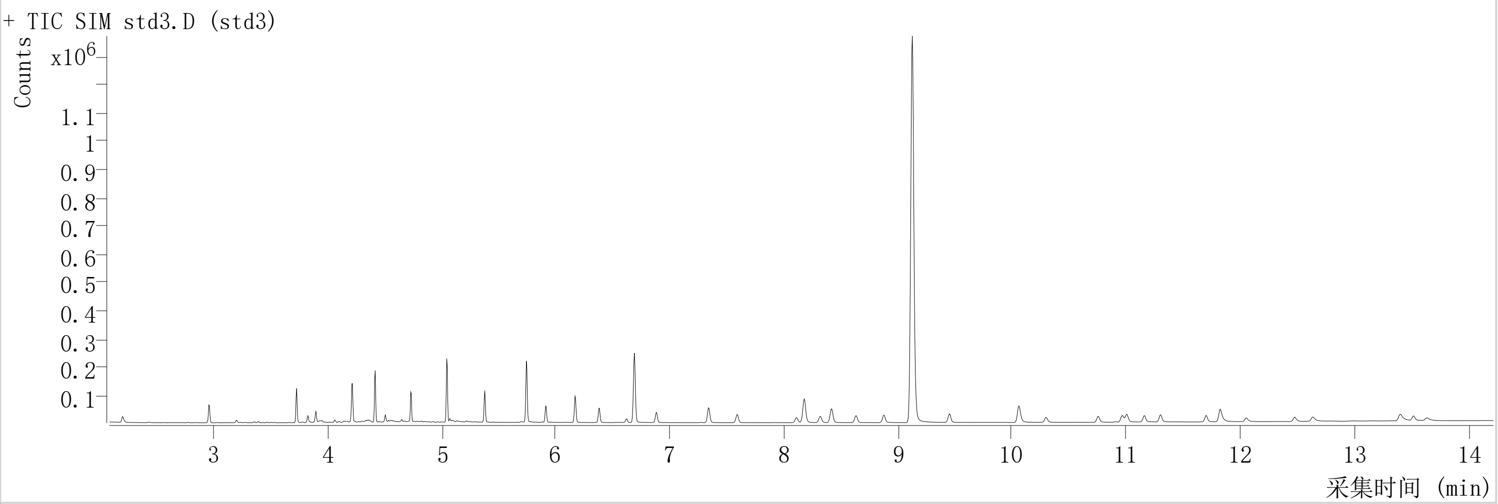

化合物	ISTD	RT	响应	ISTD 响应	响应比	最终浓度	单位
C4:0	C19:0	2.203	23261	1945904	0.0120	1.0705	ug/ml
C6:0	C19:0	2.959	47699	1945904	0.0245	1.0756	ug/ml
C8:0	C19:0	3.728	73304	1945904	0.0377	0.9105	ug/ml
C10:0	C19:0	4.417	107795	1945904	0.0554	1.1451	ug/ml
C11:0	C19:0	4.729	62803	1945904	0.0323	0.5859	ug/ml
C12:0	C19:0	5.044	137700	1945904	0.0708	1.1603	ug/ml
C13:0	C19:0	5.378	71662	1945904	0.0368	0.5884	ug/ml
C14:0	C19:0	5.742	152965	1945904	0.0786	1.4463	ug/ml
C14:1	C19:0	5.911	28748	1945904	0.0148	0.6384	ug/ml
C15:0	C19:0	6.169	73702	1945904	0.0379	0.5921	ug/ml
C15:1	C19:0	6.378	31509	1945904	0.0162	0.4641	ug/ml
C16:0	C19:0	6.690	226913	1945904	0.1166	0.8724	ug/ml
C16:1	C19:0	6.881	24334	1945904	0.0125	0.6759	ug/ml
C17:0	C19:0	7.339	63123	1945904	0.0324	0.6768	ug/ml
C17:1	C19:0	7.592	23940	1945904	0.0123	0.6337	ug/ml
C18:0	C19:0	8.179	118731	1945904	0.0610	1.3004	ug/ml
C18:1n9t	C19:0	8.317	19084	1945904	0.0098	0.5785	ug/ml
C18:1n9c	C19:0	8.419	43086	1945904	0.0221	1.0674	ug/ml
C18:2n6t	C19:0	8.633	20734	1945904	0.0107	0.6028	ug/ml
C18:2n6c	C19:0	8.877	22033	1945904	0.0113	0.6887	ug/ml
C18:3n6	C19:0	9.140	45753	1945904	0.0235	0.6652	ug/ml
C18:3n3	C19:0	9.451	24443	1945904	0.0126	0.6182	ug/ml
C20:0	C19:0	10.060	90943	1945904	0.0467	1.2129	ug/ml
C20:1	C19:0	10.296	17812	1945904	0.0092	0.6040	ug/ml
C20:2	C19:0	10.754	17325	1945904	0.0089	0.5989	ug/ml
C21:0	C19:0	10.967	41873	1945904	0.0215	0.5922	ug/ml
C20:3n6	C19:0	11.007	17167	1945904	0.0088	0.6014	ug/ml
C20:4n6	C19:0	11.163	17658	1945904	0.0091	0.6011	ug/ml
C20:3n3	C19:0	11.305	20073	1945904	0.0103	0.5904	ug/ml
C20:5n3	C19:0	11.705	20490	1945904	0.0105	0.6172	ug/ml

定量分析完成报告

化合物	ISTD	RT	响应	ISTD 响应	响应比	最终浓度	单位
C22:0	C19:0	11.825	69692	1945904	0.0358	1.1799	ug/ml
C22:1n9	C19:0	12.052	14742	1945904	0.0076	0.5461	ug/ml
C22:2n6	C19:0	12.479	13309	1945904	0.0068	0.5774	ug/ml
C23:0	C19:0	12.639	30777	1945904	0.0158	0.5905	ug/ml
C24:0	C19:0	13.404	49962	1945904	0.0257	1.1641	ug/ml
C22:6	C19:0	13.520	14842	1945904	0.0076	0.5686	ug/ml
C24:1	C19:0	13.635	12172	1945904	0.0063	0.5465	ug/ml

C4:0

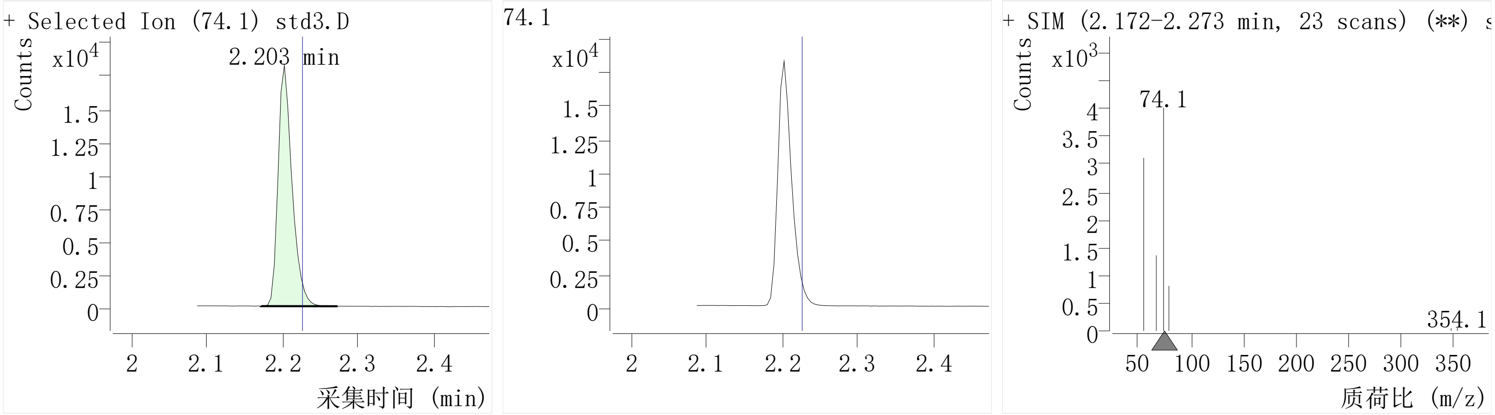

C6:0

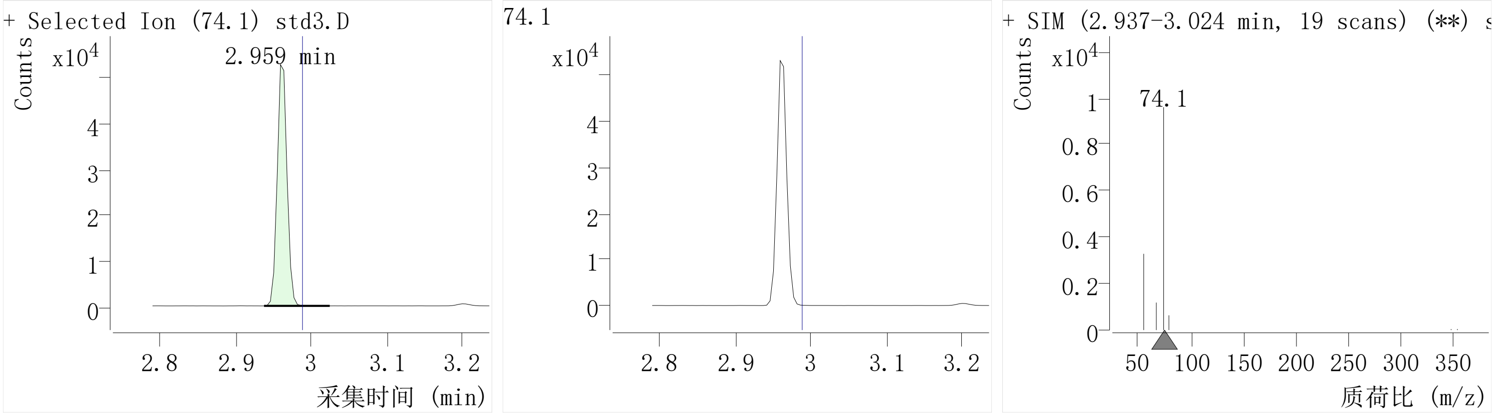

C8:0

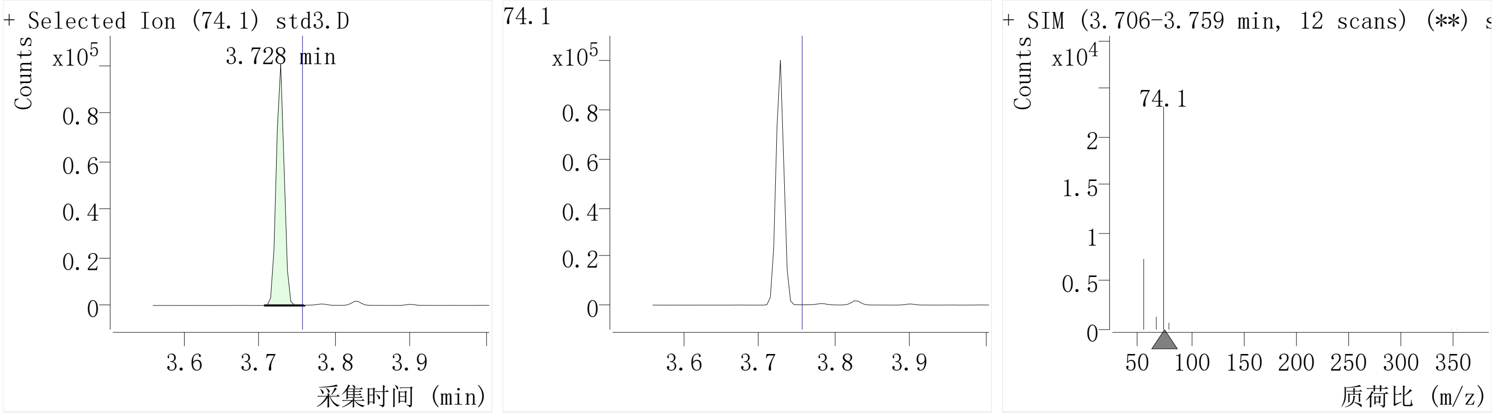

C10:0

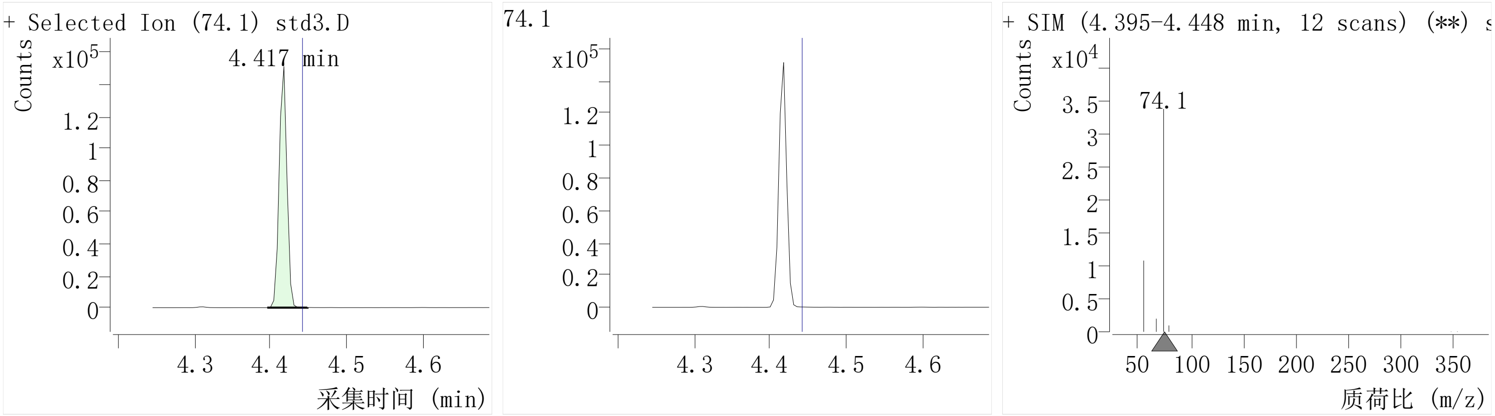

C11:0

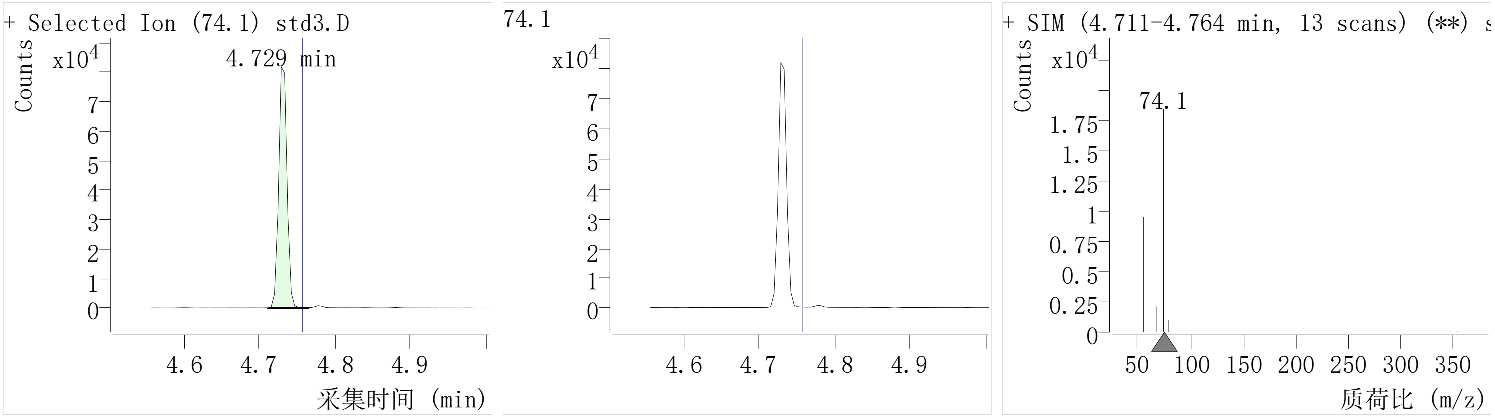

C12:0

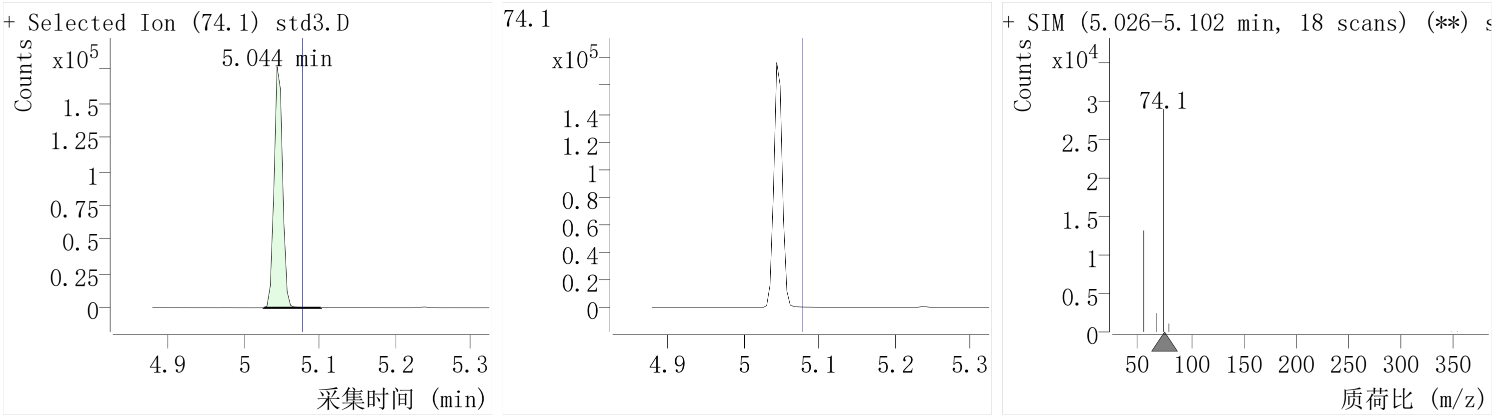

C13:0

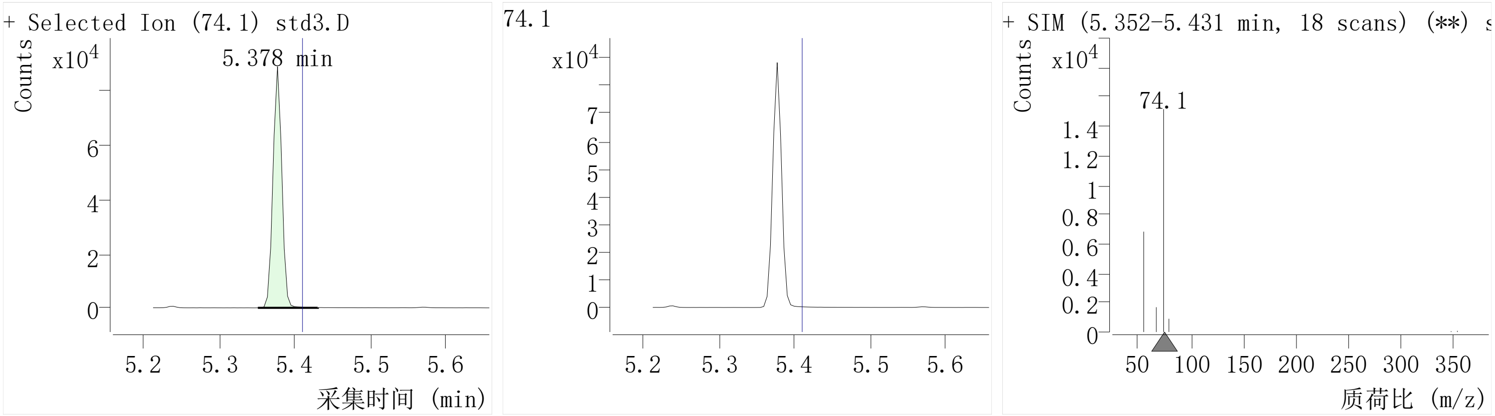

C14:0

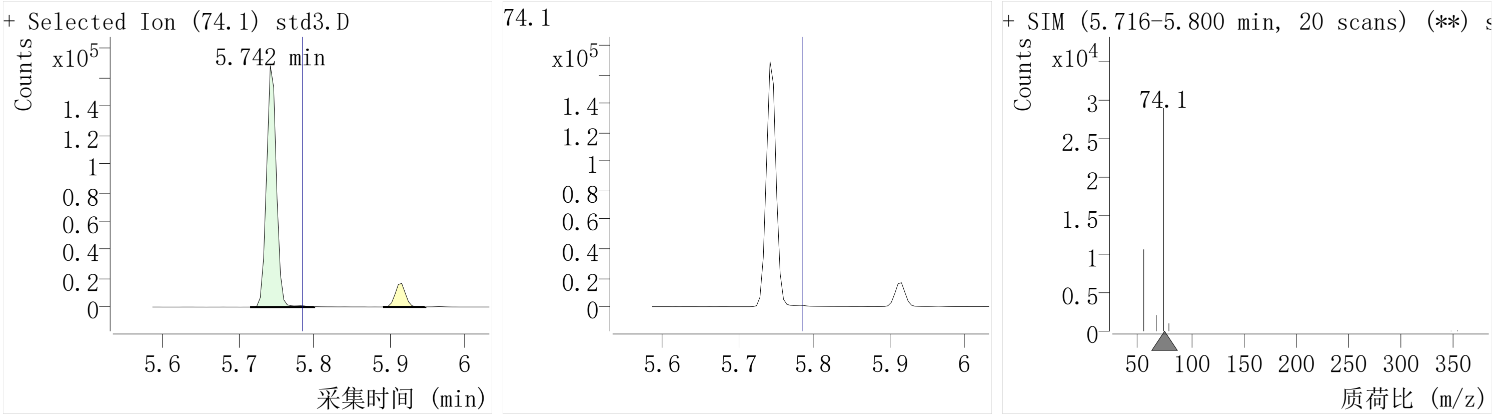

C14:1

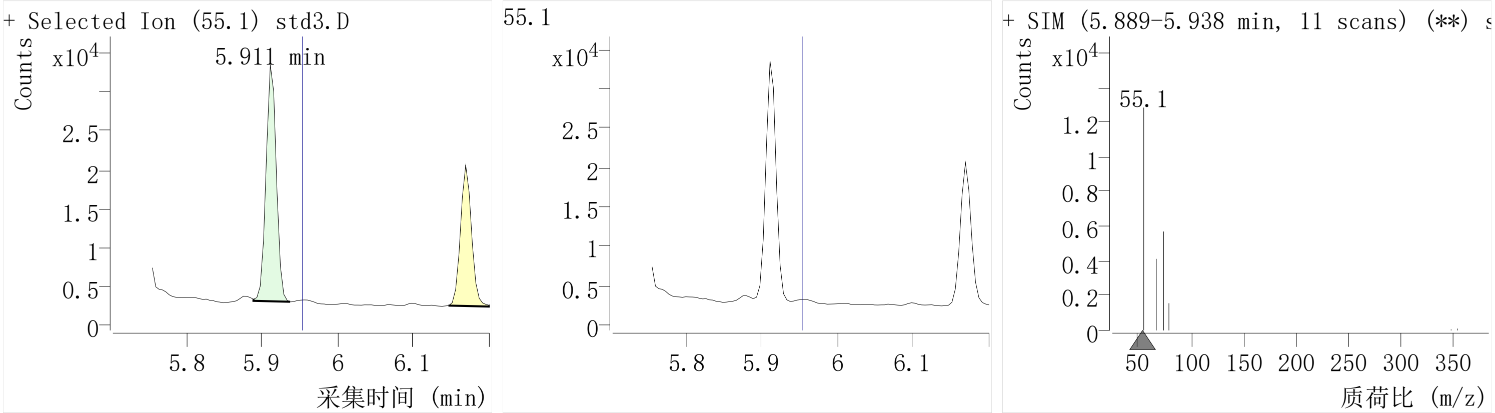

C15:0

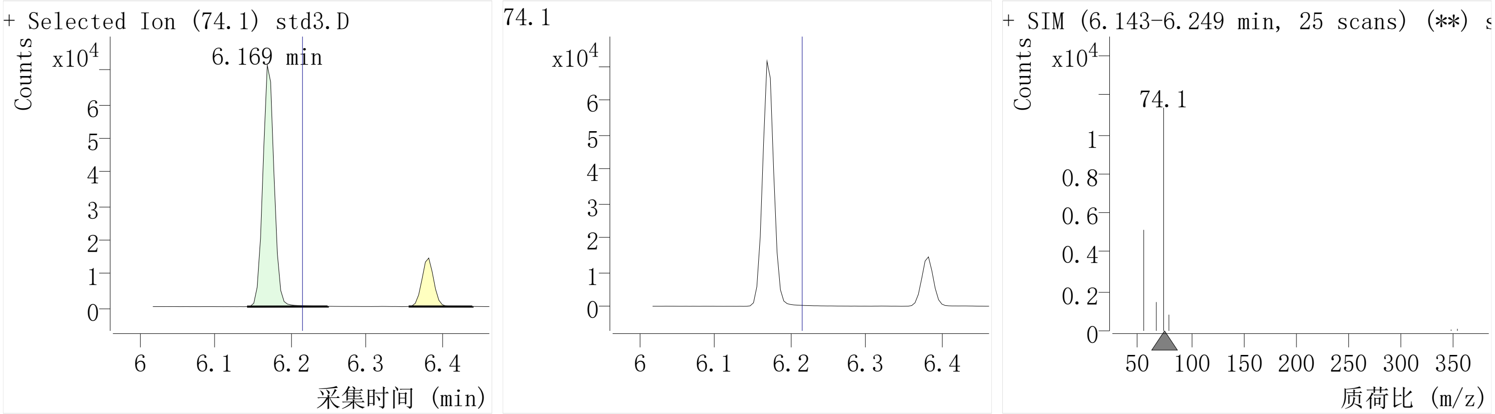

C15:1

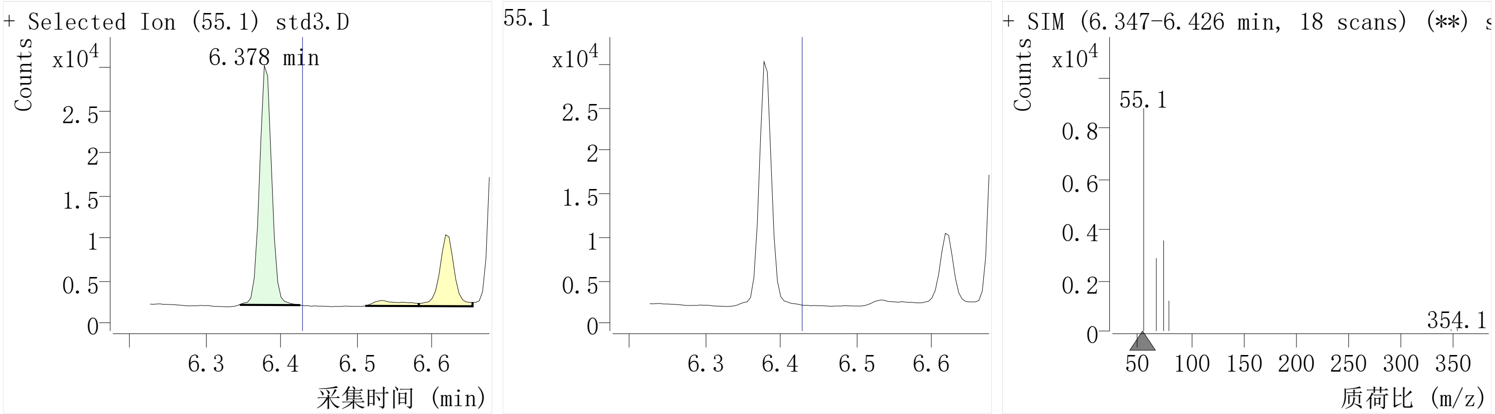

C16:0

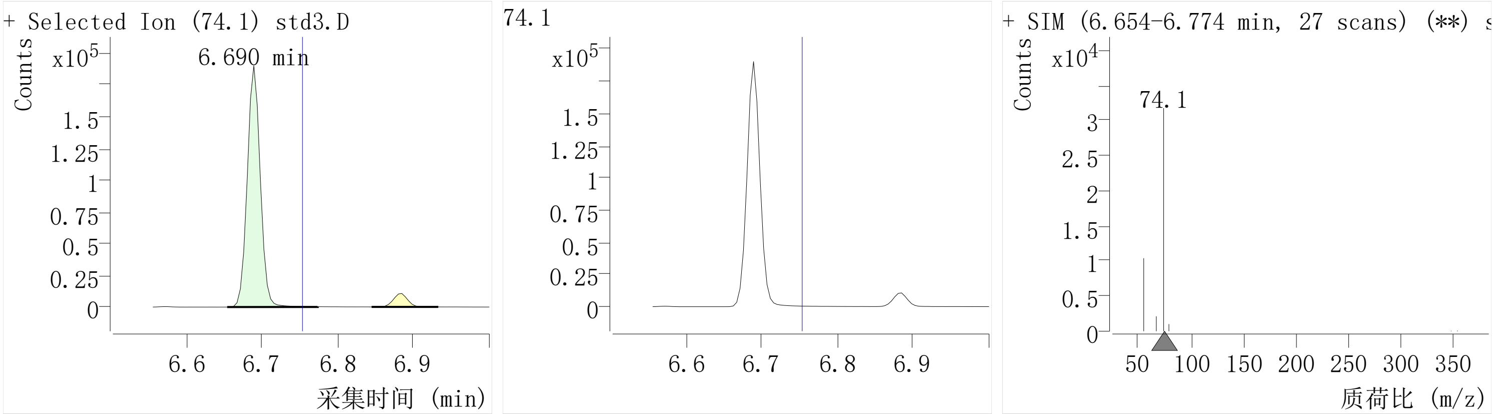

C16:1

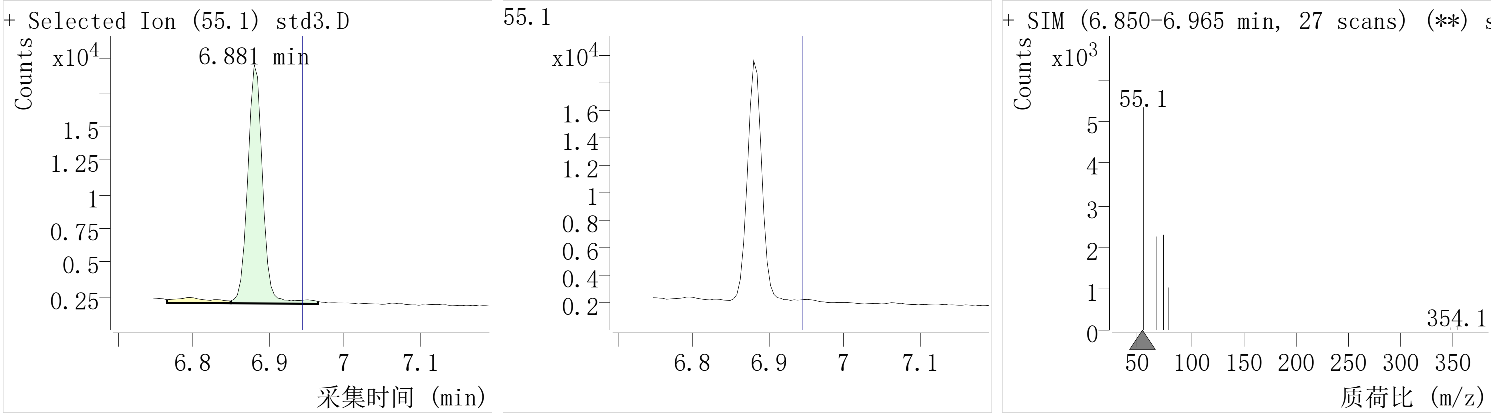

C17:0

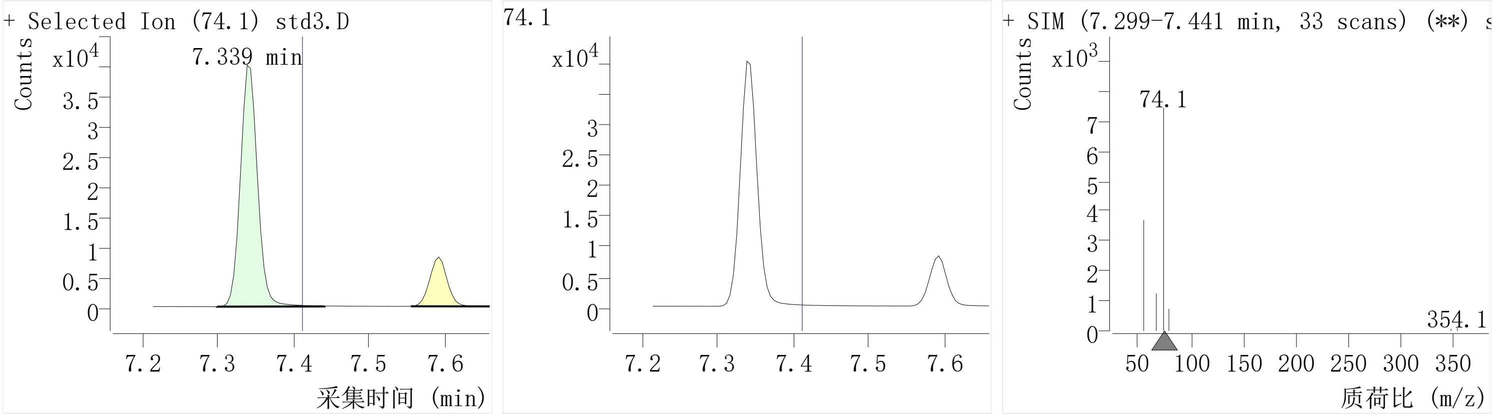

C17:1

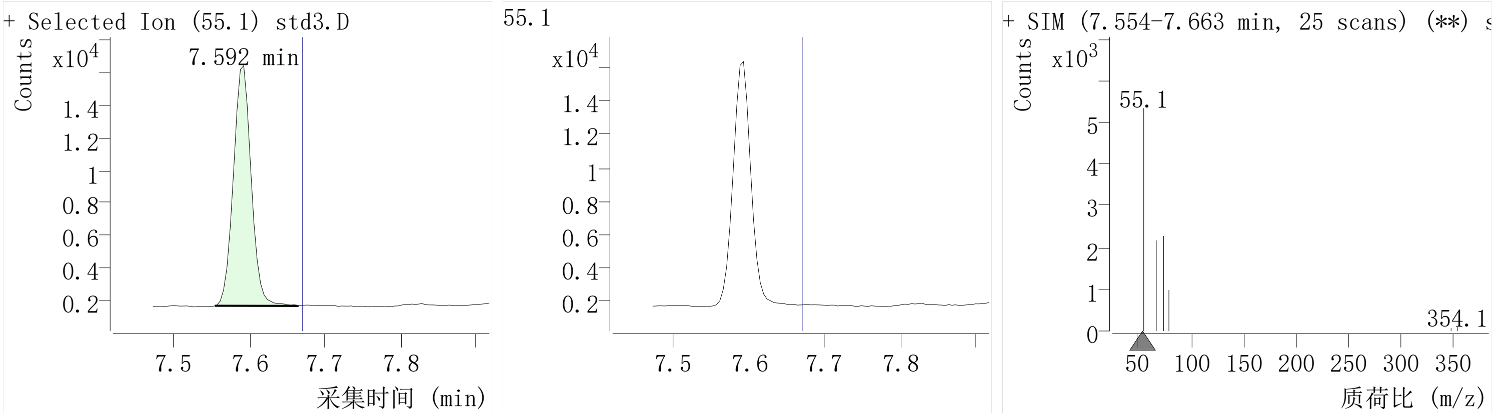

生成时间 2025/5/16 14:52

C18:0

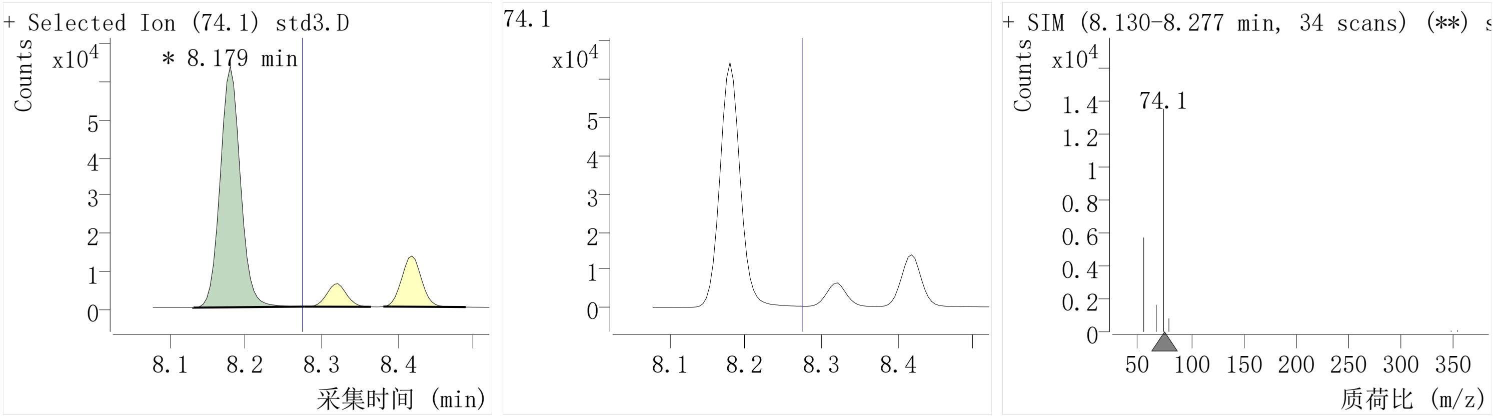

C18:1n9t

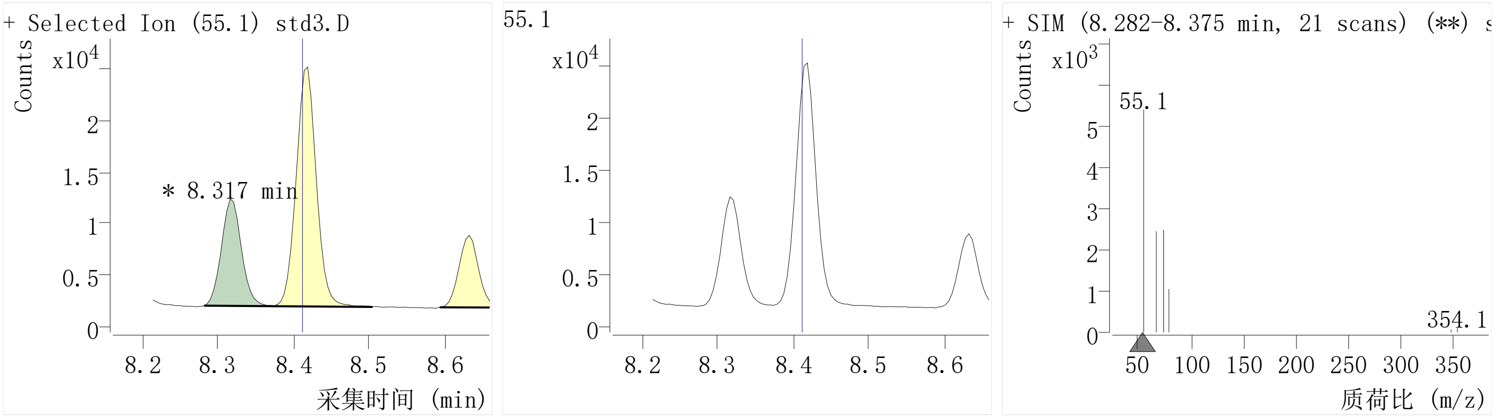

C18:1n9c

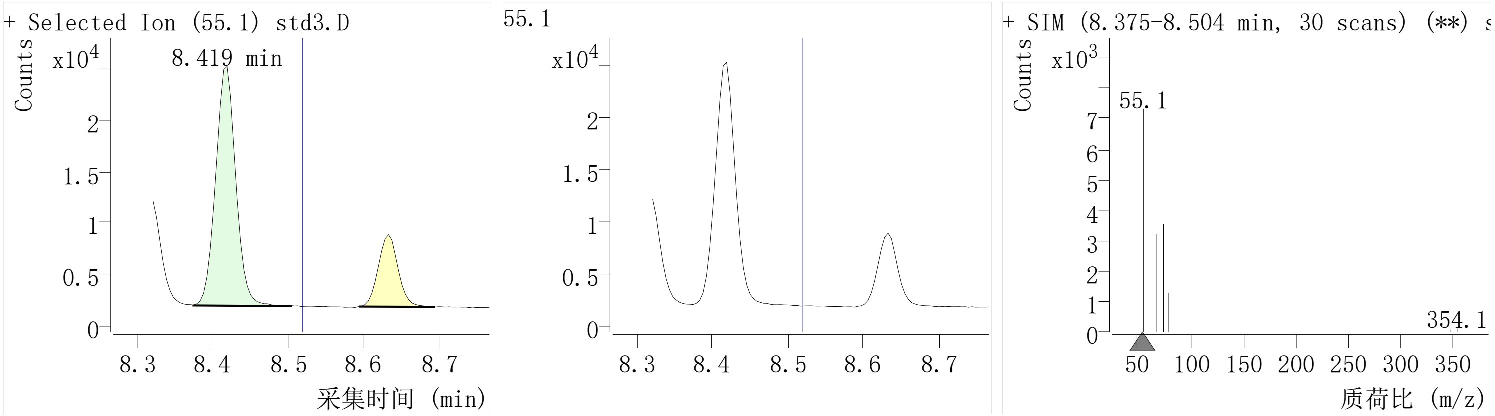

C18:2n6t

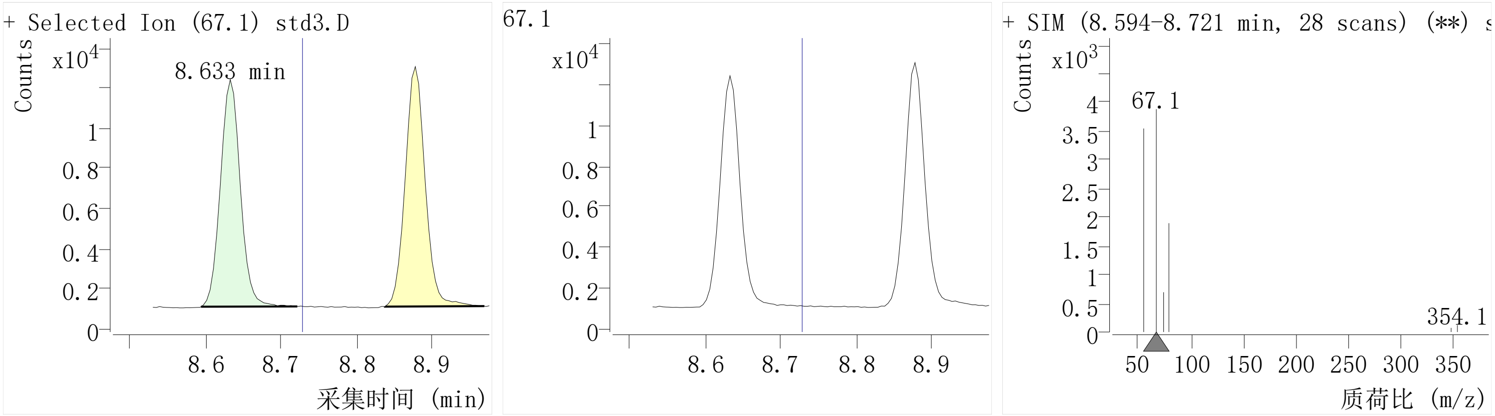

C18:2n6c

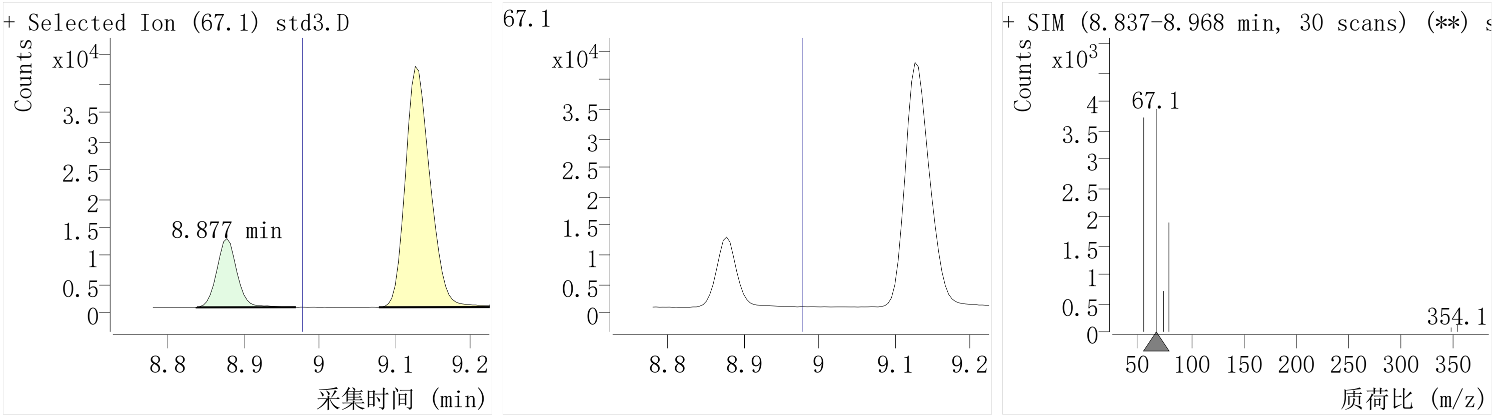

C18:3n6

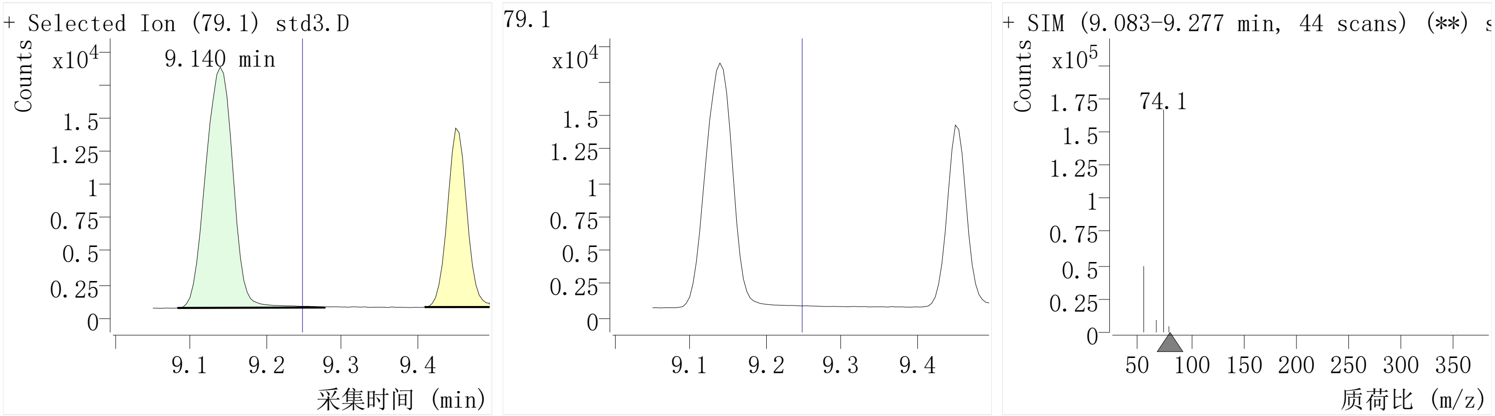

C18:3n3

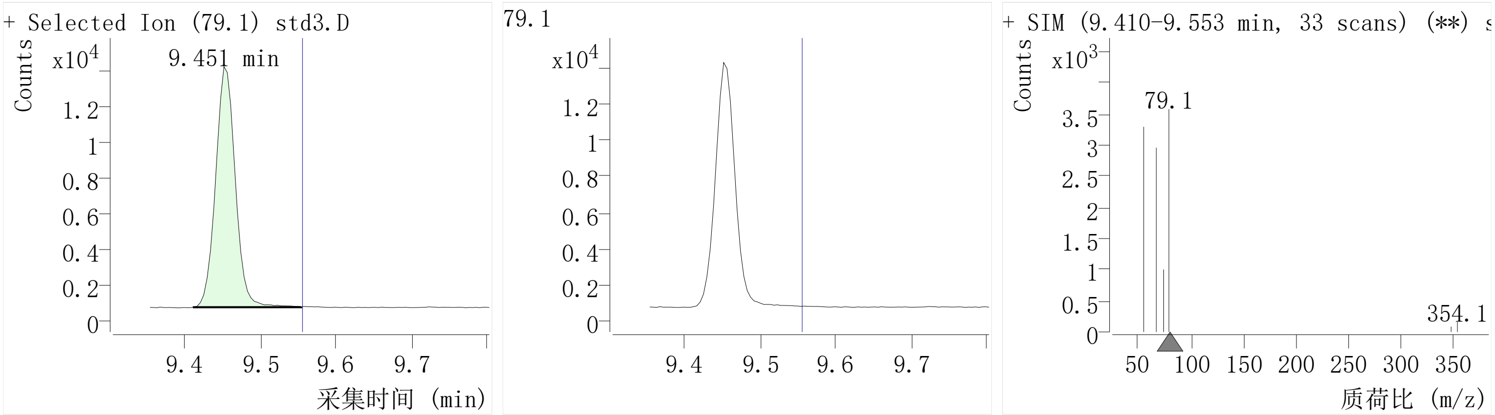

C20:0

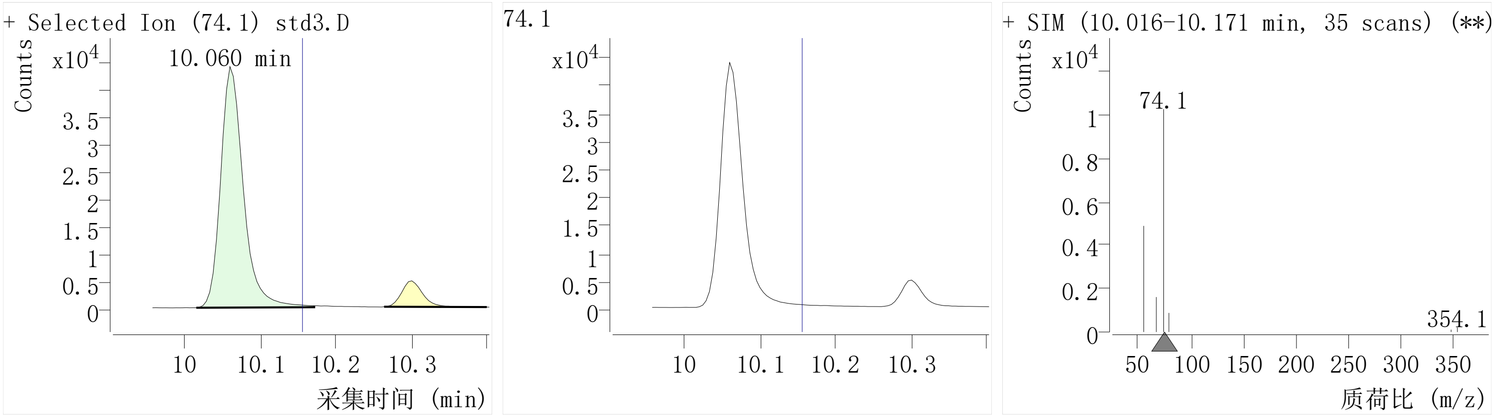

C20:1

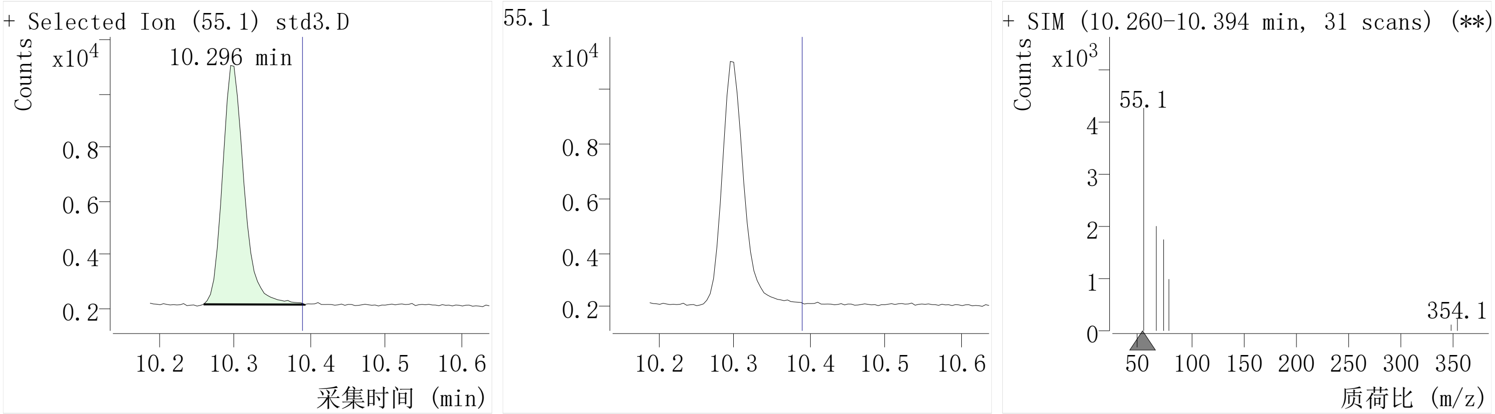

C20:2

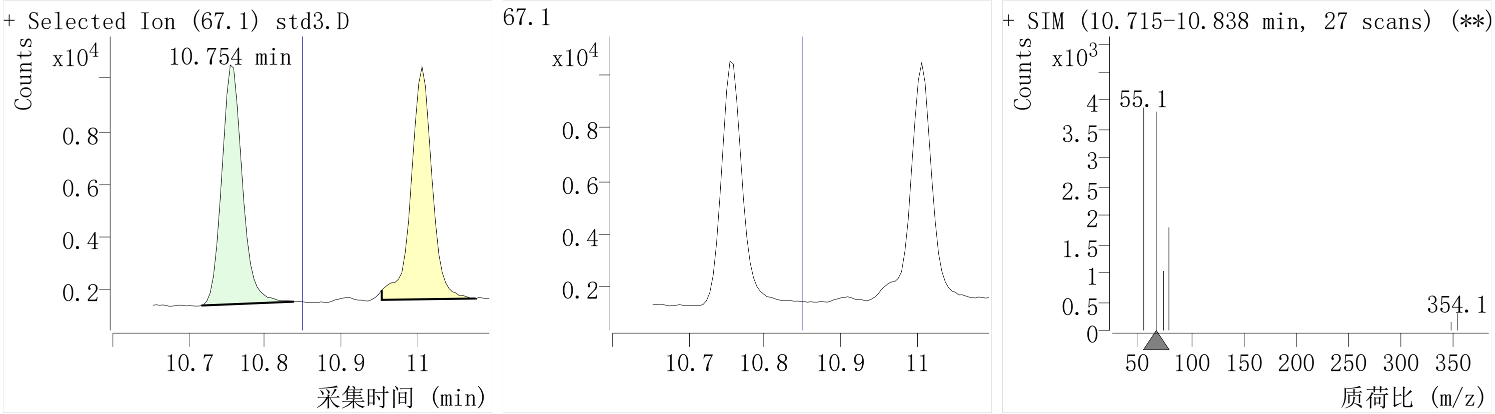

C21:0

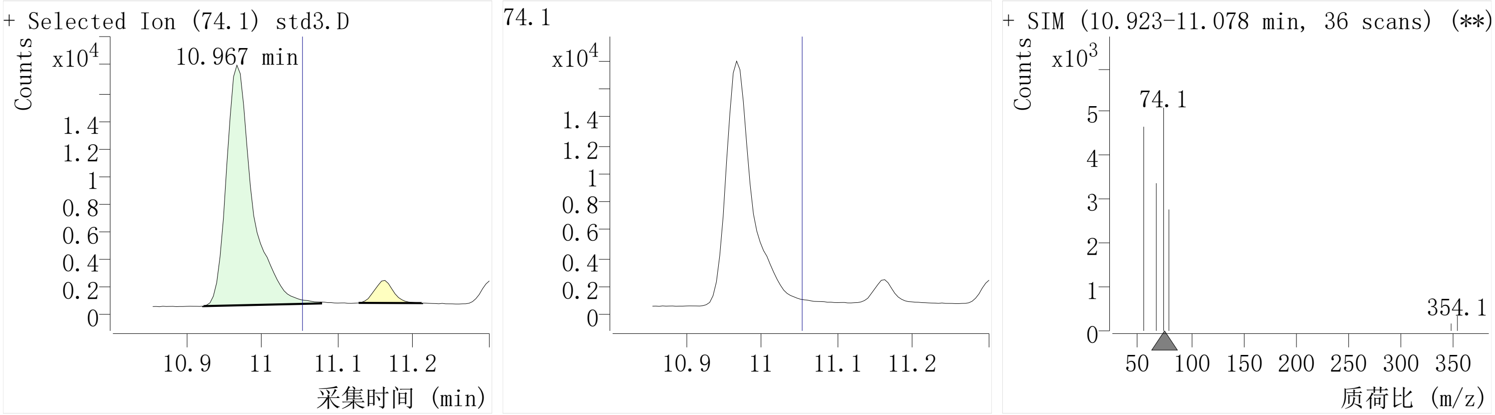

C20:3n6

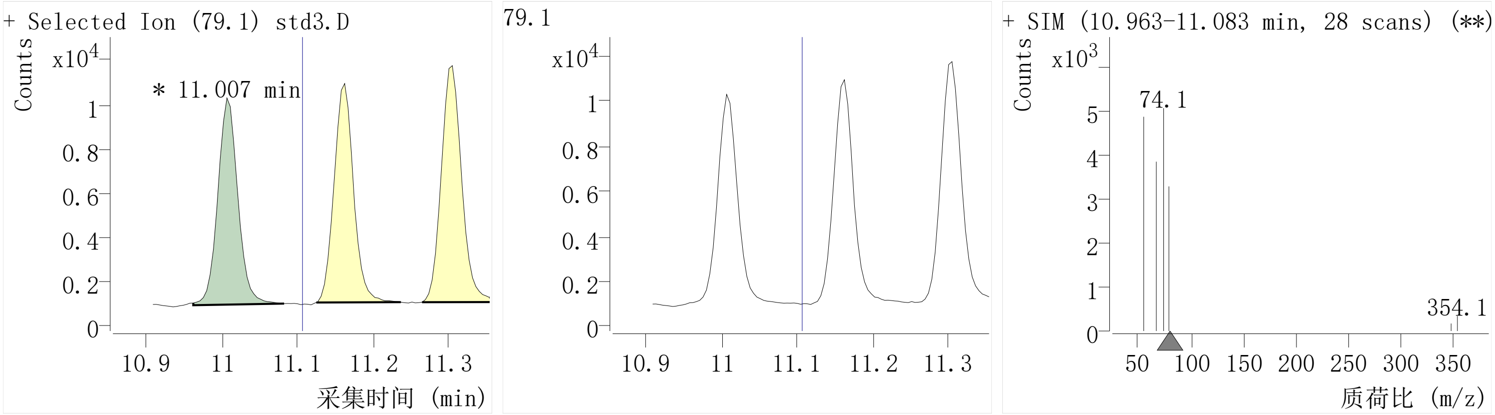

C20:4n6

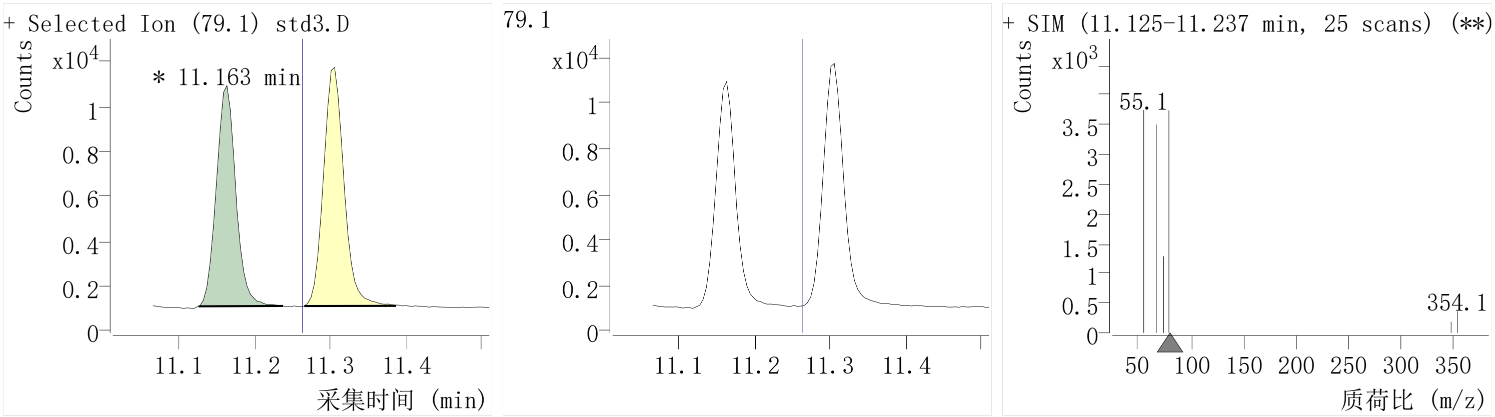

C20:3n3

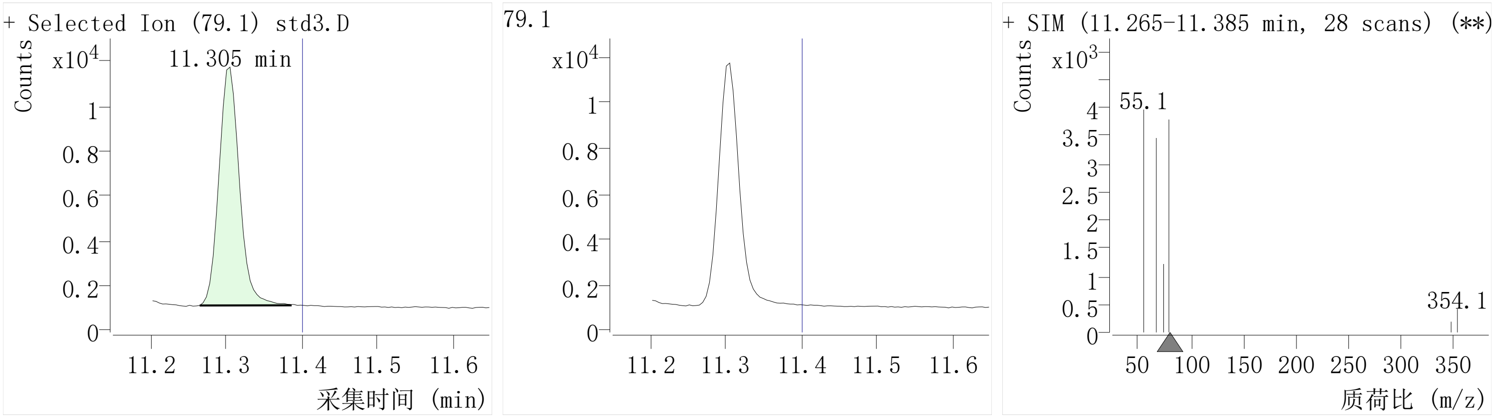

C20:5n3

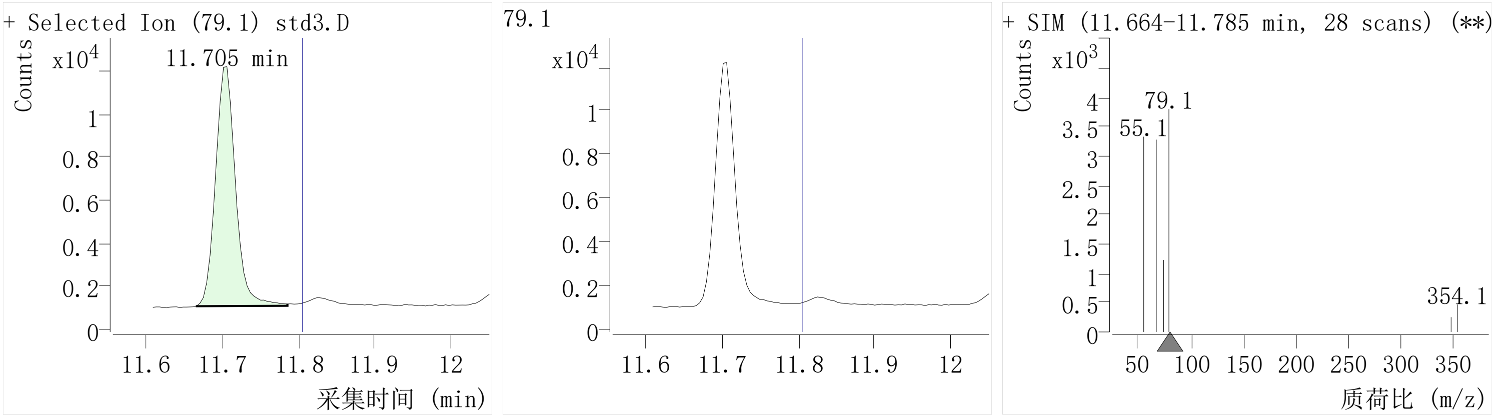

C22:0

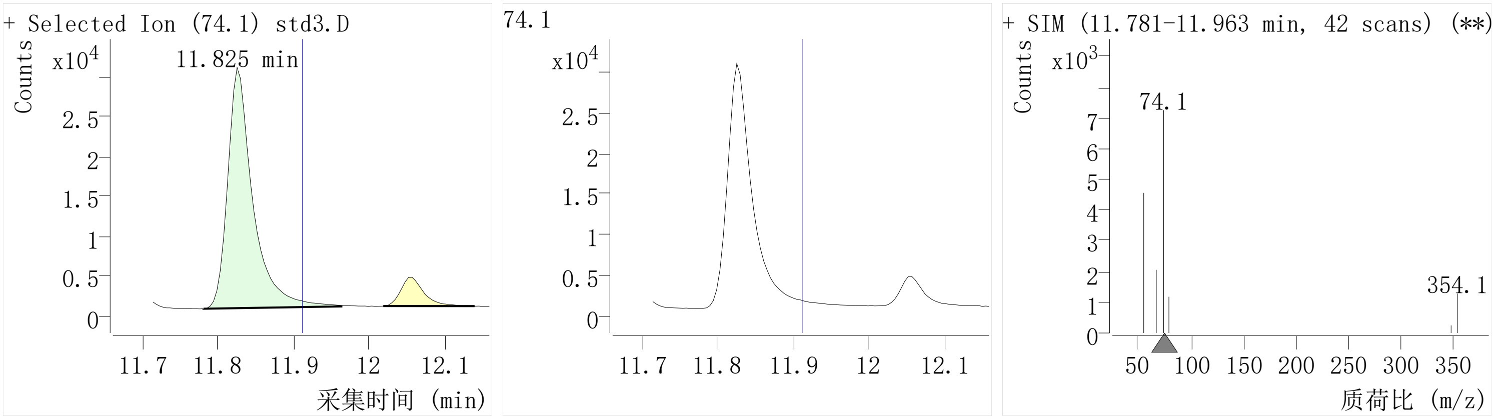

C22:1n9

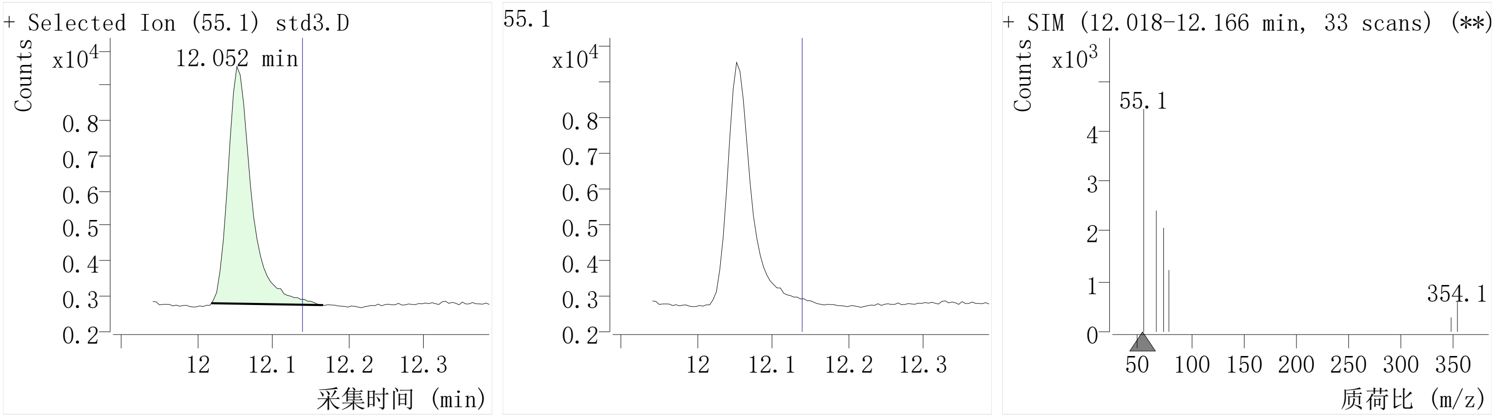

C22:2n6

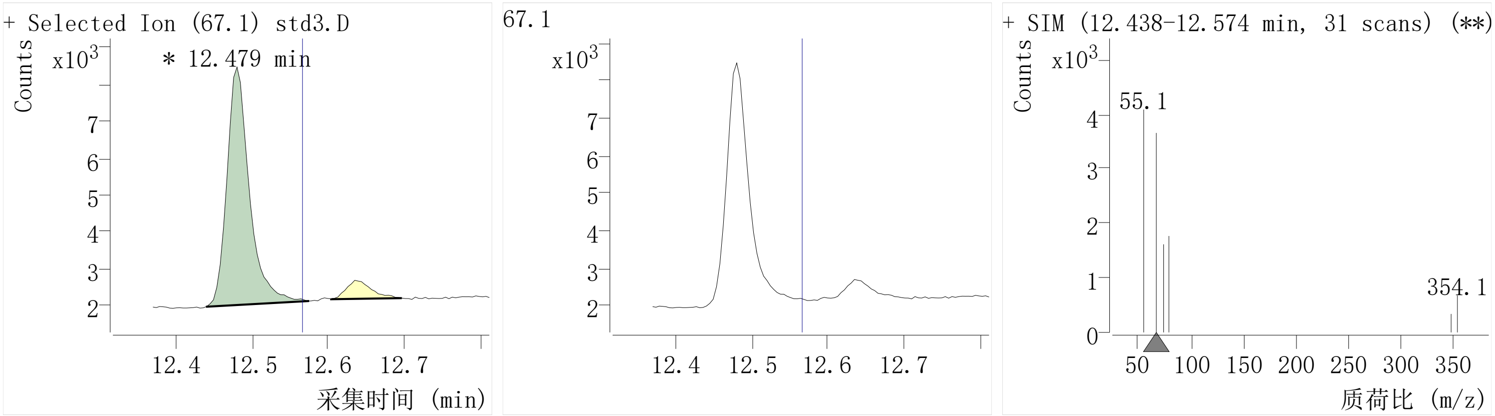

C23:0

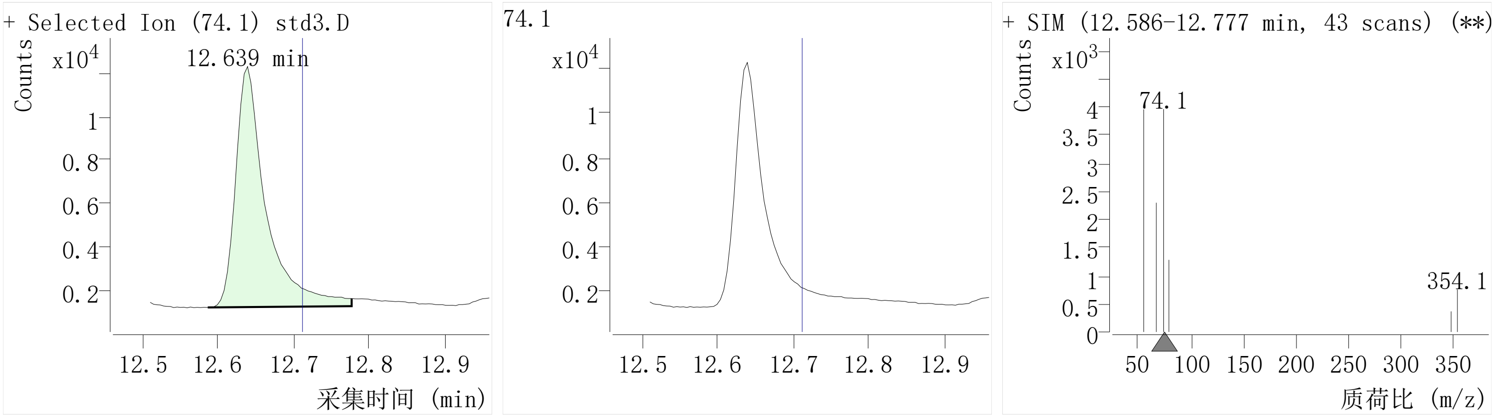

C24:0

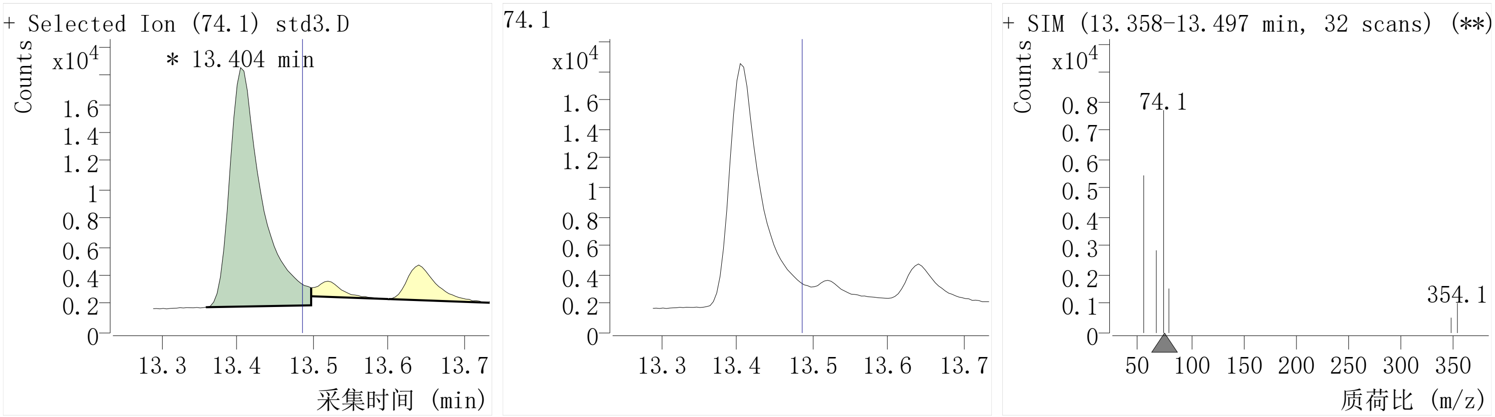

C22:6

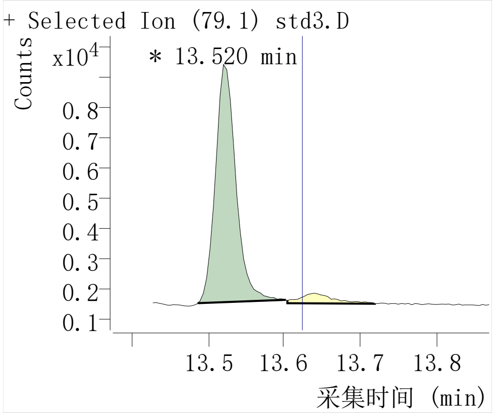
+ Selected Ion (79.1) std3.D
Counts x10^4
* 13.520 min
采集时间 (min)

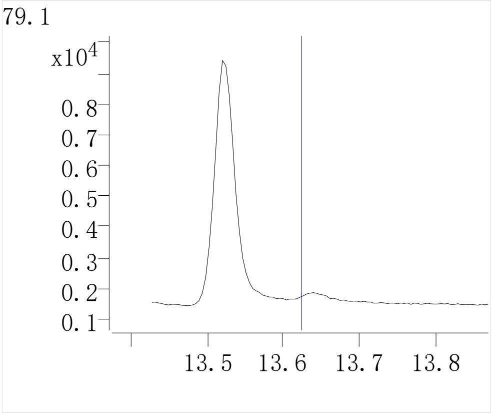
79.1
x10^4

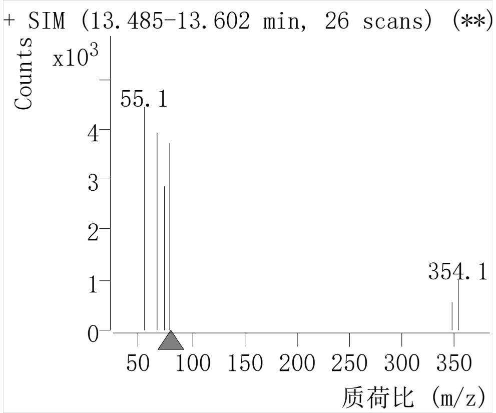
+ SIM (13.485-13.602 min, 26 scans) (**)
Counts x10^3
55.1
354.1
质荷比 (m/z)

C24:1

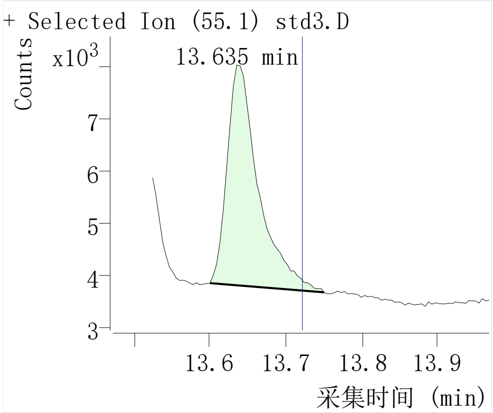
+ Selected Ion (55.1) std3.D
Counts x10^3
13.635 min
采集时间 (min)

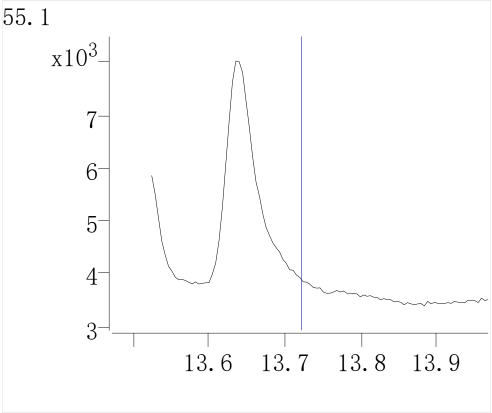
55.1
x10^3

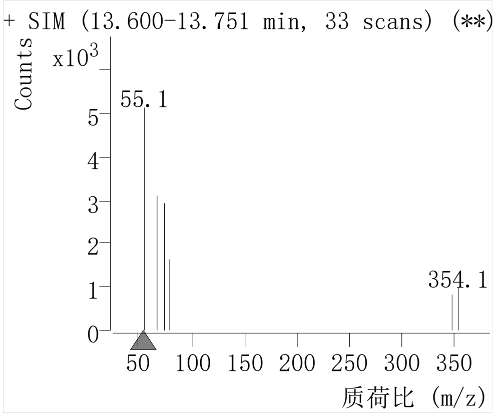
+ SIM (13.600-13.751 min, 33 scans) (**)
Counts x10^3
55.1
354.1
质荷比 (m/z)

**批处理路径**	G:\GC-MS\HX250430-4-GCMS总脂肪酸靶向检测\HX250430-4\QuantResults\HX250430-4.batch.bin		
**分析时间**	2025/5/14 16:58	**分析员姓名**	DESKTOP-M3A0GPO\omics
**报告时间**	2025/5/16 14:53:03	**报告员姓名**	DESKTOP-M3A0GPO\omics
**最近校正更新**	2025/5/14 16:58	**批处理状态**	已处理
**定量批处理版本**	10.2	**定量报告版本**	10.2

**采集时间**	2025/5/8 18:29	**数据文件**	std4.D
**样品类型**	校正	**样品名称**	std4
**稀释**	1	**采集方法**	脂肪酸

样品色谱图

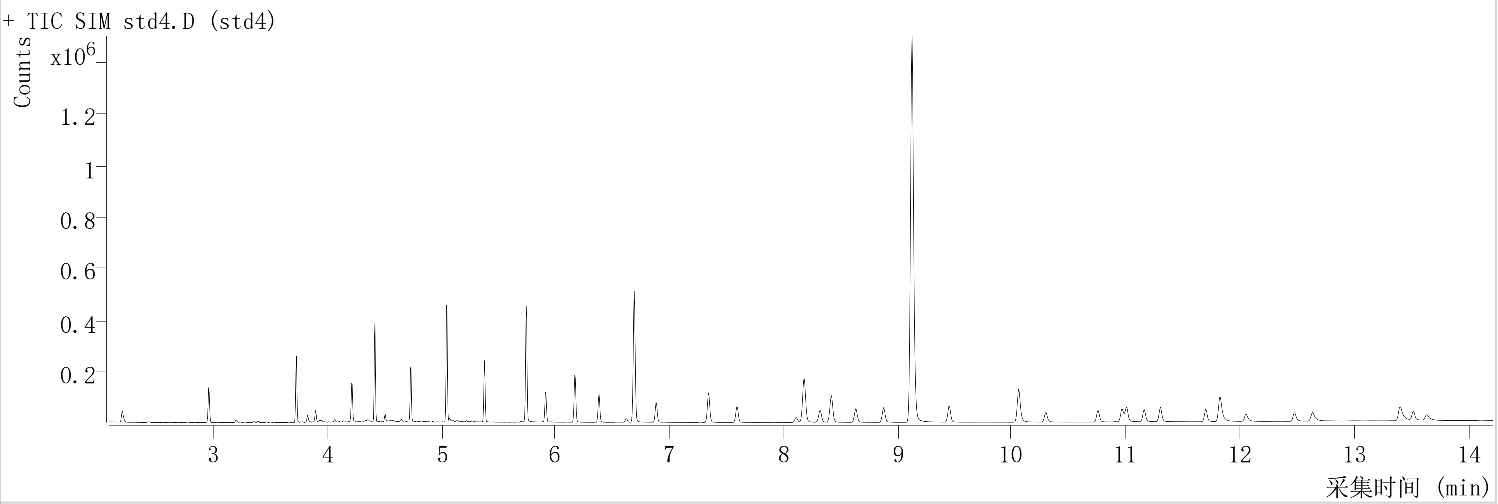

化合物	ISTD	RT	响应	ISTD 响应	响应比	最终浓度	单位
C4:0	C19:0	2.203	47125	2060032	0.0229	2.8298	ug/ml
C6:0	C19:0	2.959	99304	2060032	0.0482	2.6587	ug/ml
C8:0	C19:0	3.728	155912	2060032	0.0757	2.3736	ug/ml
C10:0	C19:0	4.417	227576	2060032	0.1105	2.6886	ug/ml
C11:0	C19:0	4.733	130127	2060032	0.0632	1.3190	ug/ml
C12:0	C19:0	5.049	285188	2060032	0.1384	2.5983	ug/ml
C13:0	C19:0	5.378	146832	2060032	0.0713	1.2667	ug/ml
C14:0	C19:0	5.742	313068	2060032	0.1520	2.8188	ug/ml
C14:1	C19:0	5.911	63277	2060032	0.0307	1.4425	ug/ml
C15:0	C19:0	6.169	150717	2060032	0.0732	1.2339	ug/ml
C15:1	C19:0	6.383	65435	2060032	0.0318	1.2733	ug/ml
C16:0	C19:0	6.690	464812	2060032	0.2256	2.1909	ug/ml
C16:1	C19:0	6.881	48292	2060032	0.0234	1.3442	ug/ml
C17:0	C19:0	7.343	129613	2060032	0.0629	1.3127	ug/ml
C17:1	C19:0	7.592	50280	2060032	0.0244	1.2857	ug/ml
C18:0	C19:0	8.179	245607	2060032	0.1192	2.4450	ug/ml
C18:1n9t	C19:0	8.317	41913	2060032	0.0203	1.2002	ug/ml
C18:1n9c	C19:0	8.419	91542	2060032	0.0444	2.2413	ug/ml
C18:2n6t	C19:0	8.633	44231	2060032	0.0215	1.1957	ug/ml
C18:2n6c	C19:0	8.877	45969	2060032	0.0223	1.2289	ug/ml
C18:3n6	C19:0	9.144	72173	2060032	0.0350	1.3035	ug/ml
C18:3n3	C19:0	9.451	50702	2060032	0.0246	1.2174	ug/ml
C20:0	C19:0	10.060	189204	2060032	0.0918	2.3507	ug/ml
C20:1	C19:0	10.300	37218	2060032	0.0181	1.1533	ug/ml
C20:2	C19:0	10.758	36144	2060032	0.0175	1.1270	ug/ml
C21:0	C19:0	10.967	86000	2060032	0.0417	1.1486	ug/ml
C20:3n6	C19:0	11.007	35762	2060032	0.0174	1.1406	ug/ml
C20:4n6	C19:0	11.163	37388	2060032	0.0181	1.1670	ug/ml
C20:3n3	C19:0	11.305	42681	2060032	0.0207	1.1342	ug/ml
C20:5n3	C19:0	11.701	42763	2060032	0.0208	1.1825	ug/ml

化合物	ISTD	RT	响应	ISTD 响应	响应比	最终浓度	单位
C22:0	C19:0	11.825	150469	2060032	0.0730	2.3852	ug/ml
C22:1n9	C19:0	12.052	32144	2060032	0.0156	1.1404	ug/ml
C22:2n6	C19:0	12.479	28462	2060032	0.0138	1.1288	ug/ml
C23:0	C19:0	12.639	65099	2060032	0.0316	1.2001	ug/ml
C24:0	C19:0	13.404	112375	2060032	0.0546	2.4661	ug/ml
C22:6	C19:0	13.524	33231	2060032	0.0161	1.1968	ug/ml
C24:1	C19:0	13.640	26091	2060032	0.0127	1.1119	ug/ml

C4:0

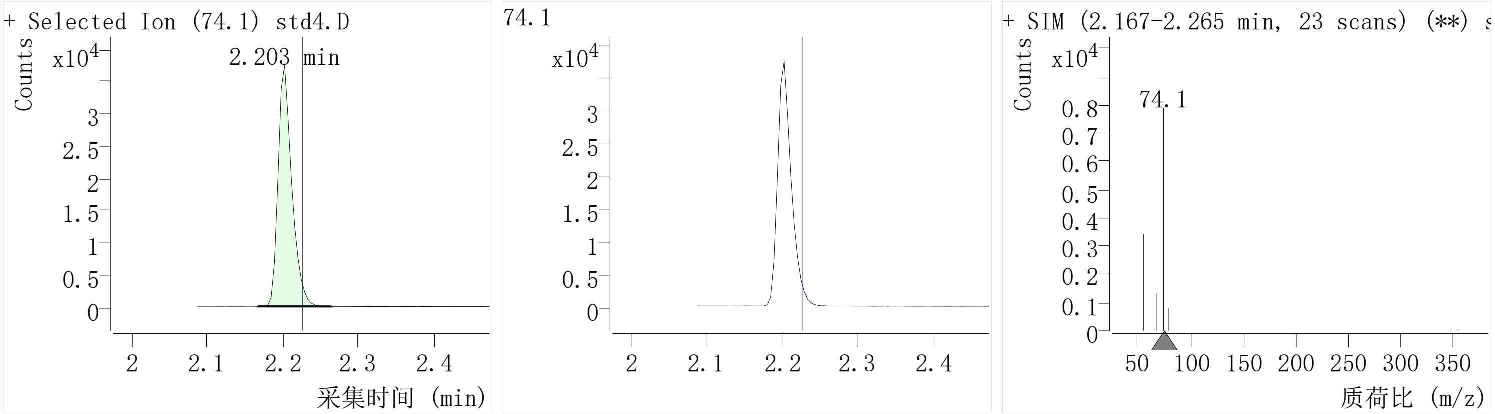

C6:0

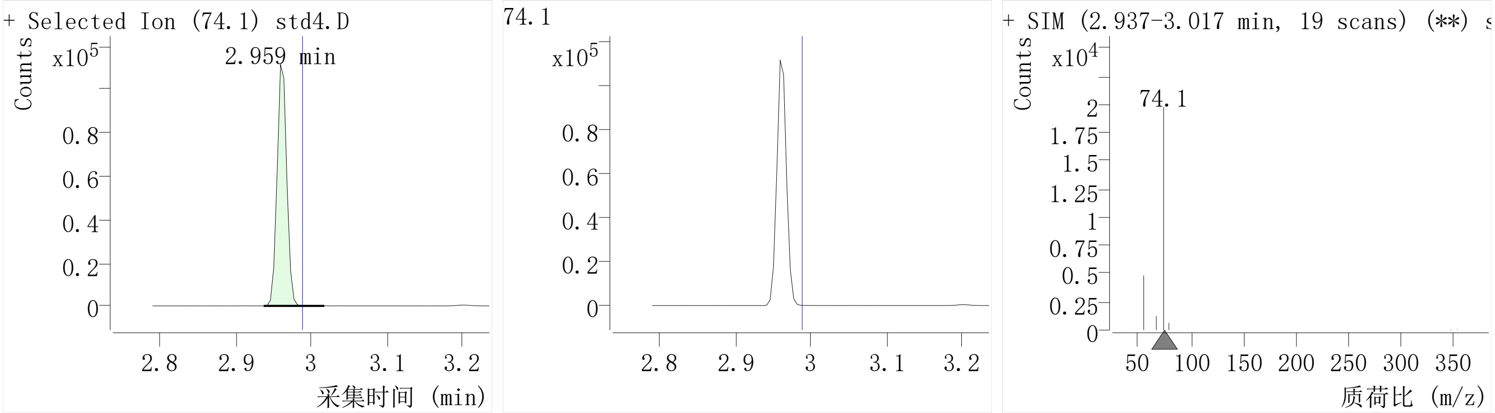

C8:0

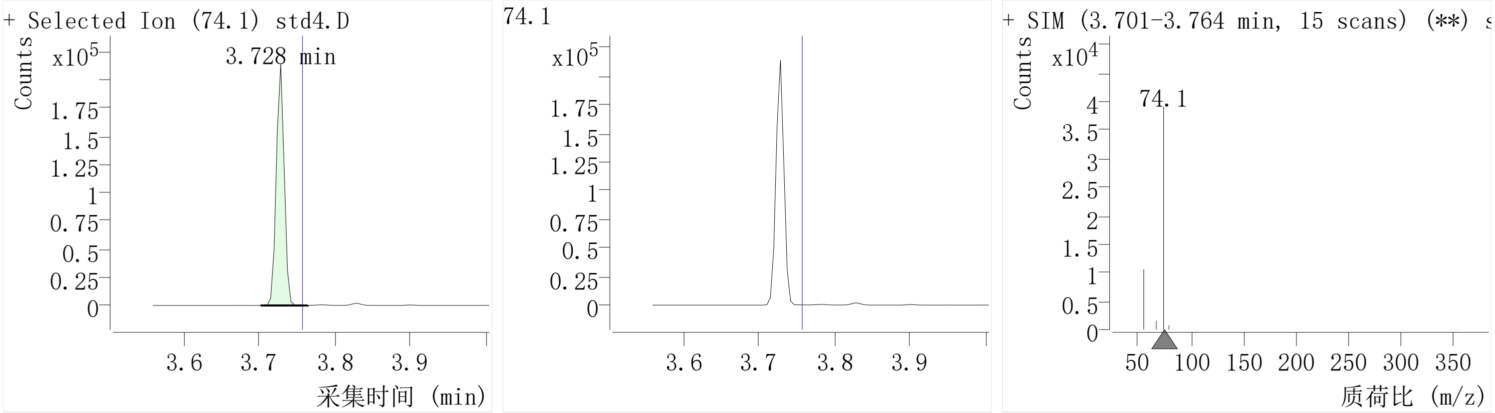

C10:0

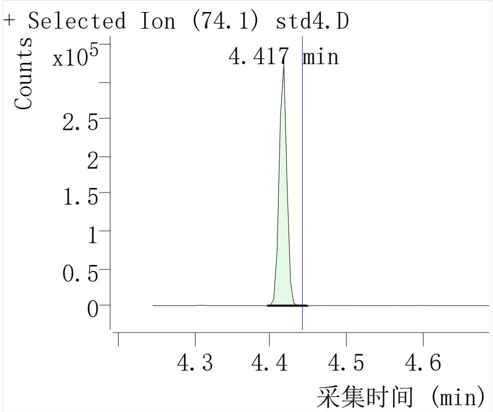

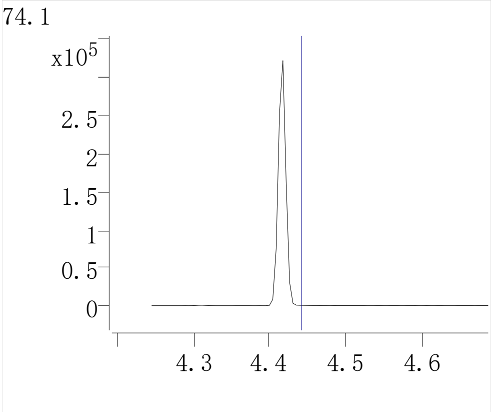

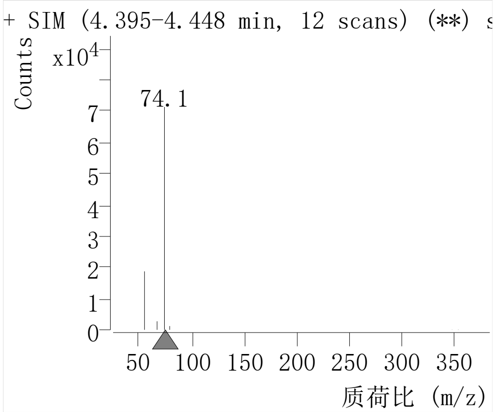

C11:0

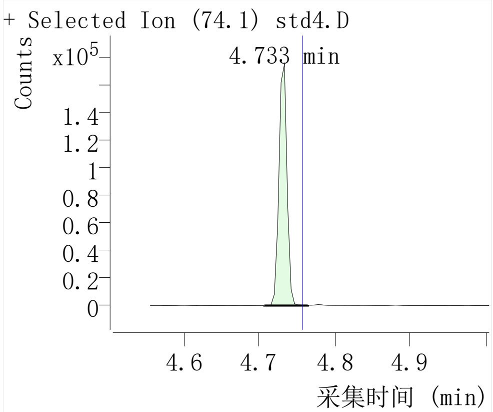

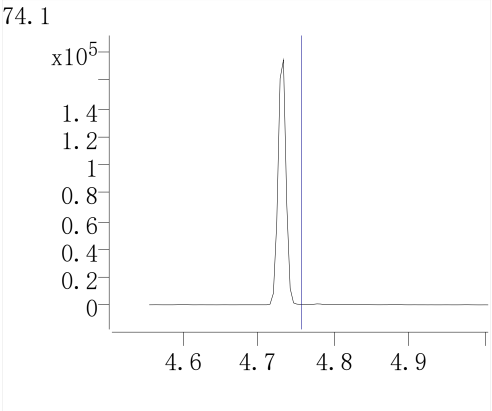

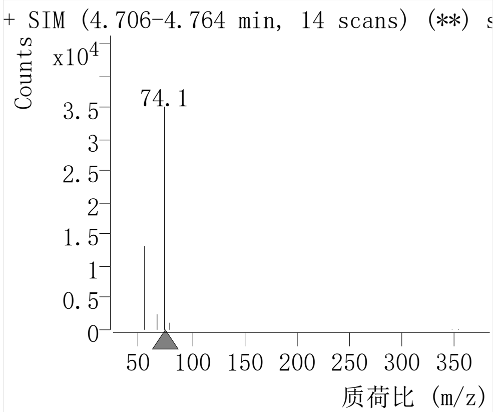

C12:0

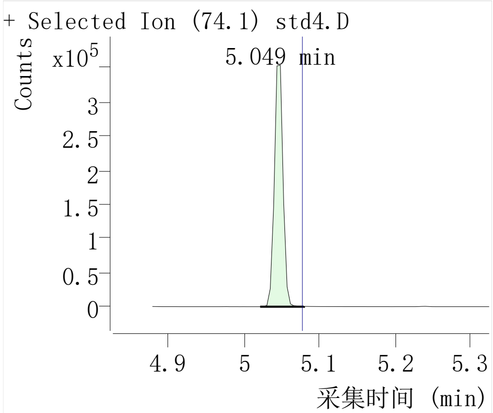

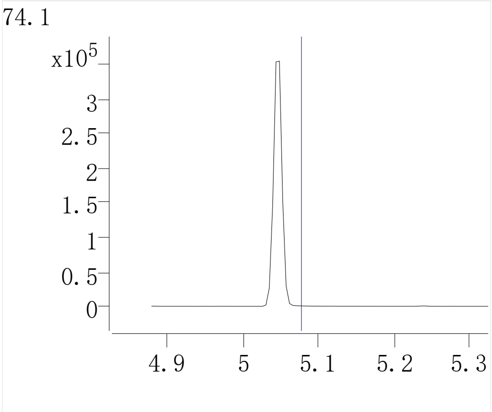

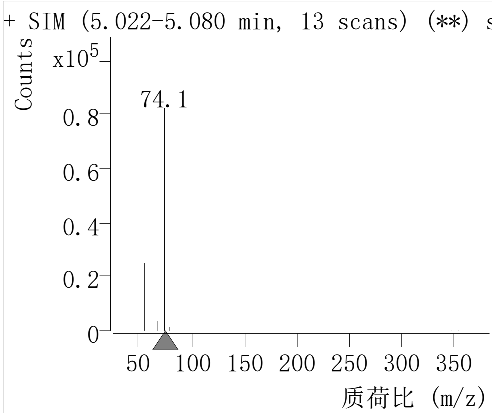

C13:0

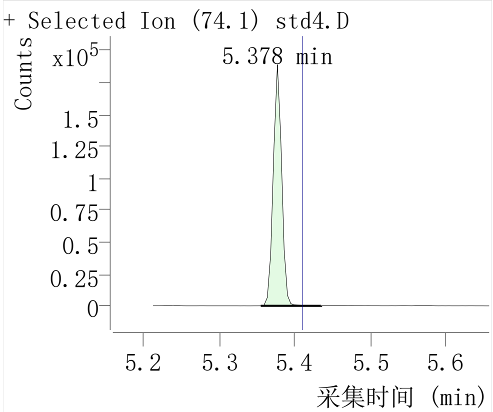

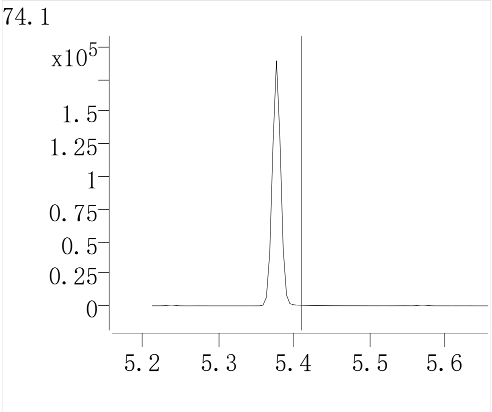

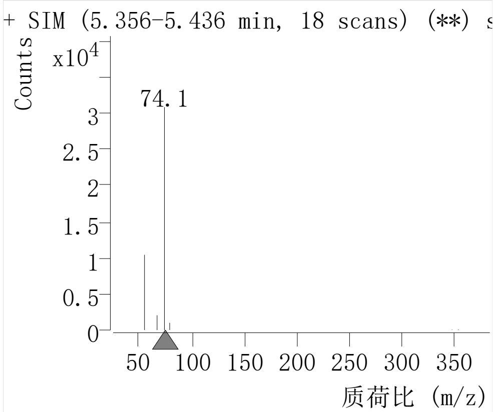

C14:0

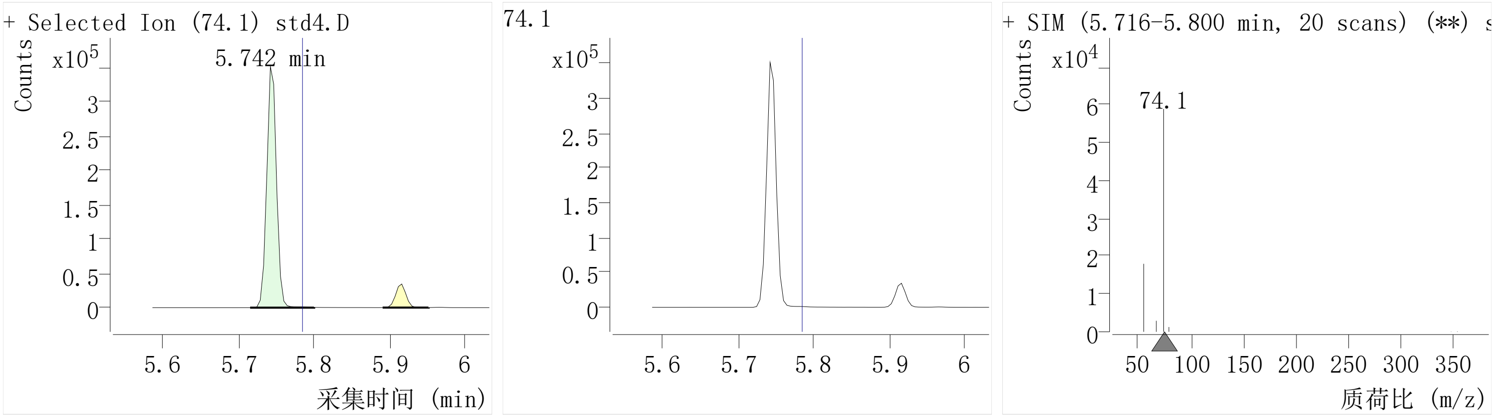

C14:1

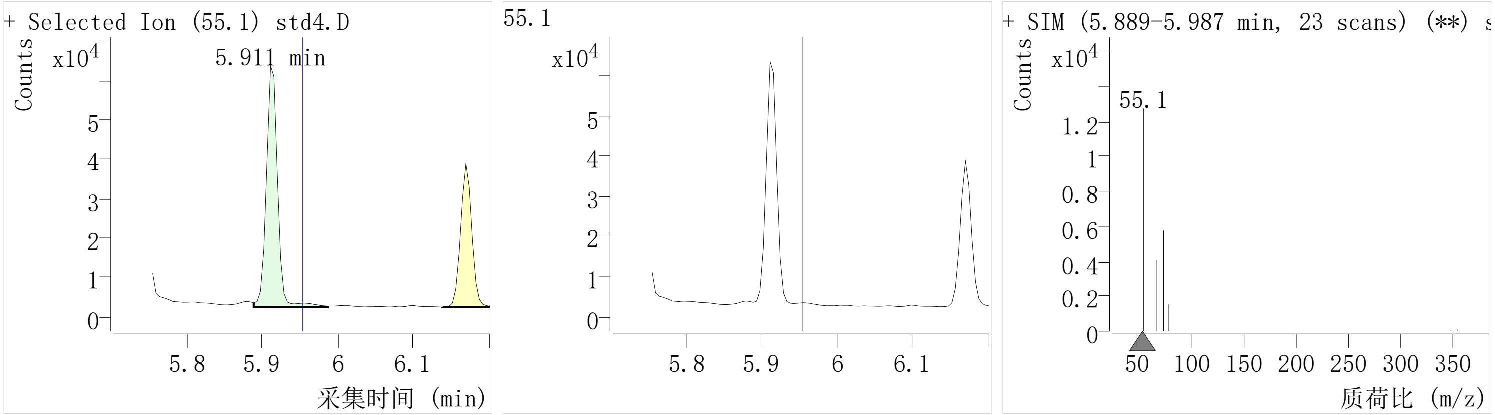

C15:0

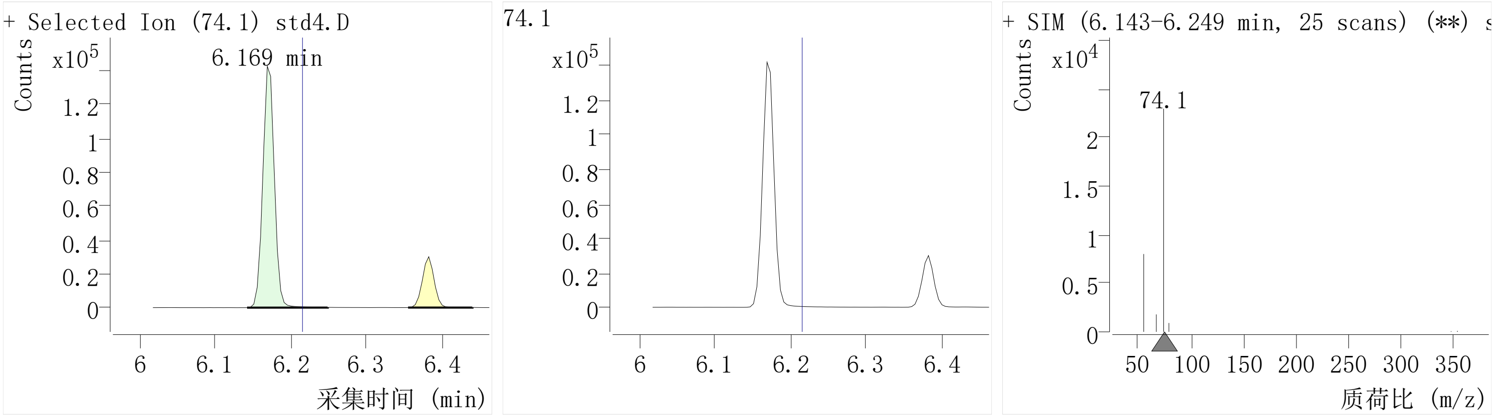

C15:1

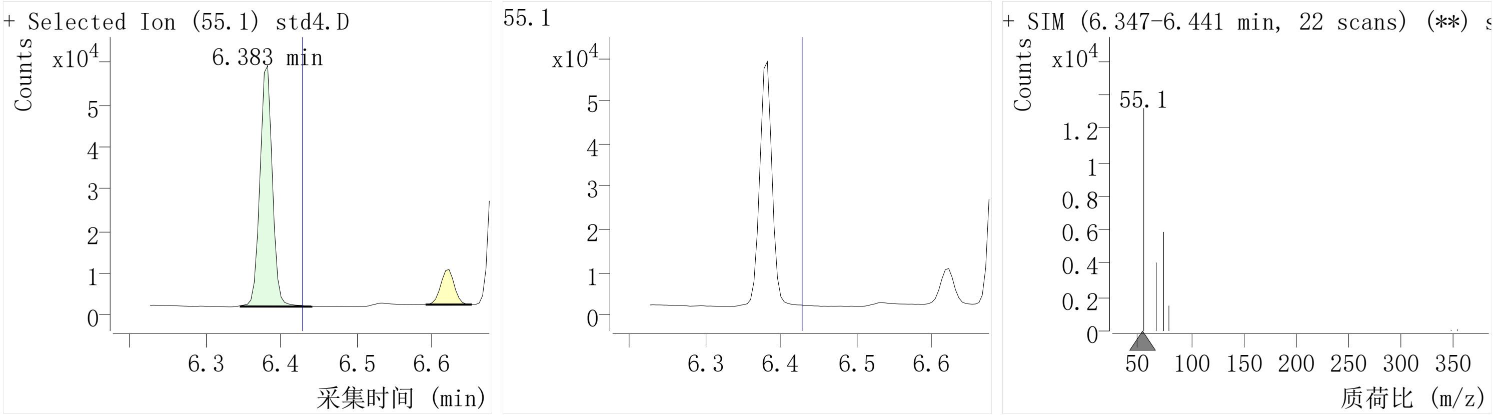

C16:0

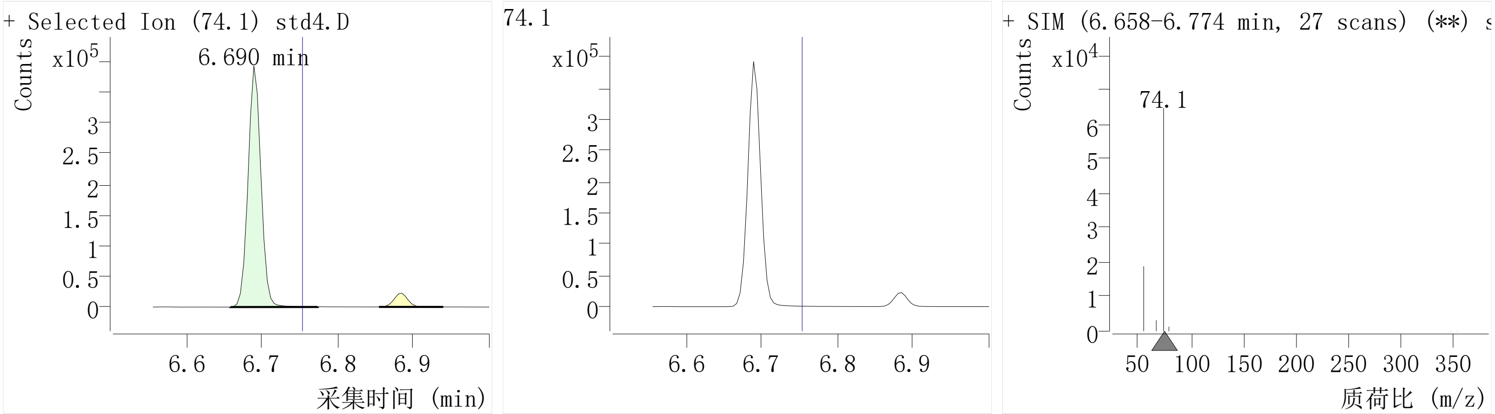

C16:1

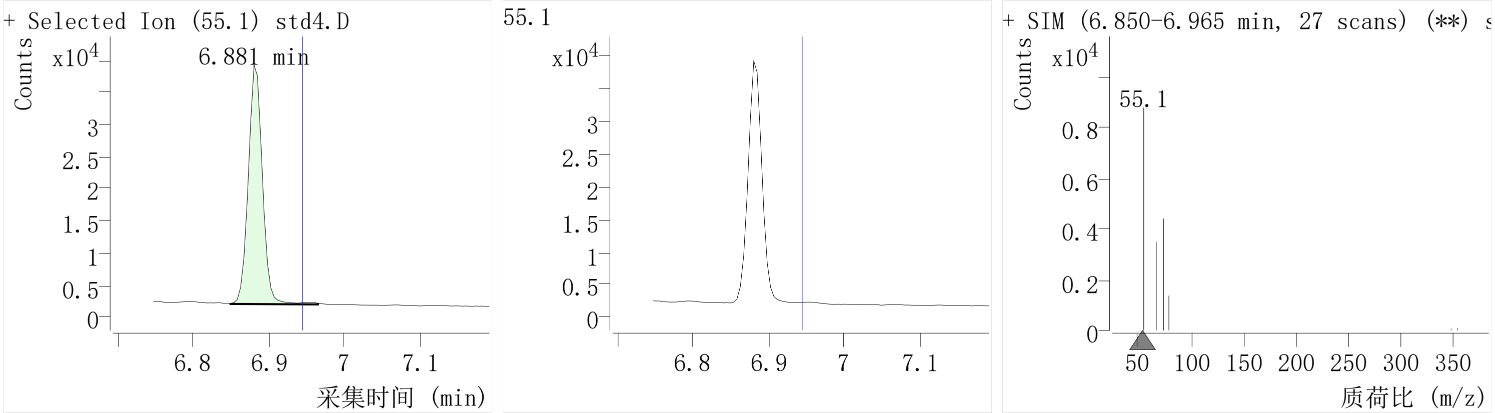

C17:0

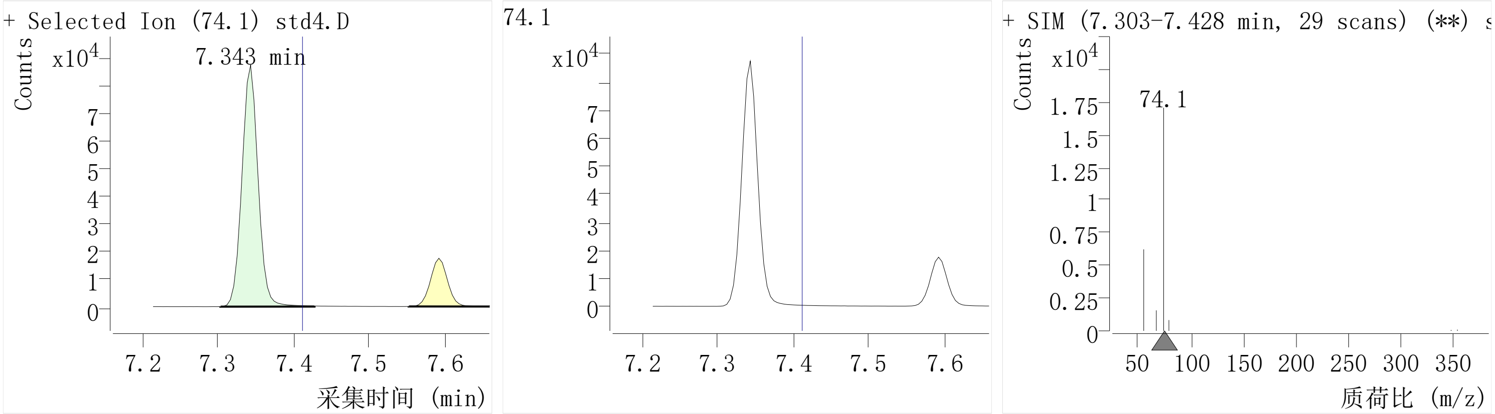

C17:1

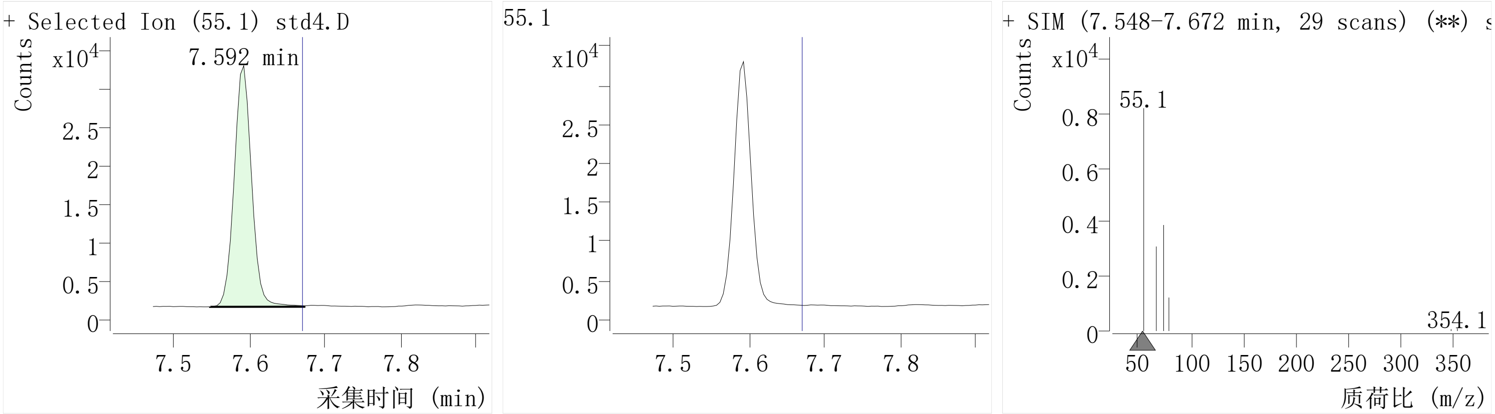

C18:0

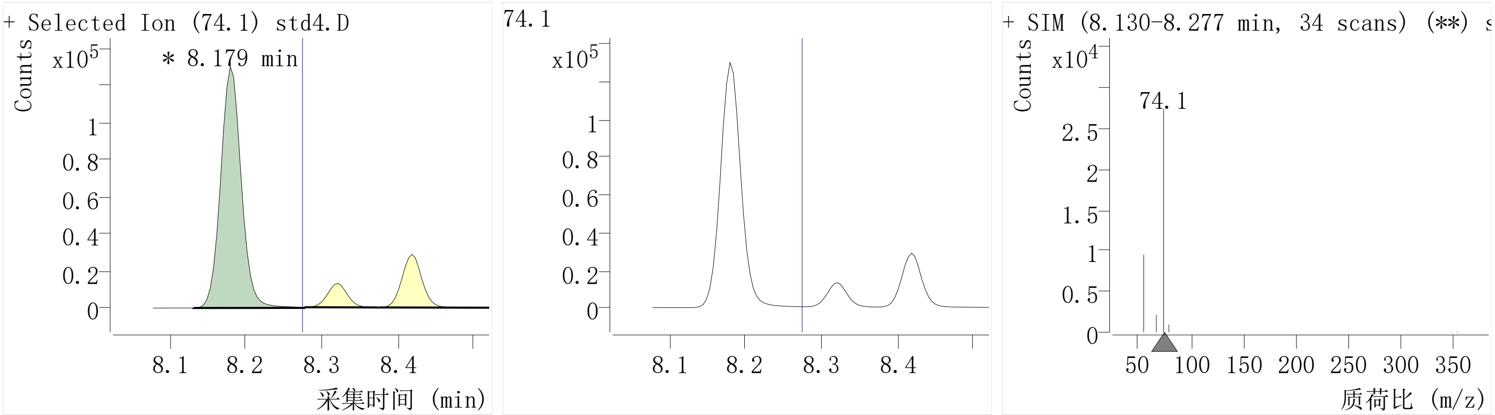

C18:1n9t

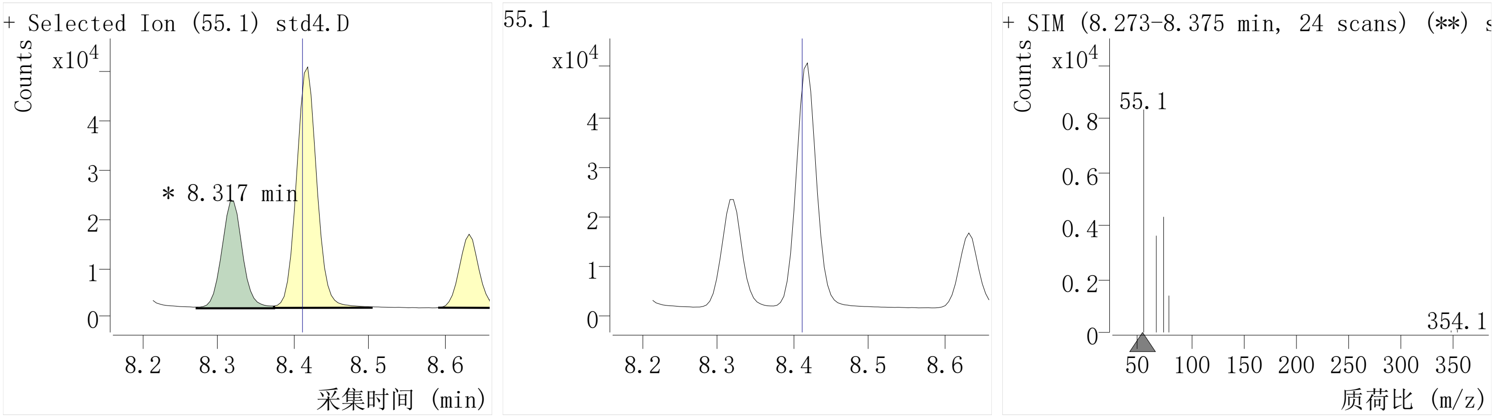

C18:1n9c

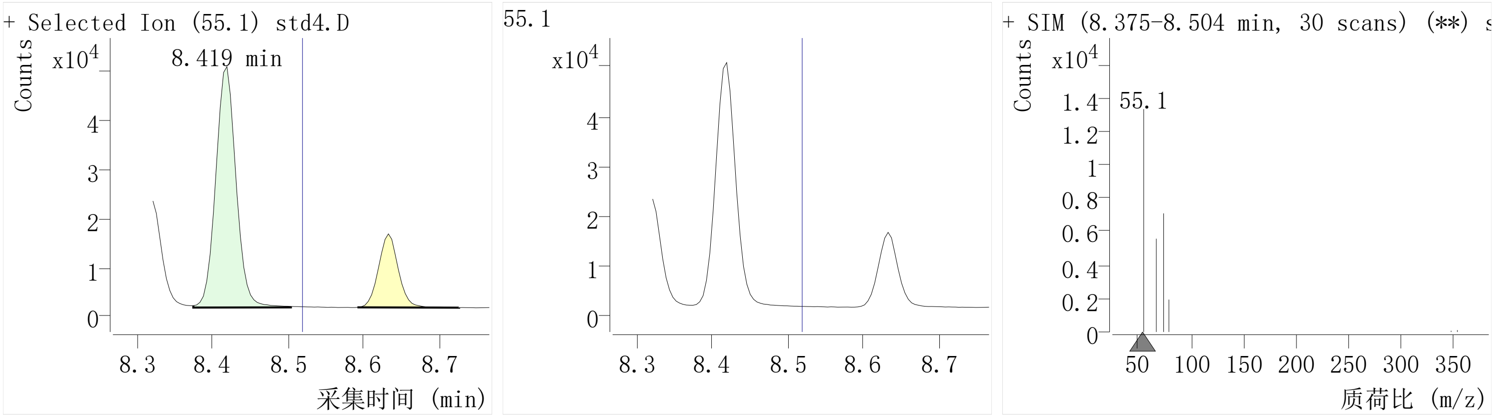

C18:2n6t

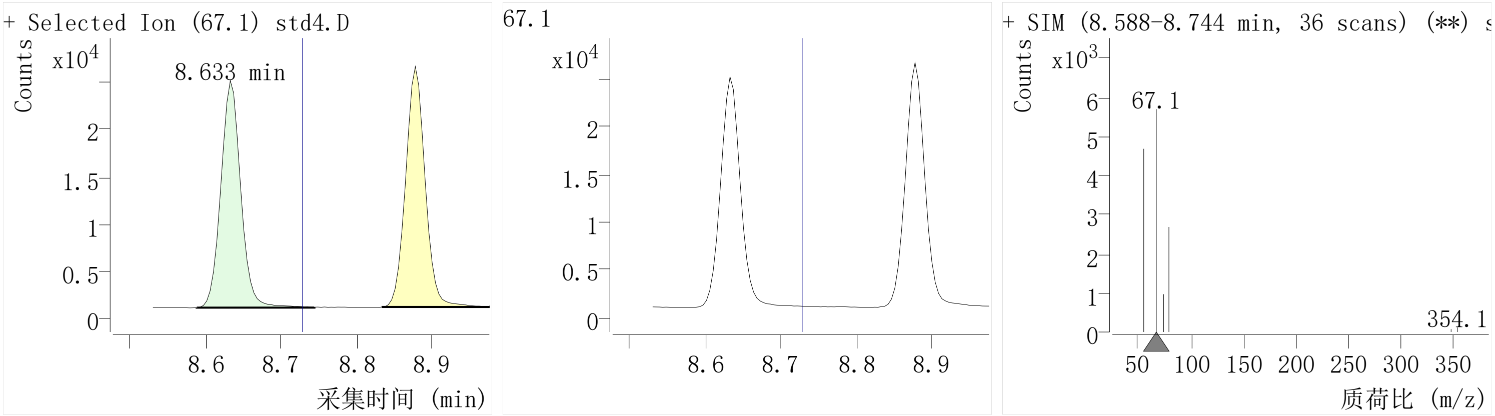

C18:2n6c

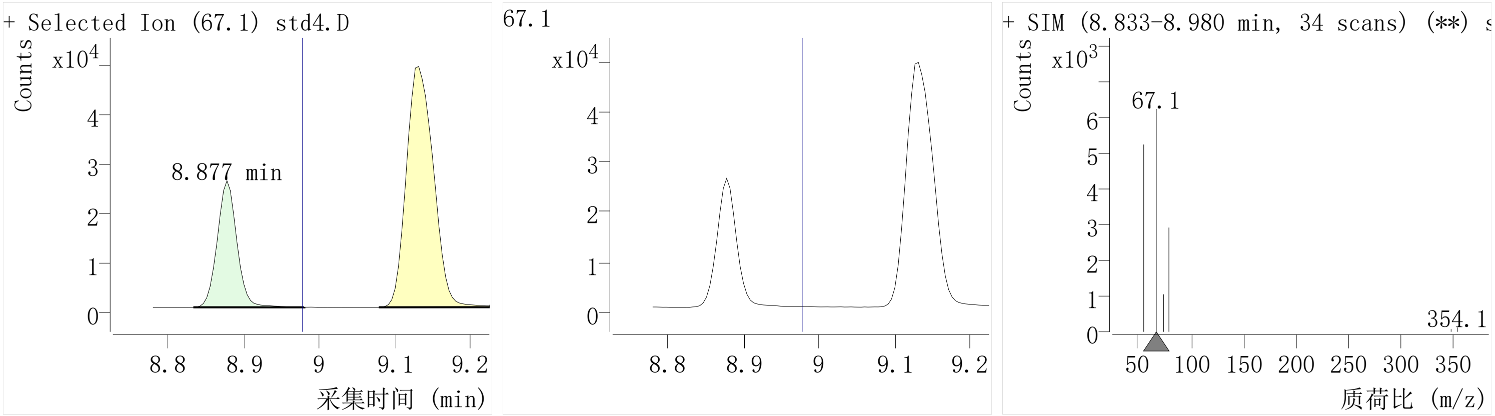

C18:3n6

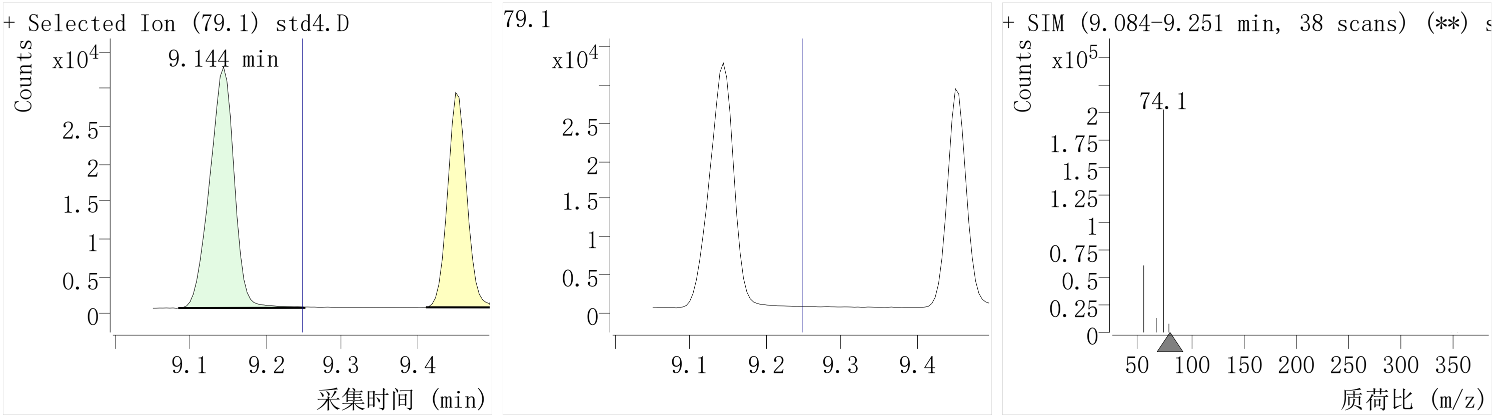

C18:3n3

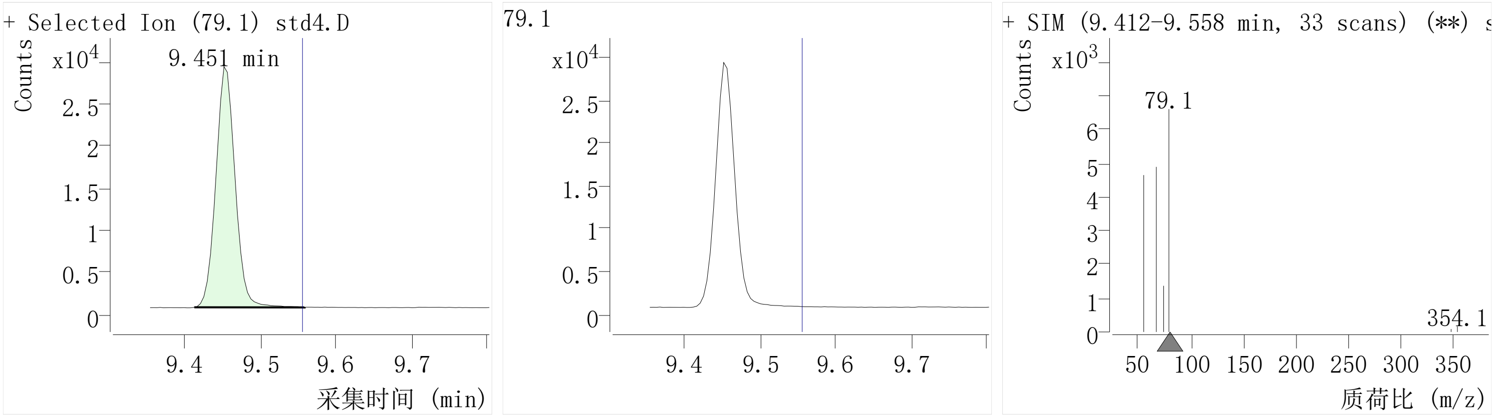

C20:0

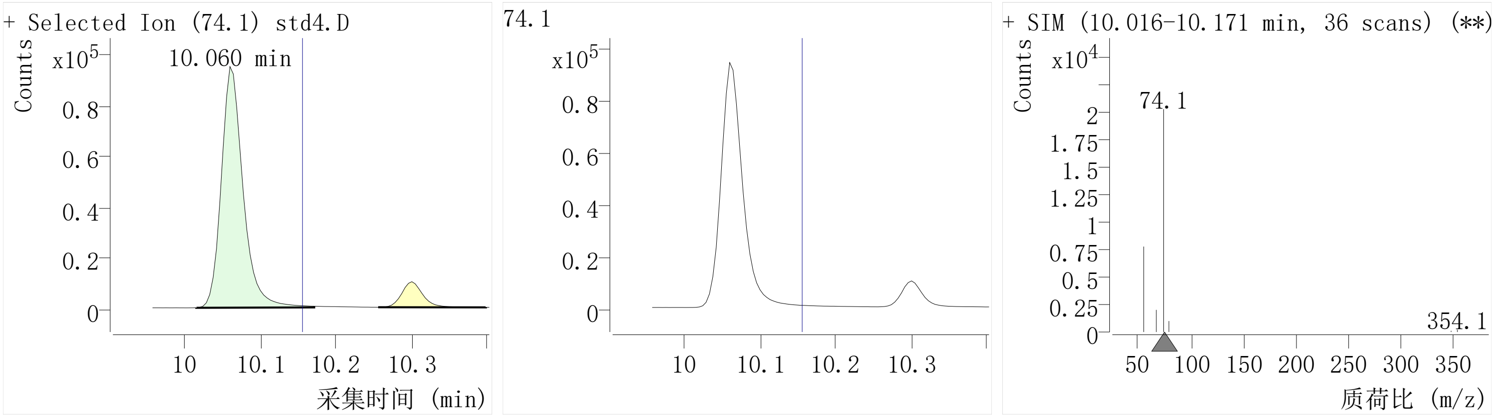

C20:1

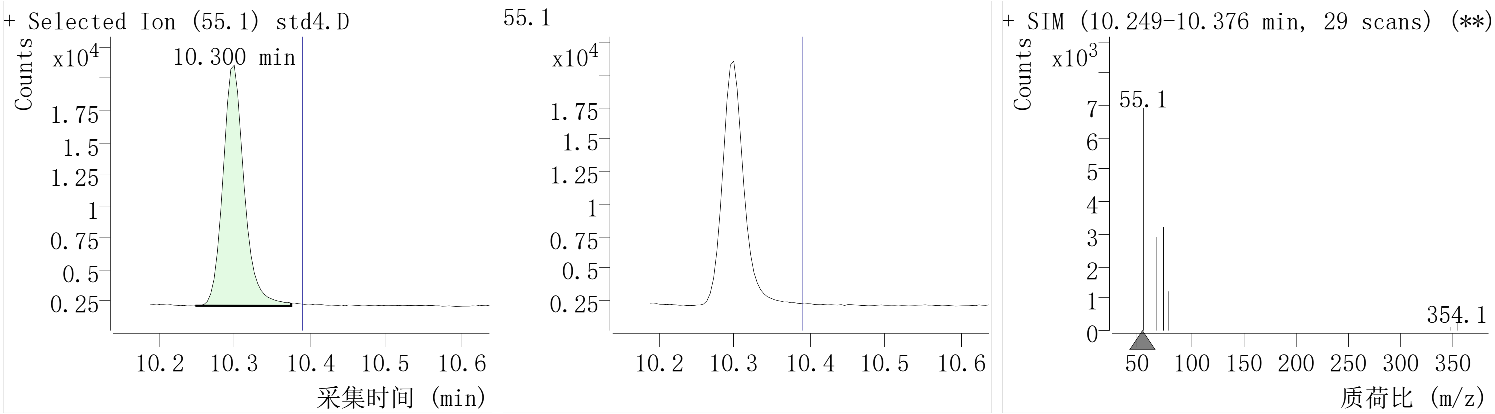

C20:2

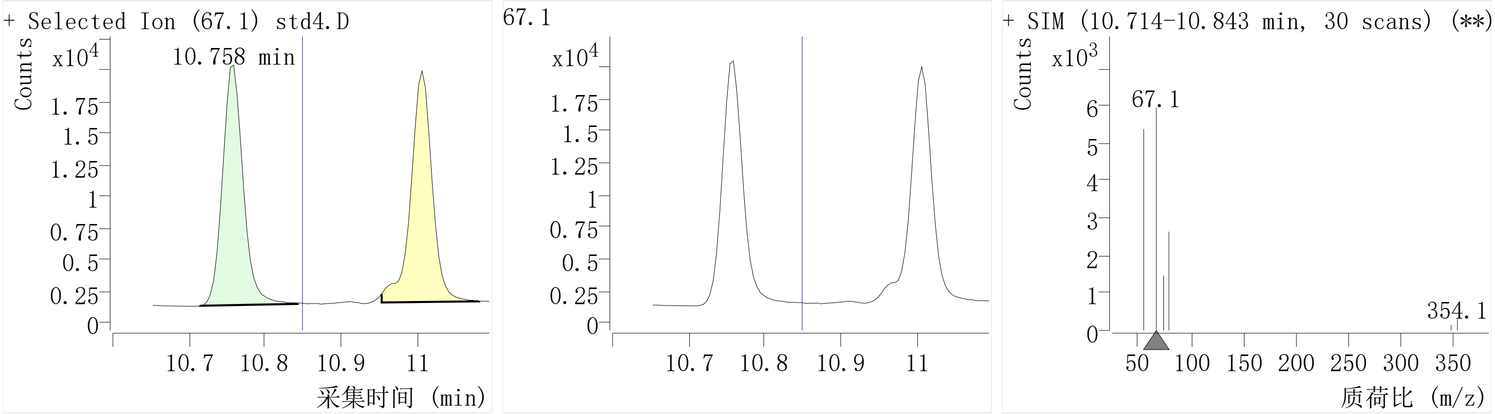

C21:0

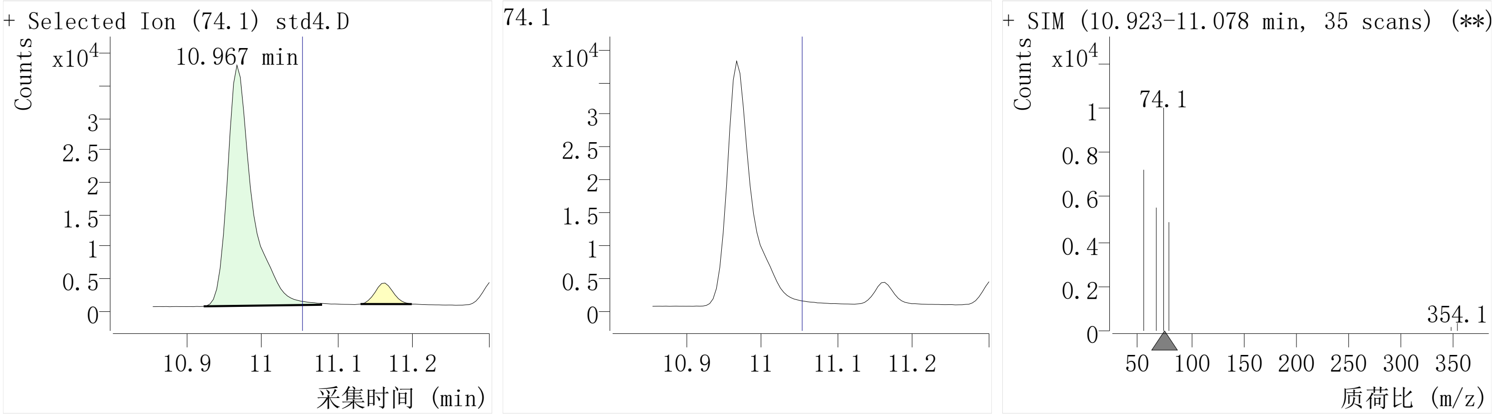

C20:3n6

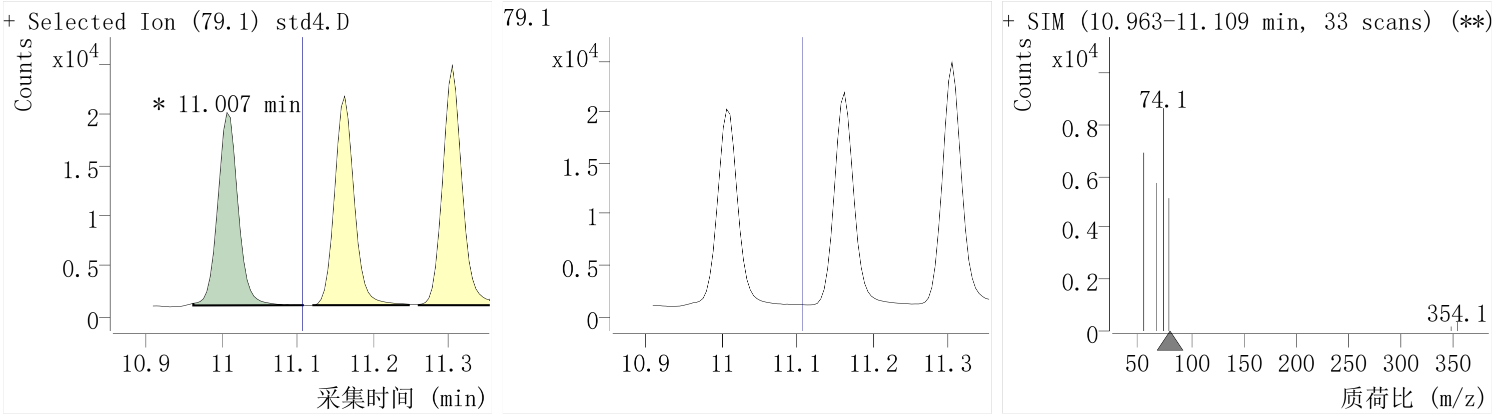

C20:4n6

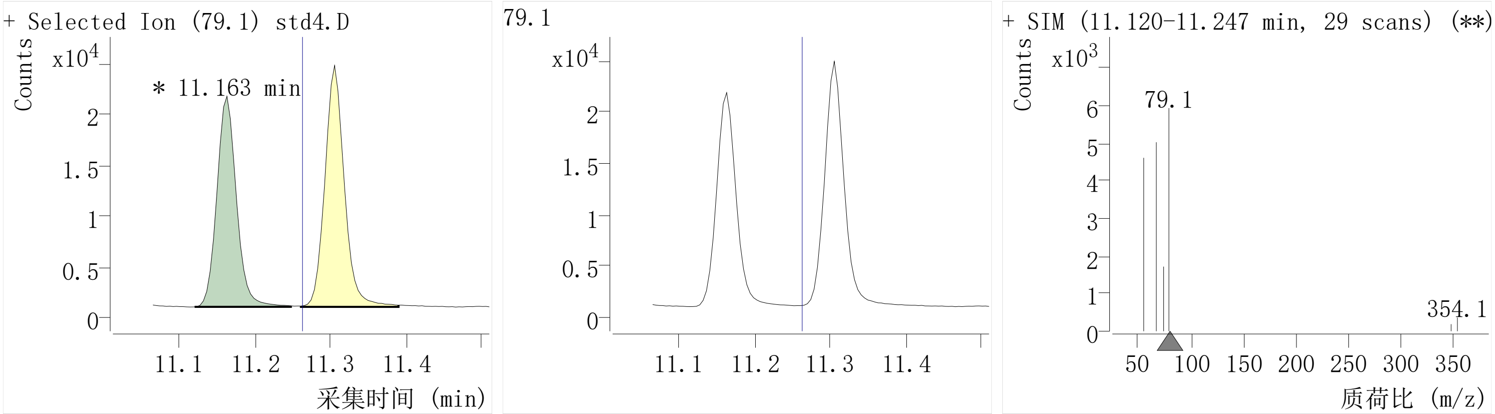

C20:3n3

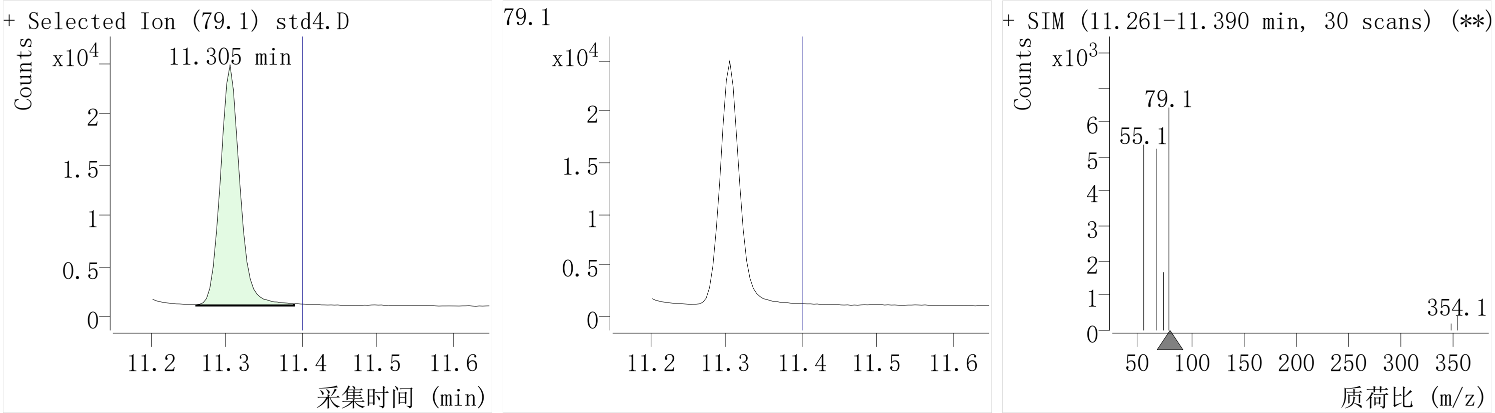

C20:5n3

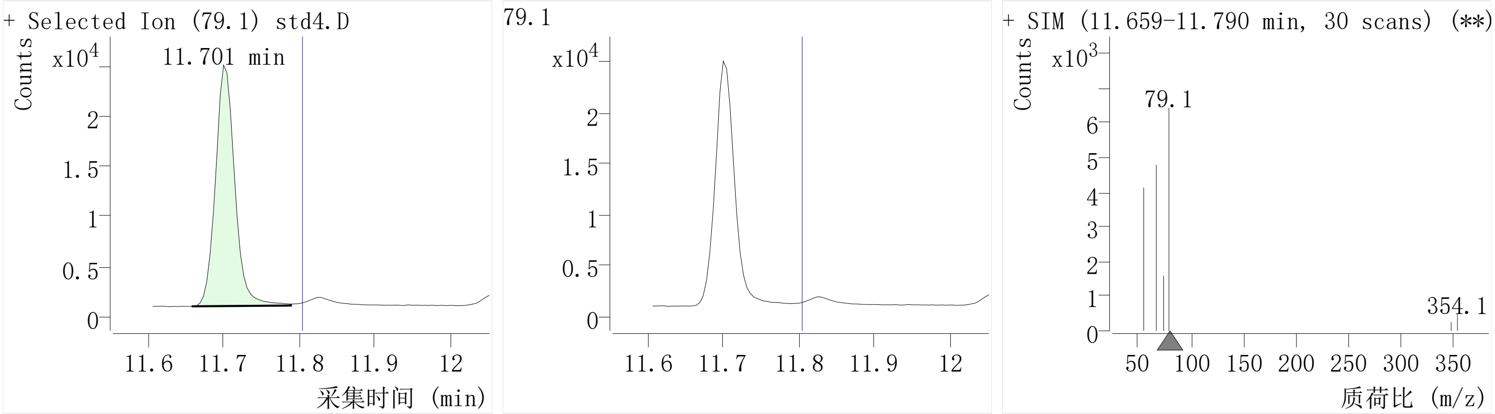

C22:0

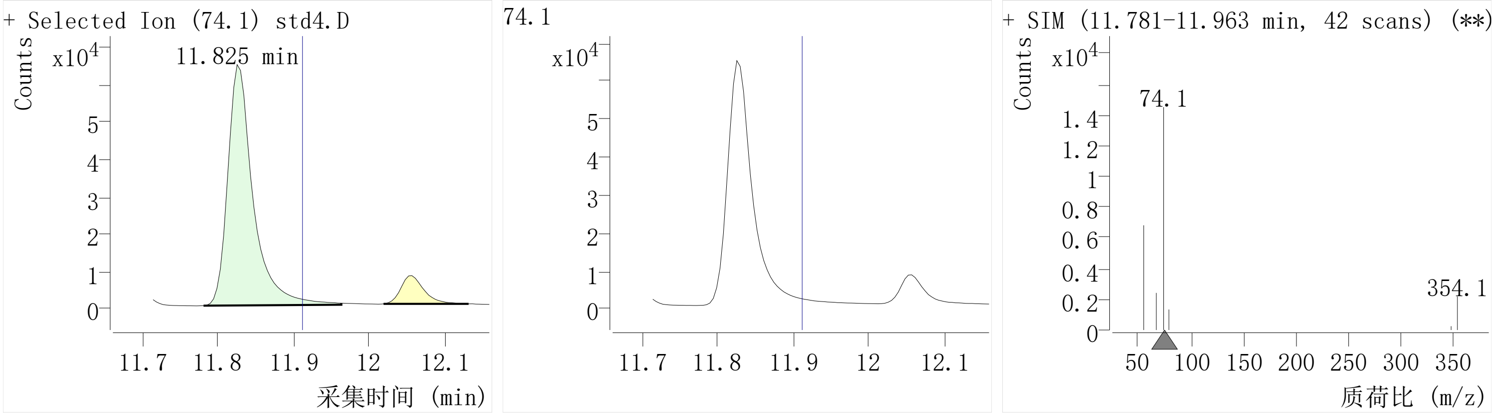

C22:1n9

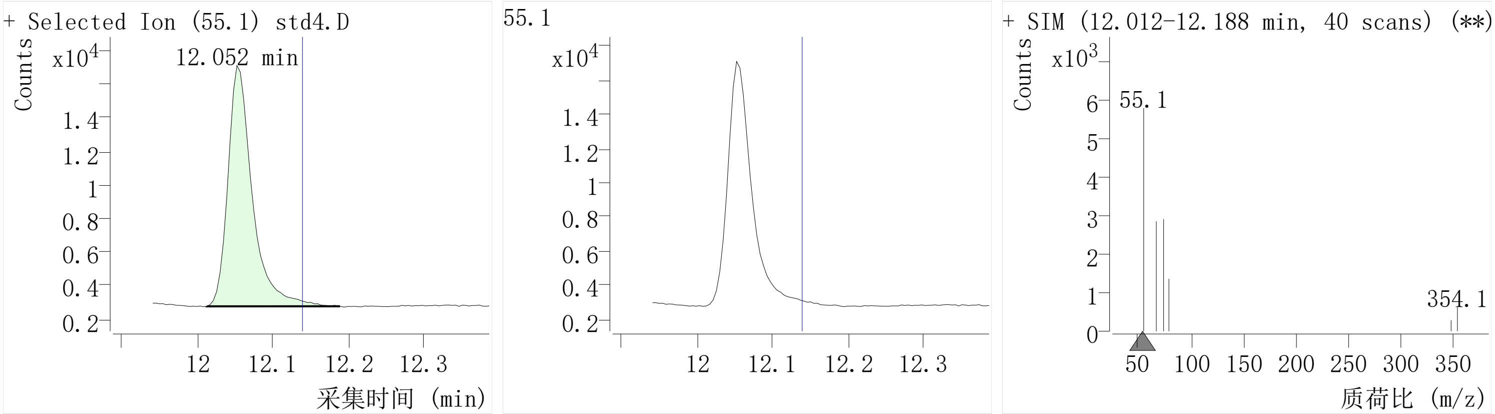

C22:2n6

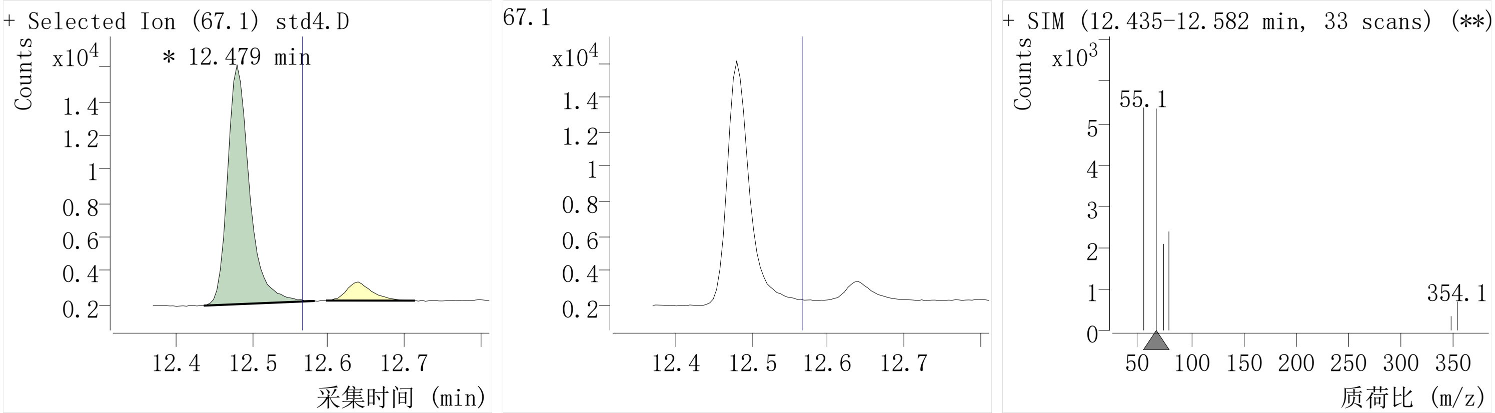

C23:0

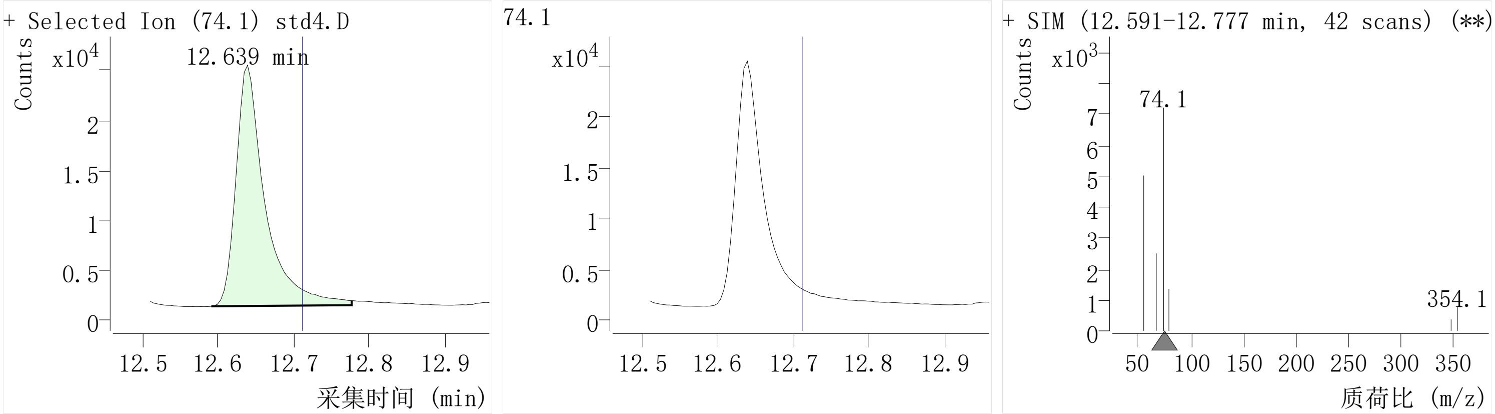

C24:0

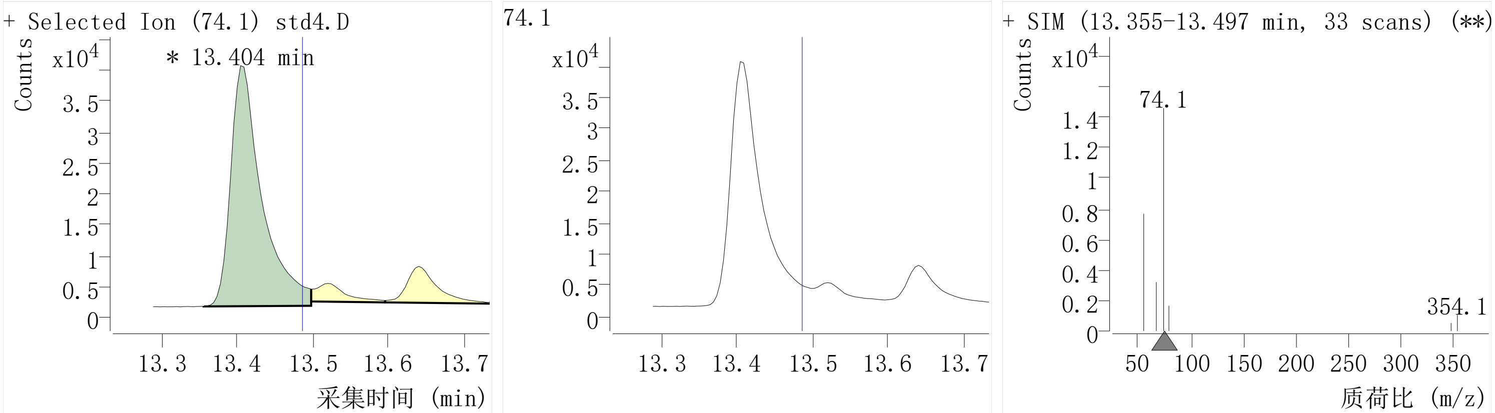

C22:6

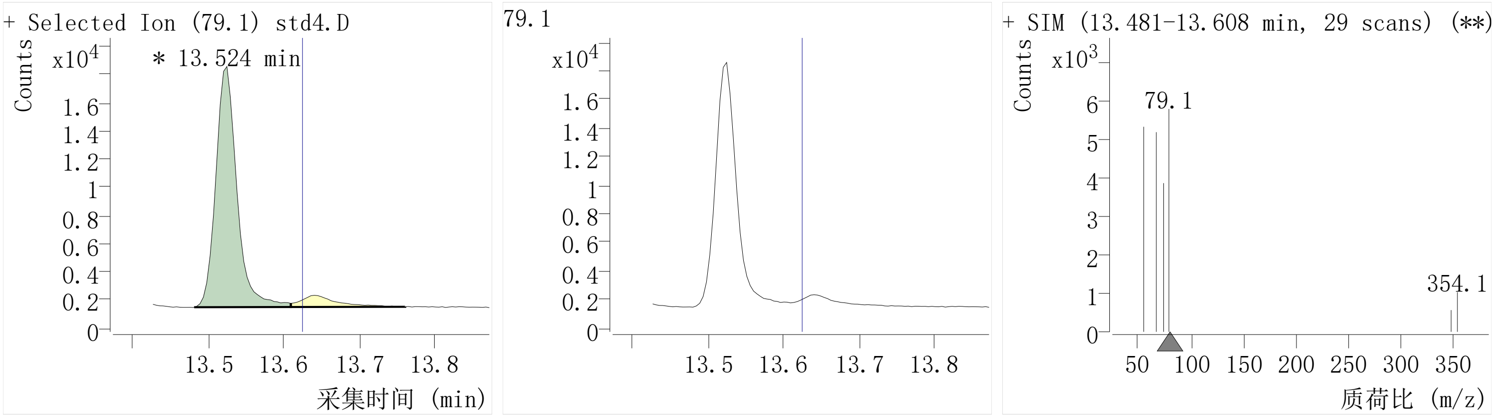
+ Selected Ion (79.1) std4.D
Counts
x10^4
* 13.524 min
1.6
1.4
1.2
1
0.8
0.6
0.4
0.2
0
13.5
13.6
13.7
13.8
采集时间 (min)
79.1
x10^4
1.6
1.4
1.2
1
0.8
0.6
0.4
0.2
0
13.5
13.6
13.7
13.8
+ SIM (13.481-13.608 min, 29 scans) (**)
Counts
x10^3
79.1
354.1
6
5
4
3
2
1
0
50
100
150
200
250
300
350
质荷比 (m/z)

C24:1

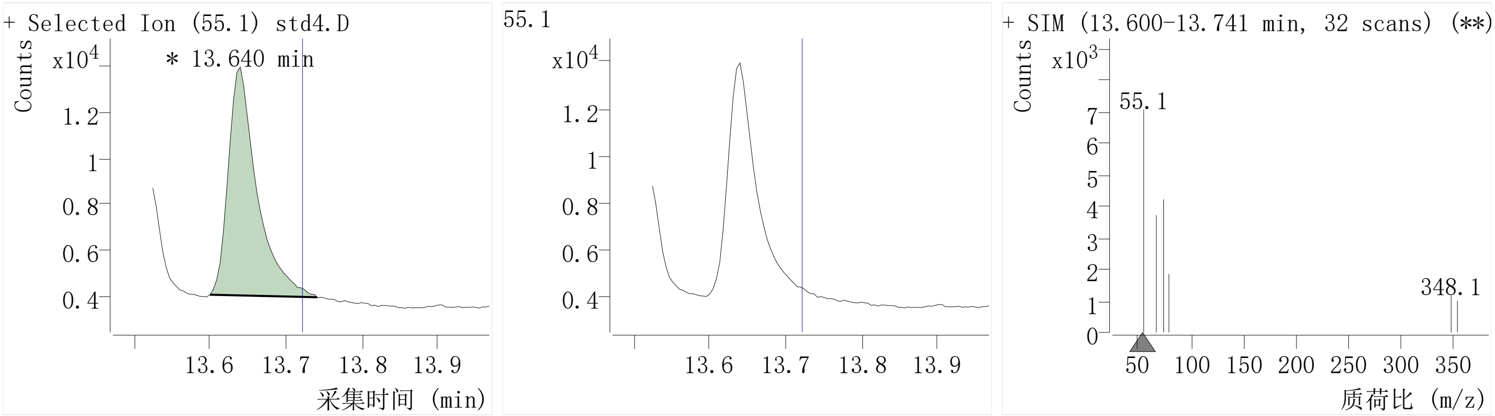
+ Selected Ion (55.1) std4.D
Counts
x10^4
* 13.640 min
1.2
1
0.8
0.6
0.4
13.6
13.7
13.8
13.9
采集时间 (min)
55.1
x10^4
1.2
1
0.8
0.6
0.4
13.6
13.7
13.8
13.9
+ SIM (13.600-13.741 min, 32 scans) (**)
Counts
x10^3
55.1
348.1
7
6
5
4
3
2
1
0
50
100
150
200
250
300
350
质荷比 (m/z)

定量分析完成报告

**批处理路径**	G:\GC-MS\HX250430-4-GCMS总脂肪酸靶向检测\HX250430-4\QuantResults\HX250430-4.batch.bin		
**分析时间**	2025/5/14 16:58	**分析员姓名**	DESKTOP-M3A0GPO\omics
**报告时间**	2025/5/16 14:53:05	**报告员姓名**	DESKTOP-M3A0GPO\omics
**最近校正更新**	2025/5/14 16:58	**批处理状态**	已处理
**定量批处理版本**	10.2	**定量报告版本**	10.2

**采集时间**	2025/5/8 18:49	**数据文件**	std5.D
**样品类型**	校正	**样品名称**	std5
**稀释**	1	**采集方法**	脂肪酸

样品色谱图

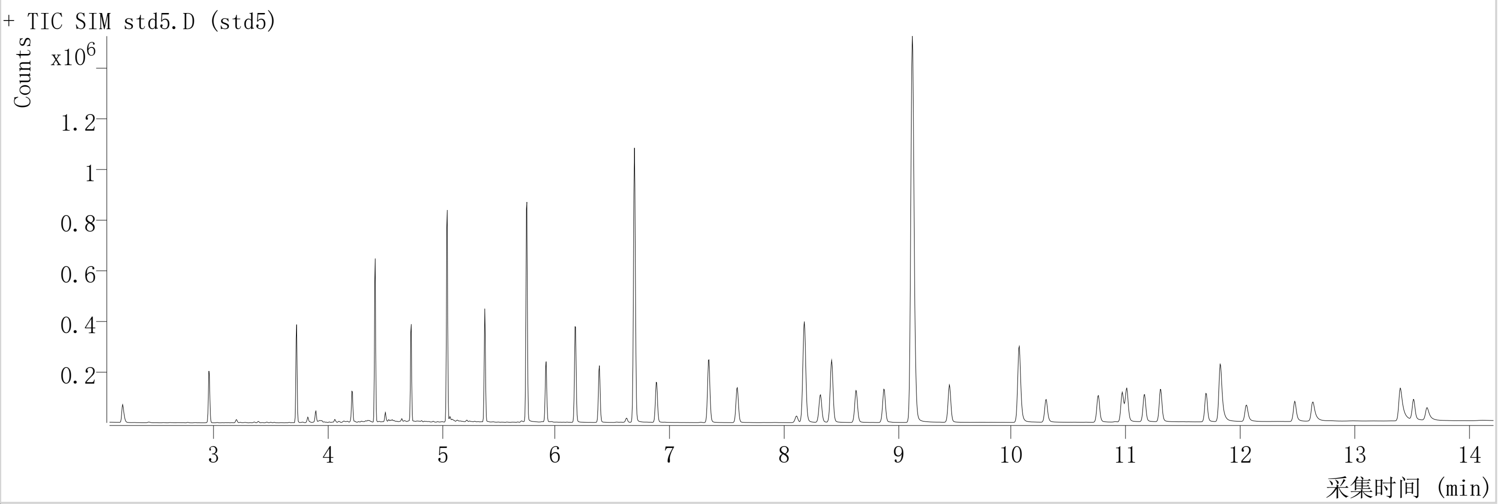

化合物	ISTD	RT	响应	ISTD 响应	响应比	最终浓度	单位
C4:0	C19:0	2.203	84256	2159349	0.0390	5.4861	ug/ml
C6:0	C19:0	2.959	155929	2159349	0.0722	4.5660	ug/ml
C8:0	C19:0	3.728	241330	2159349	0.1118	4.0542	ug/ml
C10:0	C19:0	4.417	376172	2159349	0.1742	4.7220	ug/ml
C11:0	C19:0	4.733	224035	2159349	0.1038	2.4024	ug/ml
C12:0	C19:0	5.049	516546	2159349	0.2392	5.0125	ug/ml
C13:0	C19:0	5.378	279061	2159349	0.1292	2.5281	ug/ml
C14:0	C19:0	5.747	619173	2159349	0.2867	5.4012	ug/ml
C14:1	C19:0	5.916	118980	2159349	0.0551	2.6725	ug/ml
C15:0	C19:0	6.174	310000	2159349	0.1436	2.6165	ug/ml
C15:1	C19:0	6.383	134263	2159349	0.0622	2.8539	ug/ml
C16:0	C19:0	6.689	986954	2159349	0.4571	5.8654	ug/ml
C16:1	C19:0	6.881	103309	2159349	0.0478	2.8354	ug/ml
C17:0	C19:0	7.343	287436	2159349	0.1331	2.7771	ug/ml
C17:1	C19:0	7.592	110720	2159349	0.0513	2.7327	ug/ml
C18:0	C19:0	8.179	558555	2159349	0.2587	5.2141	ug/ml
C18:1n9t	C19:0	8.321	95905	2159349	0.0444	2.6200	ug/ml
C18:1n9c	C19:0	8.419	211106	2159349	0.0978	5.1902	ug/ml
C18:2n6t	C19:0	8.633	102750	2159349	0.0476	2.6272	ug/ml
C18:2n6c	C19:0	8.877	106206	2159349	0.0492	2.5065	ug/ml
C18:3n6	C19:0	9.144	133432	2159349	0.0618	2.7858	ug/ml
C18:3n3	C19:0	9.451	117286	2159349	0.0543	2.6942	ug/ml
C20:0	C19:0	10.064	437966	2159349	0.2028	5.1500	ug/ml
C20:1	C19:0	10.300	86339	2159349	0.0400	2.5039	ug/ml
C20:2	C19:0	10.758	85641	2159349	0.0397	2.4783	ug/ml
C21:0	C19:0	10.967	196118	2159349	0.0908	2.4987	ug/ml
C20:3n6	C19:0	11.007	83881	2159349	0.0388	2.4975	ug/ml
C20:4n6	C19:0	11.163	85894	2159349	0.0398	2.5156	ug/ml
C20:3n3	C19:0	11.305	99056	2159349	0.0459	2.4493	ug/ml
C20:5n3	C19:0	11.701	98917	2159349	0.0458	2.5669	ug/ml

定量分析完成报告

化合物	ISTD	RT	响应	ISTD 响应	响应比	最终浓度	单位
C22:0	C19:0	11.825	339739	2159349	0.1573	5.1144	ug/ml
C22:1n9	C19:0	12.057	72217	2159349	0.0334	2.4611	ug/ml
C22:2n6	C19:0	12.479	64810	2159349	0.0300	2.4088	ug/ml
C23:0	C19:0	12.639	143829	2159349	0.0666	2.5521	ug/ml
C24:0	C19:0	13.404	250237	2159349	0.1159	5.2319	ug/ml
C22:6	C19:0	13.524	76310	2159349	0.0353	2.6157	ug/ml
C24:1	C19:0	13.640	56528	2159349	0.0262	2.3037	ug/ml

C4:0

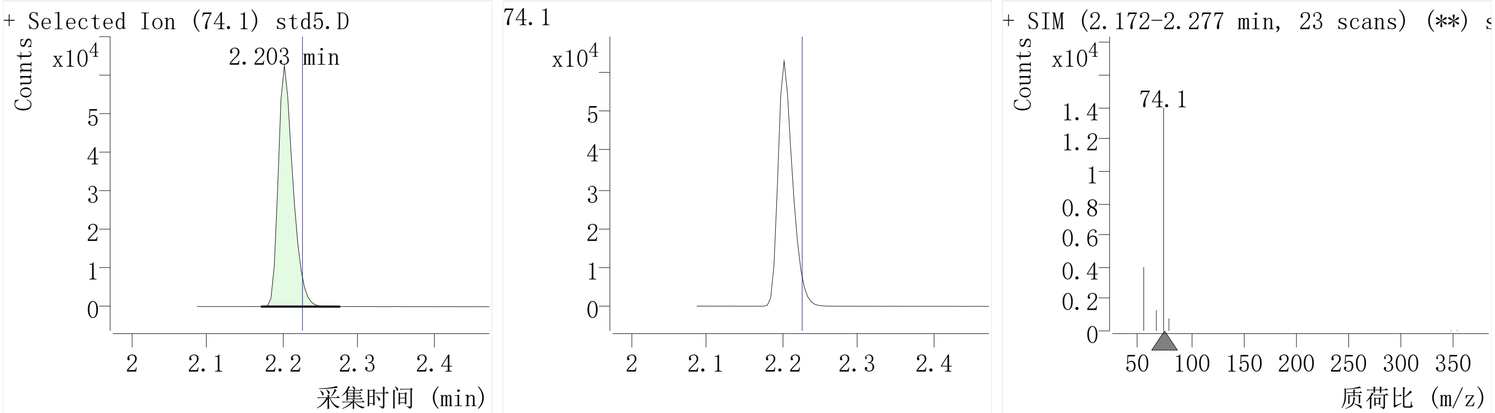

C6:0

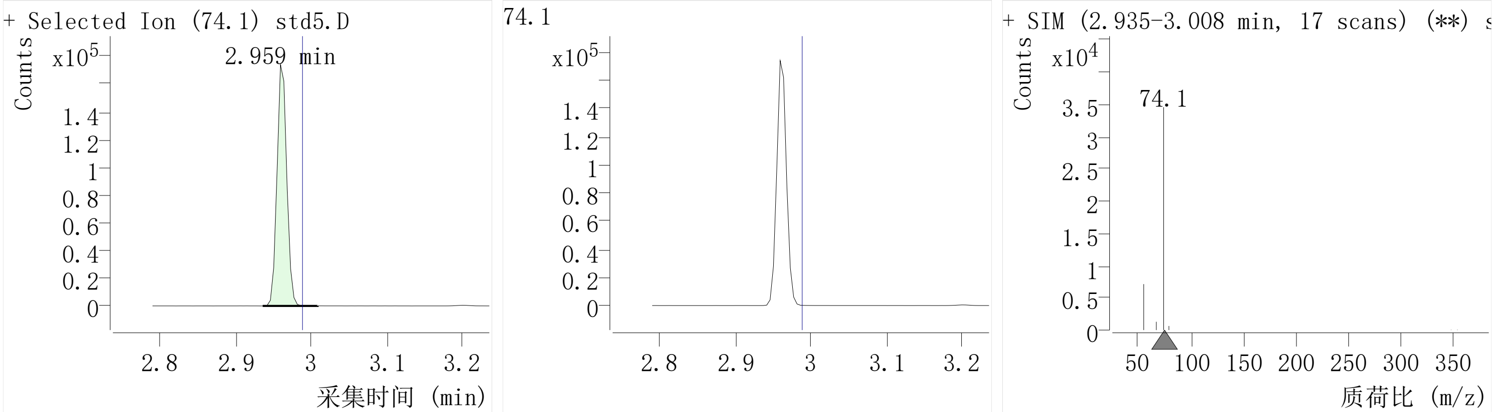

C8:0

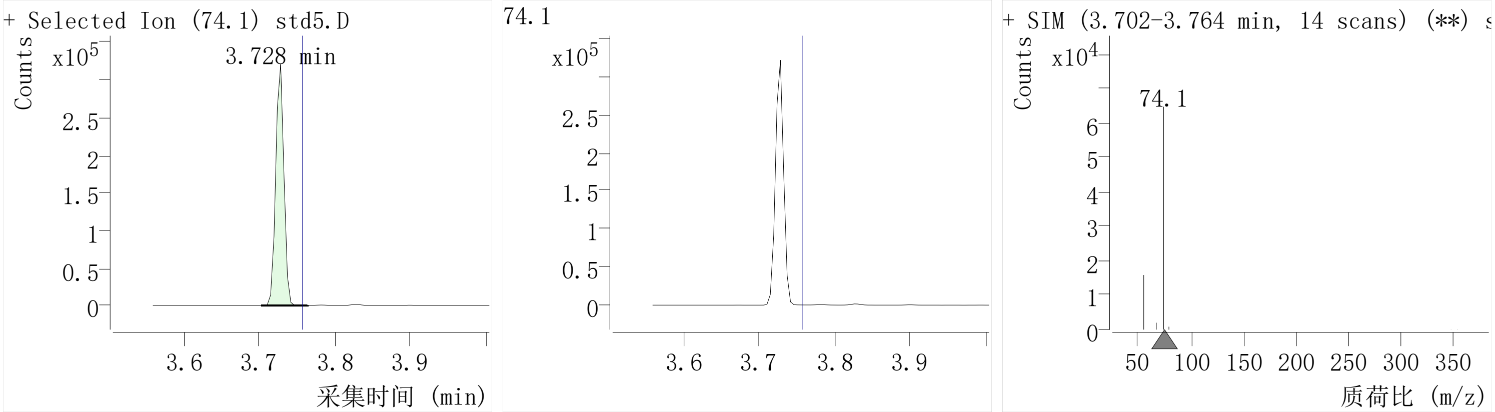

C10:0

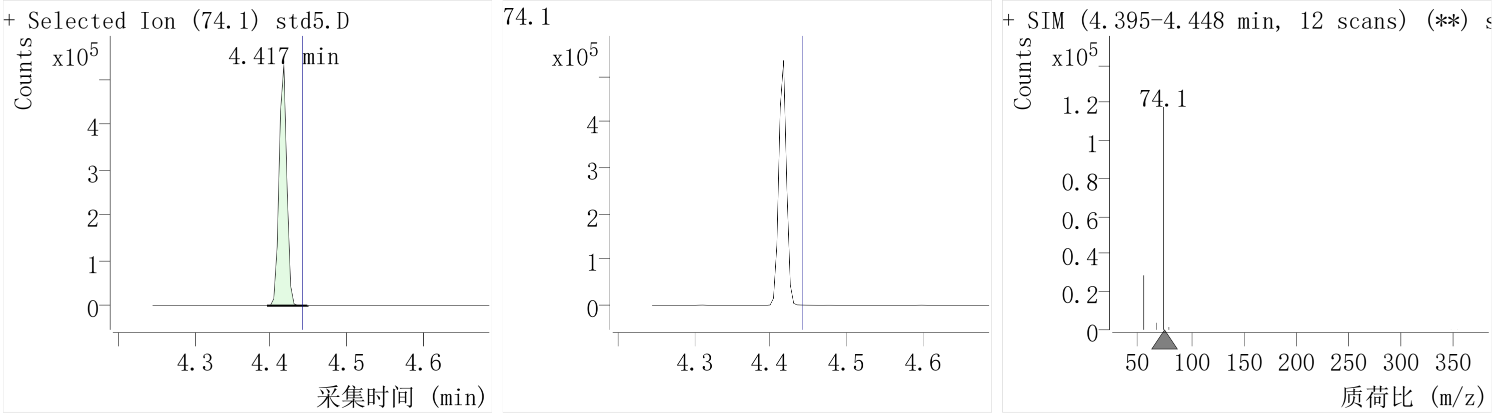

C11:0

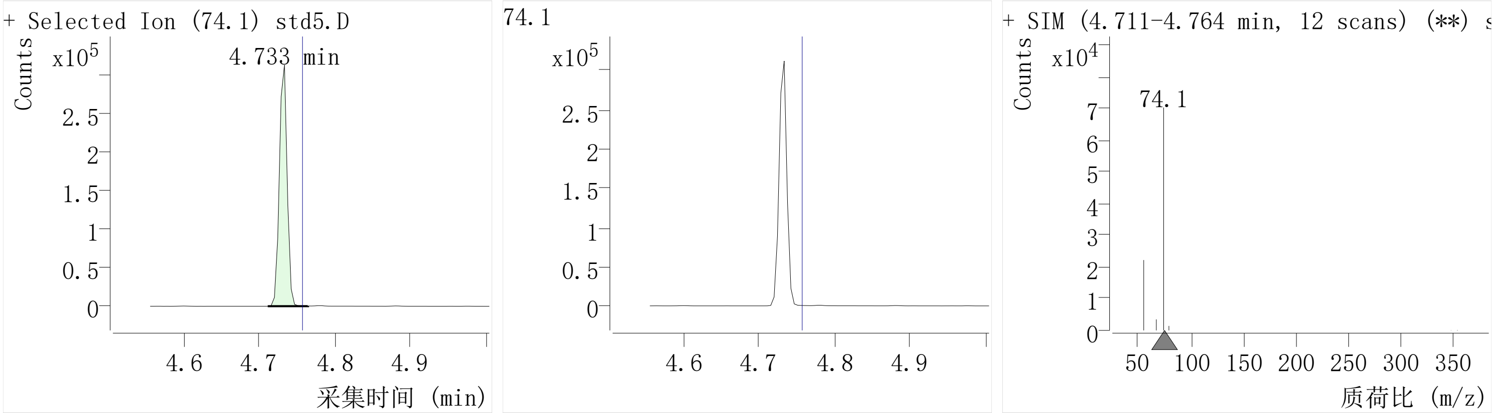

C12:0

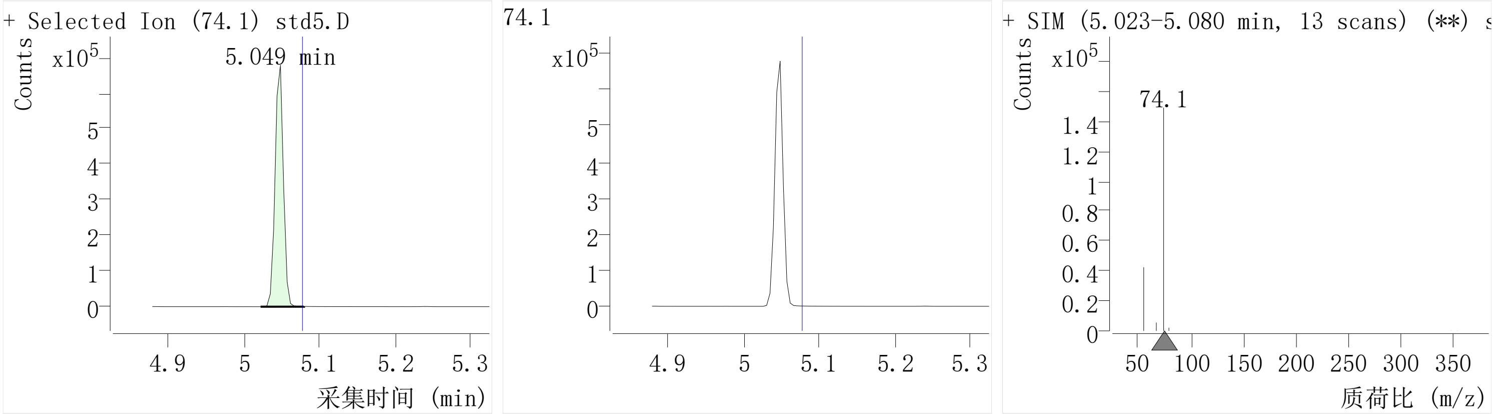

C13:0

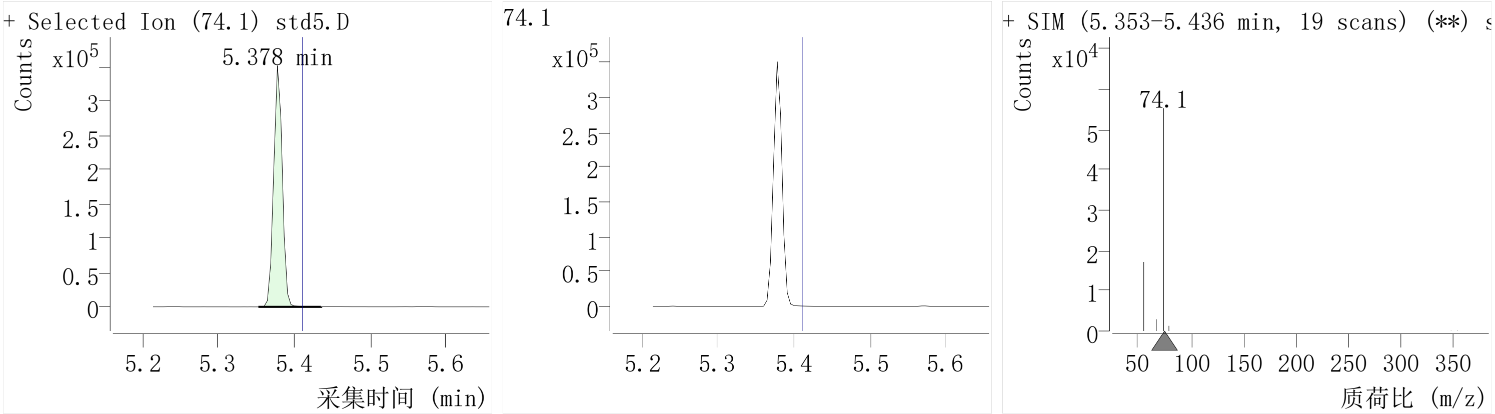

C14:0

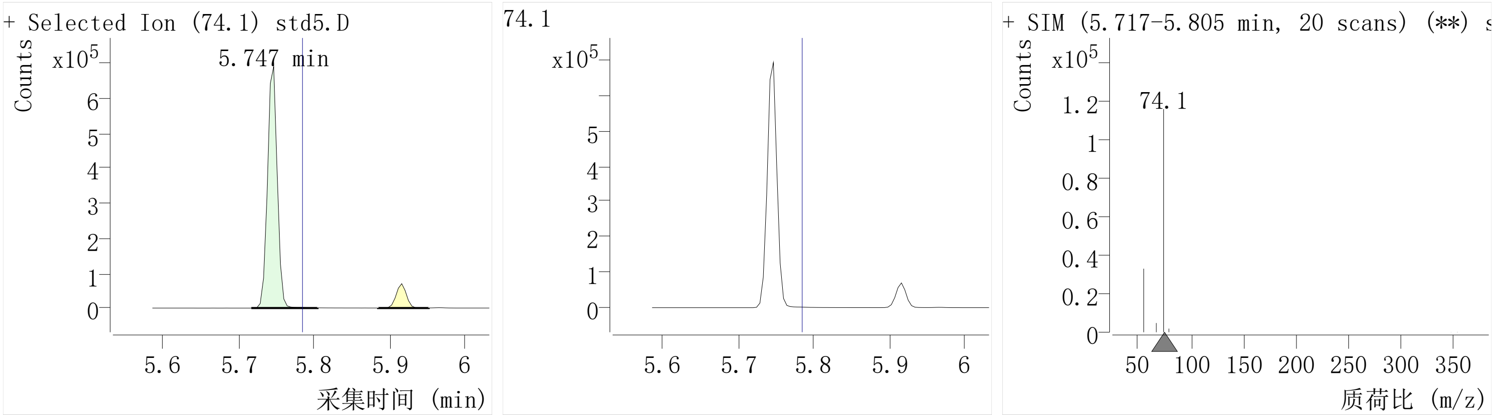

C14:1

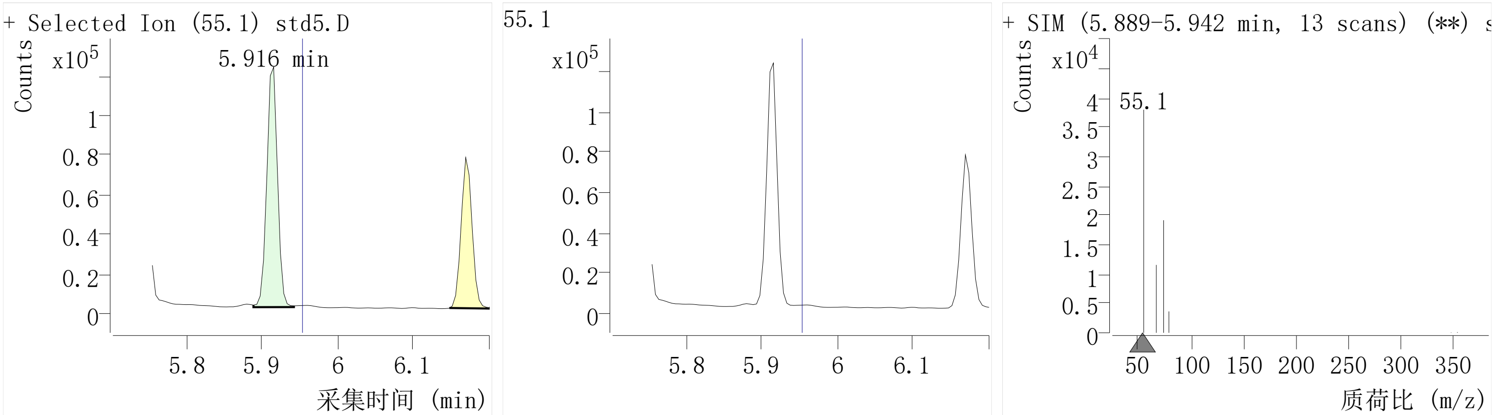

C15:0

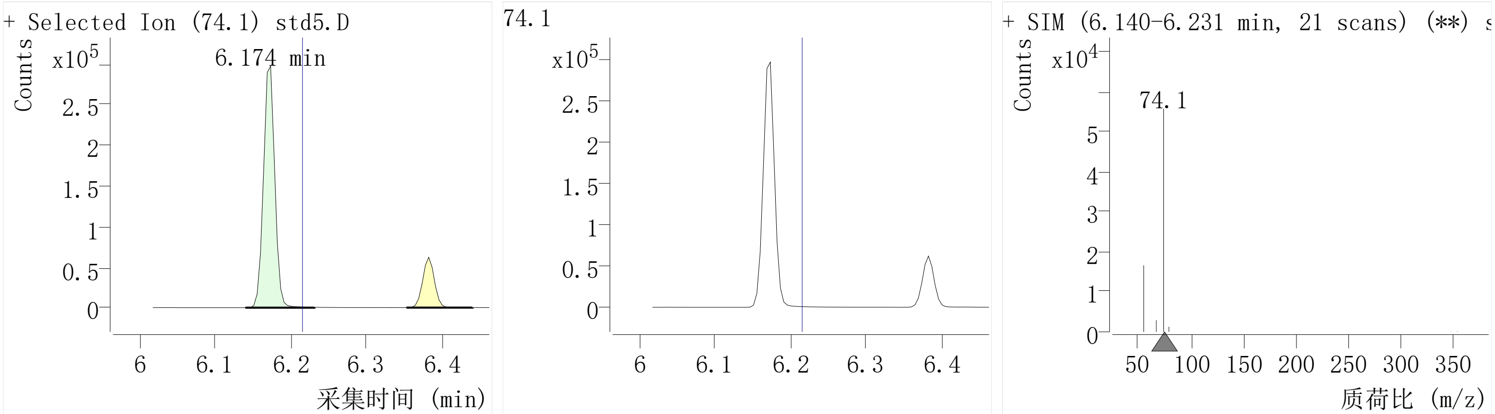

C15:1

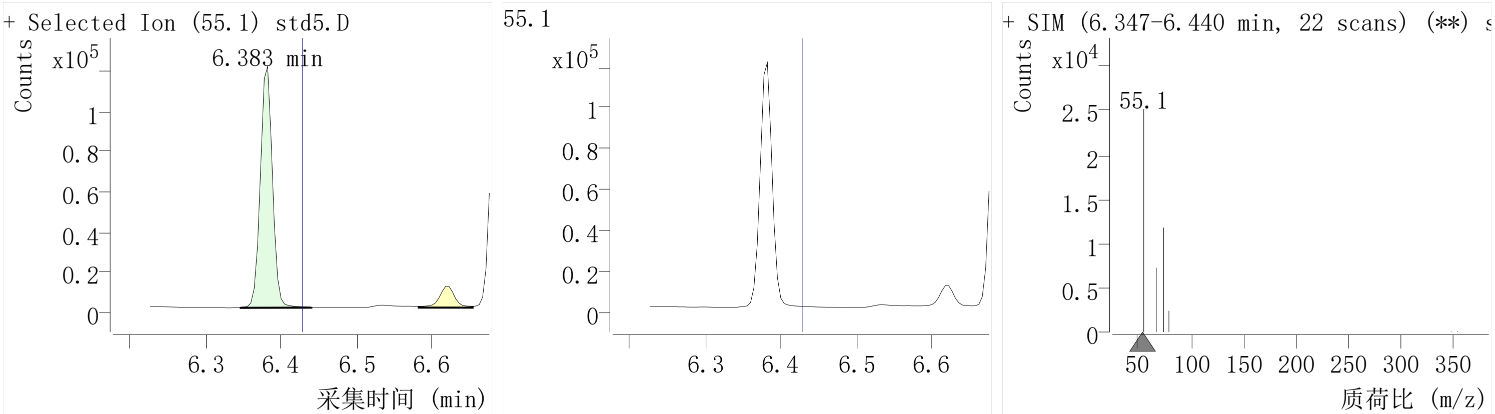

C16:0

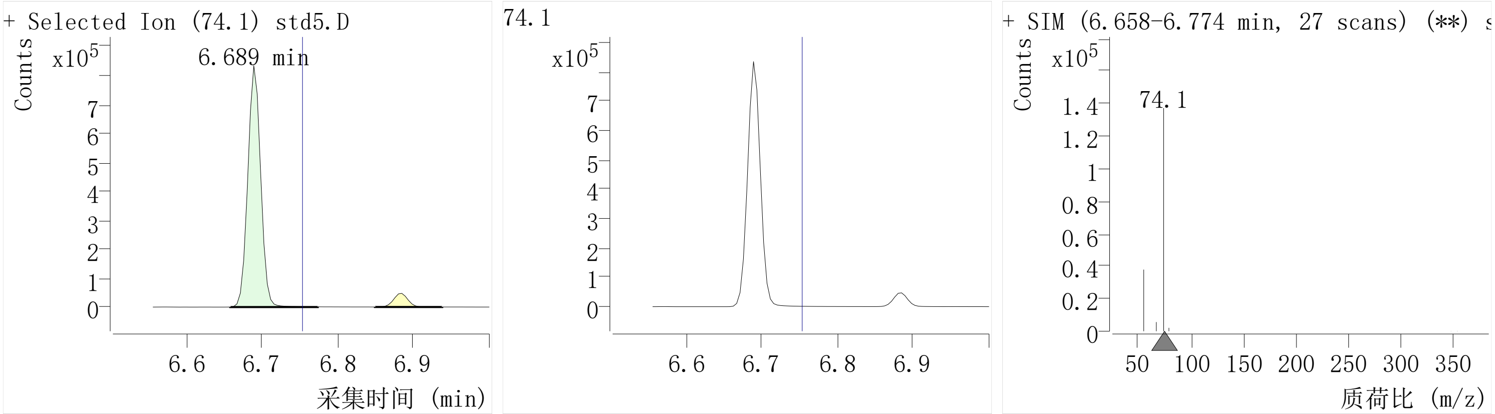

C16:1

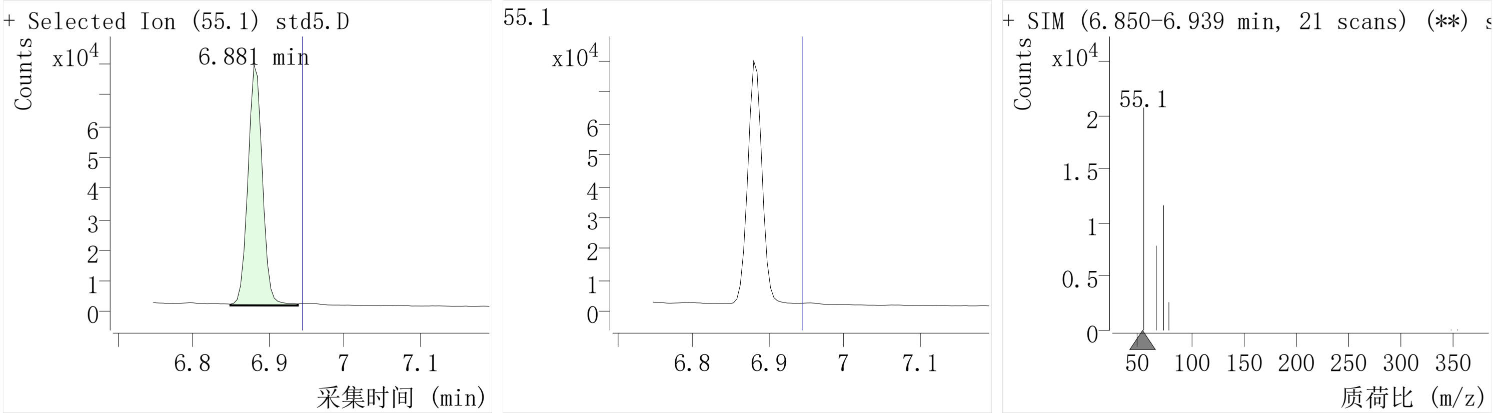

C17:0

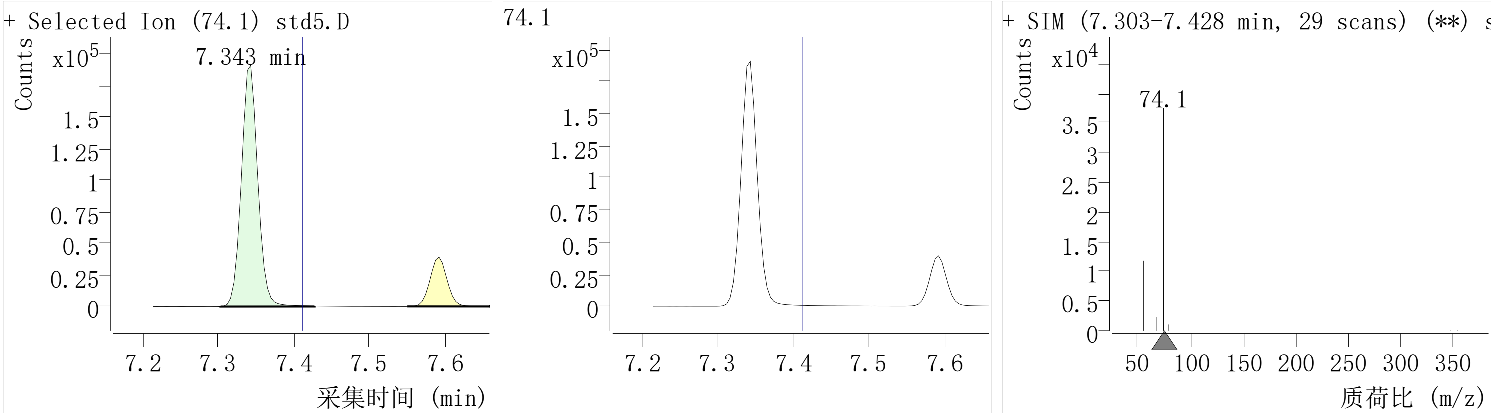

C17:1

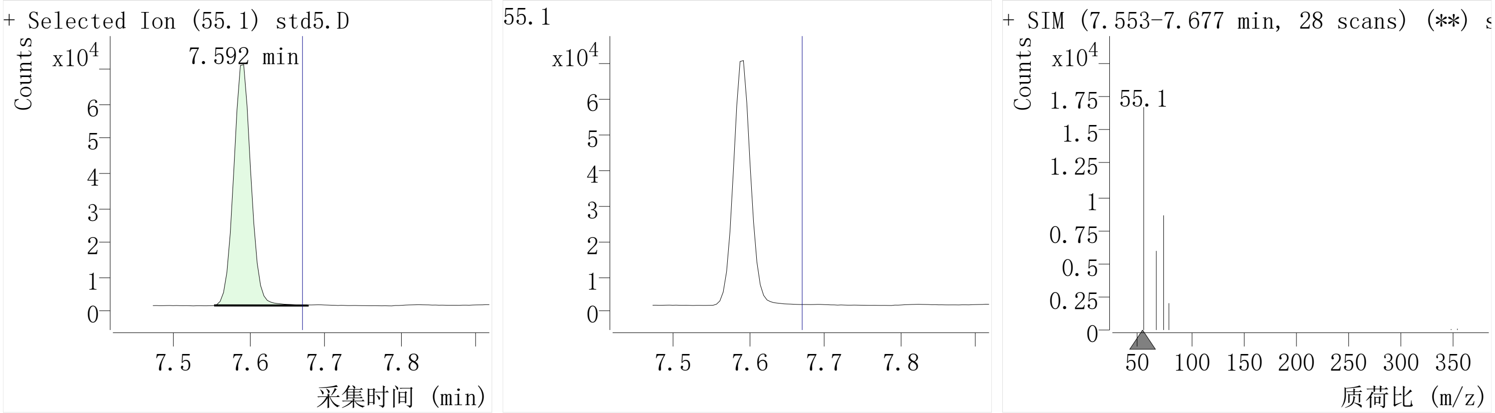

C18:0

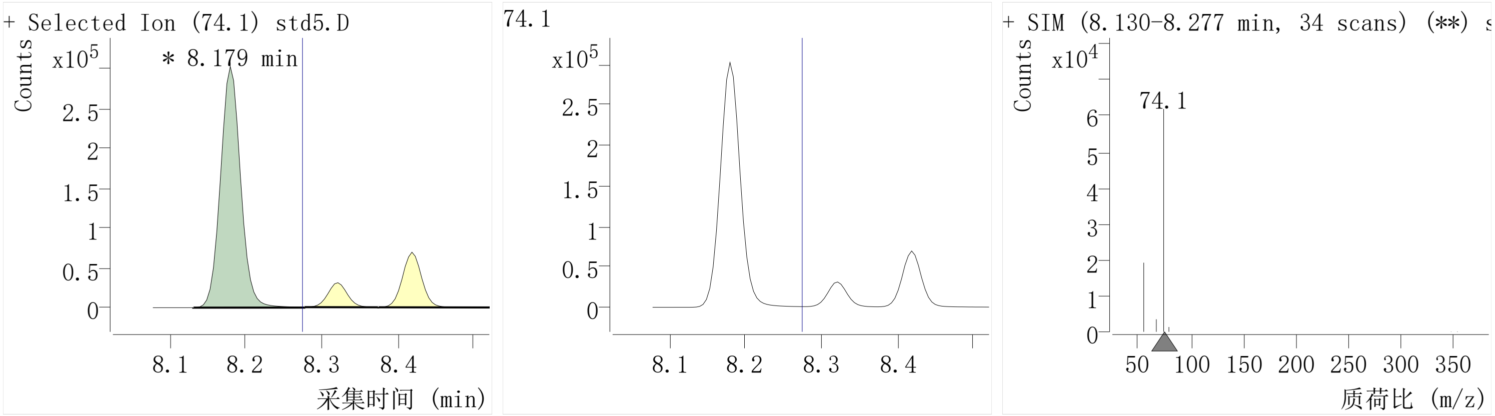

C18:1n9t

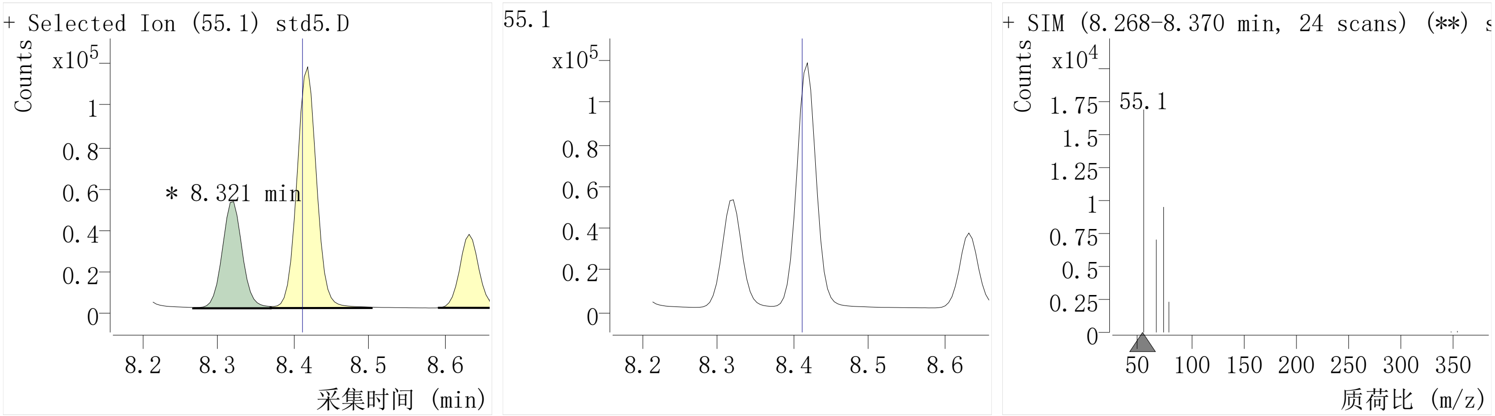

C18:1n9c

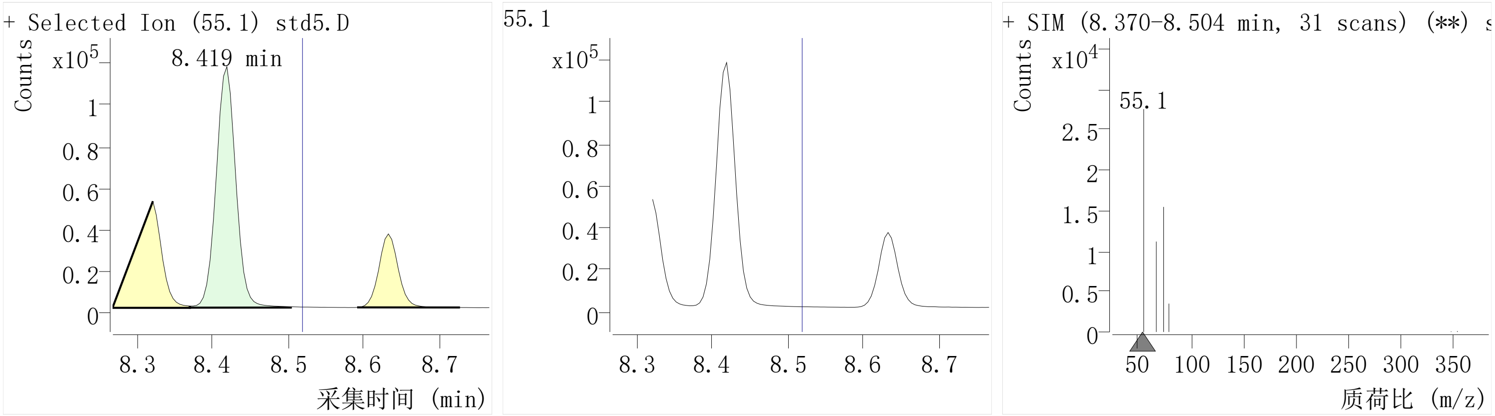

C18:2n6t

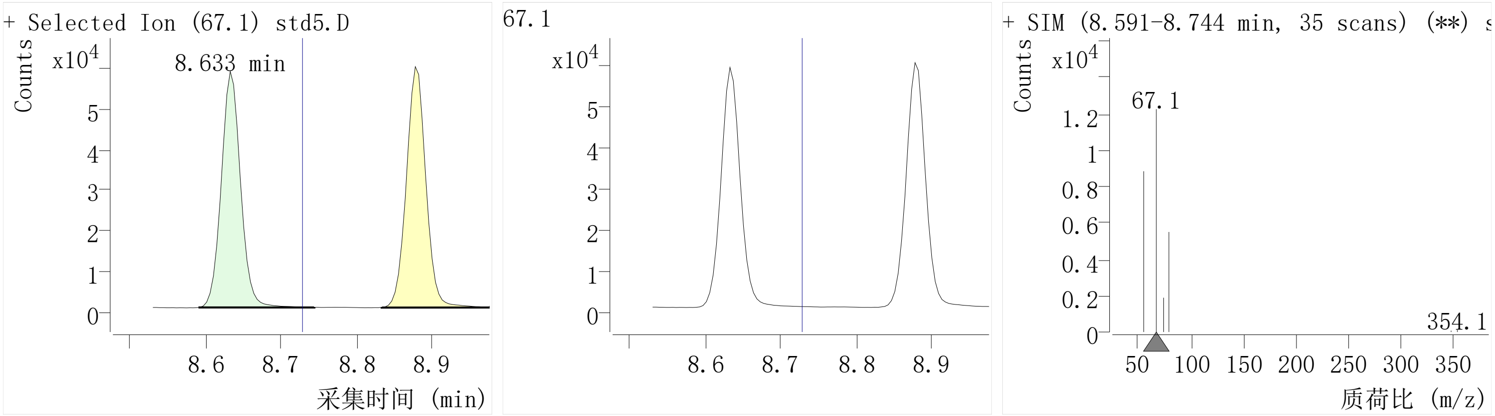

C18:2n6c

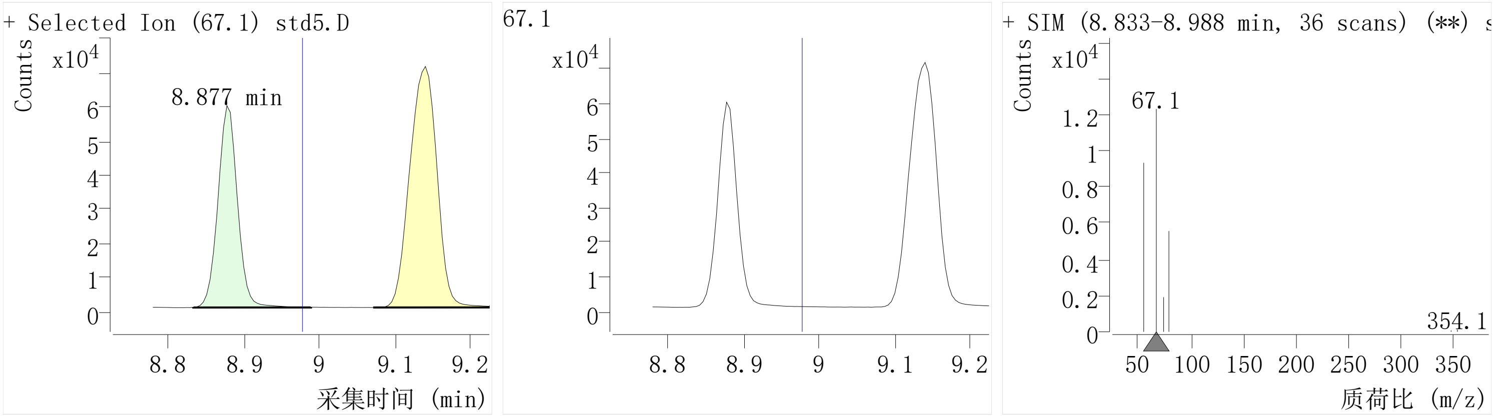

C18:3n6

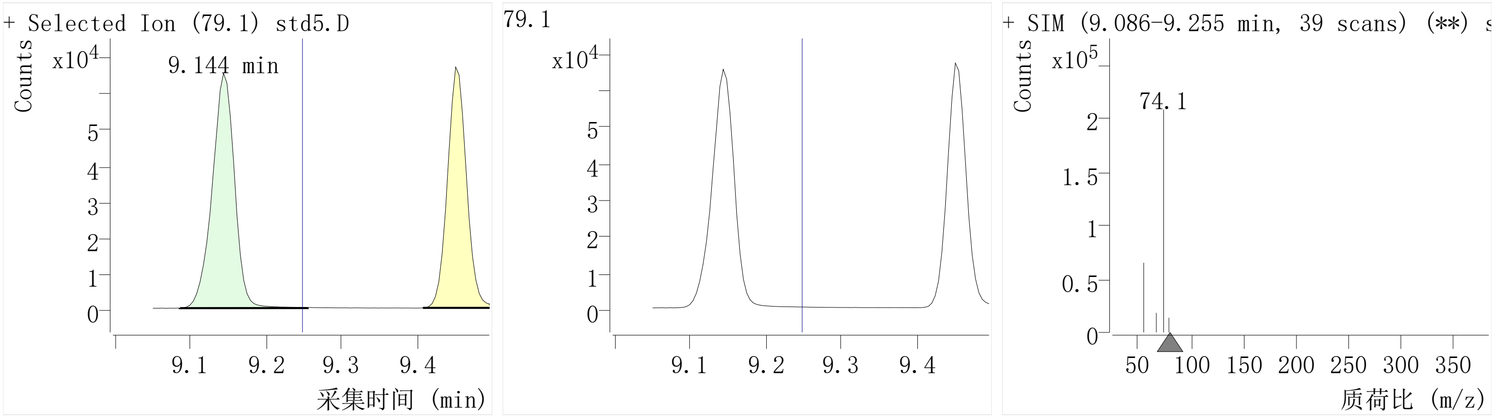

C18:3n3

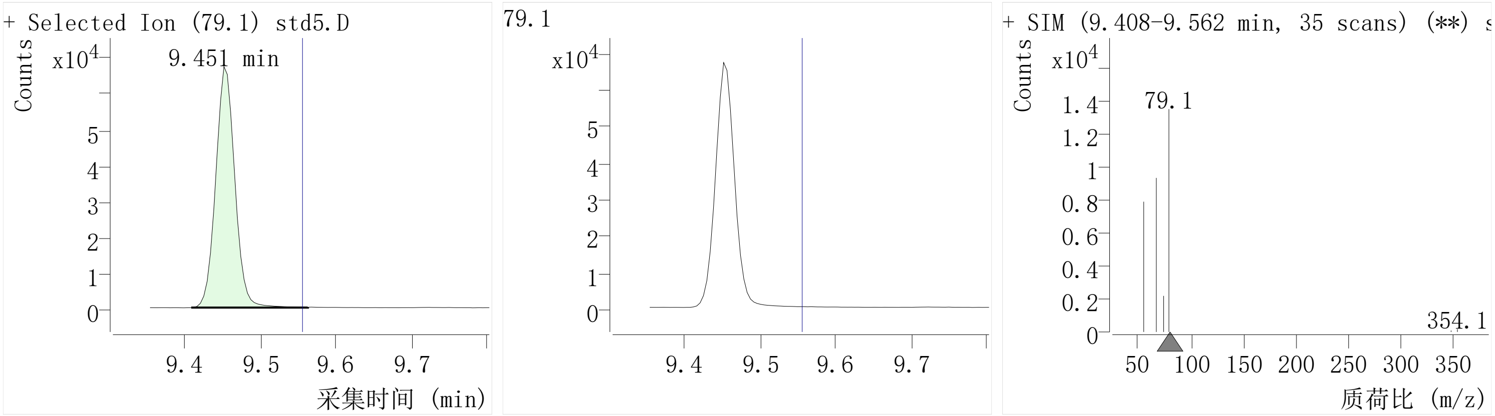

C20:0

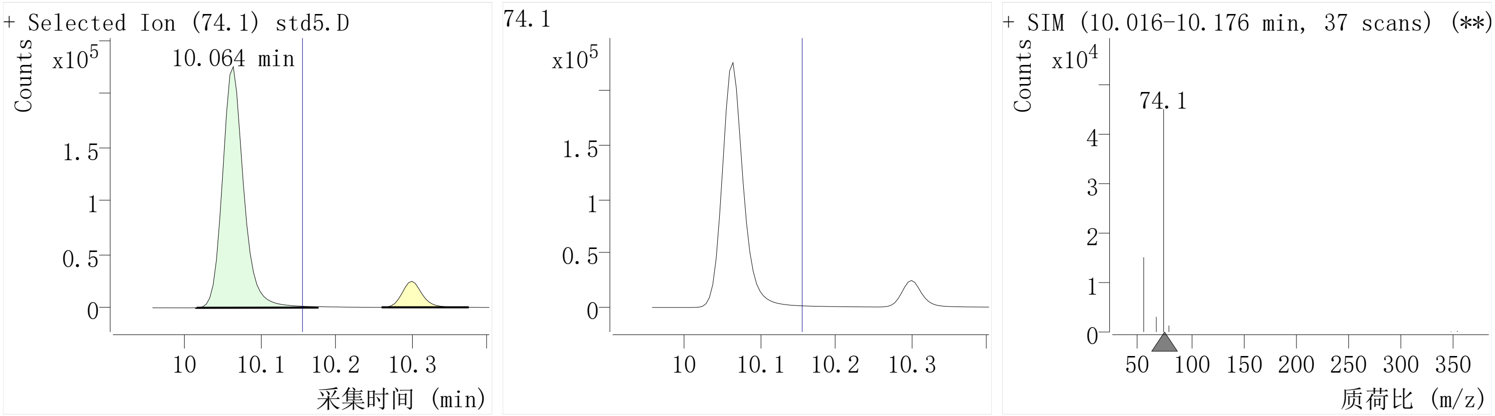

C20:1

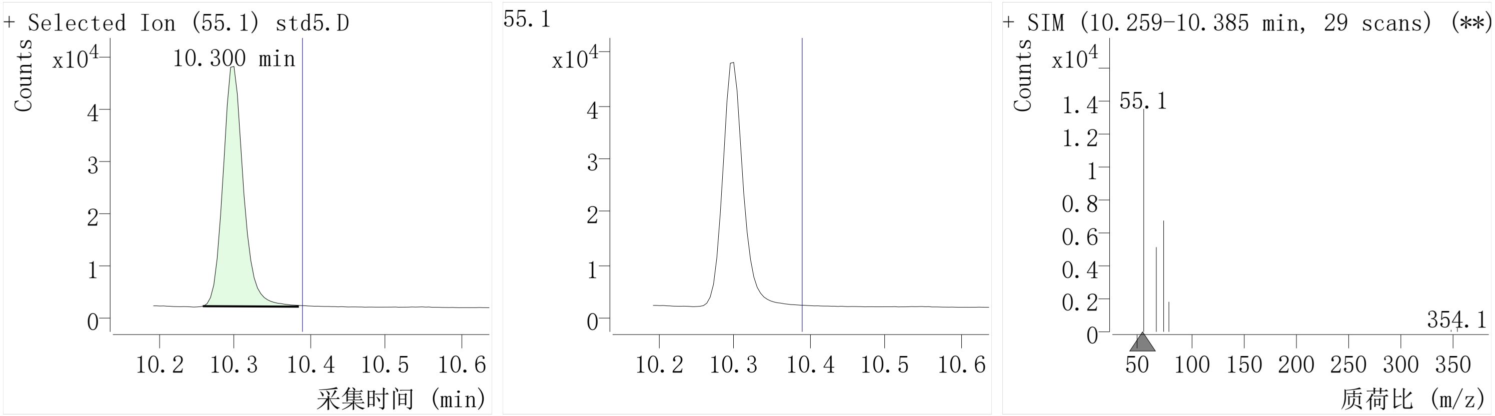

C20:2

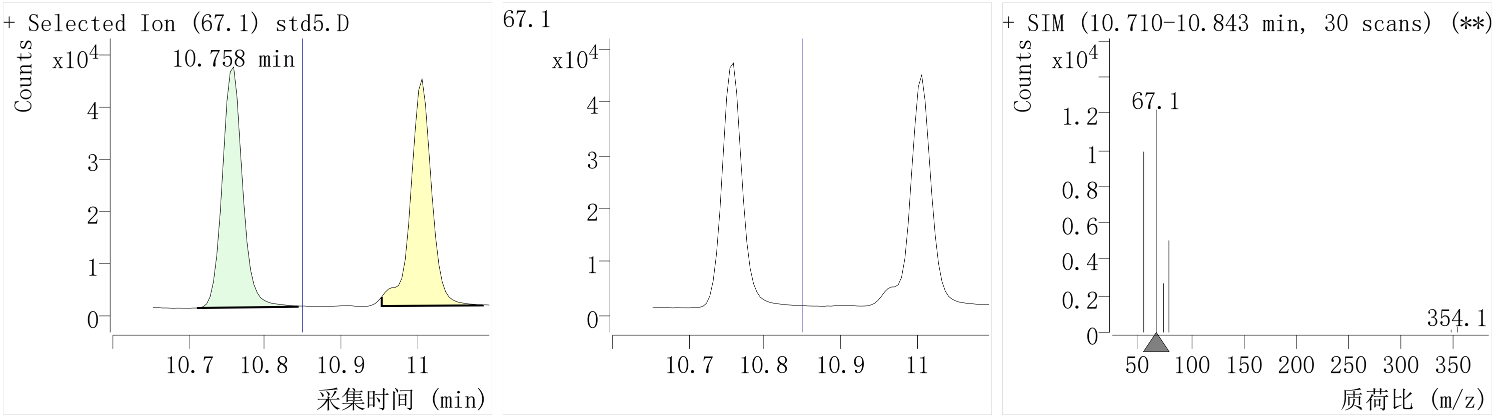

C21:0

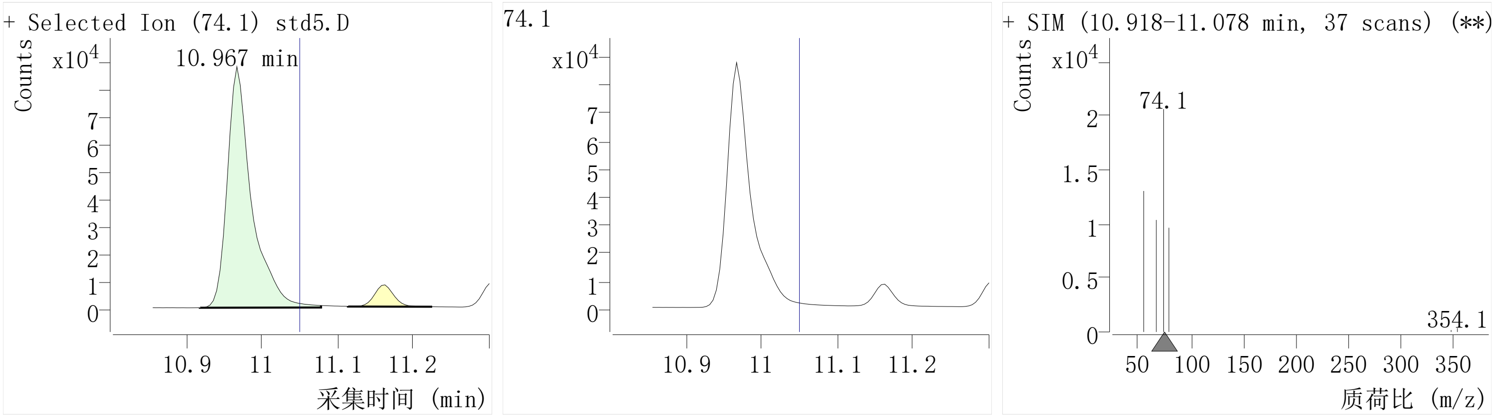

C20:3n6

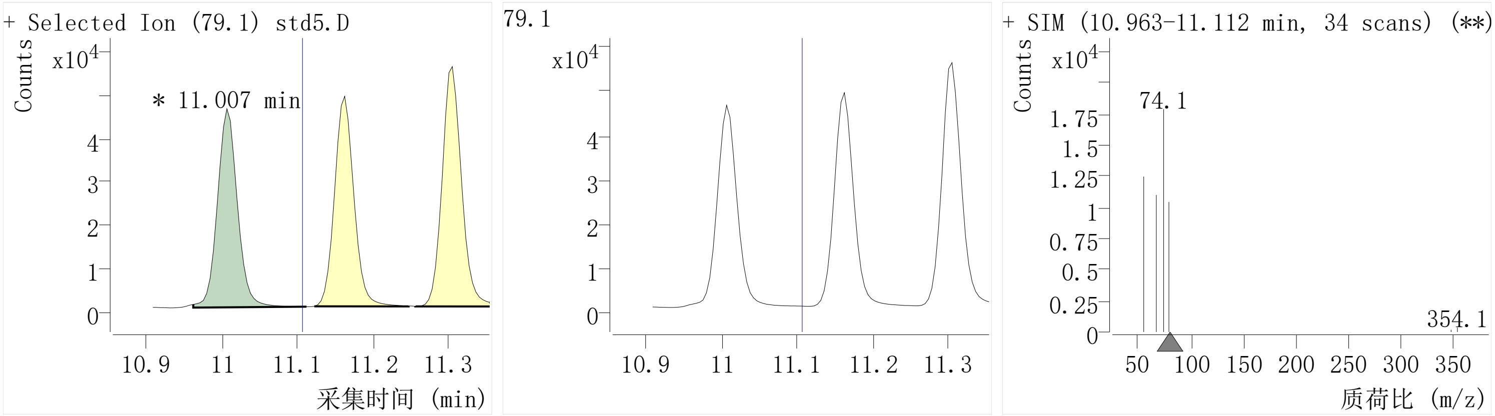

C20:4n6

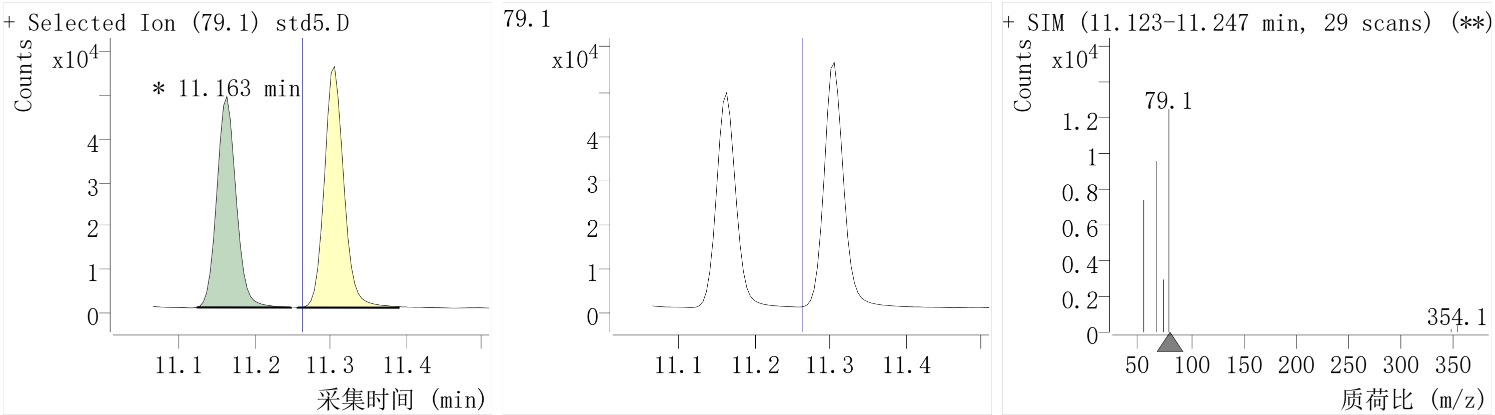

C20:3n3

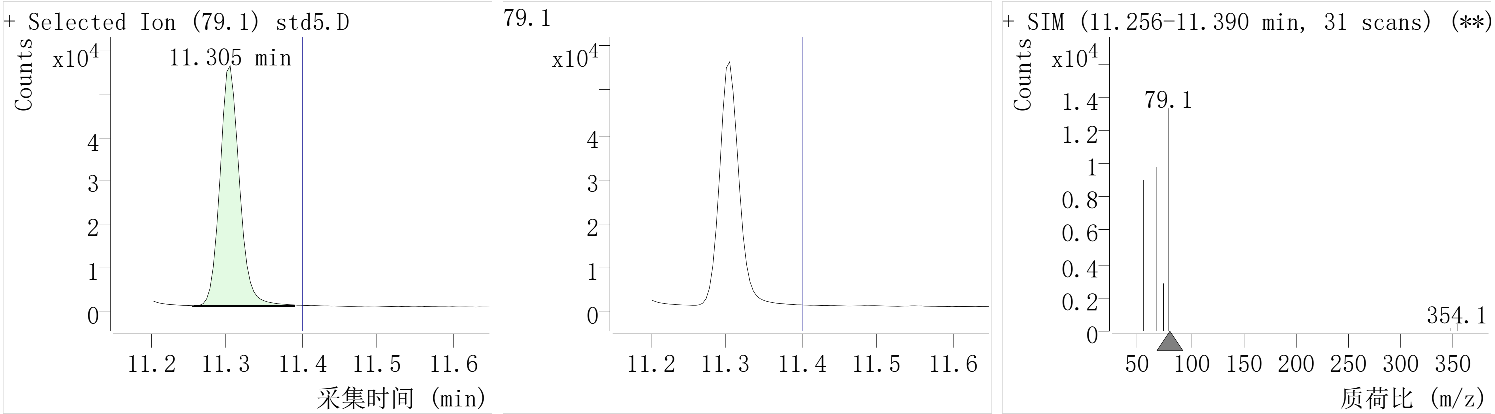

C20:5n3

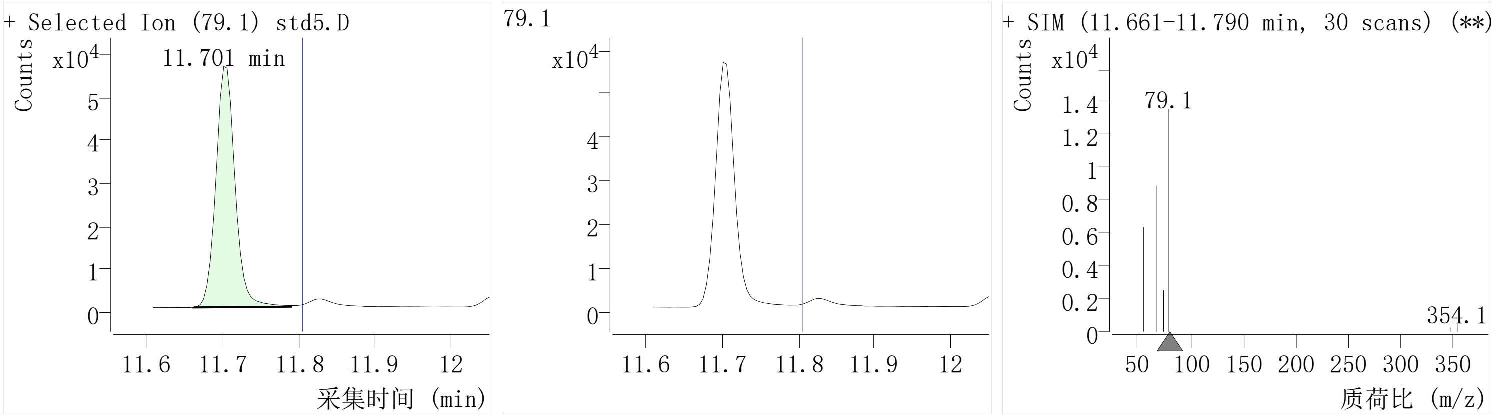

C22:0

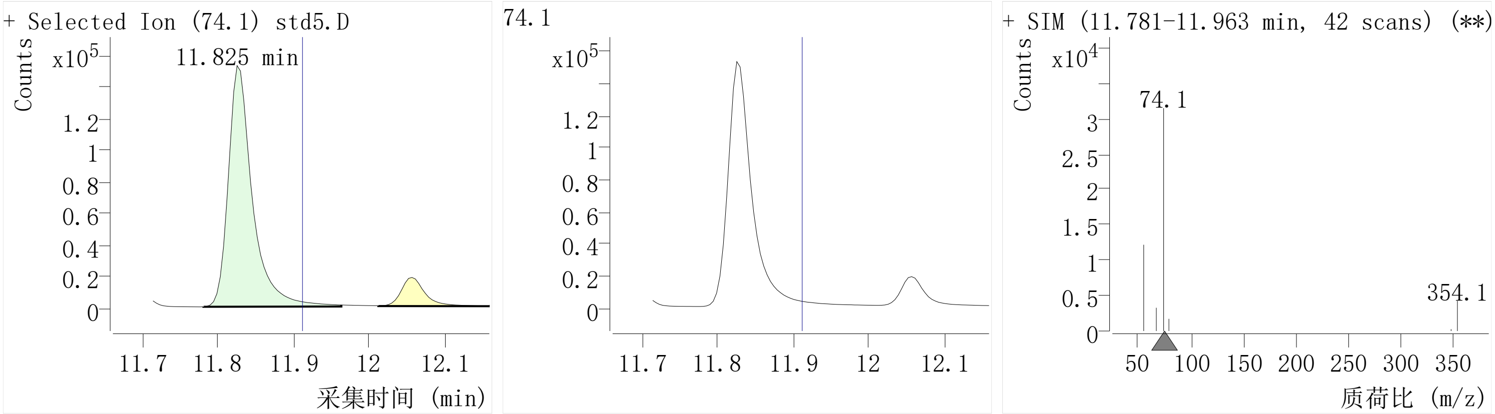

C22:1n9

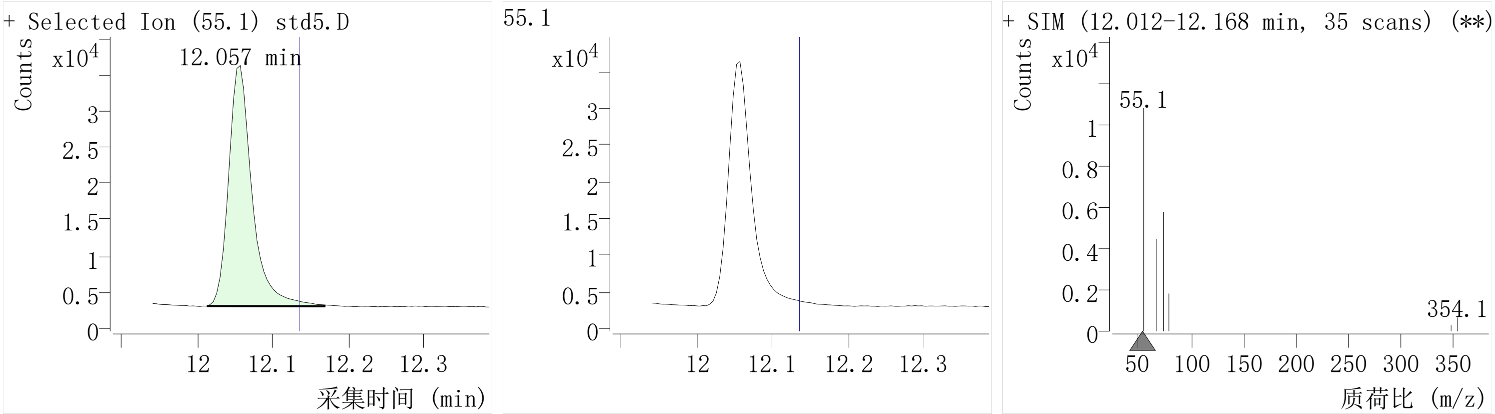

C22:2n6

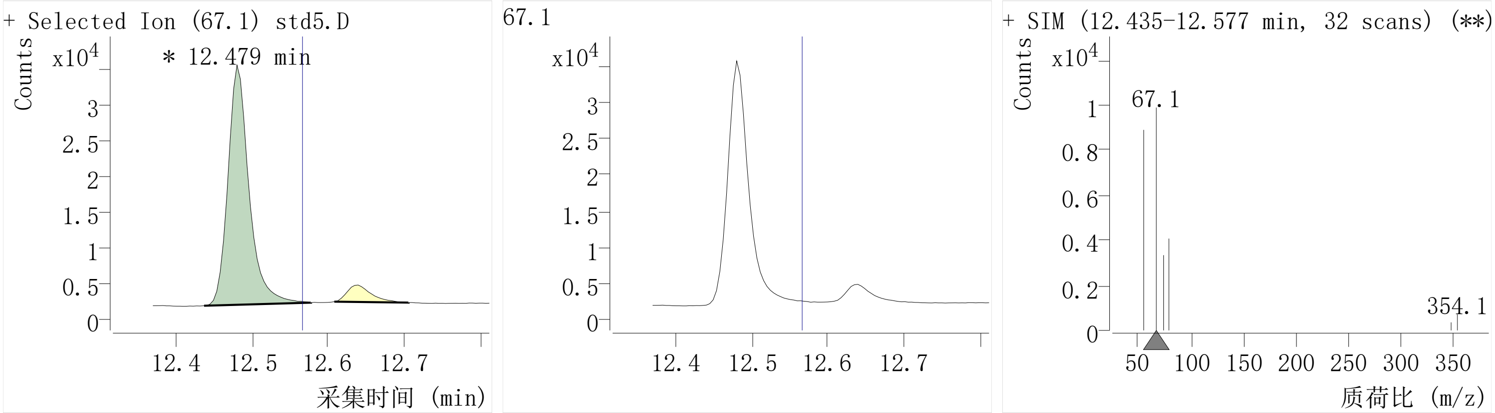

C23:0

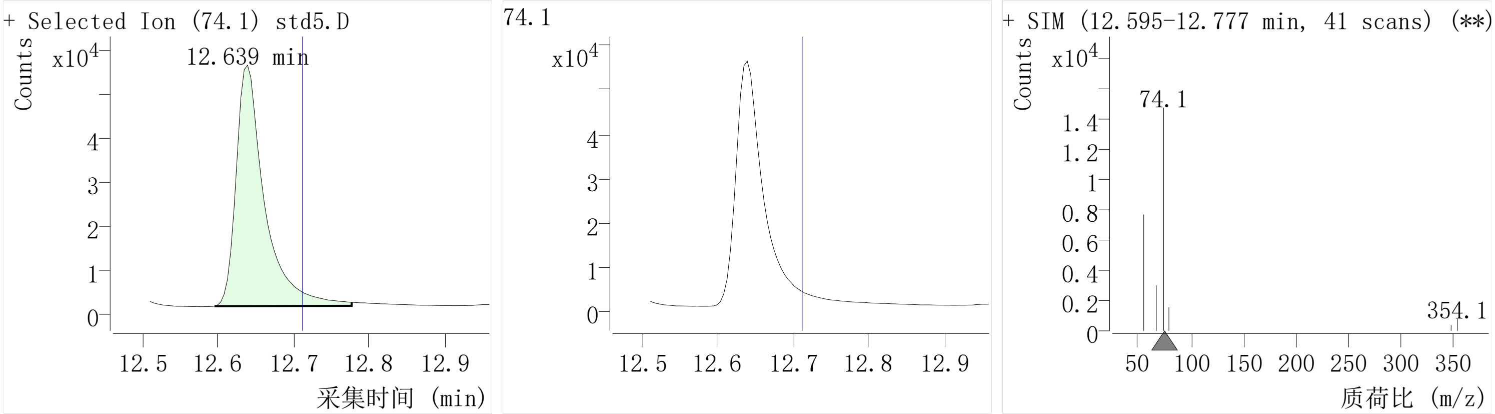

C24:0

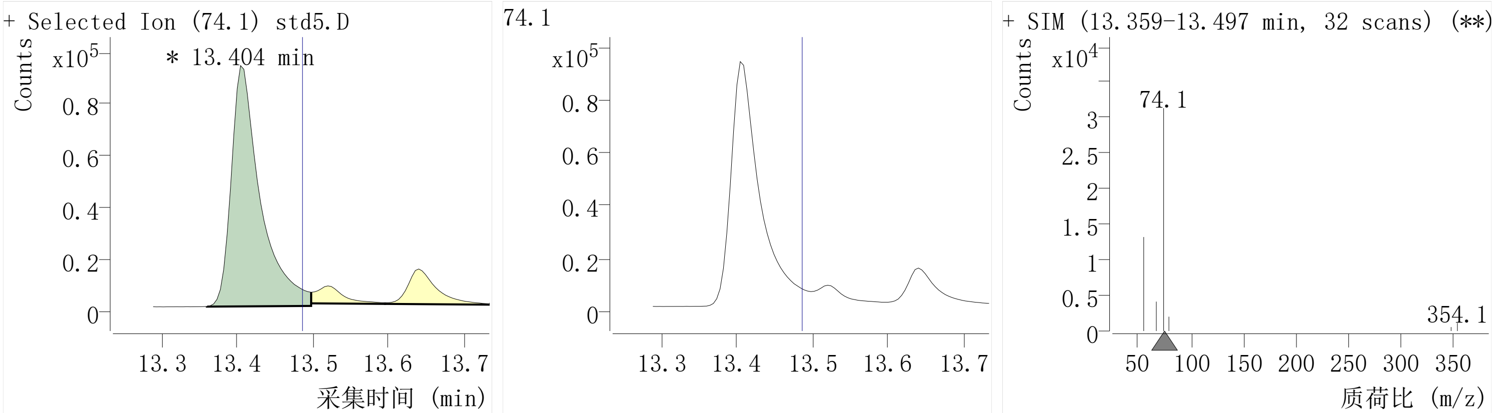

C22:6

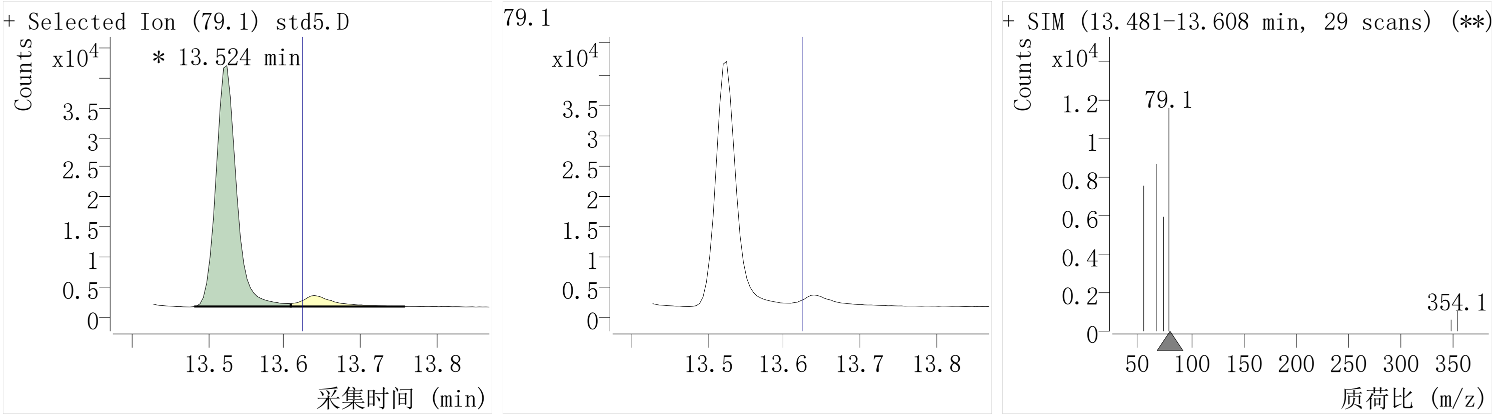
+ Selected Ion (79.1) std5.D
Counts
x10^4
* 13.524 min
3.5
3
2.5
2
1.5
1
0.5
0
13.5
13.6
13.7
13.8
采集时间 (min)
79.1
+ SIM (13.481-13.608 min, 29 scans) (**)
1.2
0.8
0.6
0.4
0.2
354.1
50
100
150
200
250
300
350
质荷比 (m/z)

C24:1

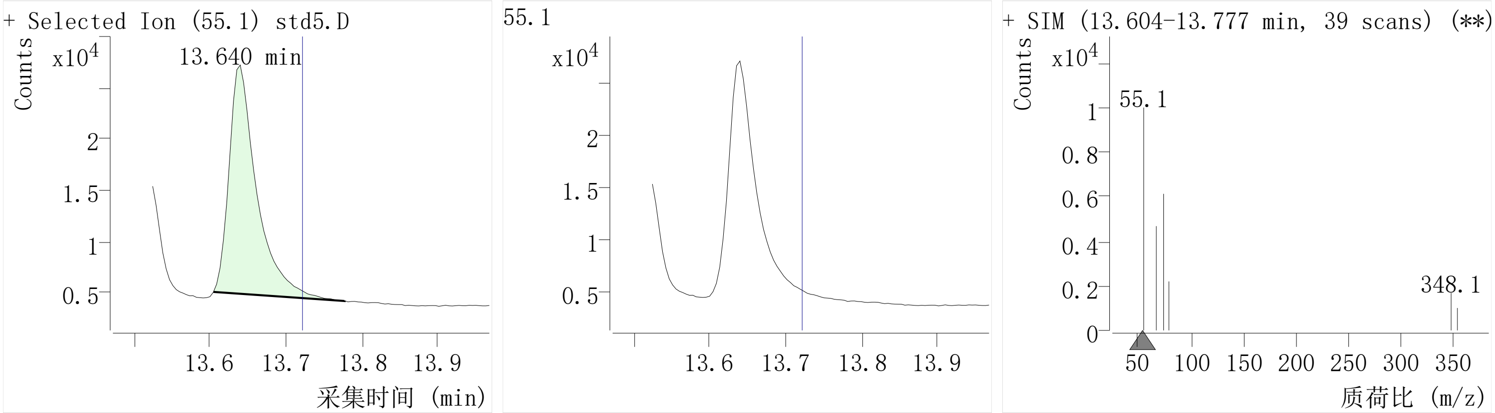
+ Selected Ion (55.1) std5.D
Counts
x10^4
13.640 min
2
1.5
1
0.5
13.6
13.7
13.8
13.9
采集时间 (min)
55.1
+ SIM (13.604-13.777 min, 39 scans) (**)
1
0.8
0.6
0.4
0.2
0
348.1
50
100
150
200
250
300
350
质荷比 (m/z)

定量分析完成报告

**批处理路径**	G:\GC-MS\HX250430-4-GCMS总脂肪酸靶向检测\HX250430-4\QuantResults\HX250430-4.batch.bin		
**分析时间**	2025/5/14 16:58	**分析员姓名**	DESKTOP-M3A0GPO\omics
**报告时间**	2025/5/16 14:53:06	**报告员姓名**	DESKTOP-M3A0GPO\omics
**最近校正更新**	2025/5/14 16:58	**批处理状态**	已处理
**定量批处理版本**	10.2	**定量报告版本**	10.2

**采集时间**	2025/5/8 19:09	**数据文件**	std6.D
**样品类型**	校正	**样品名称**	std6
**稀释**	1	**采集方法**	脂肪酸

样品色谱图

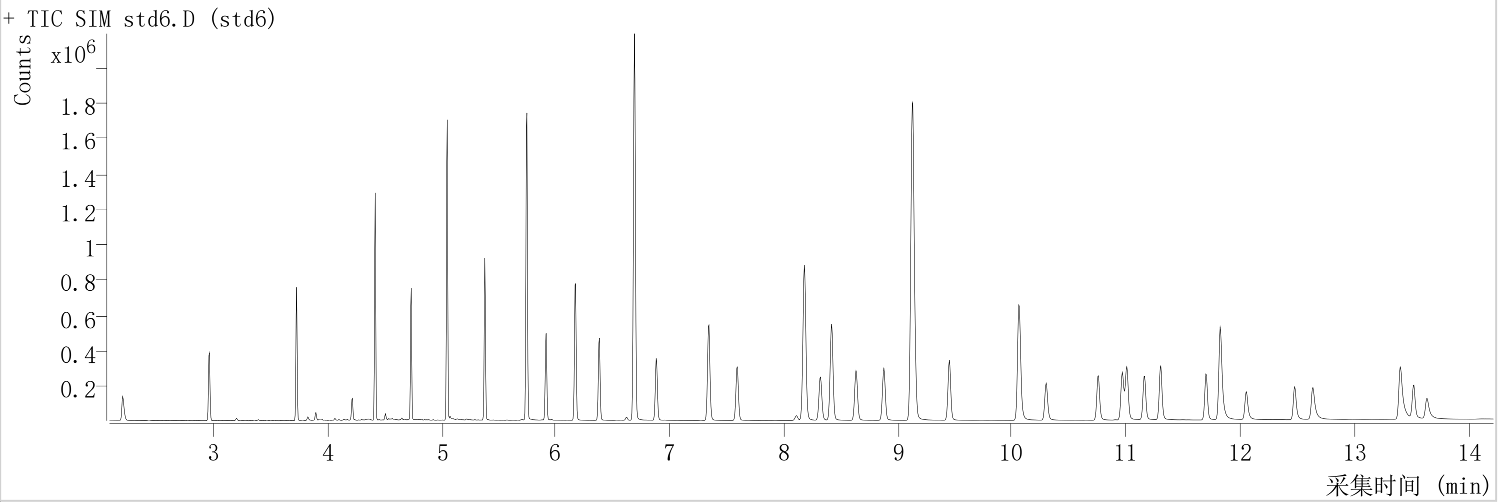

化合物	ISTD	RT	响应	ISTD 响应	响应比	最终浓度	单位
C4:0	C19:0	2.203	161600	2537627	0.0637	9.6846	ug/ml
C6:0	C19:0	2.963	302051	2537627	0.1190	8.9120	ug/ml
C8:0	C19:0	3.728	463239	2537627	0.1825	7.9535	ug/ml
C10:0	C19:0	4.417	721537	2537627	0.2843	8.6541	ug/ml
C11:0	C19:0	4.733	435048	2537627	0.1714	4.4077	ug/ml
C12:0	C19:0	5.049	1012197	2537627	0.3989	9.2644	ug/ml
C13:0	C19:0	5.378	558053	2537627	0.2199	4.6872	ug/ml
C14:0	C19:0	5.747	1247316	2537627	0.4915	9.4926	ug/ml
C14:1	C19:0	5.916	246253	2537627	0.0970	4.7880	ug/ml
C15:0	C19:0	6.174	635348	2537627	0.2504	4.8650	ug/ml
C15:1	C19:0	6.383	275315	2537627	0.1085	5.2608	ug/ml
C16:0	C19:0	6.689	2036671	2537627	0.8026	12.8648	ug/ml
C16:1	C19:0	6.881	217198	2537627	0.0856	5.1422	ug/ml
C17:0	C19:0	7.343	621619	2537627	0.2450	5.1106	ug/ml
C17:1	C19:0	7.588	240779	2537627	0.0949	5.0814	ug/ml
C18:0	C19:0	8.179	1227136	2537627	0.4836	9.7654	ug/ml
C18:1n9t	C19:0	8.317	214075	2537627	0.0844	4.9764	ug/ml
C18:1n9c	C19:0	8.419	465395	2537627	0.1834	10.1427	ug/ml
C18:2n6t	C19:0	8.633	233418	2537627	0.0920	5.0612	ug/ml
C18:2n6c	C19:0	8.877	236172	2537627	0.0931	4.6152	ug/ml
C18:3n6	C19:0	9.144	263826	2537627	0.1040	5.1221	ug/ml
C18:3n3	C19:0	9.451	263848	2537627	0.1040	5.1631	ug/ml
C20:0	C19:0	10.060	992176	2537627	0.3910	9.8963	ug/ml
C20:1	C19:0	10.300	197112	2537627	0.0777	4.8266	ug/ml
C20:2	C19:0	10.758	197958	2537627	0.0780	4.8217	ug/ml
C21:0	C19:0	10.967	443818	2537627	0.1749	4.8115	ug/ml
C20:3n6	C19:0	11.007	193575	2537627	0.0763	4.8619	ug/ml
C20:4n6	C19:0	11.163	199708	2537627	0.0787	4.9426	ug/ml
C20:3n3	C19:0	11.305	231764	2537627	0.0913	4.8260	ug/ml
C20:5n3	C19:0	11.701	224693	2537627	0.0885	4.9288	ug/ml

化合物	ISTD	RT	响应	ISTD 响应	响应比	最终浓度	单位
C22:0	C19:0	11.825	757374	2537627	0.2985	9.6836	ug/ml
C22:1n9	C19:0	12.056	162904	2537627	0.0642	4.7377	ug/ml
C22:2n6	C19:0	12.479	151778	2537627	0.0598	4.7636	ug/ml
C23:0	C19:0	12.639	317103	2537627	0.1250	4.8057	ug/ml
C24:0	C19:0	13.404	553595	2537627	0.2182	9.8434	ug/ml
C22:6	C19:0	13.524	175344	2537627	0.0691	5.1095	ug/ml
C24:1	C19:0	13.635	124624	2537627	0.0491	4.3263	ug/ml

C4:0

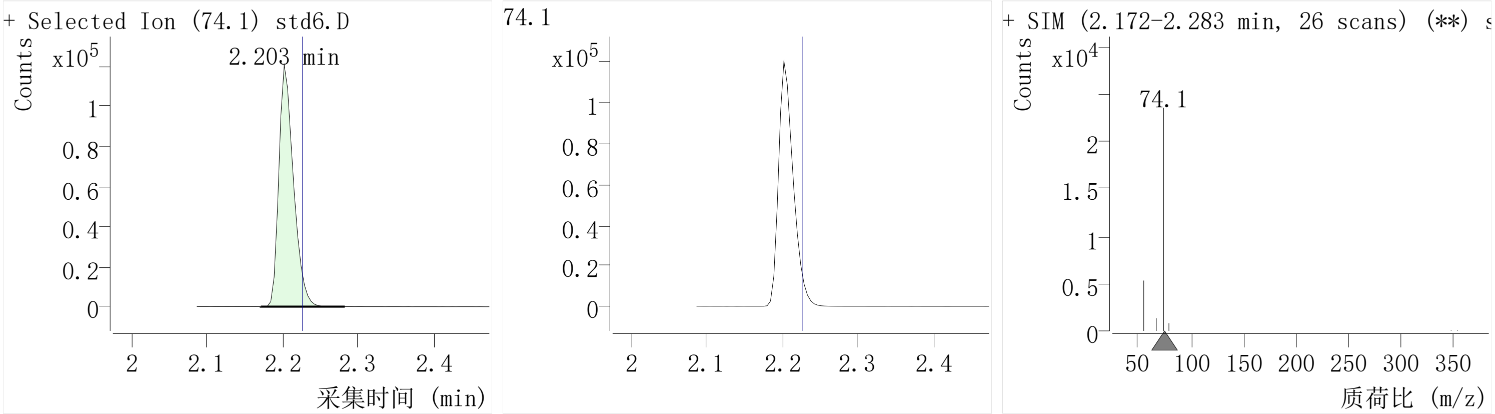

C6:0

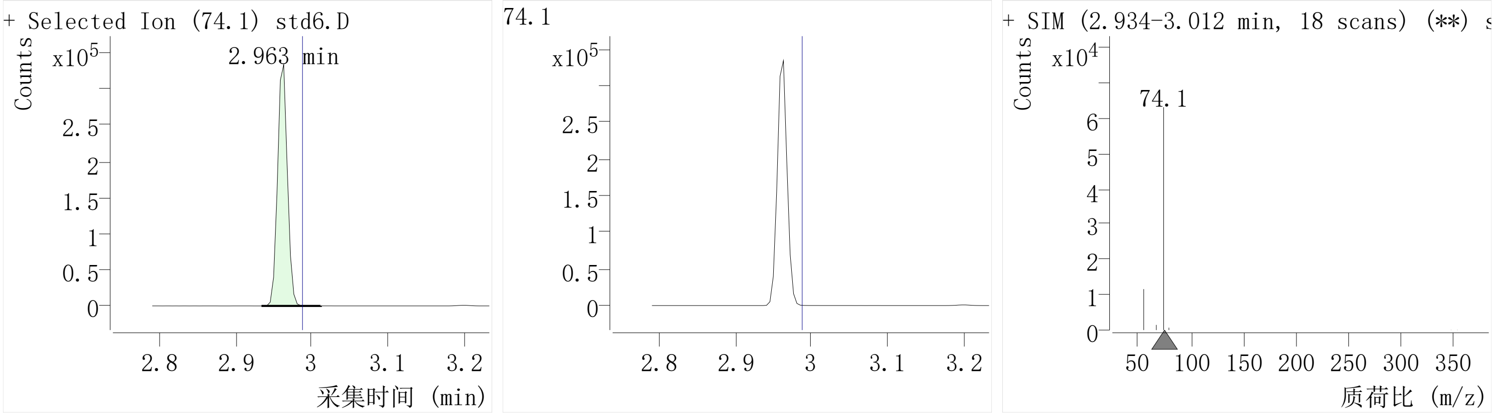

C8:0

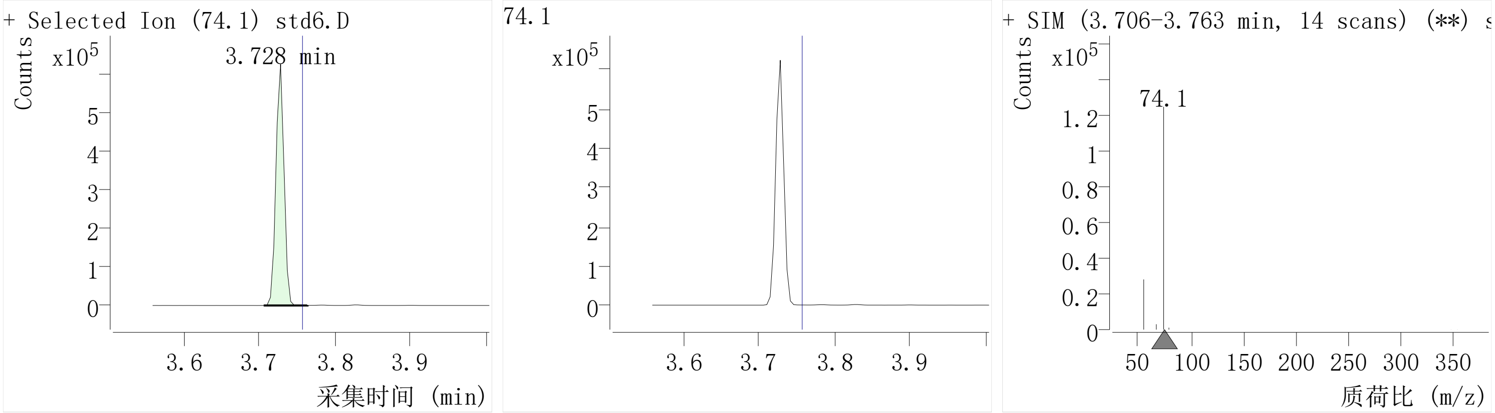

C10:0

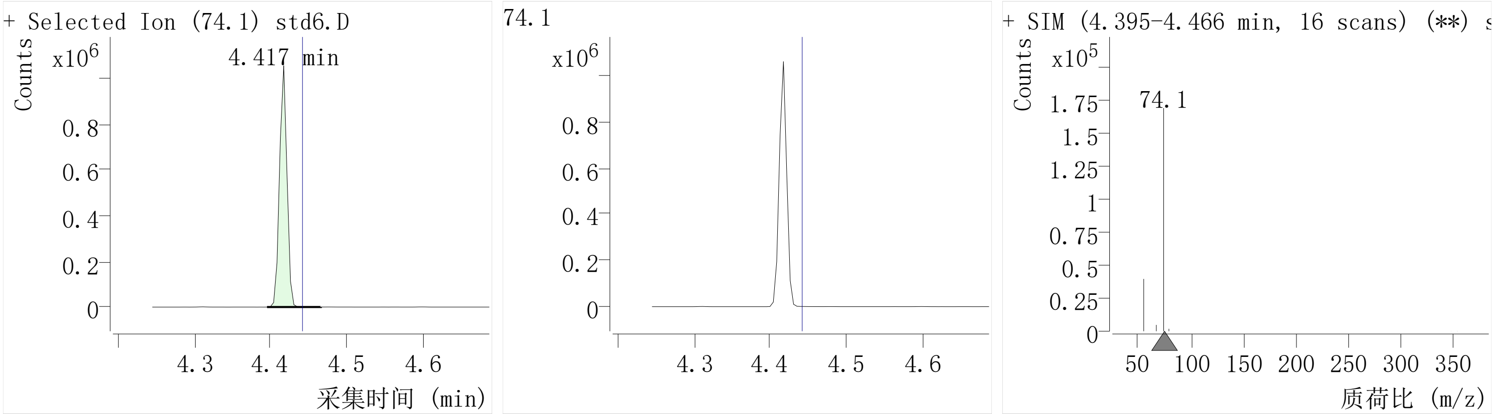

C11:0

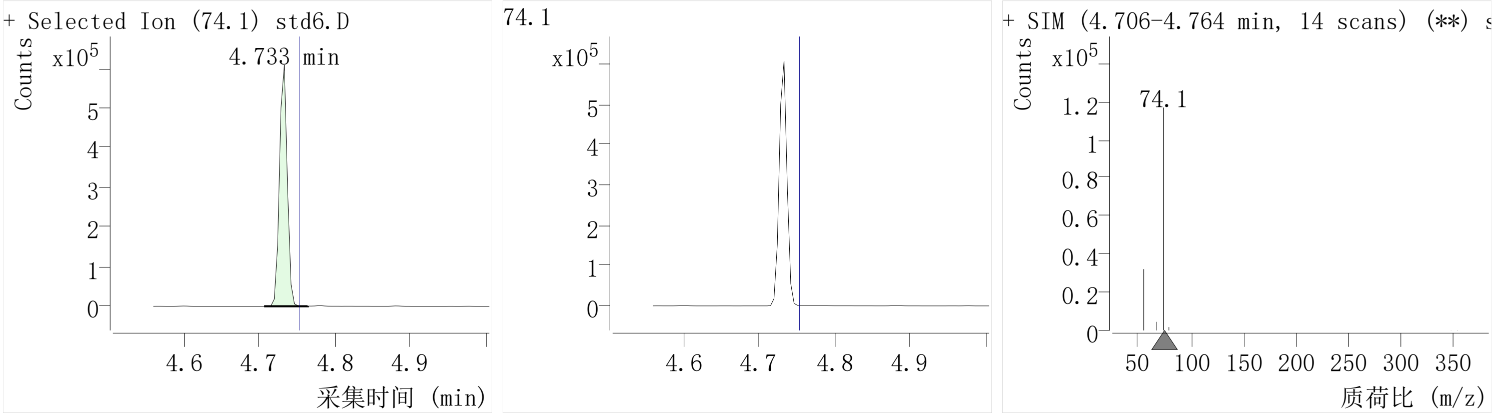

C12:0

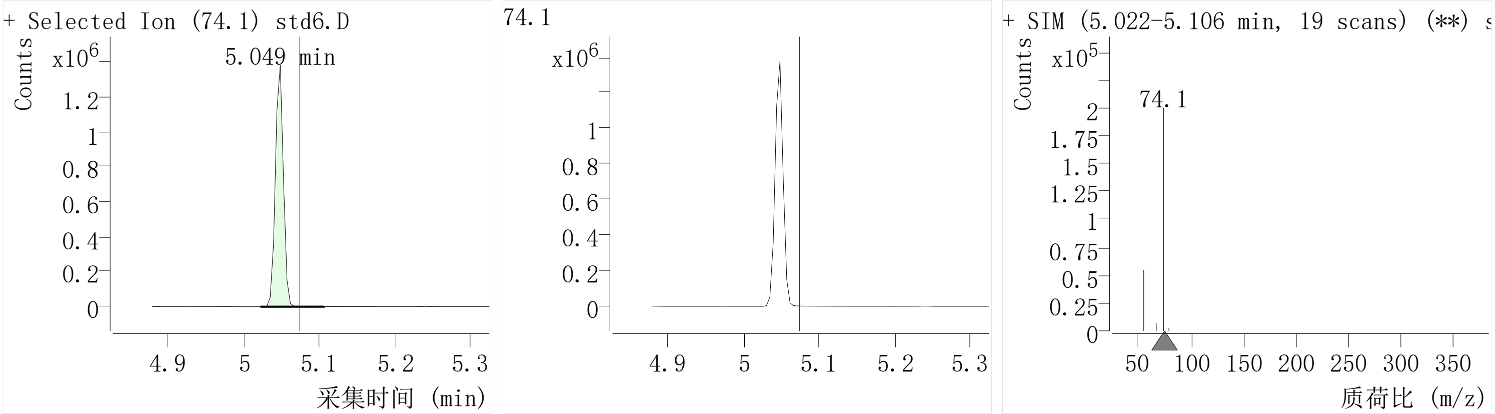

C13:0

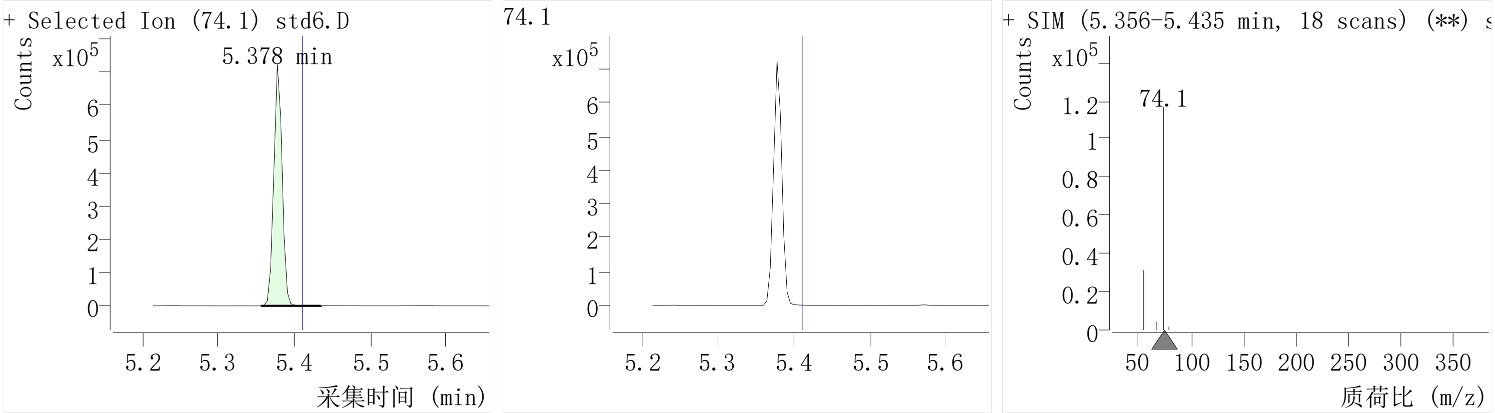

C14:0

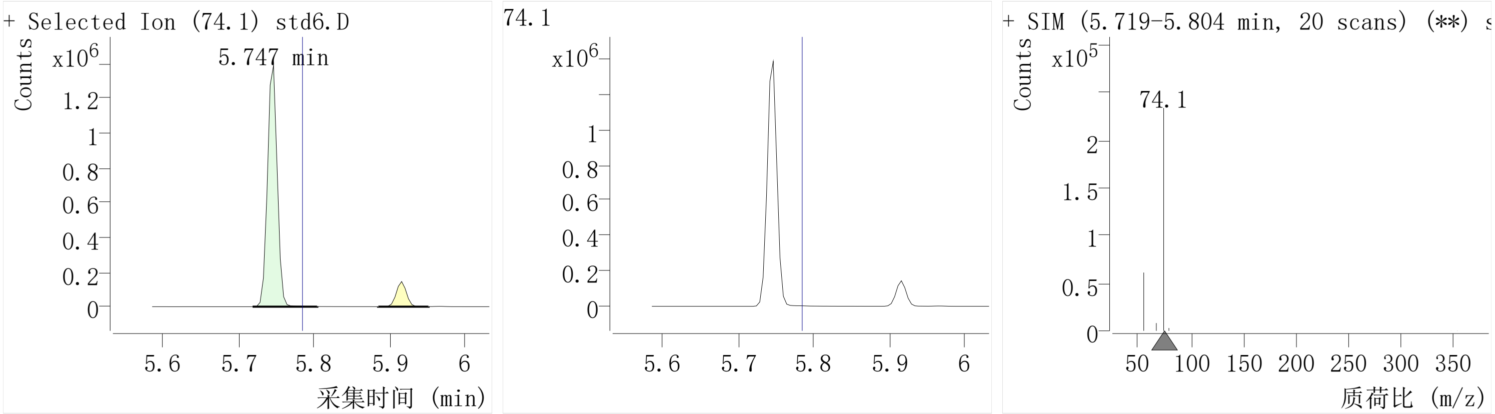

C14:1

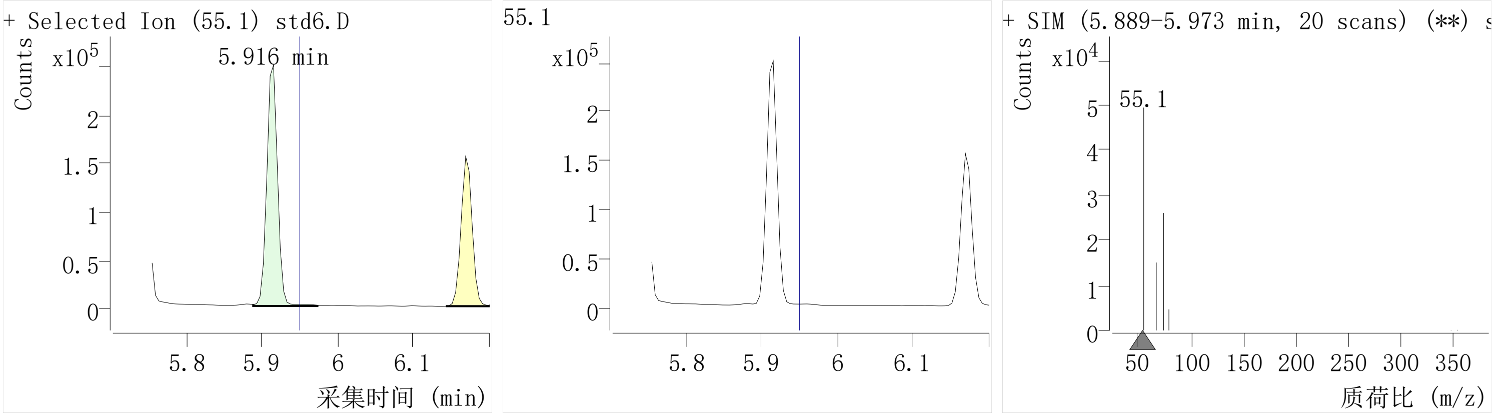

C15:0

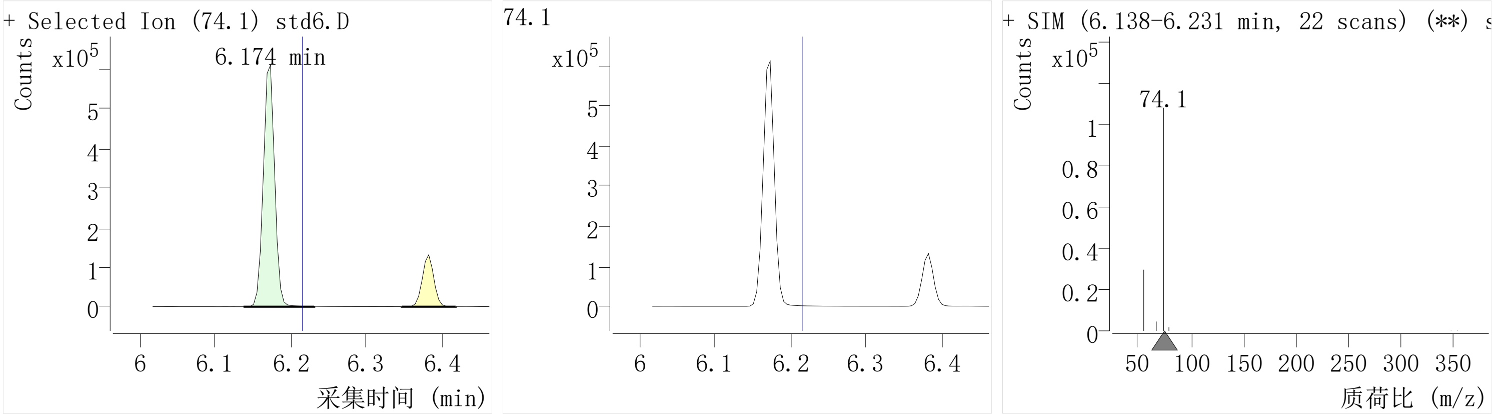

C15:1

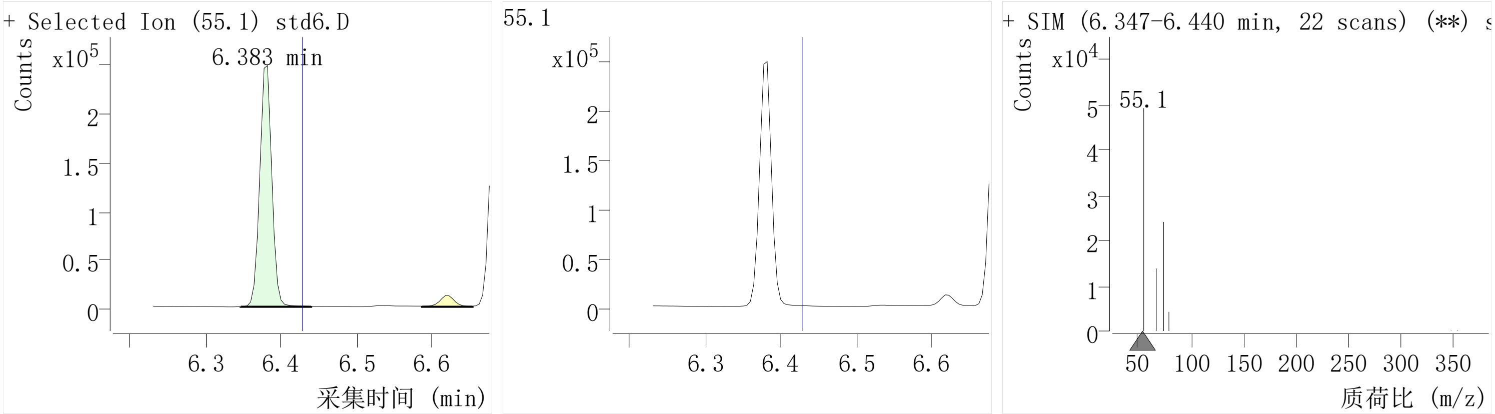

C16:0

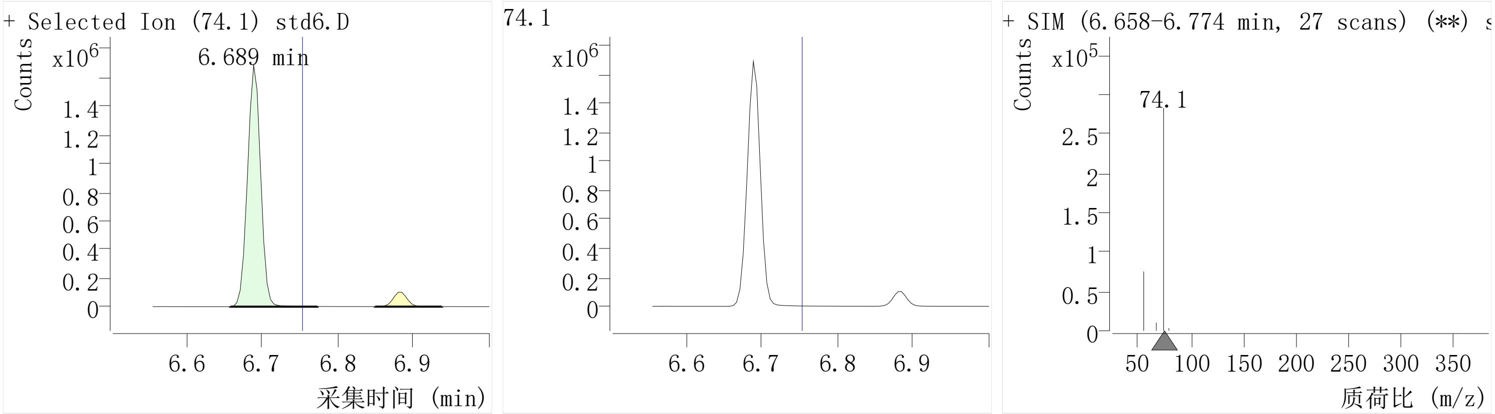

C16:1

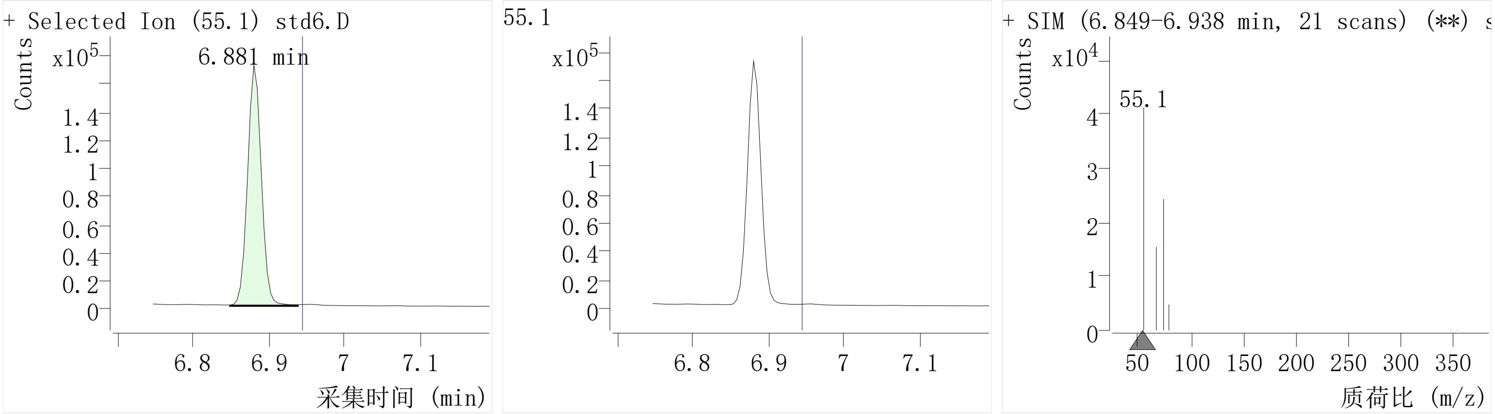

C17:0

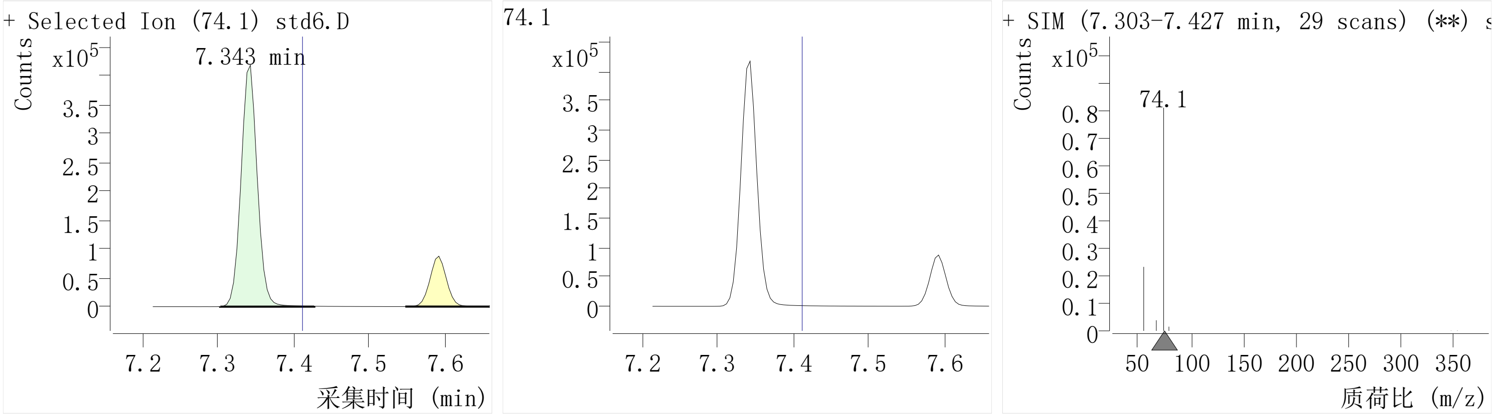

C17:1

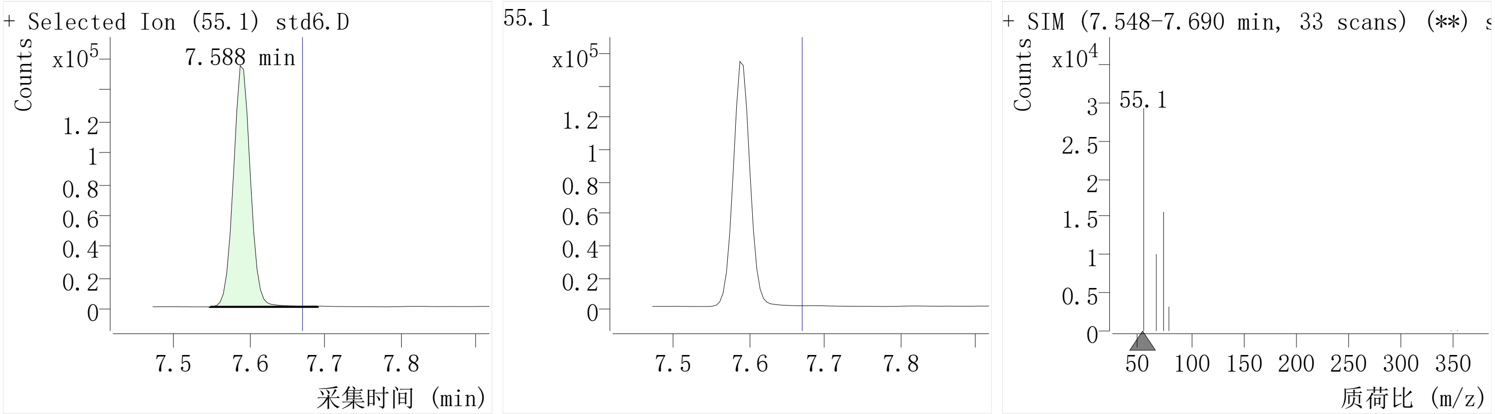

C18:0

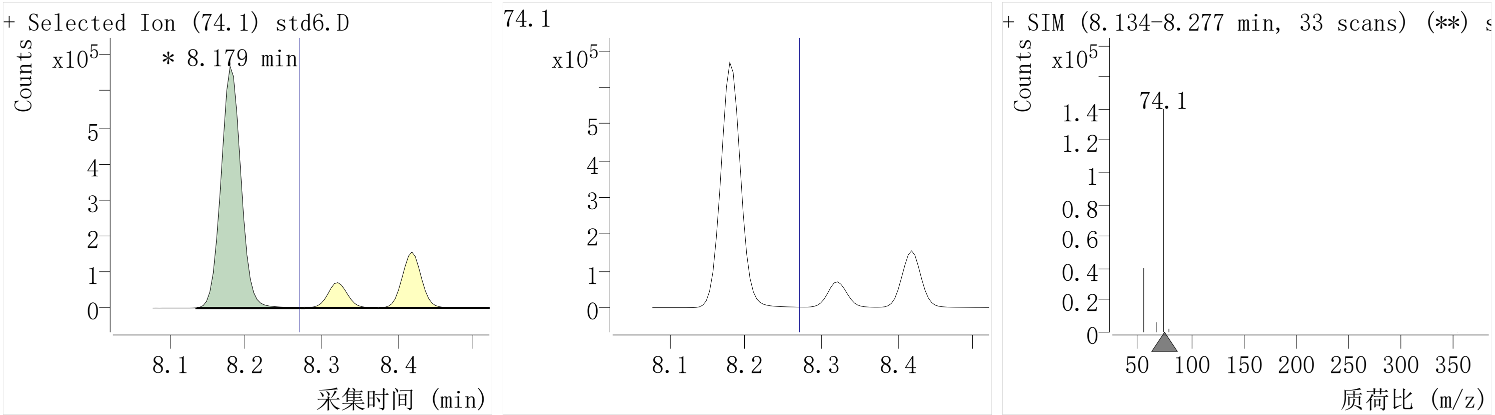

C18:1n9t

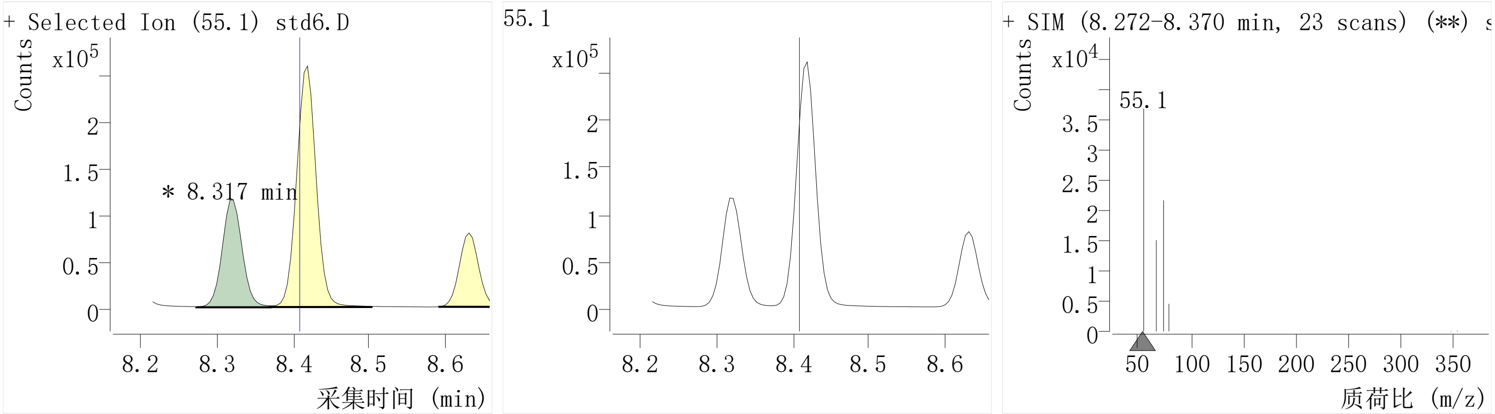

C18:1n9c

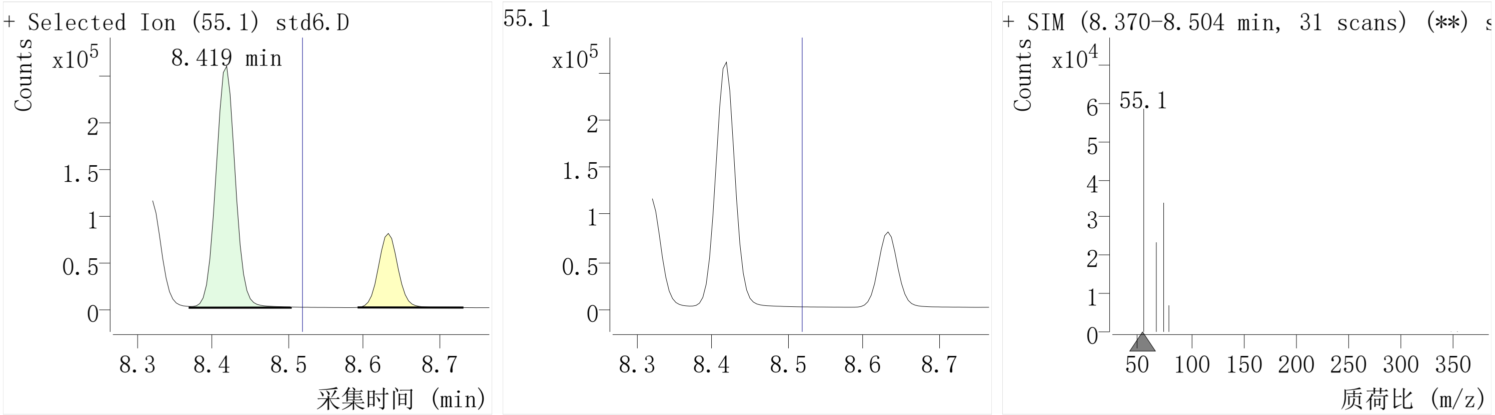

C18:2n6t

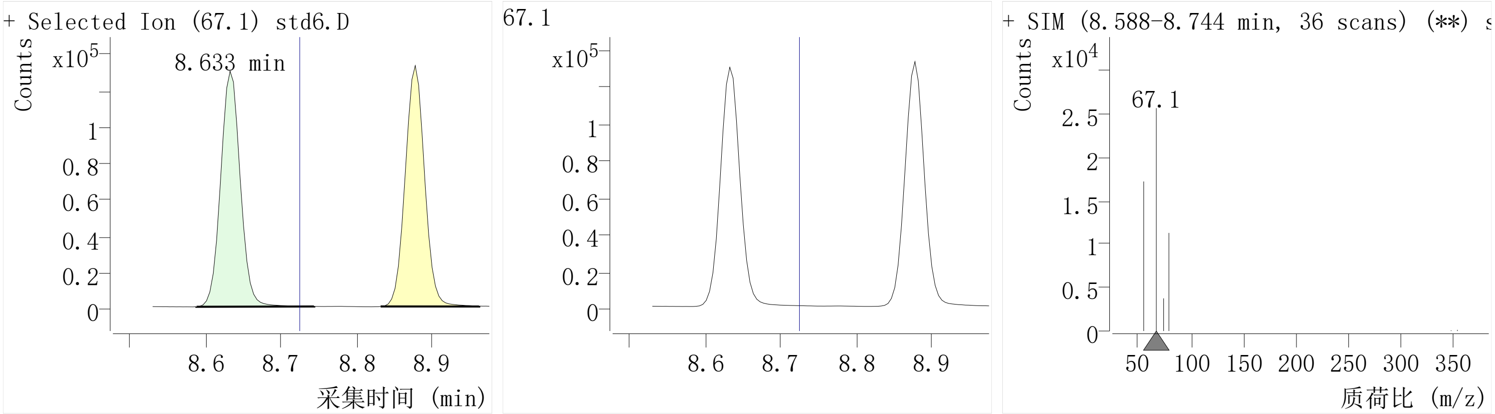

C18:2n6c

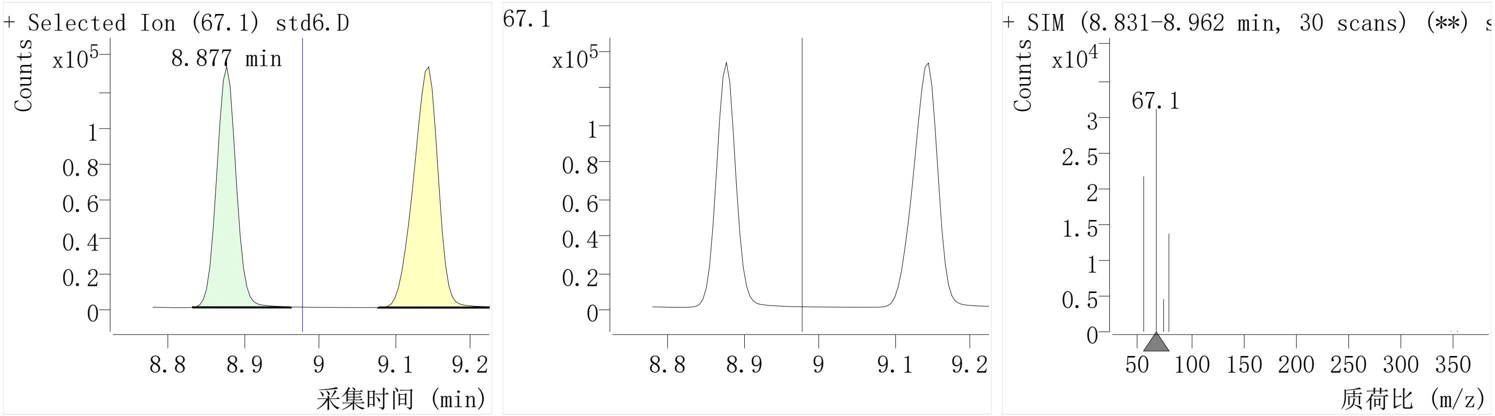

C18:3n6

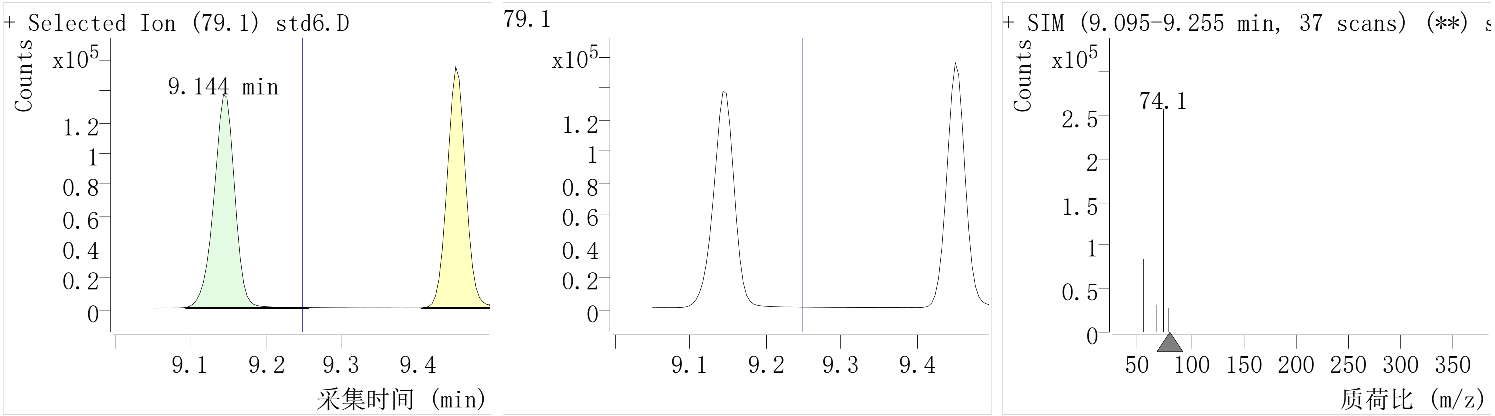

C18:3n3

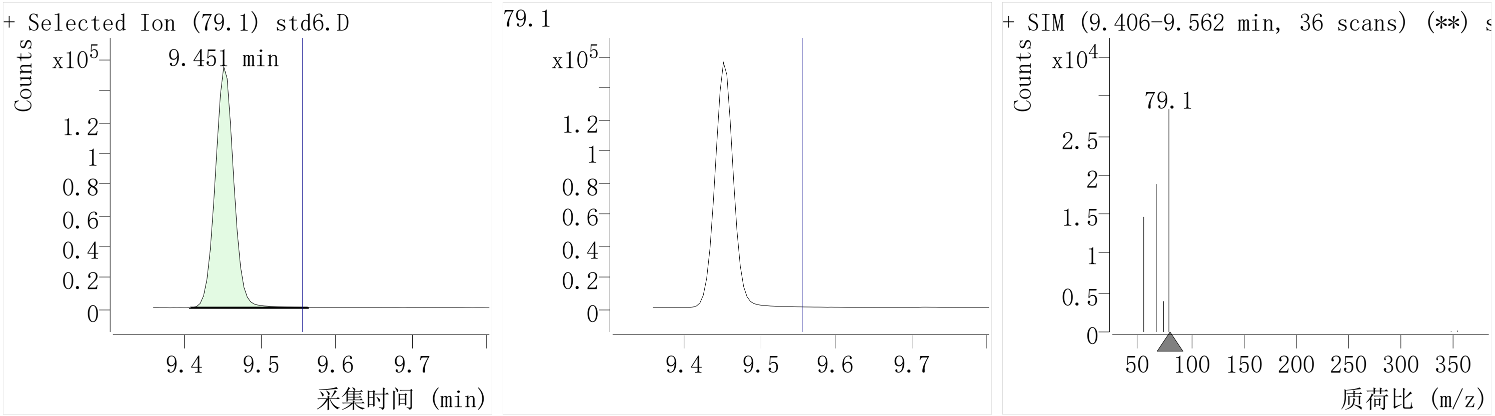

C20:0

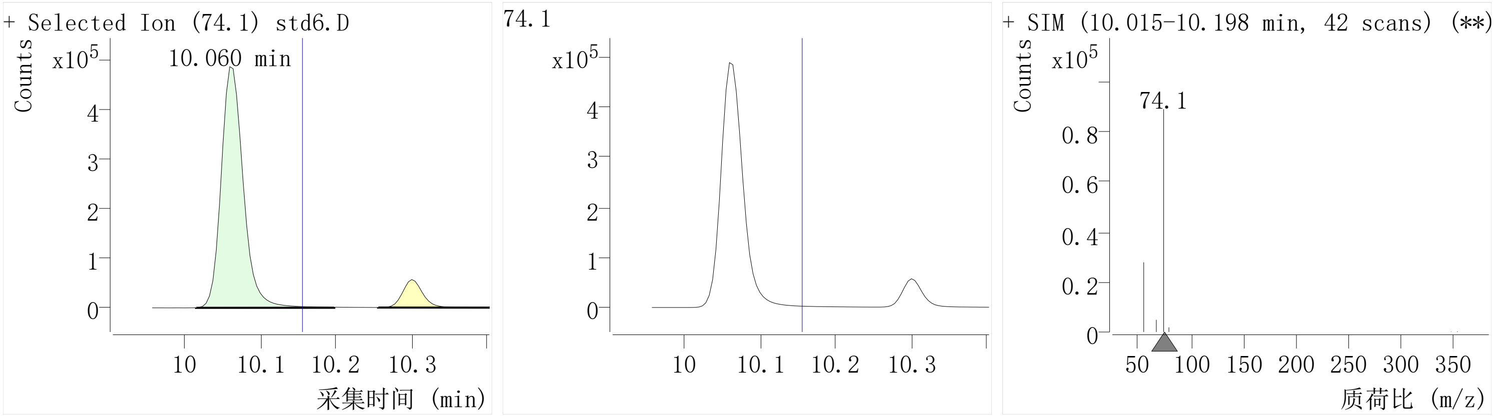

C20:1

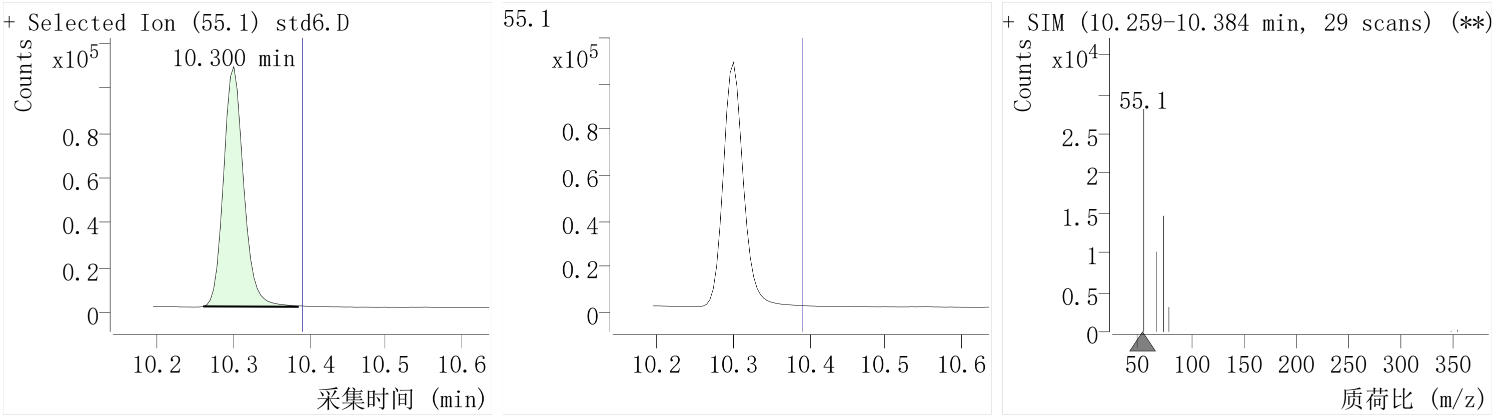

C20:2

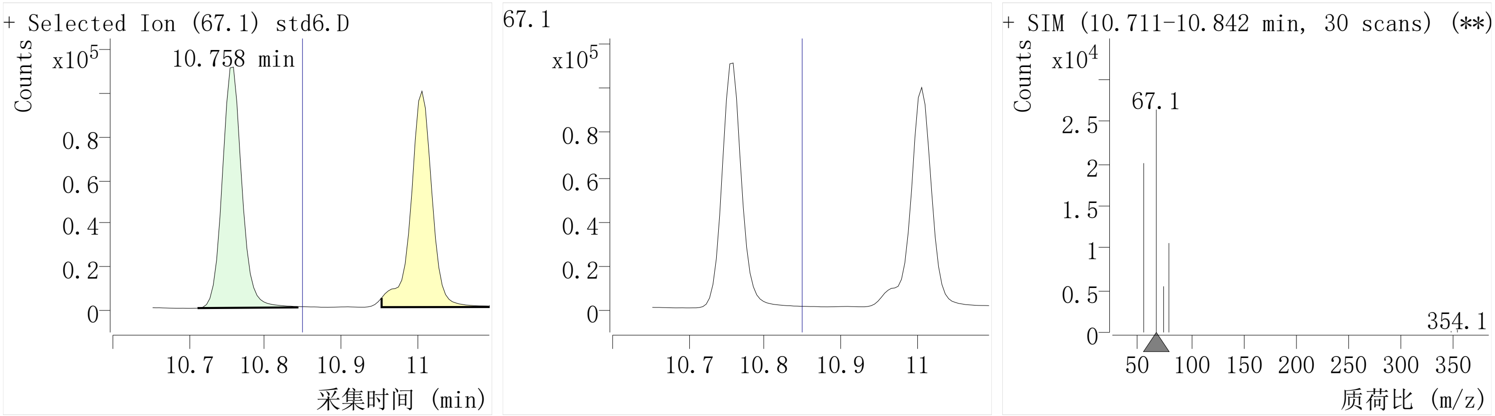

C21:0

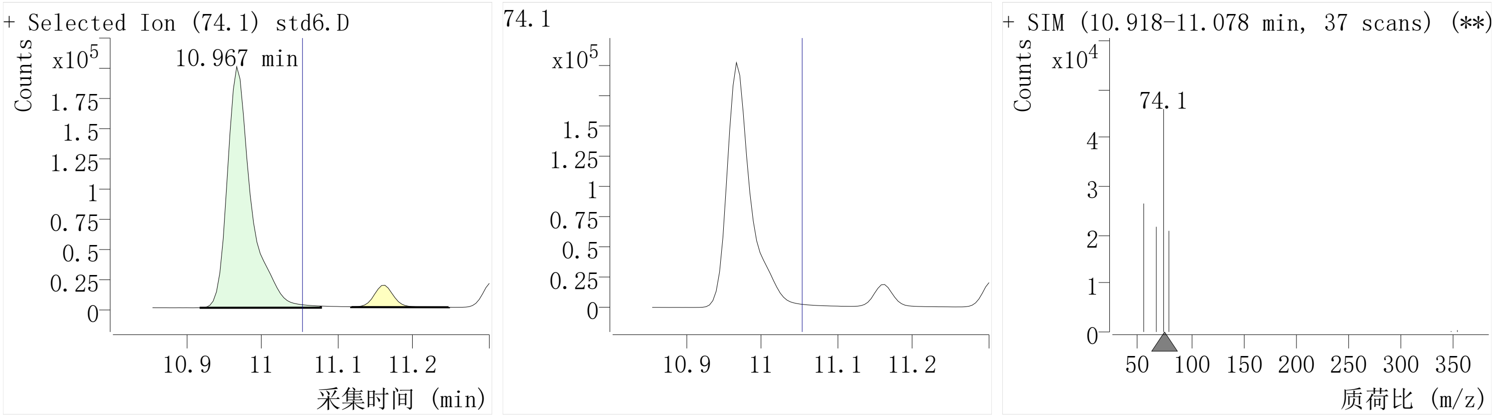

C20:3n6

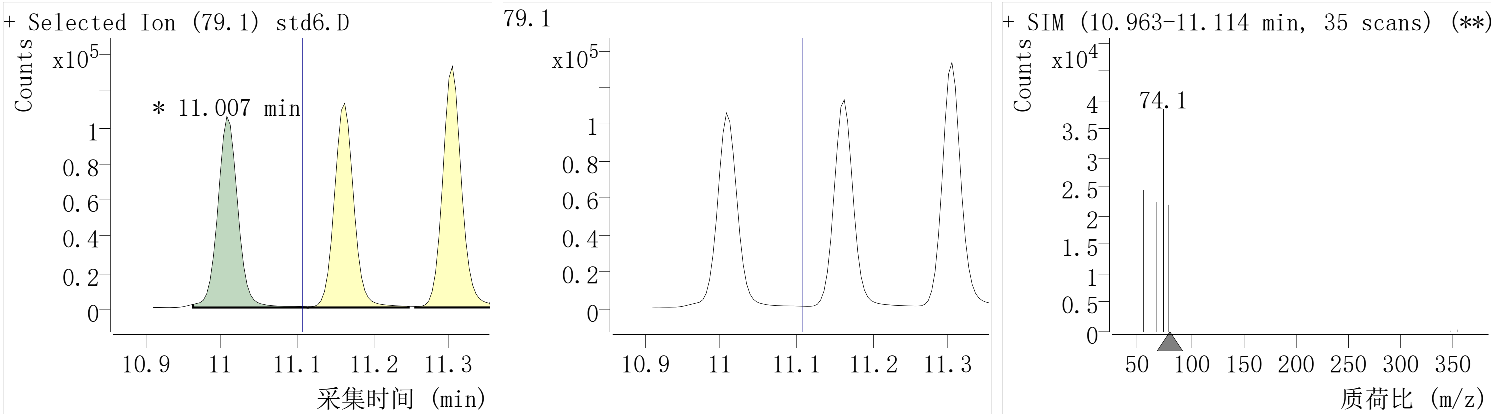

C20:4n6

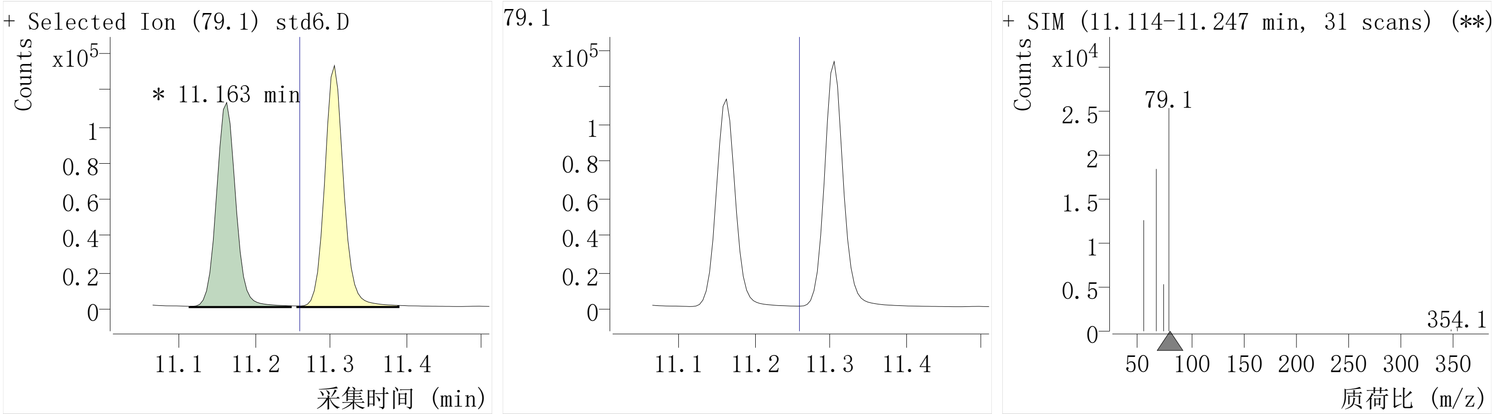

C20:3n3

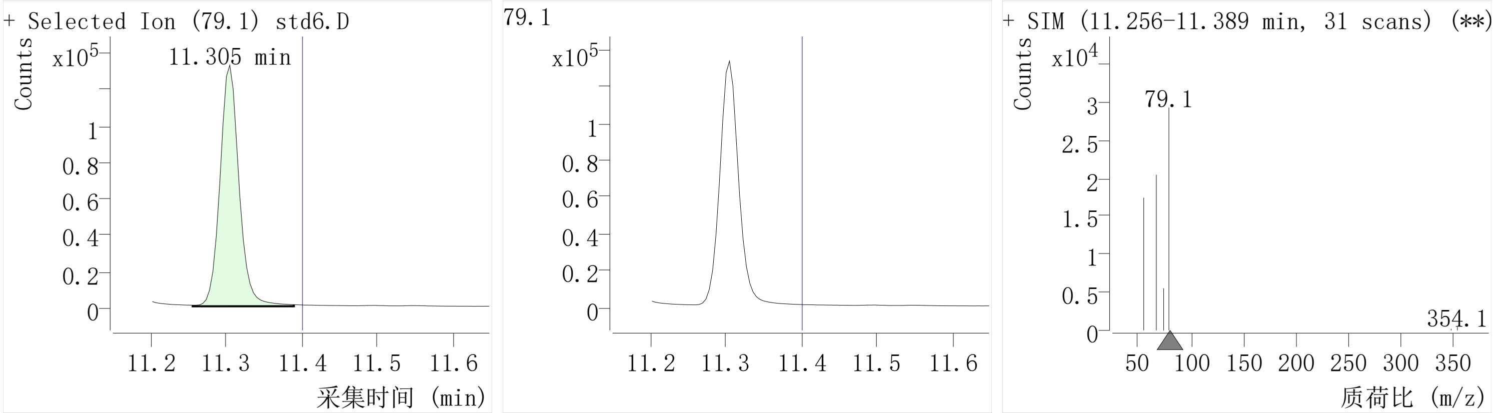

C20:5n3

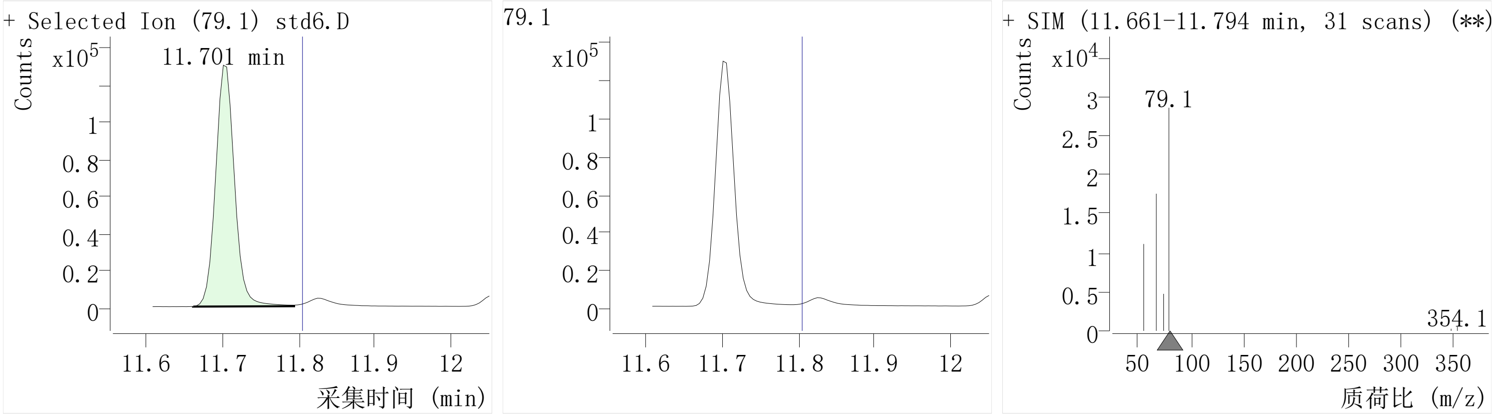

C22:0

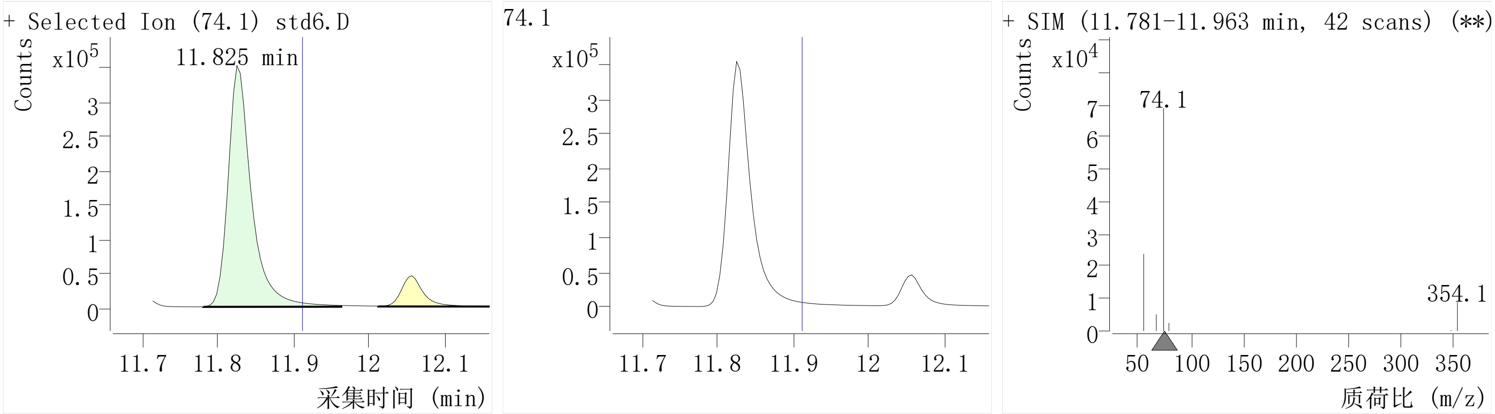

C22:1n9

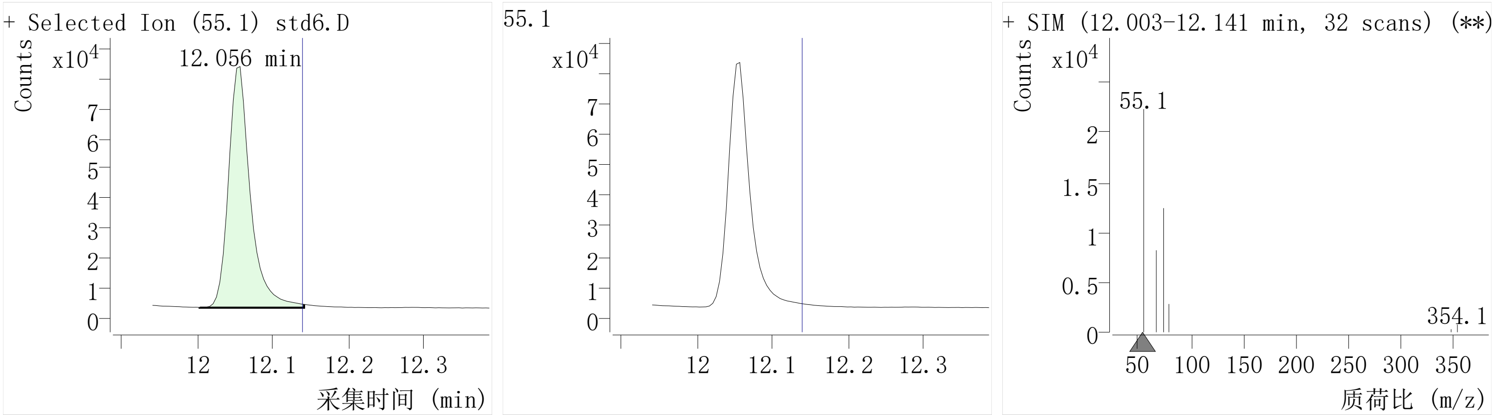

C22:2n6

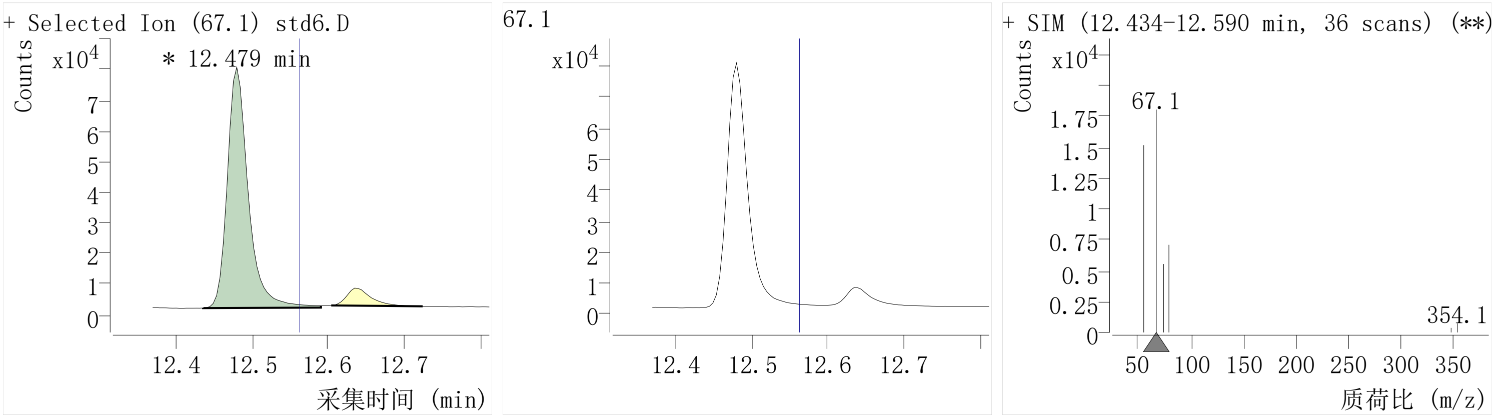

C23:0

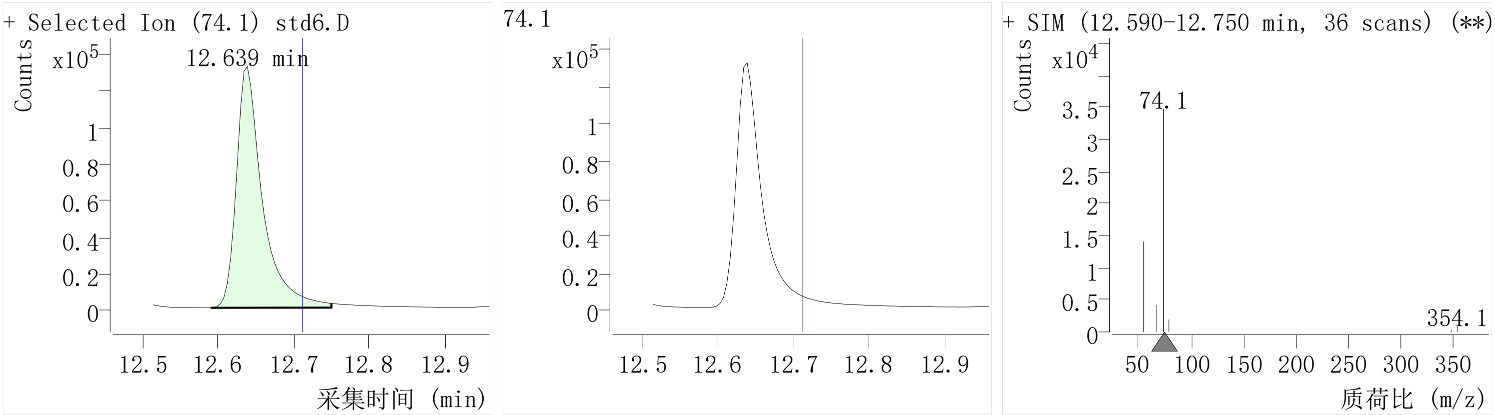

C24:0

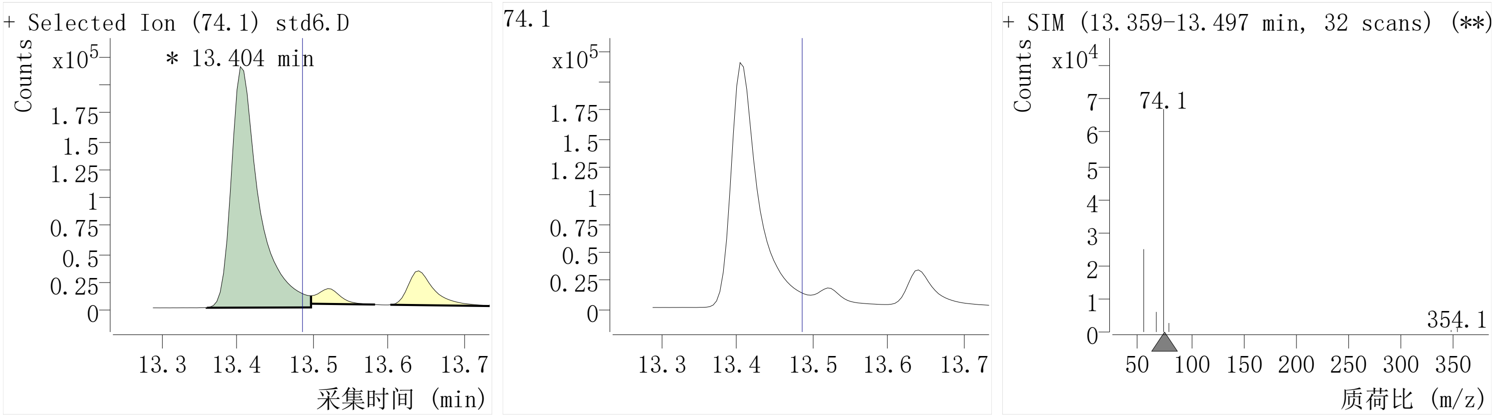

C22:6

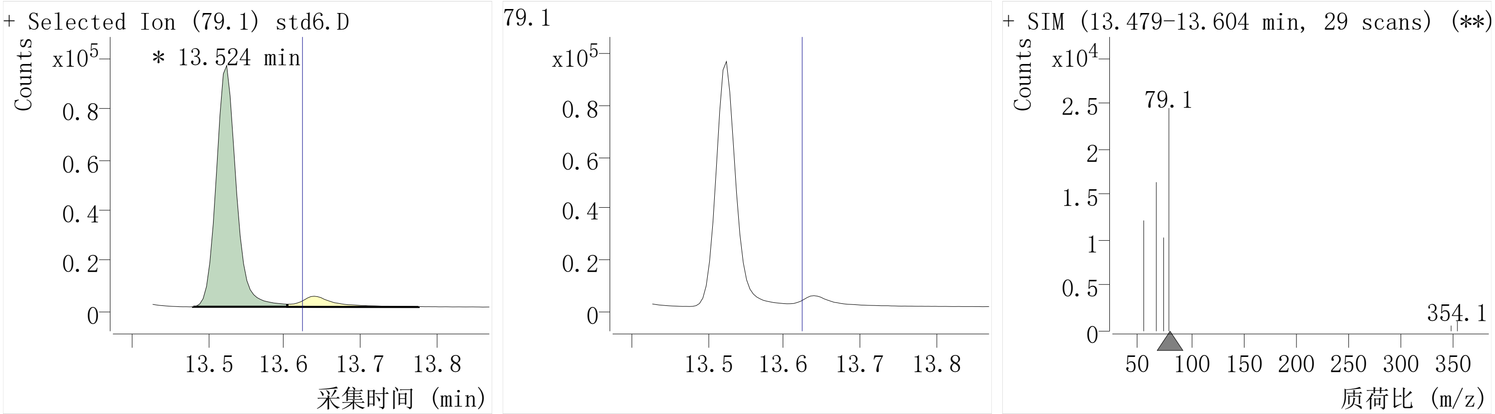
+ Selected Ion (79.1) std6.D
Counts
x10^5
* 13.524 min
0.8
0.6
0.4
0.2
0
13.5 13.6 13.7 13.8
采集时间 (min)
79.1
x10^5
0.8
0.6
0.4
0.2
0
13.5 13.6 13.7 13.8
+ SIM (13.479-13.604 min, 29 scans) (**)
Counts
x10^4
79.1
2.5
2
1.5
1
0.5
0
354.1
50 100 150 200 250 300 350
质荷比 (m/z)

C24:1

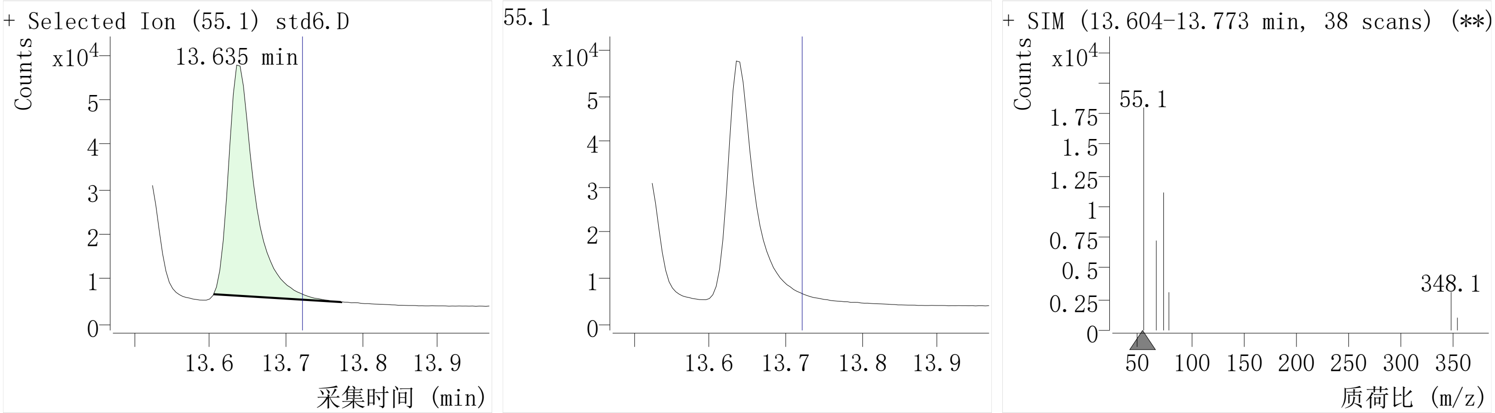
+ Selected Ion (55.1) std6.D
Counts
x10^4
13.635 min
5
4
3
2
1
0
13.6 13.7 13.8 13.9
采集时间 (min)
55.1
x10^4
5
4
3
2
1
0
13.6 13.7 13.8 13.9
+ SIM (13.604-13.773 min, 38 scans) (**)
Counts
x10^4
55.1
1.75
1.5
1.25
1
0.75
0.5
0.25
0
348.1
50 100 150 200 250 300 350
质荷比 (m/z)

**批处理路径**	G:\GC-MS\HX250430-4-GCMS总脂肪酸靶向检测\HX250430-4\QuantResults\HX250430-4.batch.bin		
**分析时间**	2025/5/14 16:58	**分析员姓名**	DESKTOP-M3A0GPO\omics
**报告时间**	2025/5/16 14:53:07	**报告员姓名**	DESKTOP-M3A0GPO\omics
**最近校正更新**	2025/5/14 16:58	**批处理状态**	已处理
**定量批处理版本**	10.2	**定量报告版本**	10.2

**采集时间**	2025/5/8 19:30	**数据文件**	std7.D
**样品类型**	校正	**样品名称**	std7
**稀释**	1	**采集方法**	脂肪酸

样品色谱图

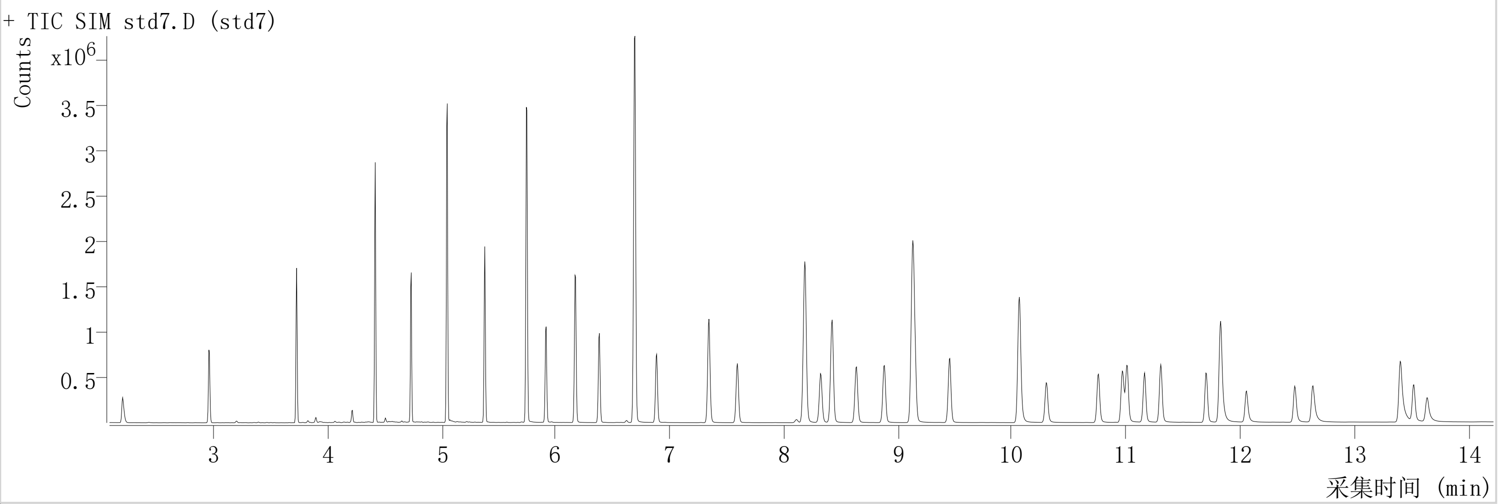

化合物	ISTD	RT	响应	ISTD 响应	响应比	最终浓度	单位
C4:0	C19:0	2.203	331688	2635656	0.1258	21.1776	ug/ml
C6:0	C19:0	2.958	629485	2635656	0.2388	22.6303	ug/ml
C8:0	C19:0	3.728	1028279	2635656	0.3901	22.5716	ug/ml
C10:0	C19:0	4.417	1614554	2635656	0.6126	22.3561	ug/ml
C11:0	C19:0	4.733	965645	2635656	0.3664	11.0348	ug/ml
C12:0	C19:0	5.048	2170310	2635656	0.8234	22.1310	ug/ml
C13:0	C19:0	5.377	1198590	2635656	0.4548	10.8981	ug/ml
C14:0	C19:0	5.747	2575499	2635656	0.9772	20.2091	ug/ml
C14:1	C19:0	5.911	520980	2635656	0.1977	9.8638	ug/ml
C15:0	C19:0	6.173	1329210	2635656	0.5043	10.6225	ug/ml
C15:1	C19:0	6.382	577044	2635656	0.2189	11.0005	ug/ml
C16:0	C19:0	6.694	4112945	2635656	1.5605	32.5265	ug/ml
C16:1	C19:0	6.880	454539	2635656	0.1725	10.4507	ug/ml
C17:0	C19:0	7.343	1294994	2635656	0.4913	10.2507	ug/ml
C17:1	C19:0	7.592	506735	2635656	0.1923	10.3260	ug/ml
C18:0	C19:0	8.183	2520868	2635656	0.9564	19.7089	ug/ml
C18:1n9t	C19:0	8.321	453645	2635656	0.1721	10.1532	ug/ml
C18:1n9c	C19:0	8.423	967718	2635656	0.3672	21.2426	ug/ml
C18:2n6t	C19:0	8.637	503880	2635656	0.1912	10.4991	ug/ml
C18:2n6c	C19:0	8.881	506011	2635656	0.1920	9.6682	ug/ml
C18:3n6	C19:0	9.148	515357	2635656	0.1955	10.1947	ug/ml
C18:3n3	C19:0	9.455	558658	2635656	0.2120	10.5321	ug/ml
C20:0	C19:0	10.064	2043689	2635656	0.7754	19.5929	ug/ml
C20:1	C19:0	10.300	421746	2635656	0.1600	9.9008	ug/ml
C20:2	C19:0	10.758	419918	2635656	0.1593	9.7903	ug/ml
C21:0	C19:0	10.967	929280	2635656	0.3526	9.6996	ug/ml
C20:3n6	C19:0	11.011	405416	2635656	0.1538	9.7588	ug/ml
C20:4n6	C19:0	11.163	416629	2635656	0.1581	9.8921	ug/ml
C20:3n3	C19:0	11.305	484842	2635656	0.1840	9.6685	ug/ml
C20:5n3	C19:0	11.705	473008	2635656	0.1795	9.9537	ug/ml

定量分析完成报告

化合物	ISTD	RT	响应	ISTD 响应	响应比	最终浓度	单位
C22:0	C19:0	11.829	1597788	2635656	0.6062	19.6482	ug/ml
C22:1n9	C19:0	12.056	345729	2635656	0.1312	9.6962	ug/ml
C22:2n6	C19:0	12.479	322970	2635656	0.1225	9.7208	ug/ml
C23:0	C19:0	12.639	685843	2635656	0.2602	10.0294	ug/ml
C24:0	C19:0	13.404	1216420	2635656	0.4615	20.8174	ug/ml
C22:6	C19:0	13.524	375373	2635656	0.1424	10.5259	ug/ml
C24:1	C19:0	13.639	306721	2635656	0.1164	10.2590	ug/ml

C4:0

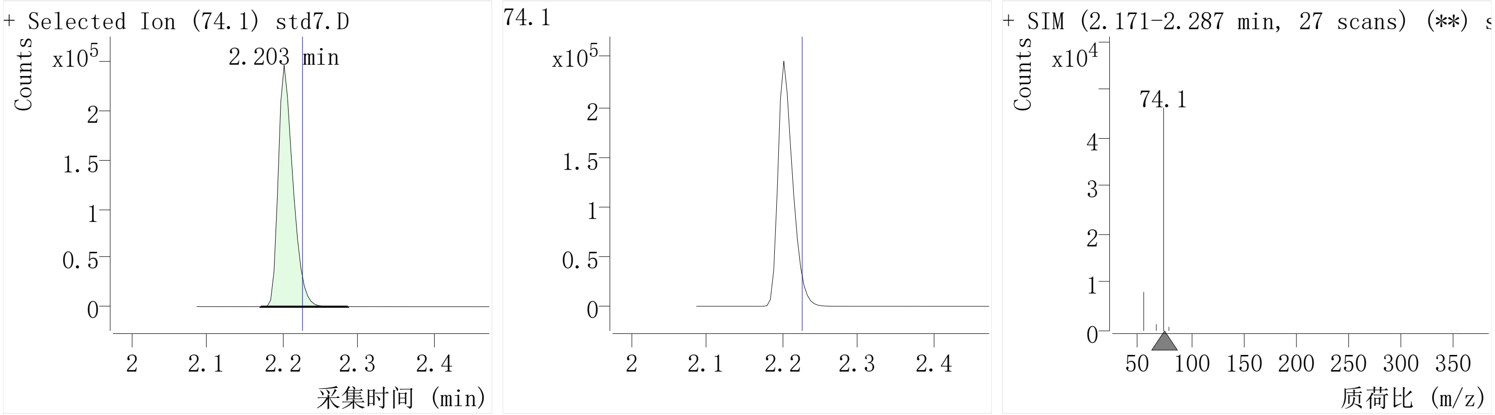

C6:0

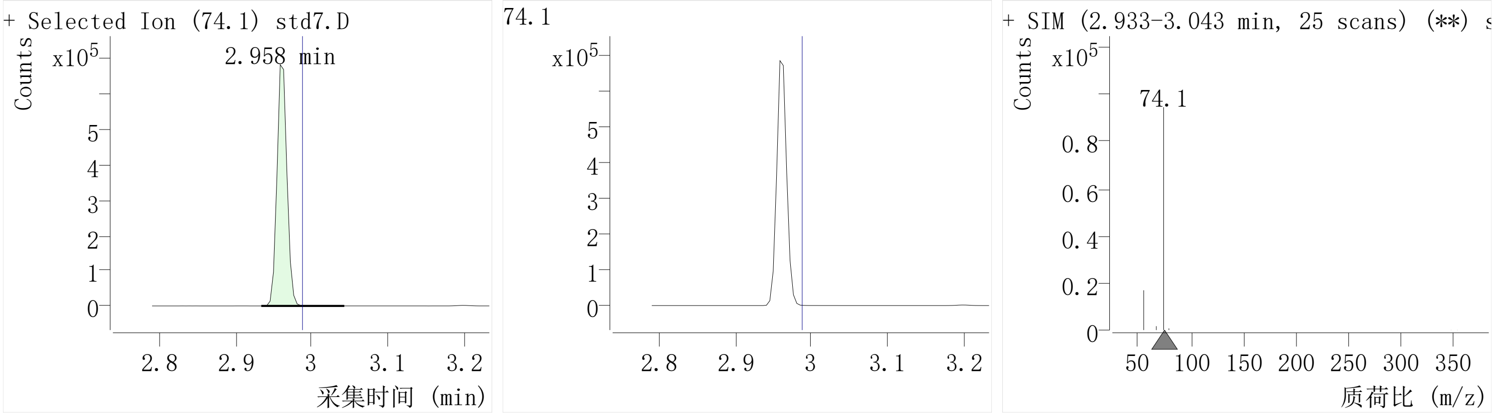

C8:0

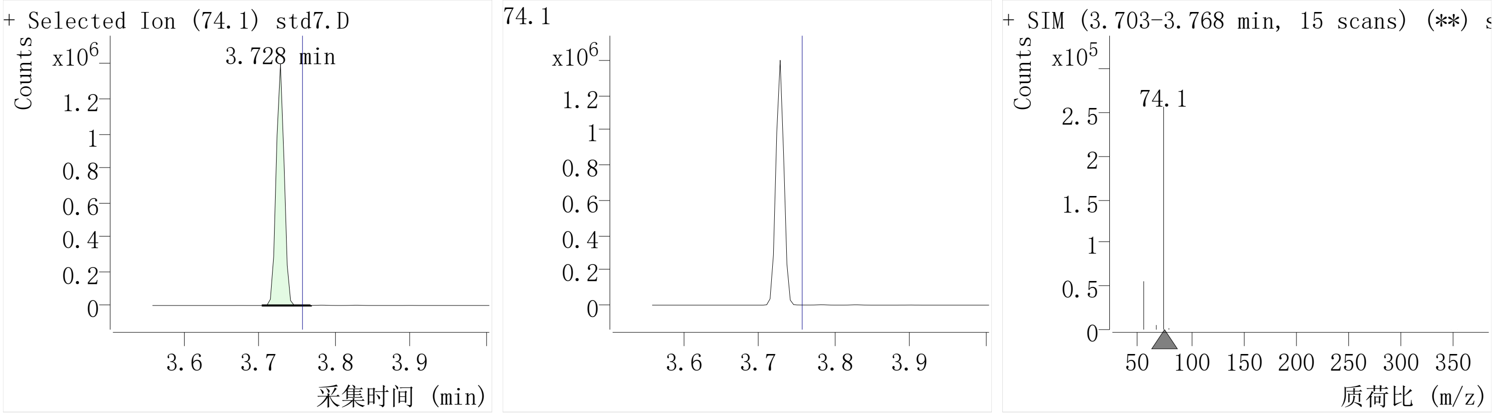

C10:0

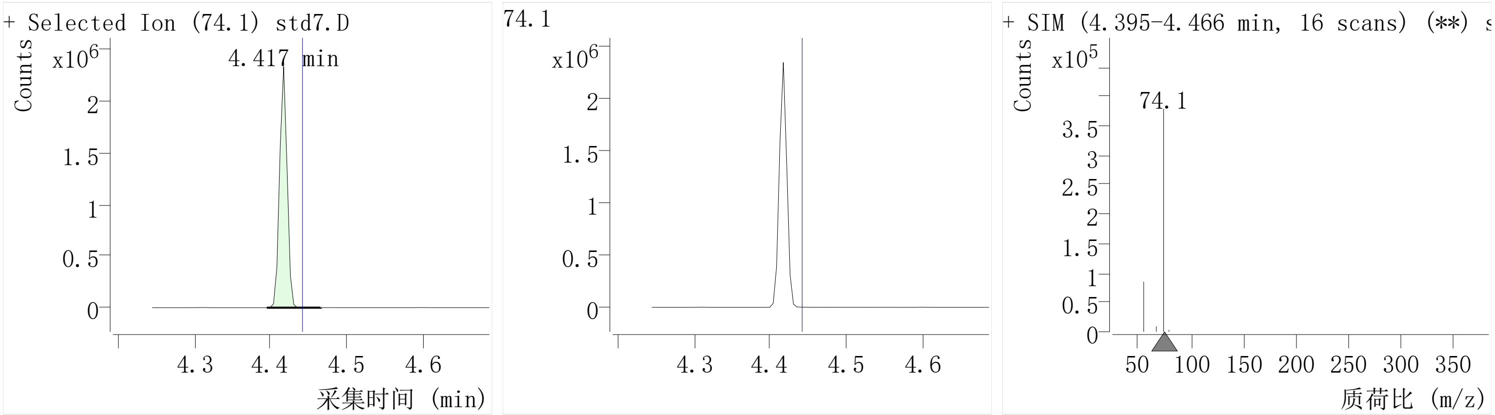

C11:0

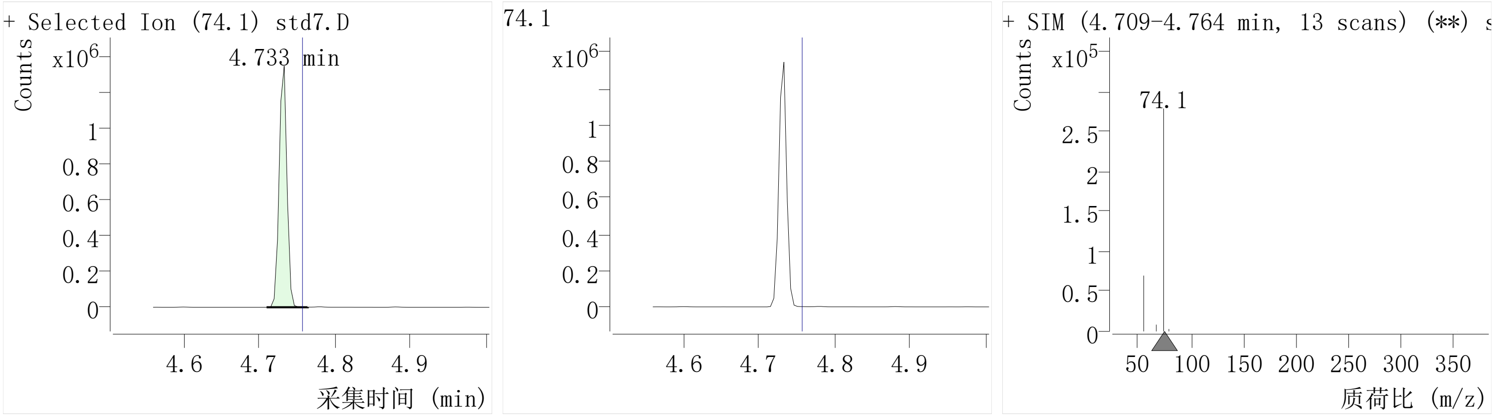

C12:0

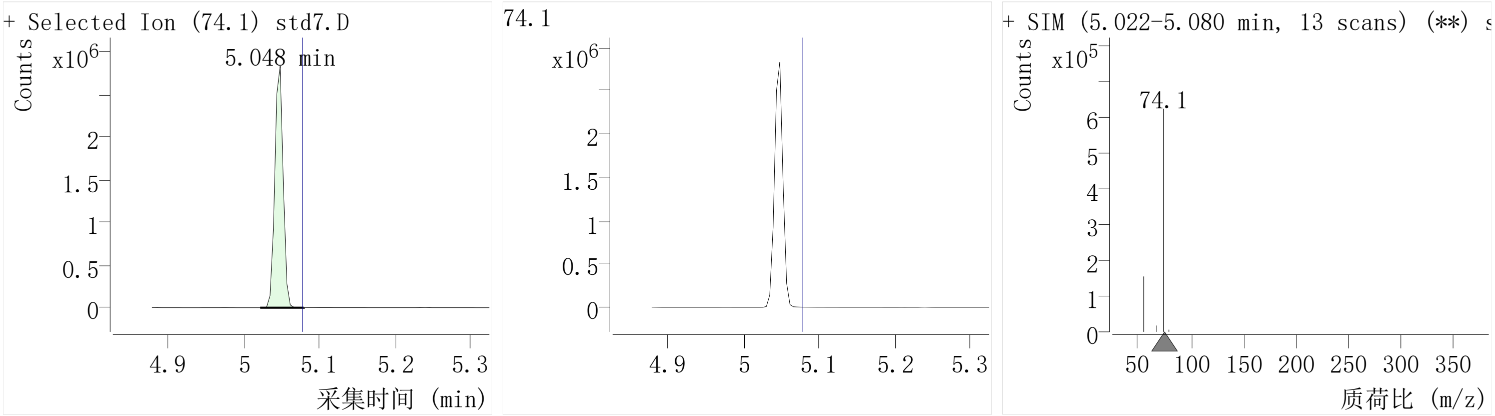

C13:0

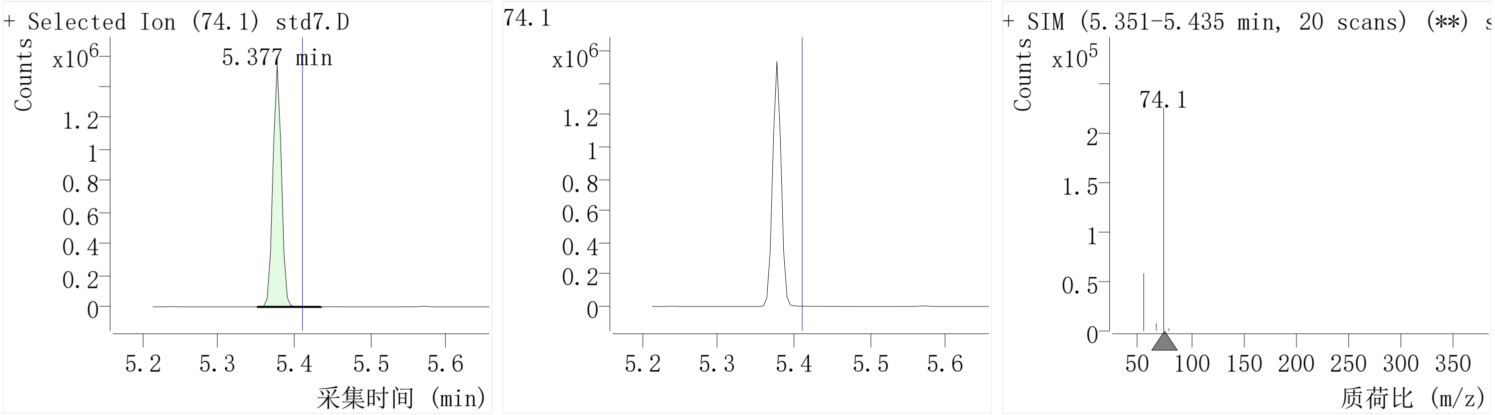

C14:0

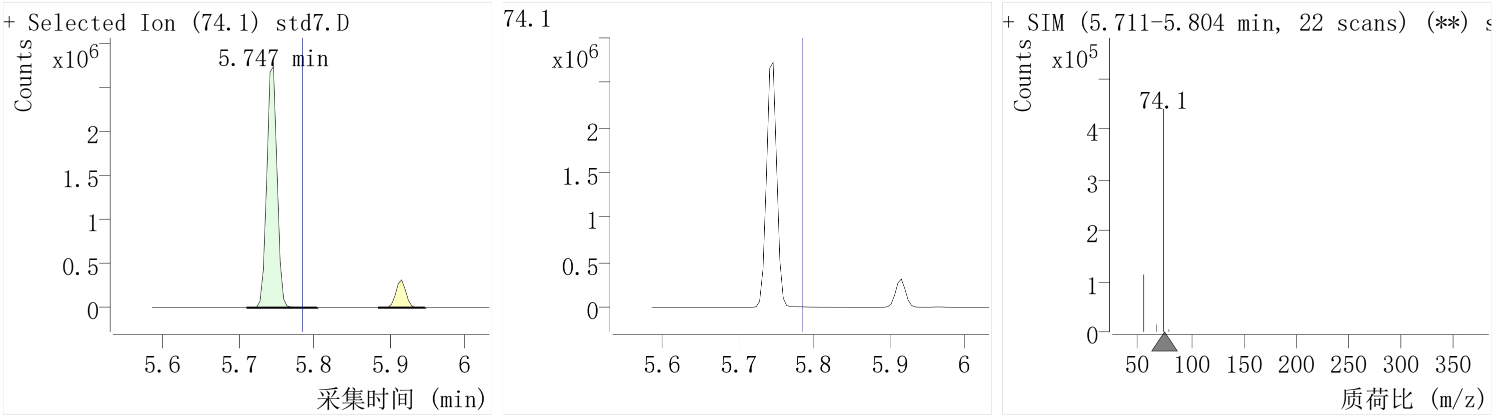

C14:1

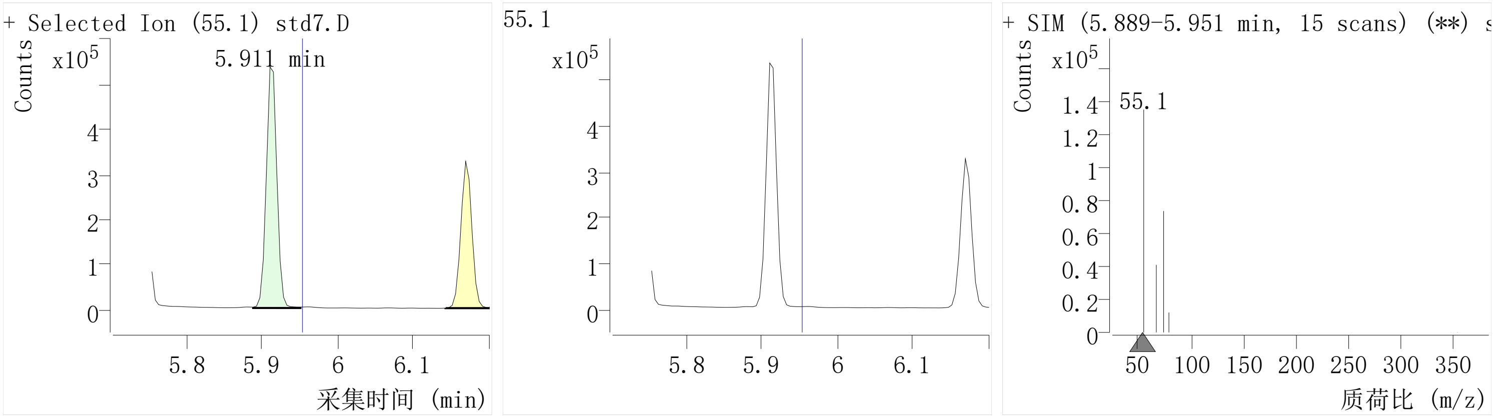

C15:0

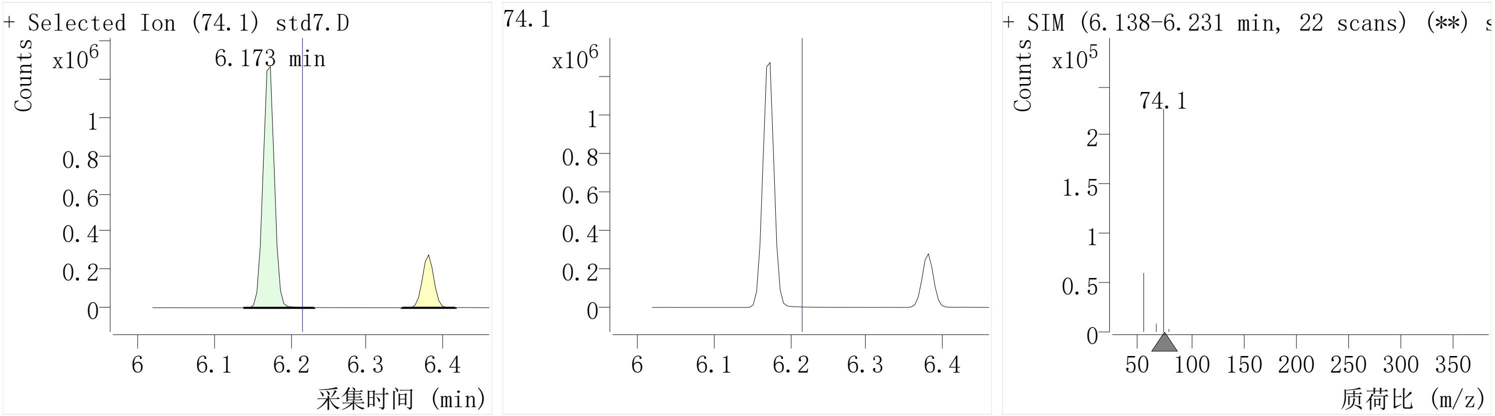

C15:1

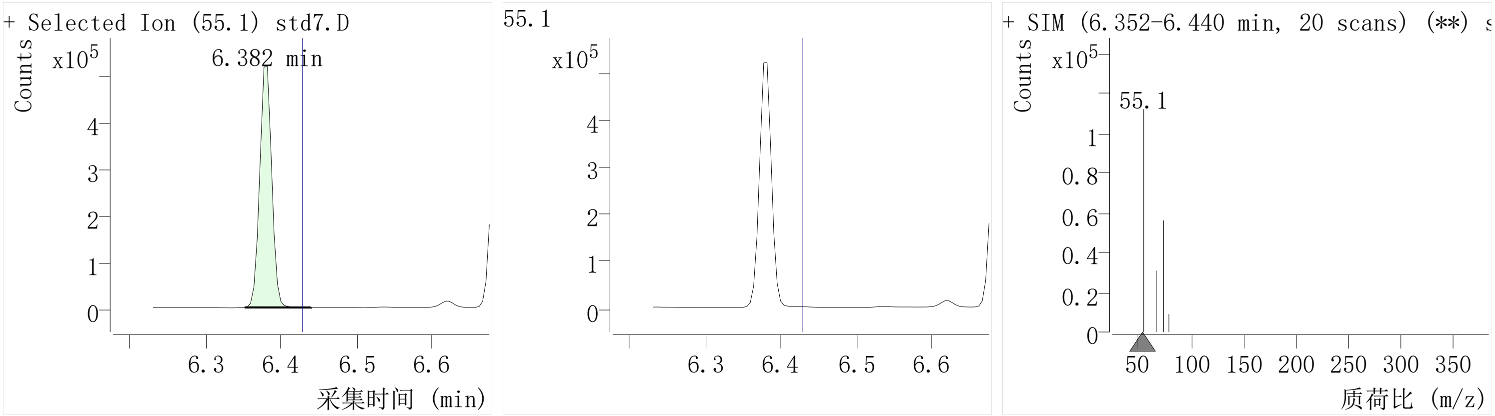

生成时间 2025/5/16 14:52

C16:0

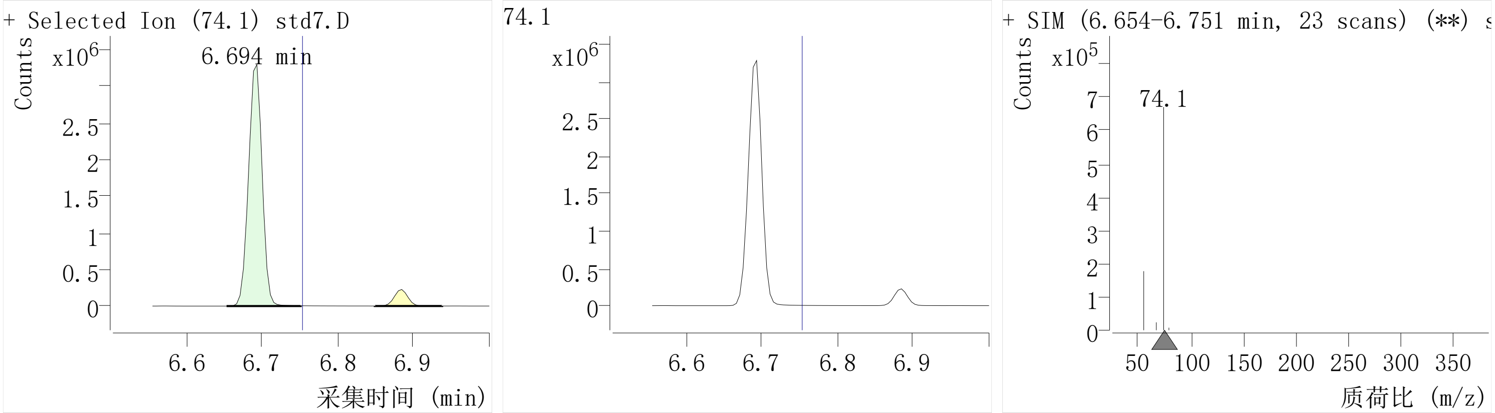

C16:1

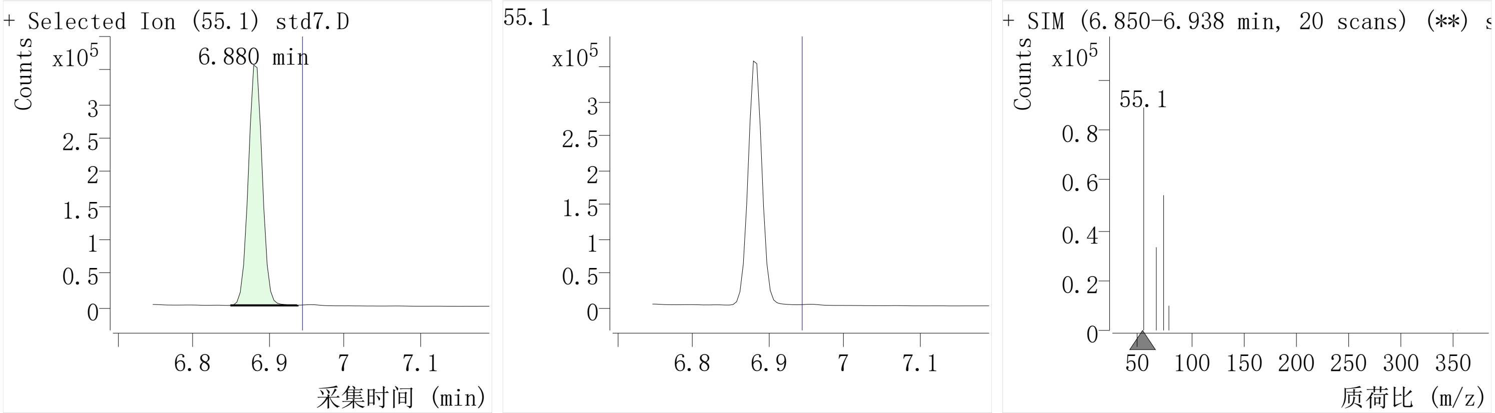

C17:0

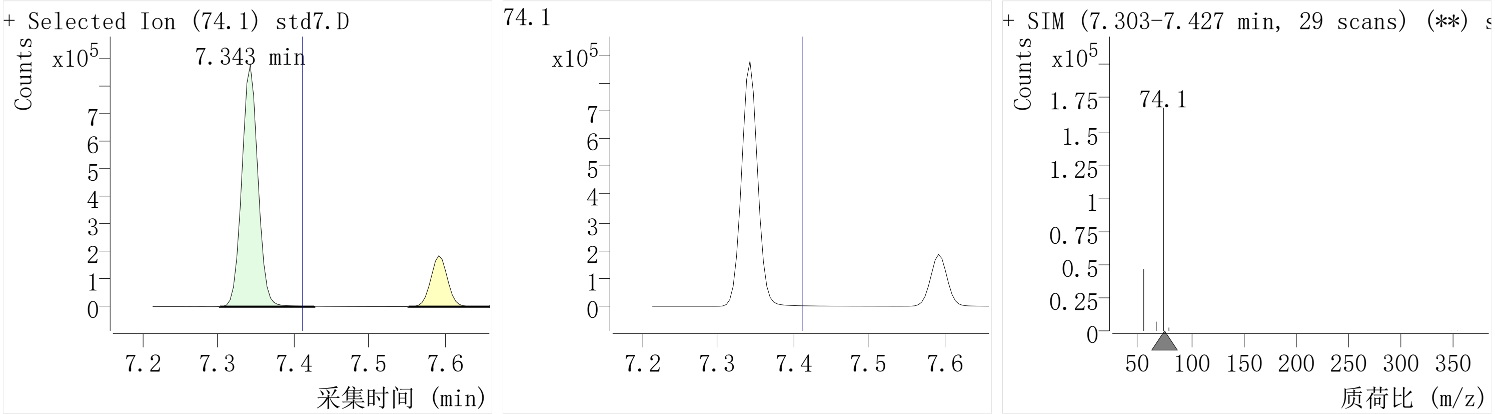

C17:1

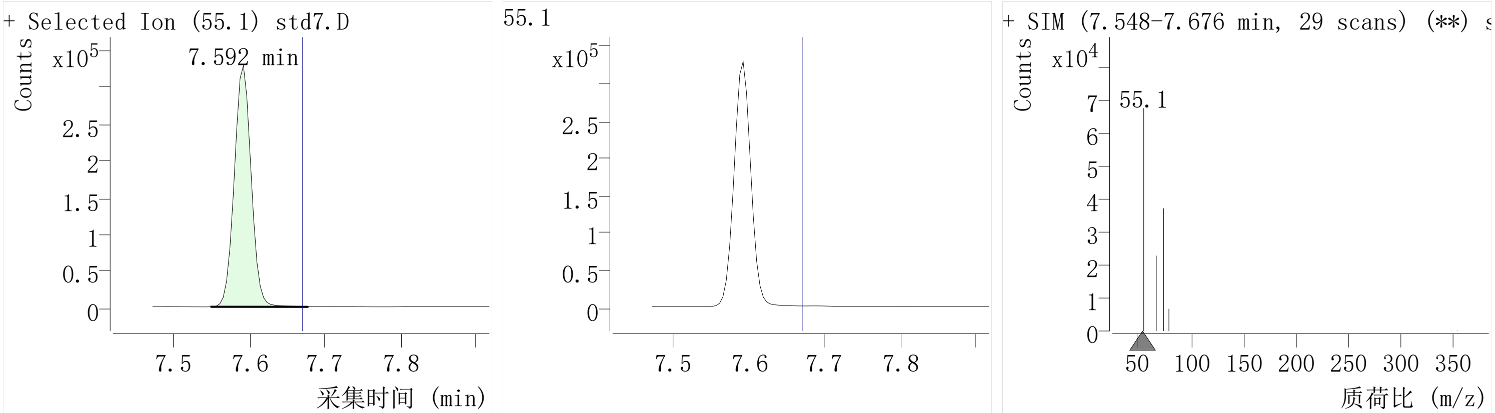

C18:0

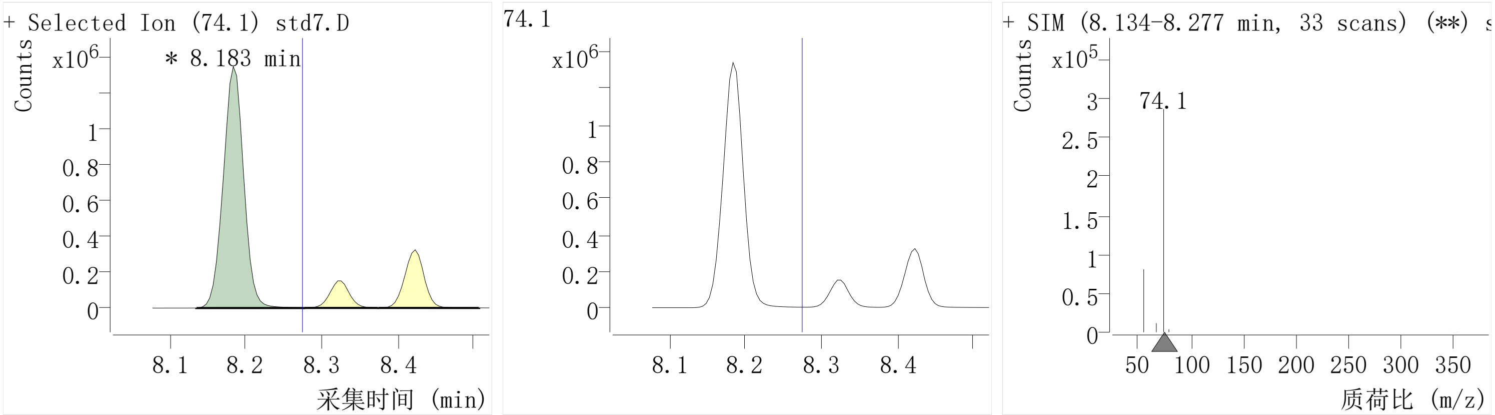

C18:1n9t

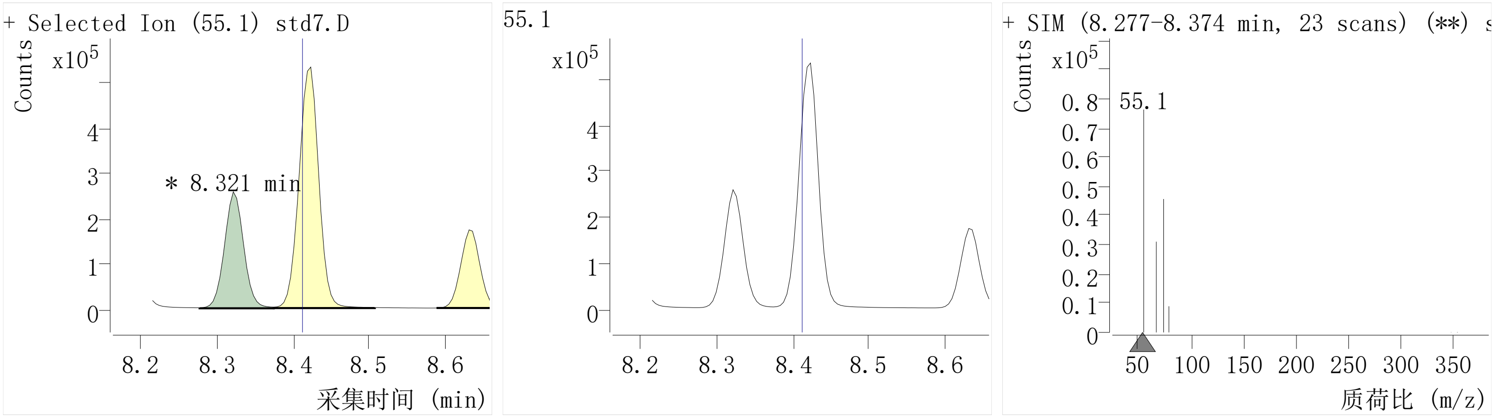

C18:1n9c

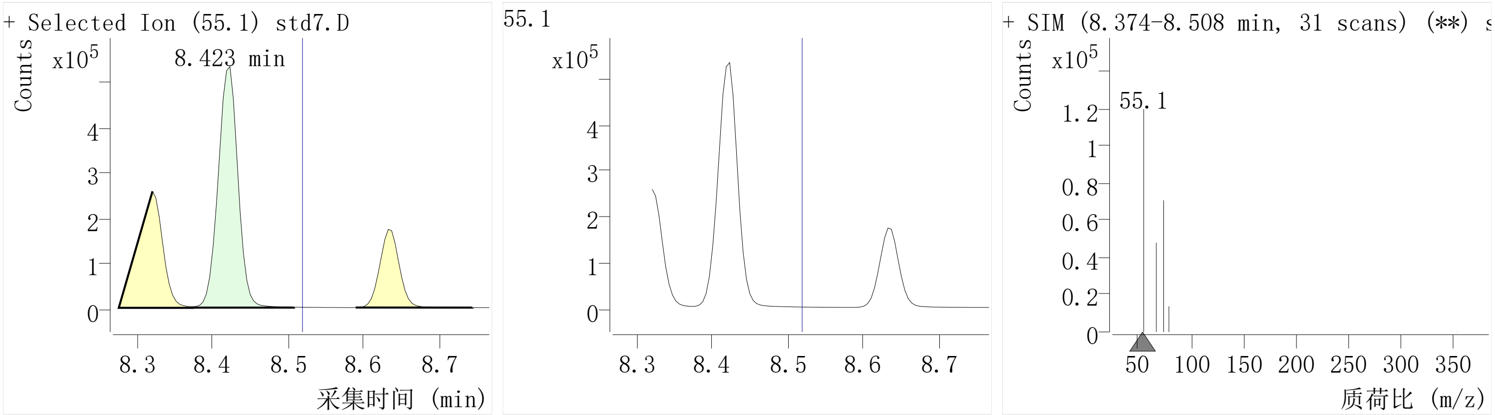

C18:2n6t

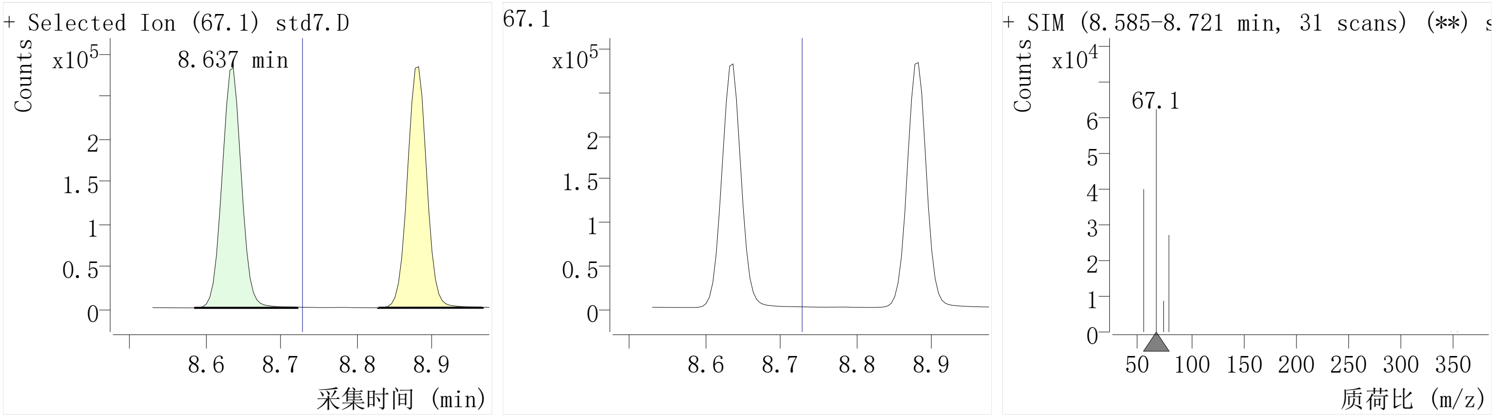

C18:2n6c

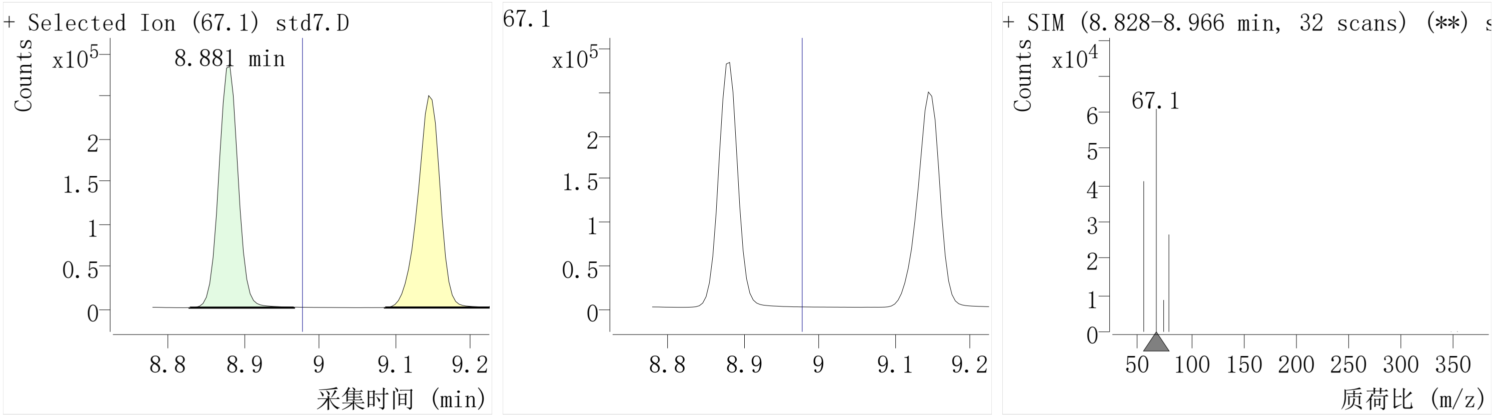

C18:3n6

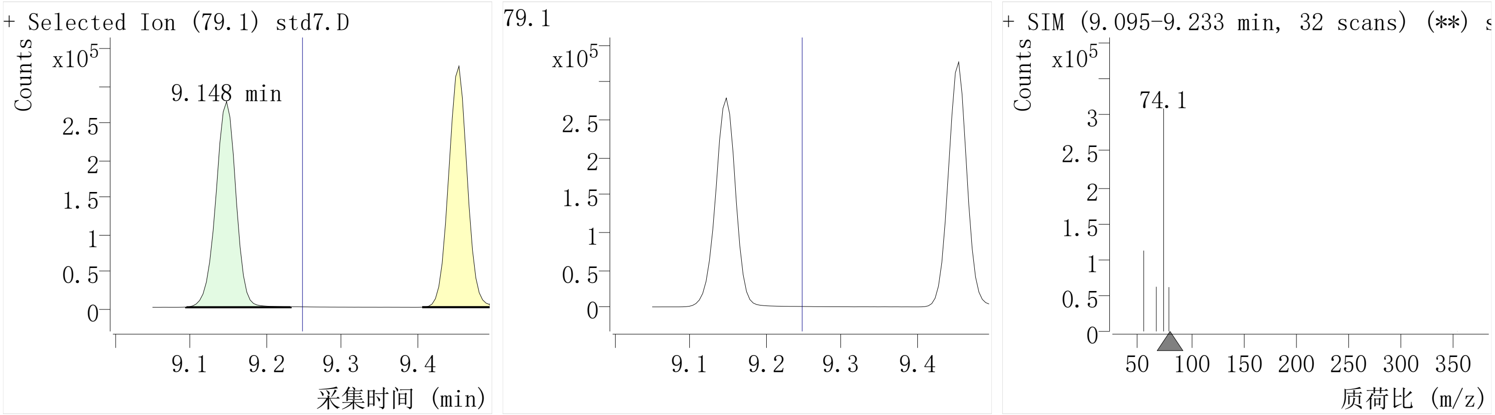

C18:3n3

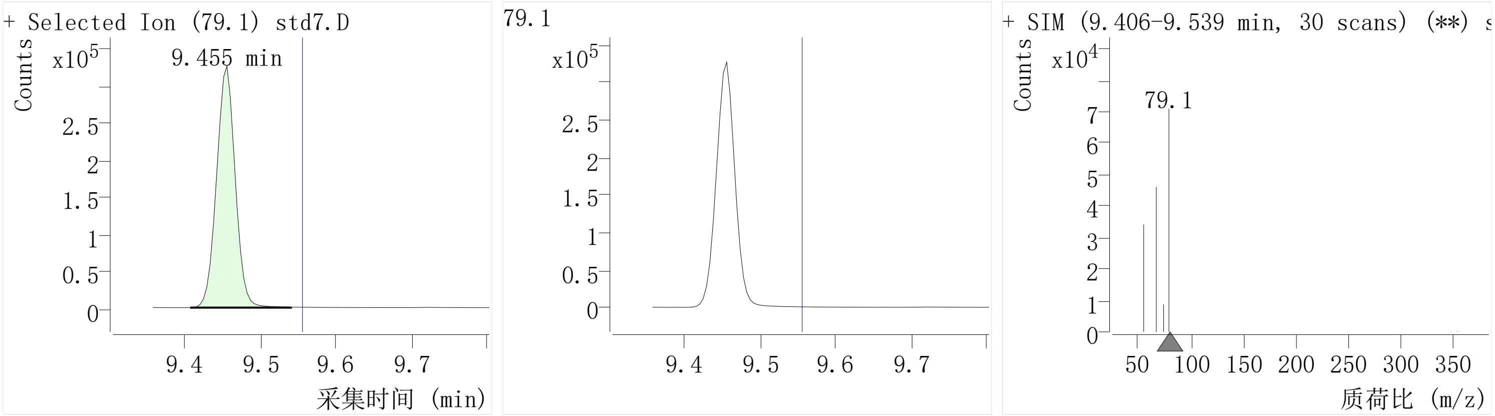

C20:0

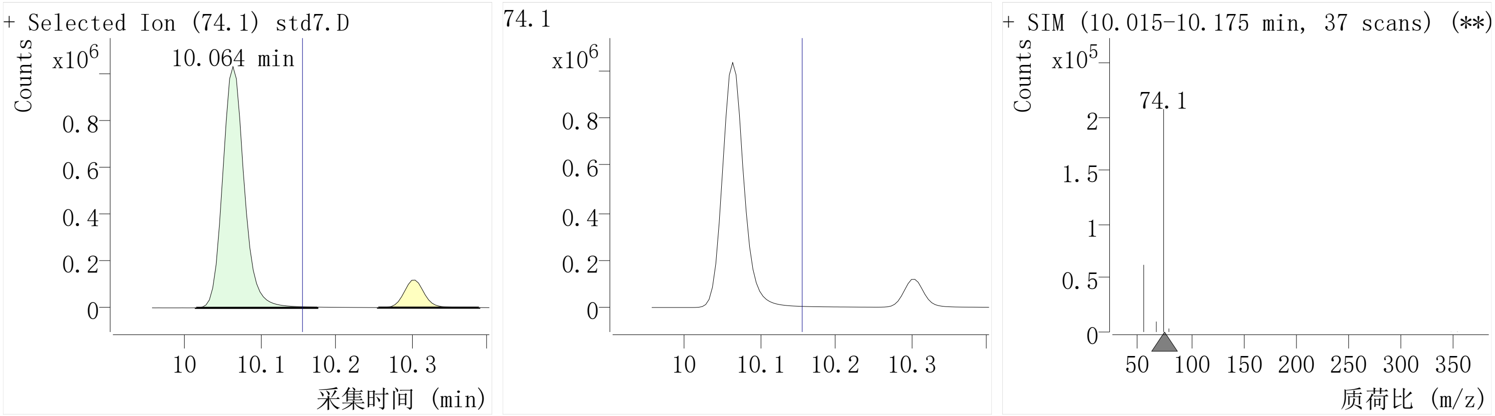

C20:1

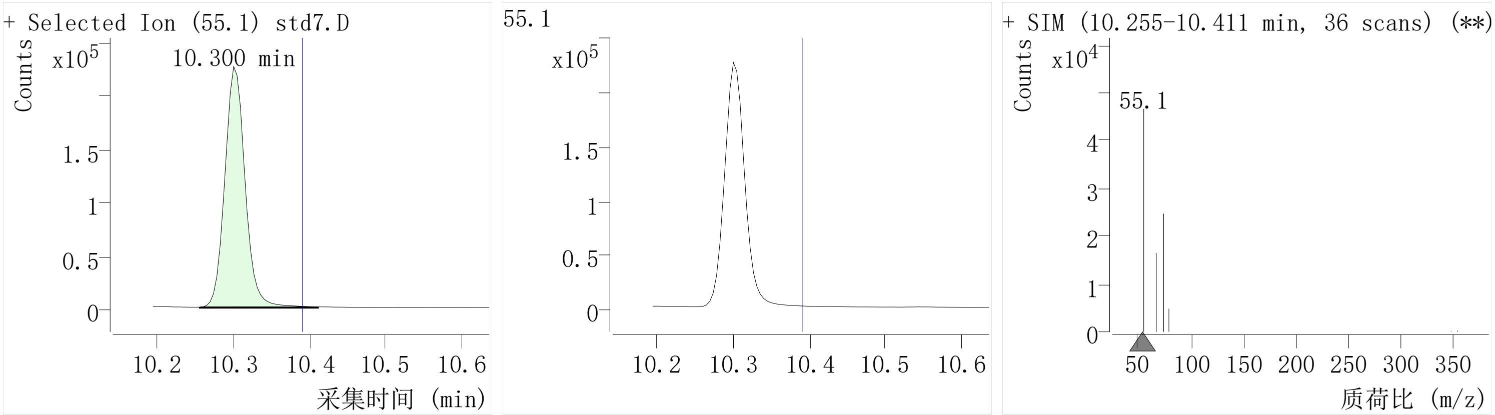

C20:2

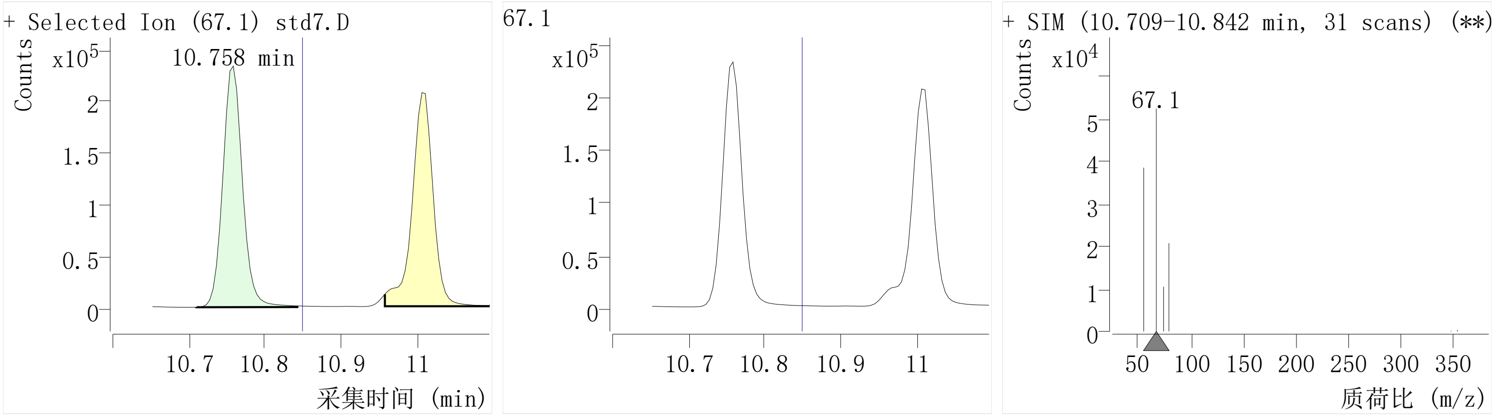

C21:0

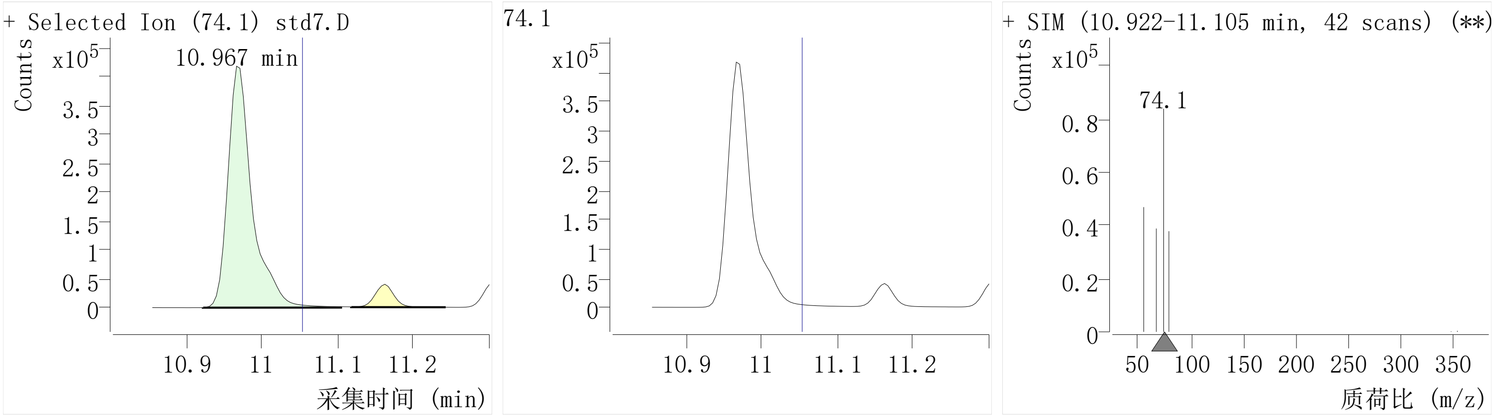

C20:3n6

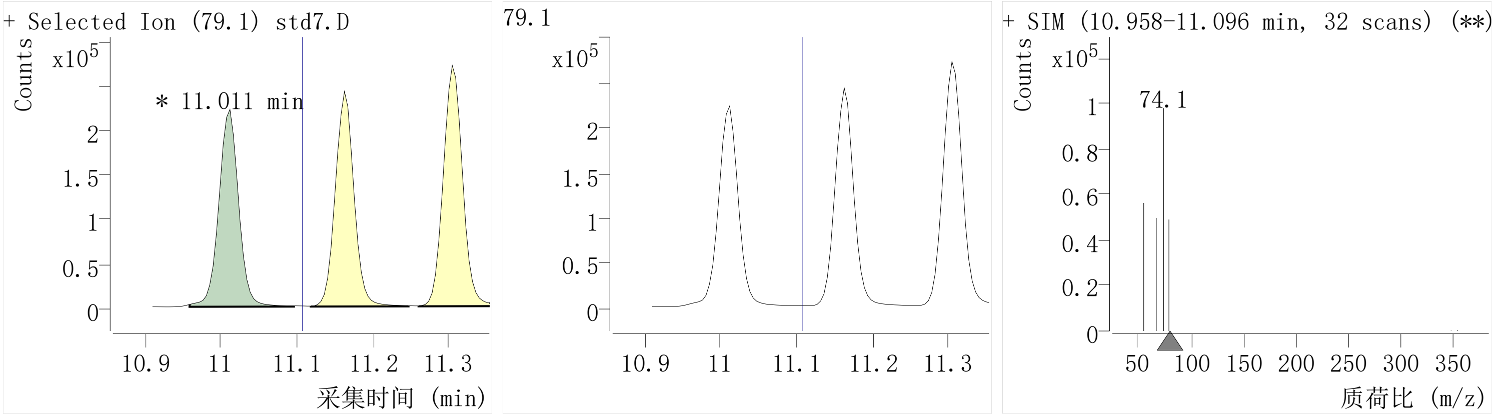

C20:4n6

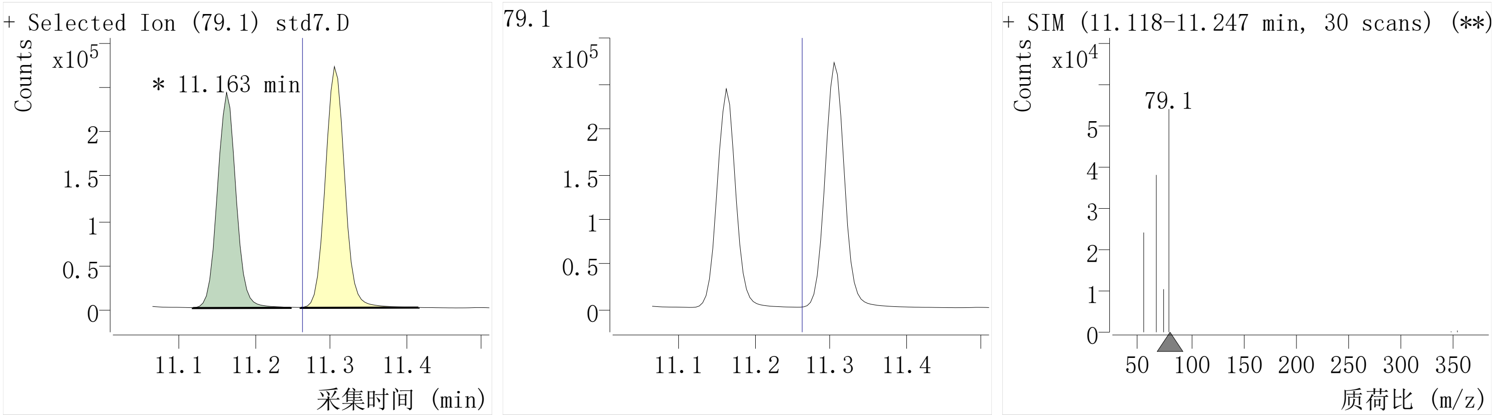

C20:3n3

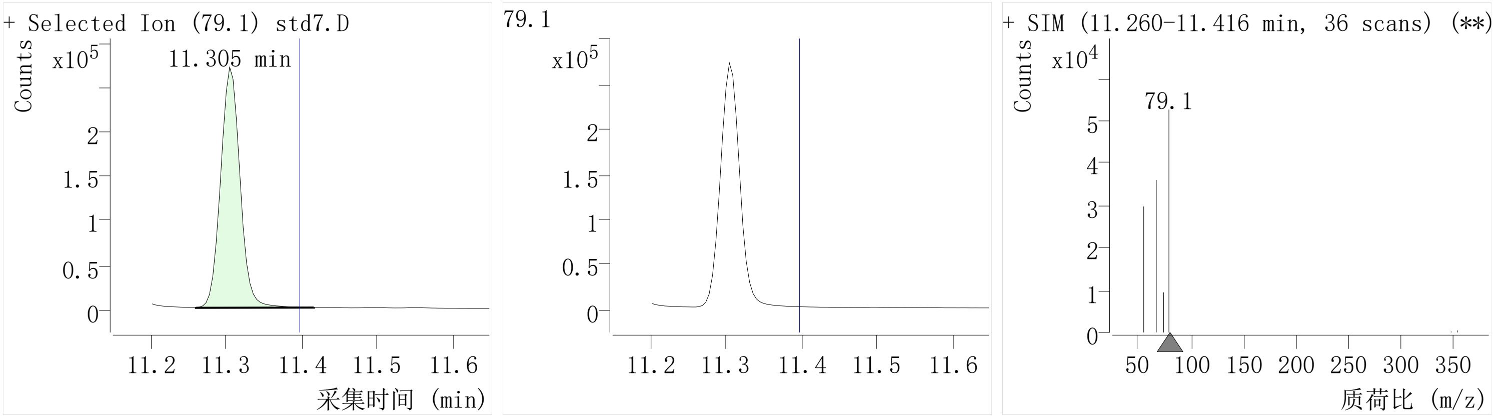

C20:5n3

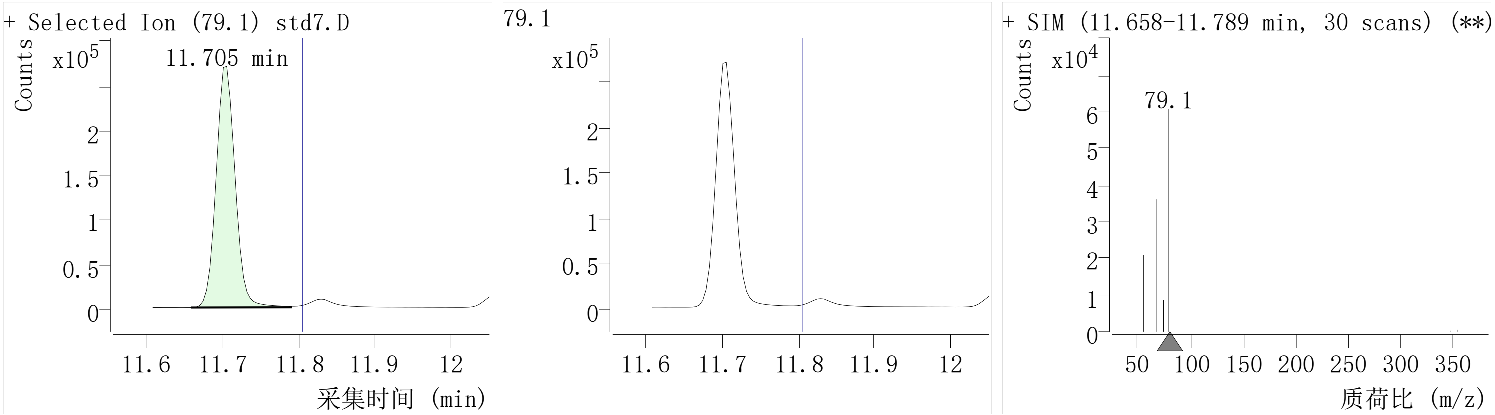

C22:0

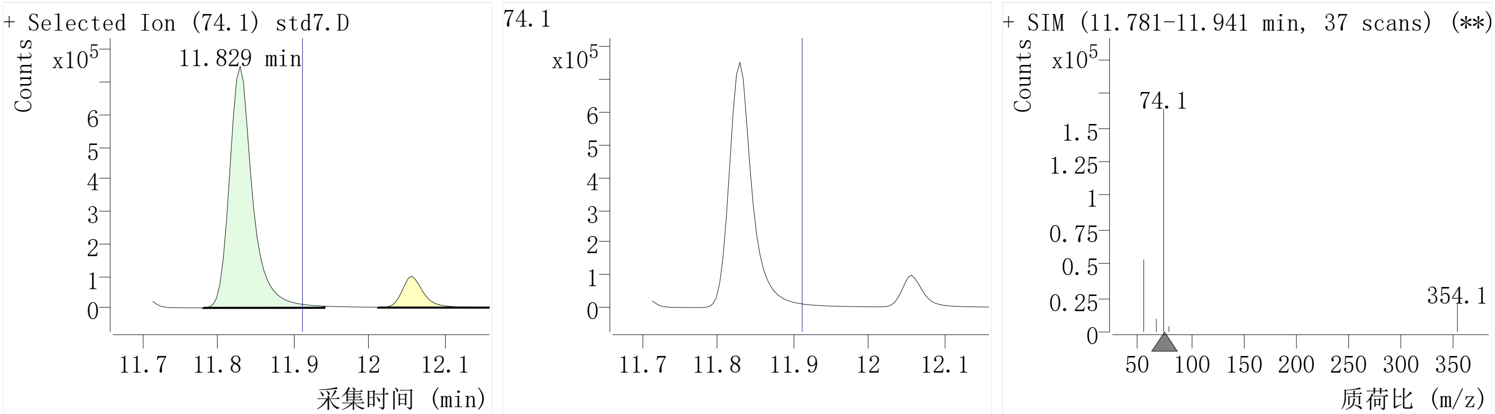

C22:1n9

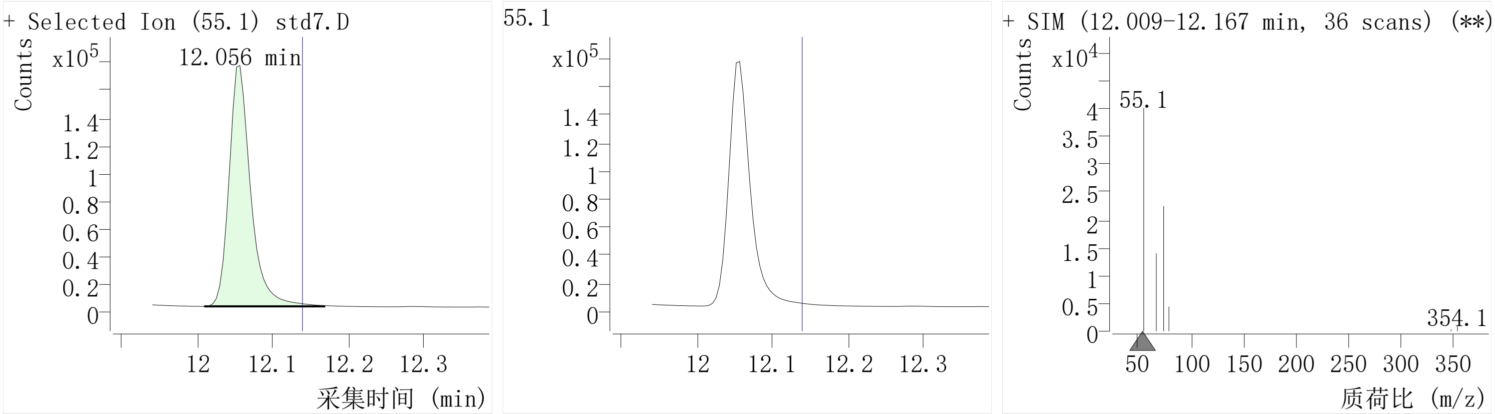

C22:2n6

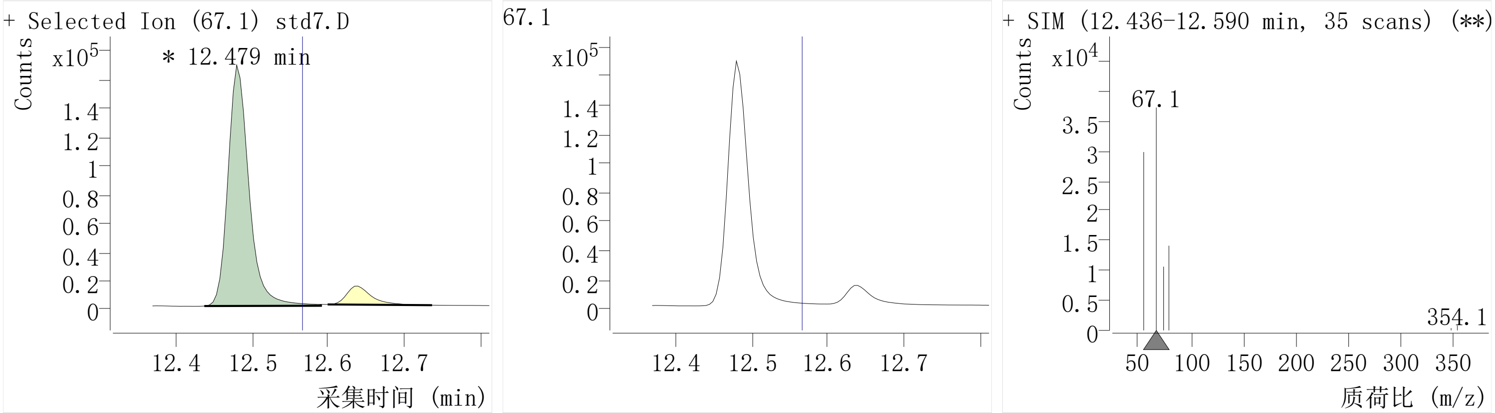

C23:0

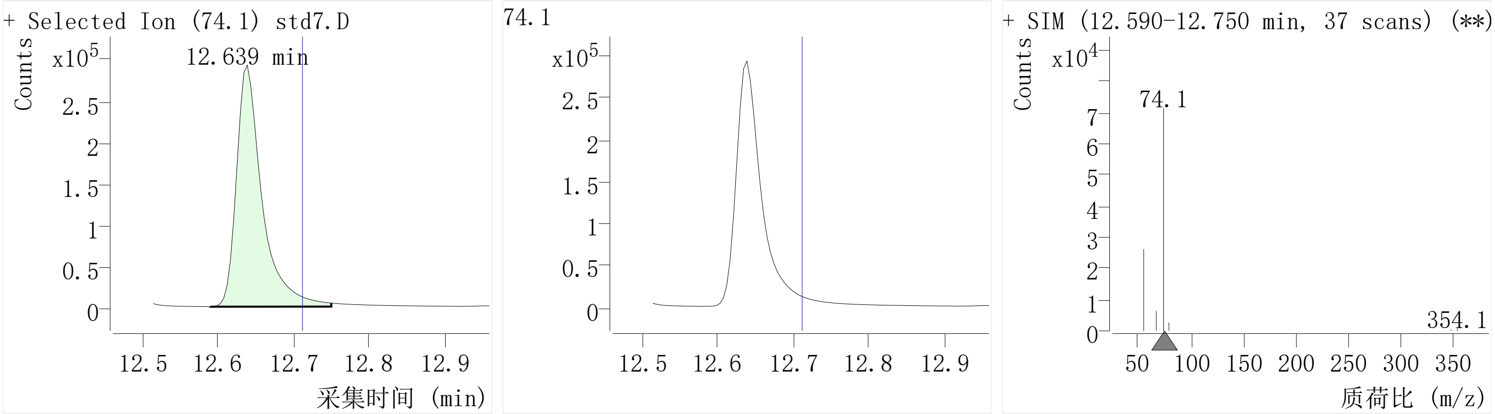

C24:0

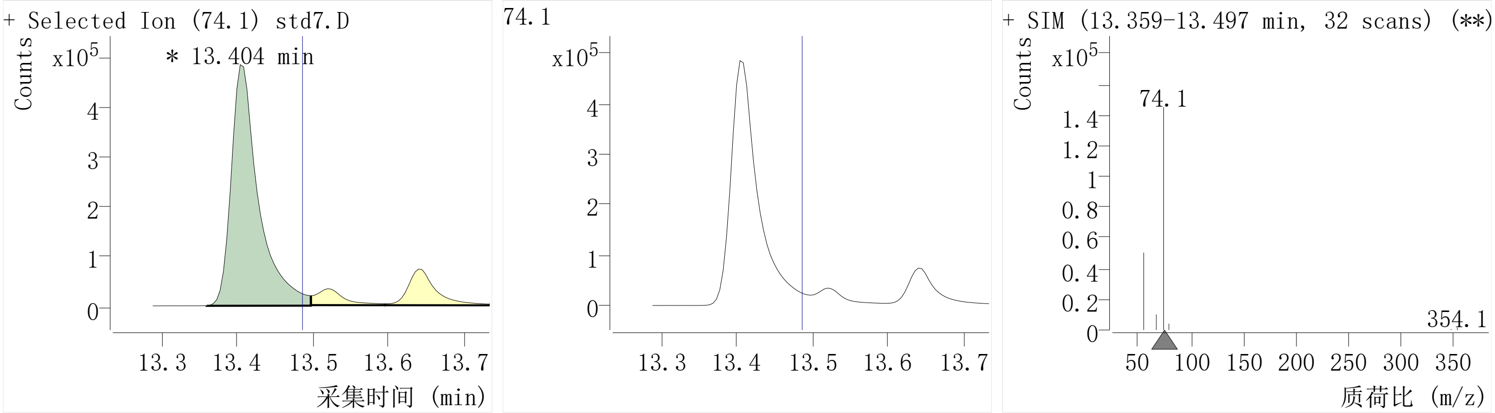

C22:6

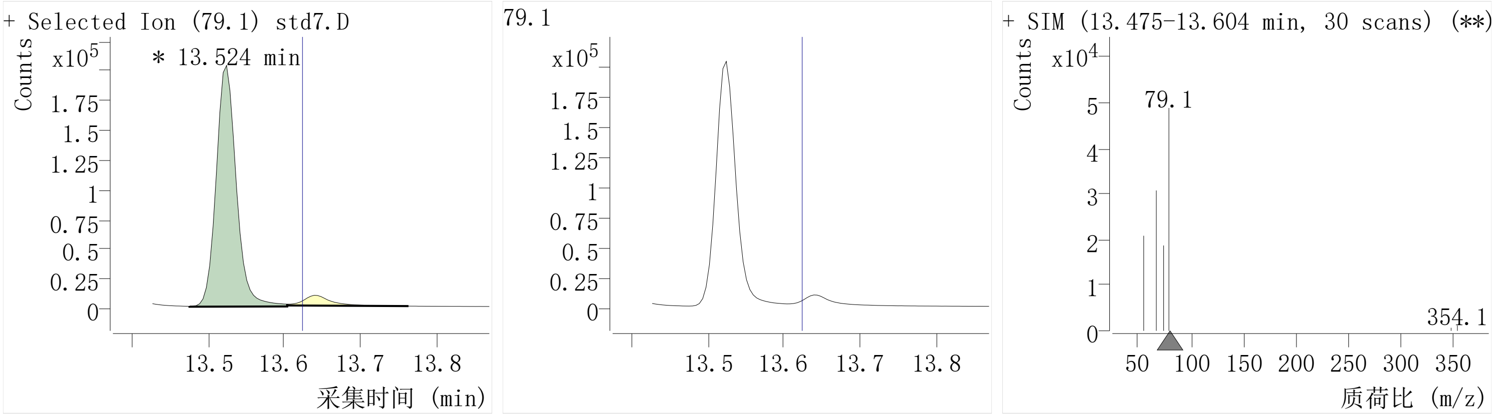
+ Selected Ion (79.1) std7.D
Counts
x10^5
* 13.524 min
1.75
1.5
1.25
1
0.75
0.5
0.25
0
13.5
13.6
13.7
13.8
采集时间 (min)
79.1
x10^5
1.75
1.5
1.25
1
0.75
0.5
0.25
0
13.5
13.6
13.7
13.8
+ SIM (13.475-13.604 min, 30 scans) (**)
Counts
x10^4
79.1
5
4
3
2
1
0
354.1
50
100
150
200
250
300
350
质荷比 (m/z)

C24:1

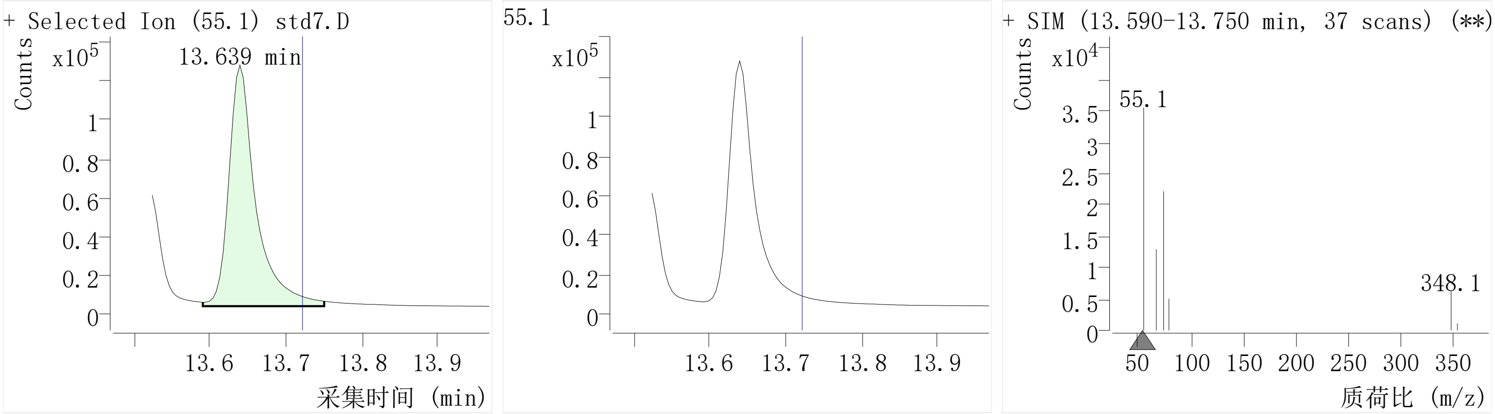
+ Selected Ion (55.1) std7.D
Counts
x10^5
13.639 min
1
0.8
0.6
0.4
0.2
0
13.6
13.7
13.8
13.9
采集时间 (min)
55.1
x10^5
1
0.8
0.6
0.4
0.2
0
13.6
13.7
13.8
13.9
+ SIM (13.590-13.750 min, 37 scans) (**)
Counts
x10^4
55.1
3.5
3
2.5
2
1.5
1
0.5
0
348.1
50
100
150
200
250
300
350
质荷比 (m/z)

定量分析完成报告

**批处理路径**	G:\GC-MS\HX250430-4-GCMS总脂肪酸靶向检测\HX250430-4\QuantResults\HX250430-4.batch.bin		
**分析时间**	2025/5/14 16:58	**分析员姓名**	DESKTOP-M3A0GPO\omics
**报告时间**	2025/5/16 14:53:09	**报告员姓名**	DESKTOP-M3A0GPO\omics
**最近校正更新**	2025/5/14 16:58	**批处理状态**	已处理
**定量批处理版本**	10.2	**定量报告版本**	10.2

**采集时间**	2025/5/8 19:50	**数据文件**	std8.D
**样品类型**	校正	**样品名称**	std8
**稀释**	1	**采集方法**	脂肪酸

样品色谱图

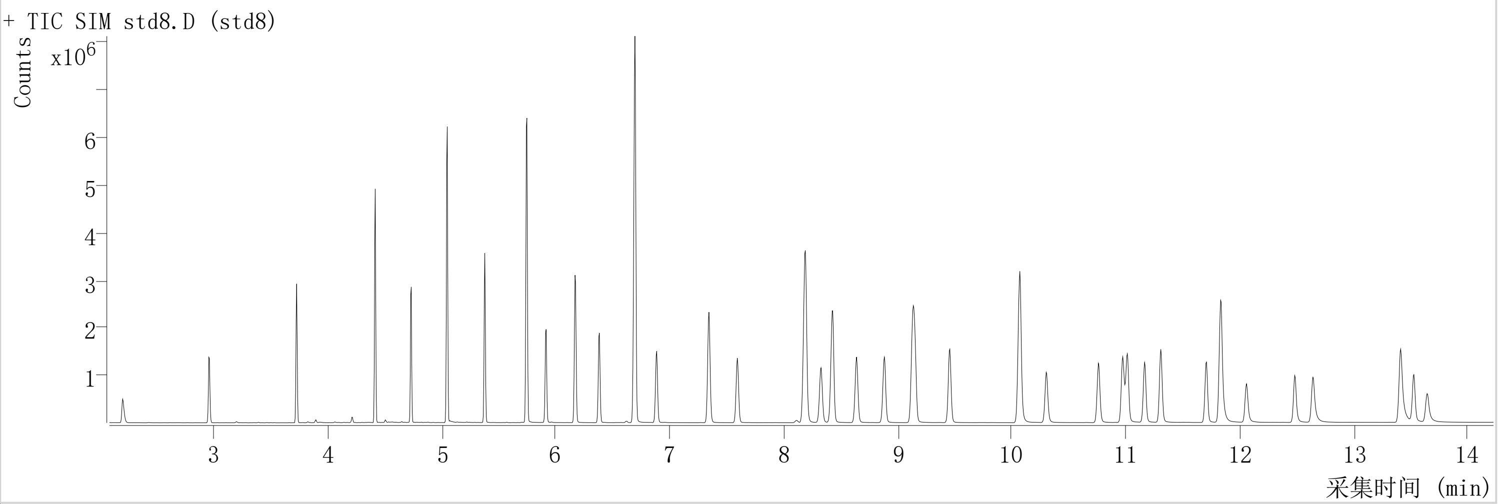

化合物	ISTD	RT	响应	ISTD 响应	响应比	最终浓度	单位
C4:0	C19:0	2.203	603804	2927430	0.2063	38.8753	ug/ml
C6:0	C19:0	2.959	1098736	2927430	0.3753	41.4364	ug/ml
C8:0	C19:0	3.728	1792155	2927430	0.6122	41.9072	ug/ml
C10:0	C19:0	4.417	2827697	2927430	0.9659	39.2609	ug/ml
C11:0	C19:0	4.733	1728180	2927430	0.5903	19.6385	ug/ml
C12:0	C19:0	5.049	3824034	2927430	1.3063	38.5262	ug/ml
C13:0	C19:0	5.378	2186179	2927430	0.7468	19.3873	ug/ml
C14:0	C19:0	5.747	4648797	2927430	1.5880	36.9235	ug/ml
C14:1	C19:0	5.911	965001	2927430	0.3296	16.5208	ug/ml
C15:0	C19:0	6.169	2539594	2927430	0.8675	19.4503	ug/ml
C15:1	C19:0	6.378	1114045	2927430	0.3806	19.3995	ug/ml
C16:0	C19:0	6.694	7743304	2927430	2.6451	67.9105	ug/ml
C16:1	C19:0	6.885	913884	2927430	0.3122	18.9893	ug/ml
C17:0	C19:0	7.343	2695183	2927430	0.9207	19.2078	ug/ml
C17:1	C19:0	7.592	1053004	2927430	0.3597	19.3441	ug/ml
C18:0	C19:0	8.188	5409063	2927430	1.8477	40.1227	ug/ml
C18:1n9t	C19:0	8.326	987844	2927430	0.3374	19.9057	ug/ml
C18:1n9c	C19:0	8.424	2049949	2927430	0.7003	42.2495	ug/ml
C18:2n6t	C19:0	8.637	1109192	2927430	0.3789	20.7899	ug/ml
C18:2n6c	C19:0	8.882	1101305	2927430	0.3762	20.2925	ug/ml
C18:3n6	C19:0	9.149	1055796	2927430	0.3607	19.3421	ug/ml
C18:3n3	C19:0	9.455	1222850	2927430	0.4177	20.7621	ug/ml
C20:0	C19:0	10.069	4700404	2927430	1.6056	40.5351	ug/ml
C20:1	C19:0	10.300	984696	2927430	0.3364	20.7685	ug/ml
C20:2	C19:0	10.758	983181	2927430	0.3359	20.5772	ug/ml
C21:0	C19:0	10.972	2175424	2927430	0.7431	20.4432	ug/ml
C20:3n6	C19:0	11.012	950912	2927430	0.3248	20.5590	ug/ml
C20:4n6	C19:0	11.163	972850	2927430	0.3323	20.7575	ug/ml
C20:3n3	C19:0	11.305	1152681	2927430	0.3938	20.6370	ug/ml
C20:5n3	C19:0	11.705	1089649	2927430	0.3722	20.6068	ug/ml

定量分析完成报告

化合物	ISTD	RT	响应	ISTD 响应	响应比	最终浓度	单位
C22:0	C19:0	11.830	3679650	2927430	1.2570	40.7174	ug/ml
C22:1n9	C19:0	12.057	820737	2927430	0.2804	20.7408	ug/ml
C22:2n6	C19:0	12.479	766807	2927430	0.2619	20.7371	ug/ml
C23:0	C19:0	12.639	1556318	2927430	0.5316	20.5117	ug/ml
C24:0	C19:0	13.408	2700382	2927430	0.9224	41.6011	ug/ml
C22:6	C19:0	13.524	896402	2927430	0.3062	22.6248	ug/ml
C24:1	C19:0	13.640	688642	2927430	0.2352	20.7429	ug/ml

C4:0

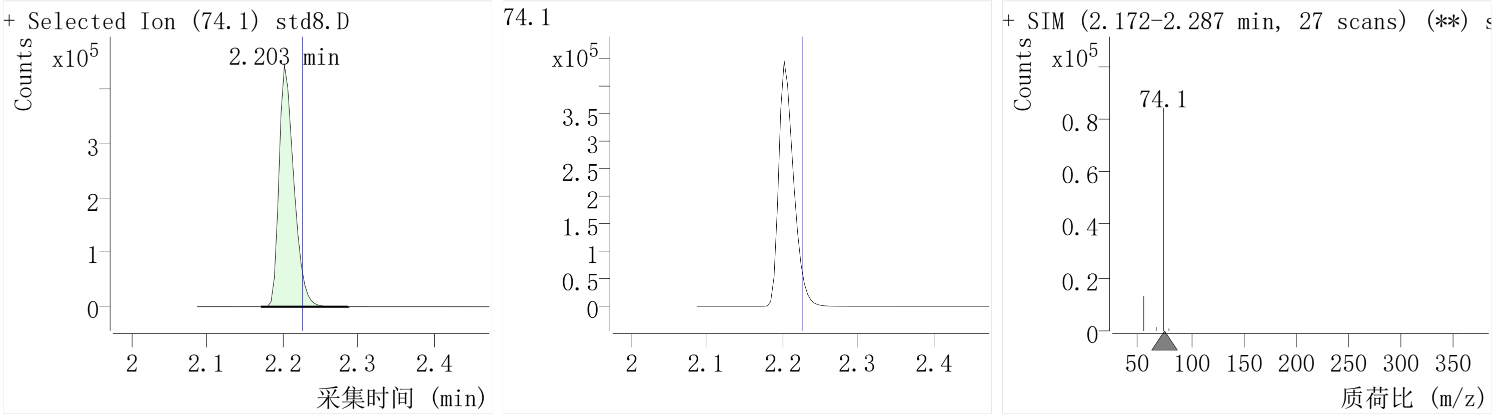

C6:0

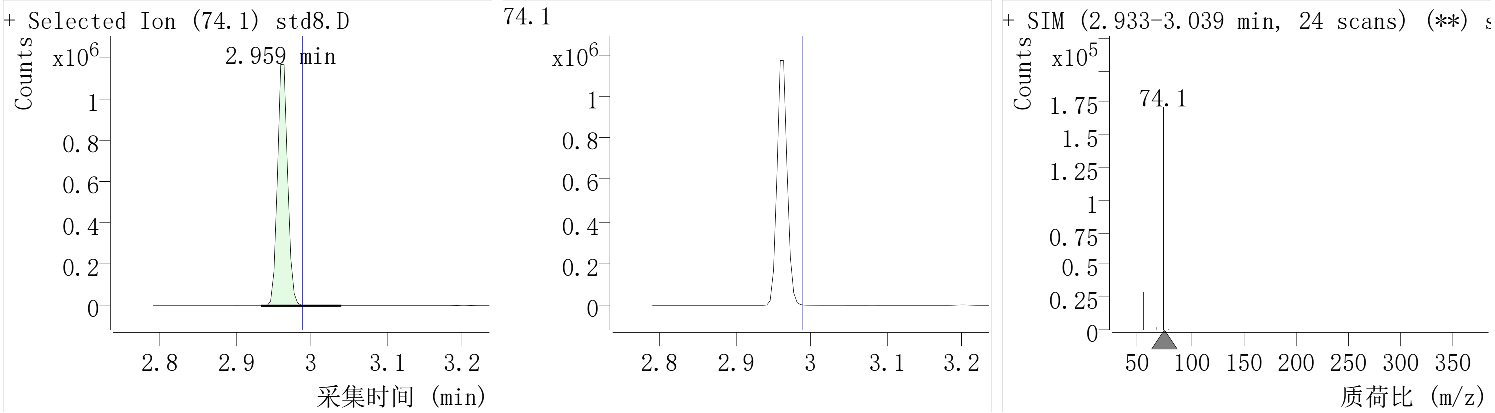

C8:0

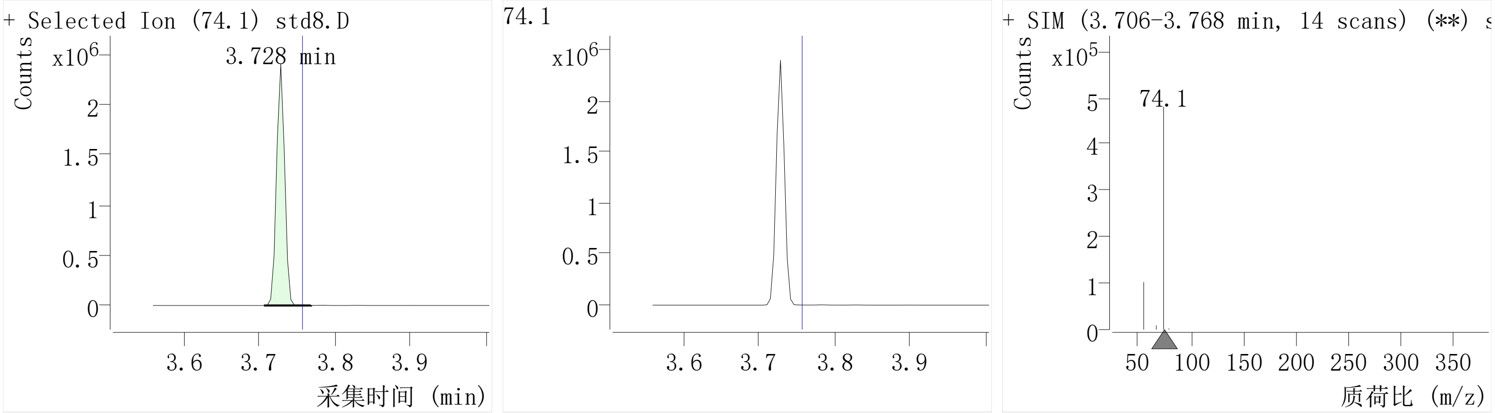

C10:0

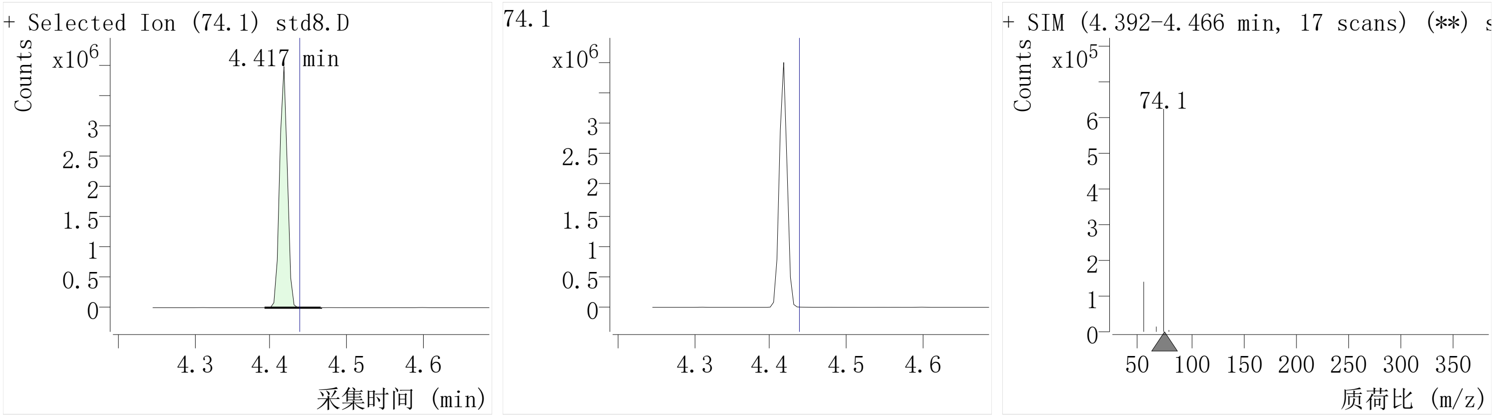

C11:0

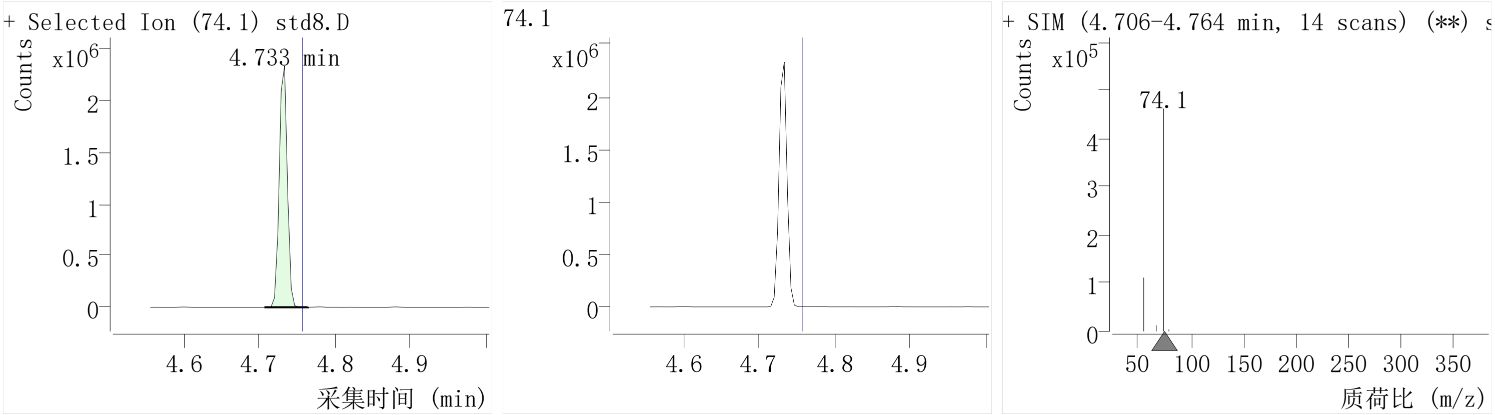

C12:0

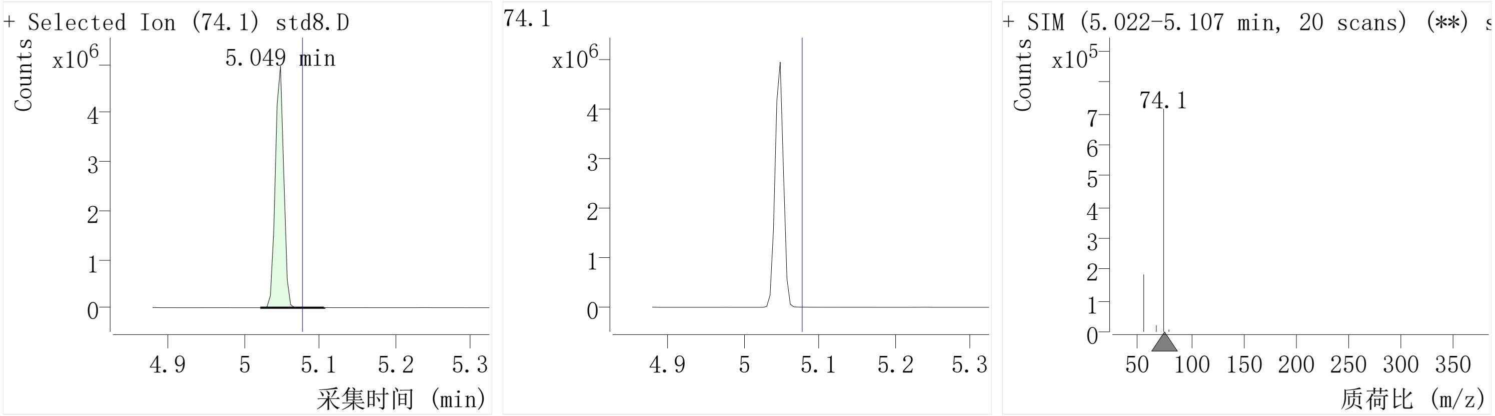

C13:0

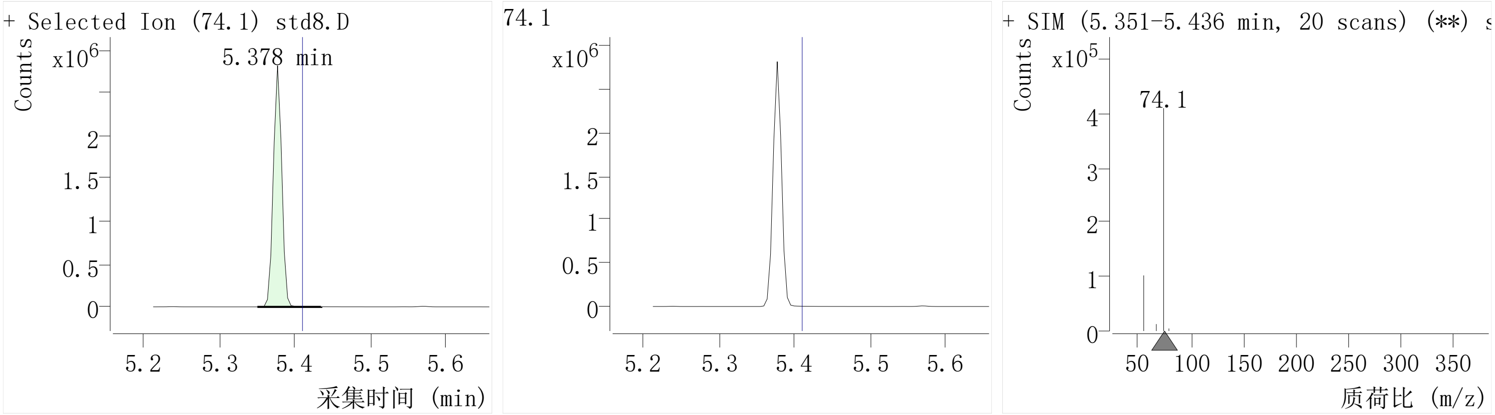

C14:0

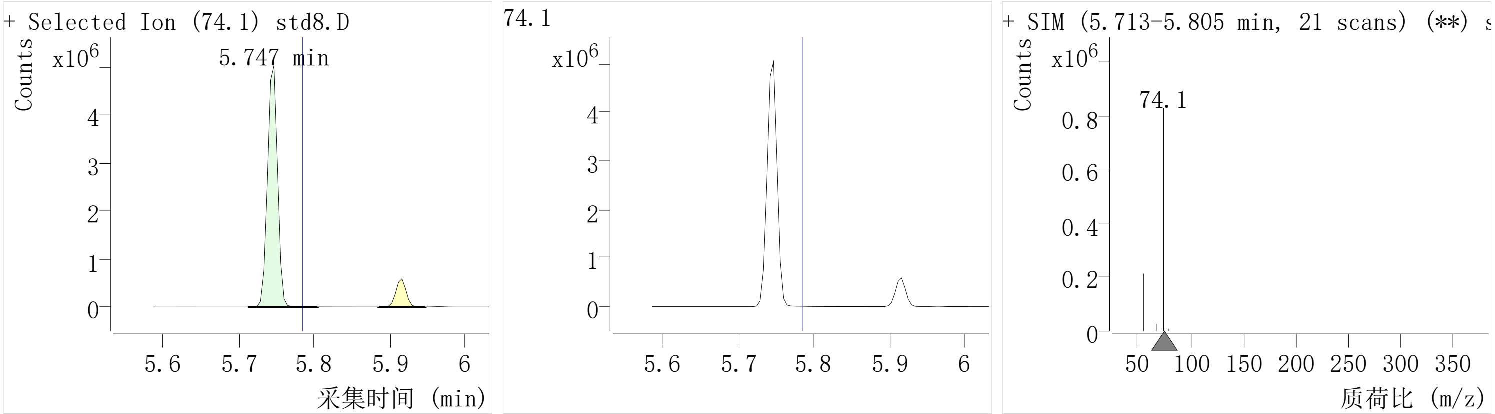

C14:1

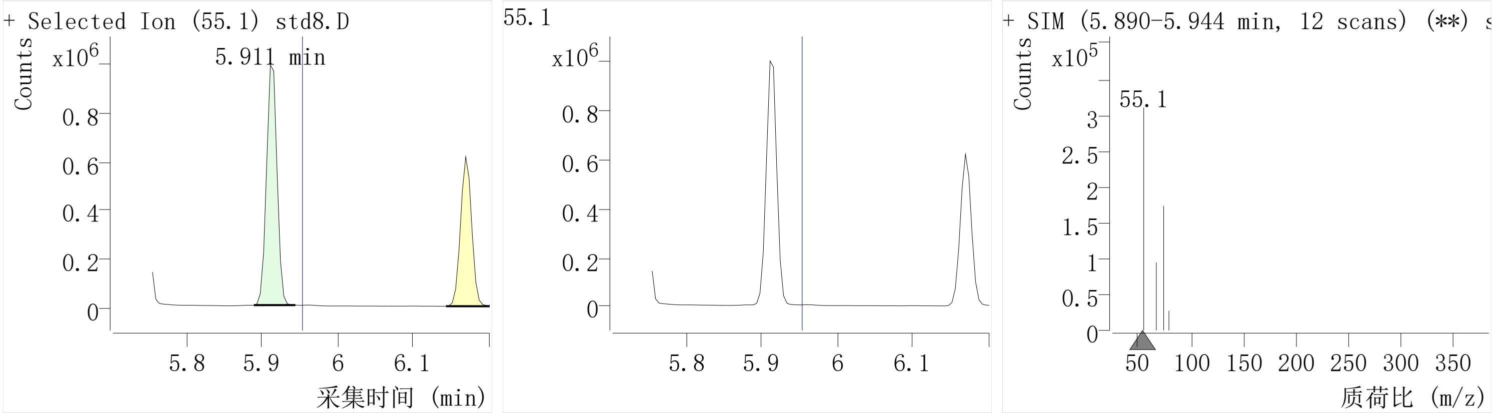

C15:0

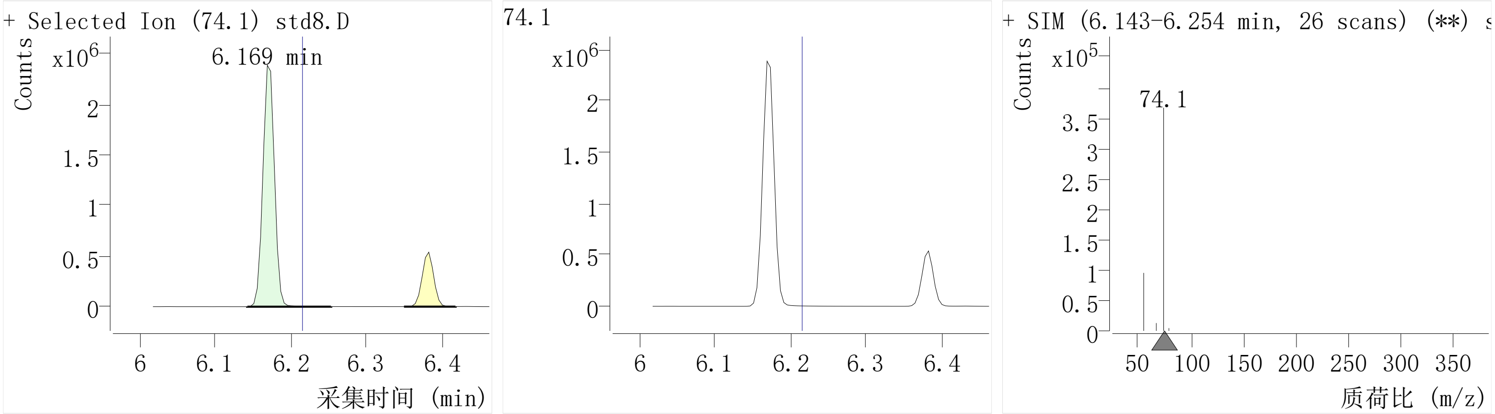

C15:1

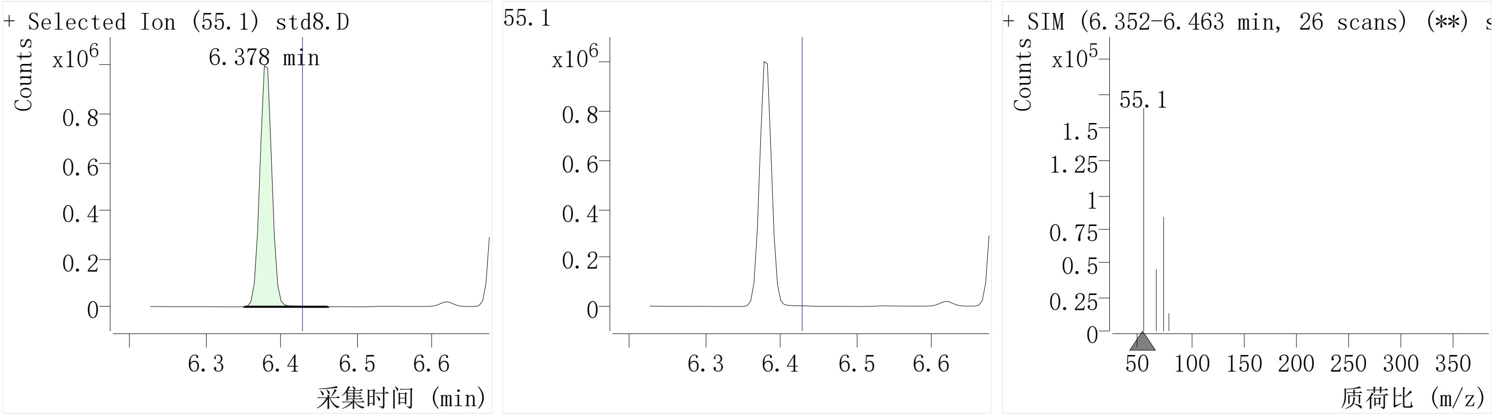

C16:0

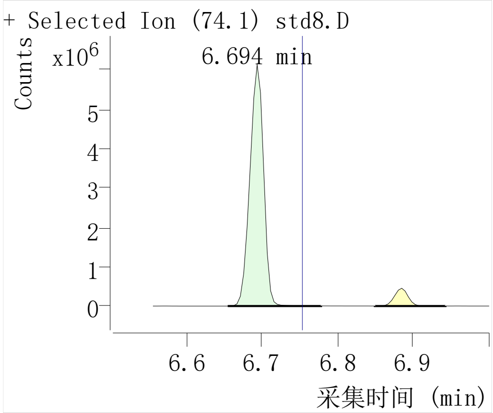

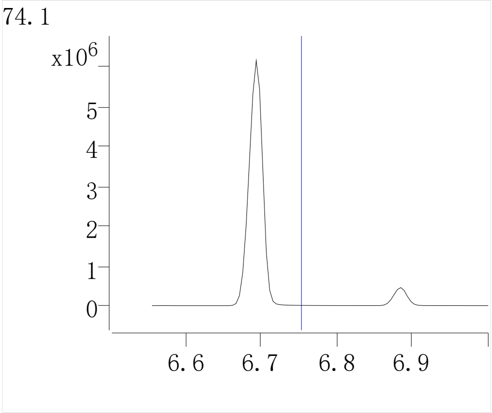

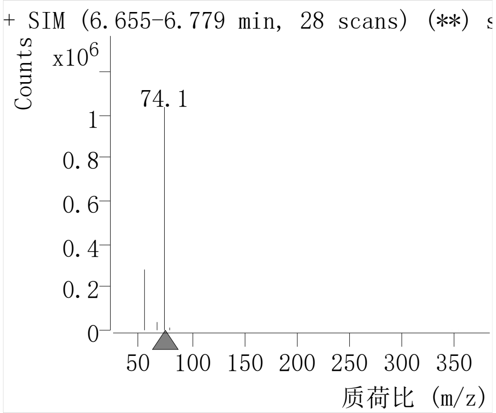

C16:1

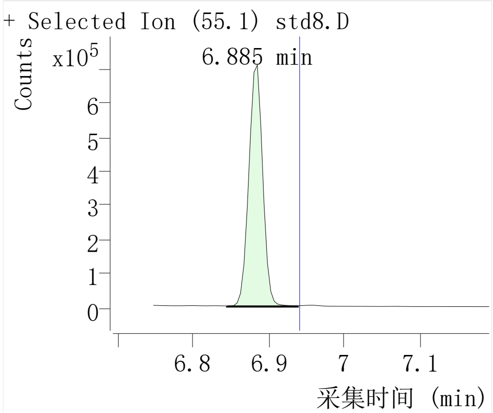

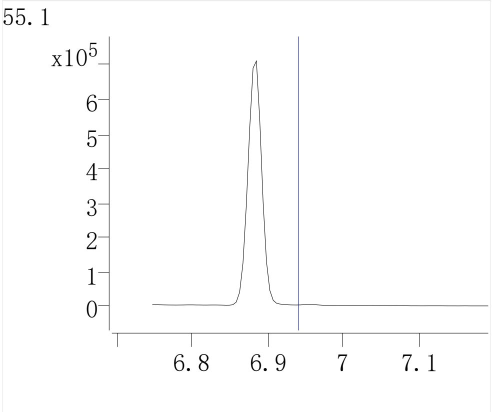

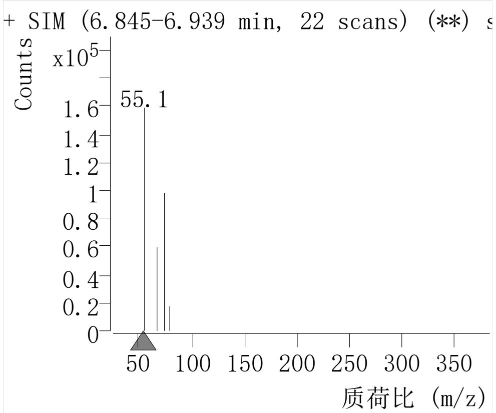

C17:0

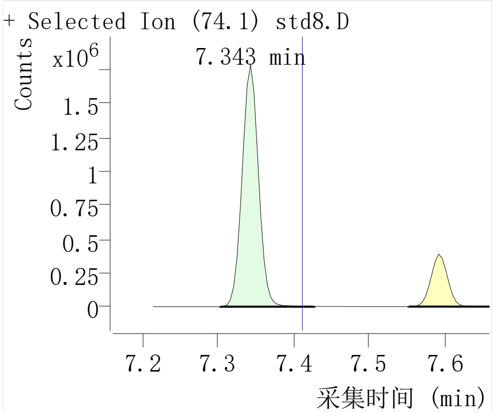

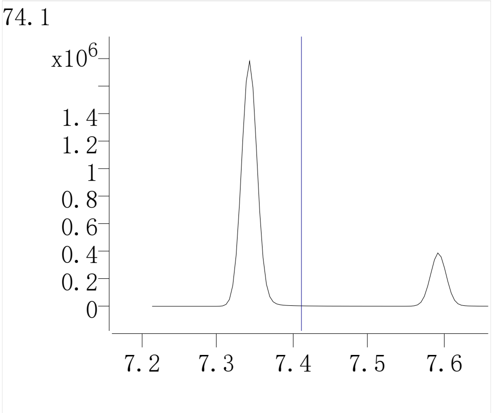

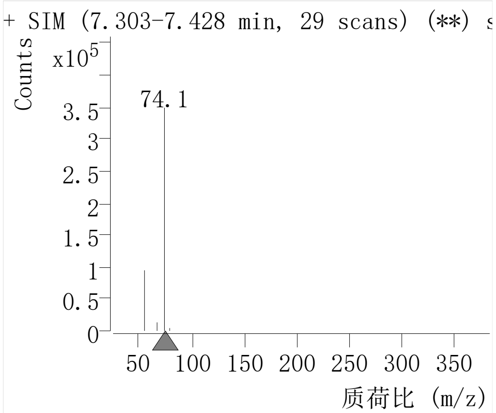

C17:1

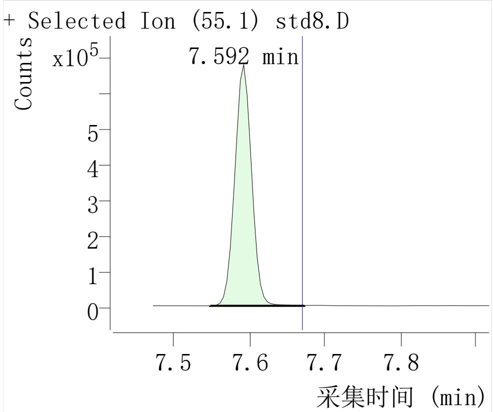

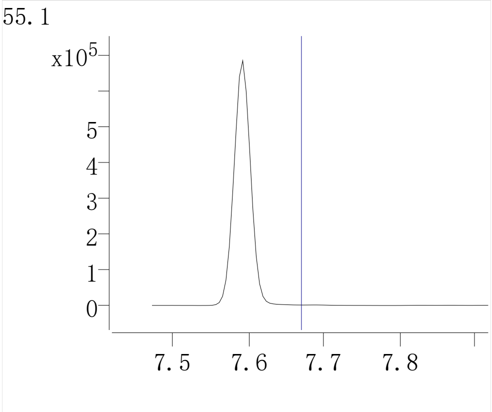

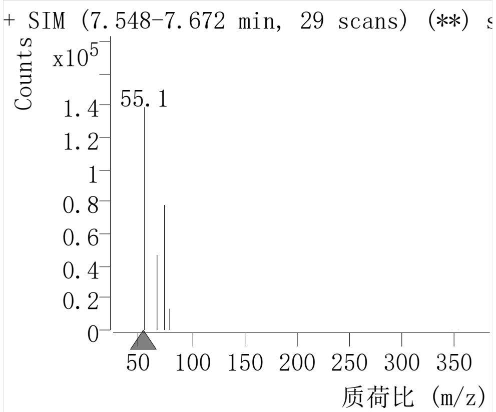

C18:0

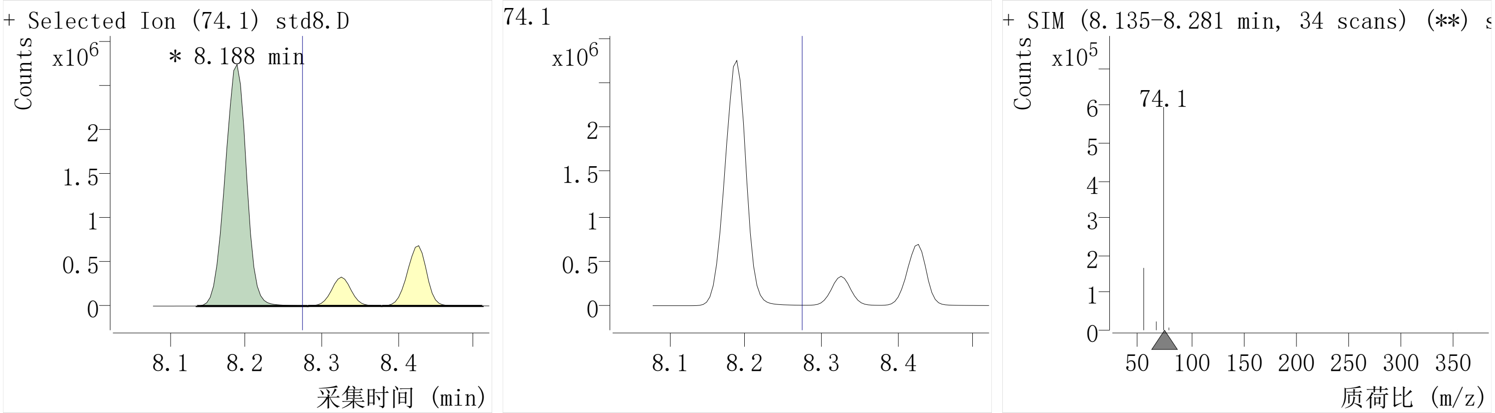

C18:1n9t

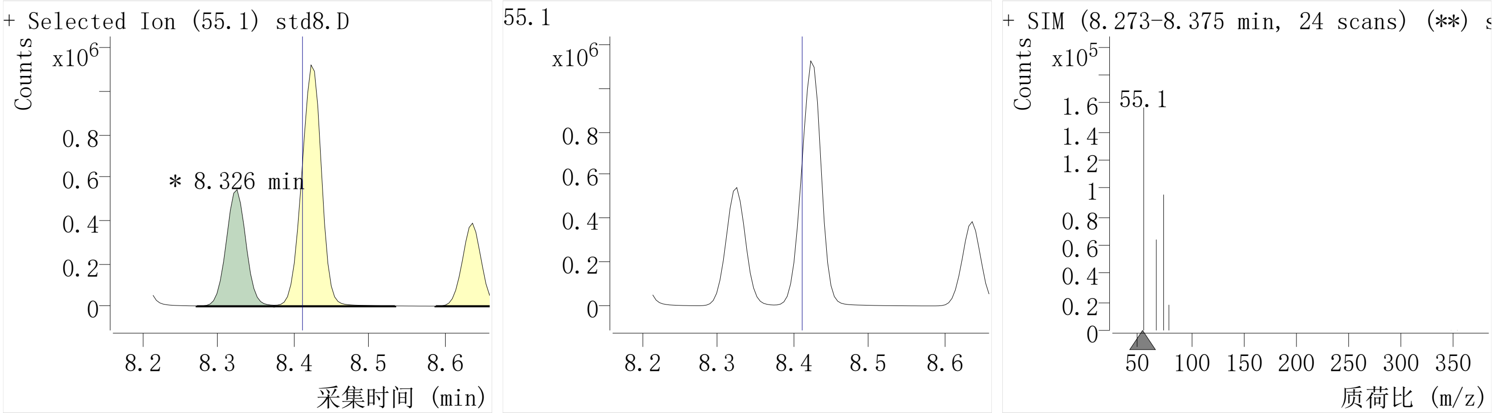

C18:1n9c

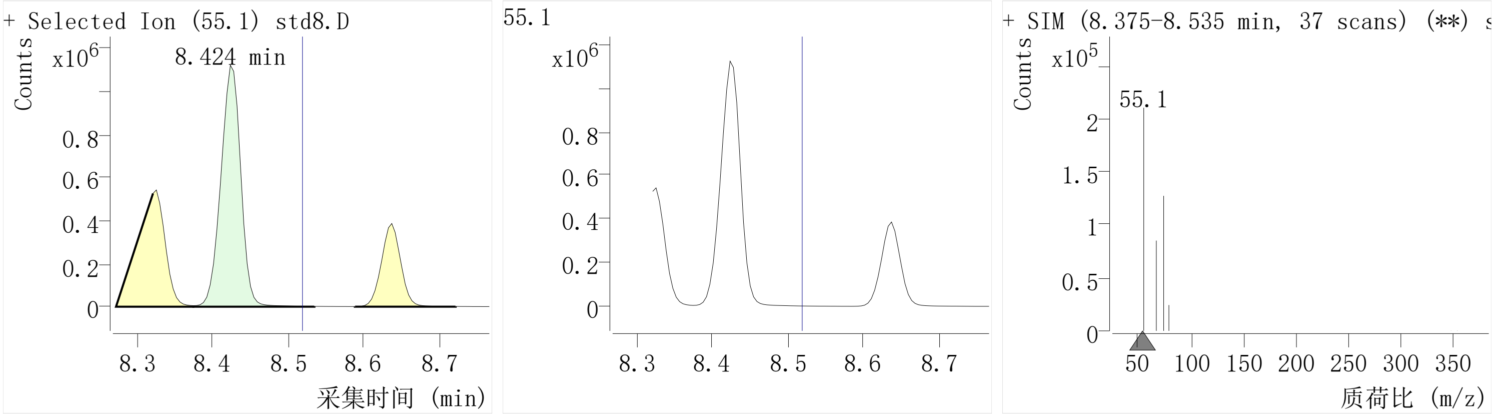

C18:2n6t

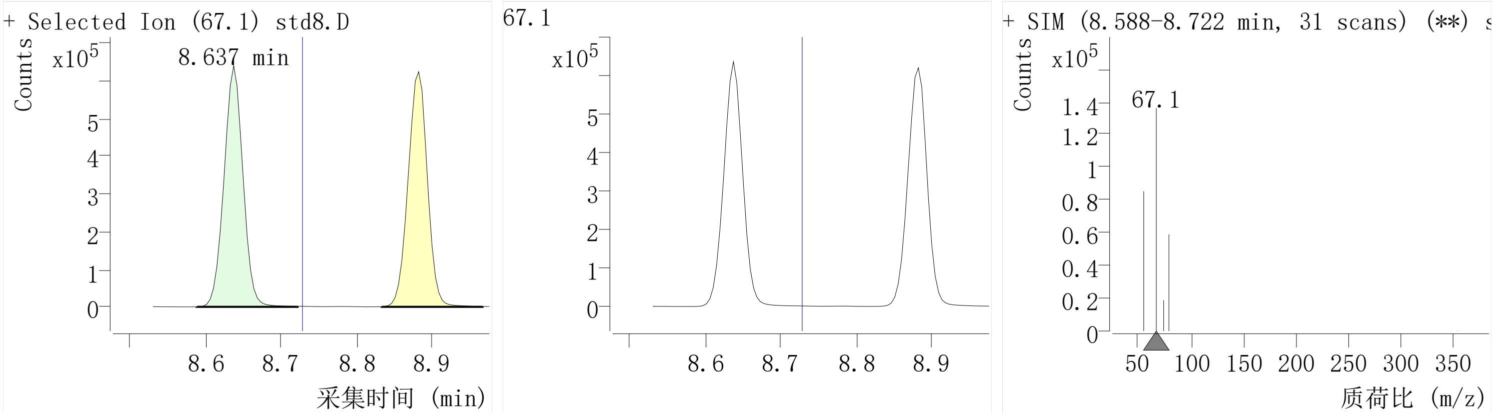

C18:2n6c

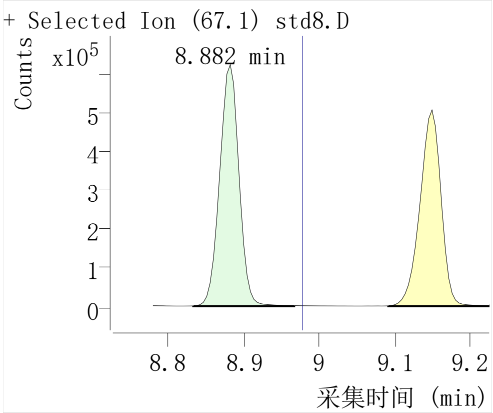

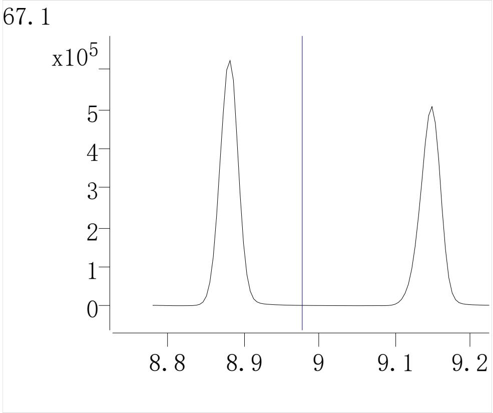

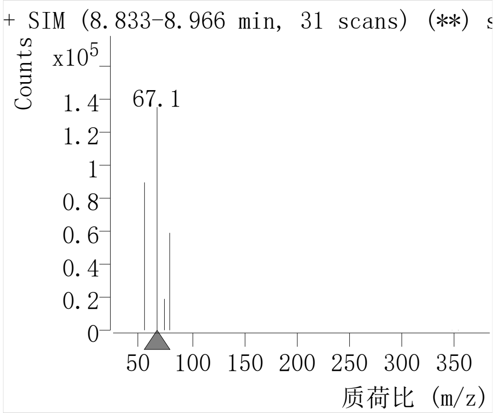

C18:3n6

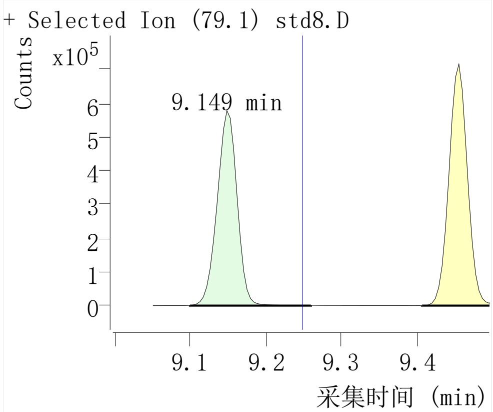

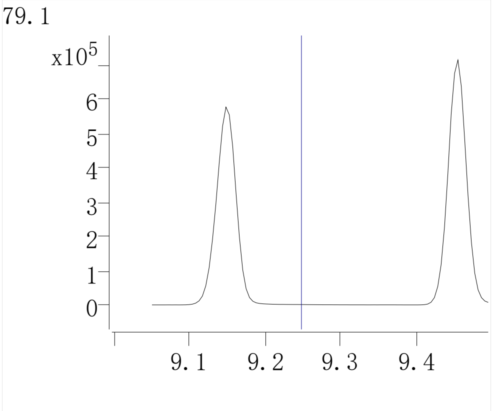

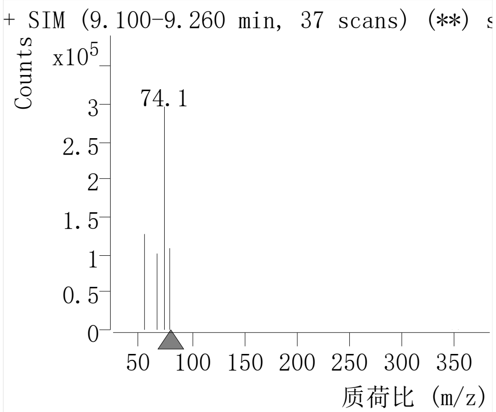

C18:3n3

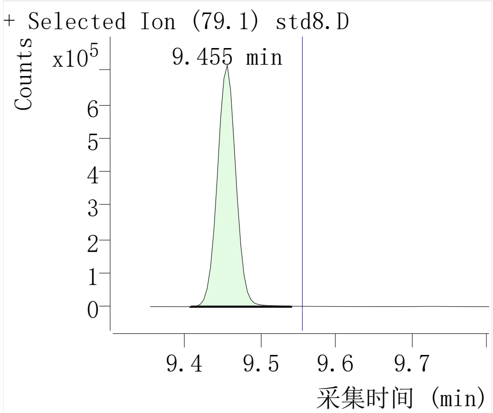

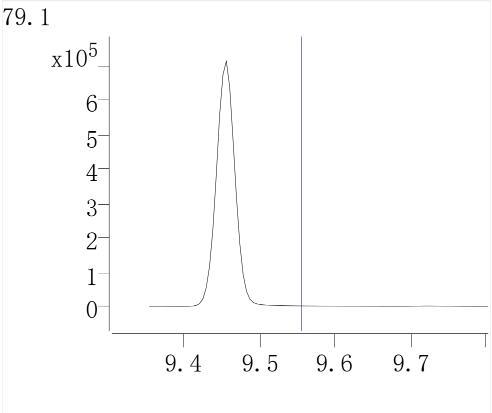

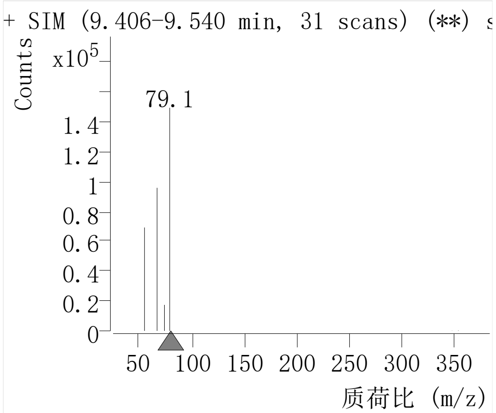

C20:0

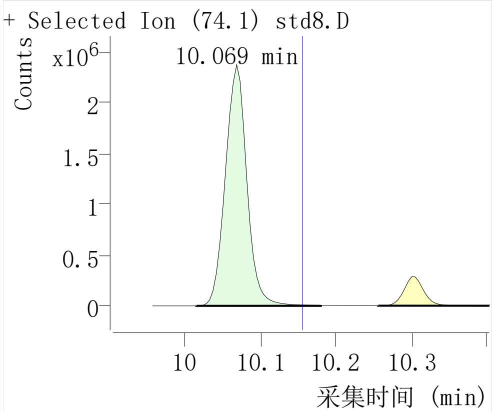

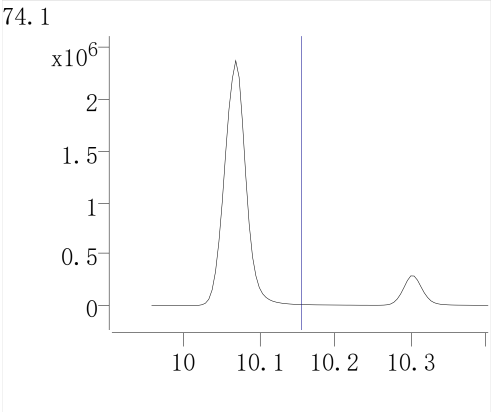

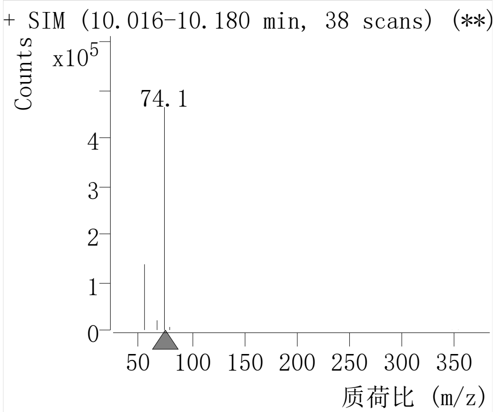

C20:1

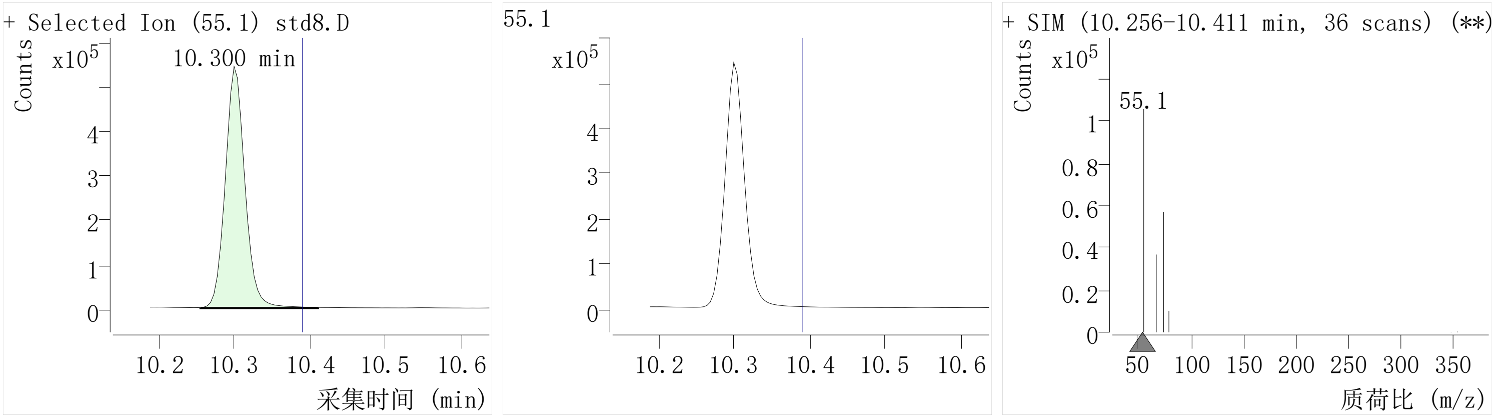

C20:2

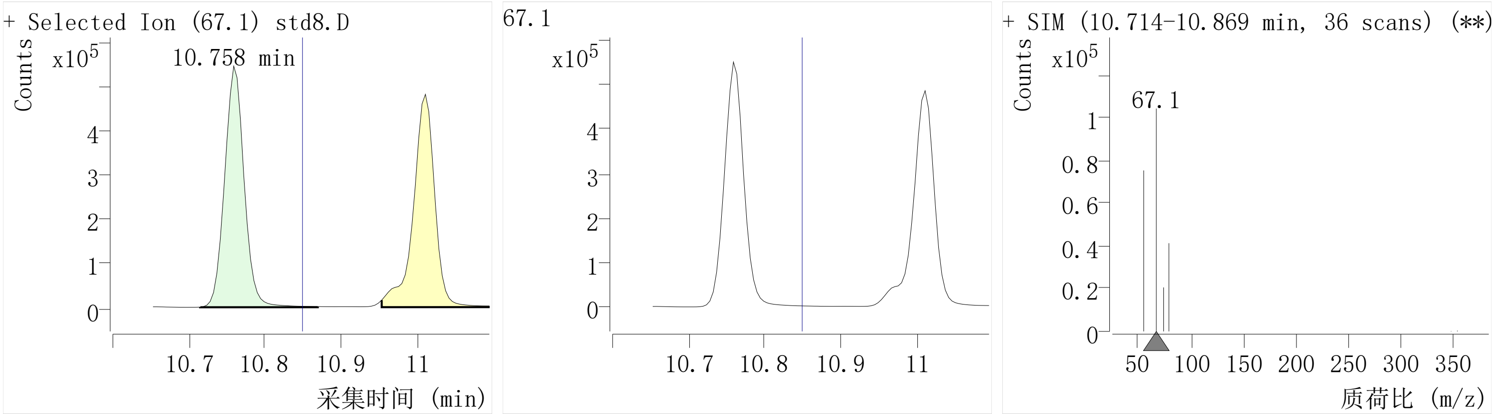

C21:0

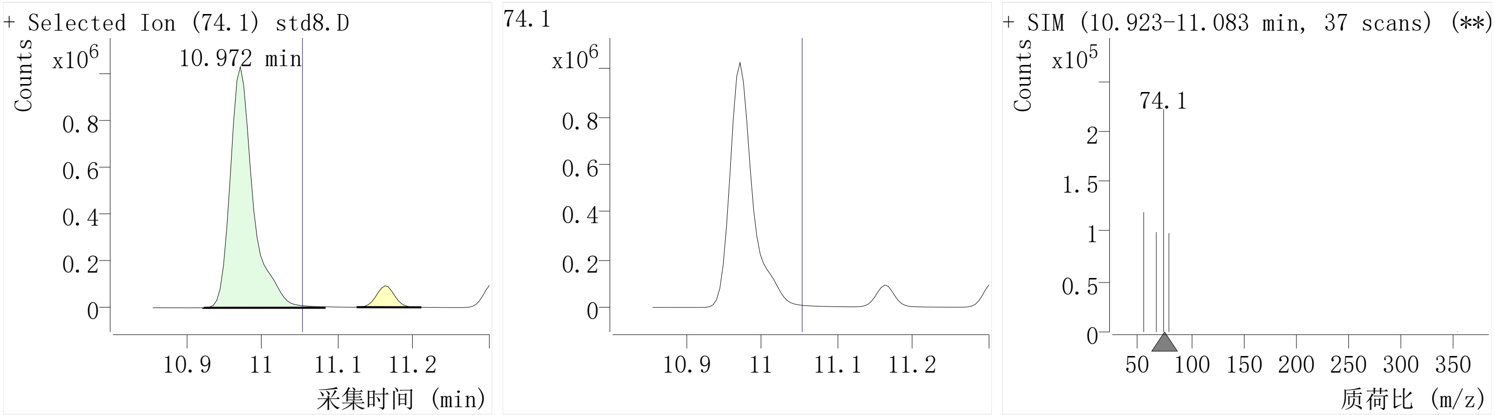

C20:3n6

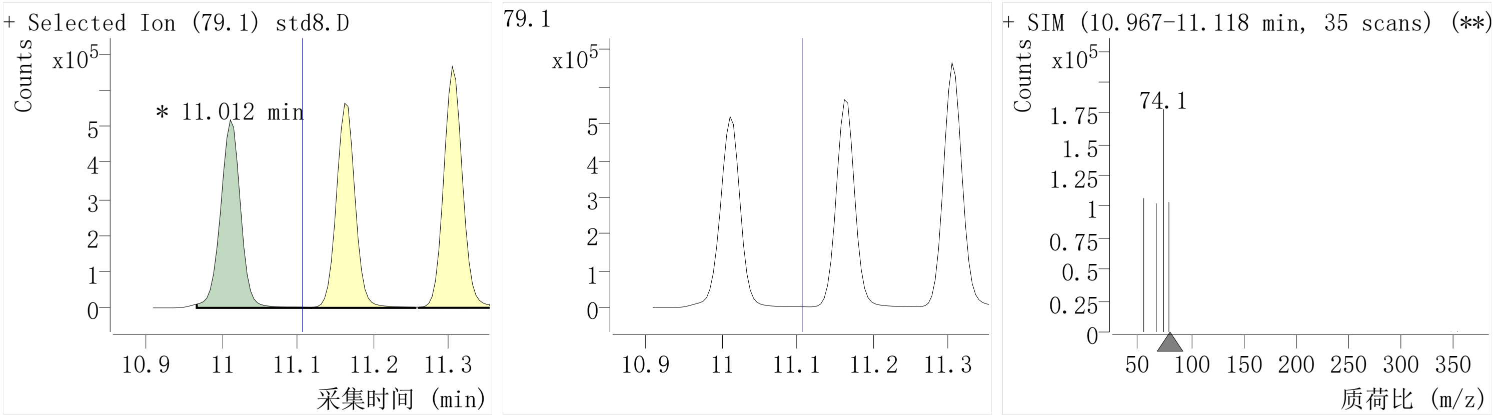

C20:4n6

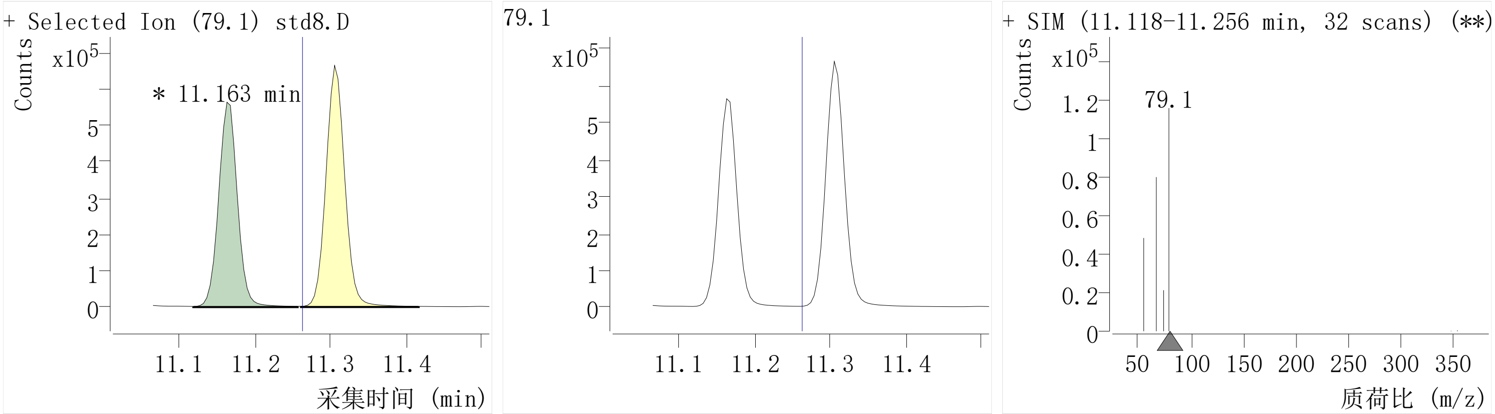

C20:3n3

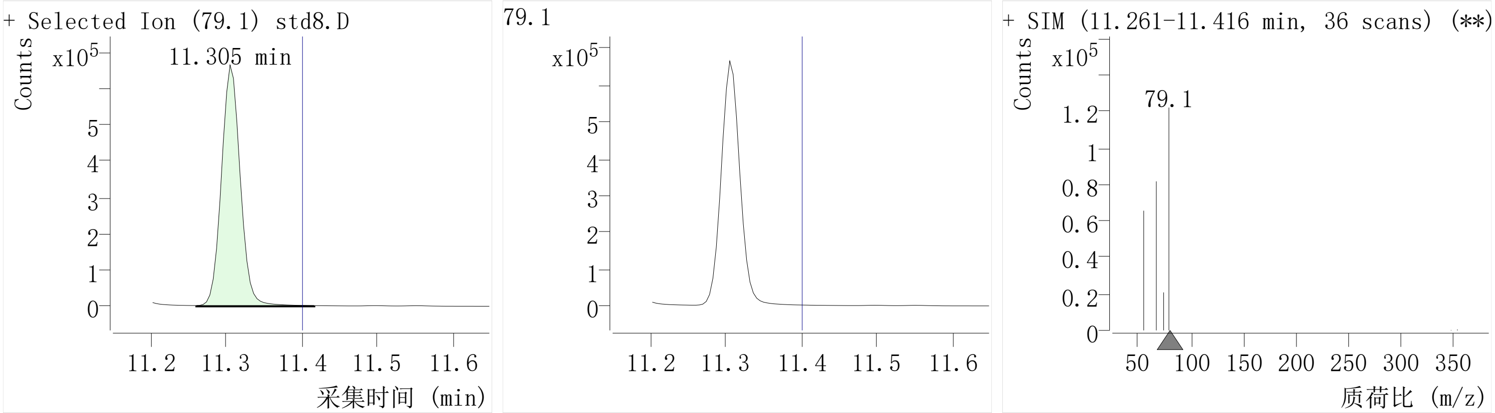

C20:5n3

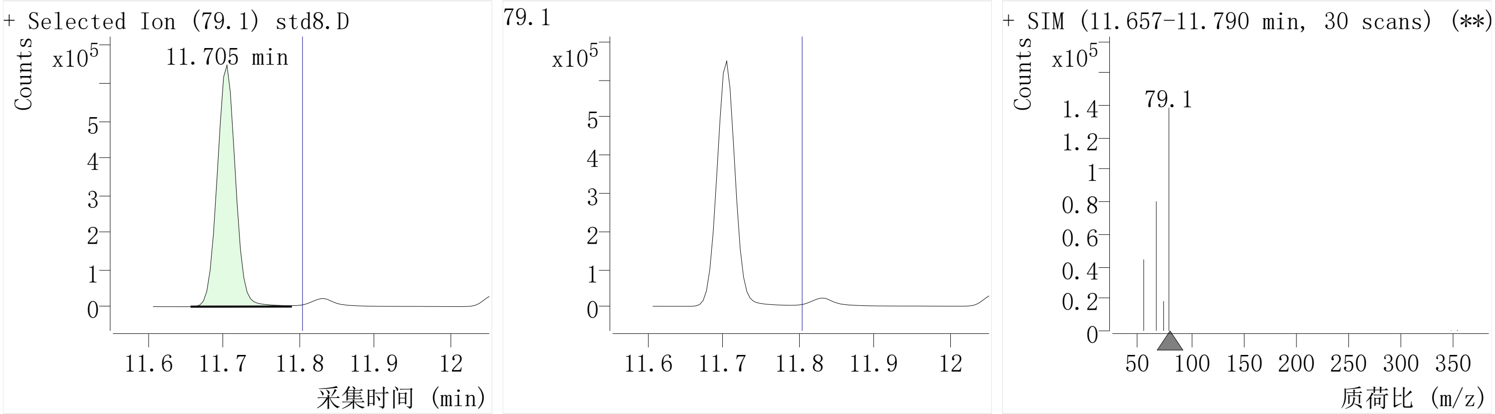

C22:0

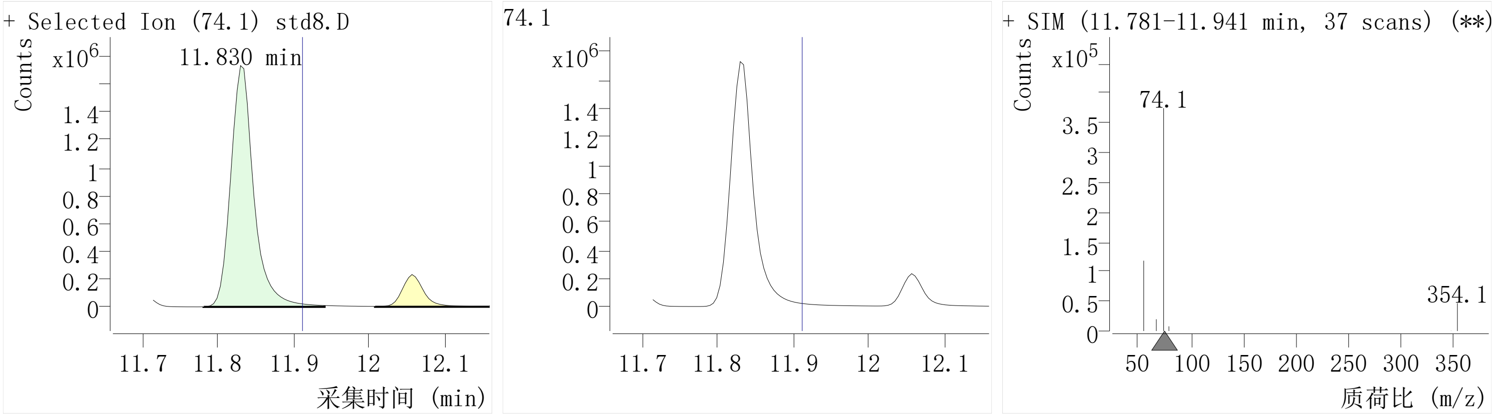

C22:1n9

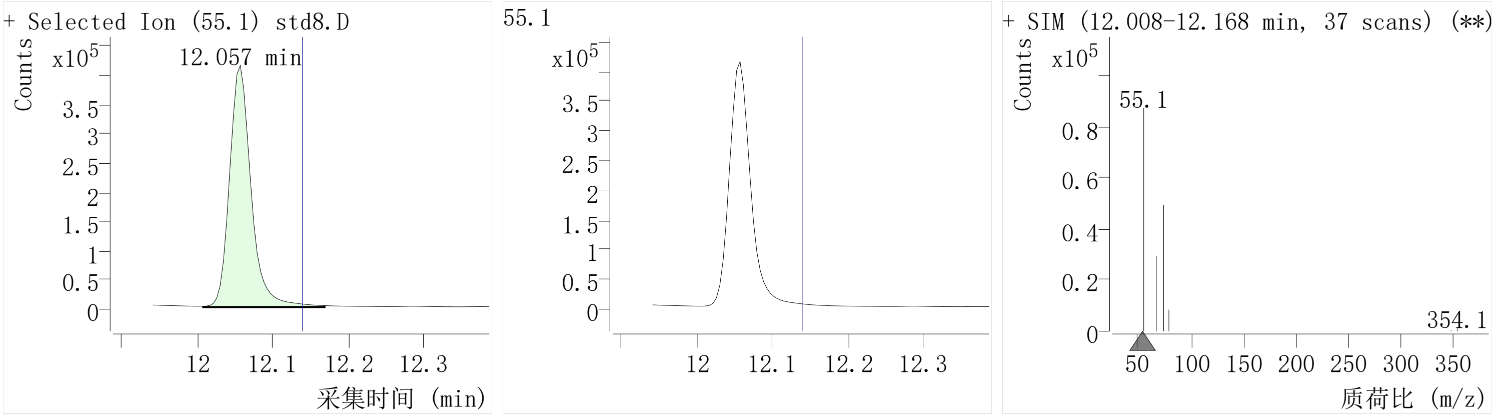

C22:2n6

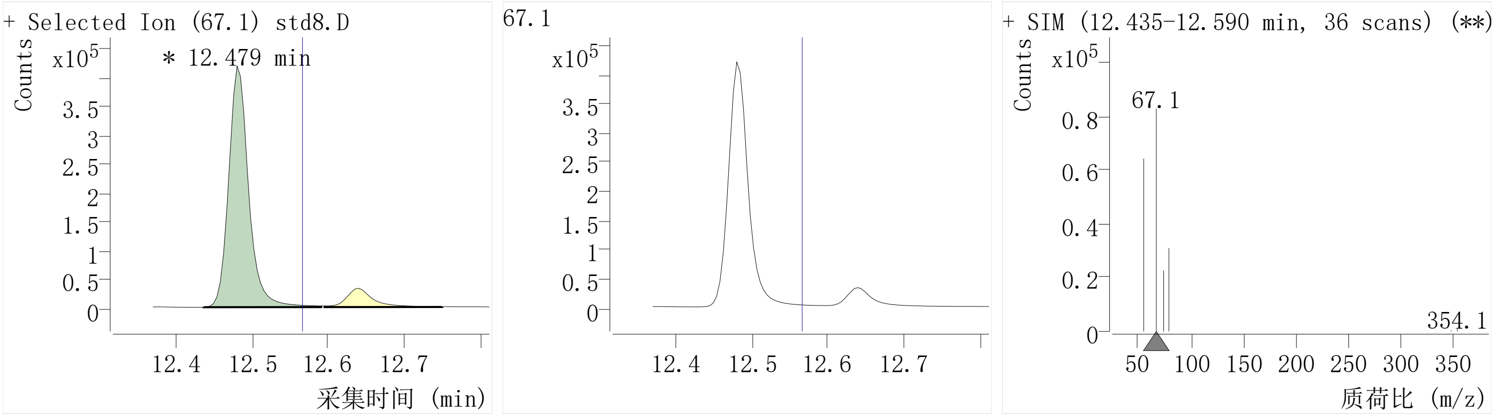

C23:0

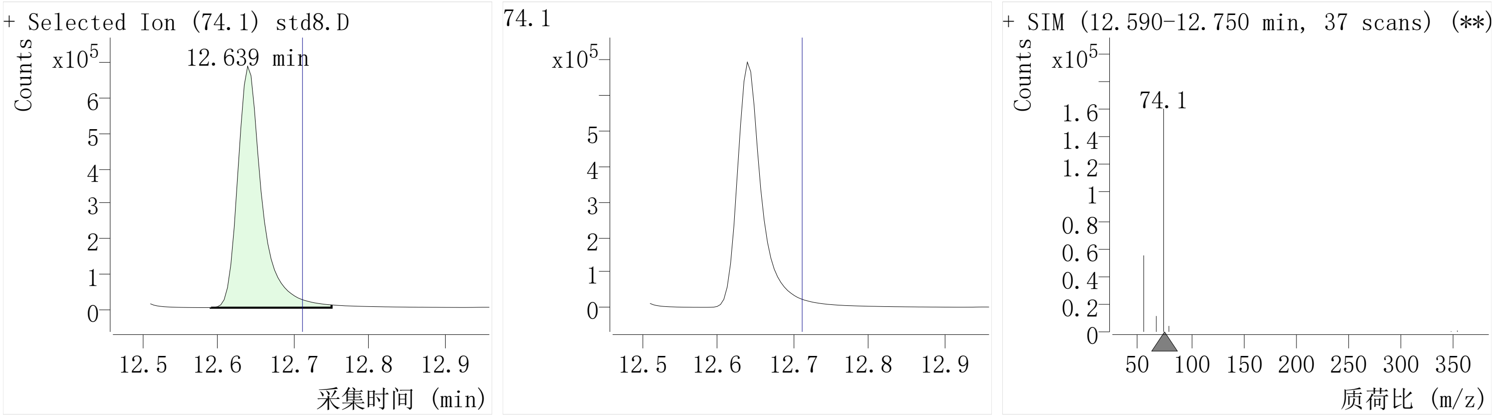

C24:0

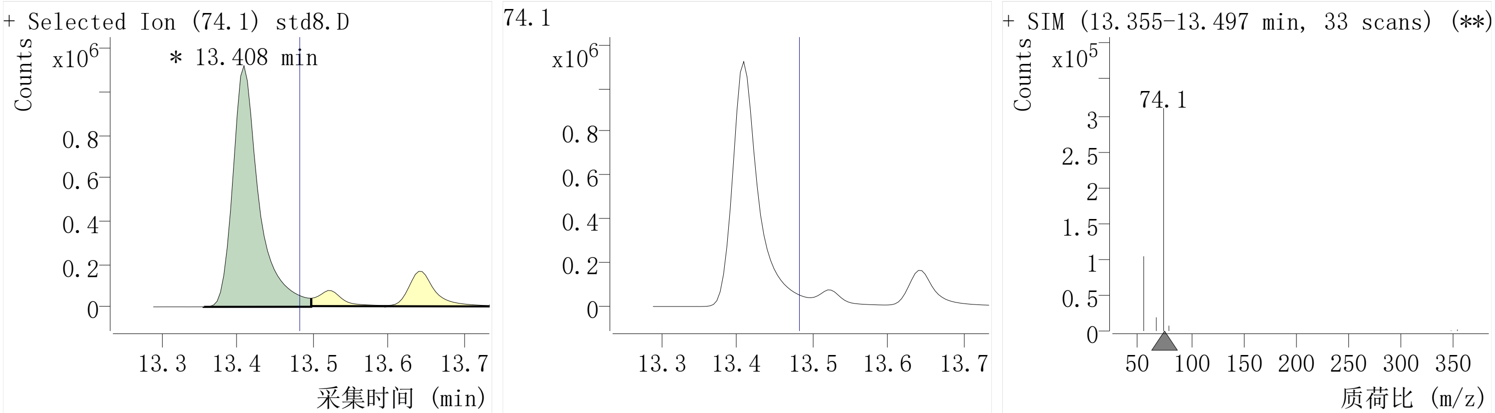

C22:6

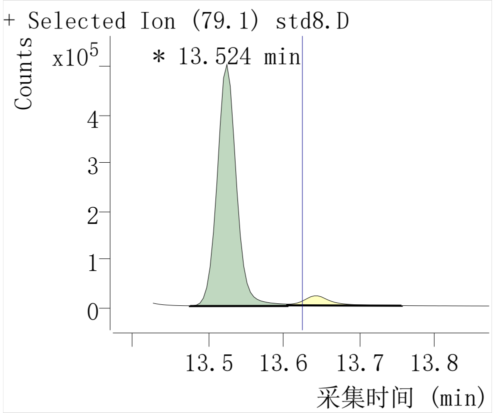
+ Selected Ion (79.1) std8.D
Counts
x10^5
* 13.524 min
4
3
2
1
0
13.5
13.6
13.7
13.8
采集时间 (min)

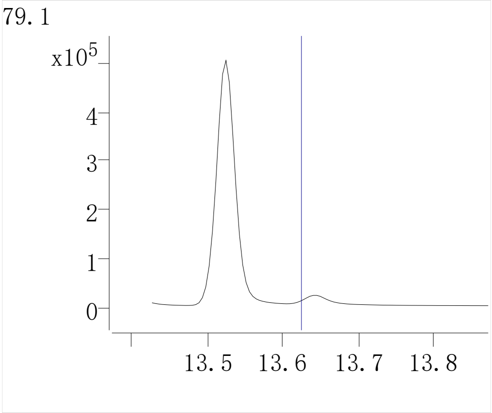
79.1
x10^5
4
3
2
1
0
13.5
13.6
13.7
13.8

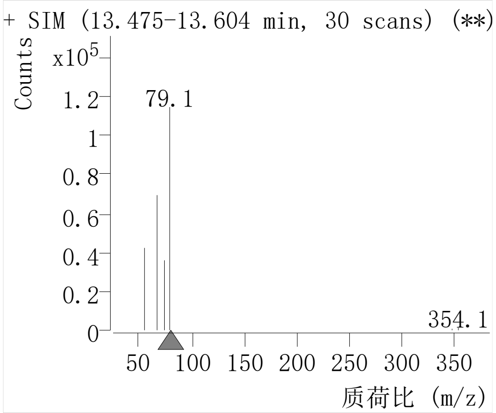
+ SIM (13.475-13.604 min, 30 scans) (**)
Counts
x10^5
1.2
1
0.8
0.6
0.4
0.2
0
79.1
354.1
50
100
150
200
250
300
350
质荷比 (m/z)

C24:1

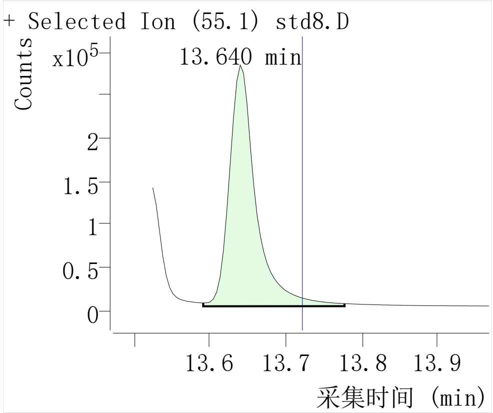
+ Selected Ion (55.1) std8.D
Counts
x10^5
13.640 min
2
1.5
1
0.5
0
13.6
13.7
13.8
13.9
采集时间 (min)

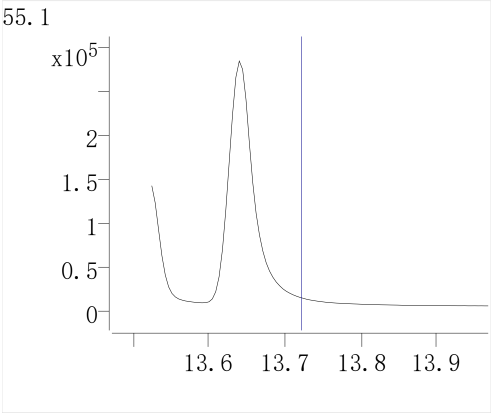
55.1
x10^5
2
1.5
1
0.5
0
13.6
13.7
13.8
13.9

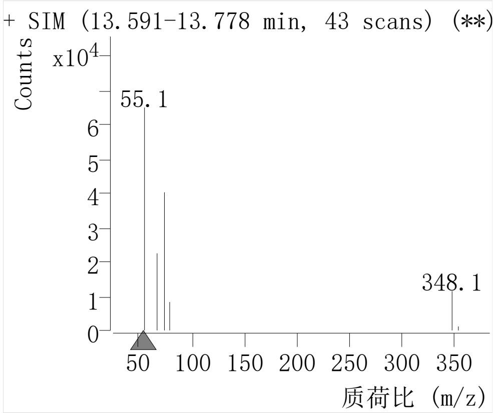
+ SIM (13.591-13.778 min, 43 scans) (**)
Counts
x10^4
55.1
6
5
4
3
2
1
0
348.1
50
100
150
200
250
300
350
质荷比 (m/z)

定量分析完成报告

**批处理路径**	G:\GC-MS\HX250430-4-GCMS总脂肪酸靶向检测\HX250430-4\QuantResults\HX250430-4.batch.bin		
**分析时间**	2025/5/14 16:58	**分析员姓名**	DESKTOP-M3A0GPO\omics
**报告时间**	2025/5/16 14:53:10	**报告员姓名**	DESKTOP-M3A0GPO\omics
**最近校正更新**	2025/5/14 16:58	**批处理状态**	已处理
**定量批处理版本**	10.2	**定量报告版本**	10.2

**采集时间**	2025/5/8 20:10	**数据文件**	std9.D
**样品类型**	校正	**样品名称**	std9
**稀释**	1	**采集方法**	脂肪酸

样品色谱图

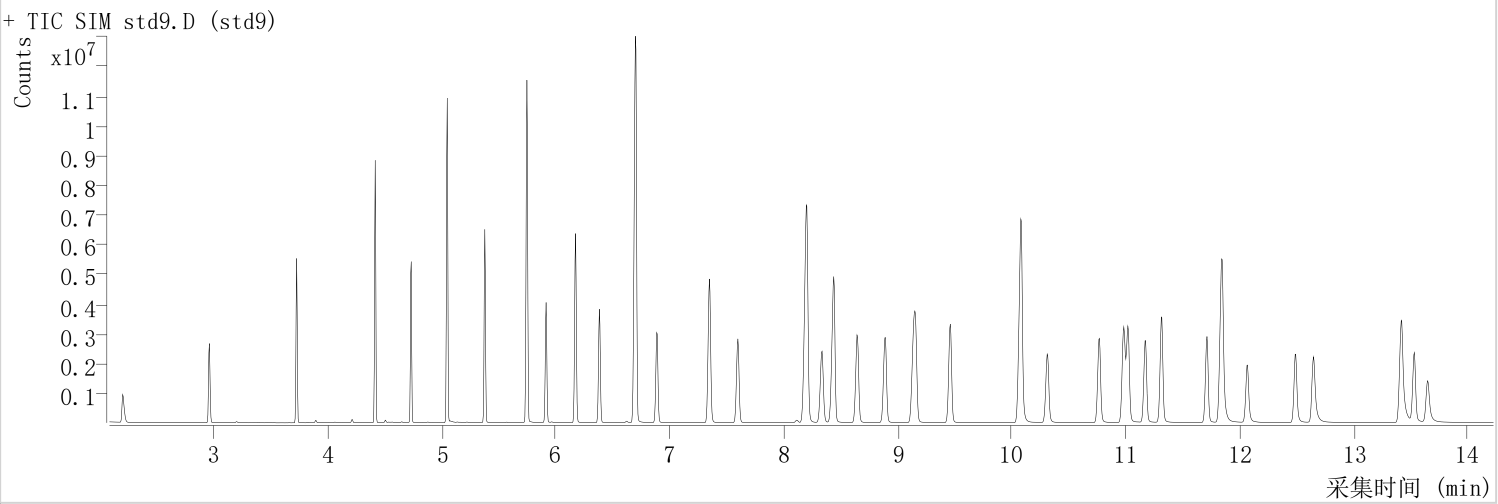

化合物	ISTD	RT	响应	ISTD 响应	响应比	最终浓度	单位
C4:0	C19:0	2.203	1129200	3505157	0.3222	80.4855	ug/ml
C6:0	C19:0	2.963	2093987	3505157	0.5974	77.1790	ug/ml
C8:0	C19:0	3.728	3387722	3505157	0.9665	78.4596	ug/ml
C10:0	C19:0	4.417	5151378	3505157	1.4697	65.9693	ug/ml
C11:0	C19:0	4.733	3258125	3505157	0.9295	33.9899	ug/ml
C12:0	C19:0	5.049	6792748	3505157	1.9379	61.8803	ug/ml
C13:0	C19:0	5.378	4183814	3505157	1.1936	33.4222	ug/ml
C14:0	C19:0	5.747	8355984	3505157	2.3839	ND	ug/ml
C14:1	C19:0	5.916	1927357	3505157	0.5499	27.6292	ug/ml
C15:0	C19:0	6.174	5044623	3505157	1.4392	34.2047	ug/ml
C15:1	C19:0	6.383	2236842	3505157	0.6382	32.7868	ug/ml
C16:0	C19:0	6.698	13522533	3505157	3.8579	114.9721	ug/ml
C16:1	C19:0	6.885	1864800	3505157	0.5320	32.4237	ug/ml
C17:0	C19:0	7.348	5748032	3505157	1.6399	34.2127	ug/ml
C17:1	C19:0	7.597	2237317	3505157	0.6383	34.3486	ug/ml
C18:0	C19:0	8.197	11509999	3505157	3.2837	80.1143	ug/ml
C18:1n9t	C19:0	8.330	2162177	3505157	0.6169	36.3881	ug/ml
C18:1n9c	C19:0	8.437	4300626	3505157	1.2269	76.7739	ug/ml
C18:2n6t	C19:0	8.642	2467607	3505157	0.7040	38.6118	ug/ml
C18:2n6c	C19:0	8.891	2429948	3505157	0.6932	42.1630	ug/ml
C18:3n6	C19:0	9.153	2245396	3505157	0.6406	34.8502	ug/ml
C18:3n3	C19:0	9.460	2708947	3505157	0.7728	38.4184	ug/ml
C20:0	C19:0	10.082	10467244	3505157	2.9862	75.3597	ug/ml
C20:1	C19:0	10.309	2250474	3505157	0.6420	39.6057	ug/ml
C20:2	C19:0	10.767	2288759	3505157	0.6530	39.9549	ug/ml
C21:0	C19:0	10.981	5116545	3505157	1.4597	40.1569	ug/ml
C20:3n6	C19:0	11.021	2213995	3505157	0.6316	39.9359	ug/ml
C20:4n6	C19:0	11.172	2217714	3505157	0.6327	39.4878	ug/ml
C20:3n3	C19:0	11.314	2680917	3505157	0.7648	40.0386	ug/ml
C20:5n3	C19:0	11.710	2503902	3505157	0.7143	39.5152	ug/ml

化合物	ISTD	RT	响应	ISTD 响应	响应比	最终浓度	单位
C22:0	C19:0	11.839	8406927	3505157	2.3984	77.6760	ug/ml
C22:1n9	C19:0	12.061	1896201	3505157	0.5410	40.0346	ug/ml
C22:2n6	C19:0	12.484	1830434	3505157	0.5222	41.3056	ug/ml
C23:0	C19:0	12.644	3602567	3505157	1.0278	39.6737	ug/ml
C24:0	C19:0	13.417	6033710	3505157	1.7214	77.6270	ug/ml
C22:6	C19:0	13.529	2149727	3505157	0.6133	45.3100	ug/ml
C24:1	C19:0	13.644	1537559	3505157	0.4387	38.6845	ug/ml

C4:0

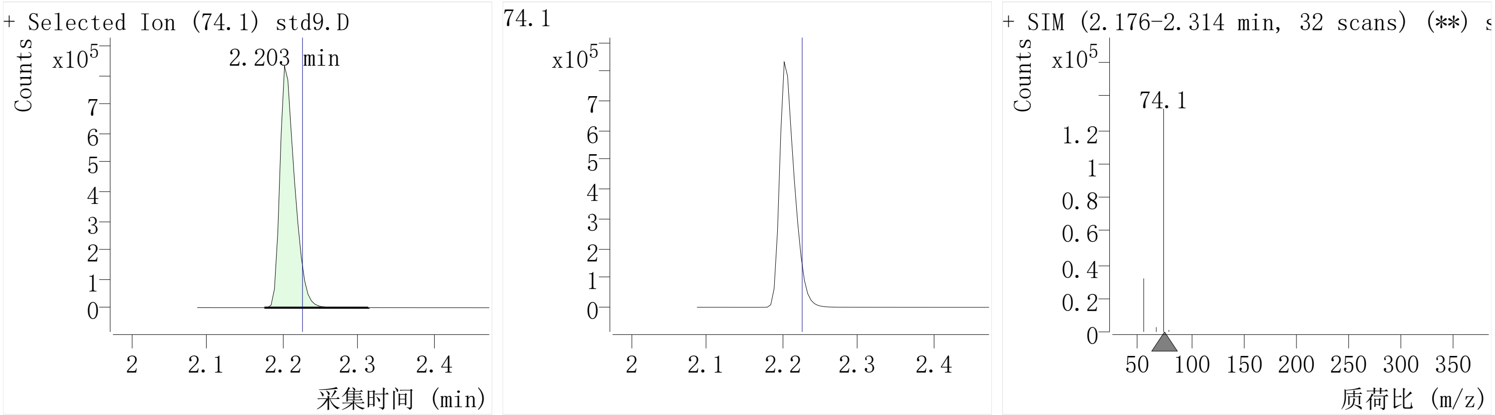

C6:0

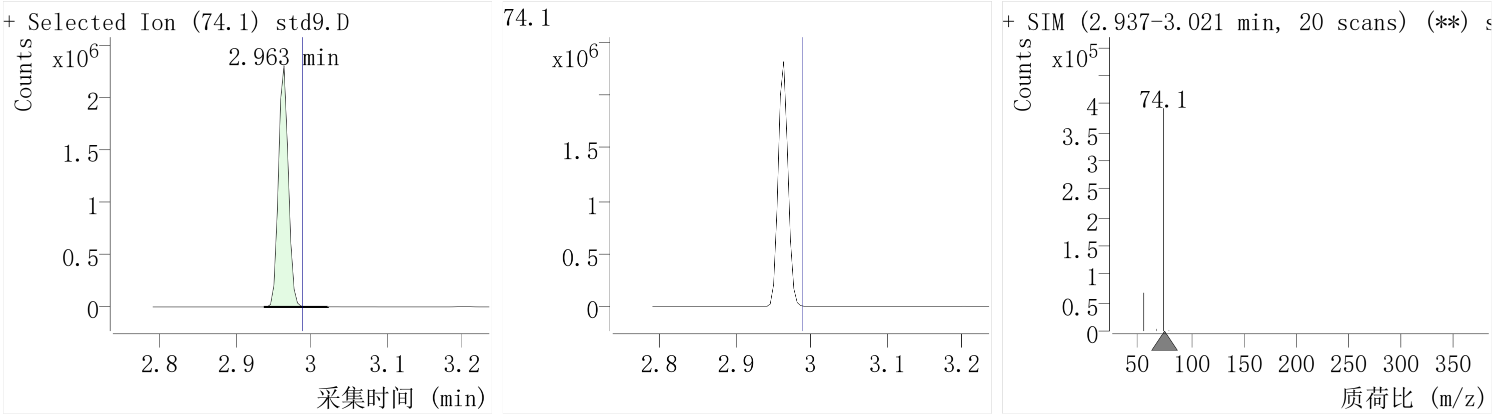

C8:0

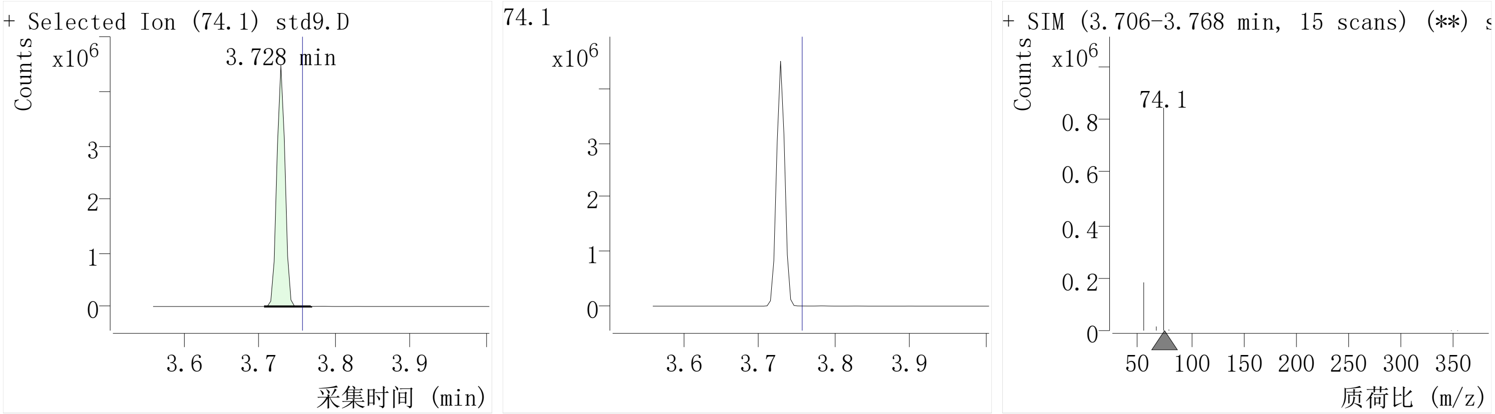

C10:0

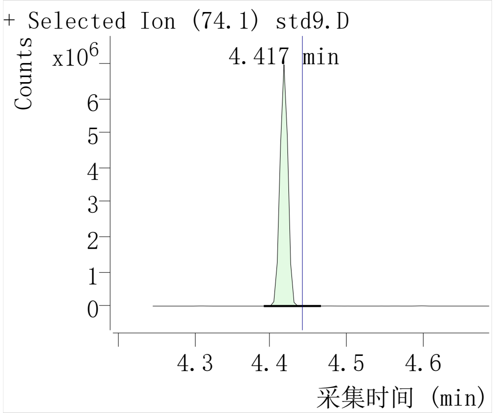

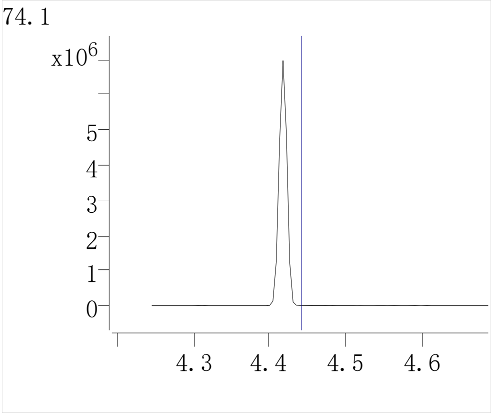

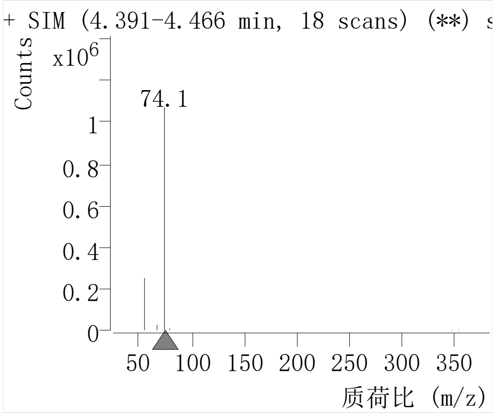

C11:0

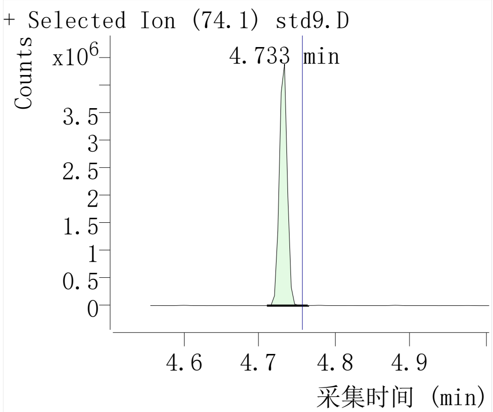

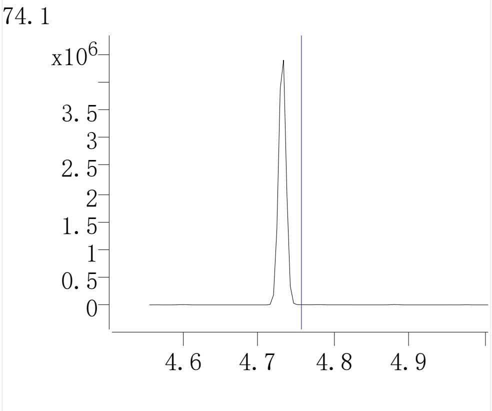

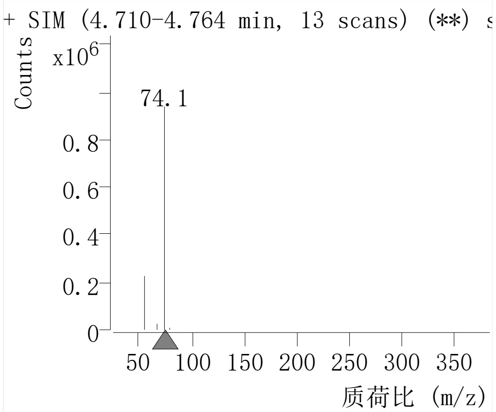

C12:0

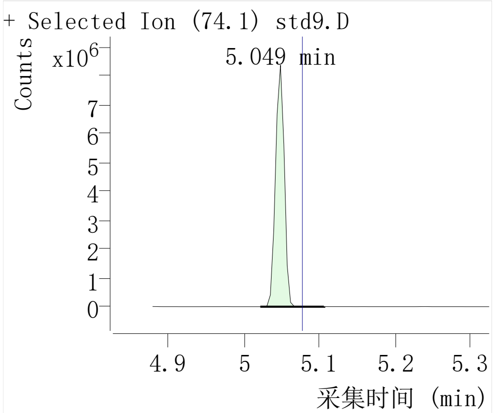

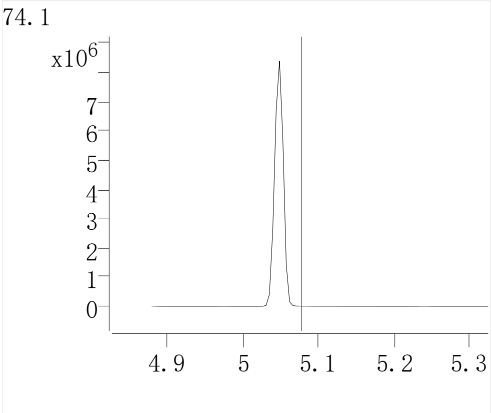

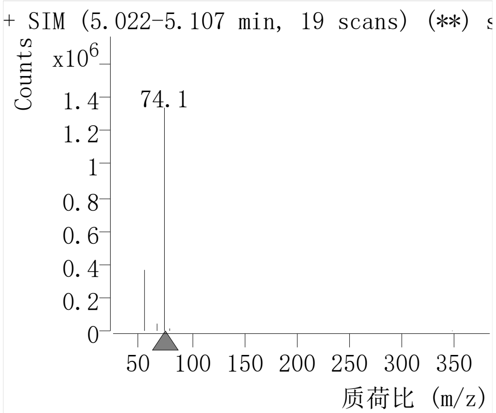

C13:0

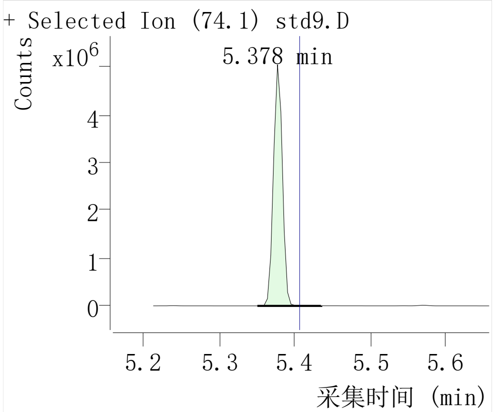

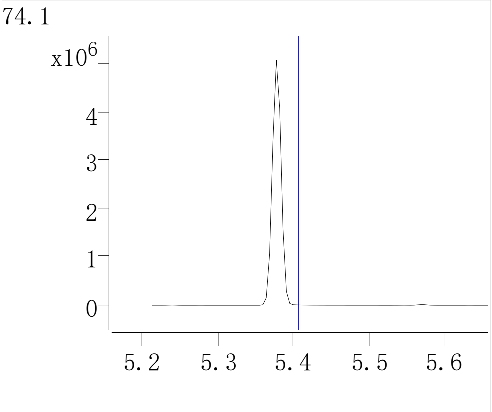

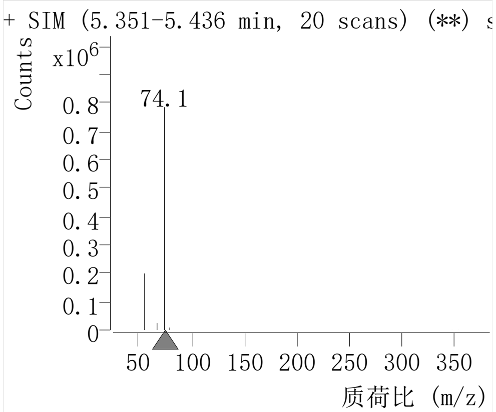

C14:0

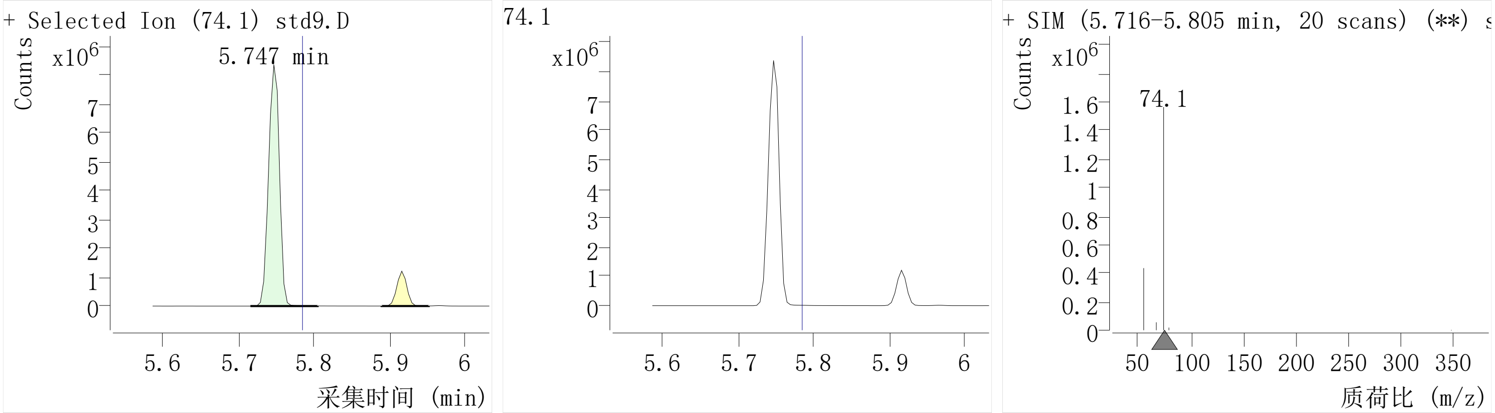

C14:1

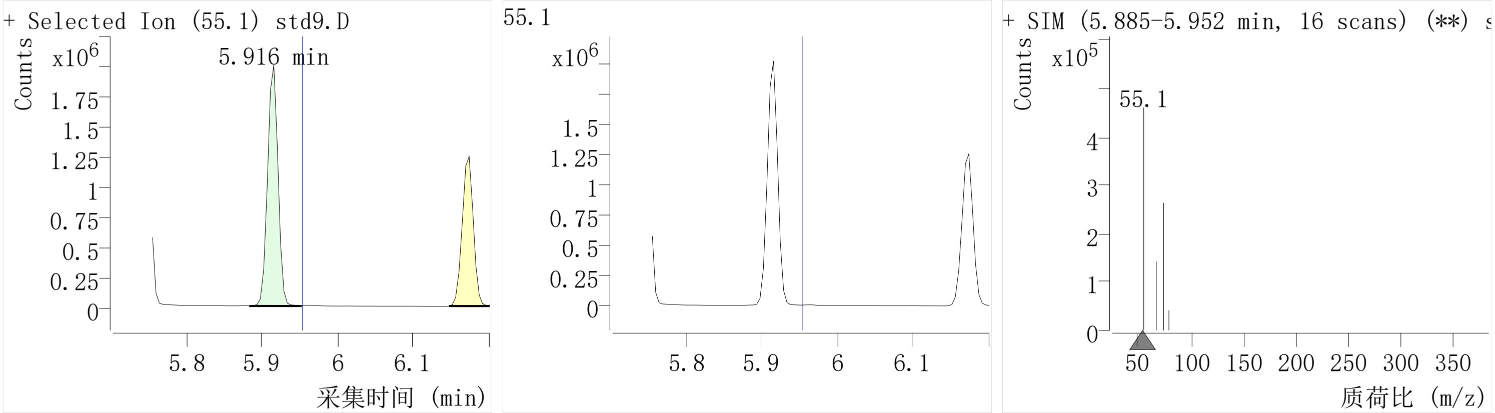

C15:0

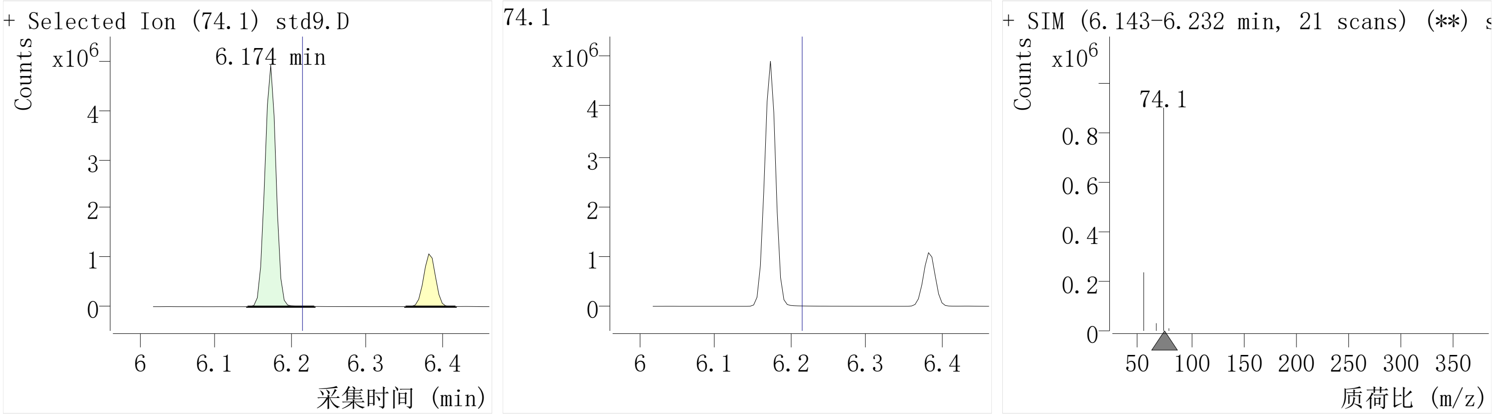

C15:1

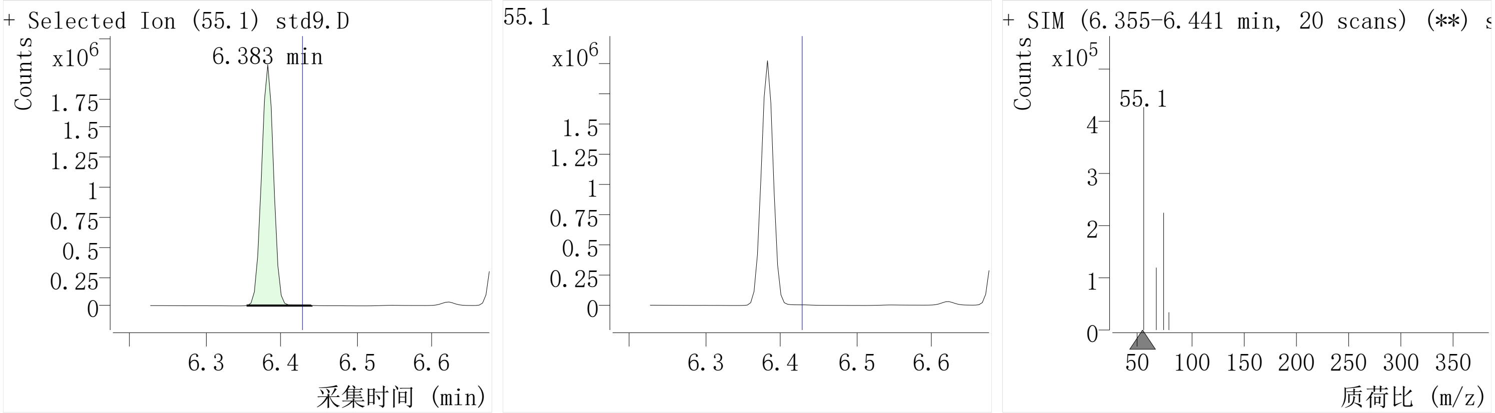

C16:0

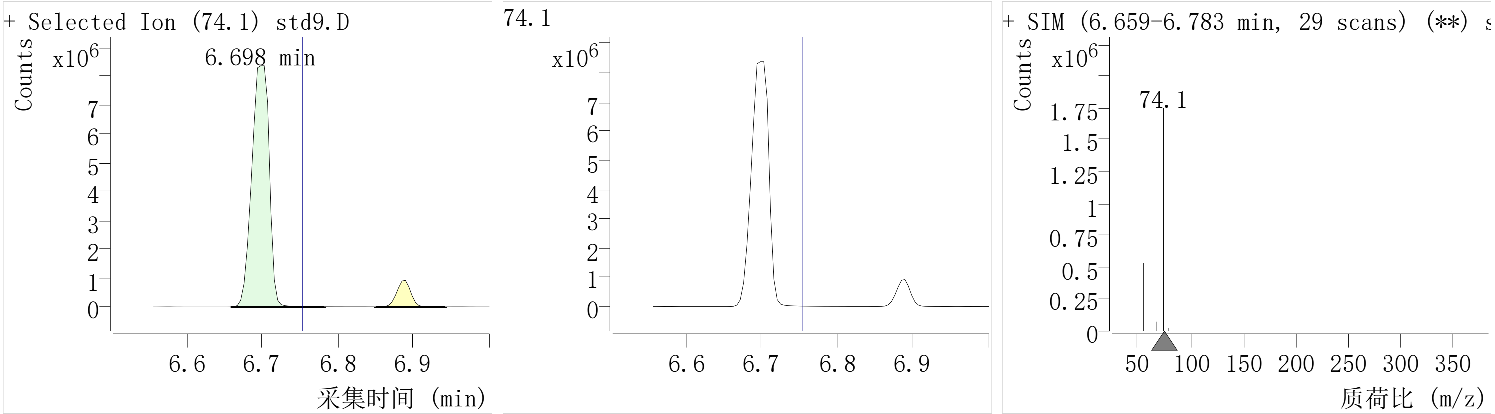

C16:1

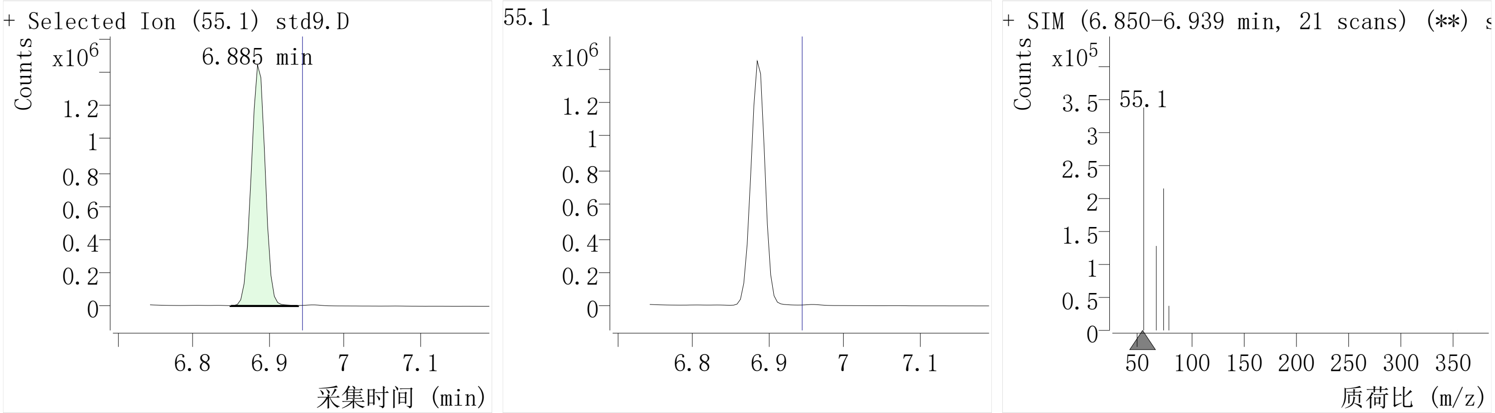

C17:0

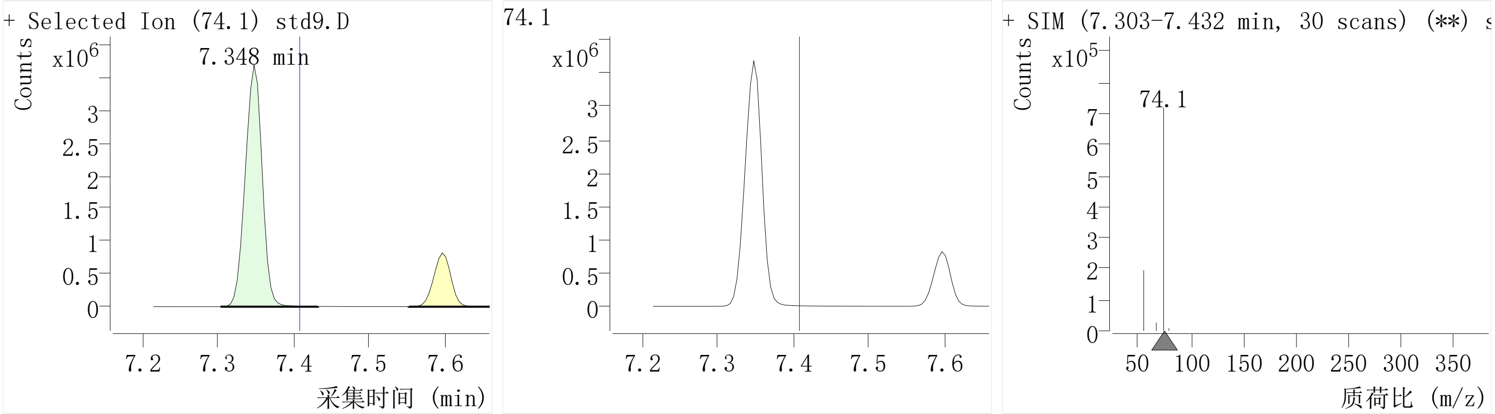

C17:1

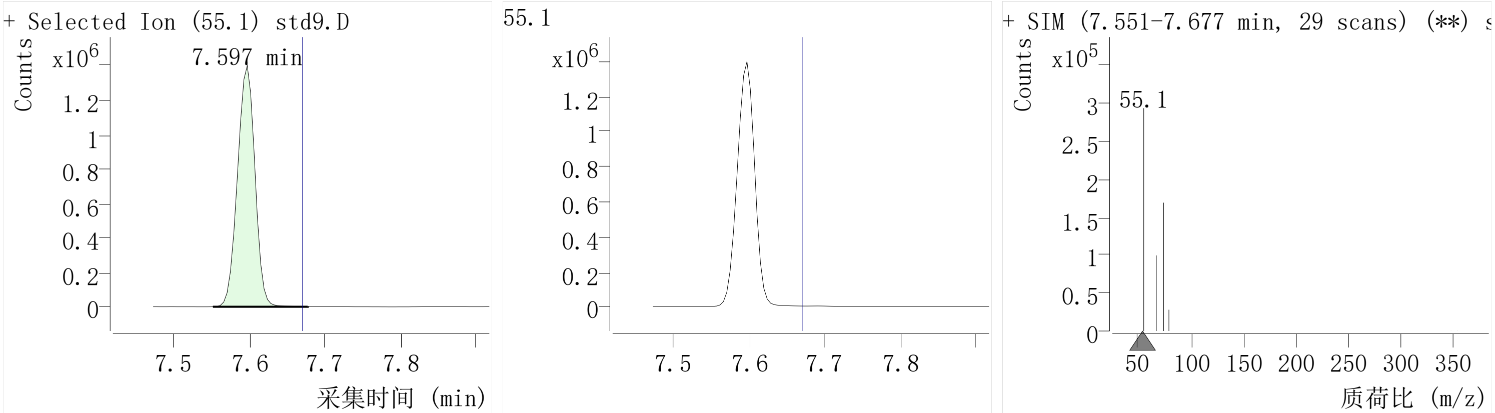

C18:0

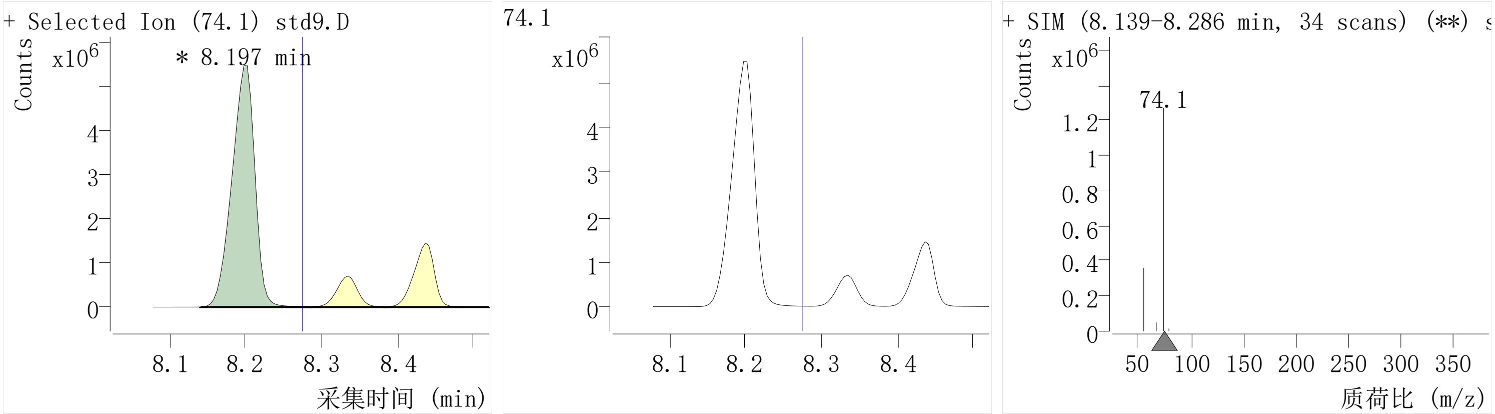

C18:1n9t

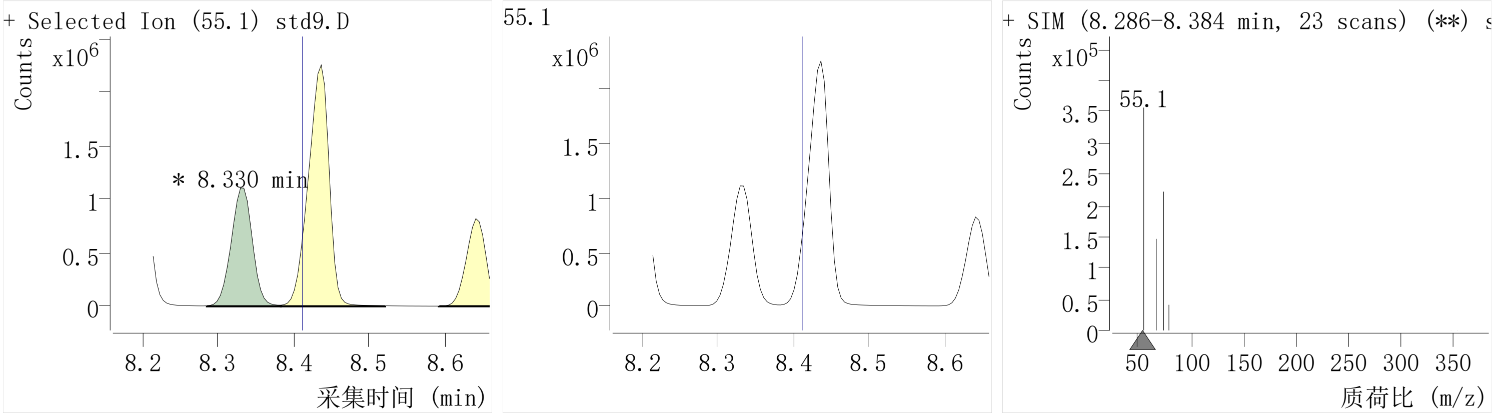

C18:1n9c

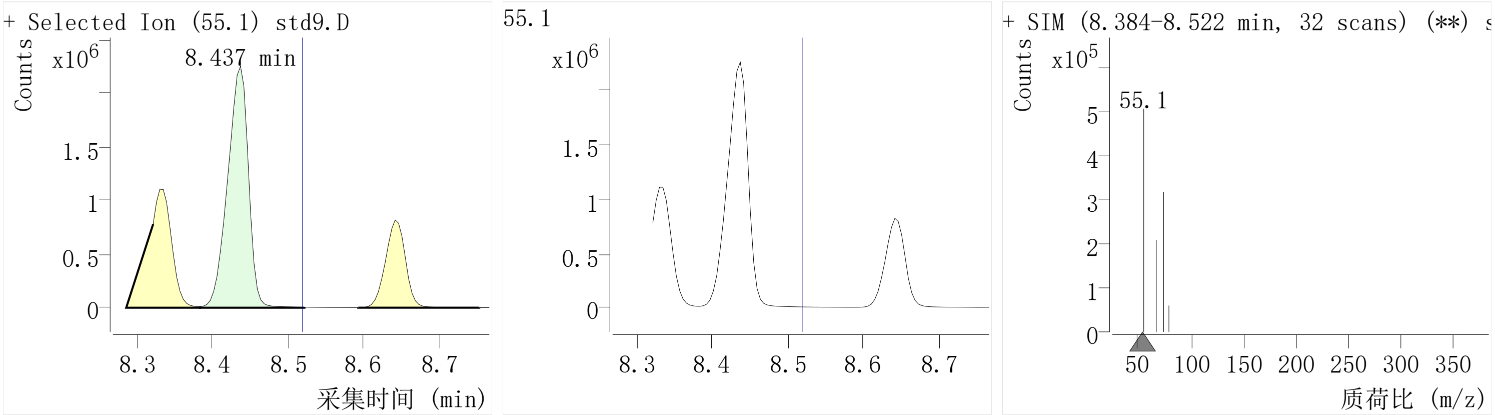

C18:2n6t

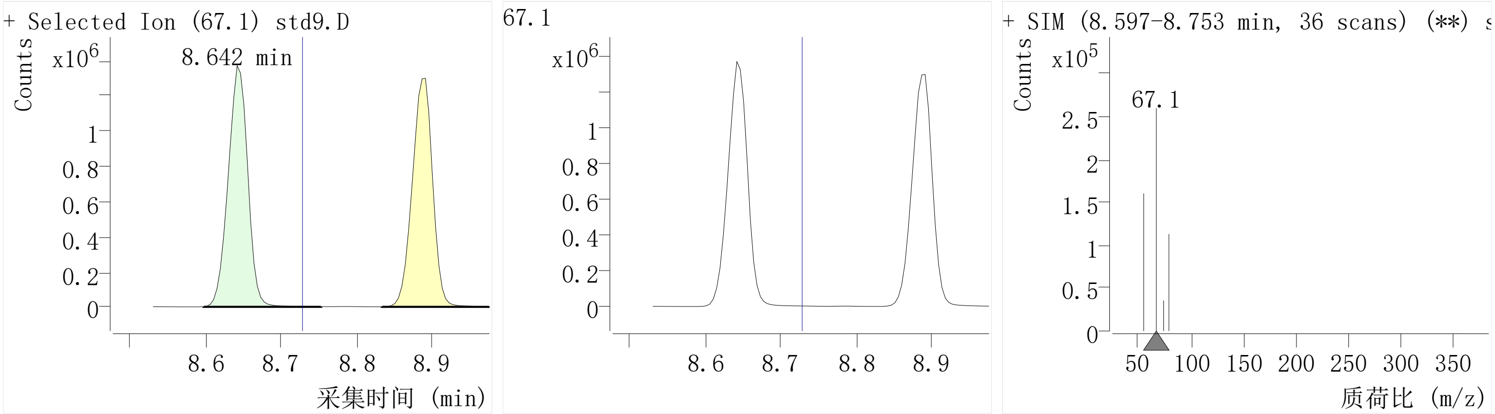

C18:2n6c

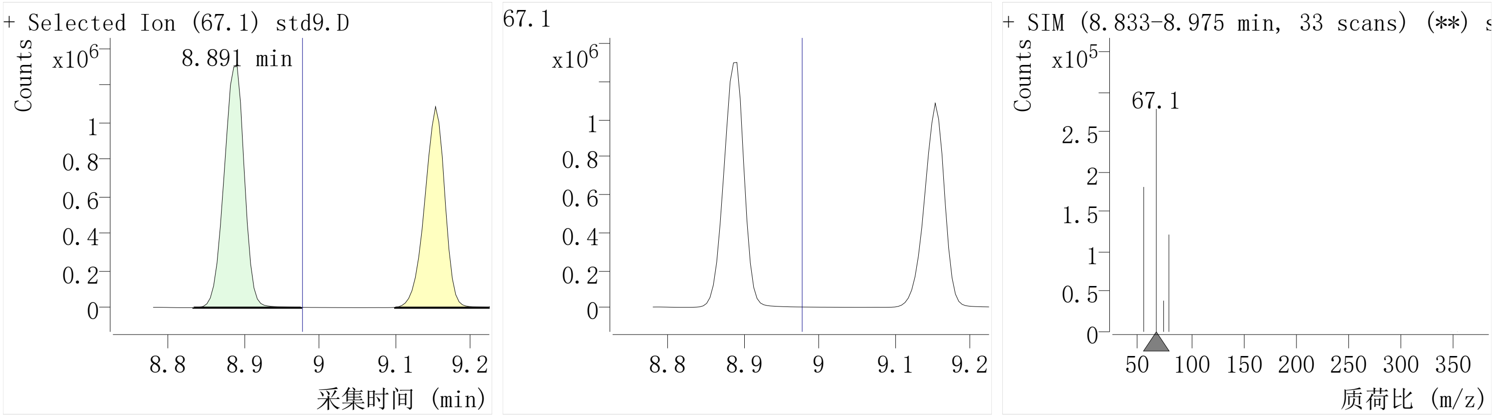

C18:3n6

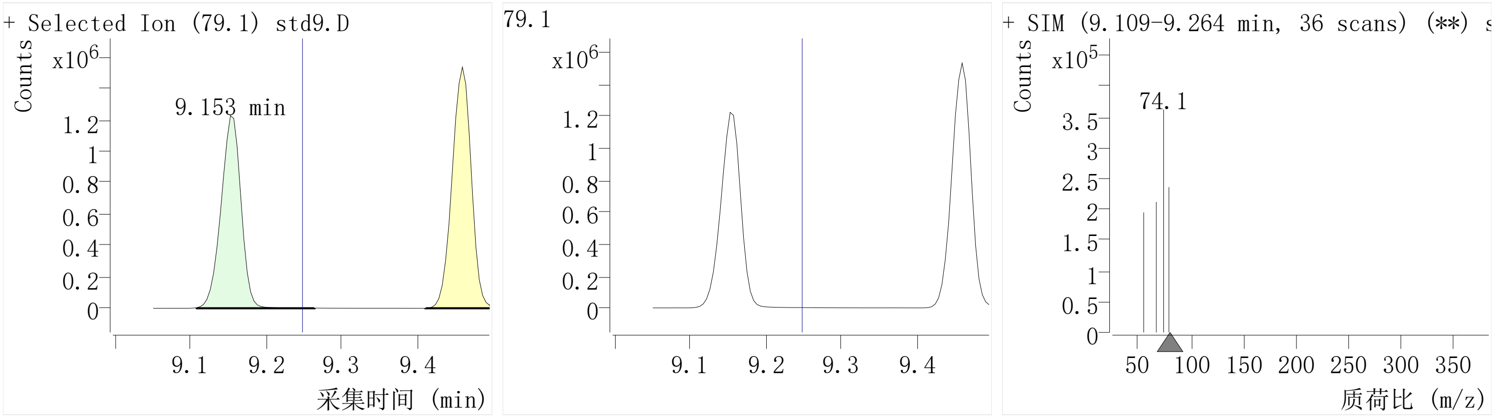

C18:3n3

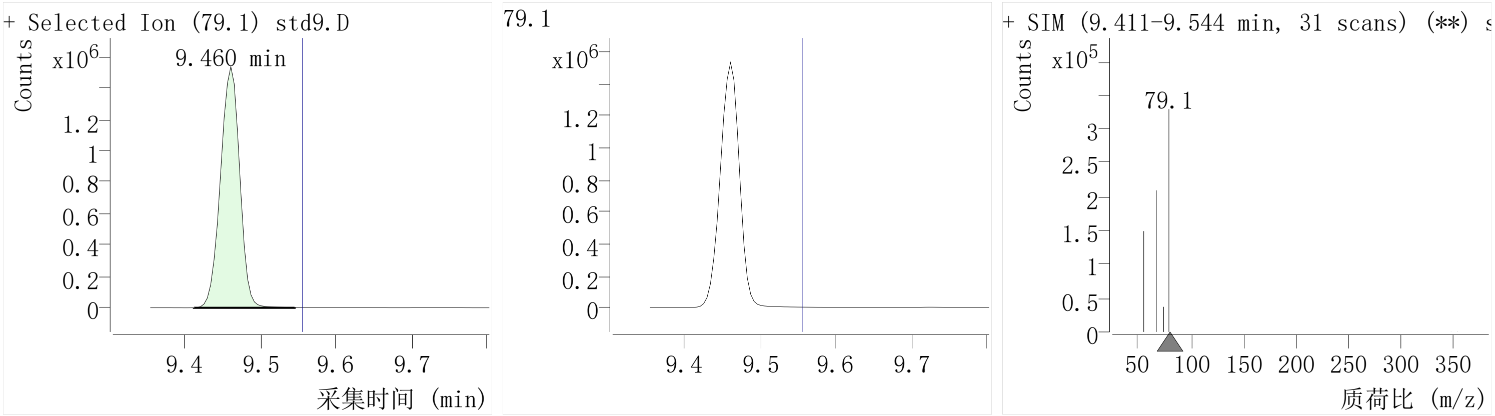

C20:0

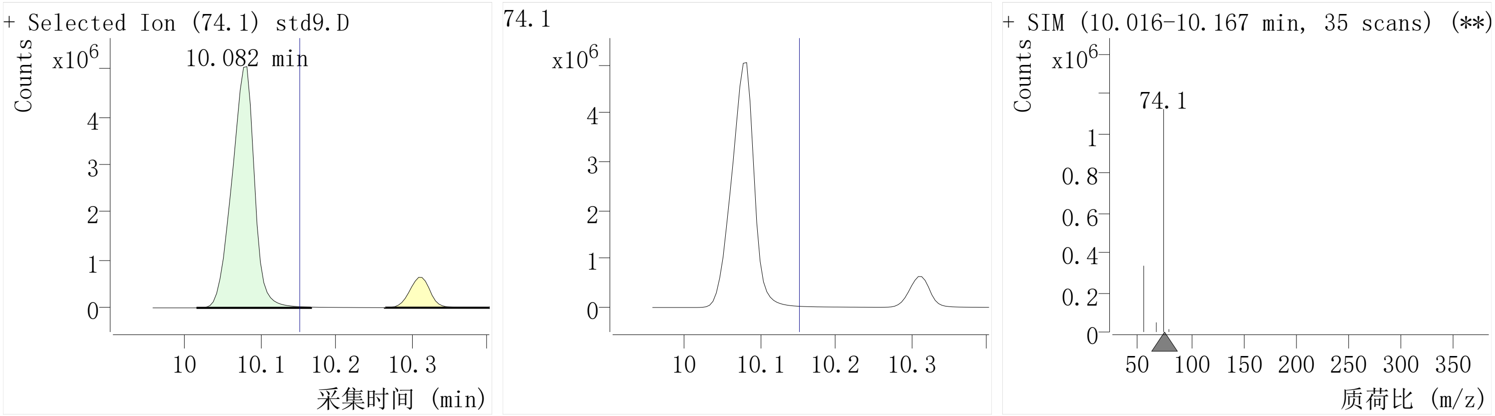

C20:1

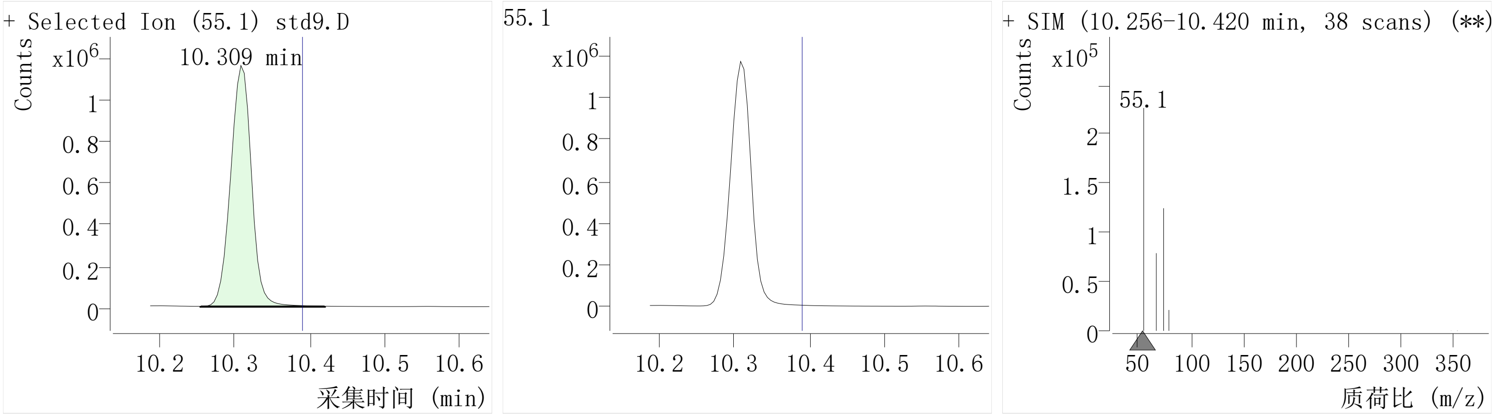

C20:2

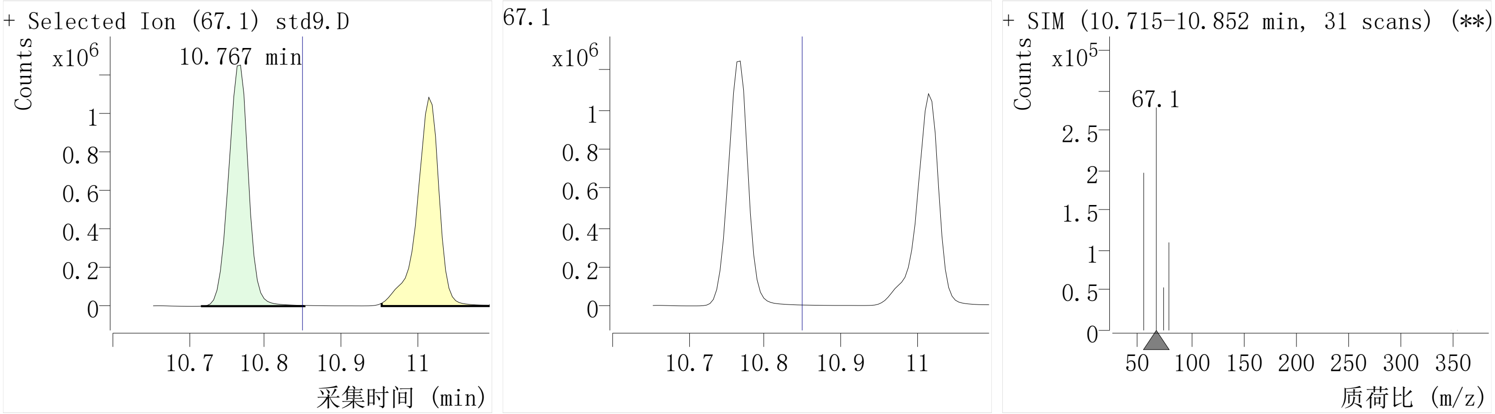

C21:0

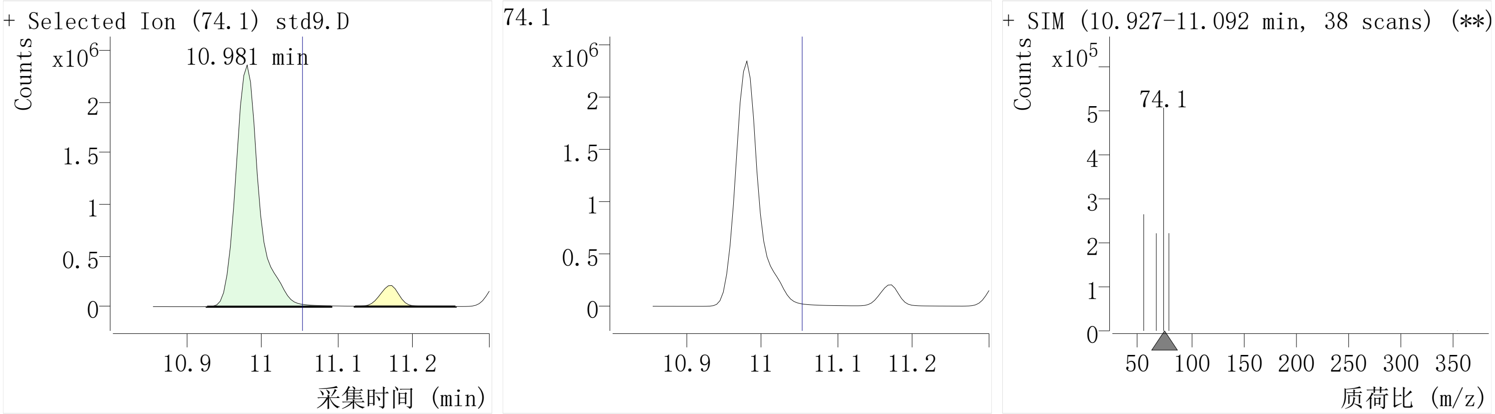

C20:3n6

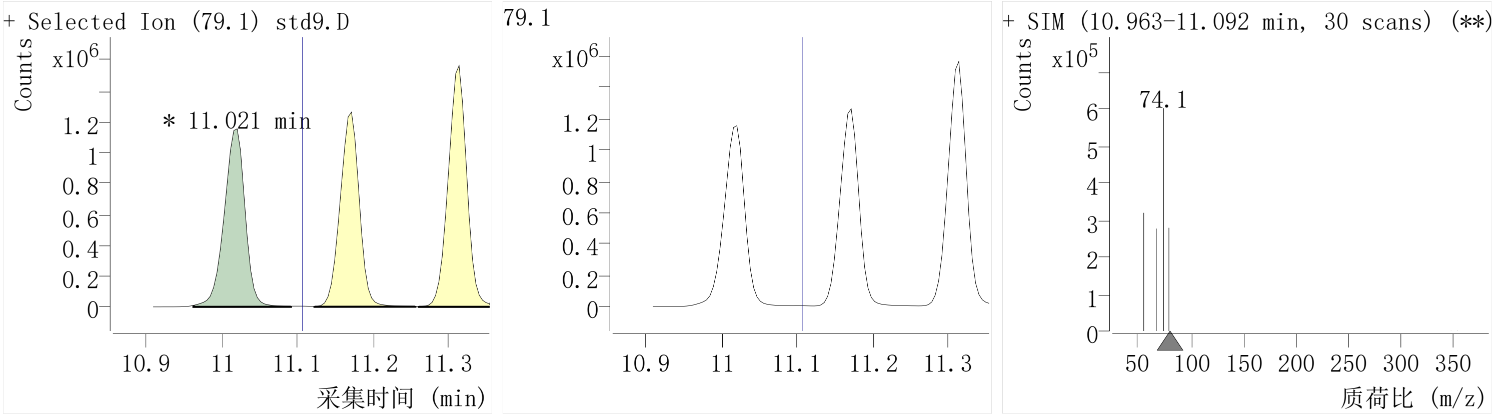

C20:4n6

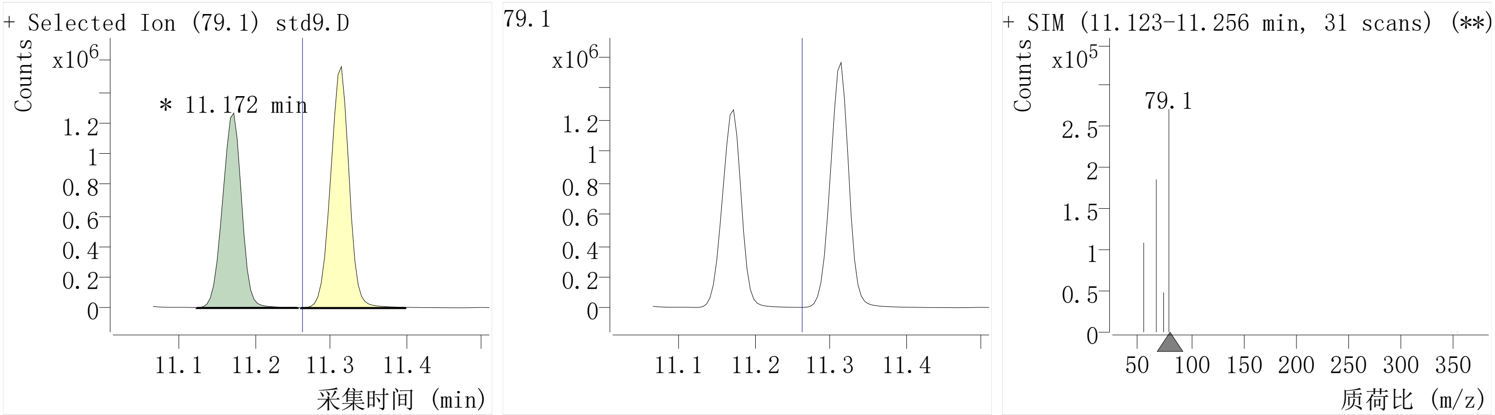

C20:3n3

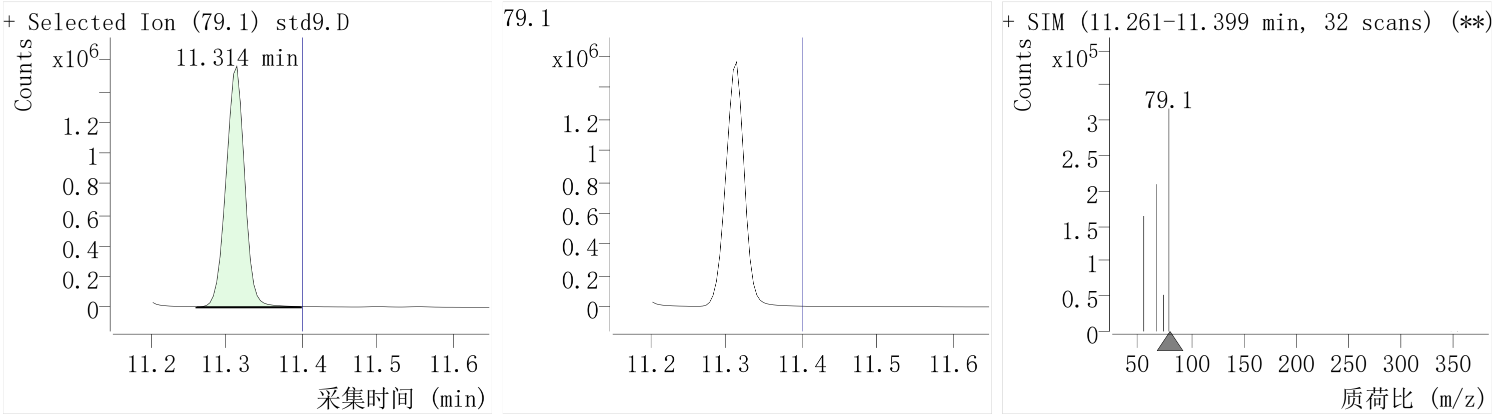

C20:5n3

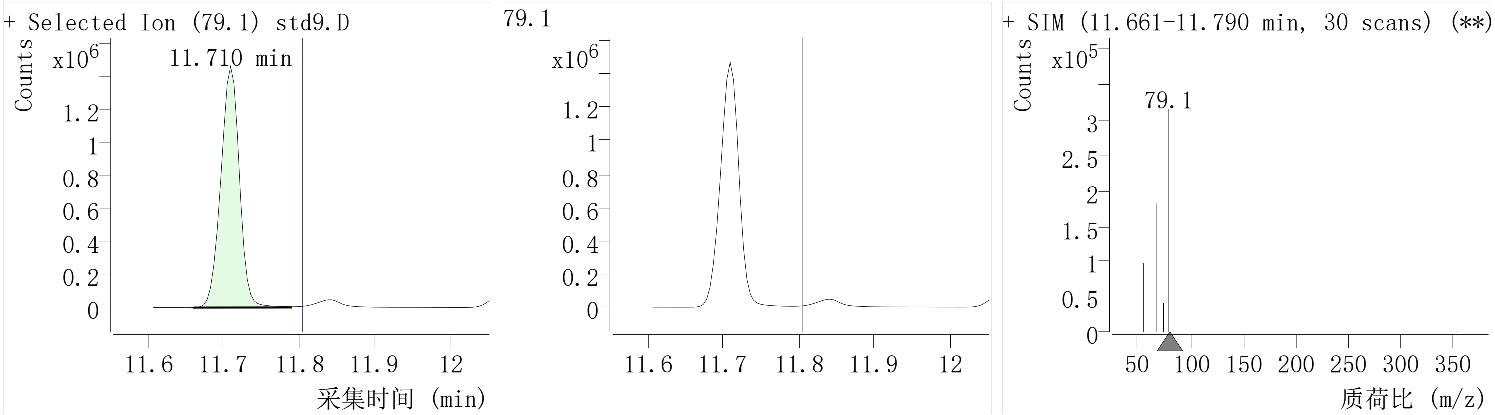

C22:0

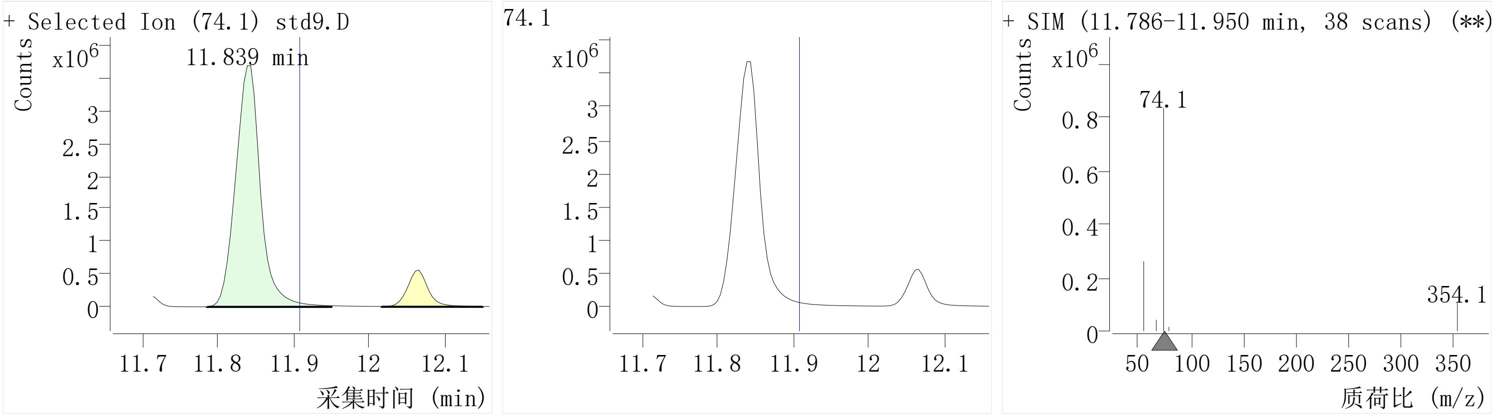

C22:1n9

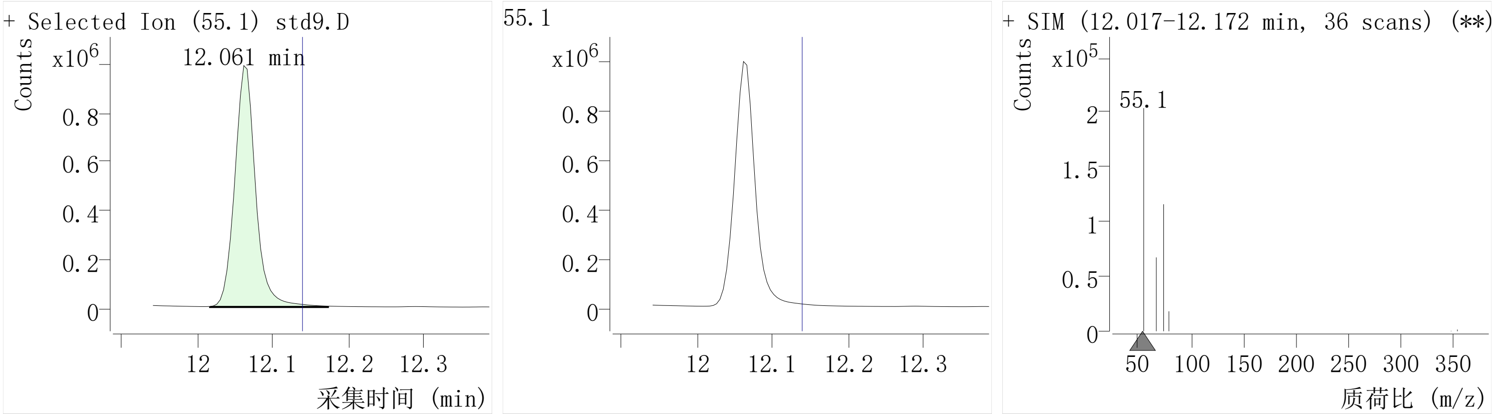

C22:2n6

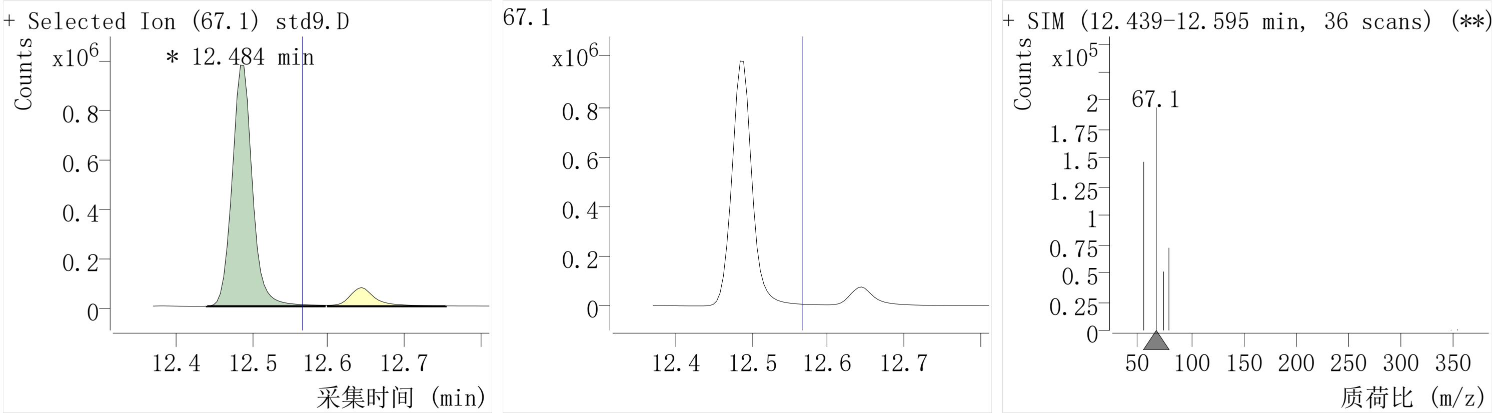

C23:0

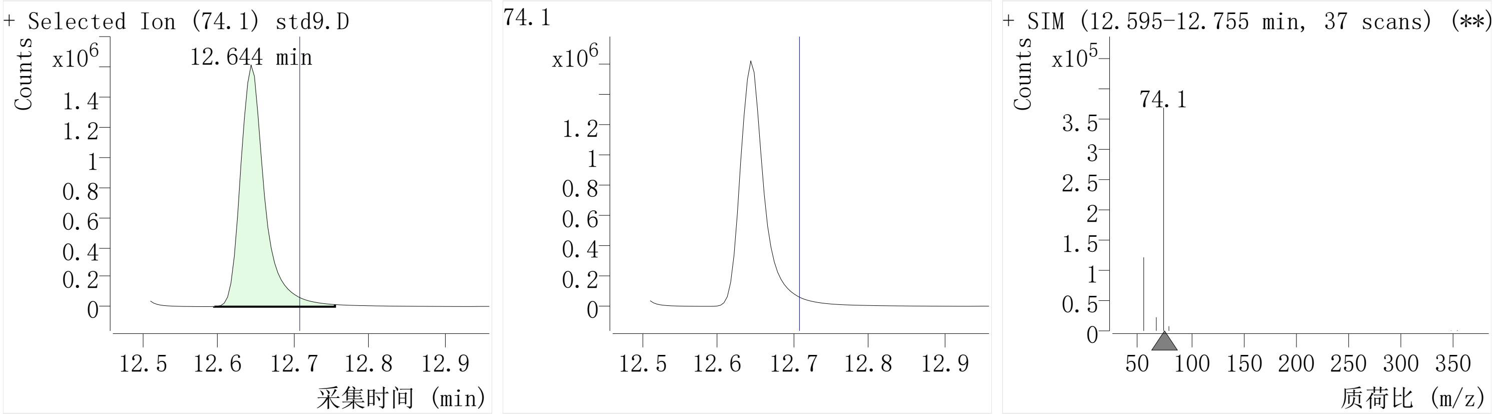

C24:0

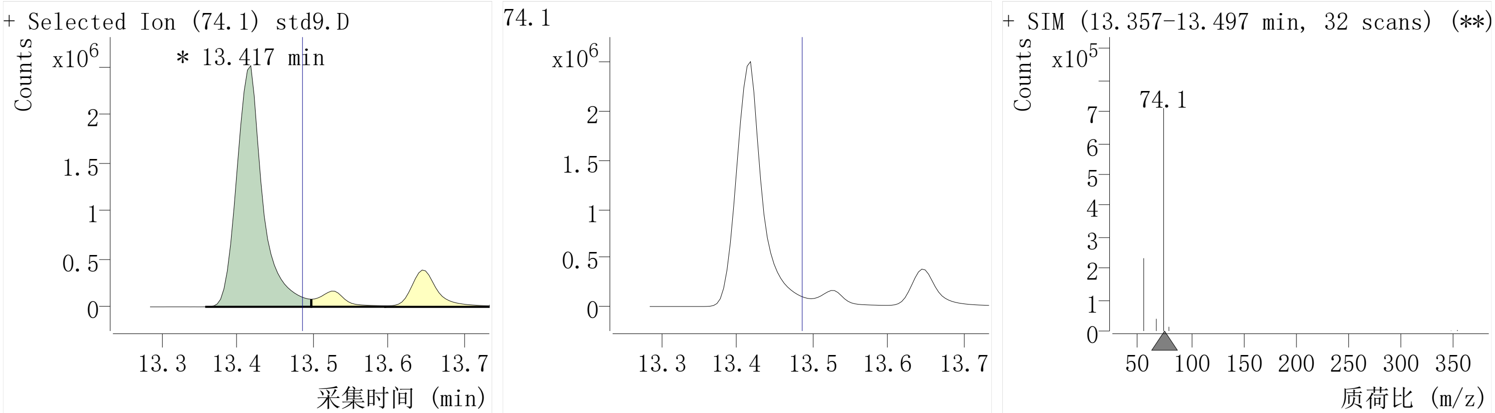

C22:6

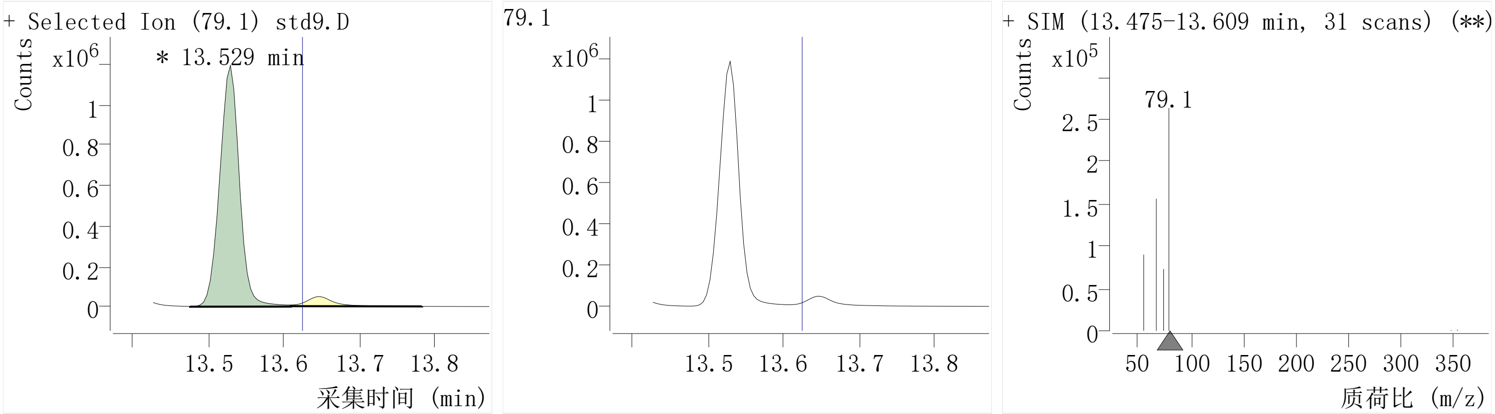

+ Selected Ion (79.1) std9.D
Counts
x10^6
* 13.529 min
1
0.8
0.6
0.4
0.2
0
13.5
13.6
13.7
13.8
采集时间 (min)
79.1
x10^6
1
0.8
0.6
0.4
0.2
0
13.5
13.6
13.7
13.8
+ SIM (13.475-13.609 min, 31 scans) (**)
Counts
x10^5
79.1
2.5
2
1.5
1
0.5
0
50
100
150
200
250
300
350
质荷比 (m/z)

C24:1

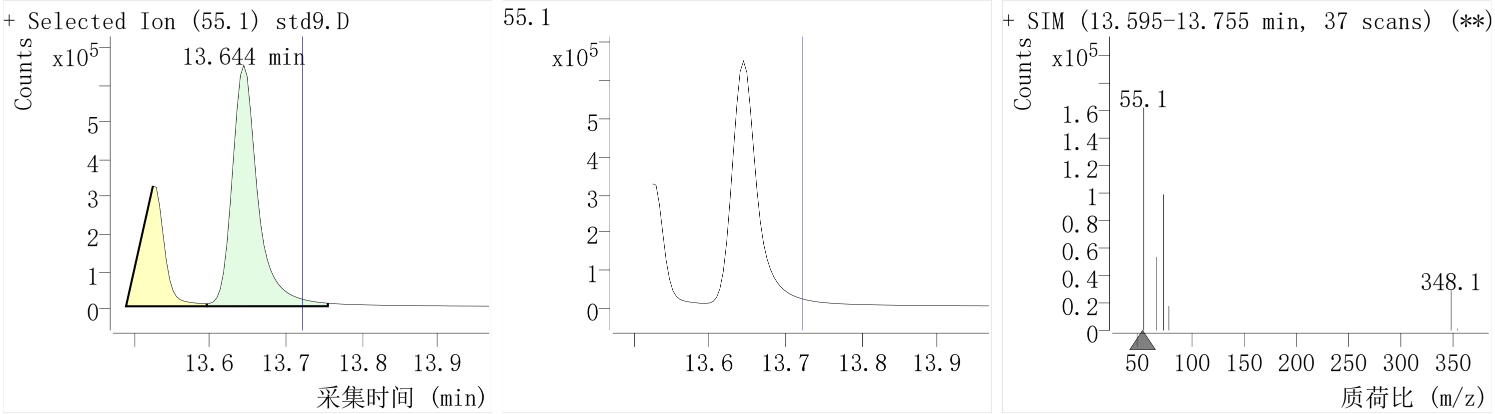

+ Selected Ion (55.1) std9.D
Counts
x10^5
13.644 min
5
4
3
2
1
0
13.6
13.7
13.8
13.9
采集时间 (min)
55.1
x10^5
5
4
3
2
1
0
13.6
13.7
13.8
13.9
+ SIM (13.595-13.755 min, 37 scans) (**)
Counts
x10^5
55.1
1.6
1.4
1.2
1
0.8
0.6
0.4
0.2
0
348.1
50
100
150
200
250
300
350
质荷比 (m/z)

**批处理路径**	G:\GC-MS\HX250430-4-GCMS总脂肪酸靶向检测\HX250430-4\QuantResults\HX250430-4.batch.bin		
**分析时间**	2025/5/14 16:58	**分析员姓名**	DESKTOP-M3A0GPO\omics
**报告时间**	2025/5/16 14:53:12	**报告员姓名**	DESKTOP-M3A0GPO\omics
**最近校正更新**	2025/5/14 16:58	**批处理状态**	已处理
**定量批处理版本**	10.2	**定量报告版本**	10.2

**采集时间**	2025/5/8 20:31	**数据文件**	std10.D
**样品类型**	校正	**样品名称**	std10
**稀释**	1	**采集方法**	脂肪酸

样品色谱图

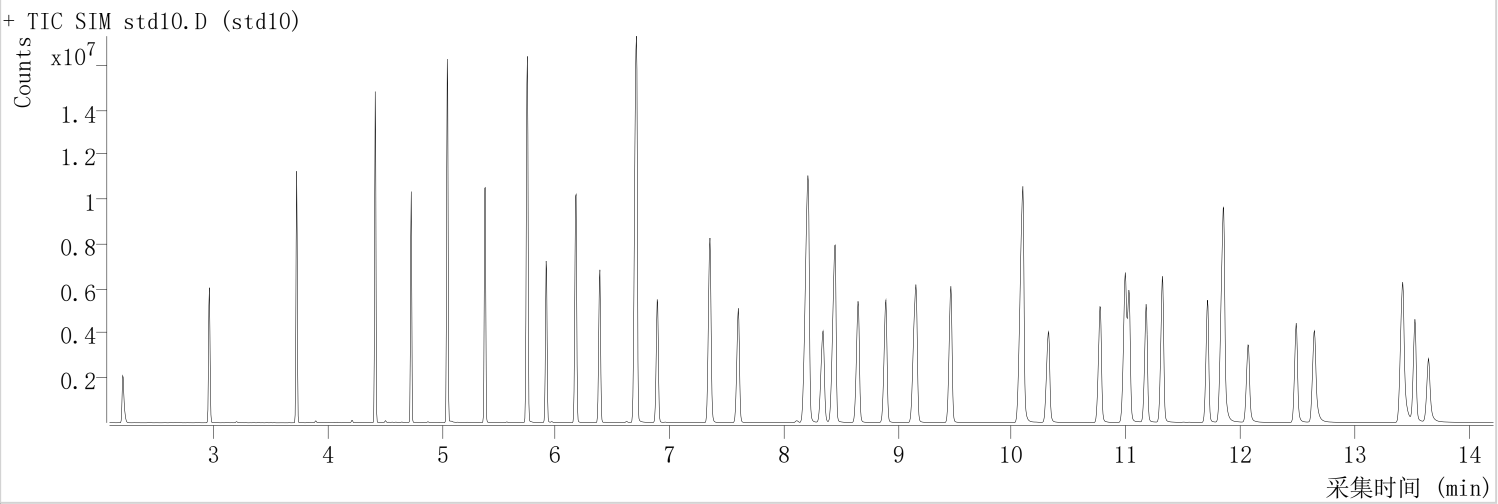

化合物	ISTD	RT	响应	ISTD 响应	响应比	最终浓度	单位
C4:0	C19:0	2.203	2300709	4076680	0.5644	ND	ug/ml
C6:0	C19:0	2.963	4667340	4076680	1.1449	184.2979	ug/ml
C8:0	C19:0	3.728	7014399	4076680	1.7206	173.2433	ug/ml
C10:0	C19:0	4.422	8122381	4076680	1.9924	96.1087	ug/ml
C11:0	C19:0	4.733	6162737	4076680	1.5117	61.1740	ug/ml
C12:0	C19:0	5.053	9395661	4076680	2.3047	76.2069	ug/ml
C13:0	C19:0	5.382	7475317	4076680	1.8337	55.0265	ug/ml
C14:0	C19:0	5.750	11343421	4076680	2.7825	ND	ug/ml
C14:1	C19:0	5.916	3591834	4076680	0.8811	44.3358	ug/ml
C15:0	C19:0	6.178	8911026	4076680	2.1859	54.5112	ug/ml
C15:1	C19:0	6.387	4130142	4076680	1.0131	52.2728	ug/ml
C16:0	C19:0	6.702	17904657	4076680	4.3920	137.7664	ug/ml
C16:1	C19:0	6.890	3434330	4076680	0.8424	51.3937	ug/ml
C17:0	C19:0	7.352	10377085	4076680	2.5455	53.1061	ug/ml
C17:1	C19:0	7.601	4118789	4076680	1.0103	54.3859	ug/ml
C18:0	C19:0	8.210	20066589	4076680	4.9223	159.9077	ug/ml
C18:1n9t	C19:0	8.344	4028333	4076680	0.9881	58.2900	ug/ml
C18:1n9c	C19:0	8.446	7665882	4076680	1.8804	120.9740	ug/ml
C18:2n6t	C19:0	8.650	4616889	4076680	1.1325	62.1033	ug/ml
C18:2n6c	C19:0	8.895	4531701	4076680	1.1116	77.8080	ug/ml
C18:3n6	C19:0	9.162	4193173	4076680	1.0286	56.3433	ug/ml
C18:3n3	C19:0	9.464	5142622	4076680	1.2615	62.7121	ug/ml
C20:0	C19:0	10.096	18731212	4076680	4.5947	115.9324	ug/ml
C20:1	C19:0	10.322	4227381	4076680	1.0370	63.9424	ug/ml
C20:2	C19:0	10.772	4407678	4076680	1.0812	66.1218	ug/ml
C21:0	C19:0	10.994	9619014	4076680	2.3595	64.9103	ug/ml
C20:3n6	C19:0	11.029	4273562	4076680	1.0483	66.2501	ug/ml
C20:4n6	C19:0	11.176	4247049	4076680	1.0418	64.9970	ug/ml
C20:3n3	C19:0	11.318	5157652	4076680	1.2652	66.1956	ug/ml
C20:5n3	C19:0	11.714	4794968	4076680	1.1762	65.0402	ug/ml

化合物	ISTD	RT	响应	ISTD 响应	响应比	最终浓度	单位
C22:0	C19:0	11.856	15354227	4076680	3.7664	121.9655	ug/ml
C22:1n9	C19:0	12.070	3624168	4076680	0.8890	65.7995	ug/ml
C22:2n6	C19:0	12.492	3557120	4076680	0.8726	68.9920	ug/ml
C23:0	C19:0	12.652	6976080	4076680	1.7112	66.0681	ug/ml
C24:0	C19:0	13.426	11459938	4076680	2.8111	126.7645	ug/ml
C22:6	C19:0	13.533	4220443	4076680	1.0353	76.4803	ug/ml
C24:1	C19:0	13.653	2974941	4076680	0.7297	64.3589	ug/ml

C4:0

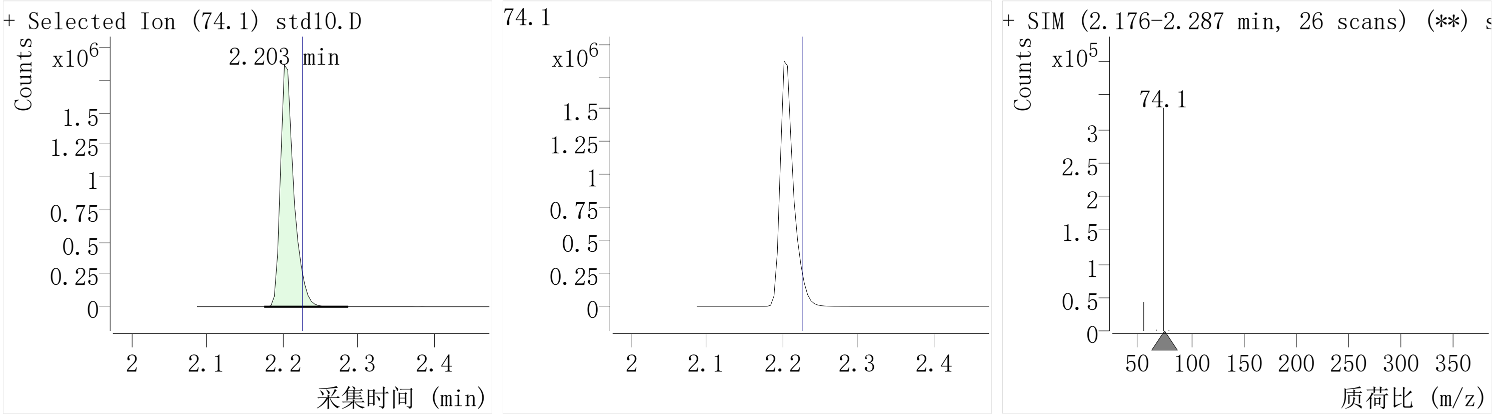

C6:0

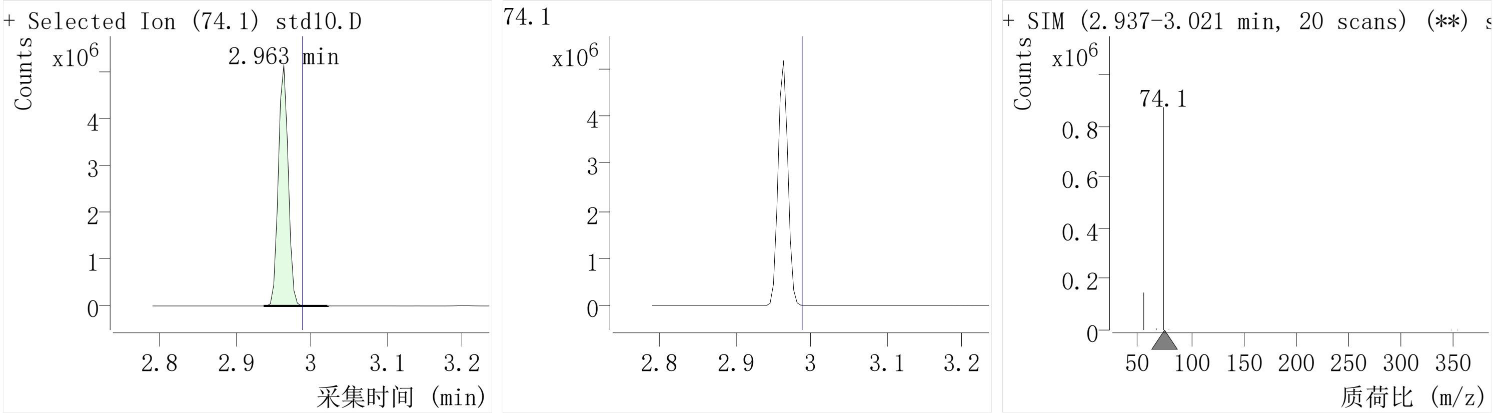

C8:0

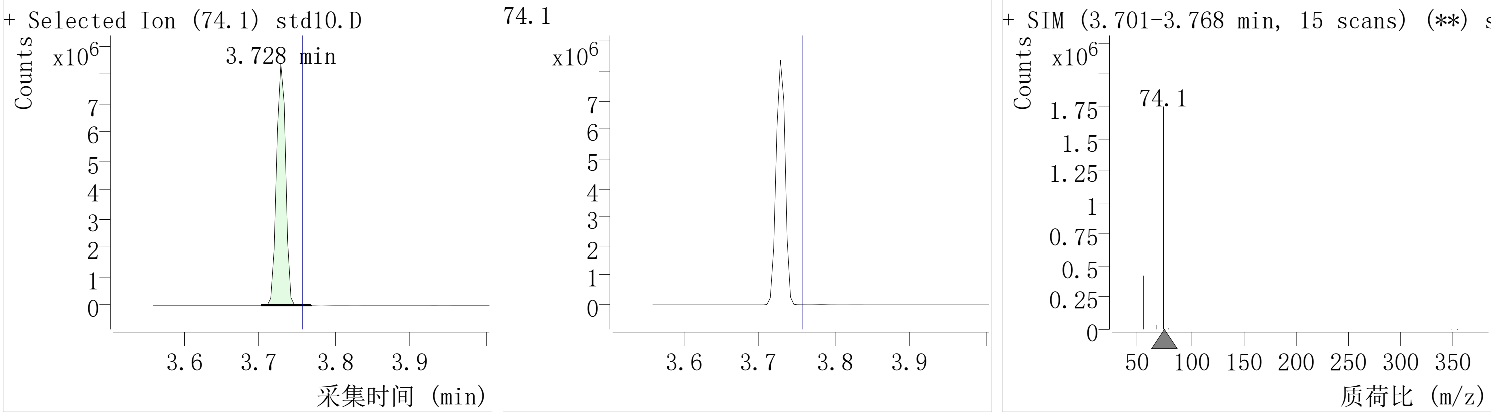

C10:0

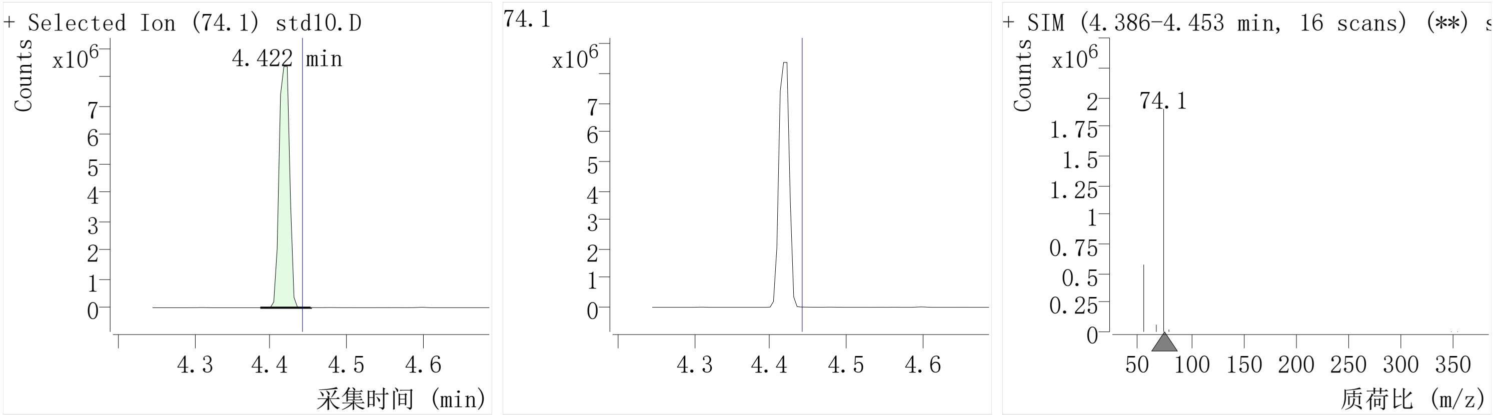

C11:0

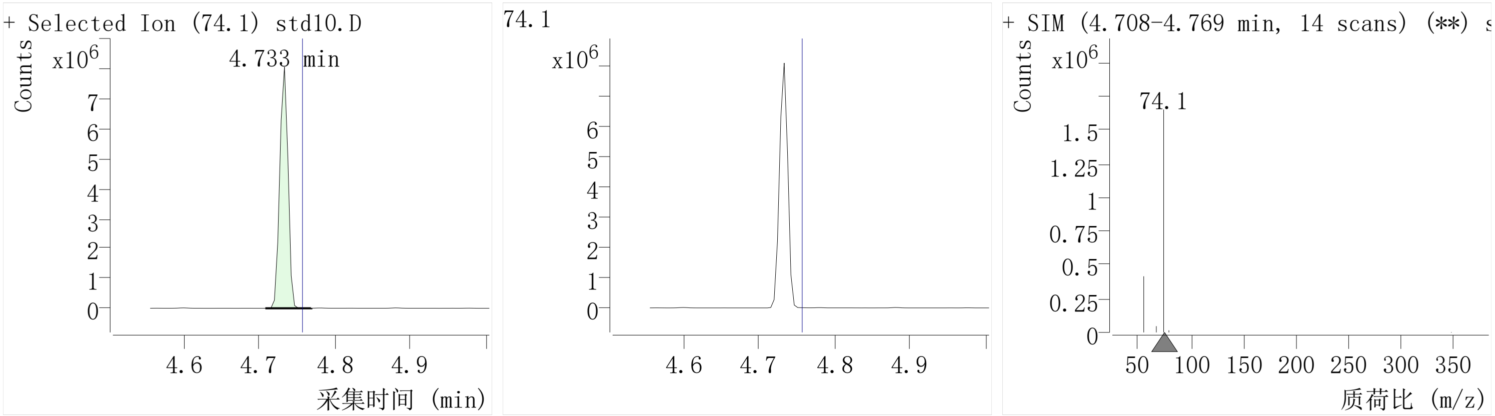

C12:0

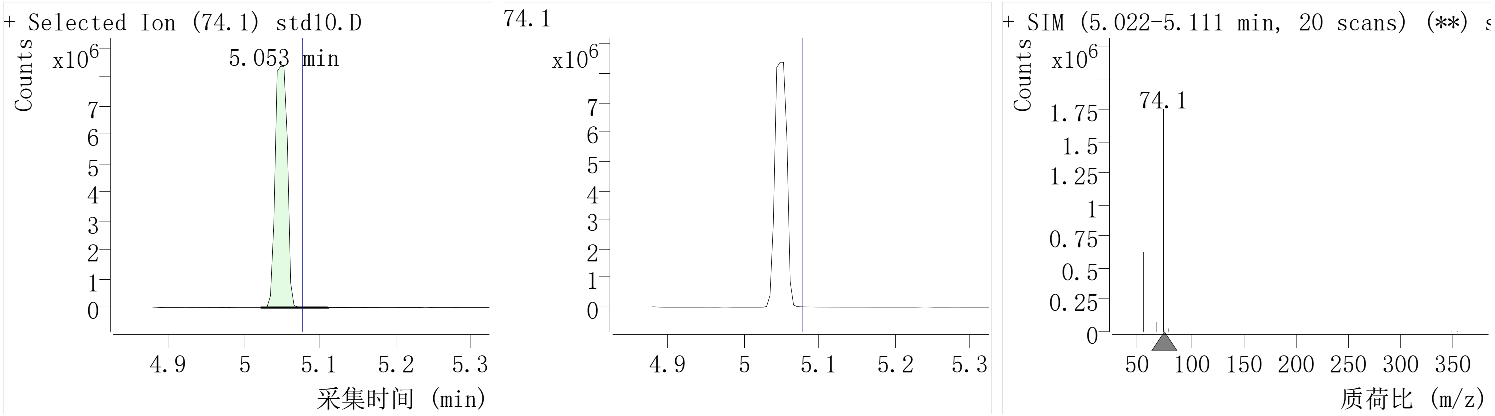

C13:0

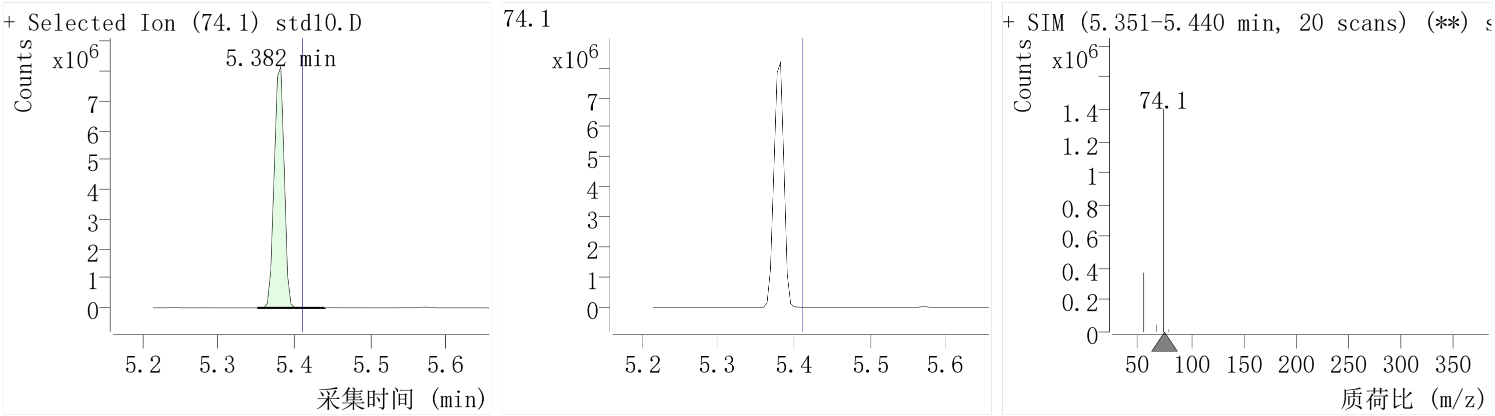

C14:0

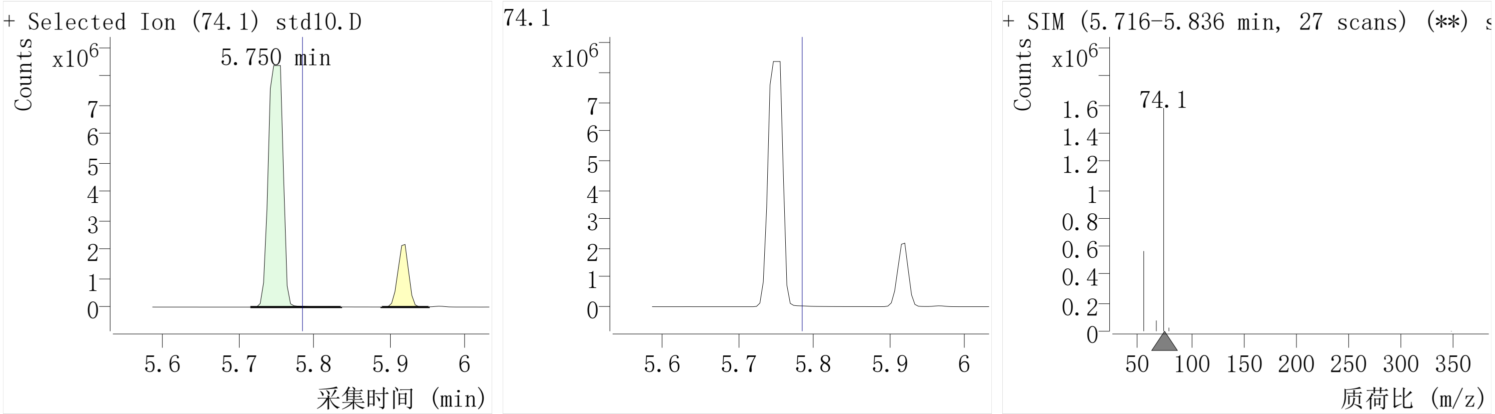

C14:1

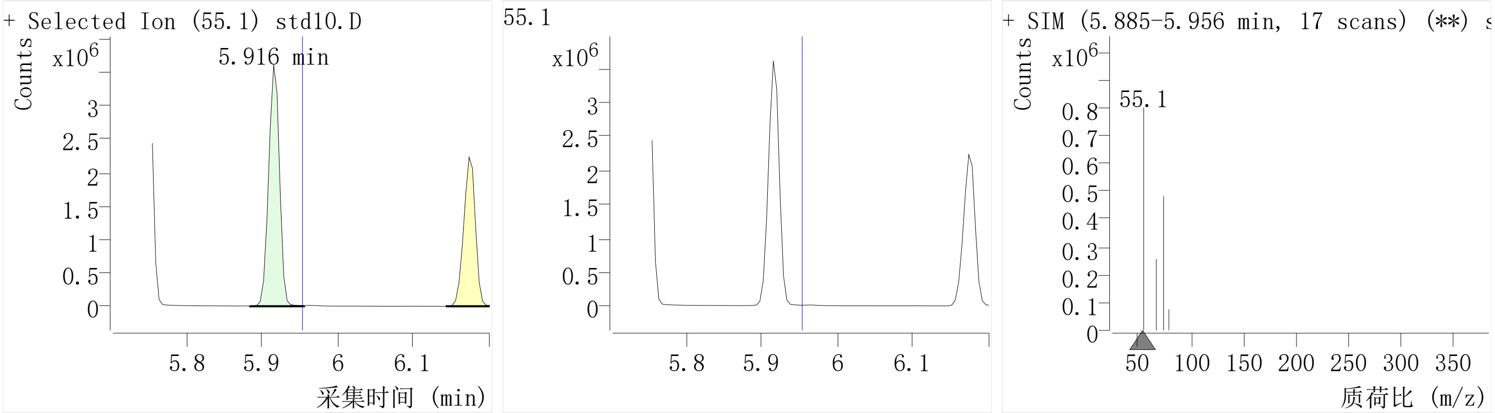

C15:0

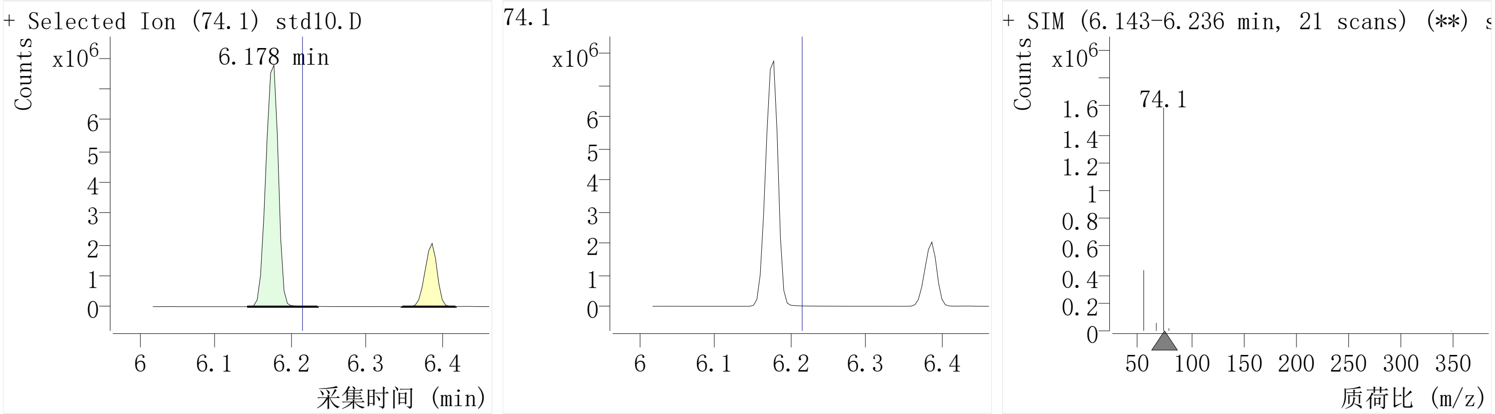

C15:1

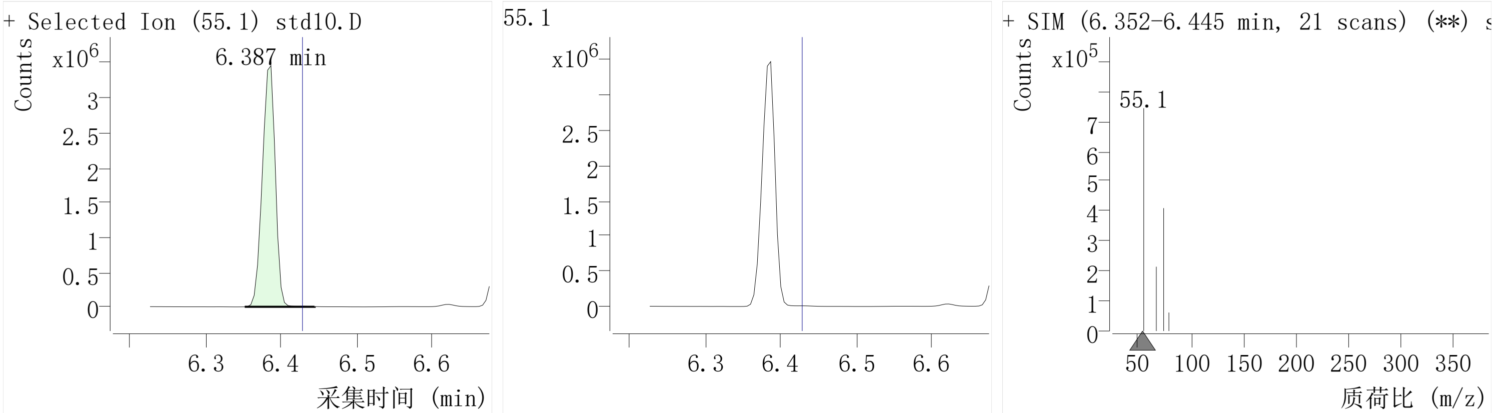

C16:0

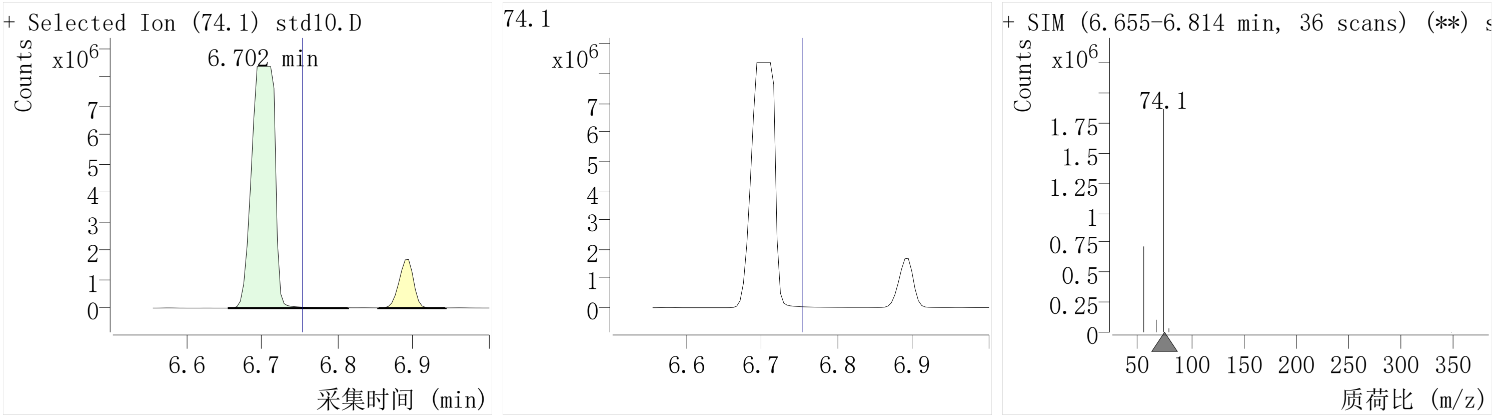

C16:1

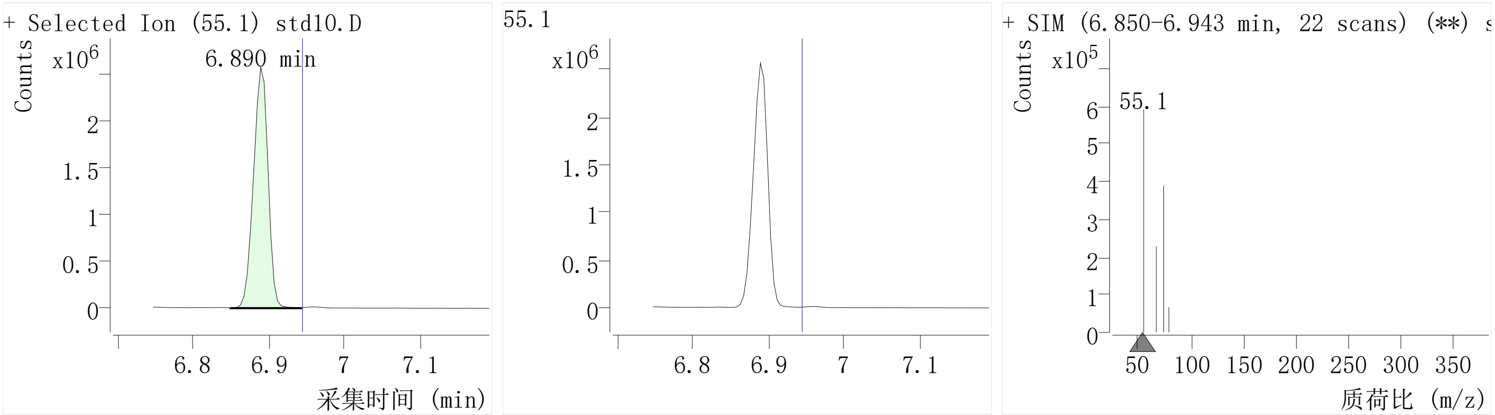

C17:0

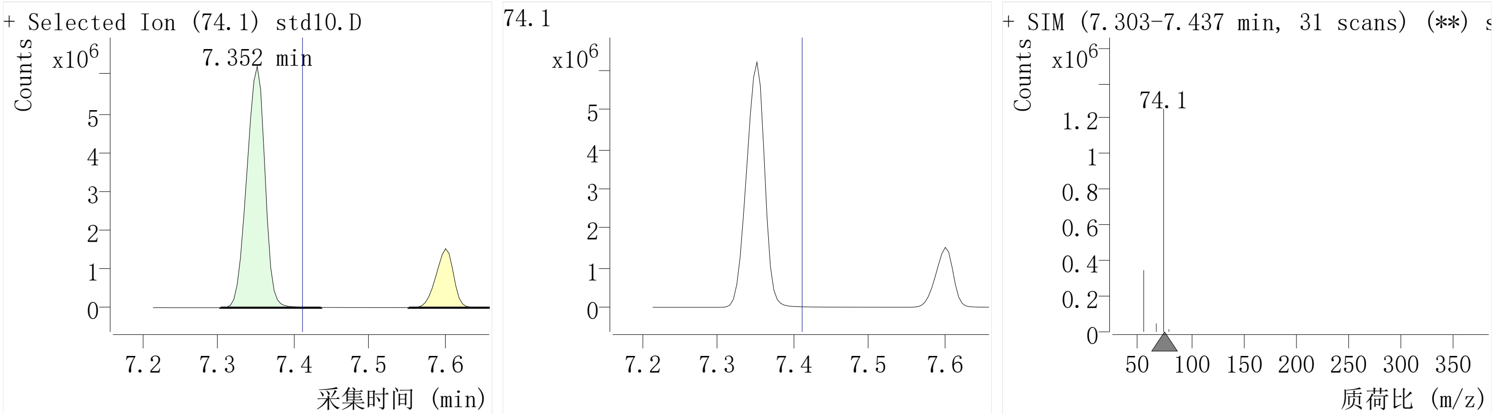

C17:1

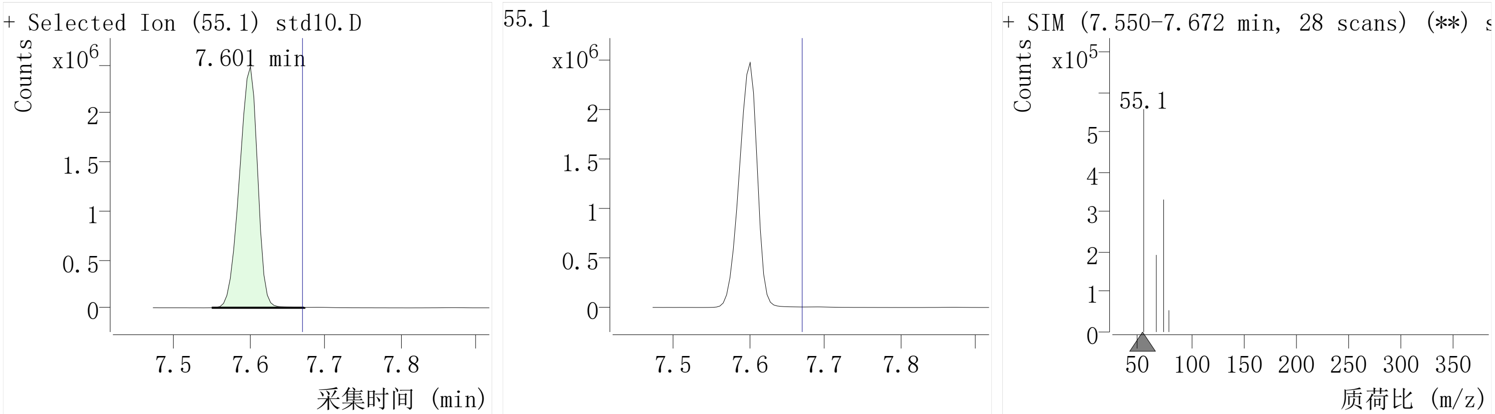

C18:0

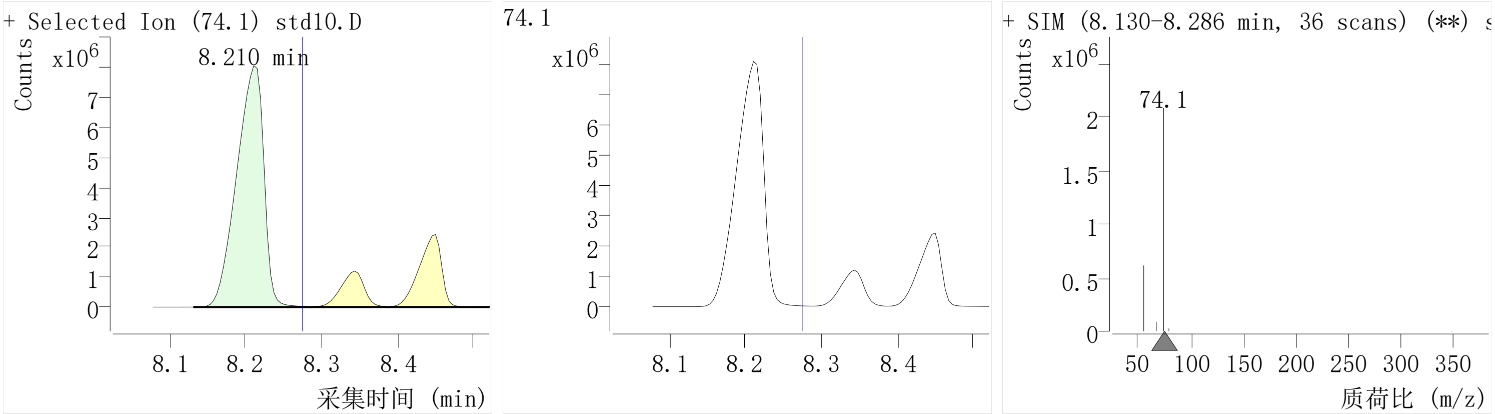

C18:1n9t

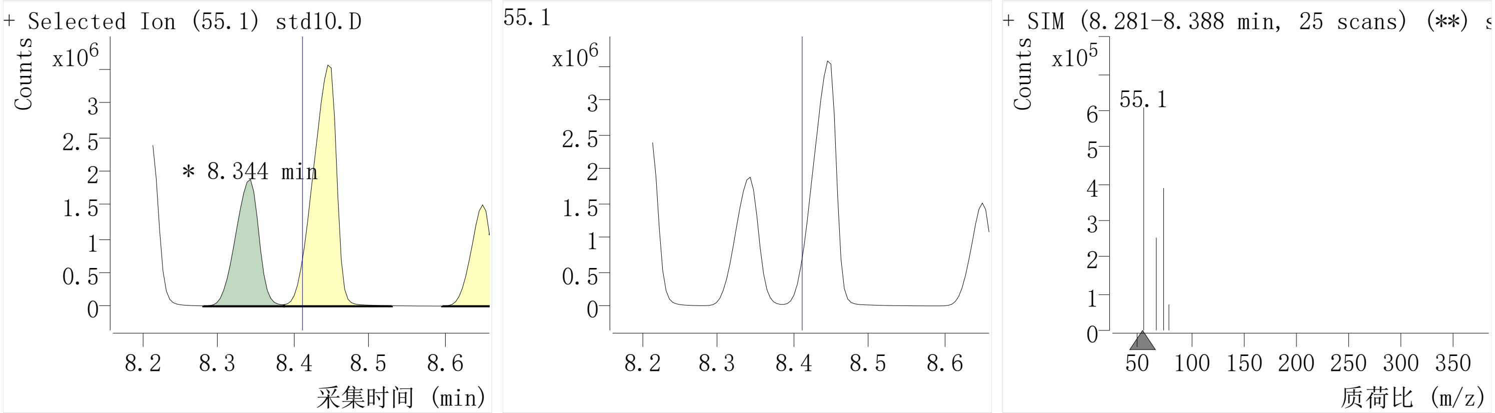

C18:1n9c

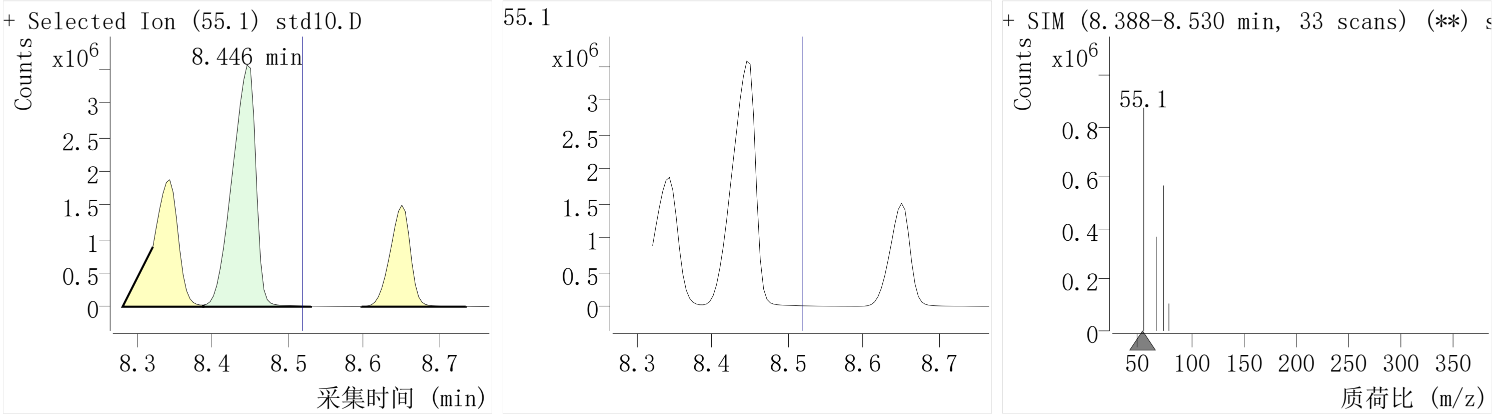

C18:2n6t

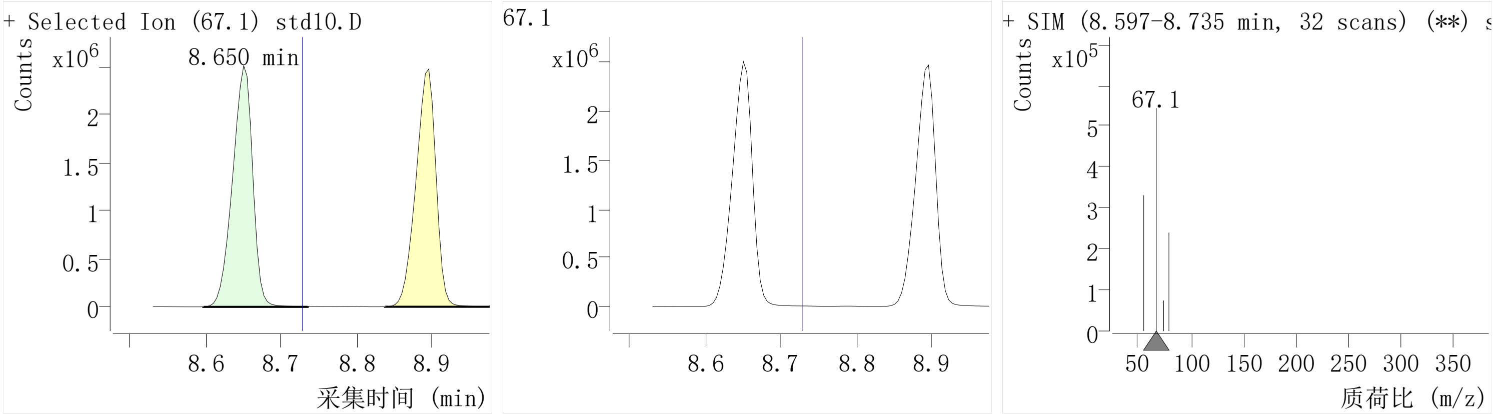

C18:2n6c

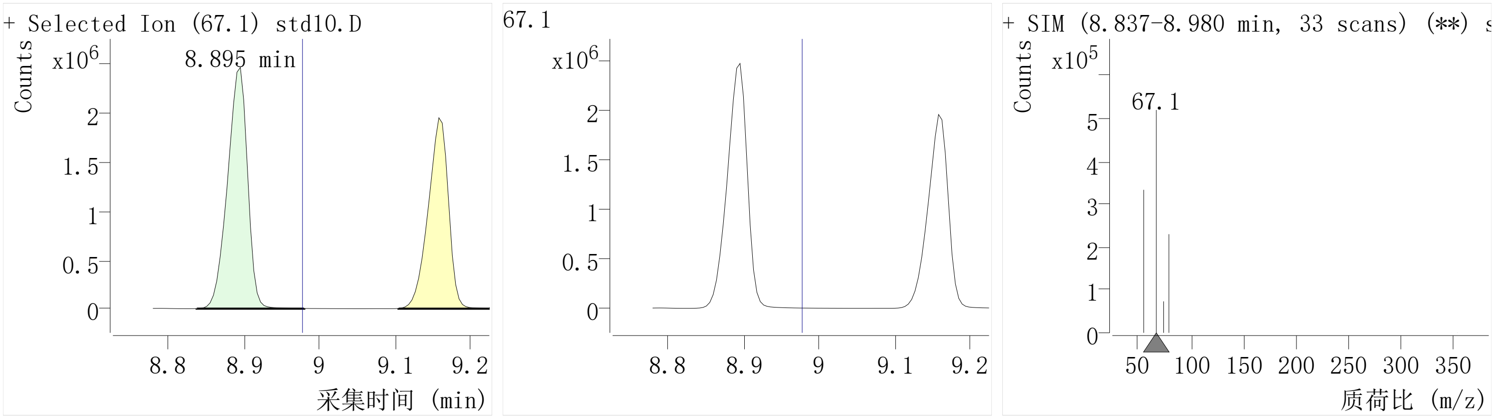

C18:3n6

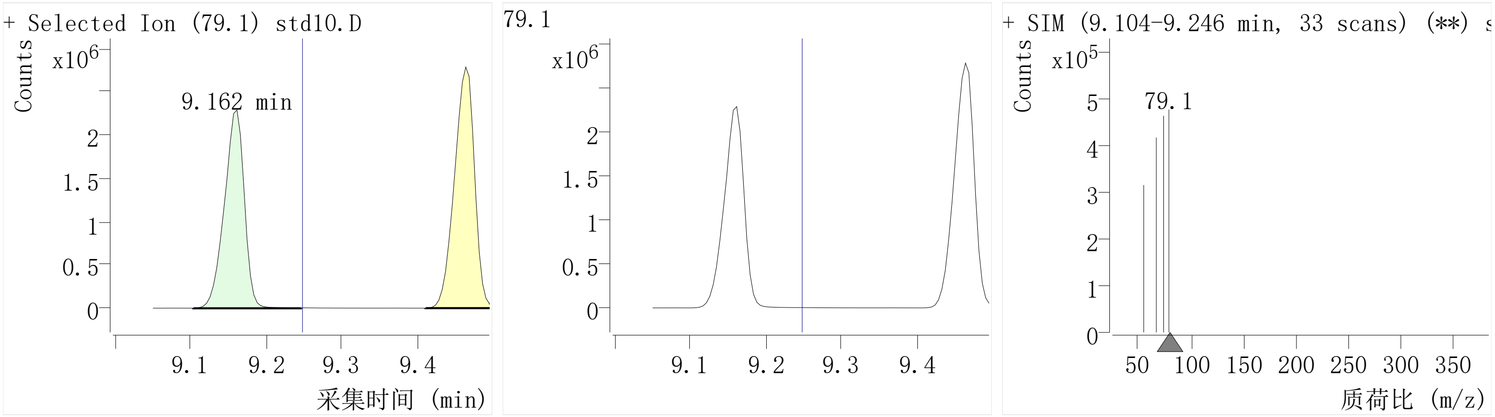

C18:3n3

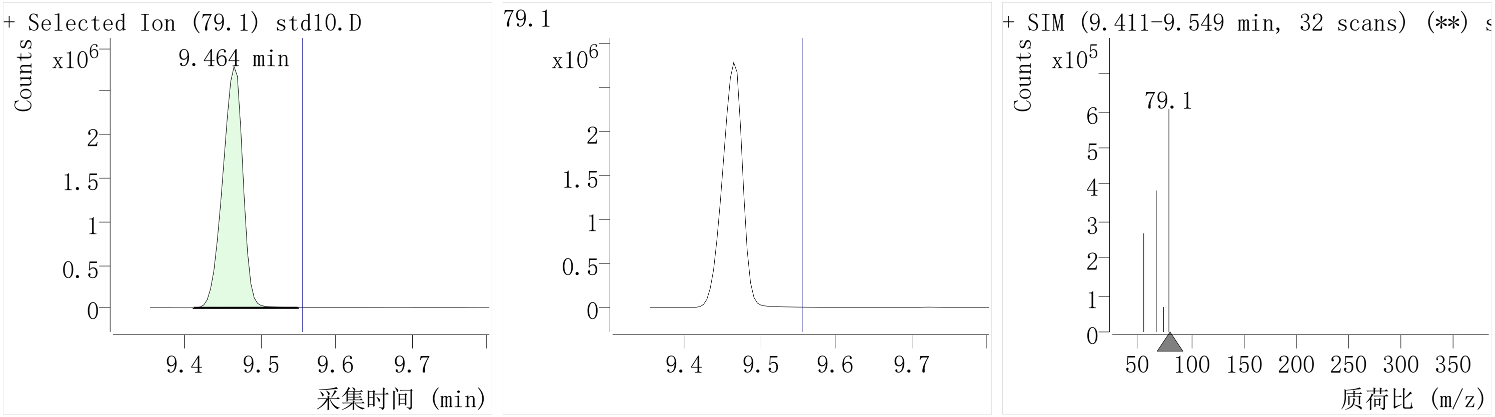

C20:0

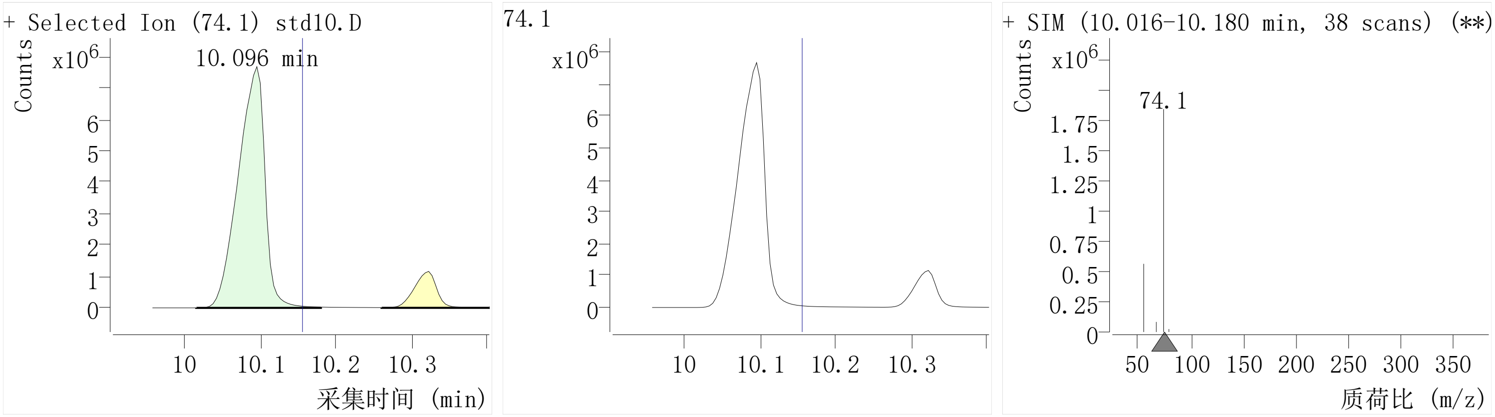

C20:1

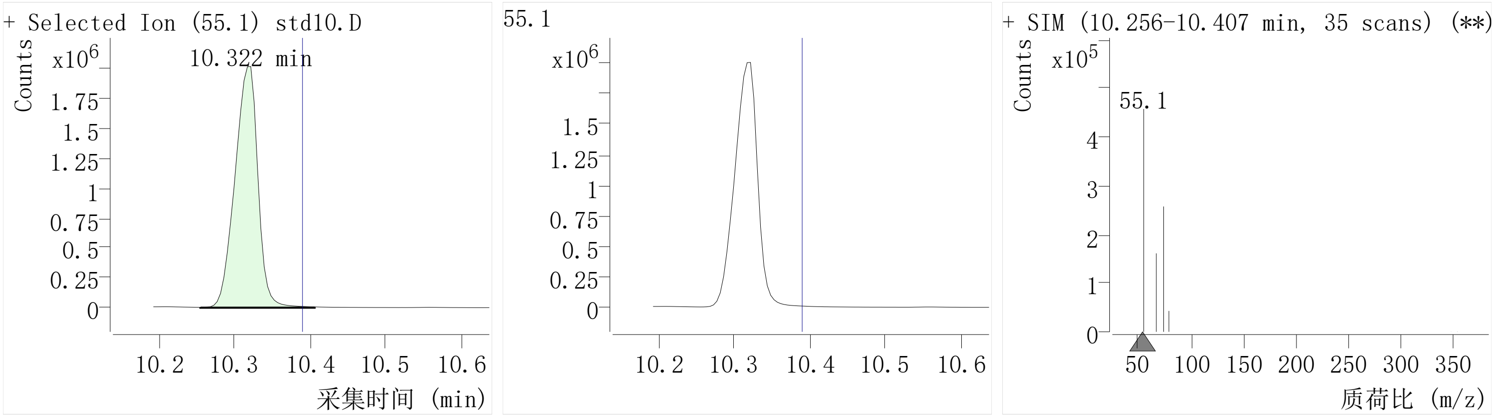

C20:2

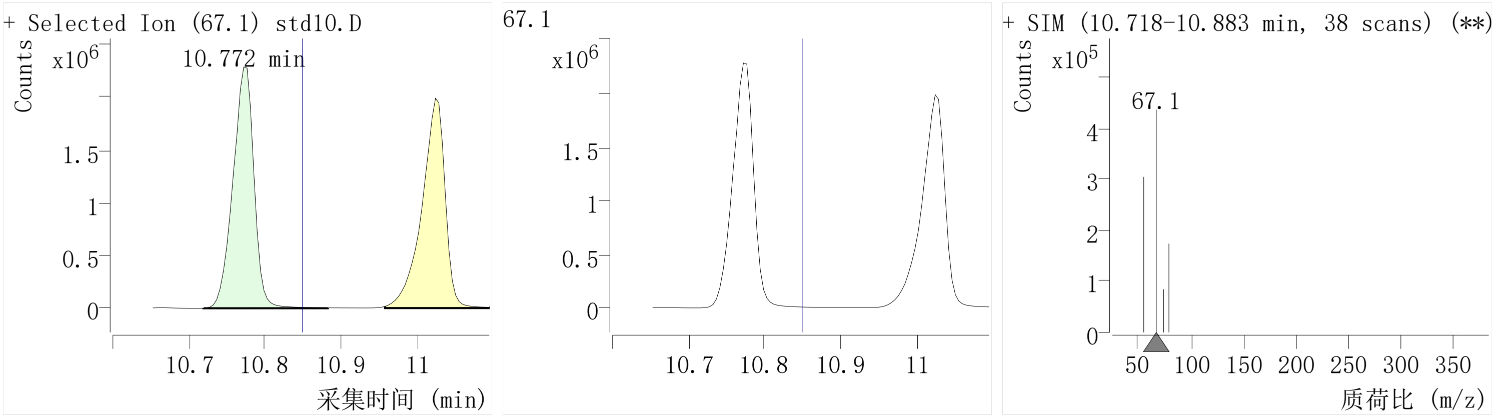

C21:0

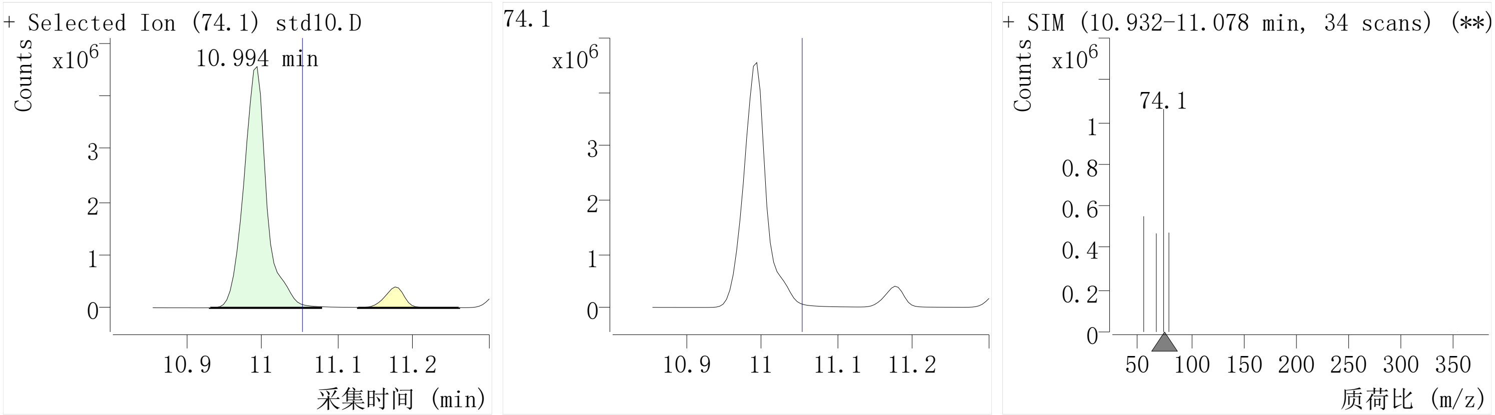

C20:3n6

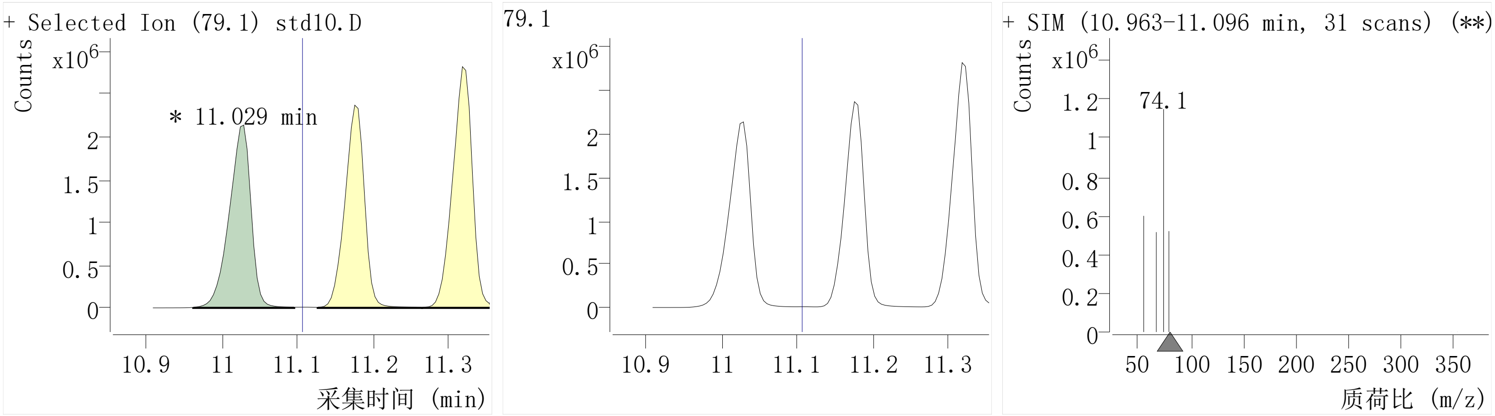

C20:4n6

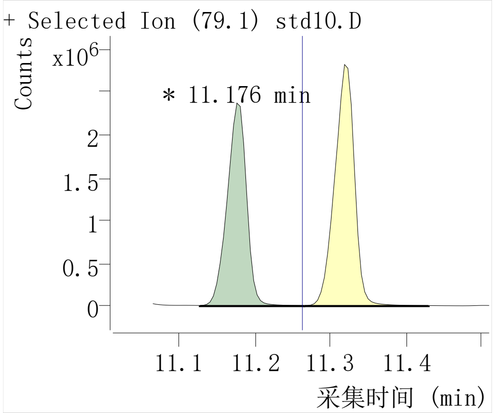

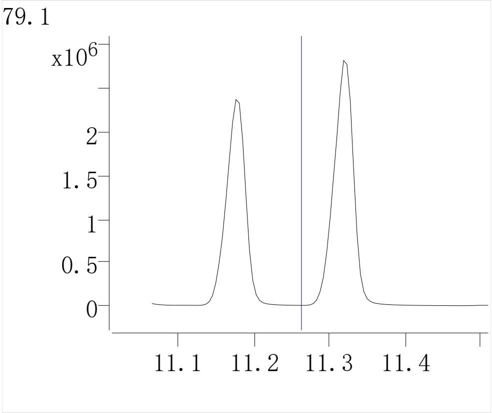

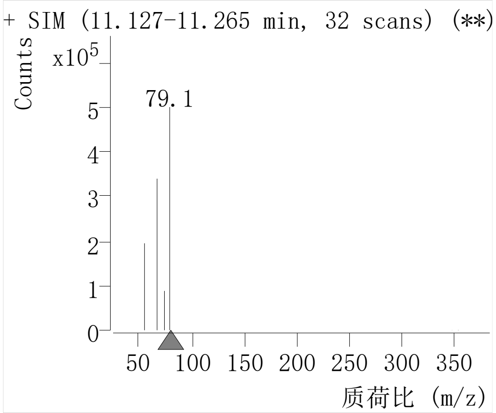

C20:3n3

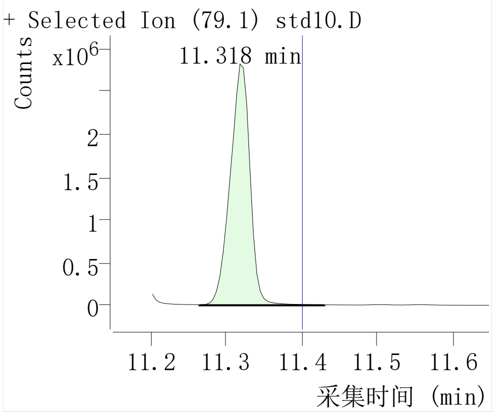

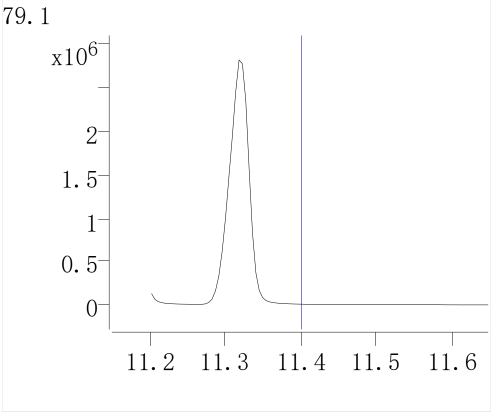

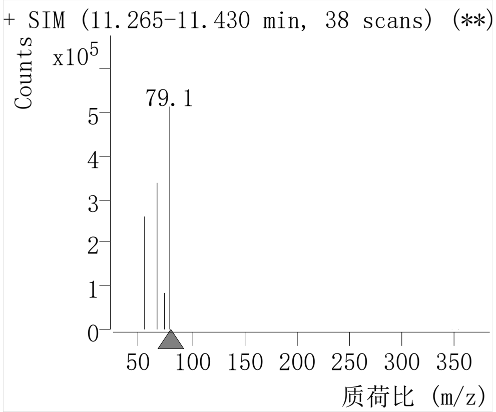

C20:5n3

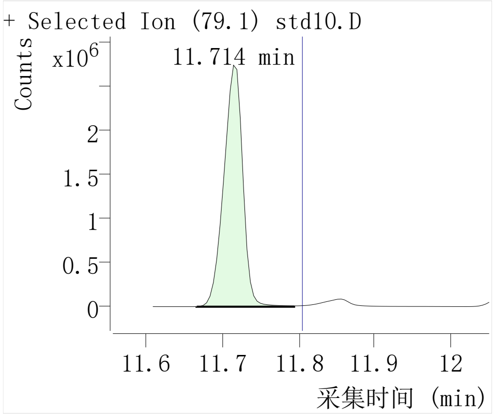

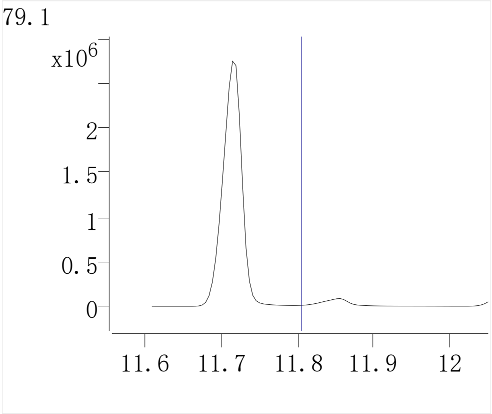

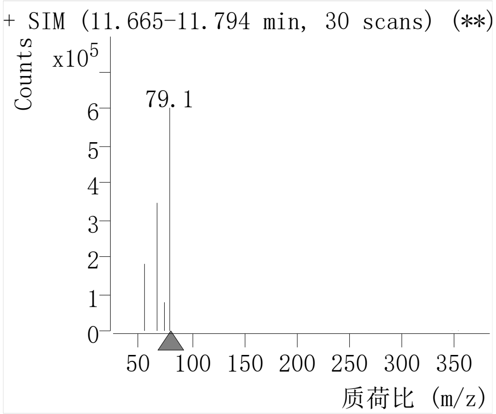

C22:0

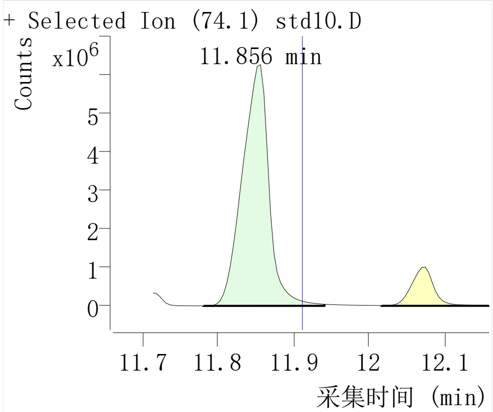

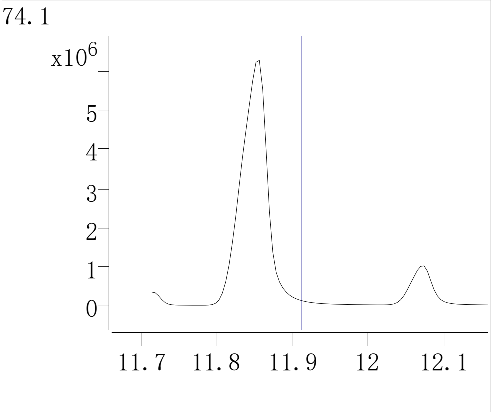

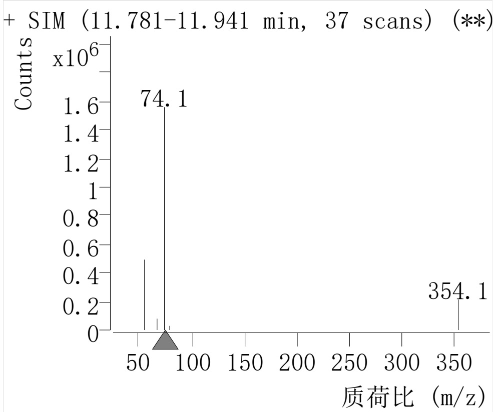

C22:1n9

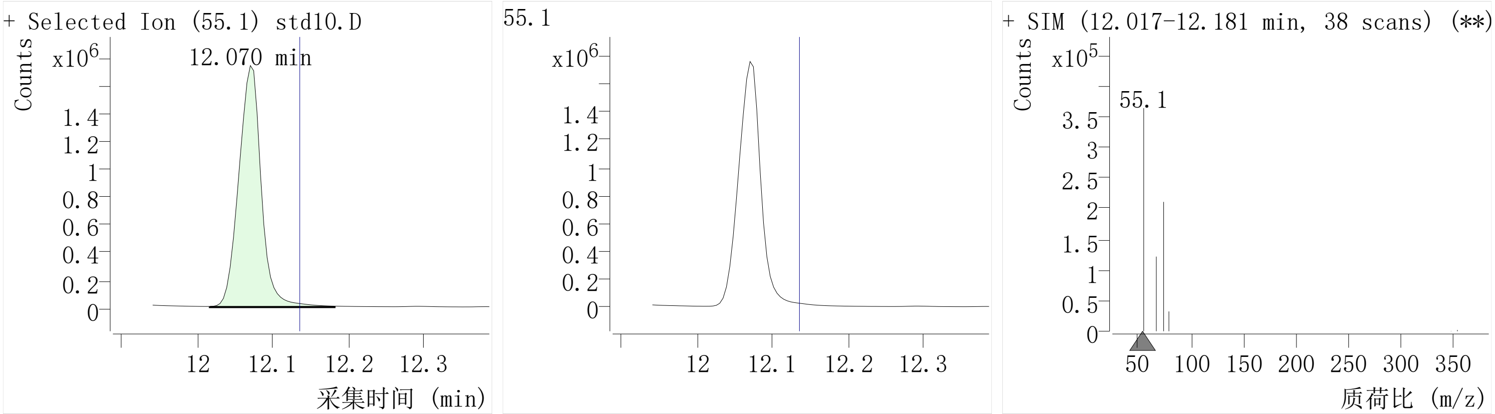

C22:2n6

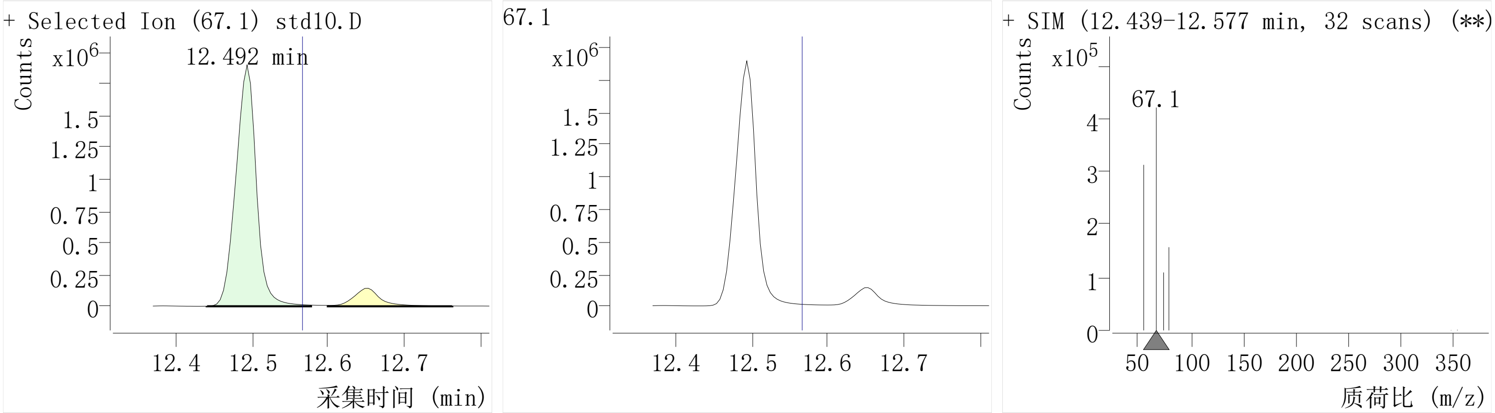

C23:0

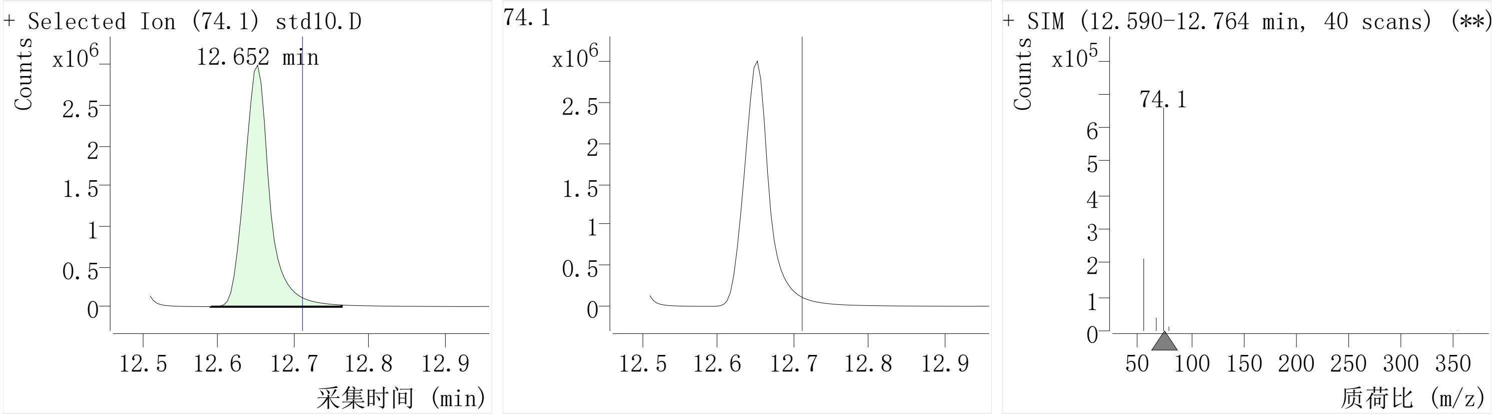

C24:0

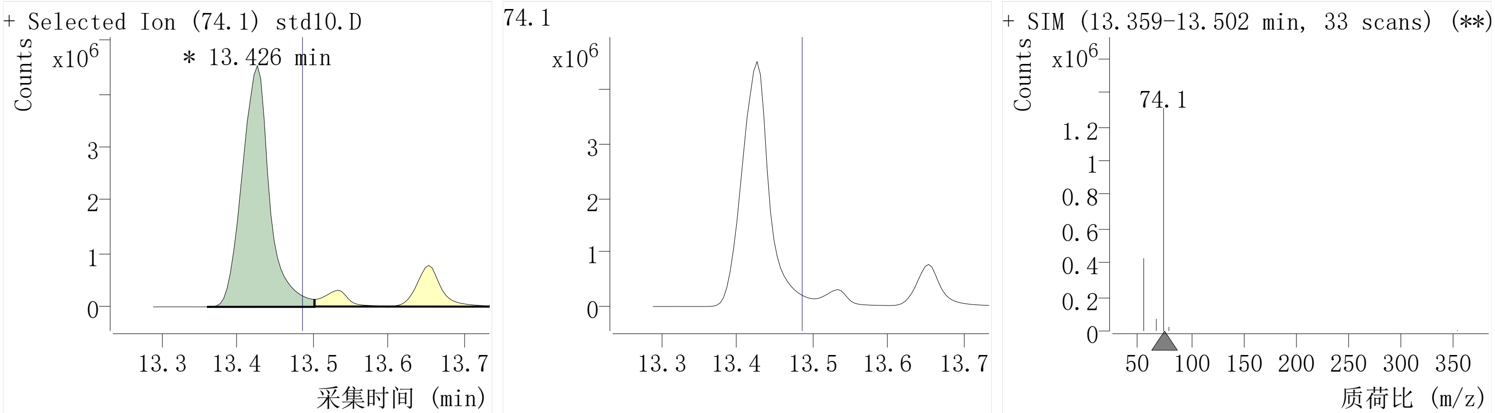

C22:6

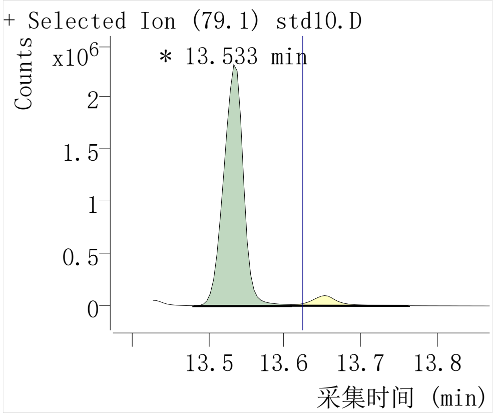
+ Selected Ion (79.1) std10.D
Counts
x10^6
* 13.533 min
采集时间 (min)

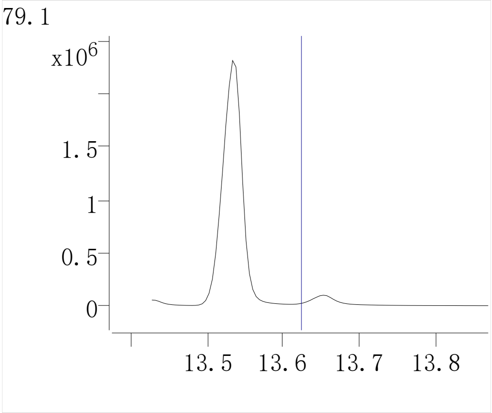
79.1
x10^6

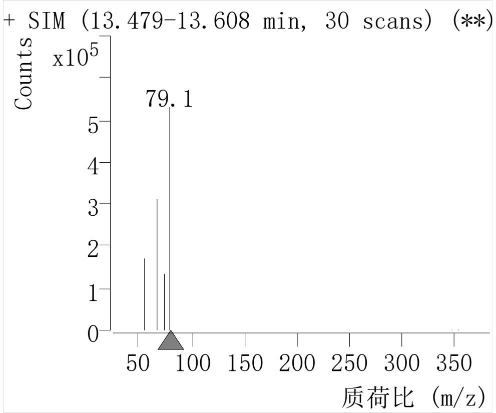
+ SIM (13.479-13.608 min, 30 scans) (**)
Counts
x10^5
79.1
质荷比 (m/z)

C24:1

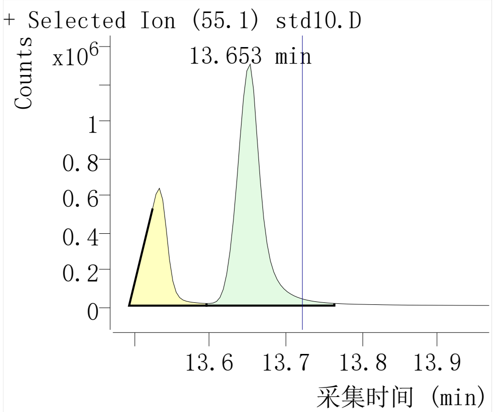
+ Selected Ion (55.1) std10.D
Counts
x10^6
13.653 min
采集时间 (min)

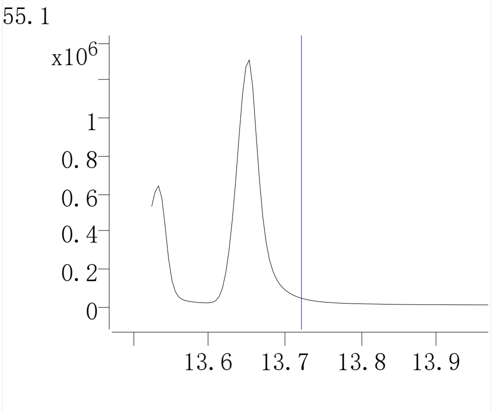
55.1
x10^6

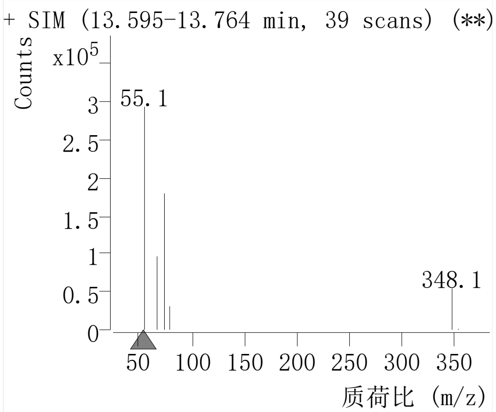
+ SIM (13.595-13.764 min, 39 scans) (**)
Counts
x10^5
55.1
348.1
质荷比 (m/z)

定量分析完成报告

**批处理路径**	G:\GC-MS\HX250430-4-GCMS总脂肪酸靶向检测\HX250430-4\QuantResults\HX250430-4.batch.bin		
**分析时间**	2025/5/14 16:58	**分析员姓名**	DESKTOP-M3A0GPO\omics
**报告时间**	2025/5/16 14:53:13	**报告员姓名**	DESKTOP-M3A0GPO\omics
**最近校正更新**	2025/5/14 16:58	**批处理状态**	已处理
**定量批处理版本**	10.2	**定量报告版本**	10.2

**采集时间**	2025/5/9 3:35	**数据文件**	qc-3.D
**样品类型**	样品	**样品名称**	qc-3
**稀释**	1	**采集方法**	脂肪酸

样品色谱图

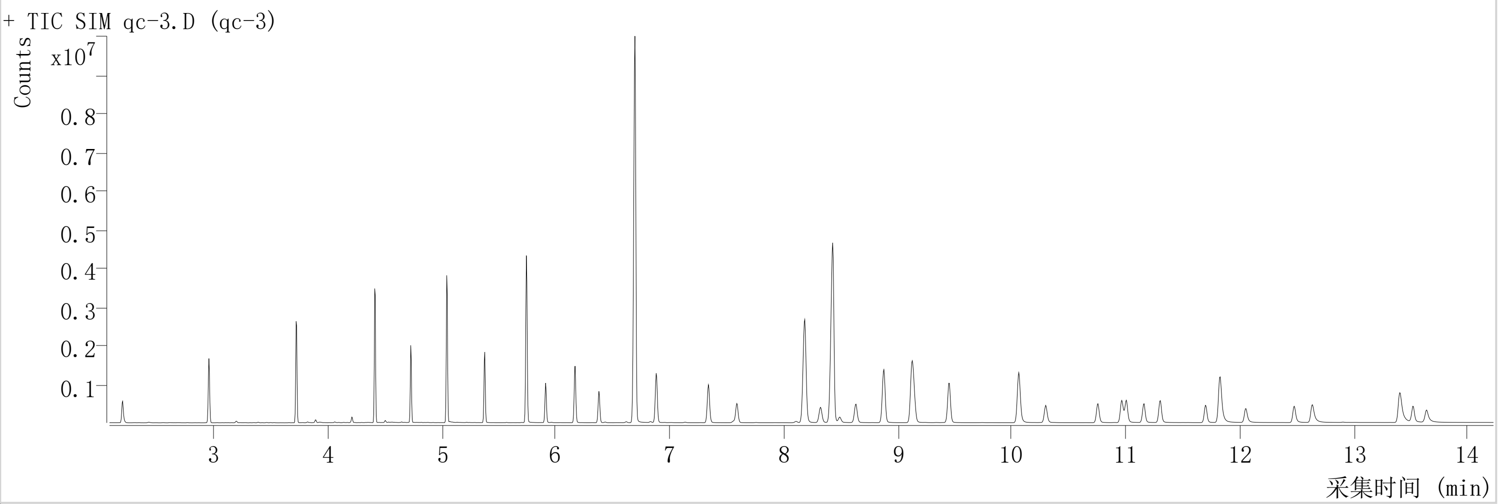

化合物	ISTD	RT	响应	ISTD 响应	响应比	最终浓度	单位
C4:0	C19:0	2.203	535644	2180317	0.2457	49.5185	ug/ml
C6:0	C19:0	2.959	1198829	2180317	0.5498	69.0697	ug/ml
C8:0	C19:0	3.724	1712973	2180317	0.7857	59.0314	ug/ml
C10:0	C19:0	4.417	2119359	2180317	0.9720	39.5682	ug/ml
C11:0	C19:0	4.729	1137858	2180317	0.5219	16.9207	ug/ml
C12:0	C19:0	5.044	2330569	2180317	1.0689	30.2779	ug/ml
C13:0	C19:0	5.378	1153928	2180317	0.5292	12.9973	ug/ml
C14:0	C19:0	5.742	3045063	2180317	1.3966	31.1327	ug/ml
C14:1	C19:0	5.911	469695	2180317	0.2154	10.7596	ug/ml
C15:0	C19:0	6.169	1139469	2180317	0.5226	11.0532	ug/ml
C15:1	C19:0	6.378	504854	2180317	0.2316	11.6560	ug/ml
C16:0	C19:0	6.694	9869330	2180317	4.5266	143.6910	ug/ml
C16:1	C19:0	6.881	776605	2180317	0.3562	21.6787	ug/ml
C17:0	C19:0	7.339	1100187	2180317	0.5046	10.5274	ug/ml
C17:1	C19:0	7.588	413093	2180317	0.1895	10.1754	ug/ml
C18:0	C19:0	8.184	3902534	2180317	1.7899	38.7198	ug/ml
C18:1n9t	C19:0	8.321	358828	2180317	0.1646	9.7083	ug/ml
C18:1n9c	C19:0	8.428	4022699	2180317	1.8450	118.5490	ug/ml
C18:2n6t	C19:0	8.633	400318	2180317	0.1836	10.0840	ug/ml
C18:2n6c	C19:0	8.877	1118607	2180317	0.5130	29.1849	ug/ml
C18:3n6	C19:0	9.144	400931	2180317	0.1839	9.5495	ug/ml
C18:3n3	C19:0	9.451	833295	2180317	0.3822	18.9956	ug/ml
C20:0	C19:0	10.060	1902344	2180317	0.8725	22.0423	ug/ml
C20:1	C19:0	10.296	427042	2180317	0.1959	12.1098	ug/ml
C20:2	C19:0	10.754	385225	2180317	0.1767	10.8512	ug/ml
C21:0	C19:0	10.963	928336	2180317	0.4258	11.7133	ug/ml
C20:3n6	C19:0	11.003	352362	2180317	0.1616	10.2508	ug/ml
C20:4n6	C19:0	11.158	377529	2180317	0.1732	10.8324	ug/ml
C20:3n3	C19:0	11.301	437705	2180317	0.2008	10.5467	ug/ml
C20:5n3	C19:0	11.696	397486	2180317	0.1823	10.1108	ug/ml

定量分析完成报告

化合物	ISTD	RT	响应	ISTD 响应	响应比	最终浓度	单位
C22:0	C19:0	11.825	1761878	2180317	0.8081	26.1840	ug/ml
C22:1n9	C19:0	12.048	377018	2180317	0.1729	12.7867	ug/ml
C22:2n6	C19:0	12.475	342315	2180317	0.1570	12.4443	ug/ml
C23:0	C19:0	12.635	798063	2180317	0.3660	14.1161	ug/ml
C24:0	C19:0	13.400	1463832	2180317	0.6714	30.2805	ug/ml
C22:6	C19:0	13.520	376923	2180317	0.1729	12.7755	ug/ml
C24:1	C19:0	13.635	367375	2180317	0.1685	14.8562	ug/ml

C4:0

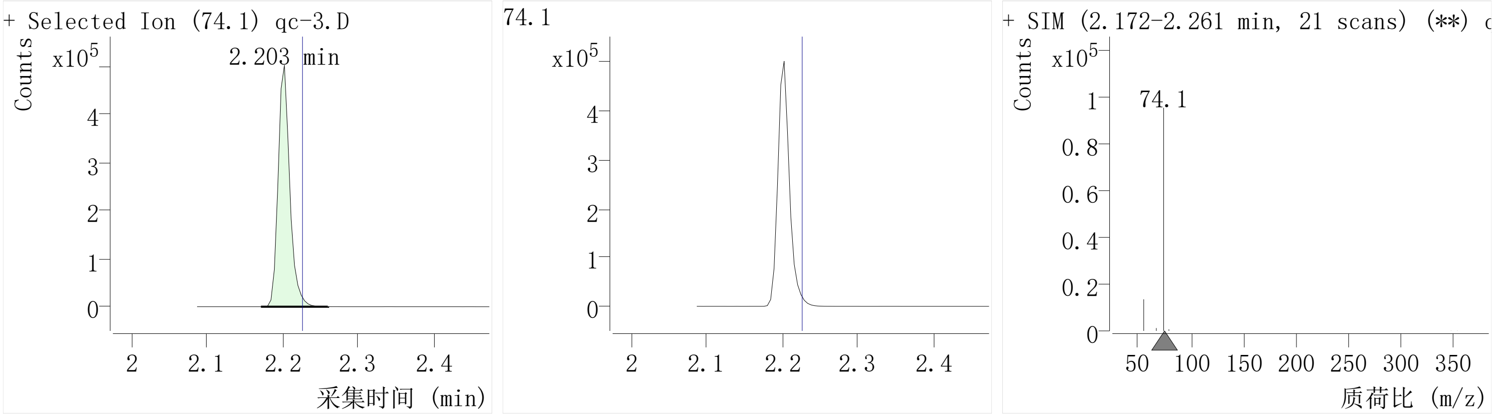

C6:0

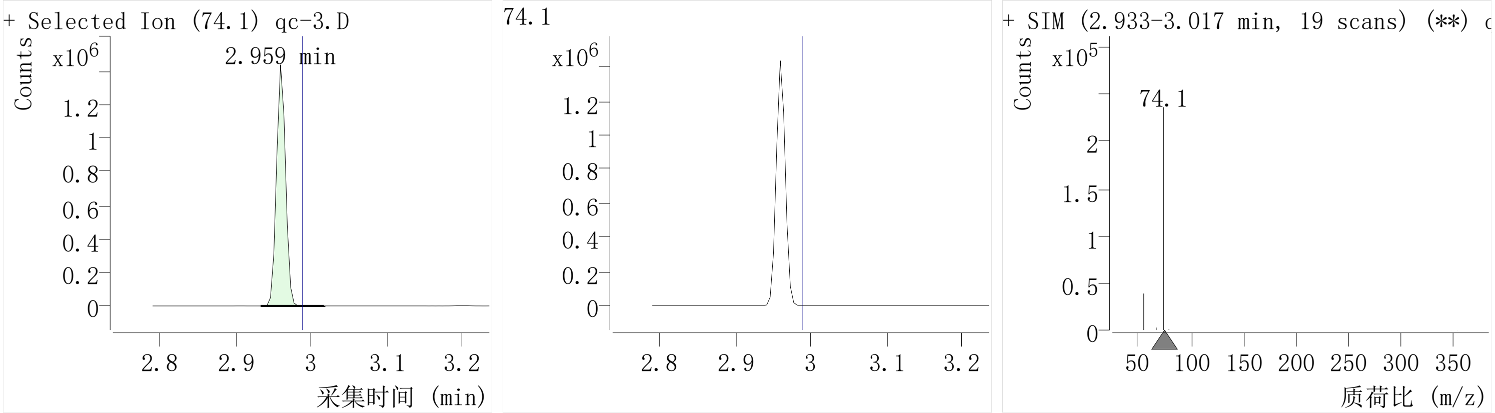

C8:0

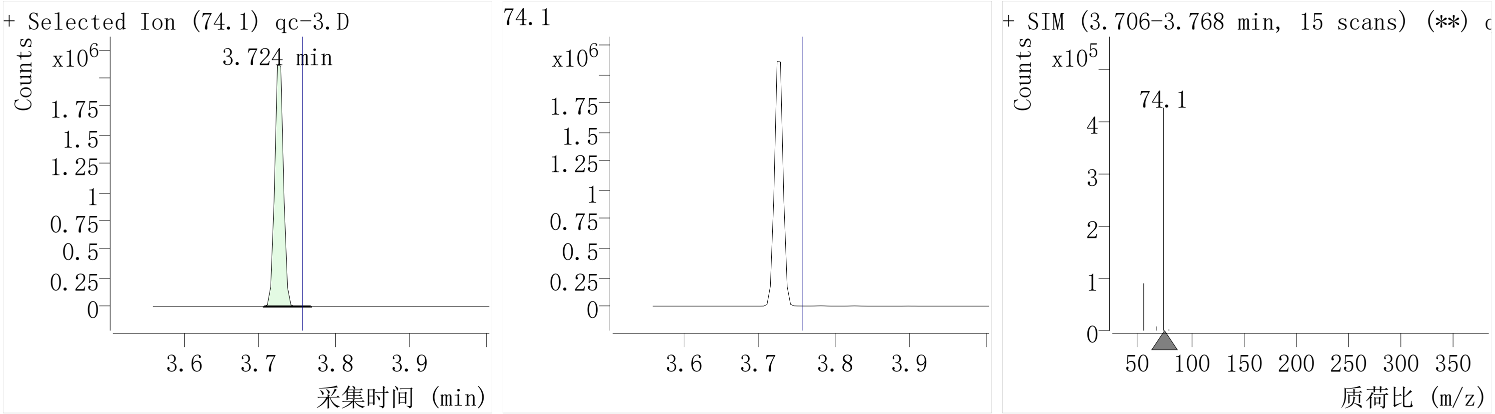

C10:0

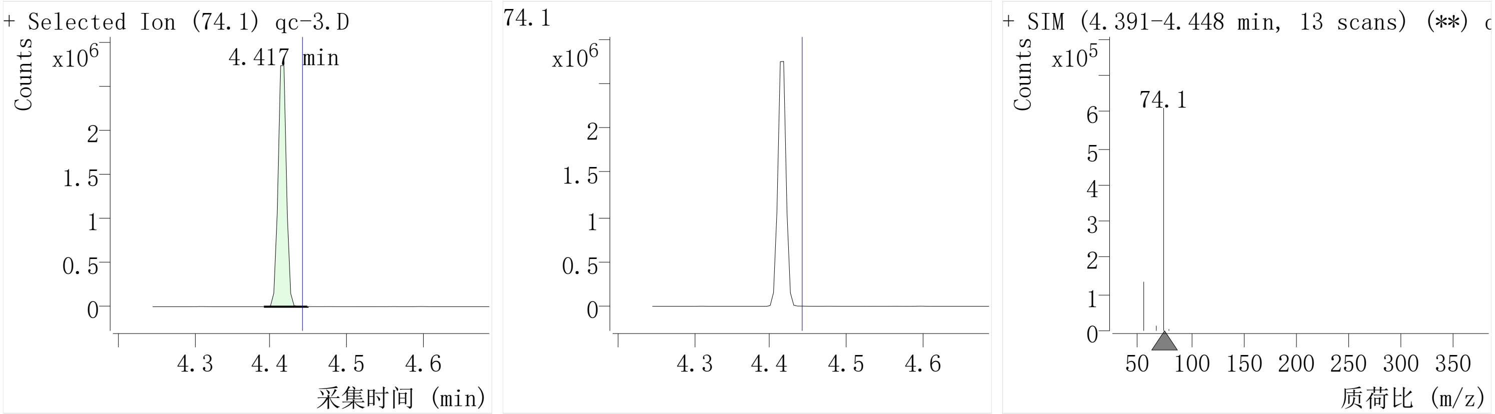

C11:0

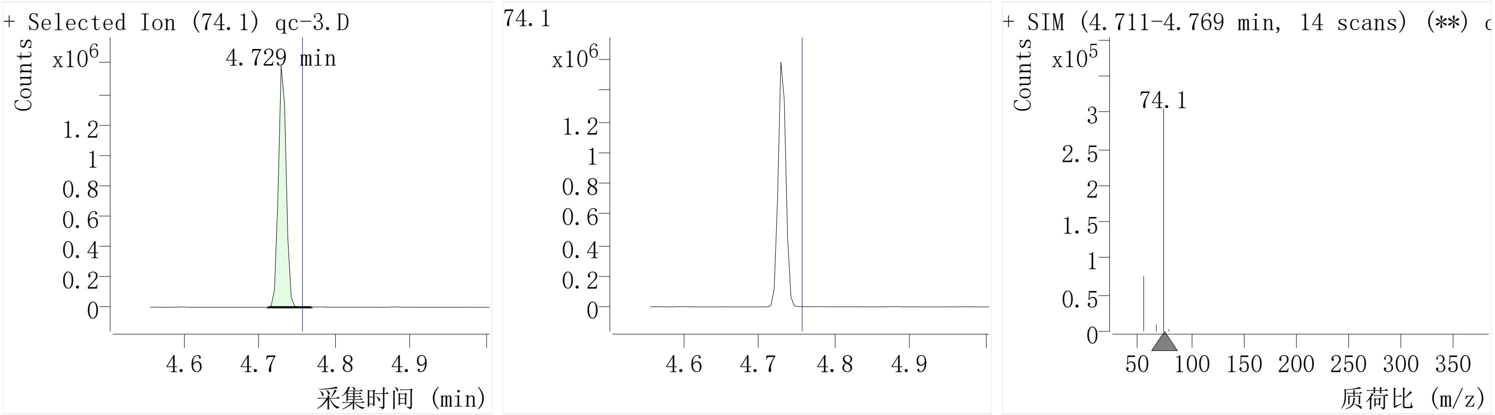

C12:0

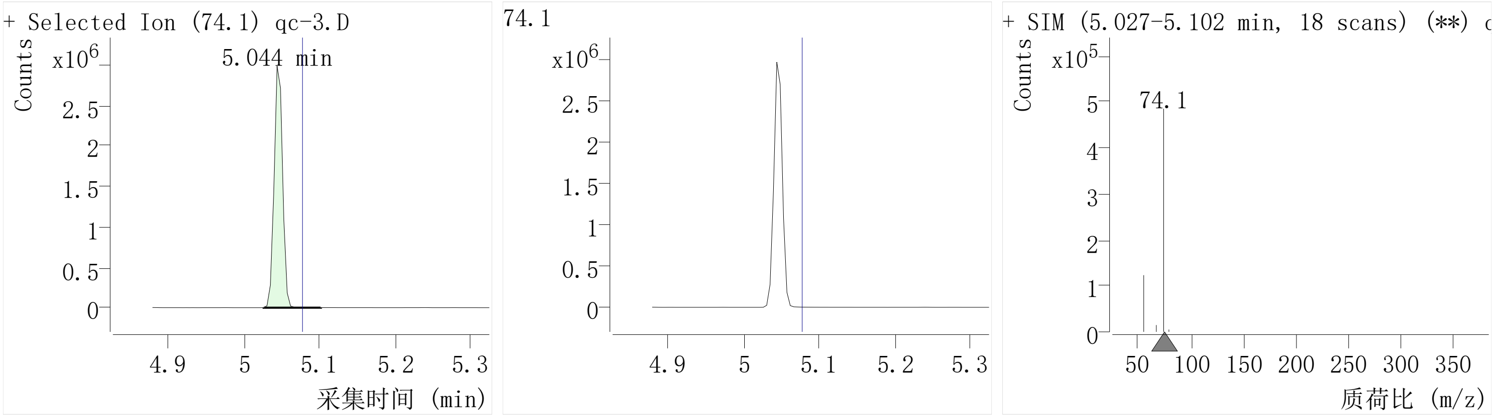

C13:0

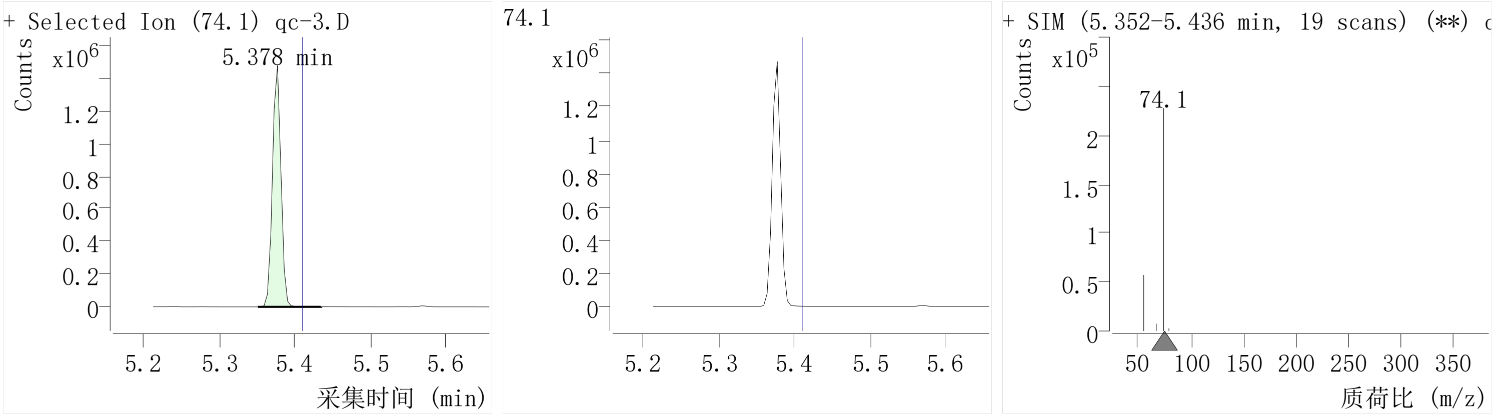

C14:0

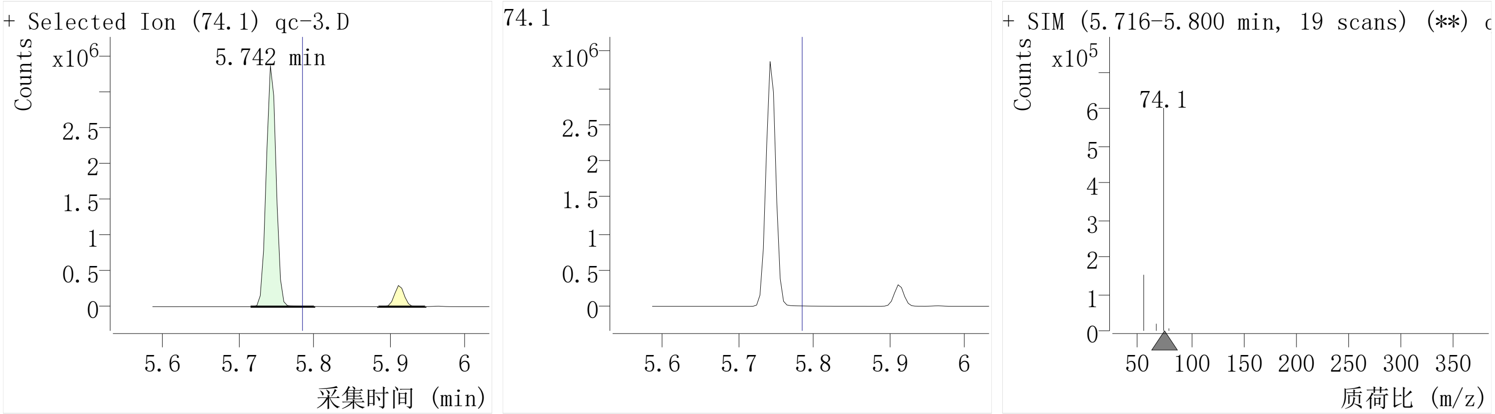

C14:1

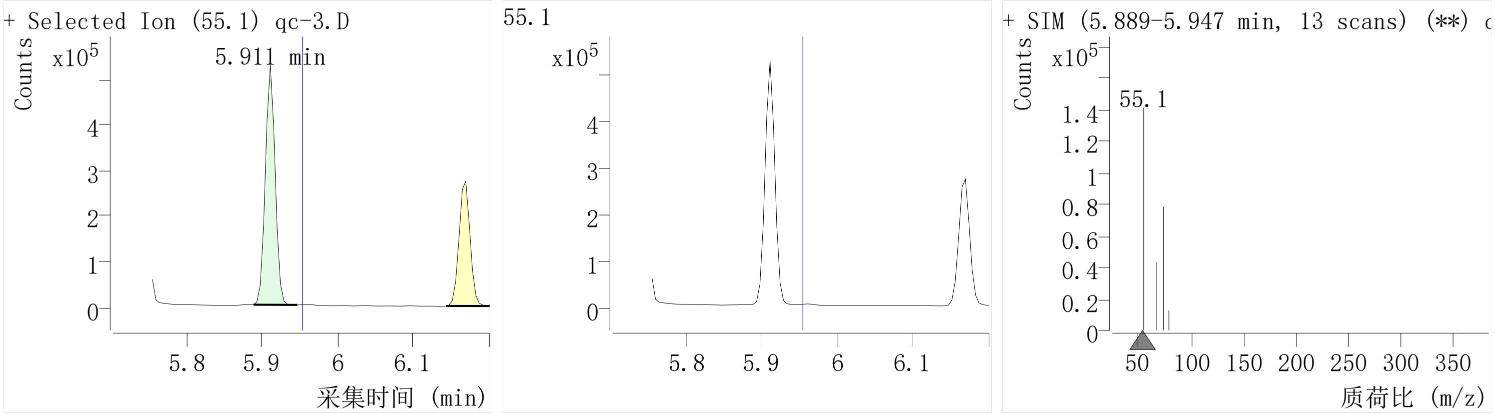

C15:0

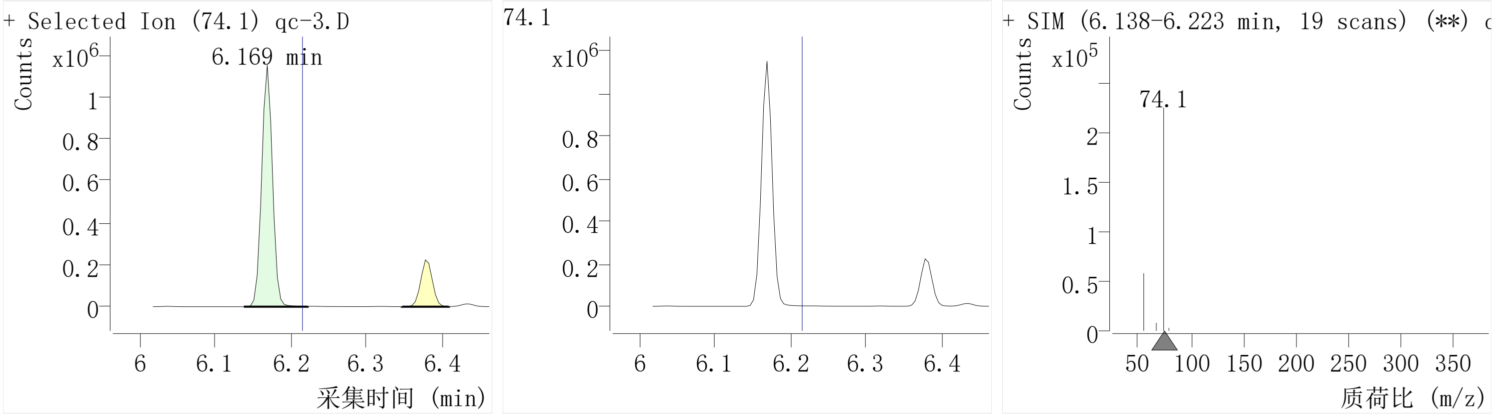

C15:1

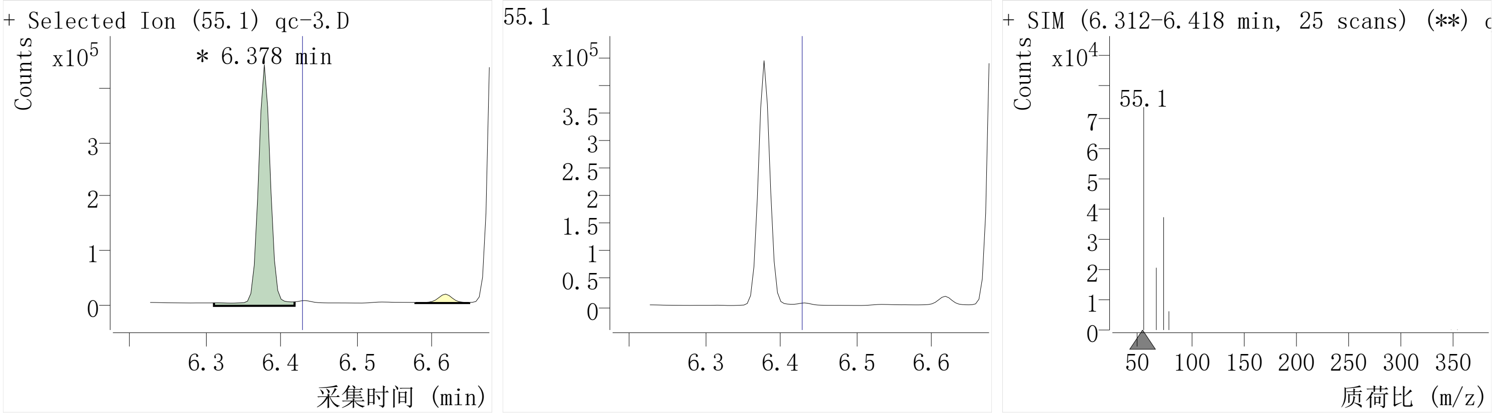

生成时间 2025/5/16 14:52

C16:0

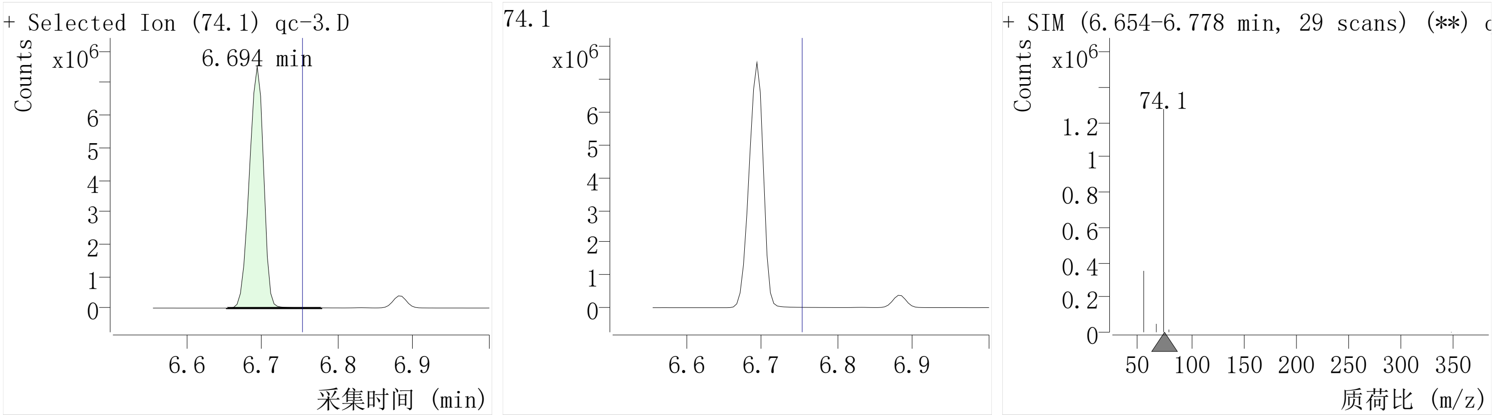

C16:1

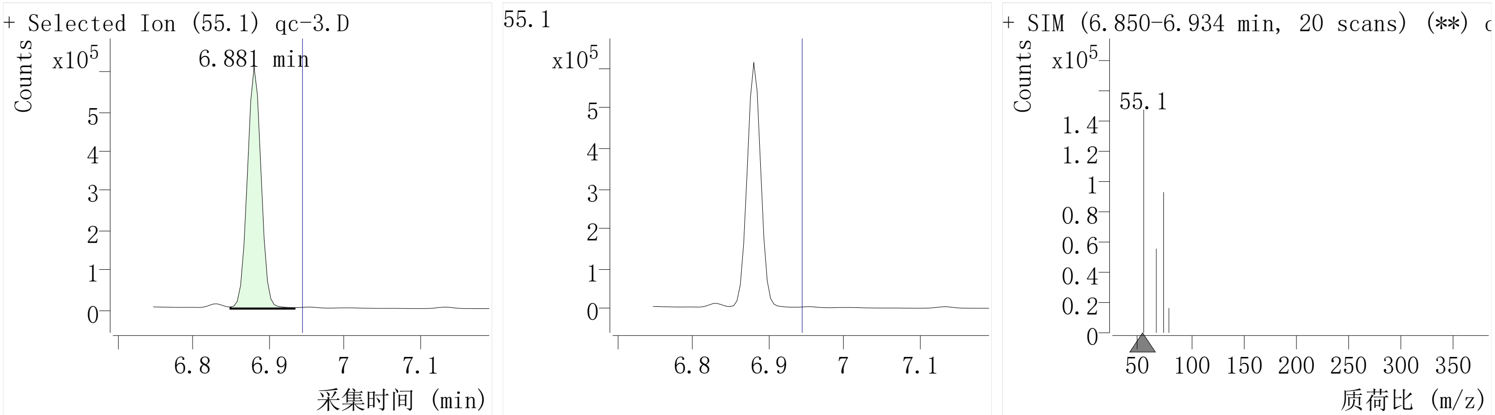

C17:0

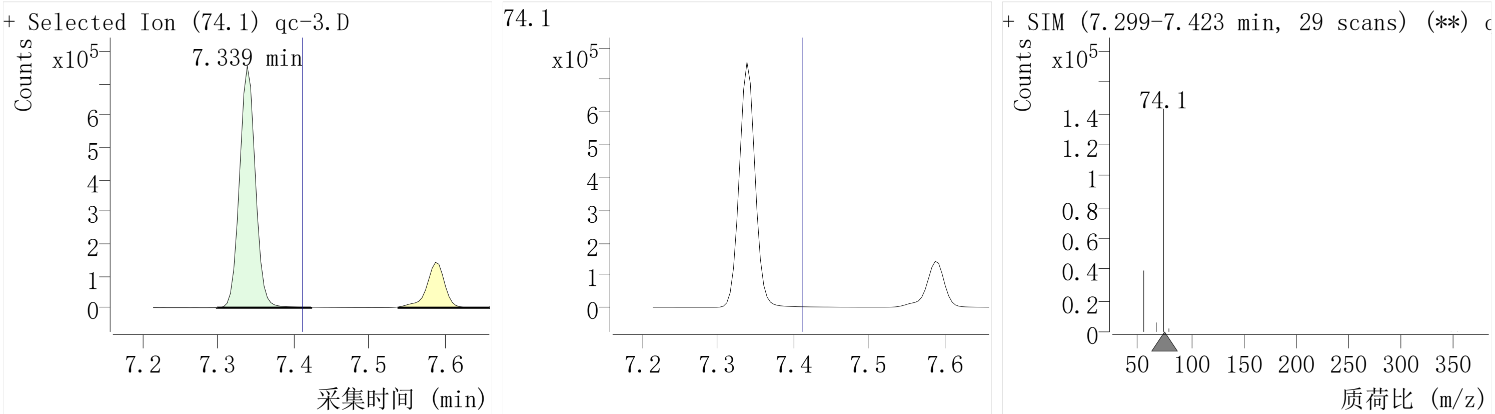

C17:1

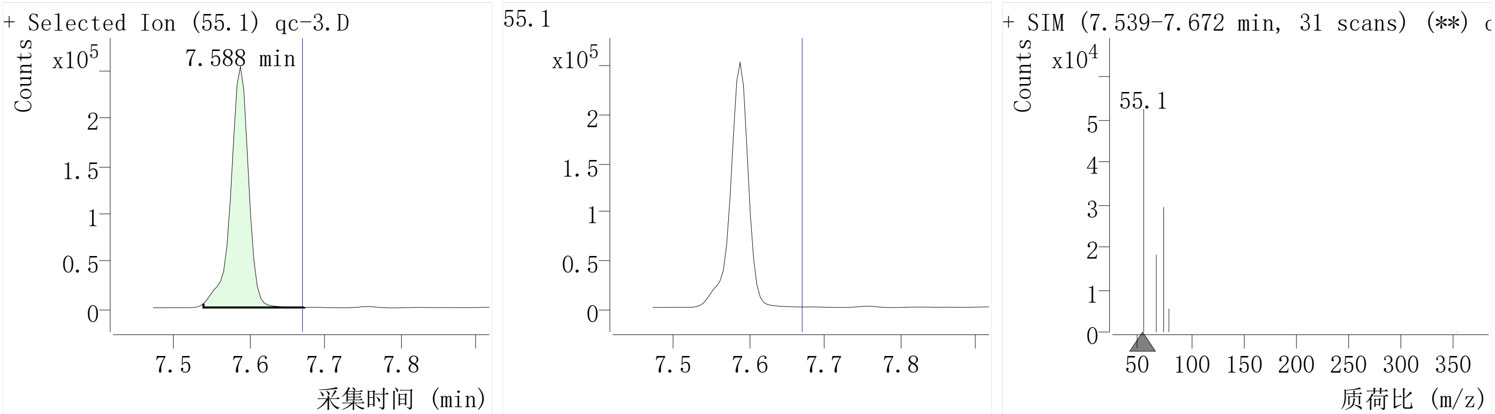

C18:0

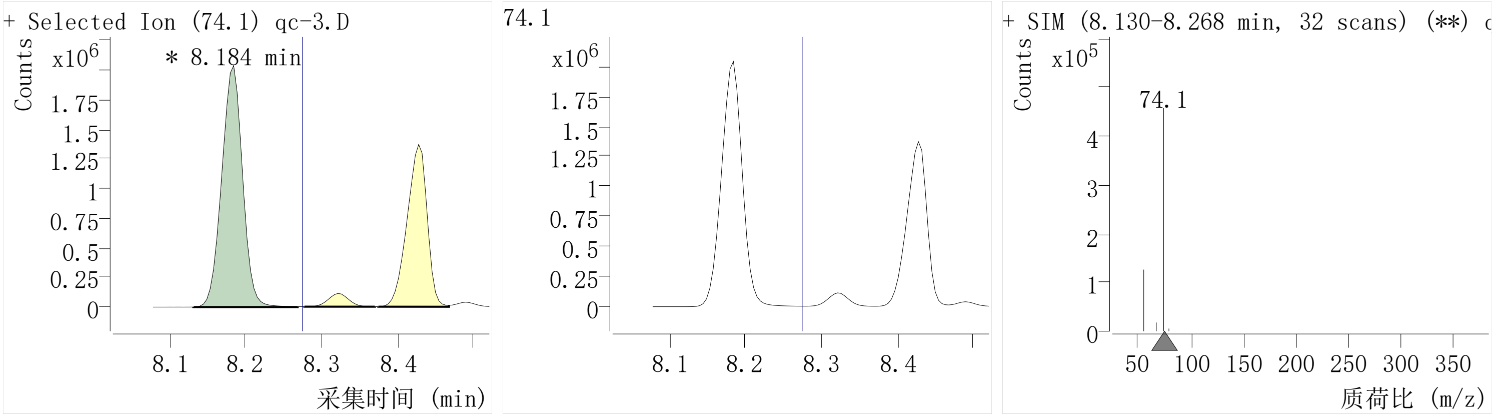

C18:1n9t

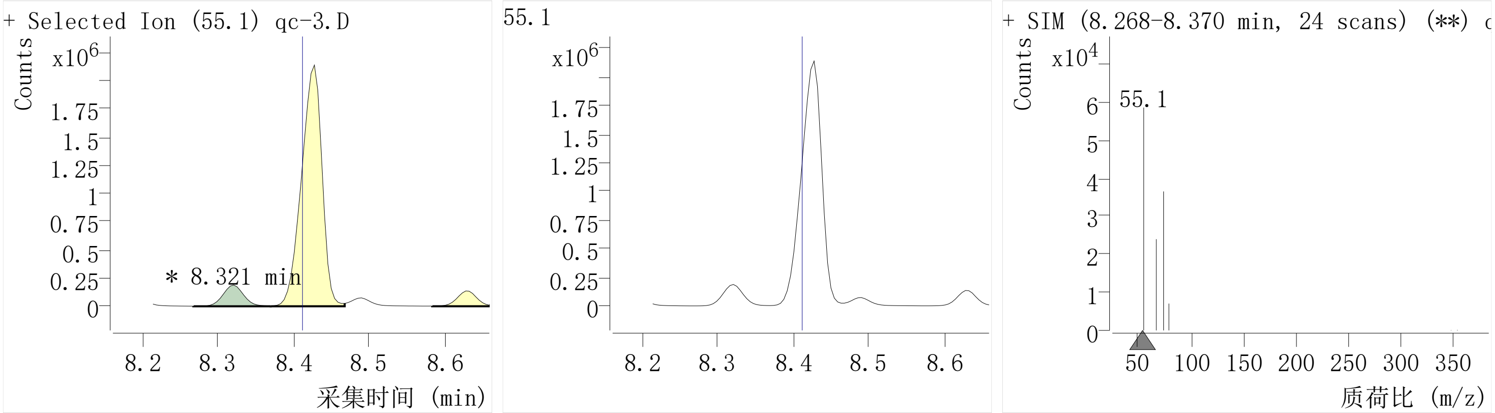

C18:1n9c

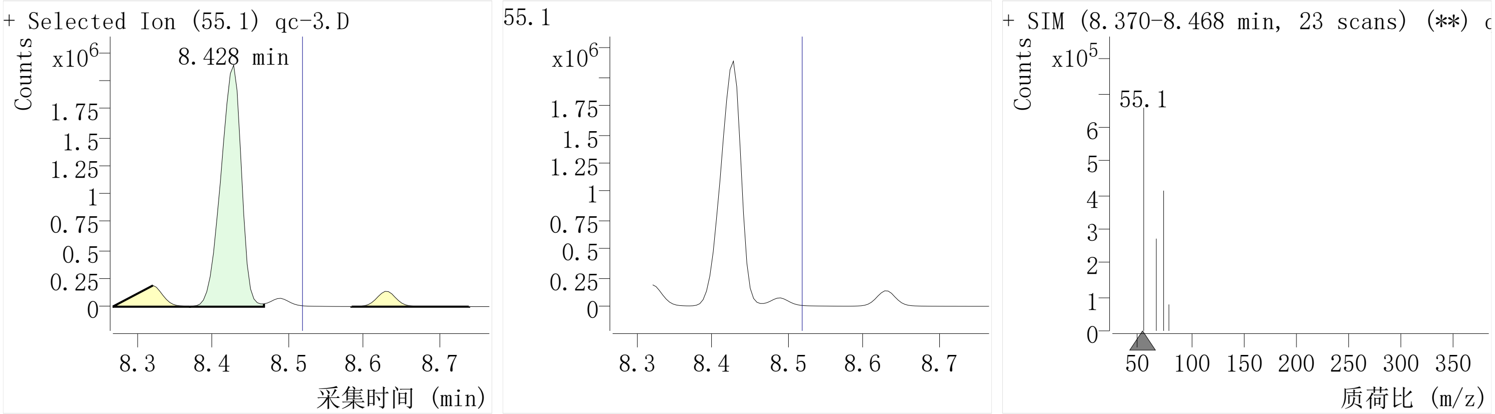

C18:2n6t

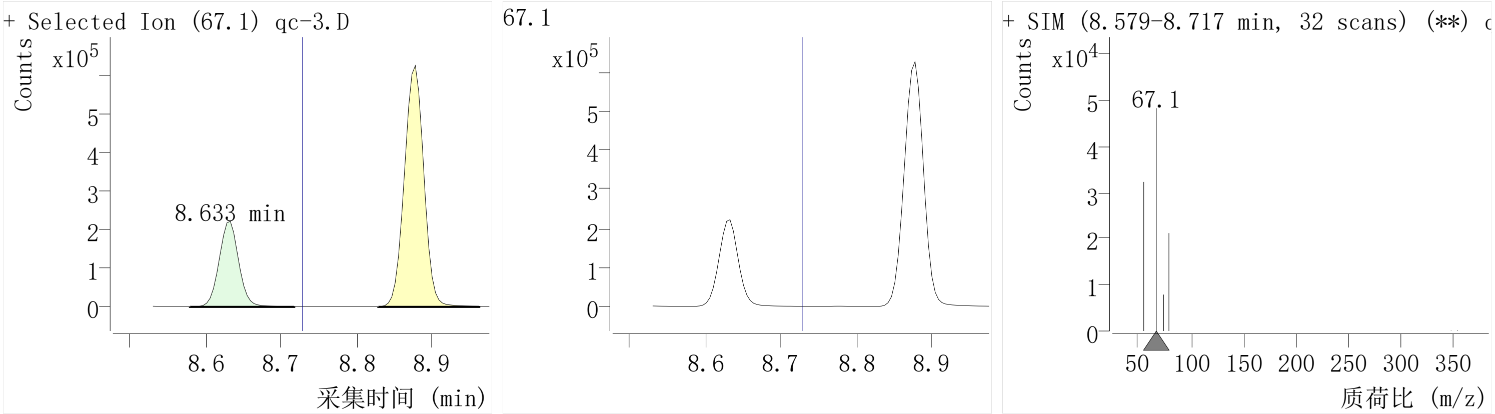

C18:2n6c

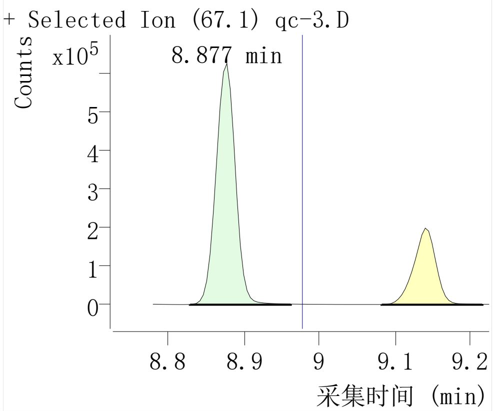

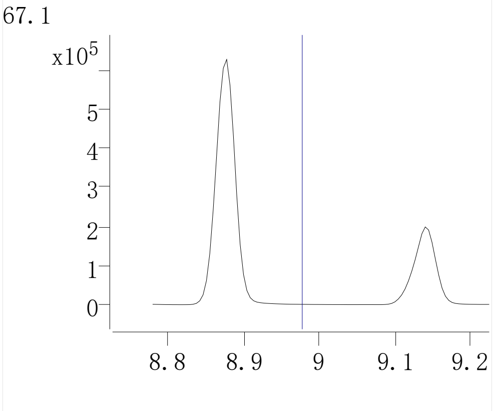

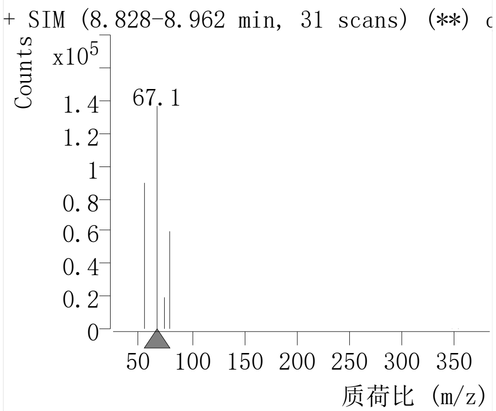

C18:3n6

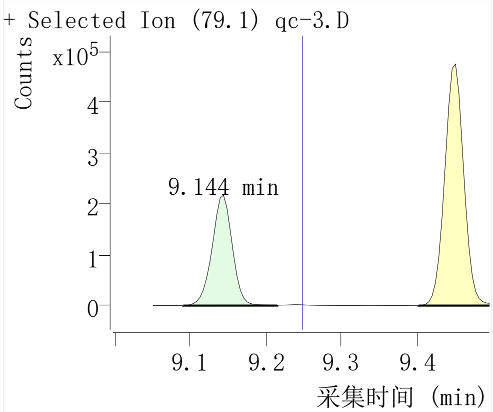

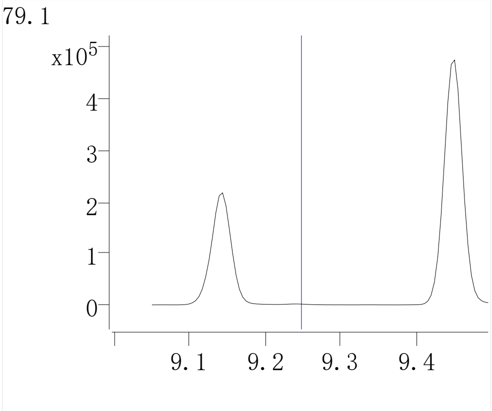

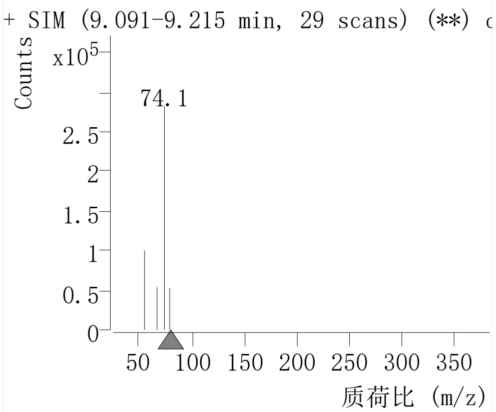

C18:3n3

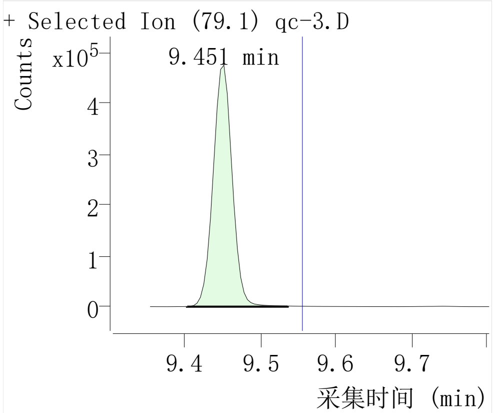

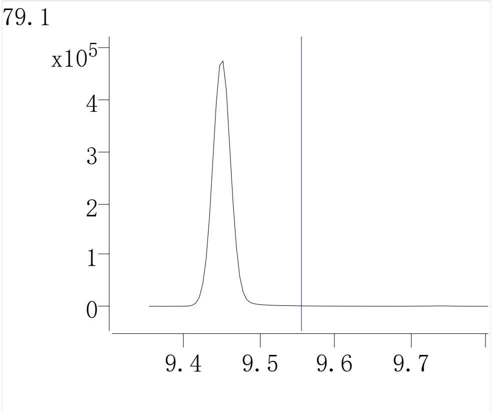

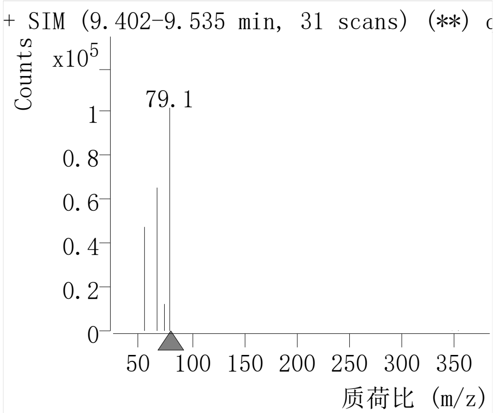

C20:0

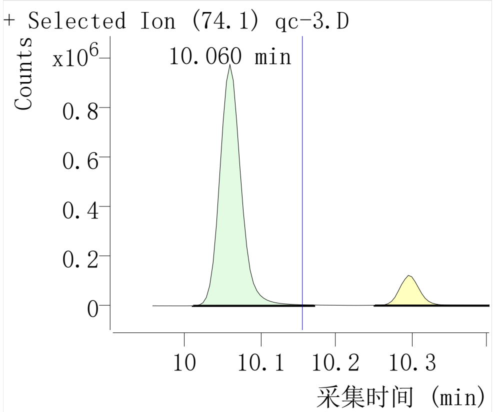

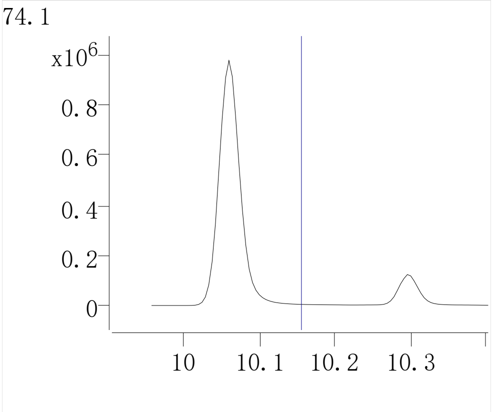

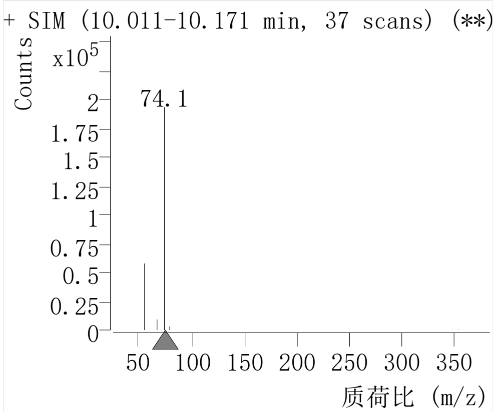

C20:1

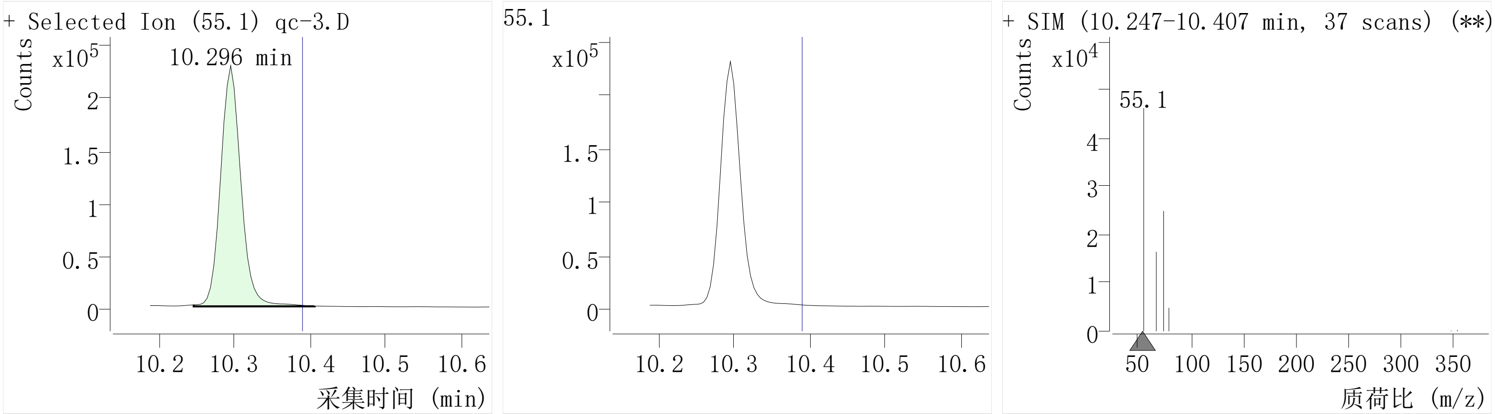

C20:2

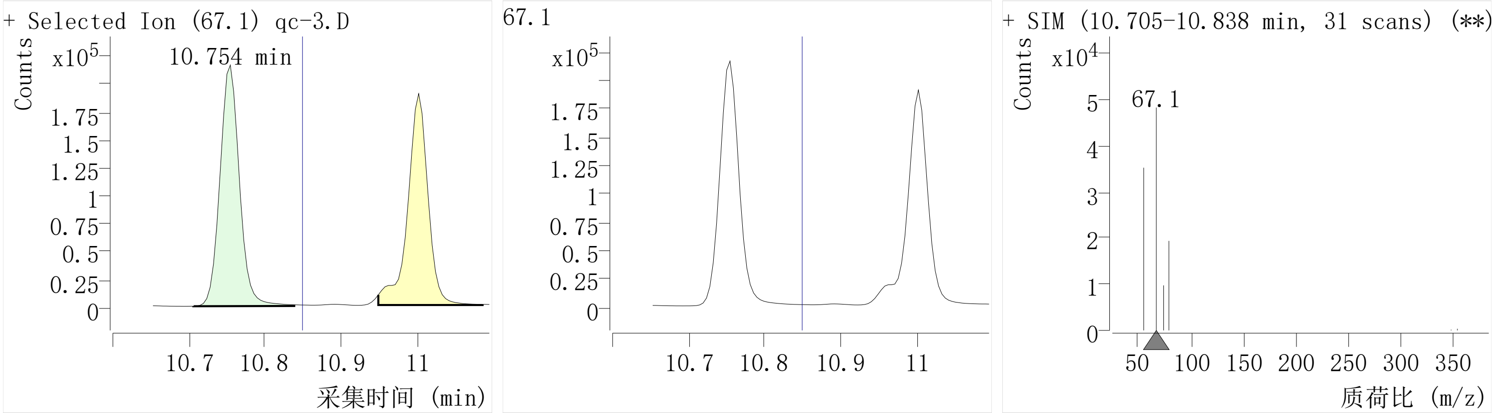

C21:0

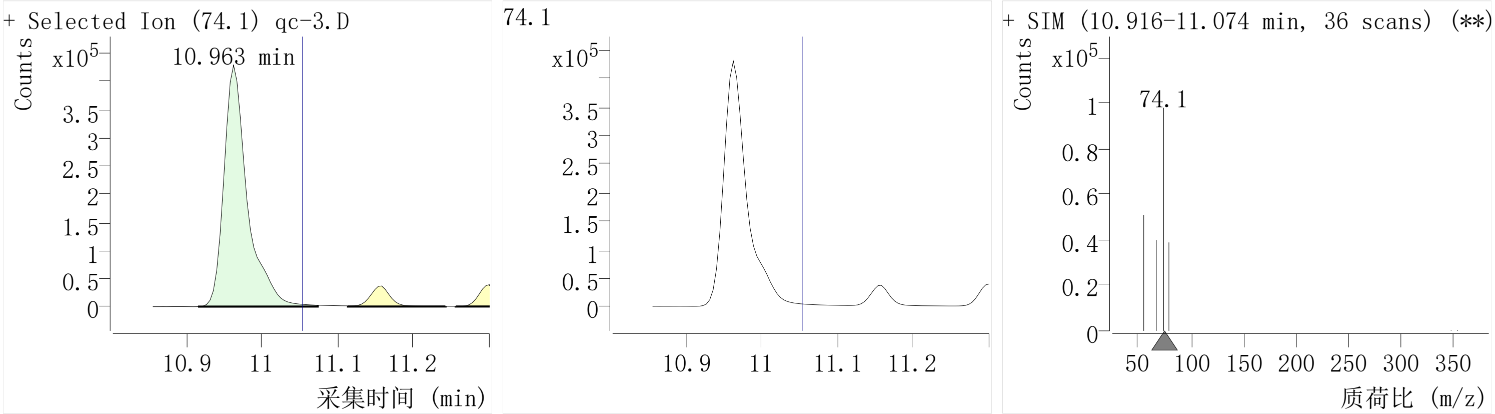

C20:3n6

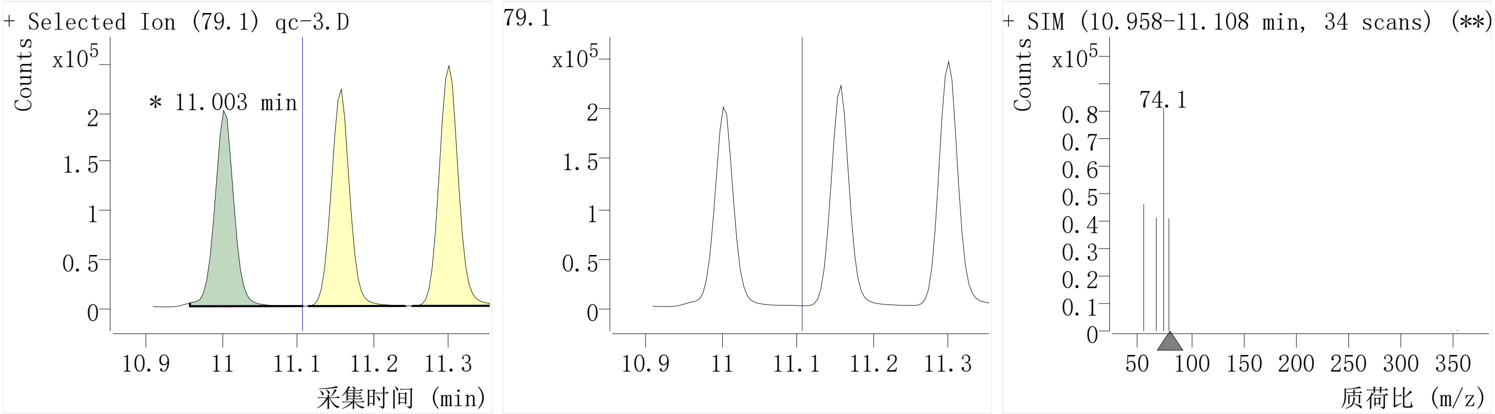

C20:4n6

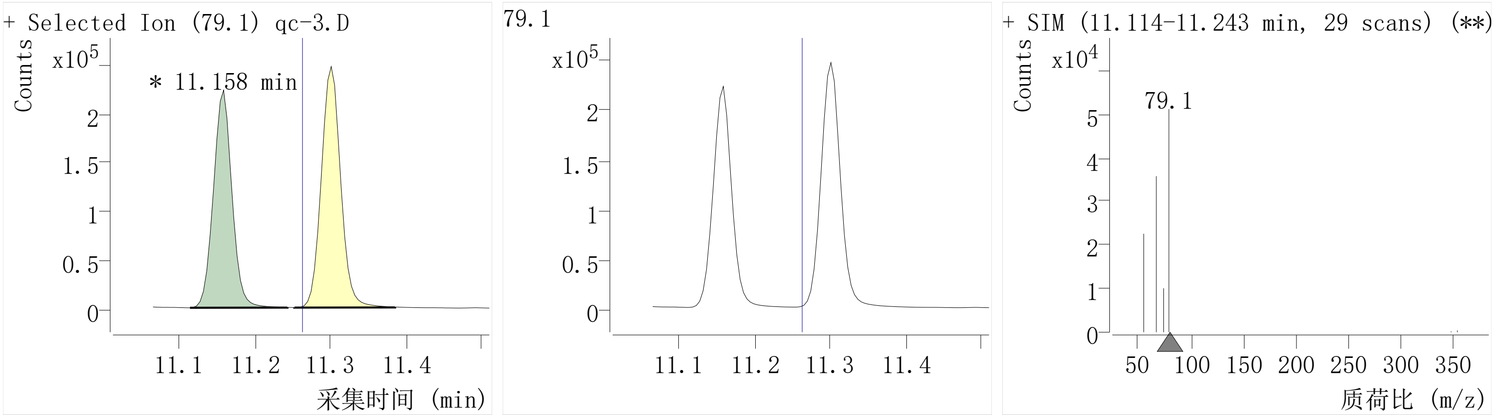

C20:3n3

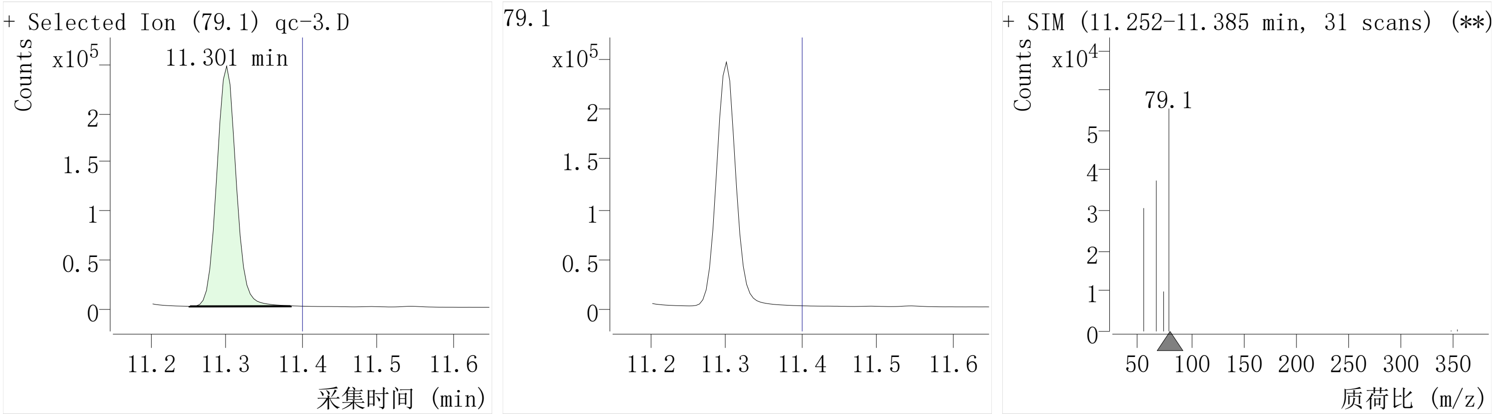

C20:5n3

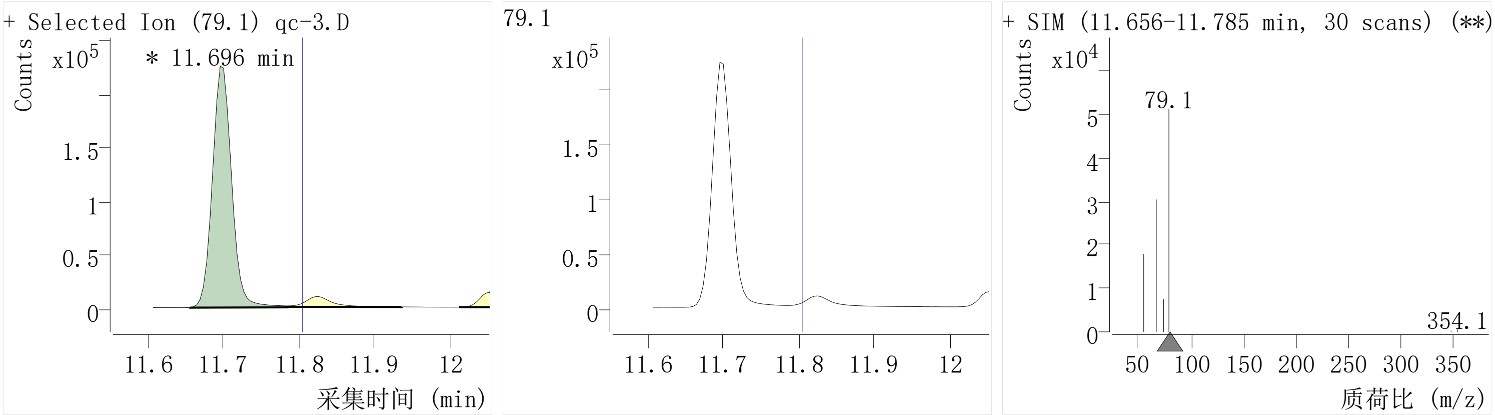

C22:0

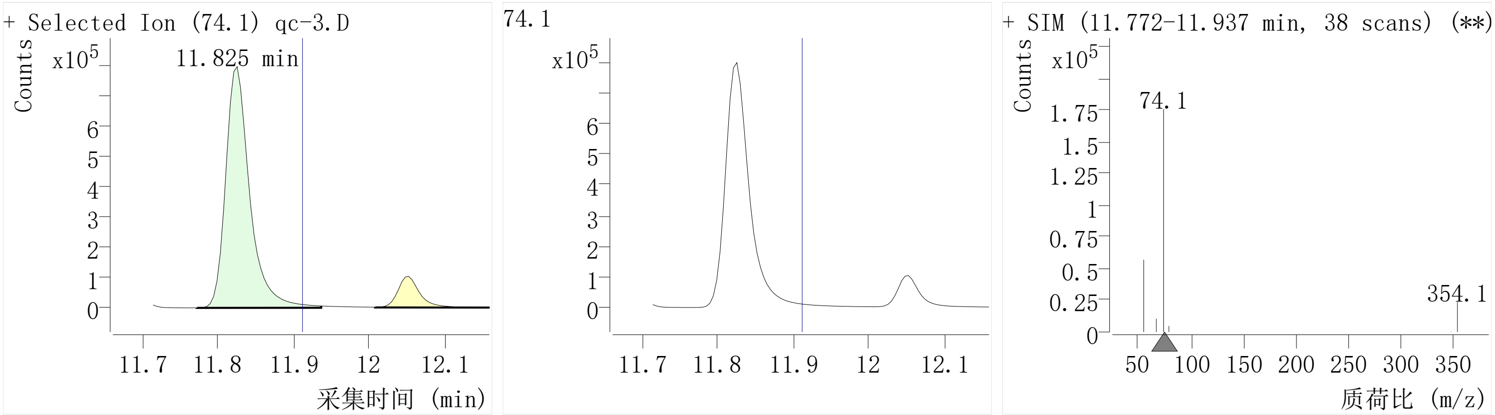

C22:1n9

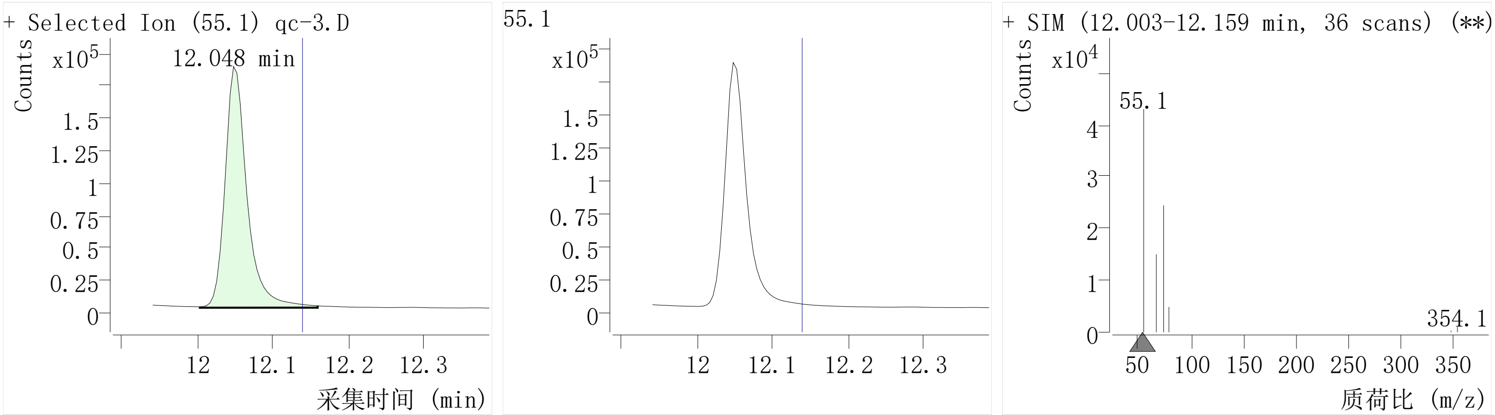

C22:2n6

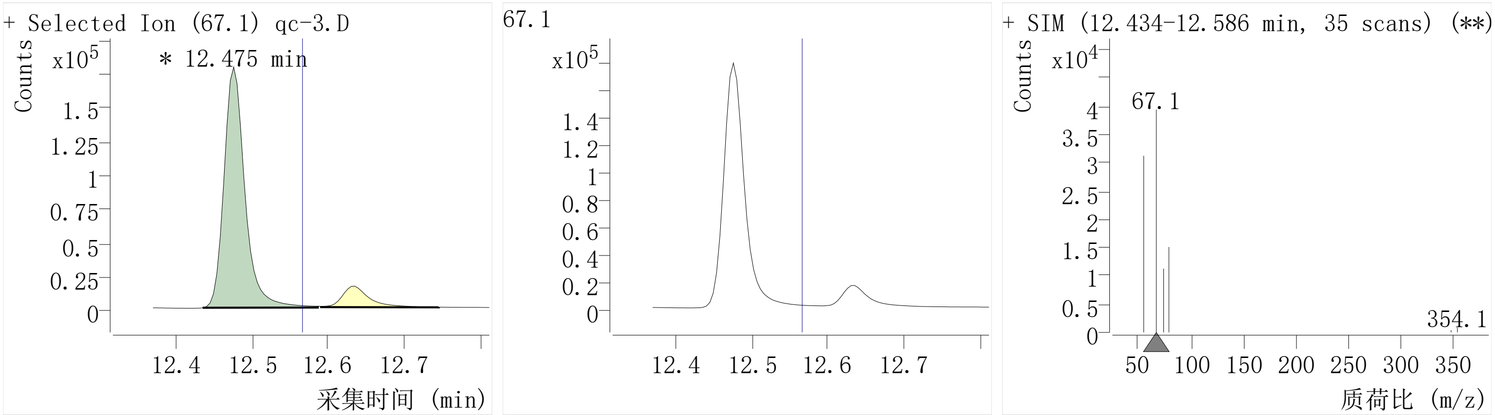

C23:0

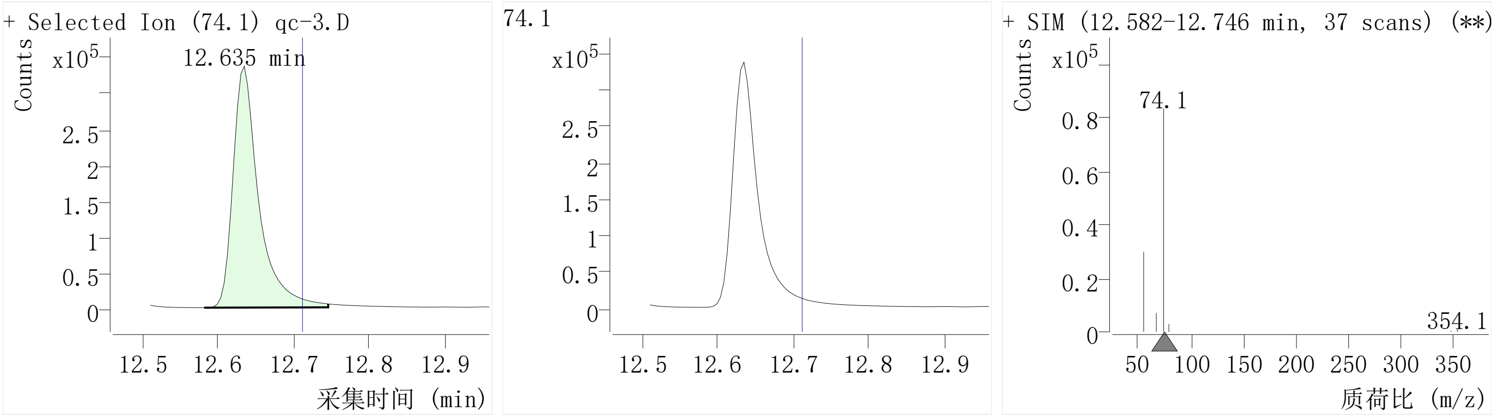

C24:0

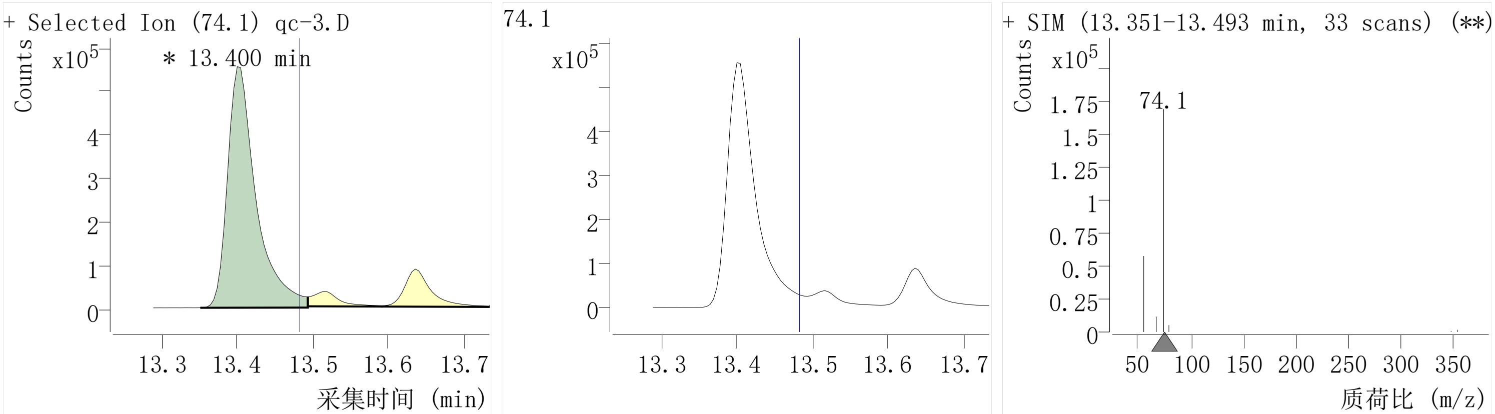

C22:6

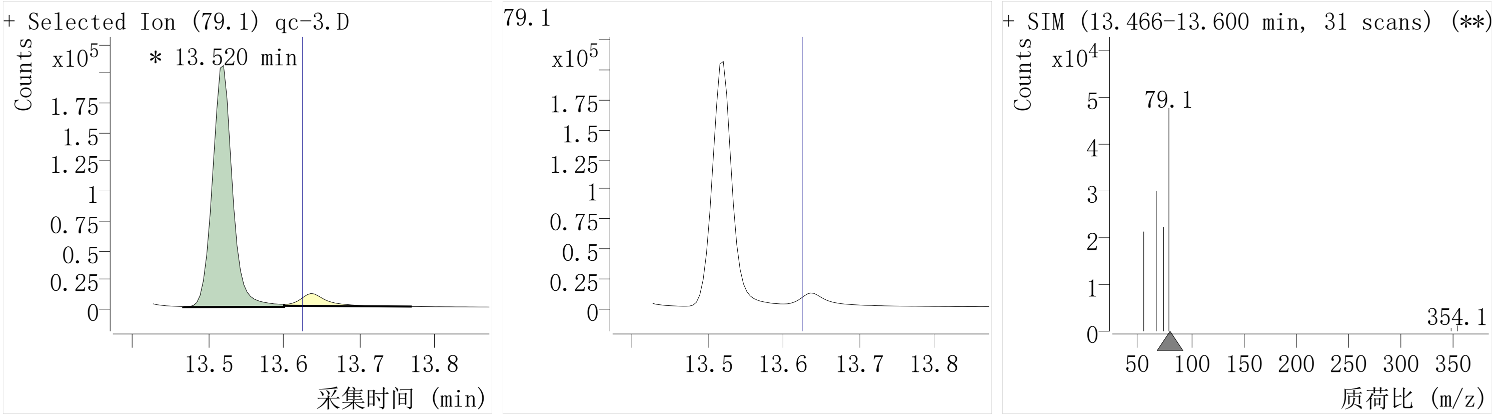
+ Selected Ion (79.1) qc-3.D
Counts
x10^5
* 13.520 min
1.75
1.5
1.25
1
0.75
0.5
0.25
0
13.5 13.6 13.7 13.8
采集时间 (min)
79.1
+ SIM (13.466-13.600 min, 31 scans) (**)
x10^4
79.1
354.1
50 100 150 200 250 300 350
质荷比 (m/z)

C24:1

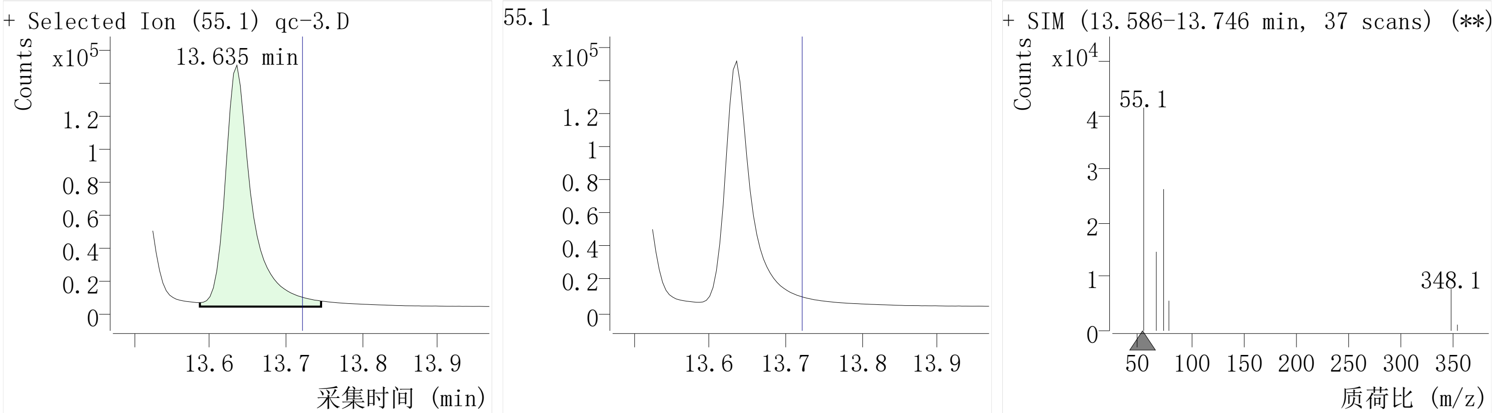
+ Selected Ion (55.1) qc-3.D
Counts
x10^5
13.635 min
1.2
1
0.8
0.6
0.4
0.2
0
13.6 13.7 13.8 13.9
采集时间 (min)
55.1
+ SIM (13.586-13.746 min, 37 scans) (**)
x10^4
55.1
348.1
50 100 150 200 250 300 350
质荷比 (m/z)

**批处理路径**	G:\GC-MS\HX250430-4-GCMS总脂肪酸靶向检测\HX250430-4\QuantResults\HX250430-4.batch.bin		
**分析时间**	2025/5/14 16:58	**分析员姓名**	DESKTOP-M3A0GPO\omics
**报告时间**	2025/5/16 14:53:14	**报告员姓名**	DESKTOP-M3A0GPO\omics
**最近校正更新**	2025/5/14 16:58	**批处理状态**	已处理
**定量批处理版本**	10.2	**定量报告版本**	10.2

**采集时间**	2025/5/9 3:55	**数据文件**	sp-17.D
**样品类型**	样品	**样品名称**	sp-17
**稀释**	1	**采集方法**	脂肪酸

样品色谱图

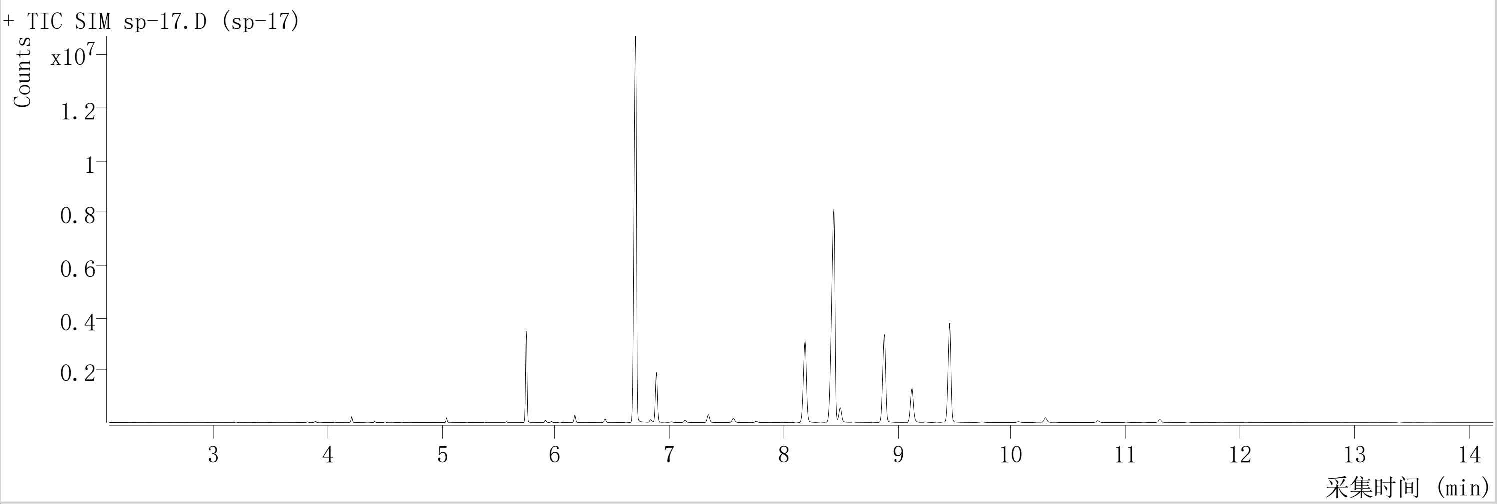

化合物	ISTD	RT	响应	ISTD 响应	响应比	最终浓度	单位
C4:0	C19:0	2.203	0	1822377	0.0000	ND	ug/ml
C6:0	C19:0	2.959	892	1822377	0.0005	0.0057	ug/ml
C8:0	C19:0	3.724	2815	1822377	0.0015	0.0113	ug/ml
C10:0	C19:0	4.413	26215	1822377	0.0144	0.2162	ug/ml
C11:0	C19:0	4.729	1887	1822377	0.0010	0.0092	ug/ml
C12:0	C19:0	5.044	99907	1822377	0.0548	0.8539	ug/ml
C13:0	C19:0	5.378	8117	1822377	0.0045	0.0506	ug/ml
C14:0	C19:0	5.742	2490228	1822377	1.3665	30.2759	ug/ml
C14:1	C19:0	5.911	41131	1822377	0.0226	1.0316	ug/ml
C15:0	C19:0	6.169	216699	1822377	0.1189	2.1207	ug/ml
C15:1	C19:0	6.432	0	1822377	0.0000	ND	ug/ml
C16:0	C19:0	6.699	15121477	1822377	8.2977	334.6394	ug/ml
C16:1	C19:0	6.885	1124711	1822377	0.6172	37.6274	ug/ml
C17:0	C19:0	7.339	350945	1822377	0.1926	4.0177	ug/ml
C17:1	C19:0	7.557	121871	1822377	0.0669	3.5729	ug/ml
C18:0	C19:0	8.188	4489811	1822377	2.4637	55.9117	ug/ml
C18:1n9t	C19:0	8.326	9818	1822377	0.0054	0.3178	ug/ml
C18:1n9c	C19:0	8.441	7757052	1822377	4.2566	288.7664	ug/ml
C18:2n6t	C19:0	8.882	0	1822377	0.0000	ND	ug/ml
C18:2n6c	C19:0	8.882	2790338	1822377	1.5312	121.4369	ug/ml
C18:3n6	C19:0	9.131	26802	1822377	0.0147	0.1774	ug/ml
C18:3n3	C19:0	9.455	3071567	1822377	1.6855	83.7927	ug/ml
C20:0	C19:0	10.060	50887	1822377	0.0279	0.7383	ug/ml
C20:1	C19:0	10.296	173478	1822377	0.0952	5.9062	ug/ml
C20:2	C19:0	10.754	55325	1822377	0.0304	1.9100	ug/ml
C21:0	C19:0	10.963	2152	1822377	0.0012	0.0327	ug/ml
C20:3n6	C19:0	11.003	8357	1822377	0.0046	0.3338	ug/ml
C20:4n6	C19:0	11.158	5289	1822377	0.0029	0.2162	ug/ml
C20:3n3	C19:0	11.301	87414	1822377	0.0480	2.5588	ug/ml
C20:5n3	C19:0	11.696	1805	1822377	0.0010	0.0900	ug/ml

化合物	ISTD	RT	响应	ISTD 响应	响应比	最终浓度	单位
C22:0	C19:0	11.821	5073	1822377	0.0028	0.1104	ug/ml
C22:1n9	C19:0	12.048	4192	1822377	0.0023	0.1555	ug/ml
C22:2n6	C19:0	12.470	820	1822377	0.0004	0.0725	ug/ml
C23:0	C19:0	12.755	0	1822377	0.0000	ND	ug/ml
C24:0	C19:0	13.399	3030	1822377	0.0017	0.0813	ug/ml
C22:6	C19:0	13.511	1336	1822377	0.0007	0.0594	ug/ml
C24:1	C19:0	13.675	0	1822377	0.0000	ND	ug/ml

C4:0

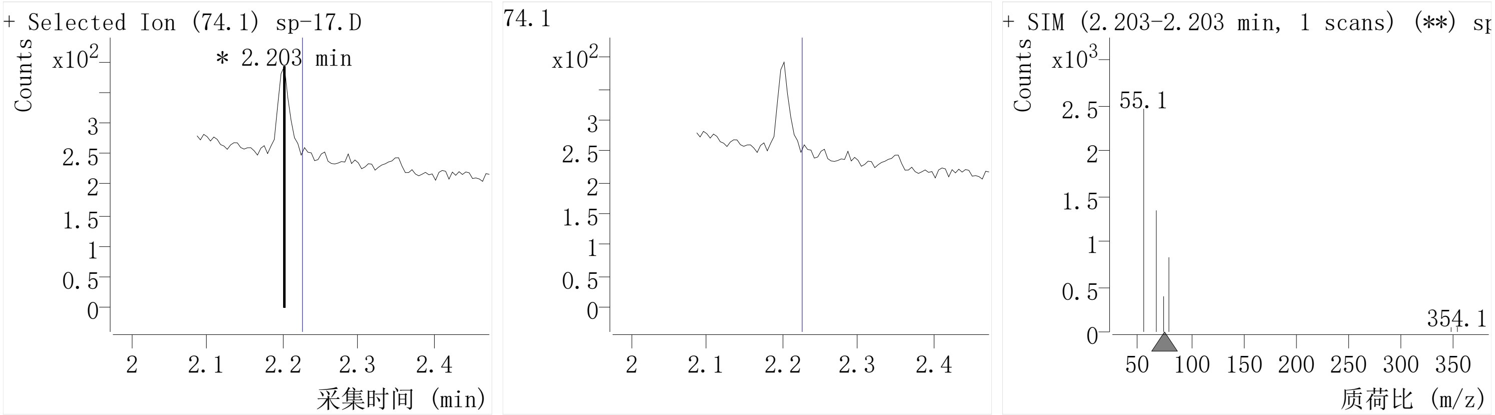

C6:0

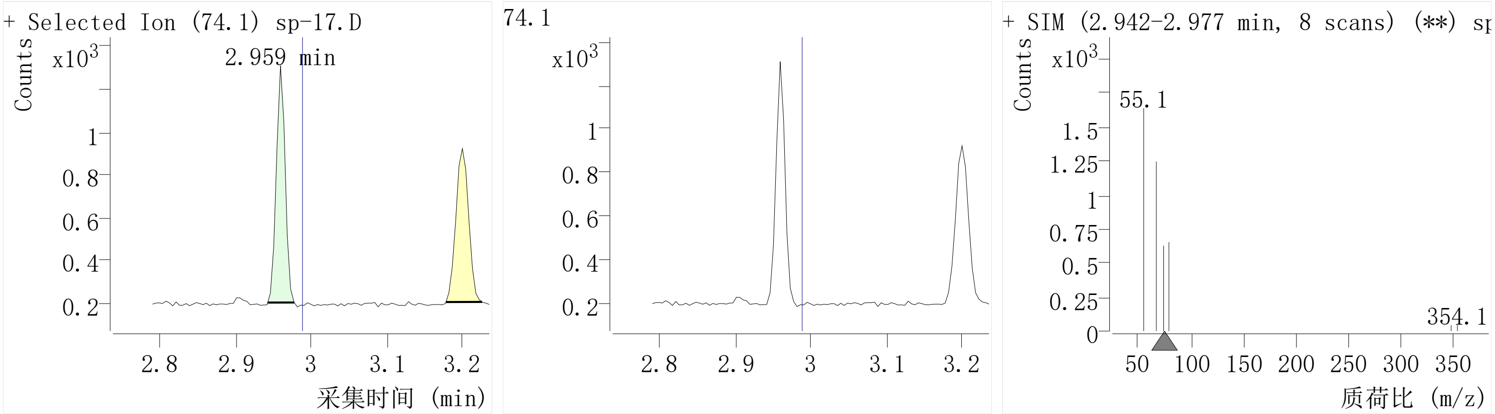

C8:0

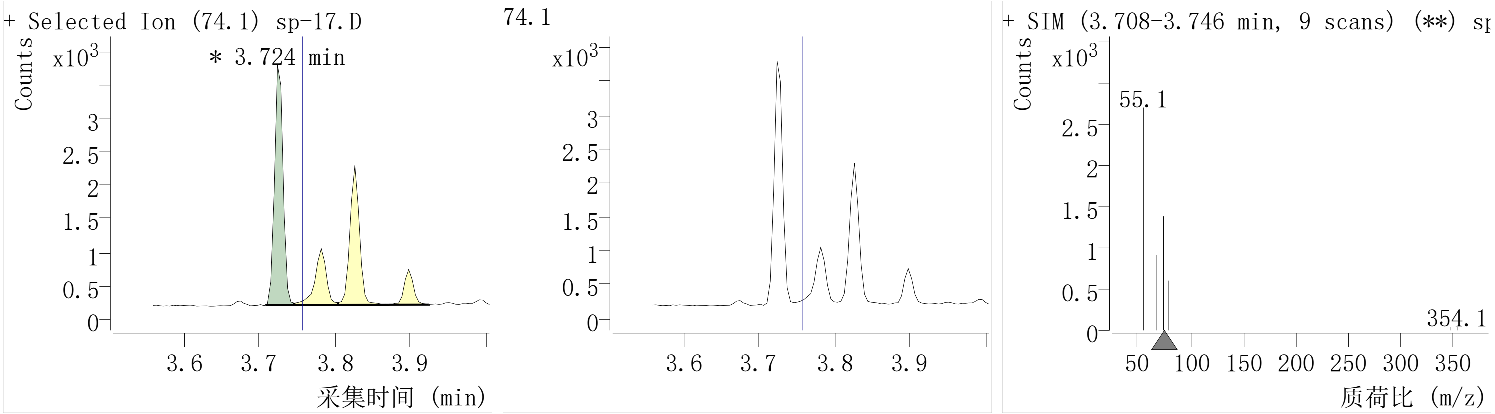

C10:0

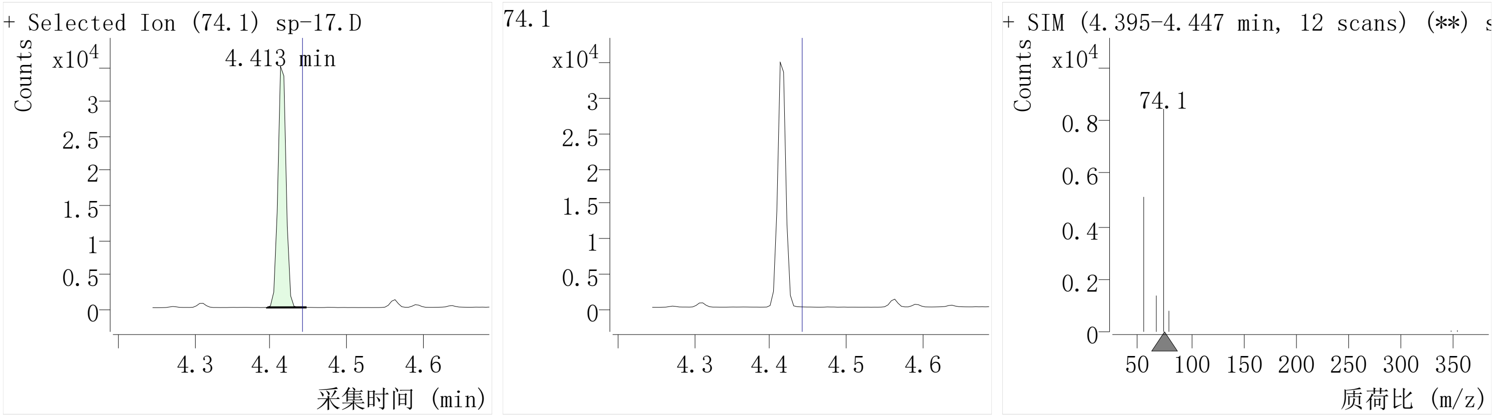

C11:0

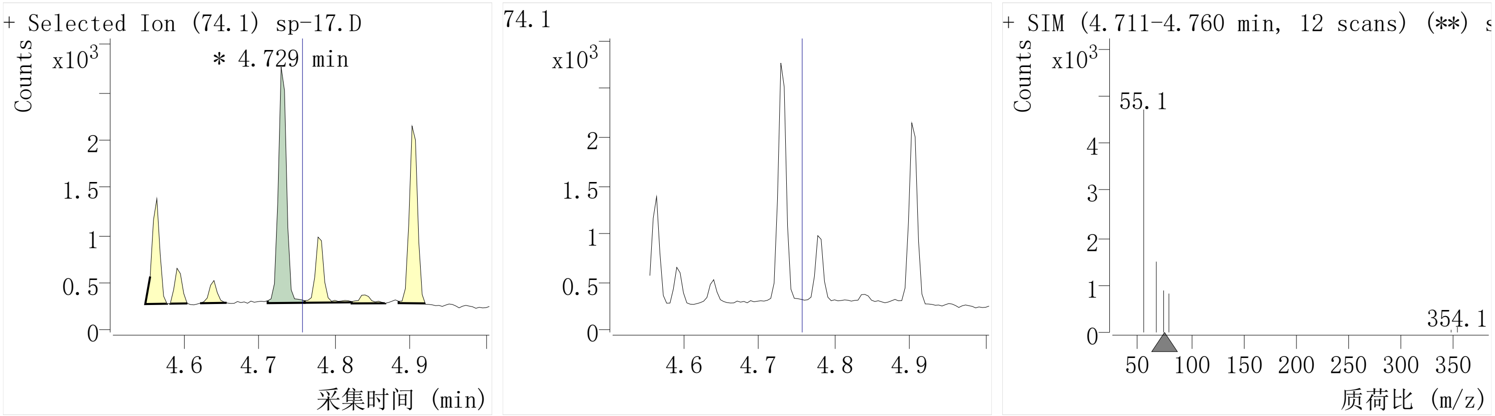

C12:0

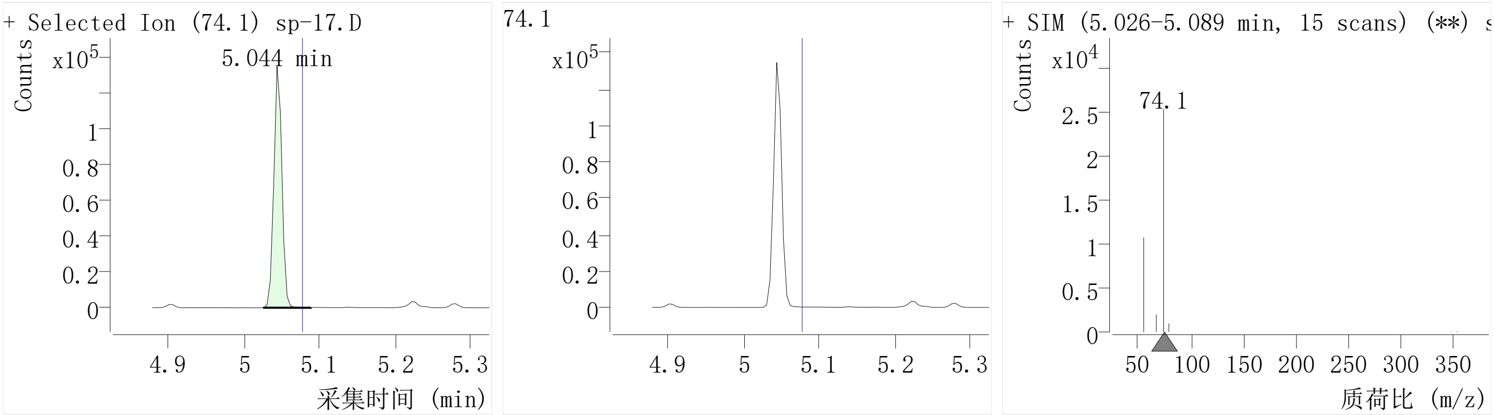

C13:0

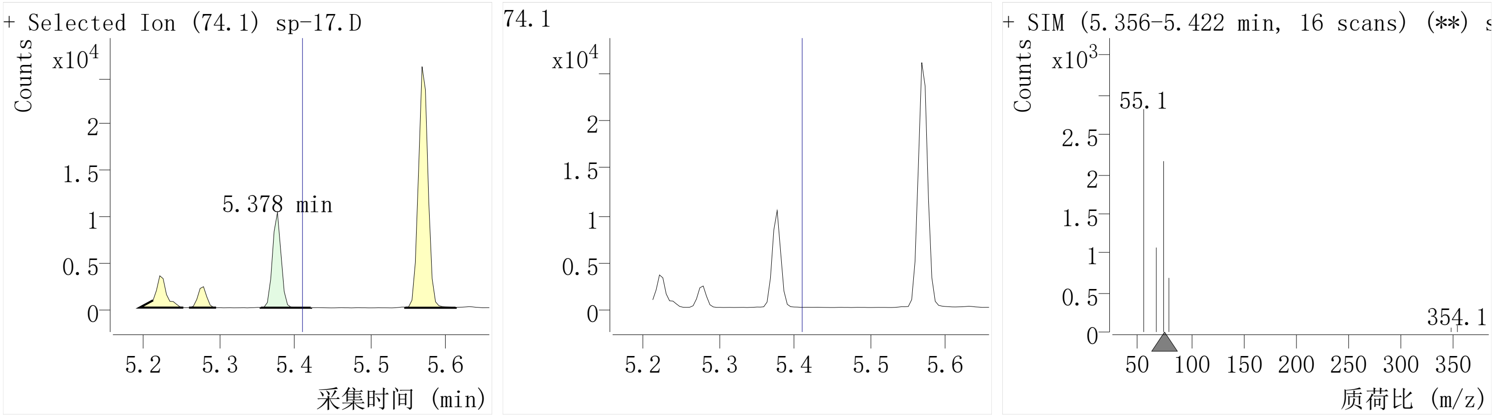

C14:0

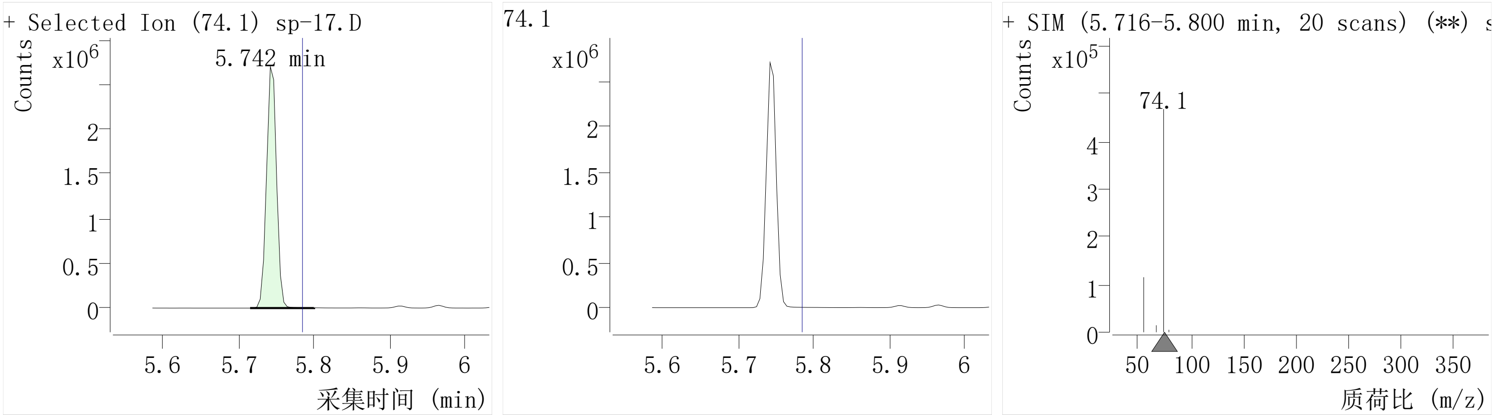

C14:1

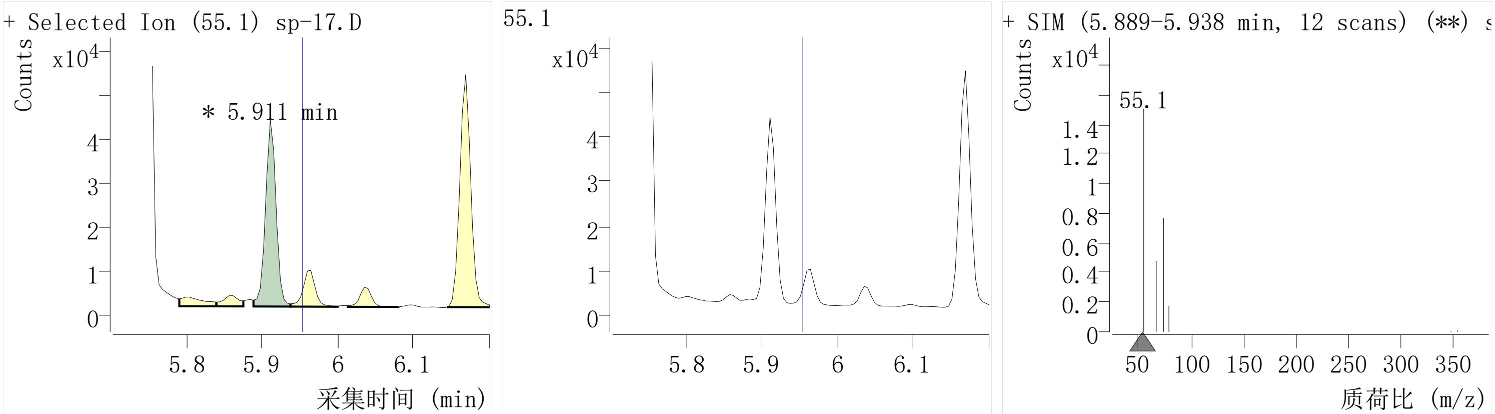

C15:0

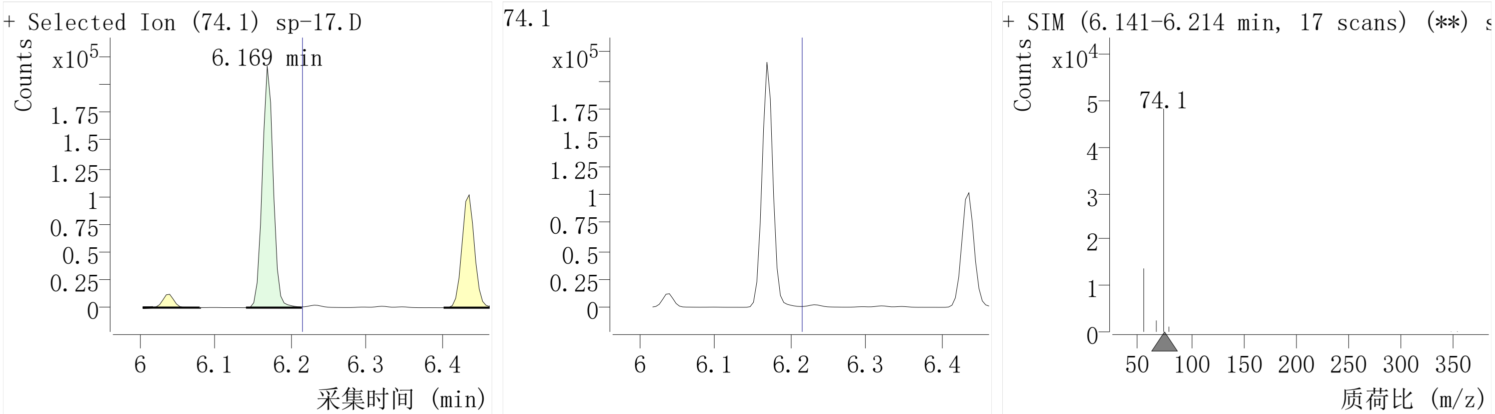

C15:1

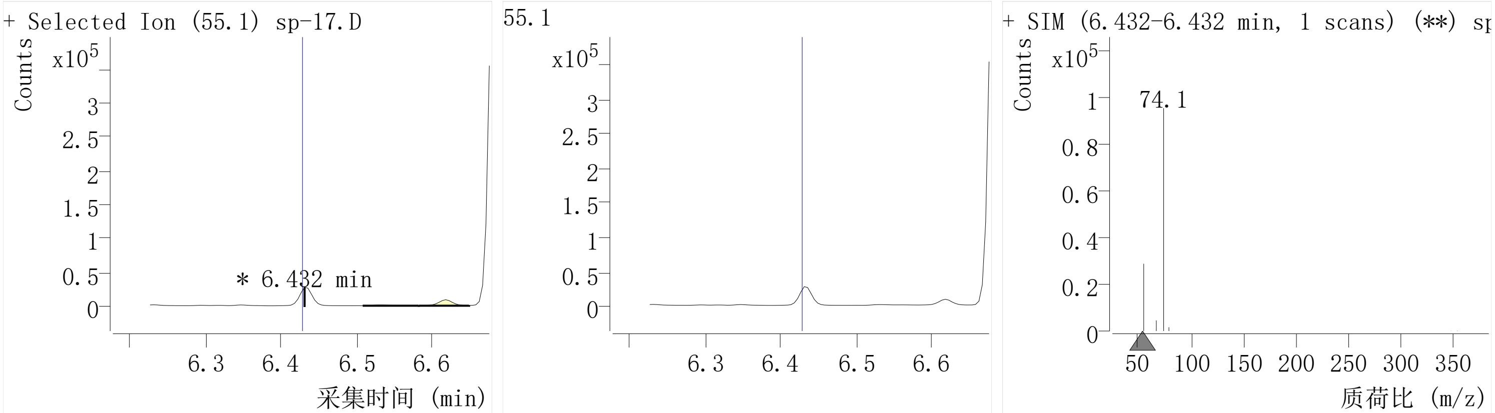

C16:0

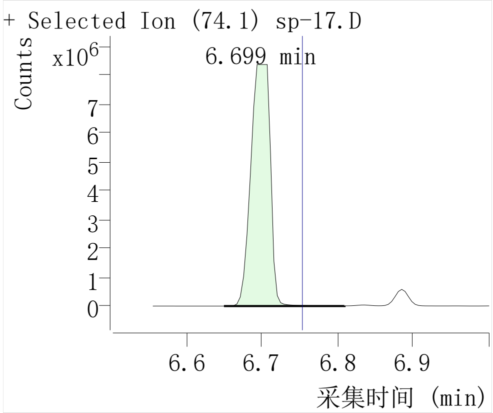

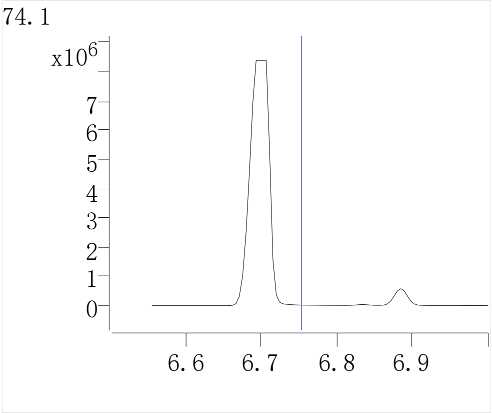

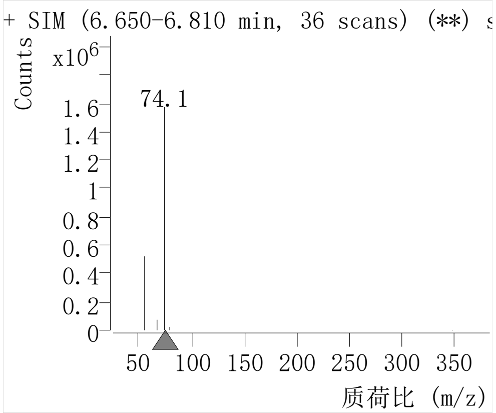

C16:1

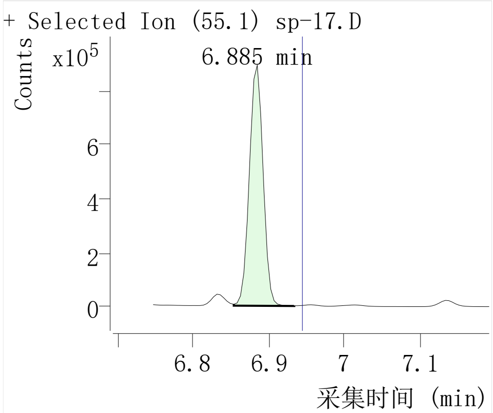

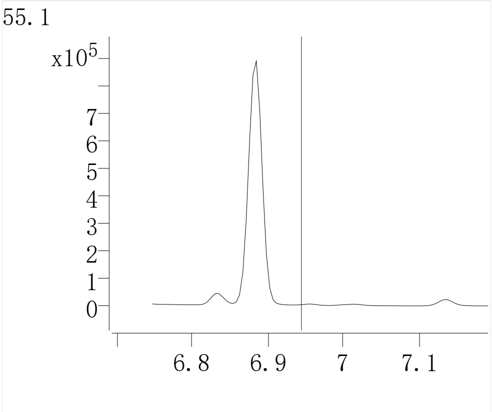

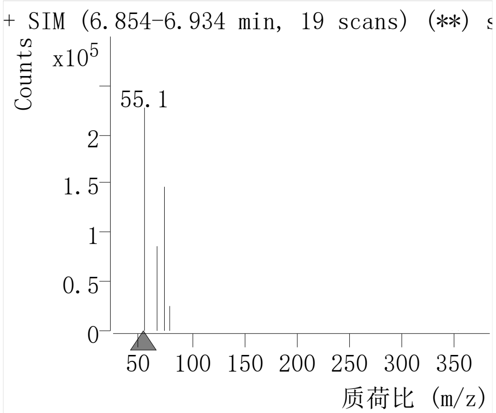

C17:0

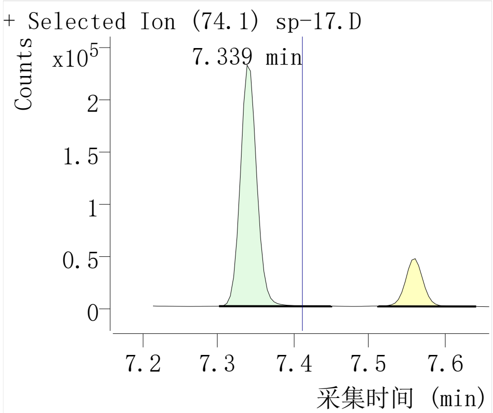

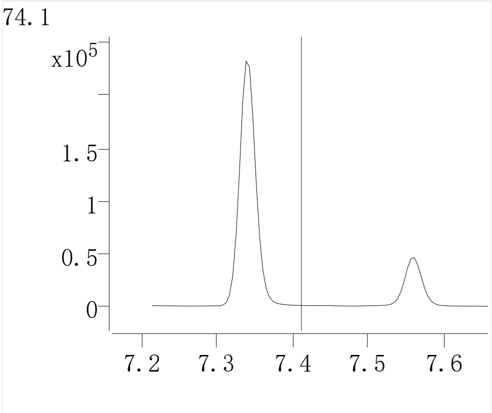

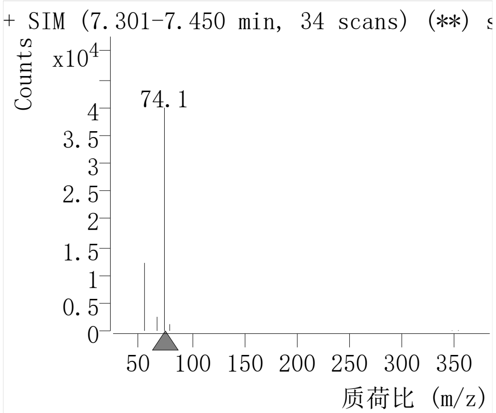

C17:1

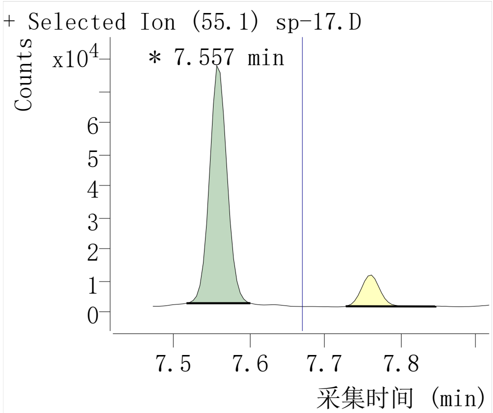

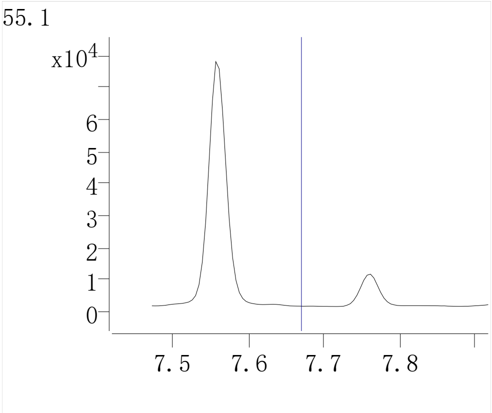

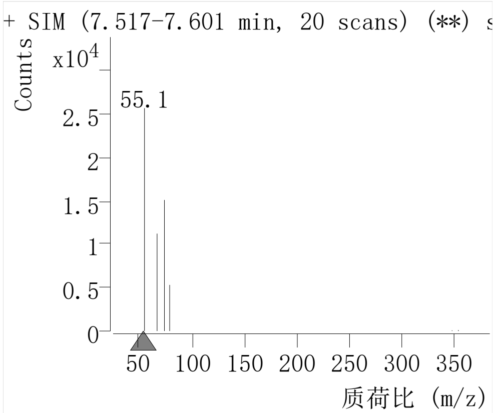

C18:0

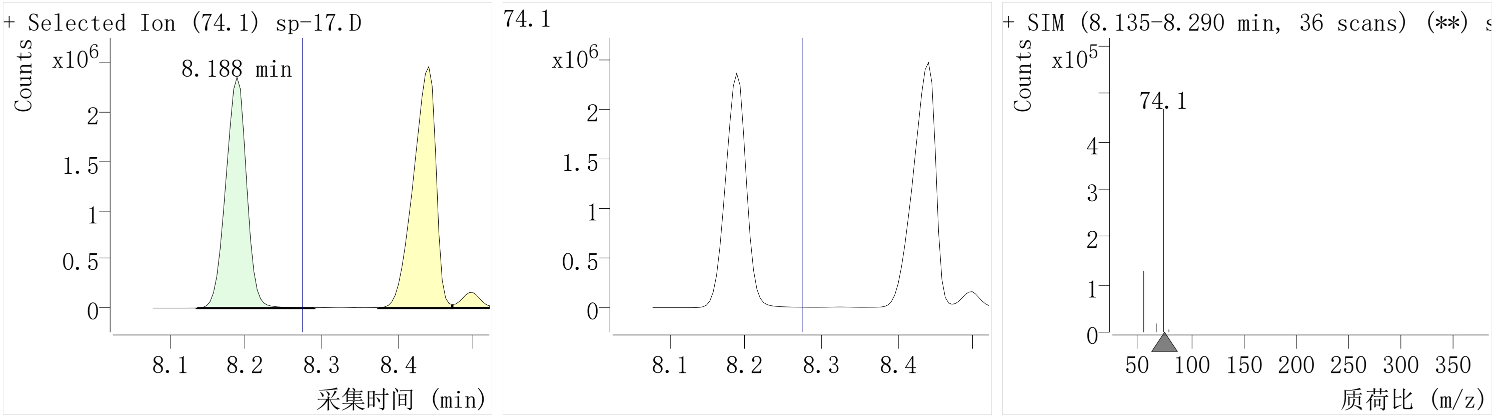

C18:1n9t

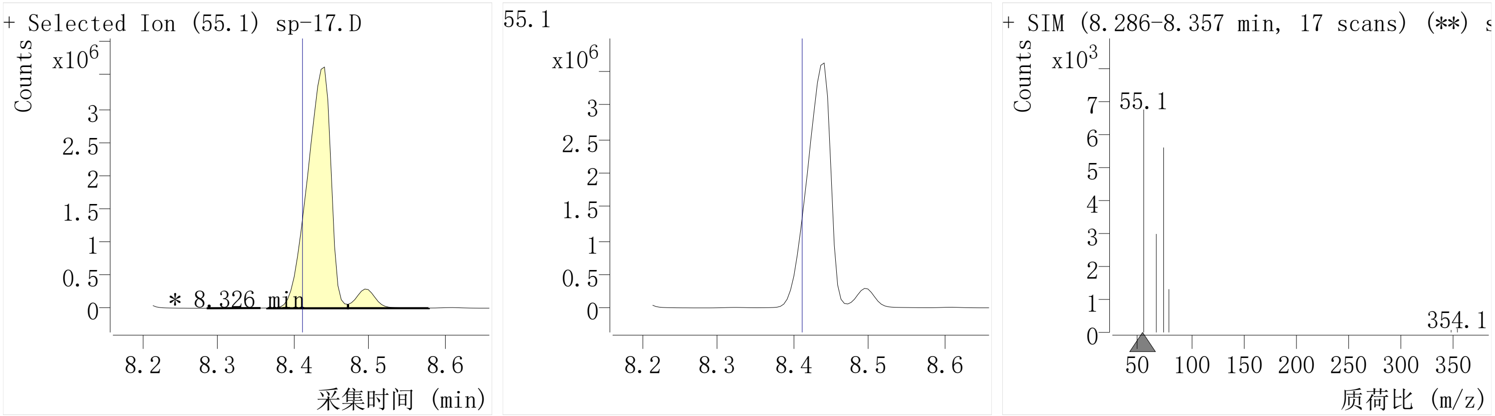

C18:1n9c

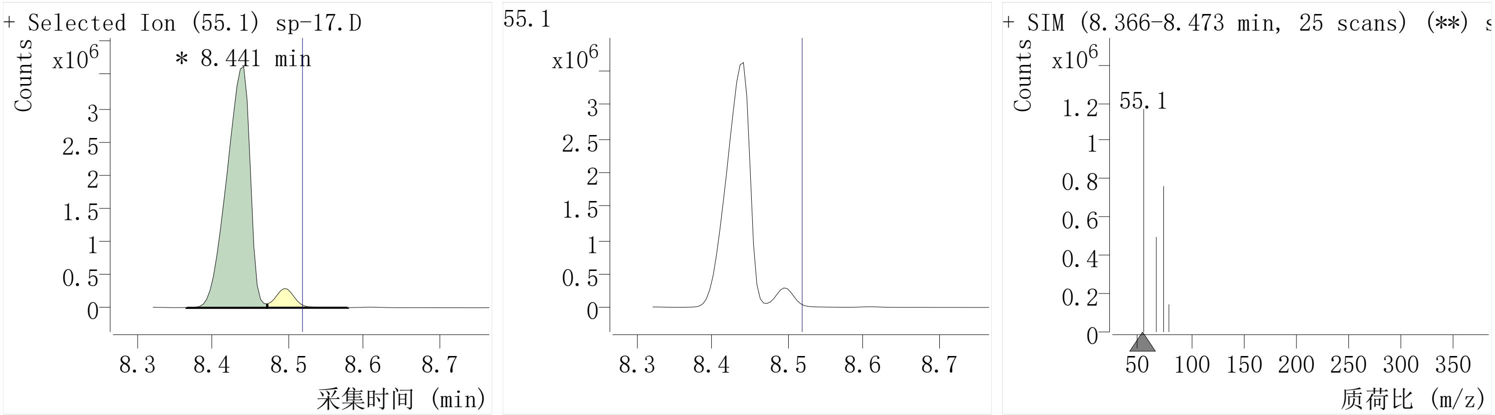

C18:2n6t

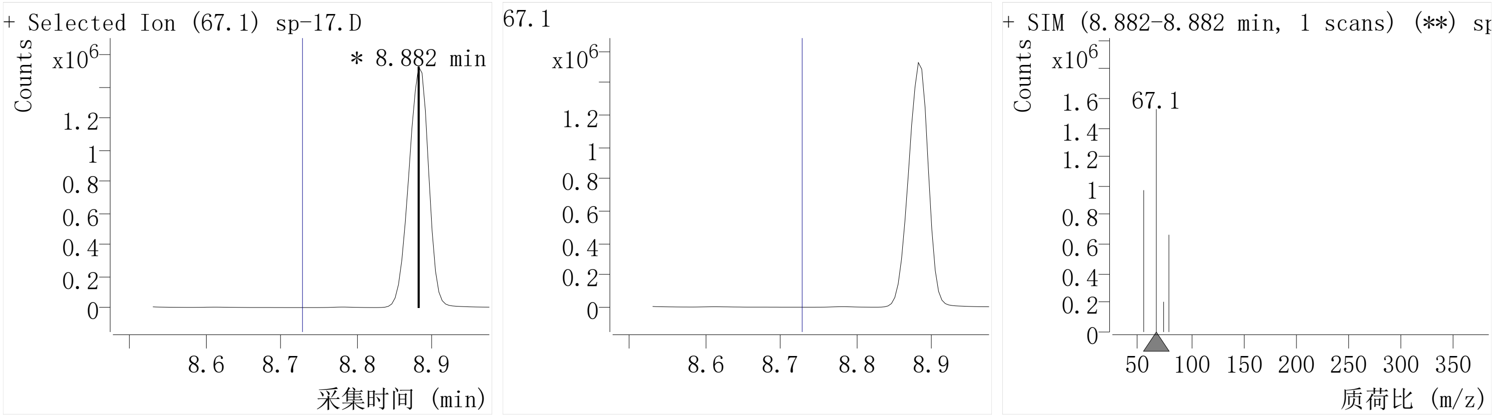

C18:2n6c

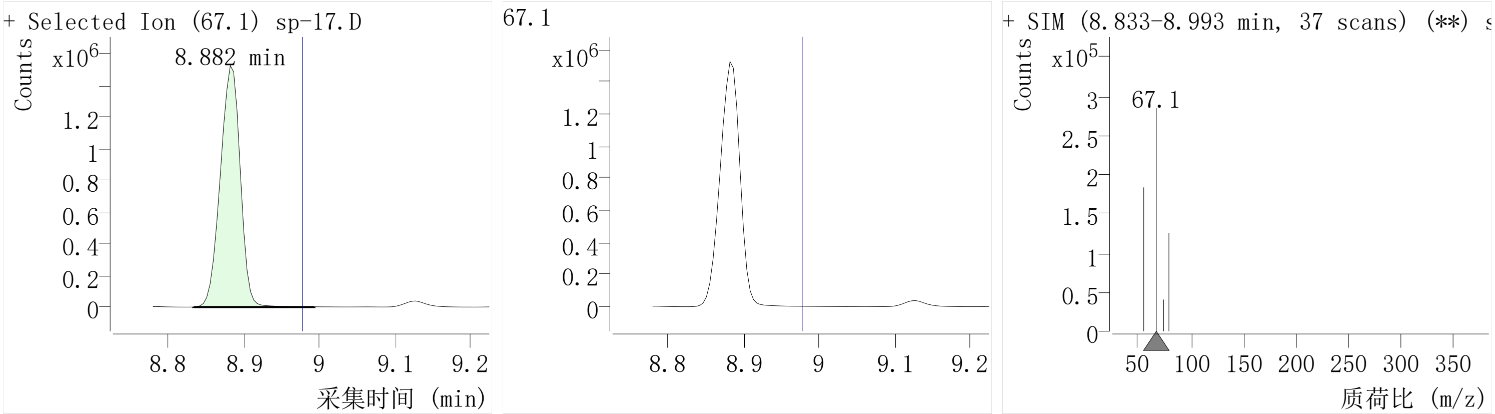

C18:3n6

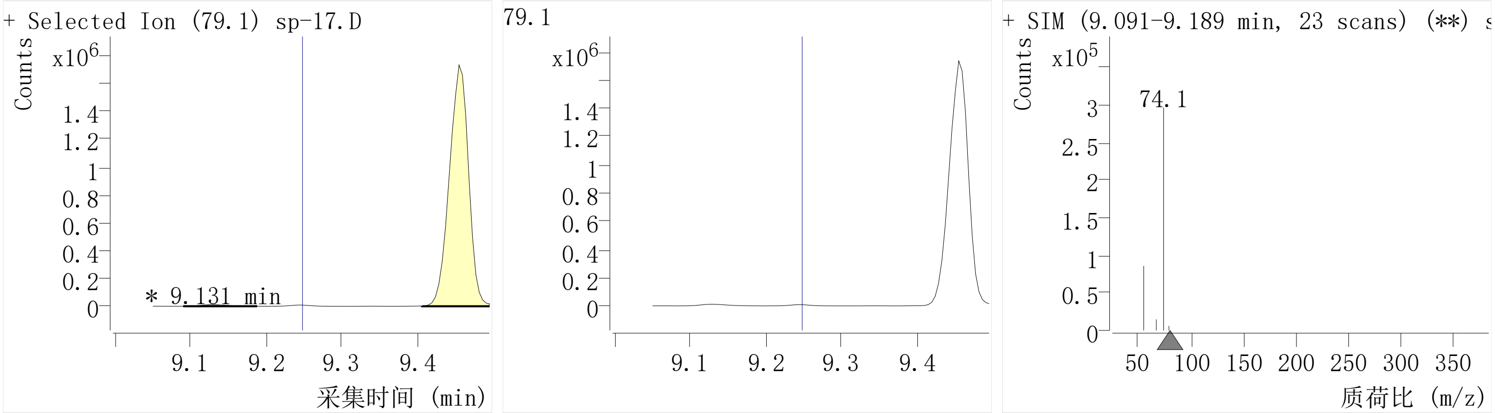

C18:3n3

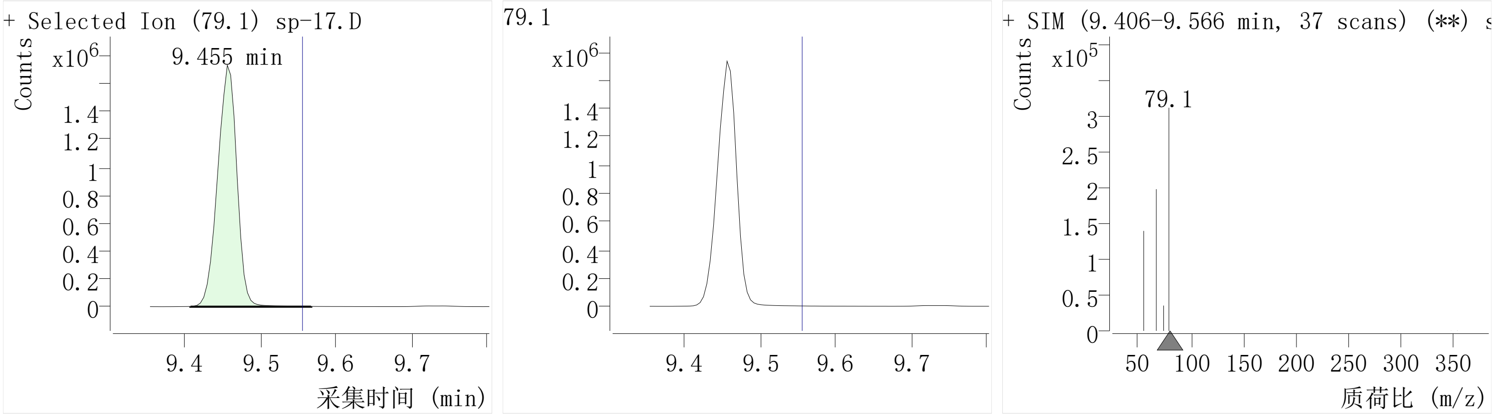

C20:0

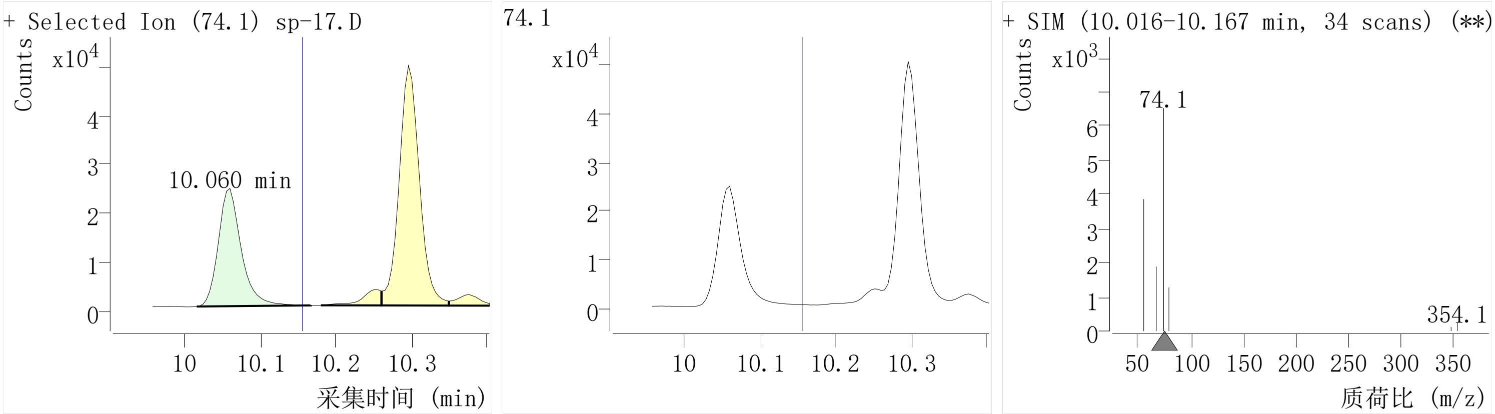

C20:1

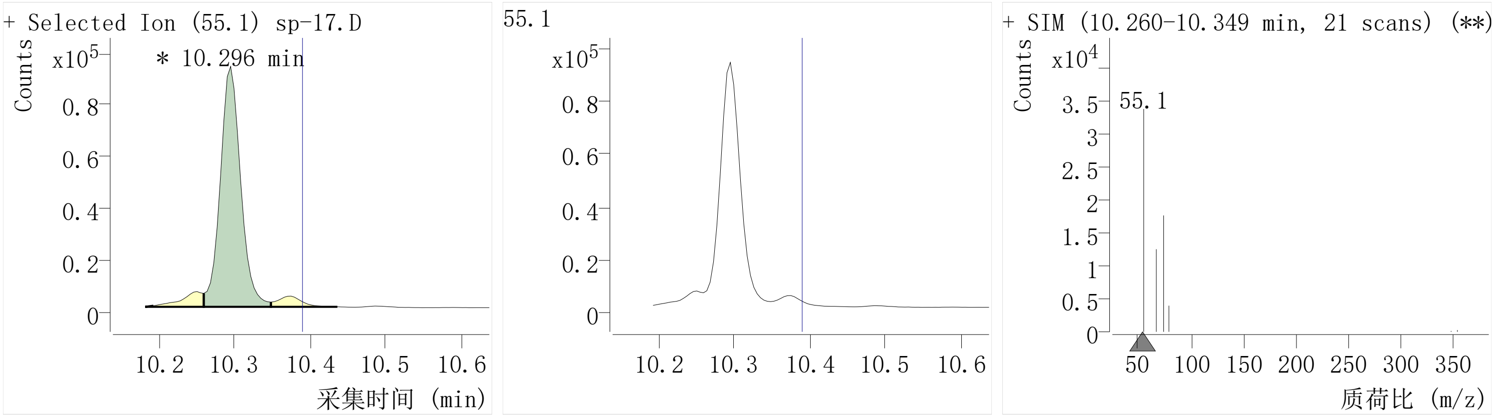

C20:2

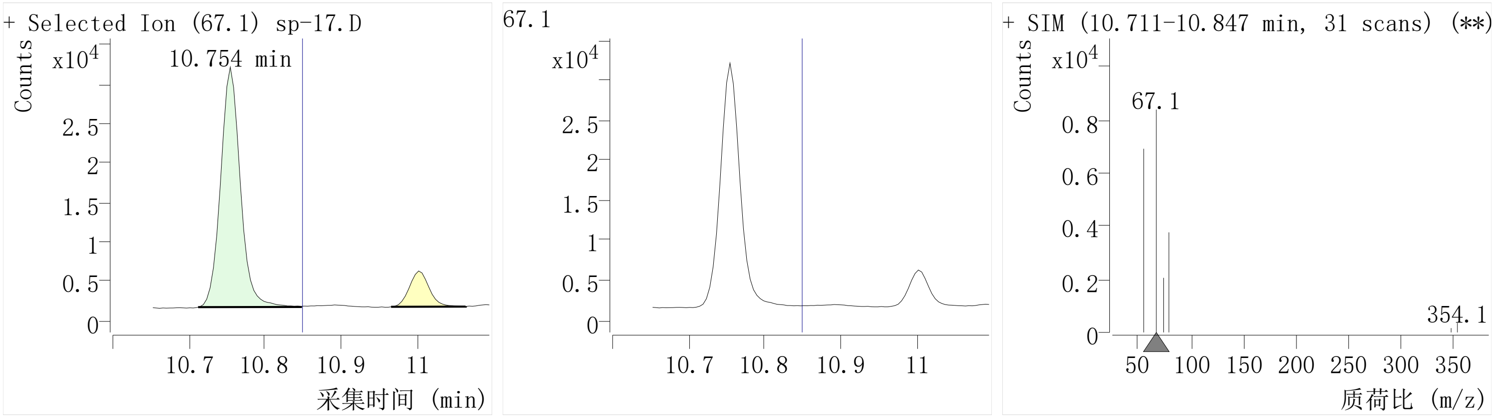

C21:0

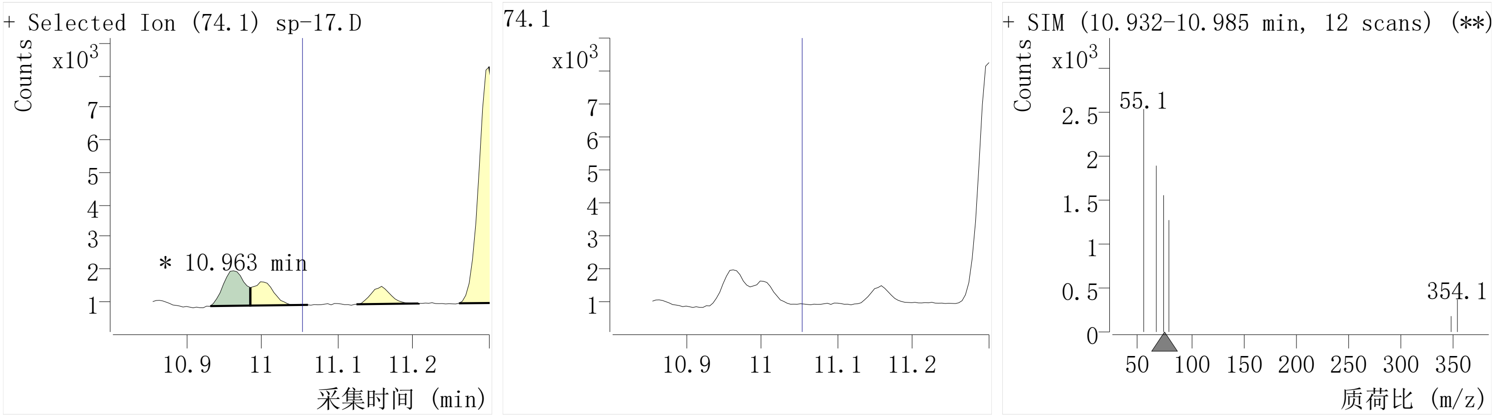

C20:3n6

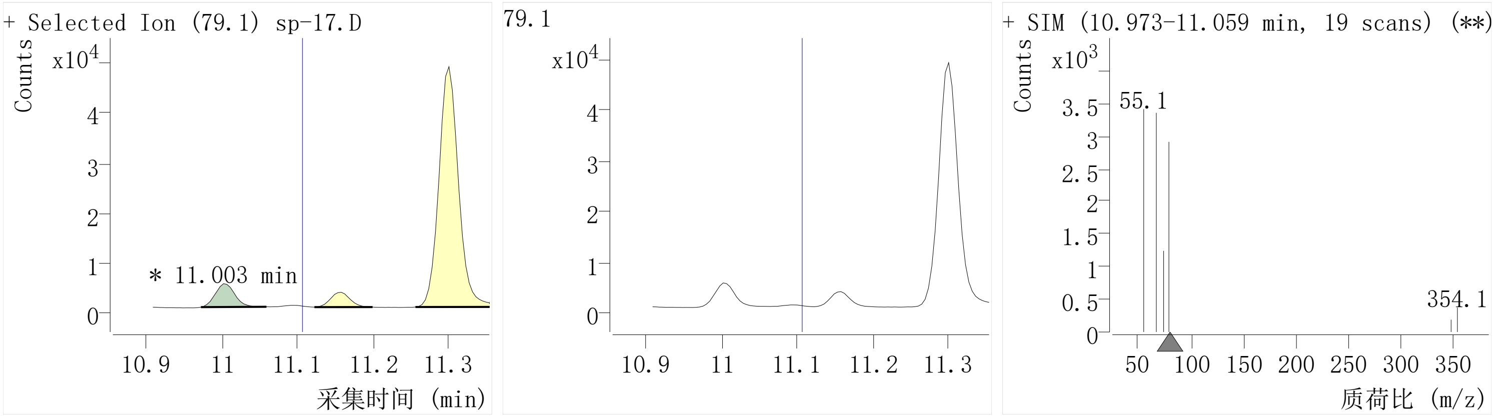

C20:4n6

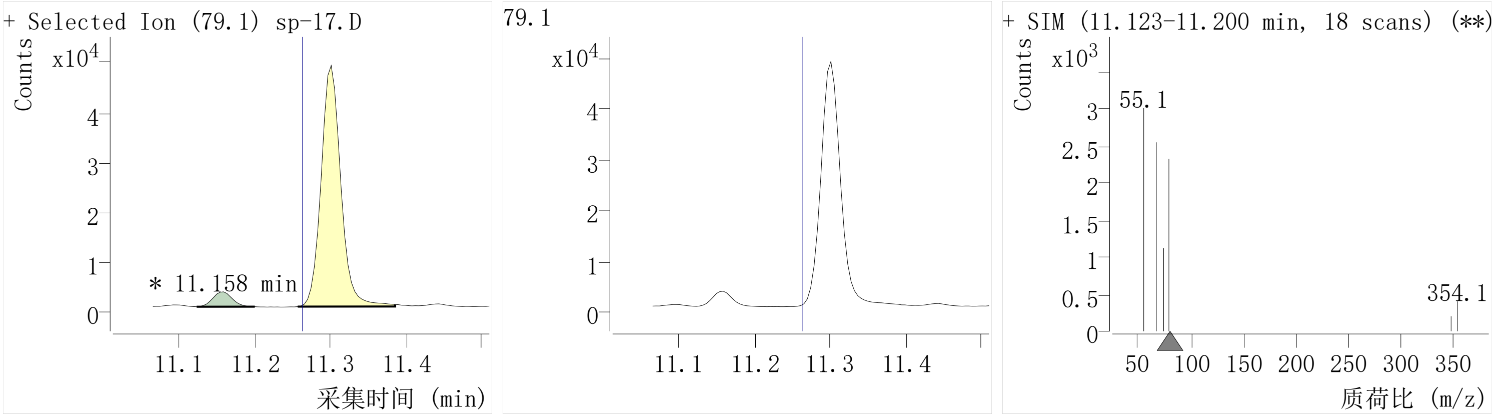

C20:3n3

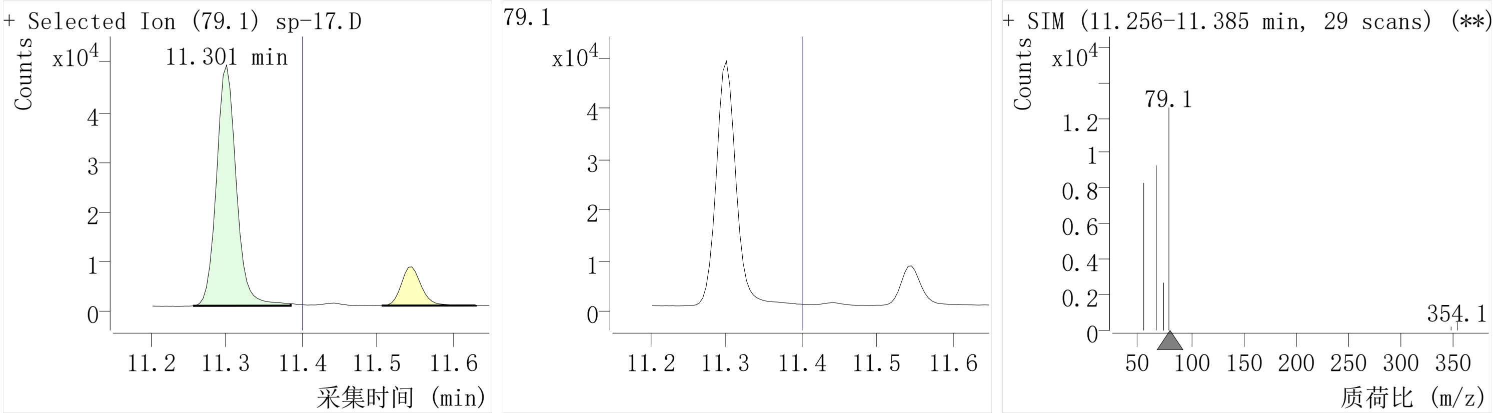

C20:5n3

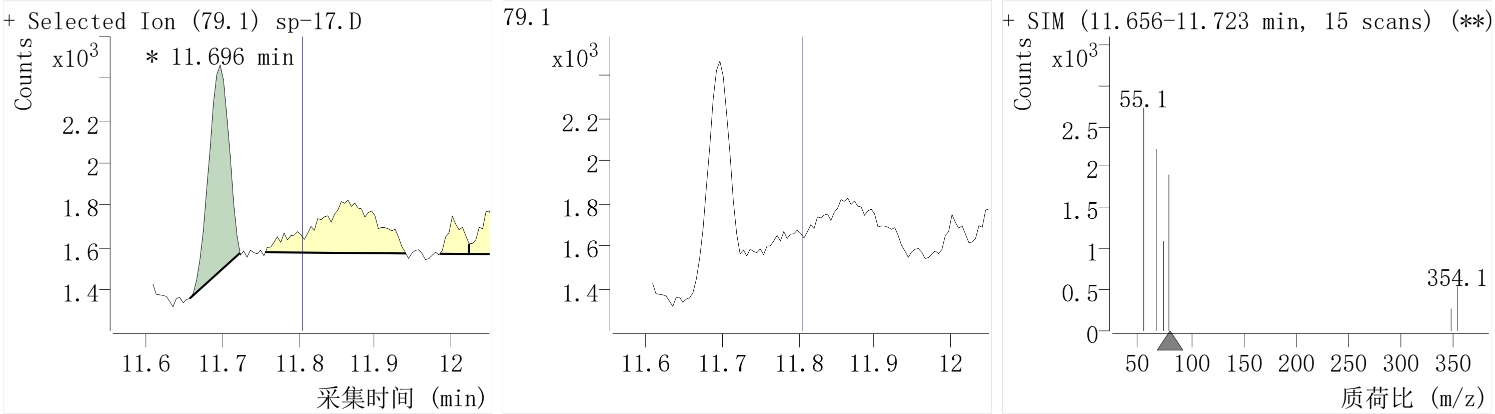

C22:0

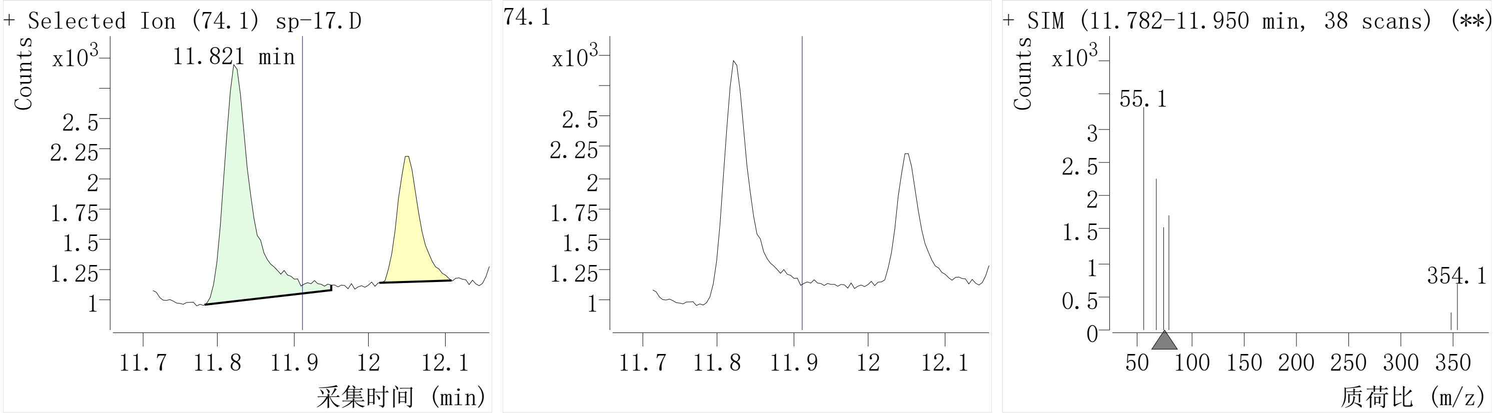

 生成时间 2025/5/16 14:52

C22:1n9

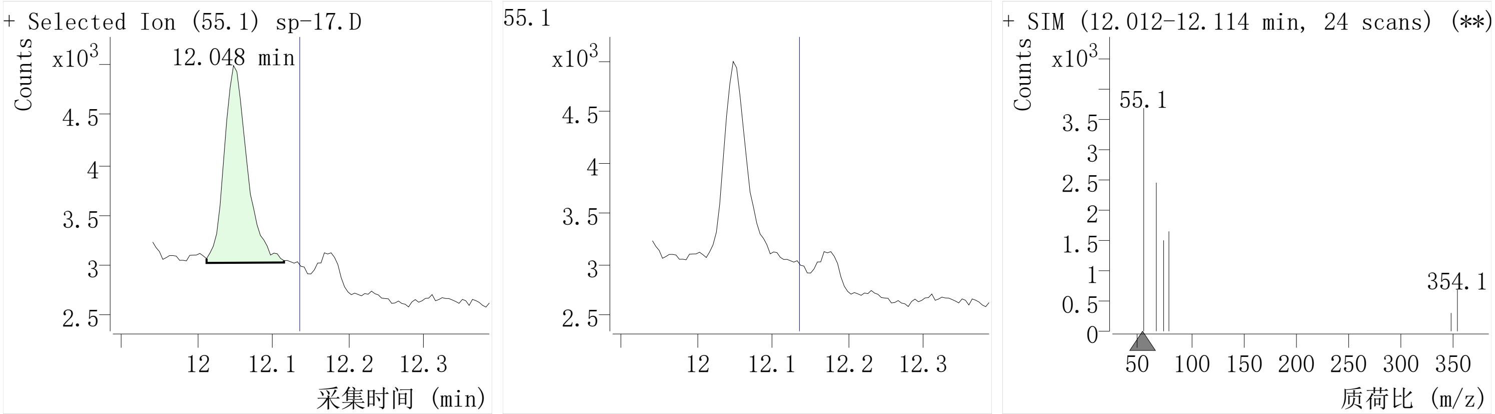

C22:2n6

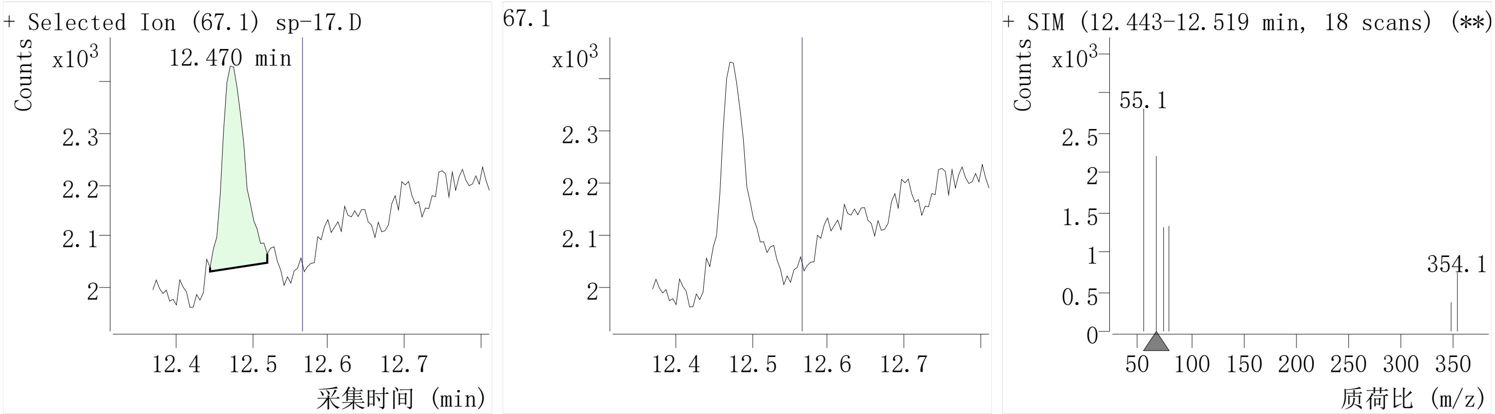

C23:0

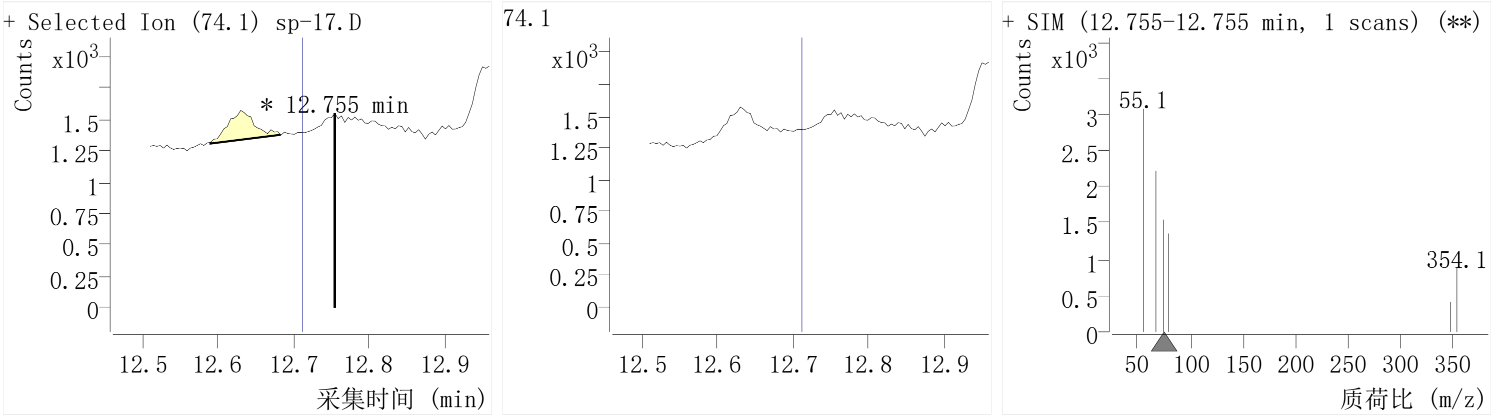

C24:0

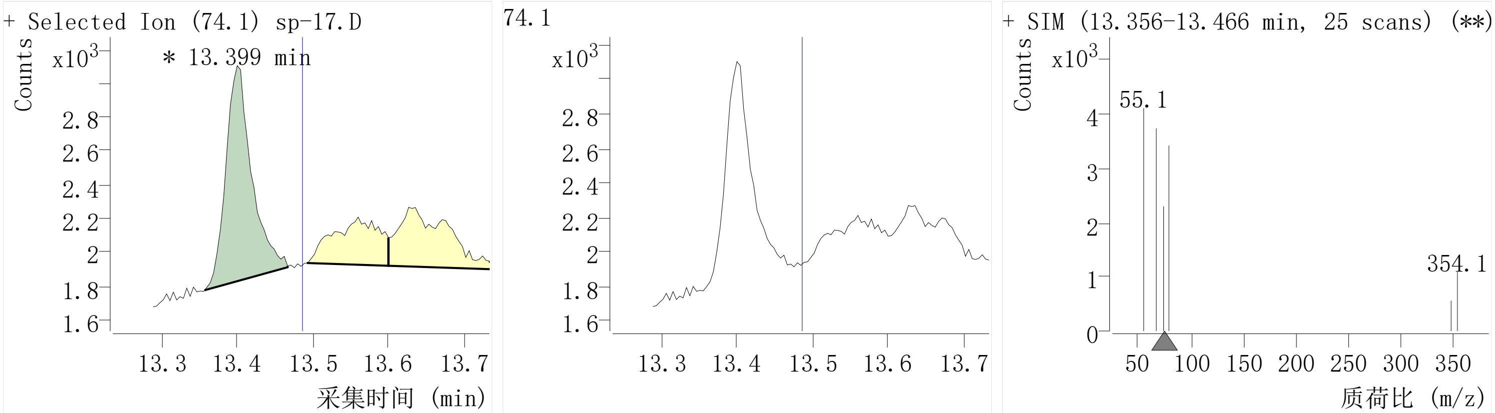

C22:6

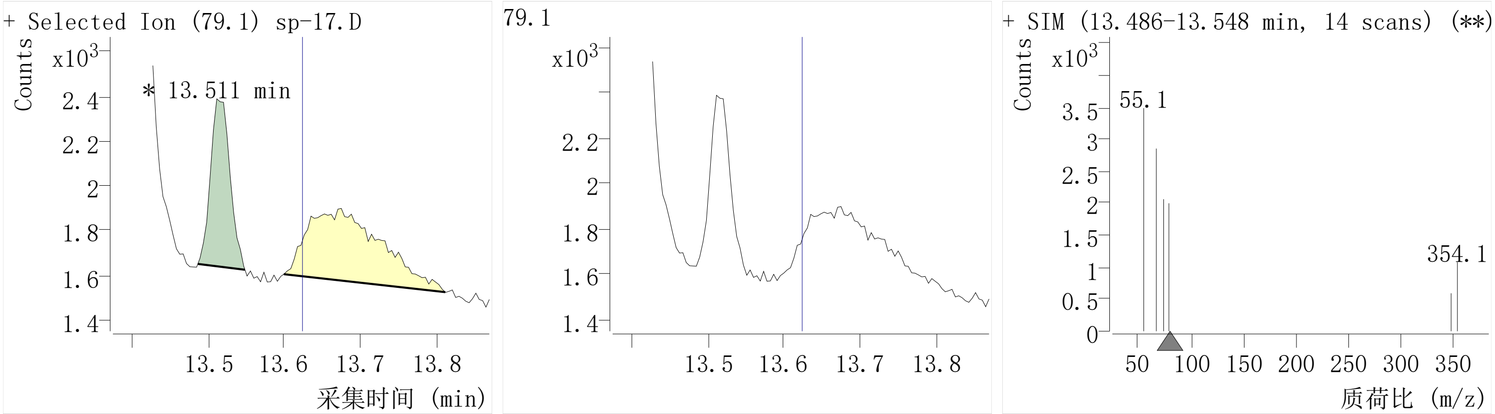
+ Selected Ion (79.1) sp-17.D
Counts
x10^3
* 13.511 min
采集时间 (min)
79.1
+ SIM (13.486-13.548 min, 14 scans) (**)
55.1
354.1
质荷比 (m/z)

C24:1

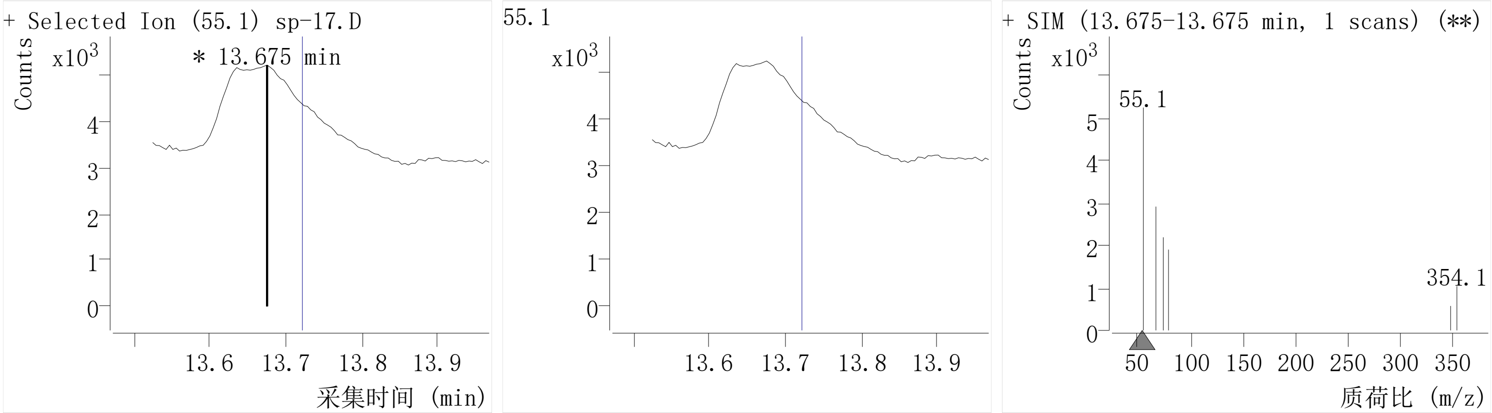
+ Selected Ion (55.1) sp-17.D
Counts
x10^3
* 13.675 min
采集时间 (min)
55.1
+ SIM (13.675-13.675 min, 1 scans) (**)
55.1
354.1
质荷比 (m/z)

定量分析完成报告

**批处理路径**	G:\GC-MS\HX250430-4-GCMS总脂肪酸靶向检测\HX250430-4\QuantResults\HX250430-4.batch.bin		
**分析时间**	2025/5/14 16:58	**分析员姓名**	DESKTOP-M3A0GPO\omics
**报告时间**	2025/5/16 14:53:16	**报告员姓名**	DESKTOP-M3A0GPO\omics
**最近校正更新**	2025/5/14 16:58	**批处理状态**	已处理
**定量批处理版本**	10.2	**定量报告版本**	10.2

**采集时间**	2025/5/9 4:15	**数据文件**	sp-18.D
**样品类型**	样品	**样品名称**	sp-18
**稀释**	1	**采集方法**	脂肪酸

样品色谱图

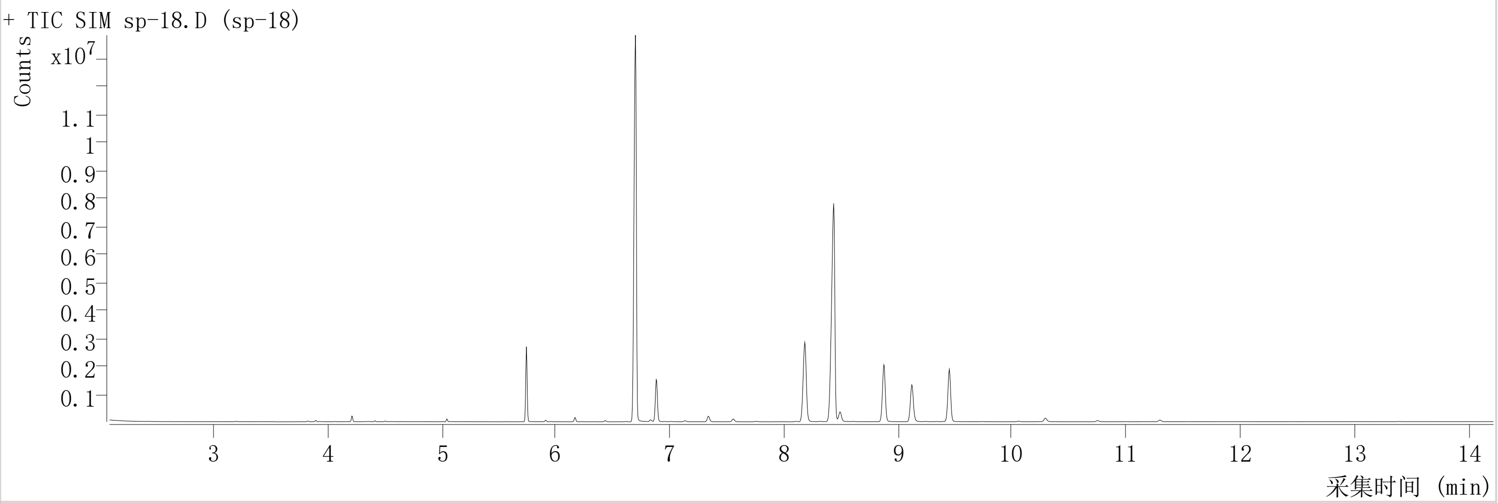

化合物	ISTD	RT	响应	ISTD 响应	响应比	最终浓度	单位
C4:0	C19:0	2.198	0	1869407	0.0000	ND	ug/ml
C6:0	C19:0	2.959	666	1869407	0.0004	0.0037	ug/ml
C8:0	C19:0	3.728	2185	1869407	0.0012	0.0077	ug/ml
C10:0	C19:0	4.417	21002	1869407	0.0112	0.1592	ug/ml
C11:0	C19:0	4.729	1024	1869407	0.0005	0.0043	ug/ml
C12:0	C19:0	5.044	61588	1869407	0.0329	0.4631	ug/ml
C13:0	C19:0	5.378	3094	1869407	0.0017	0.0160	ug/ml
C14:0	C19:0	5.742	1848245	1869407	0.9887	20.4843	ug/ml
C14:1	C19:0	5.911	28667	1869407	0.0153	0.6667	ug/ml
C15:0	C19:0	6.169	123166	1869407	0.0659	1.0978	ug/ml
C15:1	C19:0	6.432	0	1869407	0.0000	ND	ug/ml
C16:0	C19:0	6.697	13834625	1869407	7.4005	285.2698	ug/ml
C16:1	C19:0	6.881	923349	1869407	0.4939	30.0960	ug/ml
C17:0	C19:0	7.339	233475	1869407	0.1249	2.6056	ug/ml
C17:1	C19:0	7.557	75600	1869407	0.0404	2.1492	ug/ml
C18:0	C19:0	8.184	4231490	1869407	2.2635	50.5980	ug/ml
C18:1n9t	C19:0	8.321	9766	1869407	0.0052	0.3082	ug/ml
C18:1n9c	C19:0	8.437	7122606	1869407	3.8101	256.6235	ug/ml
C18:2n6t	C19:0	8.877	0	1869407	0.0000	ND	ug/ml
C18:2n6c	C19:0	8.877	1600751	1869407	0.8563	55.1359	ug/ml
C18:3n6	C19:0	9.122	20450	1869407	0.0109	ND	ug/ml
C18:3n3	C19:0	9.451	1485309	1869407	0.7945	39.4967	ug/ml
C20:0	C19:0	10.056	30985	1869407	0.0166	0.4521	ug/ml
C20:1	C19:0	10.291	126885	1869407	0.0679	4.2227	ug/ml
C20:2	C19:0	10.749	34384	1869407	0.0184	1.1788	ug/ml
C21:0	C19:0	10.958	1849	1869407	0.0010	0.0274	ug/ml
C20:3n6	C19:0	11.003	3114	1869407	0.0017	0.1494	ug/ml
C20:4n6	C19:0	11.154	3139	1869407	0.0017	0.1400	ug/ml
C20:3n3	C19:0	11.296	49892	1869407	0.0267	1.4464	ug/ml
C20:5n3	C19:0	11.696	890	1869407	0.0005	0.0615	ug/ml

化合物	ISTD	RT	响应	ISTD 响应	响应比	最终浓度	单位
C22:0	C19:0	11.821	2218	1869407	0.0012	0.0587	ug/ml
C22:1n9	C19:0	12.048	3323	1869407	0.0018	0.1168	ug/ml
C22:2n6	C19:0	12.475	615	1869407	0.0003	0.0629	ug/ml
C23:0	C19:0	12.786	0	1869407	0.0000	ND	ug/ml
C24:0	C19:0	13.395	1035	1869407	0.0006	0.0313	ug/ml
C22:6	C19:0	13.515	838	1869407	0.0004	0.0383	ug/ml
C24:1	C19:0	13.671	0	1869407	0.0000	ND	ug/ml

C4:0

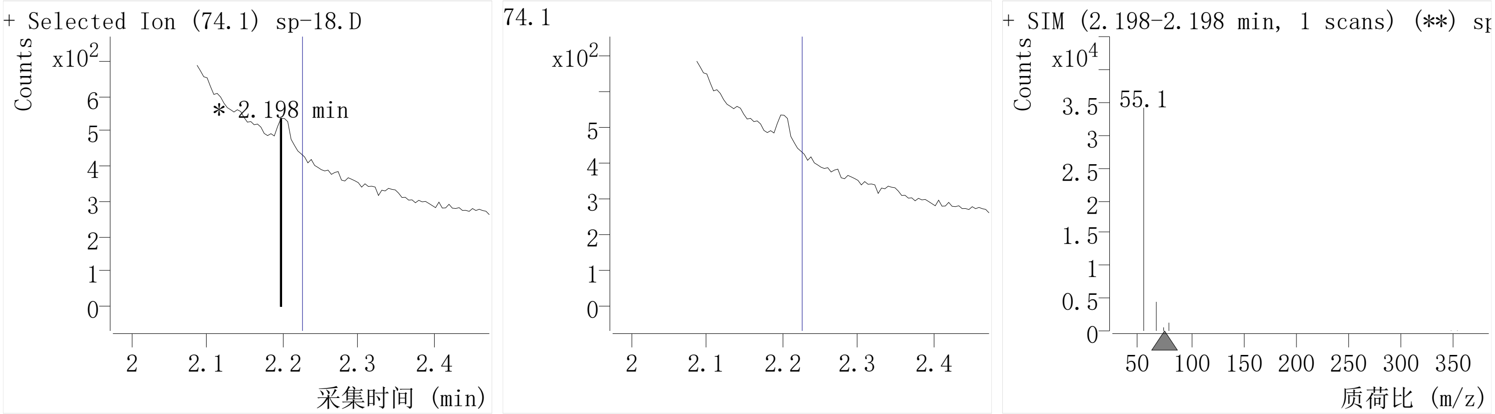

C6:0

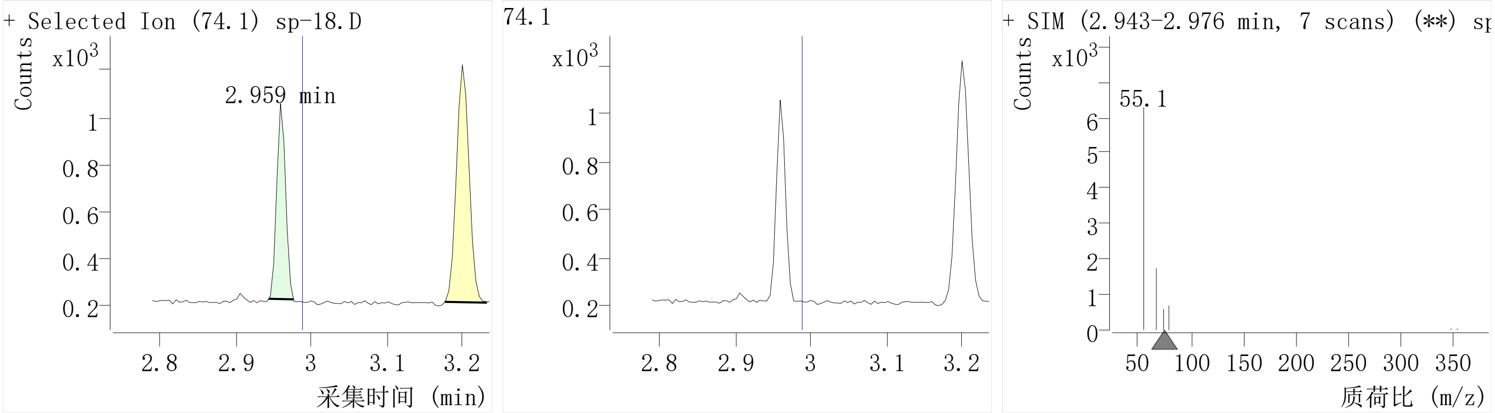

C8:0

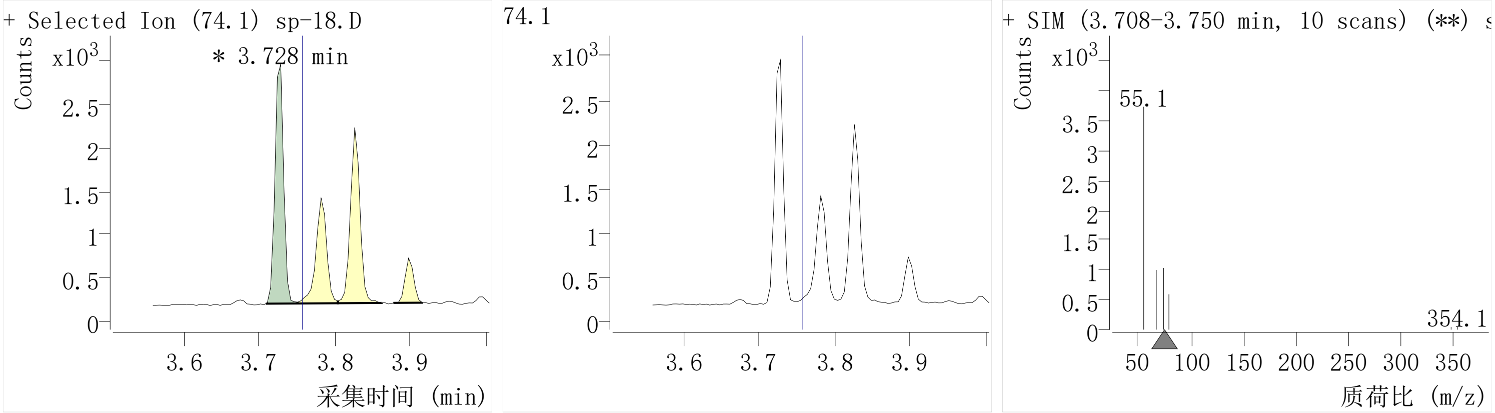

C10:0

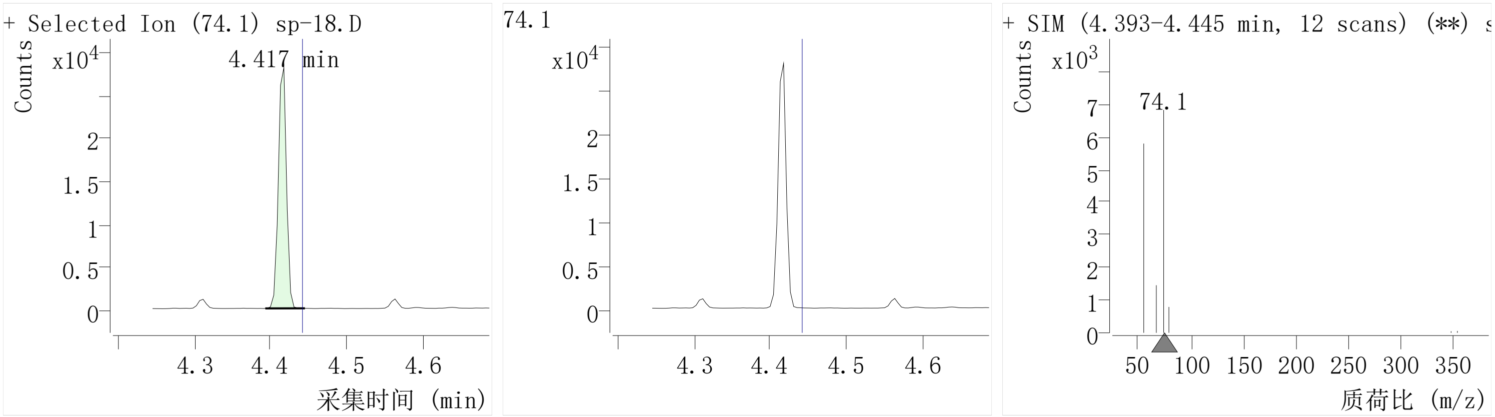

C11:0

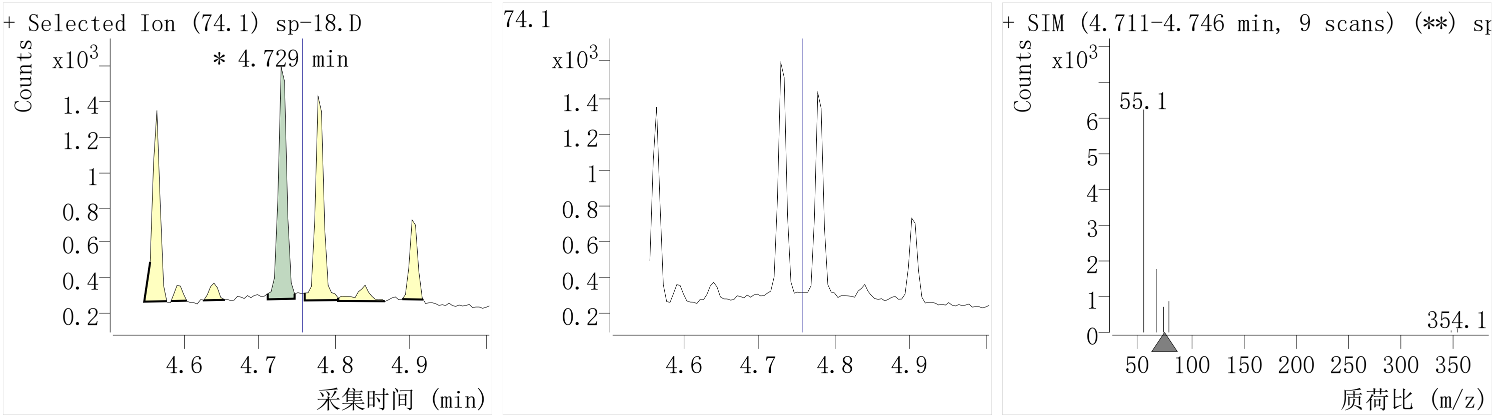

C12:0

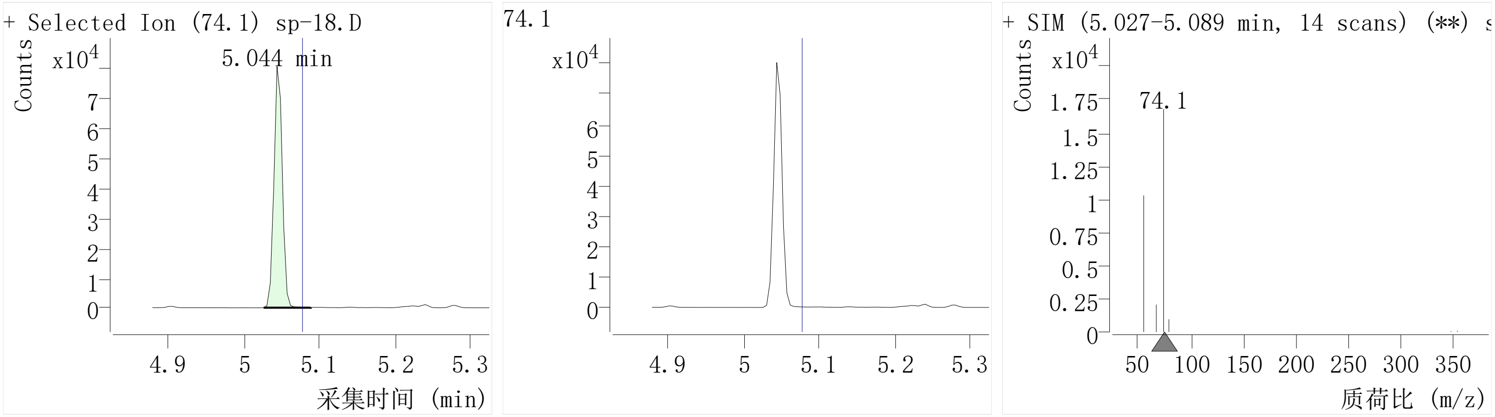

C13:0

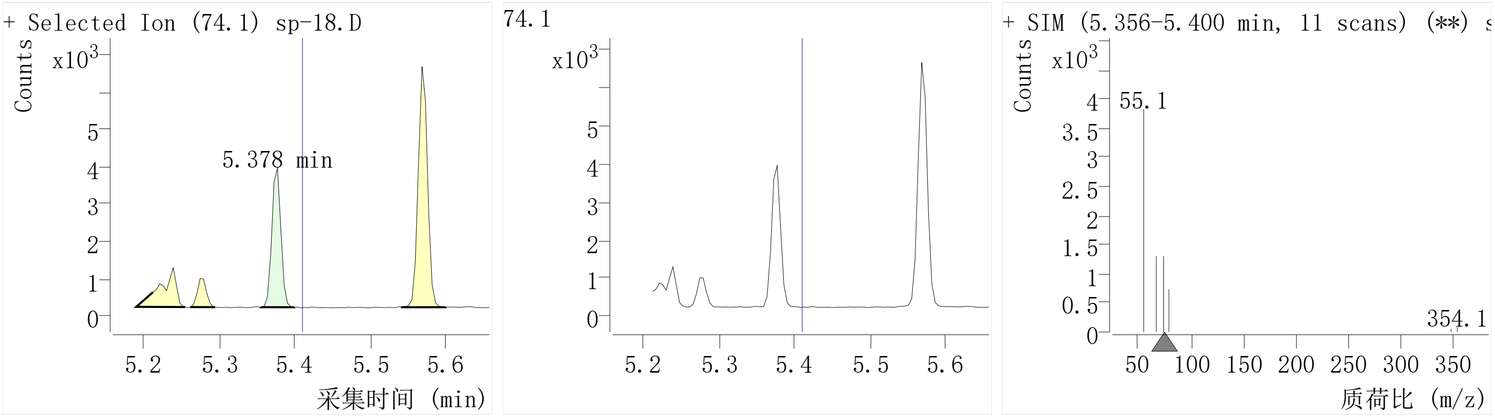

C14:0

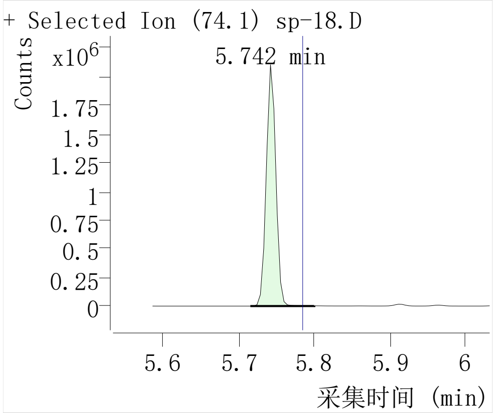

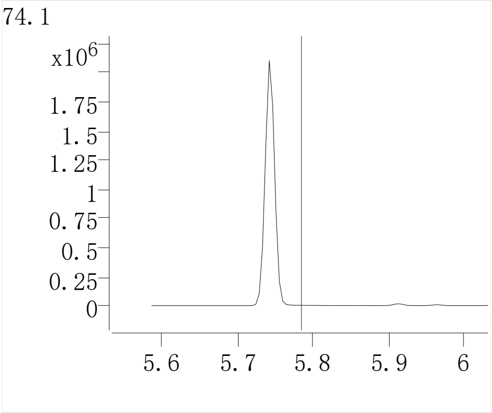

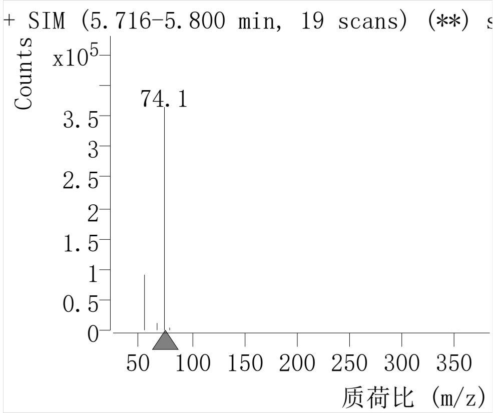

C14:1

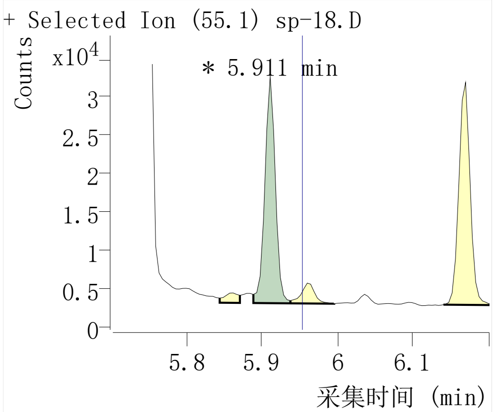

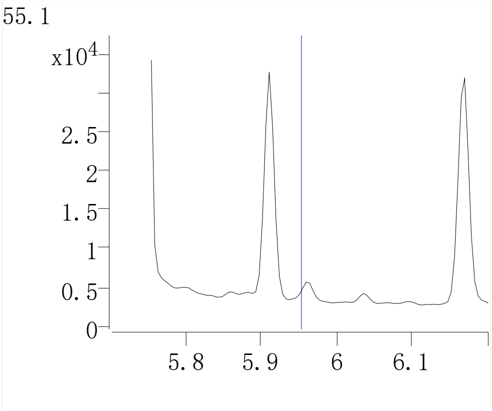

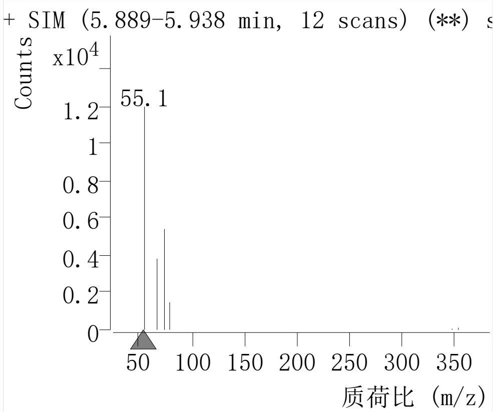

C15:0

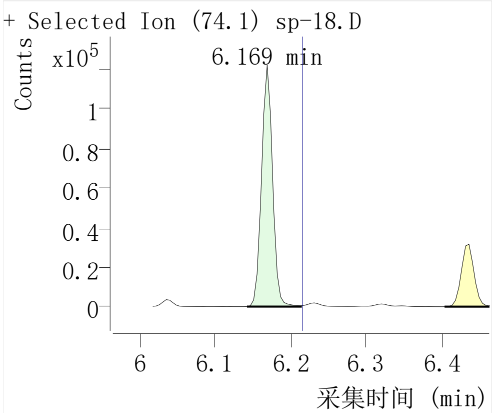

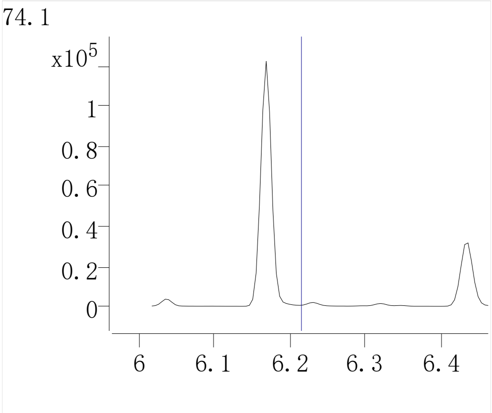

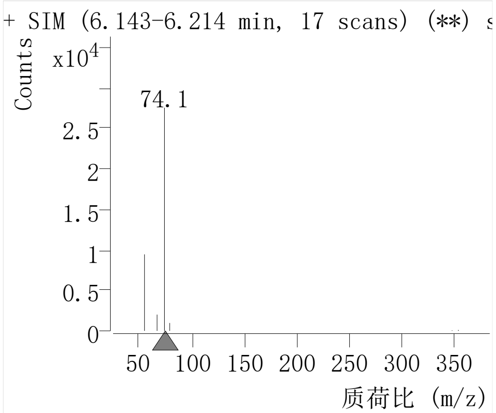

C15:1

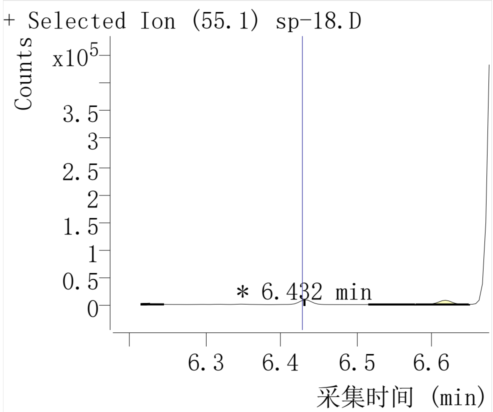

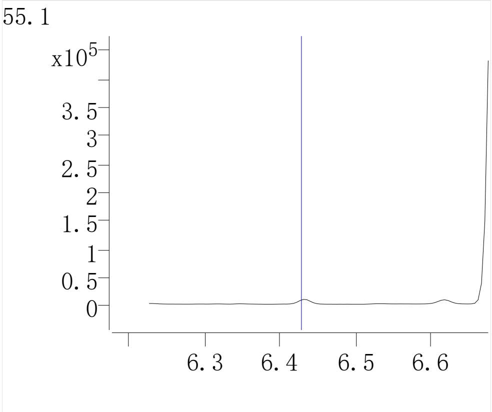

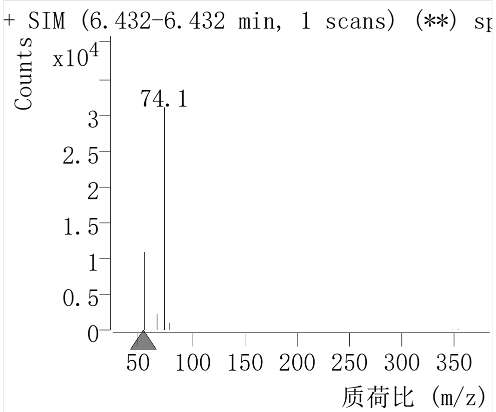

 生成时间 2025/5/16 14:52

C16:0

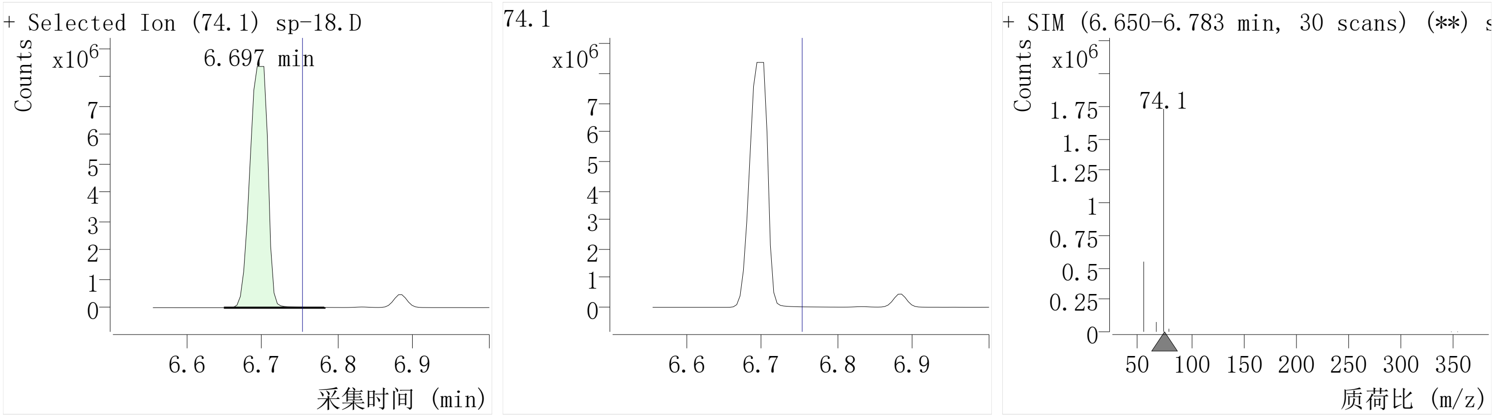

C16:1

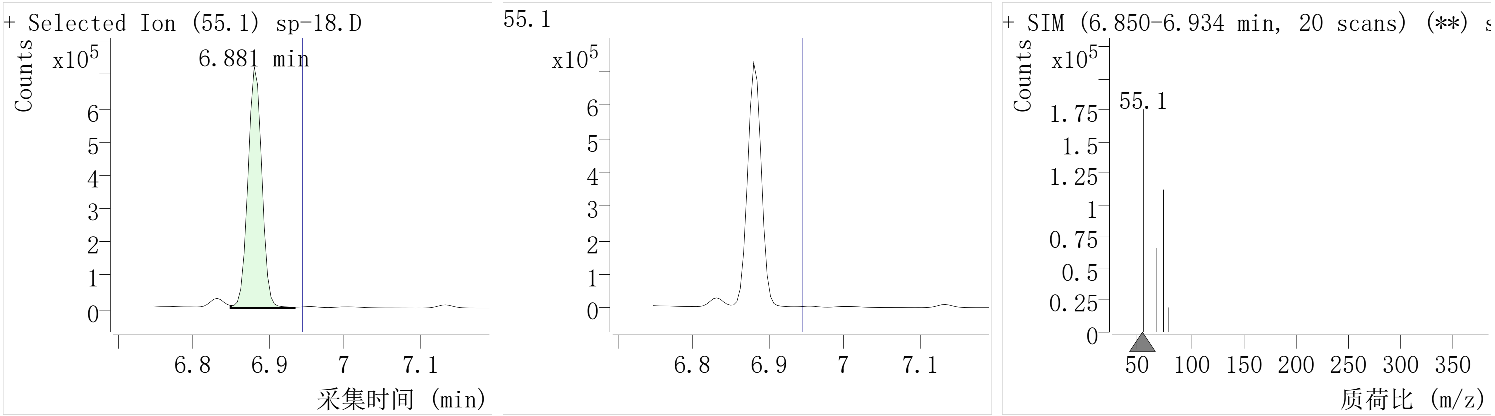

C17:0

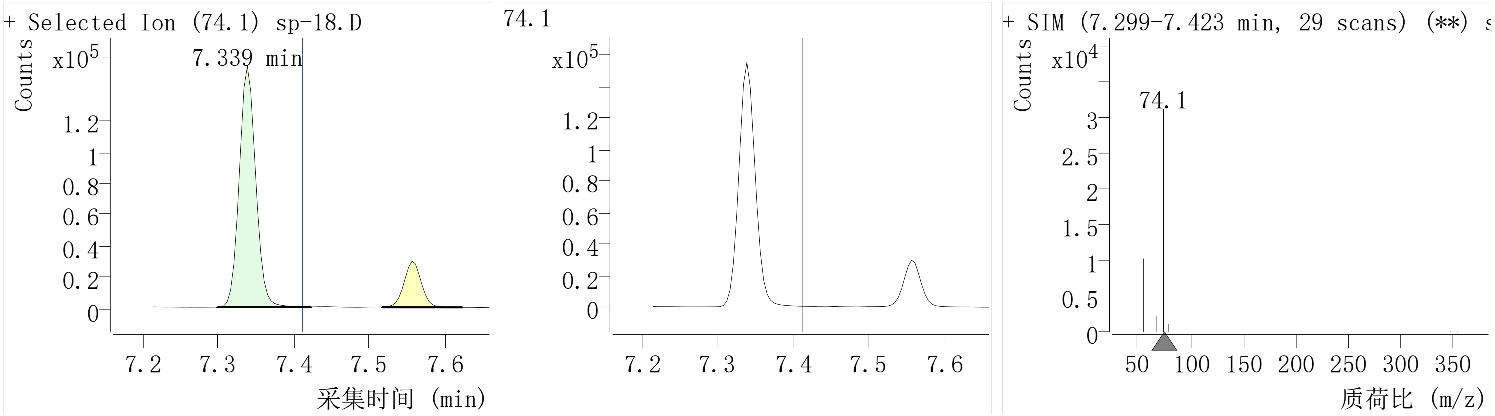

C17:1

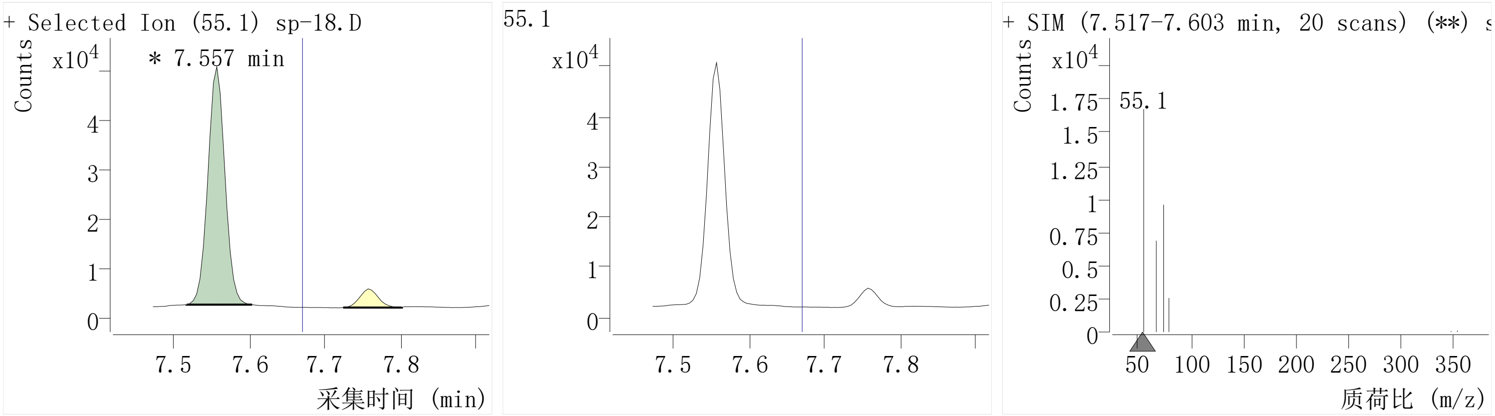

C18:0

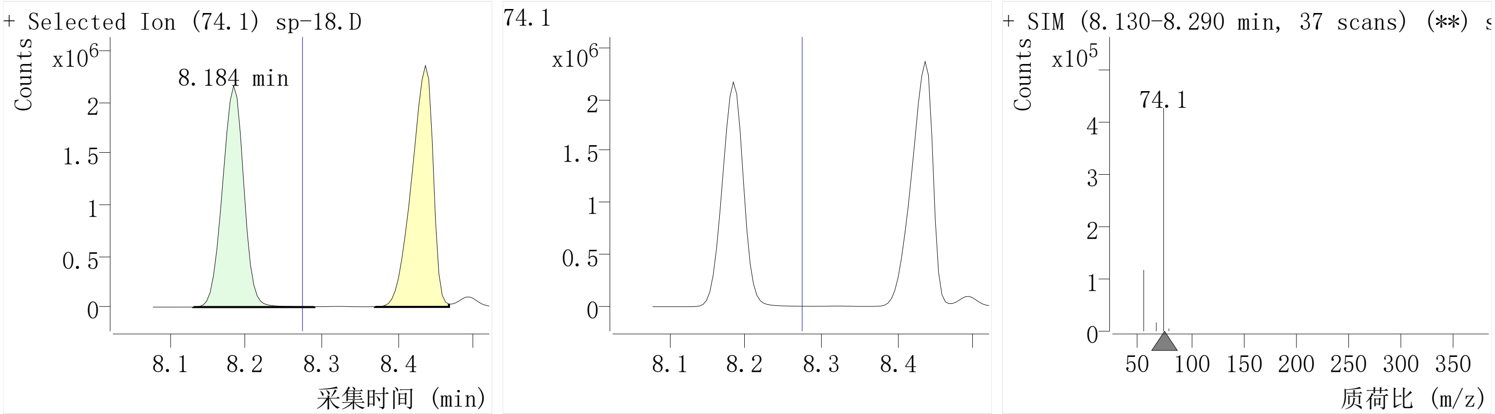

C18:1n9t

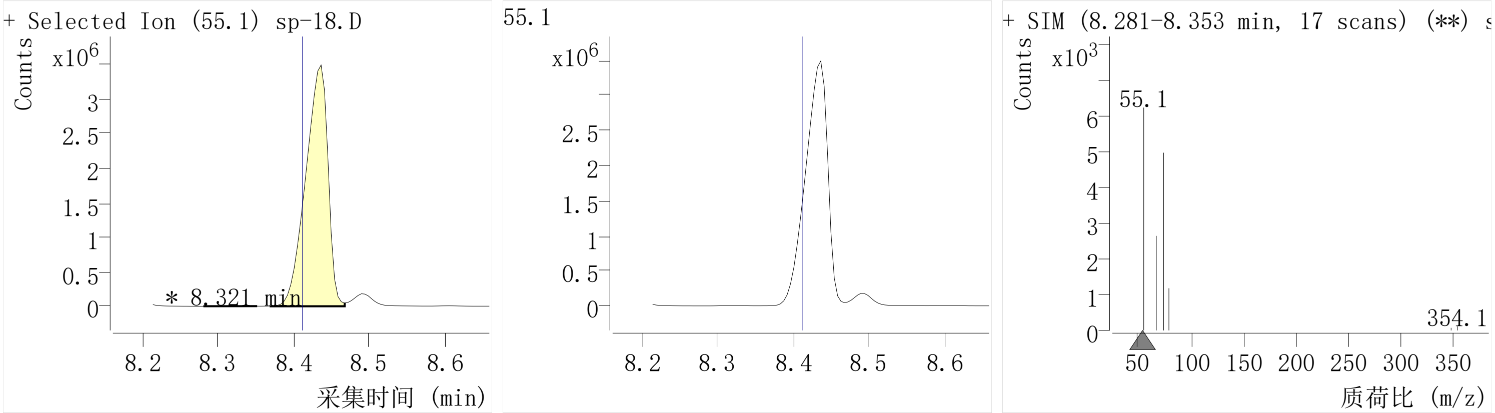

C18:1n9c

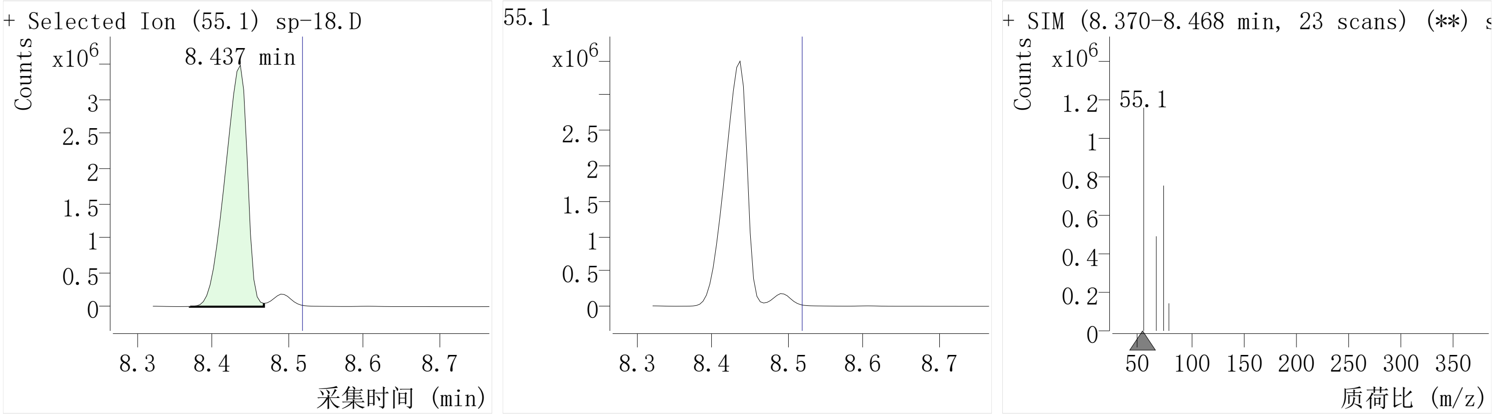

C18:2n6t

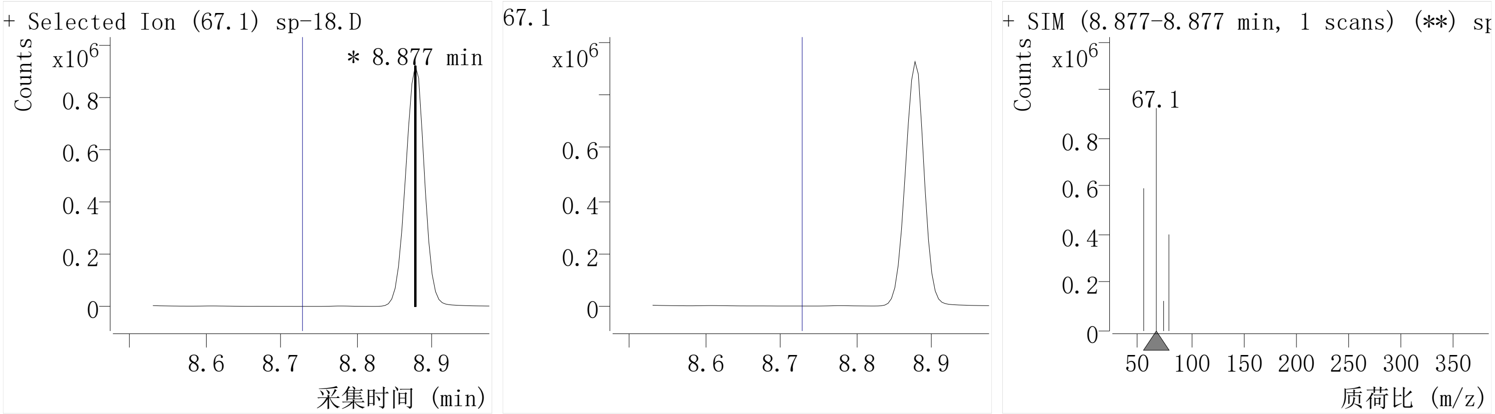

C18:2n6c

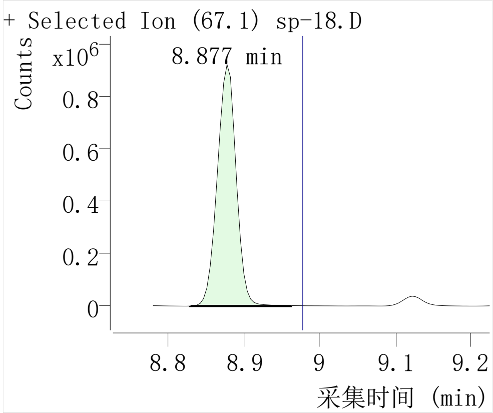

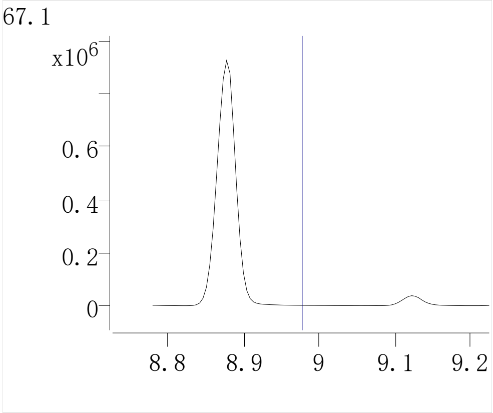

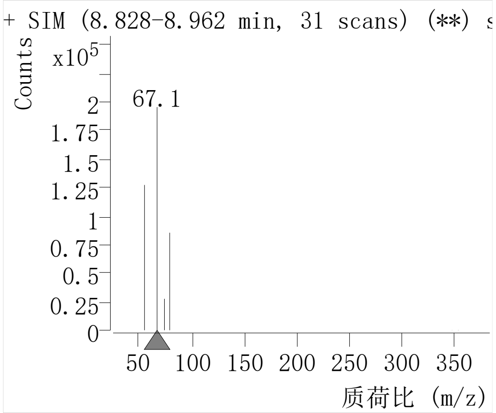

C18:3n6

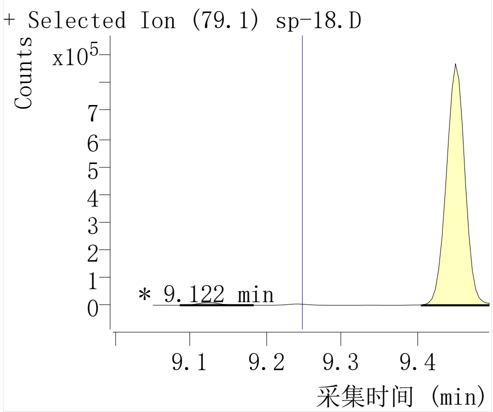

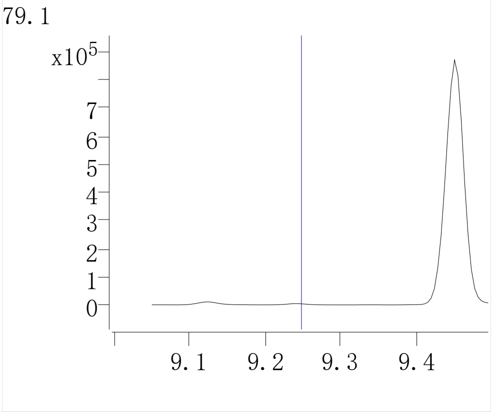

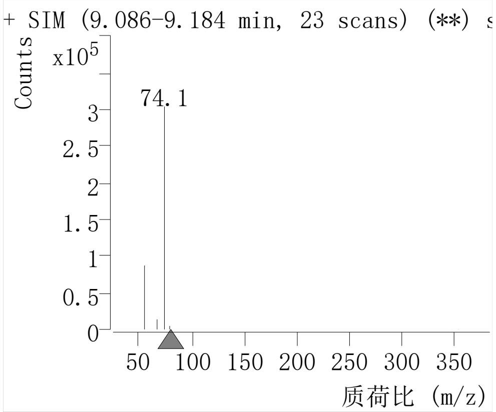

C18:3n3

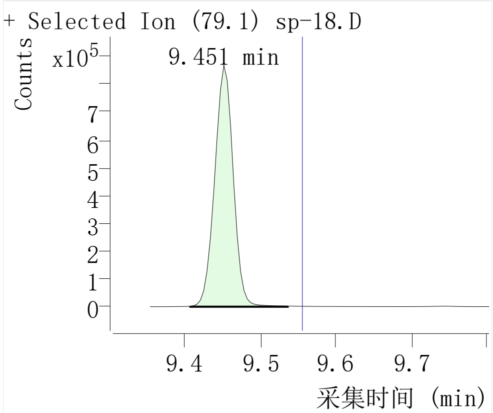

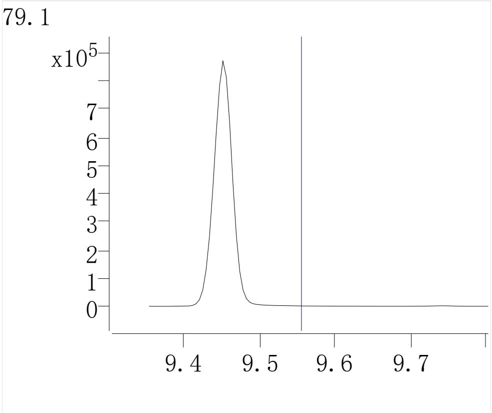

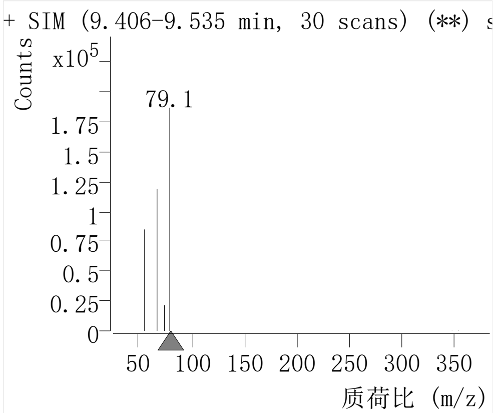

C20:0

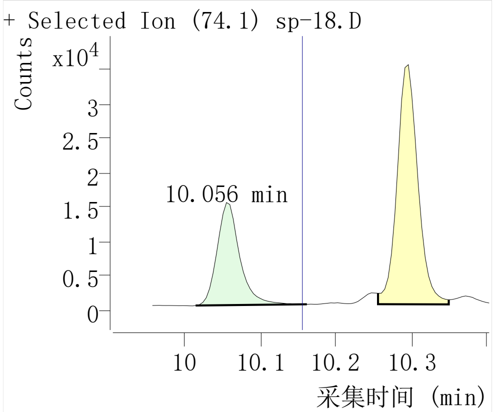

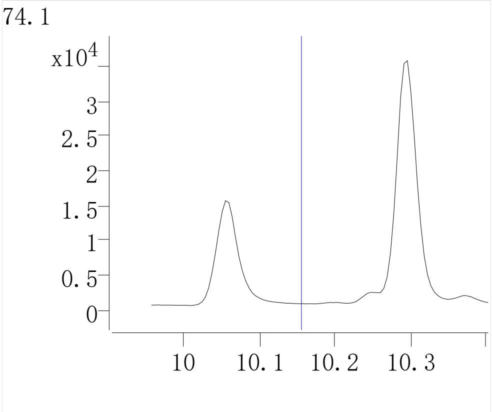

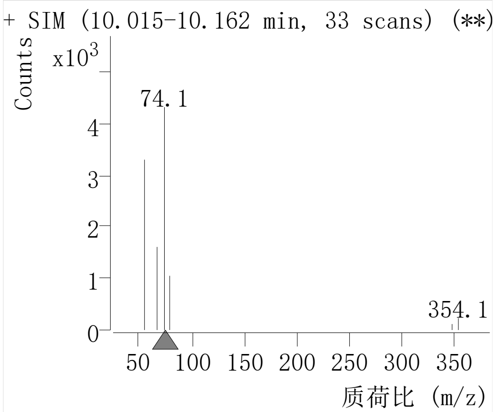

C20:1

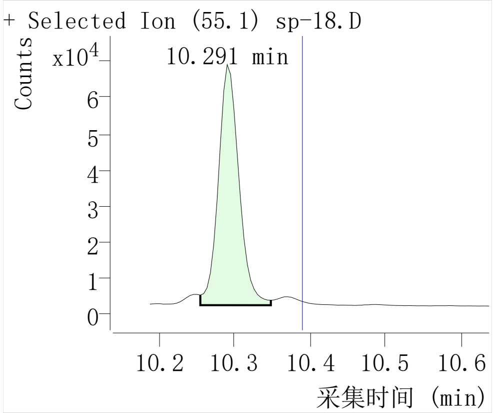

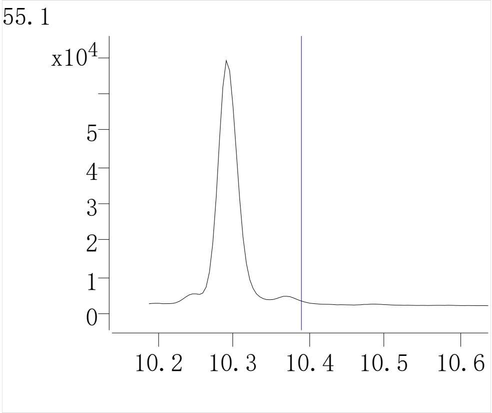

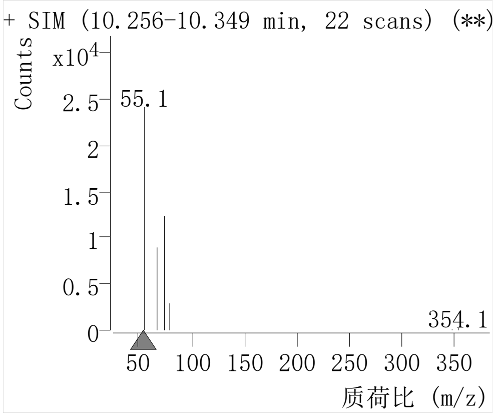

C20:2

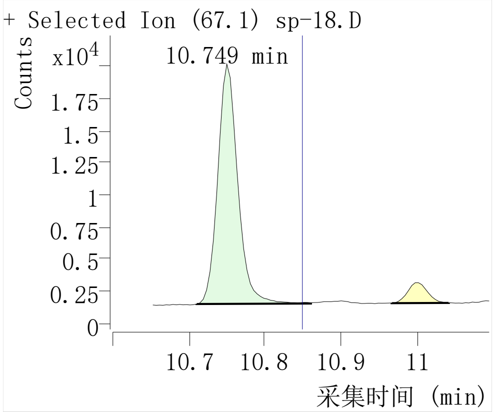

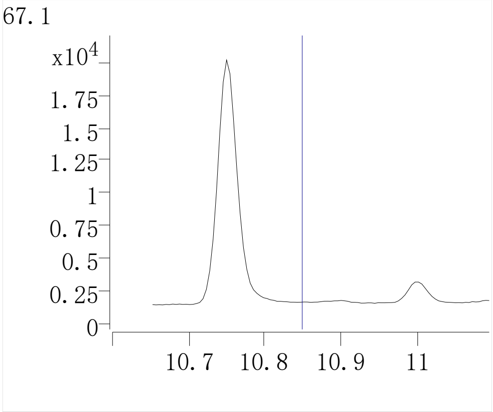

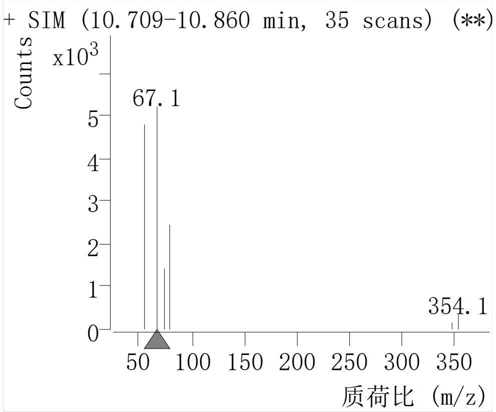

C21:0

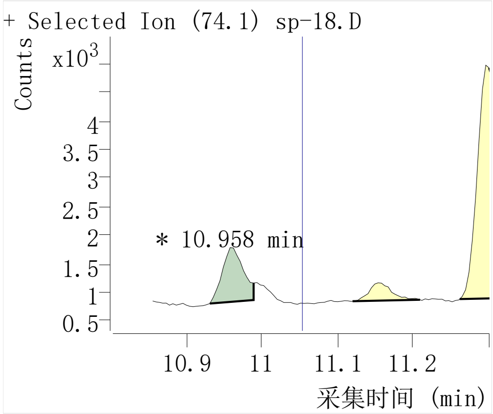

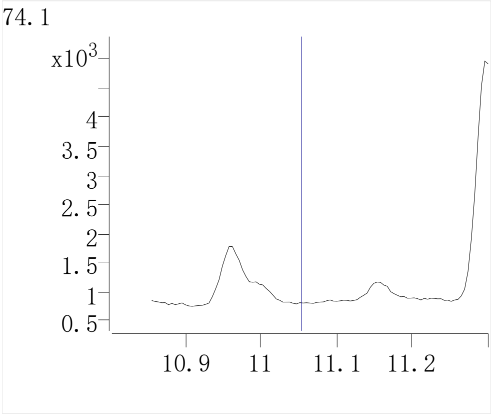

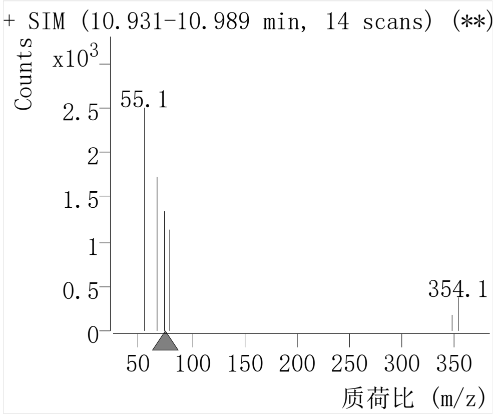

C20:3n6

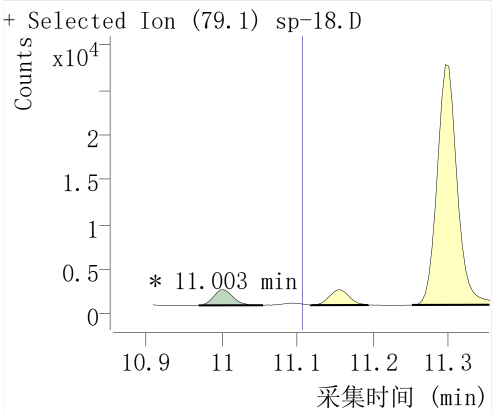

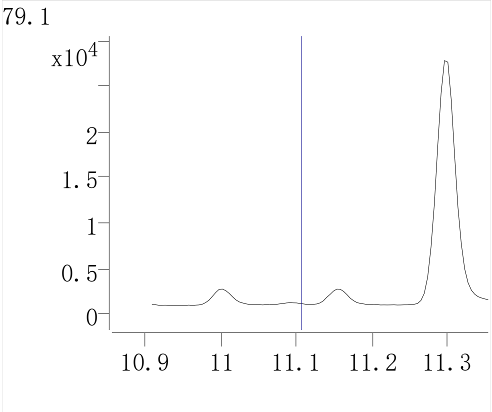

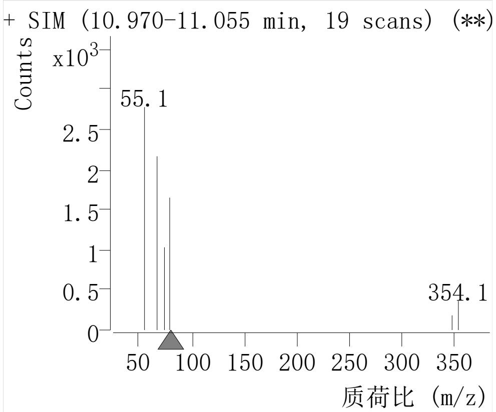

C20:4n6

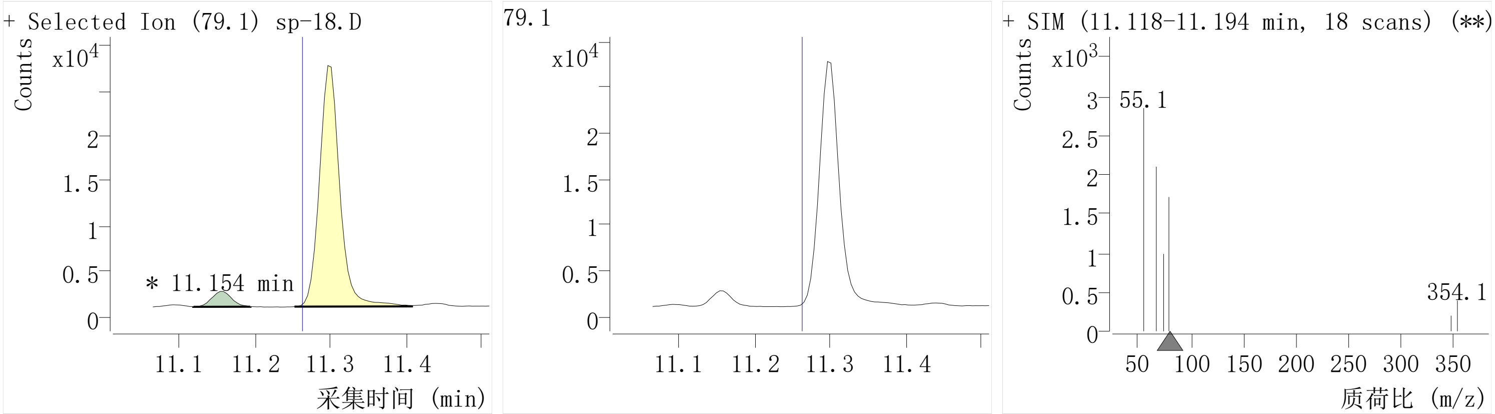

C20:3n3

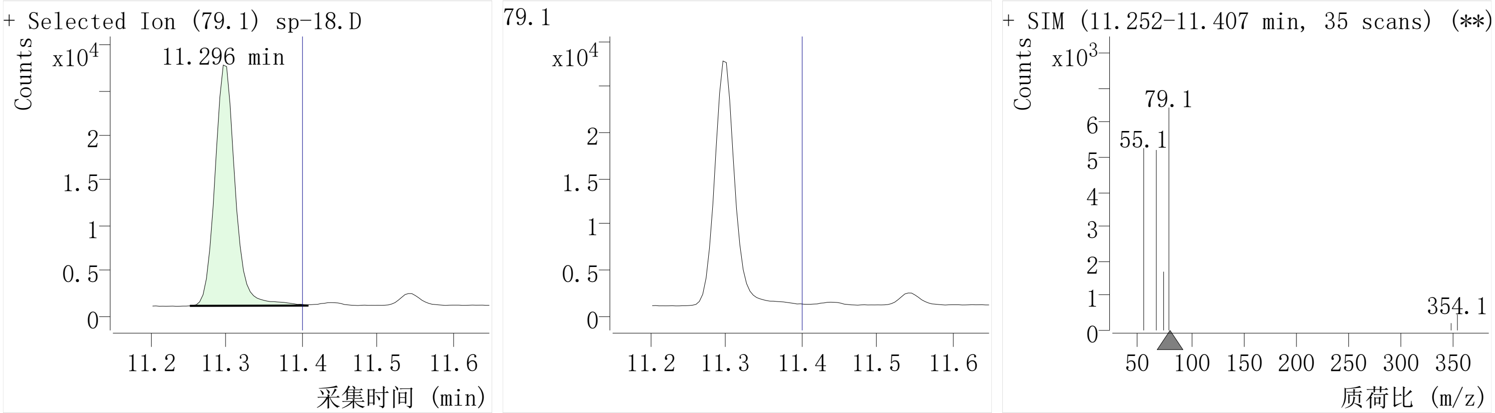

C20:5n3

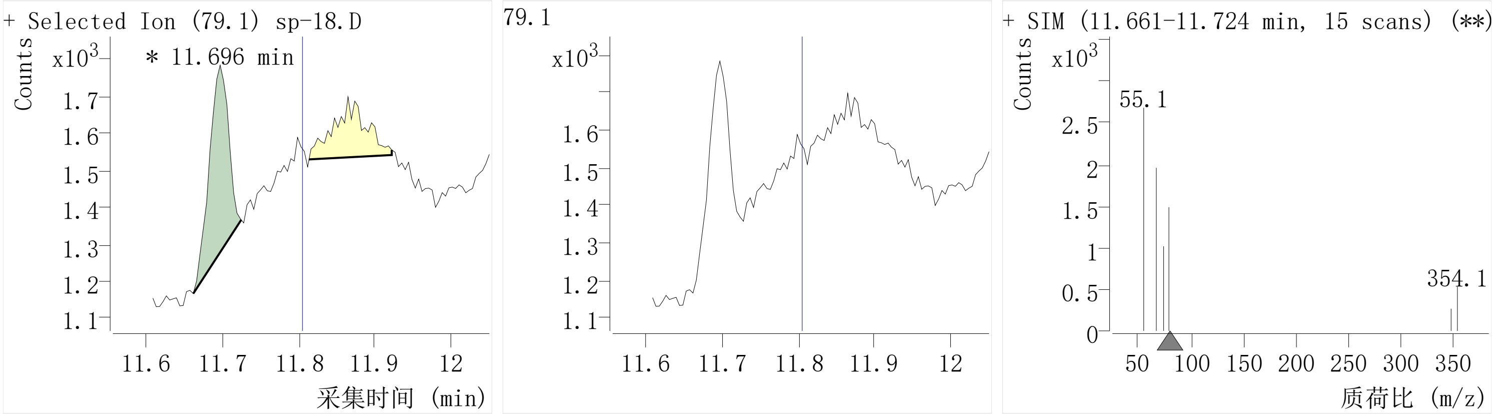

C22:0

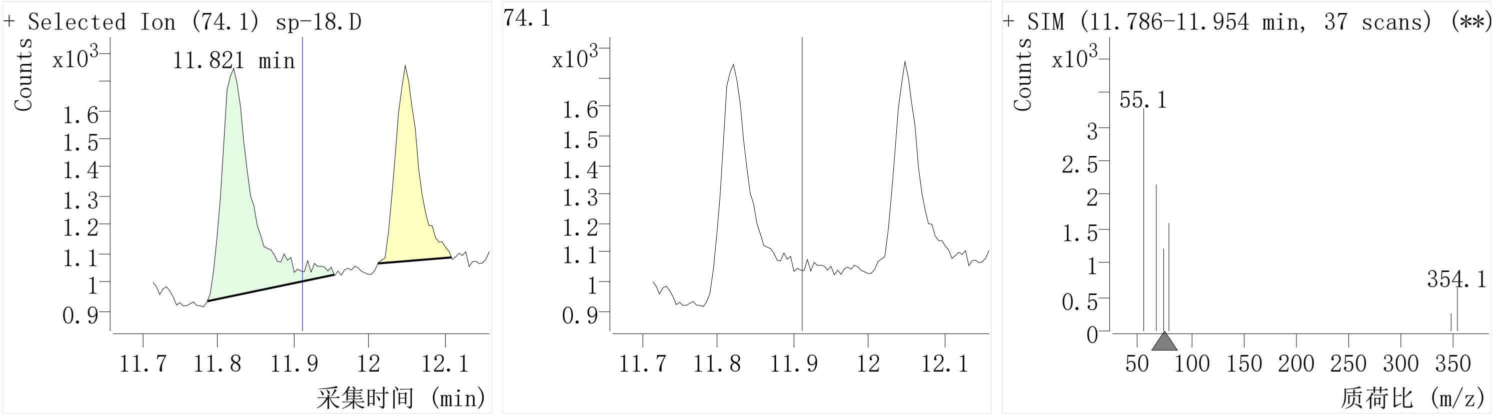

生成时间 2025/5/16 14:52

C22:1n9

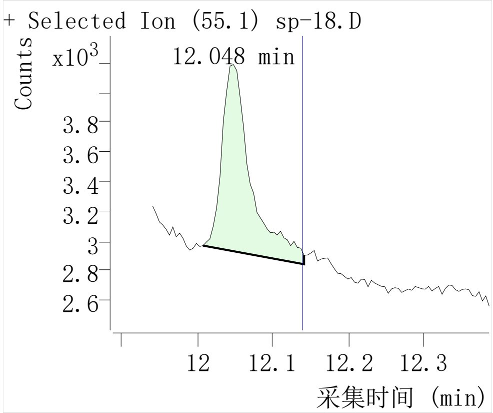

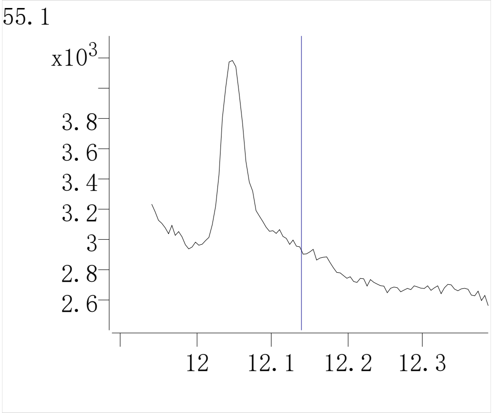

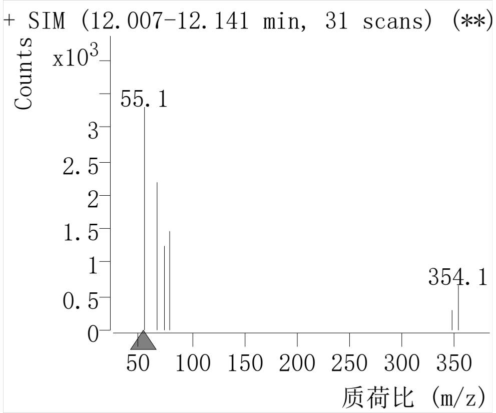

C22:2n6

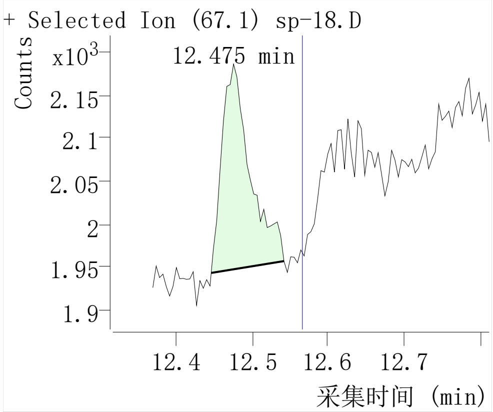

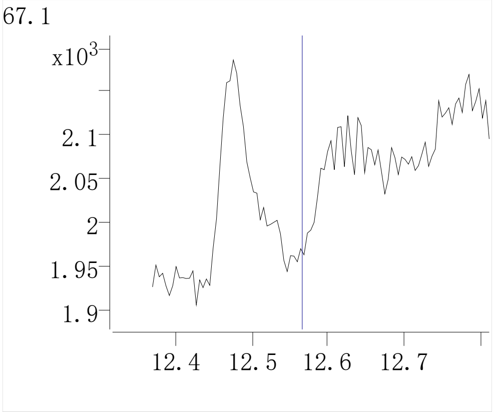

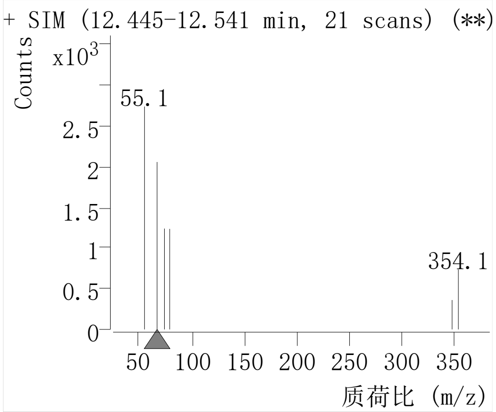

C23:0

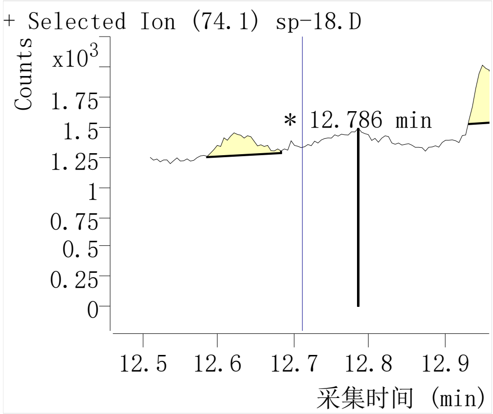

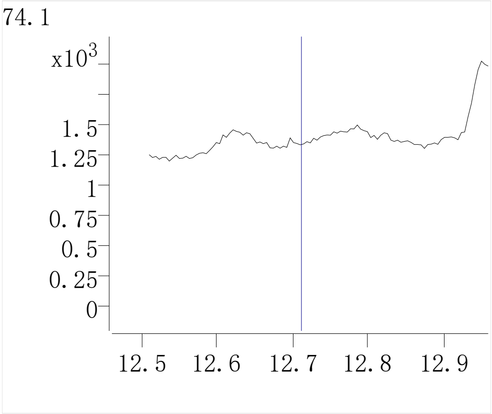

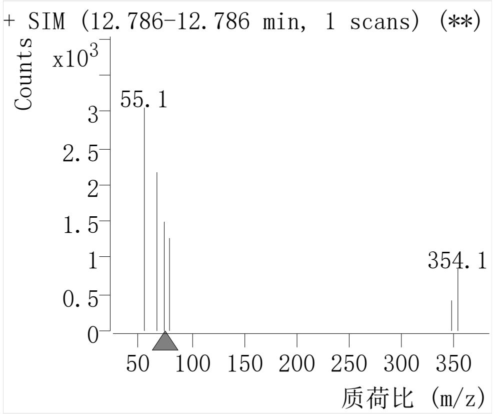

C24:0

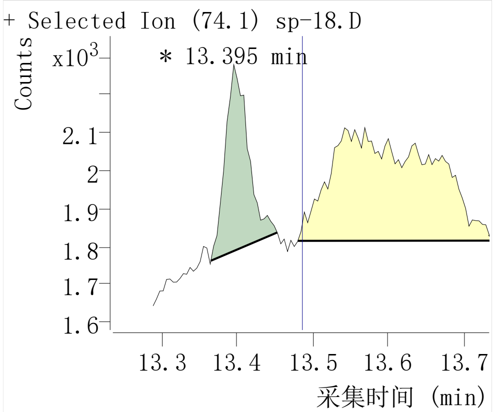

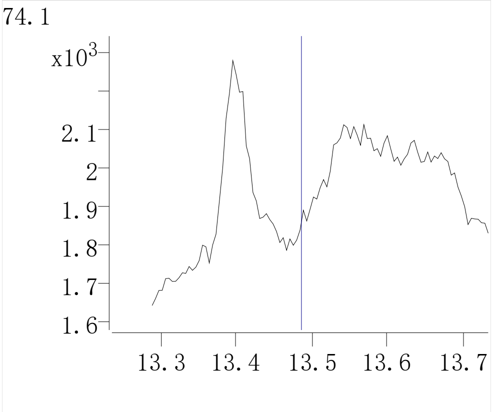

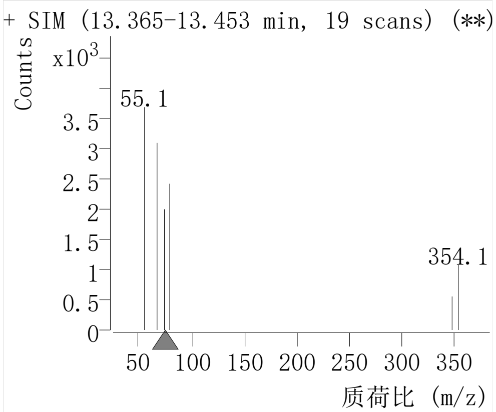

C22:6

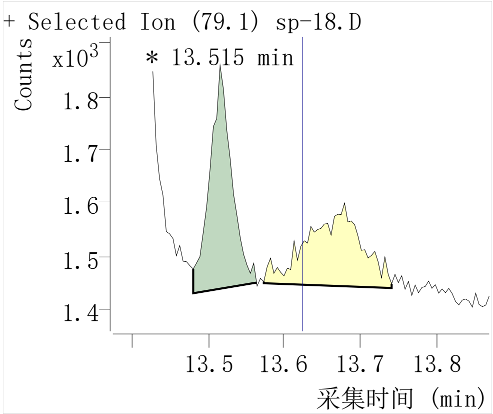
+ Selected Ion (79.1) sp-18.D
Counts
x10^3
* 13.515 min
1.8
1.7
1.6
1.5
1.4
13.5
13.6
13.7
13.8
采集时间 (min)

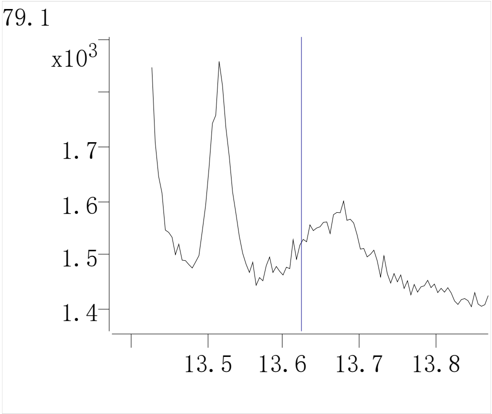
79.1
x10^3
1.7
1.6
1.5
1.4
13.5
13.6
13.7
13.8

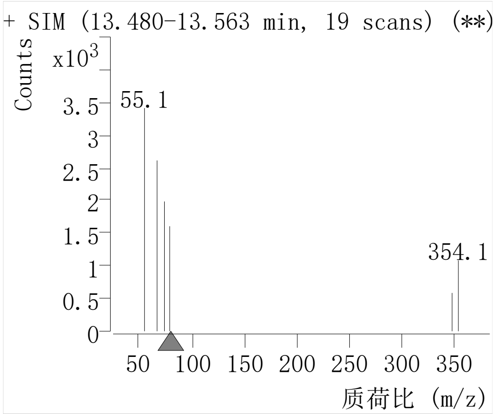
+ SIM (13.480-13.563 min, 19 scans) (**)
Counts
x10^3
55.1
354.1
3.5
3
2.5
2
1.5
1
0.5
0
50
100
150
200
250
300
350
质荷比 (m/z)

C24:1

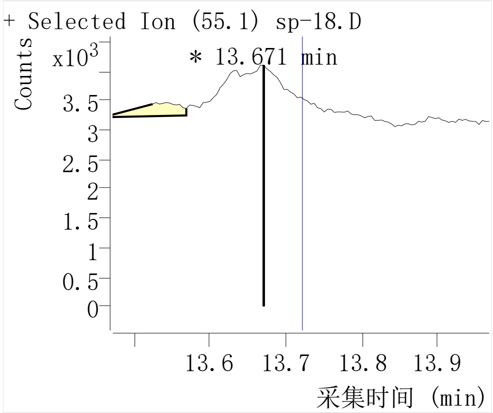
+ Selected Ion (55.1) sp-18.D
Counts
x10^3
* 13.671 min
3.5
3
2.5
2
1.5
1
0.5
0
13.6
13.7
13.8
13.9
采集时间 (min)

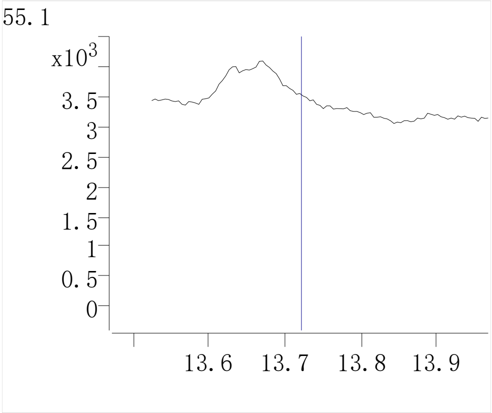
55.1
x10^3
3.5
3
2.5
2
1.5
1
0.5
0
13.6
13.7
13.8
13.9

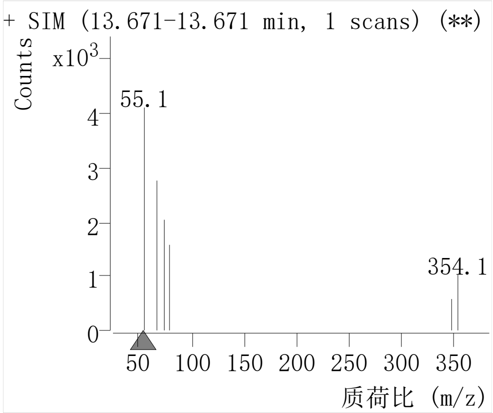
+ SIM (13.671-13.671 min, 1 scans) (**)
Counts
x10^3
55.1
354.1
4
3
2
1
0
50
100
150
200
250
300
350
质荷比 (m/z)

定量分析完成报告

**批处理路径**	G:\GC-MS\HX250430-4-GCMS总脂肪酸靶向检测\HX250430-4\QuantResults\HX250430-4.batch.bin		
**分析时间**	2025/5/14 16:58	**分析员姓名**	DESKTOP-M3A0GPO\omics
**报告时间**	2025/5/16 14:53:17	**报告员姓名**	DESKTOP-M3A0GPO\omics
**最近校正更新**	2025/5/14 16:58	**批处理状态**	已处理
**定量批处理版本**	10.2	**定量报告版本**	10.2

**采集时间**	2025/5/9 4:35	**数据文件**	sp-19.D
**样品类型**	样品	**样品名称**	sp-19
**稀释**	1	**采集方法**	脂肪酸

样品色谱图

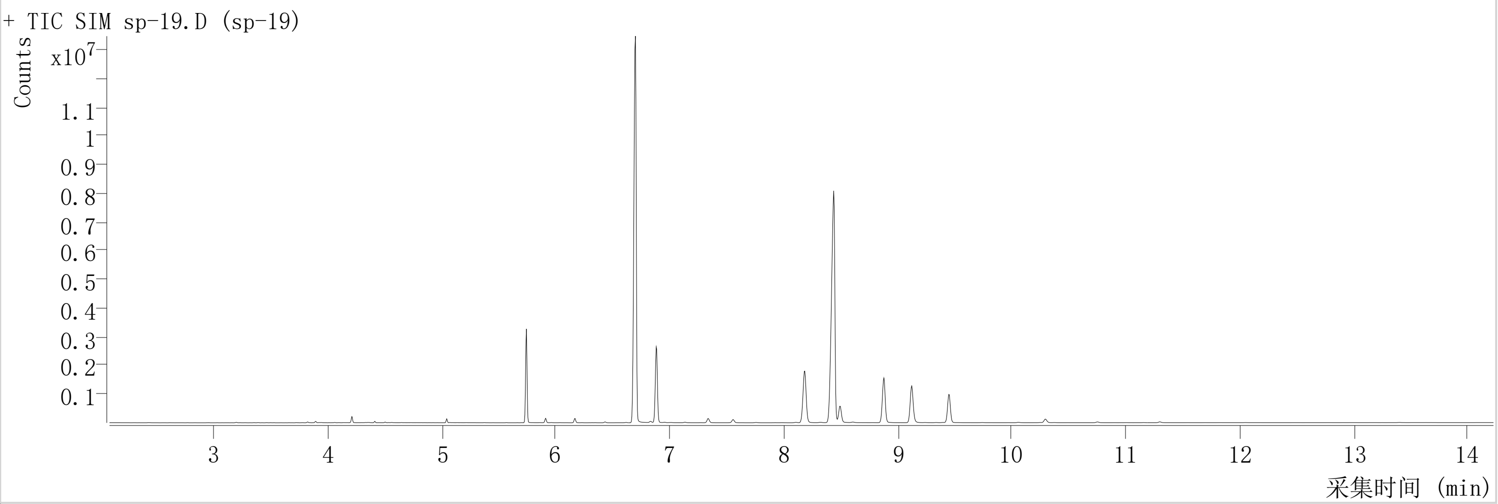

化合物	ISTD	RT	响应	ISTD 响应	响应比	最终浓度	单位
C4:0	C19:0	2.203	232	1809576	0.0001	ND	ug/ml
C6:0	C19:0	2.959	1163	1809576	0.0006	0.0082	ug/ml
C8:0	C19:0	3.724	3315	1809576	0.0018	0.0143	ug/ml
C10:0	C19:0	4.413	27830	1809576	0.0154	0.2348	ug/ml
C11:0	C19:0	4.729	1064	1809576	0.0006	0.0046	ug/ml
C12:0	C19:0	5.044	79910	1809576	0.0442	0.6585	ug/ml
C13:0	C19:0	5.373	3312	1809576	0.0018	0.0180	ug/ml
C14:0	C19:0	5.743	2252698	1809576	1.2449	26.9460	ug/ml
C14:1	C19:0	5.911	75782	1809576	0.0419	2.0055	ug/ml
C15:0	C19:0	6.169	123499	1809576	0.0682	1.1418	ug/ml
C15:1	C19:0	6.432	0	1809576	0.0000	ND	ug/ml
C16:0	C19:0	6.696	13790339	1809576	7.6208	297.1807	ug/ml
C16:1	C19:0	6.881	1592899	1809576	0.8803	53.7054	ug/ml
C17:0	C19:0	7.334	170876	1809576	0.0944	1.9701	ug/ml
C17:1	C19:0	7.552	84598	1809576	0.0468	2.4890	ug/ml
C18:0	C19:0	8.184	2677429	1809576	1.4796	31.3911	ug/ml
C18:1n9t	C19:0	8.322	10968	1809576	0.0061	0.3575	ug/ml
C18:1n9c	C19:0	8.437	7495252	1809576	4.1420	280.4966	ug/ml
C18:2n6t	C19:0	8.877	0	1809576	0.0000	ND	ug/ml
C18:2n6c	C19:0	8.877	1230662	1809576	0.6801	41.1663	ug/ml
C18:3n6	C19:0	9.122	19057	1809576	0.0105	ND	ug/ml
C18:3n3	C19:0	9.451	793437	1809576	0.4385	21.7935	ug/ml
C20:0	C19:0	10.056	23915	1809576	0.0132	0.3673	ug/ml
C20:1	C19:0	10.291	123141	1809576	0.0680	4.2335	ug/ml
C20:2	C19:0	10.749	23491	1809576	0.0130	0.8481	ug/ml
C21:0	C19:0	10.958	2240	1809576	0.0012	0.0342	ug/ml
C20:3n6	C19:0	11.003	2372	1809576	0.0013	0.1270	ug/ml
C20:4n6	C19:0	11.154	3917	1809576	0.0022	0.1702	ug/ml
C20:3n3	C19:0	11.296	24401	1809576	0.0135	0.7560	ug/ml
C20:5n3	C19:0	11.697	807	1809576	0.0004	0.0598	ug/ml

定量分析完成报告

Agilent Trusted Answers

化合物	ISTD	RT	响应	ISTD 响应	响应比	最终浓度	单位
C22:0	C19:0	11.821	1977	1809576	0.0011	0.0557	ug/ml
C22:1n9	C19:0	12.048	5136	1809576	0.0028	0.1953	ug/ml
C22:2n6	C19:0	12.475	532	1809576	0.0003	0.0602	ug/ml
C23:0	C19:0	12.768	0	1809576	0.0000	ND	ug/ml
C24:0	C19:0	13.395	1585	1809576	0.0009	0.0458	ug/ml
C22:6	C19:0	13.511	603	1809576	0.0003	0.0298	ug/ml
C24:1	C19:0	13.675	0	1809576	0.0000	ND	ug/ml

C4:0

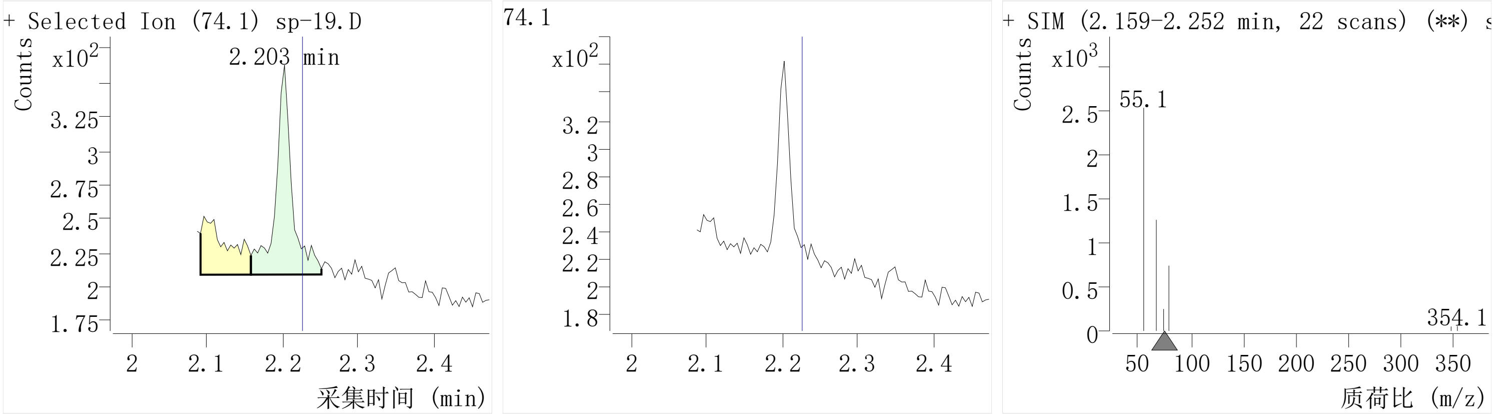

C6:0

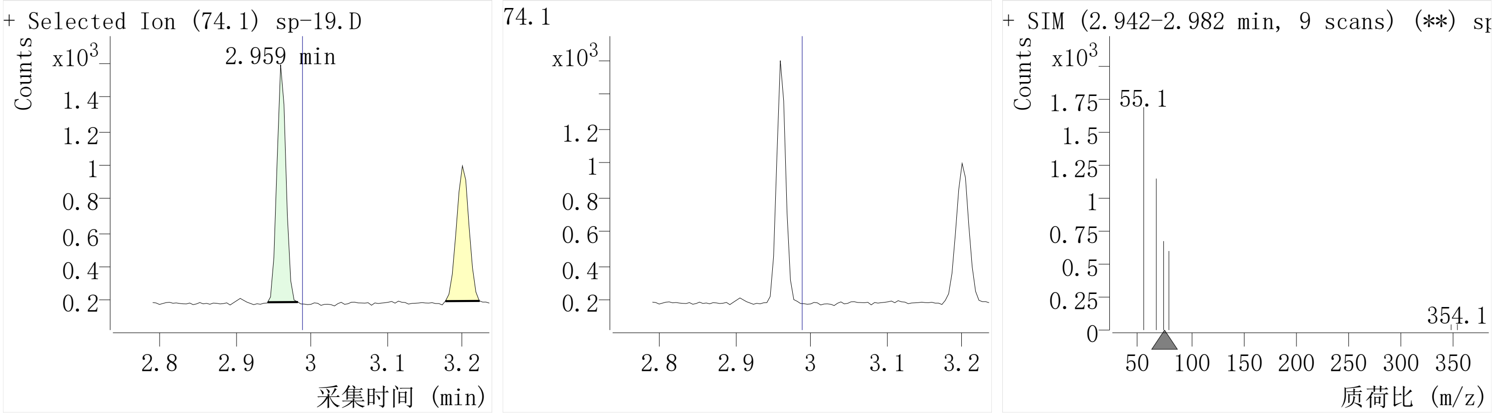

C8:0

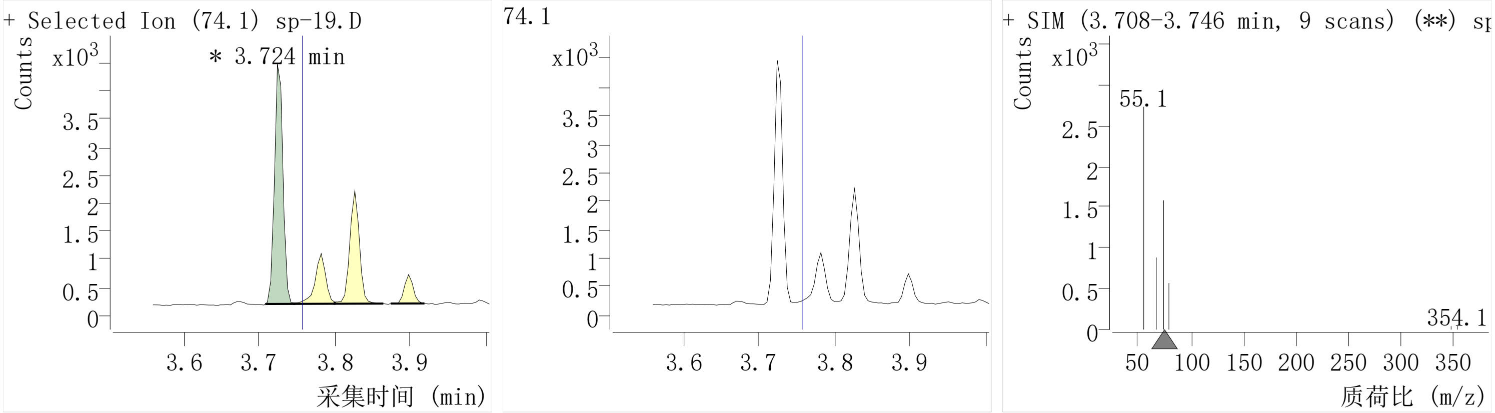

C10:0

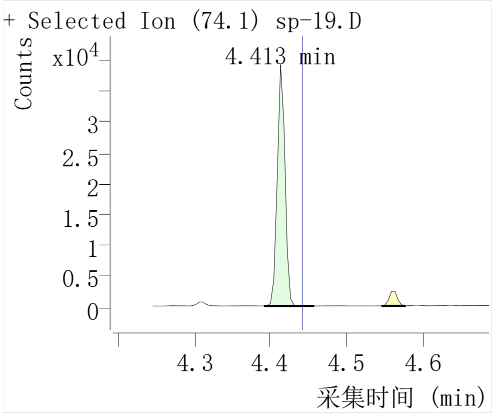

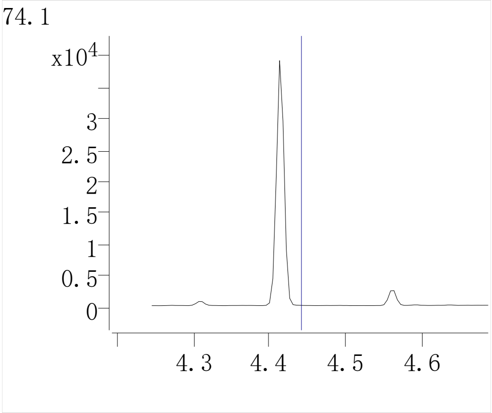

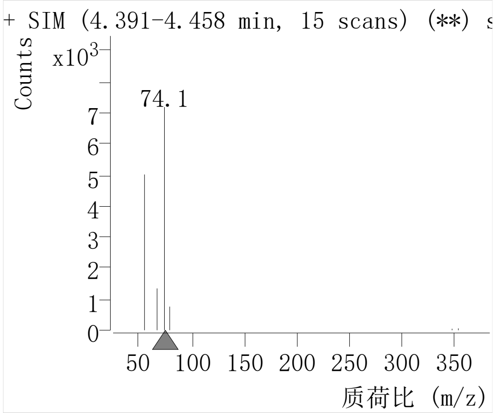

C11:0

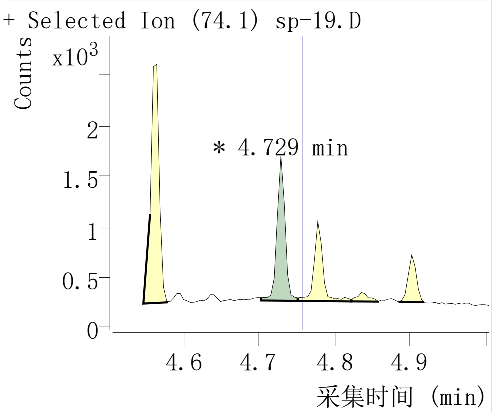

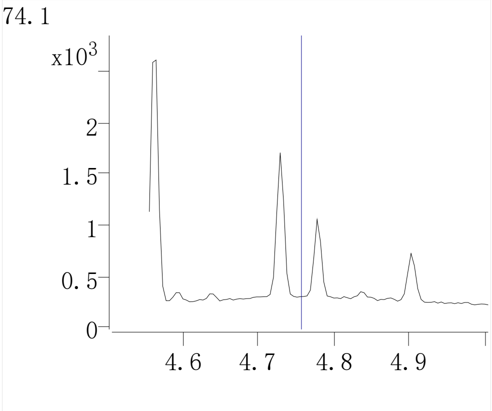

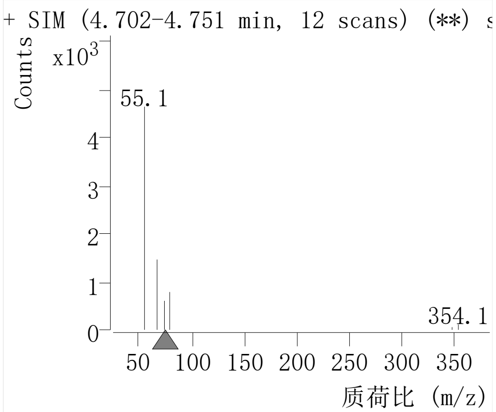

C12:0

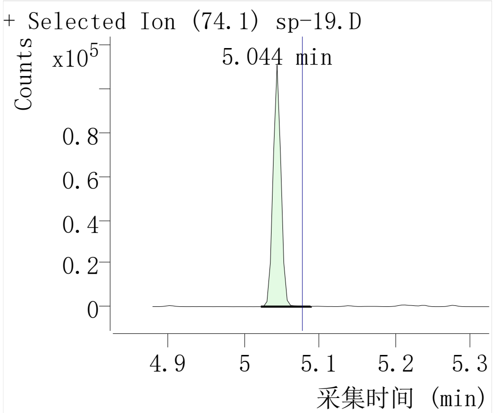

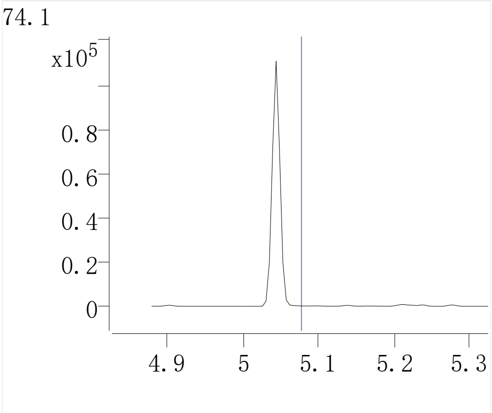

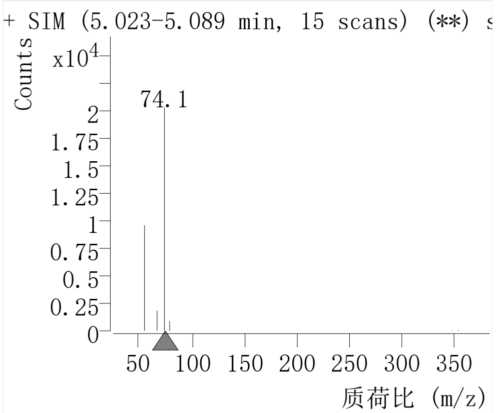

C13:0

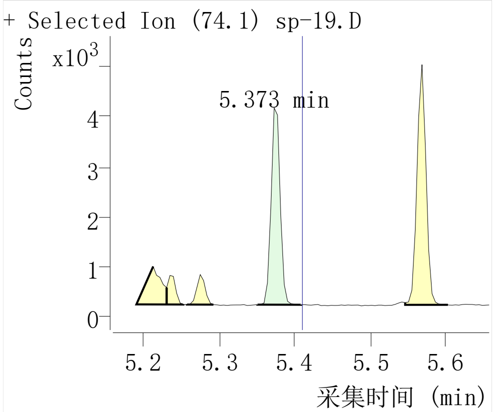

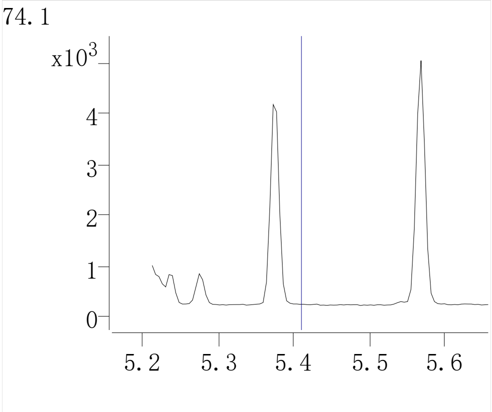

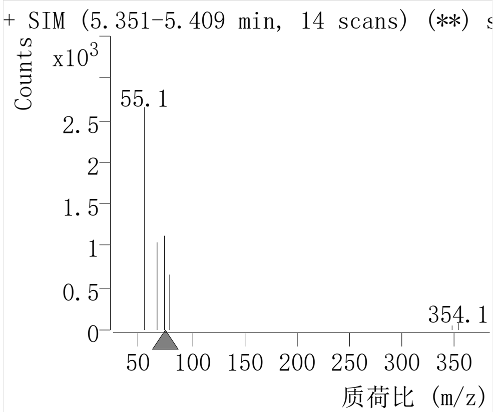

C14:0

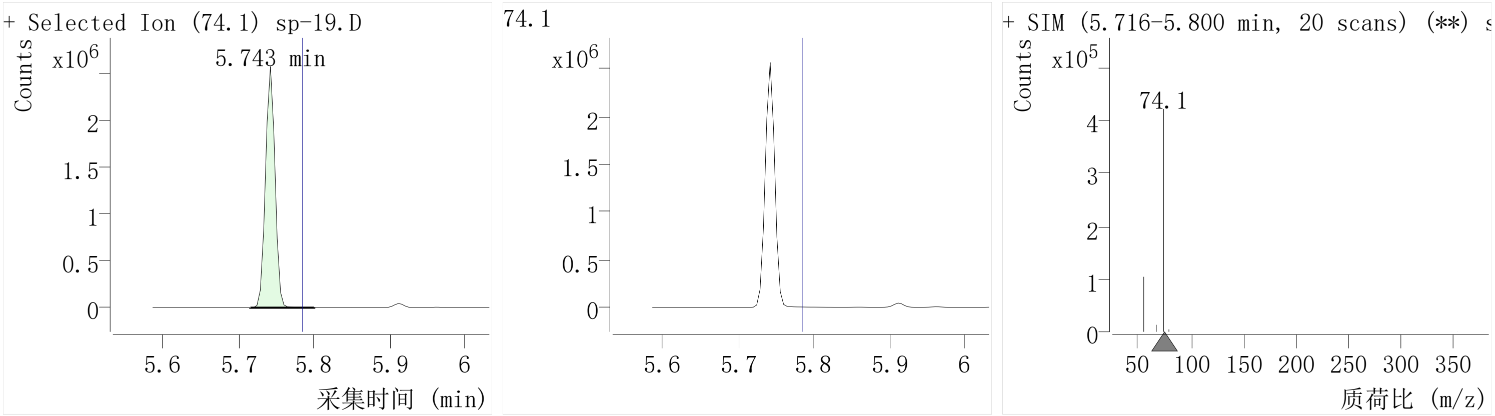

C14:1

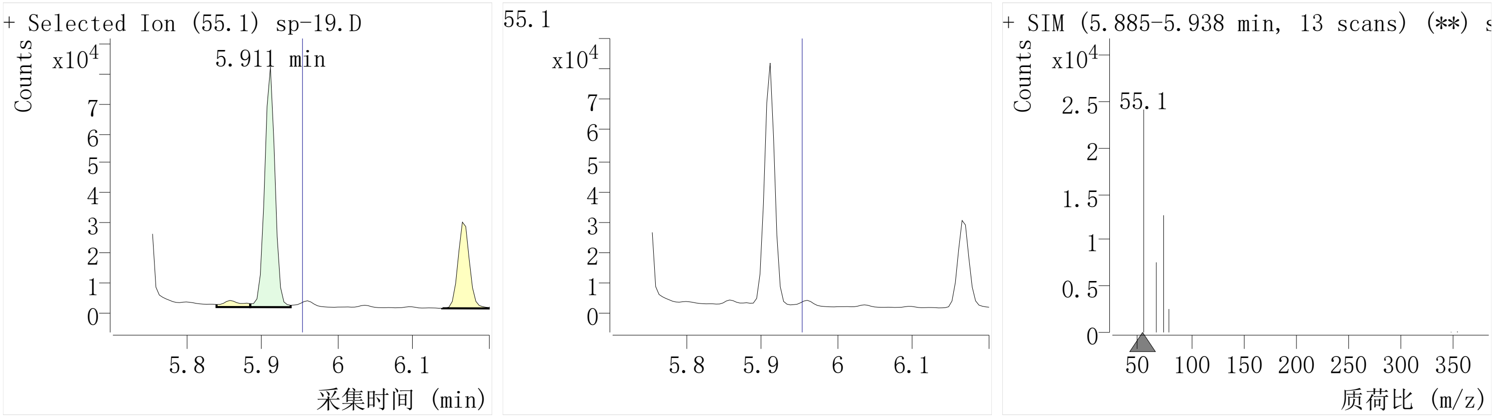

C15:0

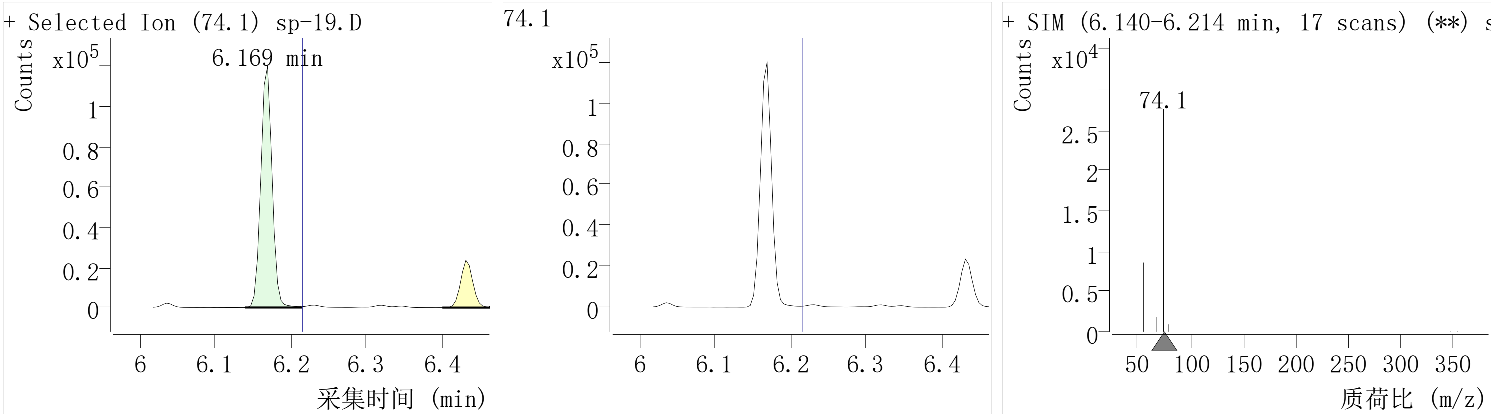

C15:1

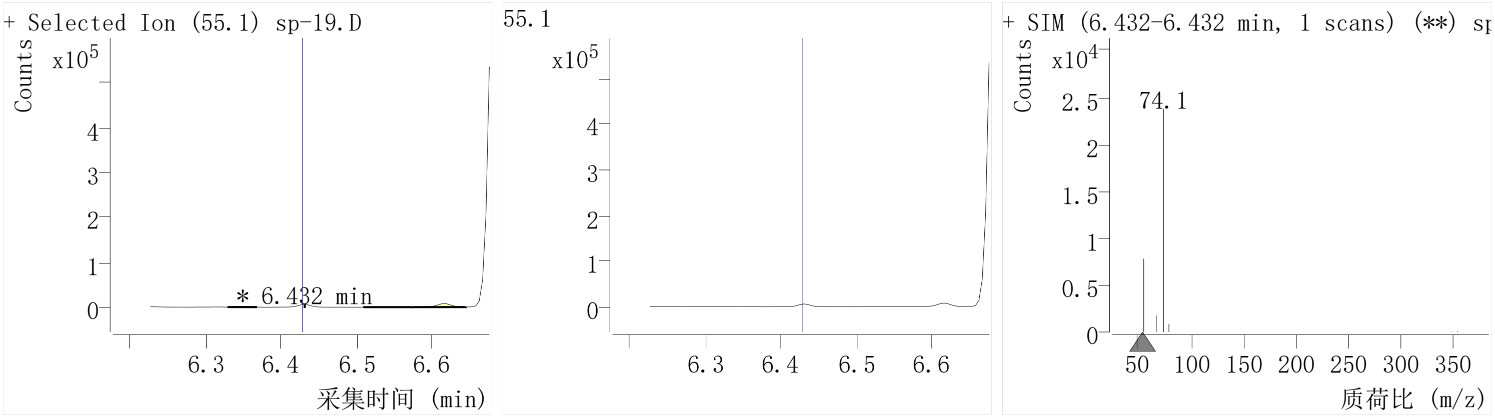

C16:0

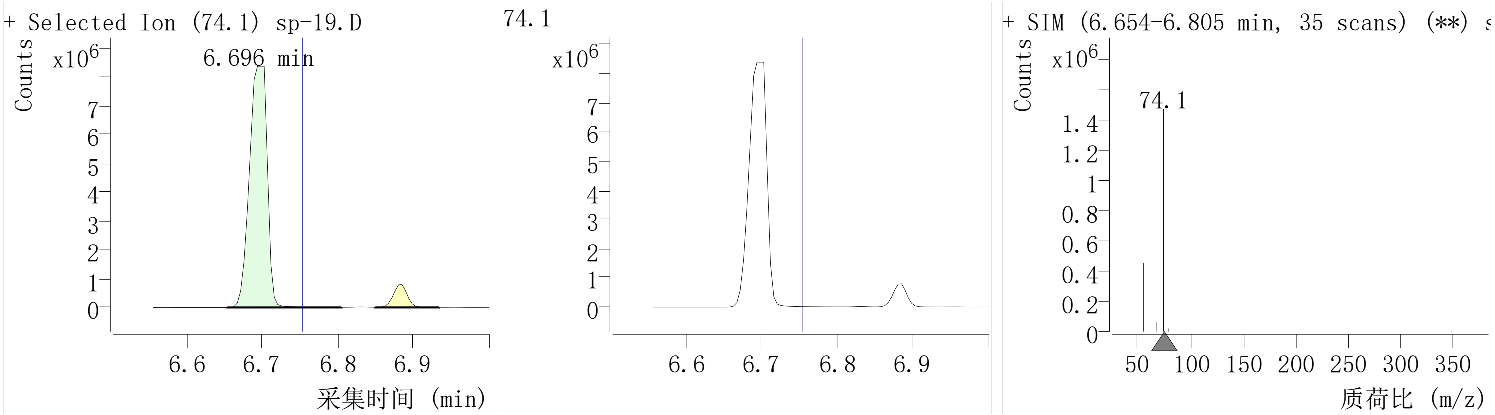

C16:1

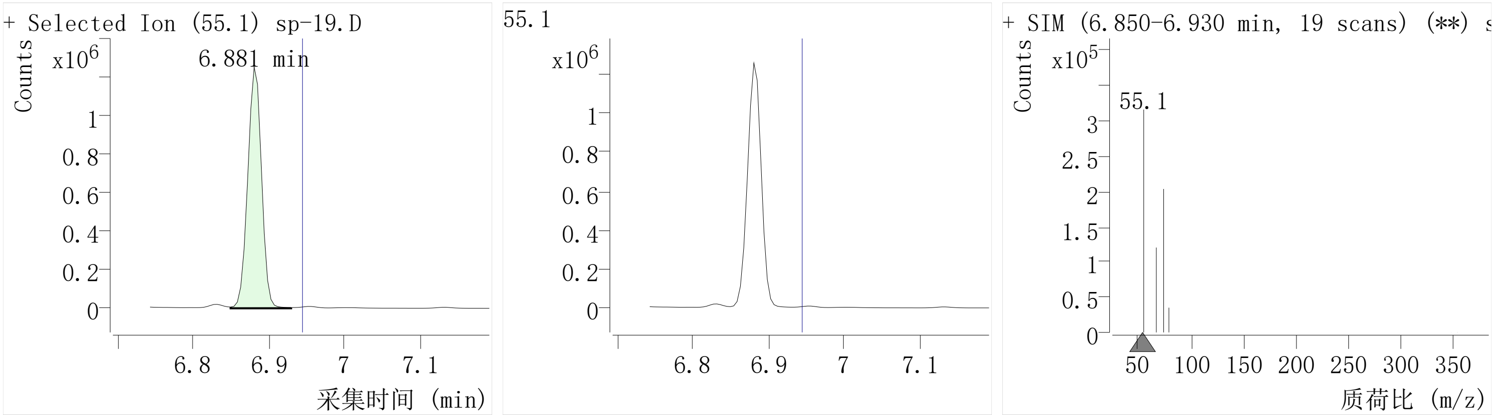

C17:0

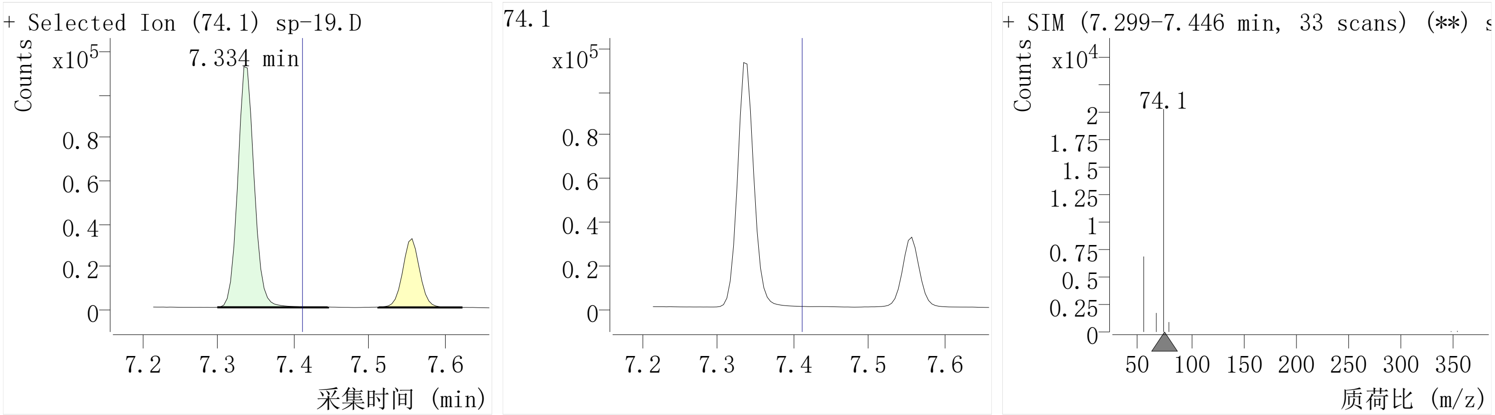

C17:1

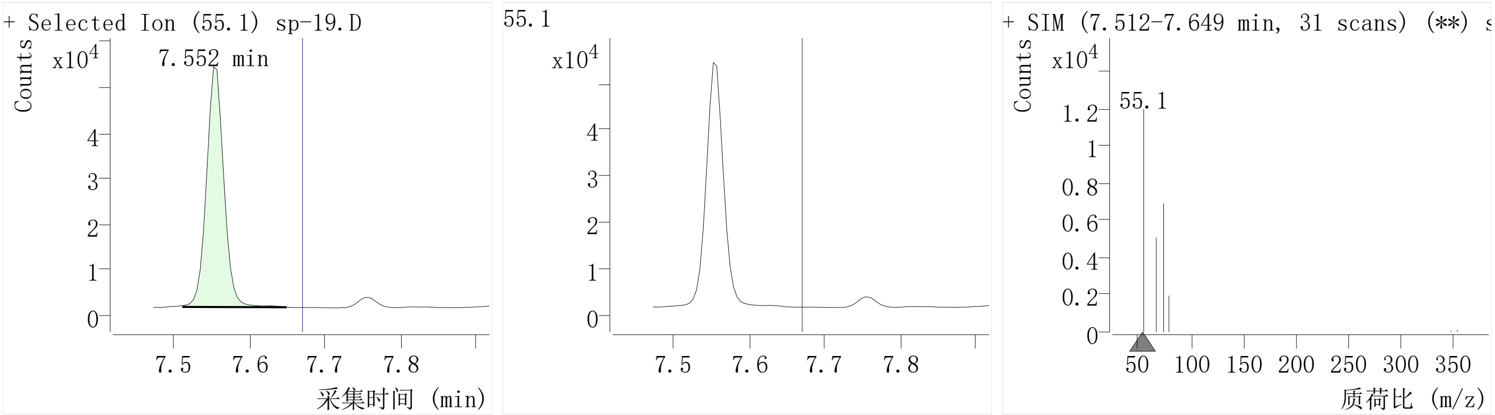

C18:0

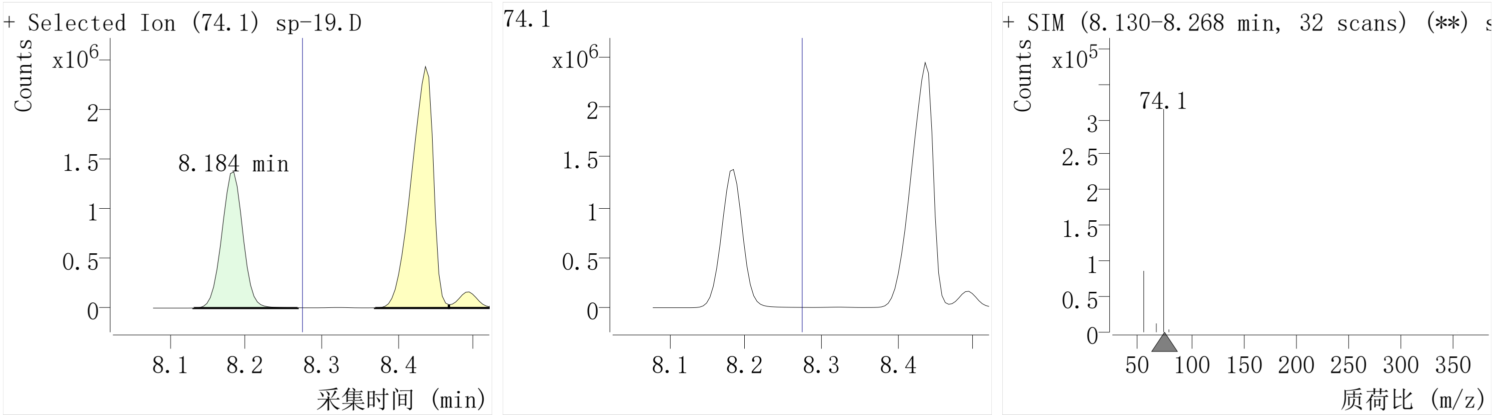

C18:1n9t

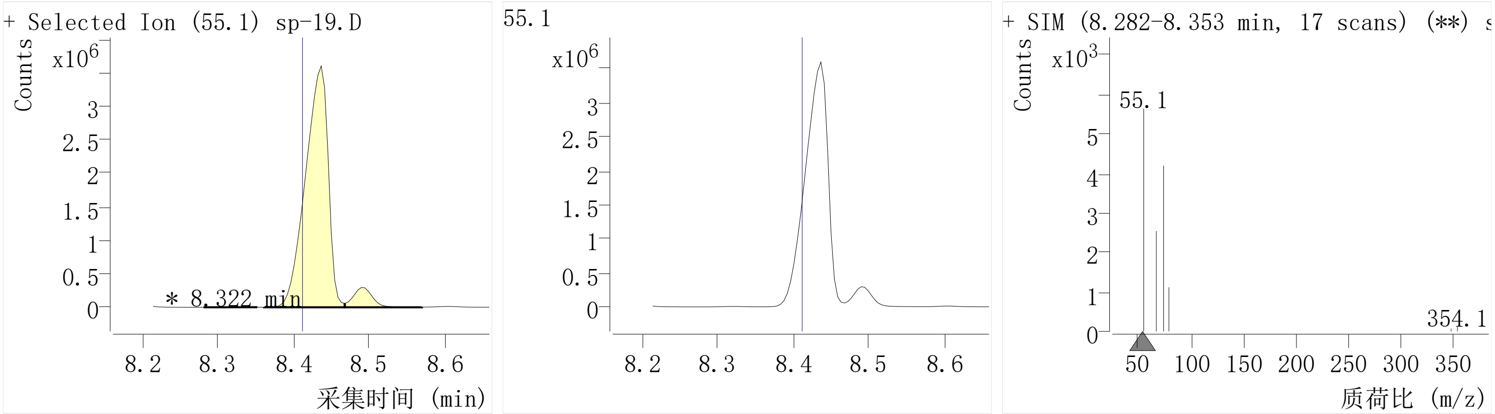

C18:1n9c

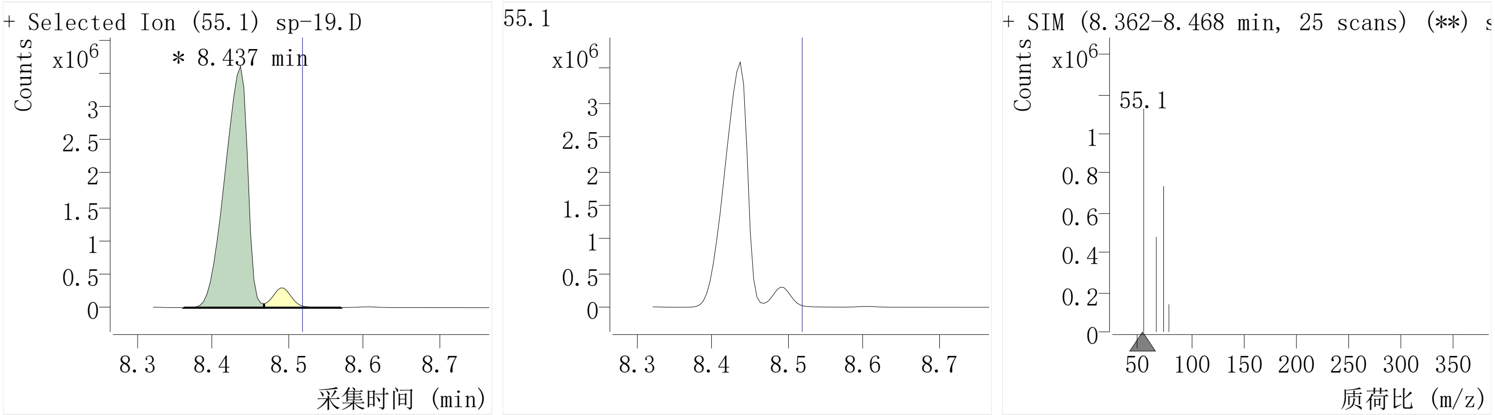

C18:2n6t

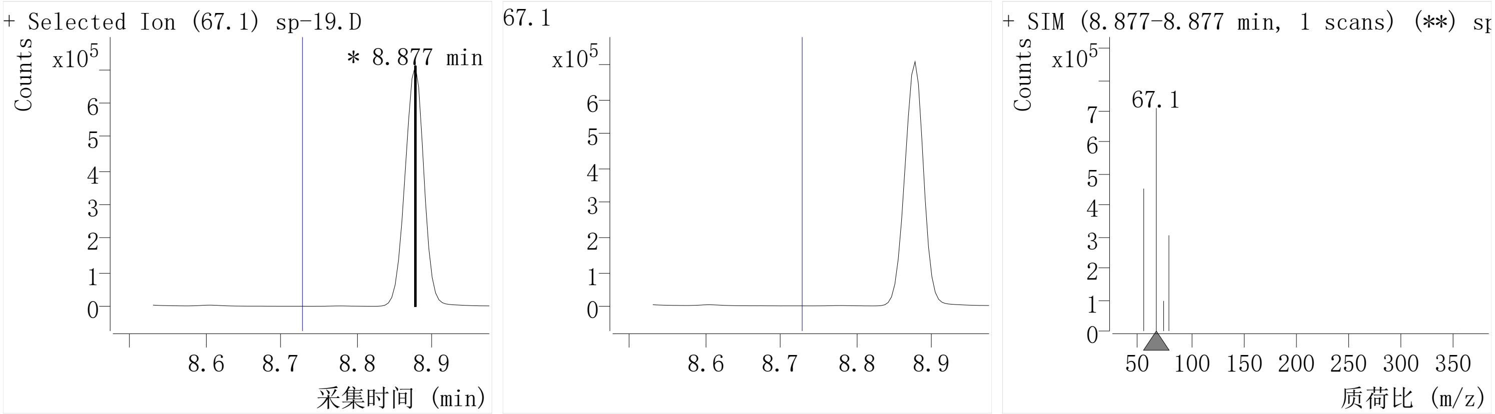

C18:2n6c

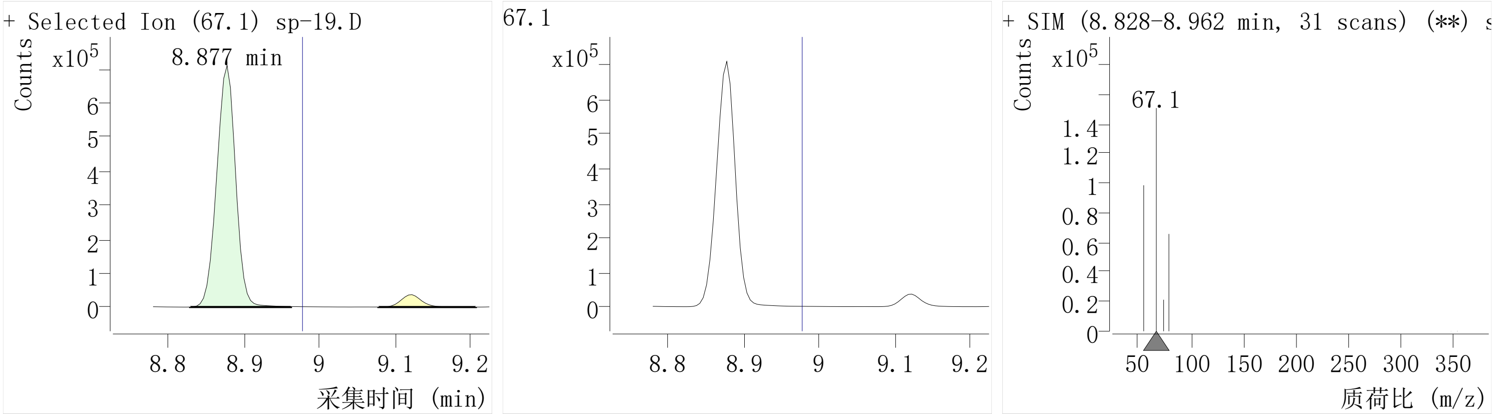

C18:3n6

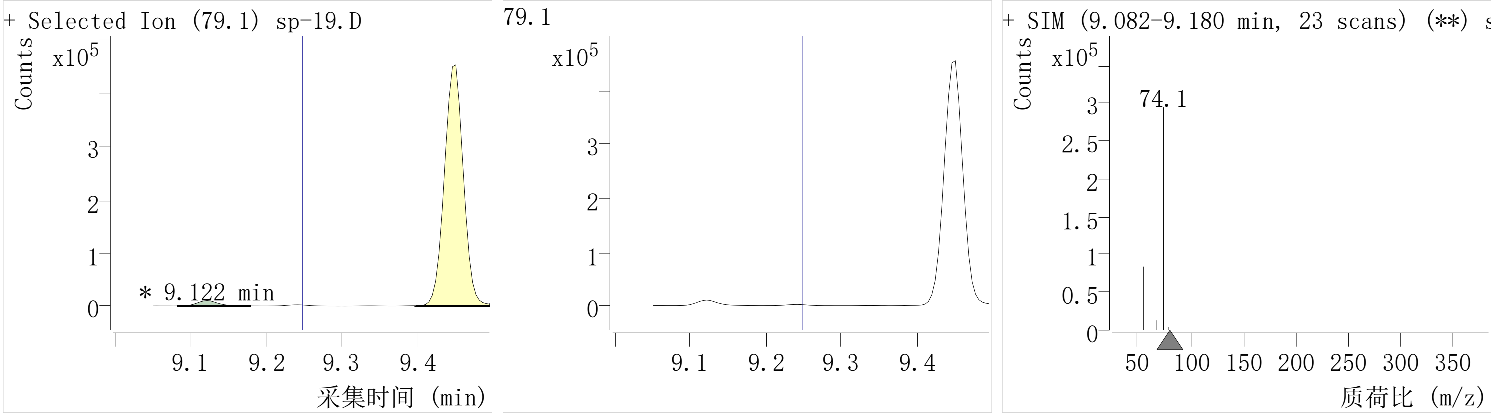

C18:3n3

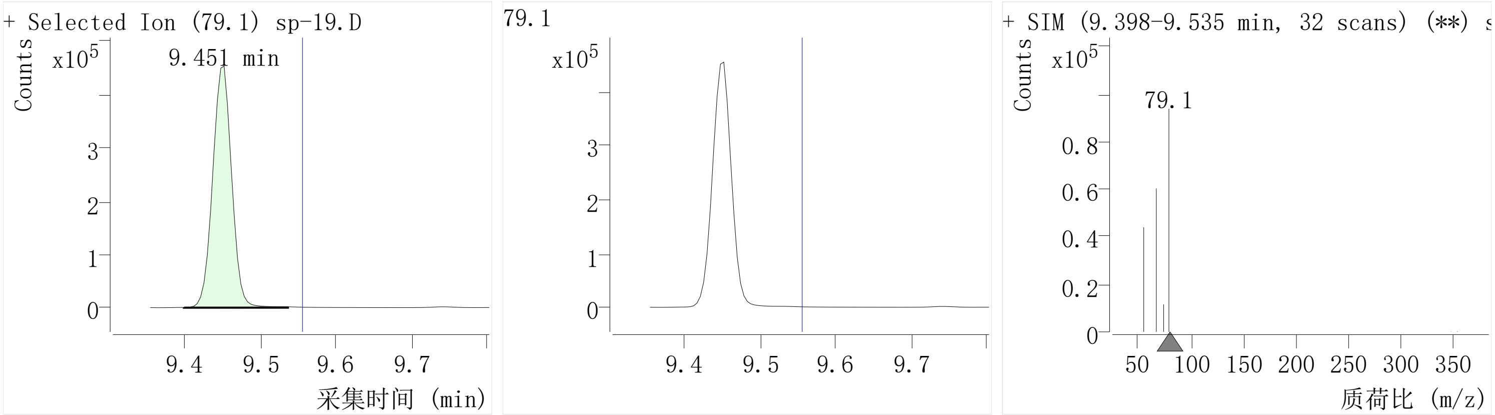

C20:0

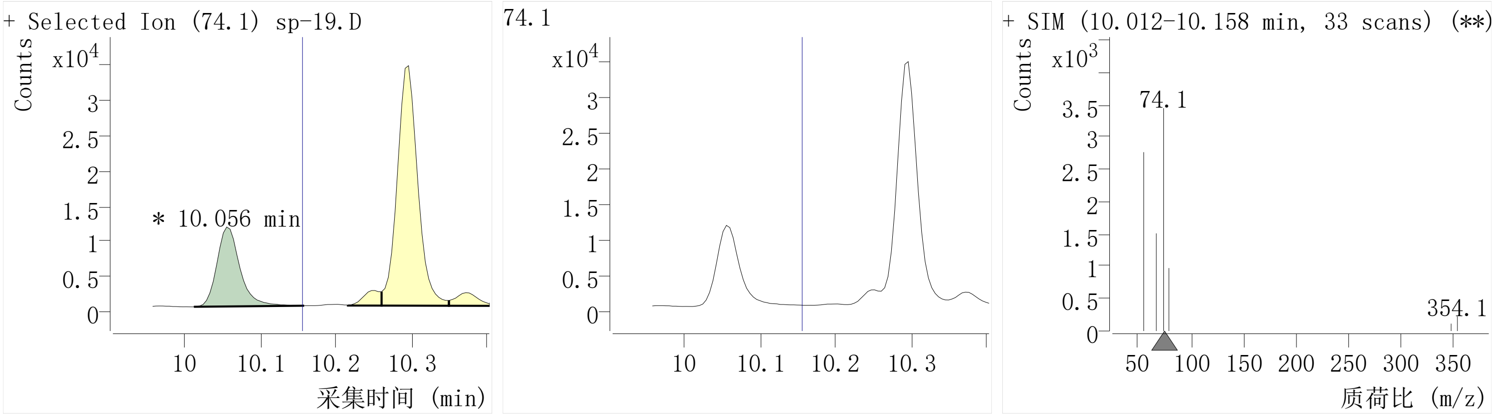

C20:1

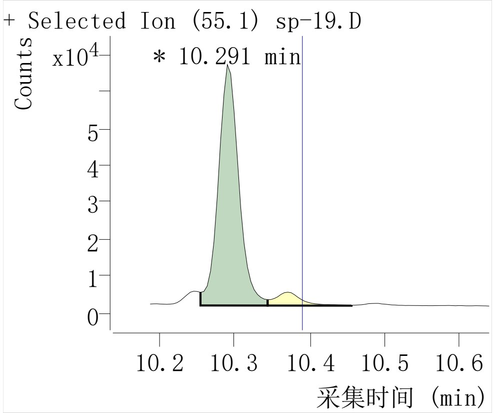

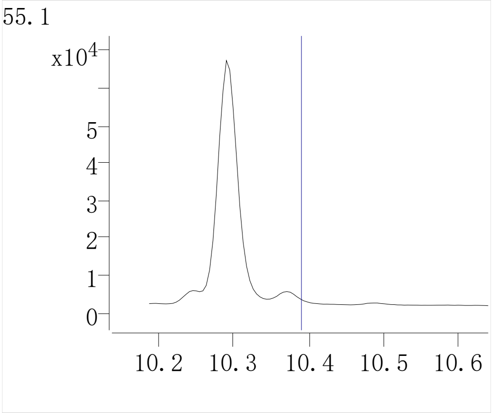

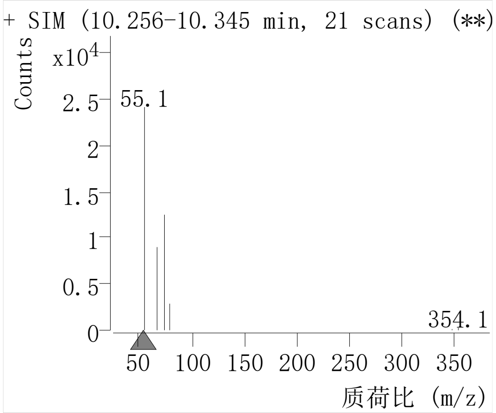

C20:2

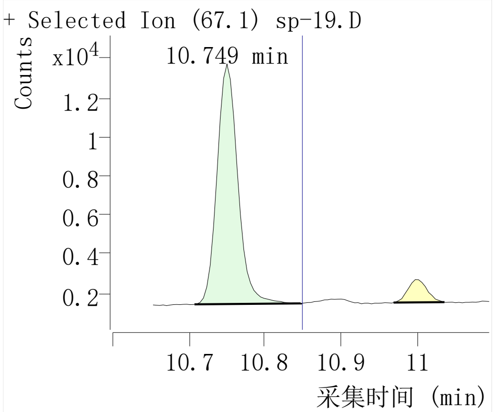

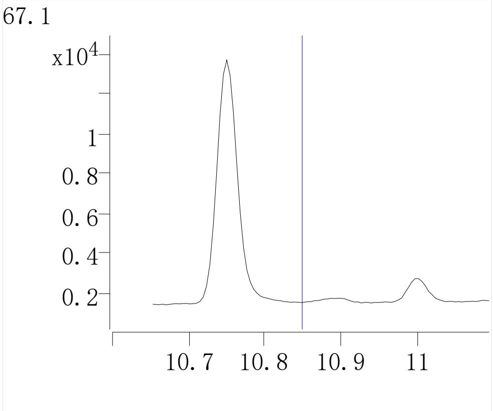

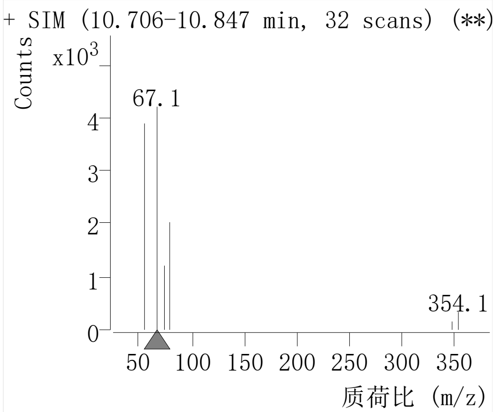

C21:0

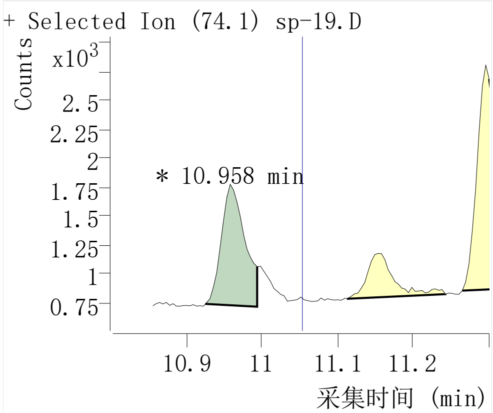

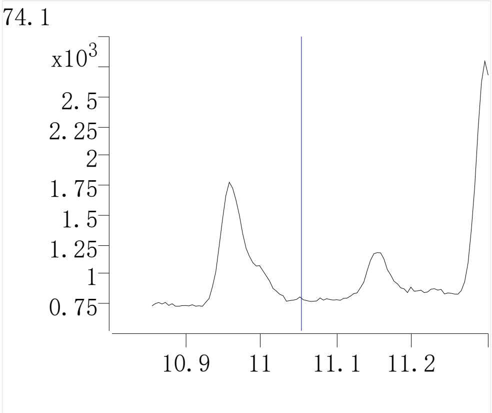

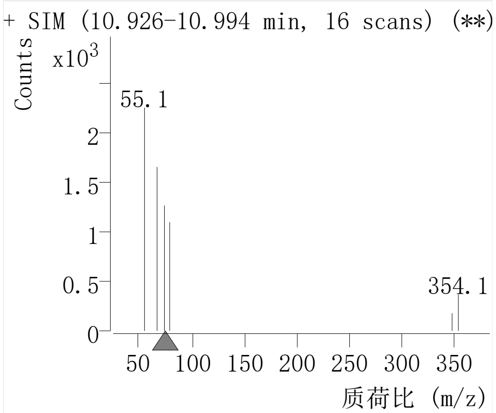

C20:3n6

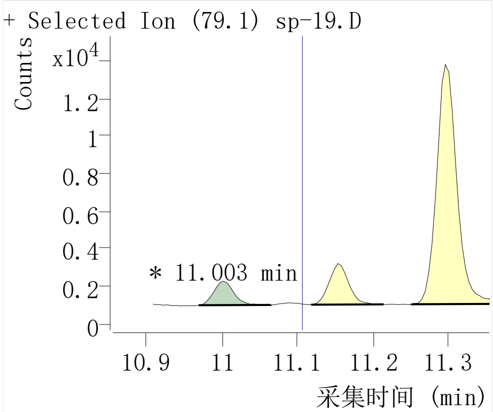

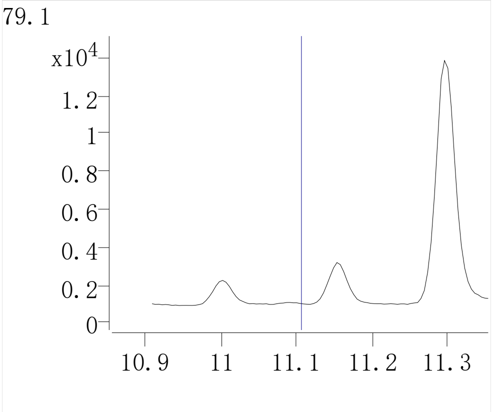

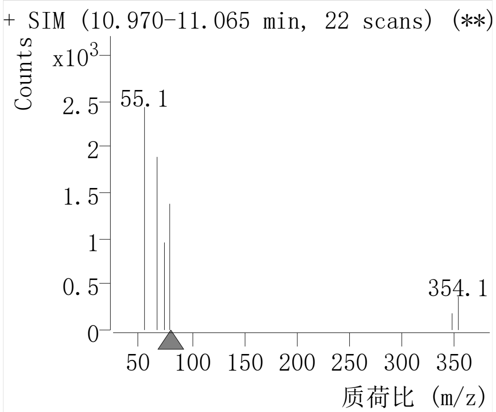

C20:4n6

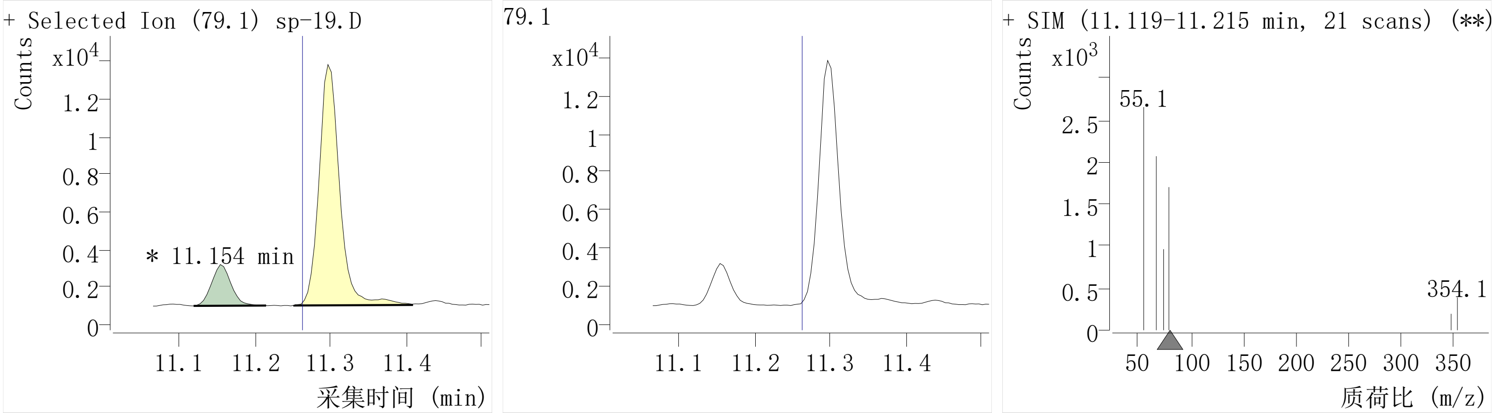

C20:3n3

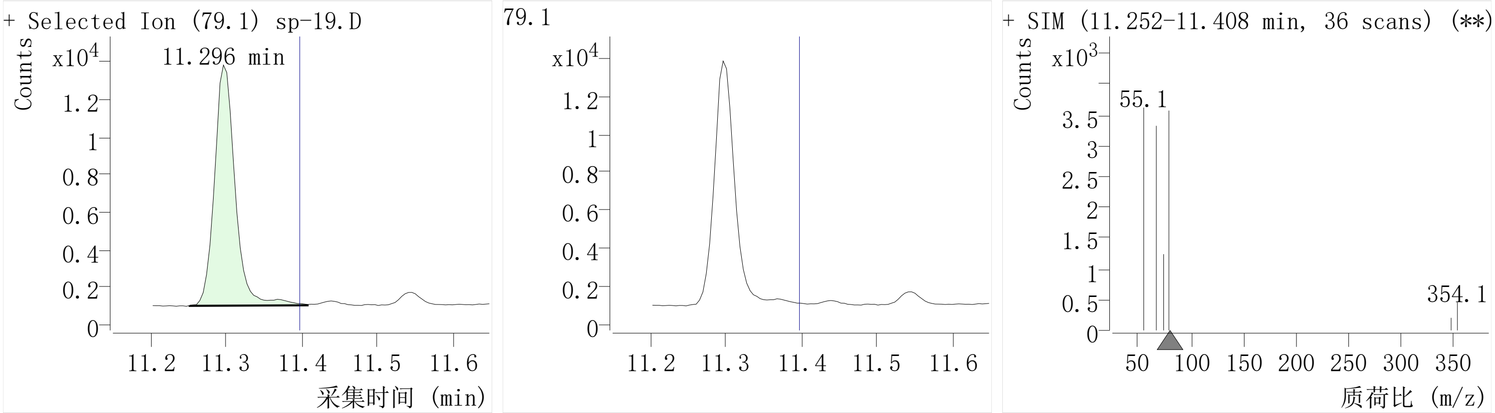

C20:5n3

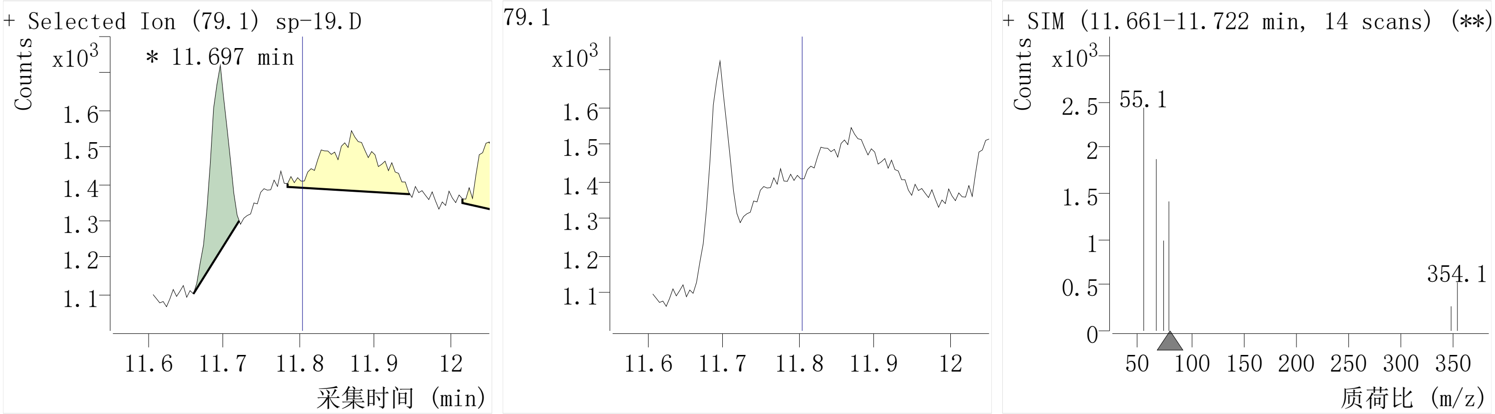

C22:0

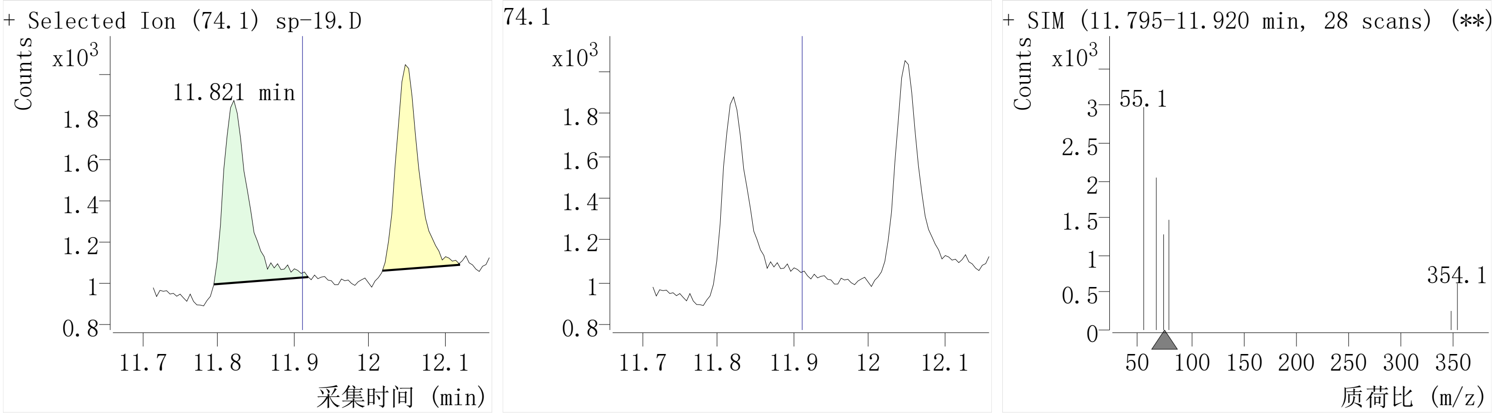

C22:1n9

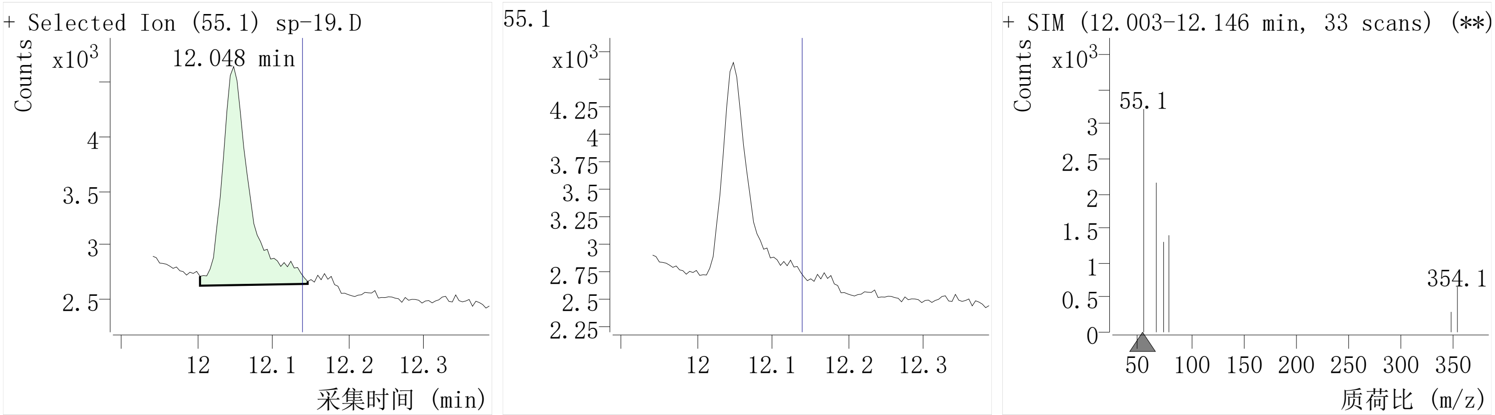

C22:2n6

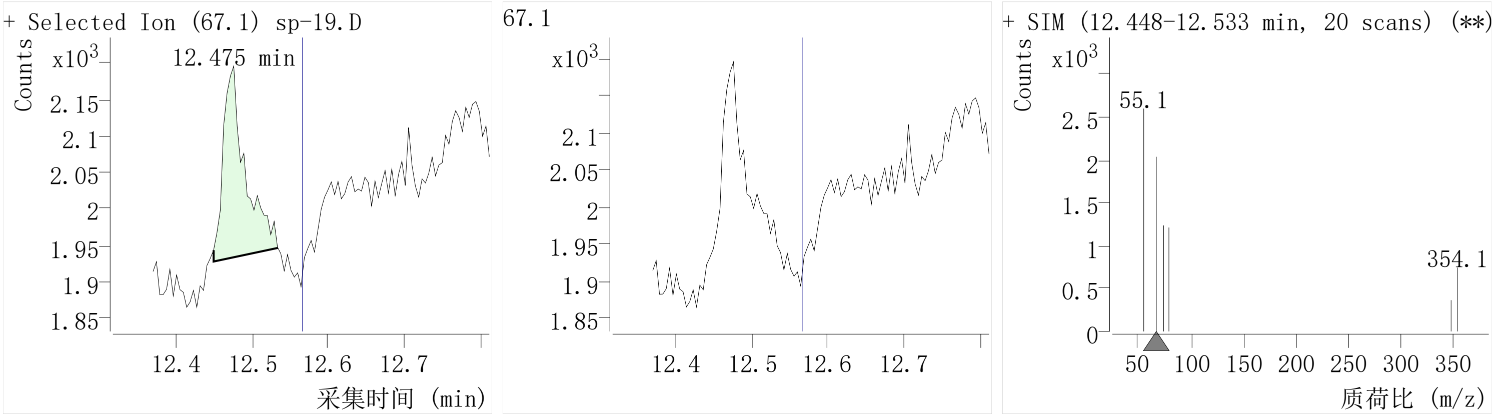

C23:0

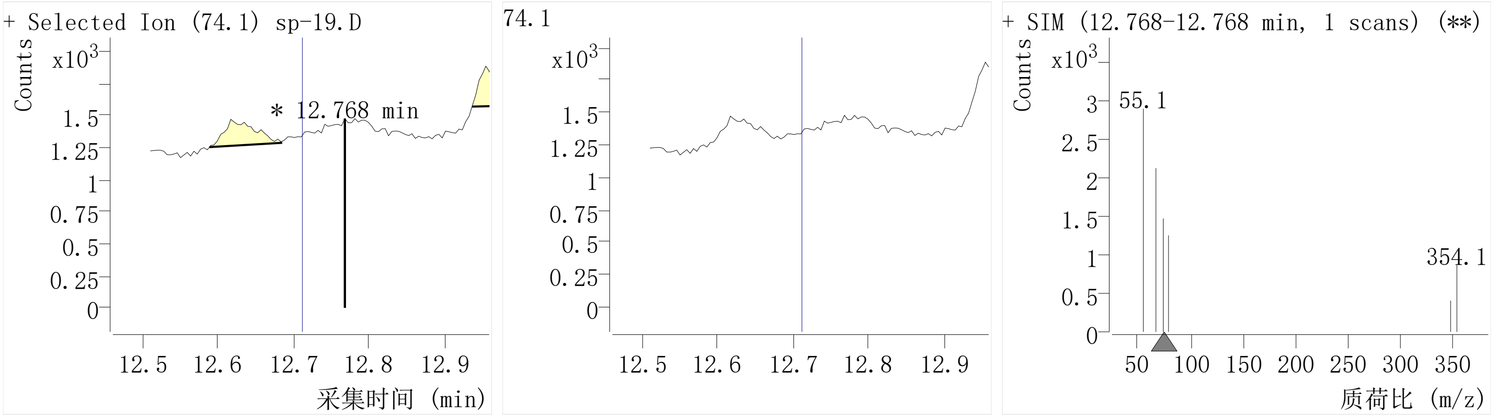

C24:0

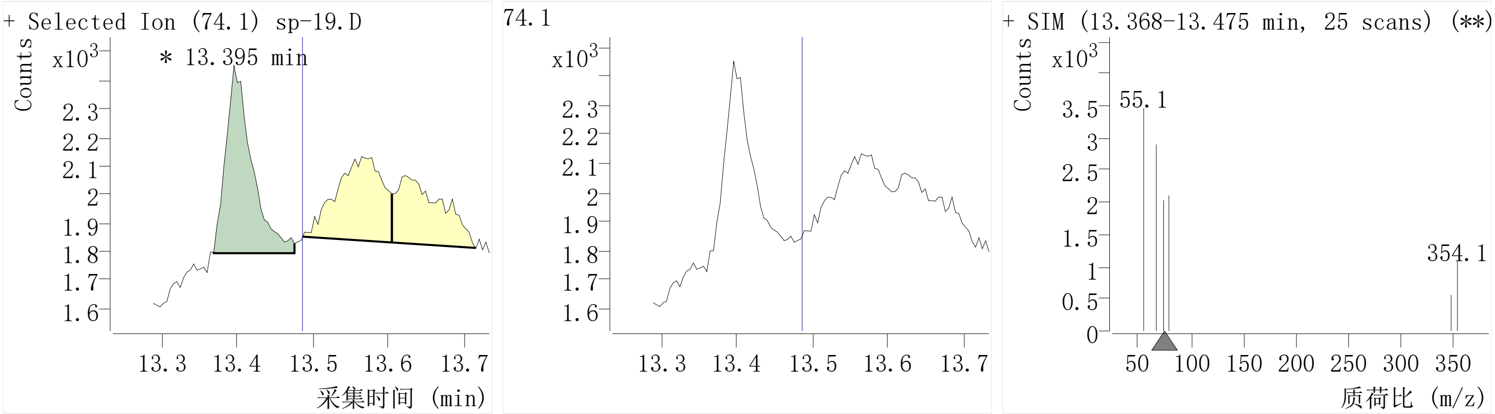

C22:6

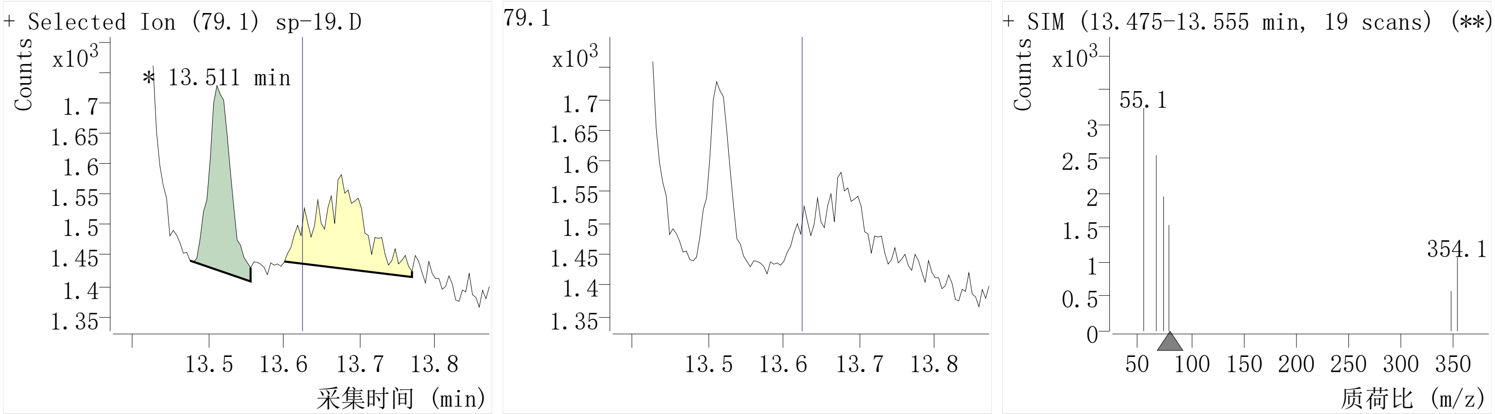
+ Selected Ion (79.1) sp-19.D
Counts
x10^3
* 13.511 min
1.7
1.65
1.6
1.55
1.5
1.45
1.4
1.35
13.5
13.6
13.7
13.8
采集时间 (min)
79.1
+ SIM (13.475-13.555 min, 19 scans) (**)
55.1
354.1
3
2.5
2
1.5
1
0.5
0
50
100
150
200
250
300
350
质荷比 (m/z)

C24:1

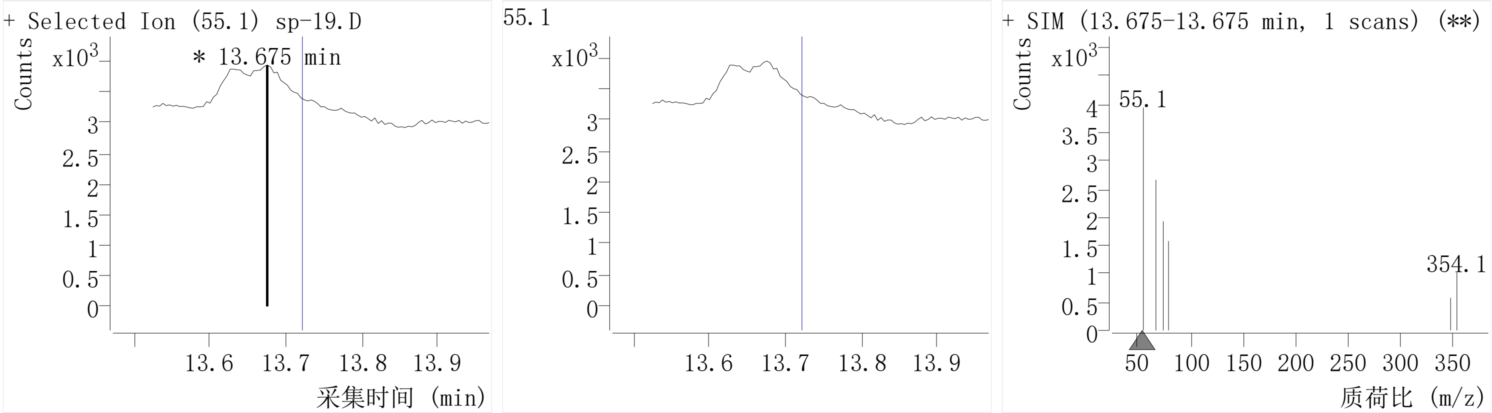
+ Selected Ion (55.1) sp-19.D
Counts
x10^3
* 13.675 min
3
2.5
2
1.5
1
0.5
0
13.6
13.7
13.8
13.9
采集时间 (min)
55.1
+ SIM (13.675-13.675 min, 1 scans) (**)
55.1
354.1
4
3.5
3
2.5
2
1.5
1
0.5
0
50
100
150
200
250
300
350
质荷比 (m/z)

**批处理路径**	G:\GC-MS\HX250430-4-GCMS总脂肪酸靶向检测\HX250430-4\QuantResults\HX250430-4.batch.bin		
**分析时间**	2025/5/14 16:58	**分析员姓名**	DESKTOP-M3A0GPO\omics
**报告时间**	2025/5/16 14:53:19	**报告员姓名**	DESKTOP-M3A0GPO\omics
**最近校正更新**	2025/5/14 16:58	**批处理状态**	已处理
**定量批处理版本**	10.2	**定量报告版本**	10.2

**采集时间**	2025/5/9 4:55	**数据文件**	sp-20.D
**样品类型**	样品	**样品名称**	sp-20
**稀释**	1	**采集方法**	脂肪酸

样品色谱图

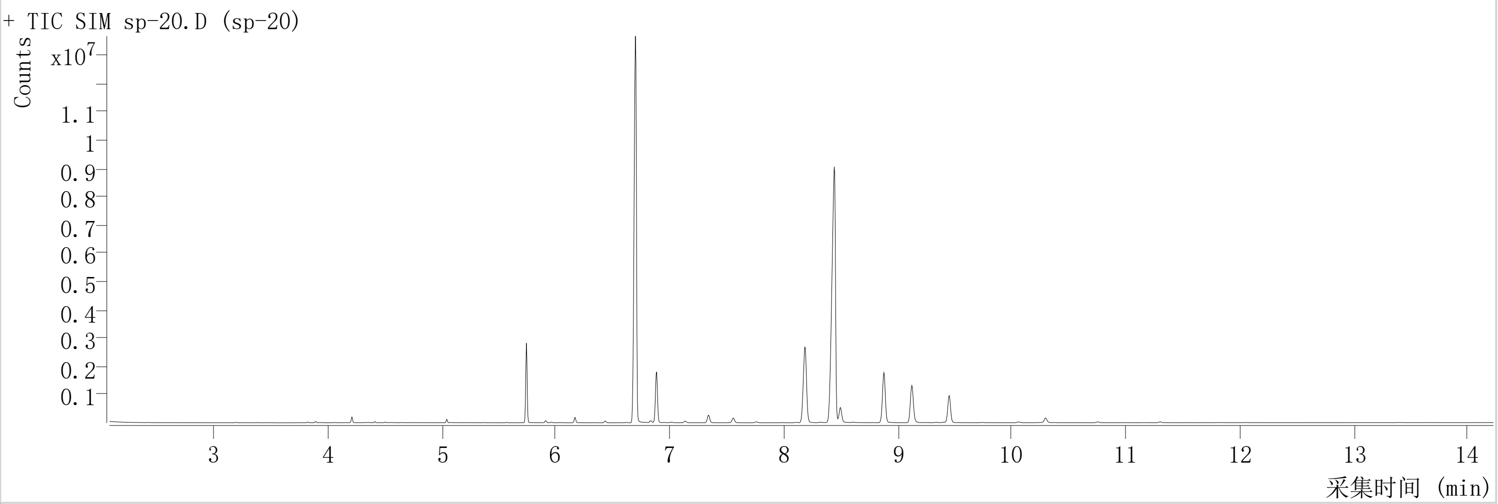

化合物	ISTD	RT	响应	ISTD 响应	响应比	最终浓度	单位
C4:0	C19:0	2.198	89	1890097	0.0000	ND	ug/ml
C6:0	C19:0	2.959	704	1890097	0.0004	0.0040	ug/ml
C8:0	C19:0	3.724	2295	1890097	0.0012	0.0081	ug/ml
C10:0	C19:0	4.413	24166	1890097	0.0128	0.1868	ug/ml
C11:0	C19:0	4.729	1366	1890097	0.0007	0.0059	ug/ml
C12:0	C19:0	5.044	70897	1890097	0.0375	0.5412	ug/ml
C13:0	C19:0	5.378	4424	1890097	0.0023	0.0240	ug/ml
C14:0	C19:0	5.742	1931585	1890097	1.0220	21.2868	ug/ml
C14:1	C19:0	5.911	38598	1890097	0.0204	0.9232	ug/ml
C15:0	C19:0	6.169	151047	1890097	0.0799	1.3615	ug/ml
C15:1	C19:0	6.432	0	1890097	0.0000	ND	ug/ml
C16:0	C19:0	6.697	13864612	1890097	7.3354	281.7729	ug/ml
C16:1	C19:0	6.881	1084691	1890097	0.5739	34.9822	ug/ml
C17:0	C19:0	7.339	310994	1890097	0.1645	3.4328	ug/ml
C17:1	C19:0	7.557	123984	1890097	0.0656	3.5040	ug/ml
C18:0	C19:0	8.184	4076298	1890097	2.1567	47.8365	ug/ml
C18:1n9t	C19:0	8.326	13116	1890097	0.0069	0.4093	ug/ml
C18:1n9c	C19:0	8.442	8925079	1890097	4.7220	322.5106	ug/ml
C18:2n6t	C19:0	8.877	0	1890097	0.0000	ND	ug/ml
C18:2n6c	C19:0	8.877	1408111	1890097	0.7450	46.1537	ug/ml
C18:3n6	C19:0	9.122	19410	1890097	0.0103	ND	ug/ml
C18:3n3	C19:0	9.451	766186	1890097	0.4054	20.1480	ug/ml
C20:0	C19:0	10.056	45475	1890097	0.0241	0.6409	ug/ml
C20:1	C19:0	10.296	161384	1890097	0.0854	5.3017	ug/ml
C20:2	C19:0	10.749	24920	1890097	0.0132	0.8605	ug/ml
C21:0	C19:0	10.963	2292	1890097	0.0012	0.0335	ug/ml
C20:3n6	C19:0	11.003	2046	1890097	0.0011	0.1126	ug/ml
C20:4n6	C19:0	11.158	3106	1890097	0.0016	0.1377	ug/ml
C20:3n3	C19:0	11.301	25166	1890097	0.0133	0.7471	ug/ml
C20:5n3	C19:0	11.696	703	1890097	0.0004	0.0558	ug/ml

化合物	ISTD	RT	响应	ISTD 响应	响应比	最终浓度	单位
C22:0	C19:0	11.821	5879	1890097	0.0031	0.1210	ug/ml
C22:1n9	C19:0	12.048	5521	1890097	0.0029	0.2015	ug/ml
C22:2n6	C19:0	12.475	650	1890097	0.0003	0.0641	ug/ml
C23:0	C19:0	12.630	0	1890097	0.0000	ND	ug/ml
C24:0	C19:0	13.400	3028	1890097	0.0016	0.0786	ug/ml
C22:6	C19:0	13.515	489	1890097	0.0003	0.0243	ug/ml
C24:1	C19:0	13.680	0	1890097	0.0000	ND	ug/ml

C4:0

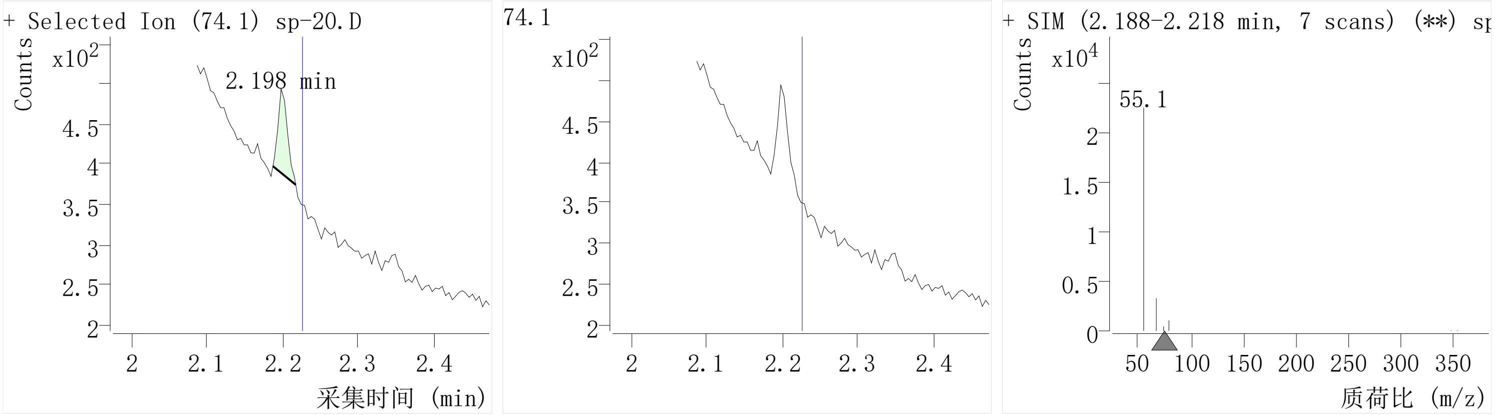

C6:0

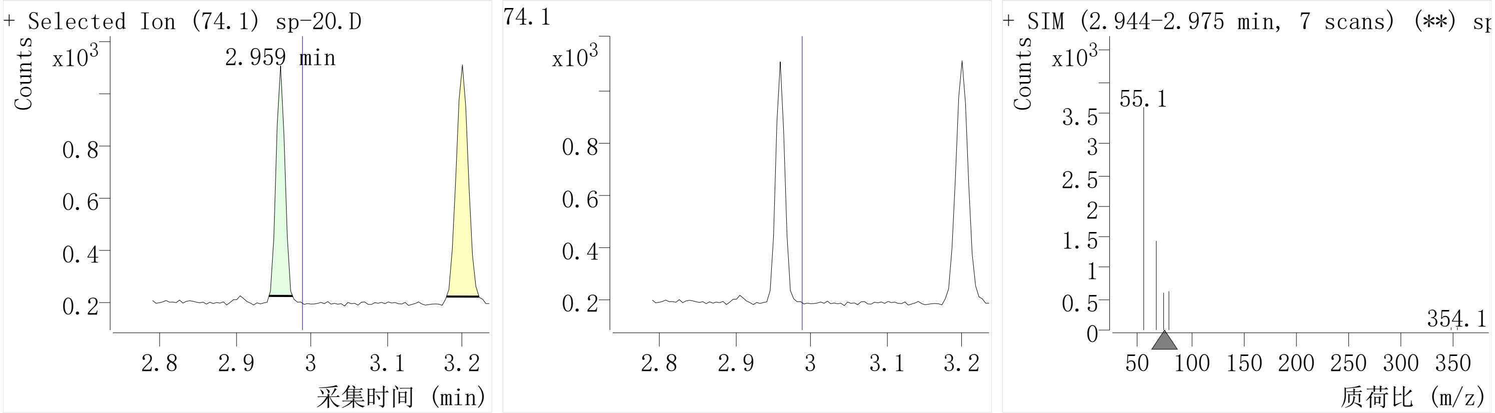

C8:0

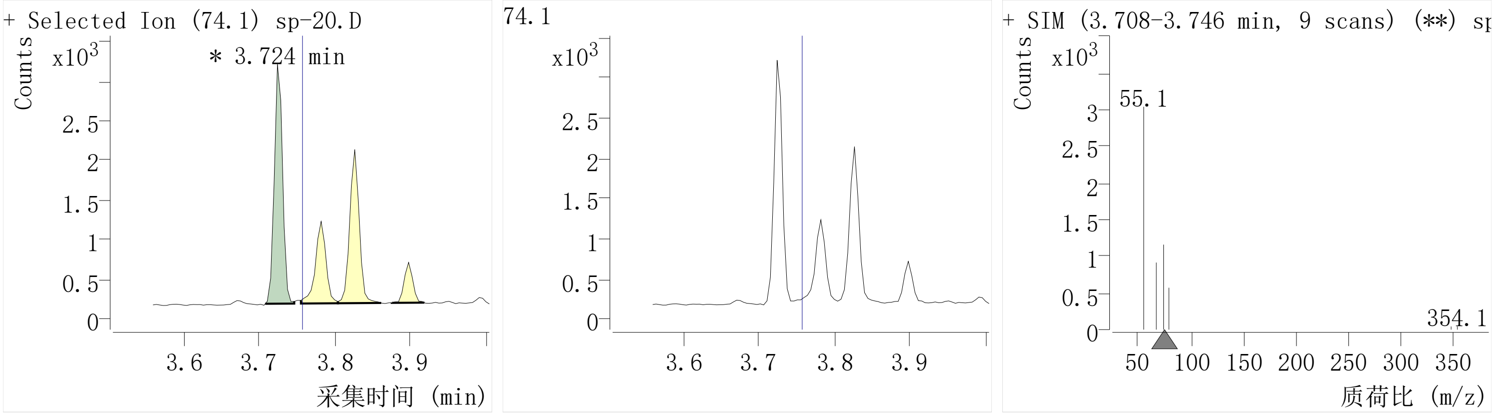

C10:0

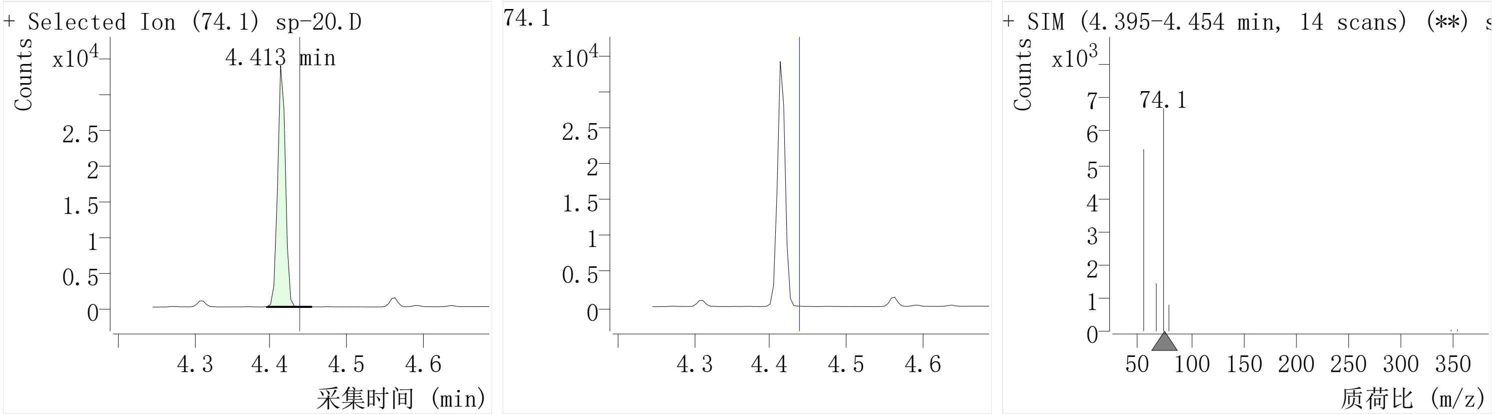

C11:0

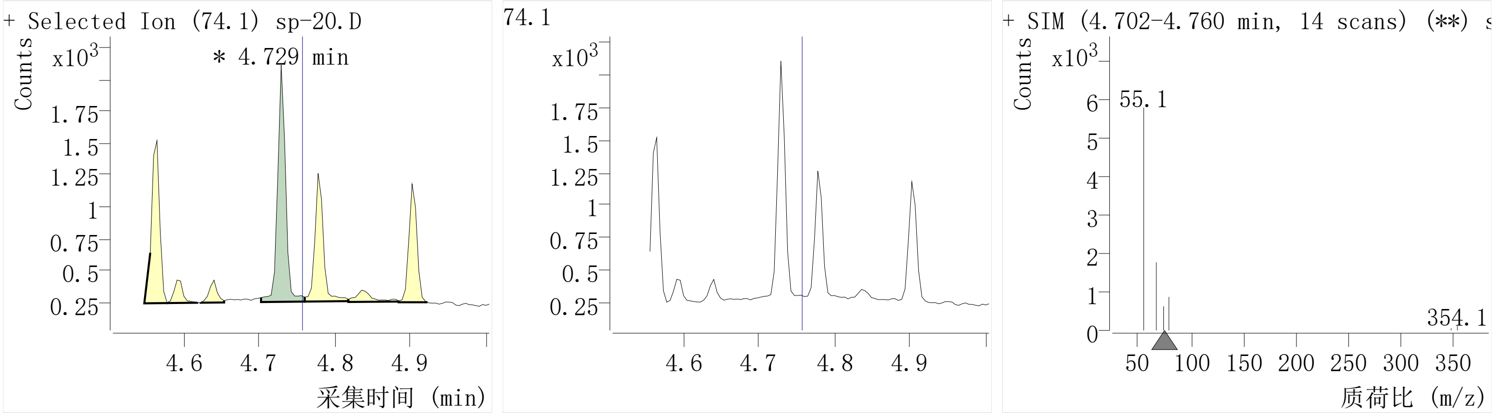

C12:0

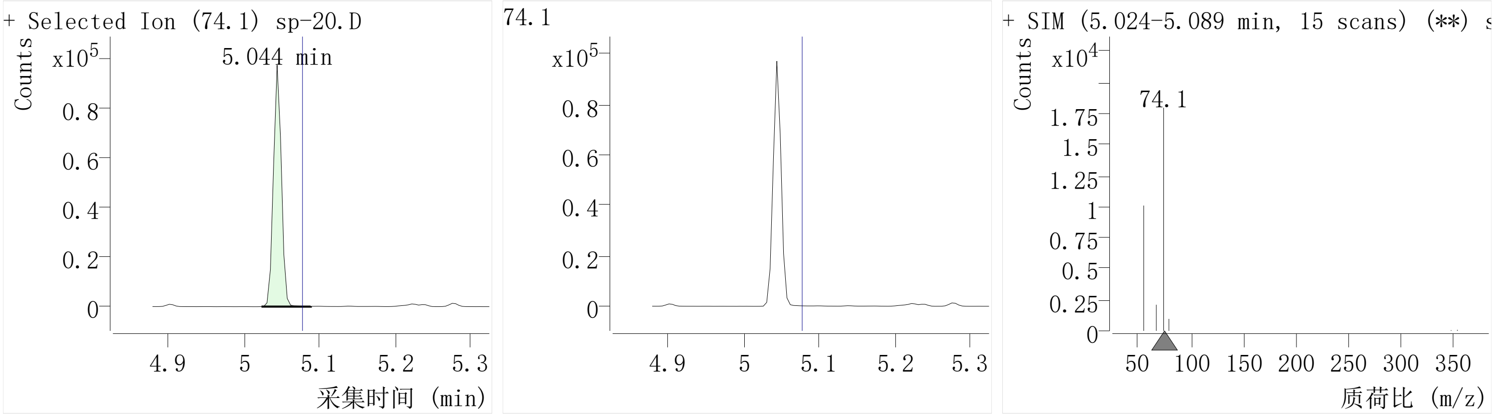

C13:0

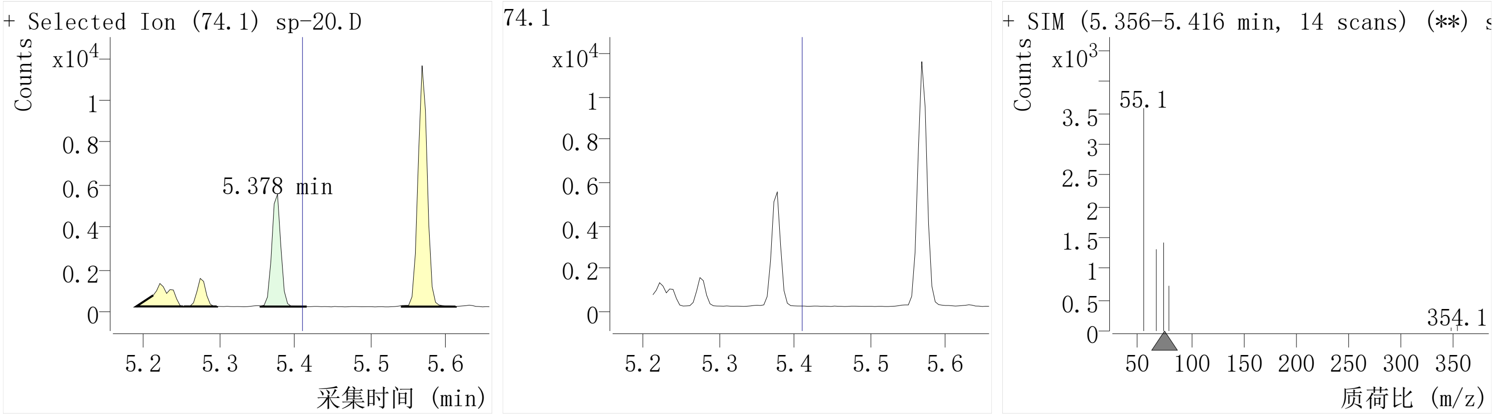

C14:0

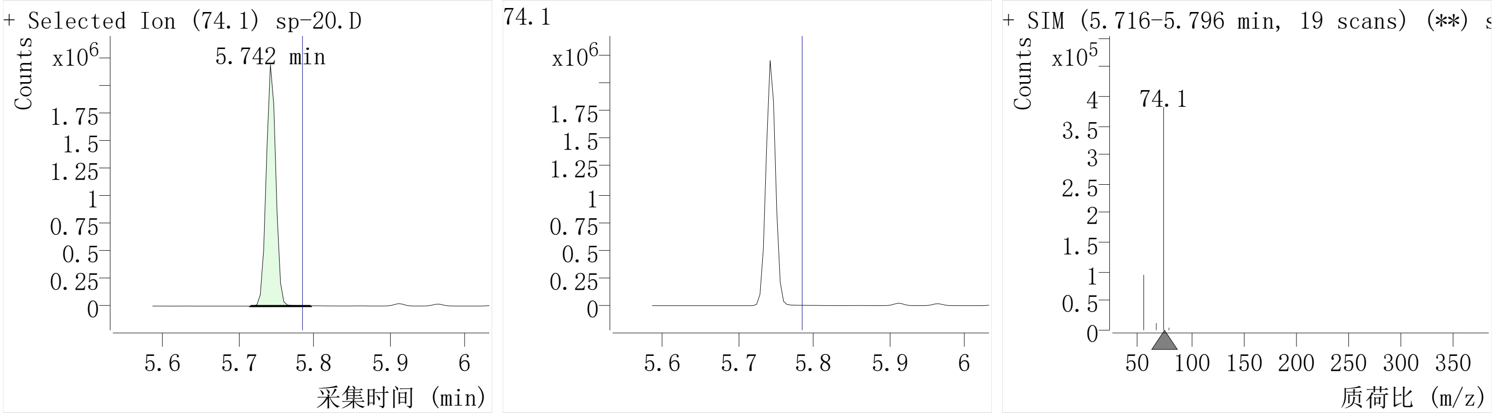

C14:1

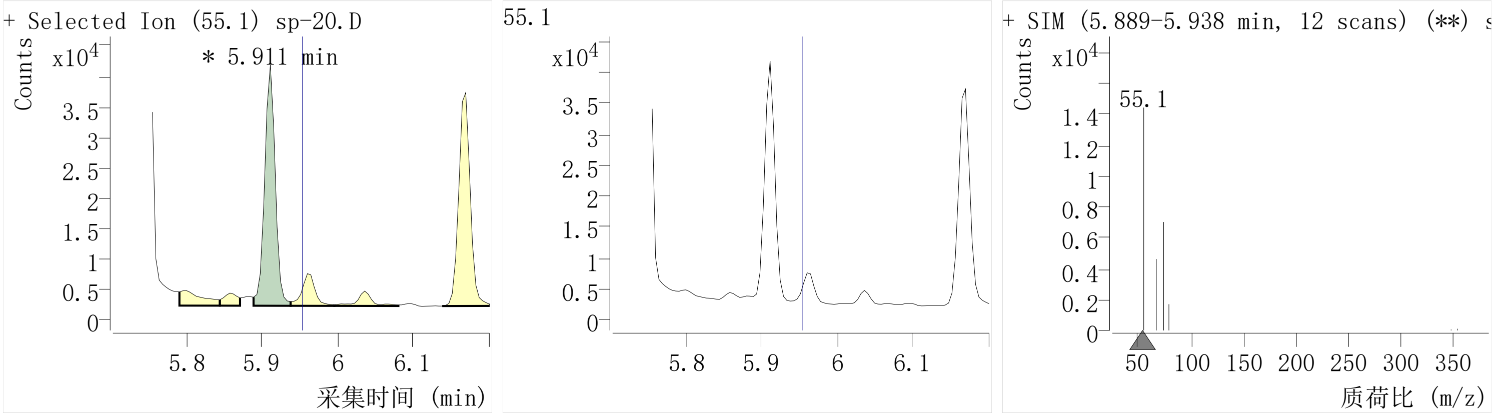

C15:0

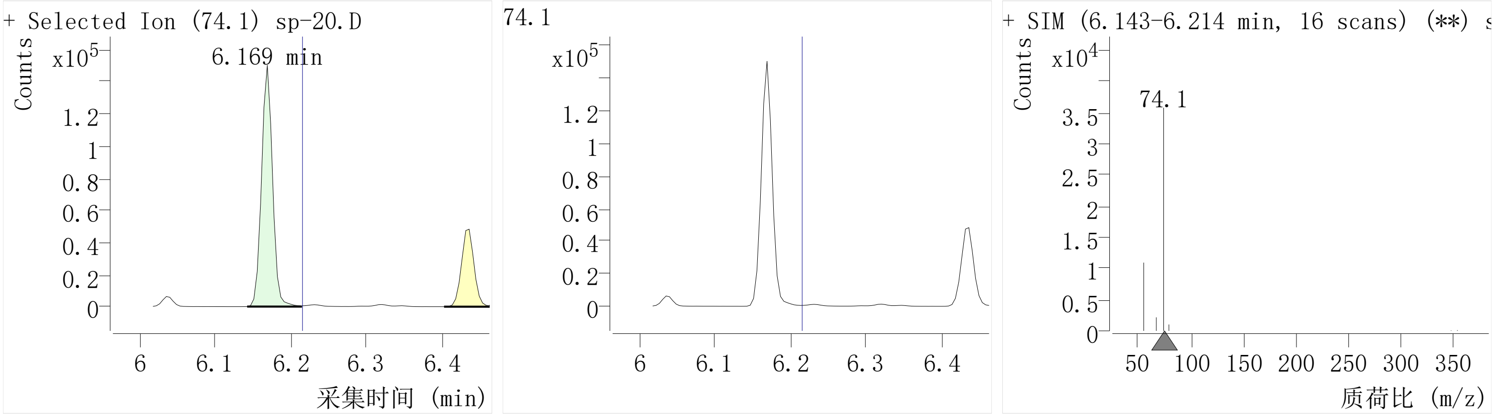

C15:1

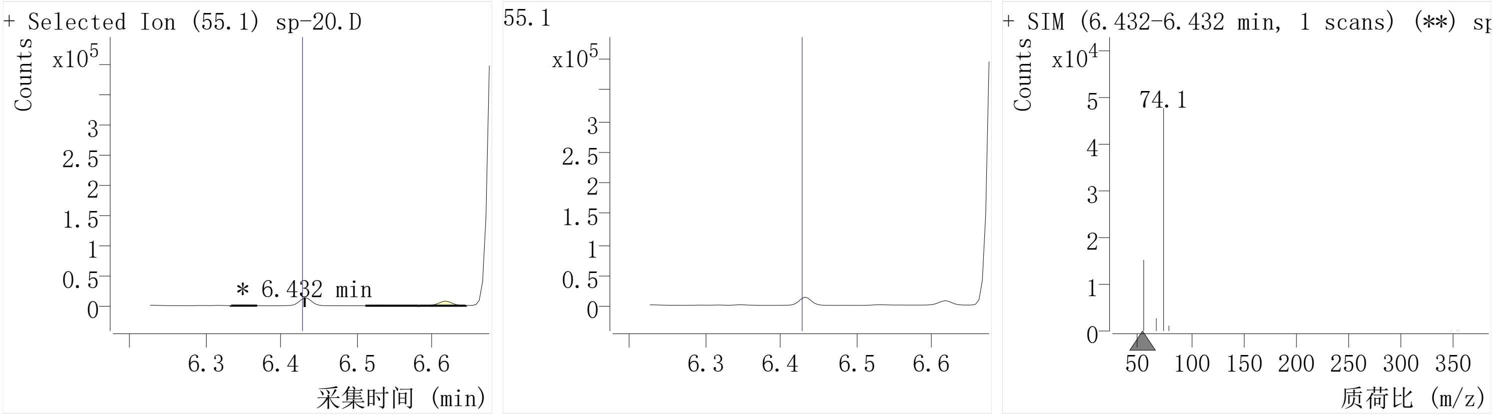

C16:0

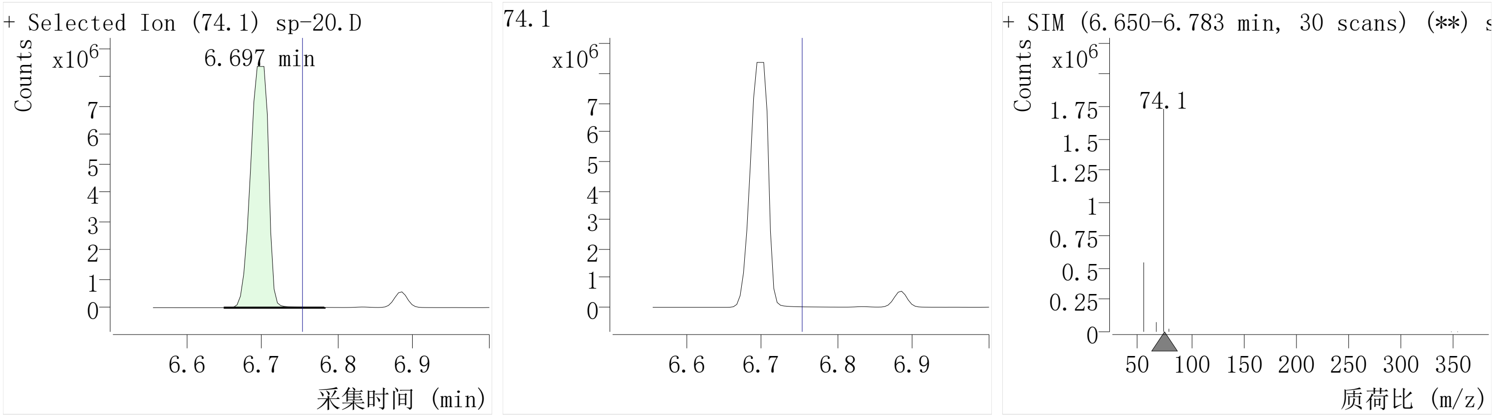

C16:1

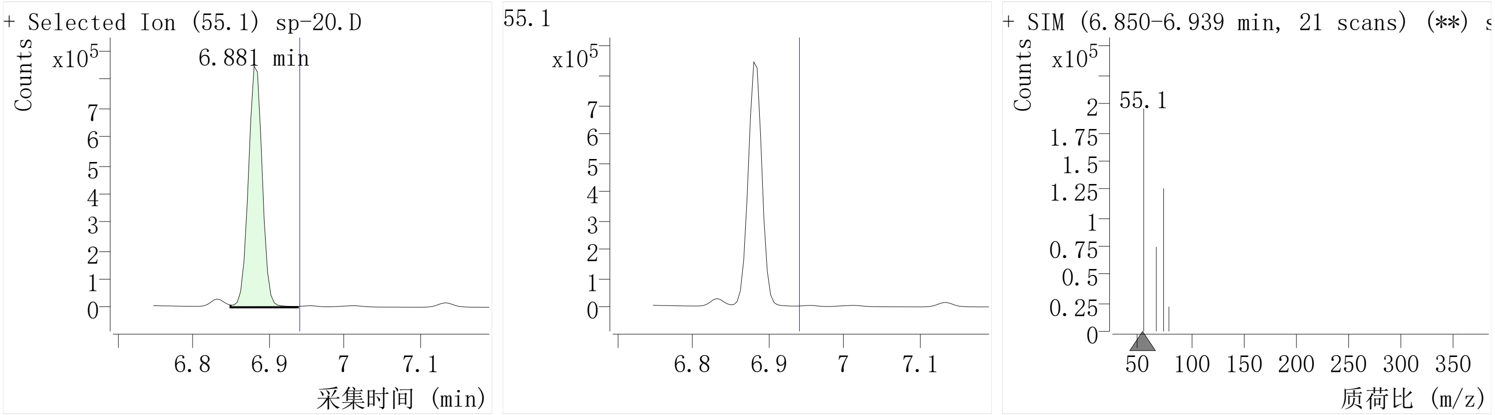

C17:0

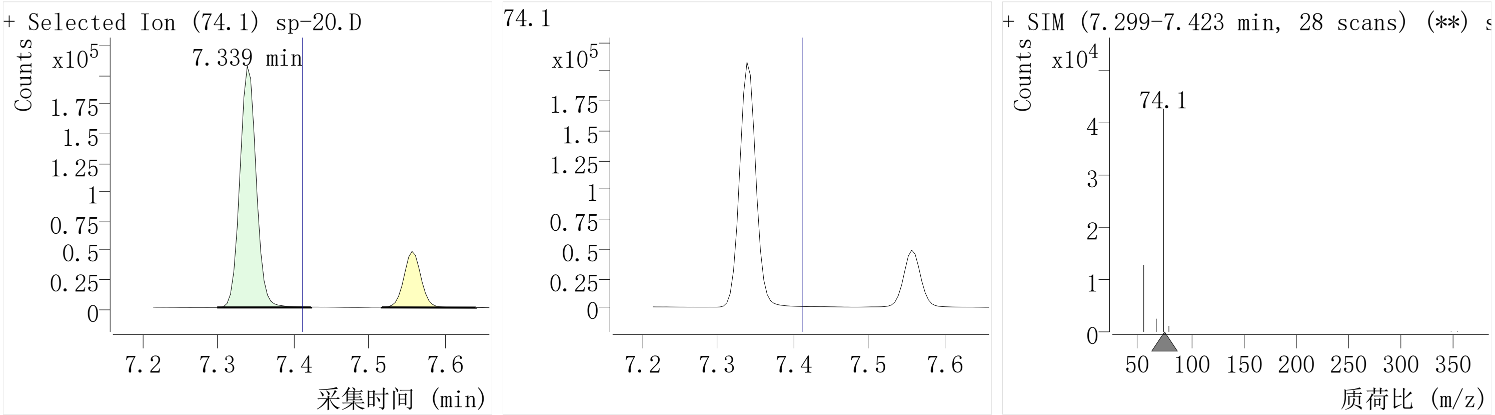

C17:1

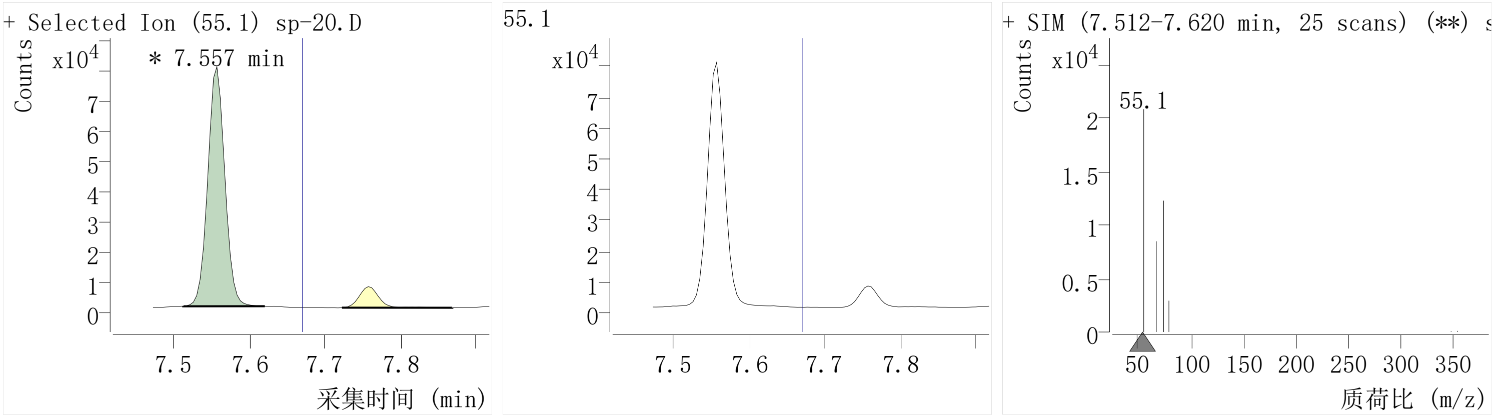

C18:0

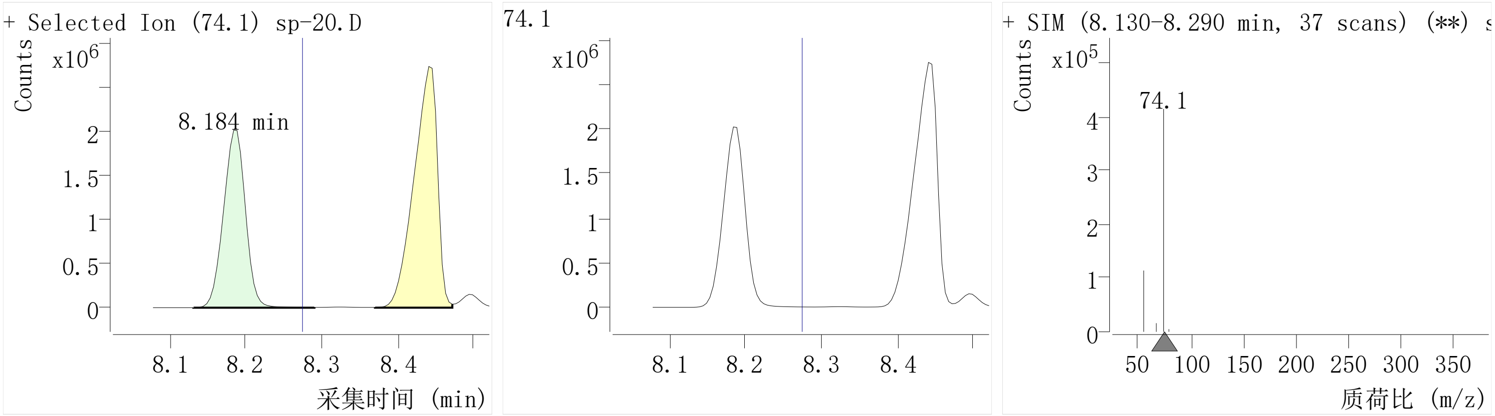

C18:1n9t

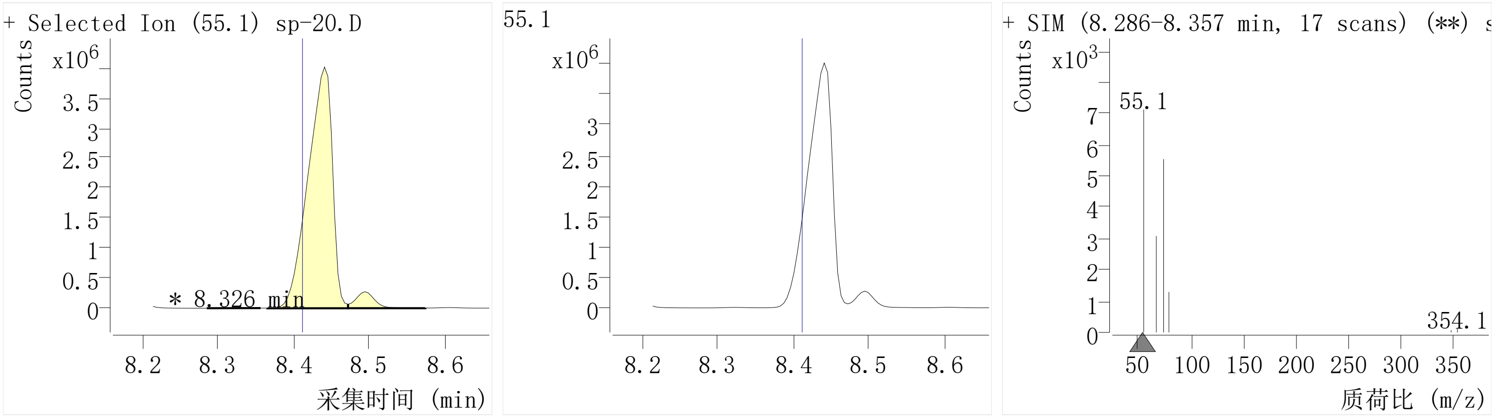

C18:1n9c

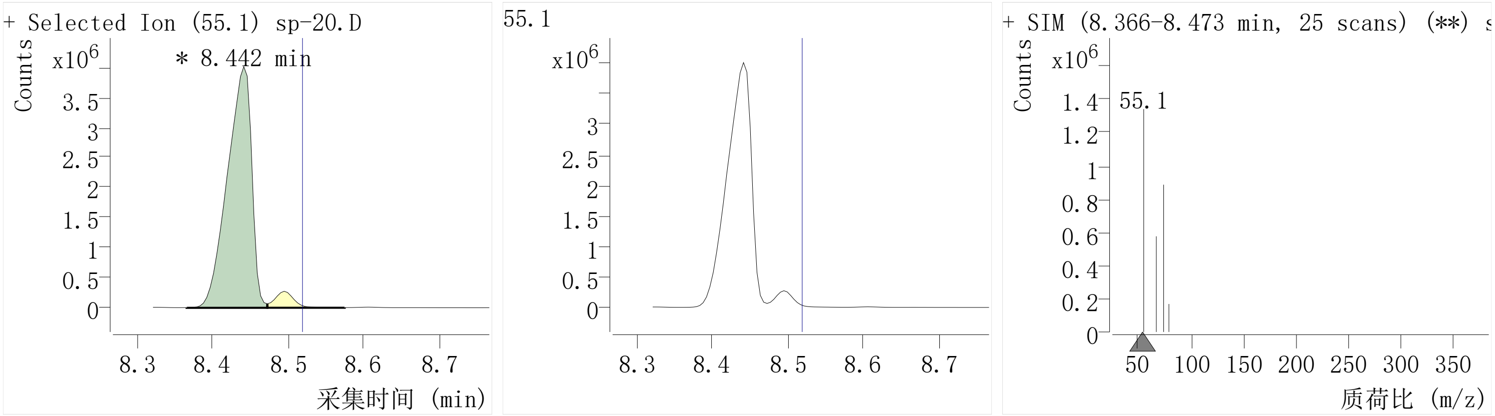

C18:2n6t

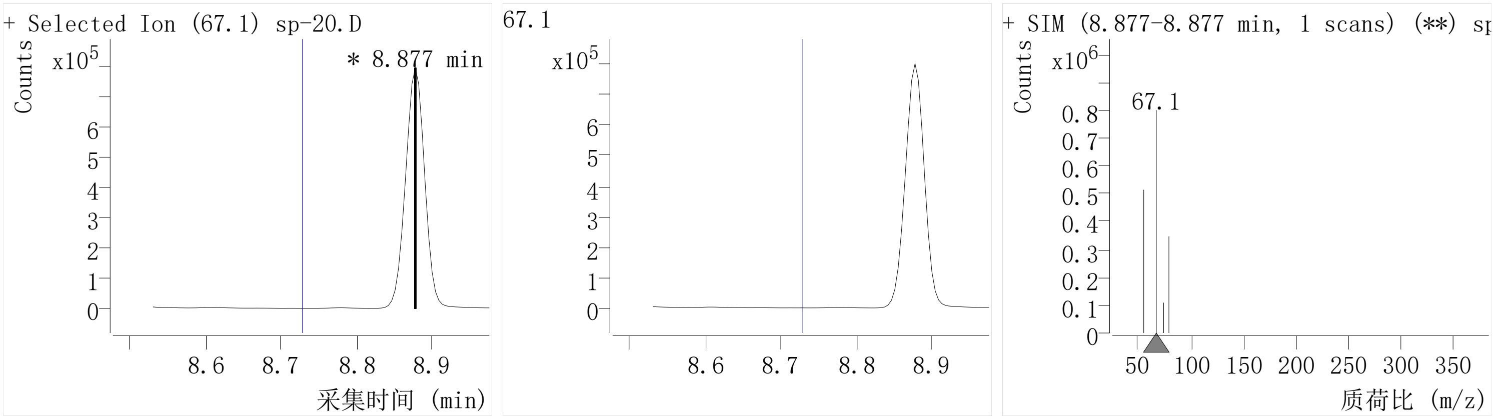

C18:2n6c

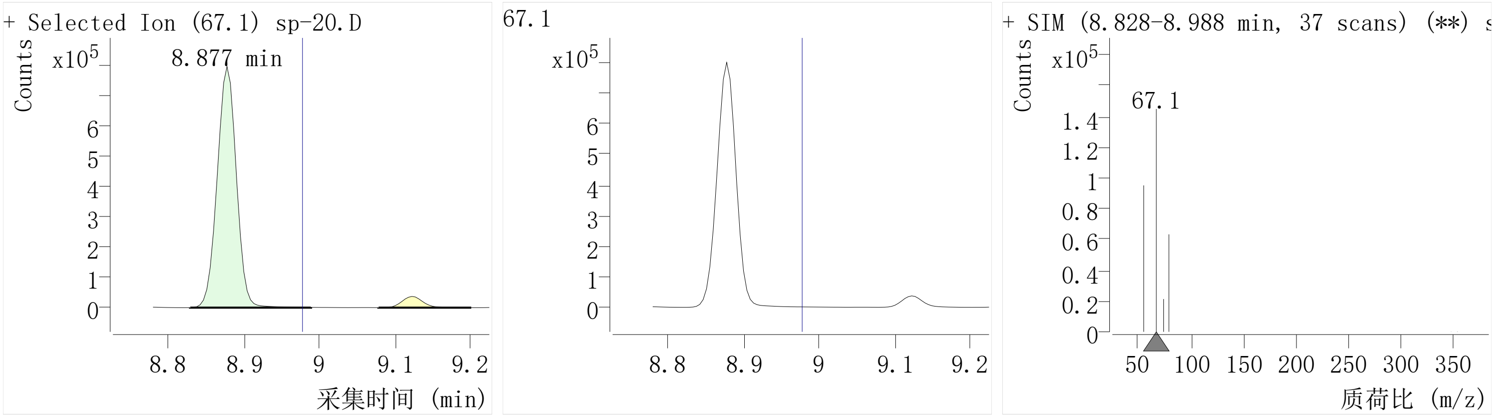

C18:3n6

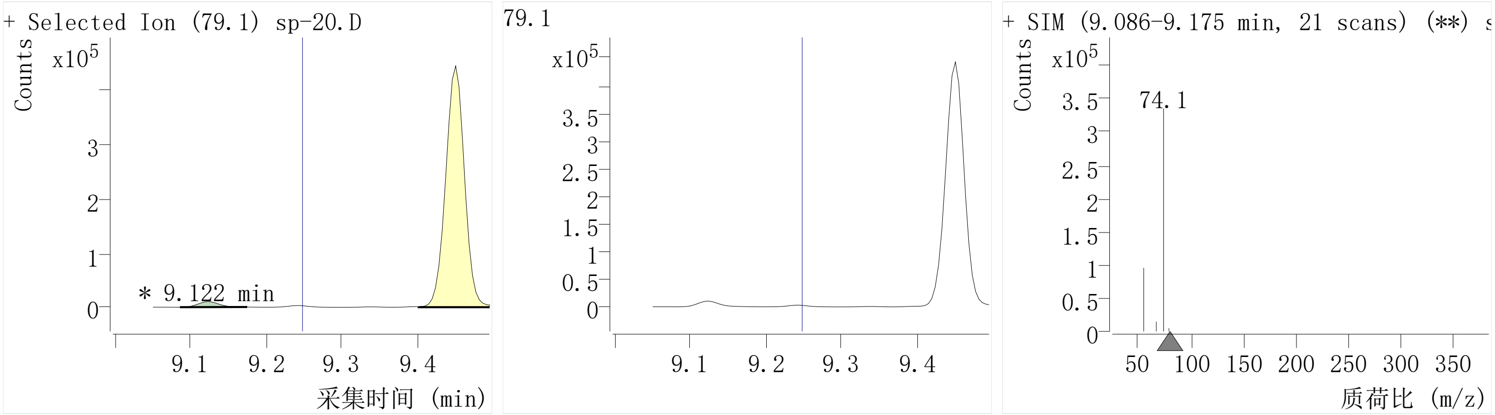

C18:3n3

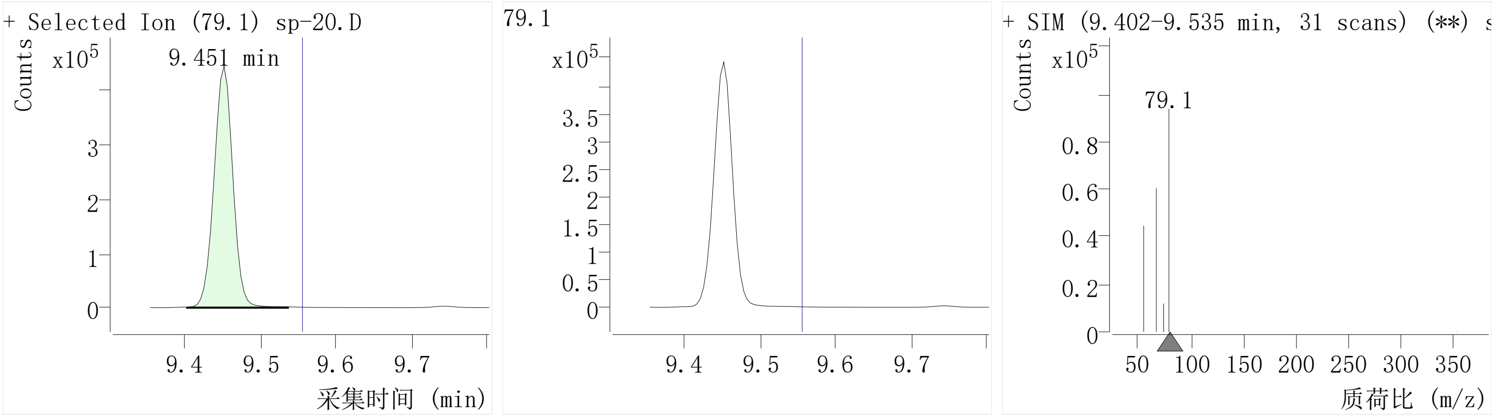

C20:0

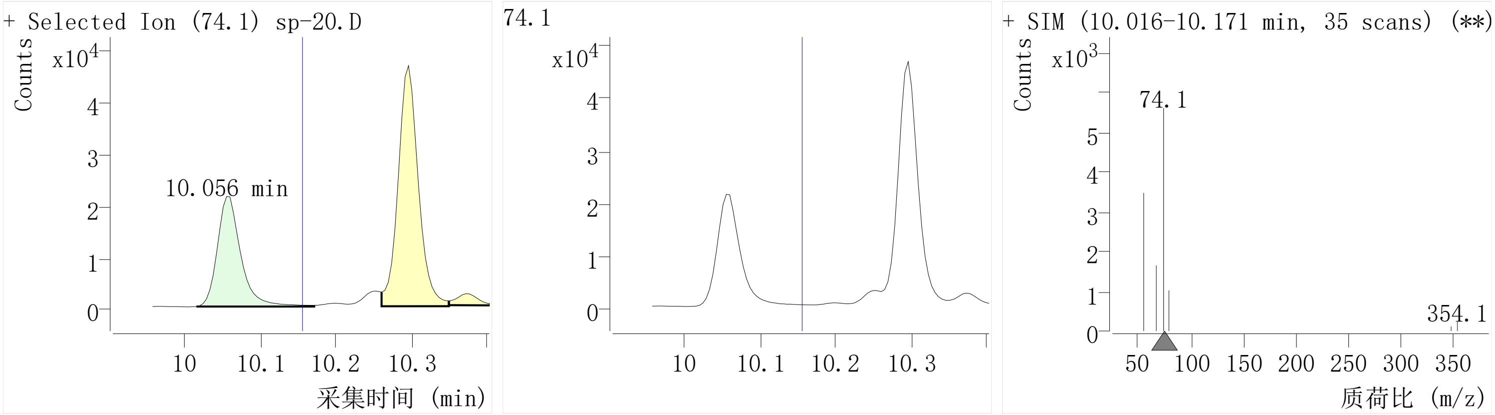

C20:1

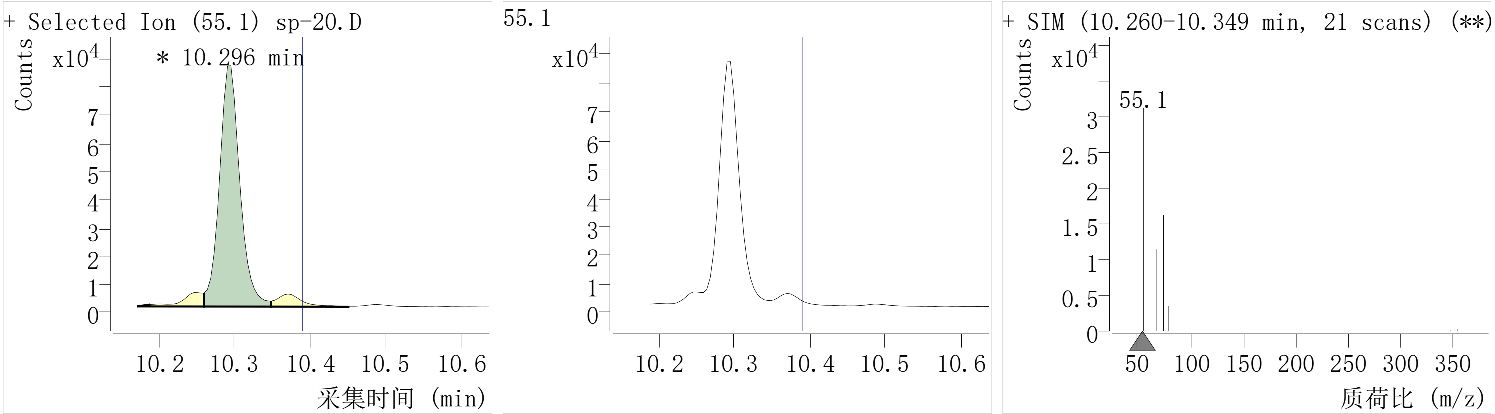

C20:2

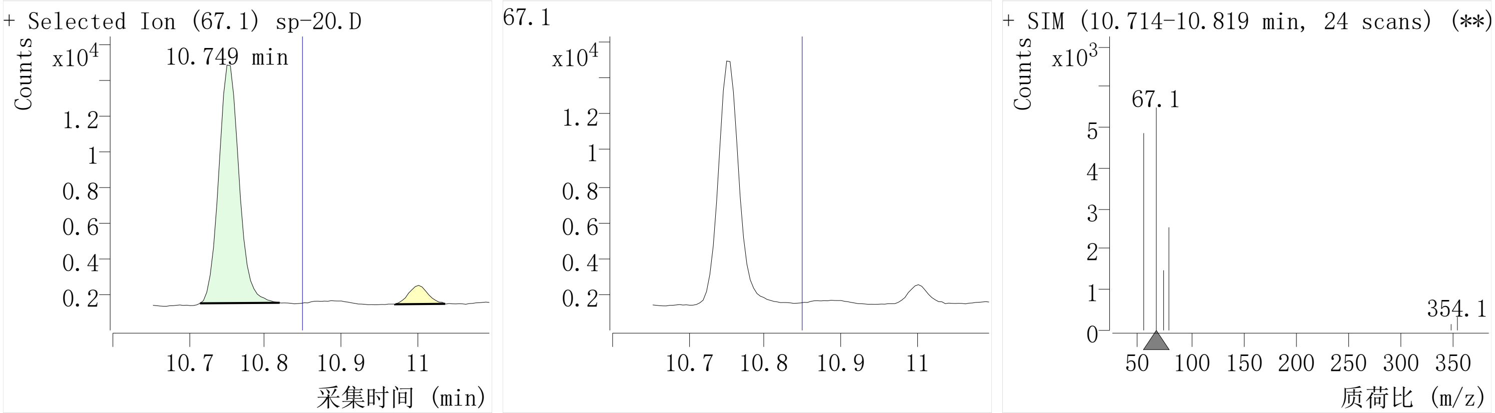

C21:0

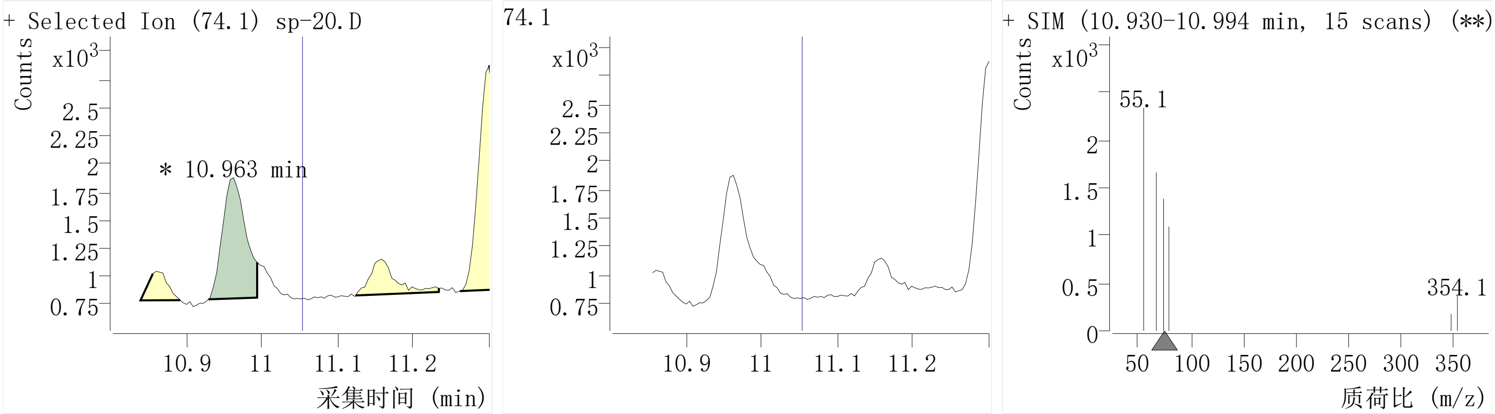

C20:3n6

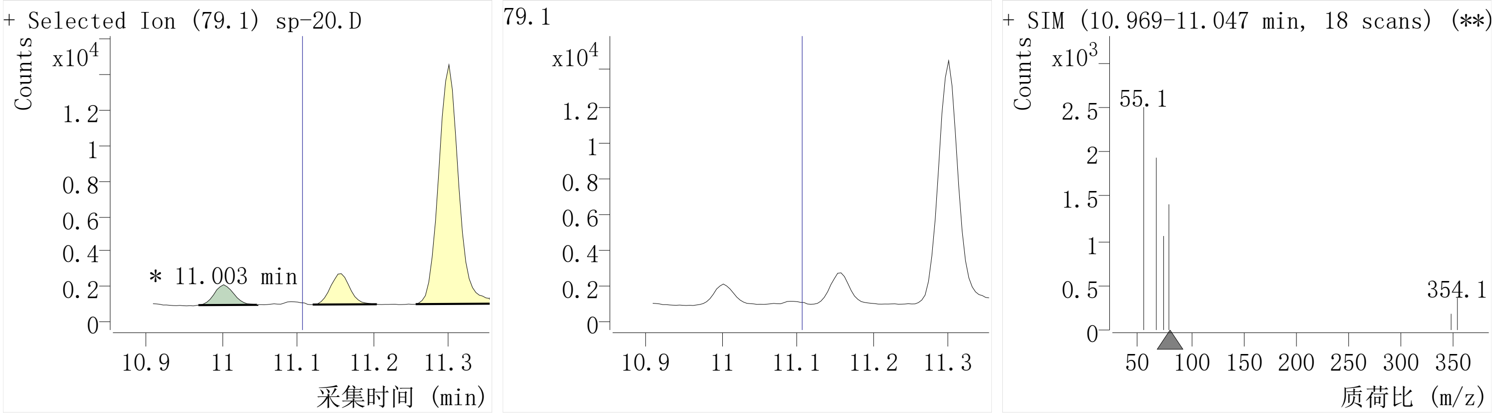

C20:4n6

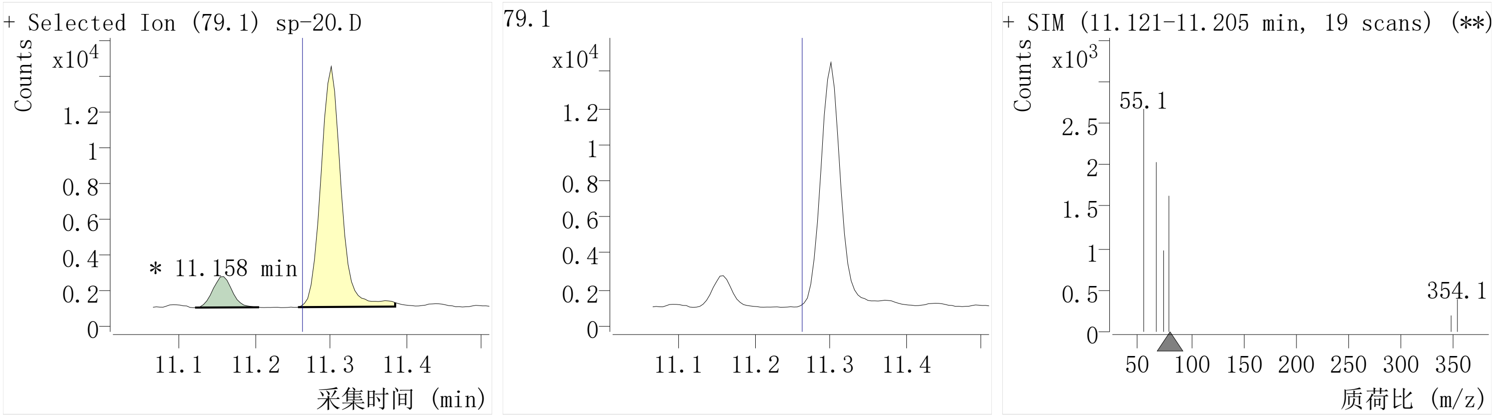

C20:3n3

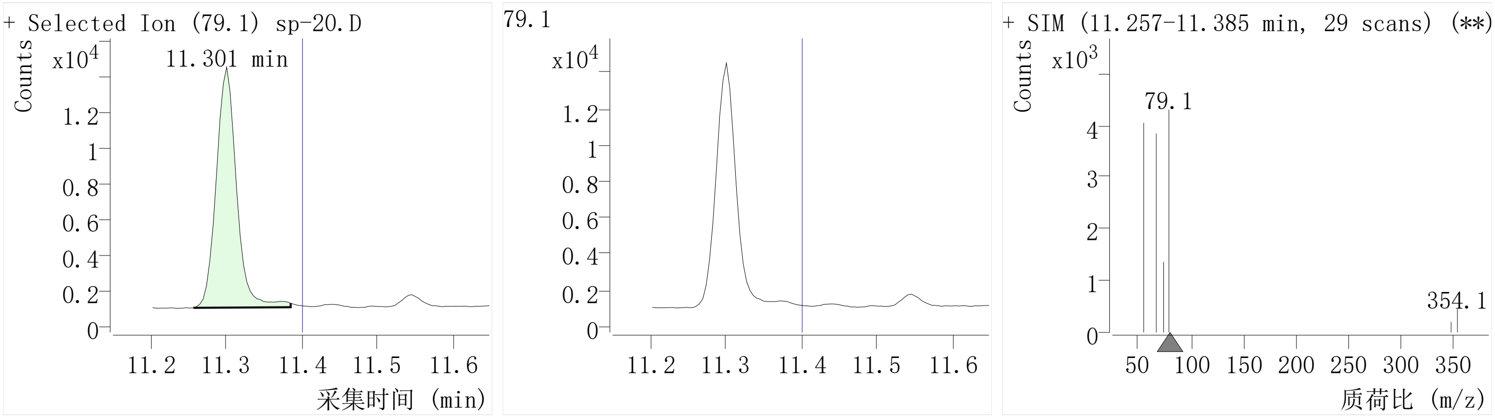

C20:5n3

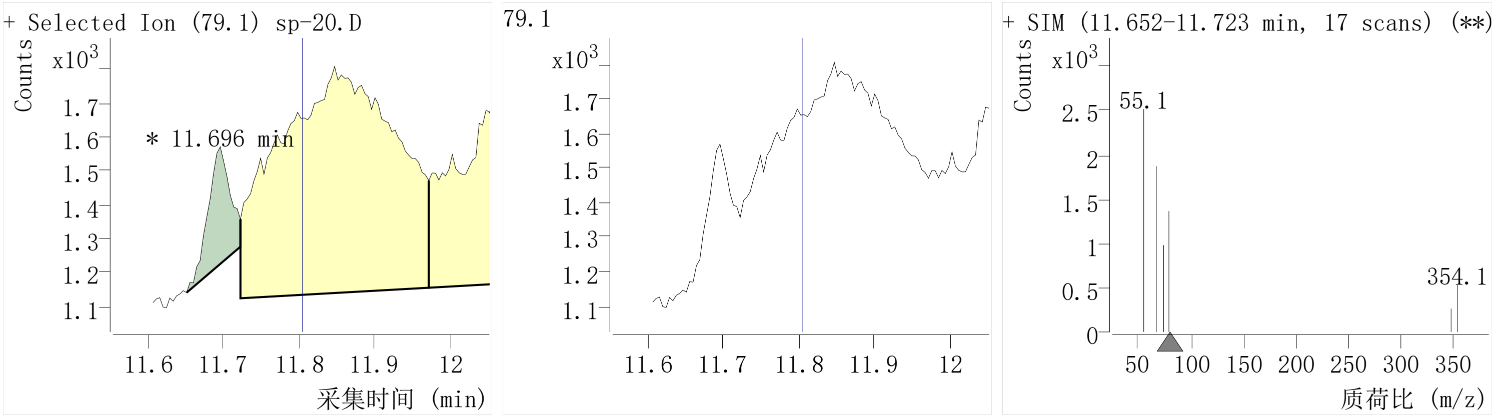

C22:0

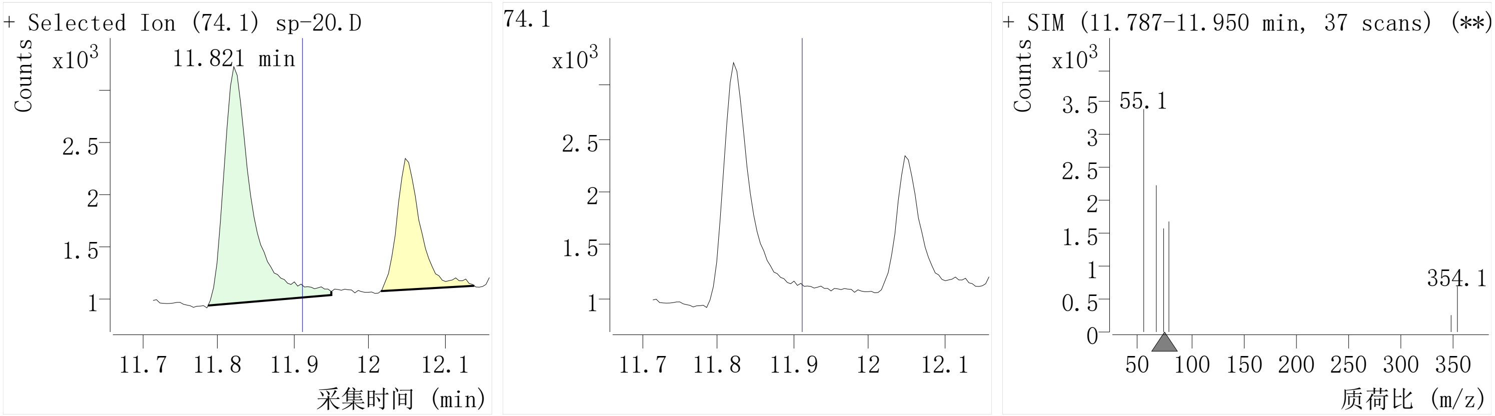

C22:1n9

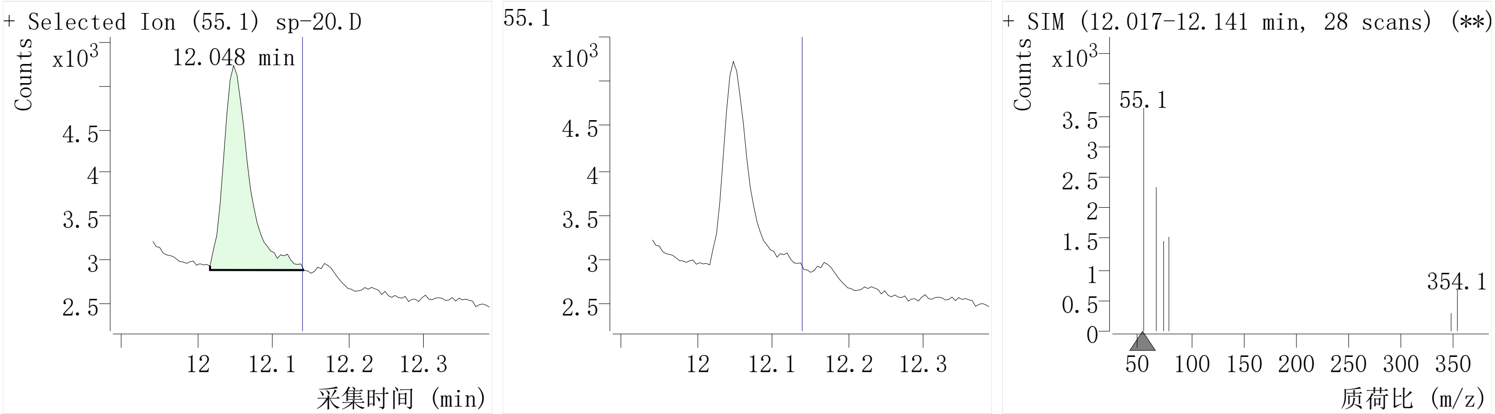

C22:2n6

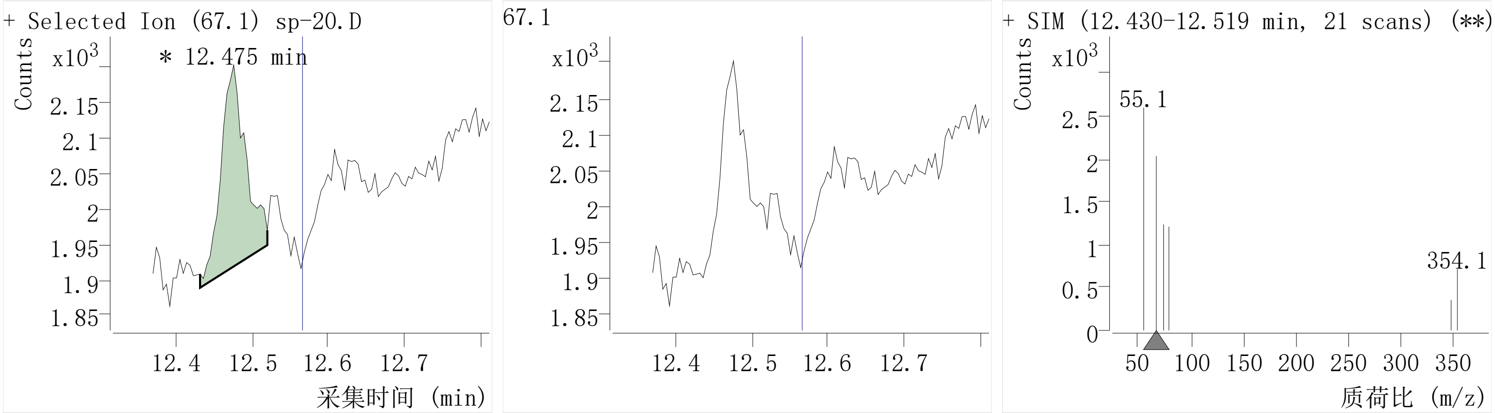

C23:0

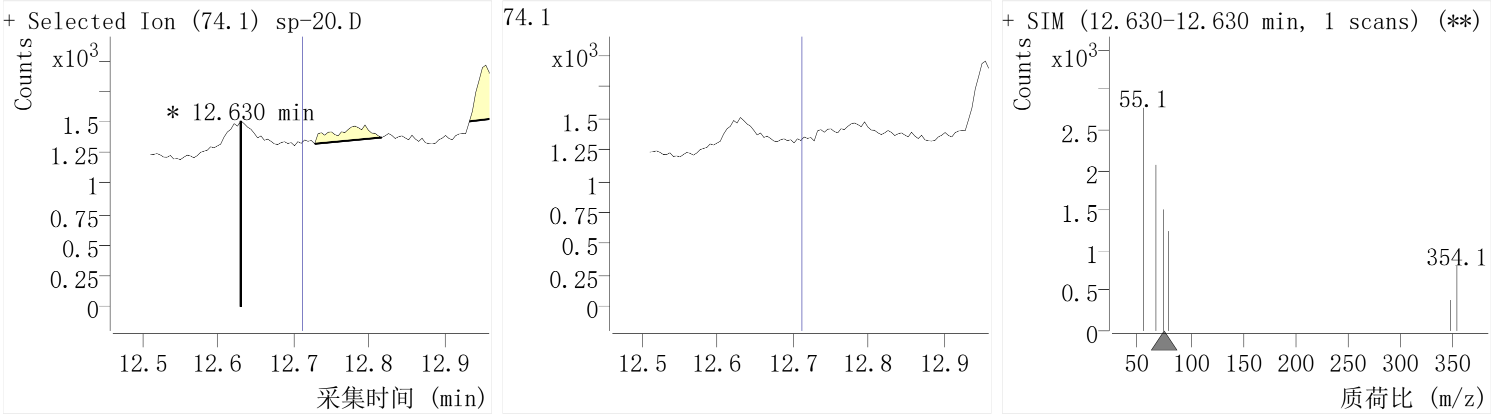

C24:0

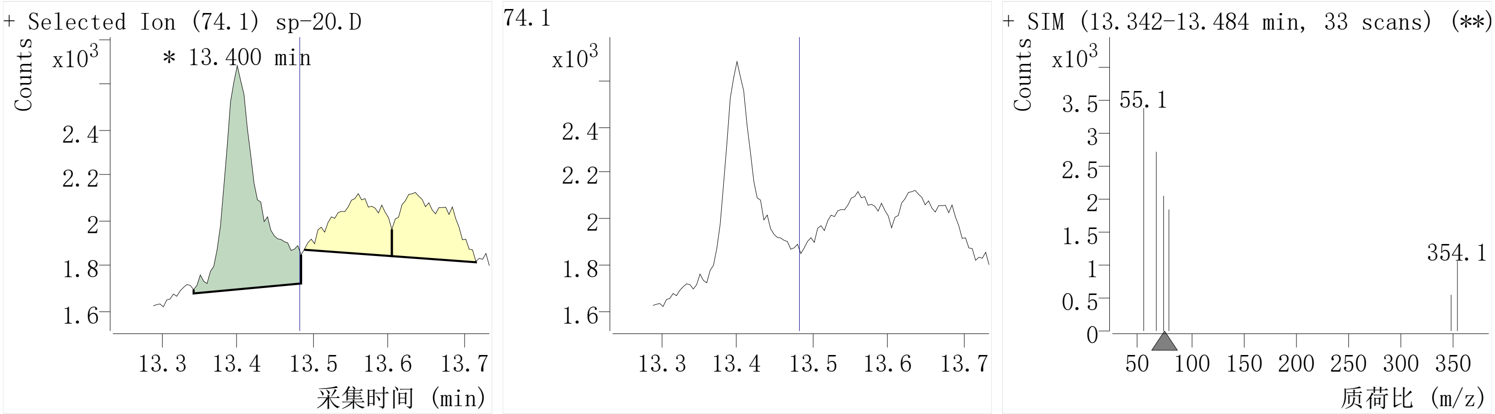

C22:6

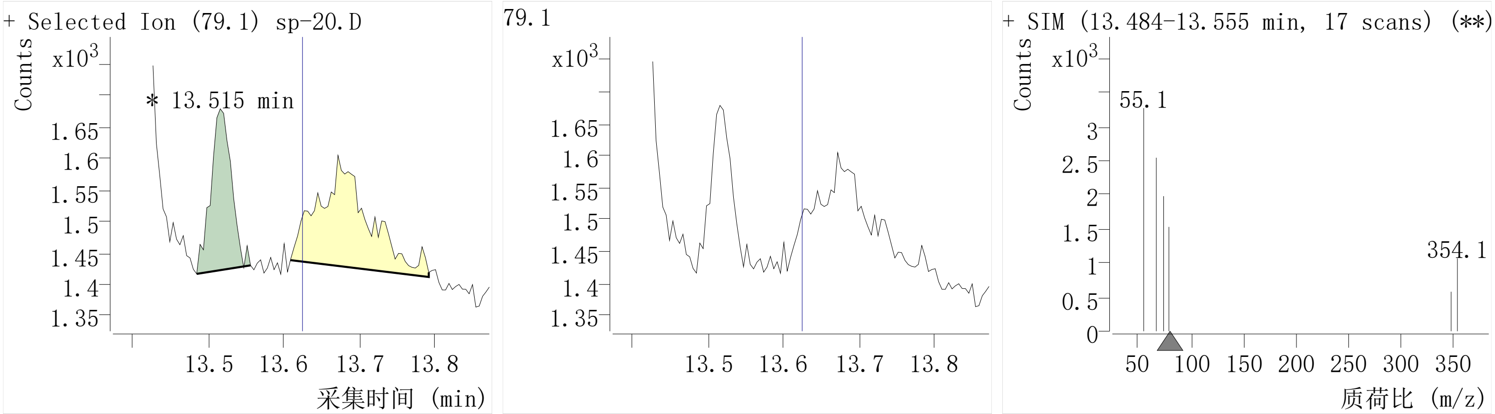
+ Selected Ion (79.1) sp-20.D
Counts
x10^3
* 13.515 min
采集时间 (min)
79.1
+ SIM (13.484-13.555 min, 17 scans) (**)
55.1
354.1
质荷比 (m/z)

C24:1

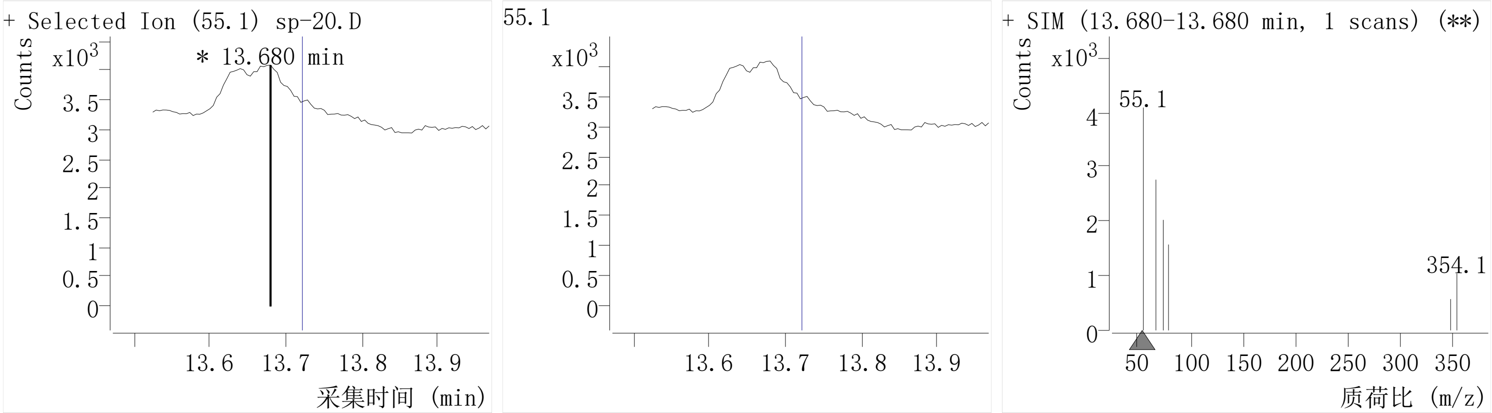
+ Selected Ion (55.1) sp-20.D
Counts
x10^3
* 13.680 min
采集时间 (min)
55.1
+ SIM (13.680-13.680 min, 1 scans) (**)
55.1
354.1
质荷比 (m/z)

**批处理路径**	G:\GC-MS\HX250430-4-GCMS总脂肪酸靶向检测\HX250430-4\QuantResults\HX250430-4.batch.bin		
**分析时间**	2025/5/14 16:58	**分析员姓名**	DESKTOP-M3A0GPO\omics
**报告时间**	2025/5/16 14:53:20	**报告员姓名**	DESKTOP-M3A0GPO\omics
**最近校正更新**	2025/5/14 16:58	**批处理状态**	已处理
**定量批处理版本**	10.2	**定量报告版本**	10.2

**采集时间**	2025/5/9 5:16	**数据文件**	sp-21.D
**样品类型**	样品	**样品名称**	sp-21
**稀释**	1	**采集方法**	脂肪酸

样品色谱图

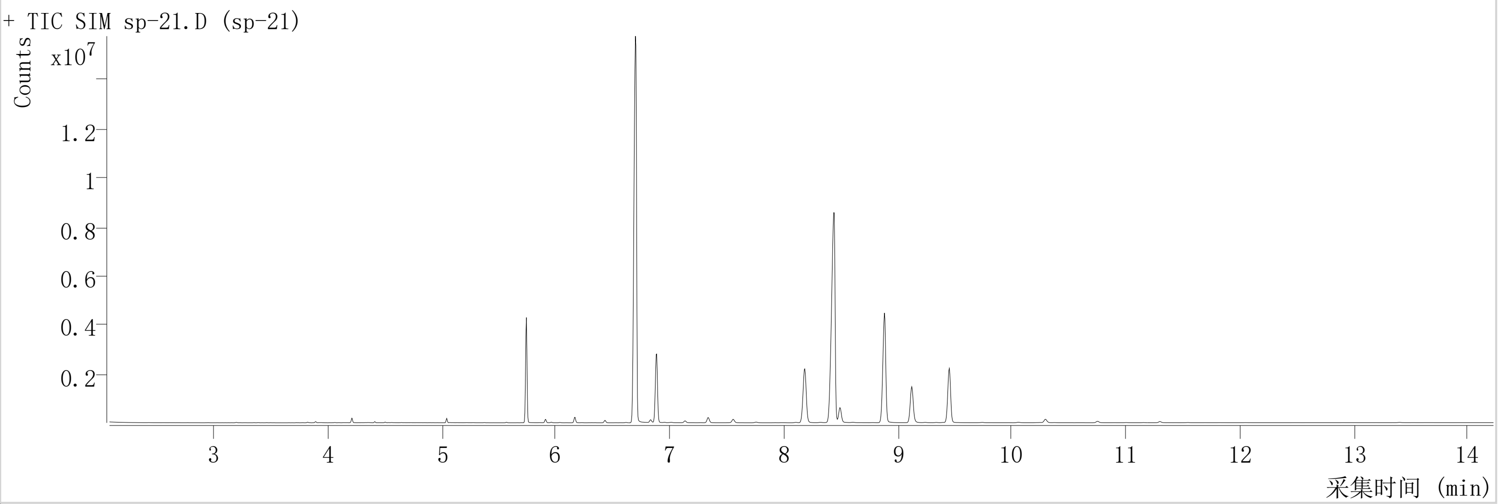

化合物	ISTD	RT	响应	ISTD 响应	响应比	最终浓度	单位
C4:0	C19:0	2.199	138	2090151	0.0001	ND	ug/ml
C6:0	C19:0	2.959	625	2090151	0.0003	0.0030	ug/ml
C8:0	C19:0	3.724	2539	2090151	0.0012	0.0081	ug/ml
C10:0	C19:0	4.413	26034	2090151	0.0125	0.1809	ug/ml
C11:0	C19:0	4.729	1288	2090151	0.0006	0.0049	ug/ml
C12:0	C19:0	5.044	104147	2090151	0.0498	0.7613	ug/ml
C13:0	C19:0	5.373	5623	2090151	0.0027	0.0282	ug/ml
C14:0	C19:0	5.742	2977132	2090151	1.4244	31.9335	ug/ml
C14:1	C19:0	5.911	64327	2090151	0.0308	1.4456	ug/ml
C15:0	C19:0	6.169	185903	2090151	0.0889	1.5341	ug/ml
C15:1	C19:0	6.432	0	2090151	0.0000	ND	ug/ml
C16:0	C19:0	6.696	16265198	2090151	7.7818	305.9795	ug/ml
C16:1	C19:0	6.881	1756328	2090151	0.8403	51.2626	ug/ml
C17:0	C19:0	7.339	246553	2090151	0.1180	2.4610	ug/ml
C17:1	C19:0	7.557	107123	2090151	0.0513	2.7314	ug/ml
C18:0	C19:0	8.184	3344449	2090151	1.6001	34.1989	ug/ml
C18:1n9t	C19:0	8.322	10204	2090151	0.0049	0.2880	ug/ml
C18:1n9c	C19:0	8.437	8396776	2090151	4.0173	271.5130	ug/ml
C18:2n6t	C19:0	8.882	0	2090151	0.0000	ND	ug/ml
C18:2n6c	C19:0	8.882	3594647	2090151	1.7198	143.7240	ug/ml
C18:3n6	C19:0	9.122	24131	2090151	0.0115	0.0022	ug/ml
C18:3n3	C19:0	9.451	1809209	2090151	0.8656	43.0293	ug/ml
C20:0	C19:0	10.056	33722	2090151	0.0161	0.4409	ug/ml
C20:1	C19:0	10.291	139426	2090151	0.0667	4.1507	ug/ml
C20:2	C19:0	10.749	49053	2090151	0.0235	1.4889	ug/ml
C21:0	C19:0	10.963	2222	2090151	0.0011	0.0294	ug/ml
C20:3n6	C19:0	11.003	5214	2090151	0.0025	0.2018	ug/ml
C20:4n6	C19:0	11.154	5563	2090151	0.0027	0.2012	ug/ml
C20:3n3	C19:0	11.296	37869	2090151	0.0181	0.9983	ug/ml
C20:5n3	C19:0	11.697	1181	2090151	0.0006	0.0664	ug/ml

定量分析完成报告

化合物	ISTD	RT	响应	ISTD 响应	响应比	最终浓度	单位
C22:0	C19:0	11.821	3786	2090151	0.0018	0.0789	ug/ml
C22:1n9	C19:0	12.048	4454	2090151	0.0021	0.1430	ug/ml
C22:2n6	C19:0	12.475	726	2090151	0.0003	0.0644	ug/ml
C23:0	C19:0	12.786	0	2090151	0.0000	ND	ug/ml
C24:0	C19:0	13.400	2466	2090151	0.0012	0.0595	ug/ml
C22:6	C19:0	13.515	1424	2090151	0.0007	0.0555	ug/ml
C24:1	C19:0	13.675	0	2090151	0.0000	ND	ug/ml

C4:0

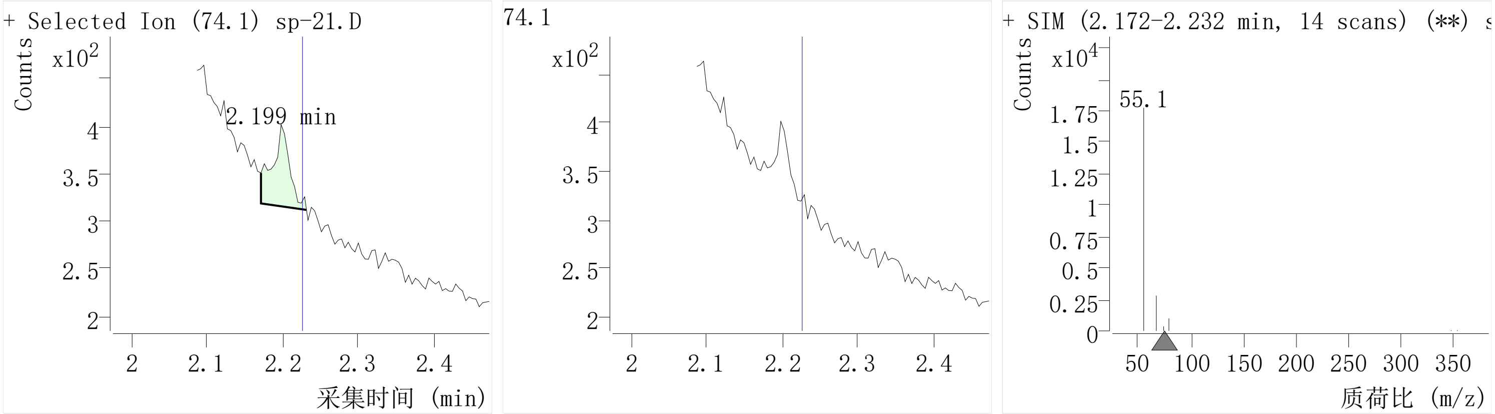

C6:0

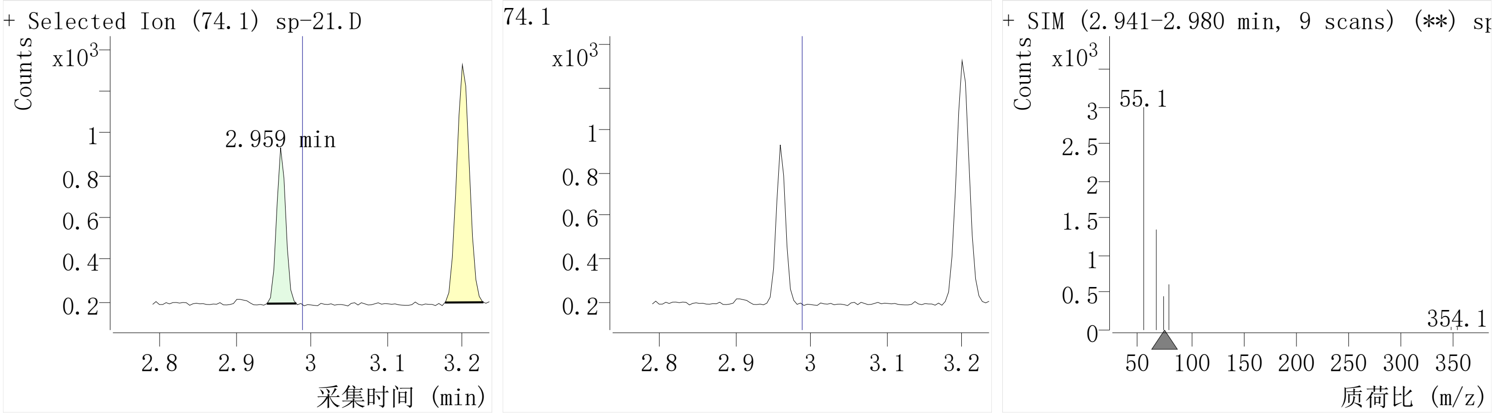

C8:0

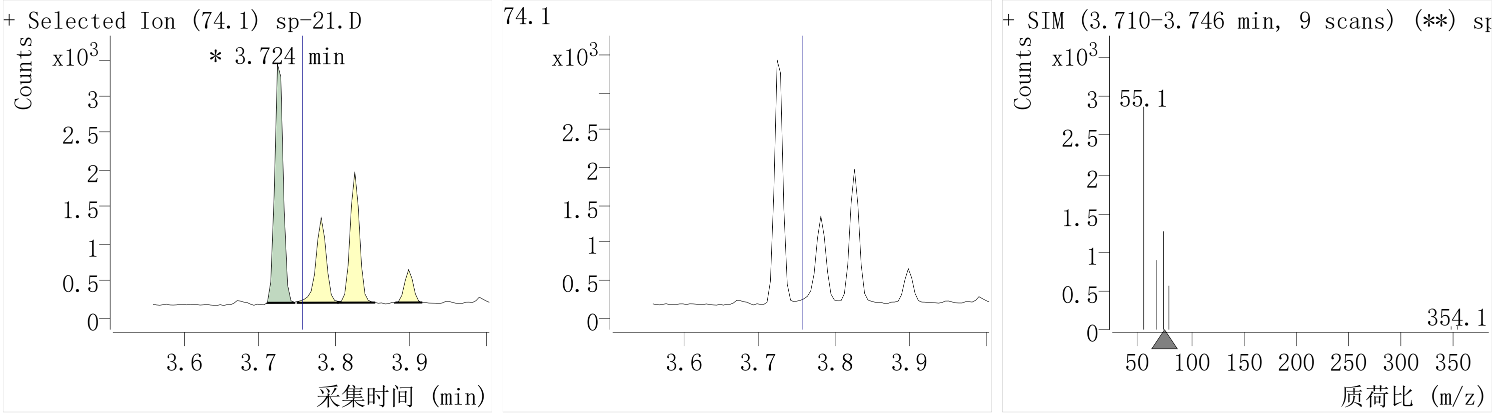

C10:0

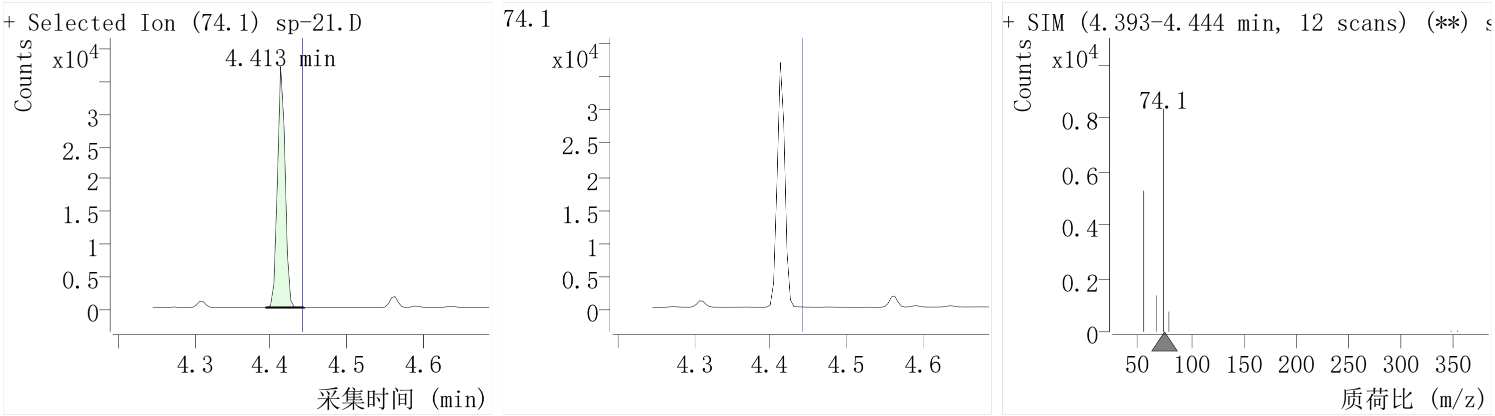

C11:0

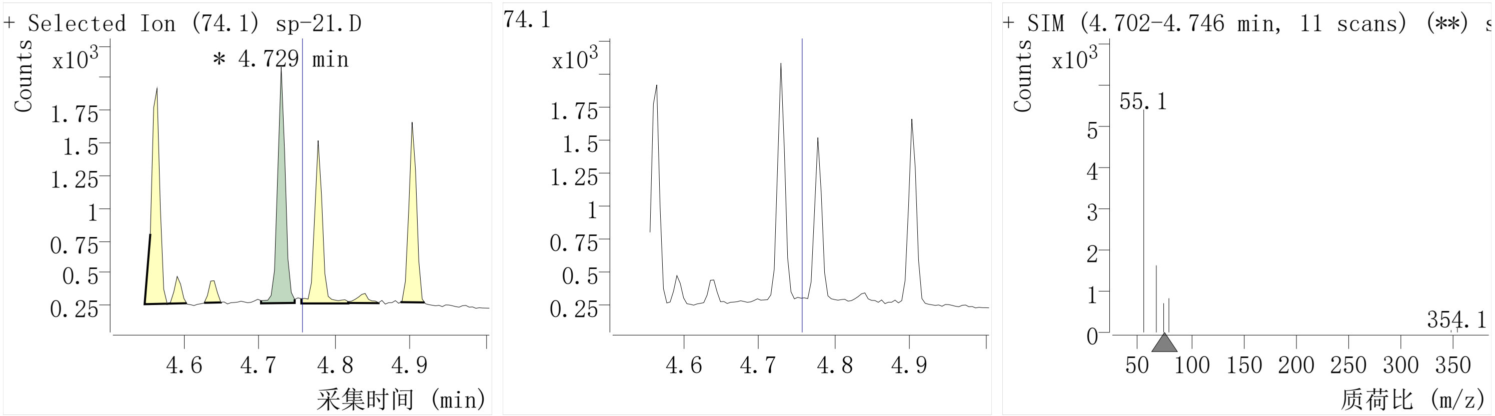

C12:0

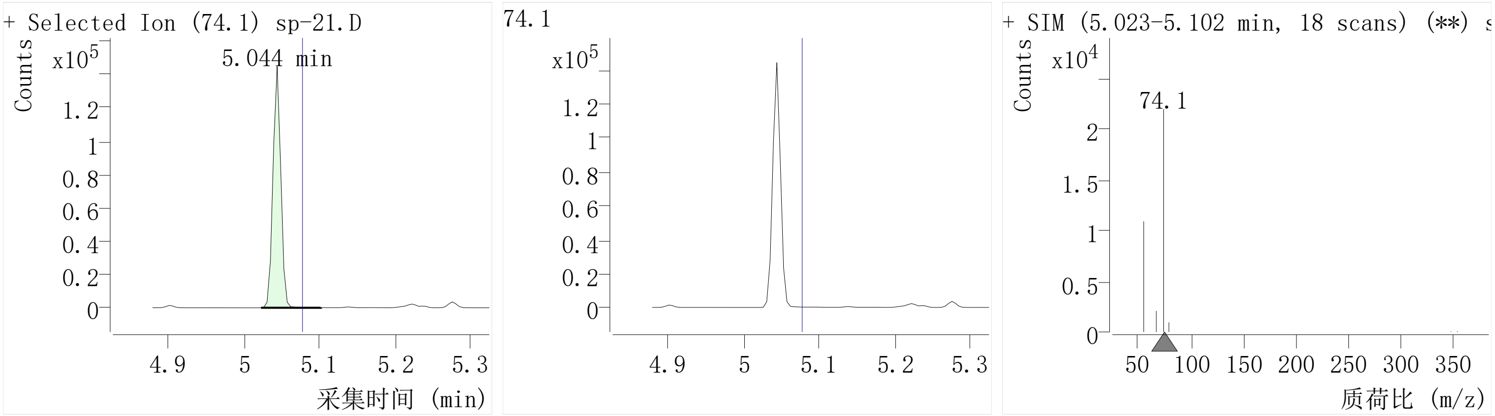

C13:0

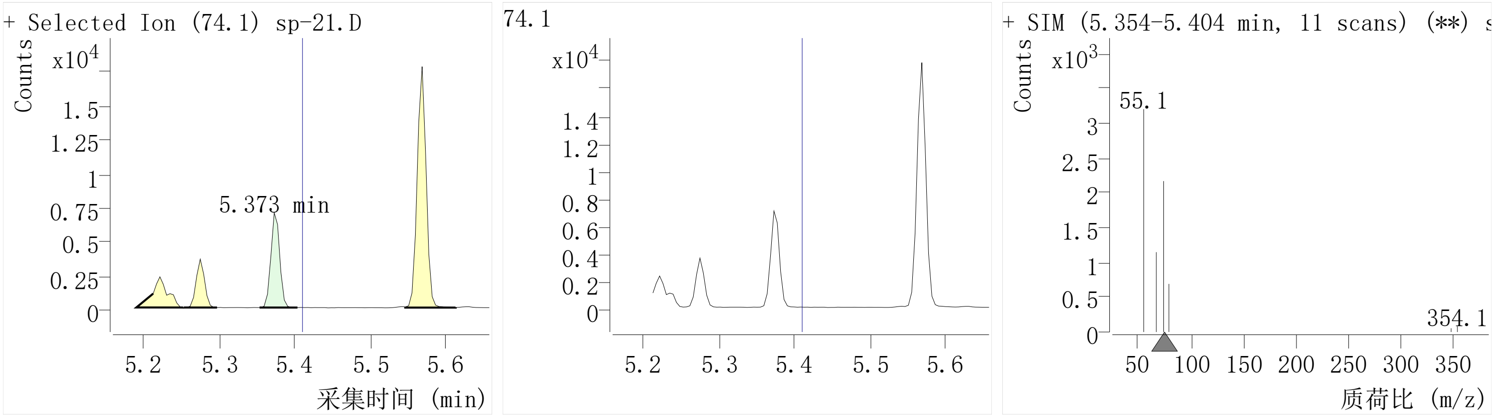

C14:0

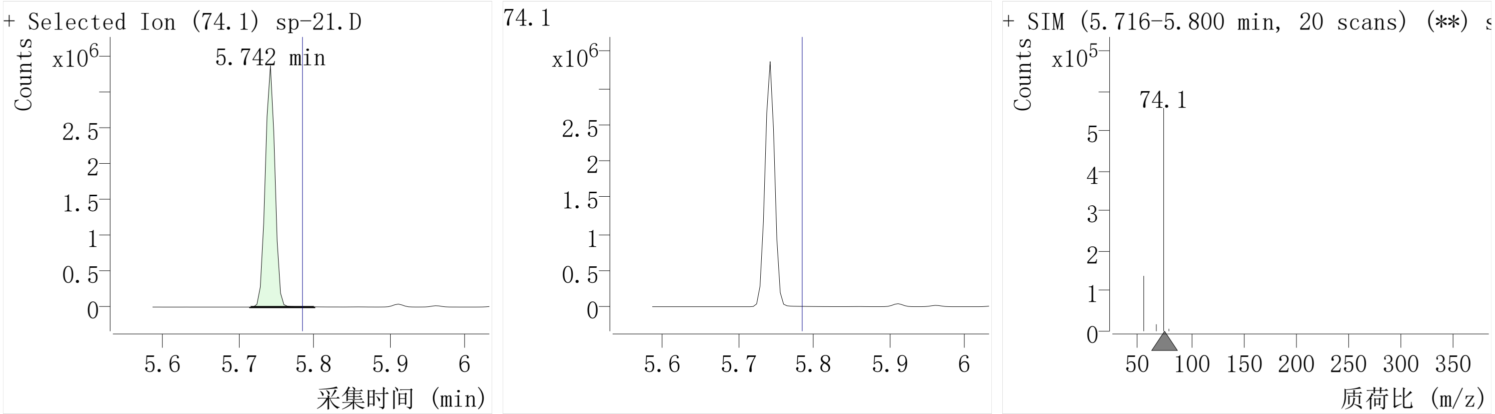

C14:1

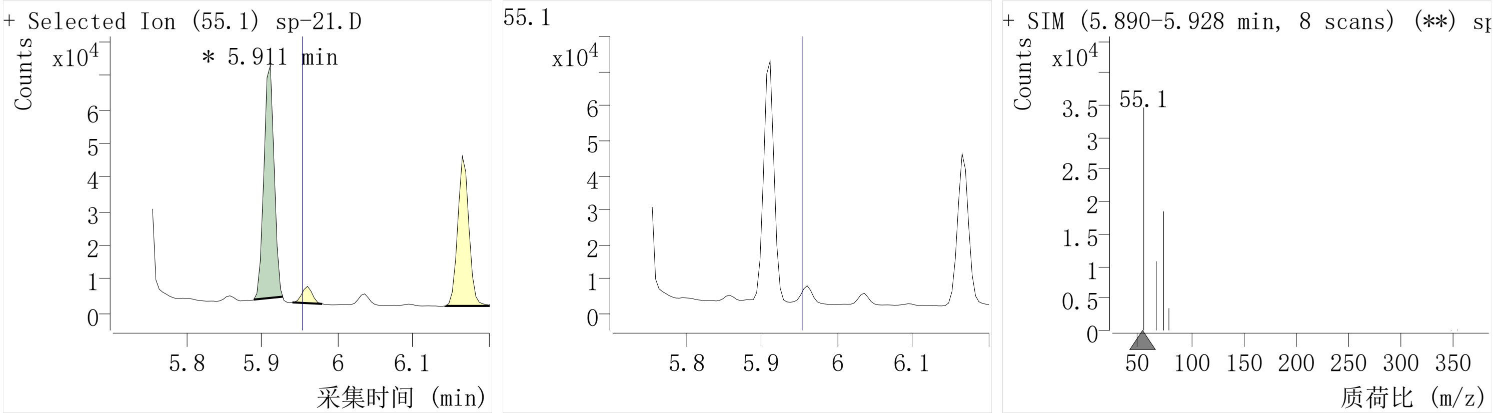

C15:0

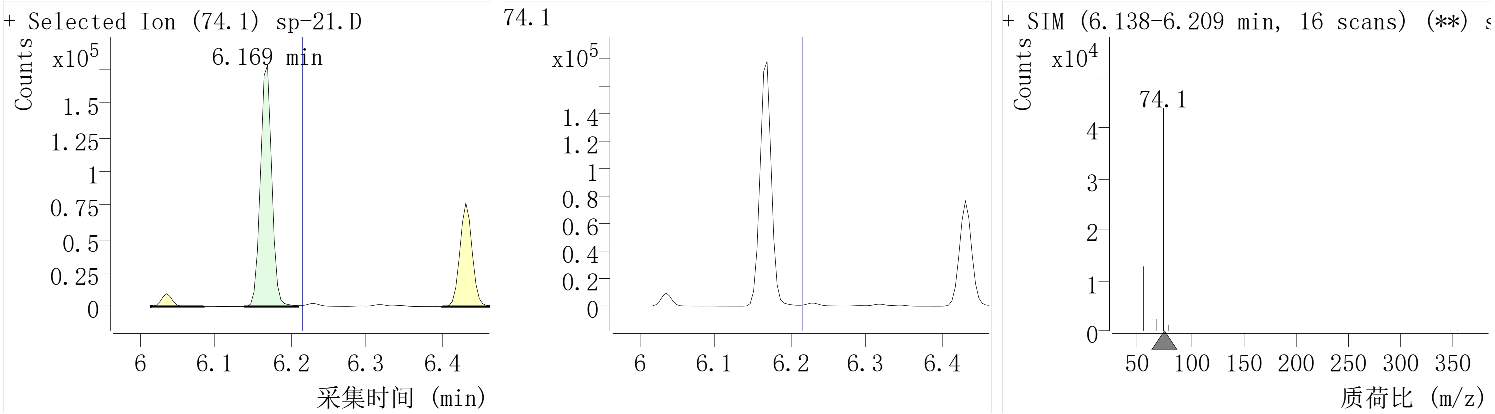

C15:1

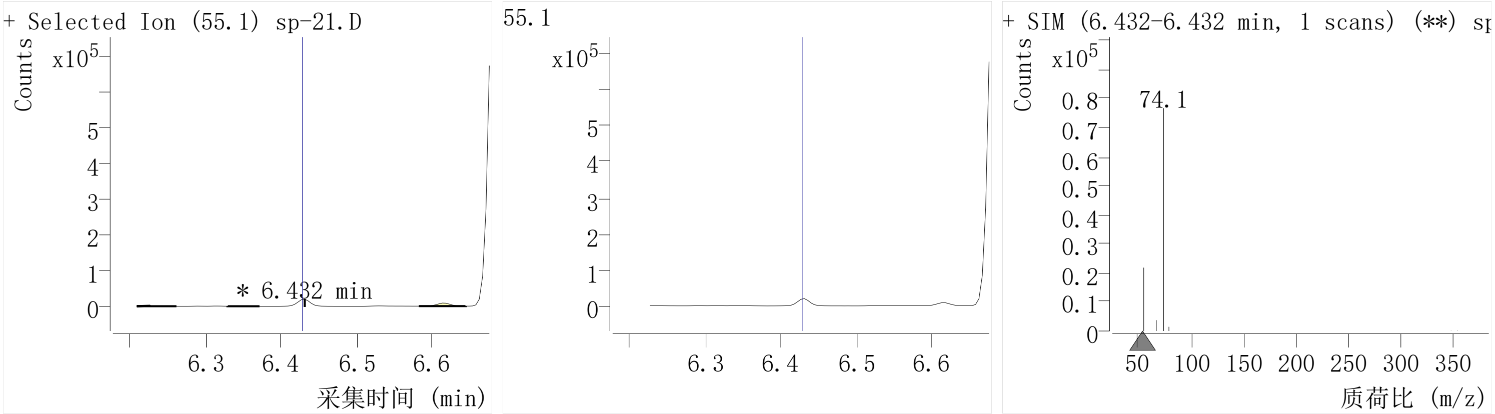

C16:0

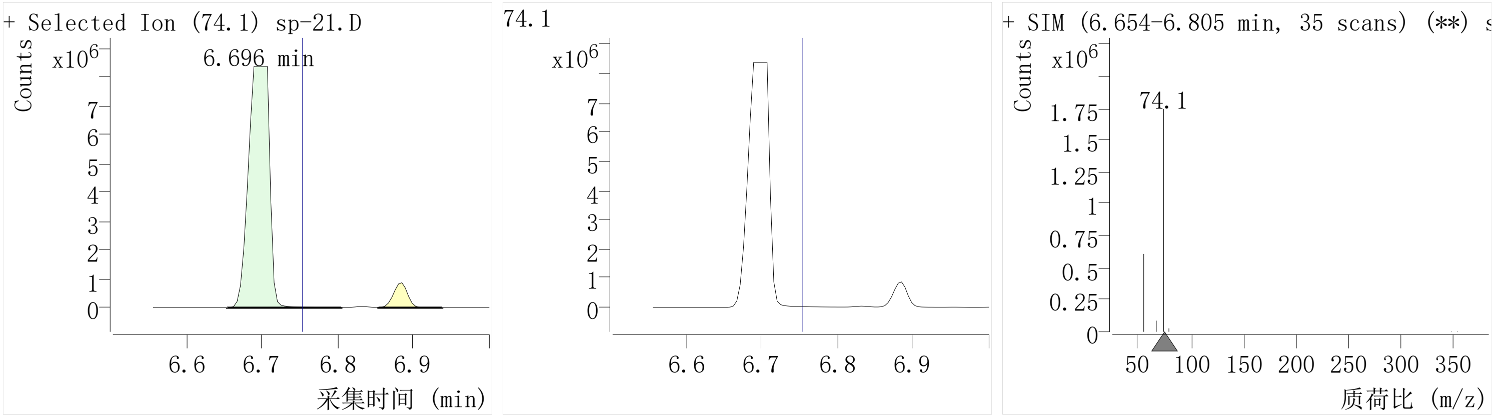

C16:1

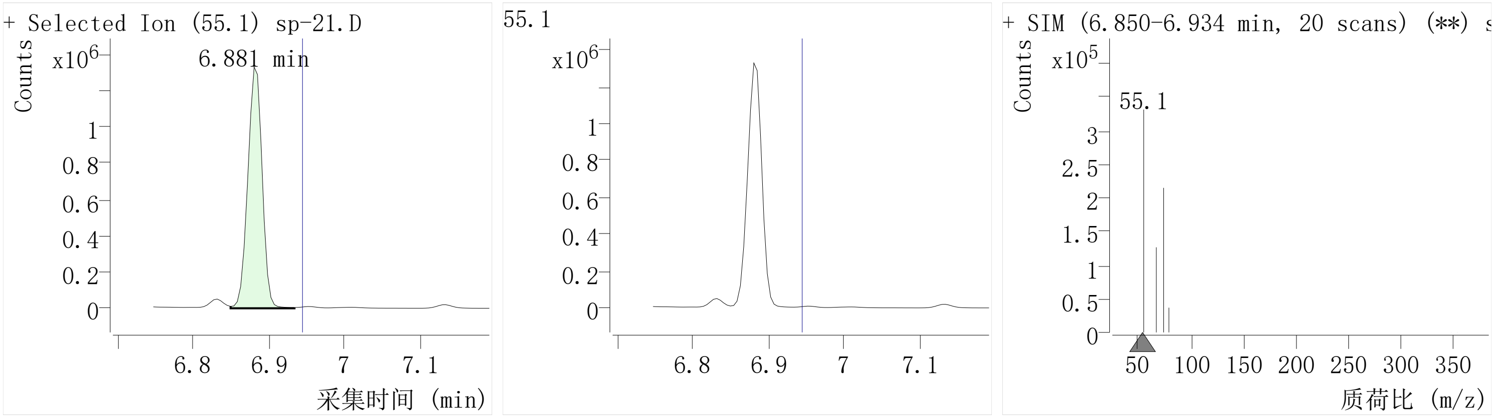

C17:0

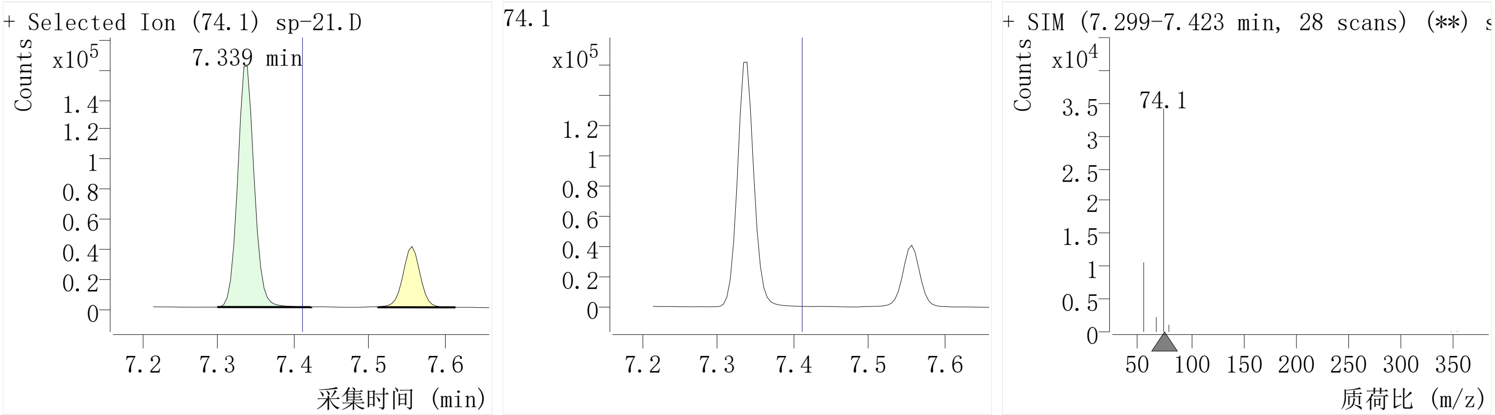

C17:1

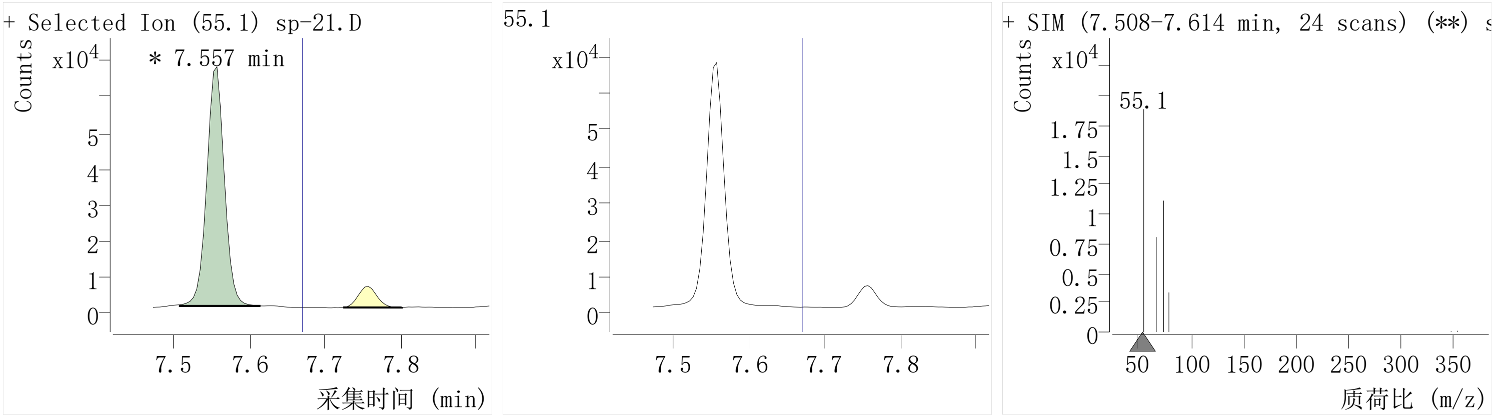

C18:0

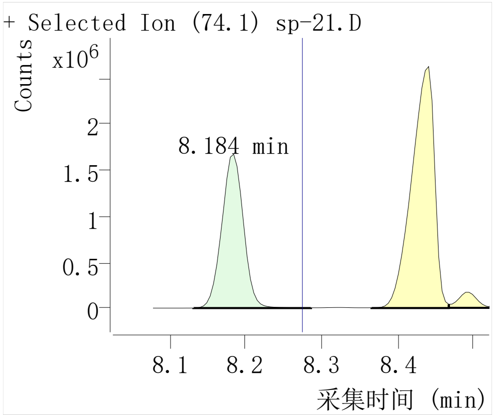

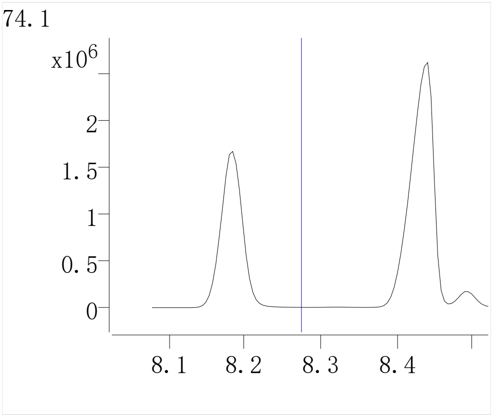

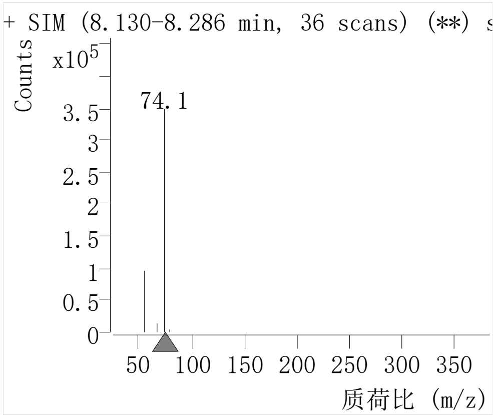

C18:1n9t

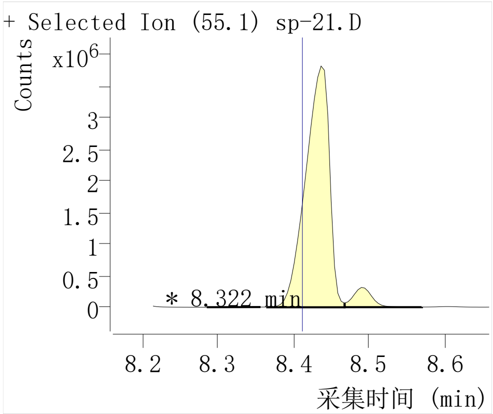

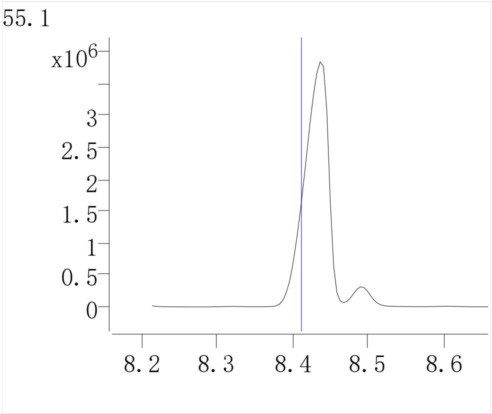

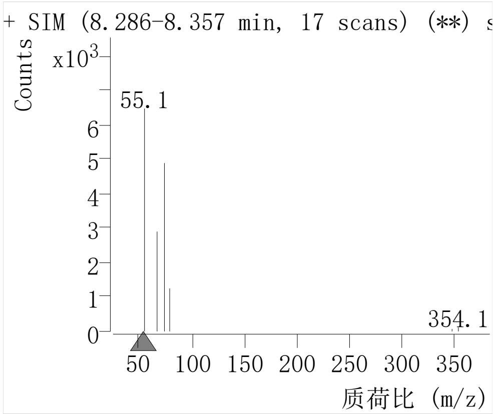

C18:1n9c

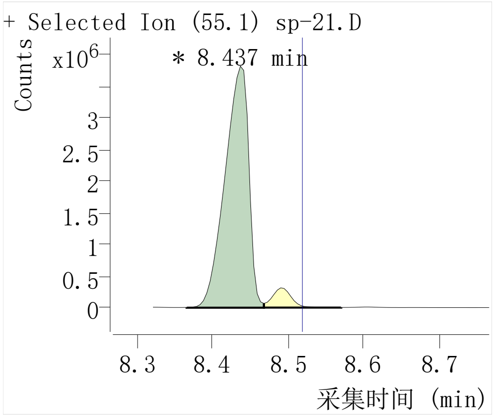

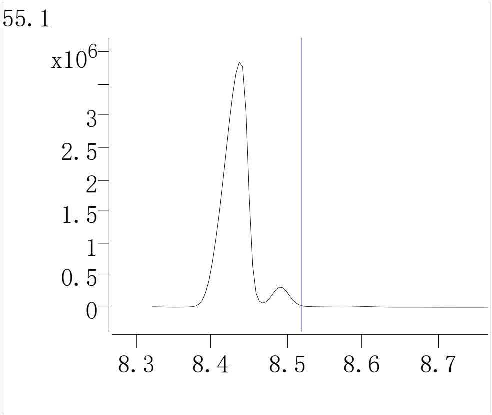

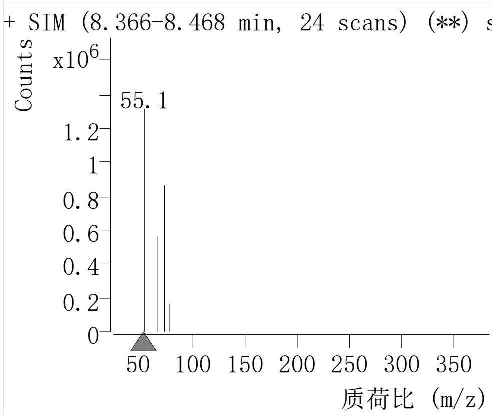

C18:2n6t

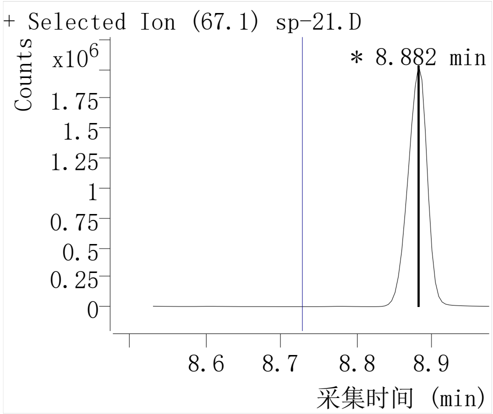

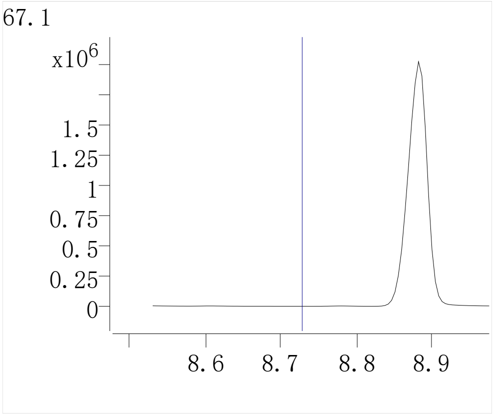

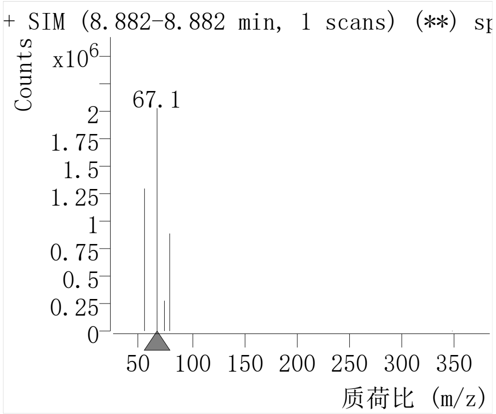

C18:2n6c

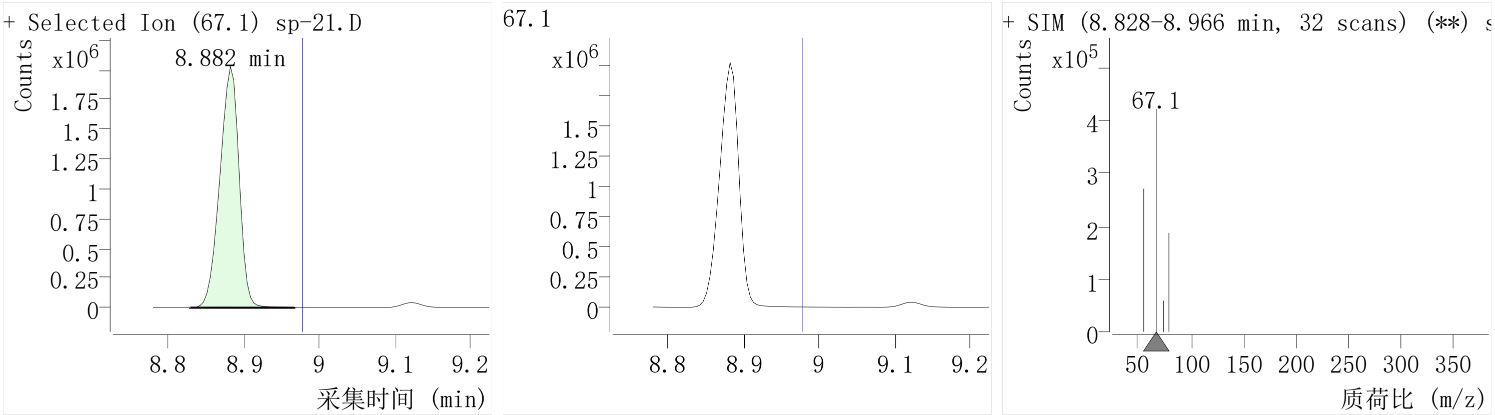

C18:3n6

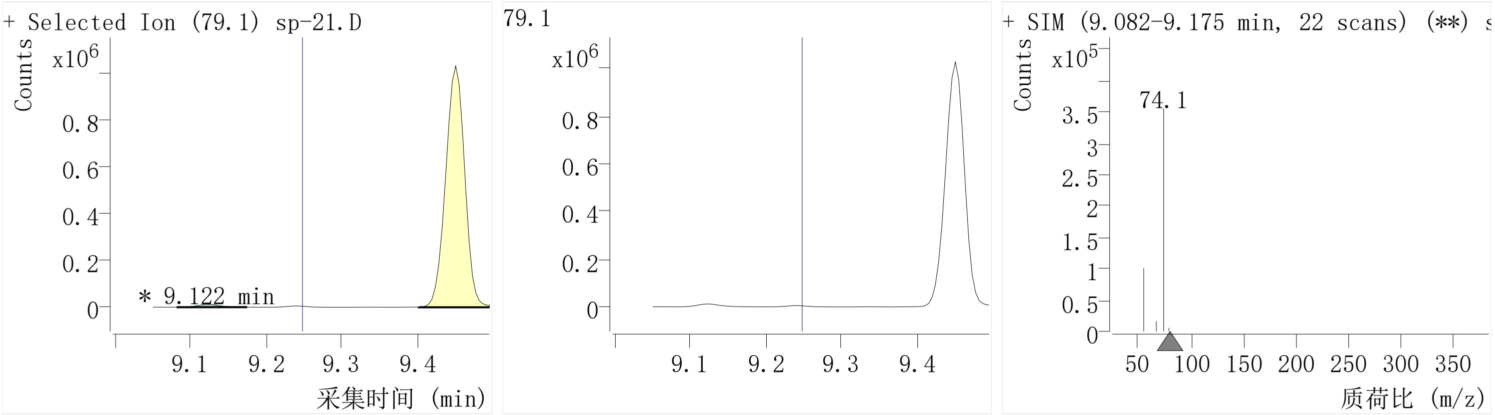

C18:3n3

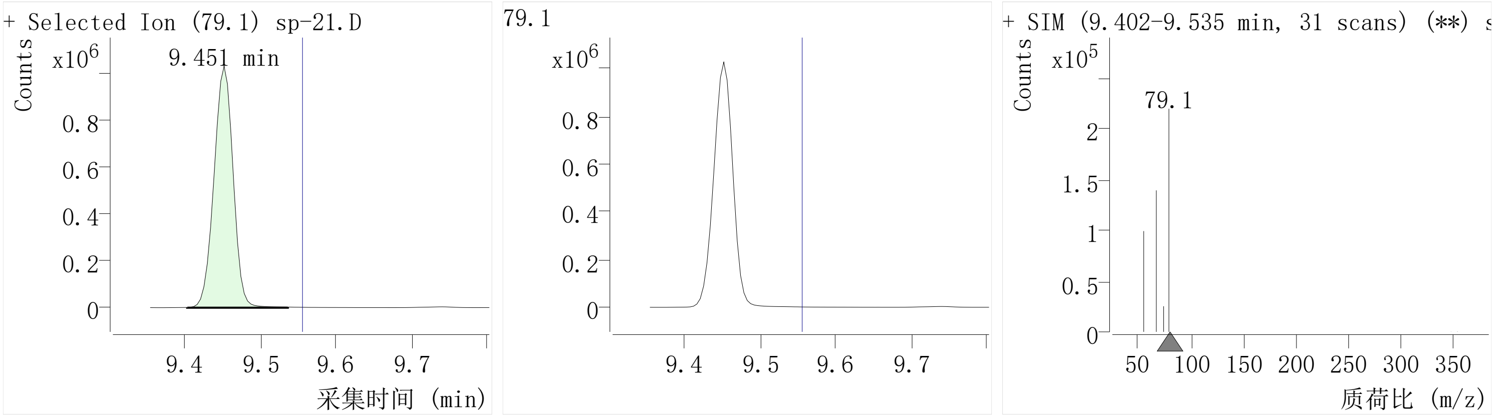

C20:0

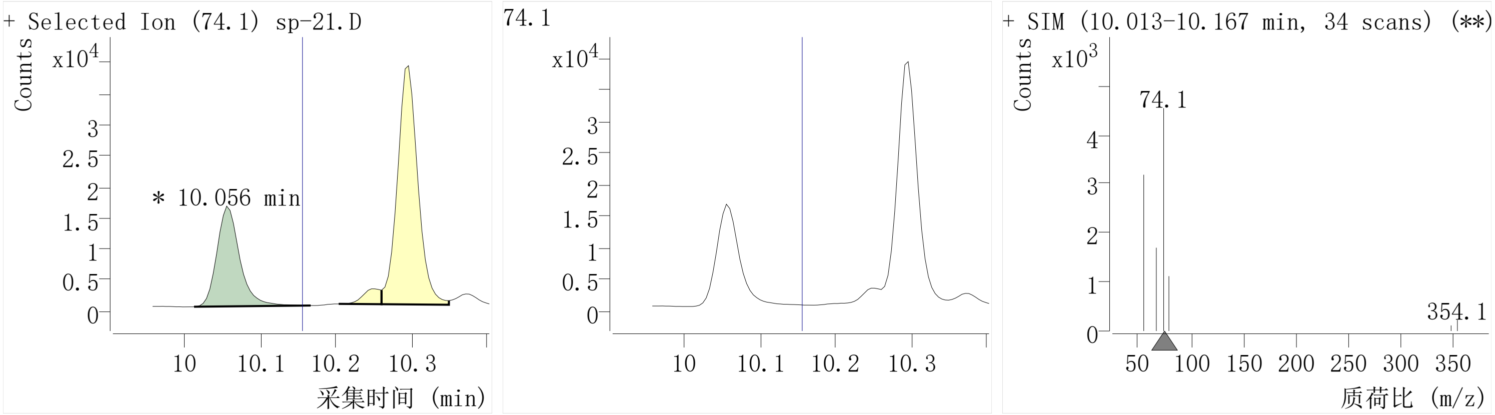

C20:1

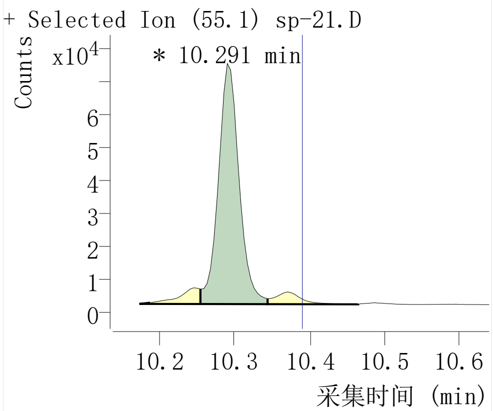

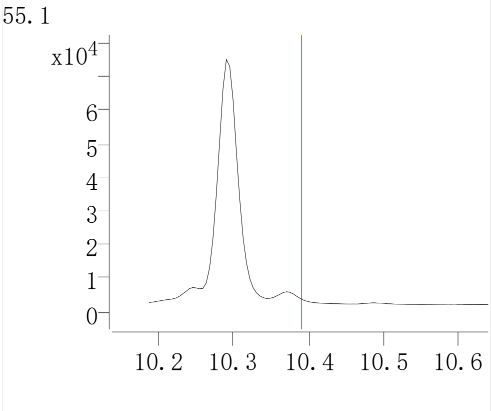

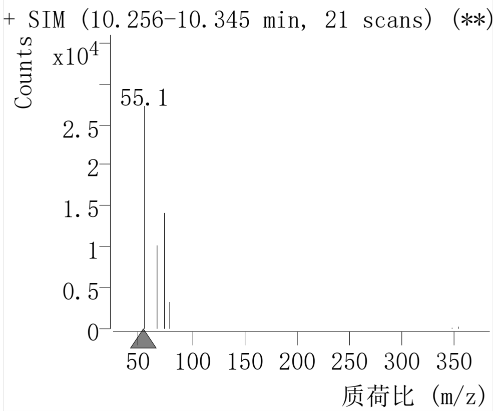

C20:2

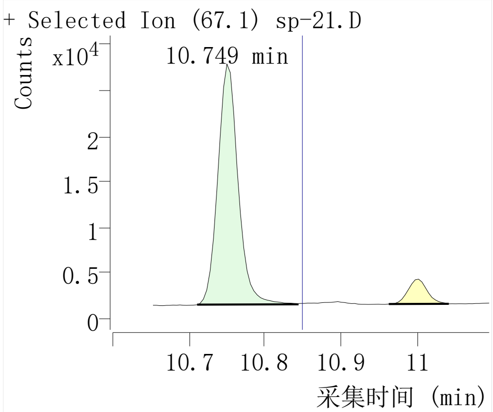

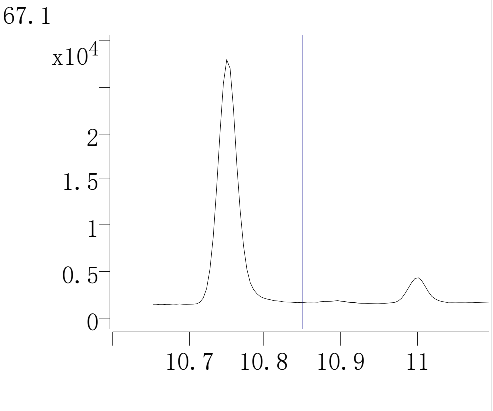

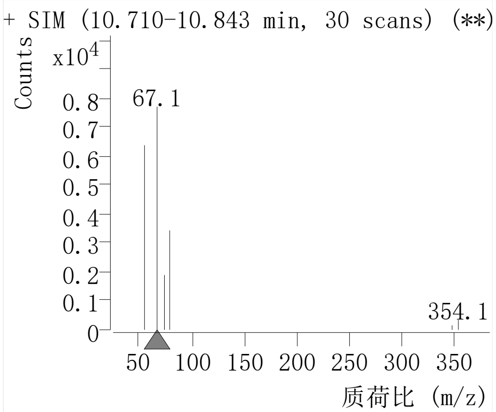

C21:0

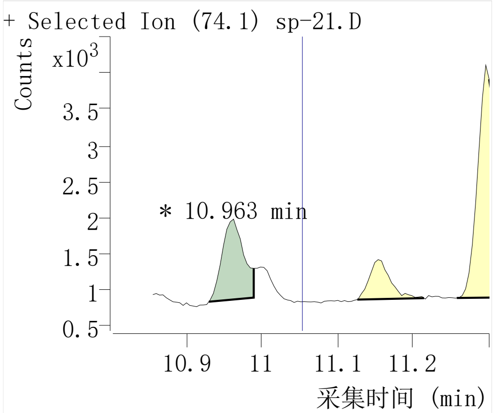

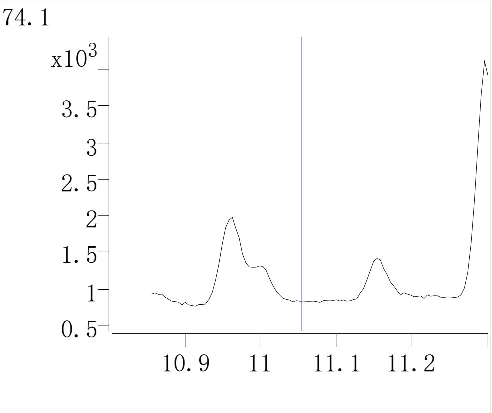

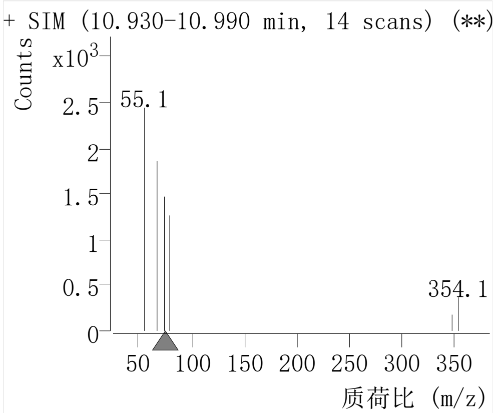

C20:3n6

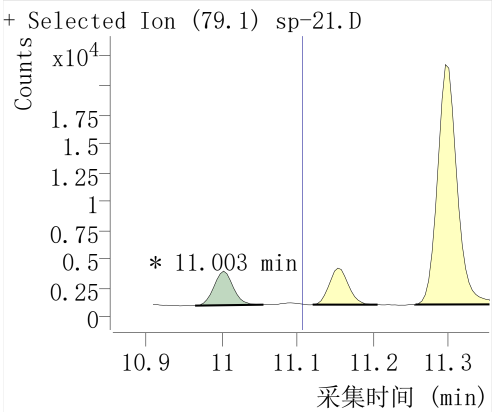

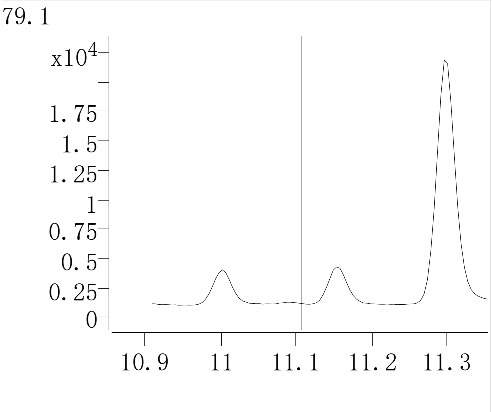

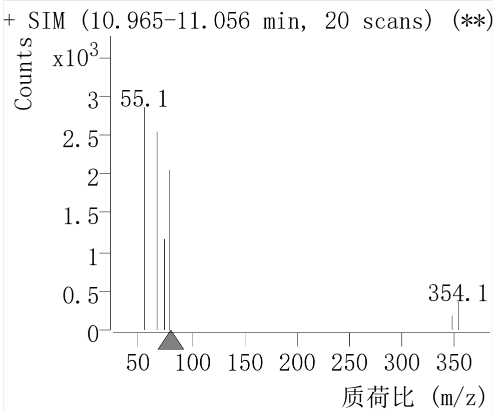

C20:4n6

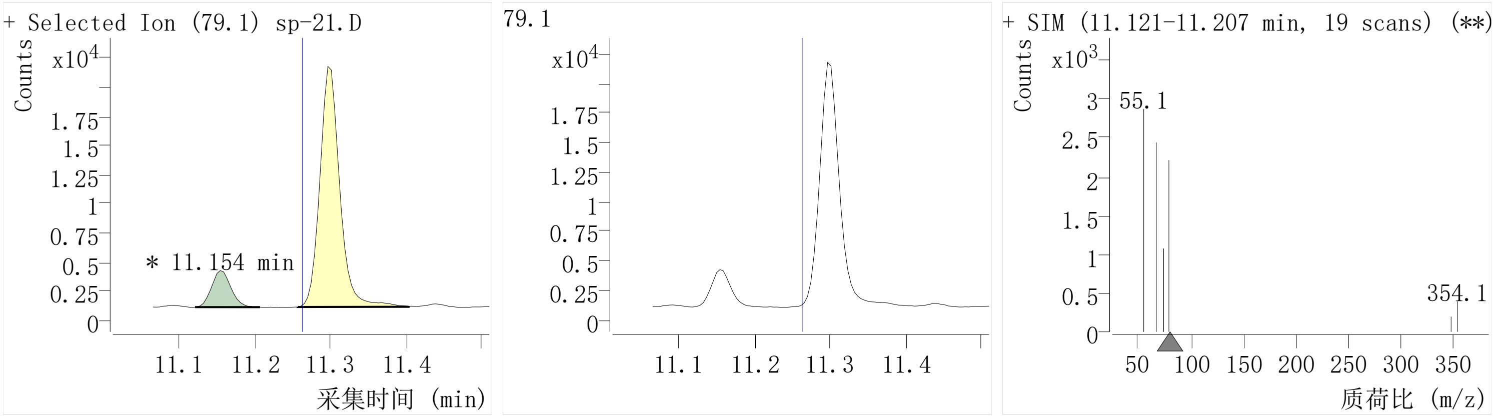

C20:3n3

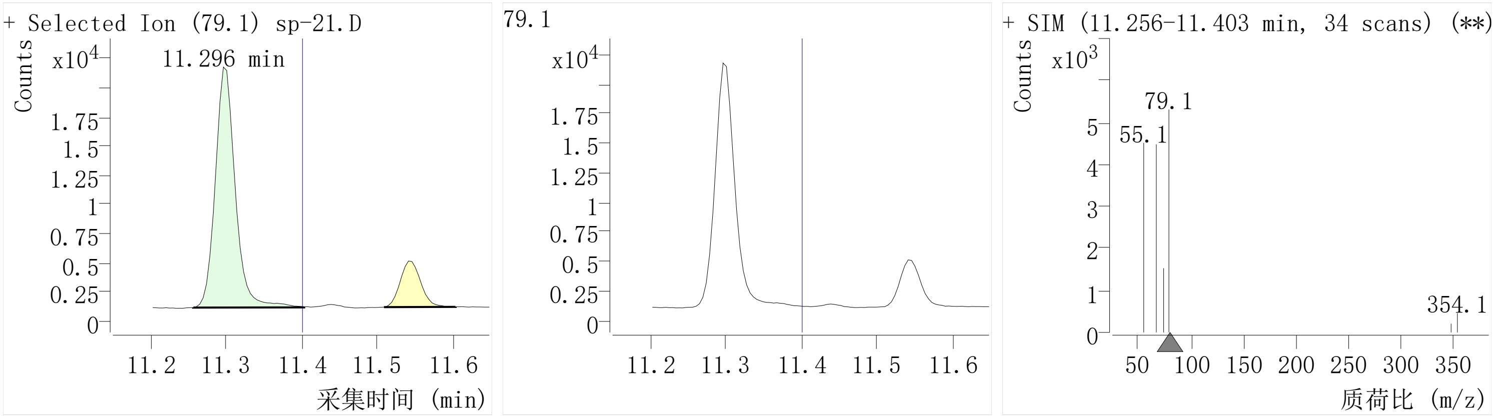

C20:5n3

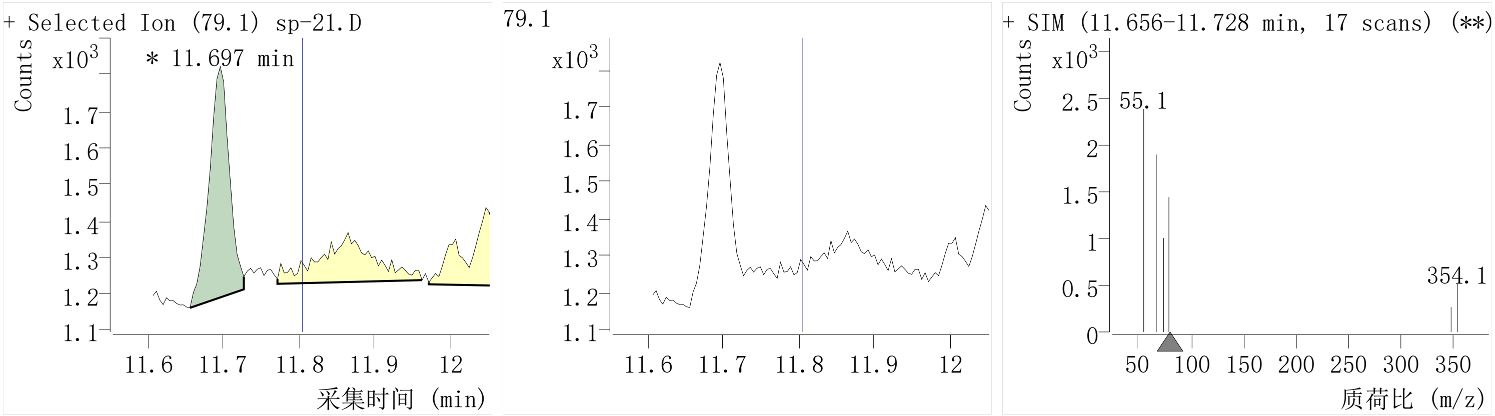

C22:0

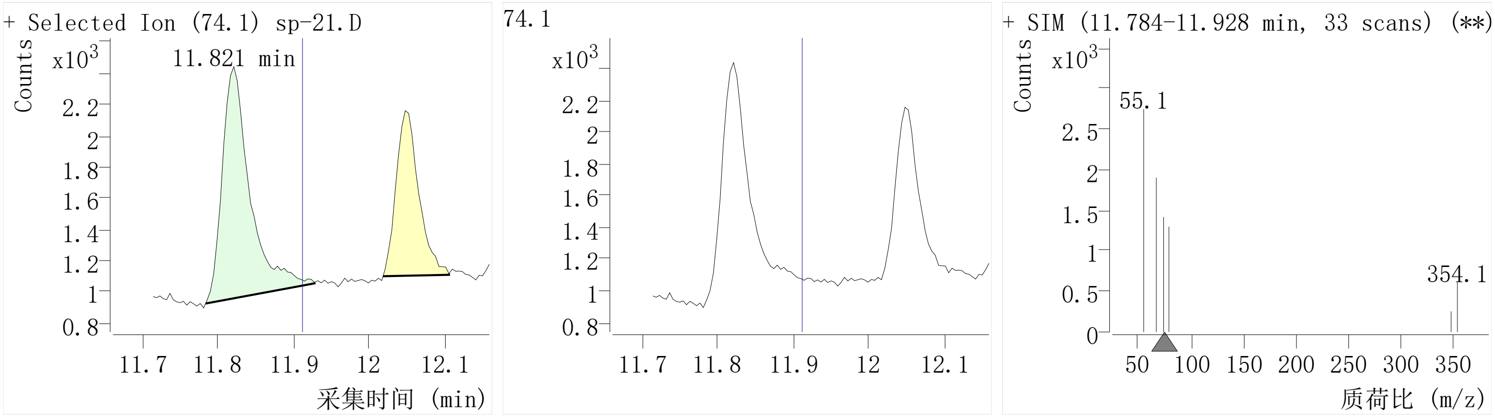

C22:1n9

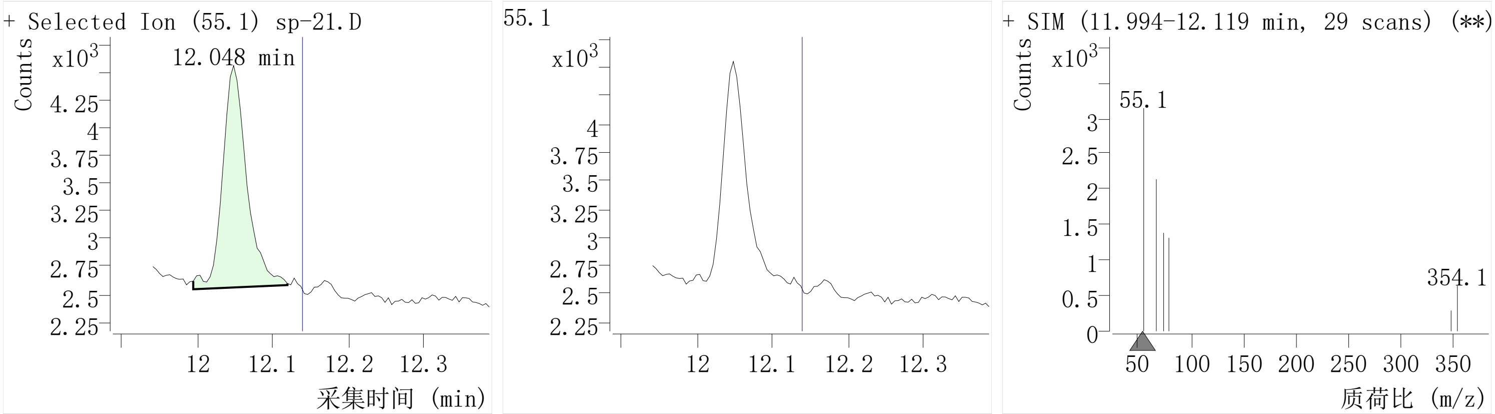

C22:2n6

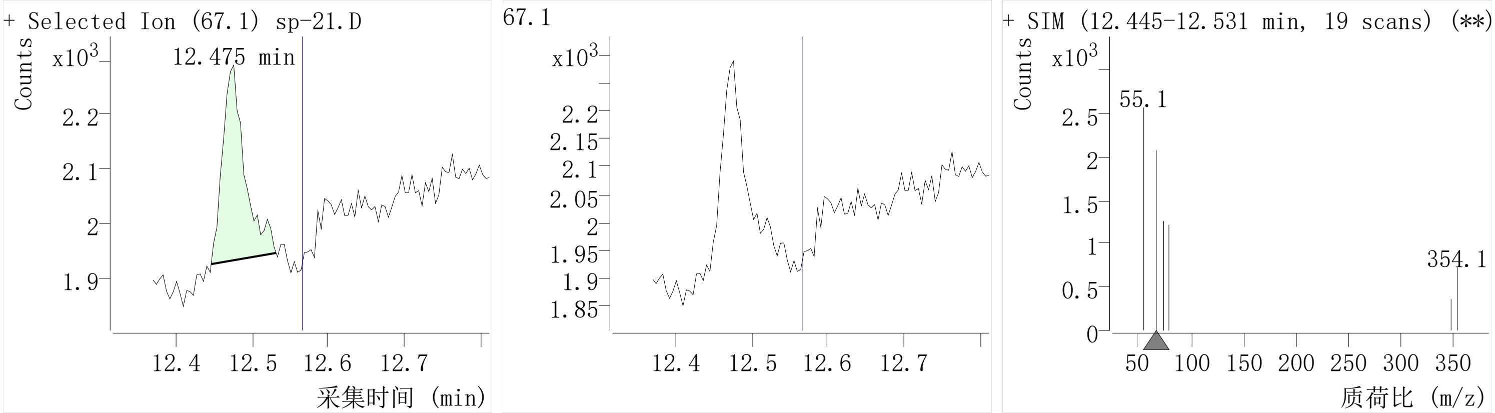

C23:0

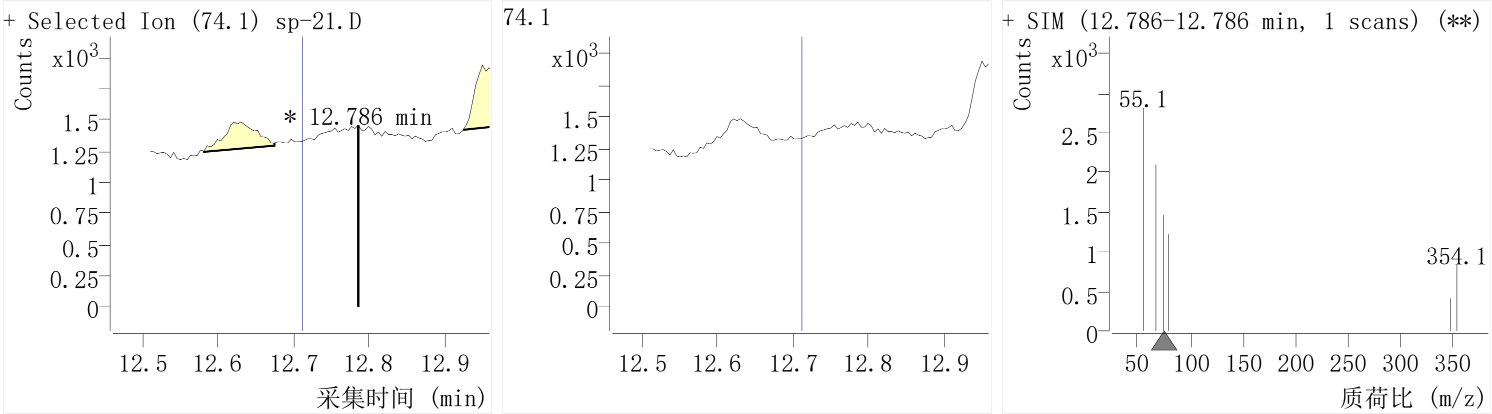

C24:0

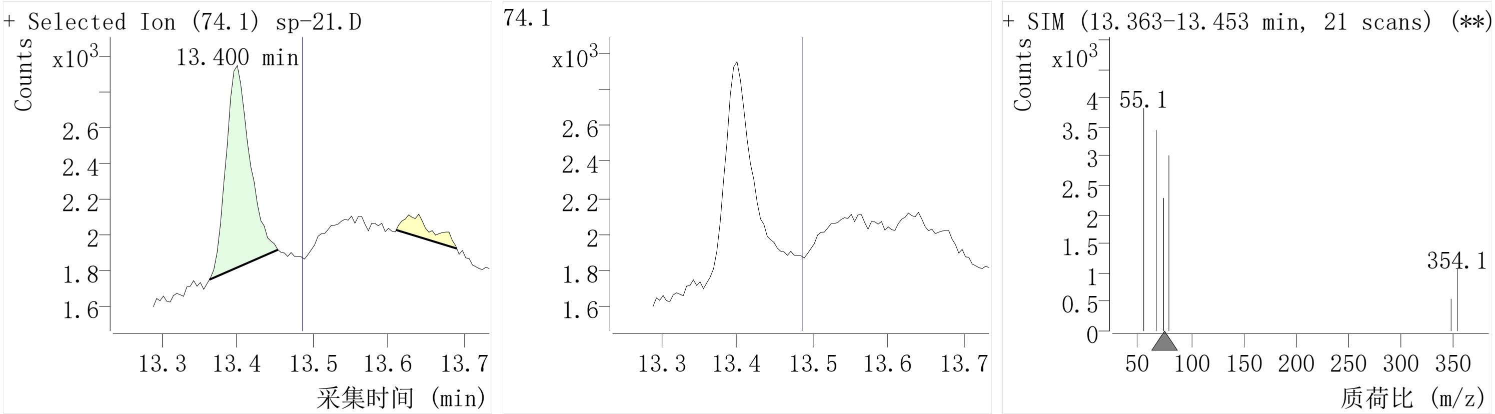

C22:6

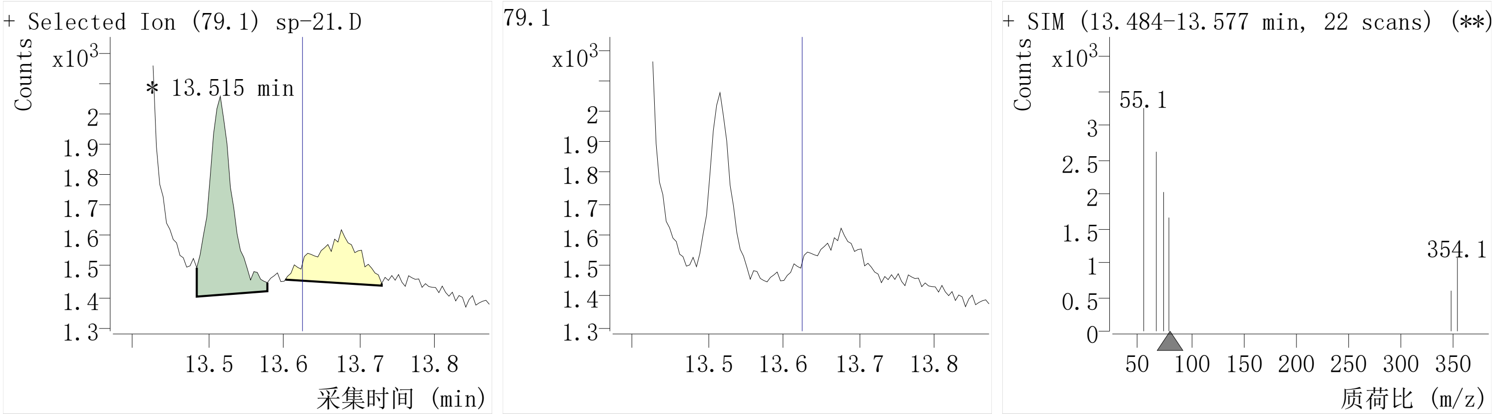
+ Selected Ion (79.1) sp-21.D
Counts
x10^3
* 13.515 min
2
1.9
1.8
1.7
1.6
1.5
1.4
1.3
13.5
13.6
13.7
13.8
采集时间 (min)
79.1
x10^3
2
1.9
1.8
1.7
1.6
1.5
1.4
1.3
13.5
13.6
13.7
13.8
+ SIM (13.484-13.577 min, 22 scans) (**)
Counts
x10^3
55.1
3
2.5
2
1.5
1
0.5
0
354.1
50
100
150
200
250
300
350
质荷比 (m/z)

C24:1

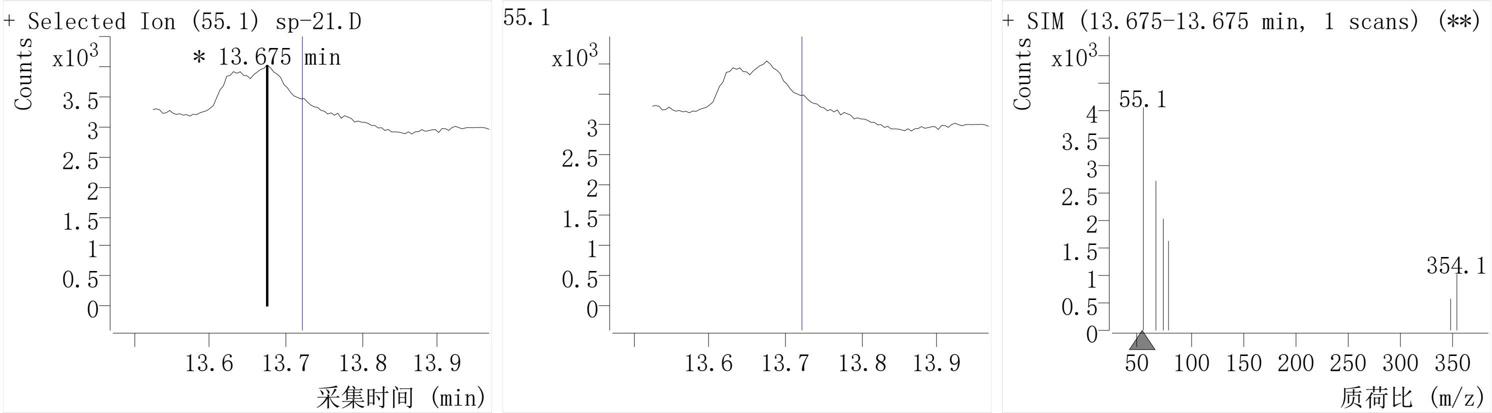
+ Selected Ion (55.1) sp-21.D
Counts
x10^3
* 13.675 min
3.5
3
2.5
2
1.5
1
0.5
0
13.6
13.7
13.8
13.9
采集时间 (min)
55.1
x10^3
3
2.5
2
1.5
1
0.5
0
13.6
13.7
13.8
13.9
+ SIM (13.675-13.675 min, 1 scans) (**)
Counts
x10^3
55.1
4
3.5
3
2.5
2
1.5
1
0.5
0
354.1
50
100
150
200
250
300
350
质荷比 (m/z)

**批处理路径**	G:\GC-MS\HX250430-4-GCMS总脂肪酸靶向检测\HX250430-4\QuantResults\HX250430-4.batch.bin		
**分析时间**	2025/5/14 16:58	**分析员姓名**	DESKTOP-M3A0GPO\omics
**报告时间**	2025/5/16 14:53:22	**报告员姓名**	DESKTOP-M3A0GPO\omics
**最近校正更新**	2025/5/14 16:58	**批处理状态**	已处理
**定量批处理版本**	10.2	**定量报告版本**	10.2

**采集时间**	2025/5/9 5:36	**数据文件**	sp-22.D
**样品类型**	样品	**样品名称**	sp-22
**稀释**	1	**采集方法**	脂肪酸

样品色谱图

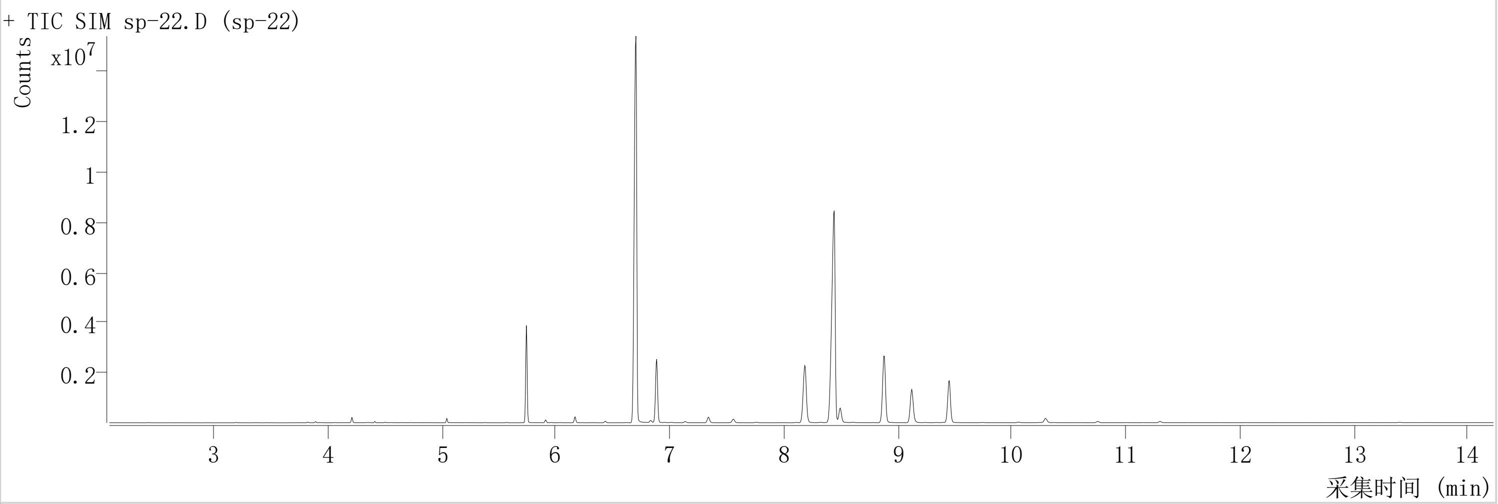

化合物	ISTD	RT	响应	ISTD 响应	响应比	最终浓度	单位
C4:0	C19:0	2.199	203	1884649	0.0001	ND	ug/ml
C6:0	C19:0	2.959	815	1884649	0.0004	0.0048	ug/ml
C8:0	C19:0	3.724	3423	1884649	0.0018	0.0142	ug/ml
C10:0	C19:0	4.413	29128	1884649	0.0155	0.2362	ug/ml
C11:0	C19:0	4.729	1349	1884649	0.0007	0.0059	ug/ml
C12:0	C19:0	5.044	100516	1884649	0.0533	0.8261	ug/ml
C13:0	C19:0	5.378	5369	1884649	0.0028	0.0301	ug/ml
C14:0	C19:0	5.743	2717254	1884649	1.4418	32.4425	ug/ml
C14:1	C19:0	5.912	53423	1884649	0.0283	1.3230	ug/ml
C15:0	C19:0	6.169	182744	1884649	0.0970	1.6892	ug/ml
C15:1	C19:0	6.432	0	1884649	0.0000	ND	ug/ml
C16:0	C19:0	6.699	15498586	1884649	8.2236	330.4794	ug/ml
C16:1	C19:0	6.885	1531725	1884649	0.8127	49.5790	ug/ml
C17:0	C19:0	7.339	253343	1884649	0.1344	2.8045	ug/ml
C17:1	C19:0	7.557	112296	1884649	0.0596	3.1803	ug/ml
C18:0	C19:0	8.184	3403211	1884649	1.8058	39.1034	ug/ml
C18:1n9t	C19:0	8.322	10498	1884649	0.0056	0.3286	ug/ml
C18:1n9c	C19:0	8.437	7970067	1884649	4.2289	286.7715	ug/ml
C18:2n6t	C19:0	8.877	0	1884649	0.0000	ND	ug/ml
C18:2n6c	C19:0	8.877	2186253	1884649	1.1600	82.4345	ug/ml
C18:3n6	C19:0	9.122	19969	1884649	0.0106	ND	ug/ml
C18:3n3	C19:0	9.451	1331090	1884649	0.7063	35.1088	ug/ml
C20:0	C19:0	10.056	40714	1884649	0.0216	0.5789	ug/ml
C20:1	C19:0	10.296	168579	1884649	0.0894	5.5521	ug/ml
C20:2	C19:0	10.754	42261	1884649	0.0224	1.4251	ug/ml
C21:0	C19:0	10.963	2159	1884649	0.0011	0.0317	ug/ml
C20:3n6	C19:0	11.003	3427	1884649	0.0018	0.1591	ug/ml
C20:4n6	C19:0	11.159	4404	1884649	0.0023	0.1810	ug/ml
C20:3n3	C19:0	11.301	39794	1884649	0.0211	1.1550	ug/ml
C20:5n3	C19:0	11.697	999	1884649	0.0005	0.0645	ug/ml

定量分析完成报告

化合物	ISTD	RT	响应	ISTD 响应	响应比	最终浓度	单位
C22:0	C19:0	11.821	4882	1884649	0.0026	0.1041	ug/ml
C22:1n9	C19:0	12.048	4980	1884649	0.0026	0.1808	ug/ml
C22:2n6	C19:0	12.470	703	1884649	0.0004	0.0664	ug/ml
C23:0	C19:0	12.782	0	1884649	0.0000	ND	ug/ml
C24:0	C19:0	13.400	3286	1884649	0.0017	0.0850	ug/ml
C22:6	C19:0	13.515	1175	1884649	0.0006	0.0513	ug/ml
C24:1	C19:0	13.675	0	1884649	0.0000	ND	ug/ml

C4:0

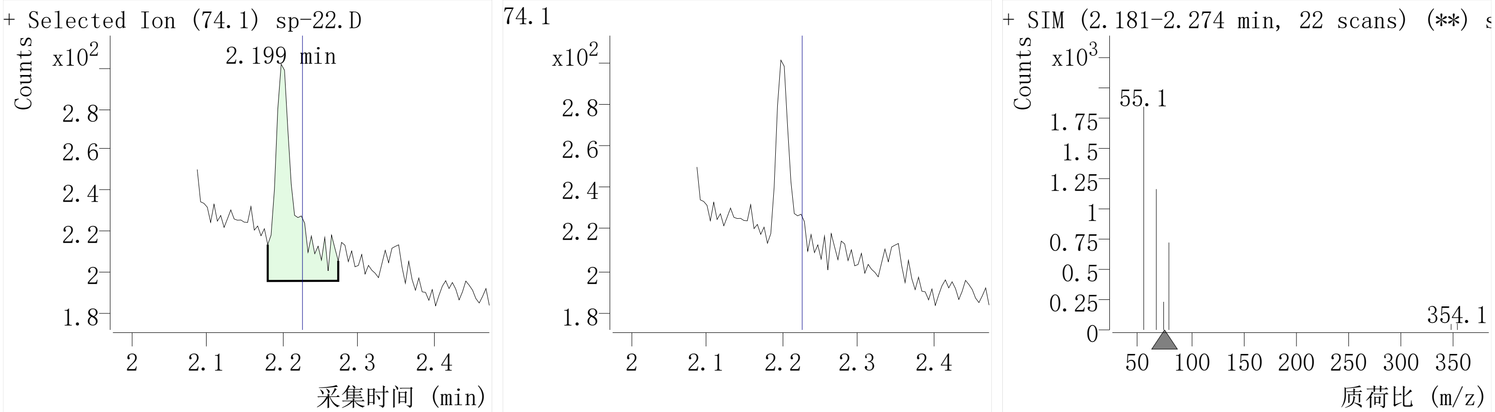

C6:0

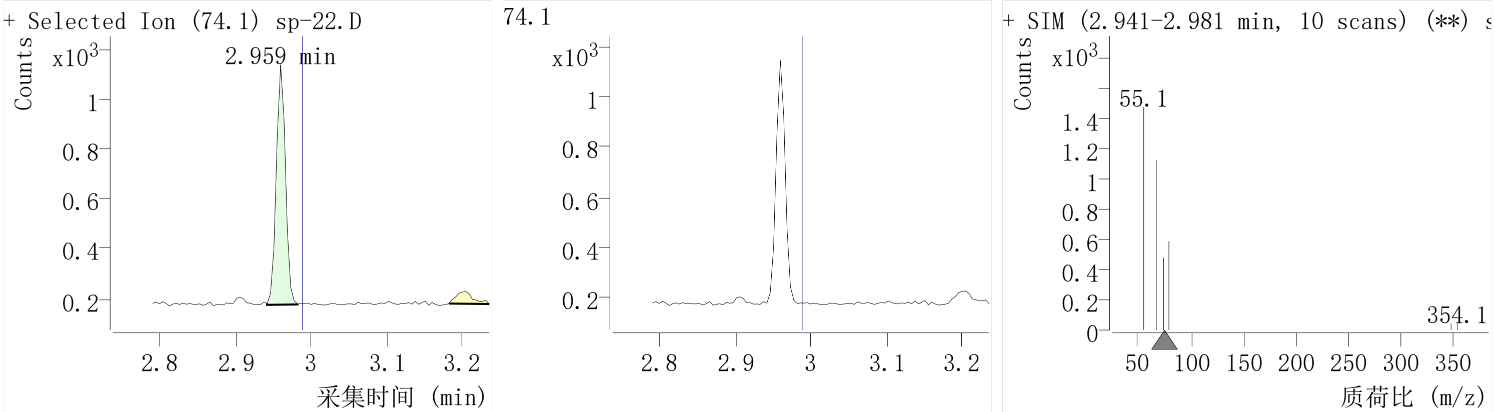

C8:0

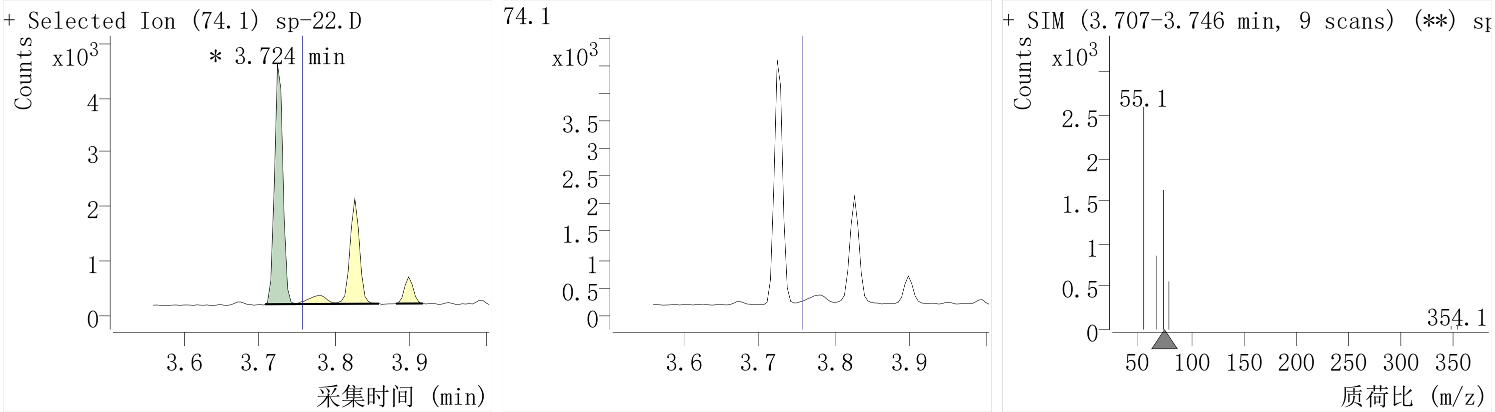

C10:0

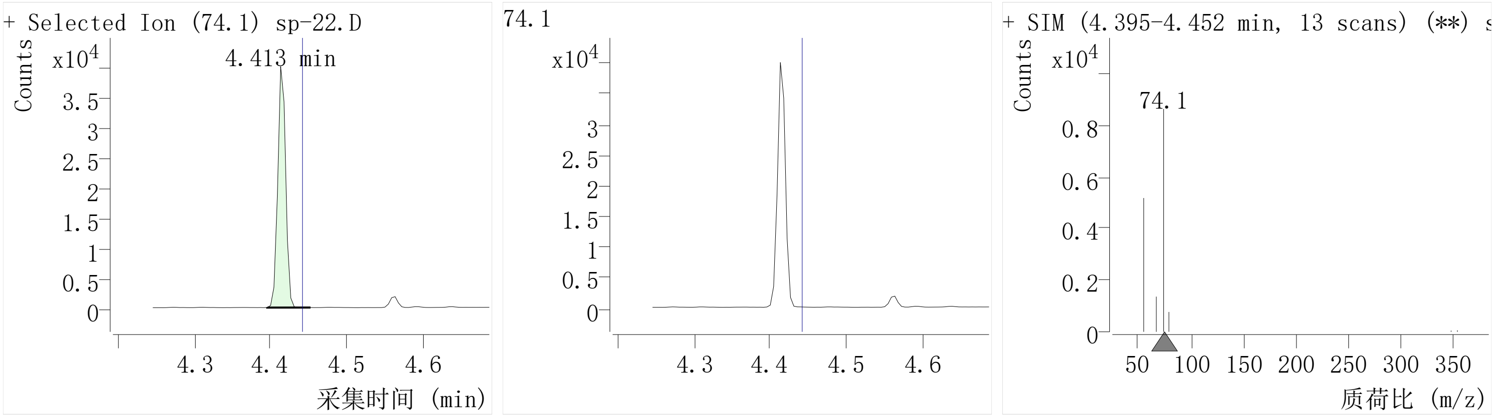

C11:0

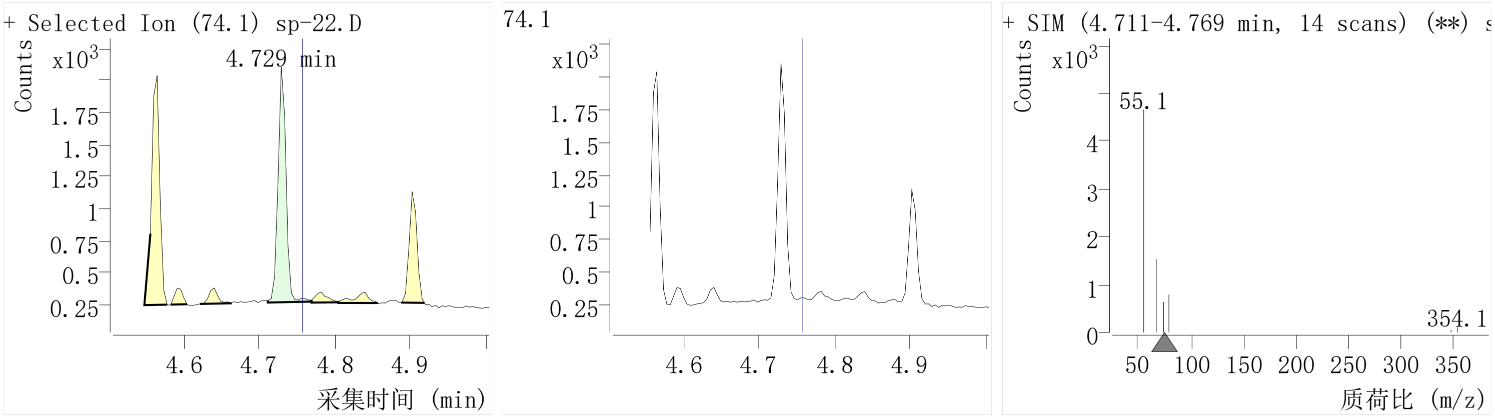

C12:0

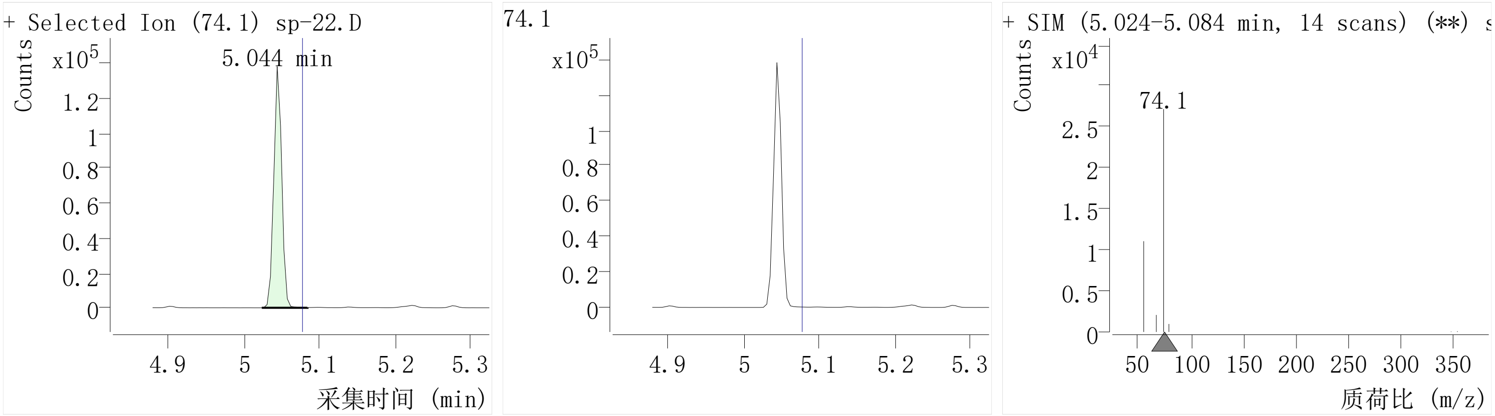

C13:0

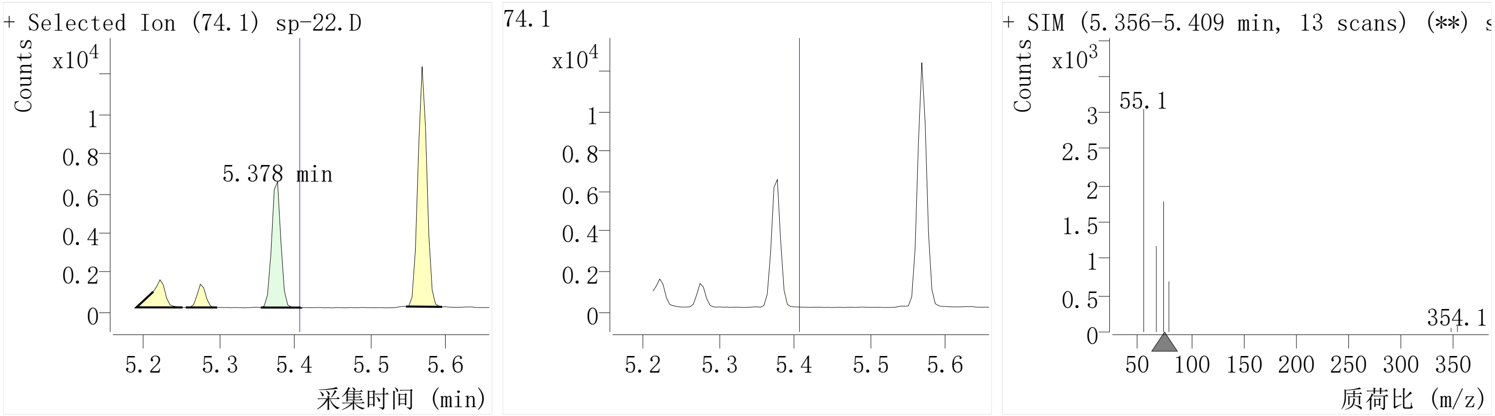

C14:0

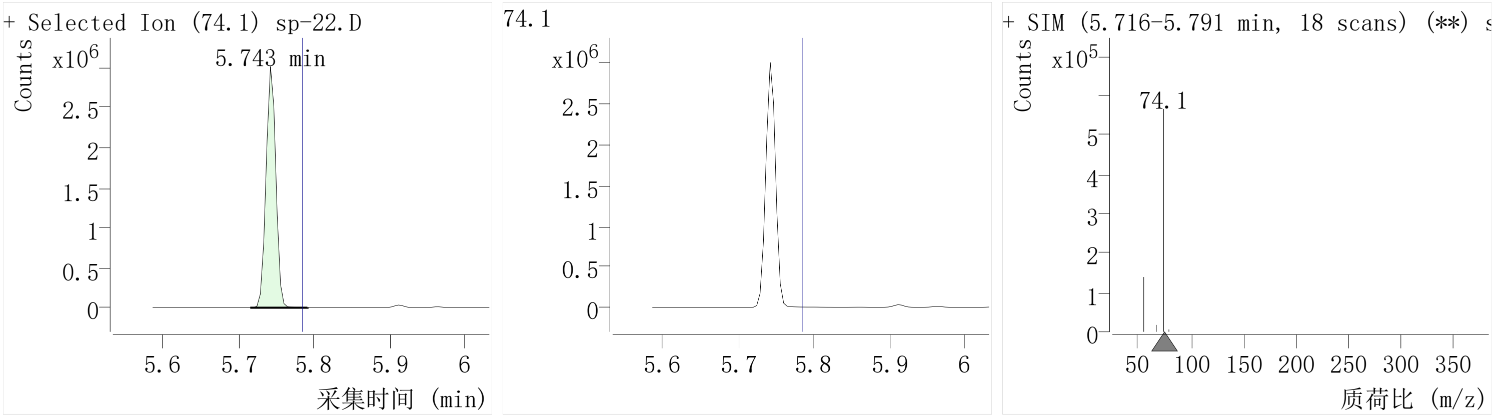

C14:1

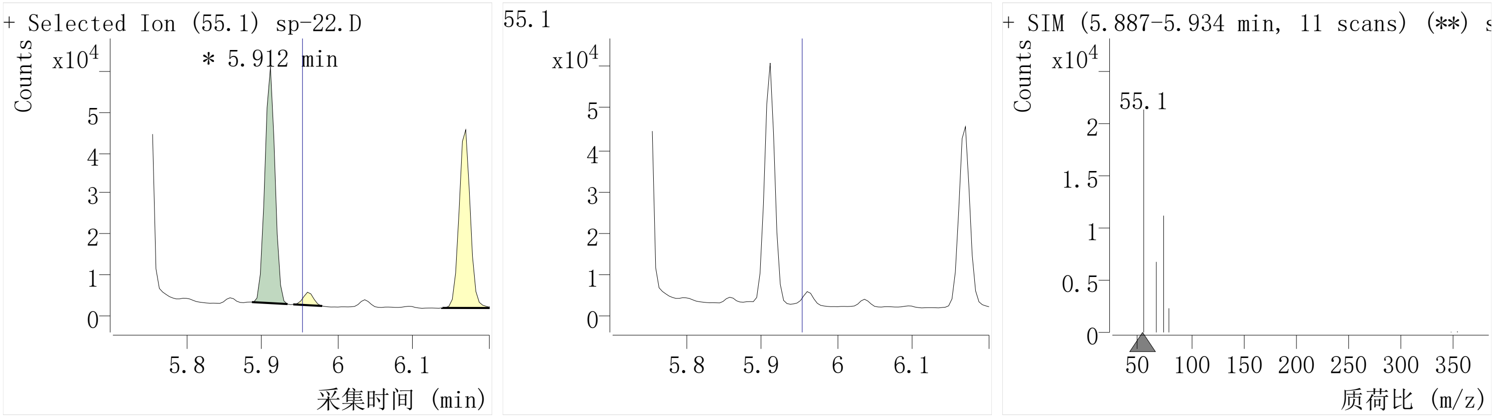

C15:0

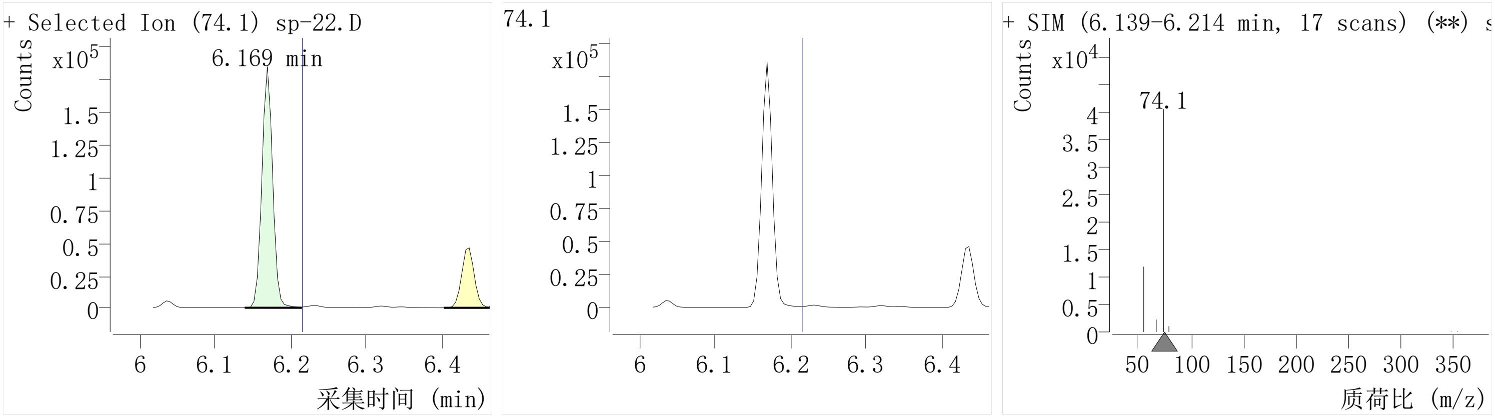

C15:1

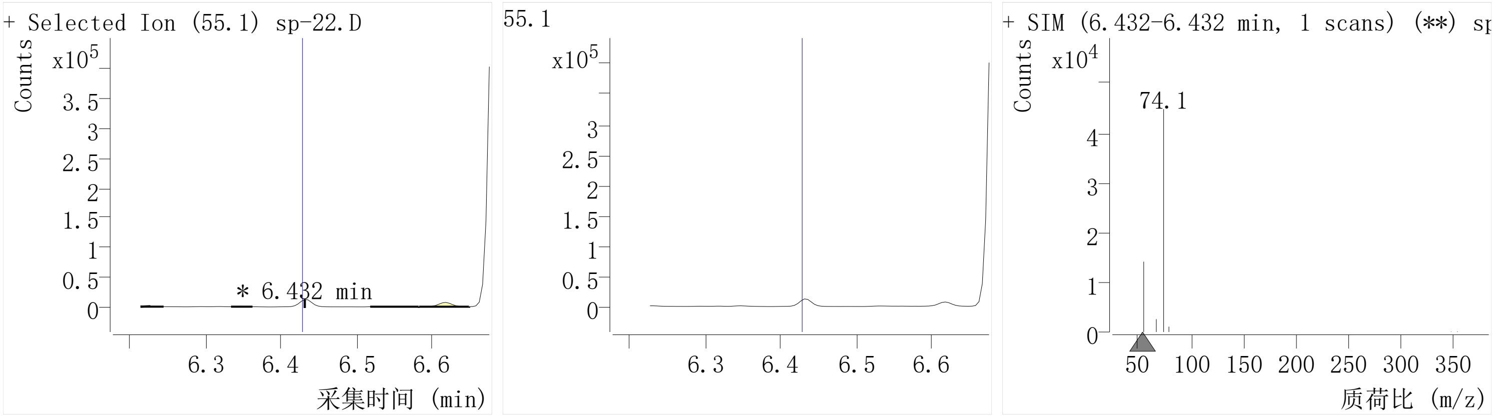

C16:0

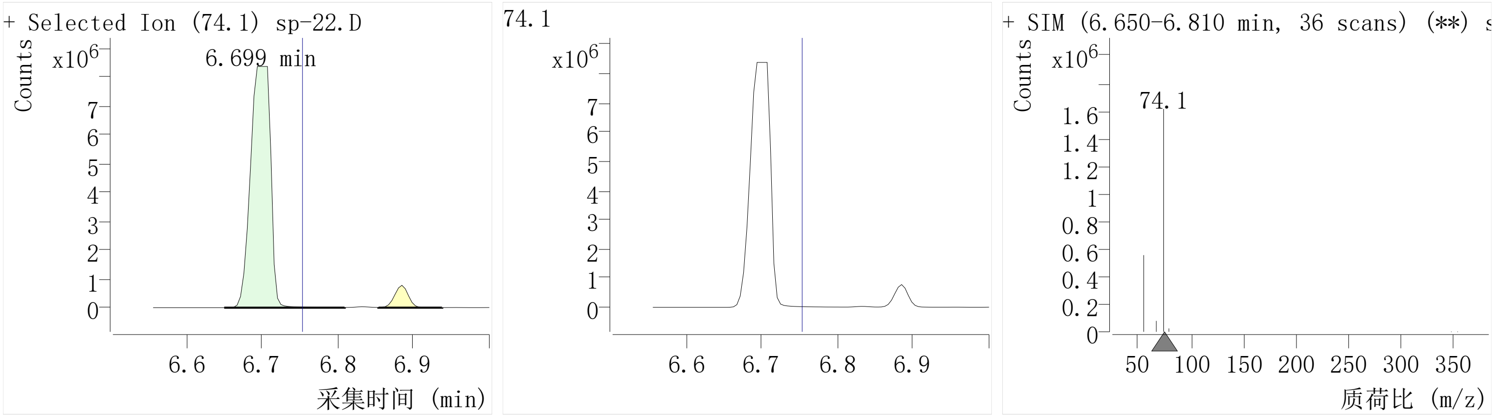

C16:1

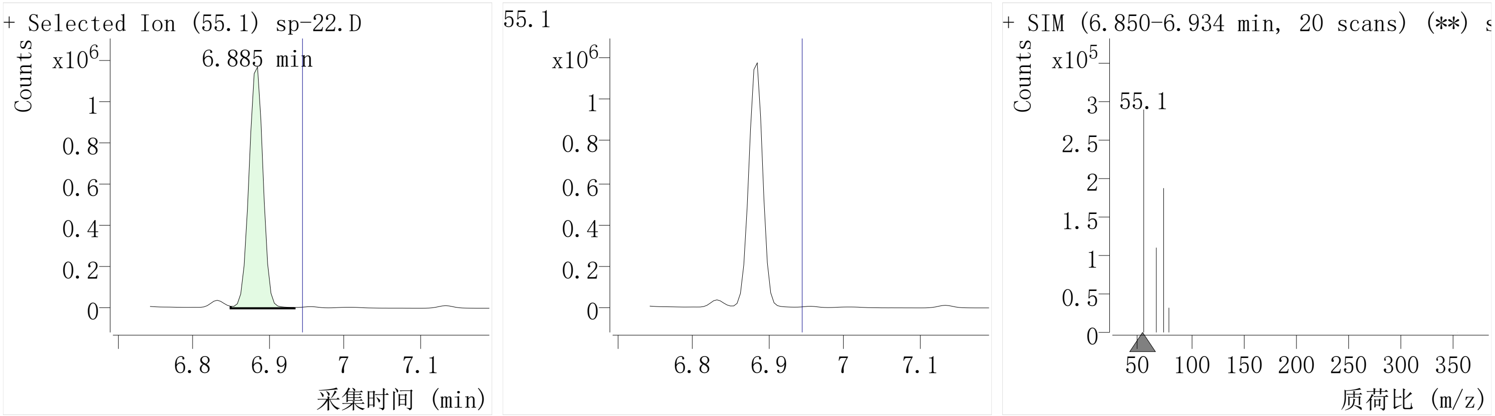

C17:0

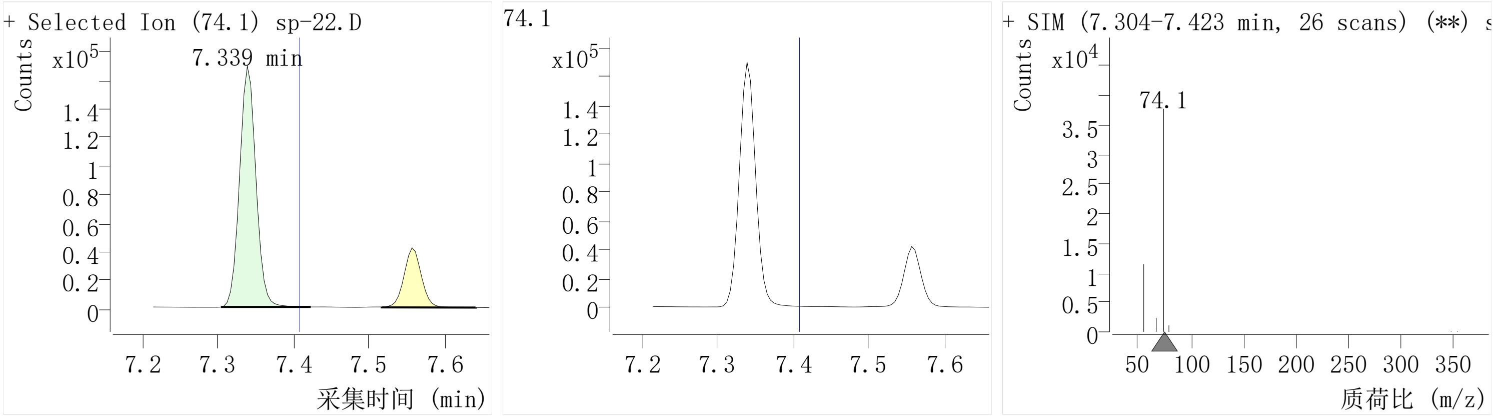

C17:1

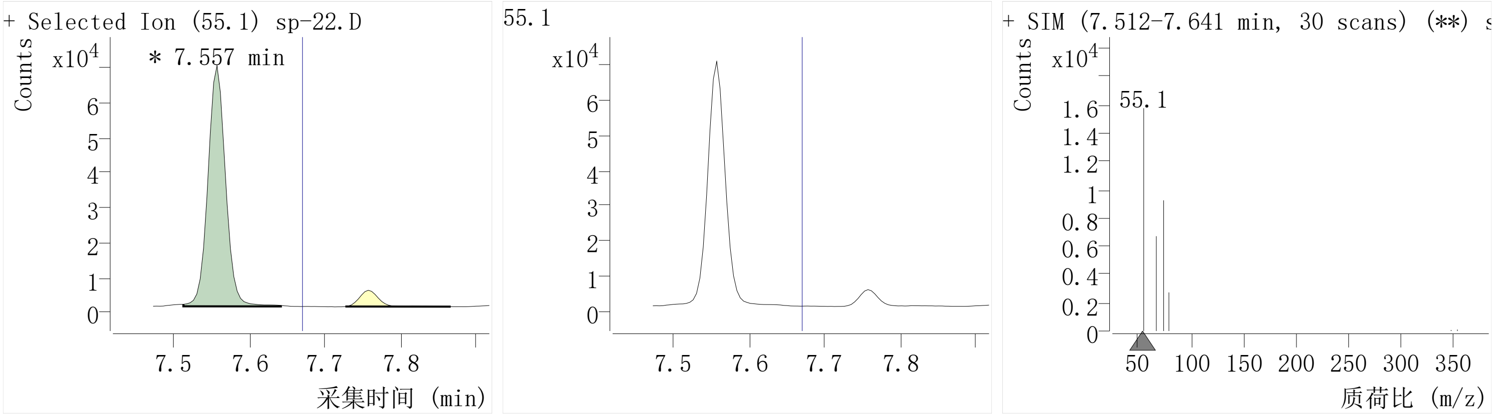

C18:0

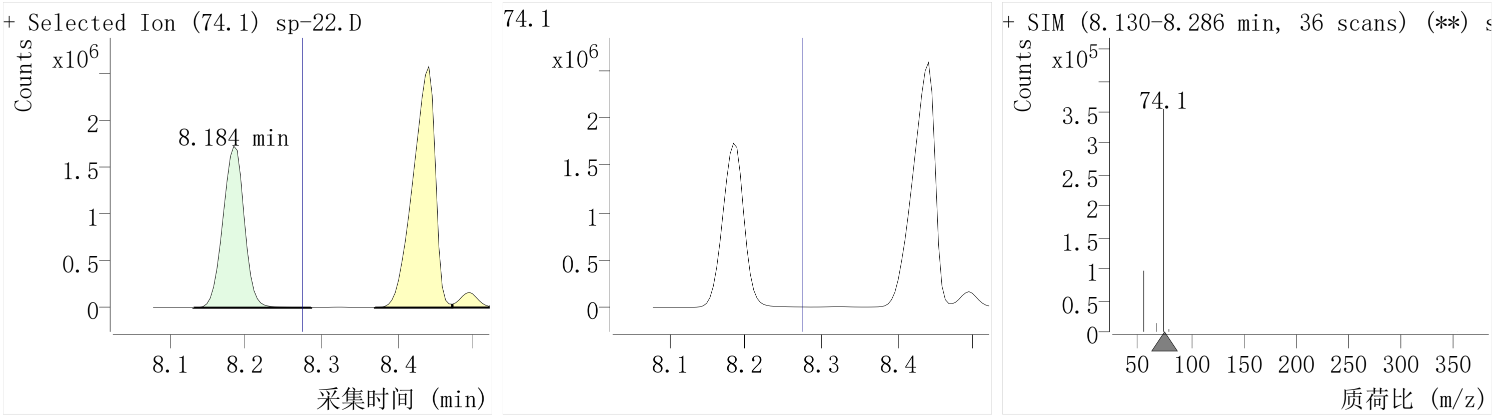

C18:1n9t

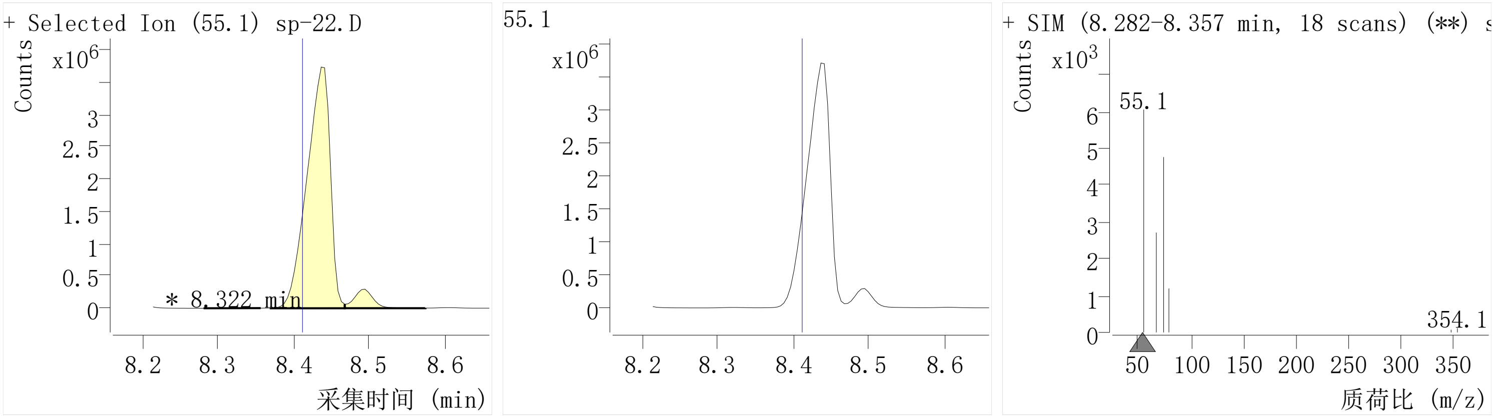

C18:1n9c

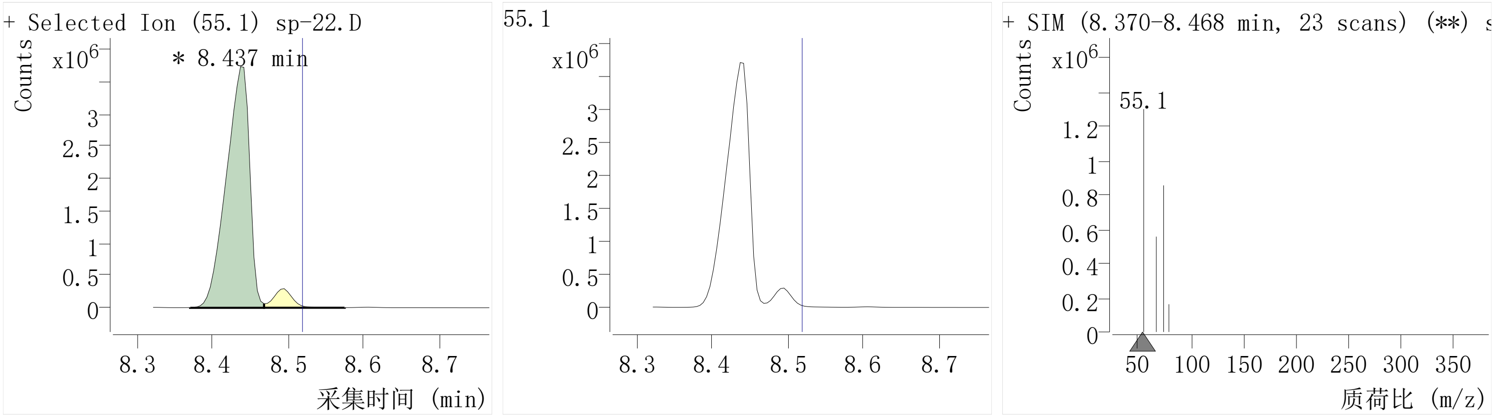

C18:2n6t

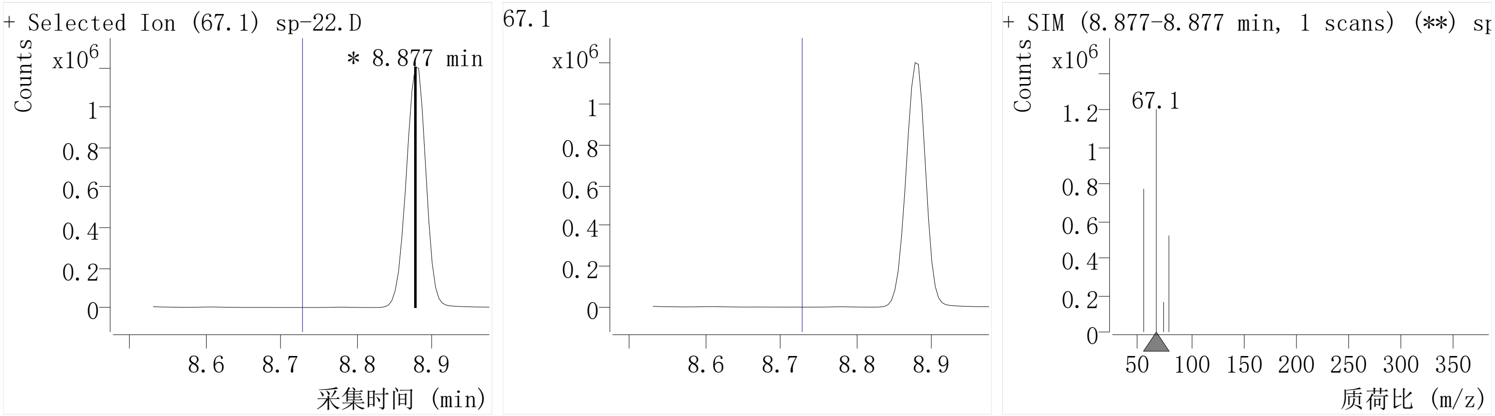

C18:2n6c

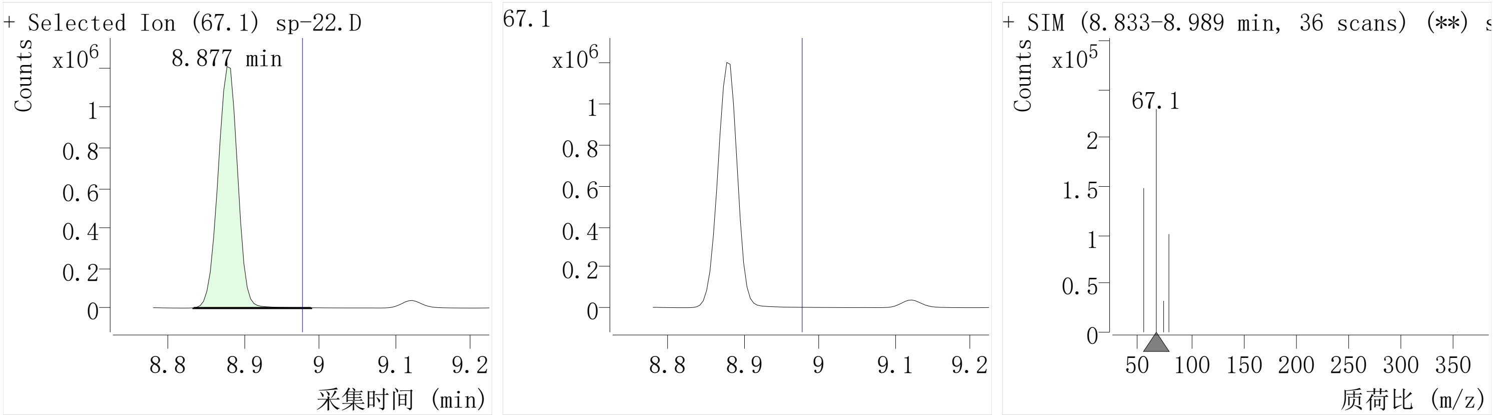

C18:3n6

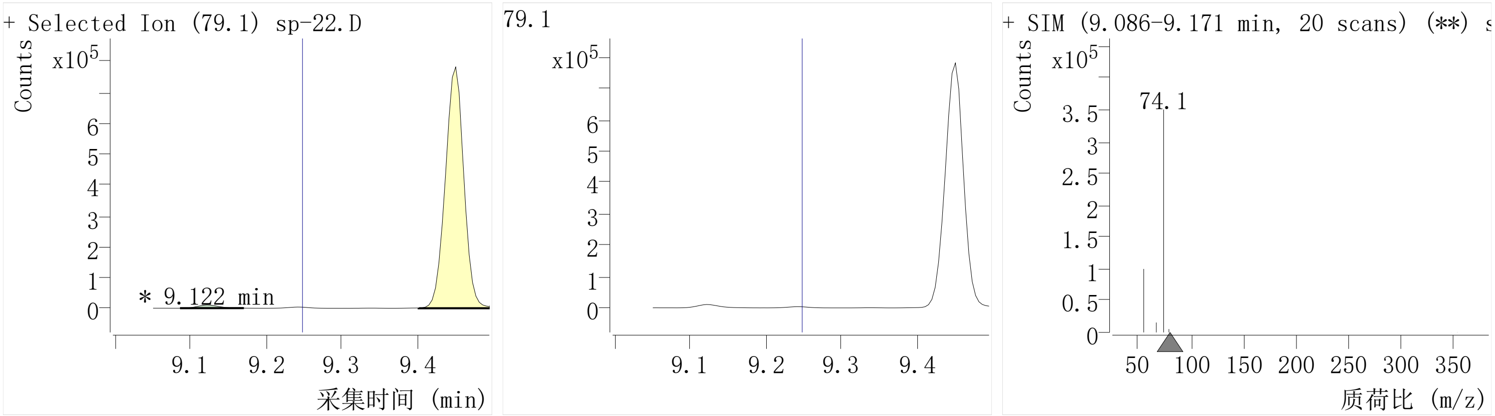

C18:3n3

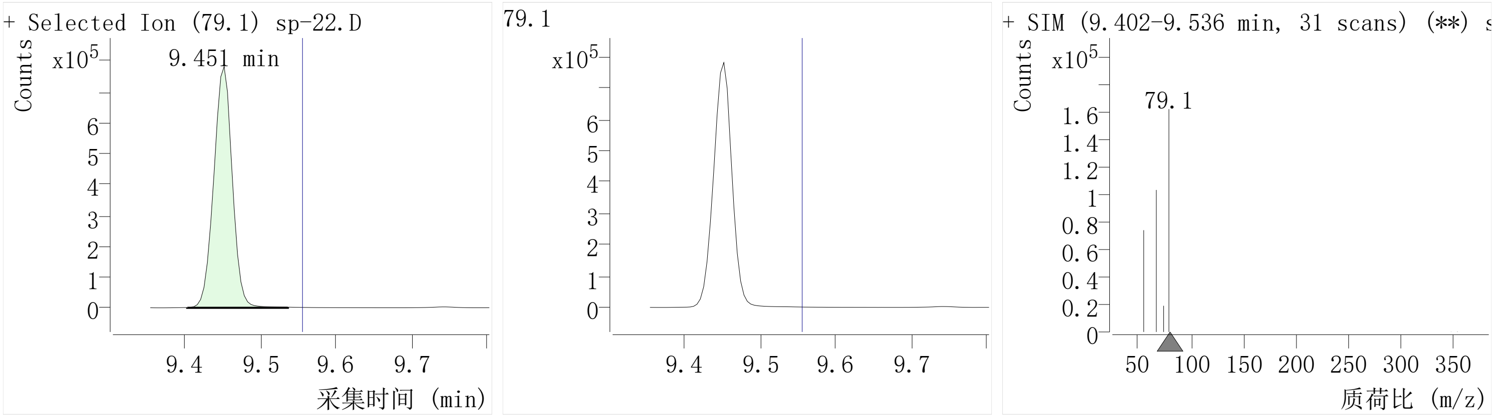

C20:0

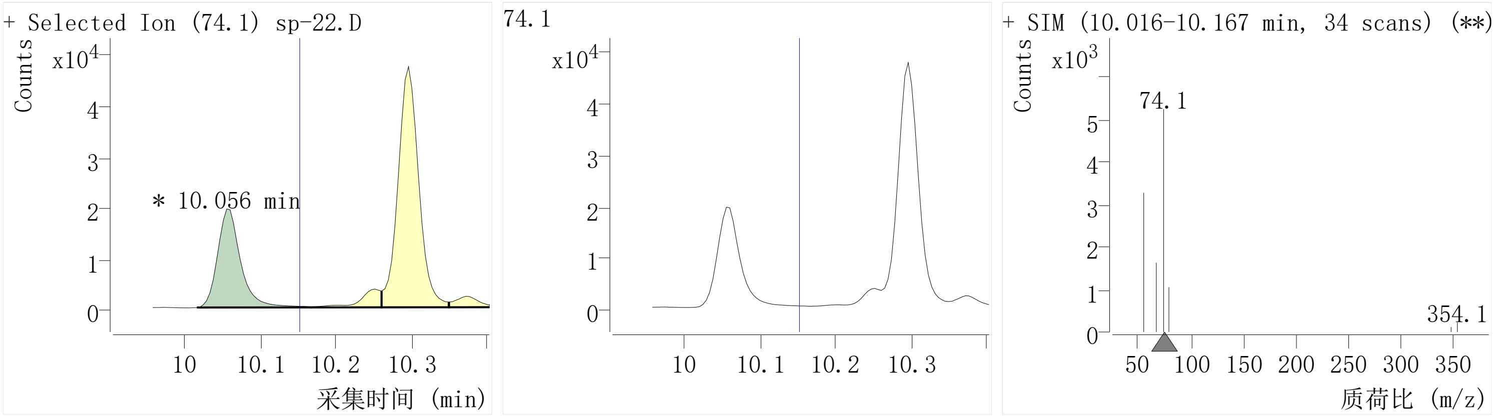

生成时间 2025/5/16 14:52

C20:1

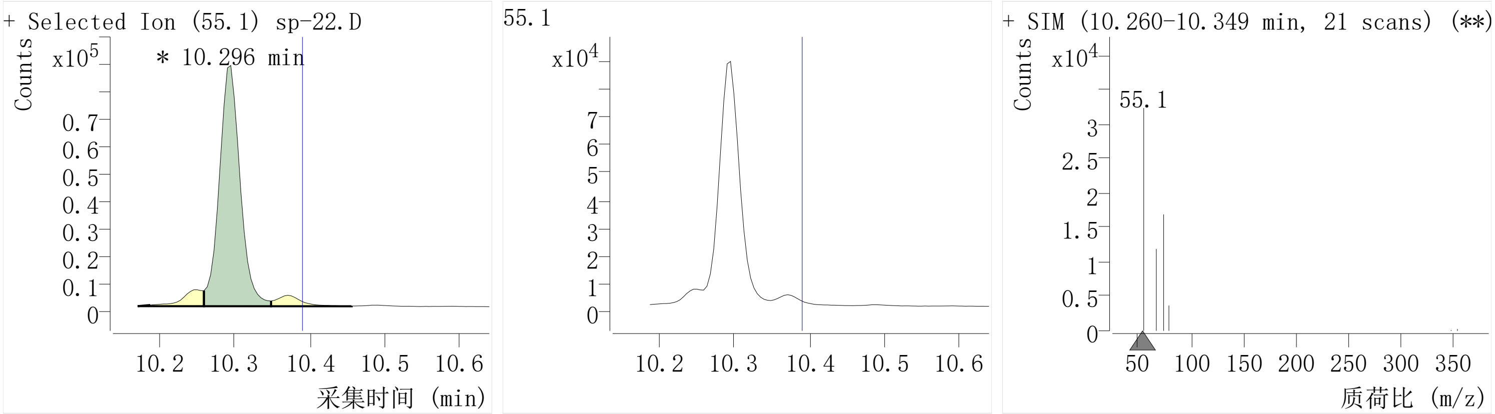

C20:2

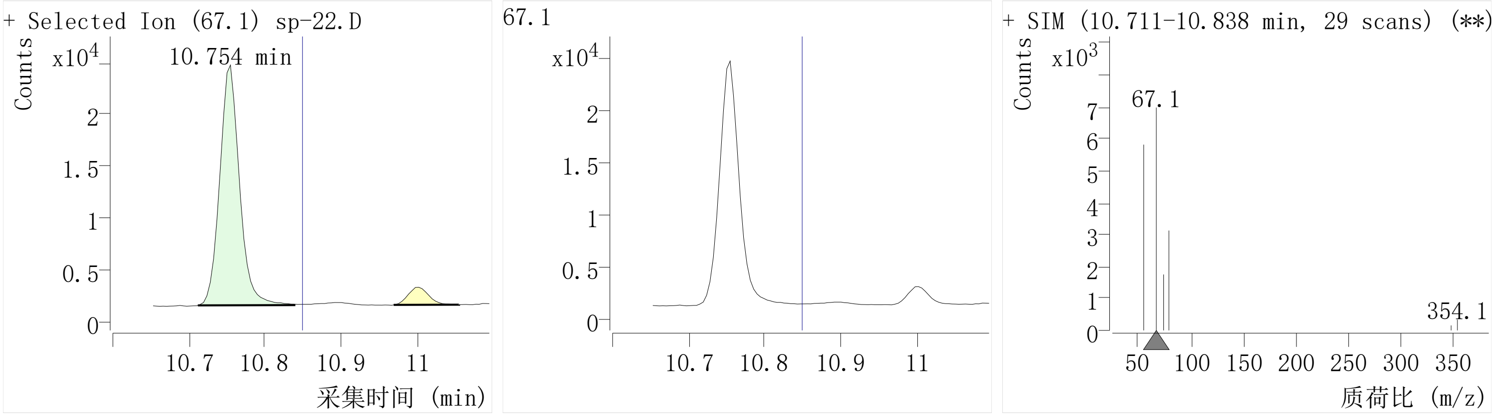

C21:0

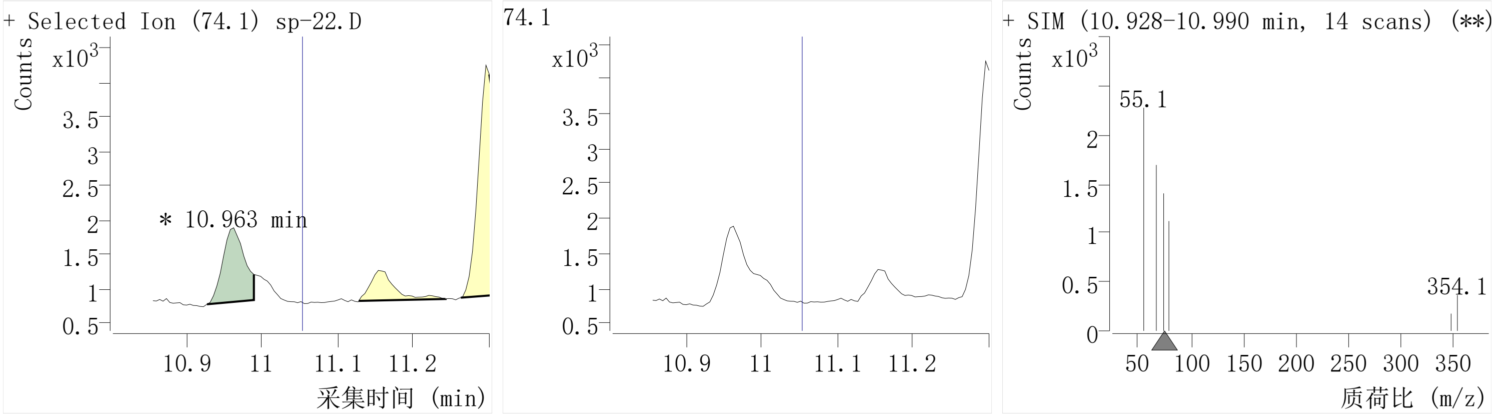

C20:3n6

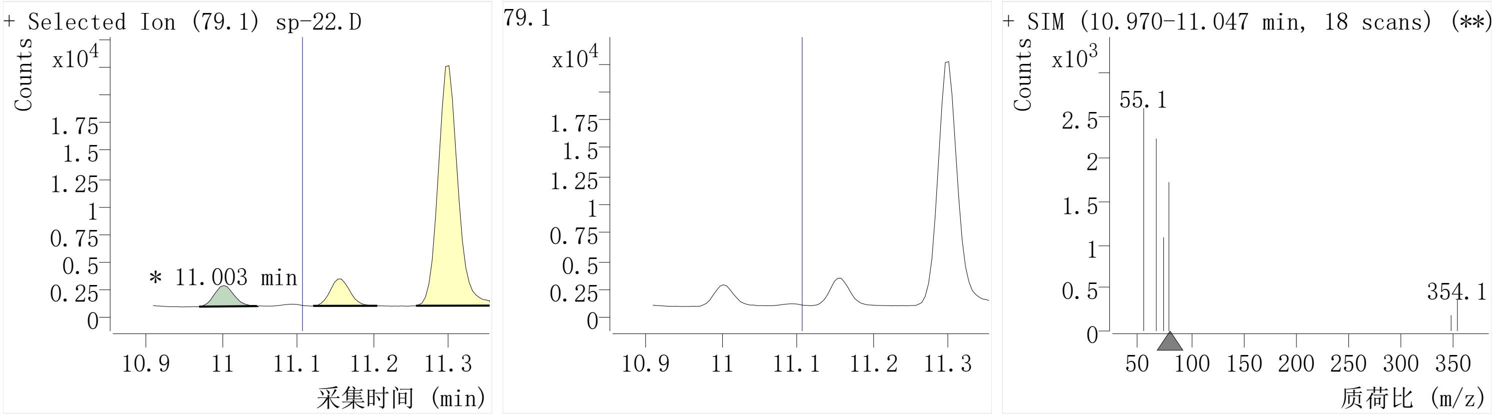

C20:4n6

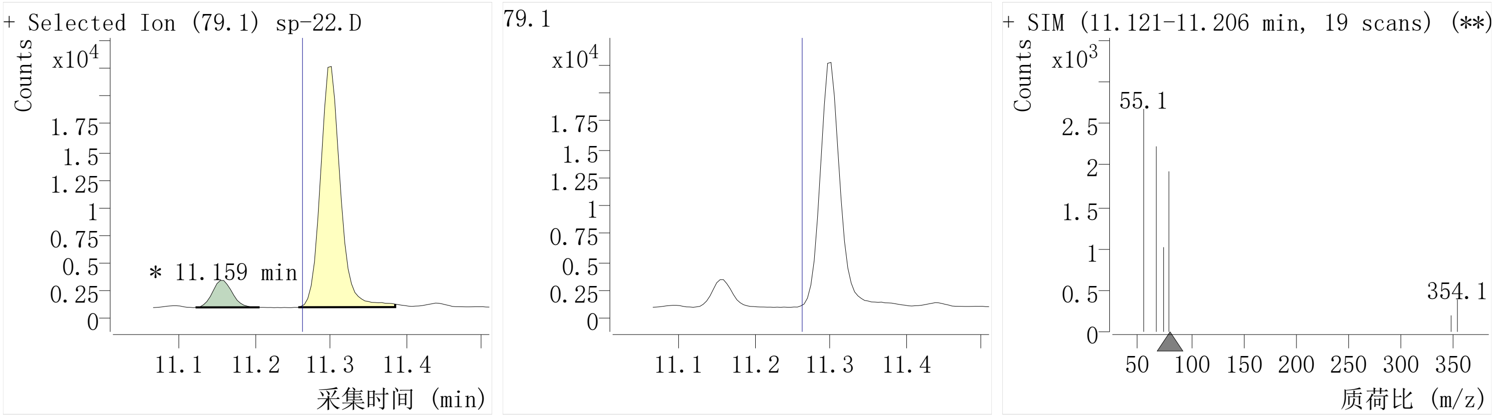

C20:3n3

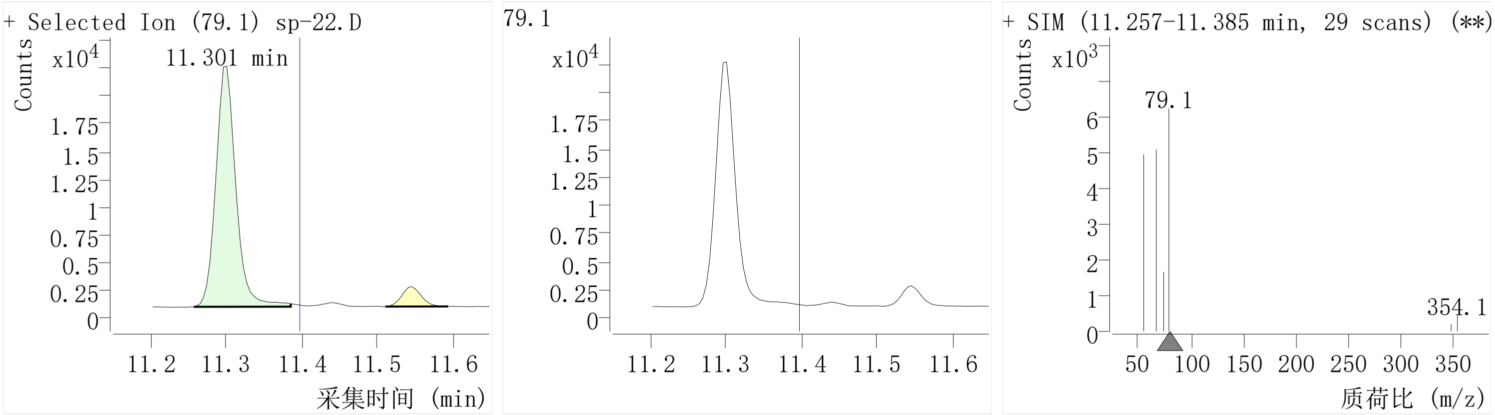

C20:5n3

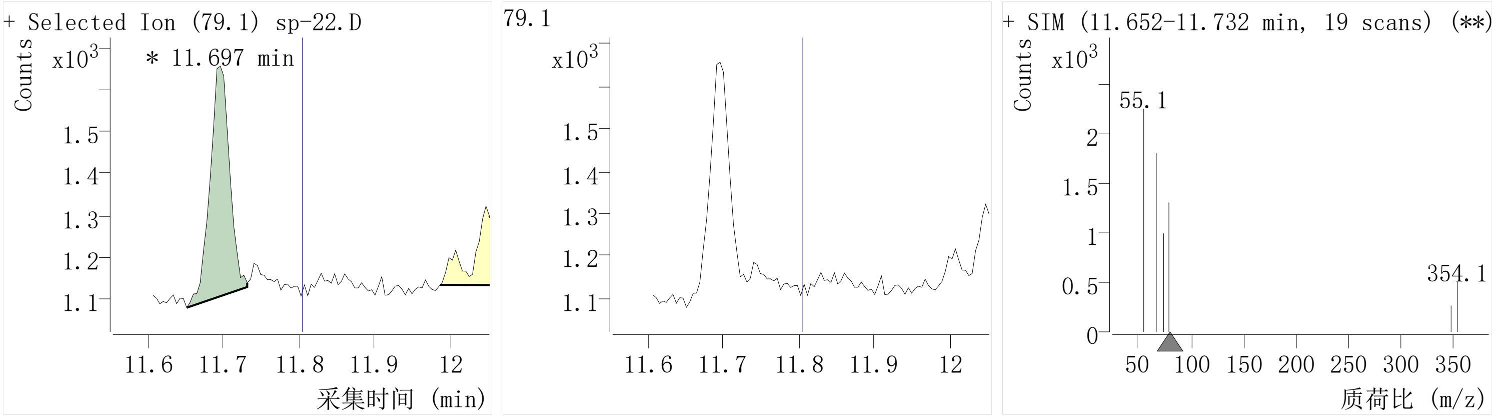

C22:0

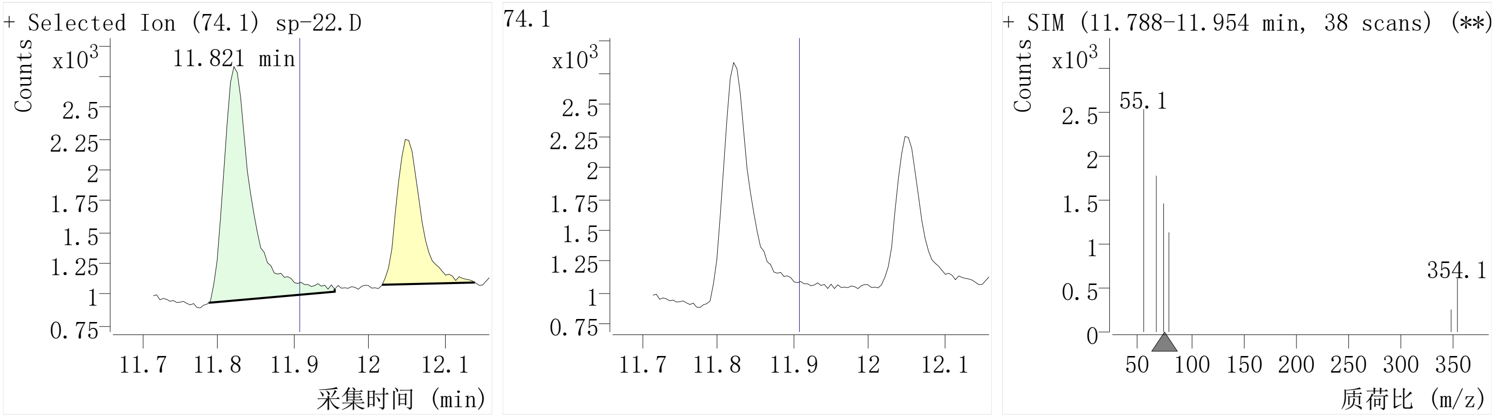

C22:1n9

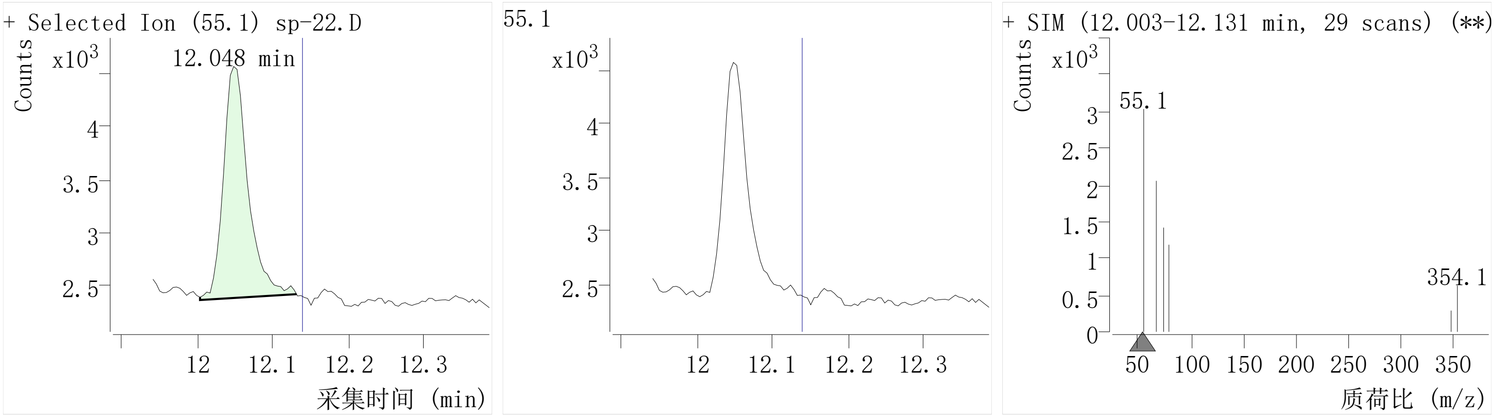

C22:2n6

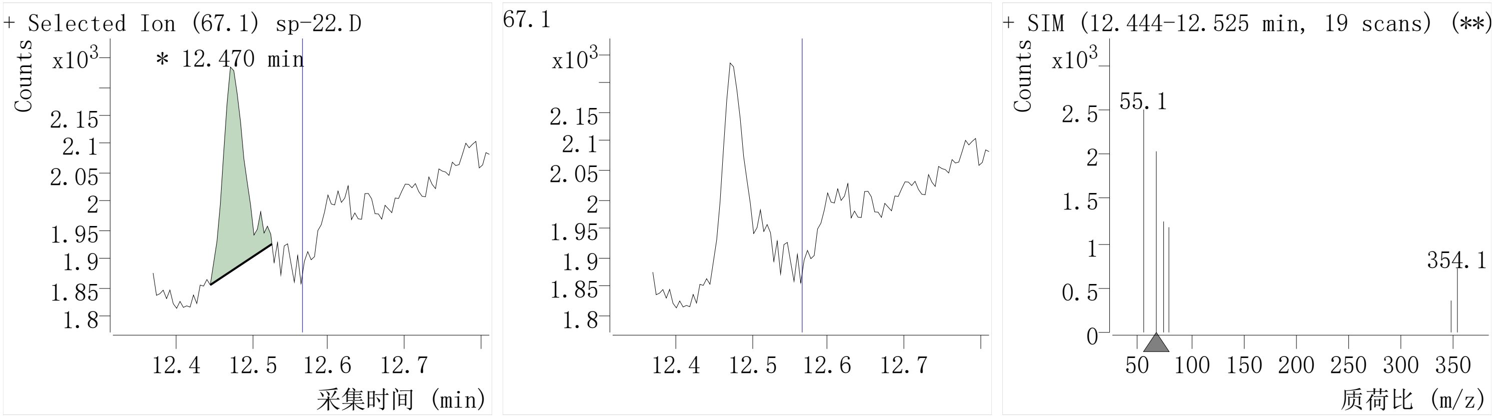

C23:0

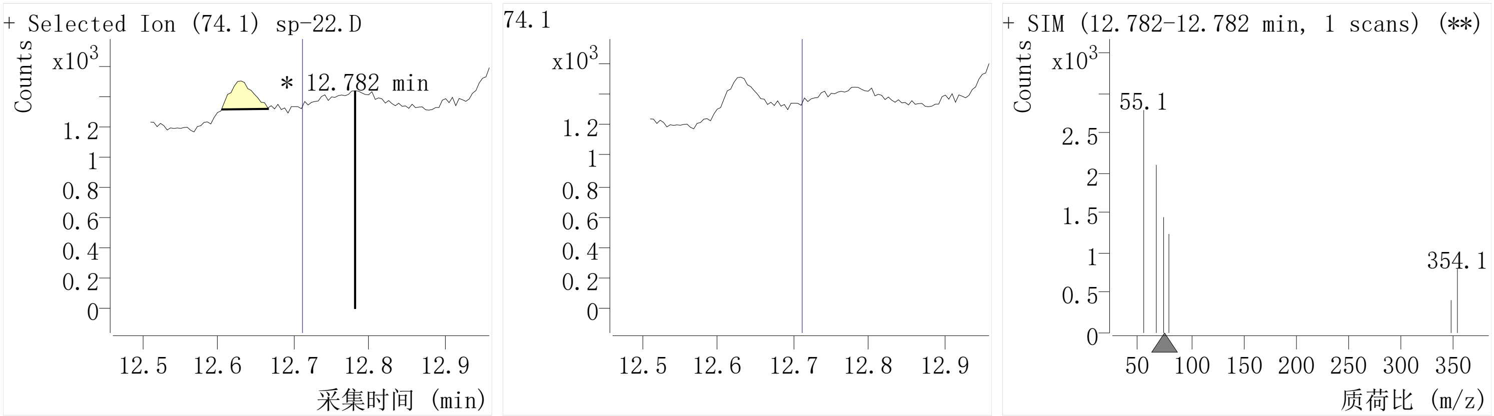

C24:0

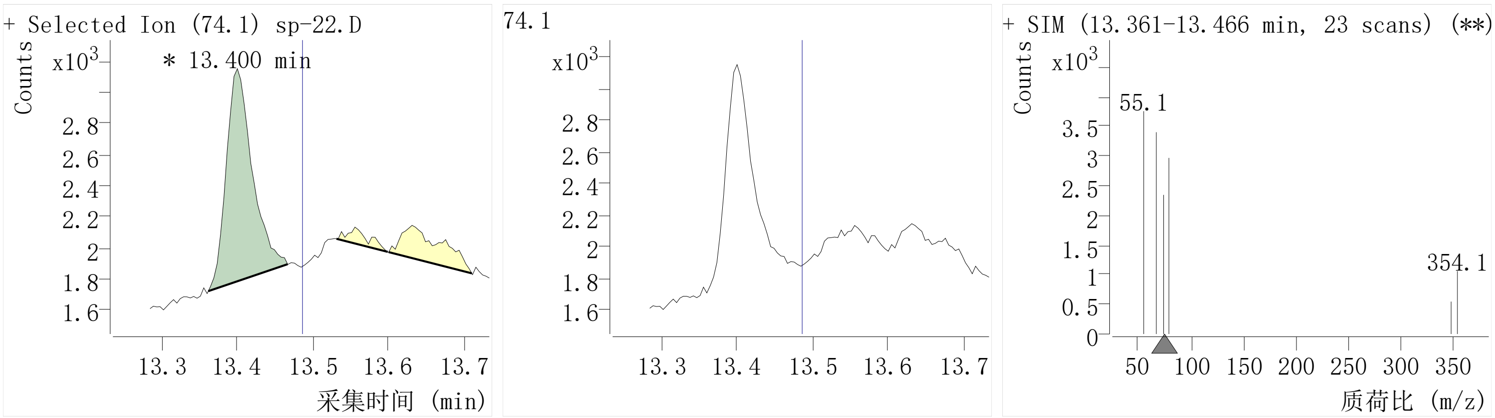

C22:6

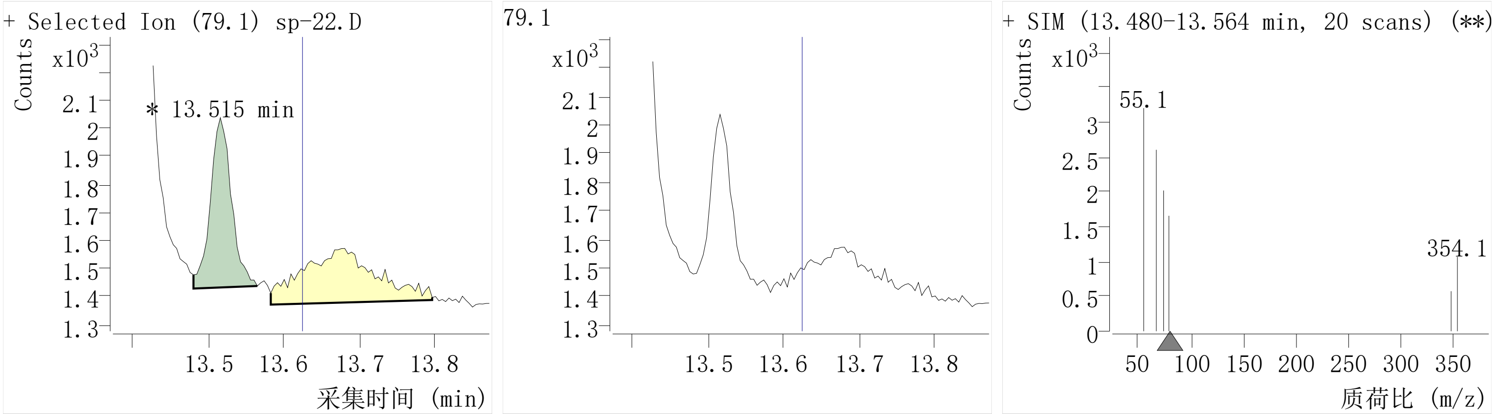
+ Selected Ion (79.1) sp-22.D
Counts
x10^3
* 13.515 min
13.5 13.6 13.7 13.8
采集时间 (min)
79.1
+ SIM (13.480-13.564 min, 20 scans) (**)
55.1
354.1
50 100 150 200 250 300 350
质荷比 (m/z)

C24:1

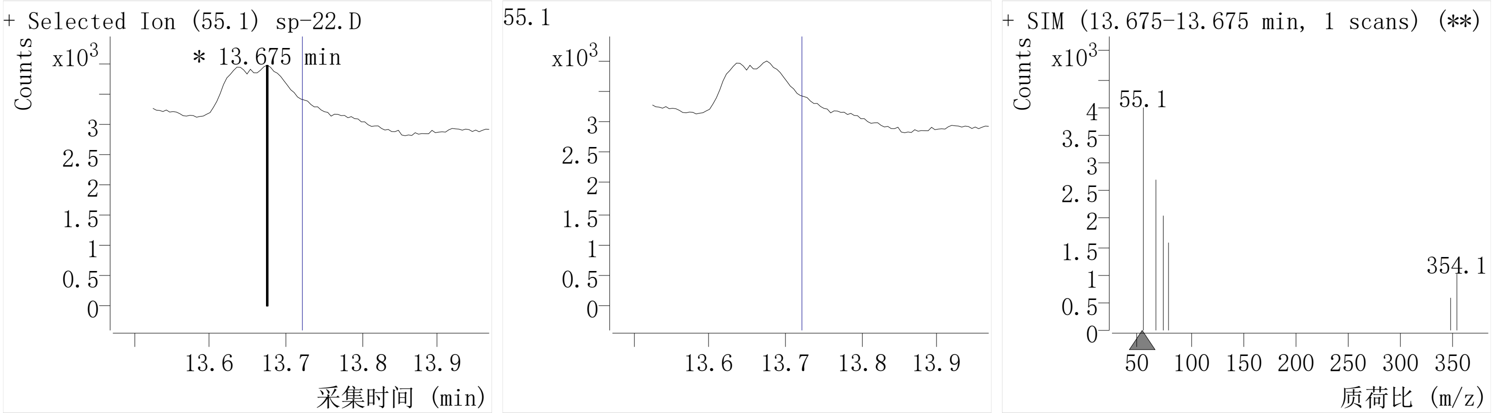
+ Selected Ion (55.1) sp-22.D
Counts
x10^3
* 13.675 min
13.6 13.7 13.8 13.9
采集时间 (min)
55.1
+ SIM (13.675-13.675 min, 1 scans) (**)
55.1
354.1
50 100 150 200 250 300 350
质荷比 (m/z)

**批处理路径**	G:\GC-MS\HX250430-4-GCMS总脂肪酸靶向检测\HX250430-4\QuantResults\HX250430-4.batch.bin		
**分析时间**	2025/5/14 16:58	**分析员姓名**	DESKTOP-M3A0GPO\omics
**报告时间**	2025/5/16 14:53:23	**报告员姓名**	DESKTOP-M3A0GPO\omics
**最近校正更新**	2025/5/14 16:58	**批处理状态**	已处理
**定量批处理版本**	10.2	**定量报告版本**	10.2

**采集时间**	2025/5/9 5:56	**数据文件**	sp-23.D
**样品类型**	样品	**样品名称**	sp-23
**稀释**	1	**采集方法**	脂肪酸

样品色谱图

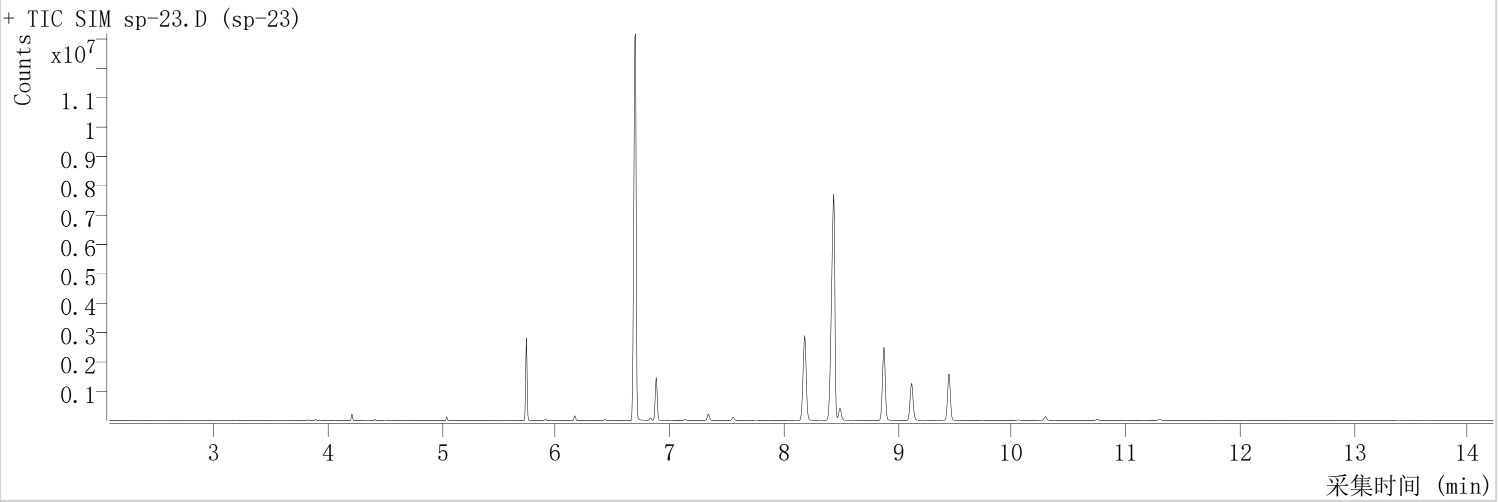

化合物	ISTD	RT	响应	ISTD 响应	响应比	最终浓度	单位
C4:0	C19:0	2.203	144	1819204	0.0001	ND	ug/ml
C6:0	C19:0	2.959	930	1819204	0.0005	0.0061	ug/ml
C8:0	C19:0	3.724	2628	1819204	0.0014	0.0103	ug/ml
C10:0	C19:0	4.413	24585	1819204	0.0135	0.2001	ug/ml
C11:0	C19:0	4.729	1183	1819204	0.0007	0.0052	ug/ml
C12:0	C19:0	5.044	73759	1819204	0.0405	0.5943	ug/ml
C13:0	C19:0	5.378	3977	1819204	0.0022	0.0221	ug/ml
C14:0	C19:0	5.743	1936363	1819204	1.0644	22.3255	ug/ml
C14:1	C19:0	5.912	29309	1819204	0.0161	0.7058	ug/ml
C15:0	C19:0	6.169	134295	1819204	0.0738	1.2462	ug/ml
C15:1	C19:0	6.432	0	1819204	0.0000	ND	ug/ml
C16:0	C19:0	6.694	13500084	1819204	7.4209	286.3638	ug/ml
C16:1	C19:0	6.881	869527	1819204	0.4780	29.1210	ug/ml
C17:0	C19:0	7.339	251696	1819204	0.1384	2.8865	ug/ml
C17:1	C19:0	7.557	83644	1819204	0.0460	2.4474	ug/ml
C18:0	C19:0	8.184	4291048	1819204	2.3588	53.1014	ug/ml
C18:1n9t	C19:0	8.322	9498	1819204	0.0052	0.3080	ug/ml
C18:1n9c	C19:0	8.437	7195558	1819204	3.9553	267.0546	ug/ml
C18:2n6t	C19:0	8.877	0	1819204	0.0000	ND	ug/ml
C18:2n6c	C19:0	8.877	1969952	1819204	1.0829	75.1104	ug/ml
C18:3n6	C19:0	9.122	19468	1819204	0.0107	ND	ug/ml
C18:3n3	C19:0	9.451	1275508	1819204	0.7011	34.8530	ug/ml
C20:0	C19:0	10.056	39958	1819204	0.0220	0.5880	ug/ml
C20:1	C19:0	10.291	129511	1819204	0.0712	4.4270	ug/ml
C20:2	C19:0	10.749	37344	1819204	0.0205	1.3092	ug/ml
C21:0	C19:0	10.958	1934	1819204	0.0011	0.0294	ug/ml
C20:3n6	C19:0	11.003	3077	1819204	0.0017	0.1510	ug/ml
C20:4n6	C19:0	11.154	4881	1819204	0.0027	0.2026	ug/ml
C20:3n3	C19:0	11.296	40279	1819204	0.0221	1.2086	ug/ml
C20:5n3	C19:0	11.692	1308	1819204	0.0007	0.0749	ug/ml

定量分析完成报告

化合物	ISTD	RT	响应	ISTD 响应	响应比	最终浓度	单位
C22:0	C19:0	11.817	3793	1819204	0.0021	0.0878	ug/ml
C22:1n9	C19:0	12.048	3545	1819204	0.0019	0.1295	ug/ml
C22:2n6	C19:0	12.470	610	1819204	0.0003	0.0634	ug/ml
C23:0	C19:0	12.621	0	1819204	0.0000	ND	ug/ml
C24:0	C19:0	13.400	1840	1819204	0.0010	0.0519	ug/ml
C22:6	C19:0	13.515	1288	1819204	0.0007	0.0575	ug/ml
C24:1	C19:0	13.671	0	1819204	0.0000	ND	ug/ml

C4:0

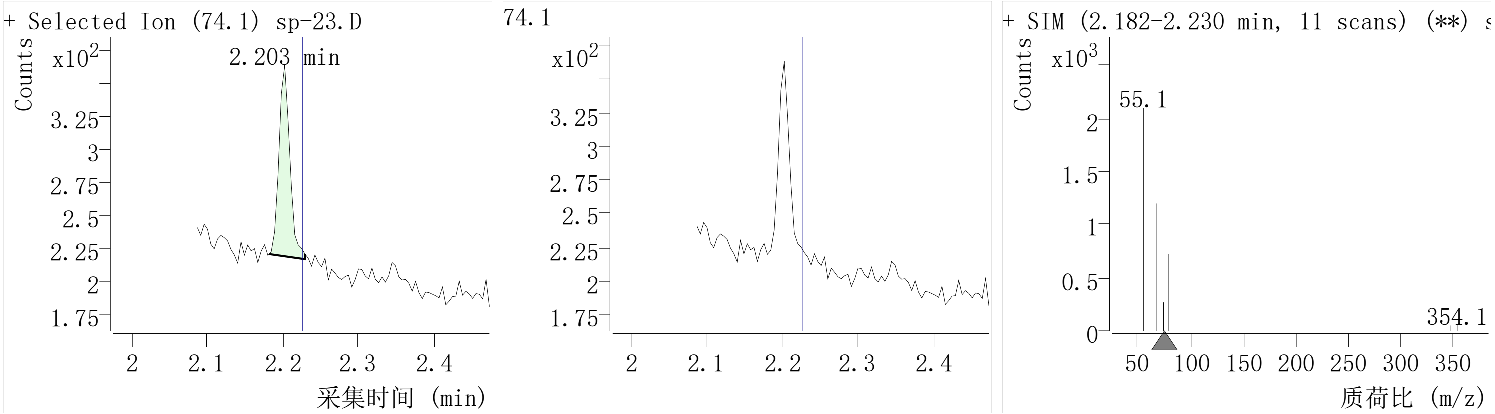

C6:0

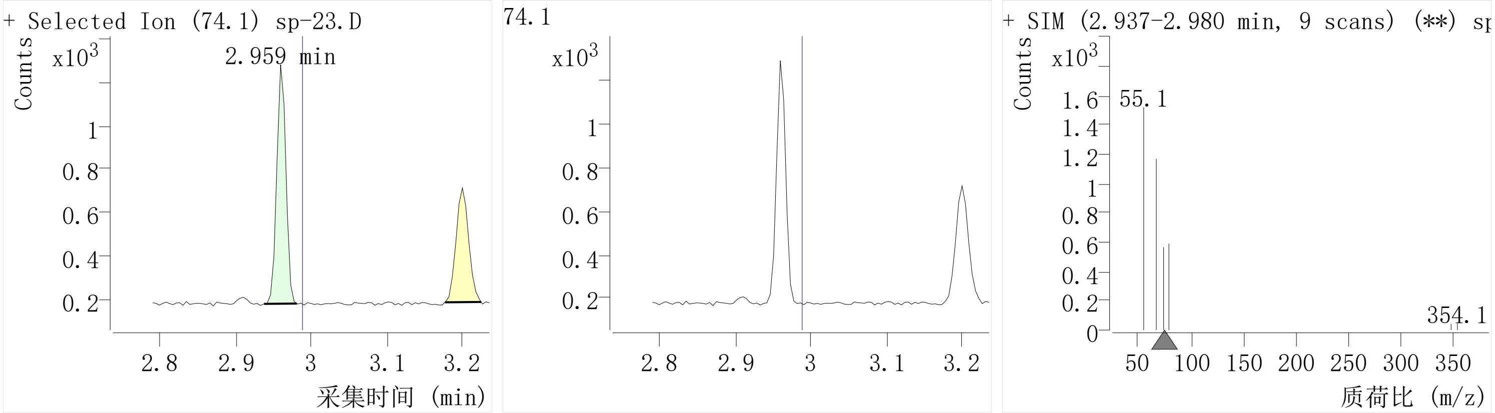

C8:0

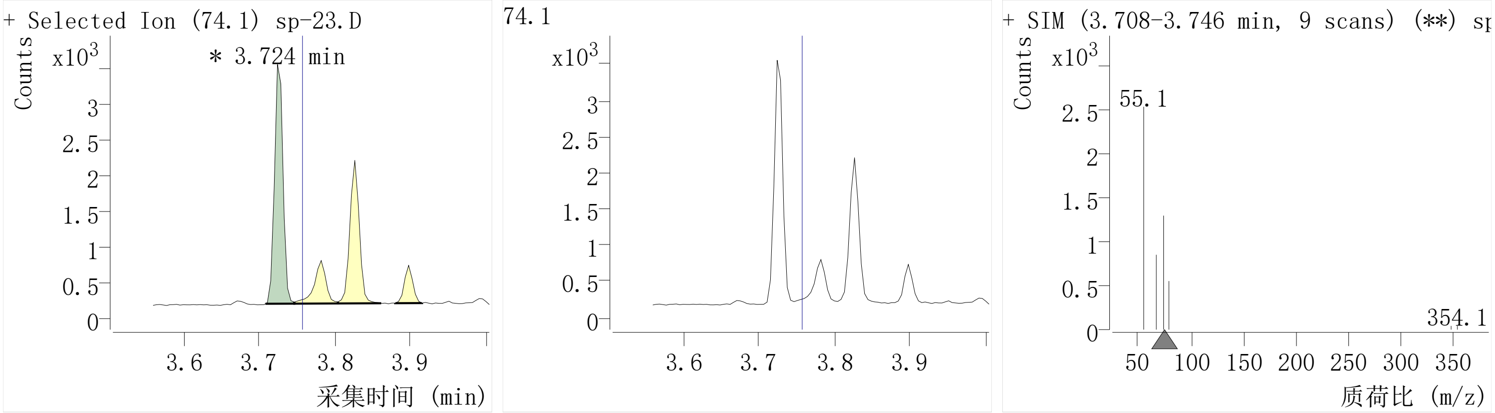

C10:0

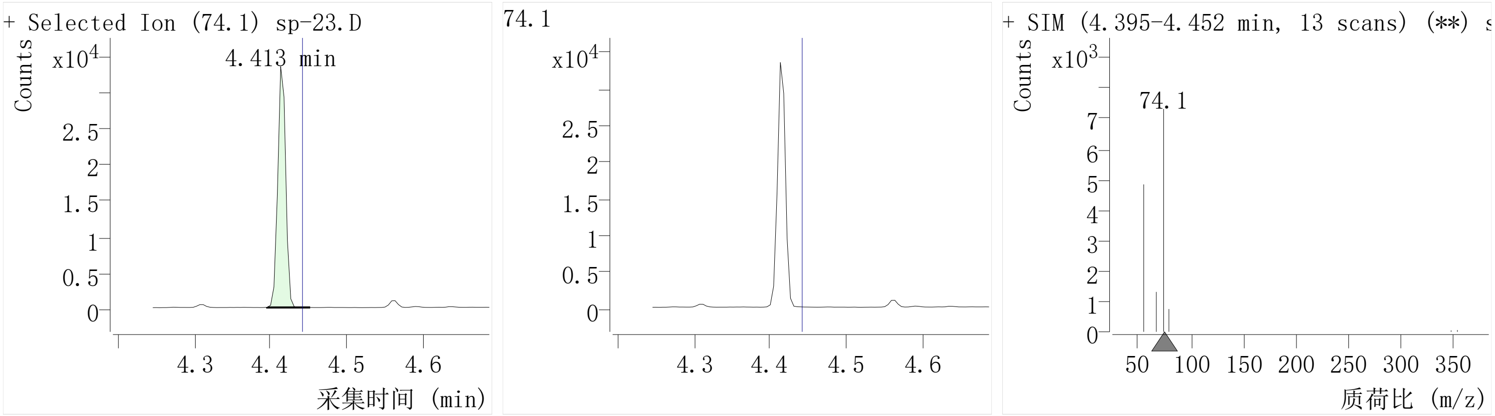

C11:0

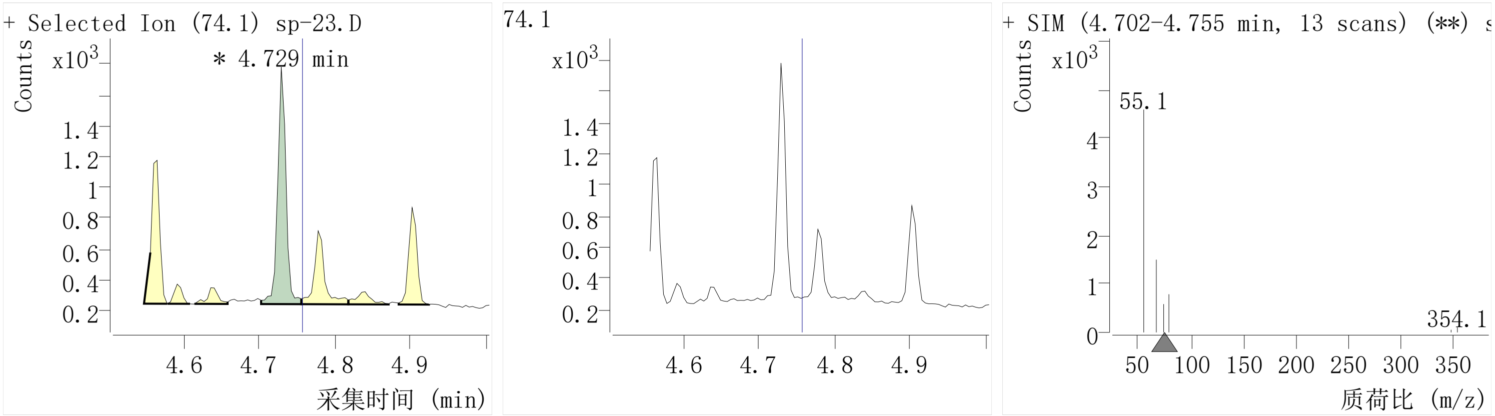

C12:0

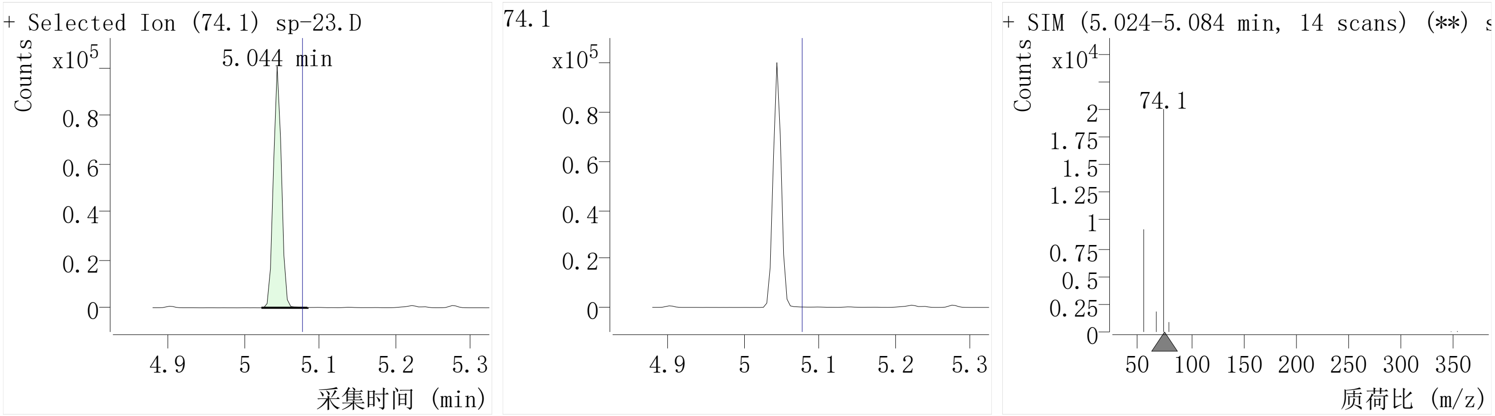

C13:0

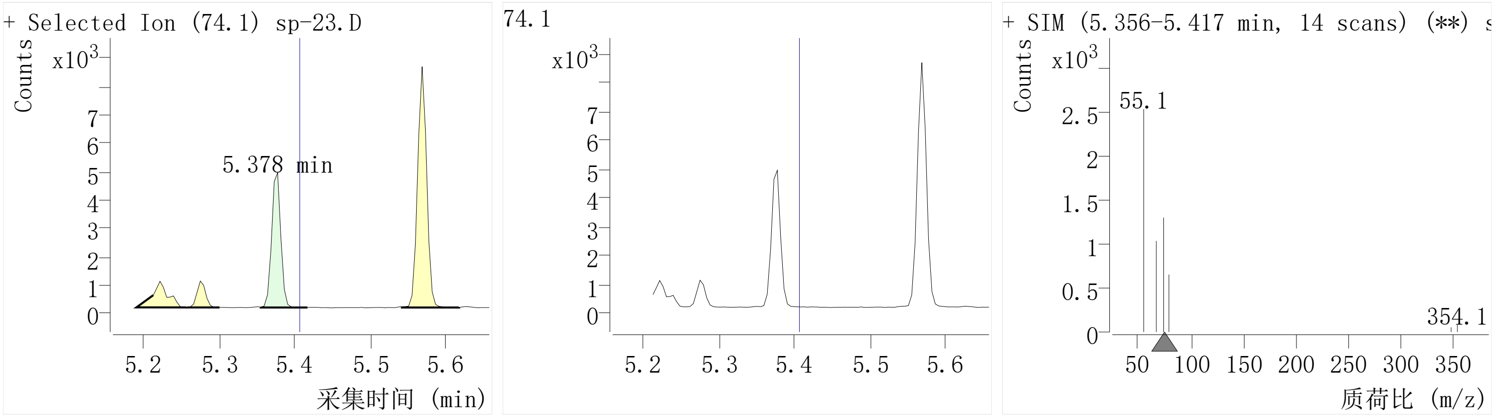

C14:0

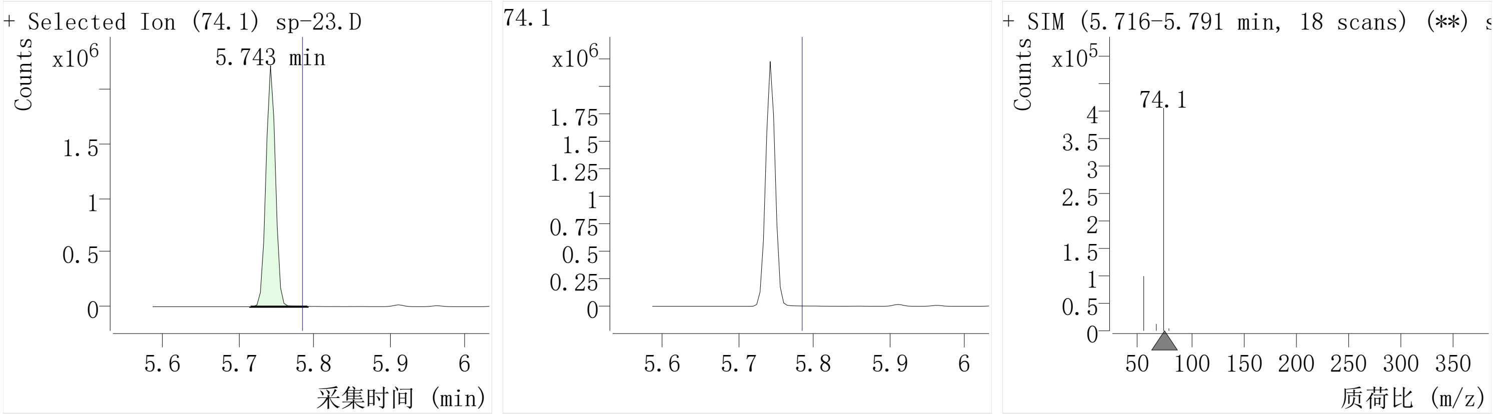

C14:1

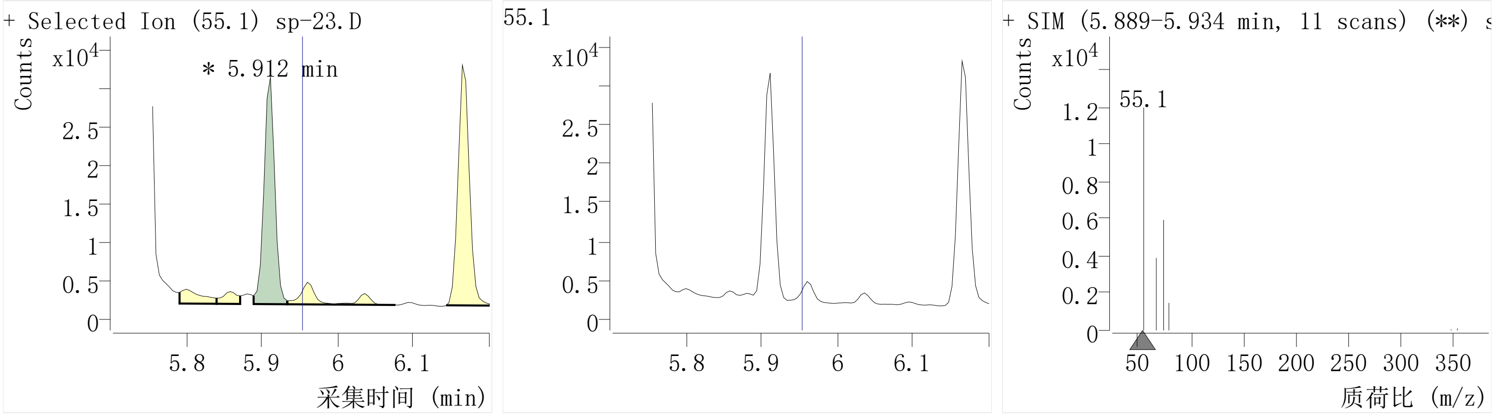

C15:0

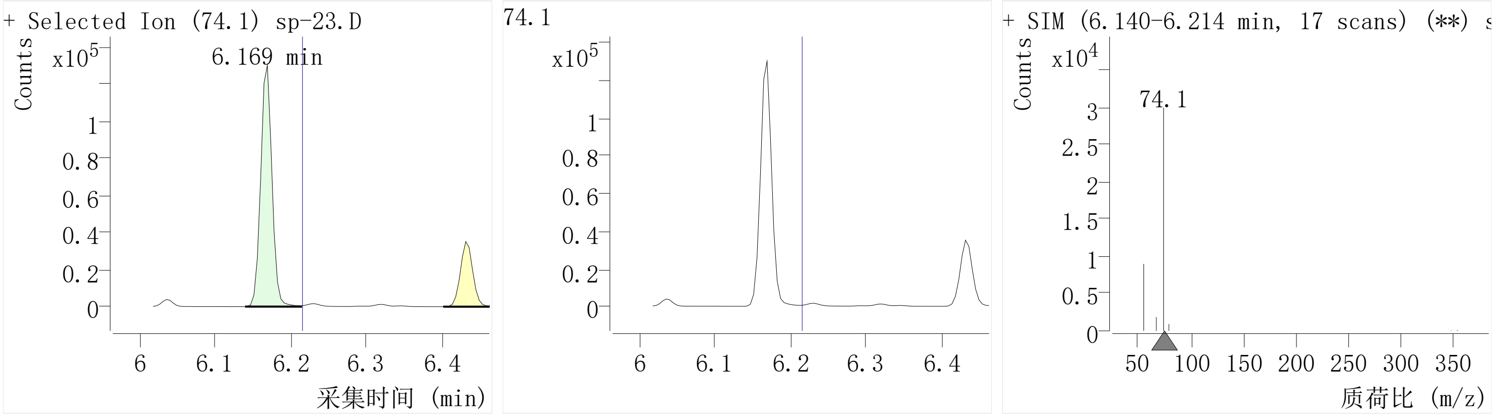

C15:1

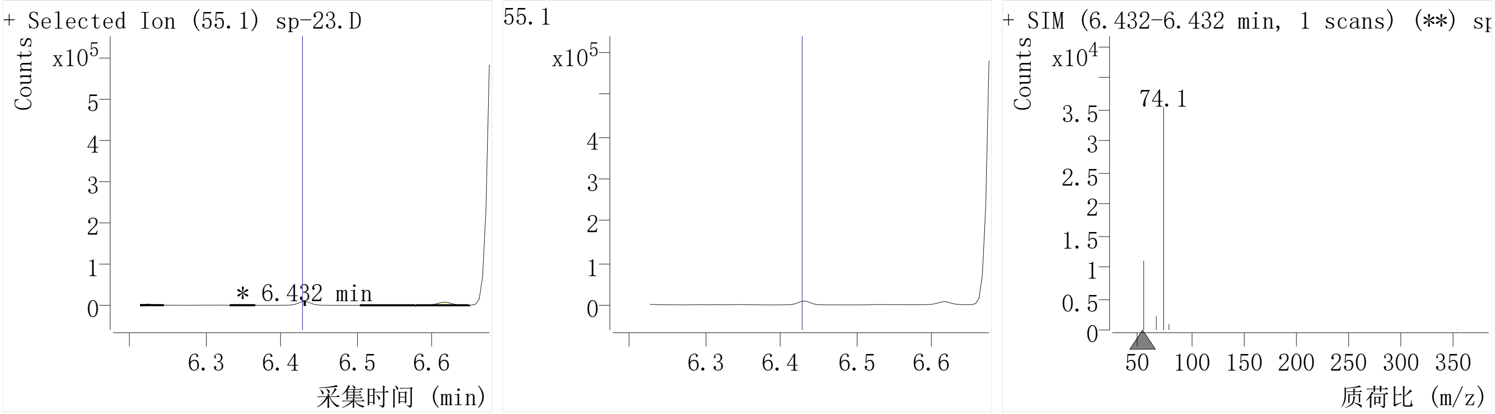

C16:0

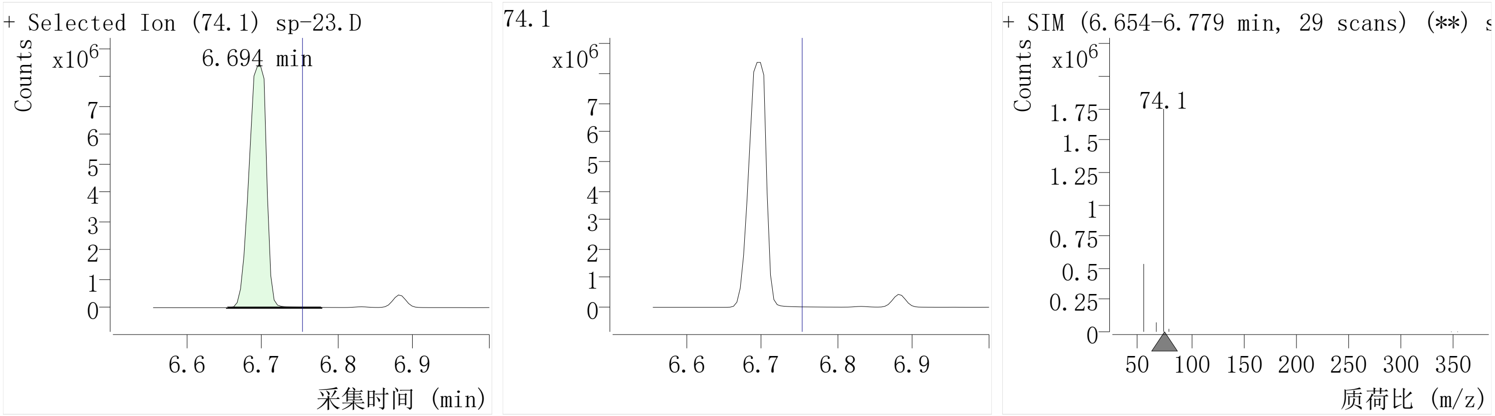

C16:1

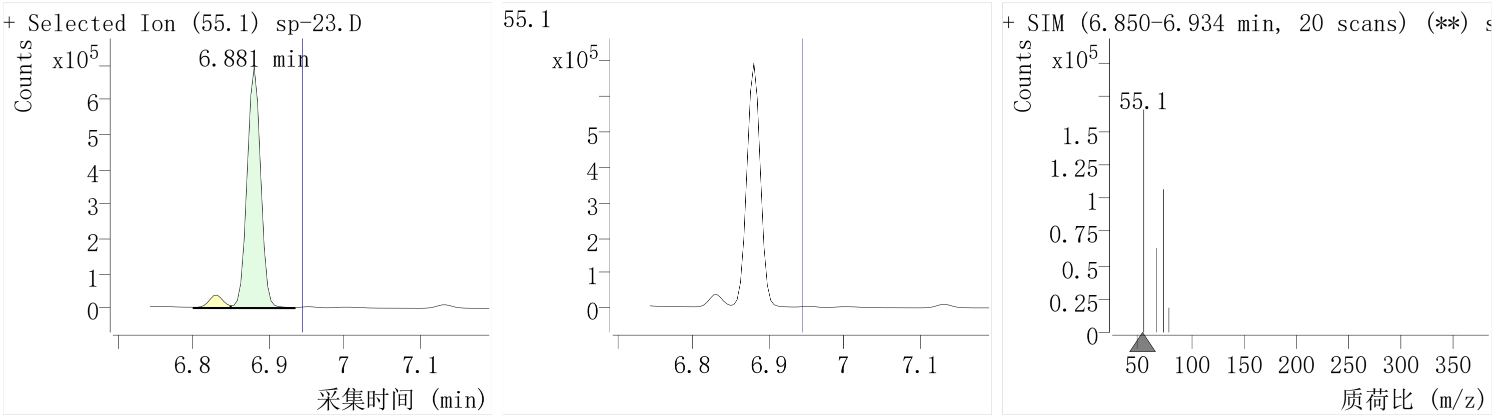

C17:0

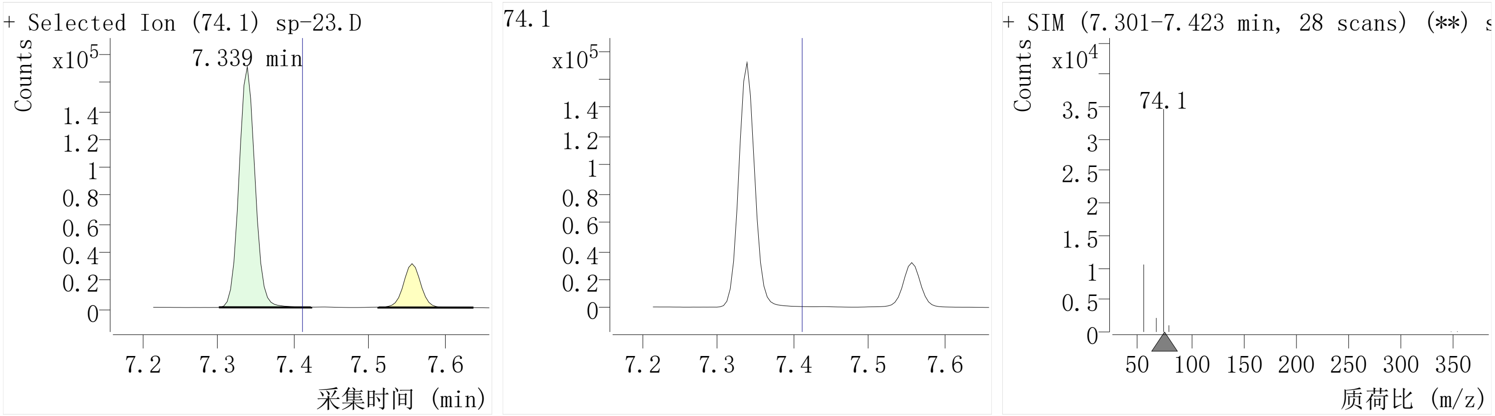

C17:1

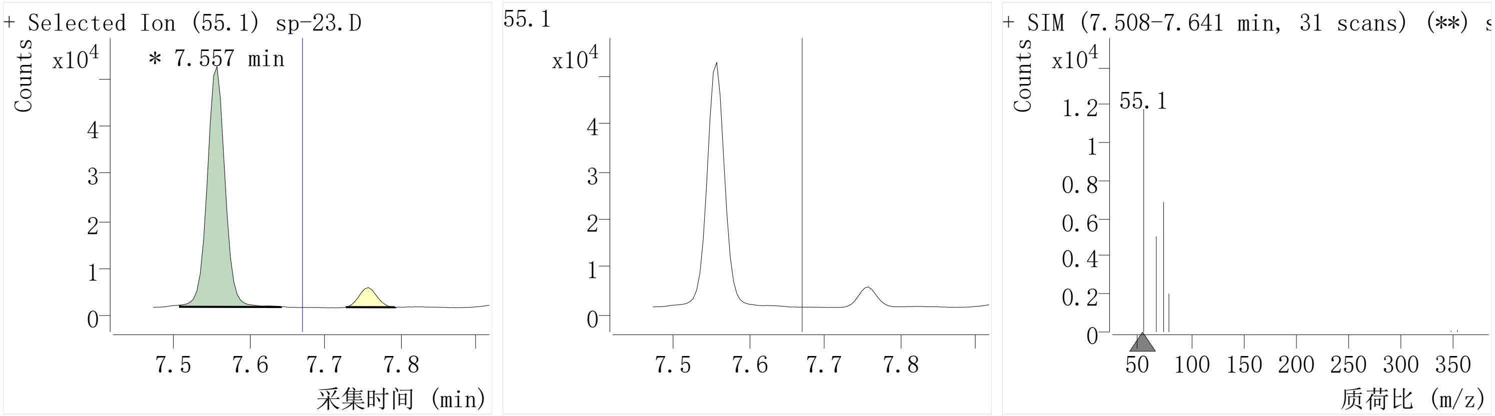

C18:0

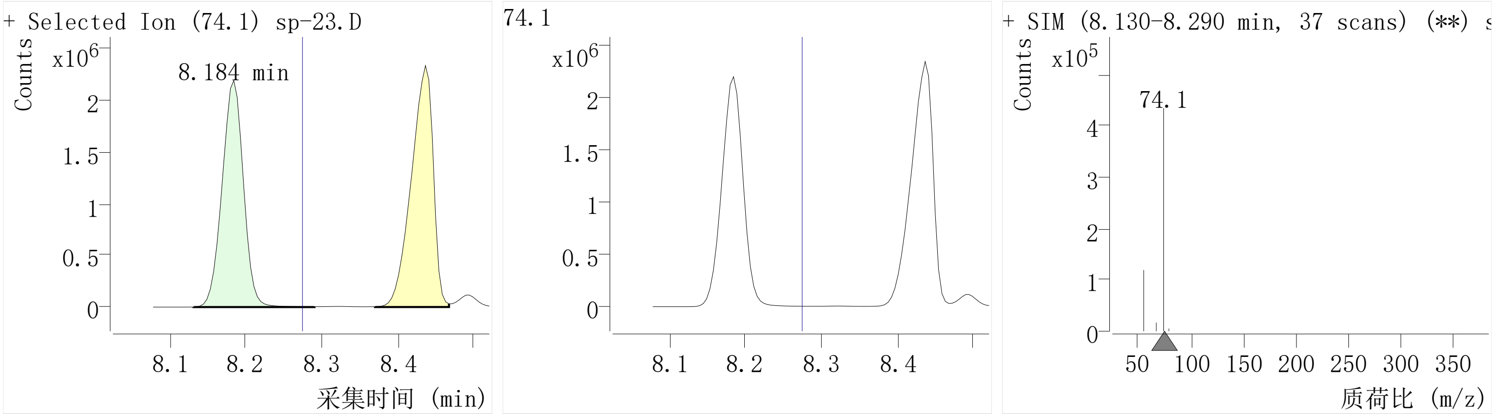

C18:1n9t

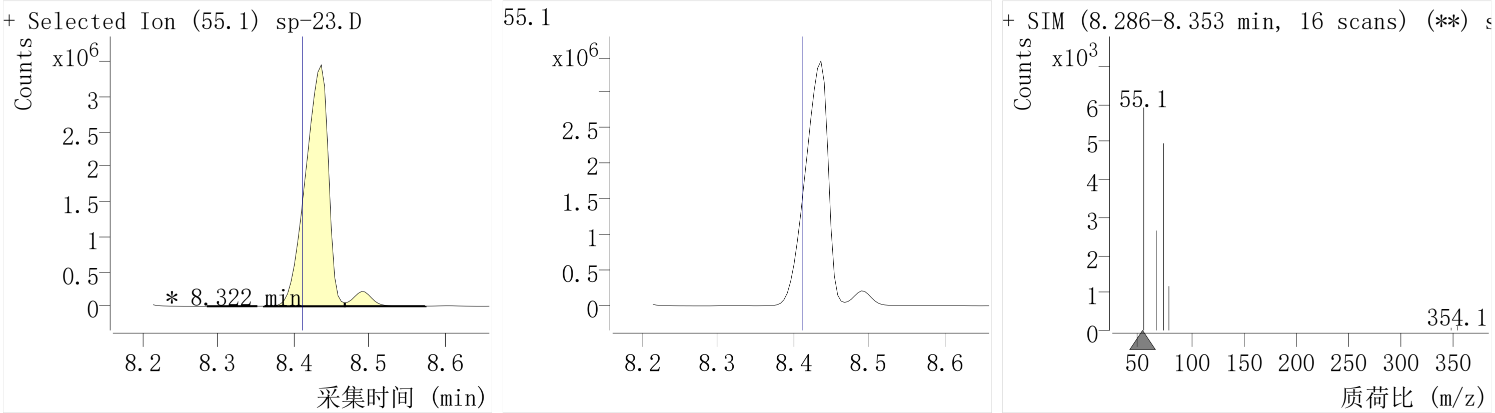

C18:1n9c

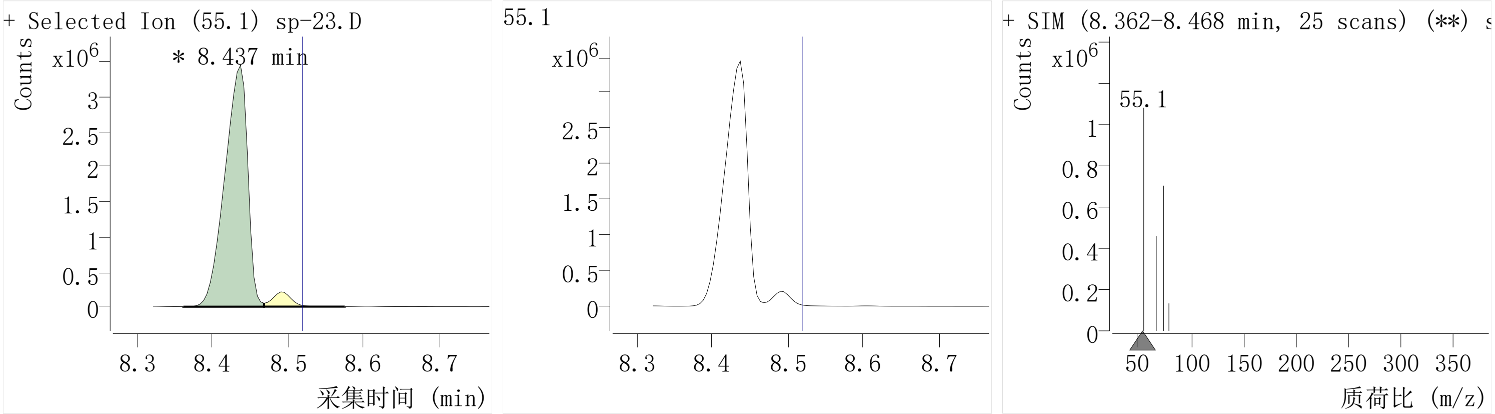

C18:2n6t

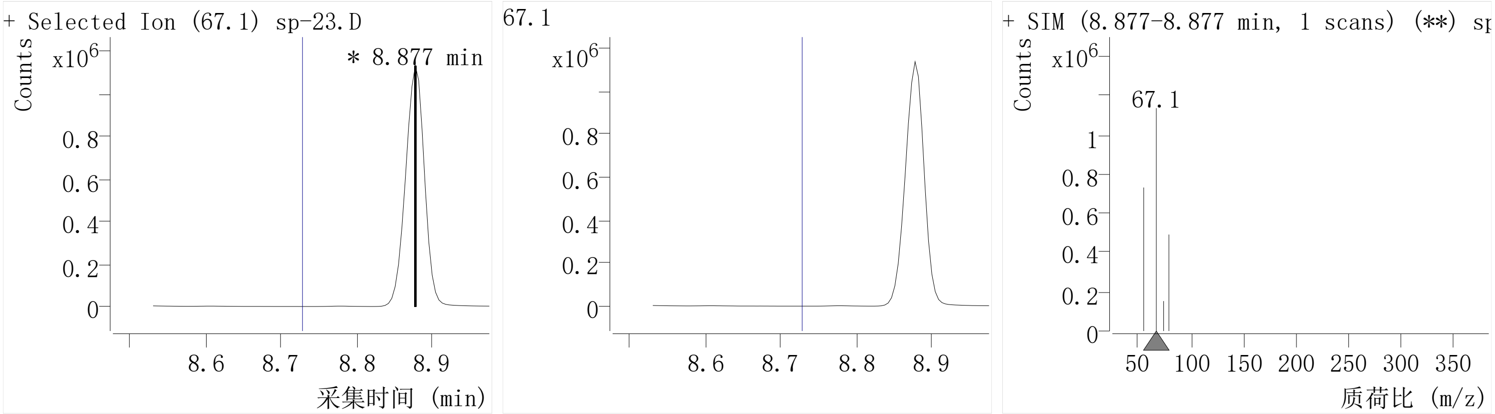

C18:2n6c

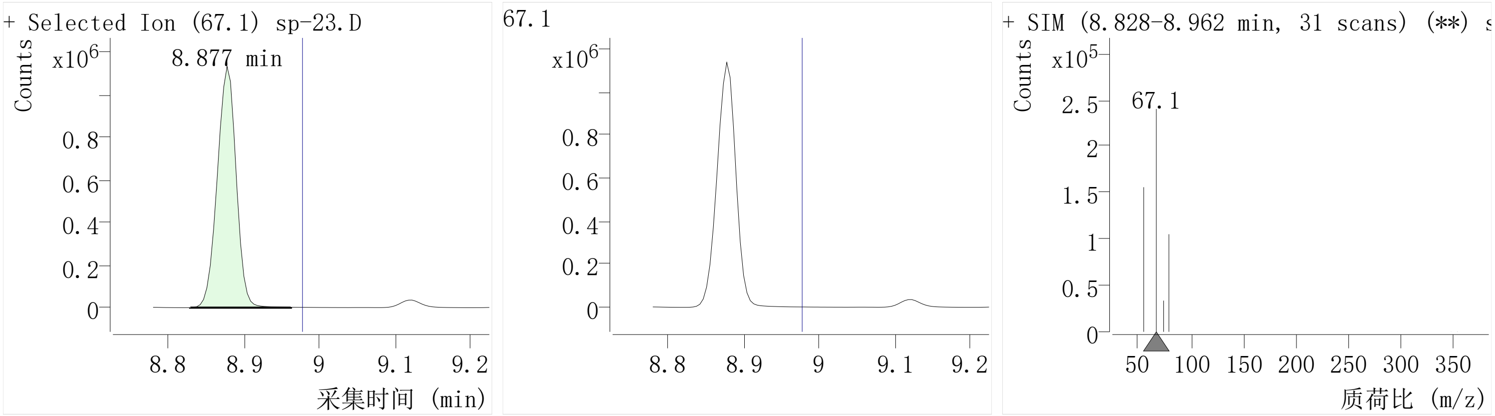

C18:3n6

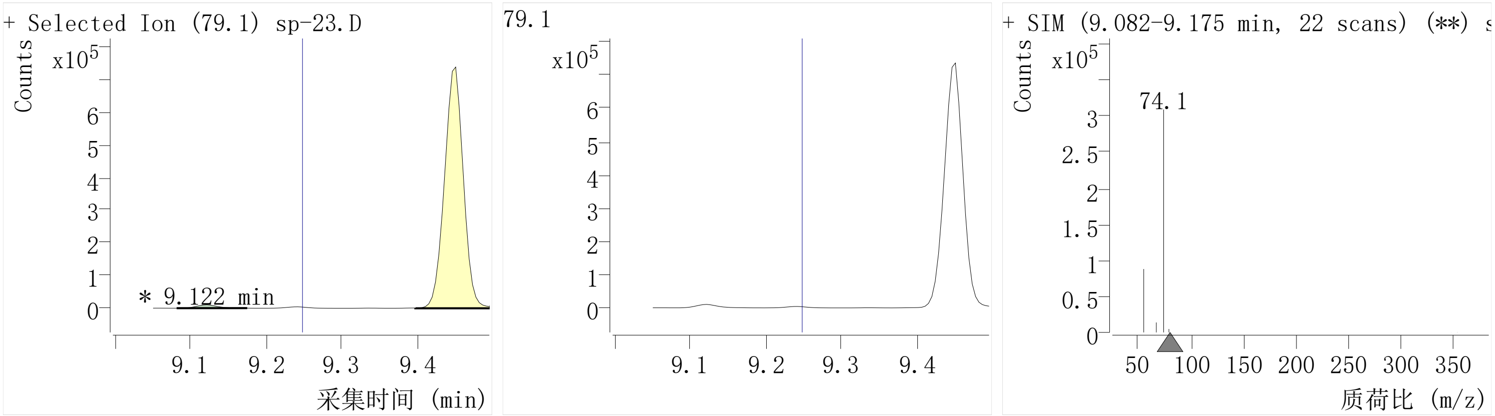

C18:3n3

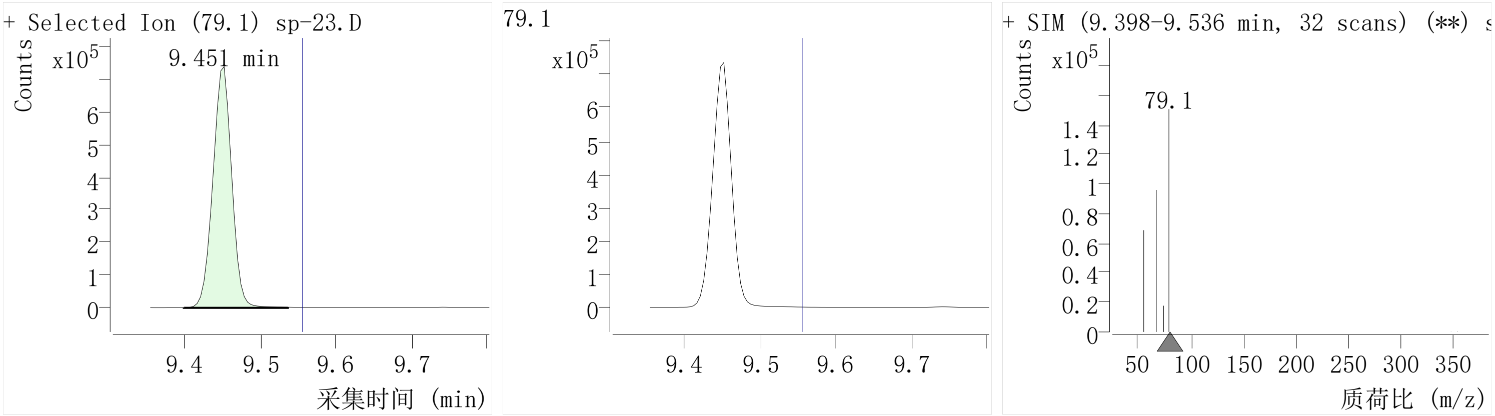

C20:0

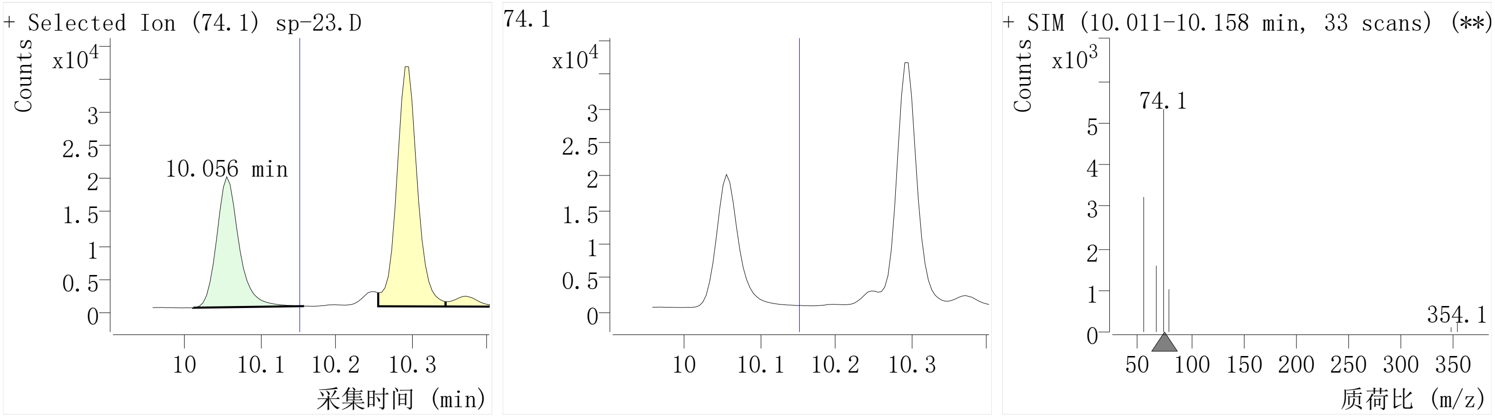

C20:1

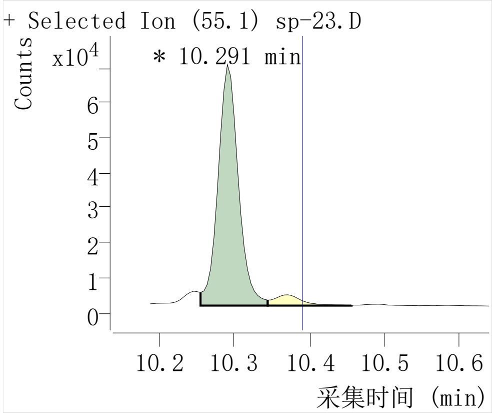

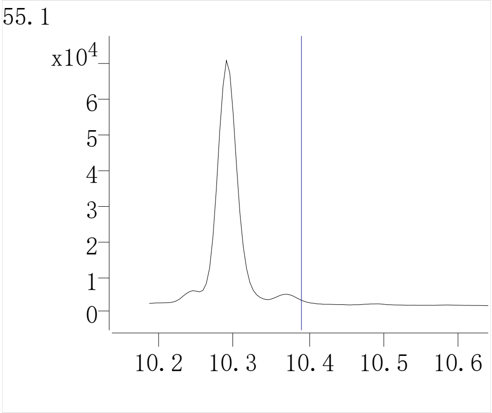

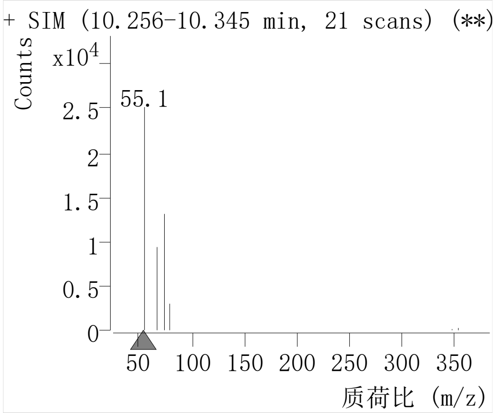

C20:2

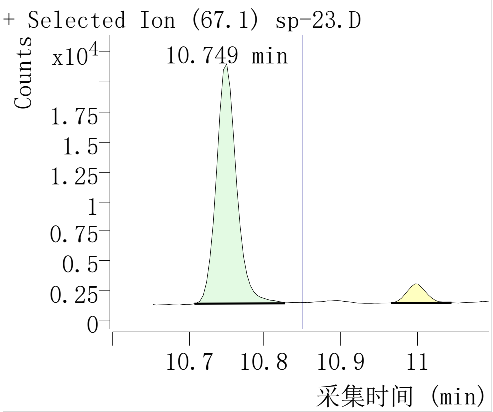

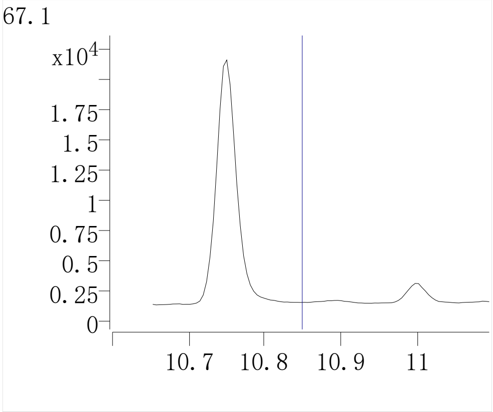

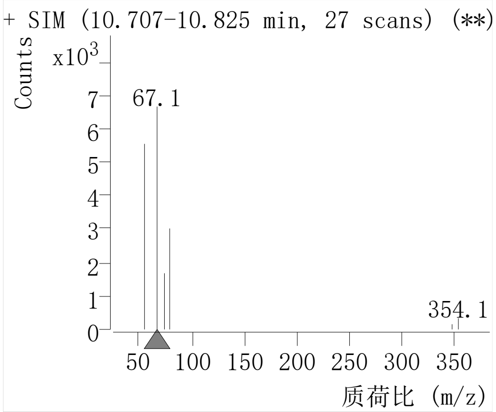

C21:0

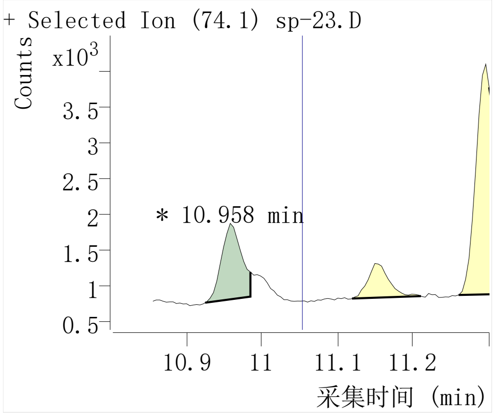

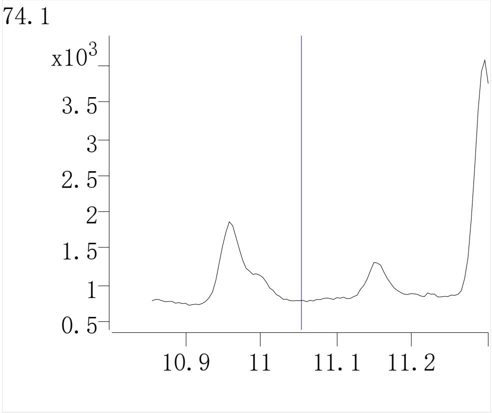

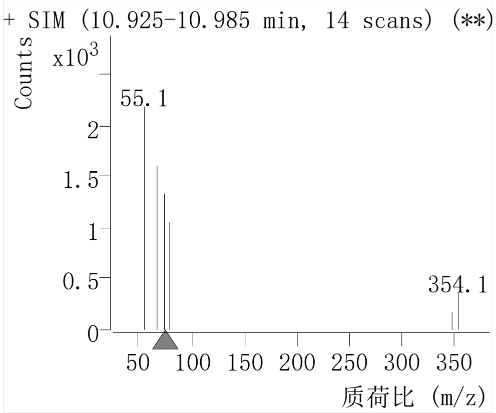

C20:3n6

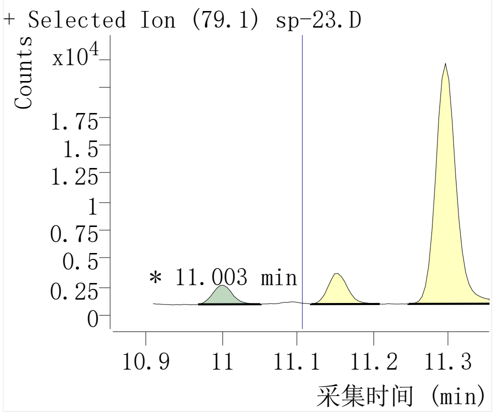

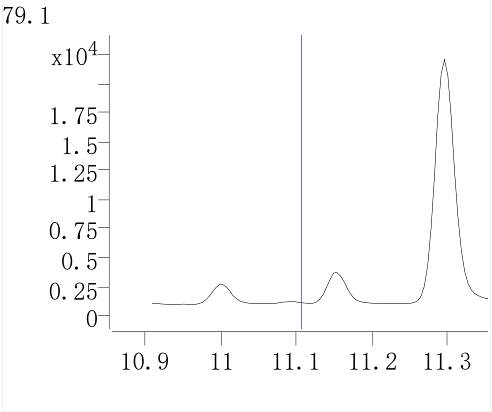

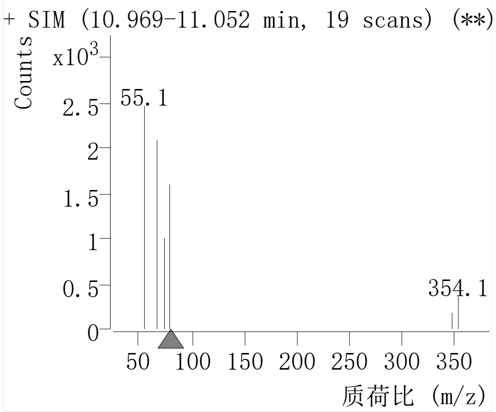

C20:4n6

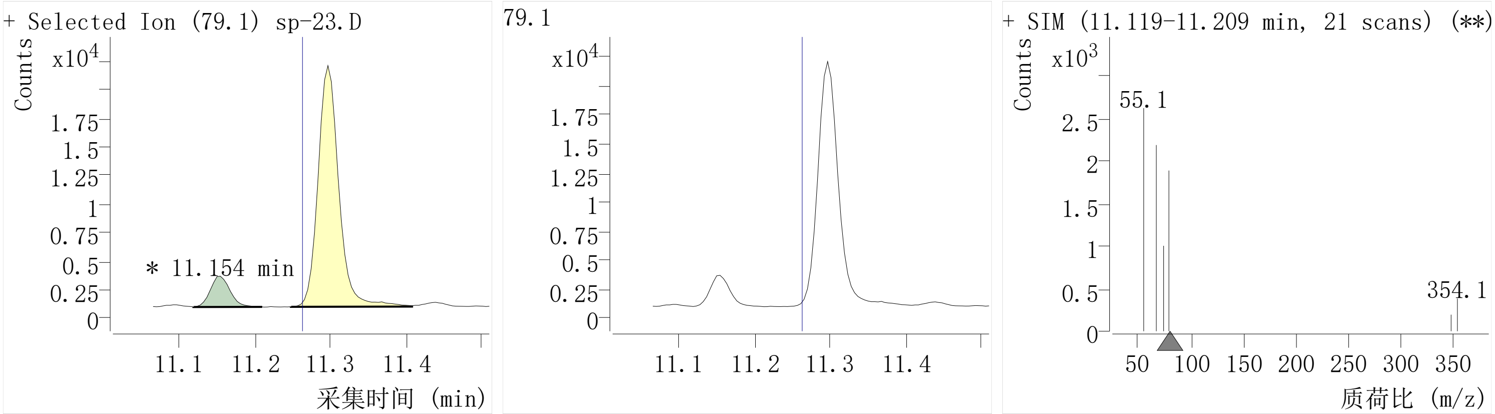

C20:3n3

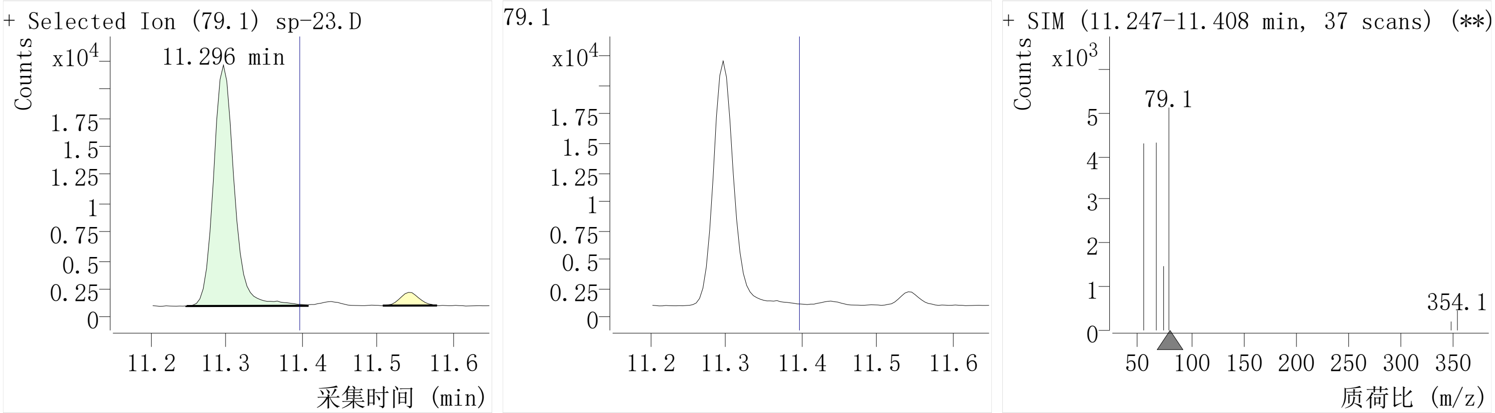

C20:5n3

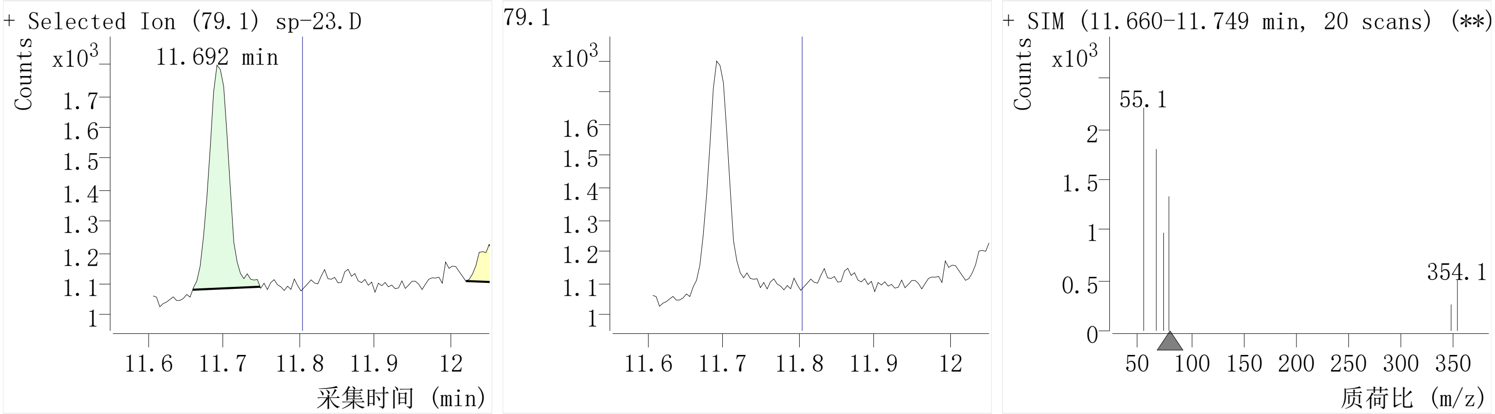

C22:0

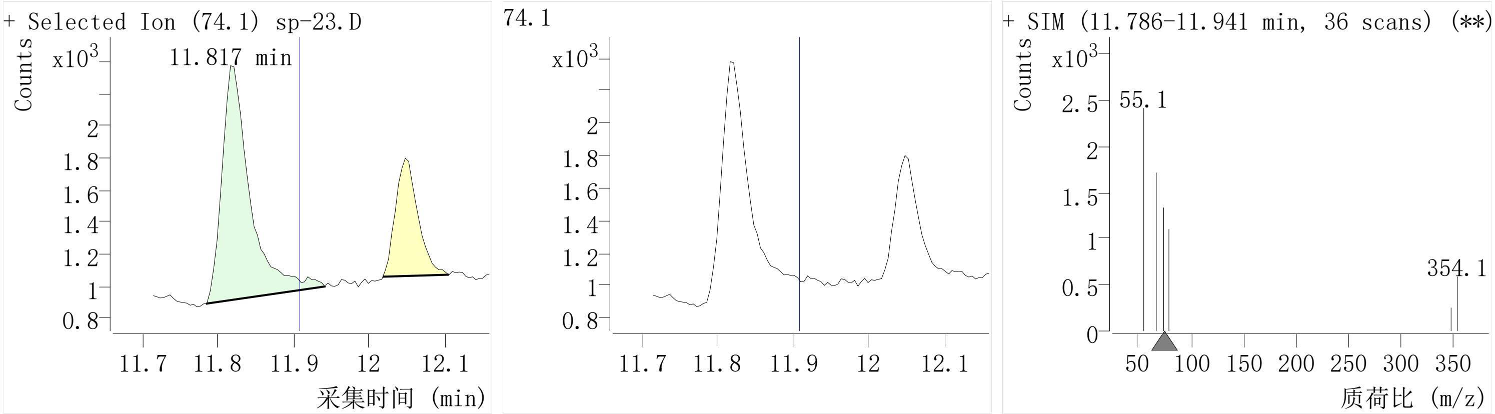

C22:1n9

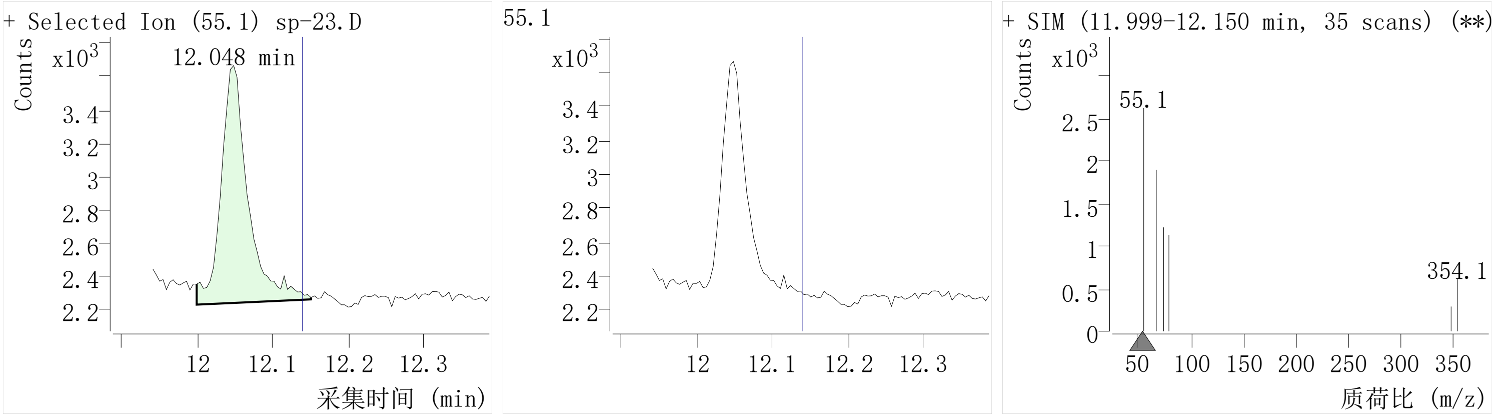

C22:2n6

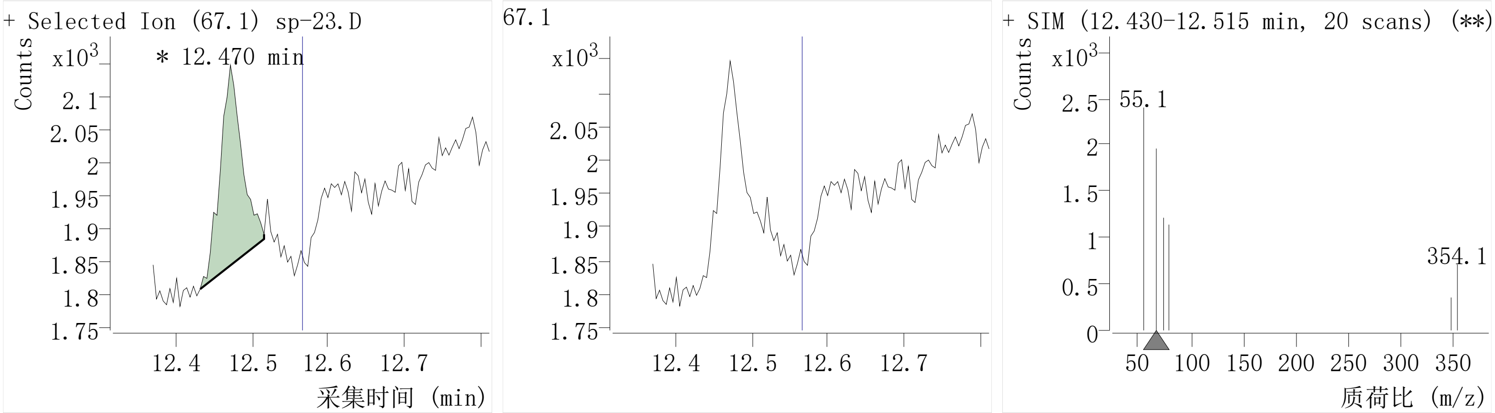

C23:0

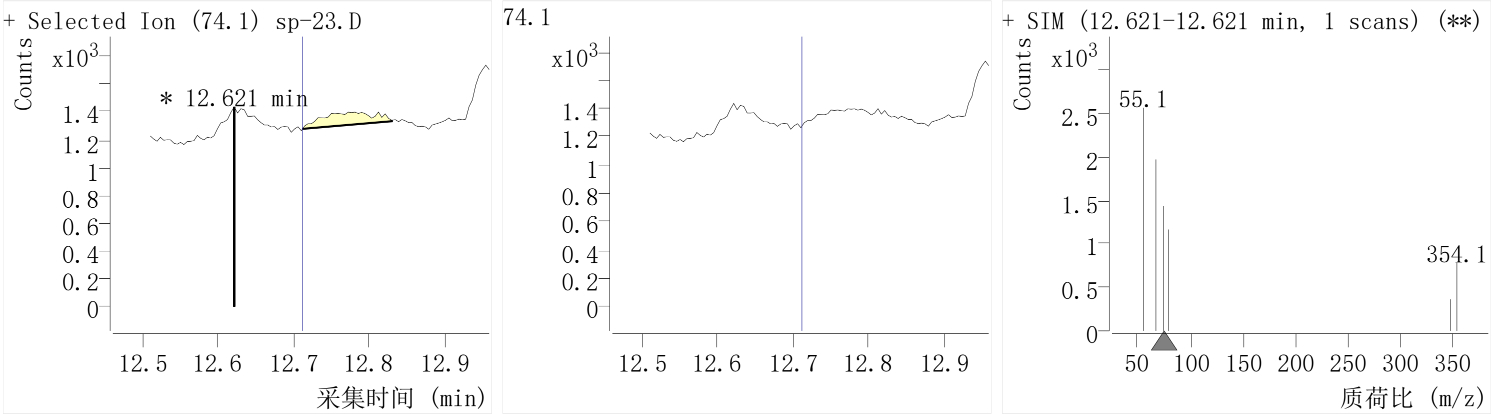

C24:0

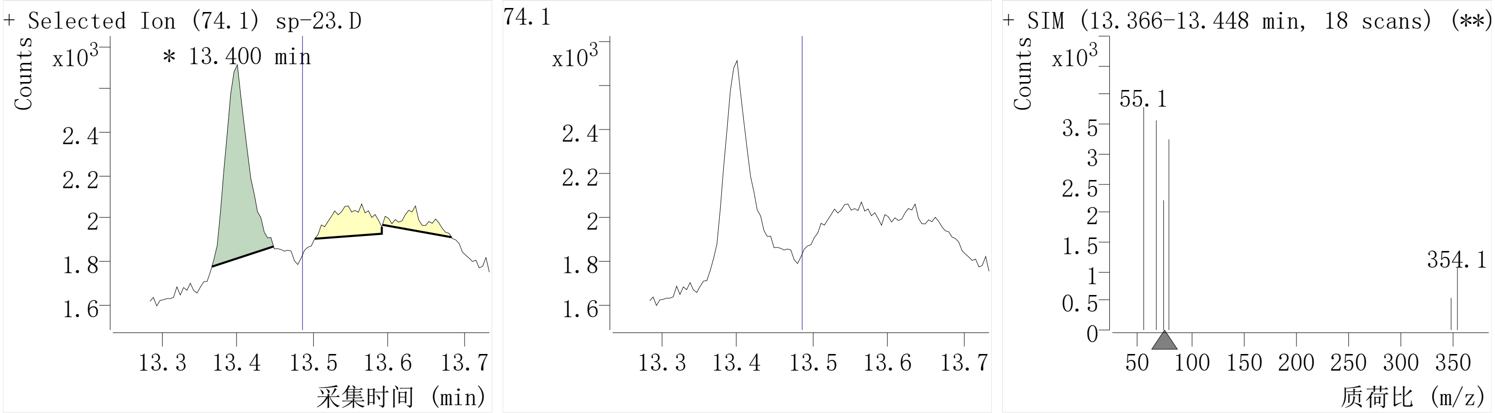

C22:6

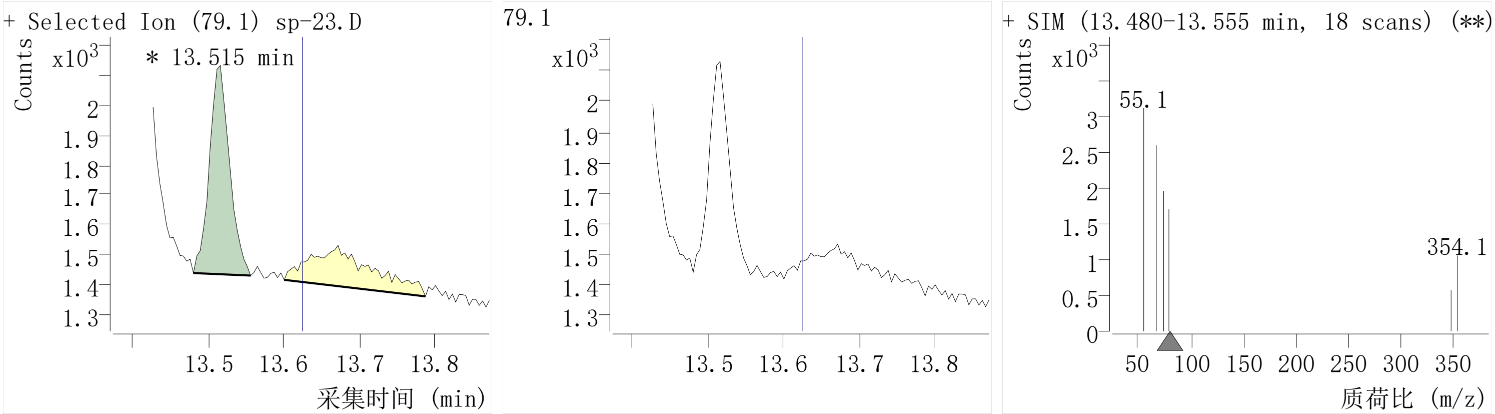
+ Selected Ion (79.1) sp-23.D
Counts
x10^3
* 13.515 min
2
1.9
1.8
1.7
1.6
1.5
1.4
1.3
13.5 13.6 13.7 13.8
采集时间 (min)
79.1
x10^3
2
1.9
1.8
1.7
1.6
1.5
1.4
1.3
13.5 13.6 13.7 13.8
+ SIM (13.480-13.555 min, 18 scans) (**)
Counts
x10^3
55.1
3
2.5
2
1.5
1
0.5
0
354.1
50 100 150 200 250 300 350
质荷比 (m/z)

C24:1

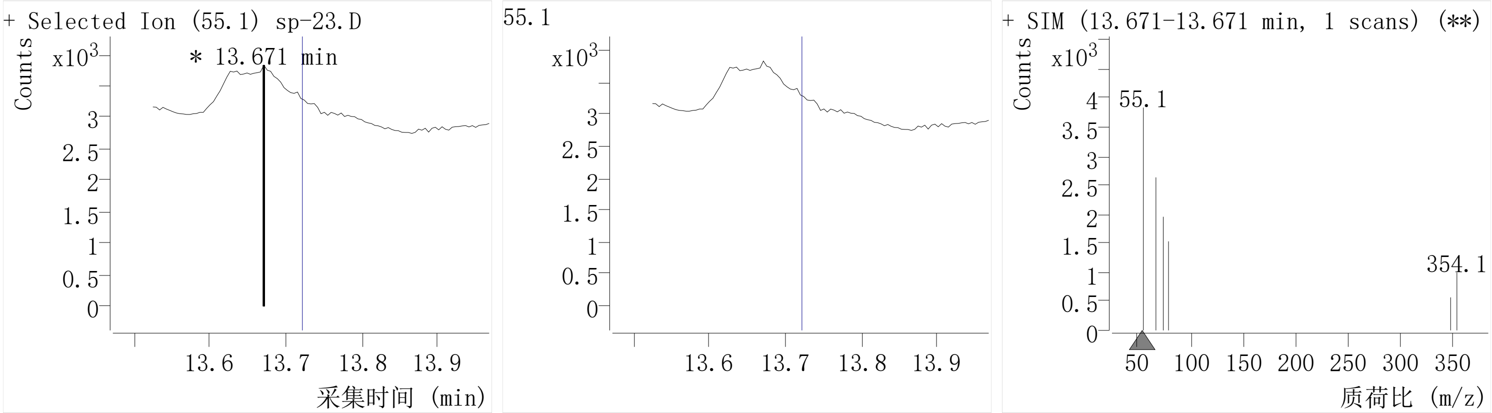
+ Selected Ion (55.1) sp-23.D
Counts
x10^3
* 13.671 min
3
2.5
2
1.5
1
0.5
0
13.6 13.7 13.8 13.9
采集时间 (min)
55.1
x10^3
3
2.5
2
1.5
1
0.5
0
13.6 13.7 13.8 13.9
+ SIM (13.671-13.671 min, 1 scans) (**)
Counts
x10^3
55.1
4
3.5
3
2.5
2
1.5
1
0.5
0
354.1
50 100 150 200 250 300 350
质荷比 (m/z)

批处理路径	G:\GC-MS\HX250430-4-GCMS总脂肪酸靶向检测\HX250430-4\QuantResults\HX250430-4.batch.bin		
分析时间	2025/5/14 16:58	分析员姓名	DESKTOP-M3A0GPO\omics
报告时间	2025/5/16 14:53:25	报告员姓名	DESKTOP-M3A0GPO\omics
最近校正更新	2025/5/14 16:58	批处理状态	已处理
定量批处理版本	10.2	定量报告版本	10.2

采集时间	2025/5/9 6:16	数据文件	sp-24.D
样品类型	样品	样品名称	sp-24
稀释	1	采集方法	脂肪酸

样品色谱图

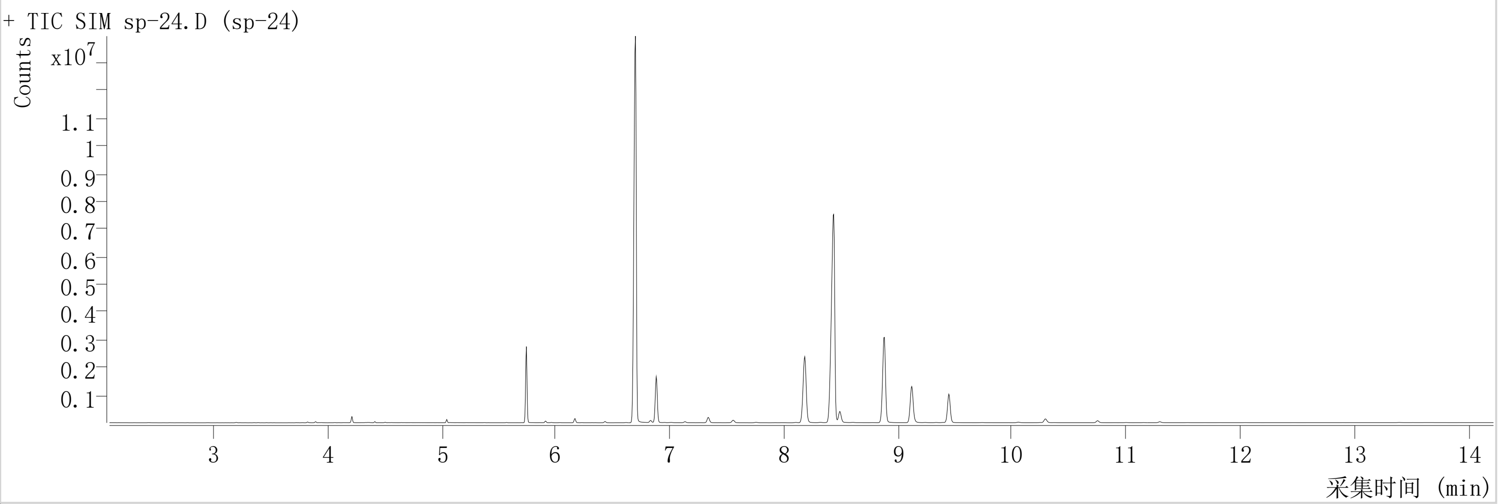

化合物	ISTD	RT	响应	ISTD 响应	响应比	最终浓度	单位
C4:0	C19:0	2.203	171	1859181	0.0001	ND	ug/ml
C6:0	C19:0	2.959	725	1859181	0.0004	0.0042	ug/ml
C8:0	C19:0	3.724	2496	1859181	0.0013	0.0093	ug/ml
C10:0	C19:0	4.413	23587	1859181	0.0127	0.1851	ug/ml
C11:0	C19:0	4.729	1092	1859181	0.0006	0.0046	ug/ml
C12:0	C19:0	5.044	68193	1859181	0.0367	0.5269	ug/ml
C13:0	C19:0	5.373	3531	1859181	0.0019	0.0188	ug/ml
C14:0	C19:0	5.742	1874195	1859181	1.0081	20.9509	ug/ml
C14:1	C19:0	5.911	31711	1859181	0.0171	0.7535	ug/ml
C15:0	C19:0	6.169	123174	1859181	0.0663	1.1046	ug/ml
C15:1	C19:0	6.432	0	1859181	0.0000	ND	ug/ml
C16:0	C19:0	6.696	14058751	1859181	7.5618	293.9782	ug/ml
C16:1	C19:0	6.881	1001124	1859181	0.5385	32.8185	ug/ml
C17:0	C19:0	7.339	226990	1859181	0.1221	2.5472	ug/ml
C17:1	C19:0	7.557	73535	1859181	0.0396	2.1013	ug/ml
C18:0	C19:0	8.184	3550711	1859181	1.9098	41.6432	ug/ml
C18:1n9t	C19:0	8.321	8991	1859181	0.0048	0.2853	ug/ml
C18:1n9c	C19:0	8.433	7089862	1859181	3.8134	256.8634	ug/ml
C18:2n6t	C19:0	8.882	0	1859181	0.0000	ND	ug/ml
C18:2n6c	C19:0	8.882	2489368	1859181	1.3390	100.4500	ug/ml
C18:3n6	C19:0	9.122	20447	1859181	0.0110	ND	ug/ml
C18:3n3	C19:0	9.446	812261	1859181	0.4369	21.7153	ug/ml
C20:0	C19:0	10.056	34102	1859181	0.0183	0.4967	ug/ml
C20:1	C19:0	10.291	136683	1859181	0.0735	4.5704	ug/ml
C20:2	C19:0	10.749	57616	1859181	0.0310	1.9485	ug/ml
C21:0	C19:0	10.958	1953	1859181	0.0011	0.0291	ug/ml
C20:3n6	C19:0	11.003	4937	1859181	0.0027	0.2119	ug/ml
C20:4n6	C19:0	11.154	4258	1859181	0.0023	0.1781	ug/ml
C20:3n3	C19:0	11.296	27756	1859181	0.0149	0.8316	ug/ml
C20:5n3	C19:0	11.696	587	1859181	0.0003	0.0527	ug/ml

定量分析完成报告

化合物	ISTD	RT	响应	ISTD 响应	响应比	最终浓度	单位
C22:0	C19:0	11.816	3538	1859181	0.0019	0.0819	ug/ml
C22:1n9	C19:0	12.048	4670	1859181	0.0025	0.1712	ug/ml
C22:2n6	C19:0	12.470	925	1859181	0.0005	0.0763	ug/ml
C23:0	C19:0	12.626	0	1859181	0.0000	ND	ug/ml
C24:0	C19:0	13.395	2028	1859181	0.0011	0.0555	ug/ml
C22:6	C19:0	13.515	662	1859181	0.0004	0.0315	ug/ml
C24:1	C19:0	13.671	0	1859181	0.0000	ND	ug/ml

C4:0

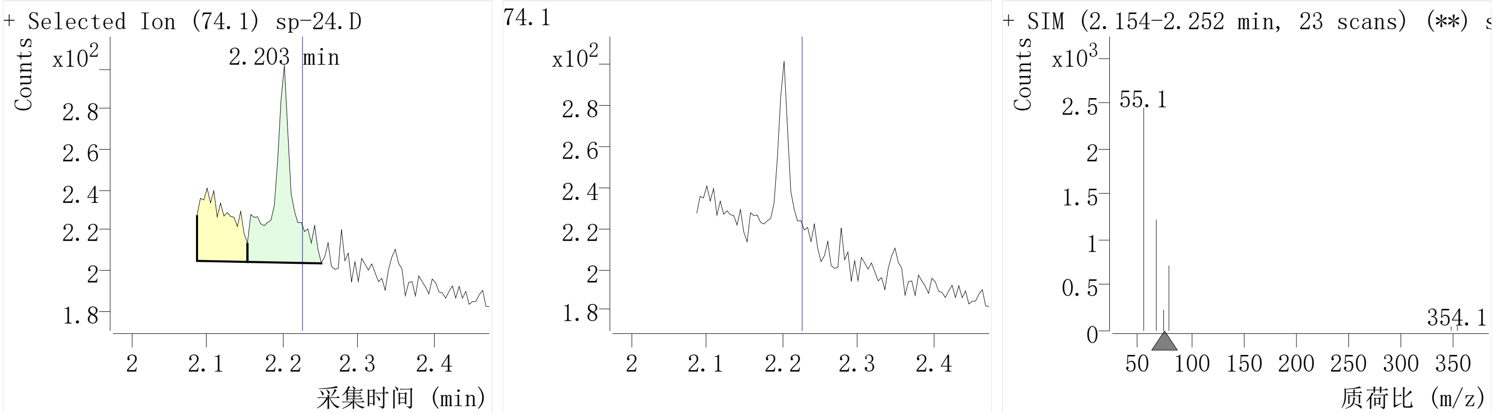

C6:0

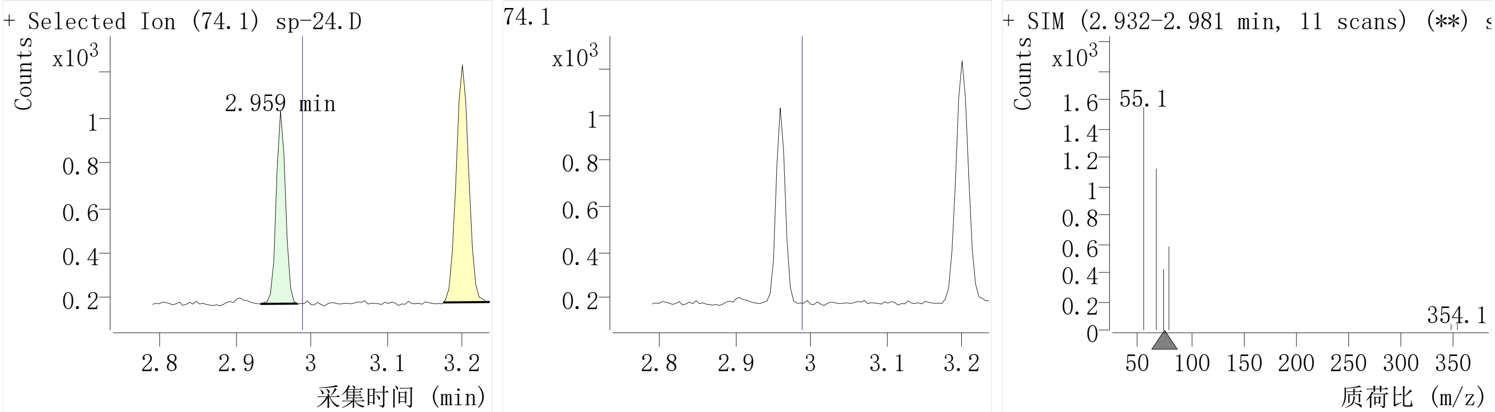

C8:0

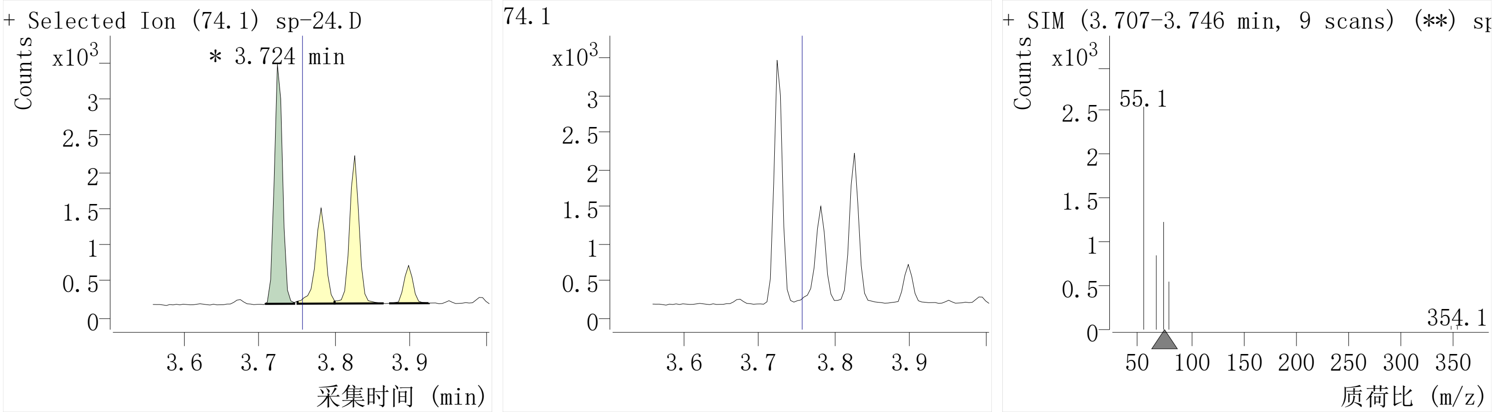

C10:0

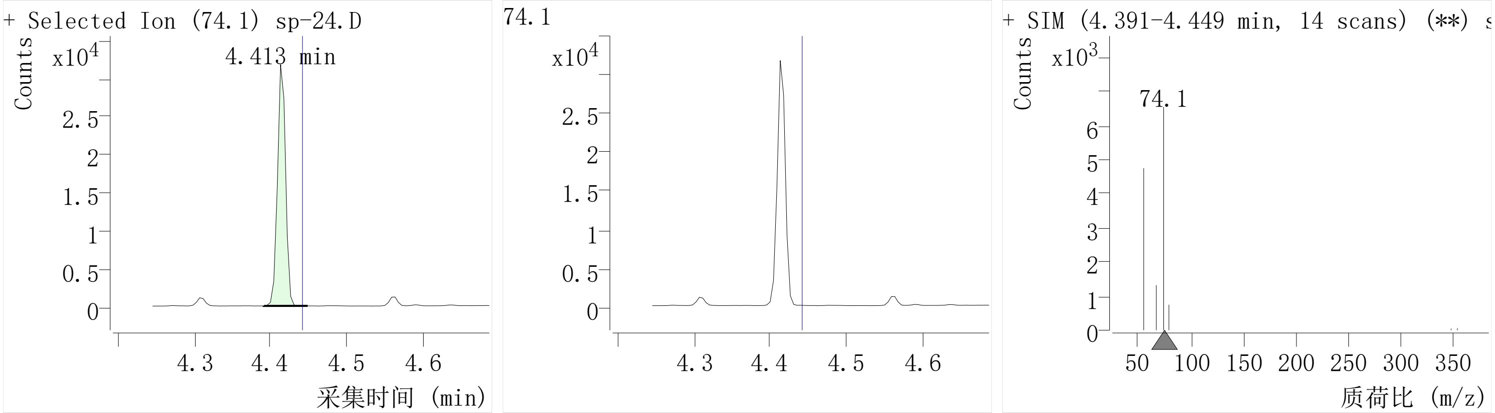

C11:0

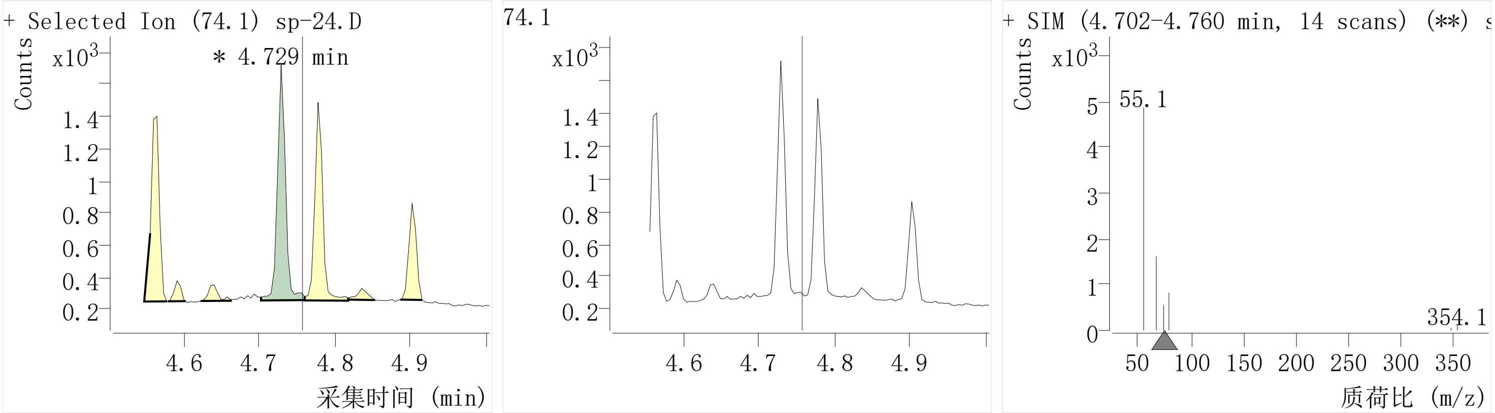

C12:0

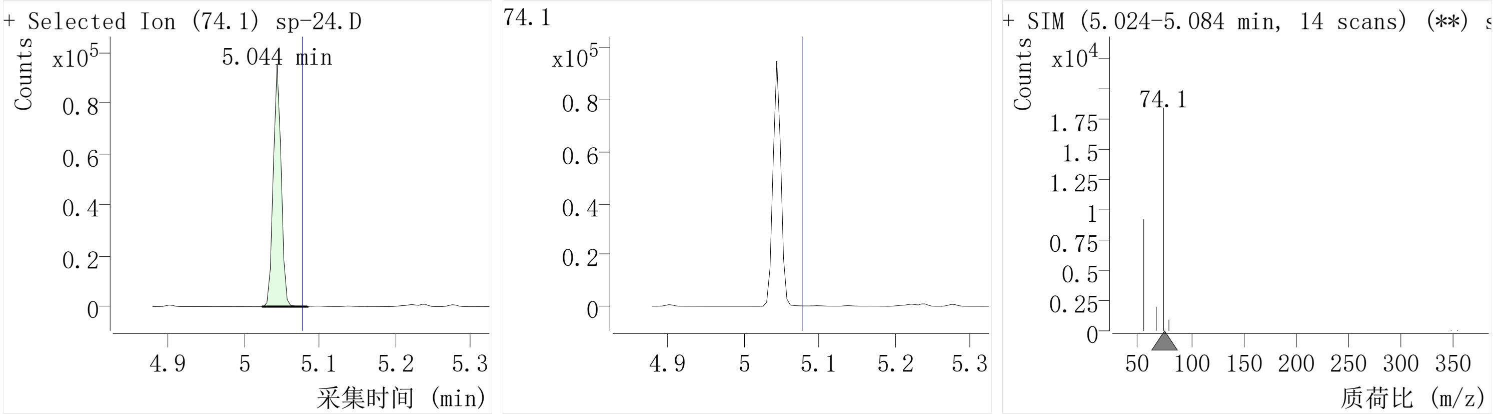

C13:0

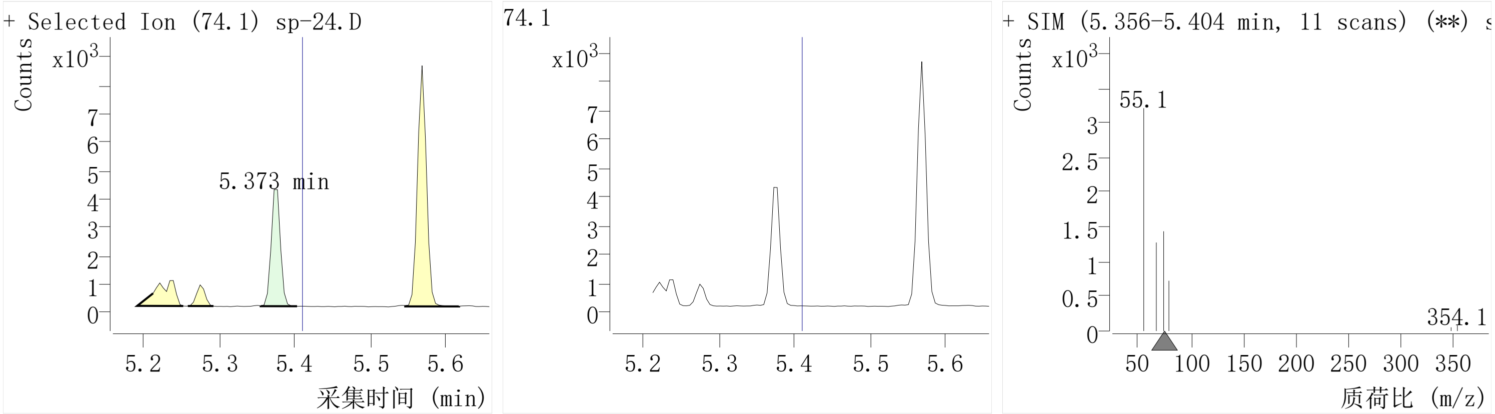

C14:0

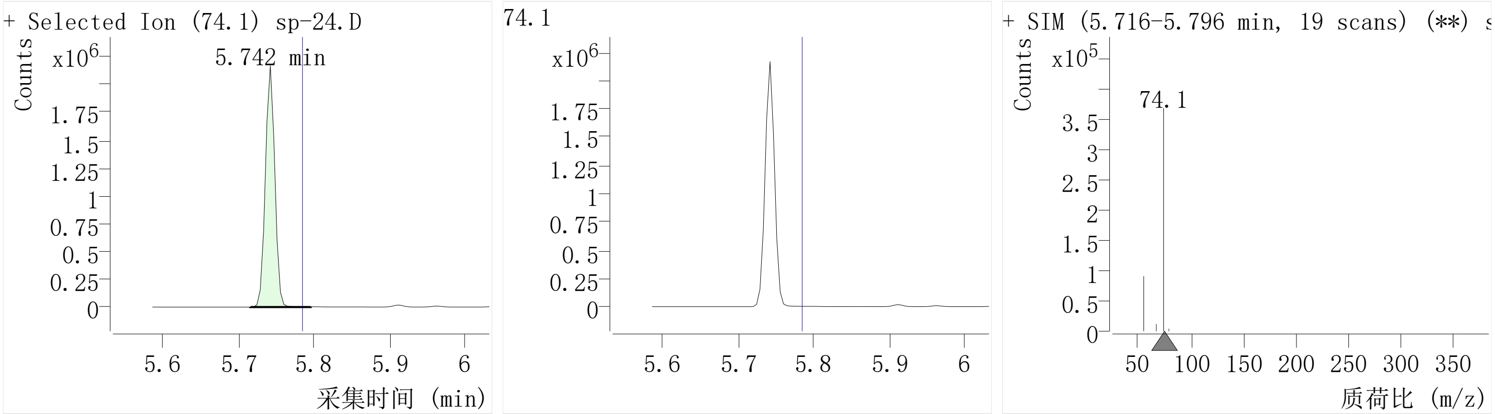

C14:1

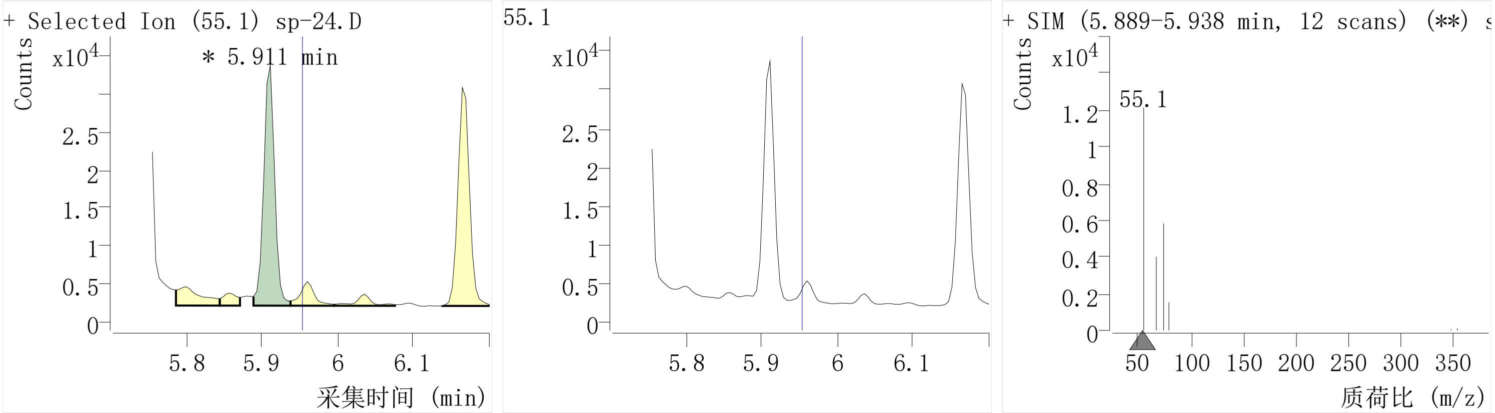

C15:0

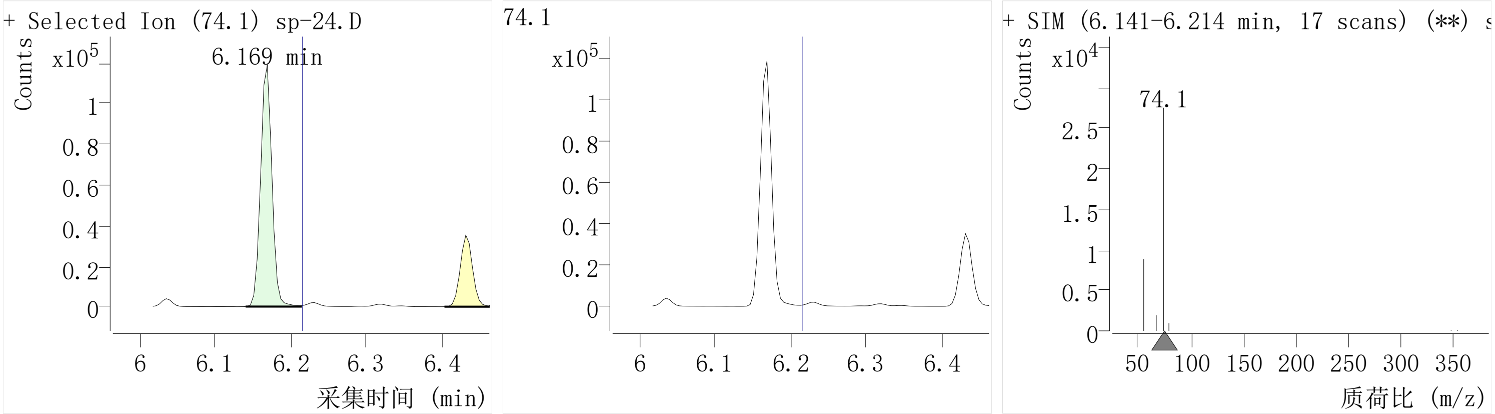

C15:1

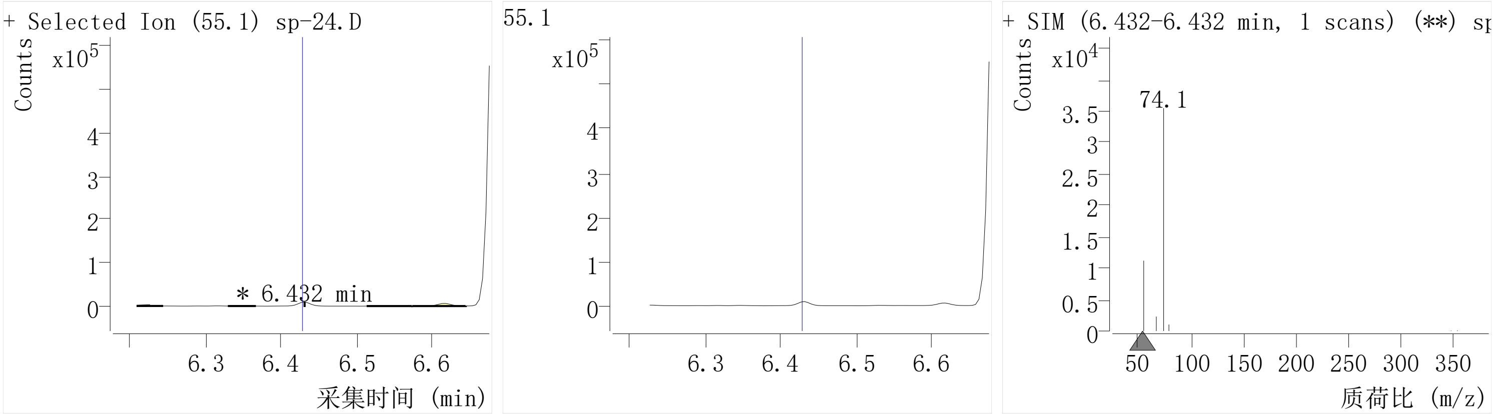

C16:0

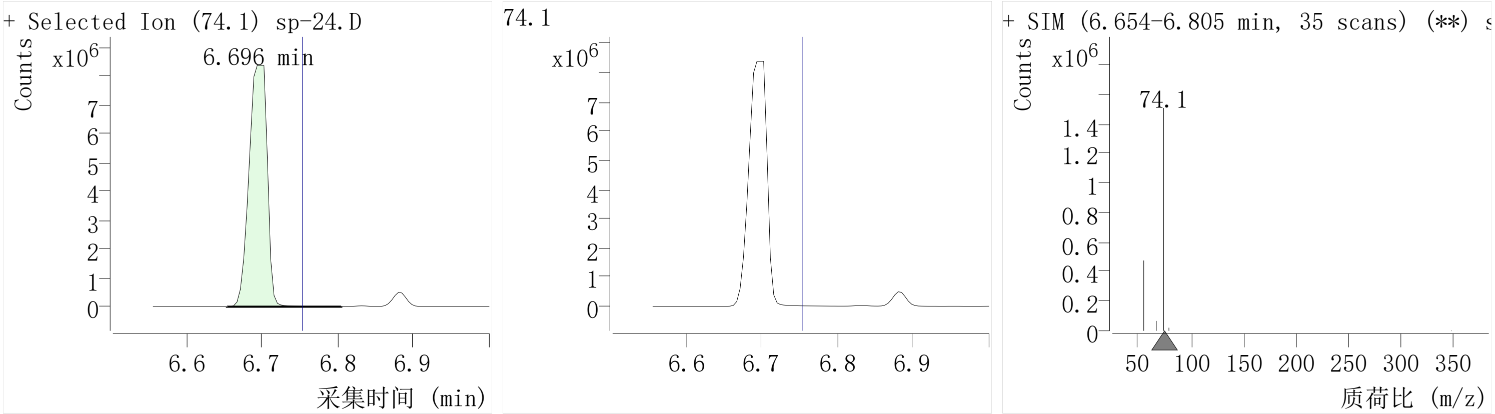

C16:1

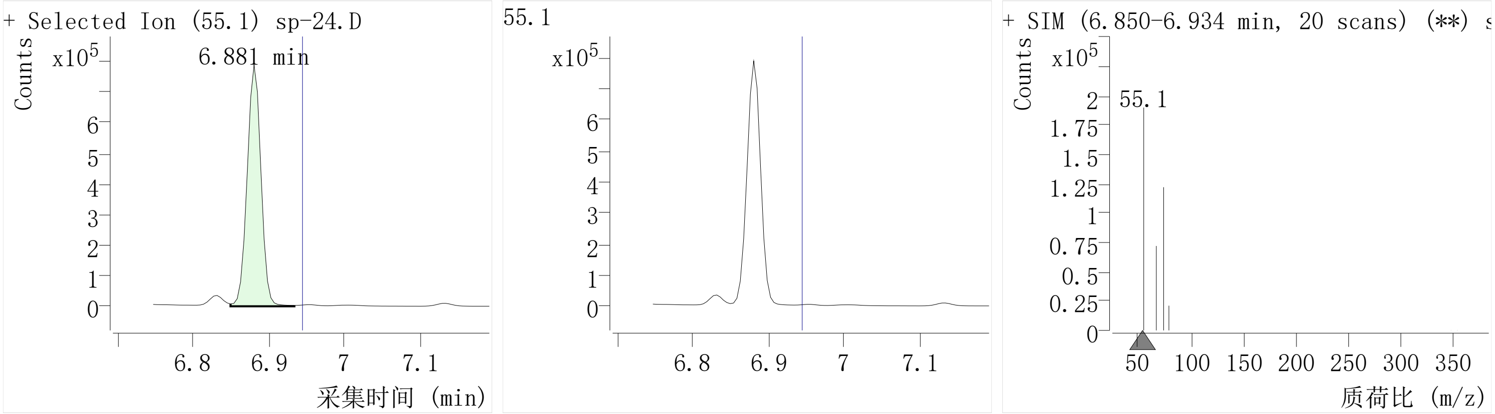

C17:0

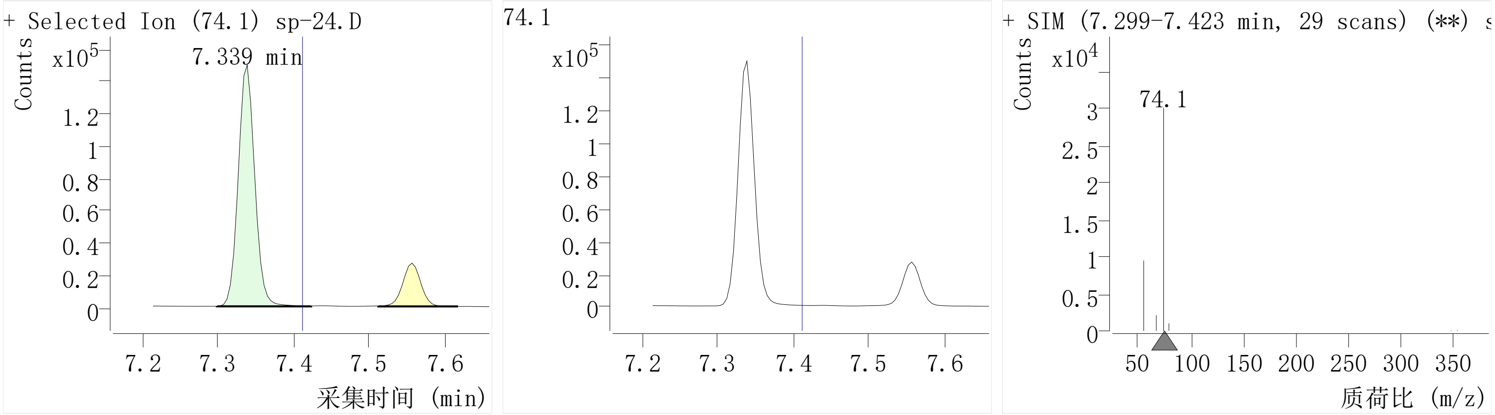

C17:1

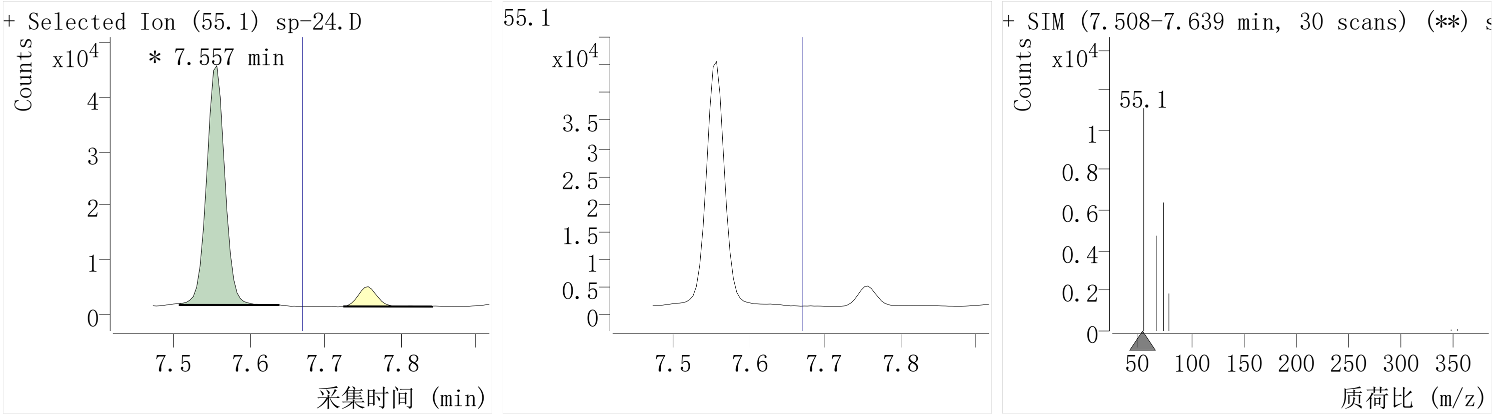

C18:0

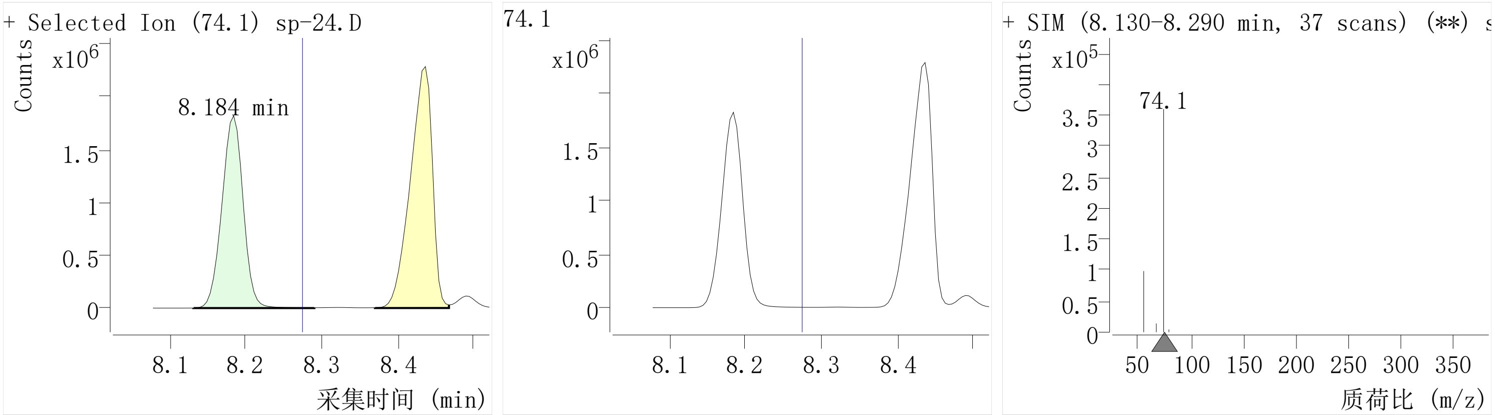

C18:1n9t

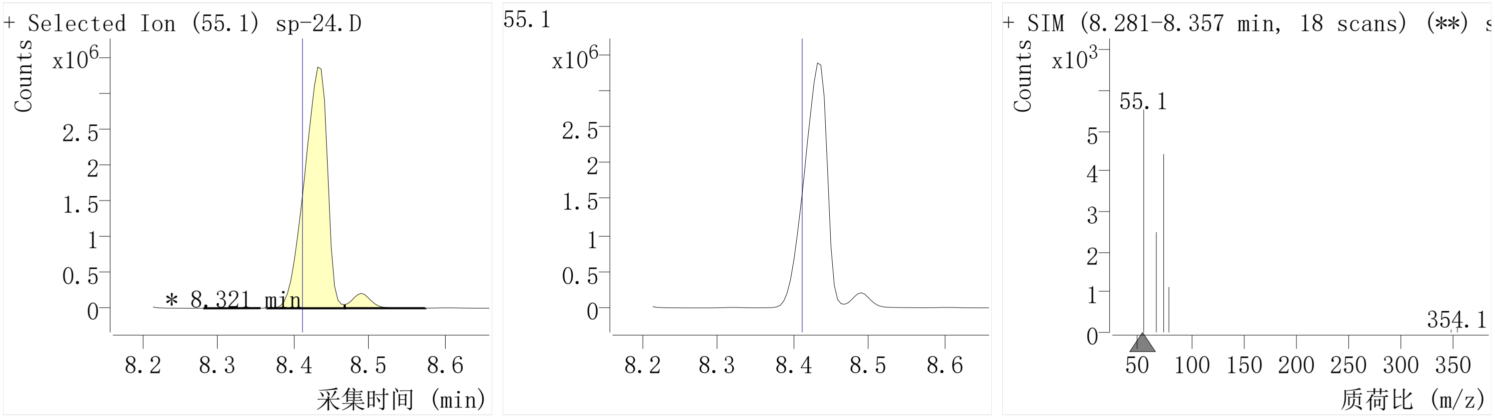

C18:1n9c

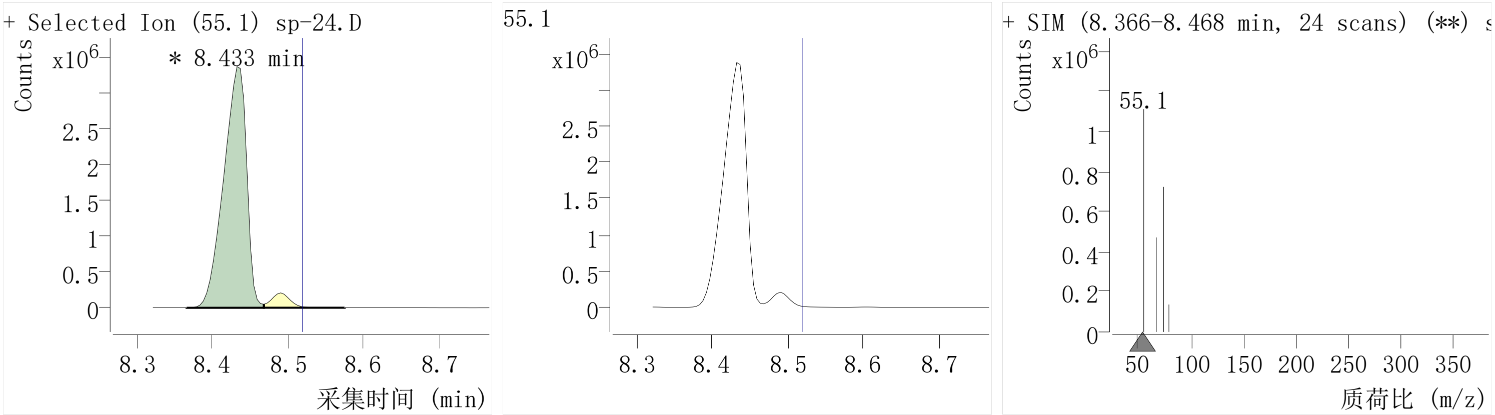

C18:2n6t

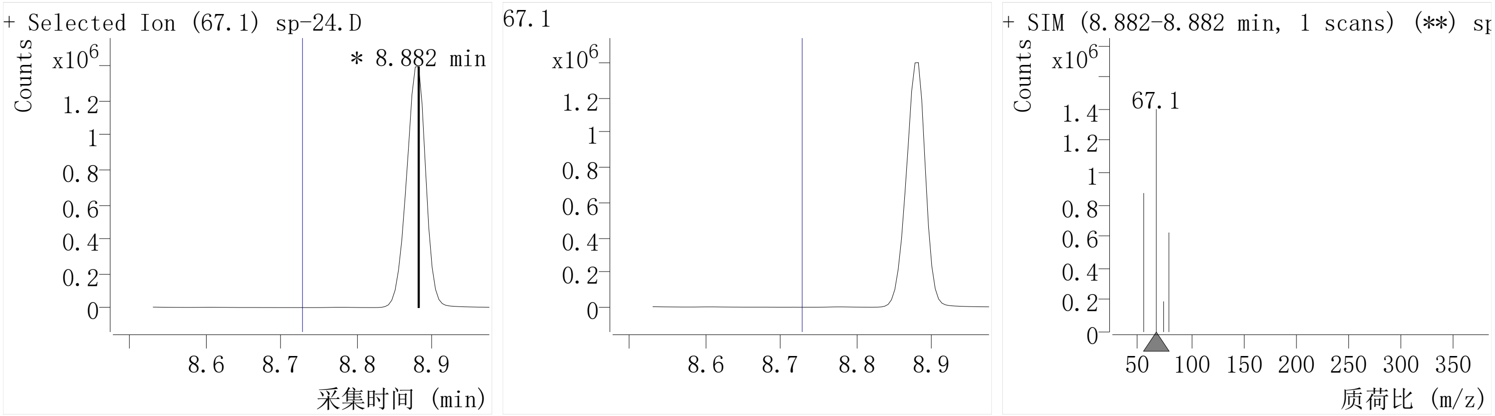

C18:2n6c

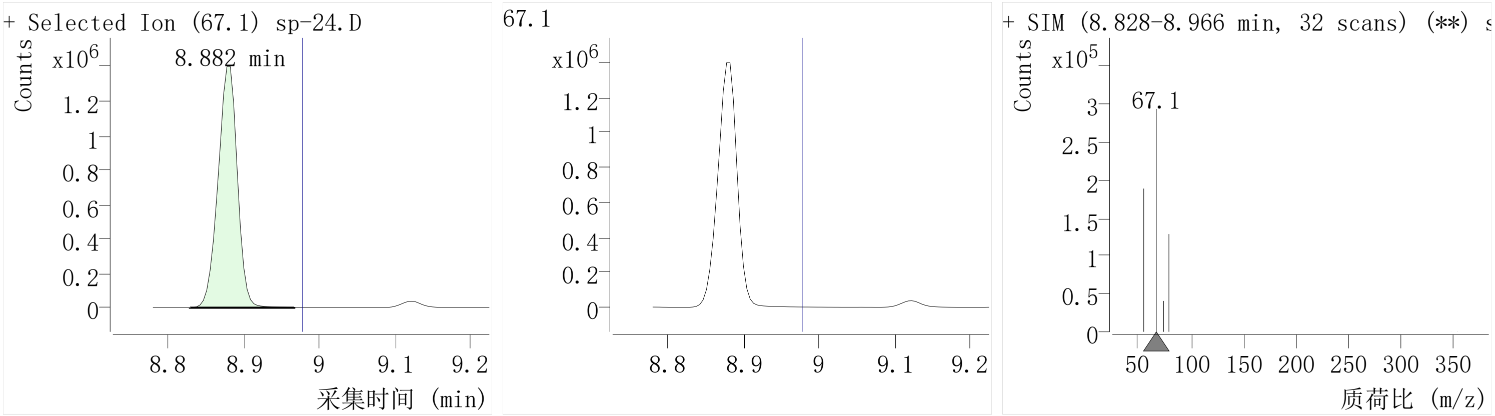

C18:3n6

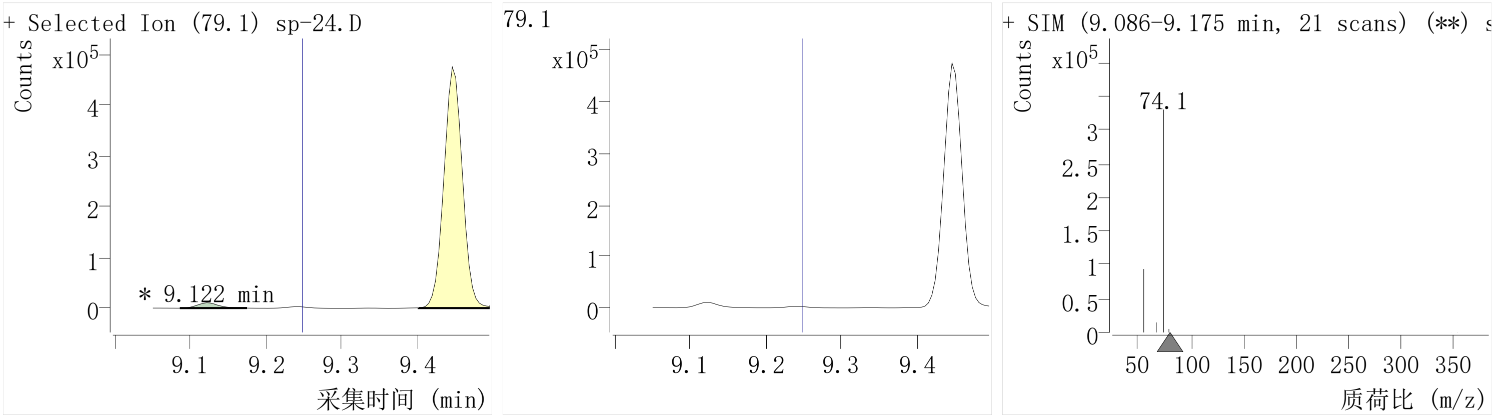

C18:3n3

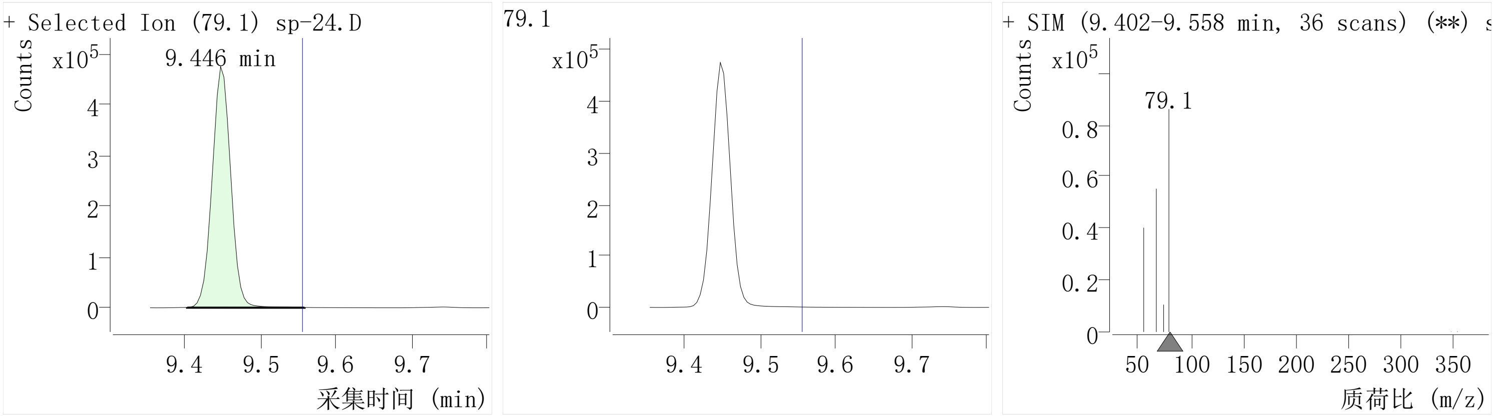

C20:0

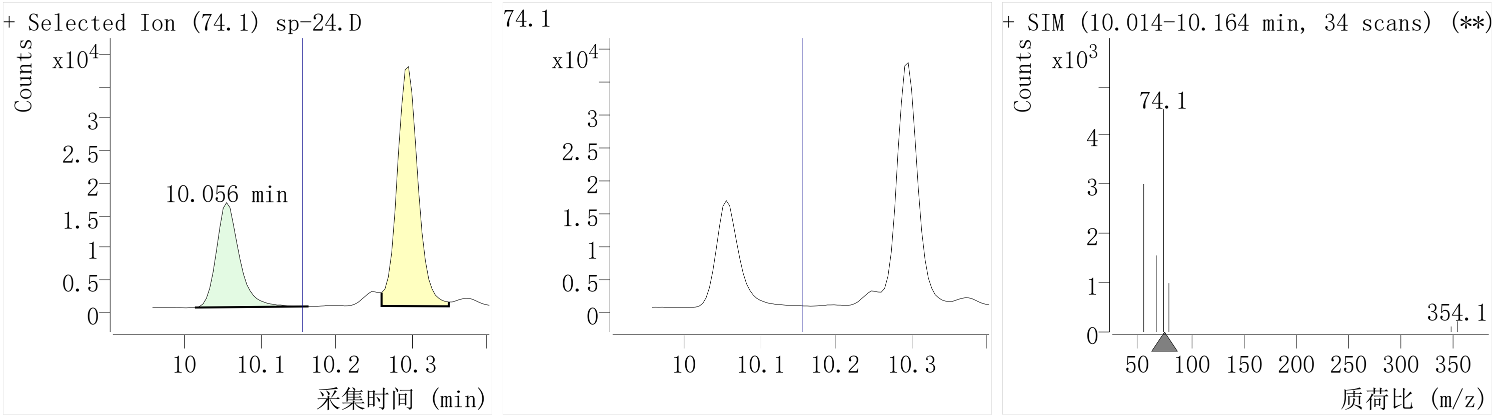

C20:1

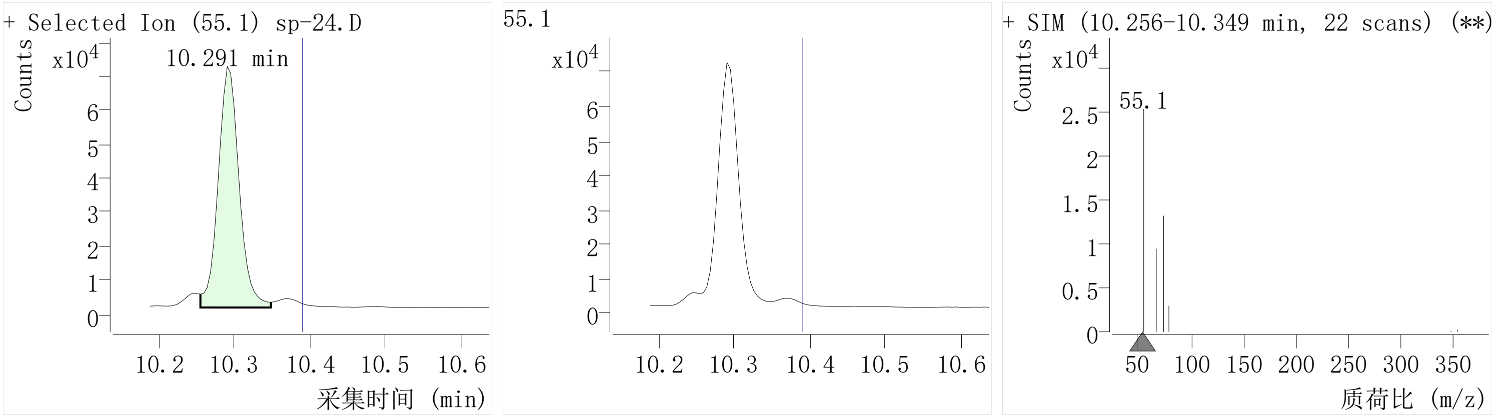

C20:2

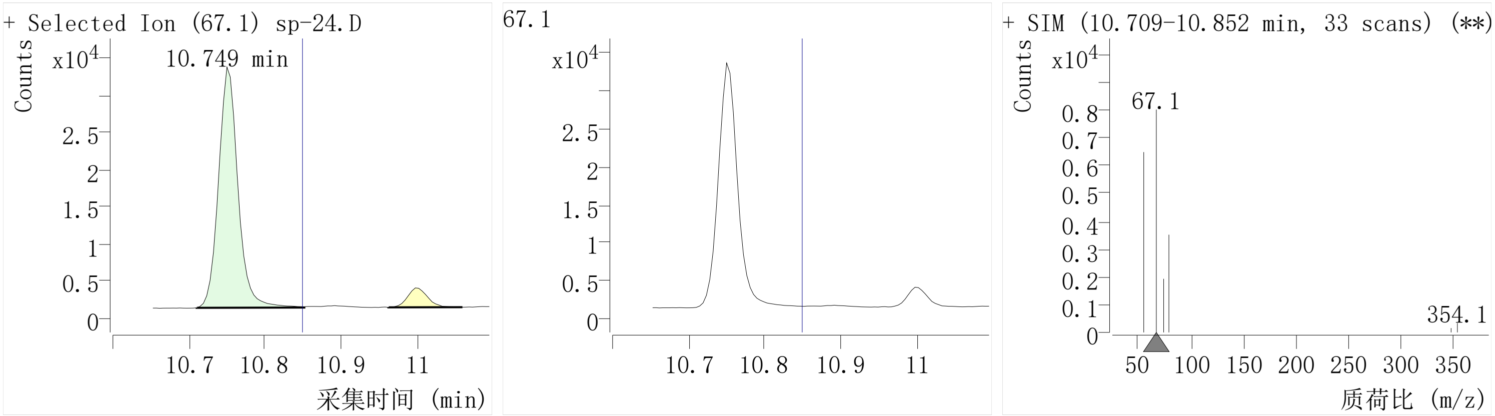

C21:0

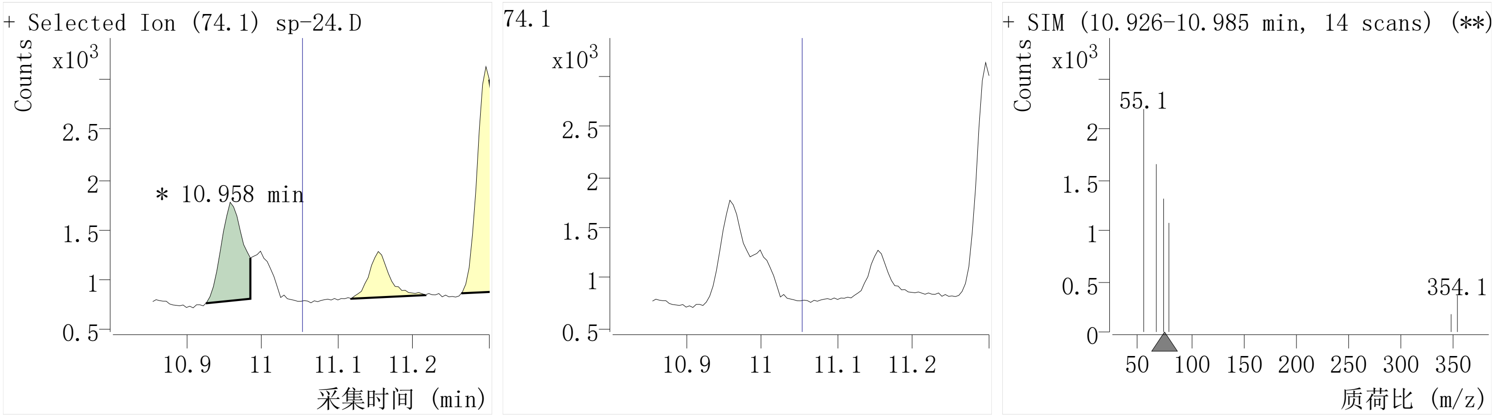

C20:3n6

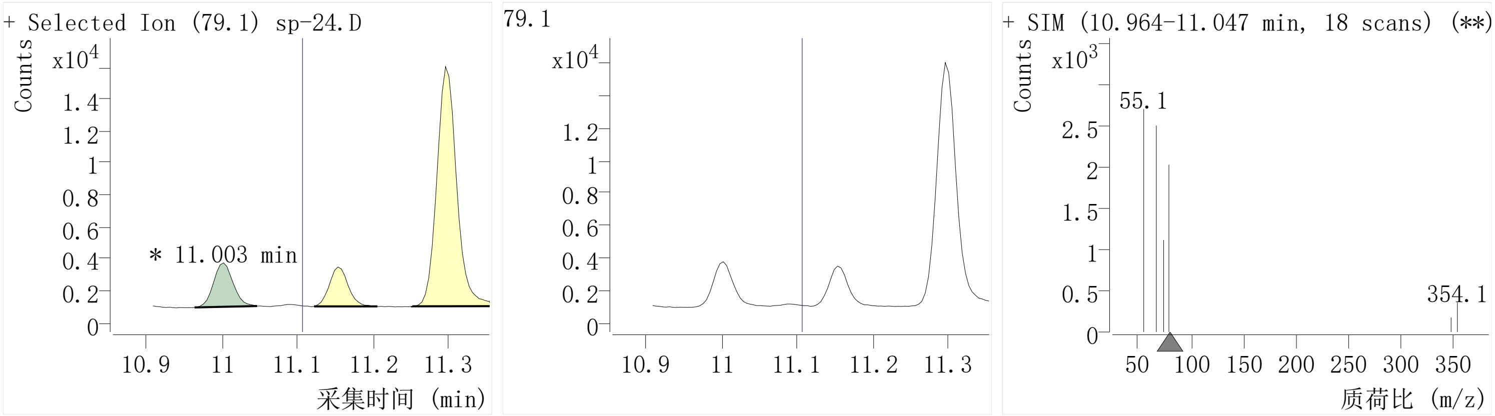

C20:4n6

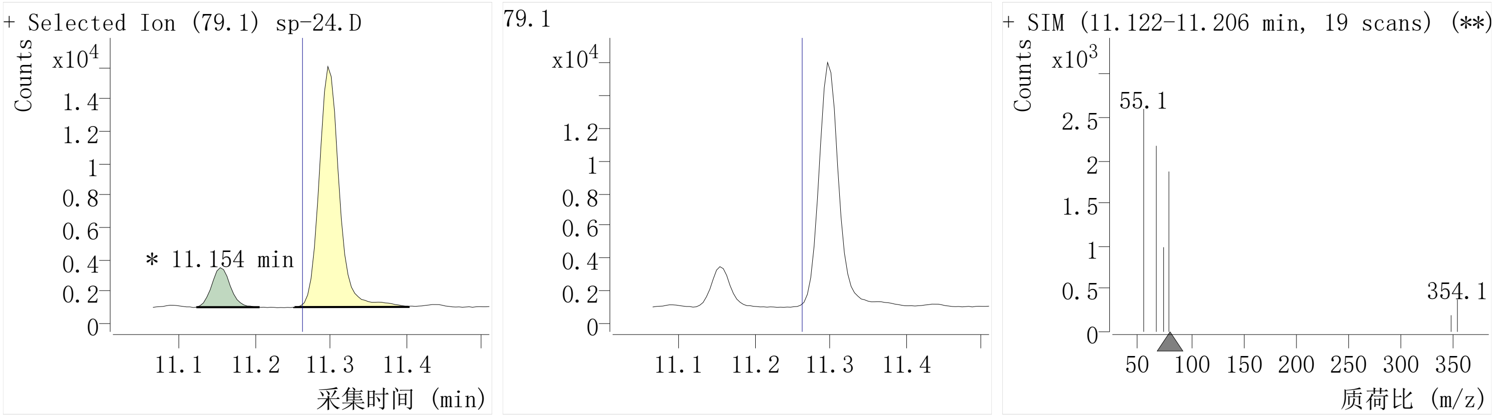

C20:3n3

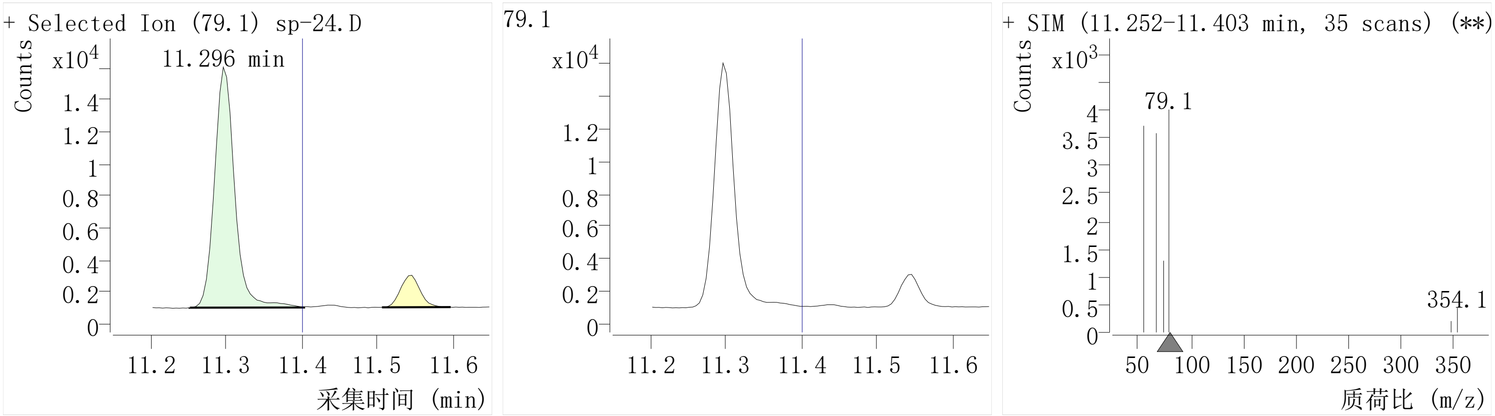

C20:5n3

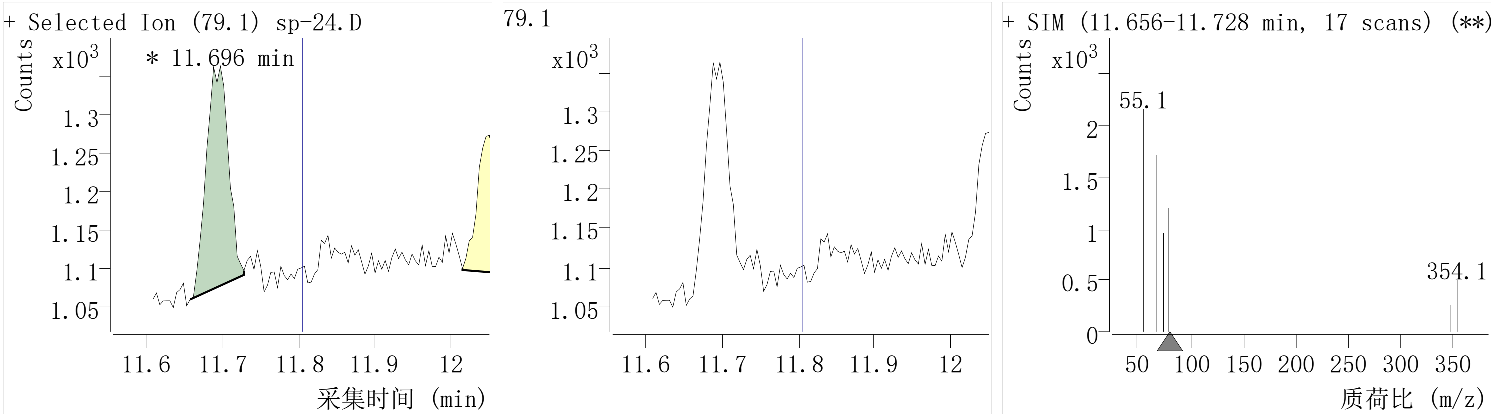

C22:0

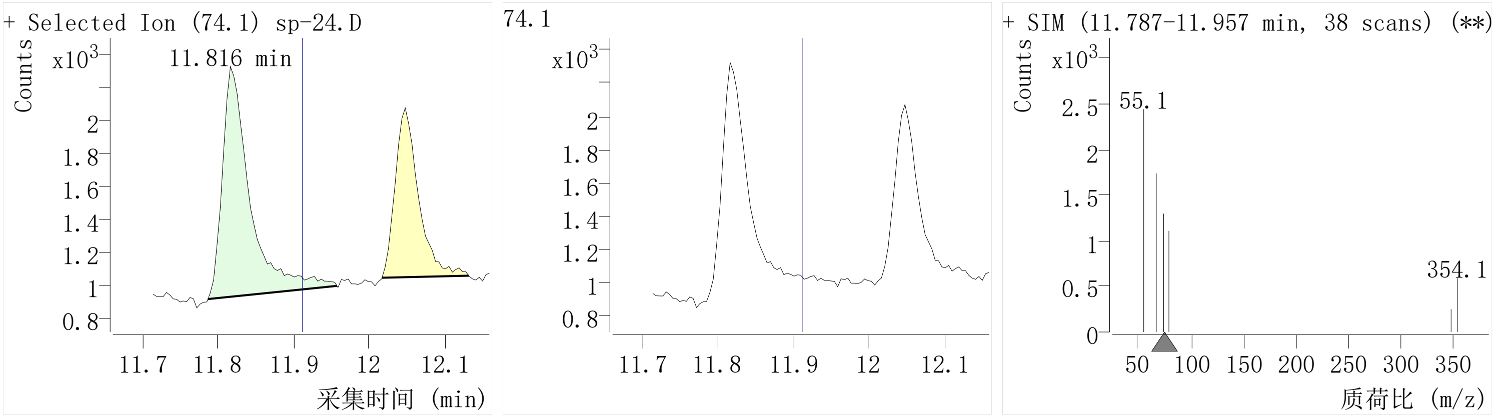

C22:1n9

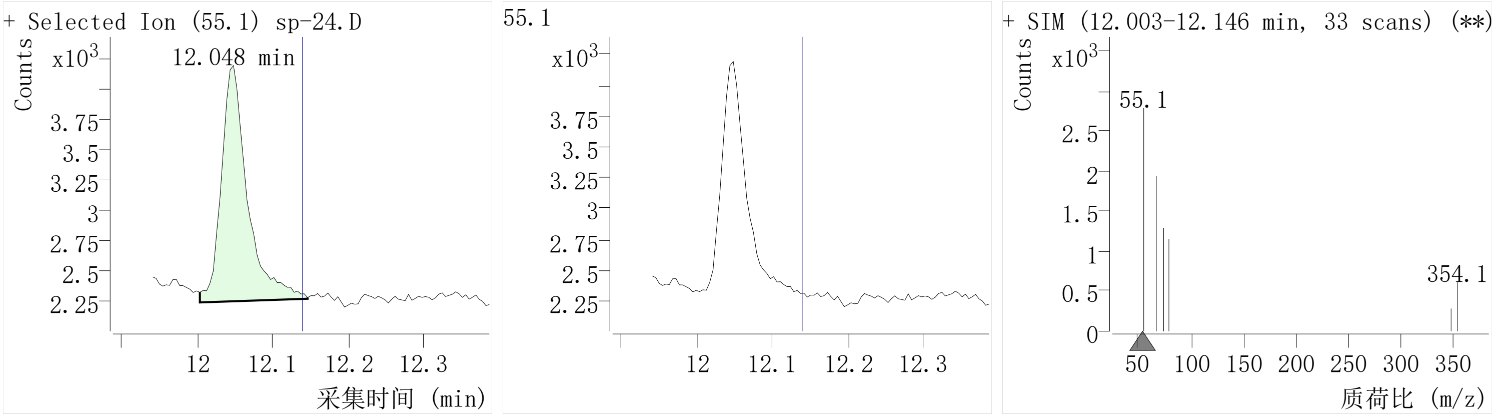

C22:2n6

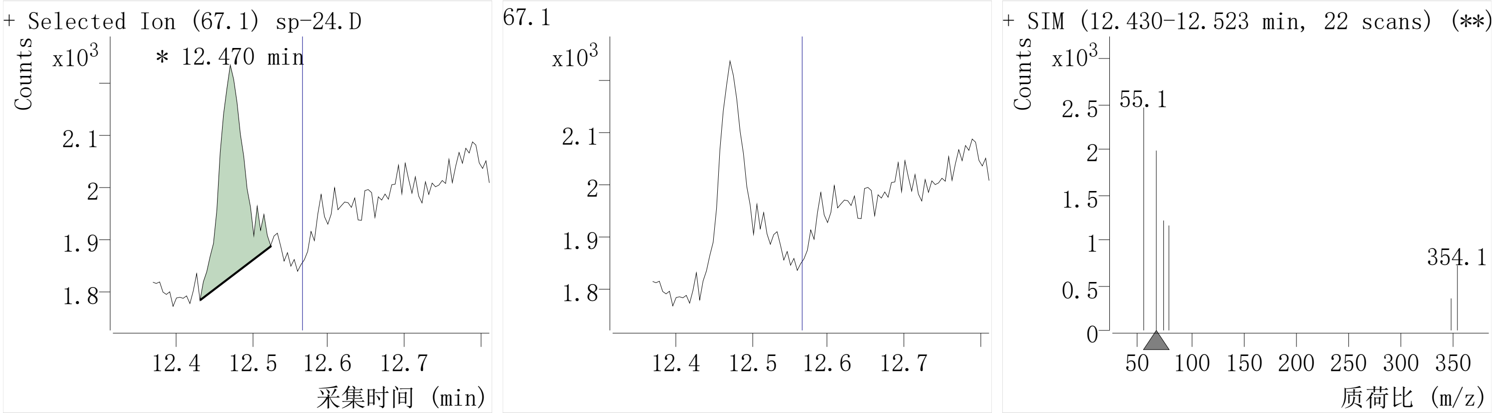

C23:0

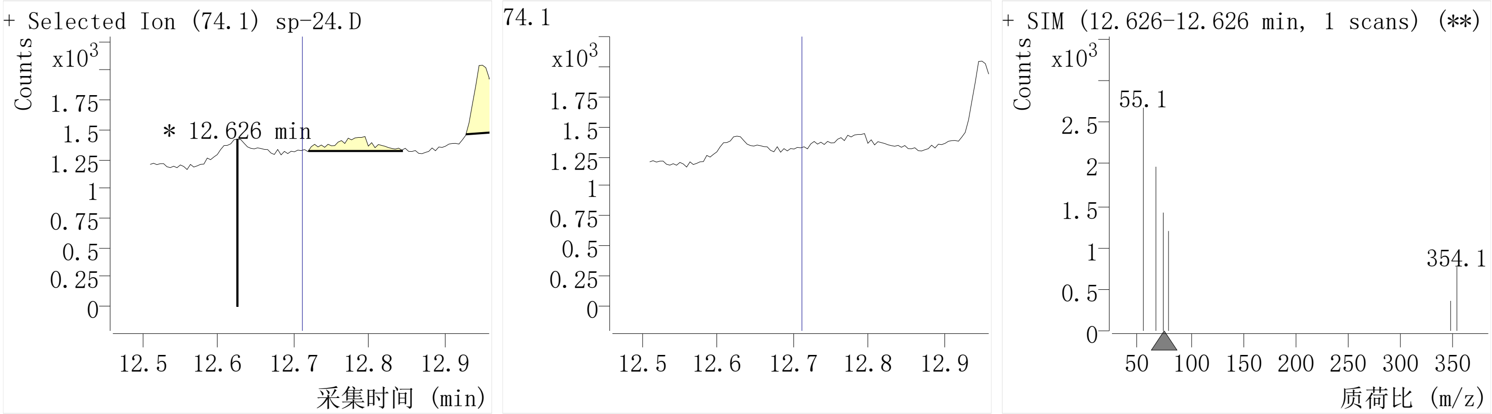

C24:0

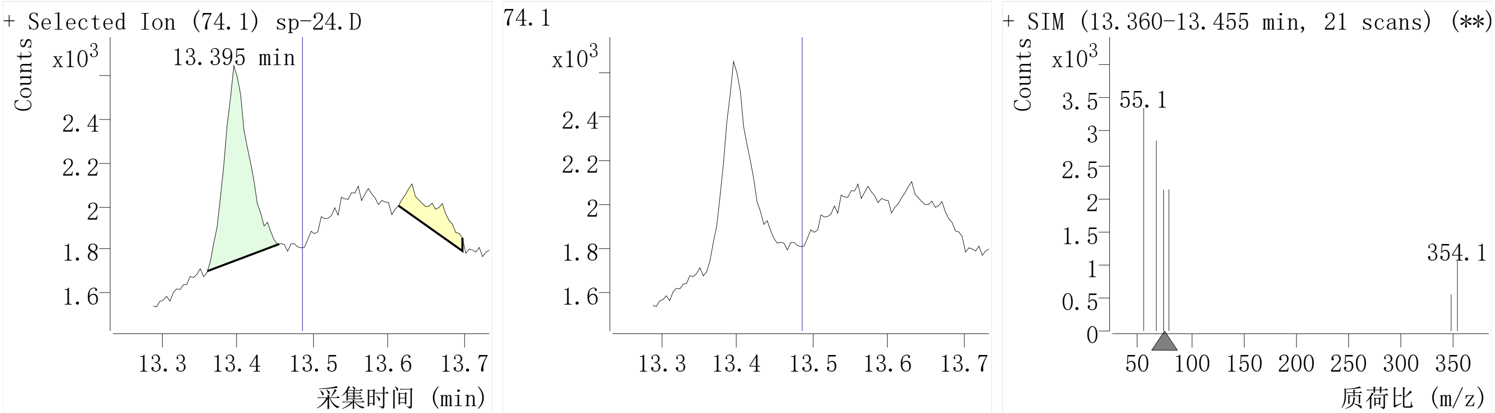

C22:6

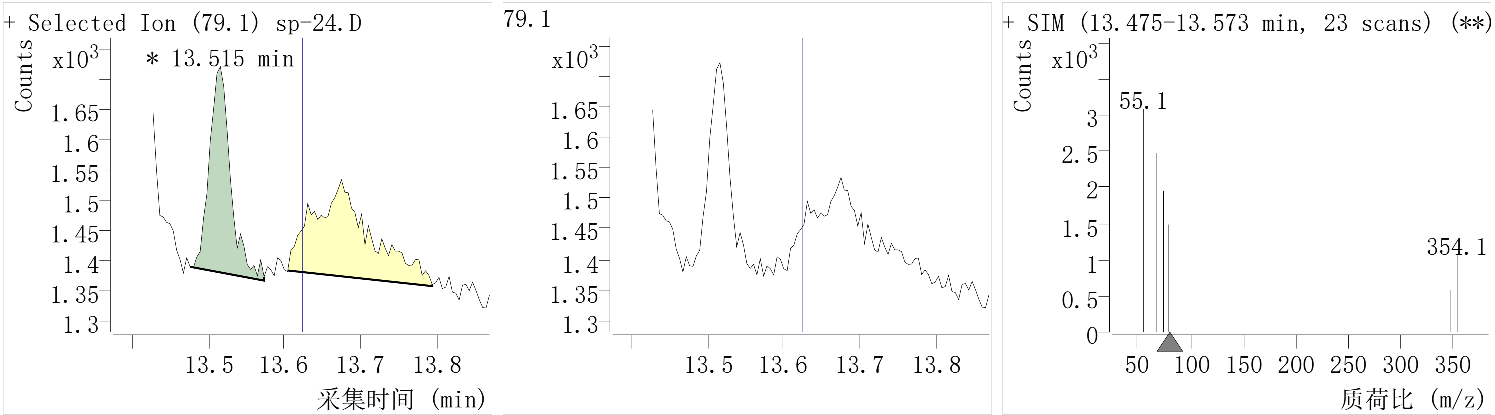
+ Selected Ion (79.1) sp-24.D
Counts
x10^3
* 13.515 min
1.65
1.6
1.55
1.5
1.45
1.4
1.35
1.3
13.5
13.6
13.7
13.8
采集时间 (min)
79.1
+ SIM (13.475-13.573 min, 23 scans) (**)
55.1
354.1
3
2.5
2
1.5
1
0.5
0
50
100
150
200
250
300
350
质荷比 (m/z)

C24:1

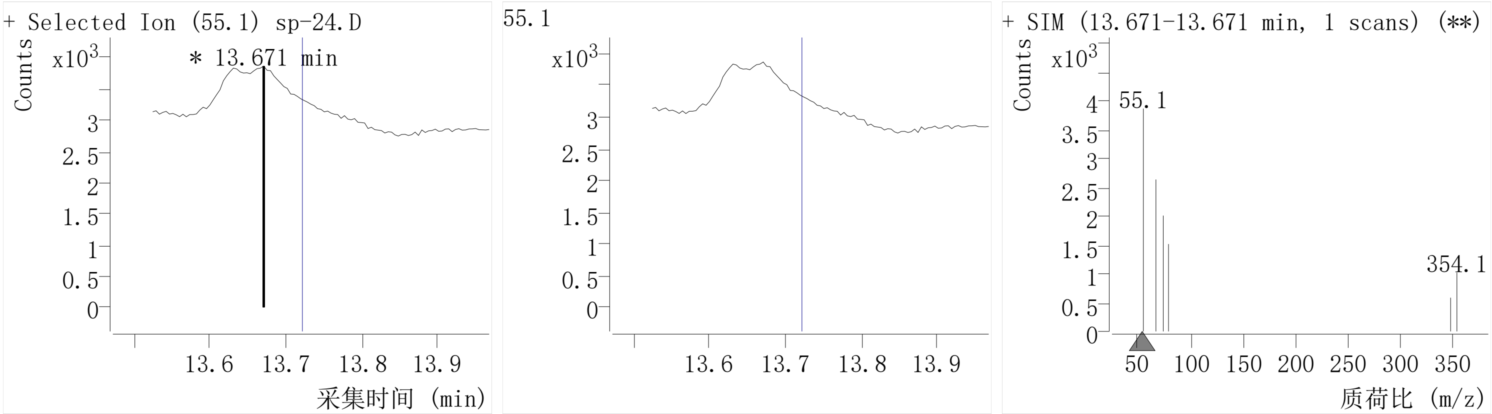
+ Selected Ion (55.1) sp-24.D
Counts
x10^3
* 13.671 min
3
2.5
2
1.5
1
0.5
0
13.6
13.7
13.8
13.9
采集时间 (min)
55.1
+ SIM (13.671-13.671 min, 1 scans) (**)
55.1
354.1
4
3.5
3
2.5
2
1.5
1
0.5
0
50
100
150
200
250
300
350
质荷比 (m/z)

**批处理路径**	G:\GC-MS\HX250430-4-GCMS总脂肪酸靶向检测\HX250430-4\QuantResults\HX250430-4.batch.bin		
**分析时间**	2025/5/14 16:58	**分析员姓名**	DESKTOP-M3A0GPO\omics
**报告时间**	2025/5/16 14:53:26	**报告员姓名**	DESKTOP-M3A0GPO\omics
**最近校正更新**	2025/5/14 16:58	**批处理状态**	已处理
**定量批处理版本**	10.2	**定量报告版本**	10.2

**采集时间**	2025/5/9 6:37	**数据文件**	qc-4.D
**样品类型**	样品	**样品名称**	qc-4
**稀释**	1	**采集方法**	脂肪酸

样品色谱图

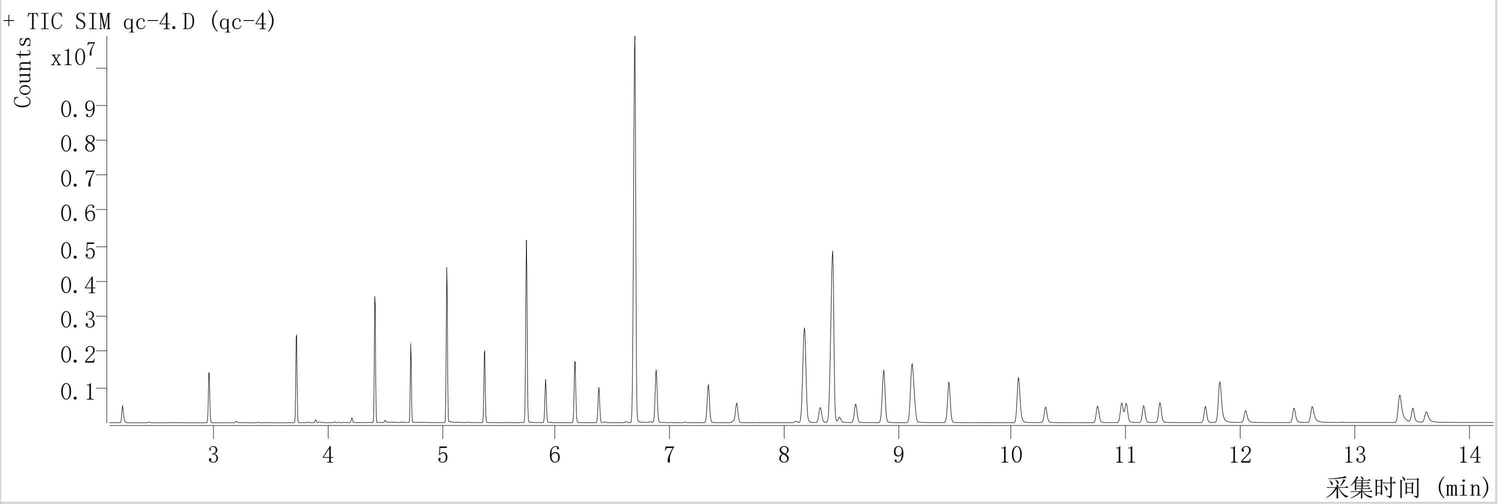

化合物	ISTD	RT	响应	ISTD 响应	响应比	最终浓度	单位
C4:0	C19:0	2.203	467088	2169029	0.2153	41.1755	ug/ml
C6:0	C19:0	2.959	1045244	2169029	0.4819	57.8937	ug/ml
C8:0	C19:0	3.728	1599633	2169029	0.7375	54.1189	ug/ml
C10:0	C19:0	4.413	2197761	2169029	1.0132	41.6526	ug/ml
C11:0	C19:0	4.729	1239199	2169029	0.5713	18.8763	ug/ml
C12:0	C19:0	5.044	2636005	2169029	1.2153	35.3255	ug/ml
C13:0	C19:0	5.378	1361368	2169029	0.6276	15.8435	ug/ml
C14:0	C19:0	5.742	3614151	2169029	1.6663	39.4991	ug/ml
C14:1	C19:0	5.911	574306	2169029	0.2648	13.2489	ug/ml
C15:0	C19:0	6.169	1365754	2169029	0.6297	13.6060	ug/ml
C15:1	C19:0	6.378	564314	2169029	0.2602	13.1432	ug/ml
C16:0	C19:0	6.694	11047058	2169029	5.0931	169.3840	ug/ml
C16:1	C19:0	6.881	898005	2169029	0.4140	25.2124	ug/ml
C17:0	C19:0	7.339	1215142	2169029	0.5602	11.6879	ug/ml
C17:1	C19:0	7.588	466644	2169029	0.2151	11.5582	ug/ml
C18:0	C19:0	8.179	4077123	2169029	1.8797	40.9039	ug/ml
C18:1n9t	C19:0	8.321	380372	2169029	0.1754	10.3447	ug/ml
C18:1n9c	C19:0	8.428	4211407	2169029	1.9416	125.1705	ug/ml
C18:2n6t	C19:0	8.628	420052	2169029	0.1937	10.6351	ug/ml
C18:2n6c	C19:0	8.877	1185916	2169029	0.5467	31.5031	ug/ml
C18:3n6	C19:0	9.144	429355	2169029	0.1979	10.3285	ug/ml
C18:3n3	C19:0	9.446	893811	2169029	0.4121	20.4816	ug/ml
C20:0	C19:0	10.056	1835193	2169029	0.8461	21.3760	ug/ml
C20:1	C19:0	10.296	417234	2169029	0.1924	11.8940	ug/ml
C20:2	C19:0	10.749	377337	2169029	0.1740	10.6852	ug/ml
C21:0	C19:0	10.963	890100	2169029	0.4104	11.2893	ug/ml
C20:3n6	C19:0	11.003	355934	2169029	0.1641	10.4080	ug/ml
C20:4n6	C19:0	11.154	384809	2169029	0.1774	11.0978	ug/ml
C20:3n3	C19:0	11.301	433169	2169029	0.1997	10.4920	ug/ml
C20:5n3	C19:0	11.696	392915	2169029	0.1811	10.0467	ug/ml

化合物	ISTD	RT	响应	ISTD 响应	响应比	最终浓度	单位
C22:0	C19:0	11.821	1687522	2169029	0.7780	25.2102	ug/ml
C22:1n9	C19:0	12.048	353032	2169029	0.1628	12.0347	ug/ml
C22:2n6	C19:0	12.475	324202	2169029	0.1495	11.8490	ug/ml
C23:0	C19:0	12.635	769809	2169029	0.3549	13.6865	ug/ml
C24:0	C19:0	13.399	1415396	2169029	0.6525	29.4311	ug/ml
C22:6	C19:0	13.515	359436	2169029	0.1657	12.2464	ug/ml
C24:1	C19:0	13.631	358876	2169029	0.1655	14.5880	ug/ml

C4:0

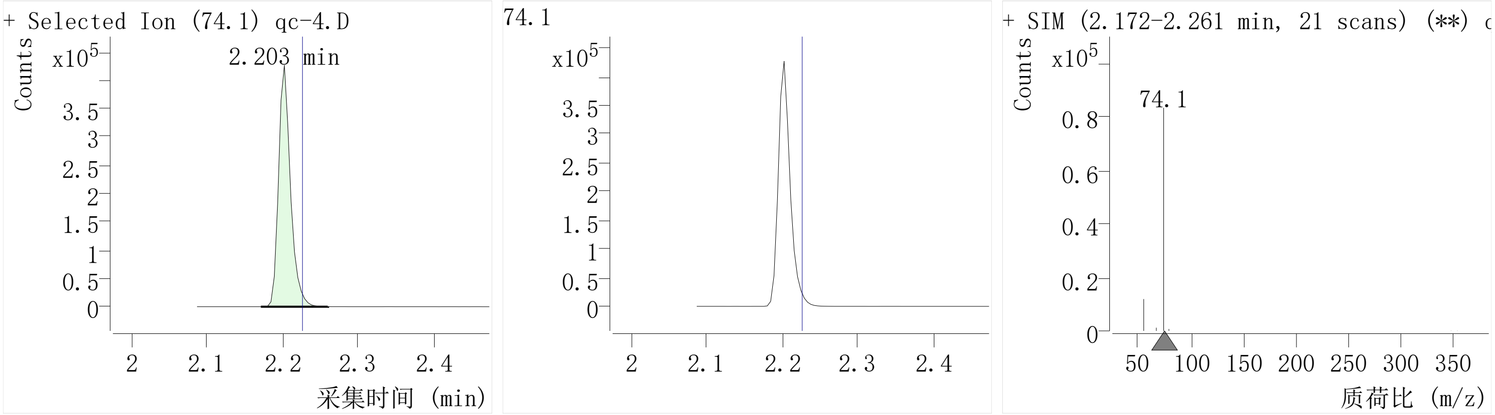

C6:0

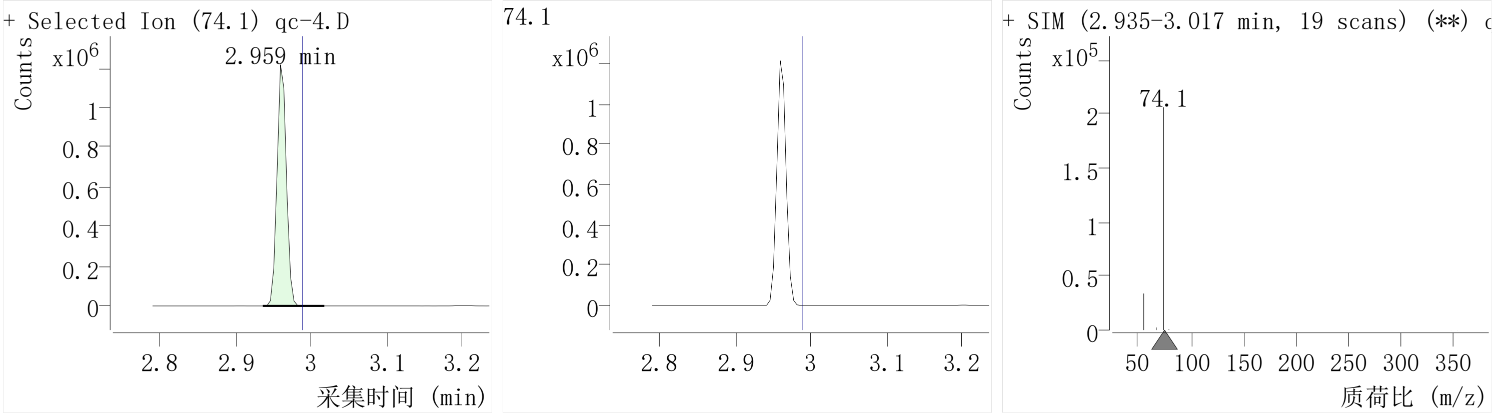

C8:0

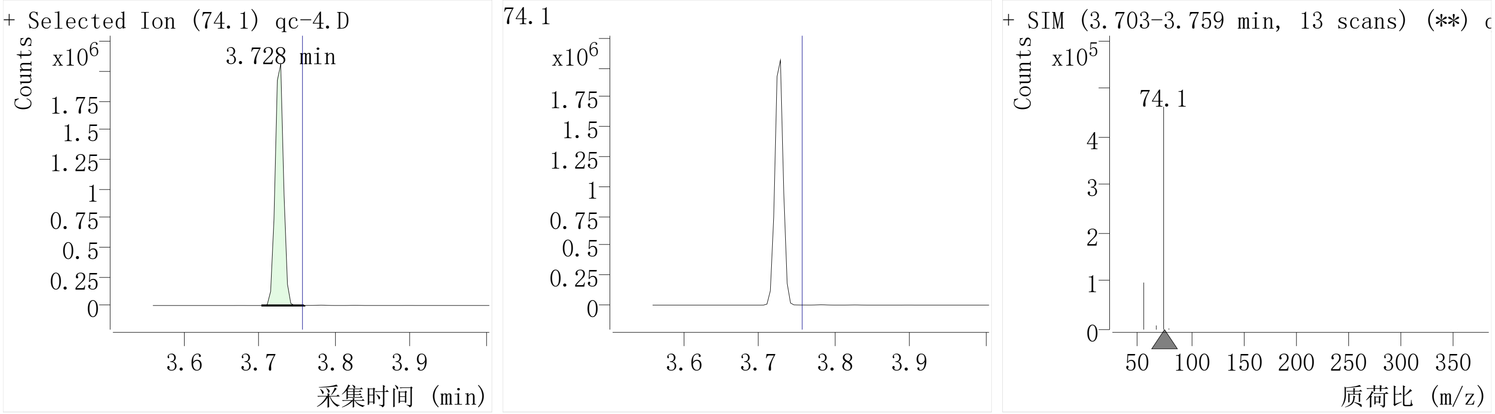

C10:0

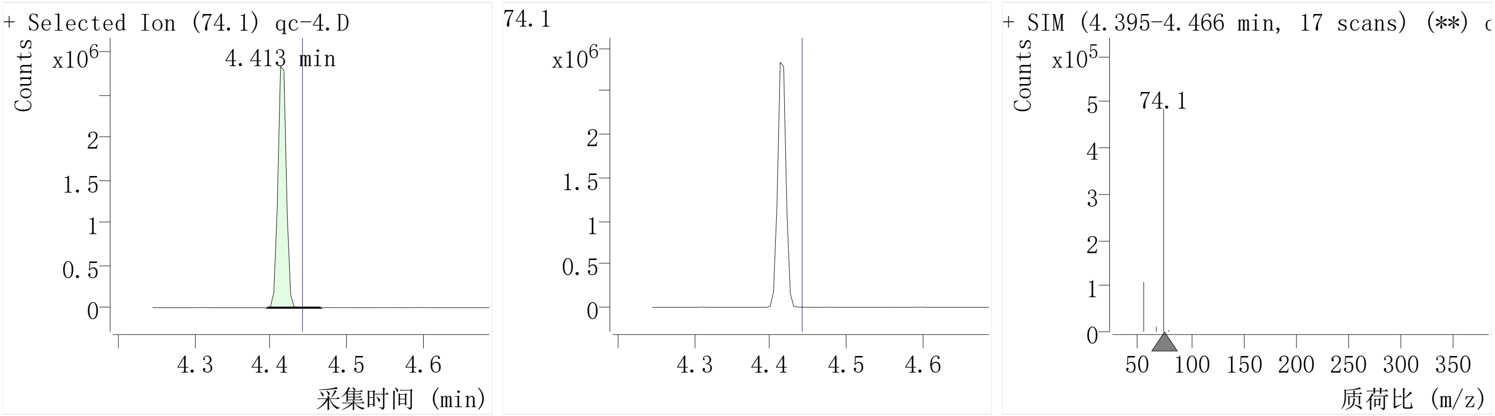

C11:0

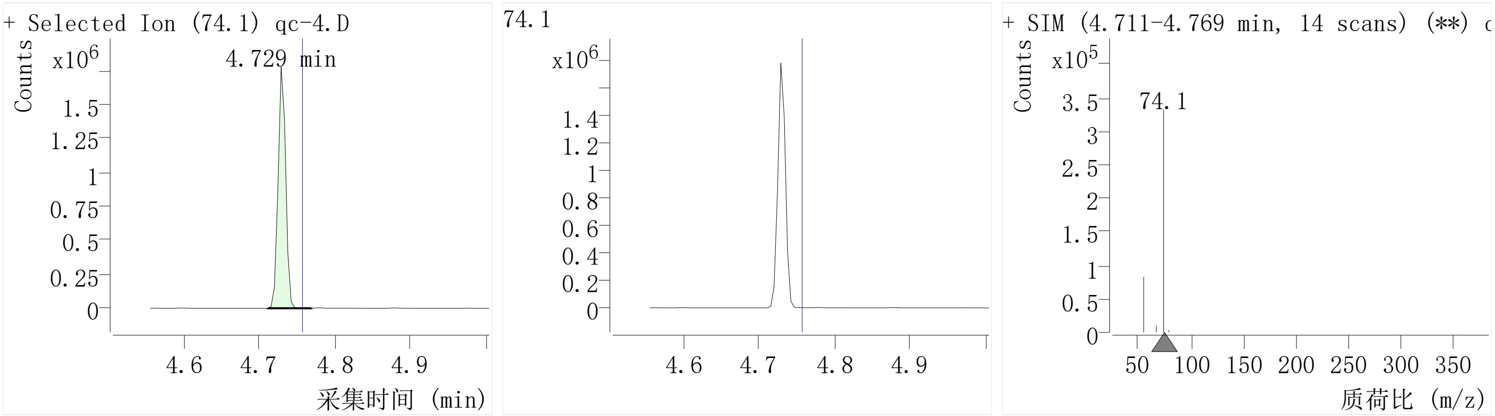

C12:0

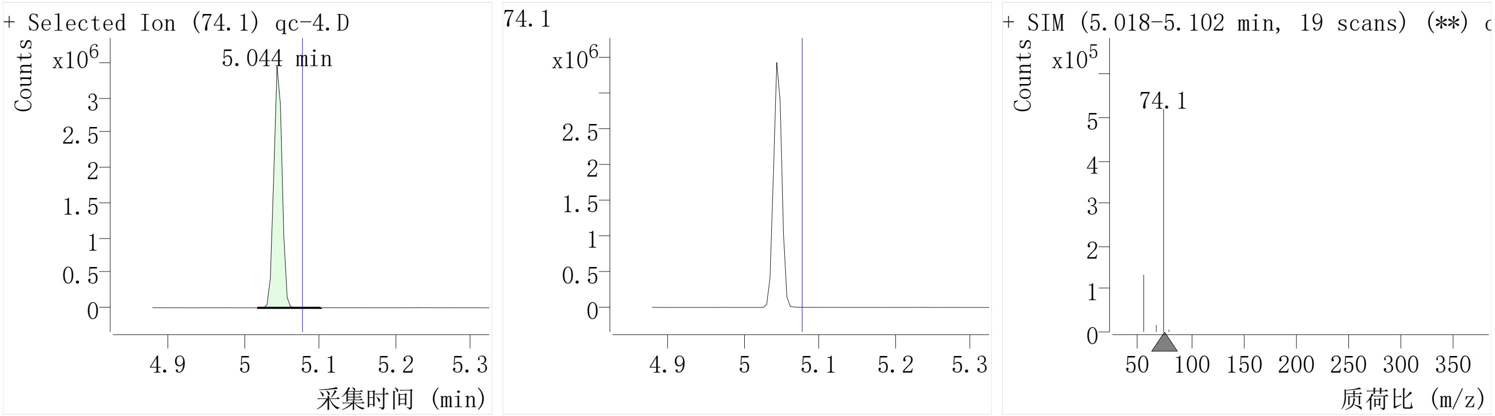

C13:0

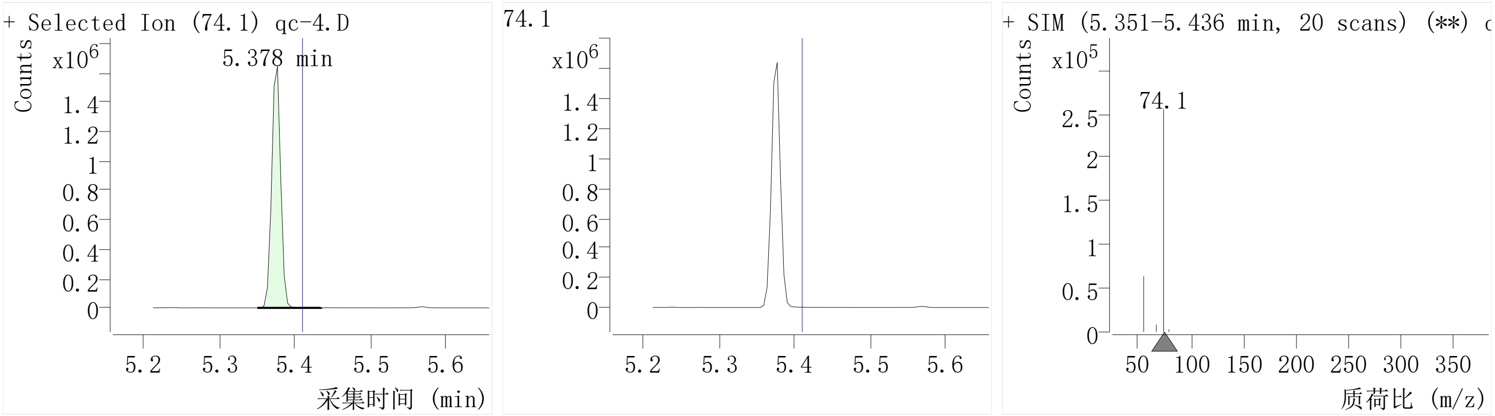

C14:0

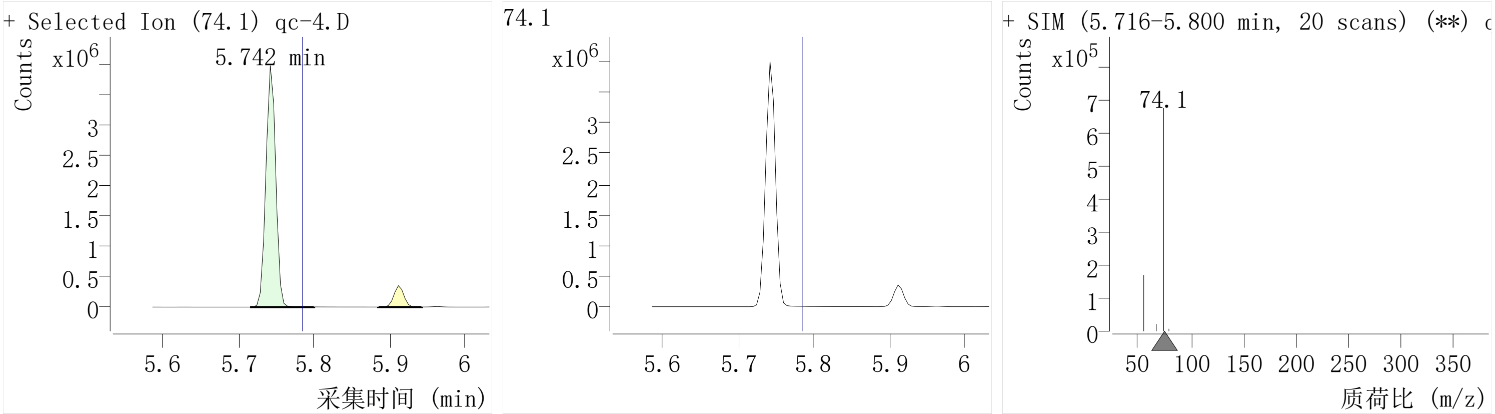

C14:1

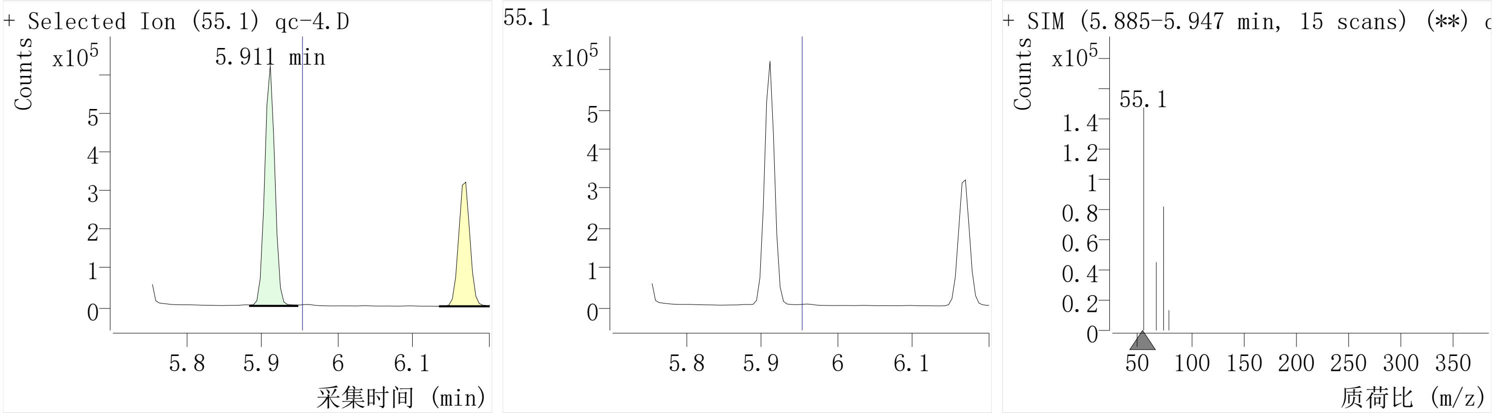

C15:0

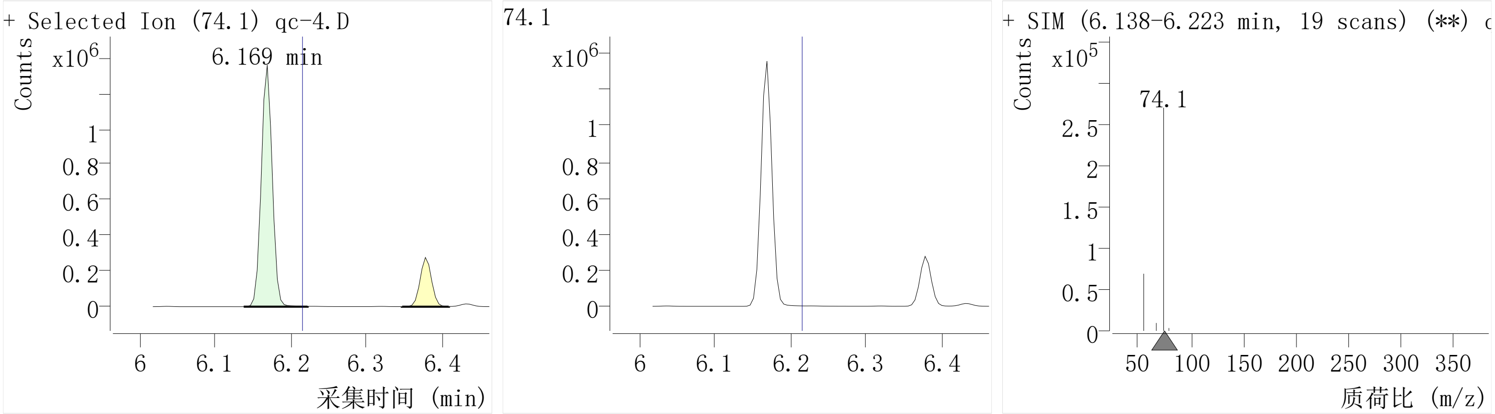

C15:1

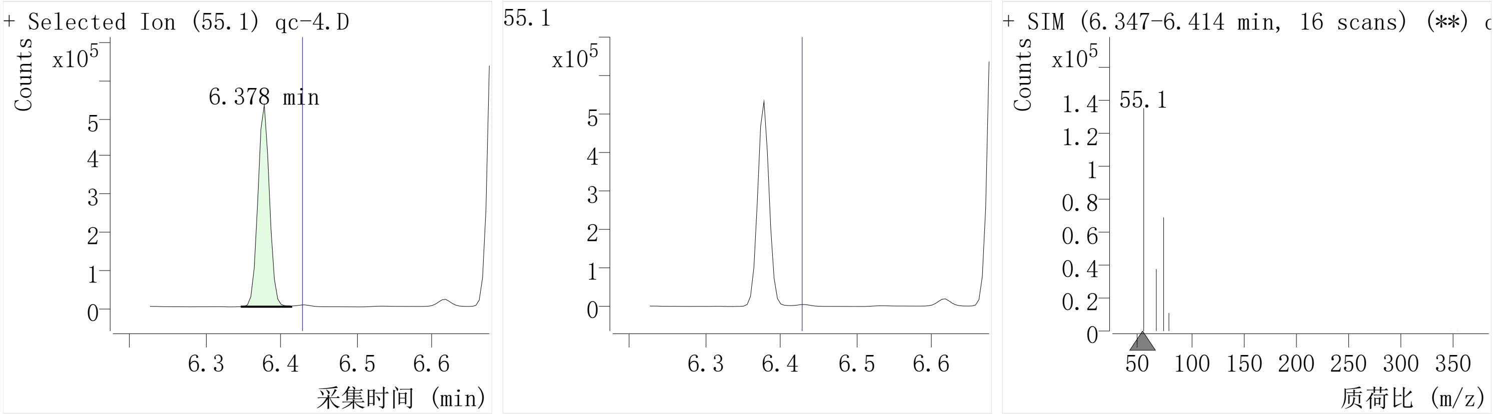

C16:0

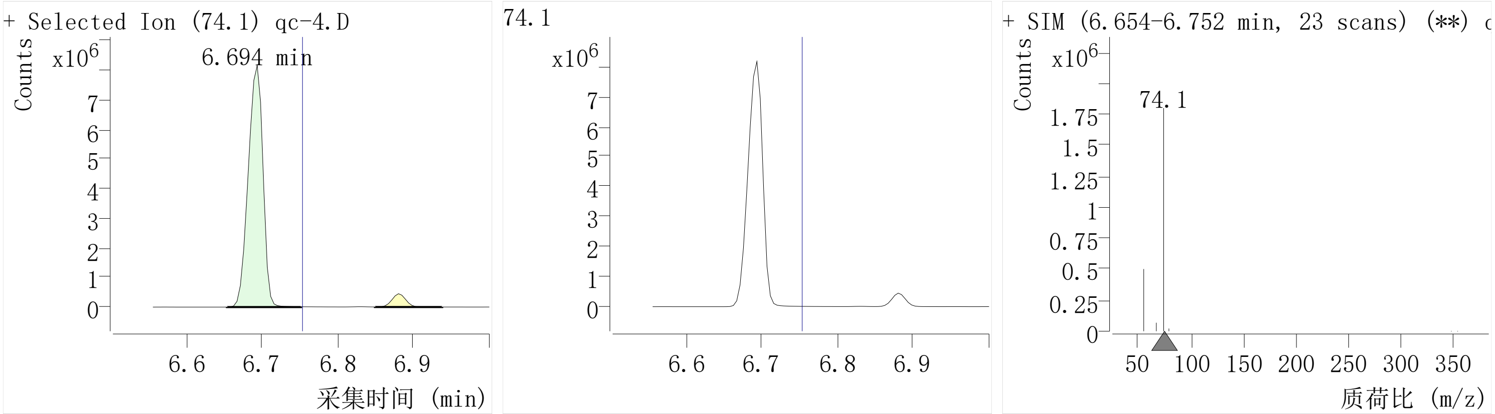

C16:1

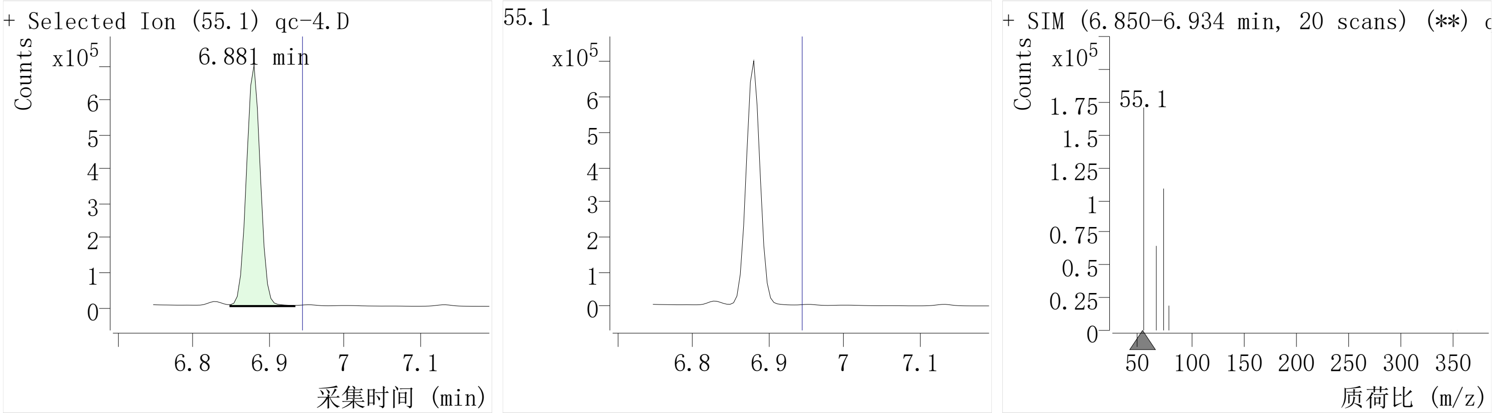

C17:0

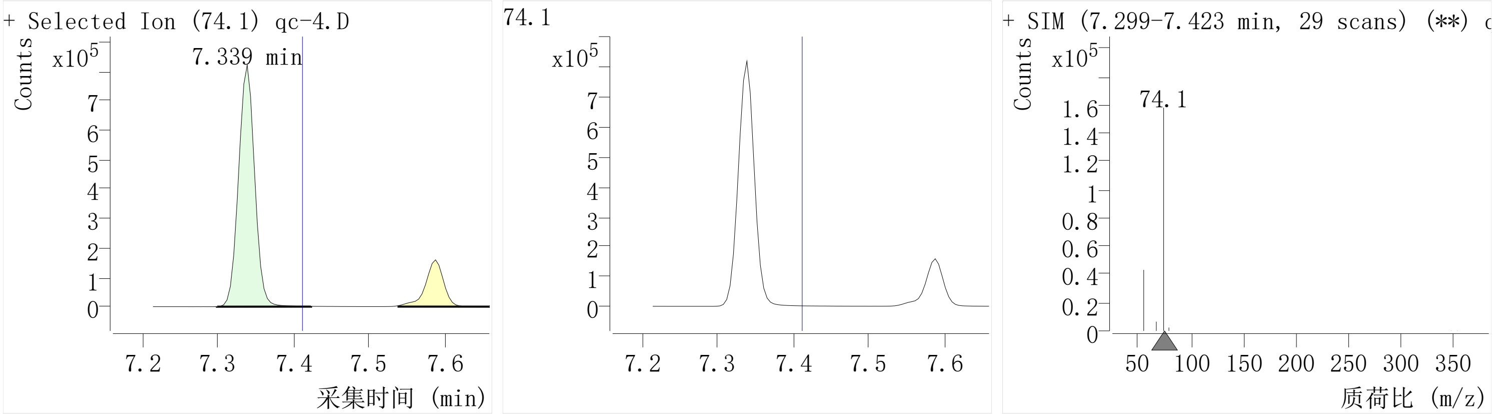

C17:1

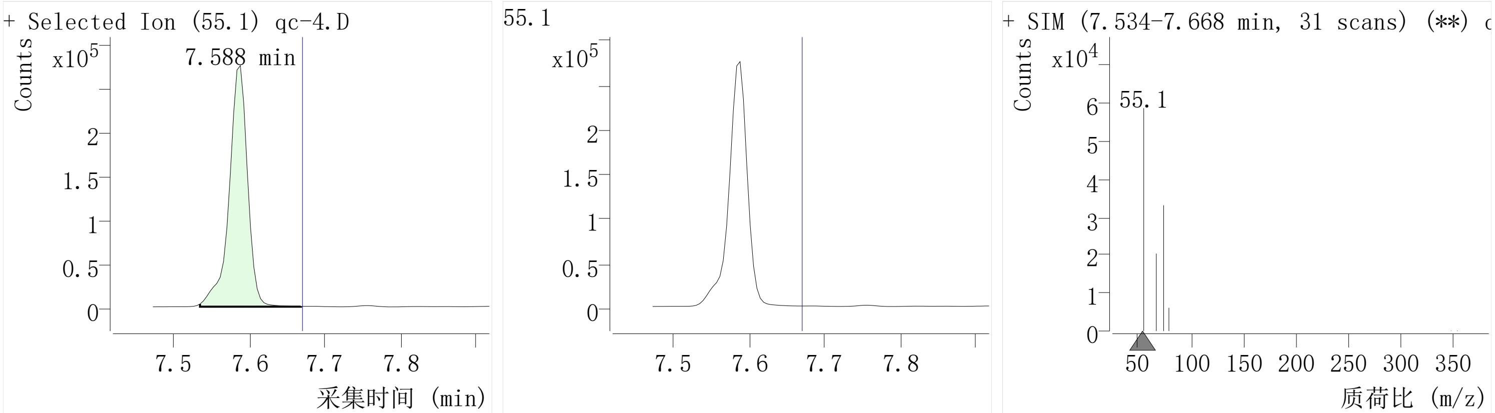

C18:0

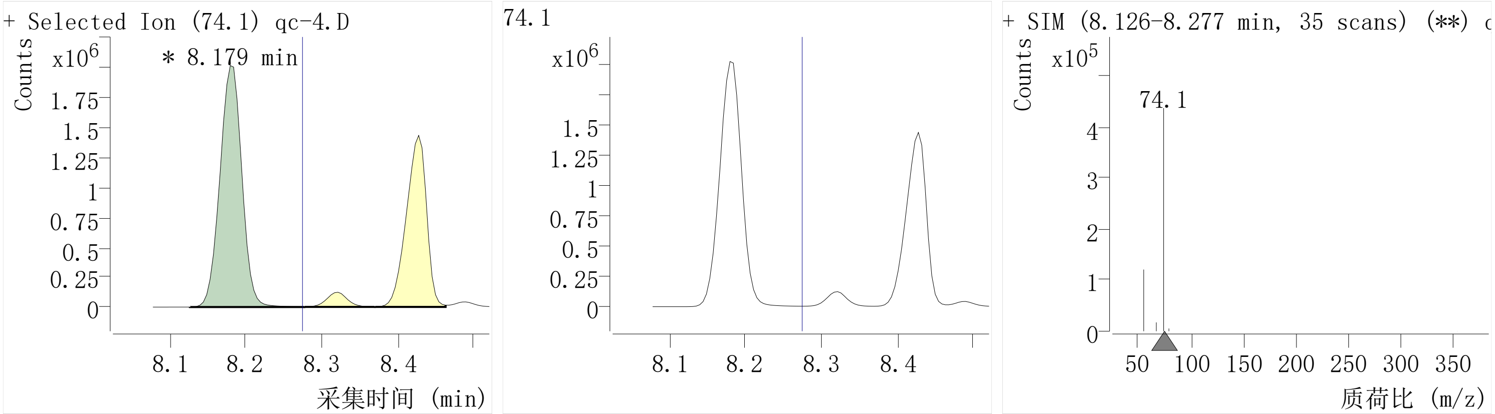

C18:1n9t

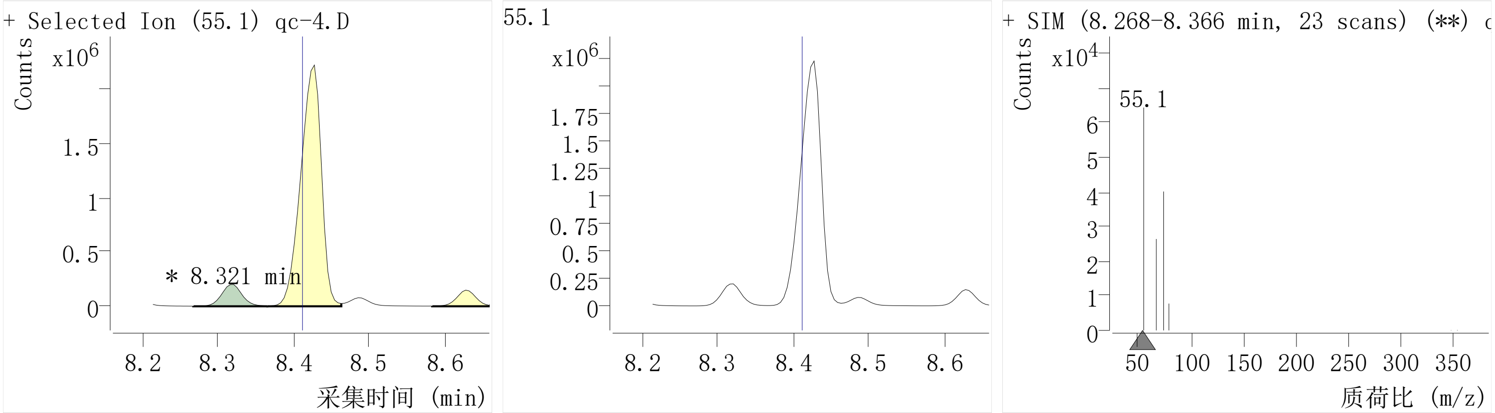

C18:1n9c

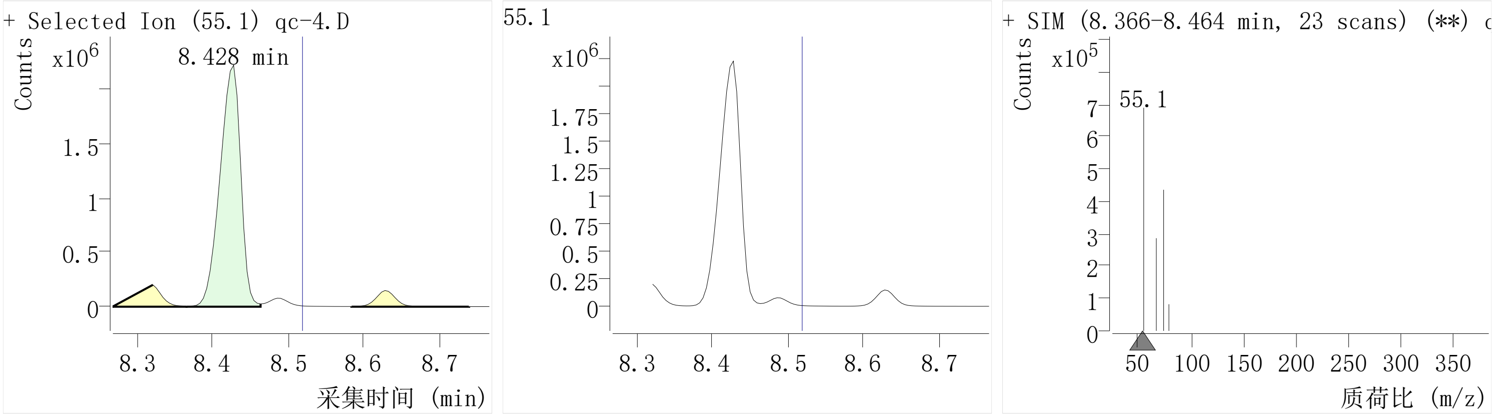

C18:2n6t

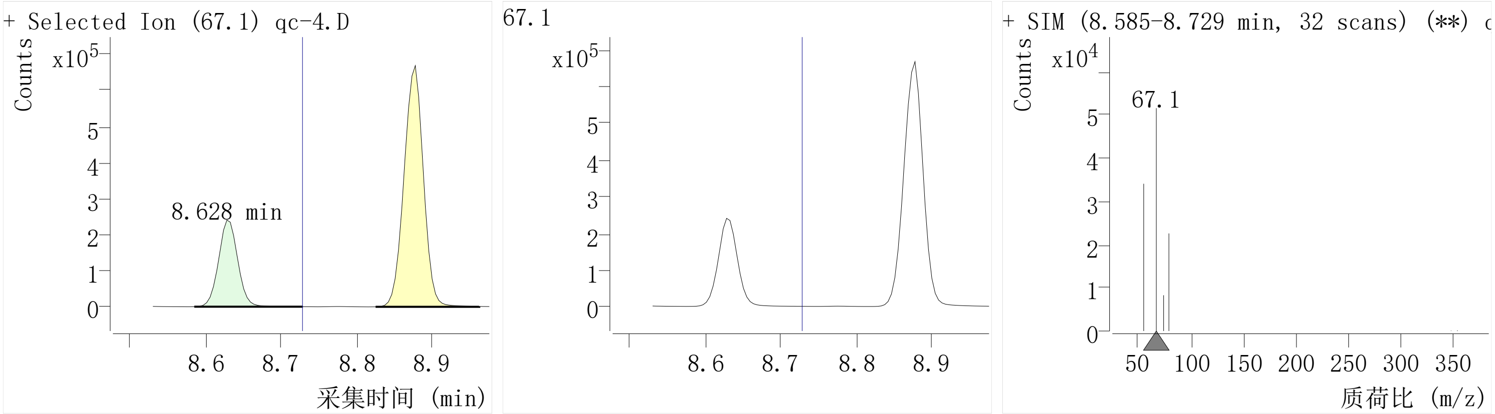

C18:2n6c

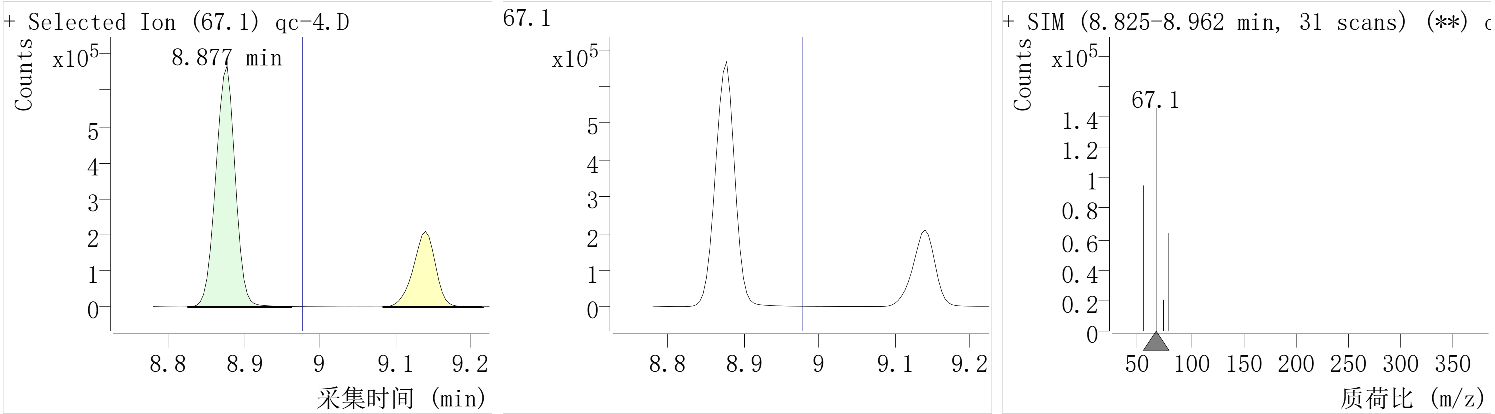

C18:3n6

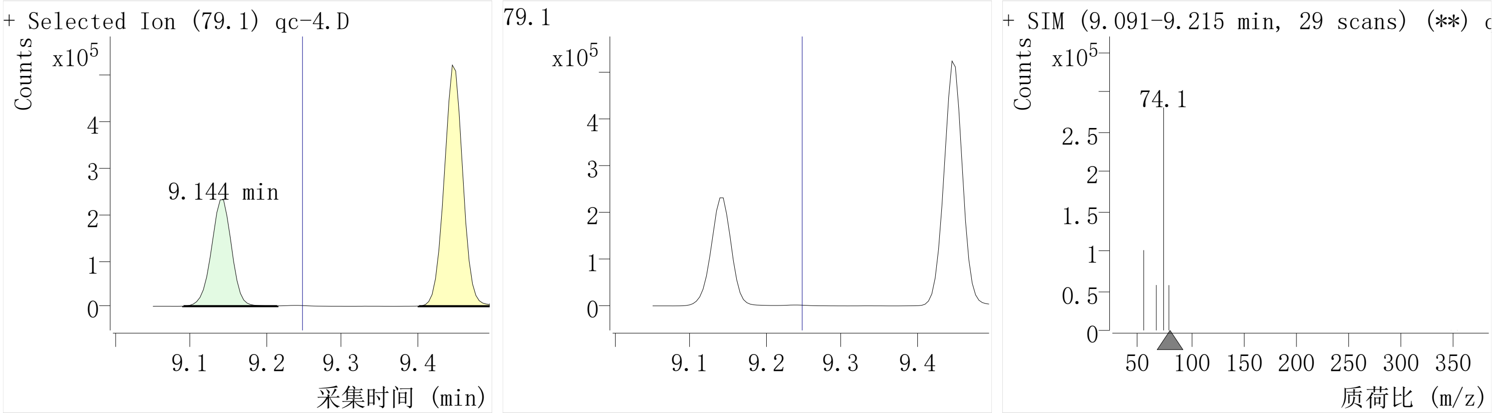

C18:3n3

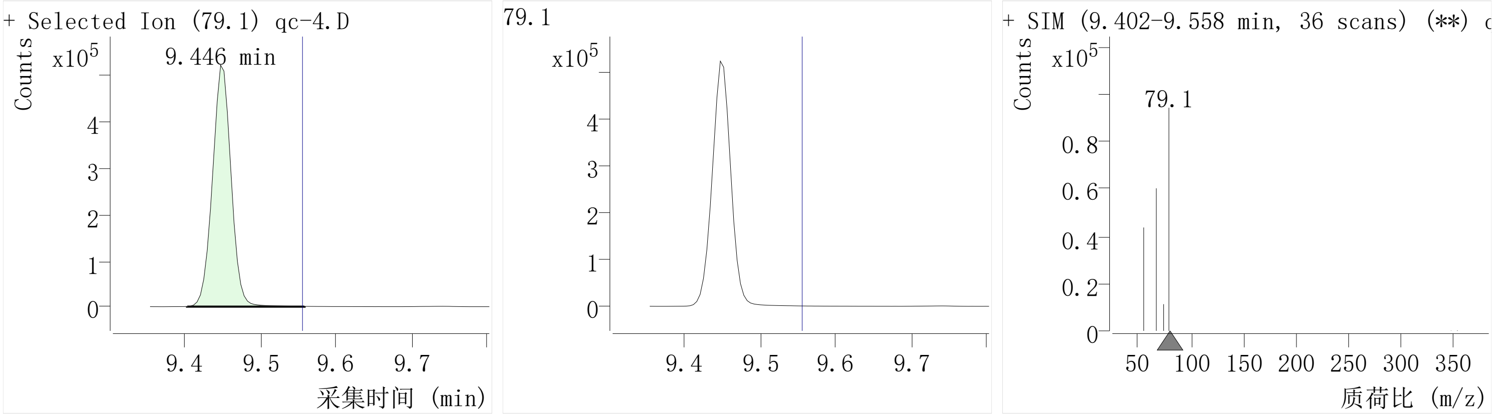

C20:0

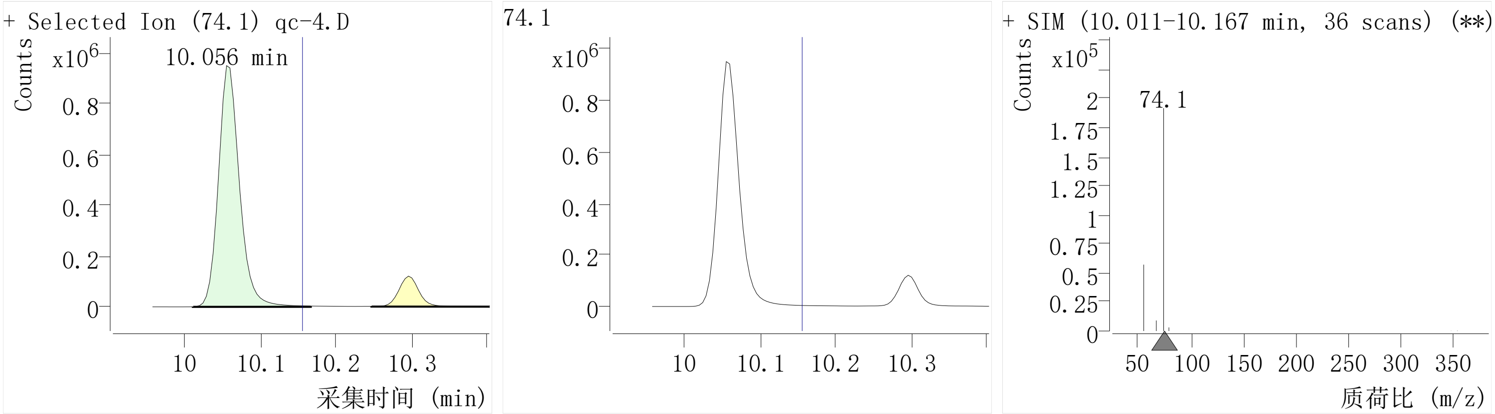

C20:1

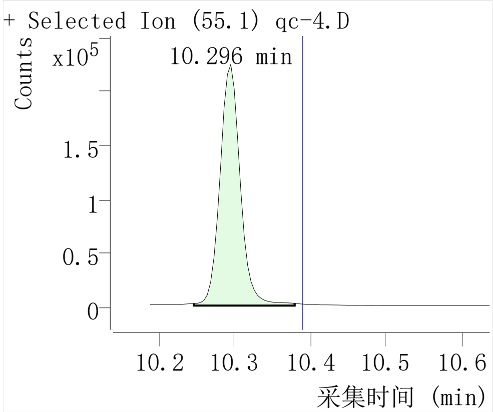

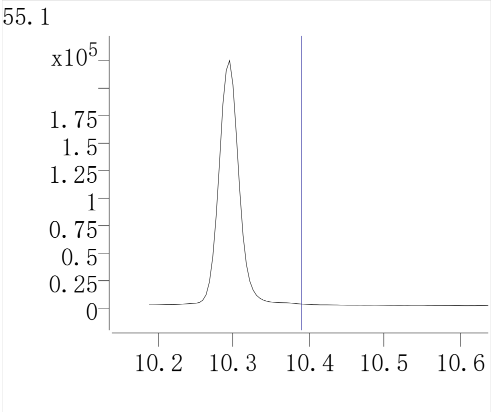

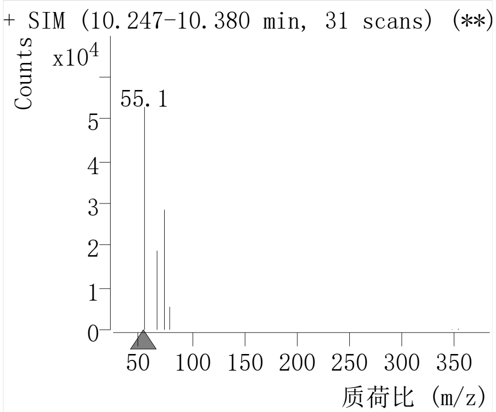

C20:2

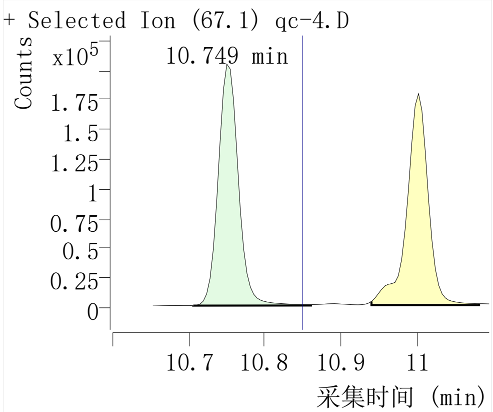

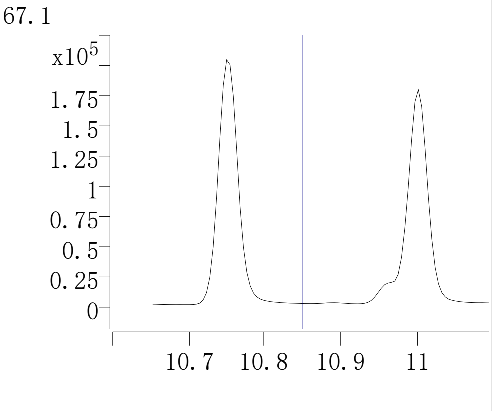

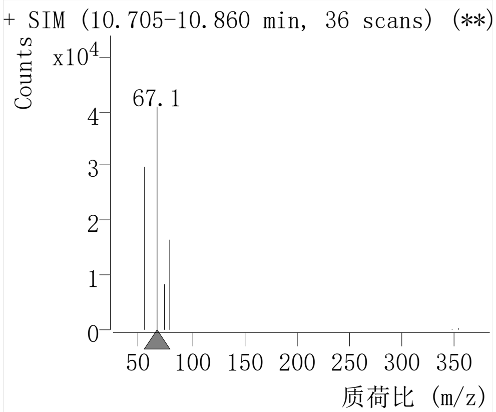

C21:0

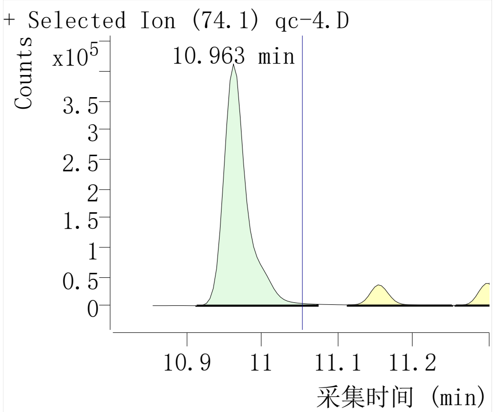

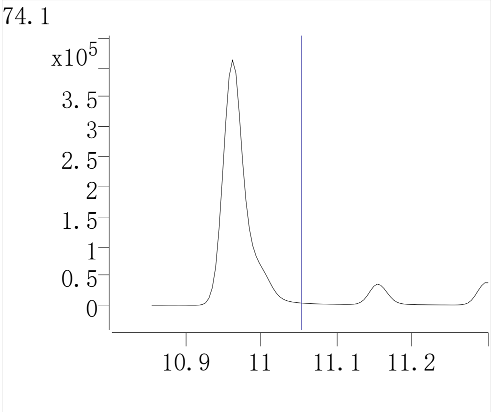

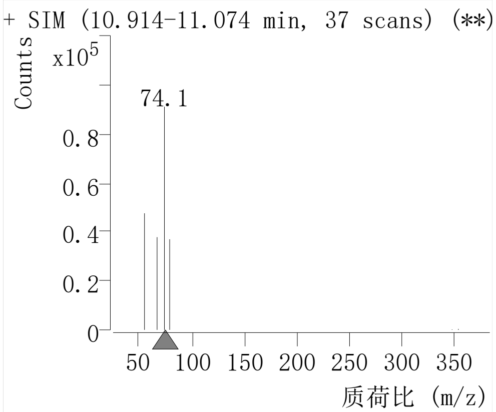

C20:3n6

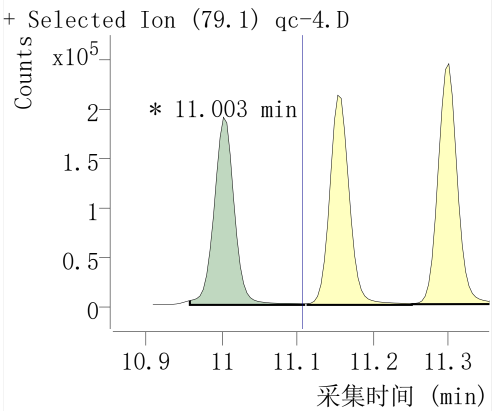

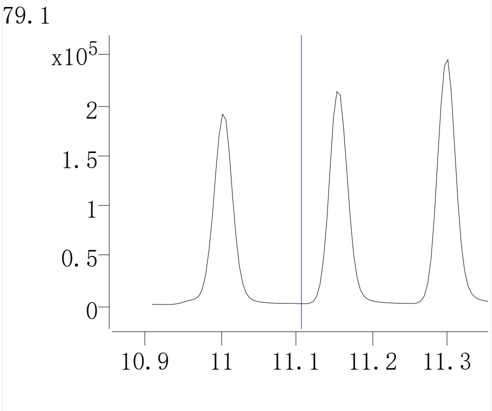

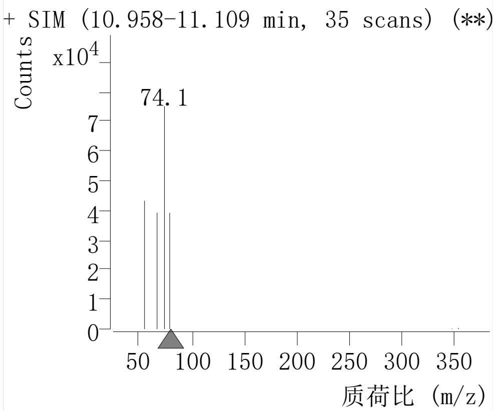

C20:4n6

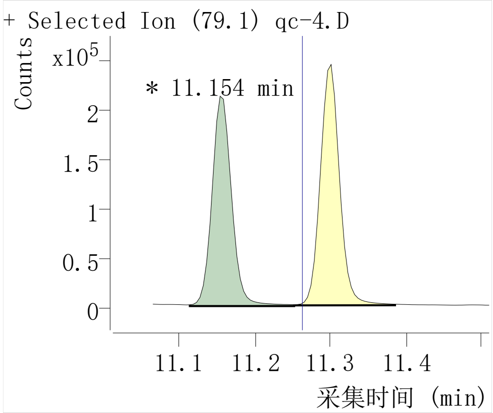

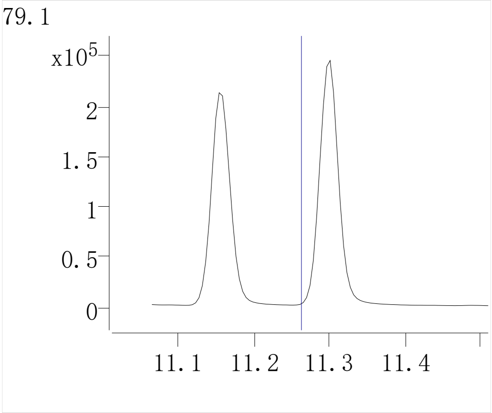

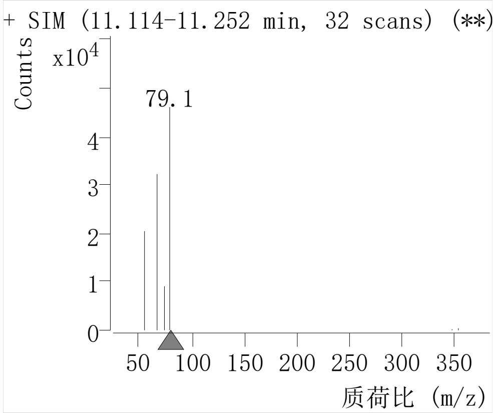

C20:3n3

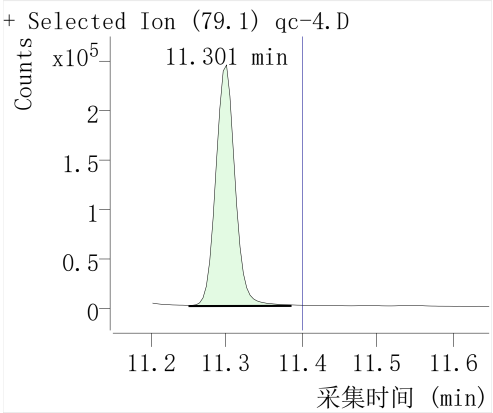

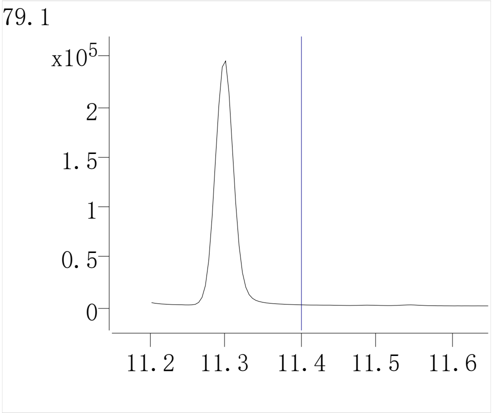

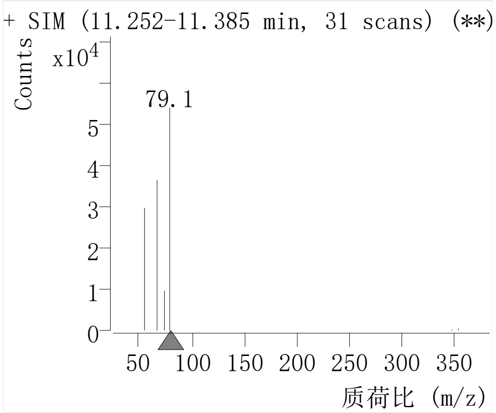

C20:5n3

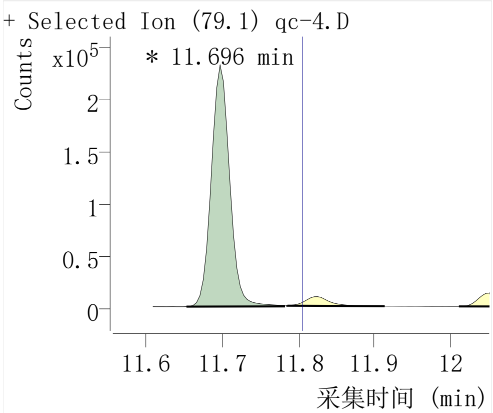

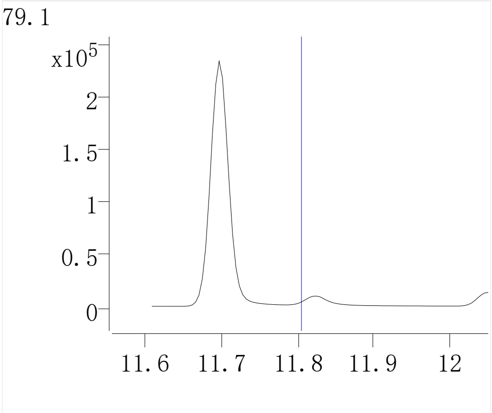

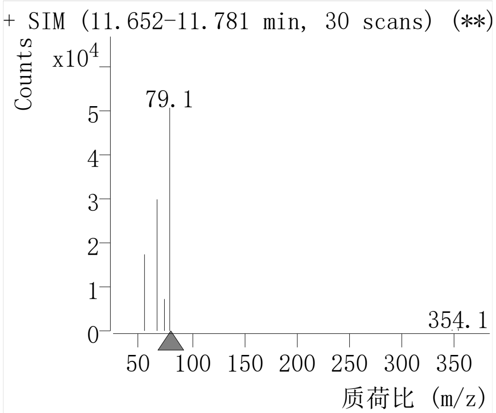

C22:0

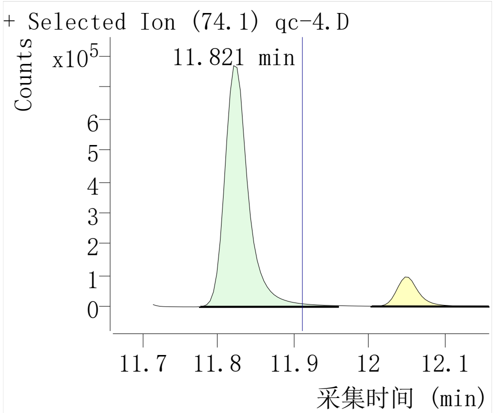

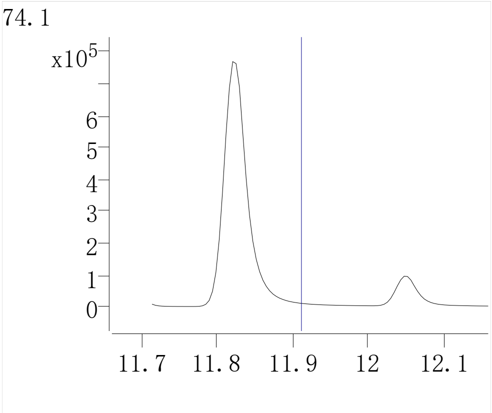

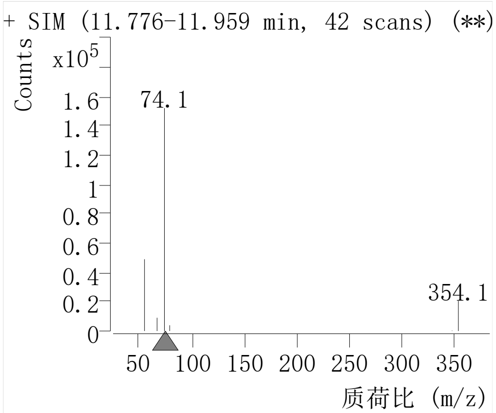

C22:1n9

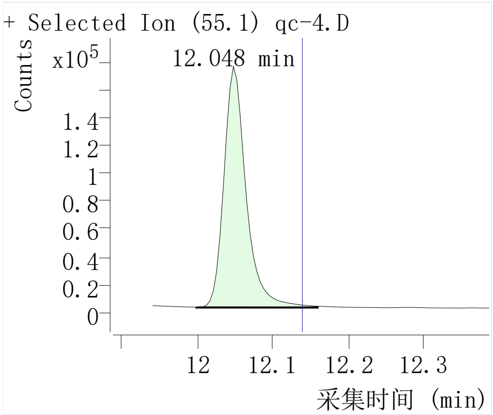

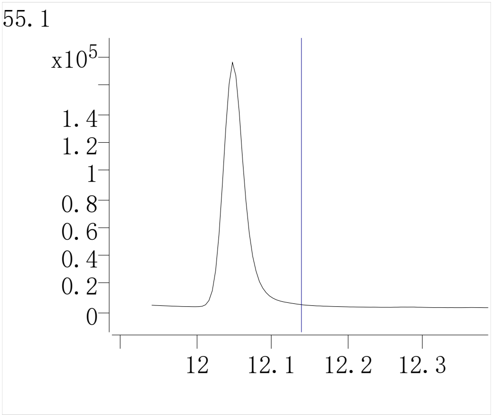

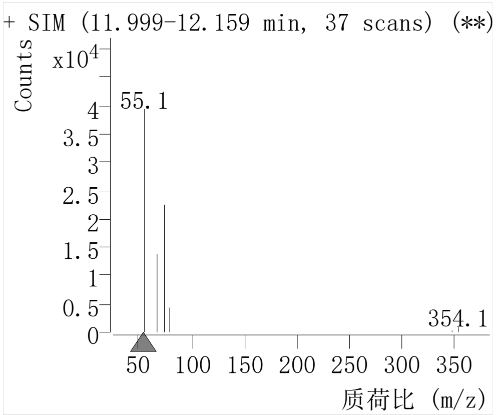

C22:2n6

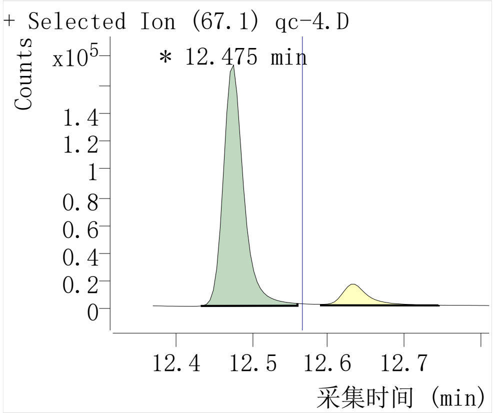

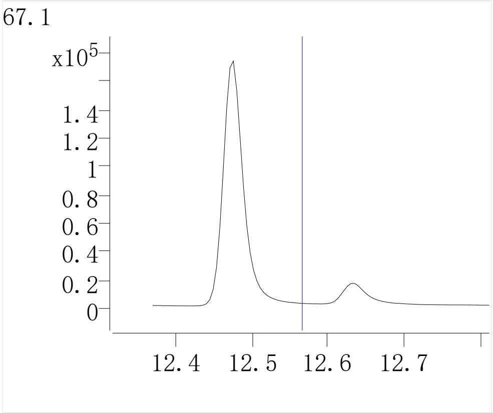

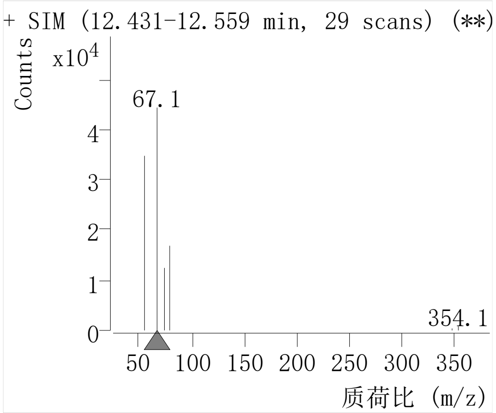

C23:0

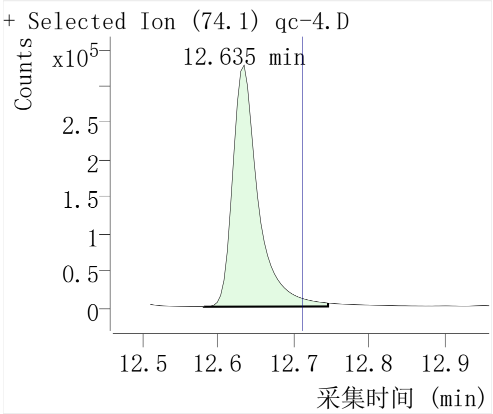

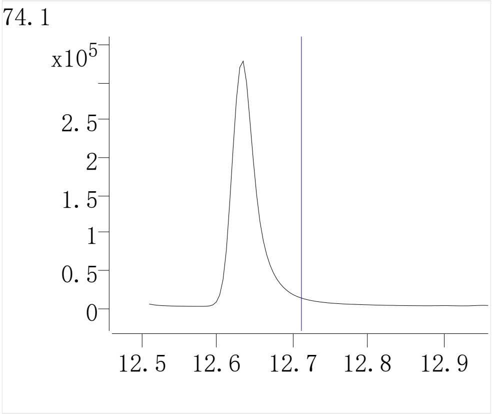

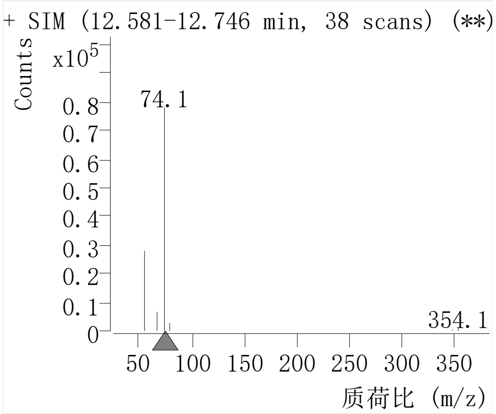

C24:0

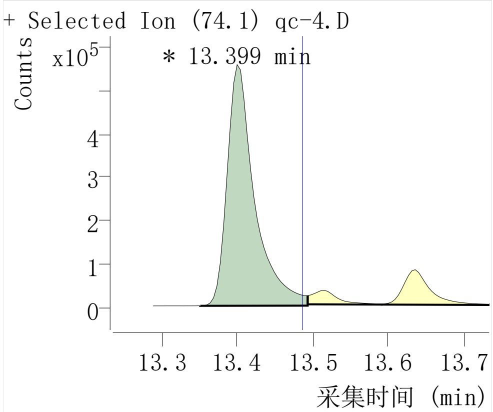

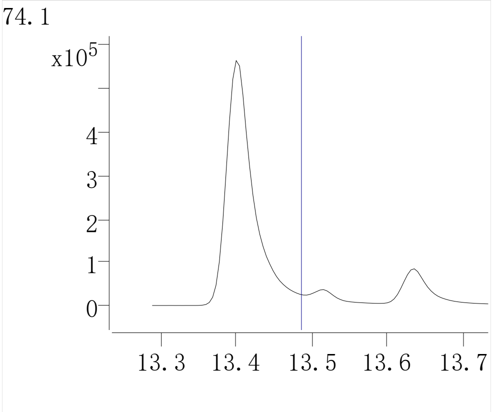

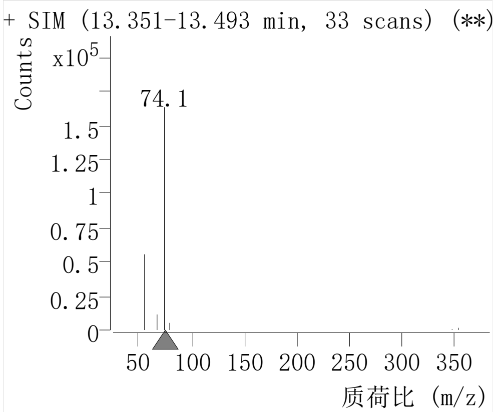

C22:6

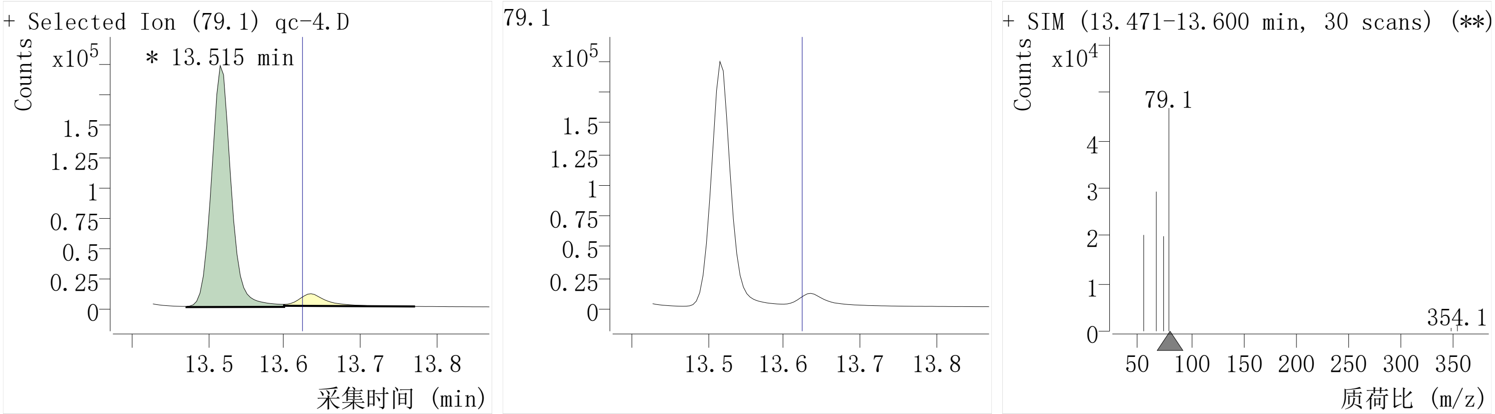
+ Selected Ion (79.1) qc-4.D
Counts
x10^5
* 13.515 min
1.5
1.25
1
0.75
0.5
0.25
0
13.5 13.6 13.7 13.8
采集时间 (min)
79.1
x10^5
1.5
1.25
1
0.75
0.5
0.25
0
13.5 13.6 13.7 13.8
+ SIM (13.471-13.600 min, 30 scans) (**)
Counts
x10^4
79.1
4
3
2
1
0
354.1
50 100 150 200 250 300 350
质荷比 (m/z)

C24:1

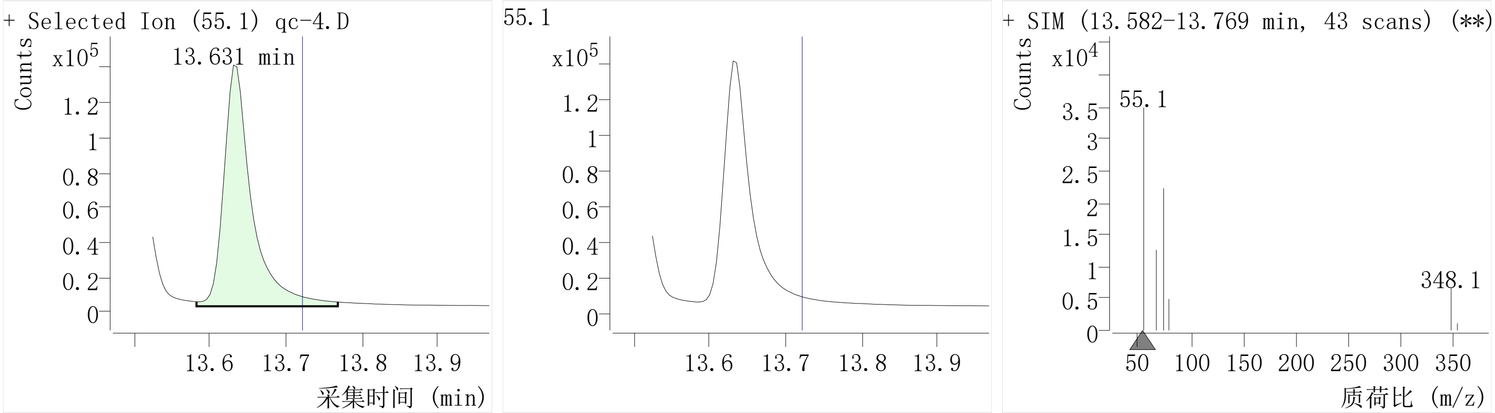
+ Selected Ion (55.1) qc-4.D
Counts
x10^5
13.631 min
1.2
1
0.8
0.6
0.4
0.2
0
13.6 13.7 13.8 13.9
采集时间 (min)
55.1
x10^5
1.2
1
0.8
0.6
0.4
0.2
0
13.6 13.7 13.8 13.9
+ SIM (13.582-13.769 min, 43 scans) (**)
Counts
x10^4
55.1
3.5
3
2.5
2
1.5
1
0.5
0
348.1
50 100 150 200 250 300 350
质荷比 (m/z)

定量分析完成报告

**批处理路径**	G:\GC-MS\HX250430-4-GCMS总脂肪酸靶向检测\HX250430-4\QuantResults\HX250430-4.batch.bin		
**分析时间**	2025/5/14 16:58	**分析员姓名**	DESKTOP-M3A0GPO\omics
**报告时间**	2025/5/16 14:53:28	**报告员姓名**	DESKTOP-M3A0GPO\omics
**最近校正更新**	2025/5/14 16:58	**批处理状态**	已处理
**定量批处理版本**	10.2	**定量报告版本**	10.2

**采集时间**	2025/5/9 6:57	**数据文件**	sp-25.D
**样品类型**	样品	**样品名称**	sp-25
**稀释**	1	**采集方法**	脂肪酸

样品色谱图

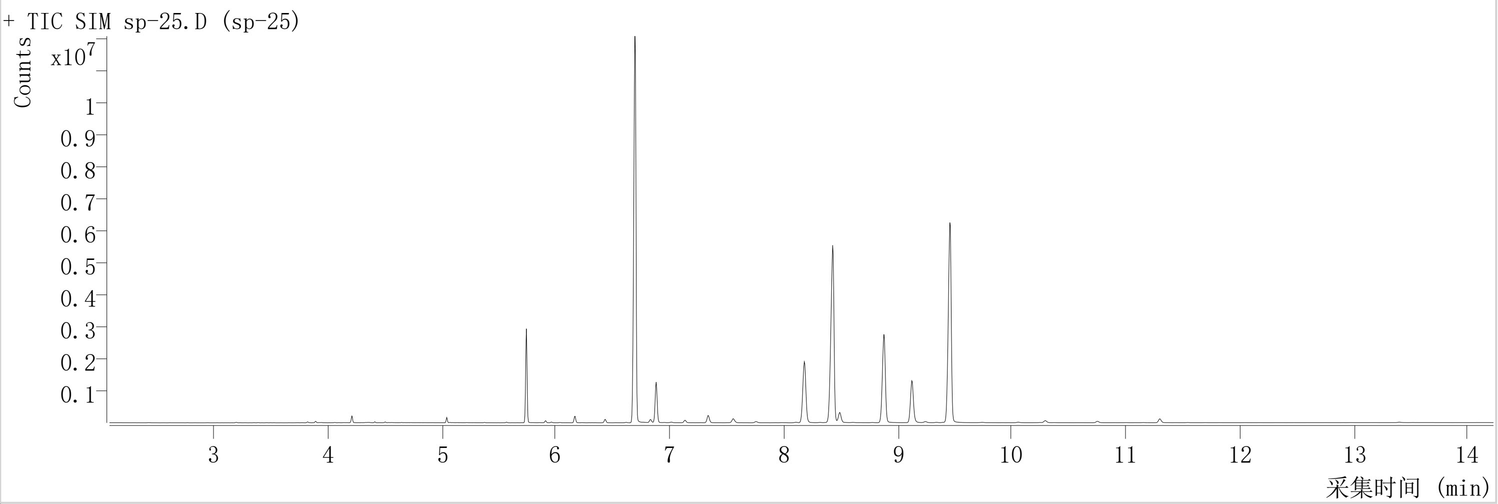

化合物	ISTD	RT	响应	ISTD 响应	响应比	最终浓度	单位
C4:0	C19:0	2.199	122	1815559	0.0001	ND	ug/ml
C6:0	C19:0	2.959	370	1815559	0.0002	0.0018	ug/ml
C8:0	C19:0	3.724	1281	1815559	0.0007	0.0039	ug/ml
C10:0	C19:0	4.413	16557	1815559	0.0091	0.1230	ug/ml
C11:0	C19:0	4.729	1522	1815559	0.0008	0.0071	ug/ml
C12:0	C19:0	5.044	95131	1815559	0.0524	0.8087	ug/ml
C13:0	C19:0	5.374	6896	1815559	0.0038	0.0421	ug/ml
C14:0	C19:0	5.743	2018957	1815559	1.1120	23.5115	ug/ml
C14:1	C19:0	5.912	32613	1815559	0.0180	0.7992	ug/ml
C15:0	C19:0	6.169	167090	1815559	0.0920	1.5936	ug/ml
C15:1	C19:0	6.432	0	1815559	0.0000	ND	ug/ml
C16:0	C19:0	6.699	12357375	1815559	6.8064	253.8346	ug/ml
C16:1	C19:0	6.881	752466	1815559	0.4145	25.2394	ug/ml
C17:0	C19:0	7.339	260771	1815559	0.1436	2.9966	ug/ml
C17:1	C19:0	7.557	98500	1815559	0.0543	2.8931	ug/ml
C18:0	C19:0	8.179	2780468	1815559	1.5315	32.5940	ug/ml
C18:1n9t	C19:0	8.317	6301	1815559	0.0035	0.2047	ug/ml
C18:1n9c	C19:0	8.428	4844008	1815559	2.6681	175.5911	ug/ml
C18:2n6t	C19:0	8.877	0	1815559	0.0000	ND	ug/ml
C18:2n6c	C19:0	8.877	2272941	1815559	1.2519	91.5053	ug/ml
C18:3n6	C19:0	9.126	21077	1815559	0.0116	0.0057	ug/ml
C18:3n3	C19:0	9.460	5202524	1815559	2.8655	142.4627	ug/ml
C20:0	C19:0	10.056	27888	1815559	0.0154	0.4214	ug/ml
C20:1	C19:0	10.292	65156	1815559	0.0359	2.2515	ug/ml
C20:2	C19:0	10.750	36672	1815559	0.0202	1.2891	ug/ml
C21:0	C19:0	10.958	2315	1815559	0.0013	0.0353	ug/ml
C20:3n6	C19:0	10.999	2411	1815559	0.0013	0.1281	ug/ml
C20:4n6	C19:0	11.154	4028	1815559	0.0022	0.1736	ug/ml
C20:3n3	C19:0	11.296	93163	1815559	0.0513	2.7338	ug/ml
C20:5n3	C19:0	11.697	2511	1815559	0.0014	0.1117	ug/ml

化合物	ISTD	RT	响应	ISTD 响应	响应比	最终浓度	单位
C22:0	C19:0	11.821	2392	1815559	0.0013	0.0629	ug/ml
C22:1n9	C19:0	12.043	1475	1815559	0.0008	0.0454	ug/ml
C22:2n6	C19:0	12.470	726	1815559	0.0004	0.0685	ug/ml
C23:0	C19:0	12.755	0	1815559	0.0000	ND	ug/ml
C24:0	C19:0	13.395	2115	1815559	0.0012	0.0589	ug/ml
C22:6	C19:0	13.511	1351	1815559	0.0007	0.0602	ug/ml
C24:1	C19:0	13.640	0	1815559	0.0000	ND	ug/ml

C4:0

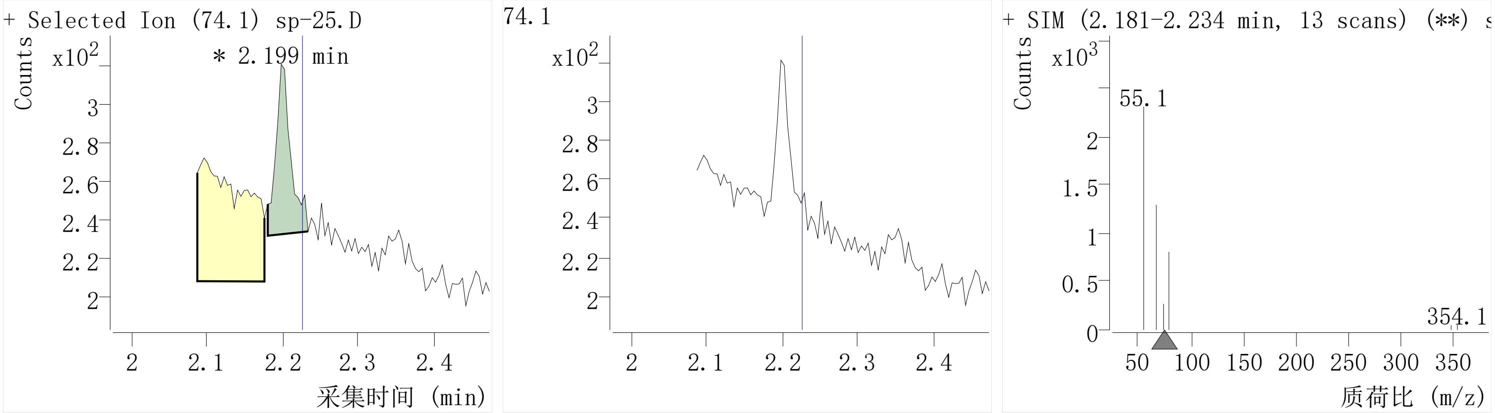

C6:0

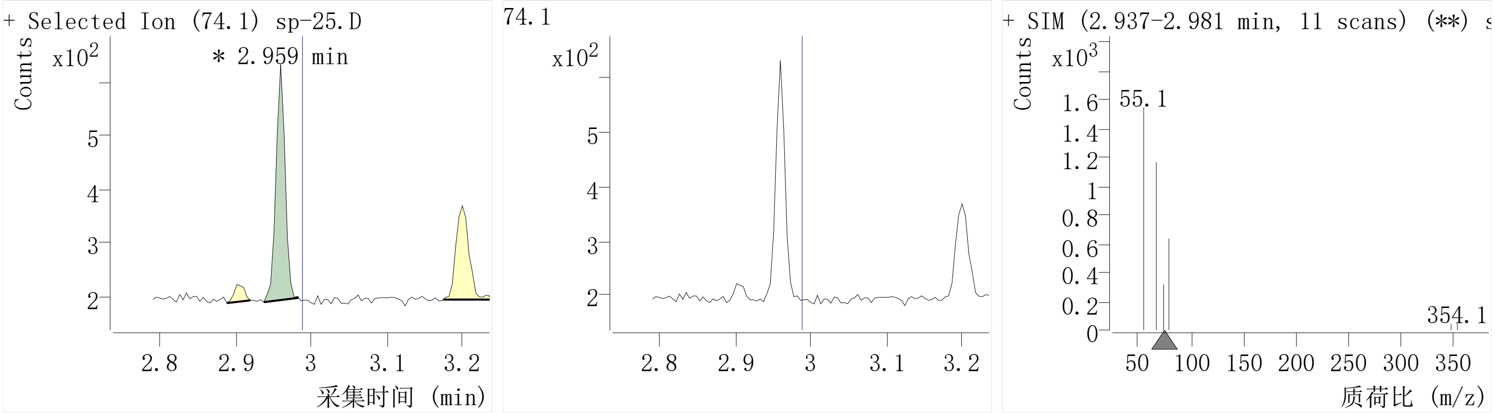

C8:0

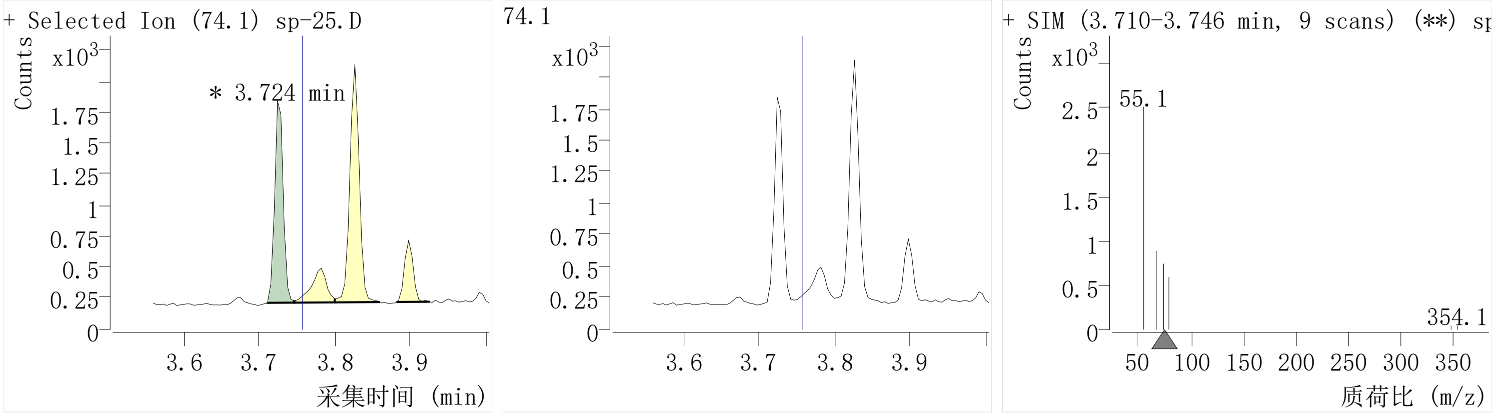

C10:0

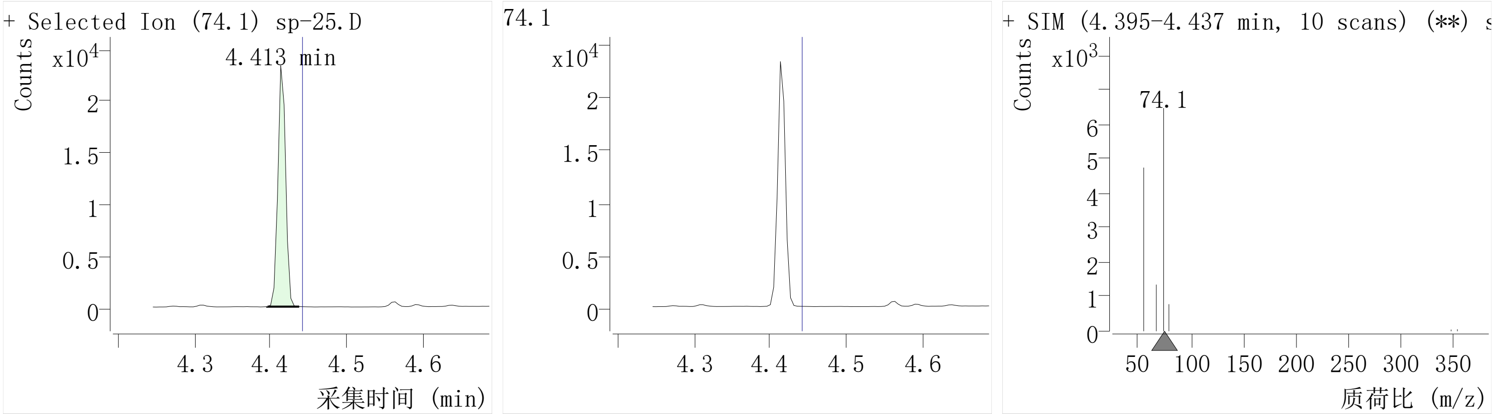

C11:0

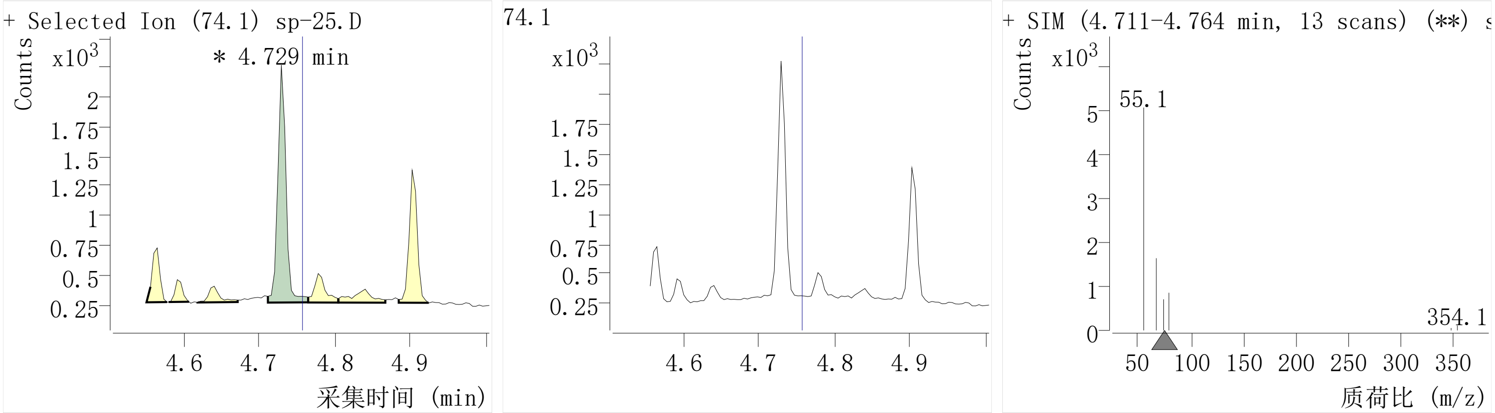

C12:0

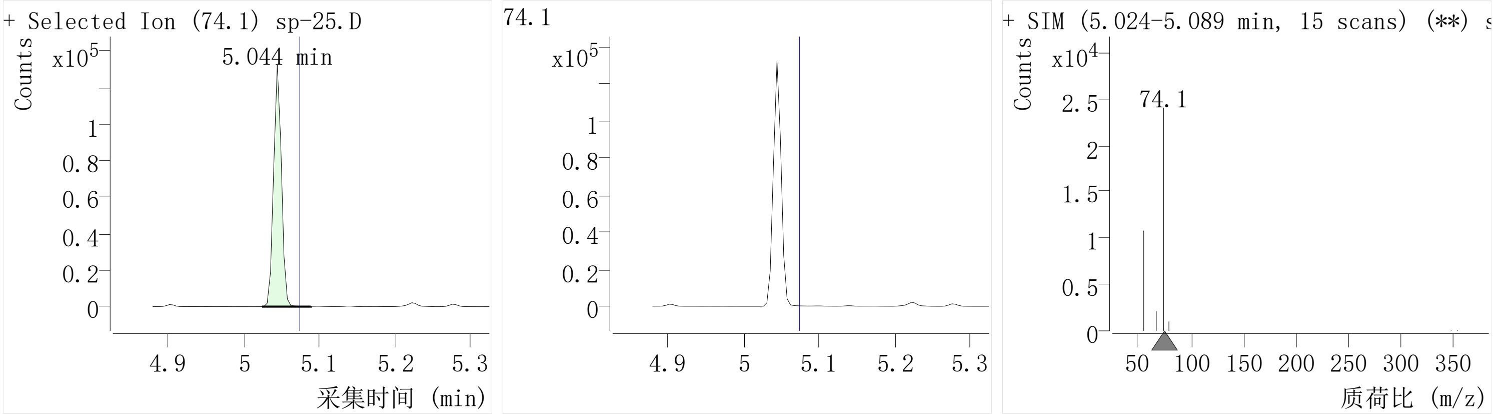

C13:0

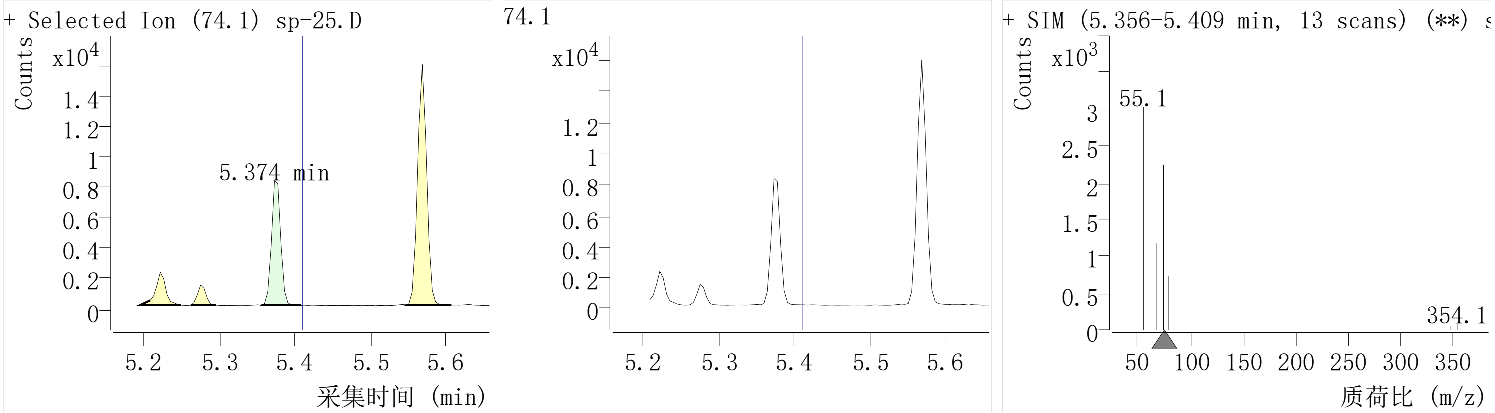

C14:0

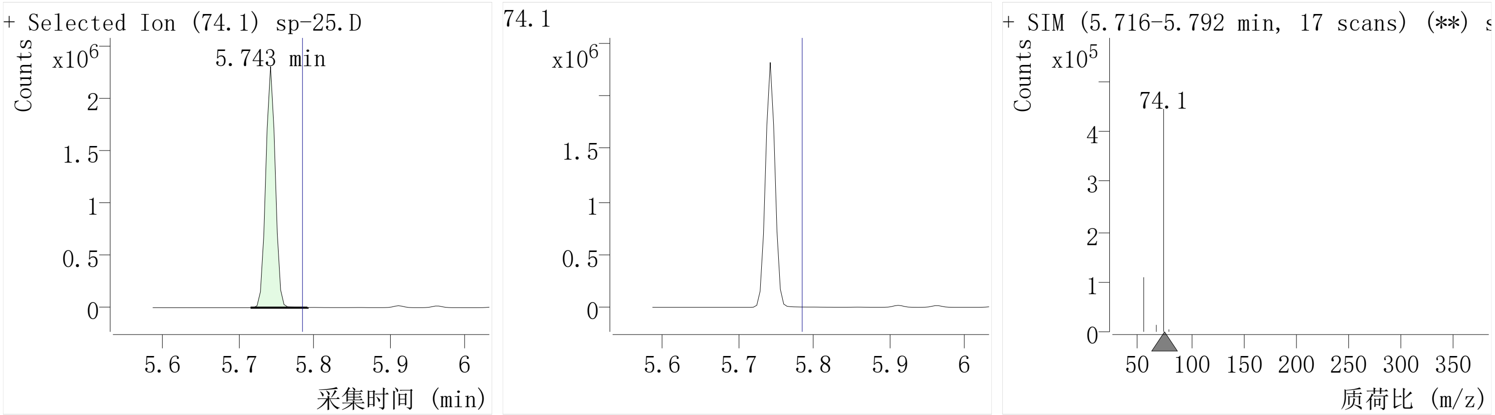

C14:1

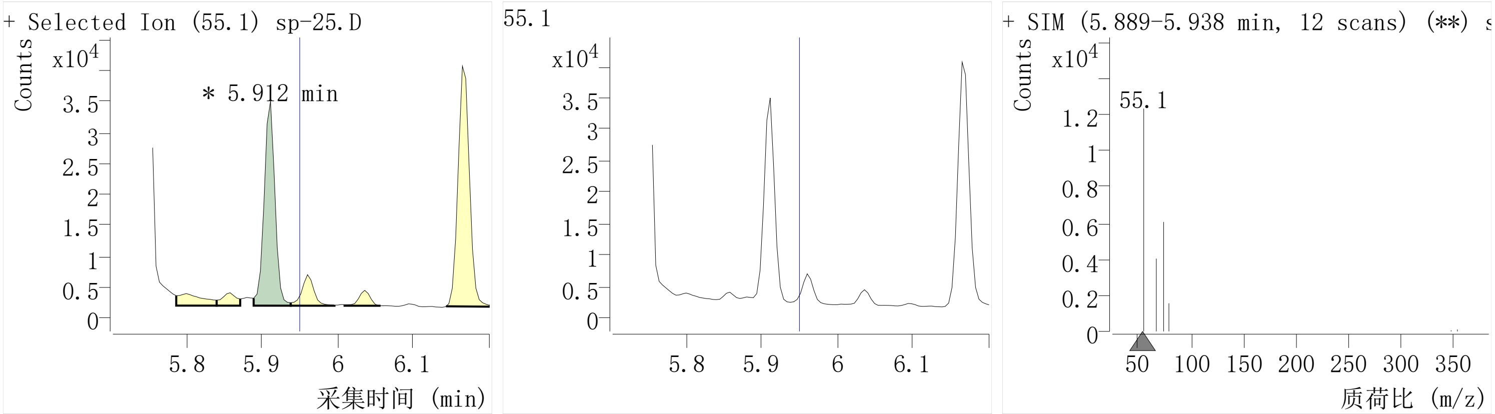

C15:0

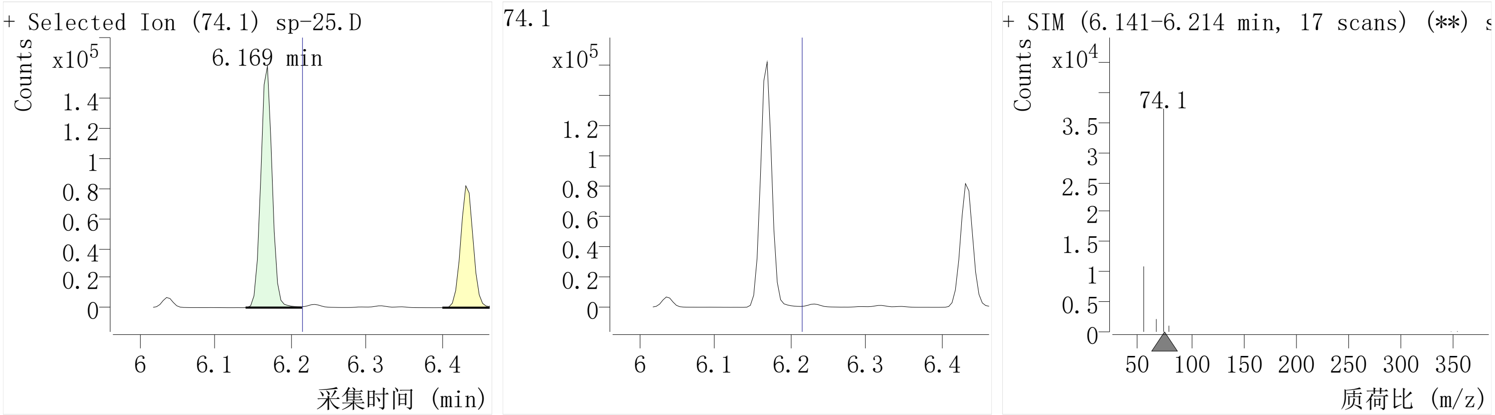

C15:1

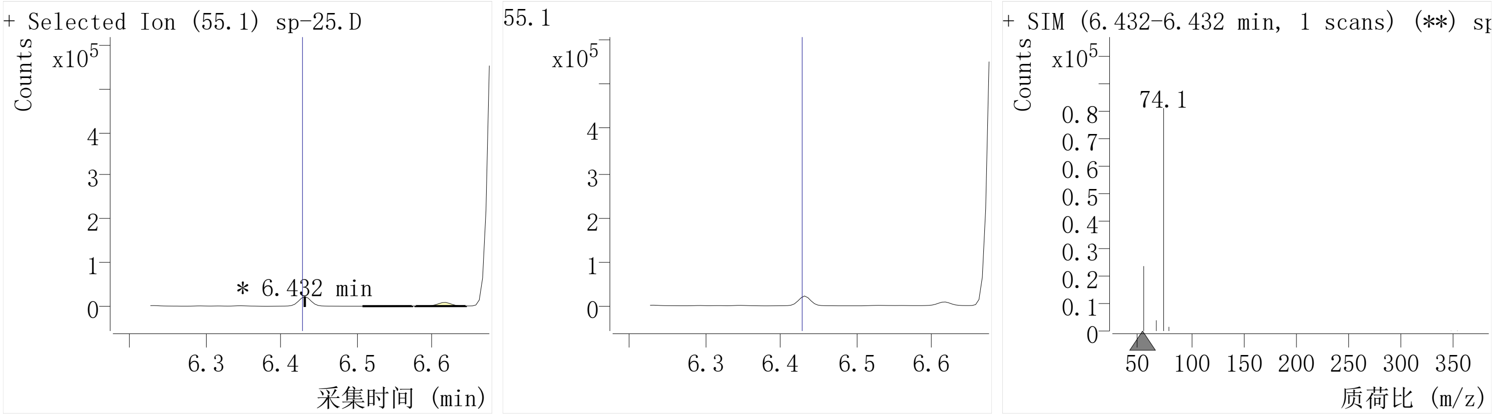

C16:0

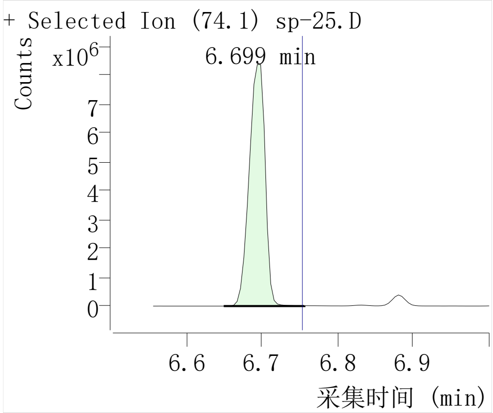

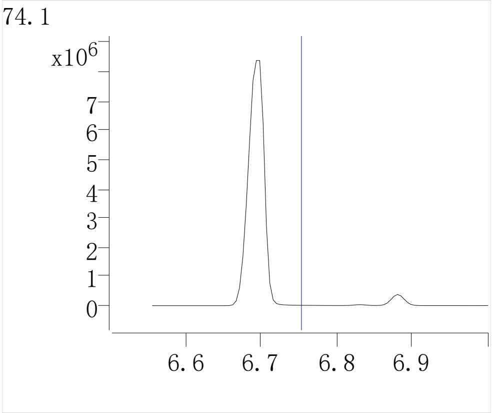

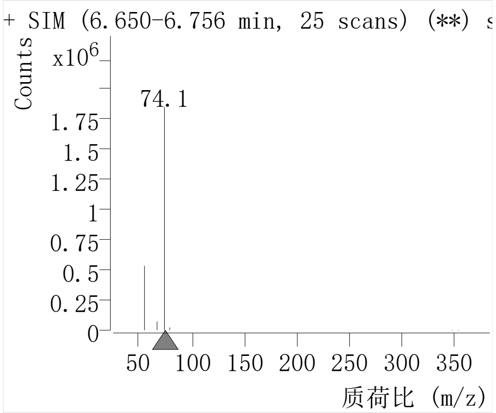

C16:1

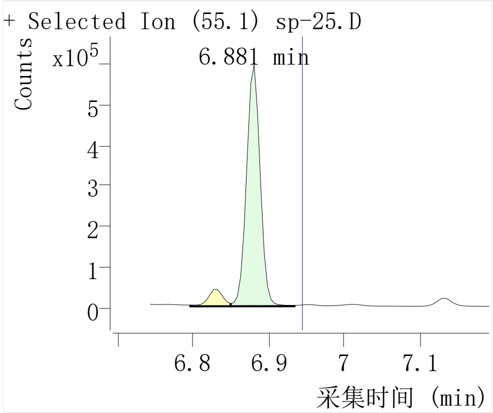

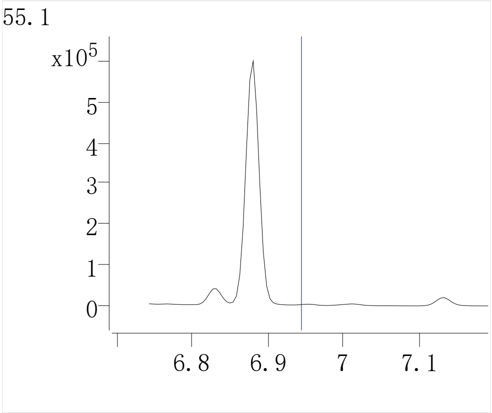

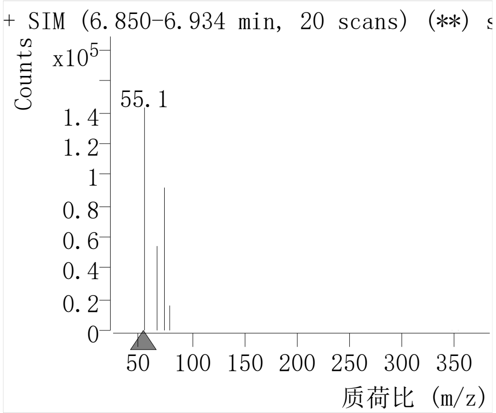

C17:0

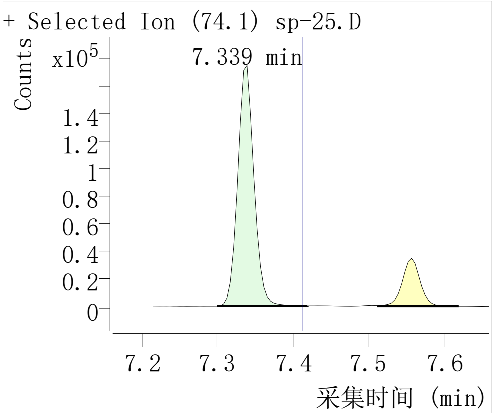

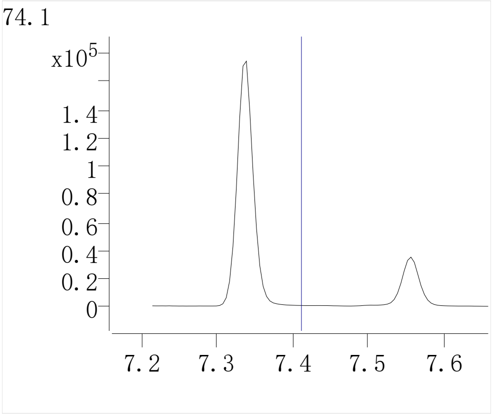

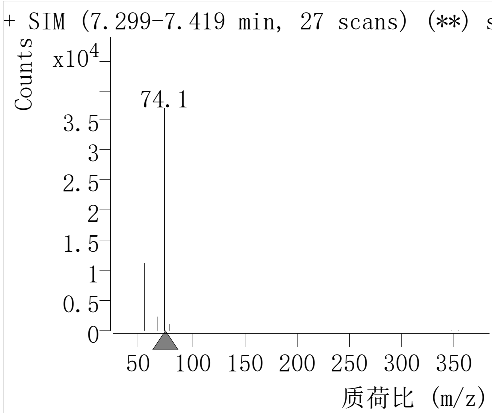

C17:1

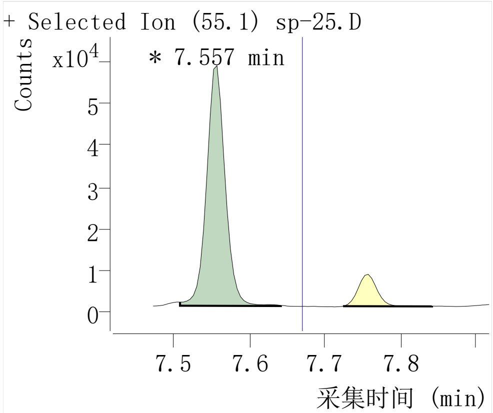

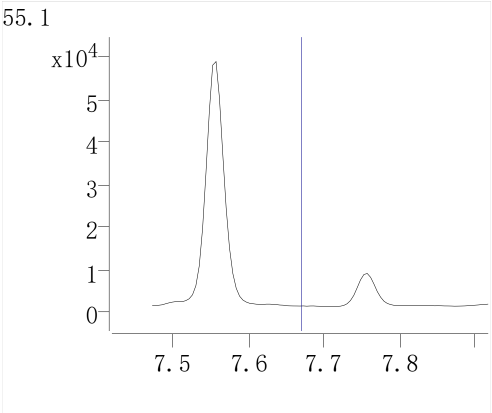

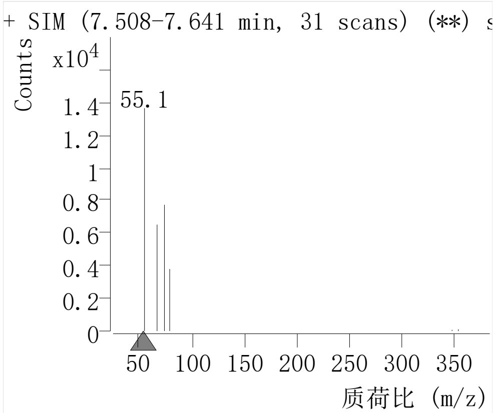

C18:0

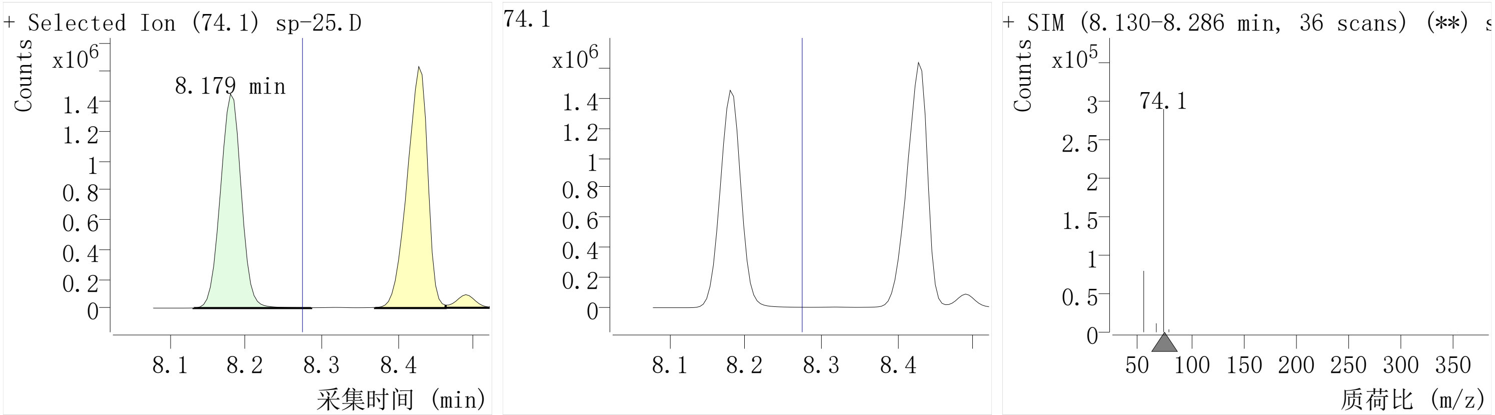

C18:1n9t

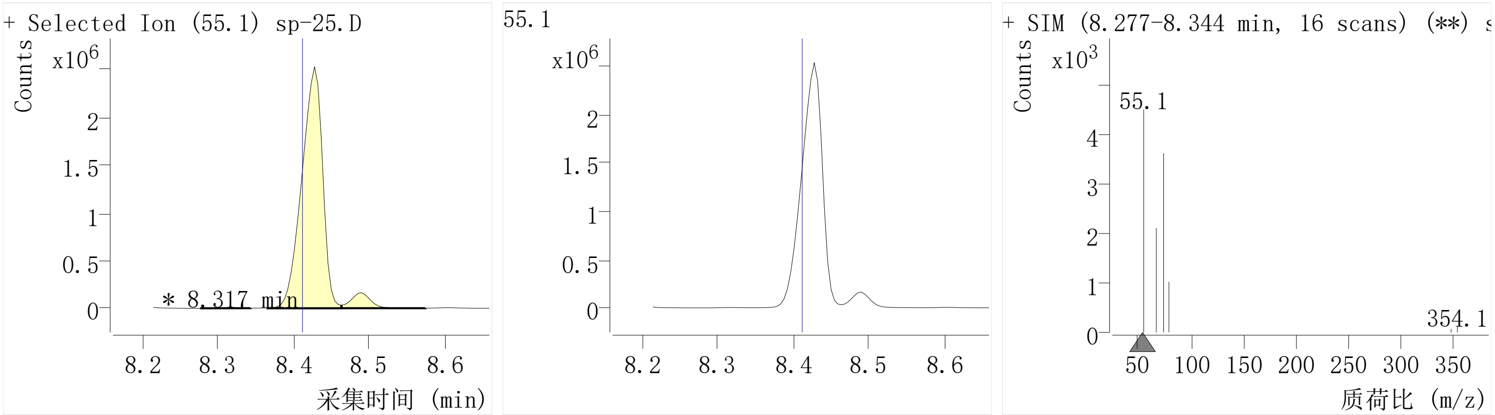

C18:1n9c

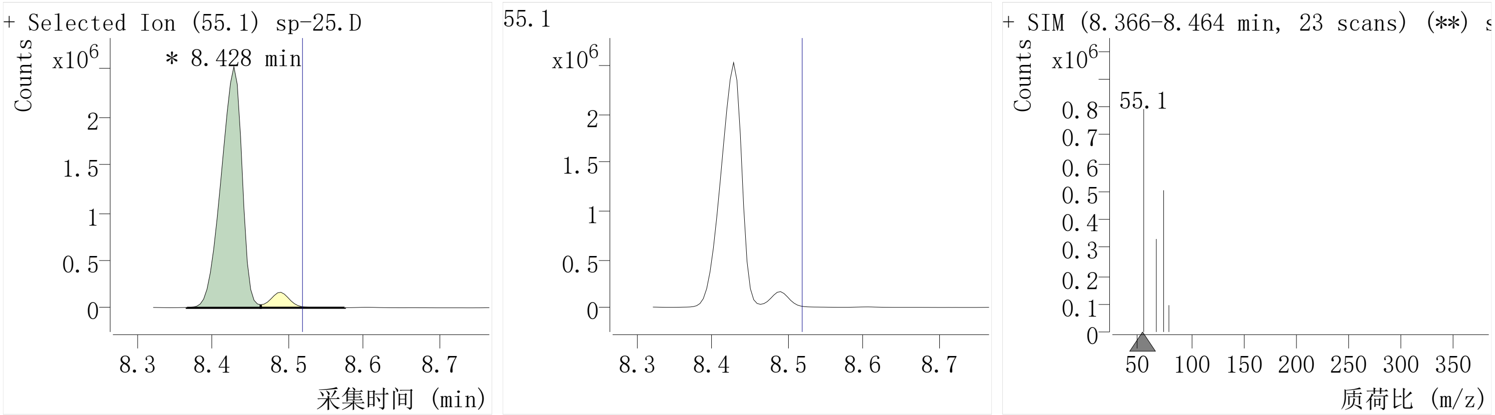

C18:2n6t

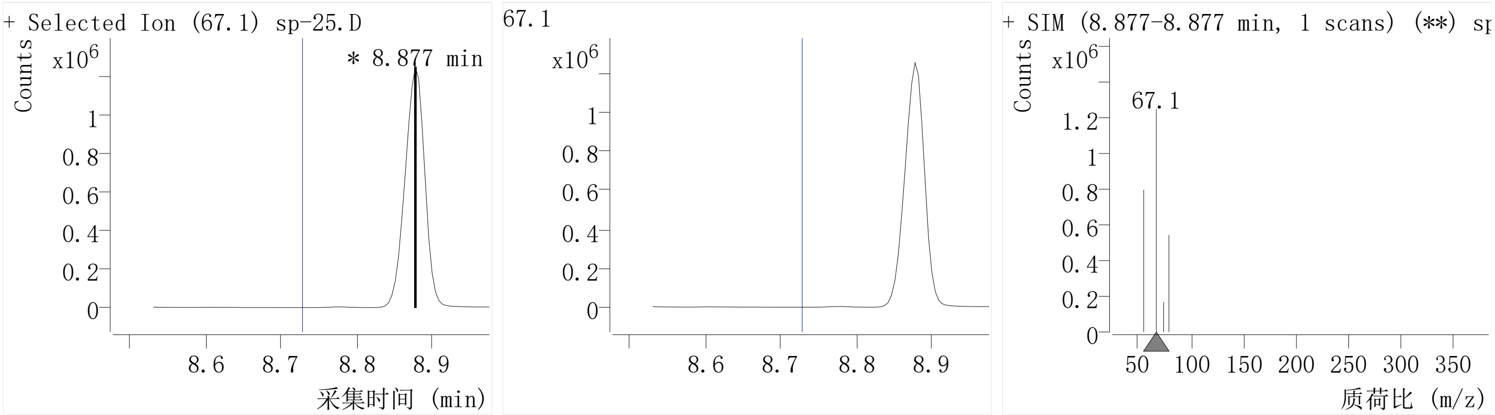

C18:2n6c

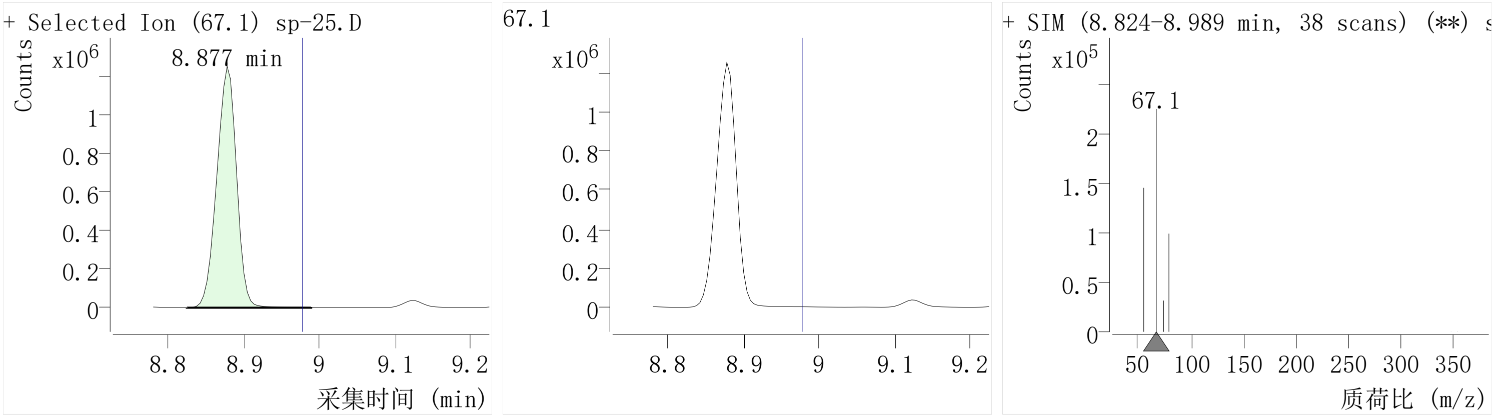

C18:3n6

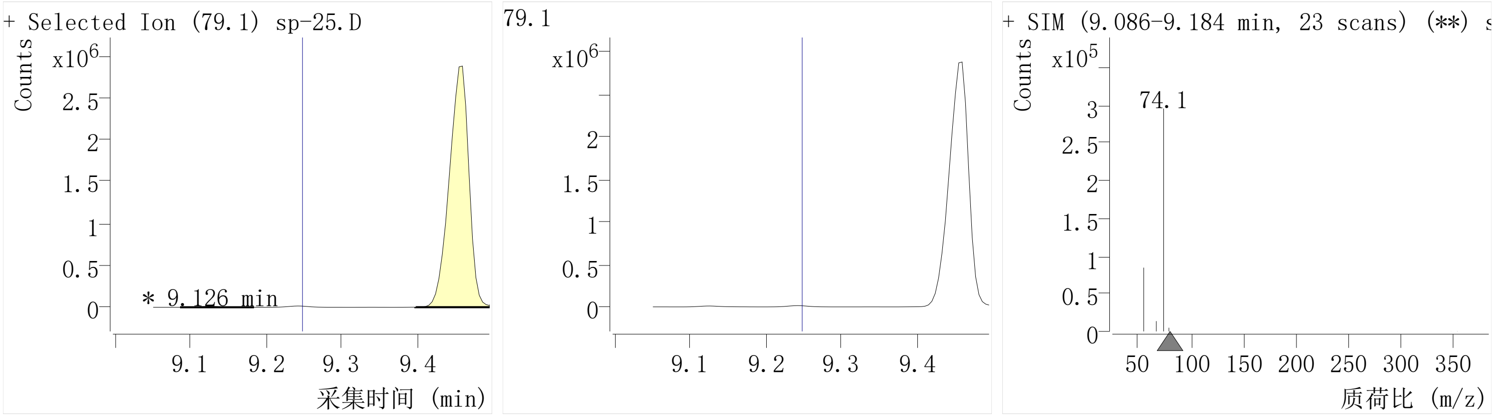

C18:3n3

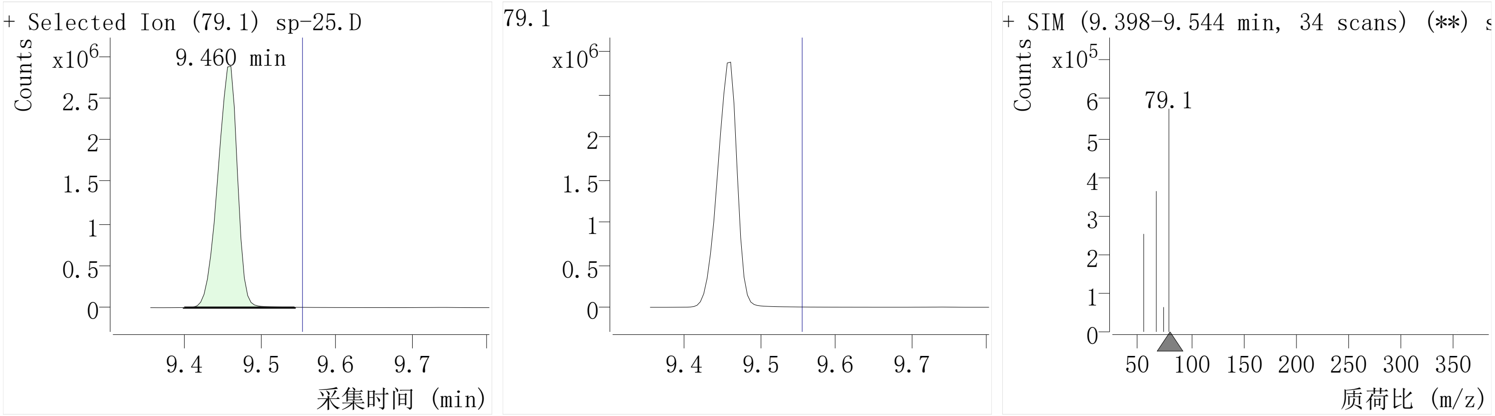

C20:0

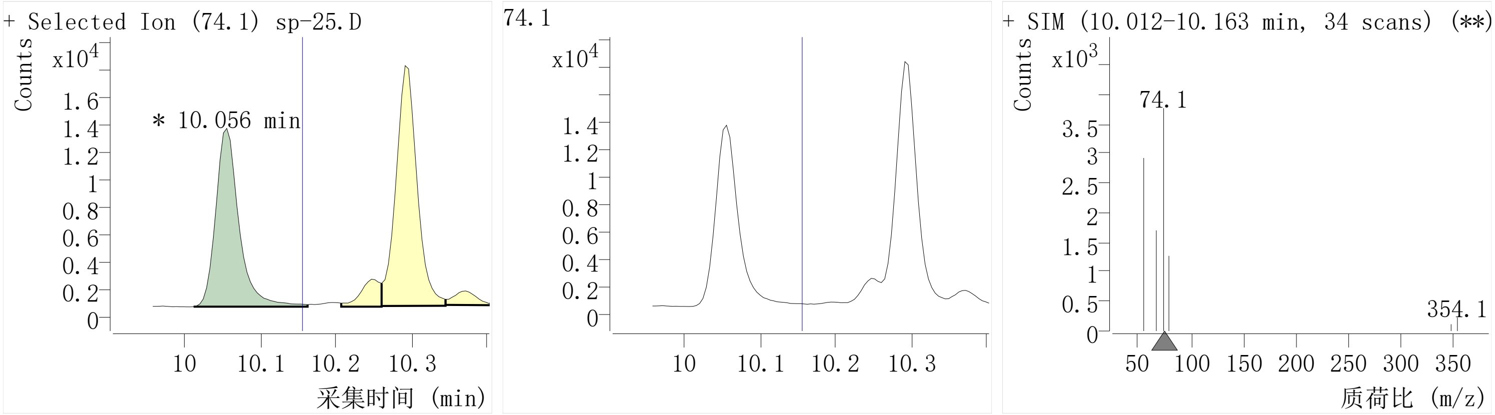

C20:1

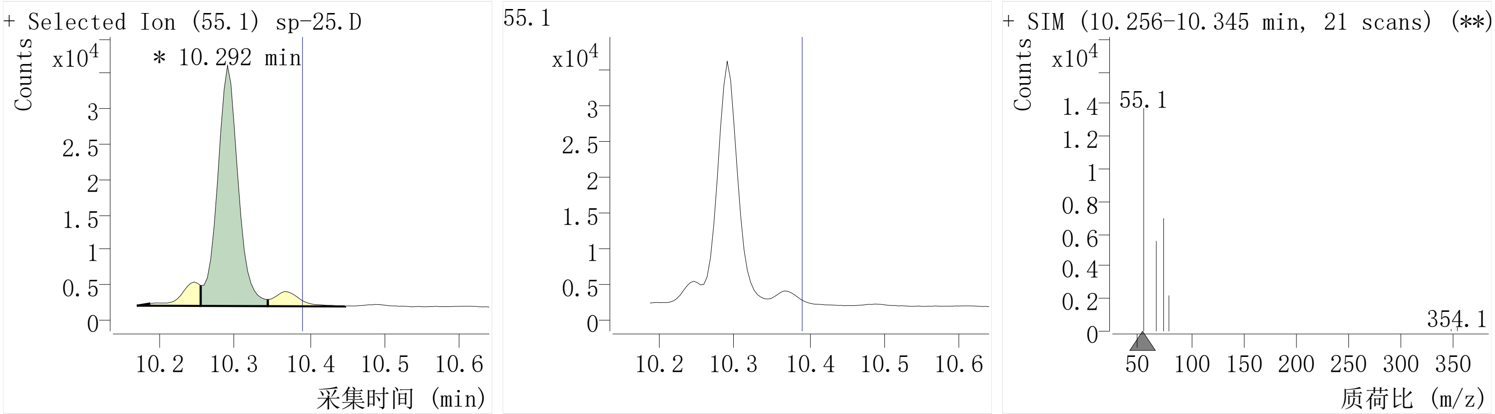

C20:2

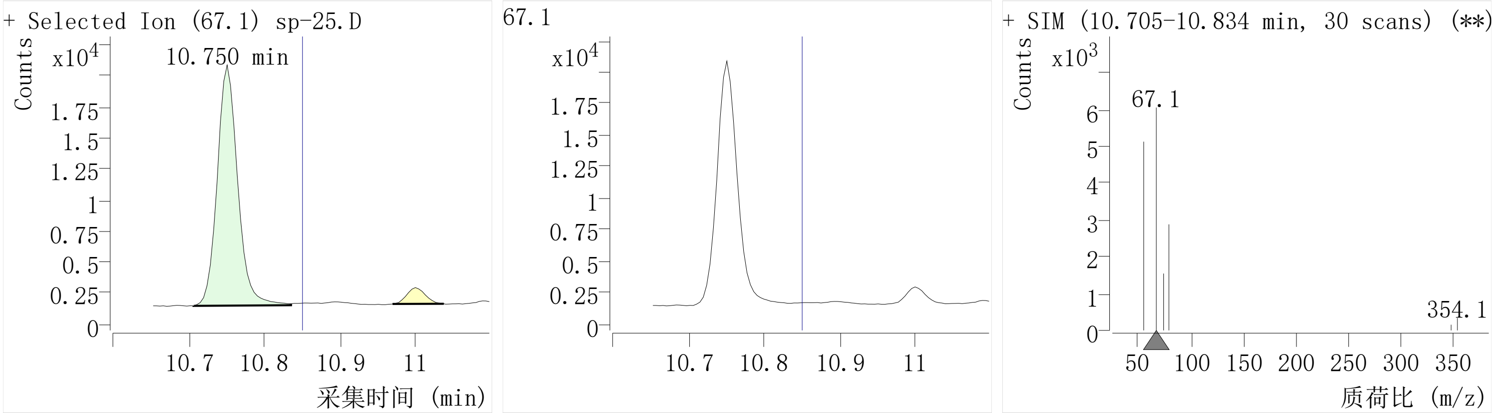

C21:0

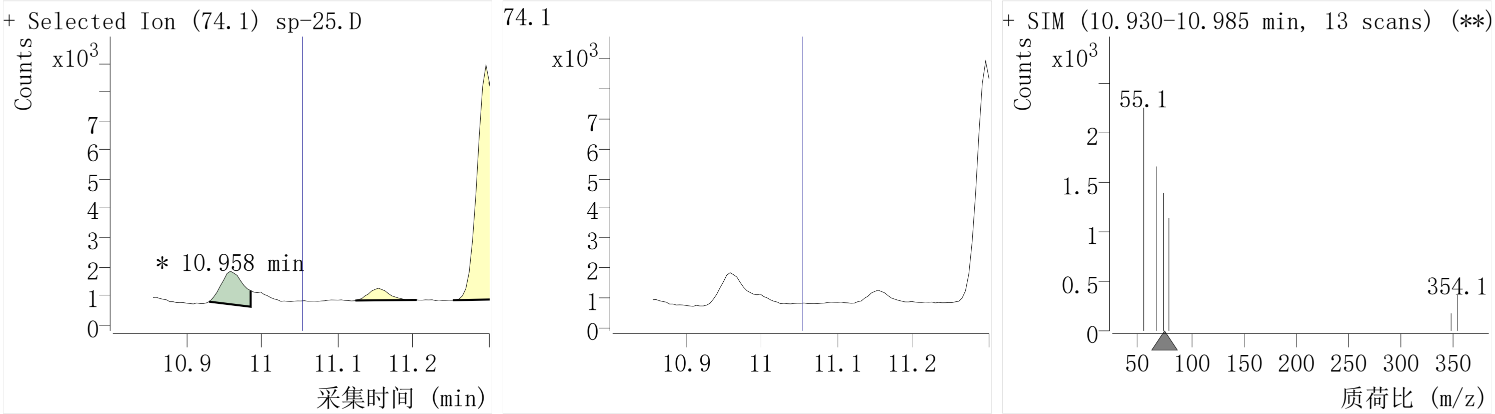

C20:3n6

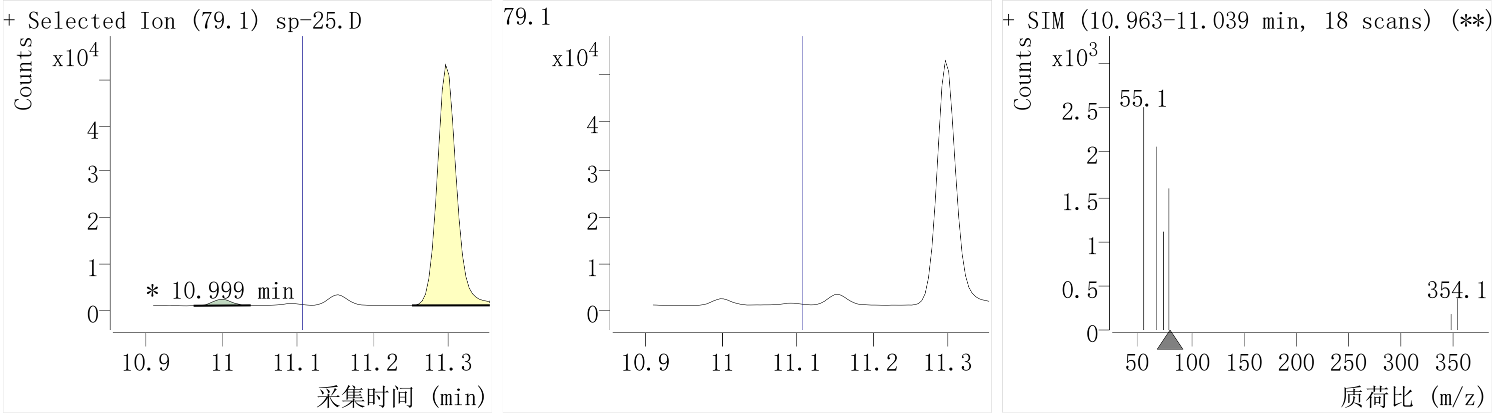

C20:4n6

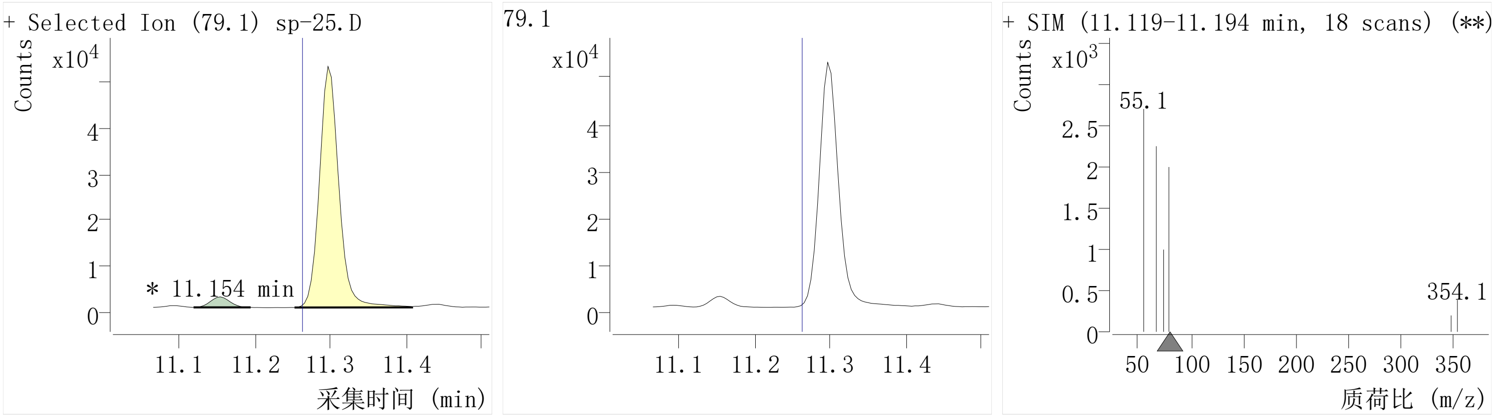

C20:3n3

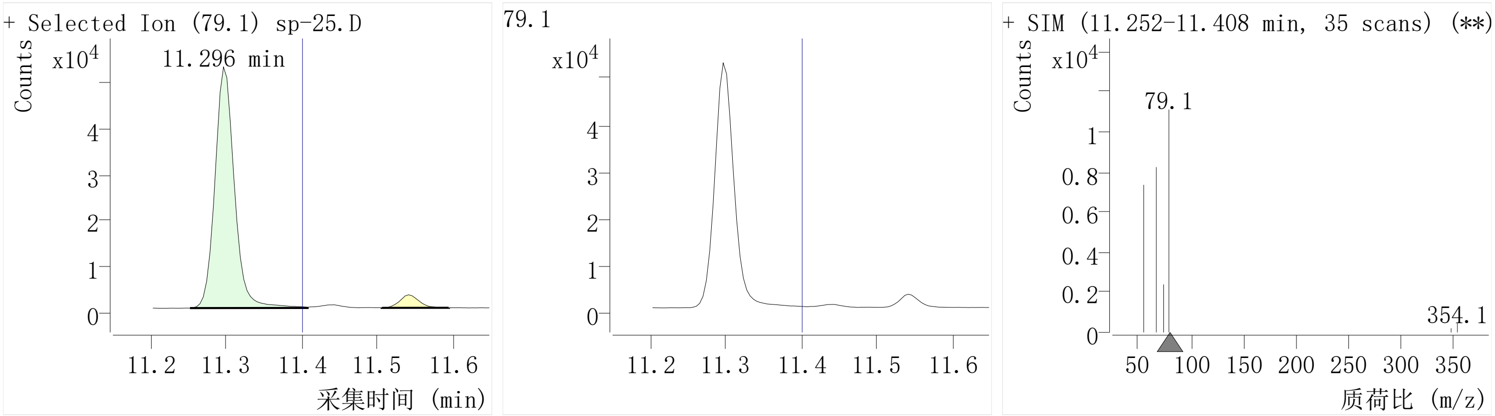

C20:5n3

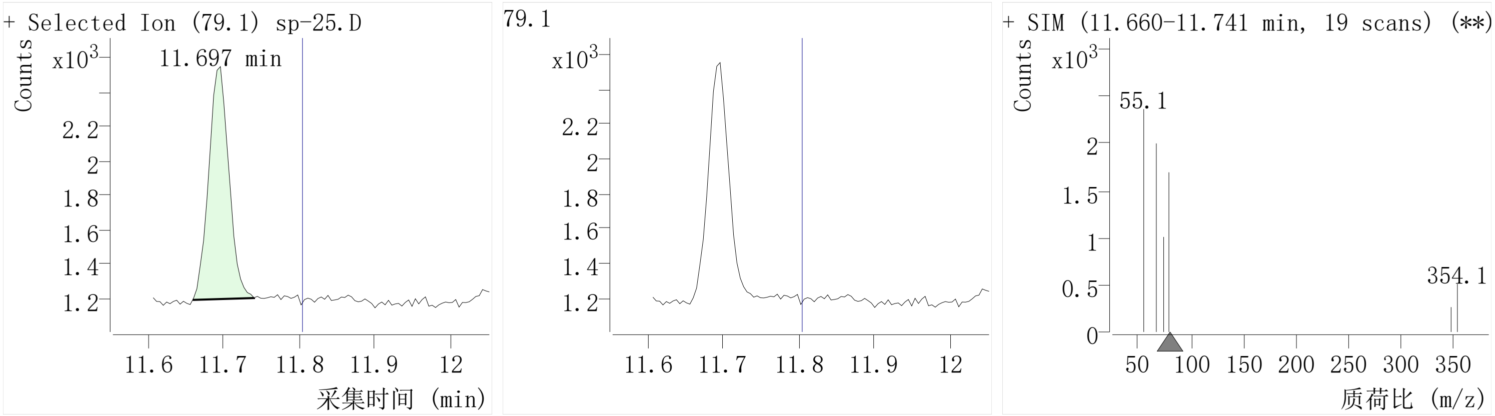

C22:0

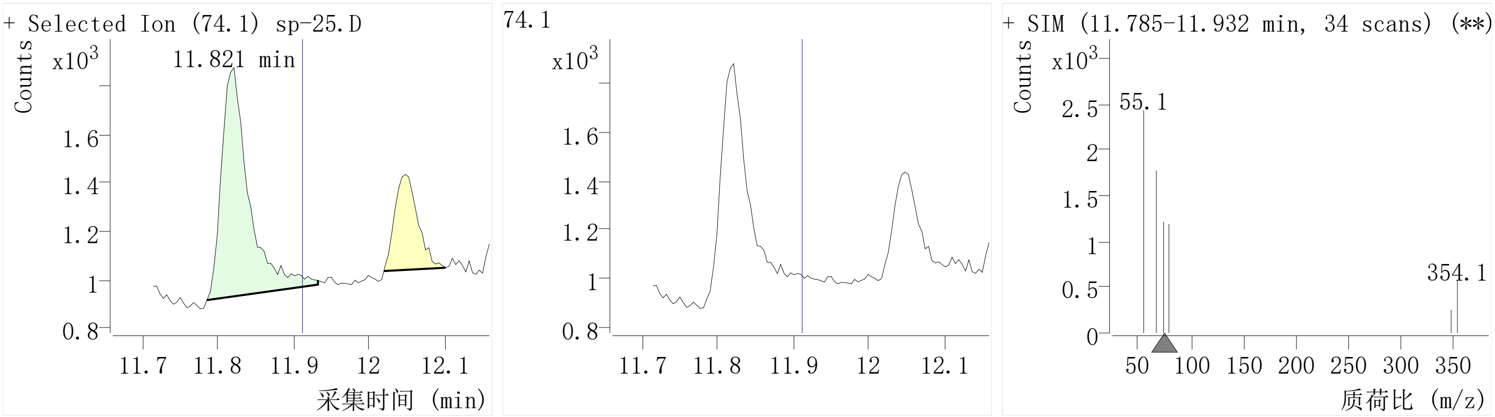

C22:1n9

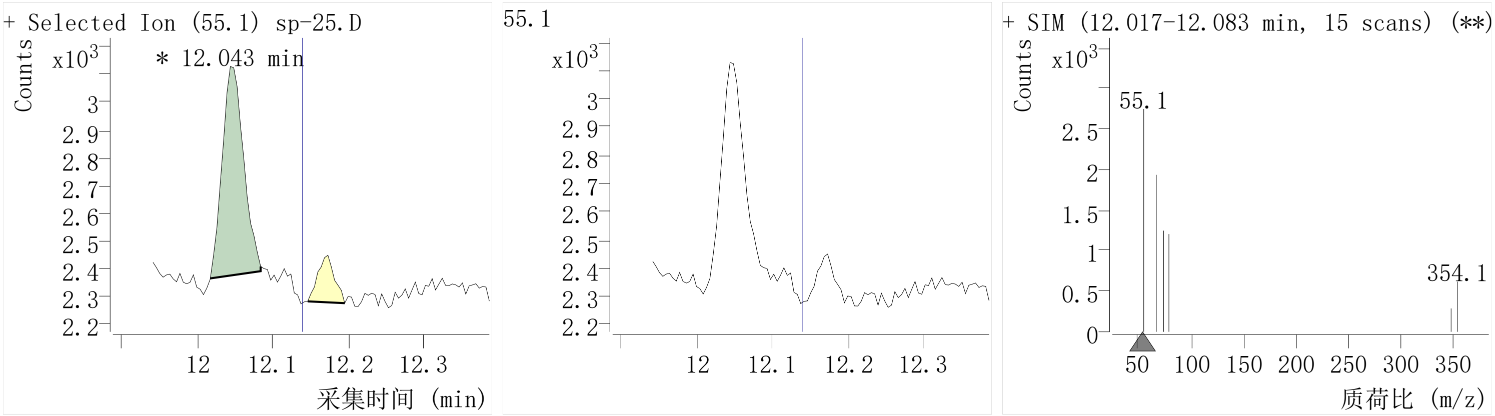

C22:2n6

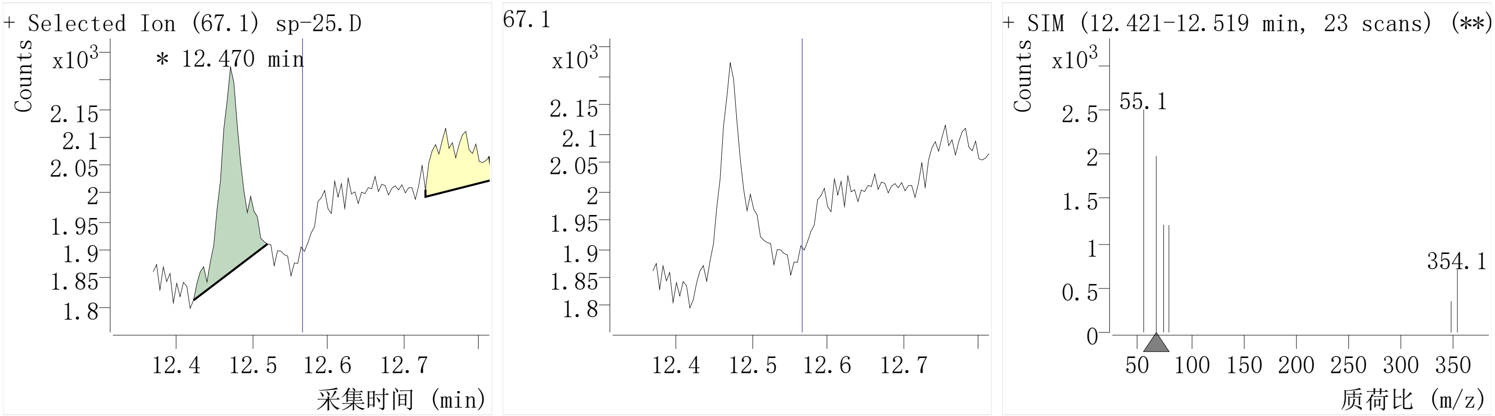

C23:0

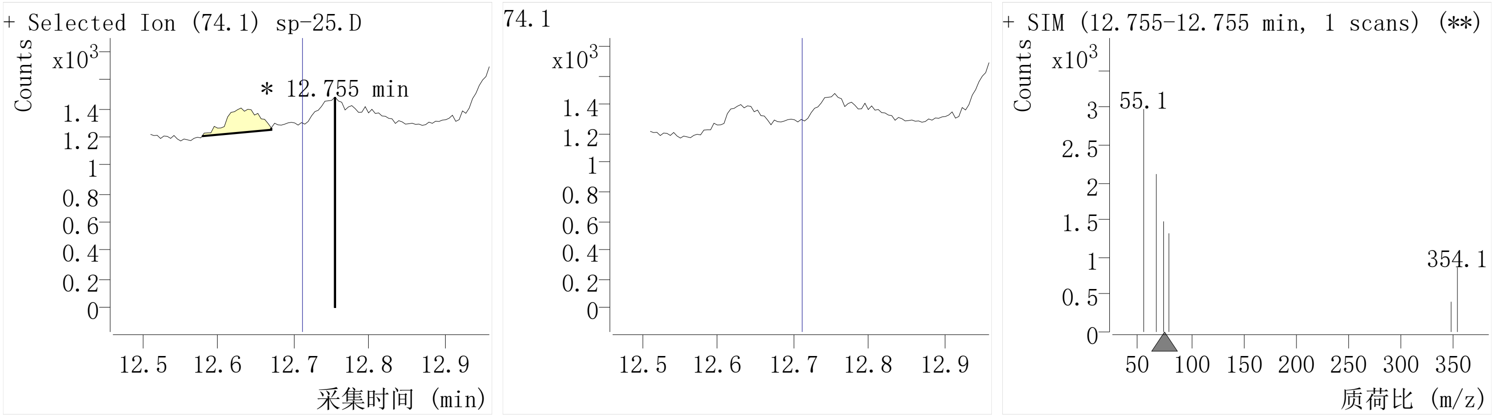

C24:0

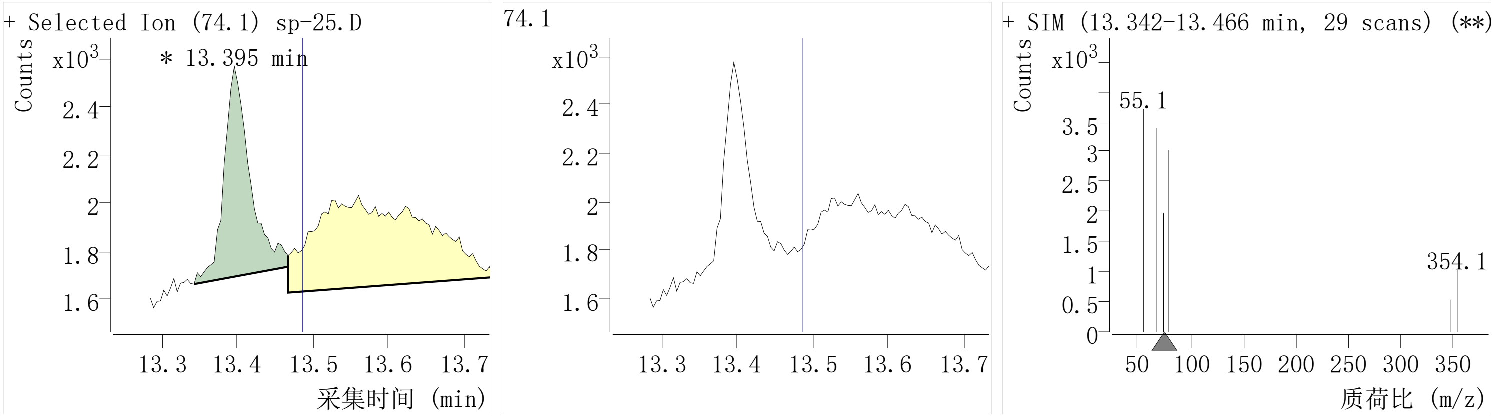

C22:6

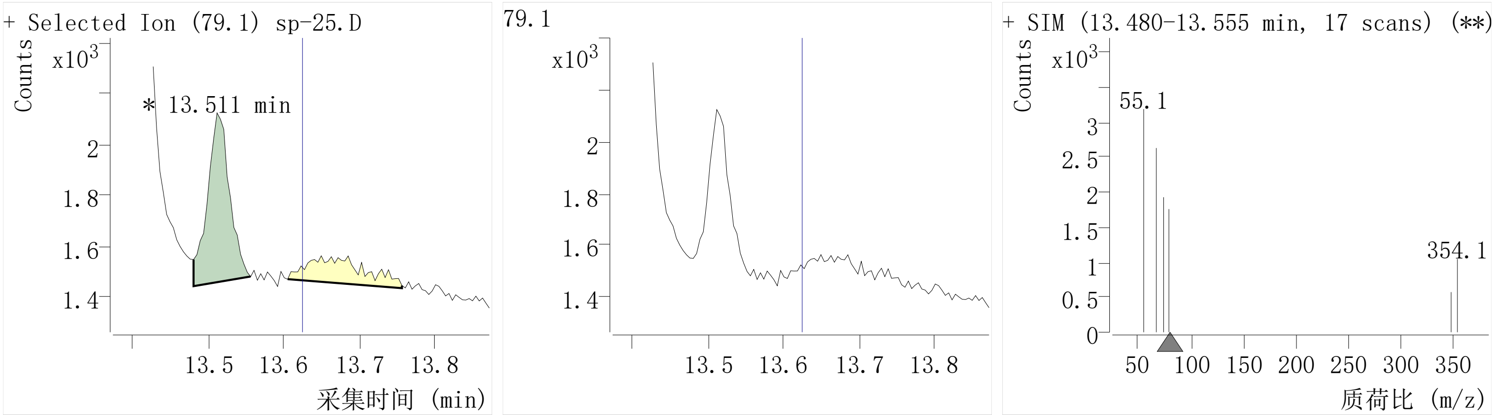
+ Selected Ion (79.1) sp-25.D
Counts
x10^3
* 13.511 min
2
1.8
1.6
1.4
13.5 13.6 13.7 13.8
采集时间 (min)
79.1
x10^3
2
1.8
1.6
1.4
13.5 13.6 13.7 13.8
+ SIM (13.480-13.555 min, 17 scans) (**)
Counts
x10^3
55.1
3
2.5
2
1.5
1
0.5
0
354.1
50 100 150 200 250 300 350
质荷比 (m/z)

C24:1

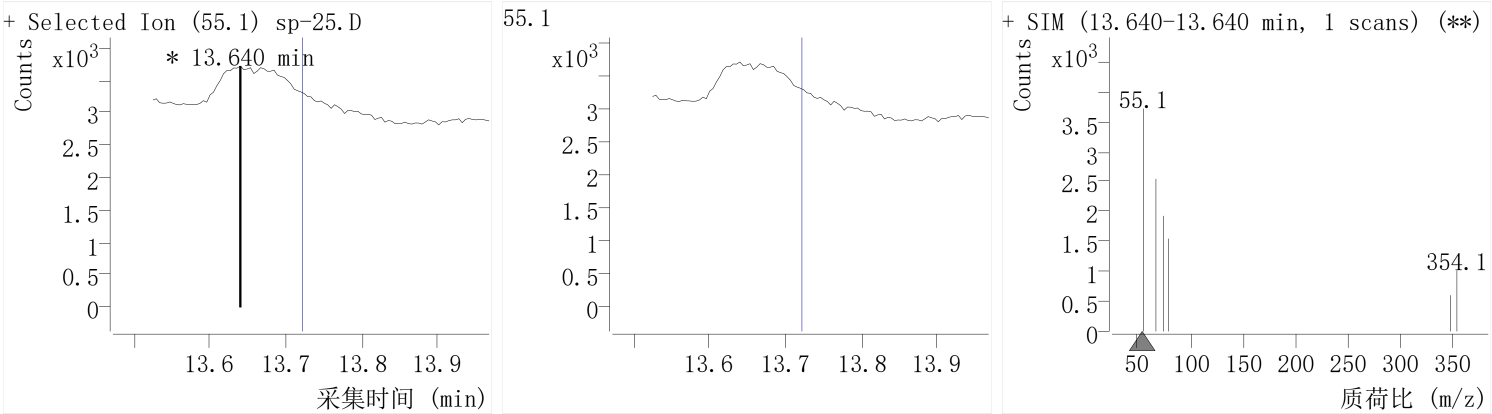
+ Selected Ion (55.1) sp-25.D
Counts
x10^3
* 13.640 min
3
2.5
2
1.5
1
0.5
0
13.6 13.7 13.8 13.9
采集时间 (min)
55.1
x10^3
3
2.5
2
1.5
1
0.5
0
13.6 13.7 13.8 13.9
+ SIM (13.640-13.640 min, 1 scans) (**)
Counts
x10^3
55.1
3.5
3
2.5
2
1.5
1
0.5
0
354.1
50 100 150 200 250 300 350
质荷比 (m/z)

**批处理路径**	G:\GC-MS\HX250430-4-GCMS总脂肪酸靶向检测\HX250430-4\QuantResults\HX250430-4.batch.bin		
**分析时间**	2025/5/14 16:58	**分析员姓名**	DESKTOP-M3A0GPO\omics
**报告时间**	2025/5/16 14:53:29	**报告员姓名**	DESKTOP-M3A0GPO\omics
**最近校正更新**	2025/5/14 16:58	**批处理状态**	已处理
**定量批处理版本**	10.2	**定量报告版本**	10.2

**采集时间**	2025/5/9 7:17	**数据文件**	sp-26.D
**样品类型**	样品	**样品名称**	sp-26
**稀释**	1	**采集方法**	脂肪酸

样品色谱图

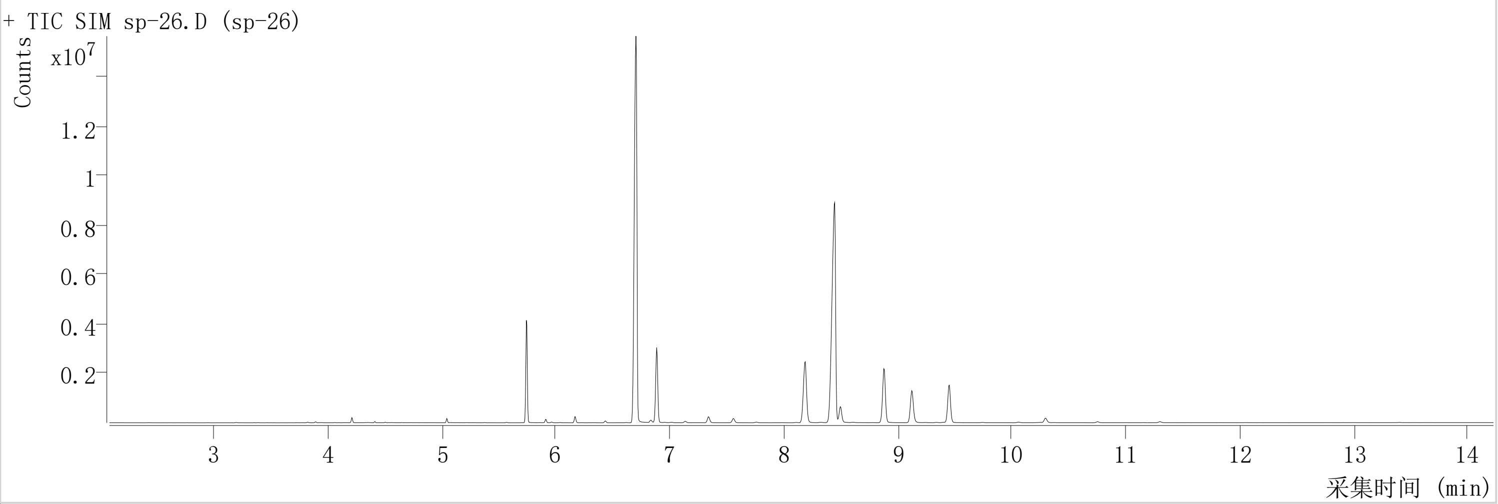

化合物	ISTD	RT	响应	ISTD 响应	响应比	最终浓度	单位
C4:0	C19:0	2.198	91	1841988	0.0000	ND	ug/ml
C6:0	C19:0	2.959	571	1841988	0.0003	0.0031	ug/ml
C8:0	C19:0	3.724	2405	1841988	0.0013	0.0090	ug/ml
C10:0	C19:0	4.413	26483	1841988	0.0144	0.2160	ug/ml
C11:0	C19:0	4.729	1364	1841988	0.0007	0.0061	ug/ml
C12:0	C19:0	5.044	103098	1841988	0.0560	0.8754	ug/ml
C13:0	C19:0	5.378	5817	1841988	0.0032	0.0339	ug/ml
C14:0	C19:0	5.742	2997518	1841988	1.6273	38.2002	ug/ml
C14:1	C19:0	5.911	69355	1841988	0.0377	1.7924	ug/ml
C15:0	C19:0	6.169	197248	1841988	0.1071	1.8869	ug/ml
C15:1	C19:0	6.432	0	1841988	0.0000	ND	ug/ml
C16:0	C19:0	6.699	16266664	1841988	8.8310	365.0225	ug/ml
C16:1	C19:0	6.885	1796563	1841988	0.9753	59.5157	ug/ml
C17:0	C19:0	7.339	281191	1841988	0.1527	3.1849	ug/ml
C17:1	C19:0	7.557	131720	1841988	0.0715	3.8225	ug/ml
C18:0	C19:0	8.188	3682425	1841988	1.9992	43.8563	ug/ml
C18:1n9t	C19:0	8.326	12081	1841988	0.0066	0.3869	ug/ml
C18:1n9c	C19:0	8.442	8835371	1841988	4.7966	327.9417	ug/ml
C18:2n6t	C19:0	8.877	0	1841988	0.0000	ND	ug/ml
C18:2n6c	C19:0	8.877	1716583	1841988	0.9319	61.5508	ug/ml
C18:3n6	C19:0	9.122	19043	1841988	0.0103	ND	ug/ml
C18:3n3	C19:0	9.451	1216822	1841988	0.6606	32.8378	ug/ml
C20:0	C19:0	10.056	40645	1841988	0.0221	0.5906	ug/ml
C20:1	C19:0	10.296	178140	1841988	0.0967	5.9997	ug/ml
C20:2	C19:0	10.749	33485	1841988	0.0182	1.1657	ug/ml
C21:0	C19:0	10.963	2159	1841988	0.0012	0.0324	ug/ml
C20:3n6	C19:0	11.003	2744	1841988	0.0015	0.1383	ug/ml
C20:4n6	C19:0	11.154	3610	1841988	0.0020	0.1575	ug/ml
C20:3n3	C19:0	11.301	35571	1841988	0.0193	1.0606	ug/ml
C20:5n3	C19:0	11.696	663	1841988	0.0004	0.0551	ug/ml

定量分析完成报告

化合物	ISTD	RT	响应	ISTD 响应	响应比	最终浓度	单位
C22:0	C19:0	11.821	4275	1841988	0.0023	0.0954	ug/ml
C22:1n9	C19:0	12.048	4960	1841988	0.0027	0.1846	ug/ml
C22:2n6	C19:0	12.470	697	1841988	0.0004	0.0669	ug/ml
C23:0	C19:0	12.786	0	1841988	0.0000	ND	ug/ml
C24:0	C19:0	13.395	3298	1841988	0.0018	0.0871	ug/ml
C22:6	C19:0	13.515	687	1841988	0.0004	0.0328	ug/ml
C24:1	C19:0	13.631	0	1841988	0.0000	ND	ug/ml

C4:0

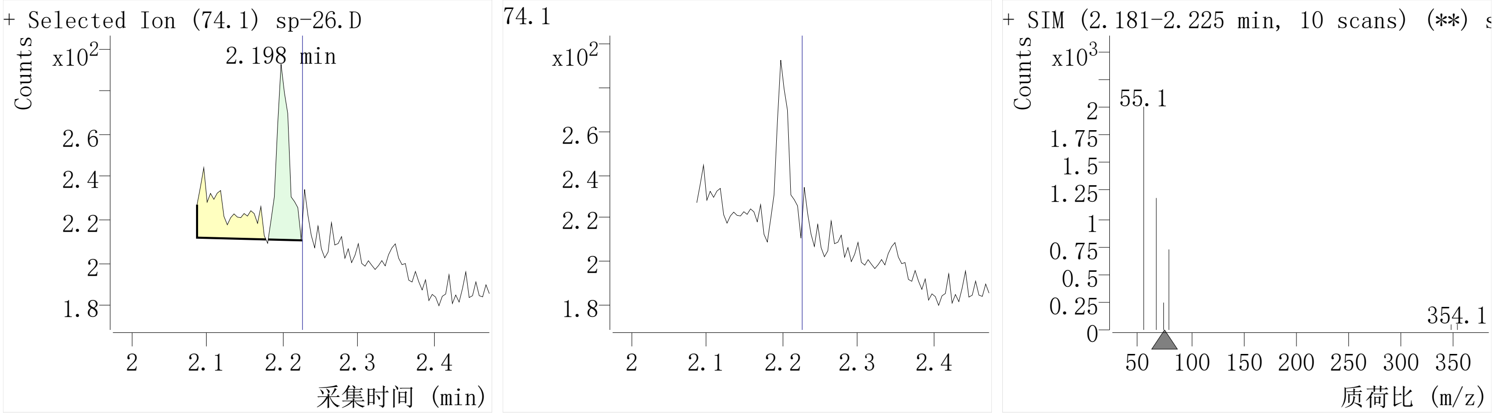

C6:0

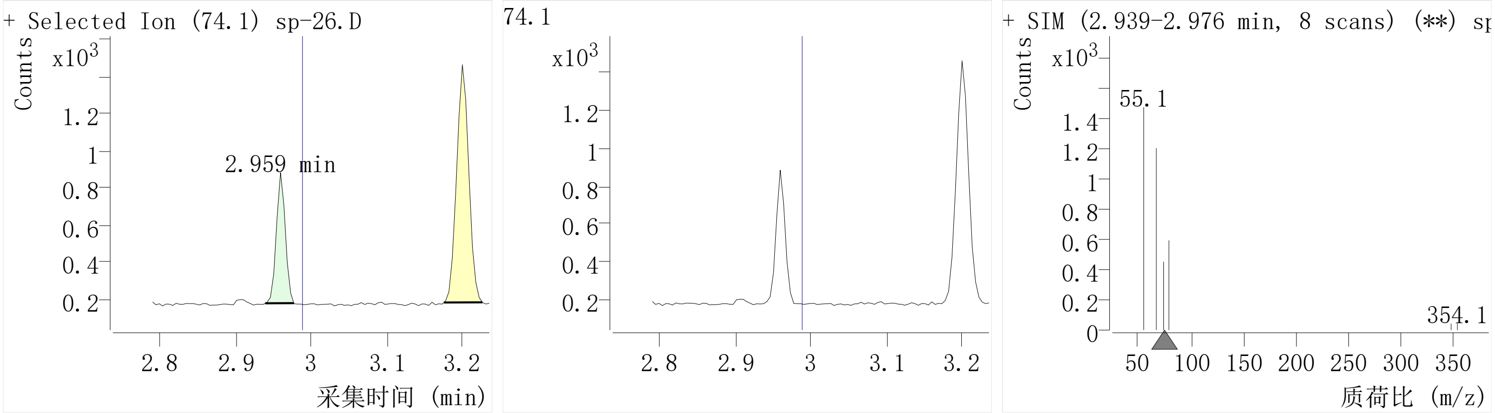

C8:0

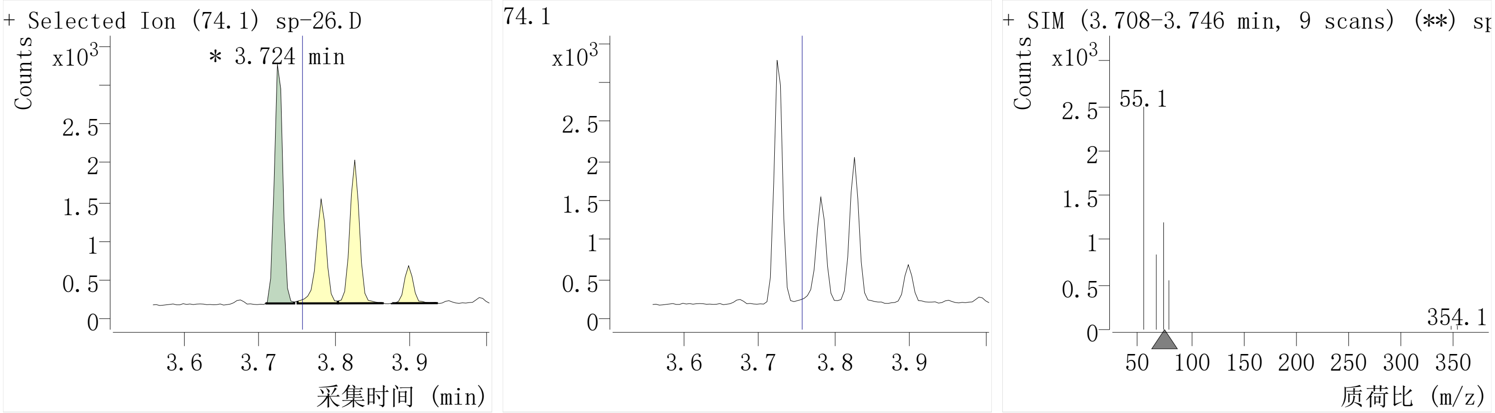

C10:0

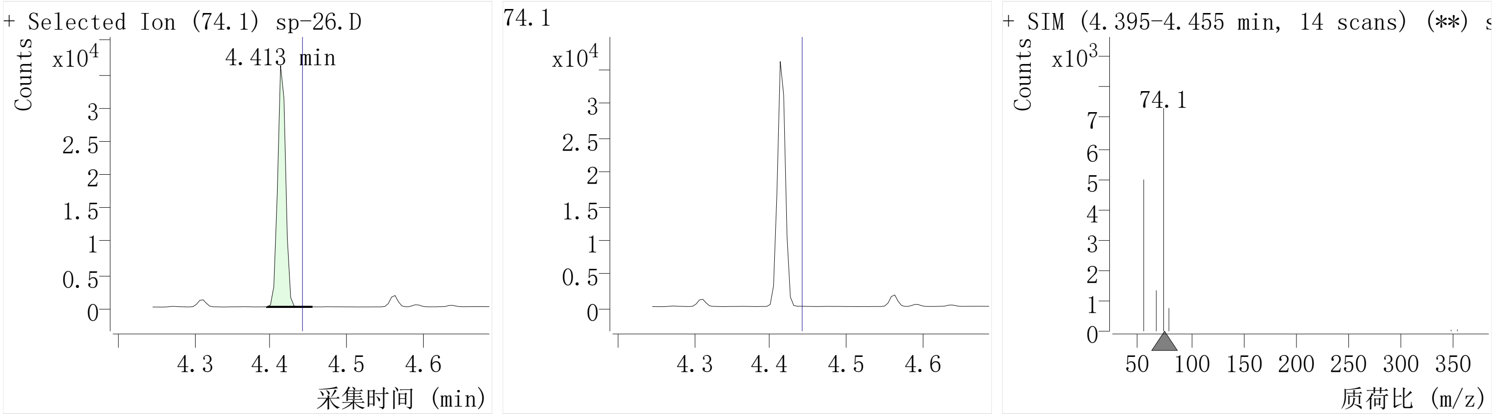

C11:0

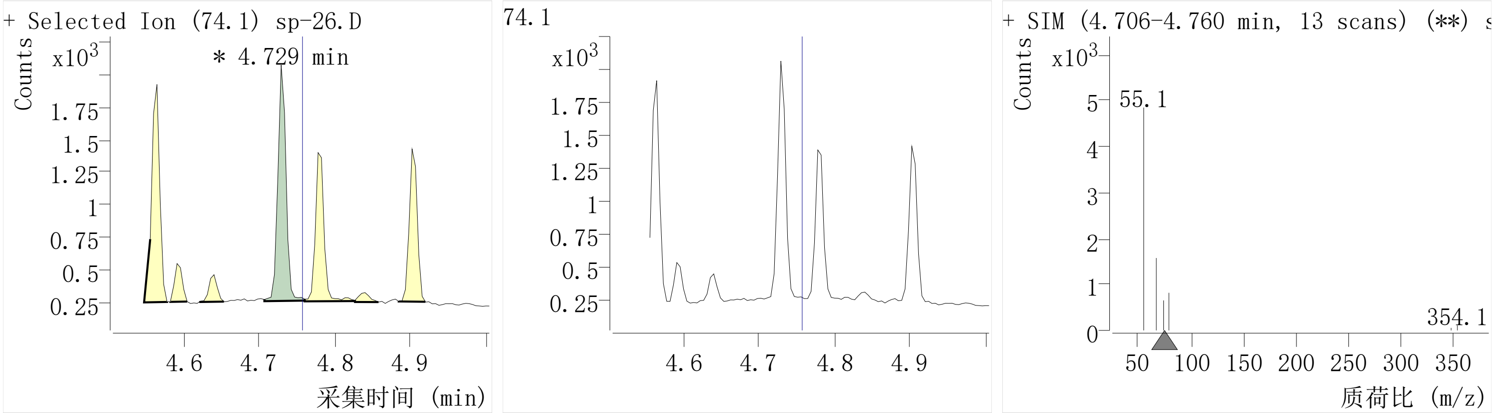

C12:0

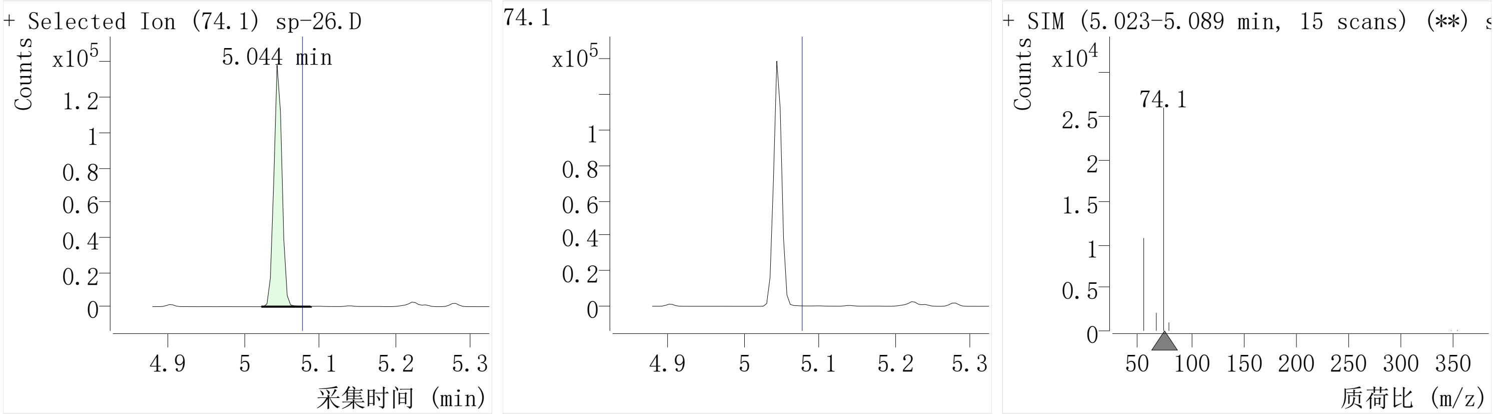

C13:0

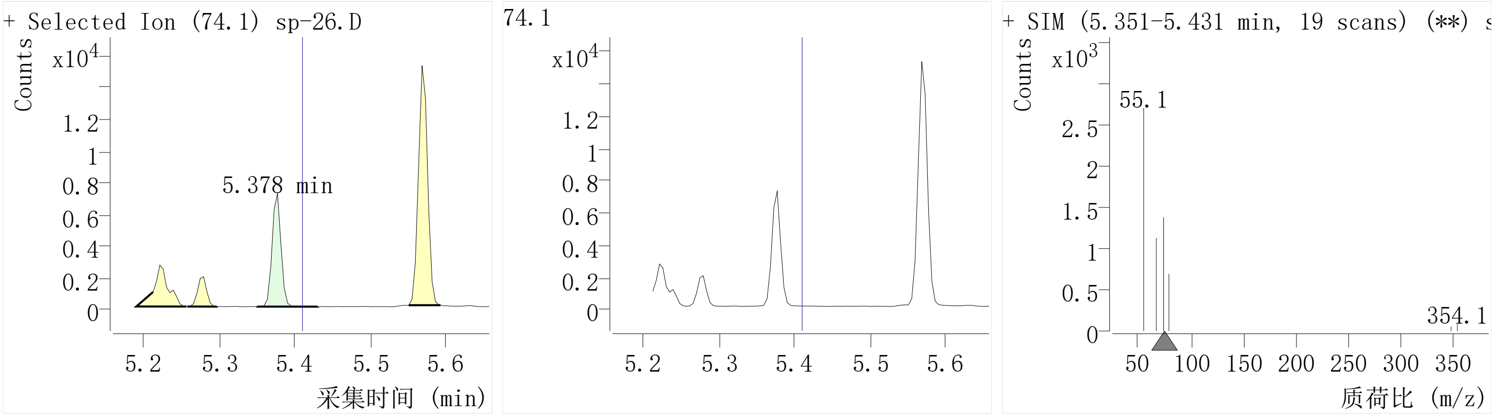

C14:0

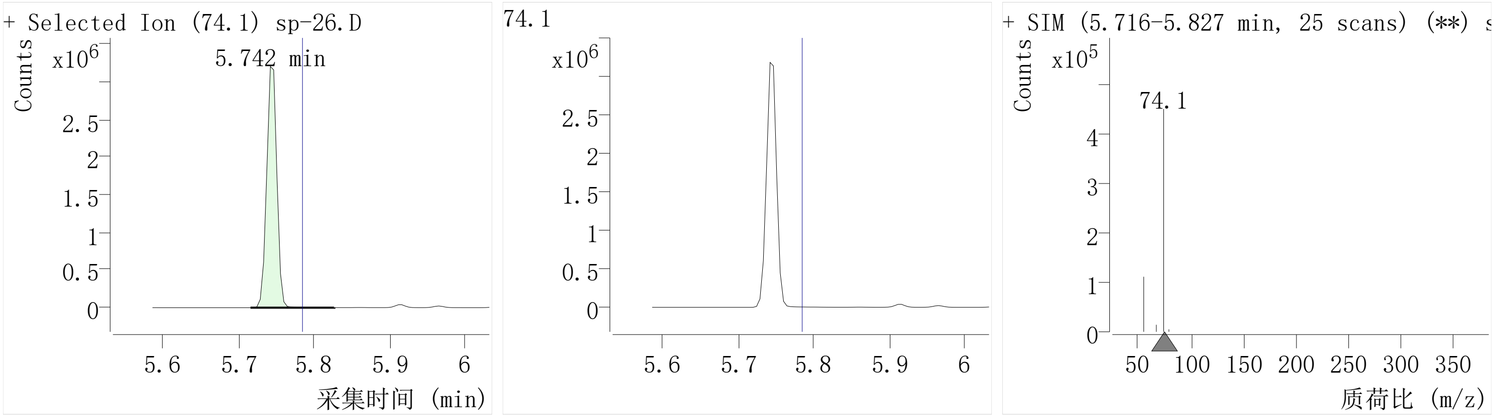

C14:1

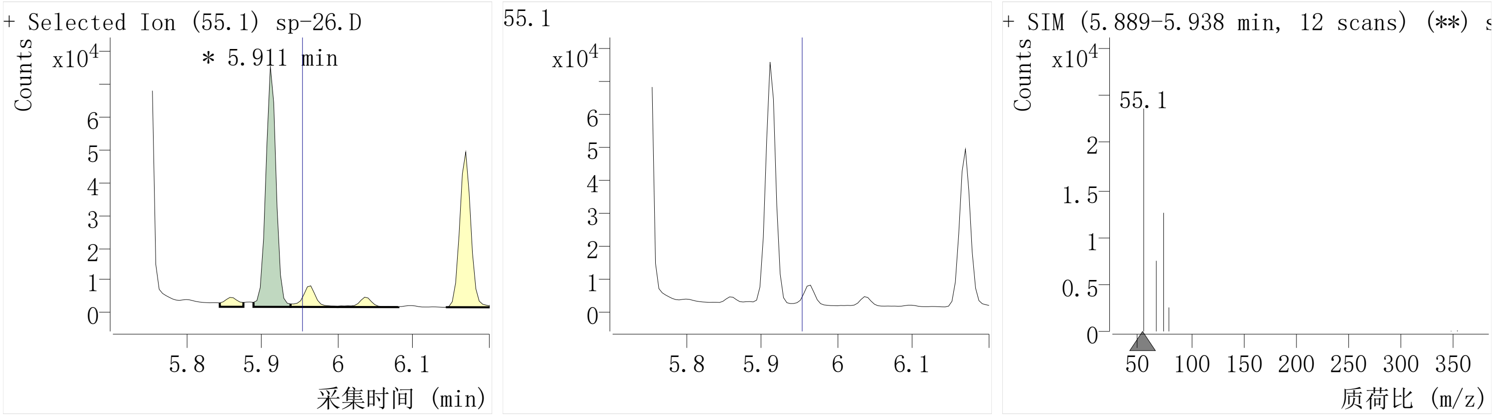

C15:0

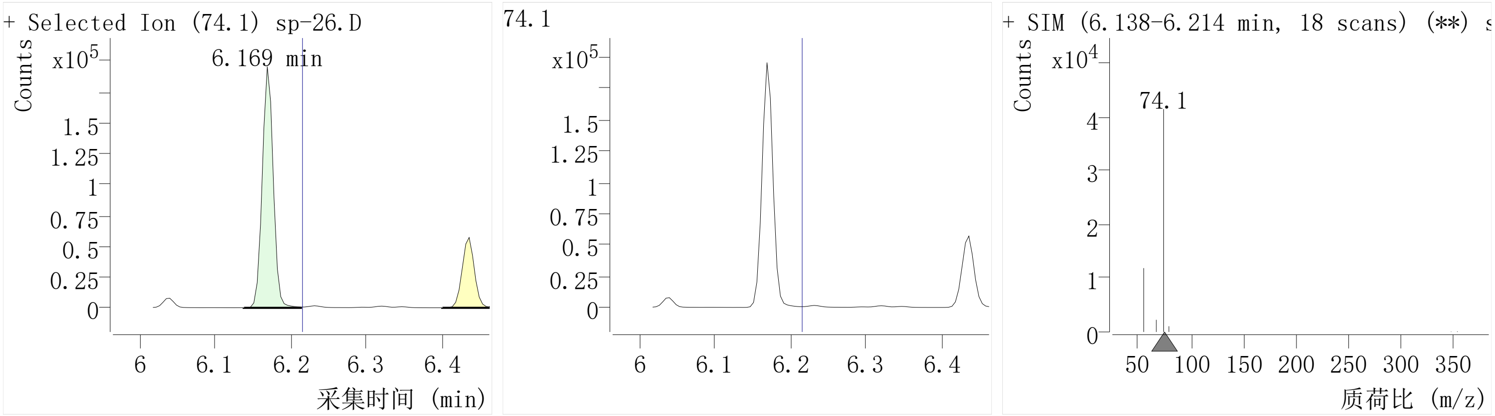

C15:1

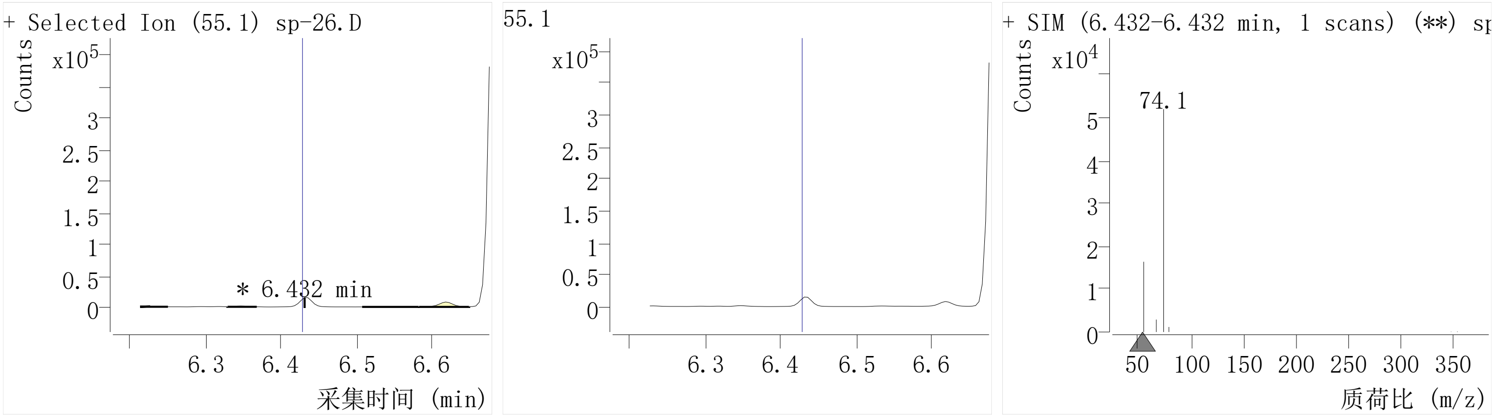

C16:0

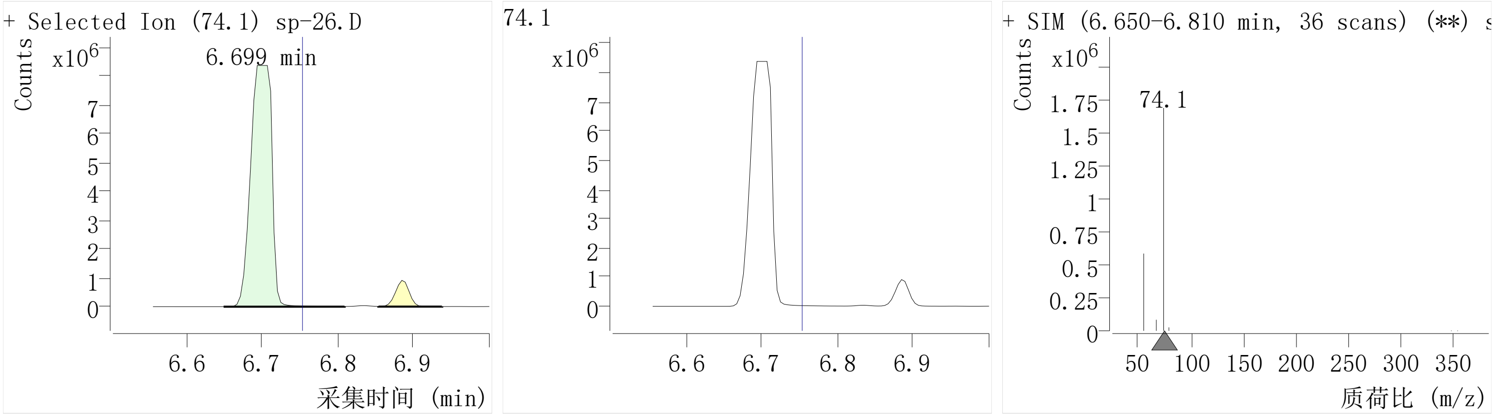

C16:1

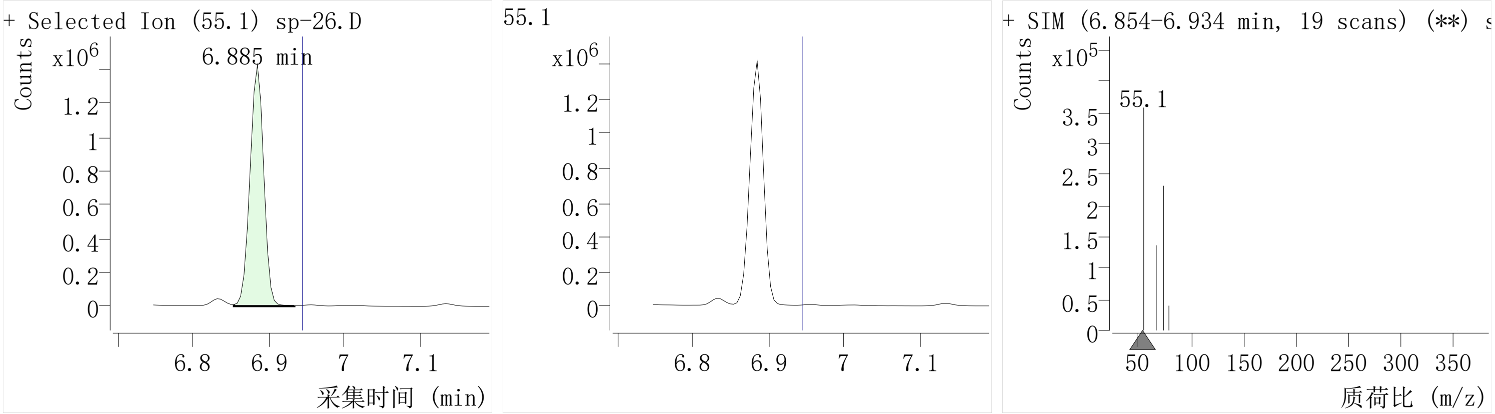

C17:0

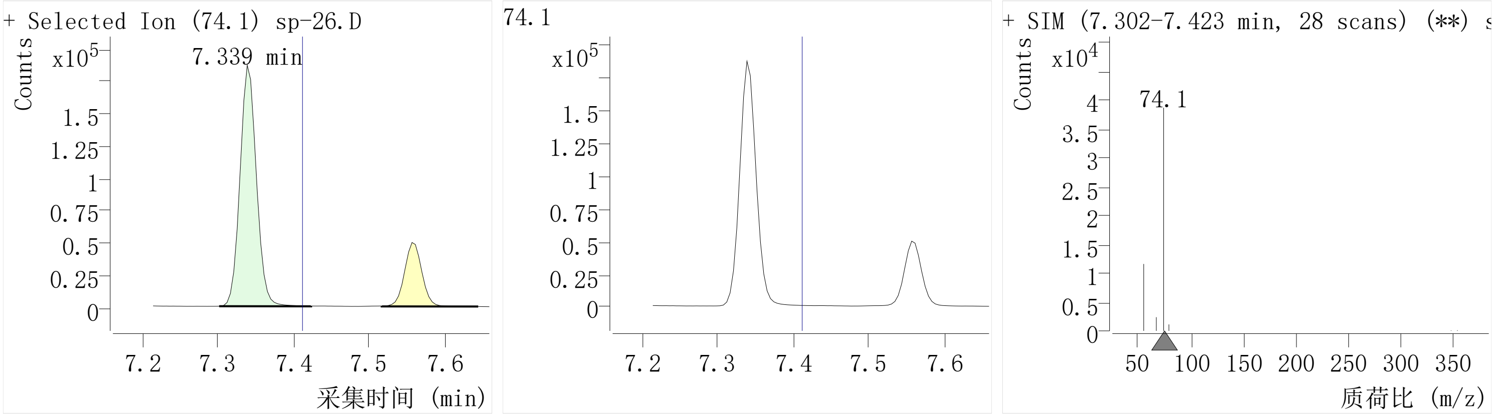

C17:1

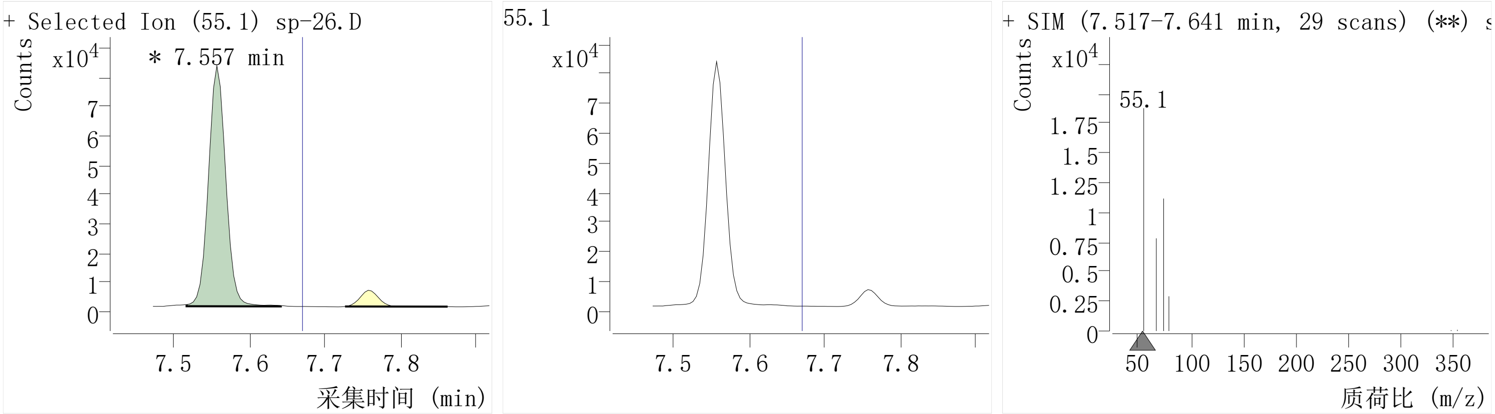

C18:0

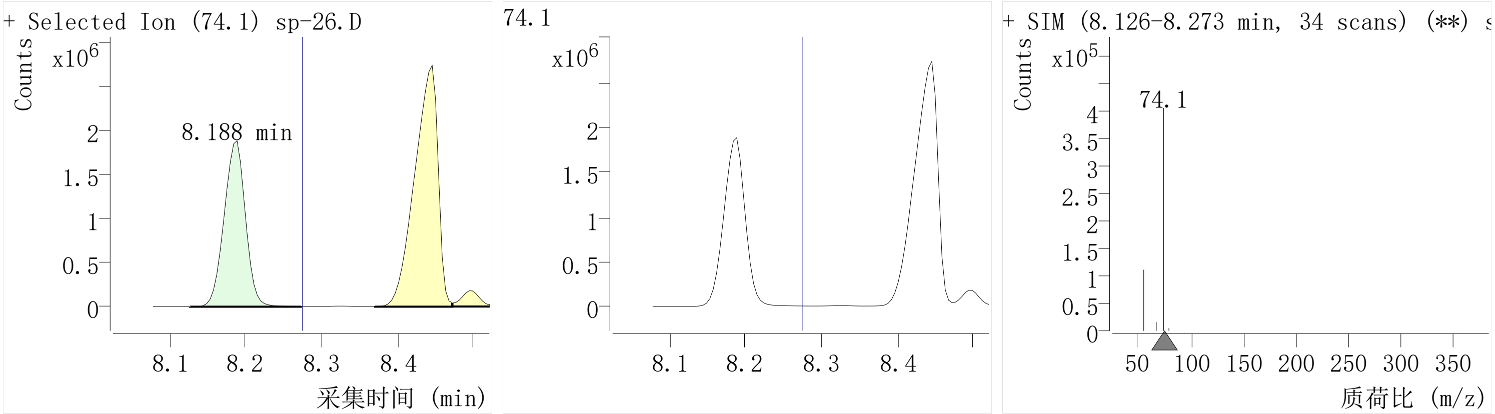

C18:1n9t

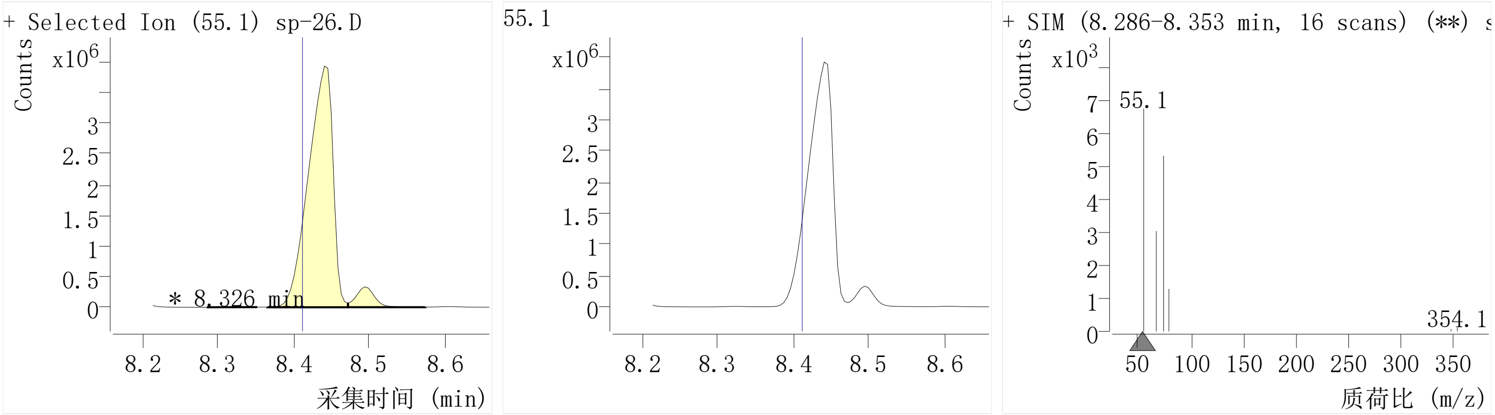

C18:1n9c

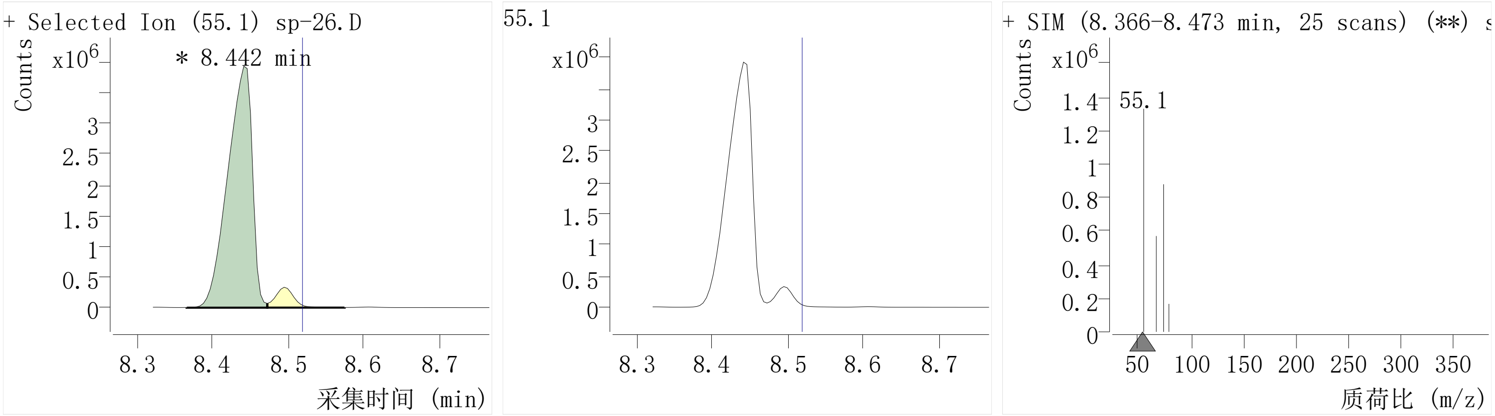

C18:2n6t

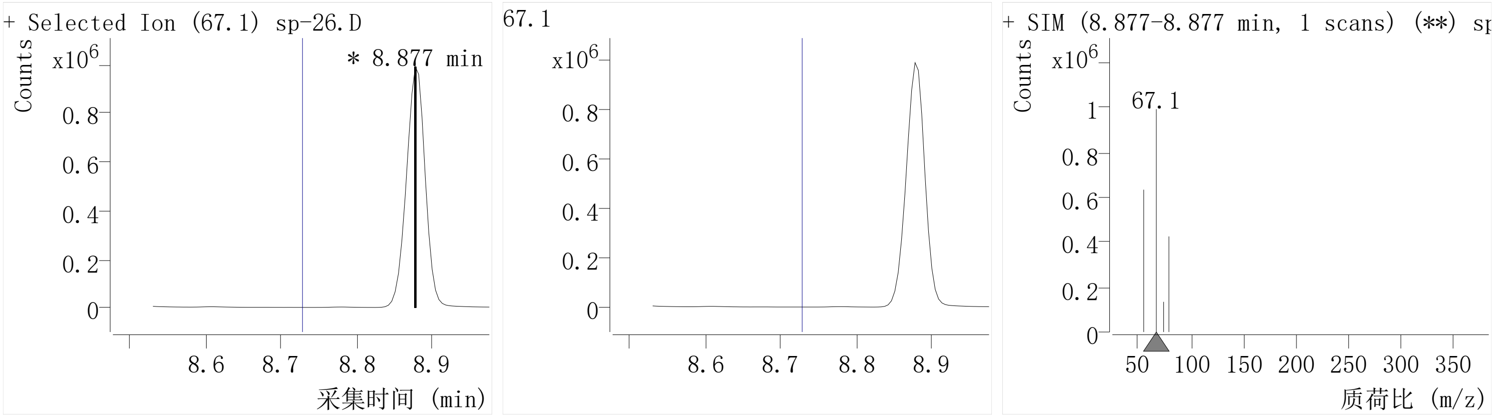

C18:2n6c

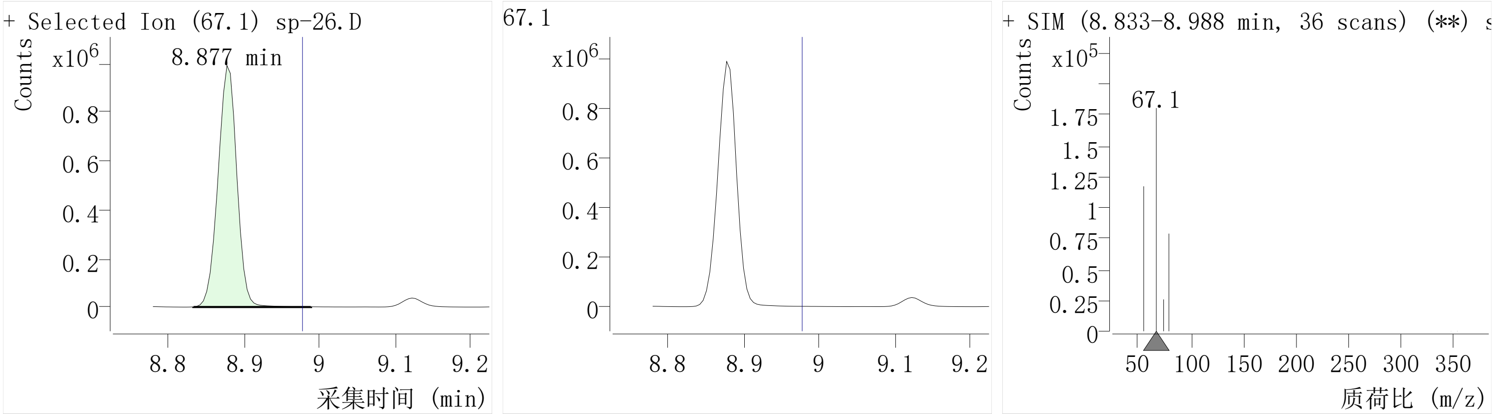

C18:3n6

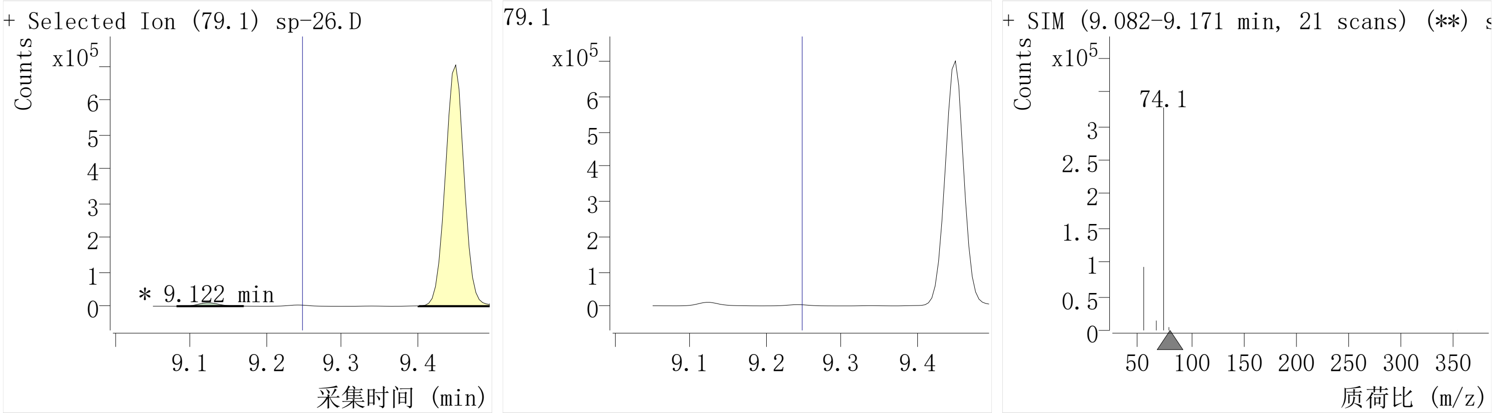

C18:3n3

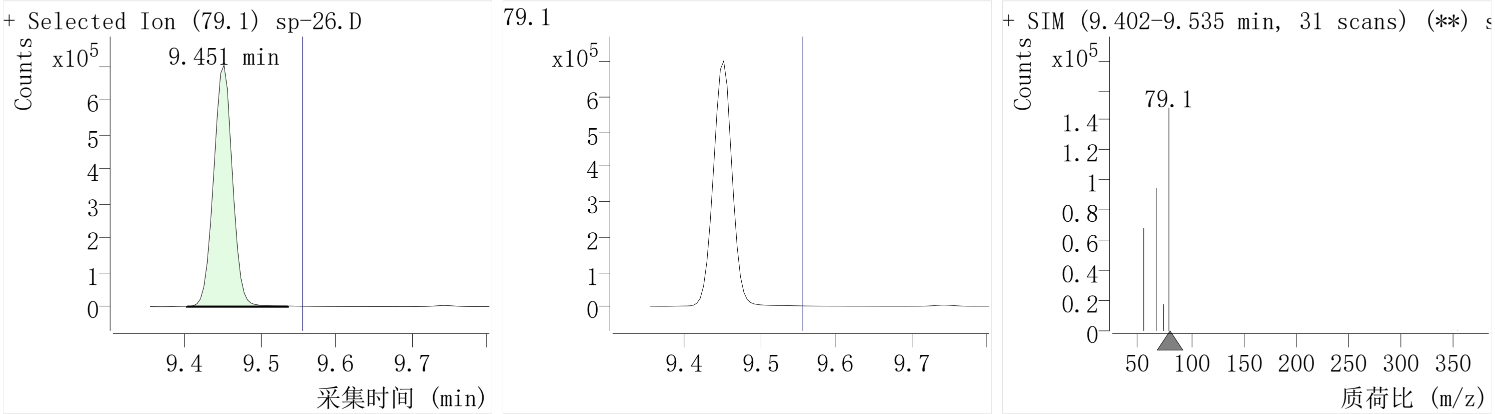

C20:0

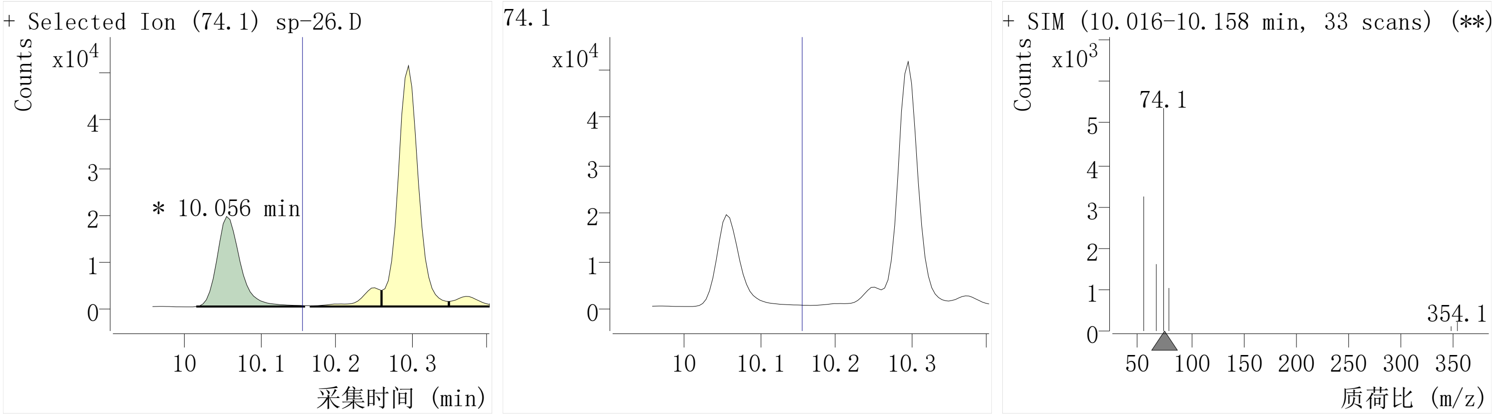

C20:1

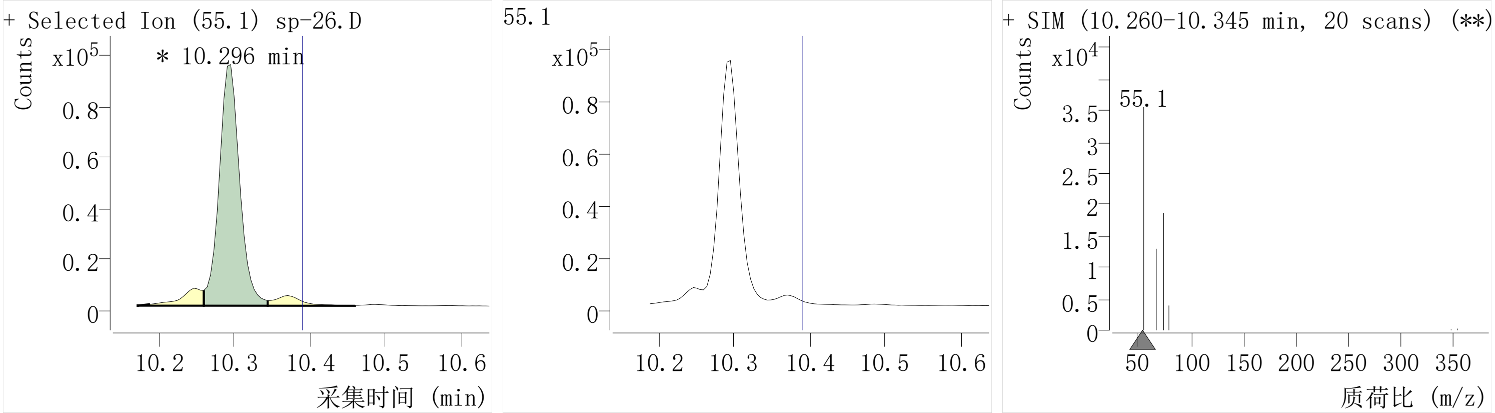

C20:2

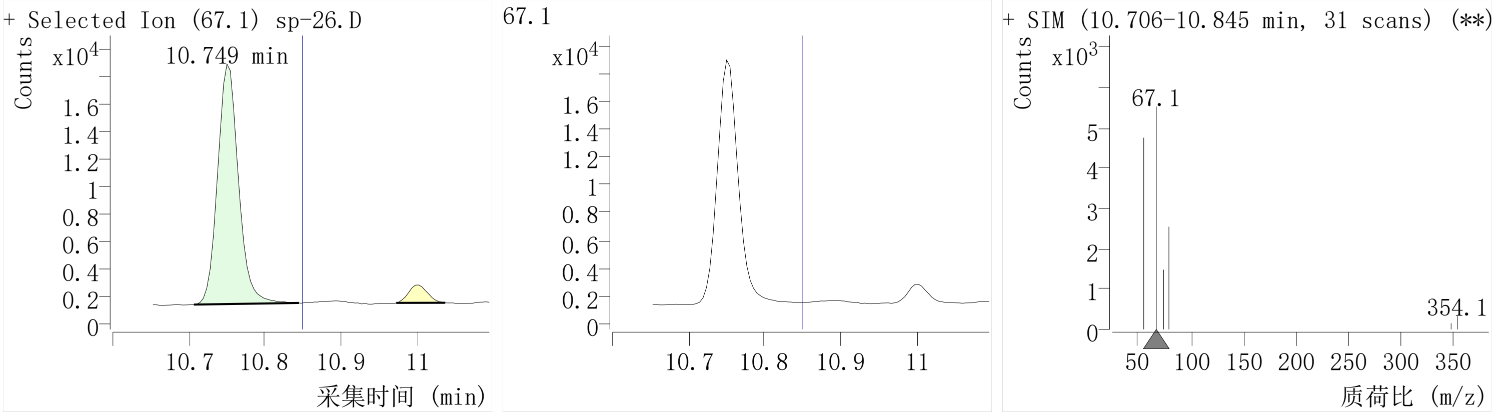

C21:0

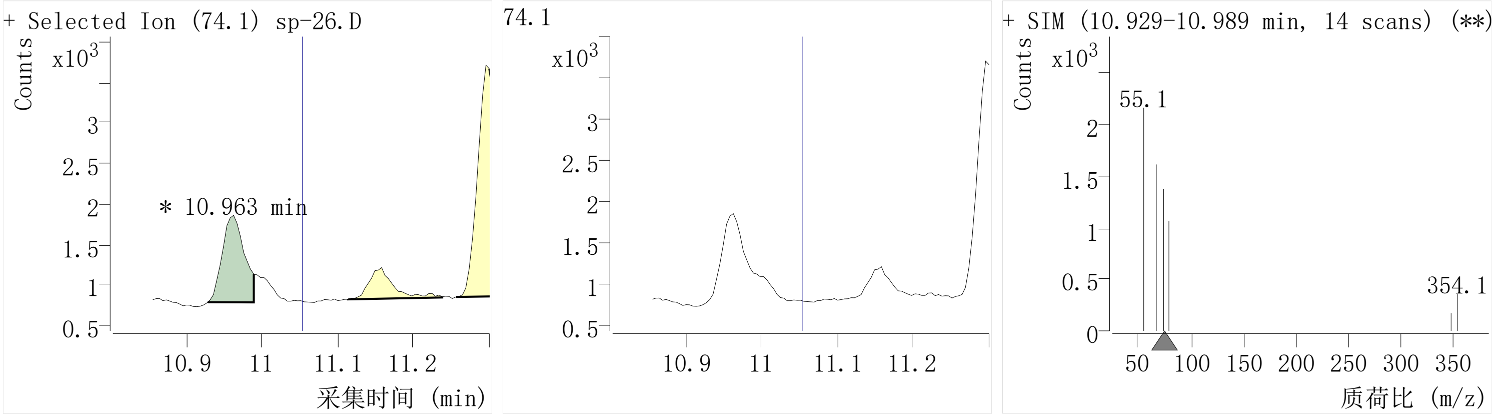

C20:3n6

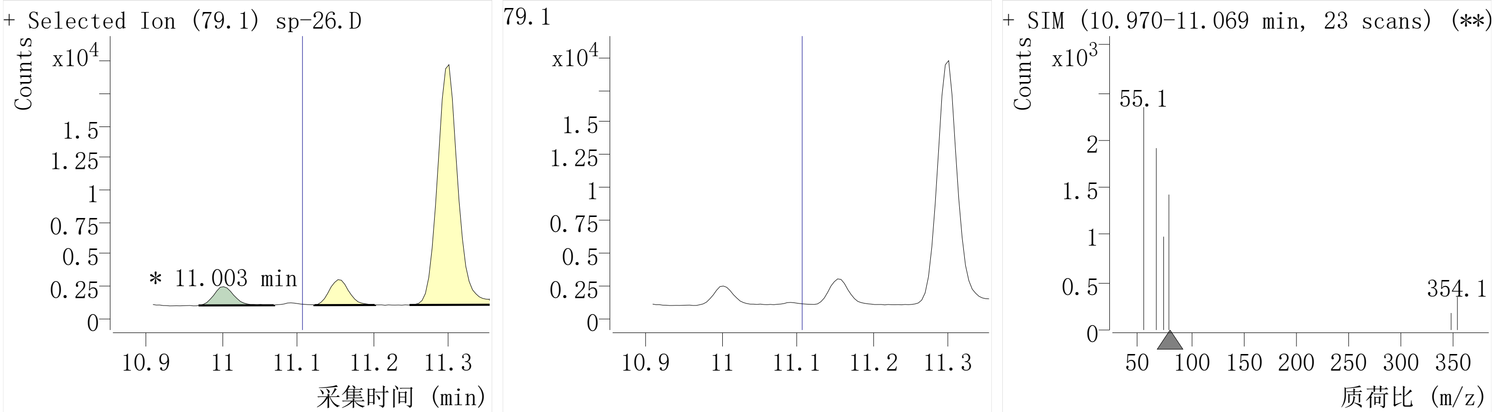

C20:4n6

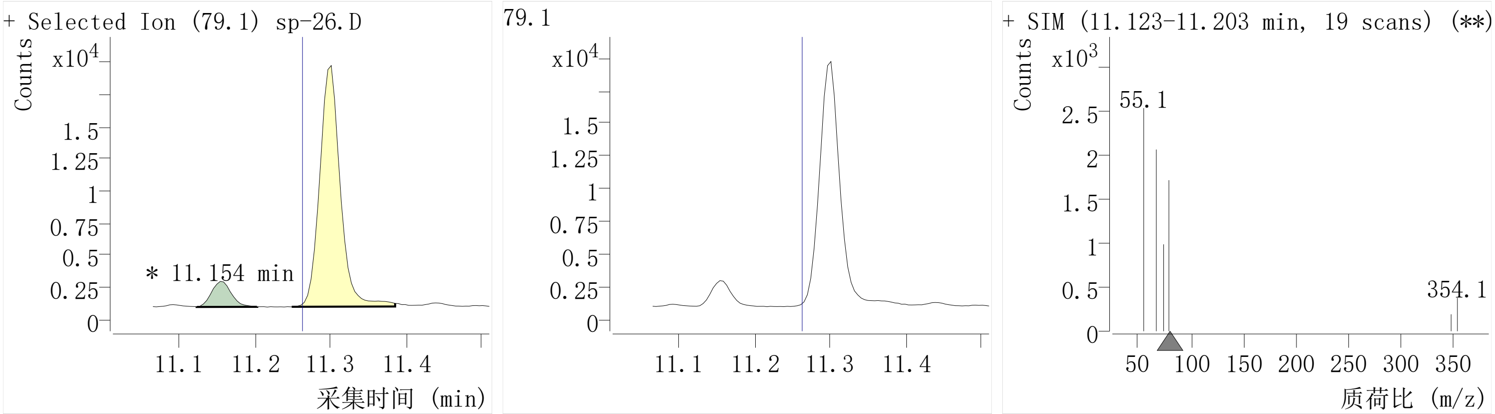

C20:3n3

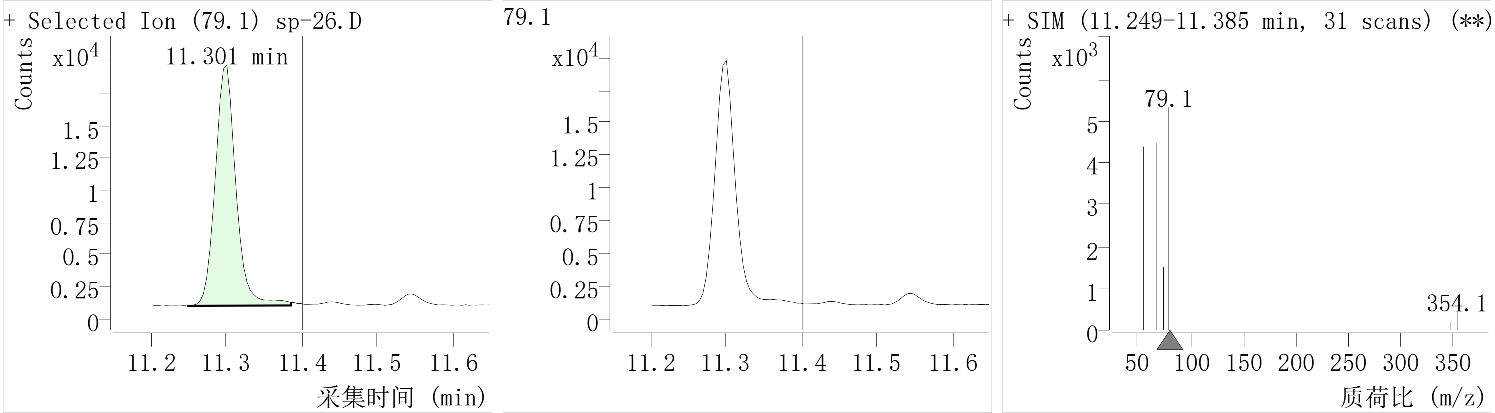

C20:5n3

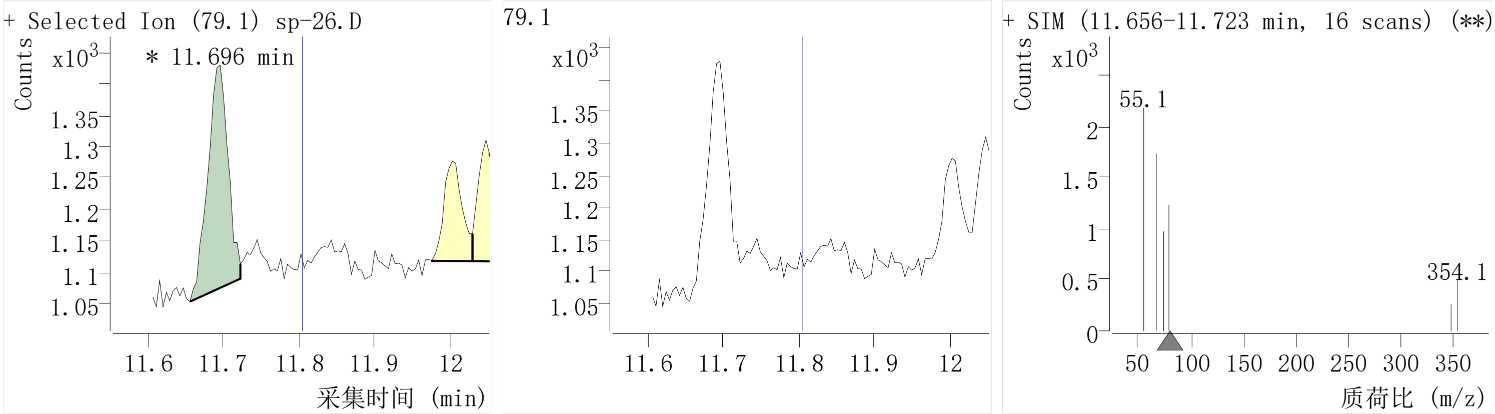

C22:0

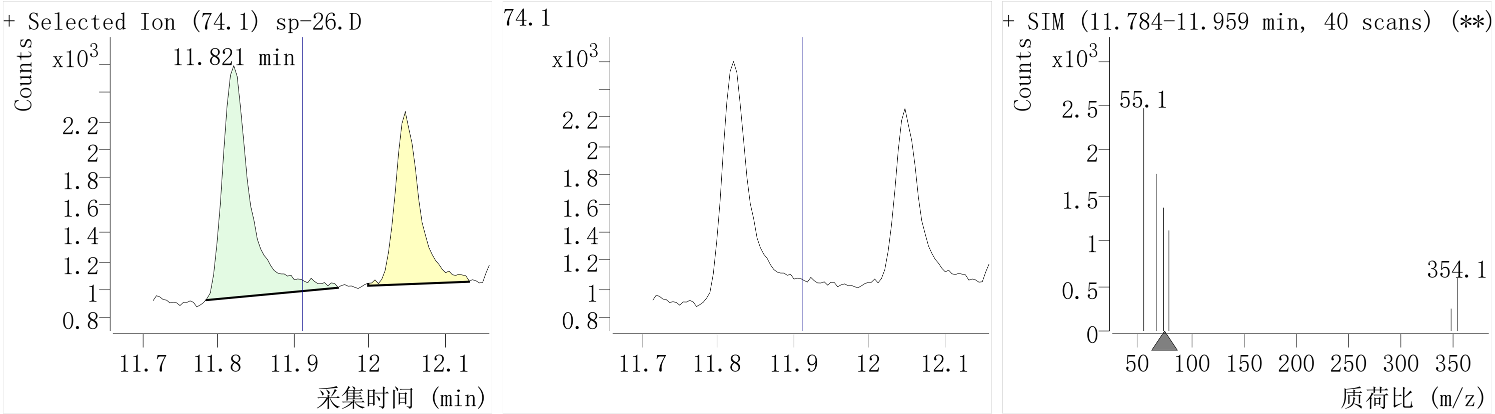

C22:1n9

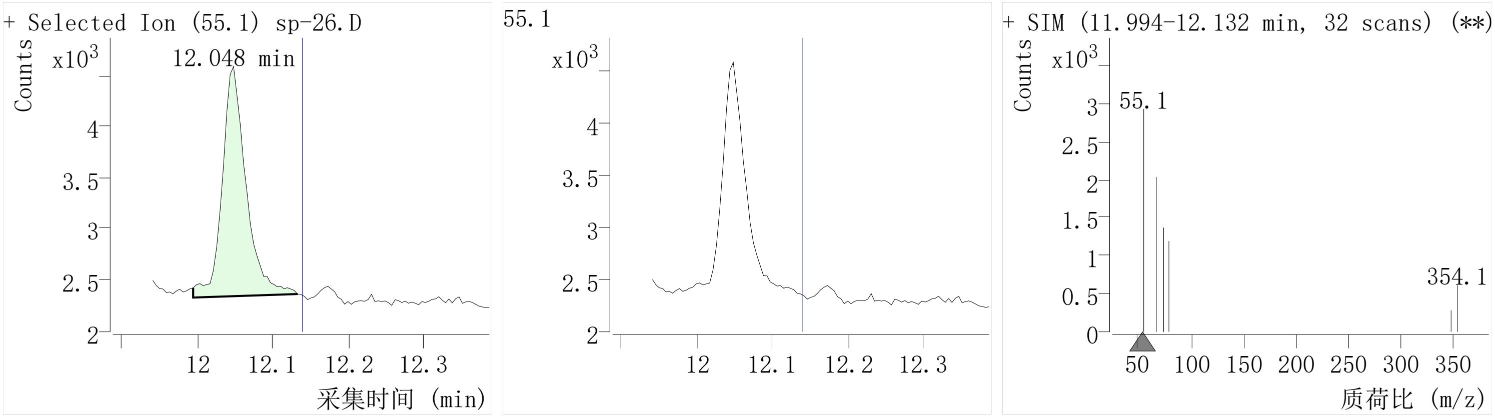

C22:2n6

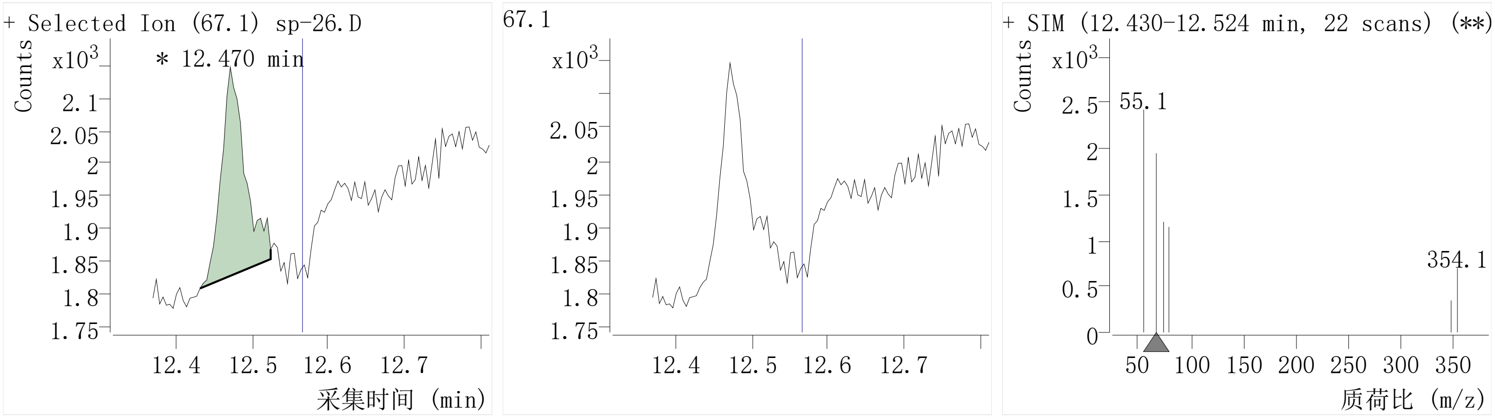

C23:0

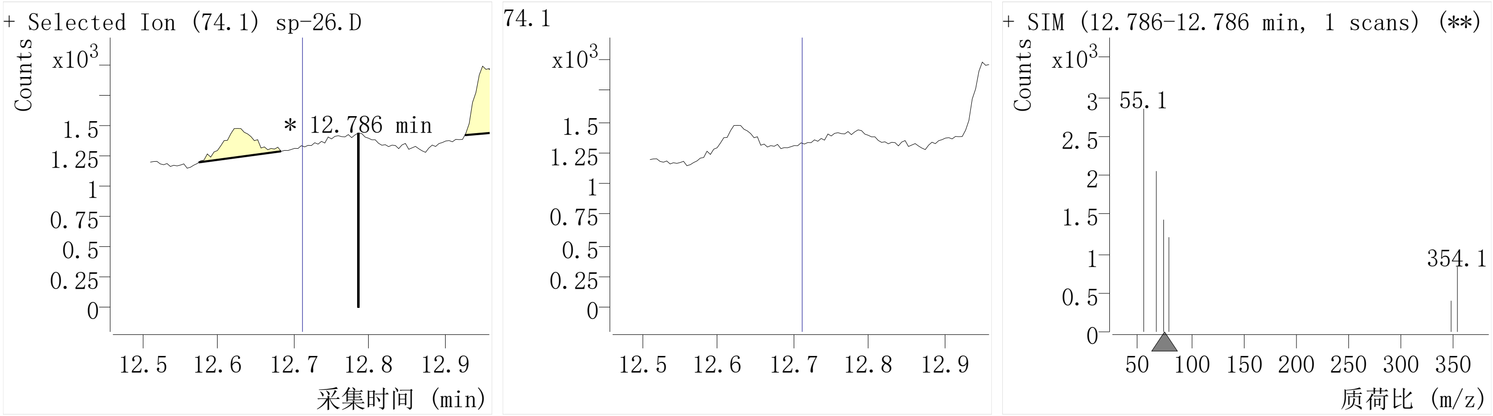

C24:0

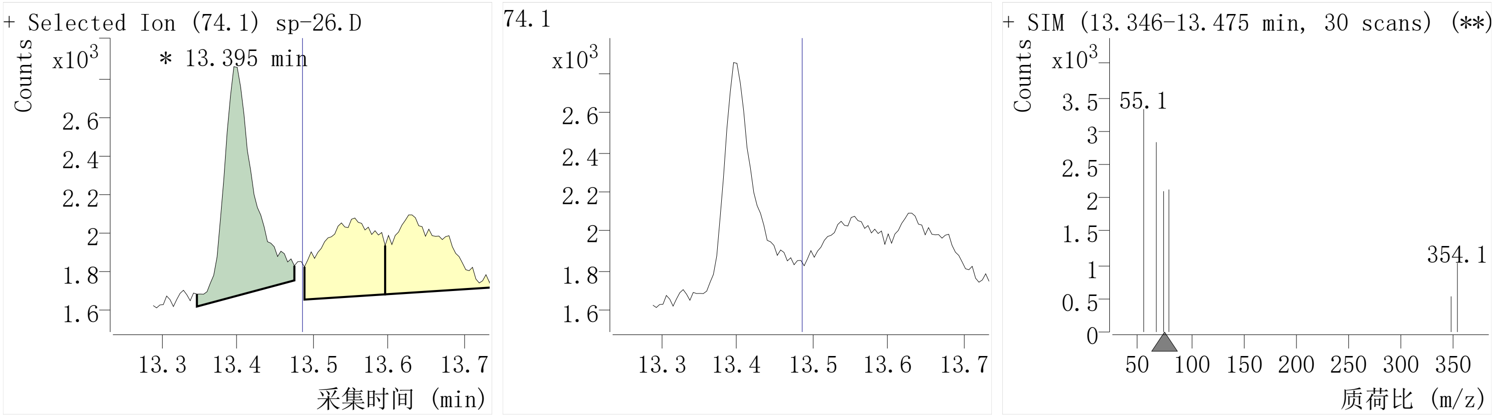

C22:6

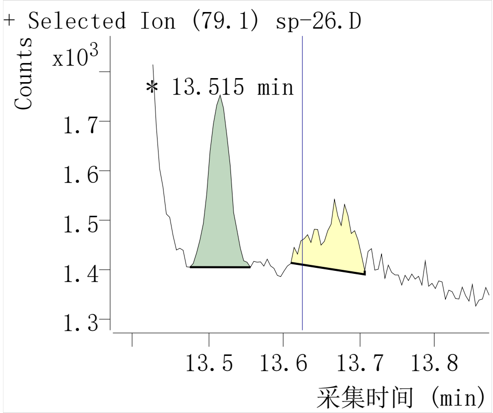
+ Selected Ion (79.1) sp-26.D
Counts
x10^3
* 13.515 min
1.7
1.6
1.5
1.4
1.3
13.5
13.6
13.7
13.8
采集时间 (min)

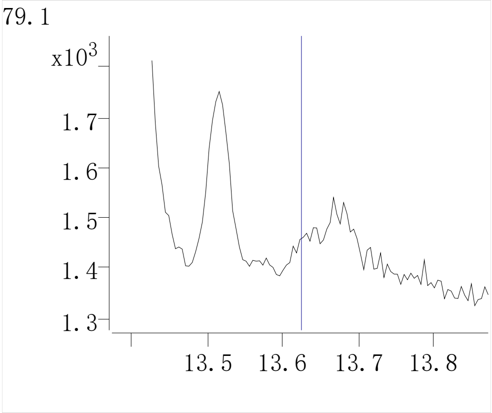
79.1
x10^3
1.7
1.6
1.5
1.4
1.3
13.5
13.6
13.7
13.8

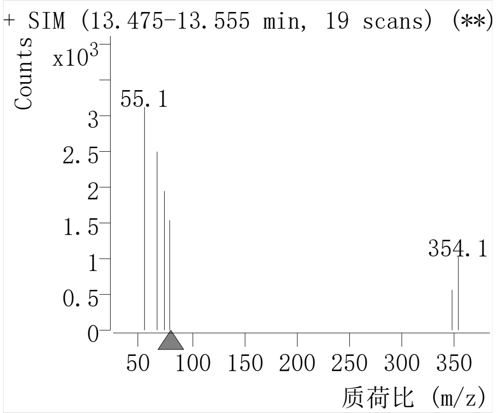
+ SIM (13.475-13.555 min, 19 scans) (**)
Counts
x10^3
55.1
354.1
3
2.5
2
1.5
1
0.5
0
50
100
150
200
250
300
350
质荷比 (m/z)

C24:1

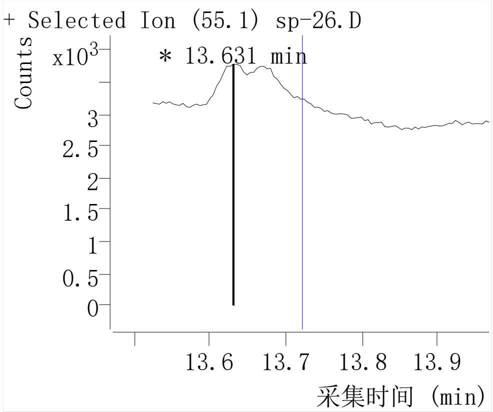
+ Selected Ion (55.1) sp-26.D
Counts
x10^3
* 13.631 min
3
2.5
2
1.5
1
0.5
0
13.6
13.7
13.8
13.9
采集时间 (min)

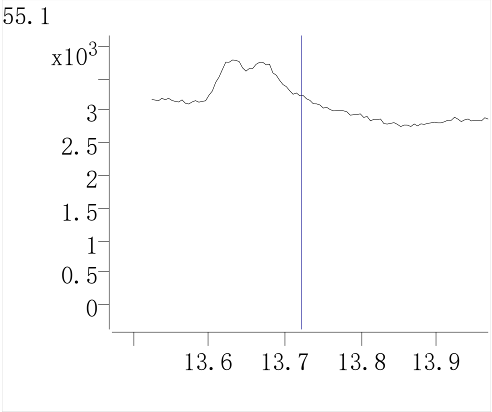
55.1
x10^3
3
2.5
2
1.5
1
0.5
0
13.6
13.7
13.8
13.9

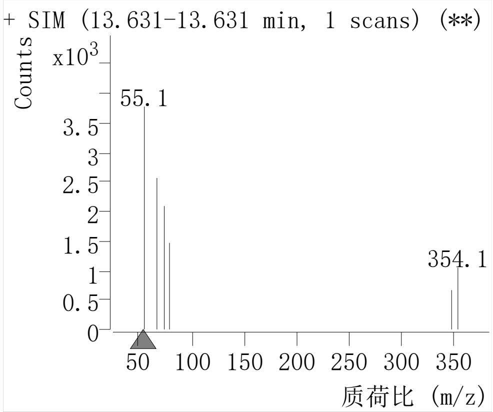
+ SIM (13.631-13.631 min, 1 scans) (**)
Counts
x10^3
55.1
354.1
3.5
3
2.5
2
1.5
1
0.5
0
50
100
150
200
250
300
350
质荷比 (m/z)

定量分析完成报告

**批处理路径**	G:\GC-MS\HX250430-4-GCMS总脂肪酸靶向检测\HX250430-4\QuantResults\HX250430-4.batch.bin		
**分析时间**	2025/5/14 16:58	**分析员姓名**	DESKTOP-M3A0GPO\omics
**报告时间**	2025/5/16 14:53:31	**报告员姓名**	DESKTOP-M3A0GPO\omics
**最近校正更新**	2025/5/14 16:58	**批处理状态**	已处理
**定量批处理版本**	10.2	**定量报告版本**	10.2

**采集时间**	2025/5/9 7:37	**数据文件**	sp-27.D
**样品类型**	样品	**样品名称**	sp-27
**稀释**	1	**采集方法**	脂肪酸

样品色谱图

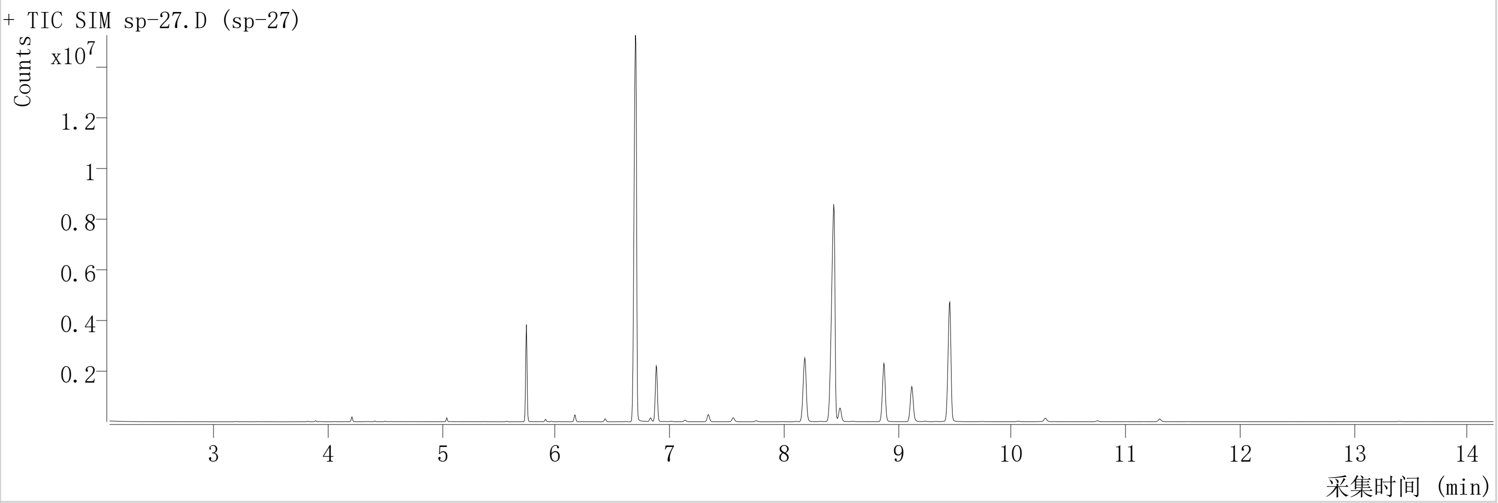

化合物	ISTD	RT	响应	ISTD 响应	响应比	最终浓度	单位
C4:0	C19:0	2.199	27	1938349	0.0000	ND	ug/ml
C6:0	C19:0	2.959	326	1938349	0.0002	0.0014	ug/ml
C8:0	C19:0	3.724	1331	1938349	0.0007	0.0037	ug/ml
C10:0	C19:0	4.413	18718	1938349	0.0097	0.1321	ug/ml
C11:0	C19:0	4.729	1388	1938349	0.0007	0.0059	ug/ml
C12:0	C19:0	5.044	90389	1938349	0.0466	0.7030	ug/ml
C13:0	C19:0	5.373	6601	1938349	0.0034	0.0371	ug/ml
C14:0	C19:0	5.743	2640268	1938349	1.3621	30.1533	ug/ml
C14:1	C19:0	5.912	44072	1938349	0.0227	1.0400	ug/ml
C15:0	C19:0	6.169	219592	1938349	0.1133	2.0092	ug/ml
C15:1	C19:0	6.432	0	1938349	0.0000	ND	ug/ml
C16:0	C19:0	6.698	15651428	1938349	8.0746	322.1577	ug/ml
C16:1	C19:0	6.881	1349551	1938349	0.6962	42.4595	ug/ml
C17:0	C19:0	7.339	326975	1938349	0.1687	3.5193	ug/ml
C17:1	C19:0	7.557	122779	1938349	0.0633	3.3826	ug/ml
C18:0	C19:0	8.184	3722941	1938349	1.9207	41.9104	ug/ml
C18:1n9t	C19:0	8.322	9964	1938349	0.0051	0.3032	ug/ml
C18:1n9c	C19:0	8.437	8104375	1938349	4.1811	283.3158	ug/ml
C18:2n6t	C19:0	8.877	0	1938349	0.0000	ND	ug/ml
C18:2n6c	C19:0	8.877	1814962	1938349	0.9363	61.9340	ug/ml
C18:3n6	C19:0	9.122	22400	1938349	0.0116	0.0028	ug/ml
C18:3n3	C19:0	9.455	3899922	1938349	2.0120	100.0261	ug/ml
C20:0	C19:0	10.056	33573	1938349	0.0173	0.4709	ug/ml
C20:1	C19:0	10.291	137013	1938349	0.0707	4.3959	ug/ml
C20:2	C19:0	10.749	29862	1938349	0.0154	0.9962	ug/ml
C21:0	C19:0	10.958	2316	1938349	0.0012	0.0331	ug/ml
C20:3n6	C19:0	11.003	2913	1938349	0.0015	0.1391	ug/ml
C20:4n6	C19:0	11.154	3602	1938349	0.0019	0.1511	ug/ml
C20:3n3	C19:0	11.296	88100	1938349	0.0455	2.4273	ug/ml
C20:5n3	C19:0	11.697	1794	1938349	0.0009	0.0863	ug/ml

化合物	ISTD	RT	响应	ISTD 响应	响应比	最终浓度	单位
C22:0	C19:0	11.817	2133	1938349	0.0011	0.0559	ug/ml
C22:1n9	C19:0	12.043	2747	1938349	0.0014	0.0901	ug/ml
C22:2n6	C19:0	12.470	599	1938349	0.0003	0.0614	ug/ml
C23:0	C19:0	12.746	0	1938349	0.0000	ND	ug/ml
C24:0	C19:0	13.395	1745	1938349	0.0009	0.0469	ug/ml
C22:6	C19:0	13.511	826	1938349	0.0004	0.0367	ug/ml
C24:1	C19:0	13.666	0	1938349	0.0000	ND	ug/ml

C4:0

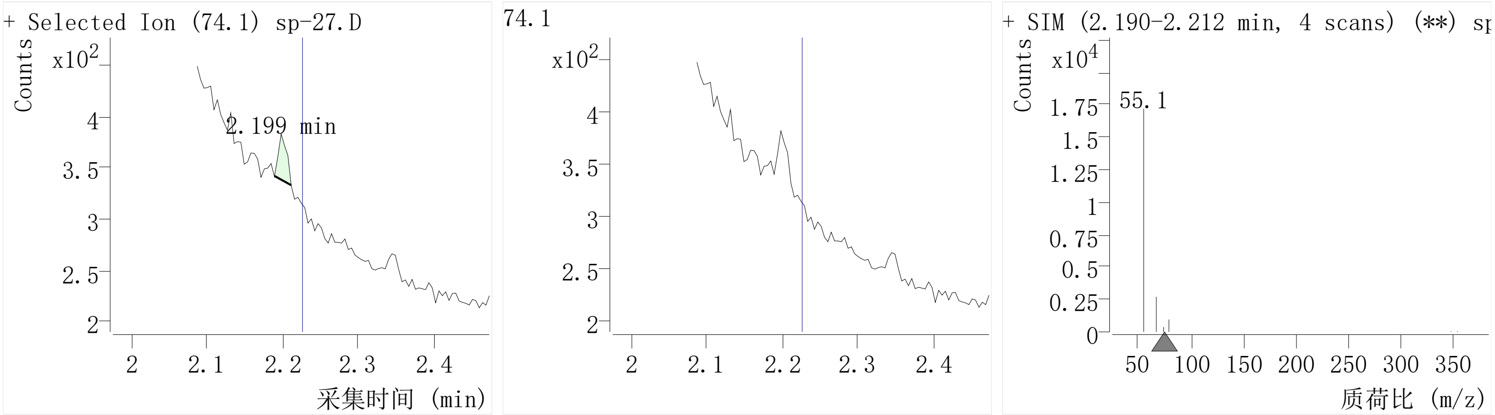

C6:0

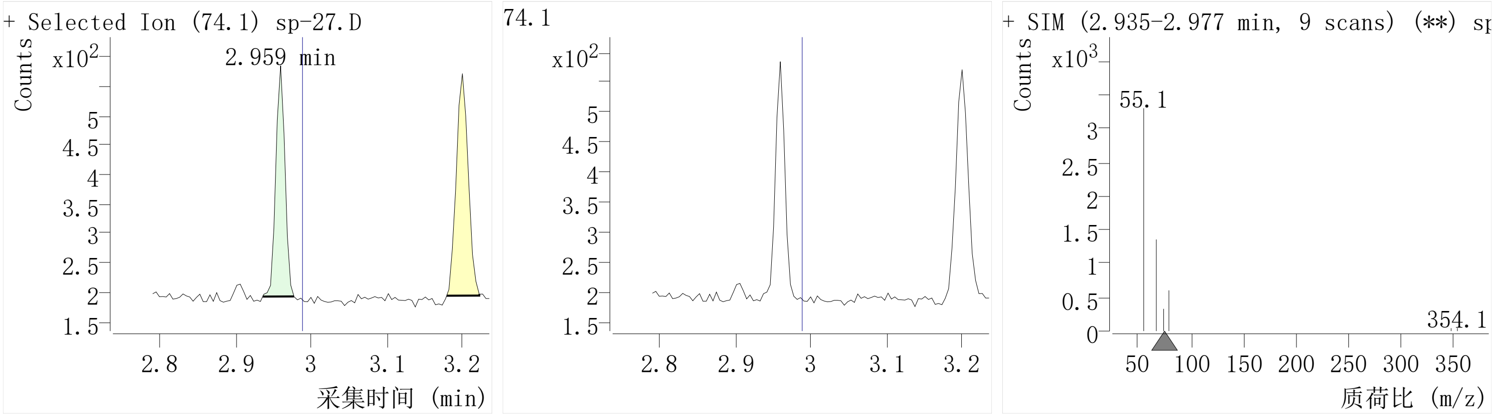

C8:0

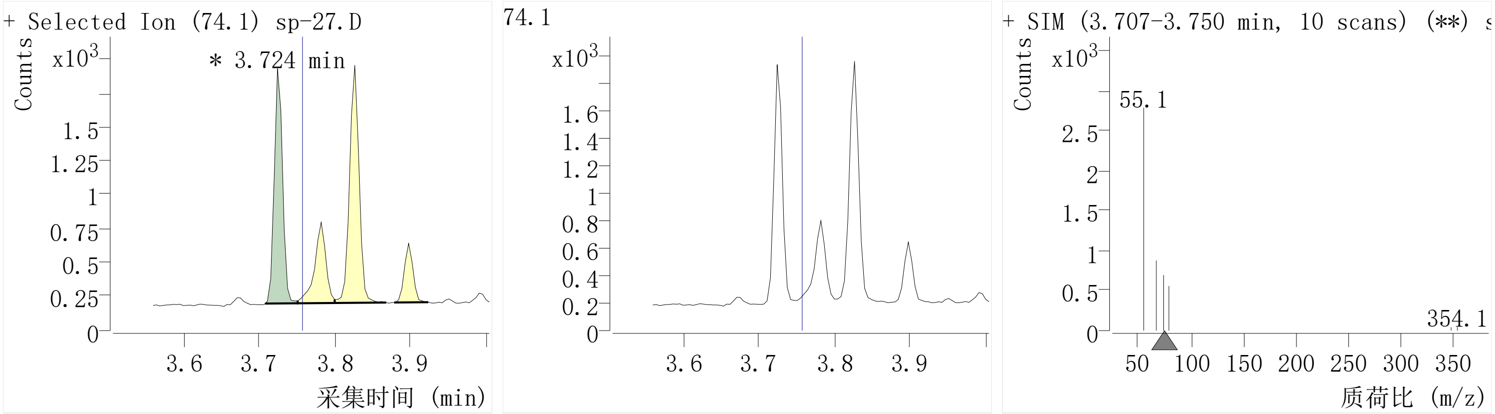

C10:0

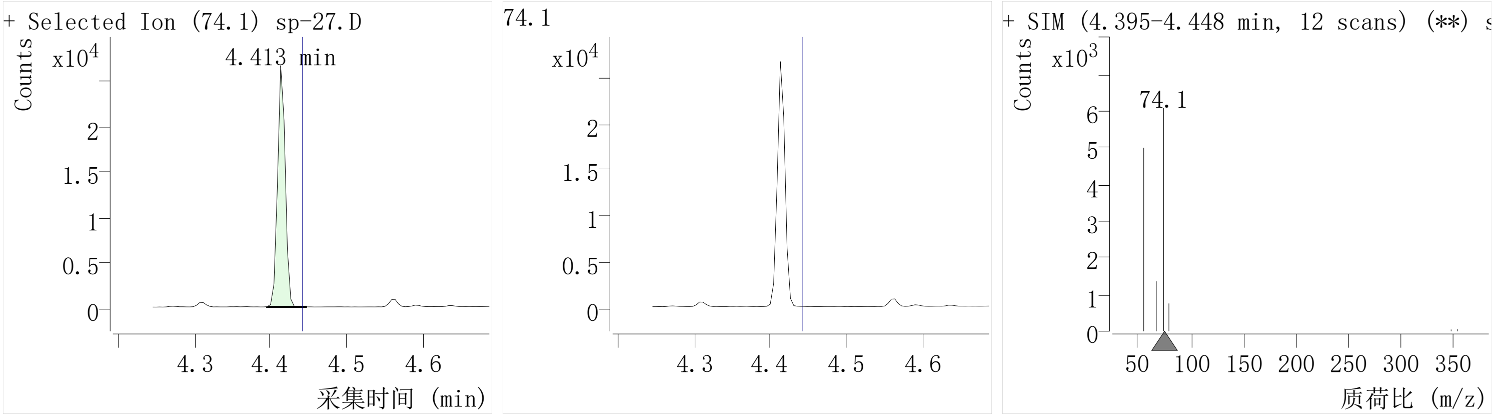

C11:0

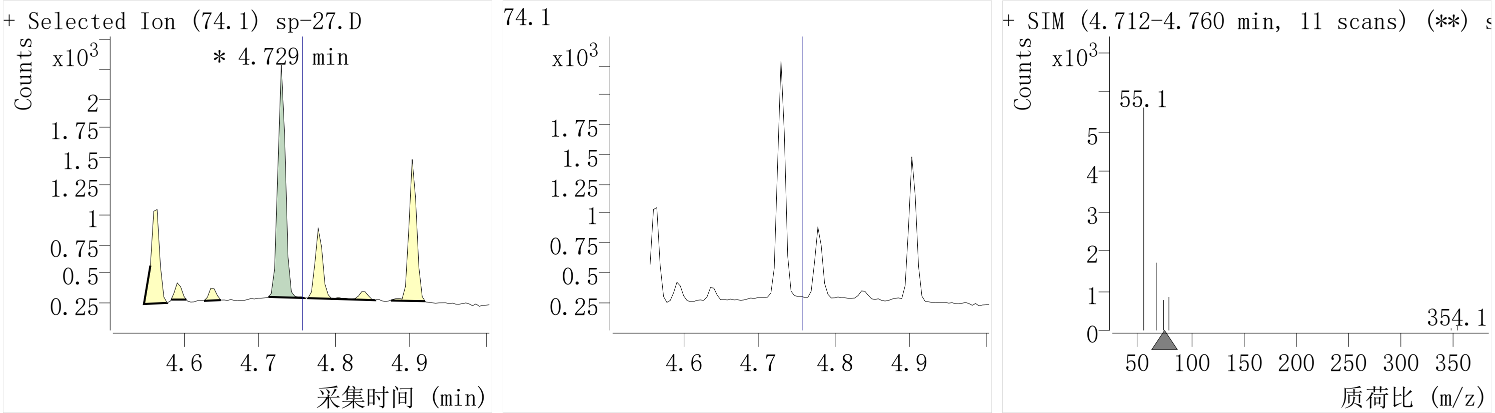

C12:0

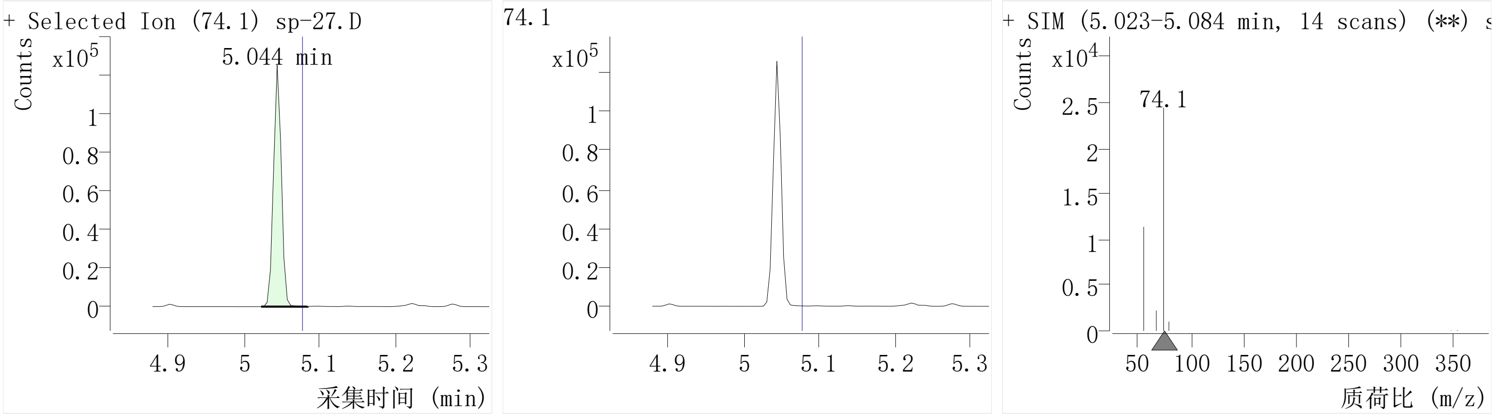

C13:0

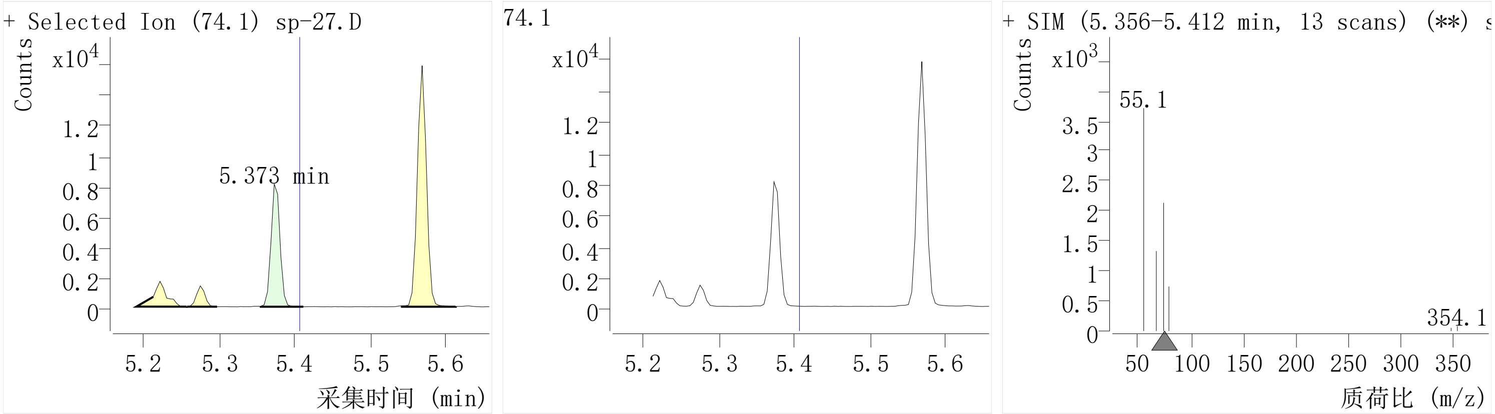

C14:0

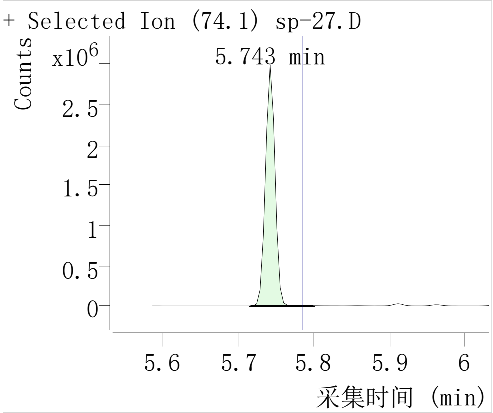

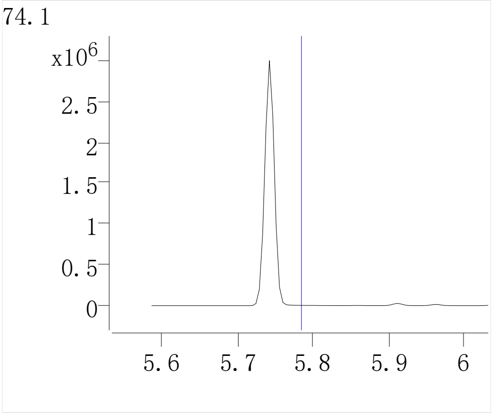

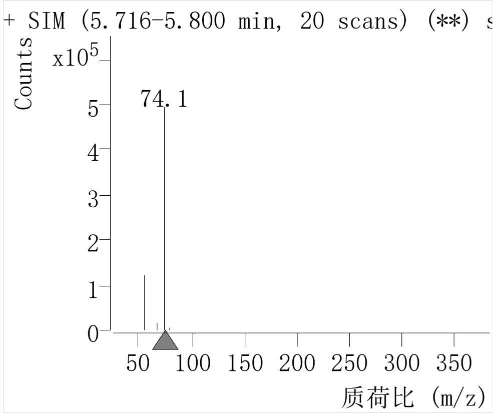

C14:1

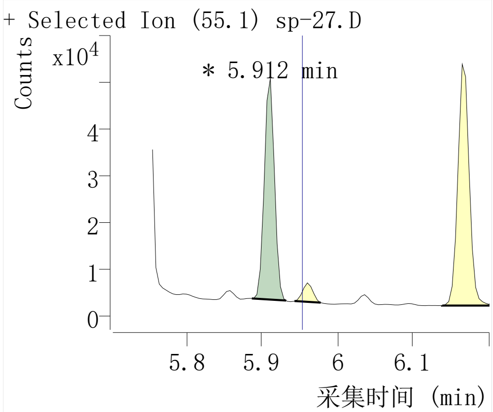

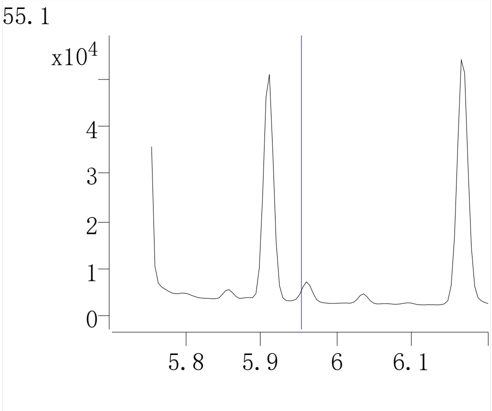

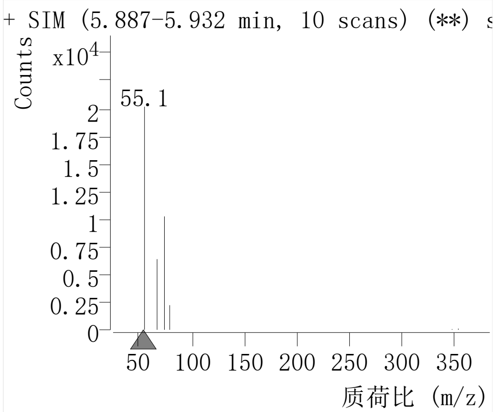

C15:0

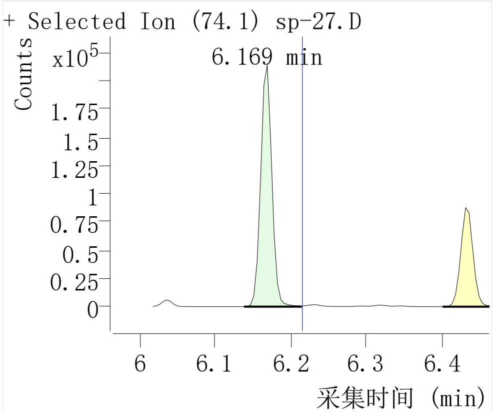

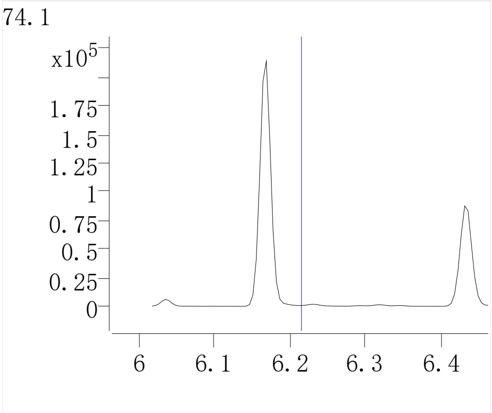

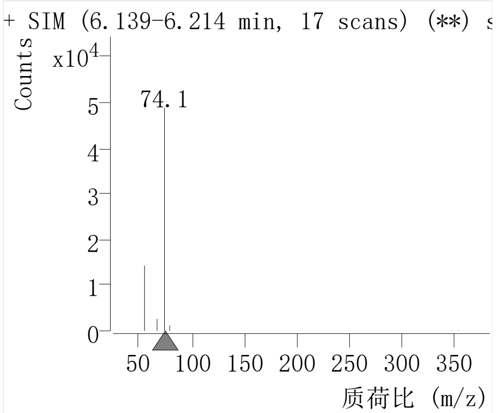

C15:1

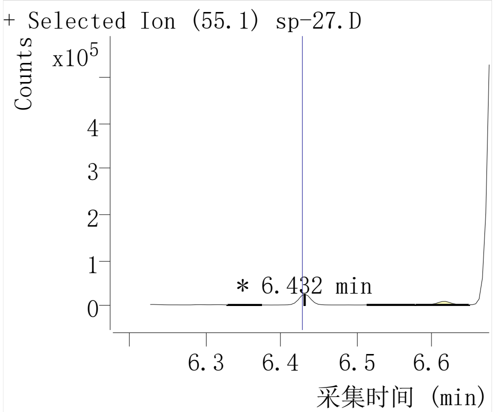

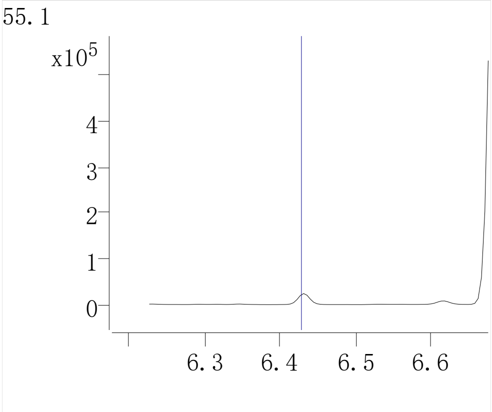

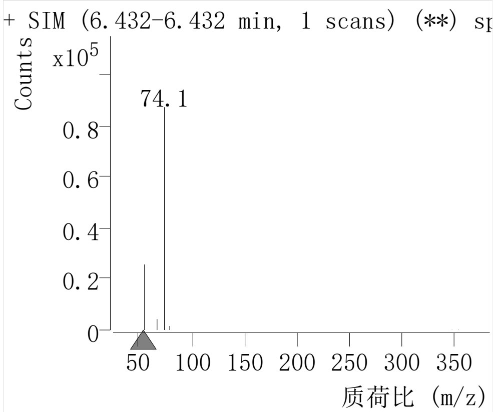

C16:0

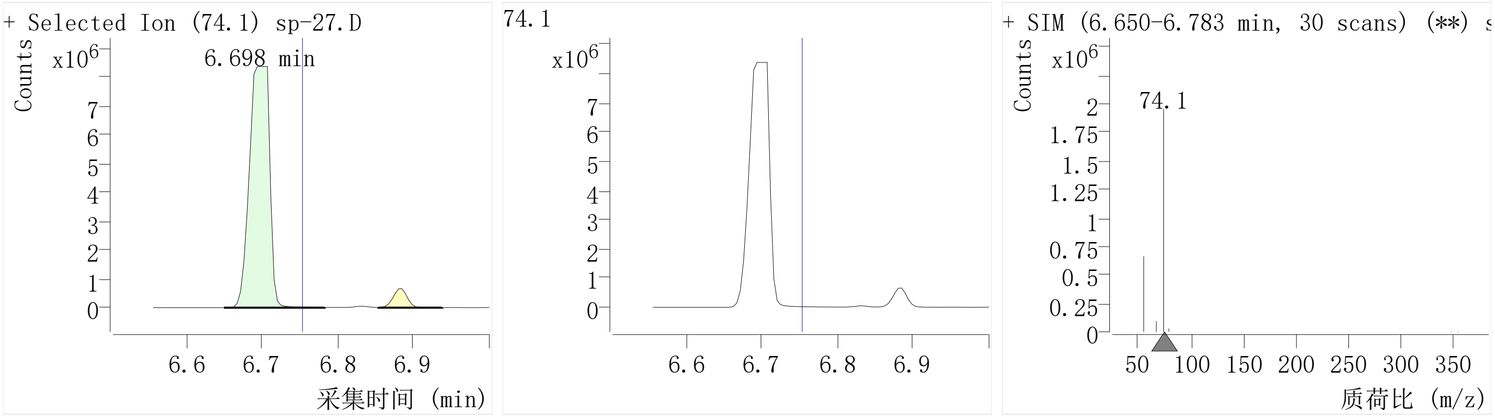

C16:1

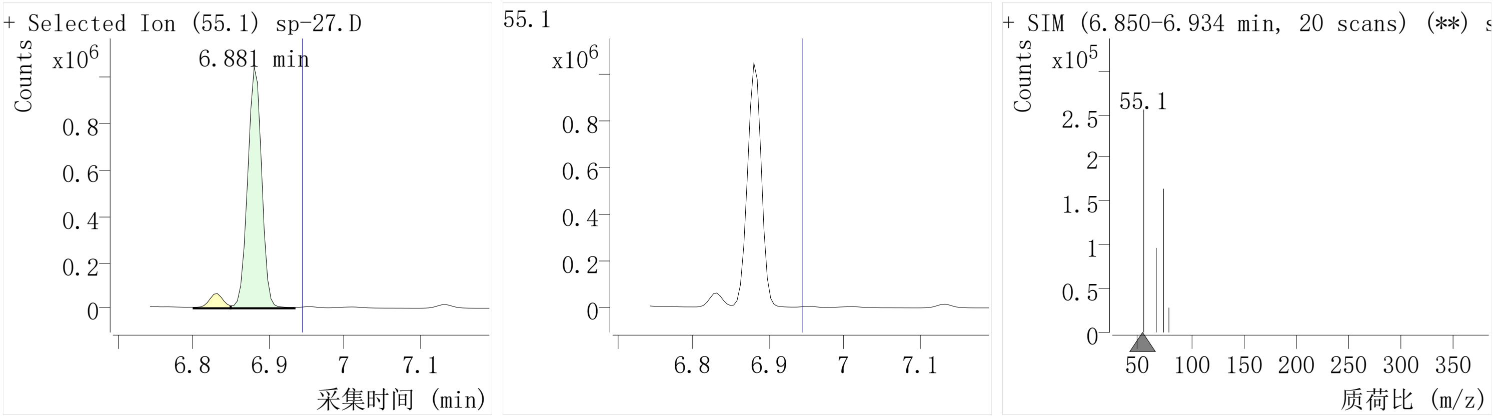

C17:0

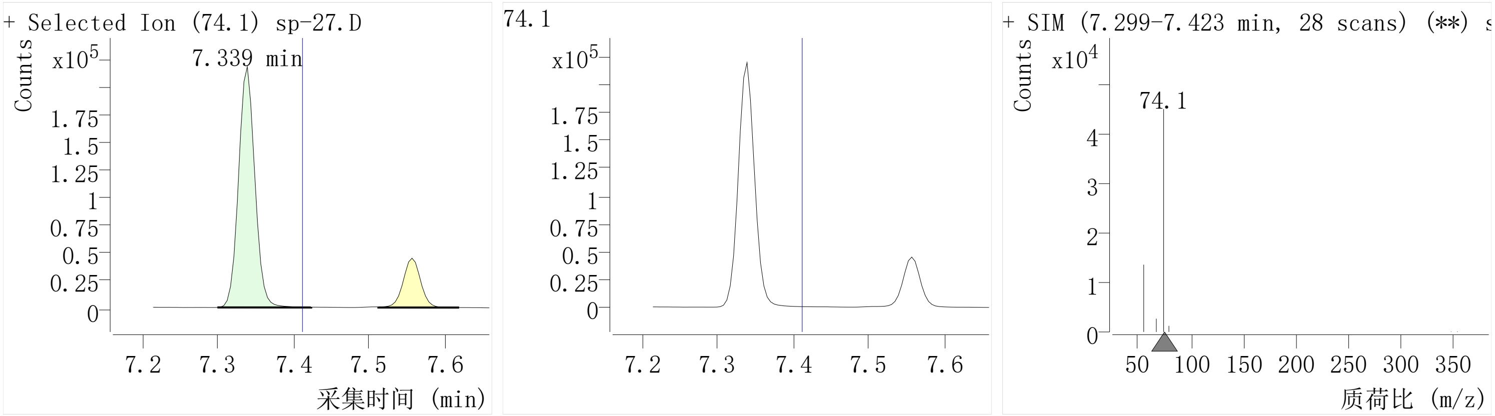

C17:1

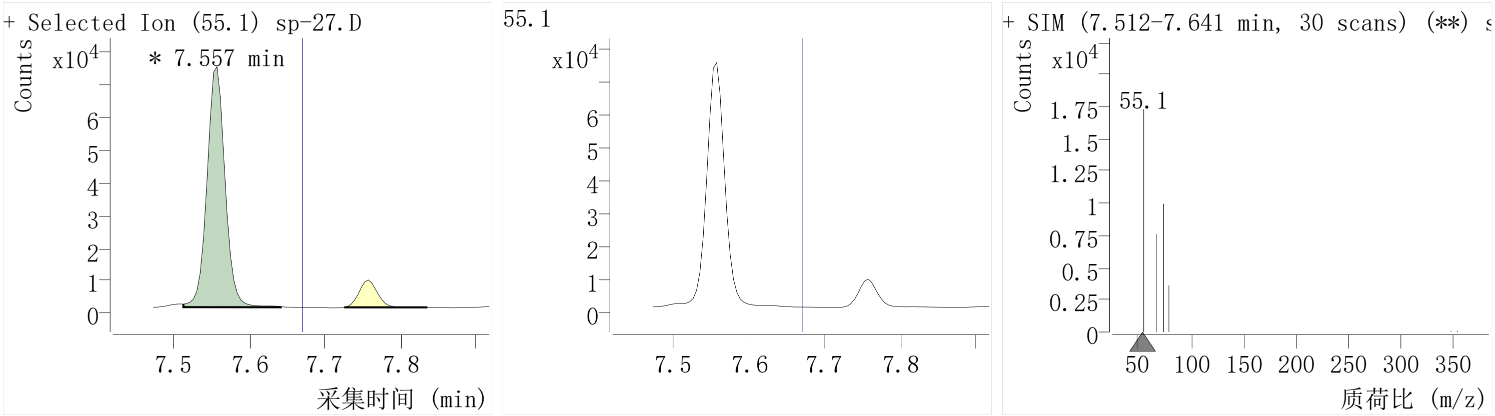

C18:0

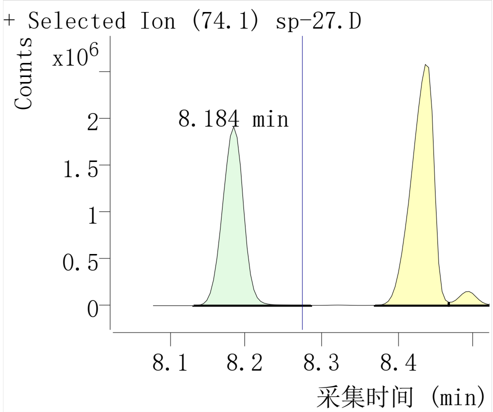

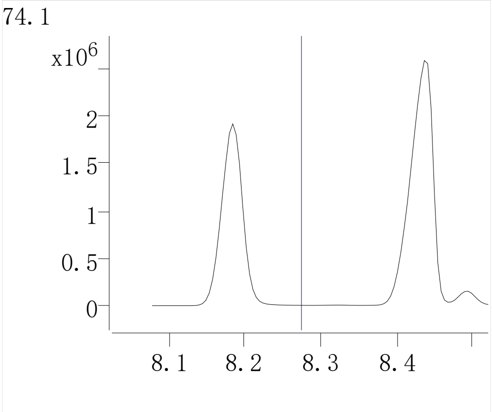

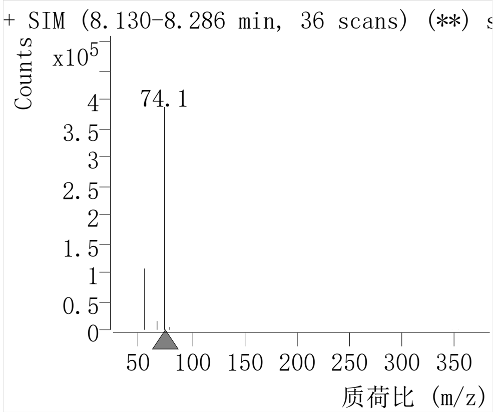

C18:1n9t

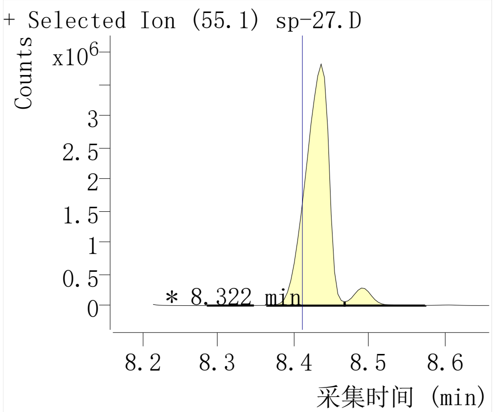

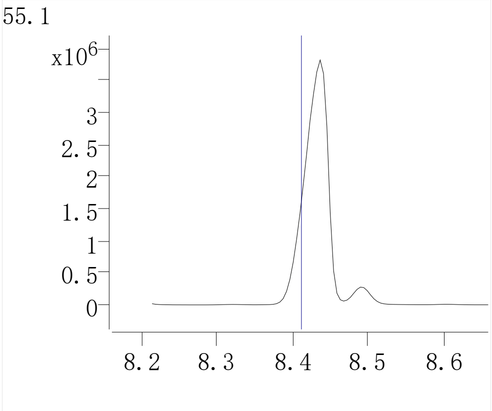

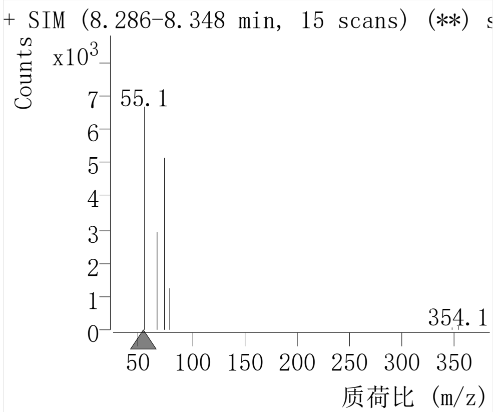

C18:1n9c

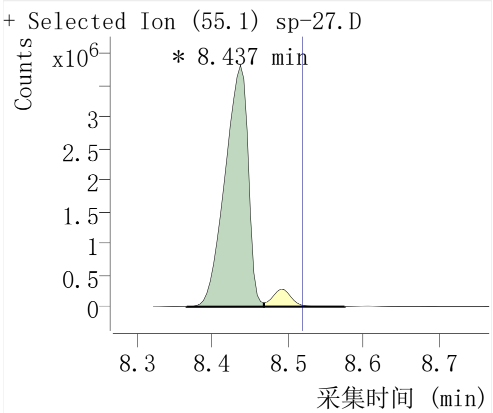

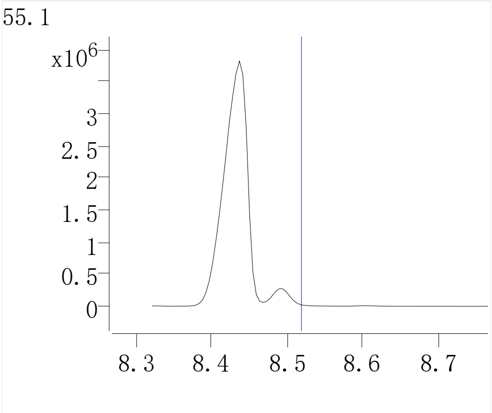

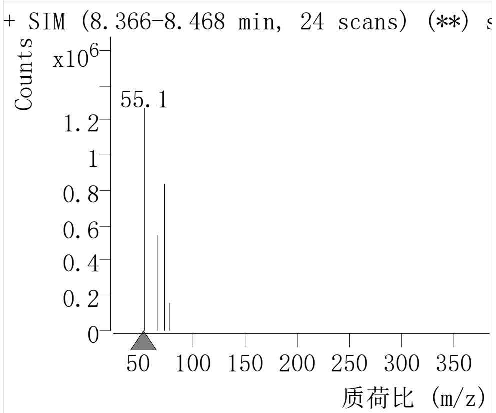

C18:2n6t

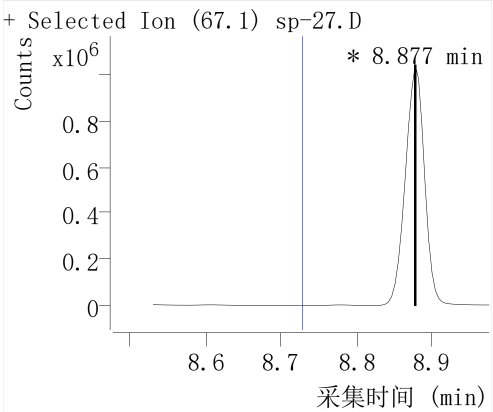

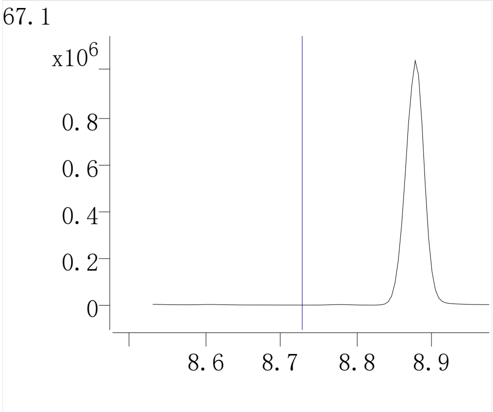

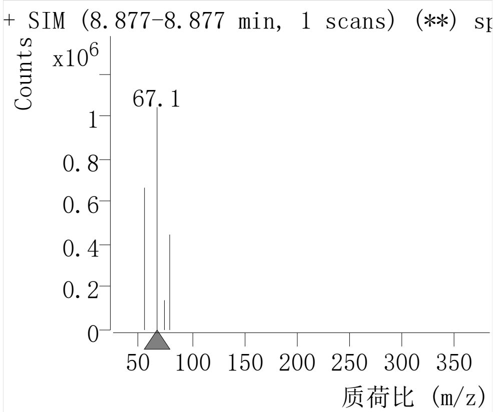

C18:2n6c

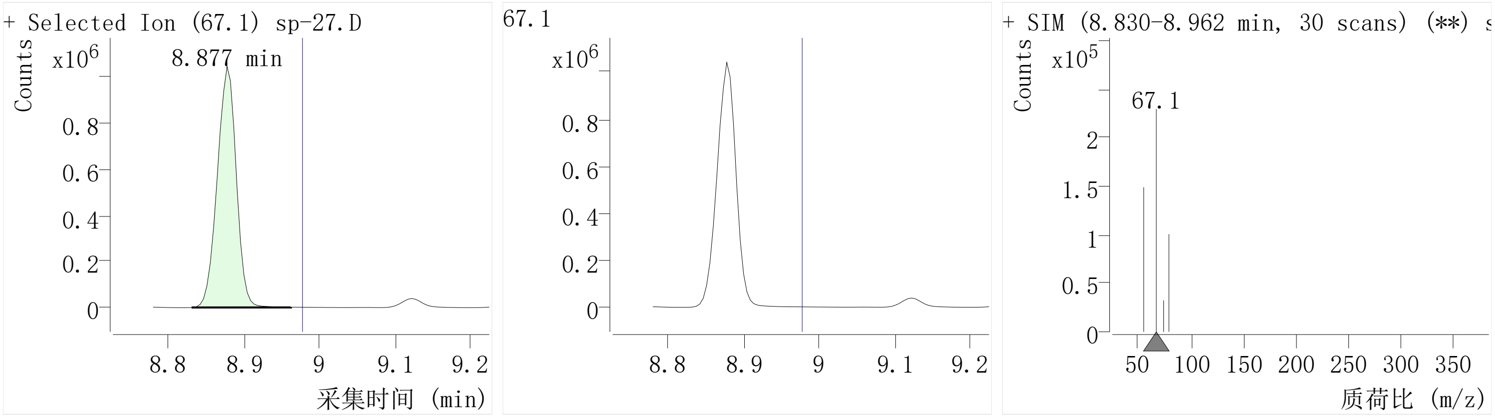

C18:3n6

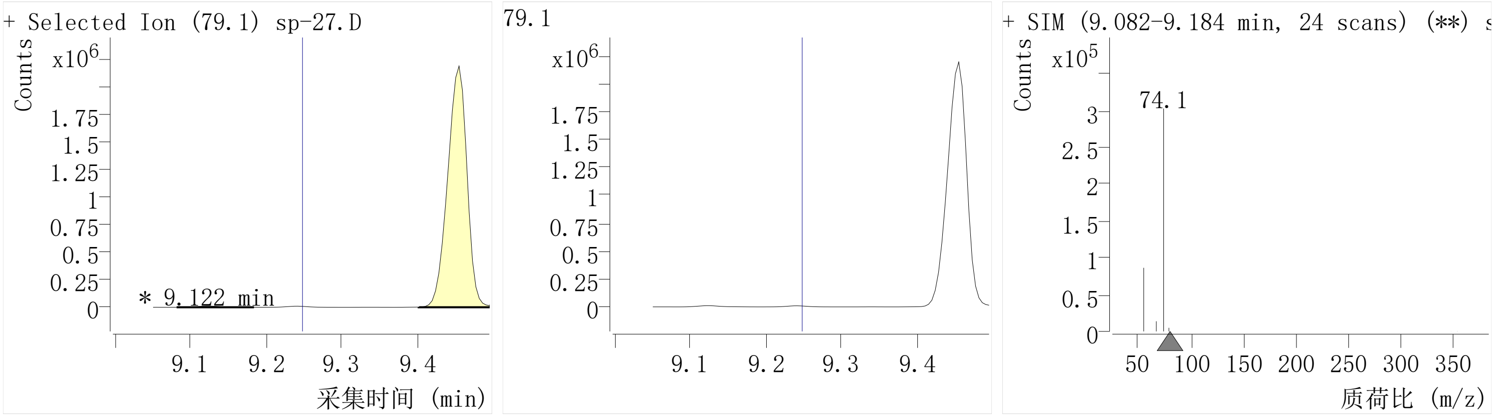

C18:3n3

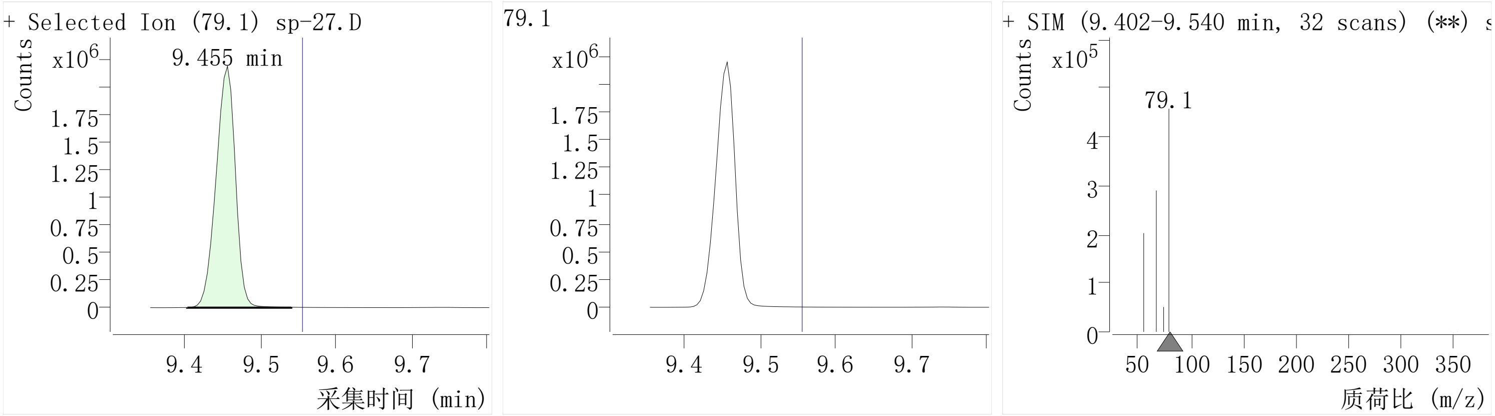

C20:0

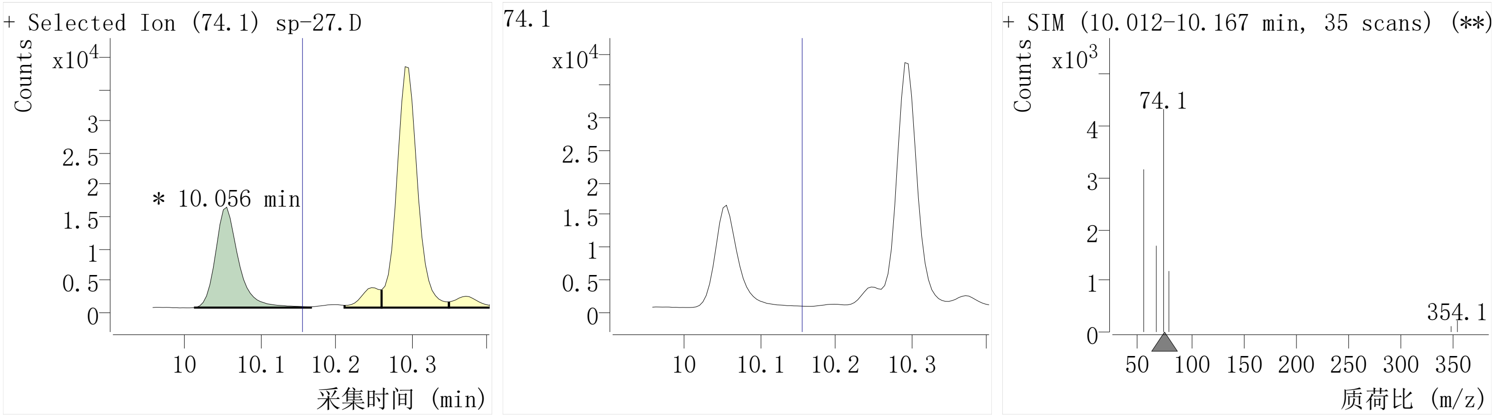

C20:1

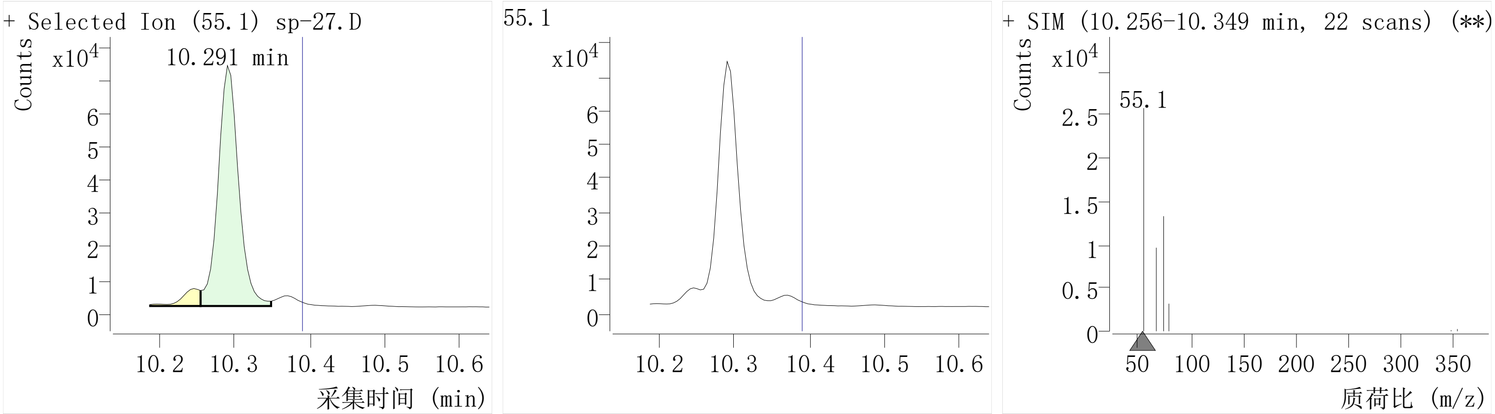

C20:2

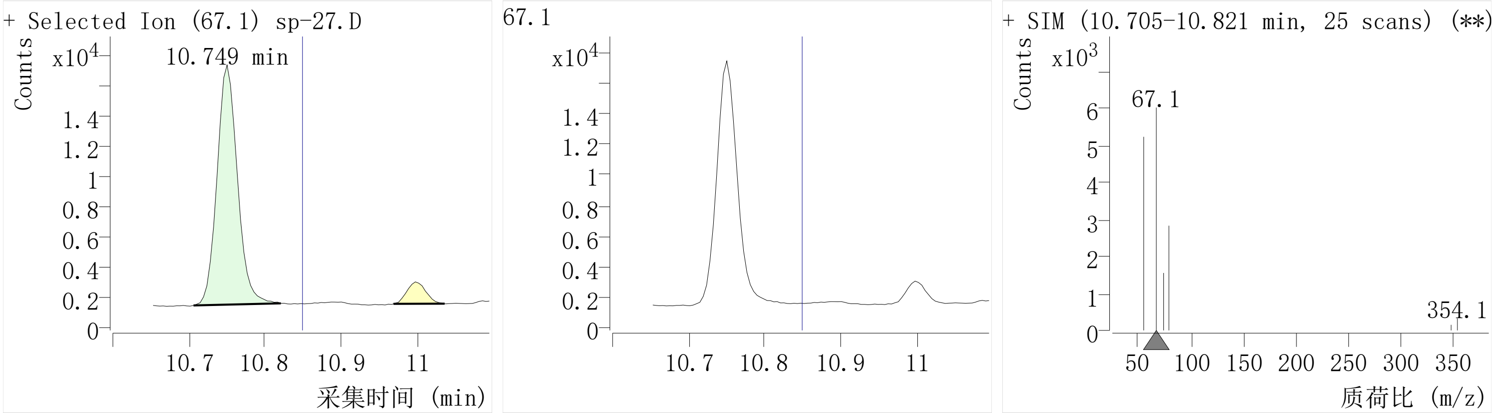

C21:0

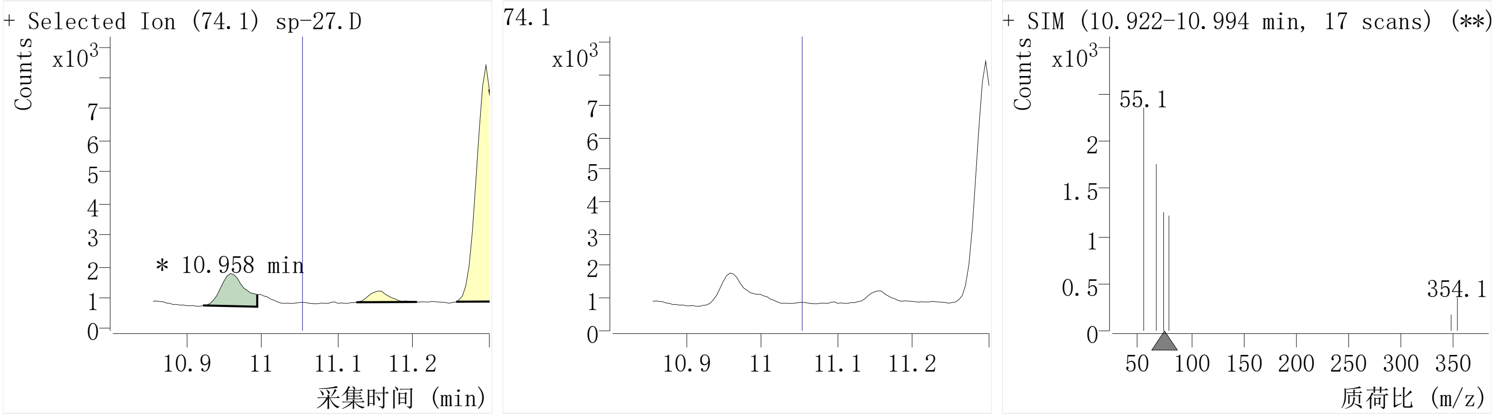

C20:3n6

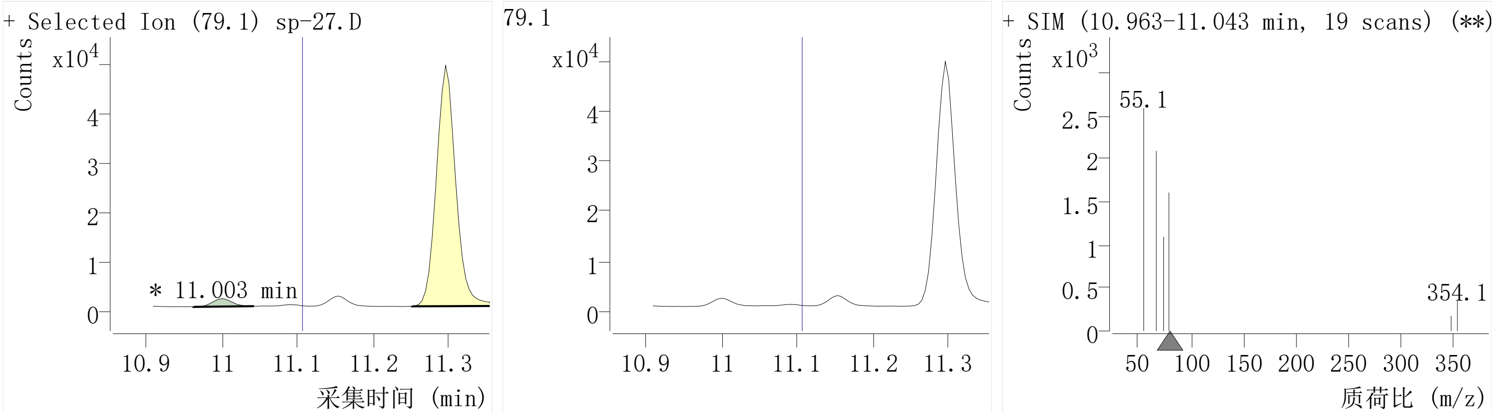

C20:4n6

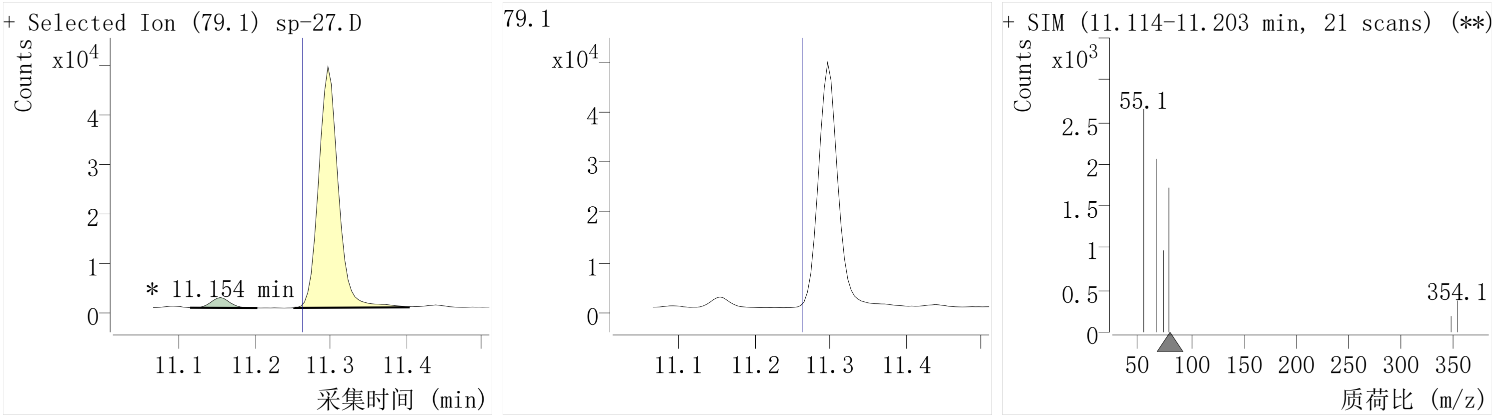

C20:3n3

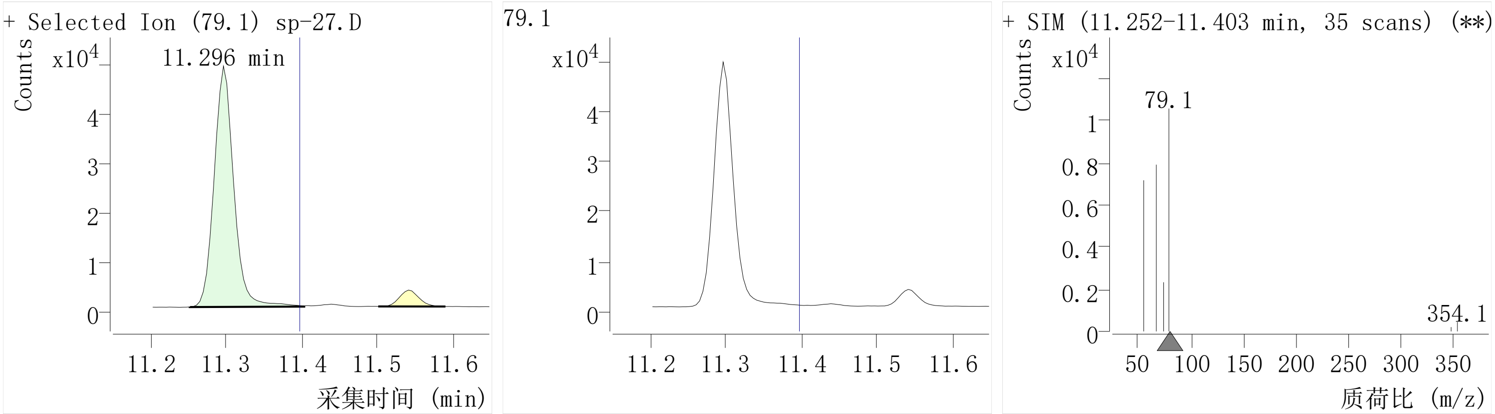

C20:5n3

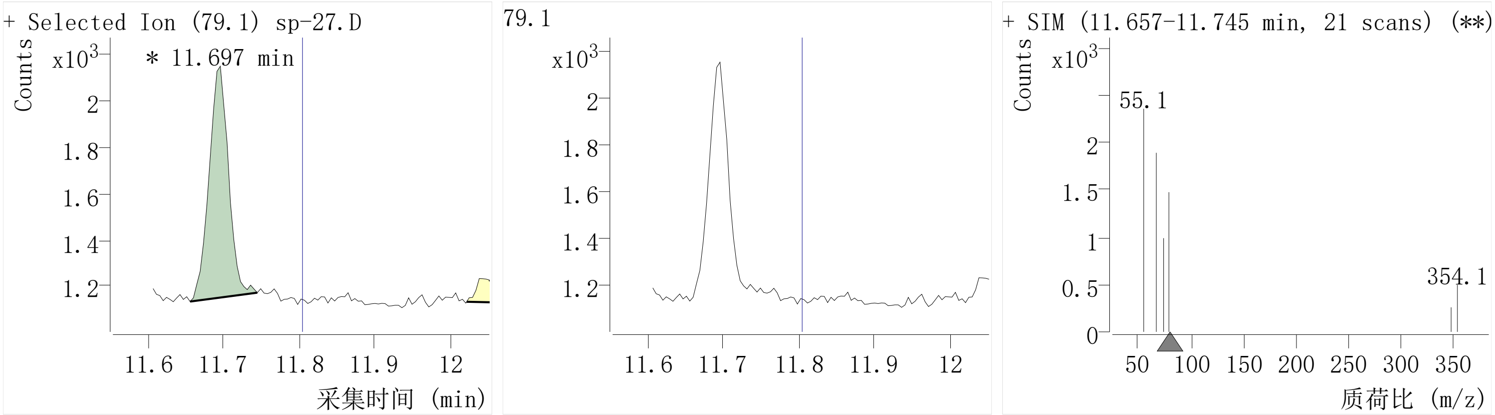

C22:0

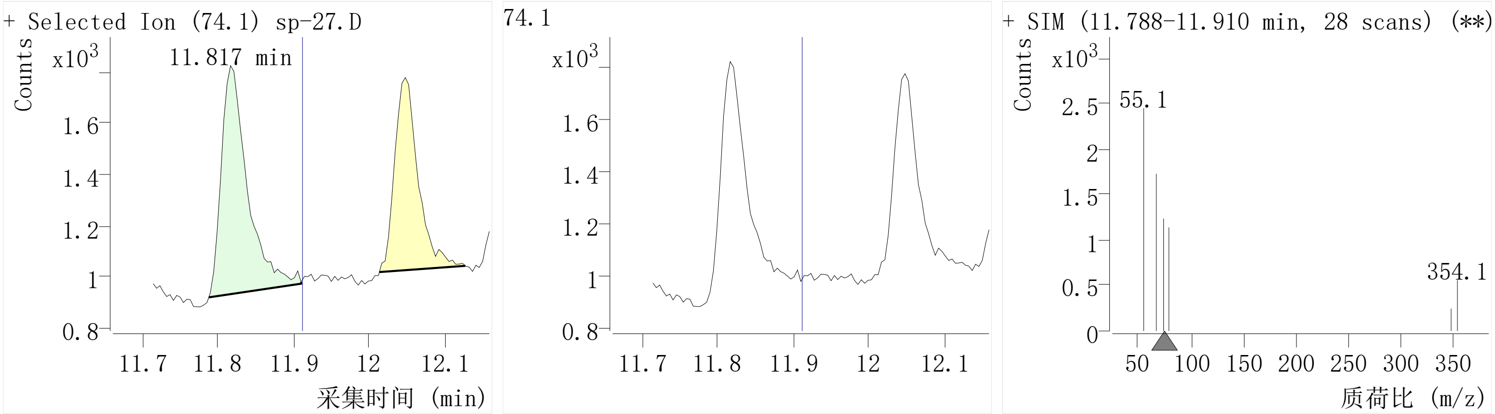

C22:1n9

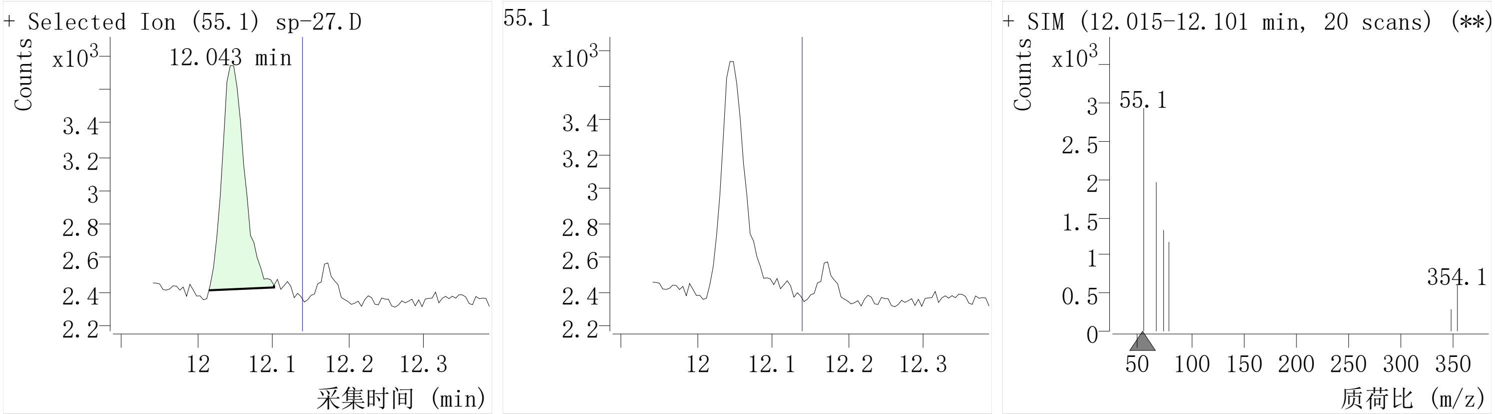

C22:2n6

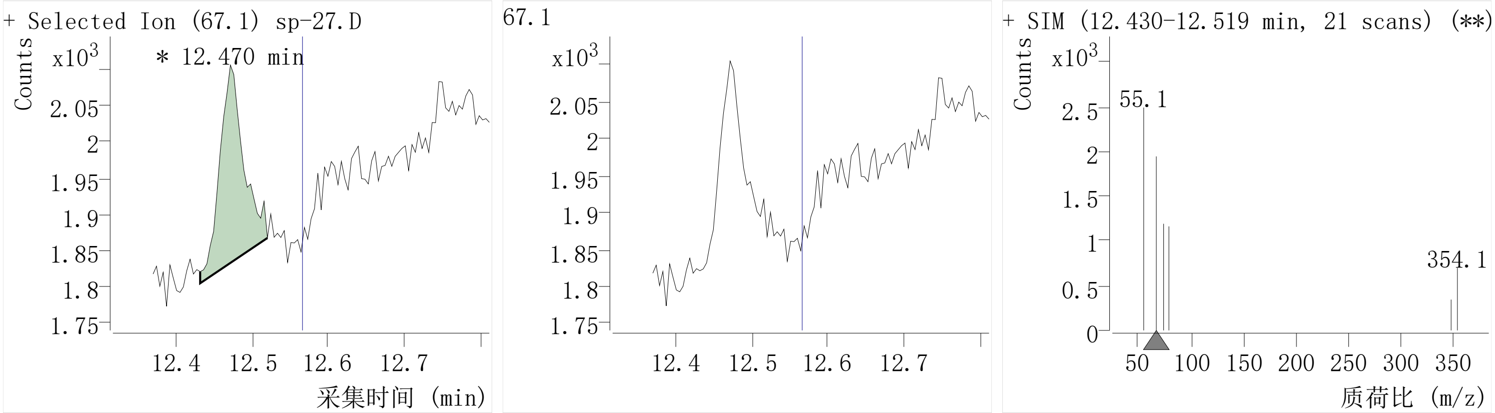

C23:0

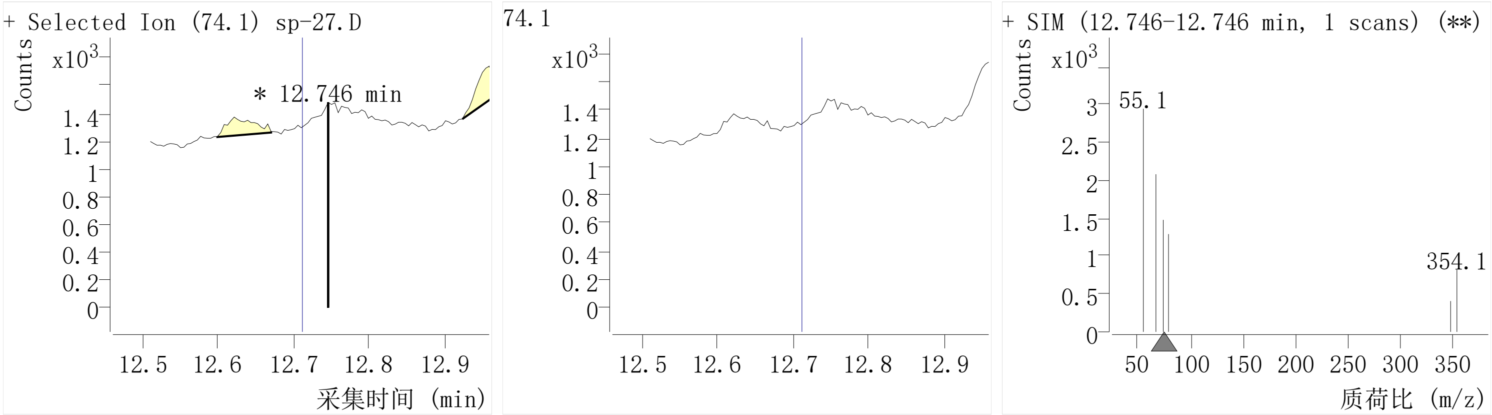

C24:0

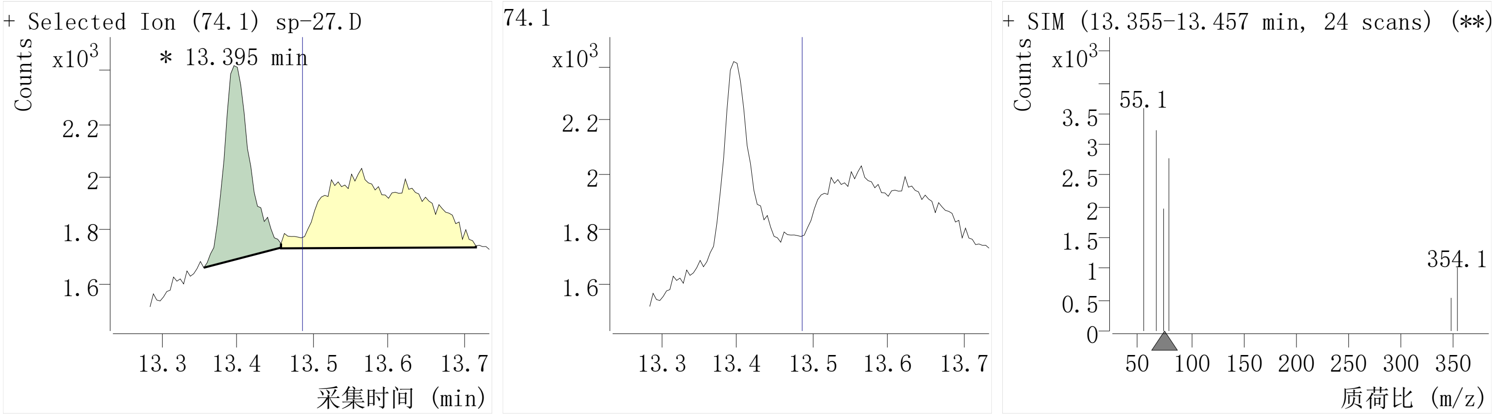

C22:6

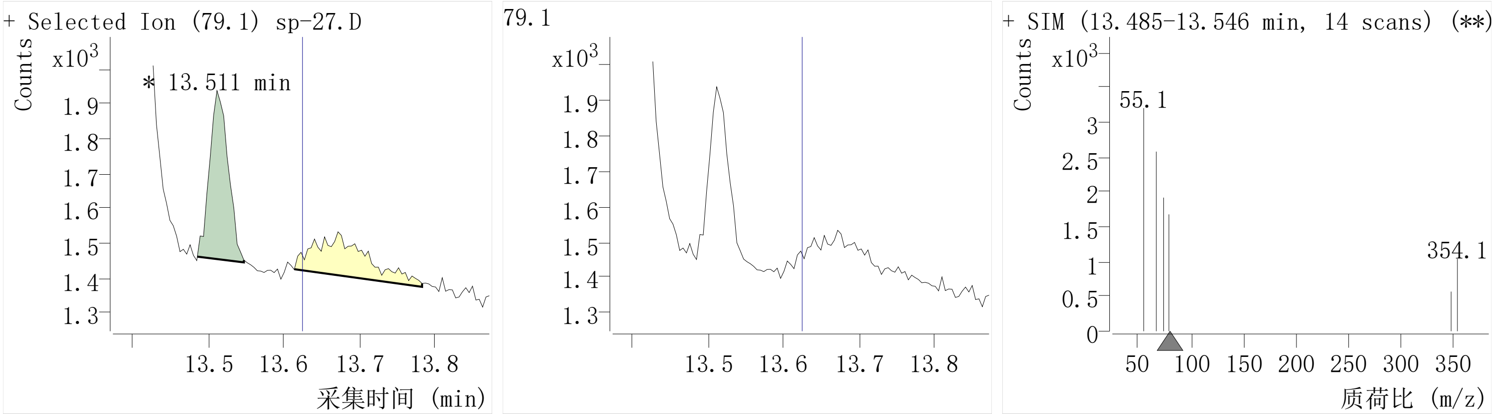
+ Selected Ion (79.1) sp-27.D
Counts
x10^3
* 13.511 min
1.9
1.8
1.7
1.6
1.5
1.4
1.3
13.5 13.6 13.7 13.8
采集时间 (min)
79.1
x10^3
1.9
1.8
1.7
1.6
1.5
1.4
1.3
13.5 13.6 13.7 13.8
+ SIM (13.485-13.546 min, 14 scans) (**)
Counts
x10^3
55.1
3
2.5
2
1.5
1
0.5
0
354.1
50 100 150 200 250 300 350
质荷比 (m/z)

C24:1

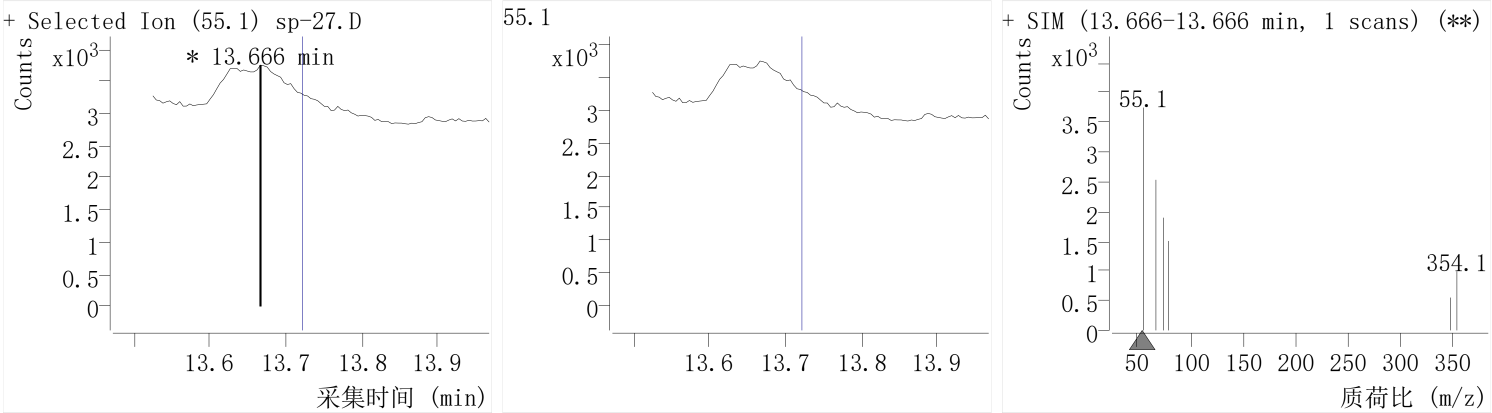
+ Selected Ion (55.1) sp-27.D
Counts
x10^3
* 13.666 min
3
2.5
2
1.5
1
0.5
0
13.6 13.7 13.8 13.9
采集时间 (min)
55.1
x10^3
3
2.5
2
1.5
1
0.5
0
13.6 13.7 13.8 13.9
+ SIM (13.666-13.666 min, 1 scans) (**)
Counts
x10^3
55.1
3.5
3
2.5
2
1.5
1
0.5
0
354.1
50 100 150 200 250 300 350
质荷比 (m/z)

定量分析完成报告

**批处理路径**	G:\GC-MS\HX250430-4-GCMS总脂肪酸靶向检测\HX250430-4\QuantResults\HX250430-4.batch.bin		
**分析时间**	2025/5/14 16:58	**分析员姓名**	DESKTOP-M3A0GPO\omics
**报告时间**	2025/5/16 14:53:32	**报告员姓名**	DESKTOP-M3A0GPO\omics
**最近校正更新**	2025/5/14 16:58	**批处理状态**	已处理
**定量批处理版本**	10.2	**定量报告版本**	10.2

**采集时间**	2025/5/9 7:57	**数据文件**	sp-28.D
**样品类型**	样品	**样品名称**	sp-28
**稀释**	1	**采集方法**	脂肪酸

样品色谱图

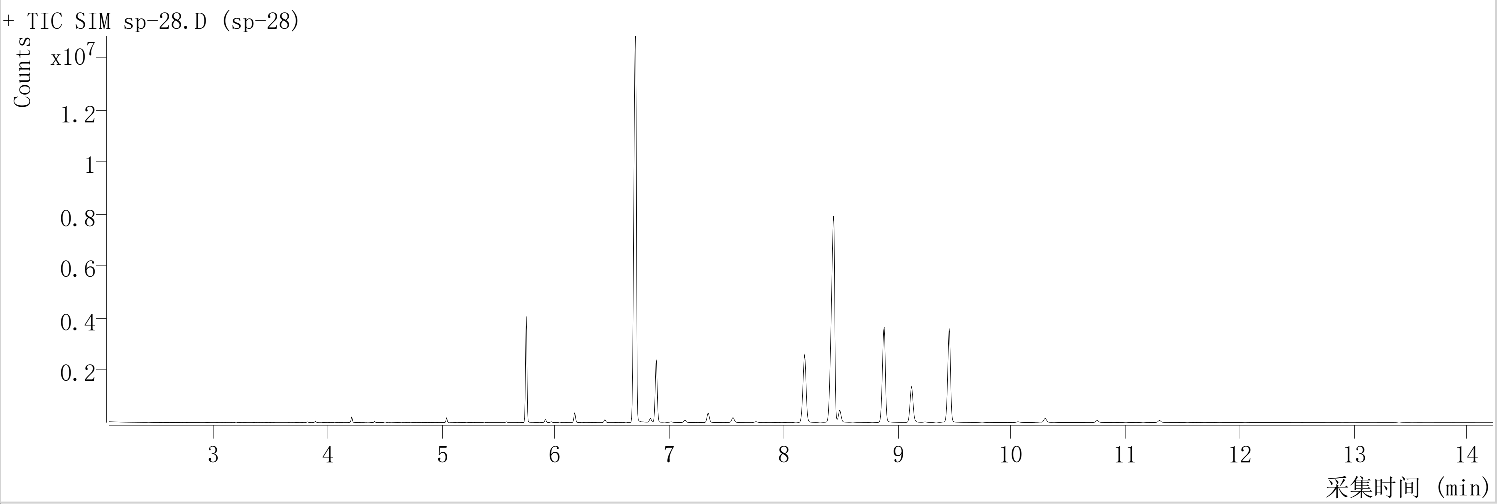

化合物	ISTD	RT	响应	ISTD 响应	响应比	最终浓度	单位
C4:0	C19:0	2.203	51	1948544	0.0000	ND	ug/ml
C6:0	C19:0	2.959	528	1948544	0.0003	0.0026	ug/ml
C8:0	C19:0	3.724	1948	1948544	0.0010	0.0062	ug/ml
C10:0	C19:0	4.413	22503	1948544	0.0115	0.1648	ug/ml
C11:0	C19:0	4.729	2044	1948544	0.0010	0.0093	ug/ml
C12:0	C19:0	5.044	101737	1948544	0.0522	0.8053	ug/ml
C13:0	C19:0	5.378	9142	1948544	0.0047	0.0538	ug/ml
C14:0	C19:0	5.743	2838545	1948544	1.4568	32.8837	ug/ml
C14:1	C19:0	5.912	54850	1948544	0.0281	1.3130	ug/ml
C15:0	C19:0	6.169	295228	1948544	0.1515	2.7786	ug/ml
C15:1	C19:0	6.423	0	1948544	0.0000	ND	ug/ml
C16:0	C19:0	6.698	15768732	1948544	8.0926	323.1574	ug/ml
C16:1	C19:0	6.881	1419469	1948544	0.7285	44.4297	ug/ml
C17:0	C19:0	7.339	414948	1948544	0.2130	4.4428	ug/ml
C17:1	C19:0	7.557	147672	1948544	0.0758	4.0528	ug/ml
C18:0	C19:0	8.184	3822693	1948544	1.9618	42.9275	ug/ml
C18:1n9t	C19:0	8.322	8493	1948544	0.0044	0.2571	ug/ml
C18:1n9c	C19:0	8.437	7518298	1948544	3.8584	260.0917	ug/ml
C18:2n6t	C19:0	8.882	0	1948544	0.0000	ND	ug/ml
C18:2n6c	C19:0	8.882	3012368	1948544	1.5460	123.1250	ug/ml
C18:3n6	C19:0	9.122	23459	1948544	0.0120	0.0296	ug/ml
C18:3n3	C19:0	9.451	2844212	1948544	1.4597	72.5656	ug/ml
C20:0	C19:0	10.056	46732	1948544	0.0240	0.6389	ug/ml
C20:1	C19:0	10.292	149551	1948544	0.0768	4.7696	ug/ml
C20:2	C19:0	10.750	60892	1948544	0.0313	1.9644	ug/ml
C21:0	C19:0	10.959	2435	1948544	0.0012	0.0346	ug/ml
C20:3n6	C19:0	10.999	4691	1948544	0.0024	0.1963	ug/ml
C20:4n6	C19:0	11.154	6793	1948544	0.0035	0.2526	ug/ml
C20:3n3	C19:0	11.296	60467	1948544	0.0310	1.6734	ug/ml
C20:5n3	C19:0	11.692	1758	1948544	0.0009	0.0851	ug/ml

化合物	ISTD	RT	响应	ISTD 响应	响应比	最终浓度	单位
C22:0	C19:0	11.821	3822	1948544	0.0020	0.0838	ug/ml
C22:1n9	C19:0	12.048	3902	1948544	0.0020	0.1335	ug/ml
C22:2n6	C19:0	12.470	750	1948544	0.0004	0.0674	ug/ml
C23:0	C19:0	12.751	0	1948544	0.0000	ND	ug/ml
C24:0	C19:0	13.395	2850	1948544	0.0015	0.0723	ug/ml
C22:6	C19:0	13.515	1730	1948544	0.0009	0.0708	ug/ml
C24:1	C19:0	13.667	0	1948544	0.0000	ND	ug/ml

C4:0

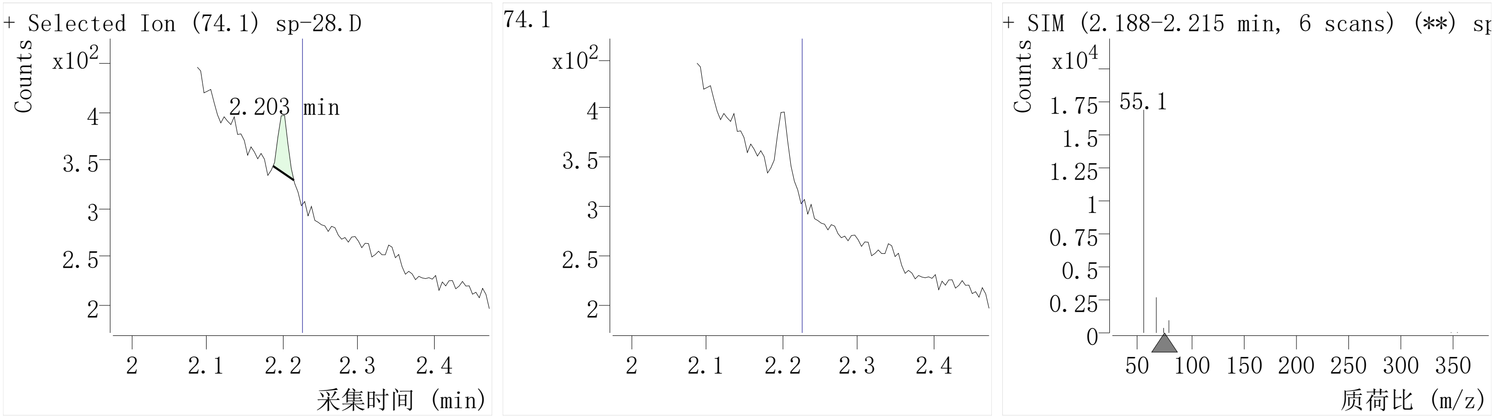

C6:0

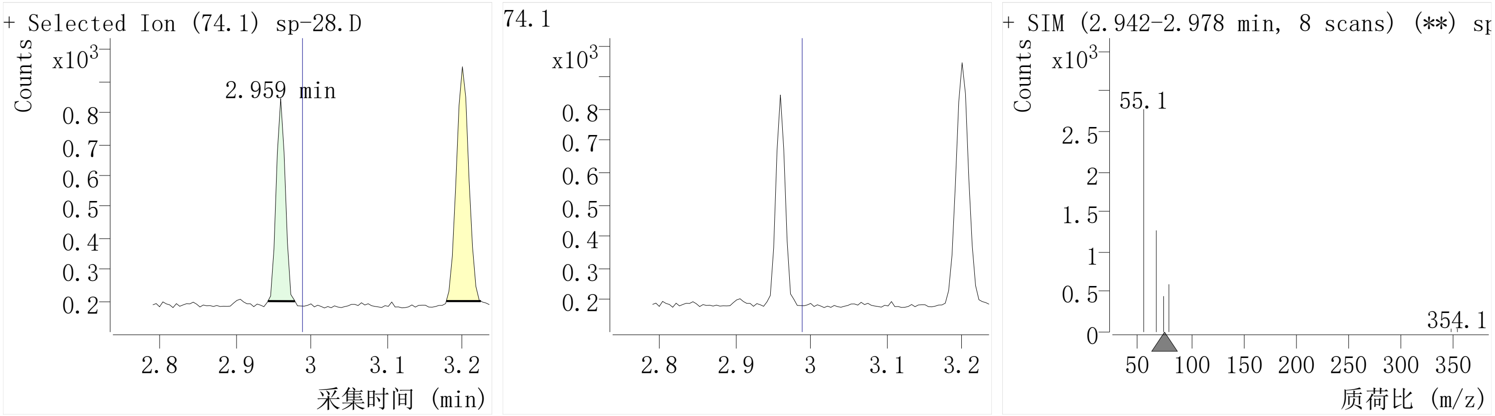

C8:0

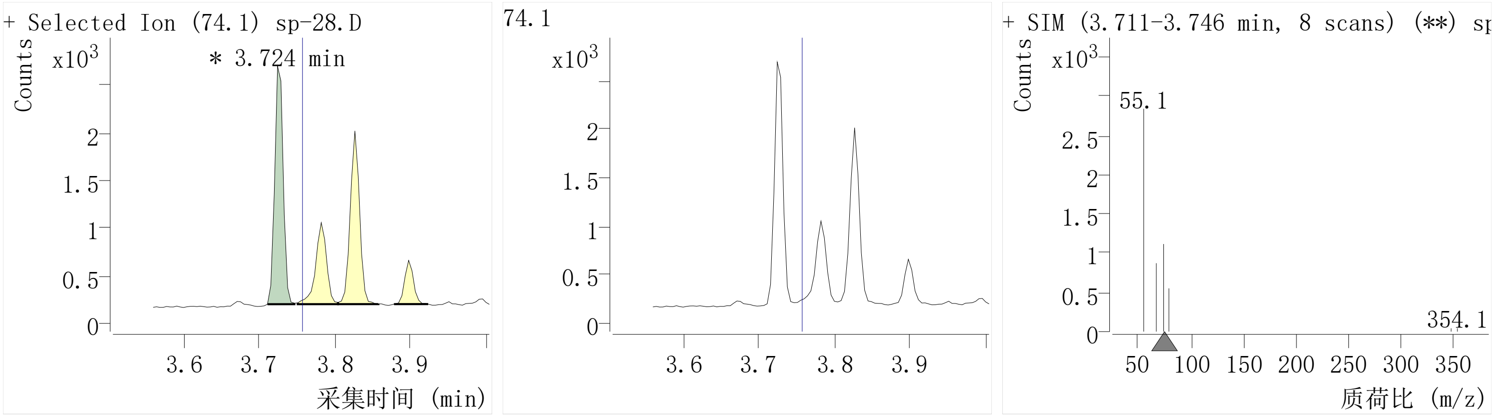

 生成时间 2025/5/16 14:52

C10:0

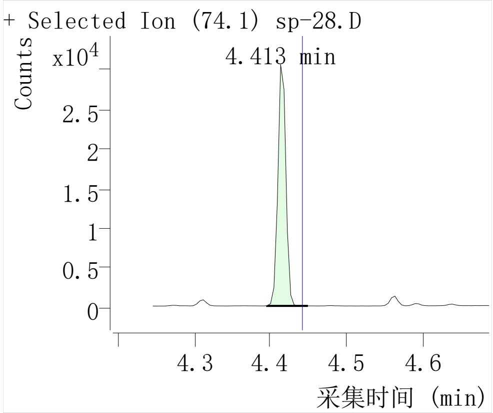

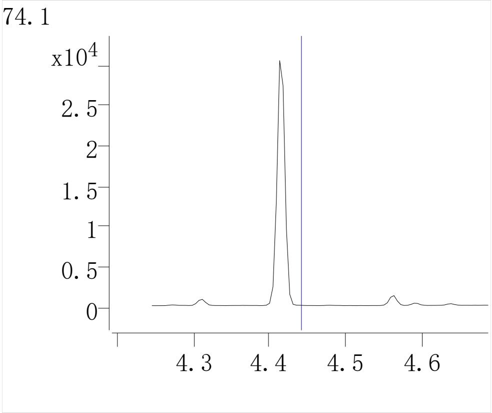

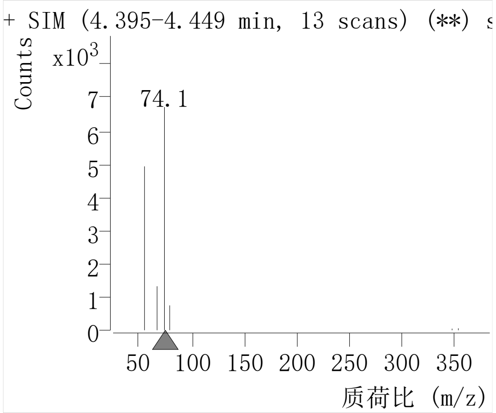

C11:0

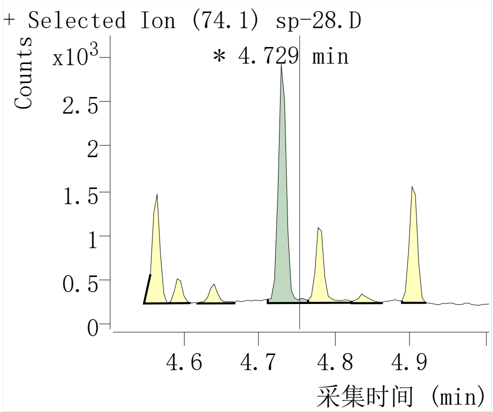

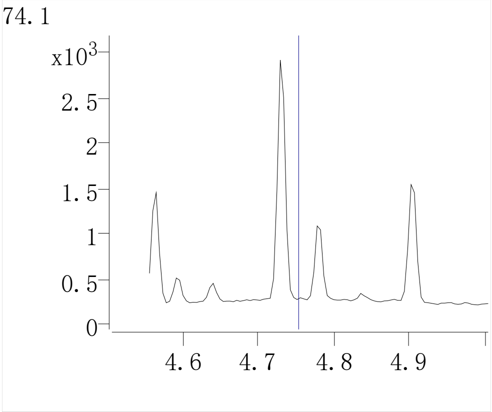

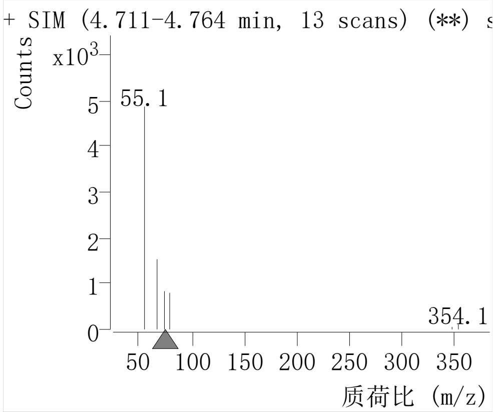

C12:0

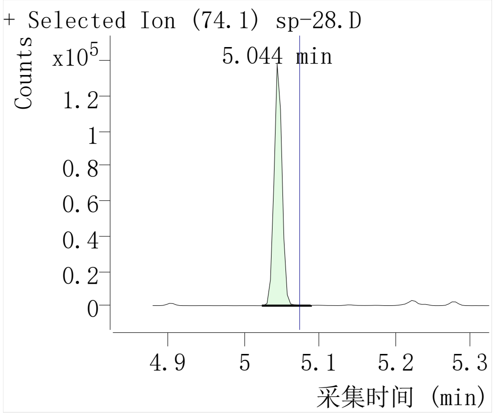

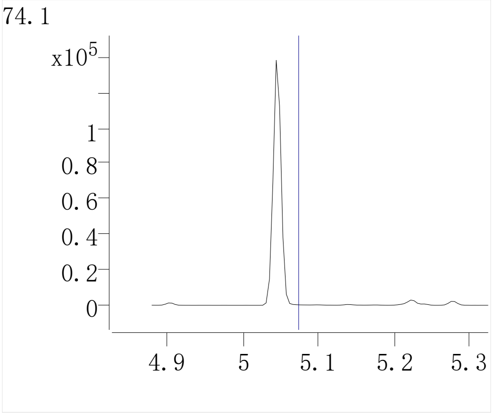

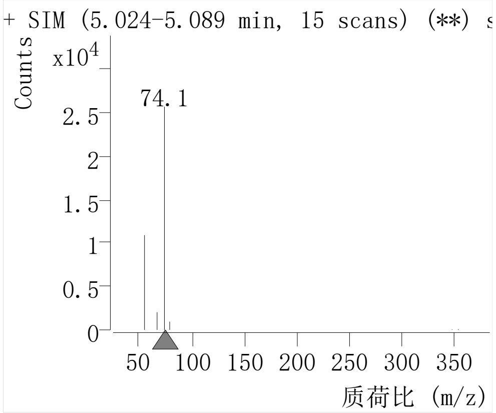

C13:0

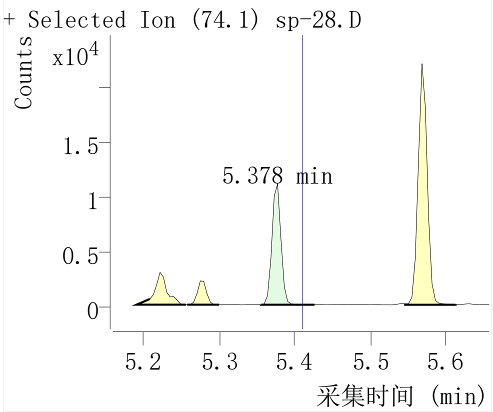

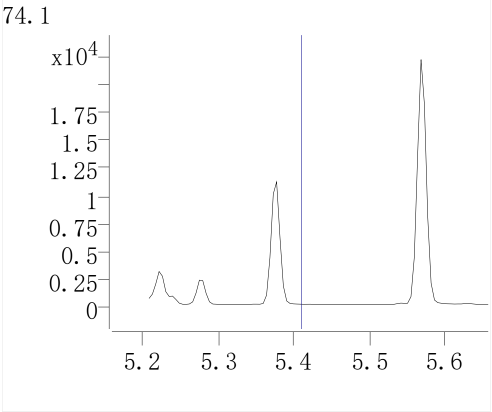

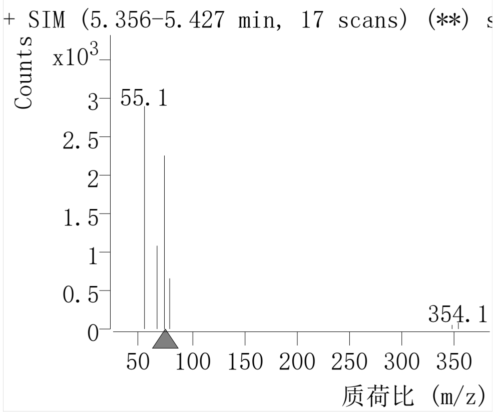

C14:0

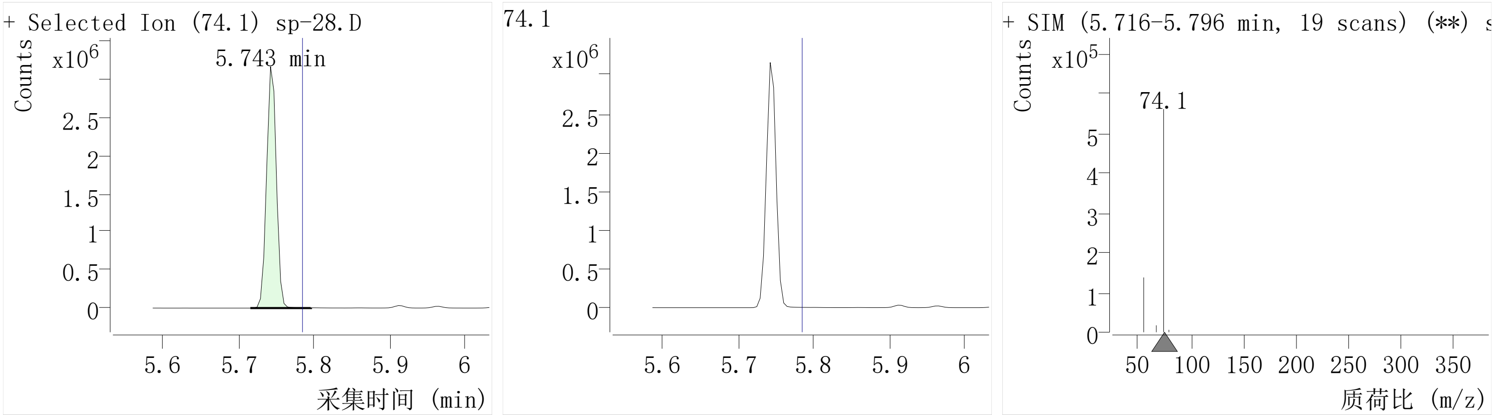

C14:1

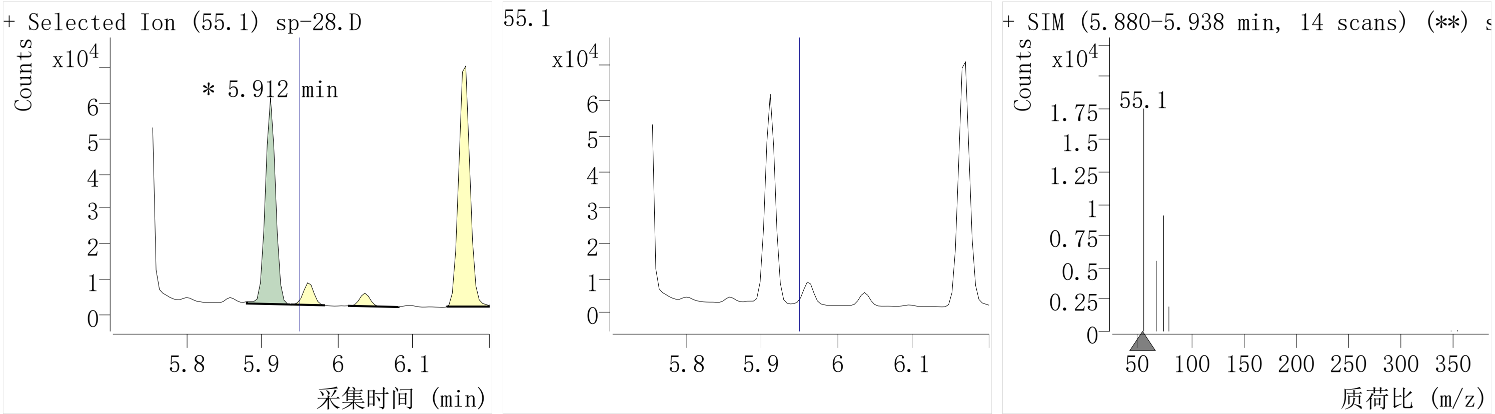

C15:0

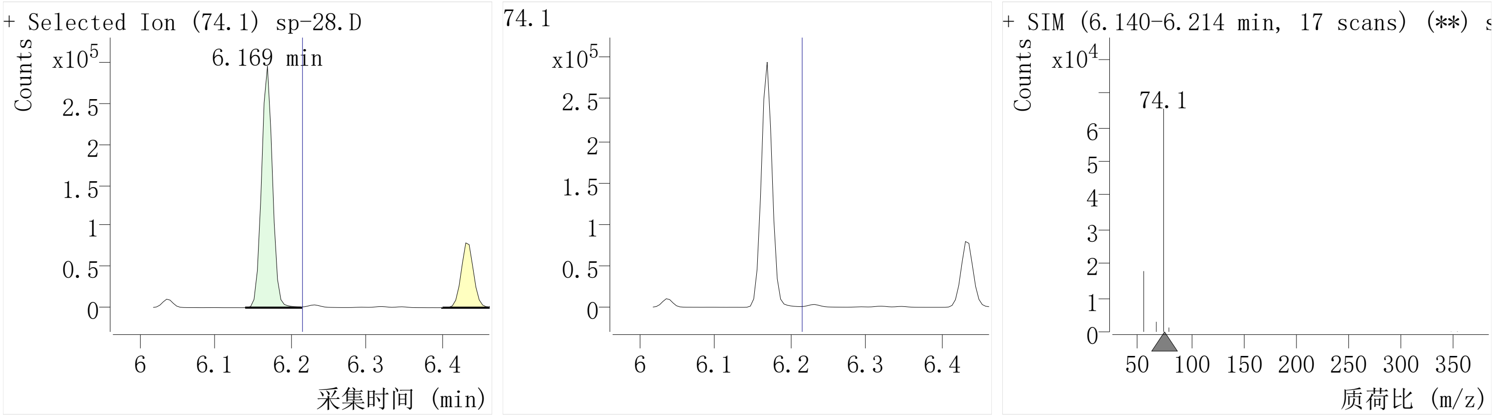

C15:1

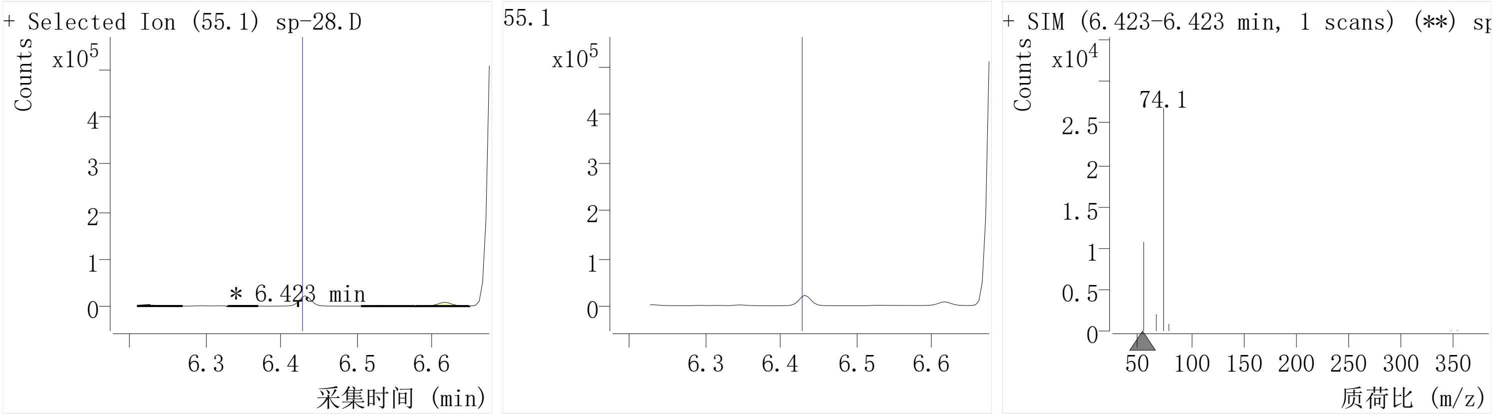

C16:0

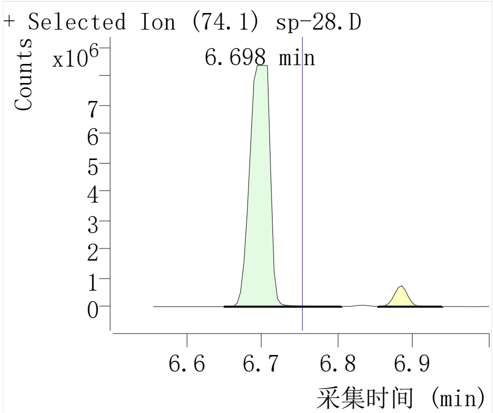

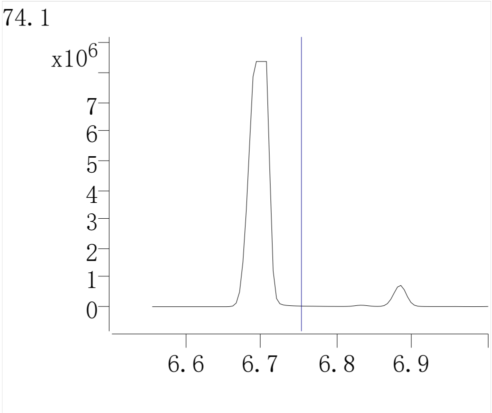

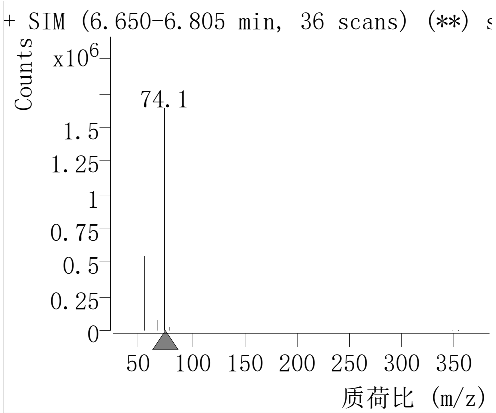

C16:1

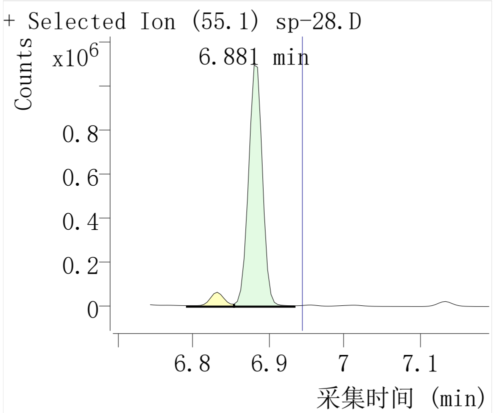

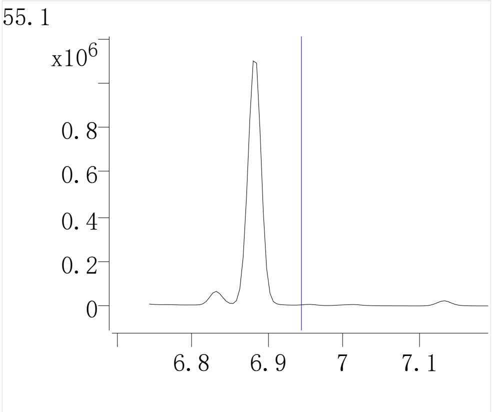

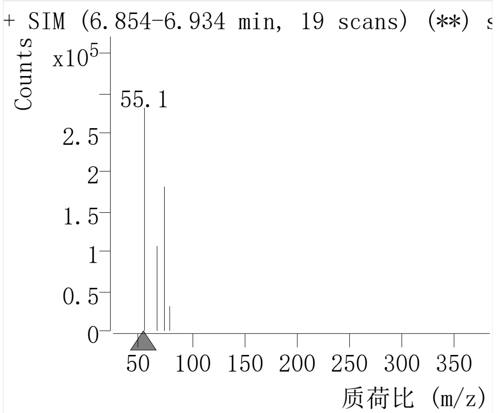

C17:0

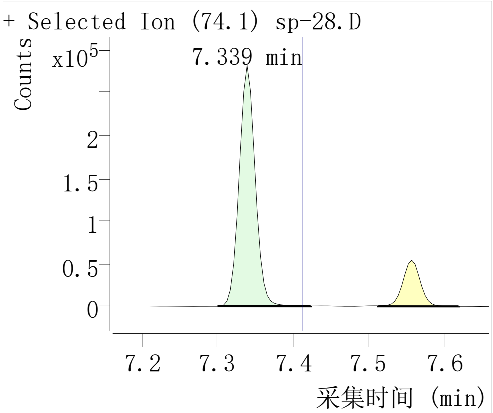

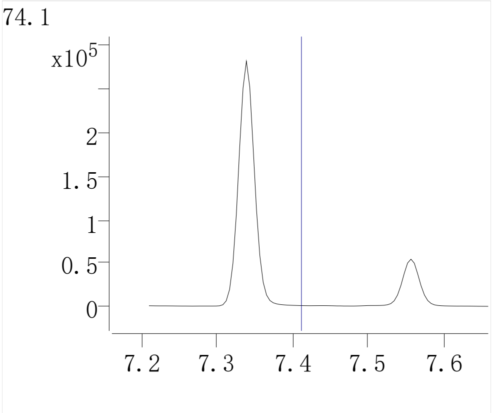

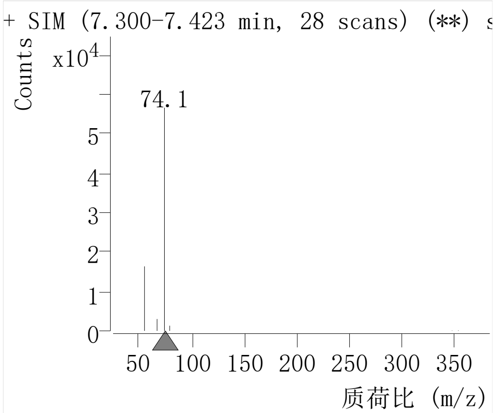

C17:1

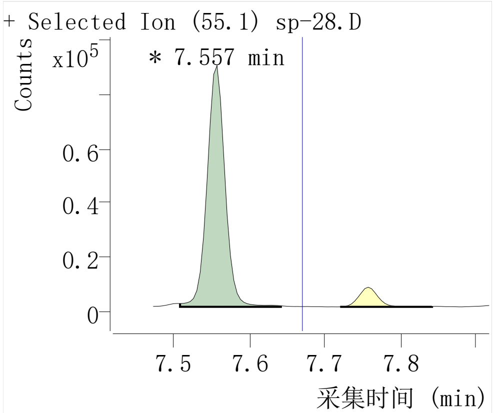

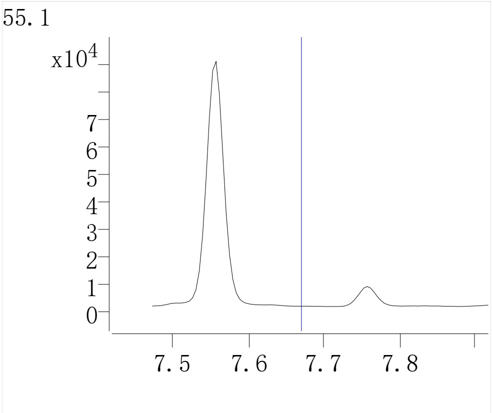

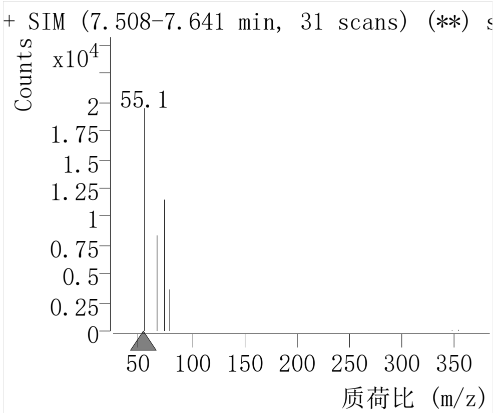

C18:0

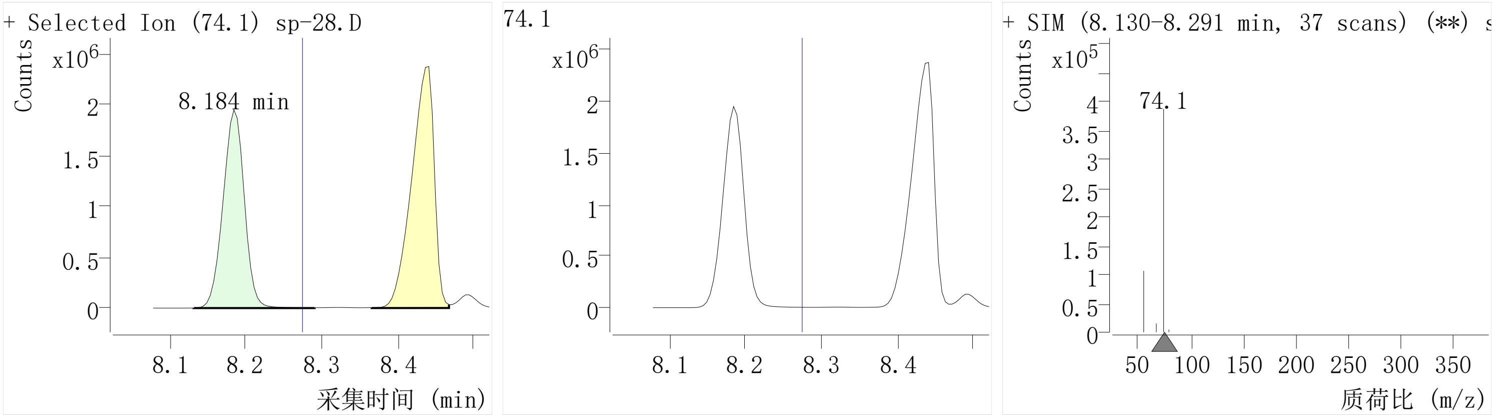

C18:1n9t

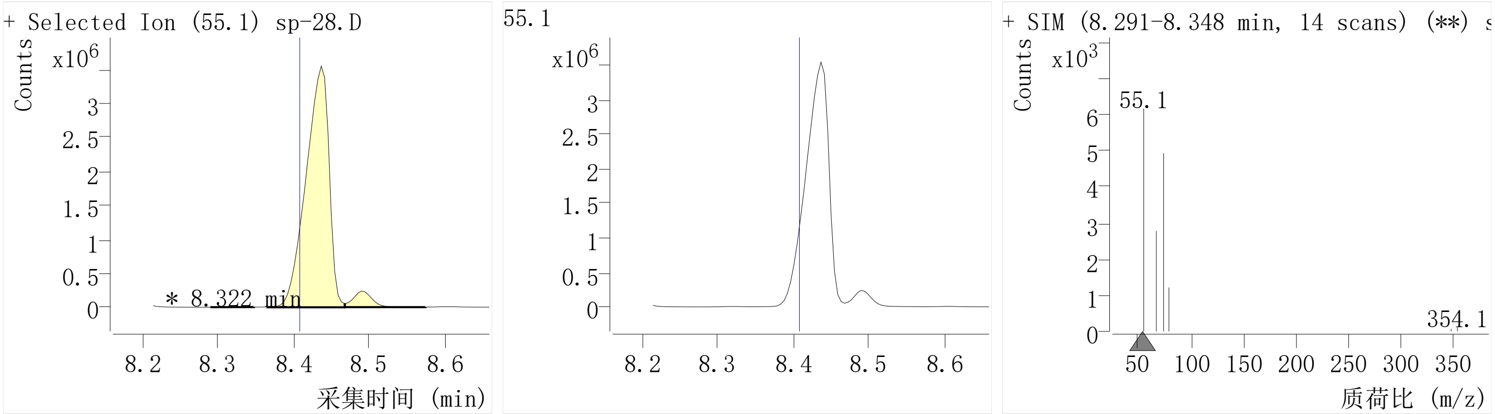

C18:1n9c

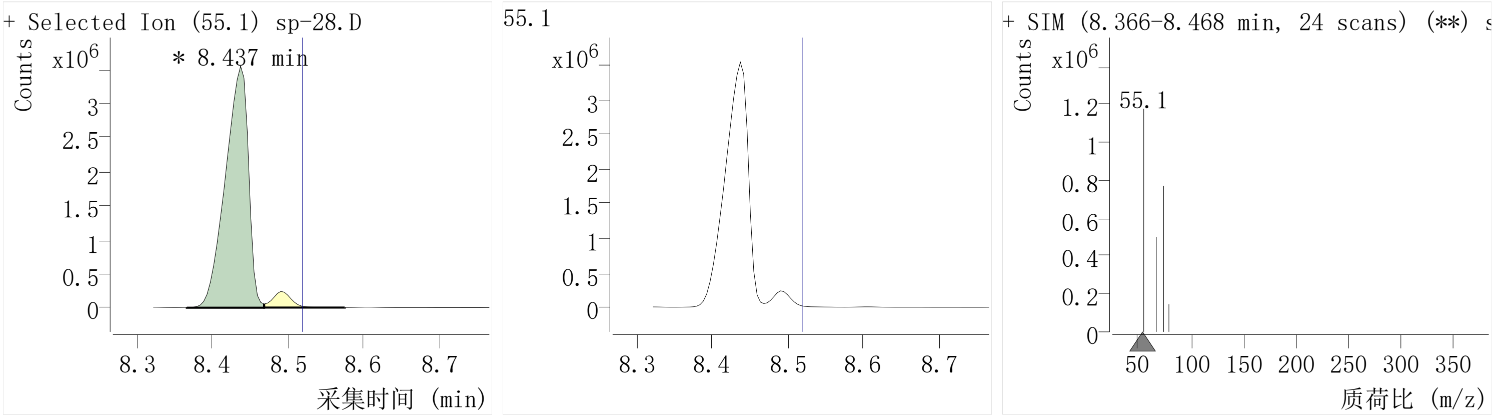

C18:2n6t

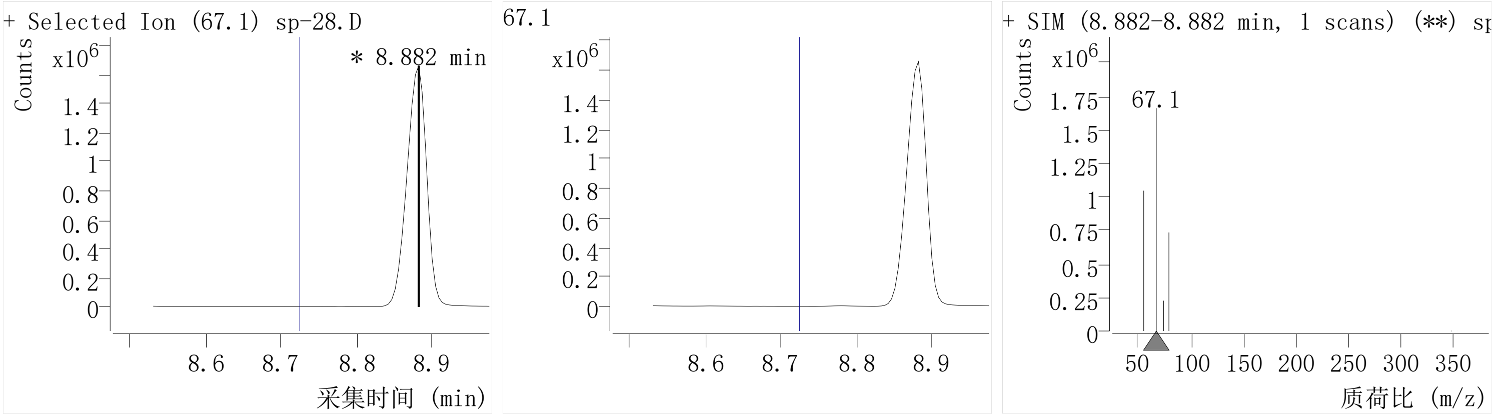

C18:2n6c

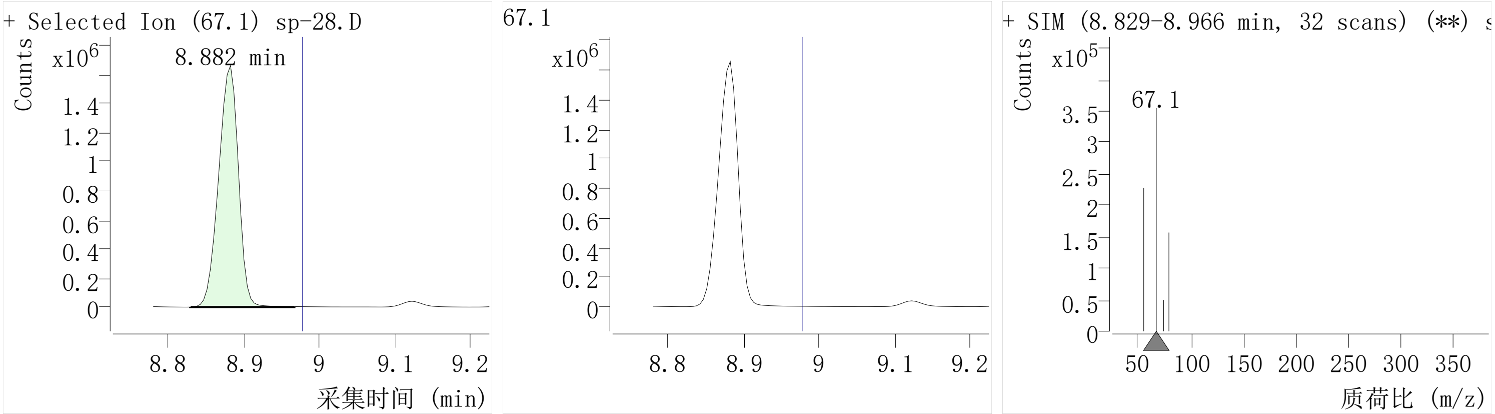

C18:3n6

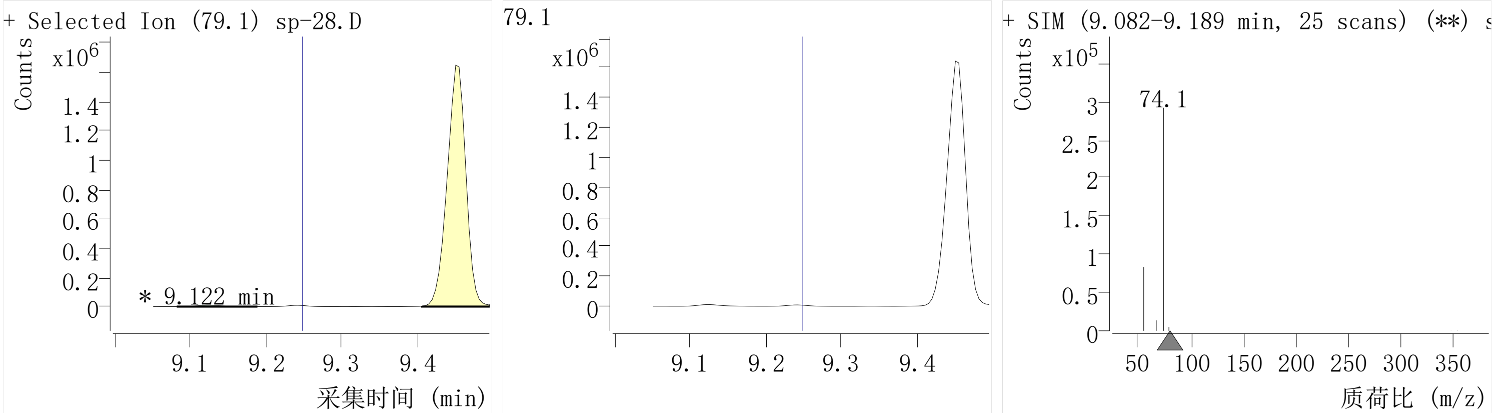

C18:3n3

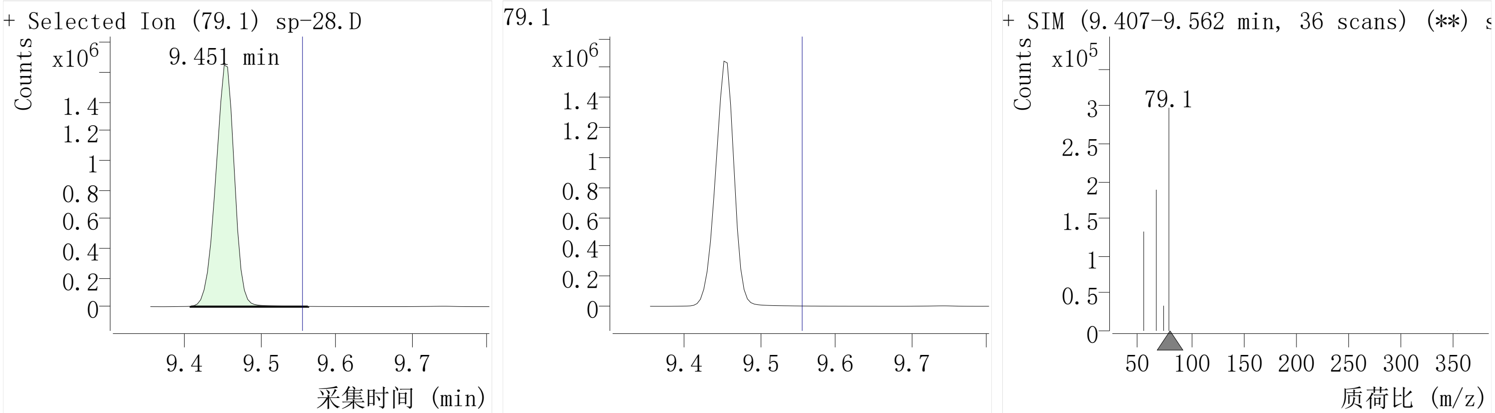

C20:0

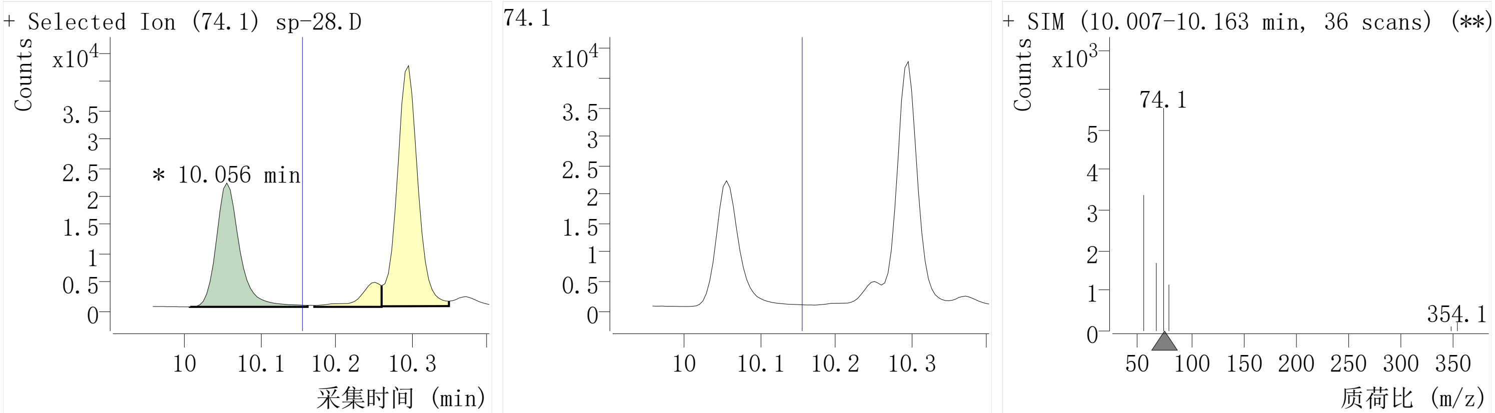

C20:1

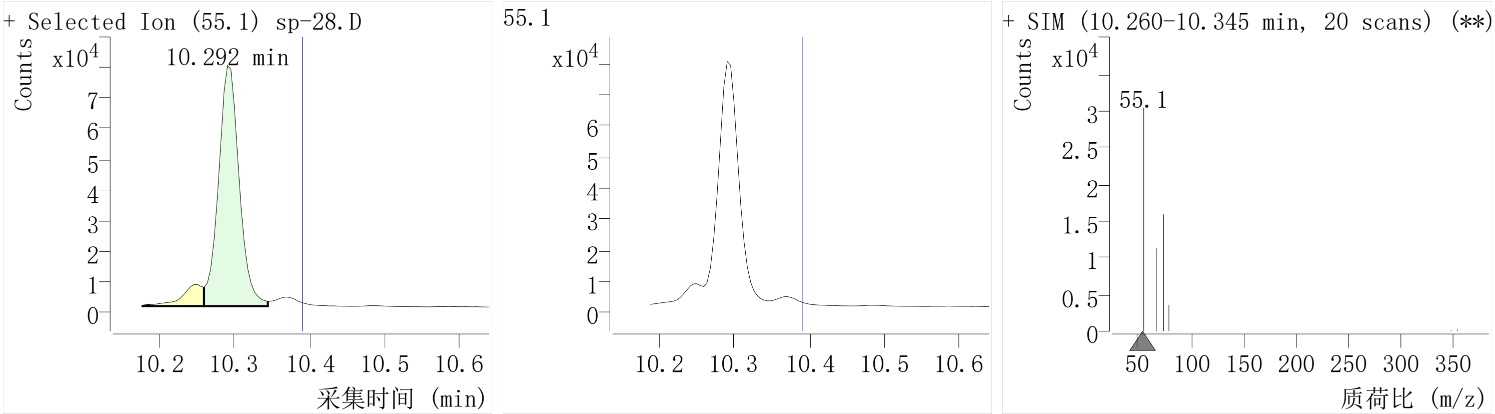

C20:2

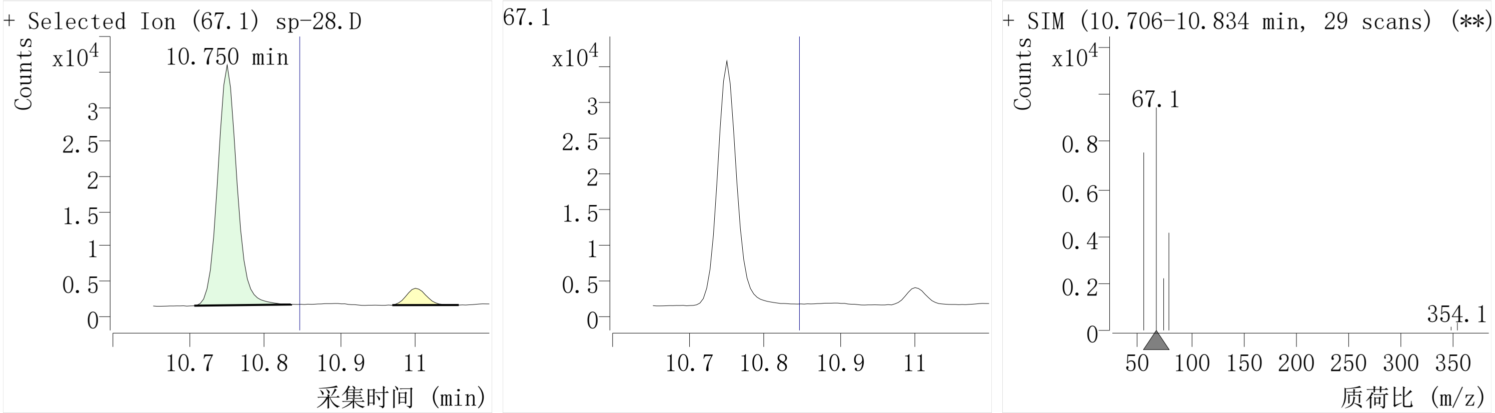

C21:0

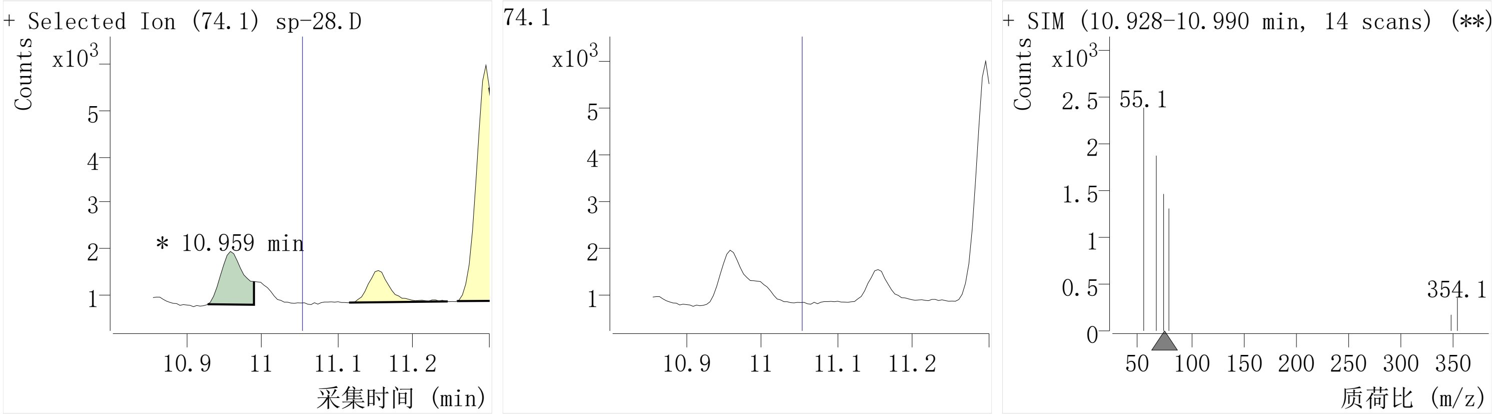

C20:3n6

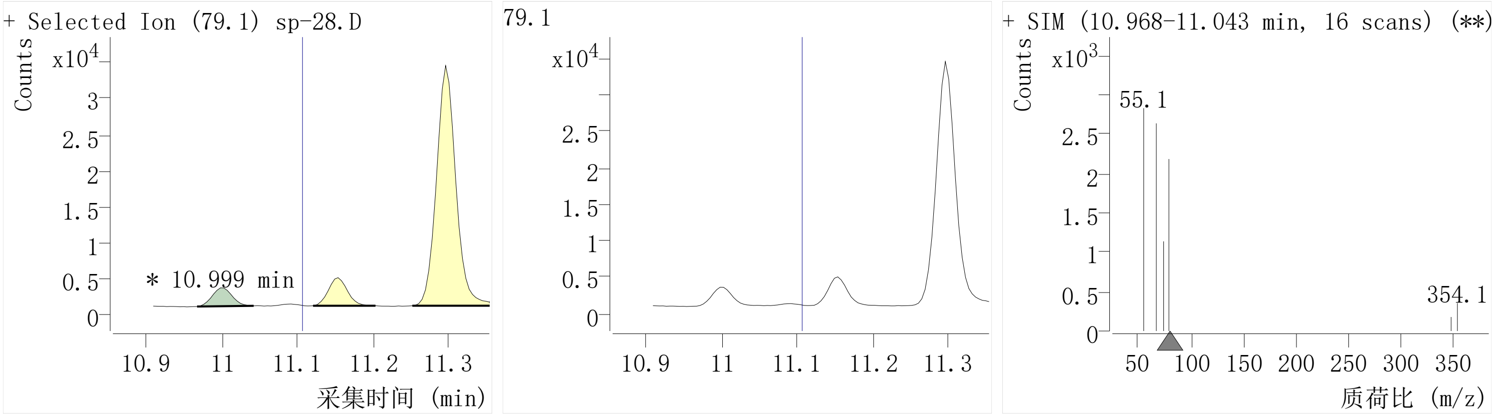

C20:4n6

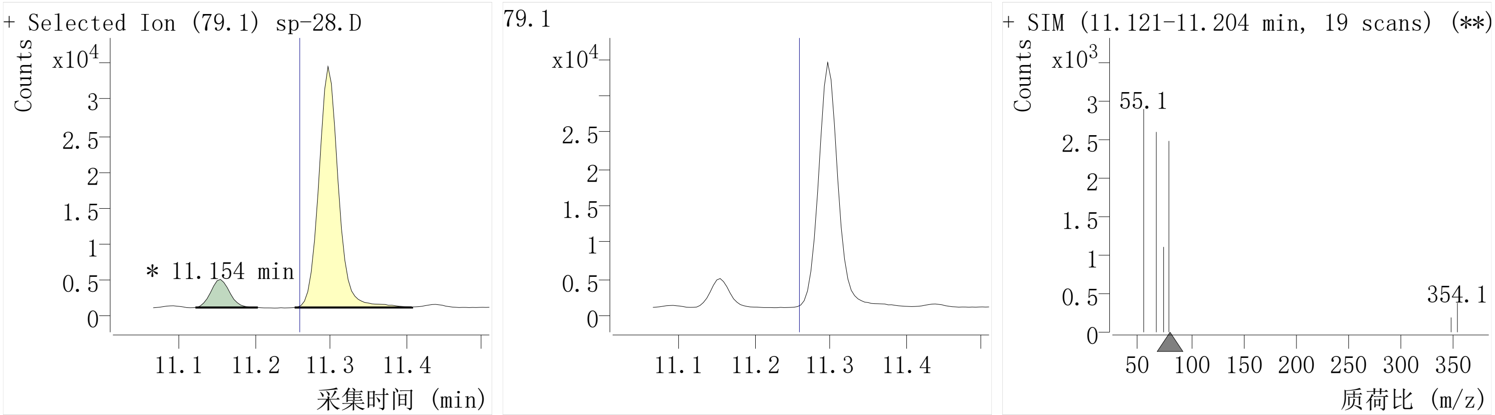

C20:3n3

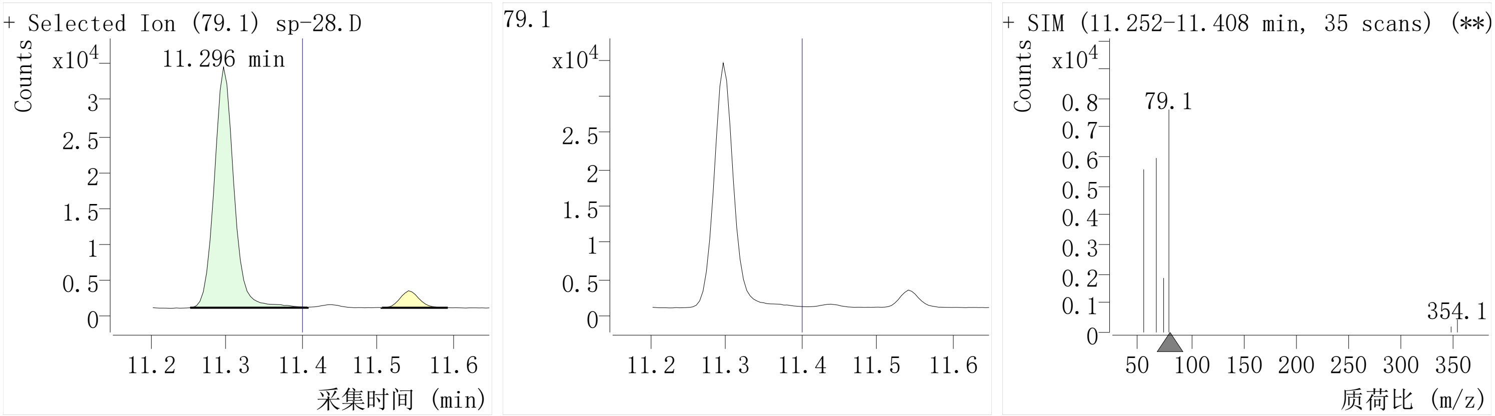

C20:5n3

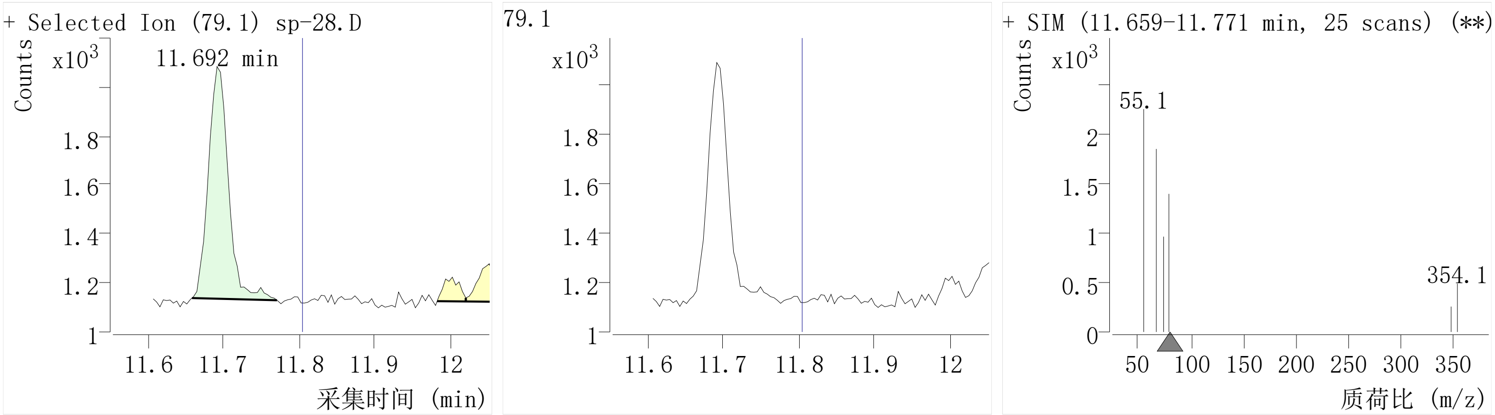

C22:0

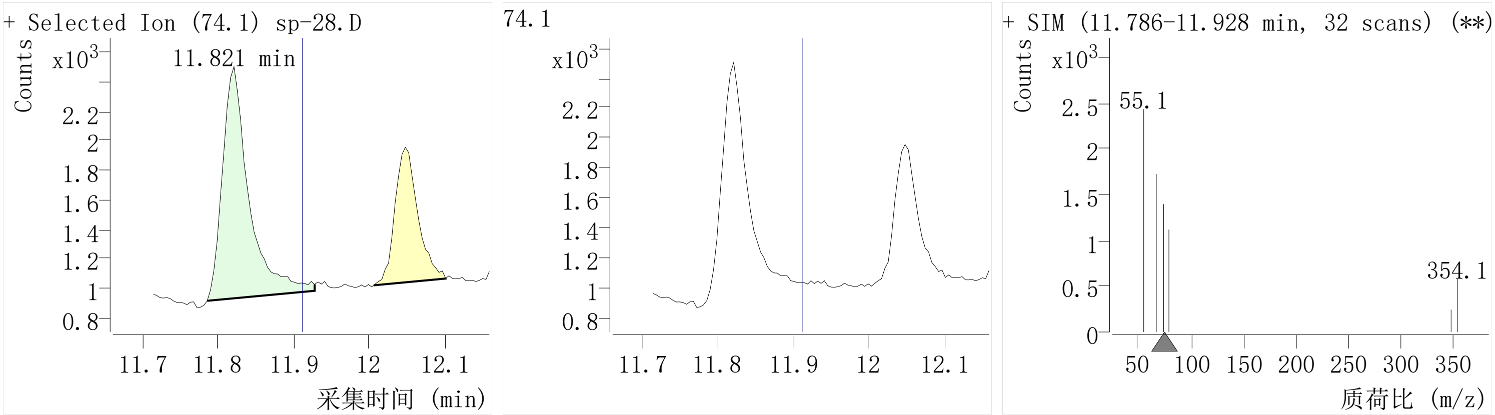

C22:1n9

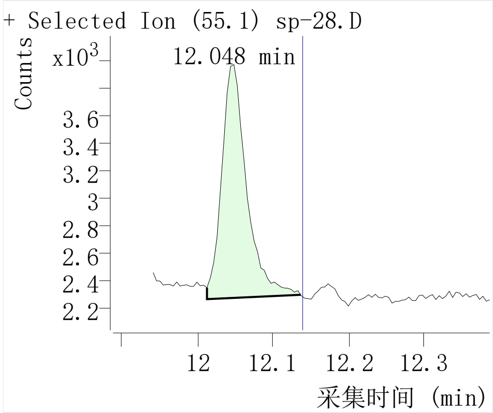

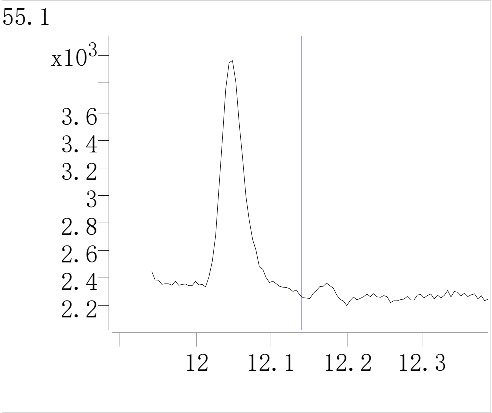

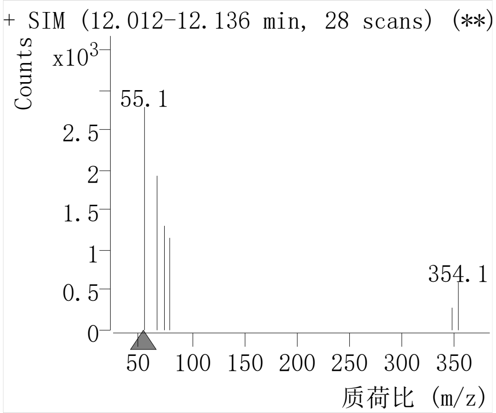

C22:2n6

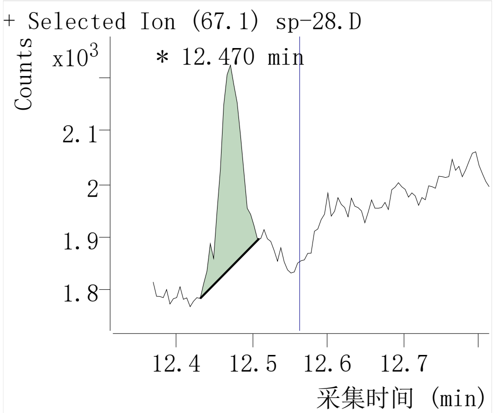

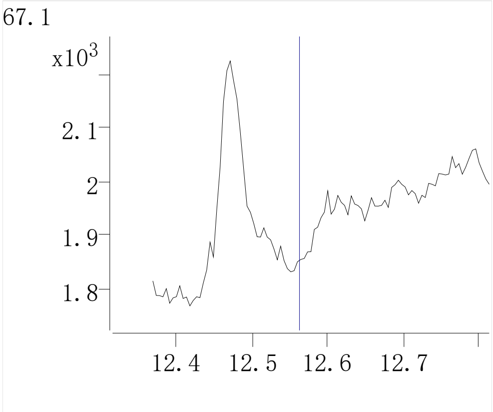

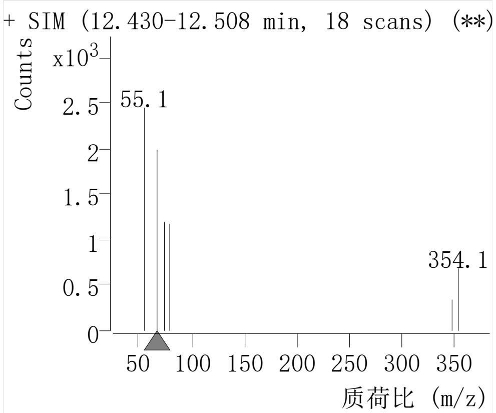

C23:0

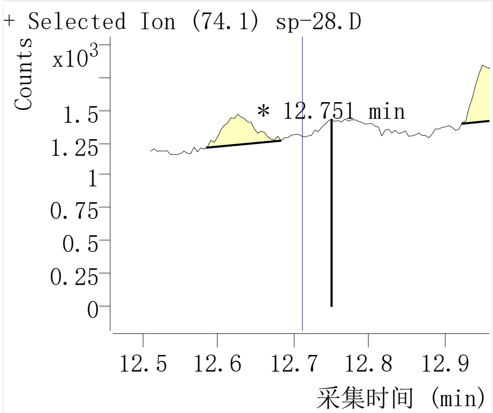

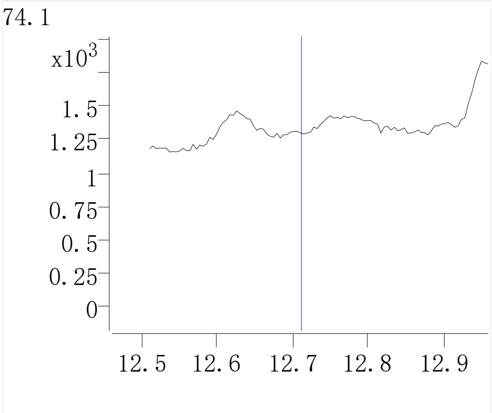

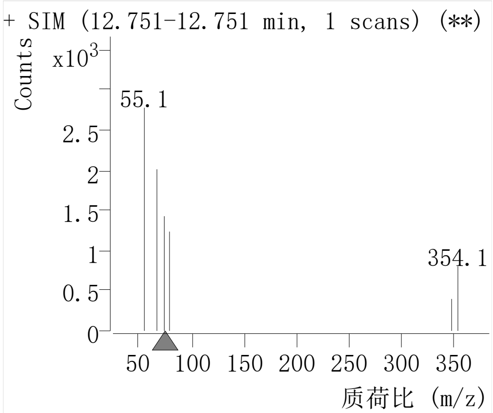

C24:0

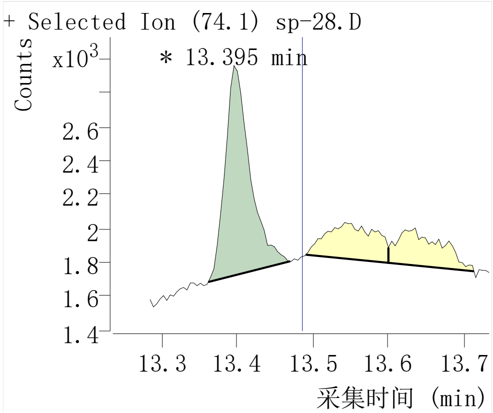

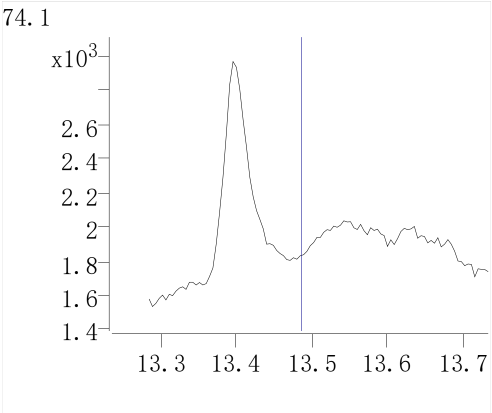

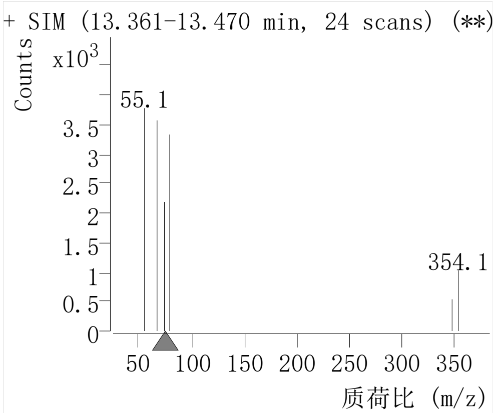

C22:6

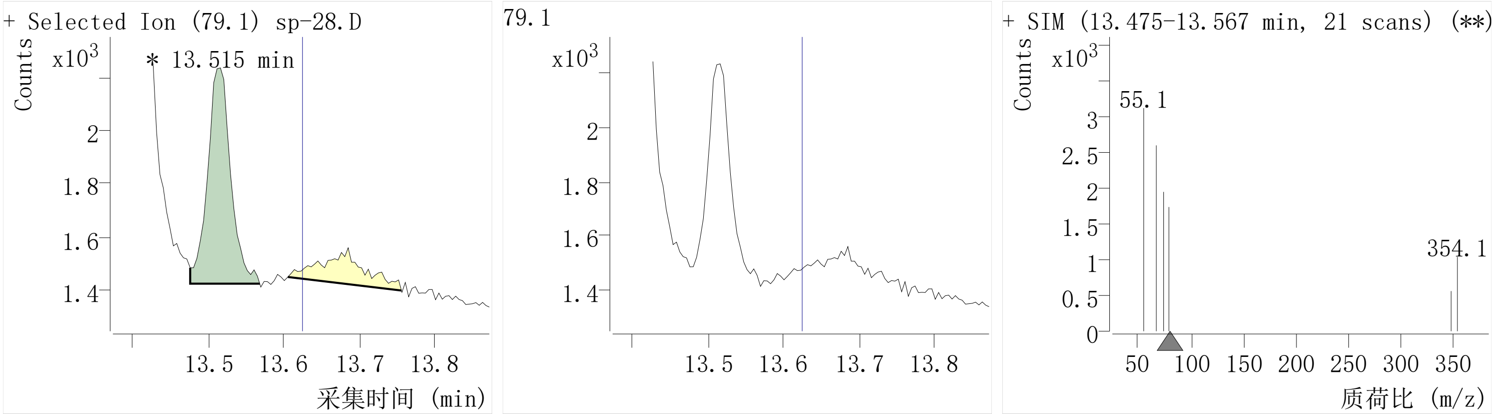
+ Selected Ion (79.1) sp-28.D
Counts
x10^3
* 13.515 min
2
1.8
1.6
1.4
13.5
13.6
13.7
13.8
采集时间 (min)
79.1
+ SIM (13.475-13.567 min, 21 scans) (**)
55.1
354.1
3
2.5
1.5
1
0.5
0
50
100
150
200
250
300
350
质荷比 (m/z)

C24:1

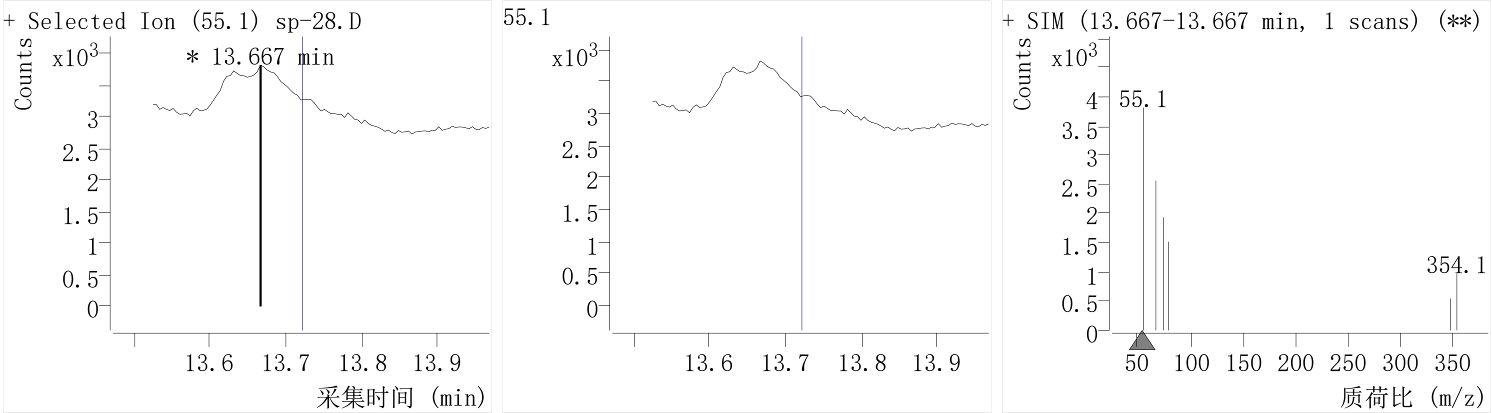
+ Selected Ion (55.1) sp-28.D
Counts
x10^3
* 13.667 min
3
2.5
2
1.5
1
0.5
0
13.6
13.7
13.8
13.9
采集时间 (min)
55.1
+ SIM (13.667-13.667 min, 1 scans) (**)
55.1
354.1
4
3.5
50
100
150
200
250
300
350
质荷比 (m/z)

定量分析完成报告

**批处理路径**	G:\GC-MS\HX250430-4-GCMS总脂肪酸靶向检测\HX250430-4\QuantResults\HX250430-4.batch.bin		
**分析时间**	2025/5/14 16:58	**分析员姓名**	DESKTOP-M3A0GPO\omics
**报告时间**	2025/5/16 14:53:34	**报告员姓名**	DESKTOP-M3A0GPO\omics
**最近校正更新**	2025/5/14 16:58	**批处理状态**	已处理
**定量批处理版本**	10.2	**定量报告版本**	10.2

**采集时间**	2025/5/9 8:18	**数据文件**	sp-29.D
**样品类型**	样品	**样品名称**	sp-29
**稀释**	1	**采集方法**	脂肪酸

样品色谱图

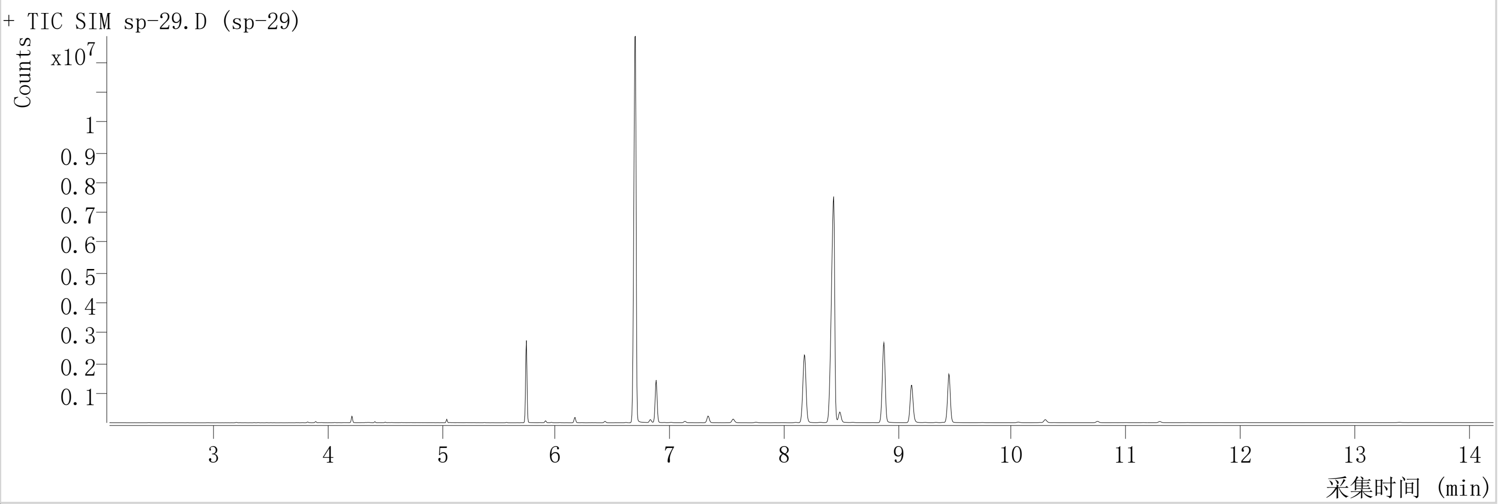

化合物	ISTD	RT	响应	ISTD 响应	响应比	最终浓度	单位
C4:0	C19:0	2.203	128	1770121	0.0001	ND	ug/ml
C6:0	C19:0	2.959	569	1770121	0.0003	0.0033	ug/ml
C8:0	C19:0	3.724	1795	1770121	0.0010	0.0064	ug/ml
C10:0	C19:0	4.413	20412	1770121	0.0115	0.1644	ug/ml
C11:0	C19:0	4.728	1441	1770121	0.0008	0.0069	ug/ml
C12:0	C19:0	5.044	66885	1770121	0.0378	0.5460	ug/ml
C13:0	C19:0	5.373	4370	1770121	0.0025	0.0255	ug/ml
C14:0	C19:0	5.742	1897708	1770121	1.0721	22.5151	ug/ml
C14:1	C19:0	5.911	31441	1770121	0.0178	0.7891	ug/ml
C15:0	C19:0	6.169	146788	1770121	0.0829	1.4188	ug/ml
C15:1	C19:0	6.432	0	1770121	0.0000	ND	ug/ml
C16:0	C19:0	6.698	13199847	1770121	7.4570	288.3121	ug/ml
C16:1	C19:0	6.881	830552	1770121	0.4692	28.5854	ug/ml
C17:0	C19:0	7.334	263685	1770121	0.1490	3.1078	ug/ml
C17:1	C19:0	7.557	95545	1770121	0.0540	2.8782	ug/ml
C18:0	C19:0	8.179	3384934	1770121	1.9123	41.7032	ug/ml
C18:1n9t	C19:0	8.321	9220	1770121	0.0052	0.3073	ug/ml
C18:1n9c	C19:0	8.437	7034089	1770121	3.9738	268.3820	ug/ml
C18:2n6t	C19:0	8.877	0	1770121	0.0000	ND	ug/ml
C18:2n6c	C19:0	8.877	2146466	1770121	1.2126	87.5779	ug/ml
C18:3n6	C19:0	9.122	19717	1770121	0.0111	ND	ug/ml
C18:3n3	C19:0	9.446	1277163	1770121	0.7215	35.8661	ug/ml
C20:0	C19:0	10.056	35069	1770121	0.0198	0.5337	ug/ml
C20:1	C19:0	10.291	101616	1770121	0.0574	3.5776	ug/ml
C20:2	C19:0	10.749	37759	1770121	0.0213	1.3583	ug/ml
C21:0	C19:0	10.958	2254	1770121	0.0013	0.0352	ug/ml
C20:3n6	C19:0	11.003	2813	1770121	0.0016	0.1446	ug/ml
C20:4n6	C19:0	11.154	3335	1770121	0.0019	0.1527	ug/ml
C20:3n3	C19:0	11.296	32372	1770121	0.0183	1.0072	ug/ml
C20:5n3	C19:0	11.692	829	1770121	0.0005	0.0611	ug/ml

定量分析完成报告

化合物	ISTD	RT	响应	ISTD 响应	响应比	最终浓度	单位
C22:0	C19:0	11.821	3875	1770121	0.0022	0.0912	ug/ml
C22:1n9	C19:0	12.043	3320	1770121	0.0019	0.1241	ug/ml
C22:2n6	C19:0	12.474	606	1770121	0.0003	0.0640	ug/ml
C23:0	C19:0	12.772	0	1770121	0.0000	ND	ug/ml
C24:0	C19:0	13.399	3004	1770121	0.0017	0.0829	ug/ml
C22:6	C19:0	13.515	789	1770121	0.0004	0.0381	ug/ml
C24:1	C19:0	13.671	0	1770121	0.0000	ND	ug/ml

C4:0

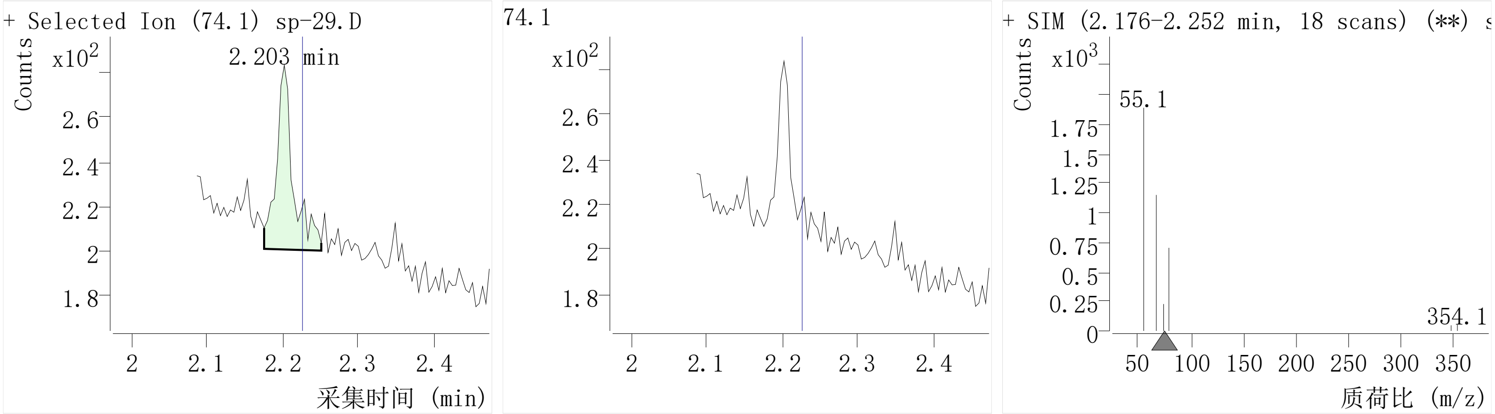

C6:0

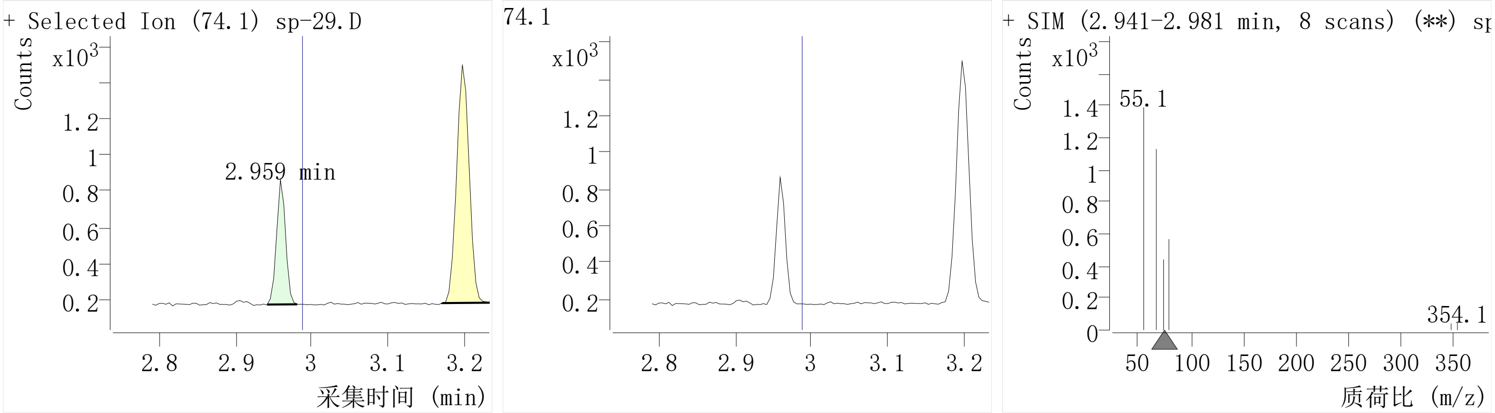

C8:0

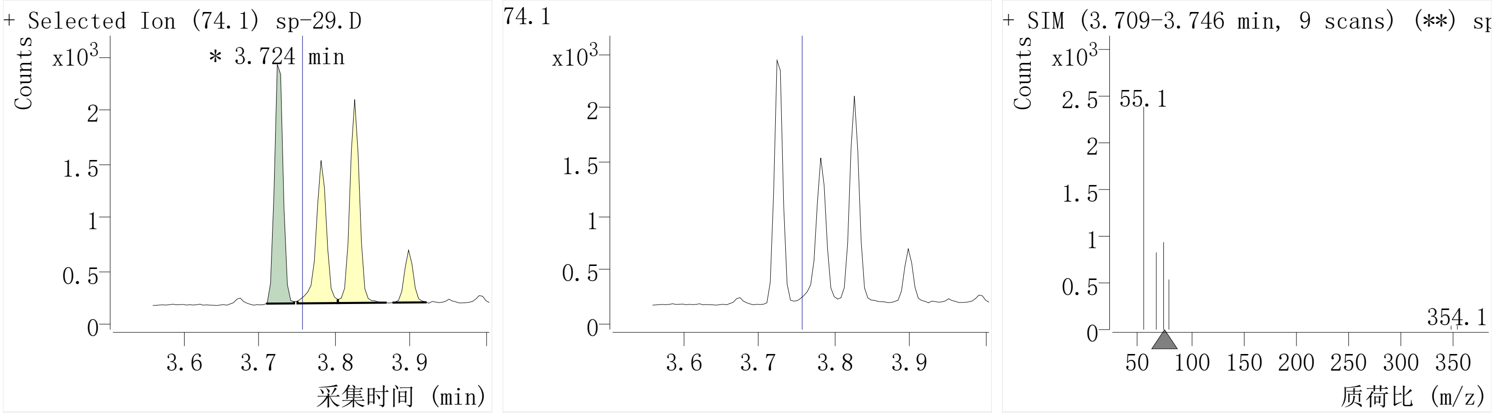

C10:0

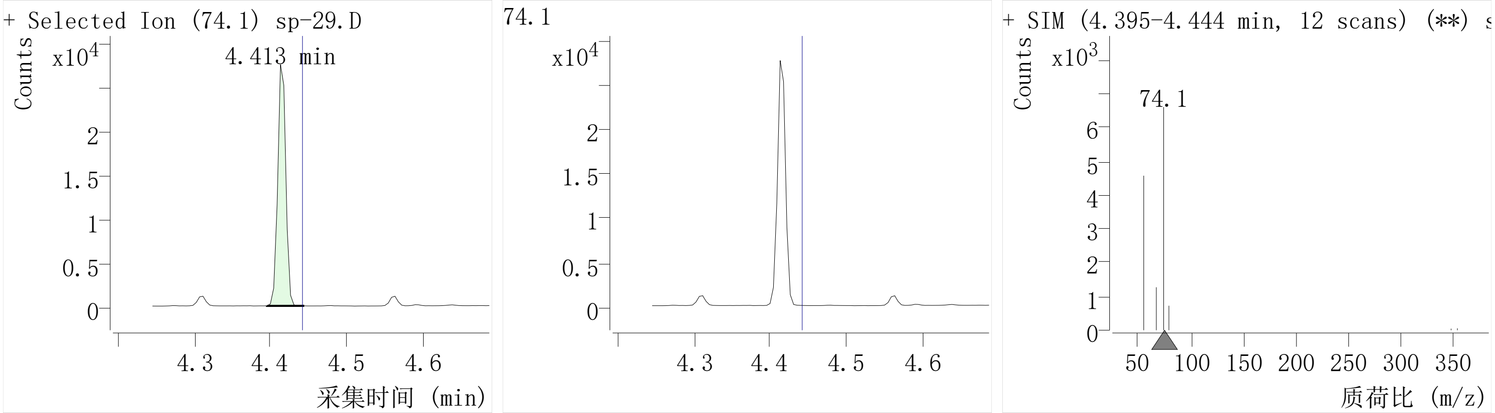

C11:0

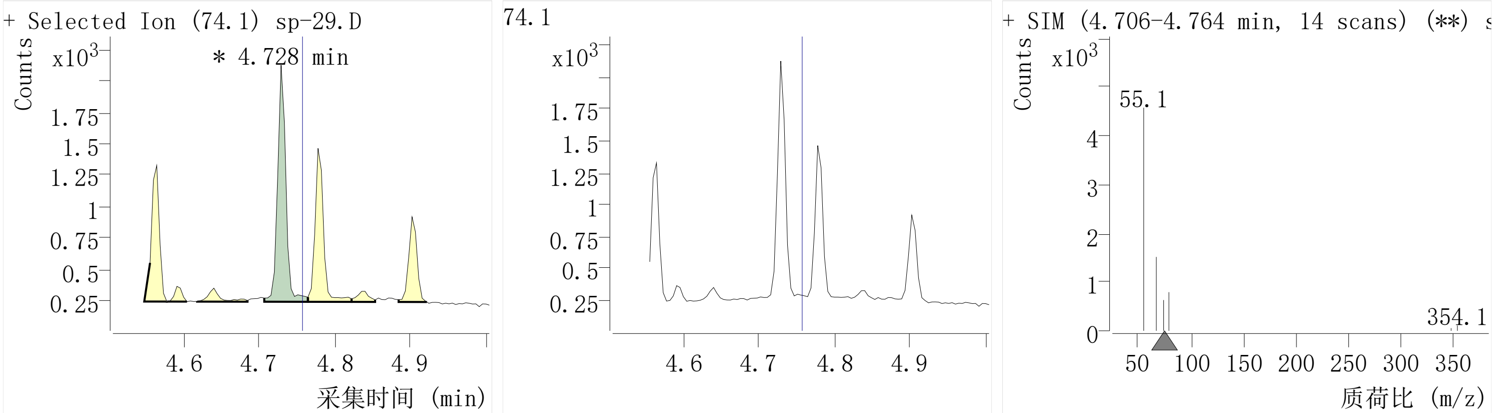

C12:0

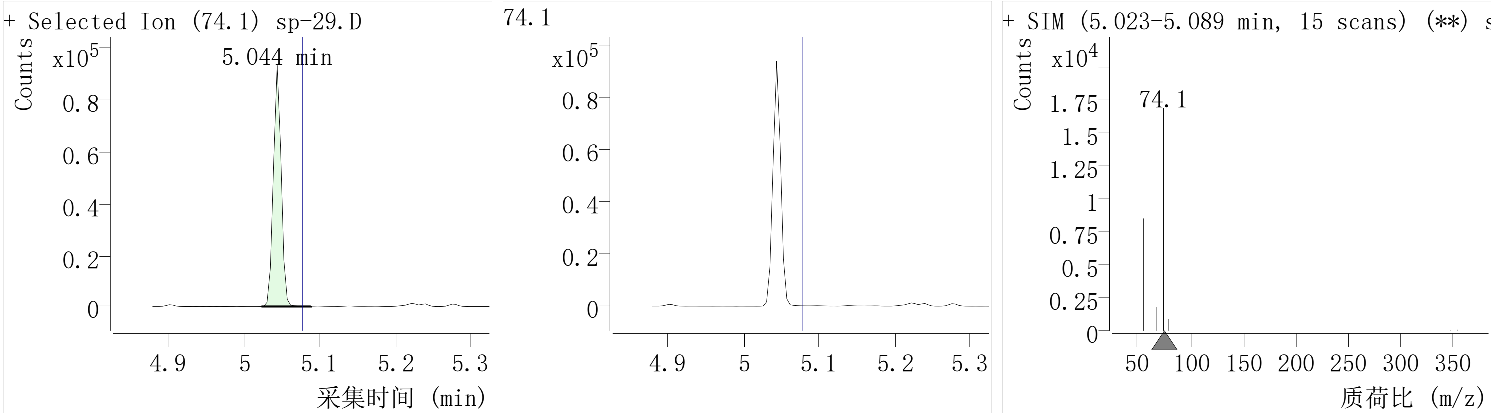

C13:0

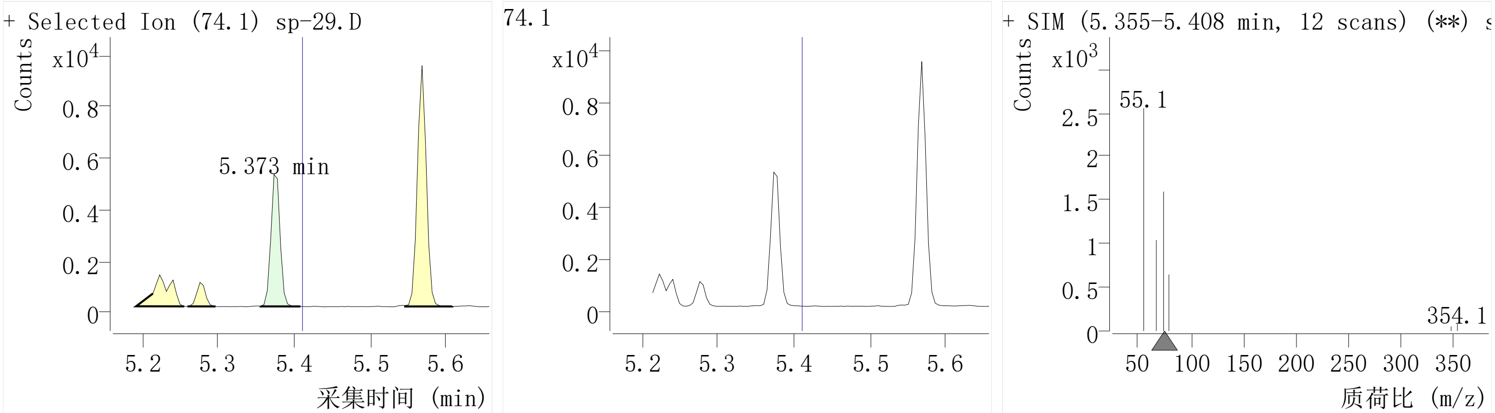

C14:0

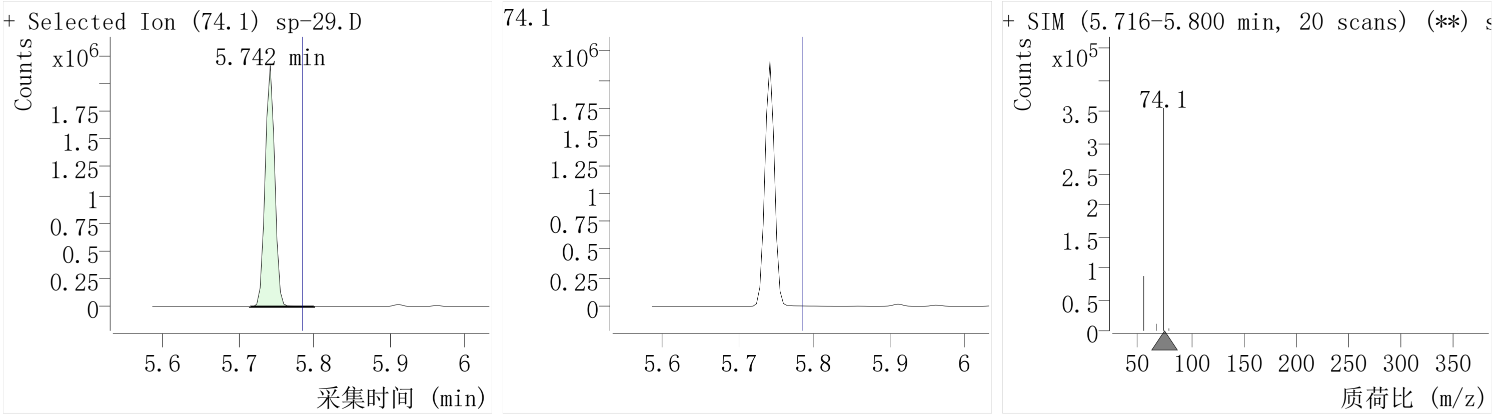

C14:1

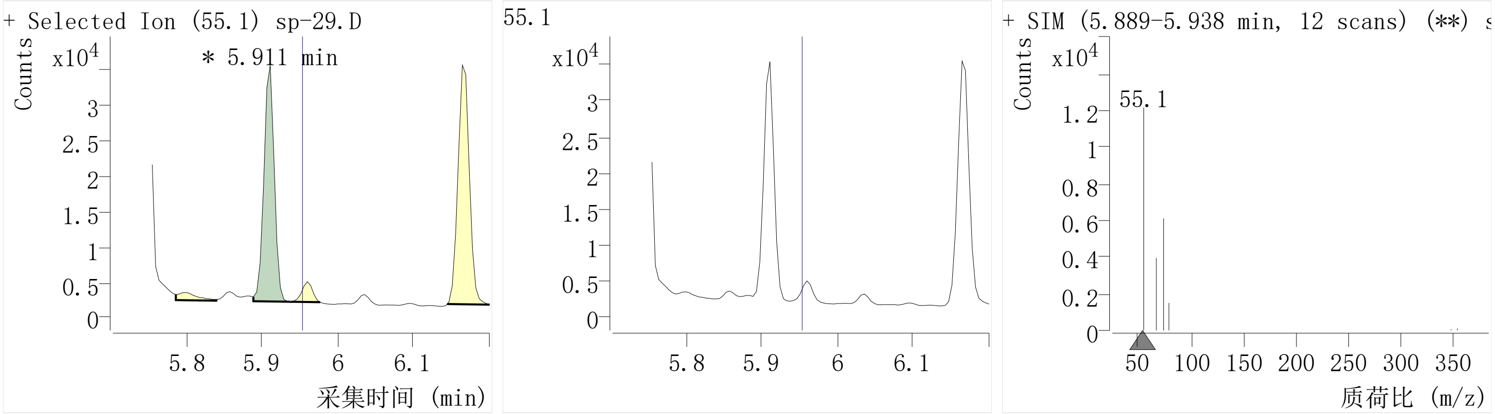

C15:0

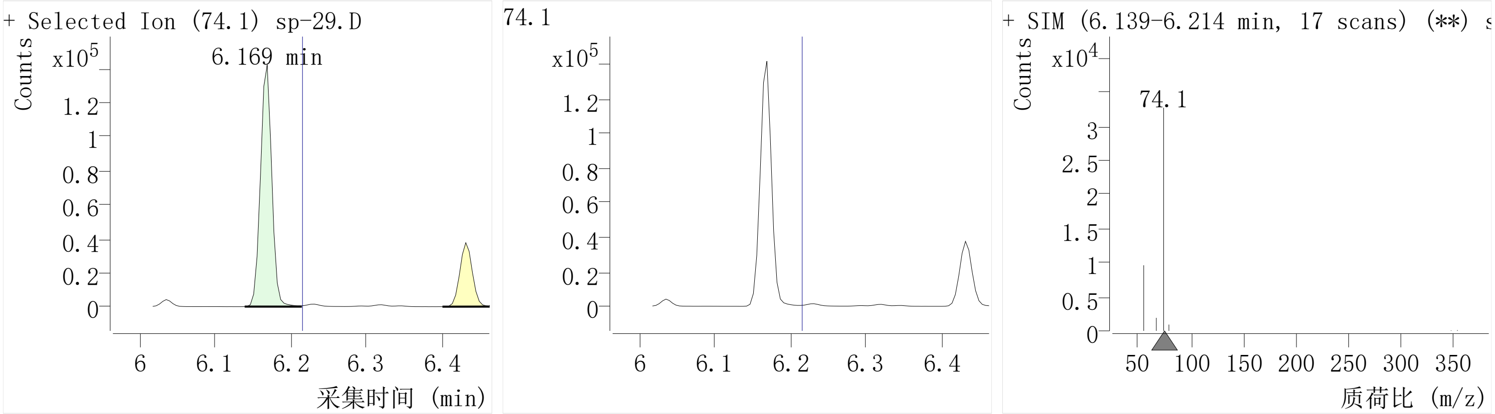

C15:1

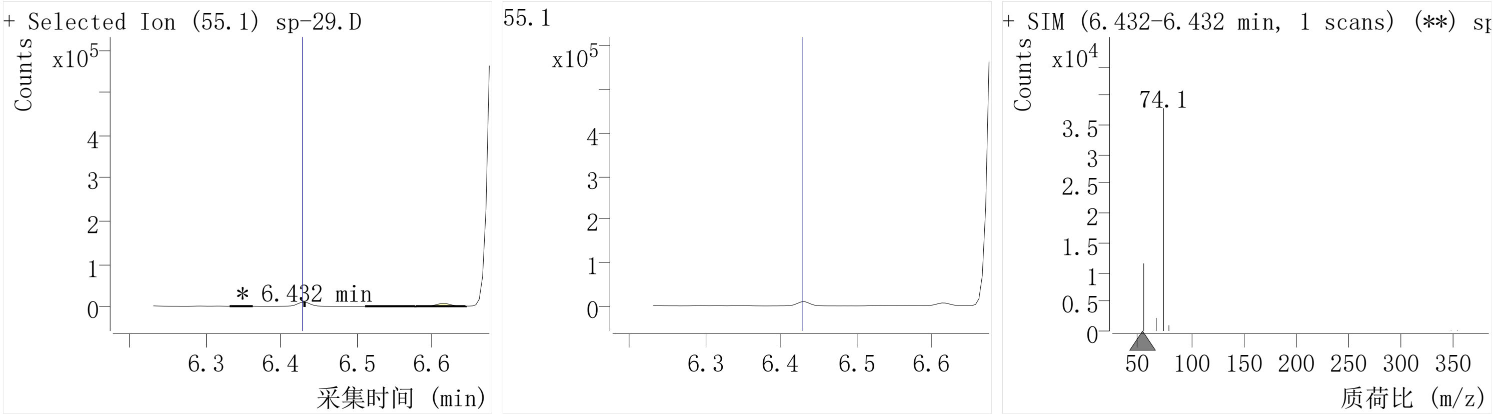

C16:0

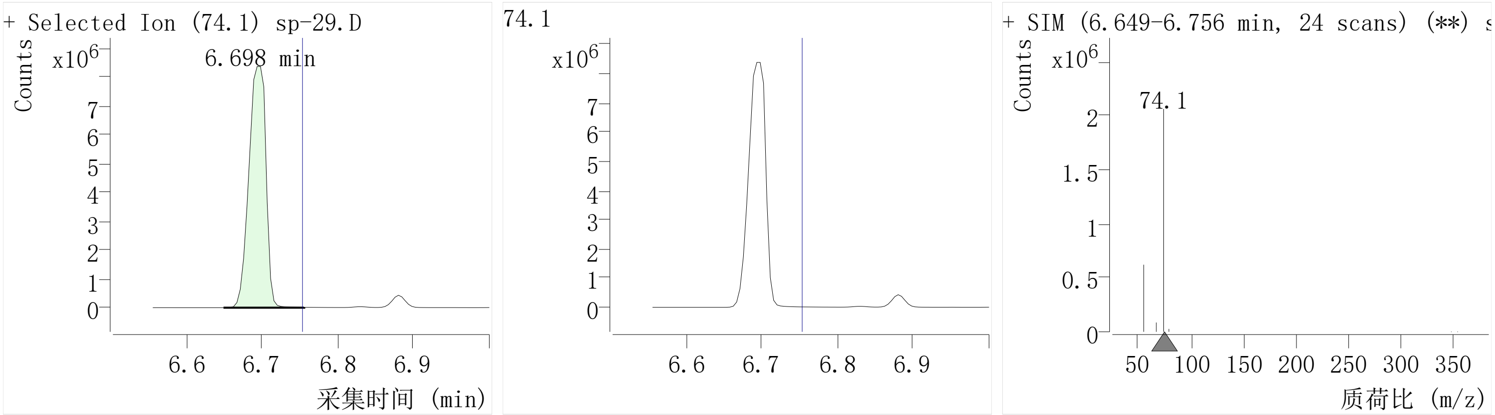

C16:1

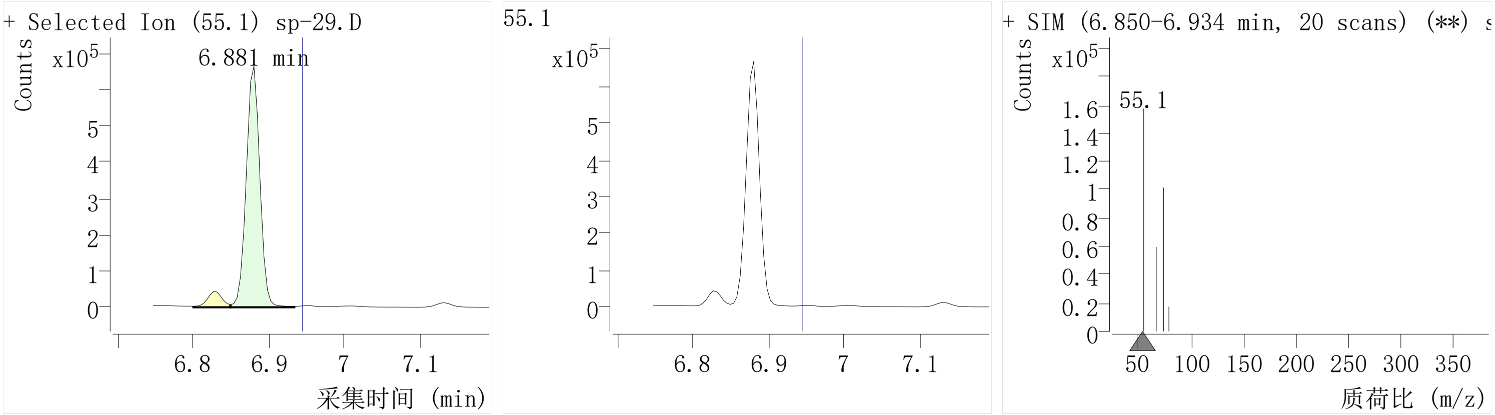

C17:0

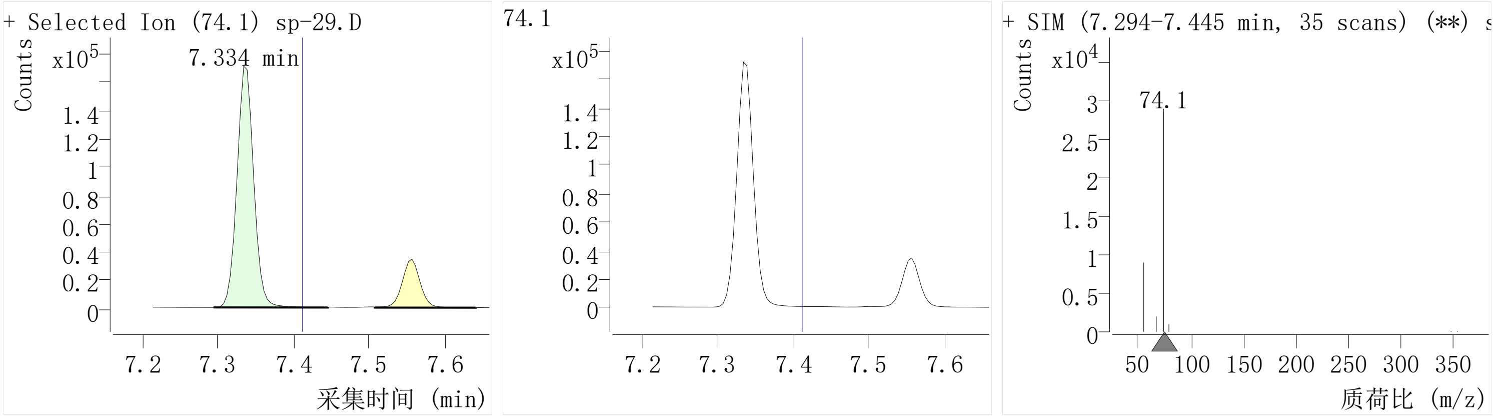

C17:1

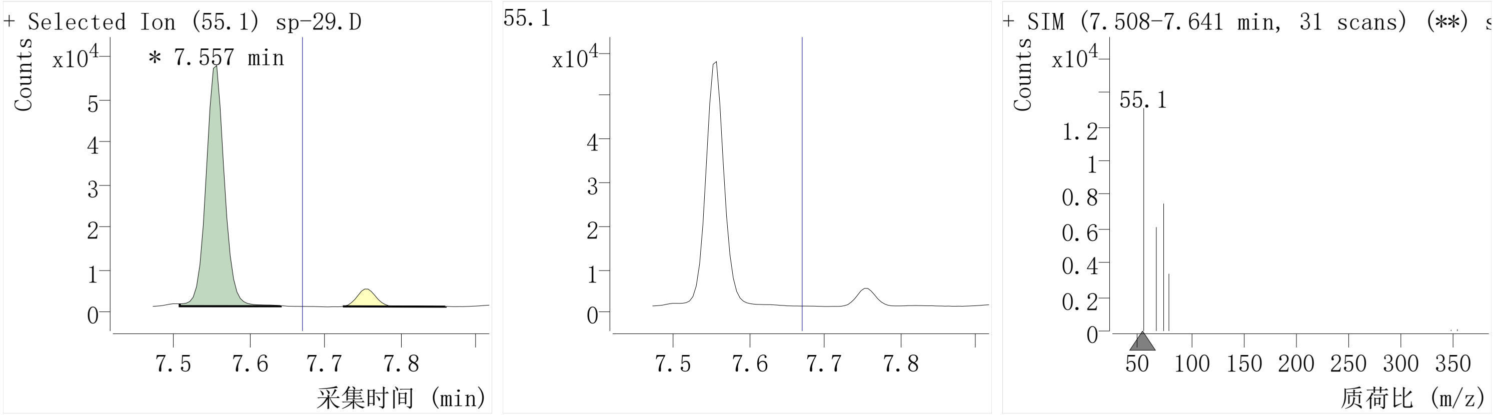

C18:0

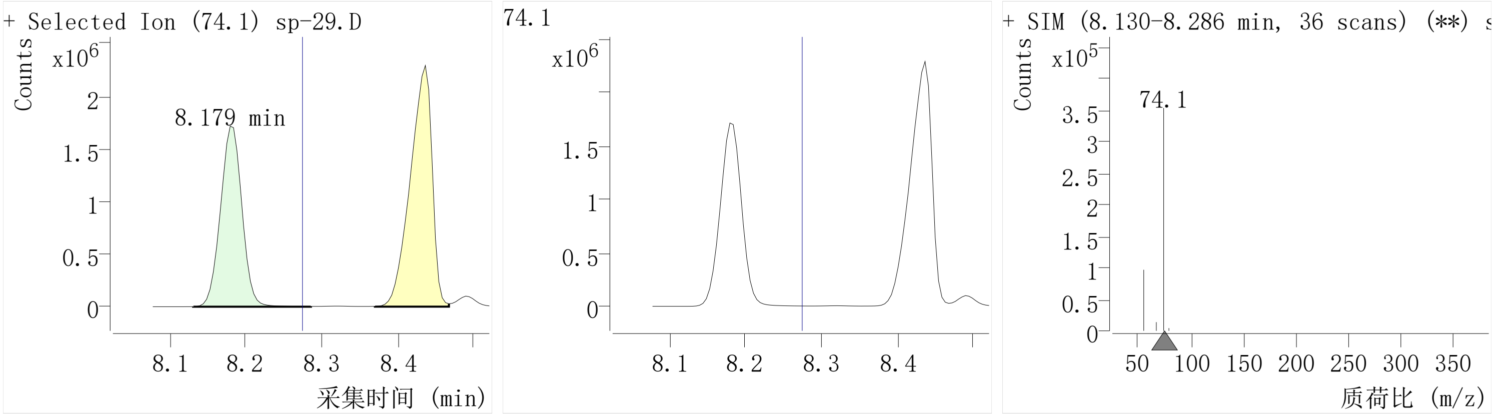

C18:1n9t

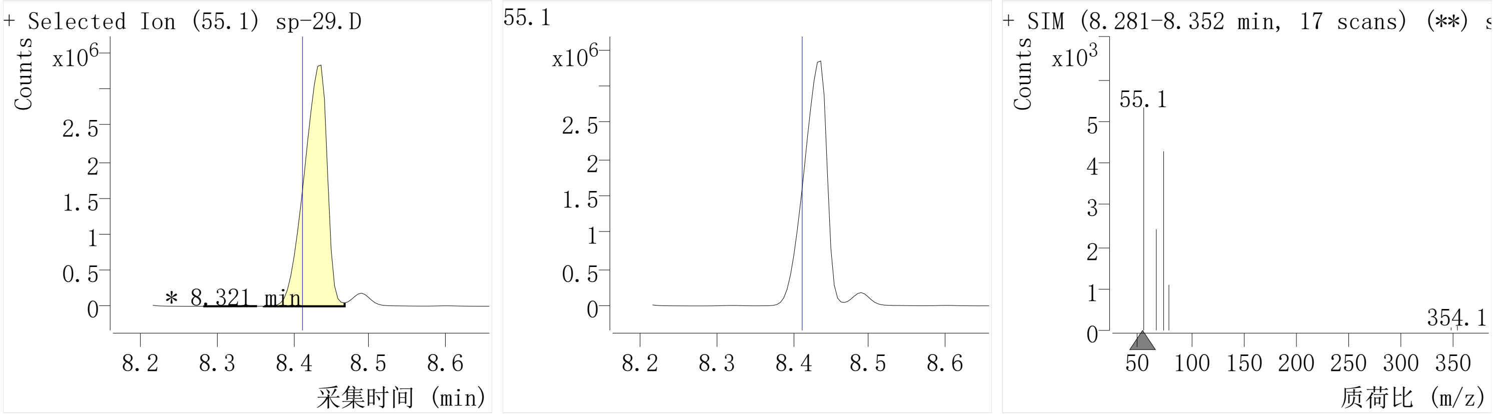

C18:1n9c

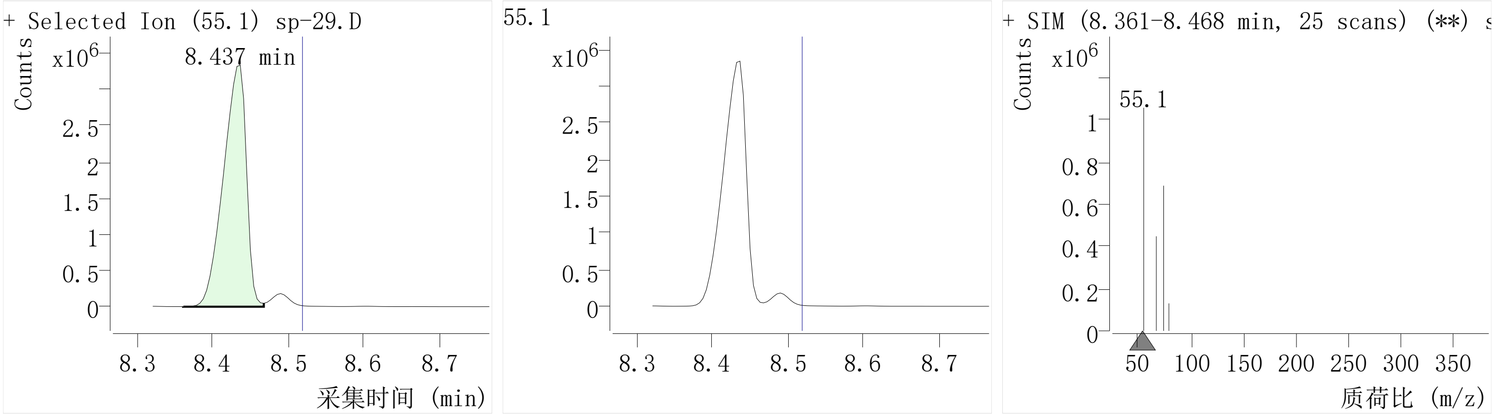

C18:2n6t

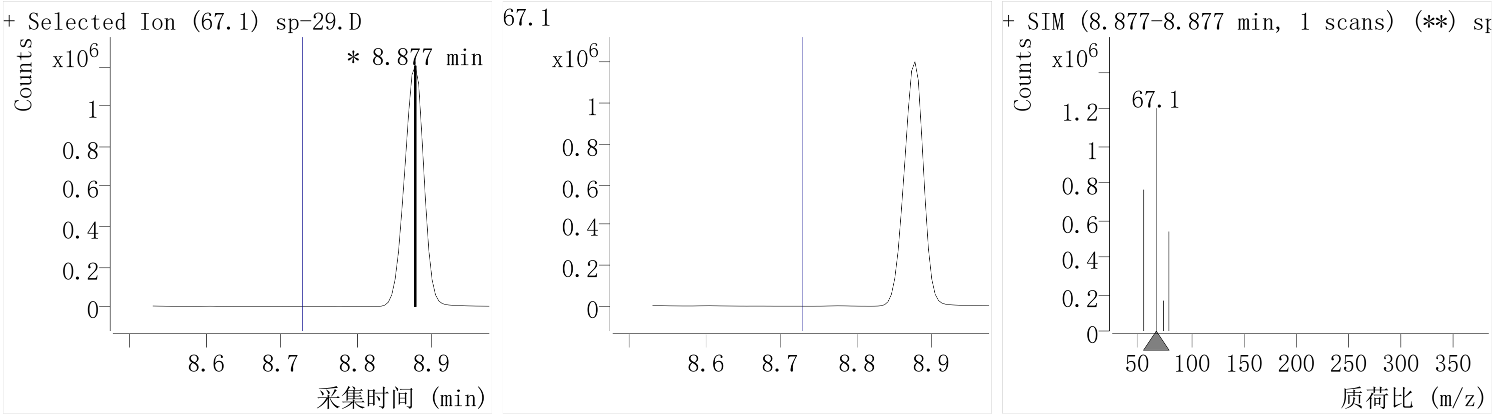

C18:2n6c

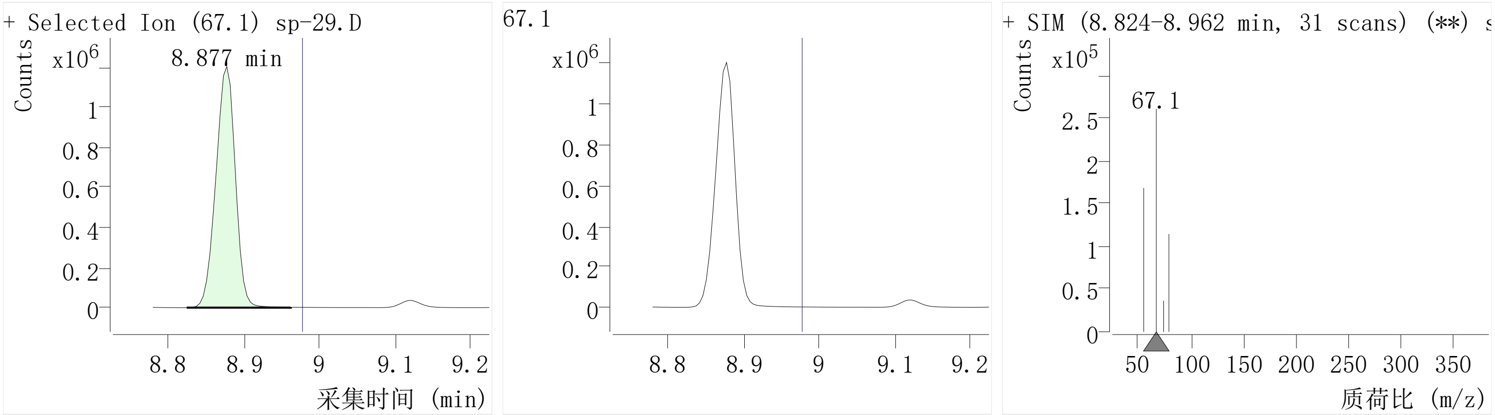

C18:3n6

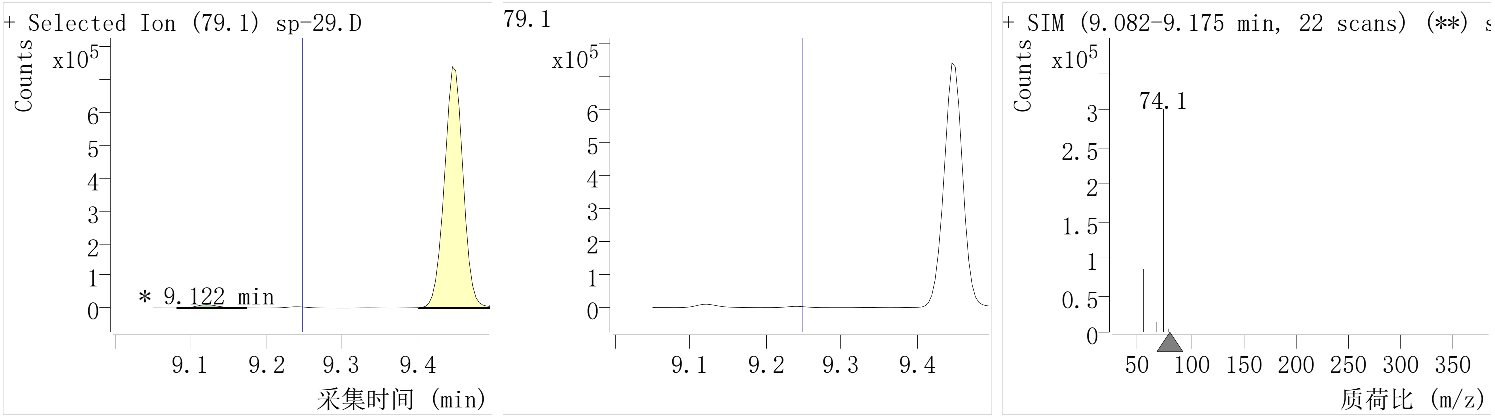

C18:3n3

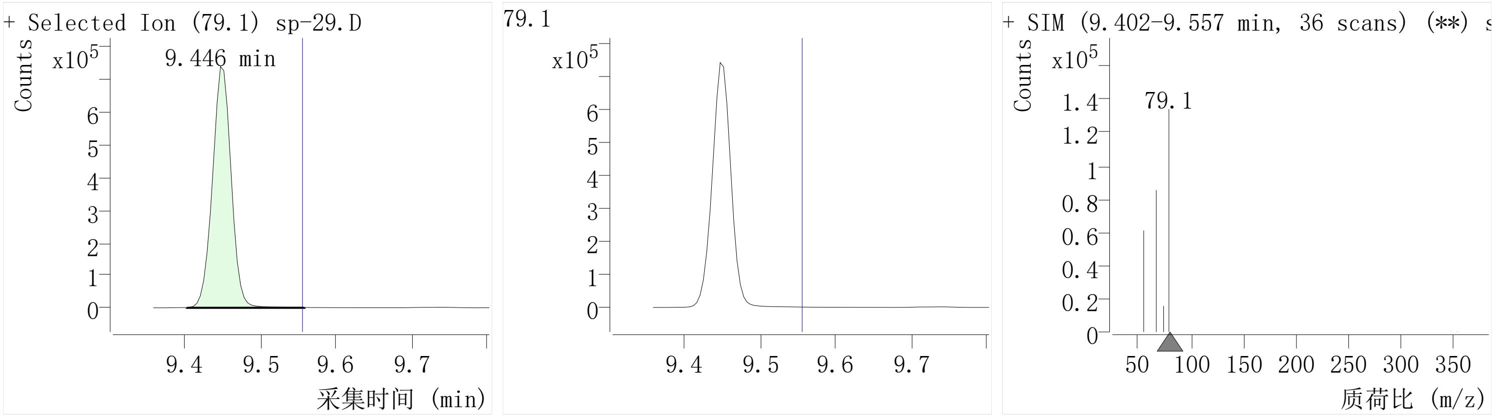

C20:0

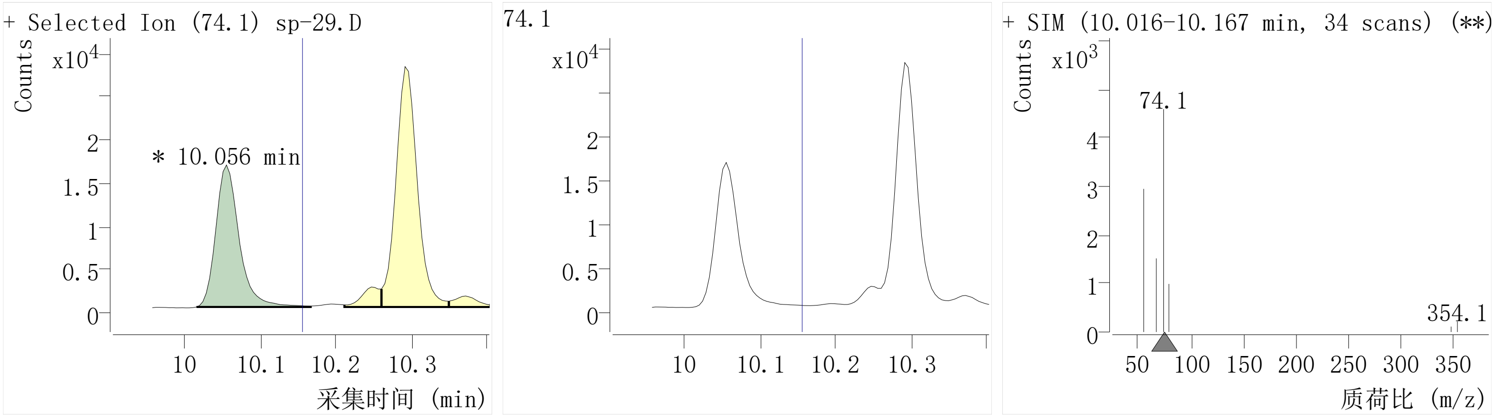

C20:1

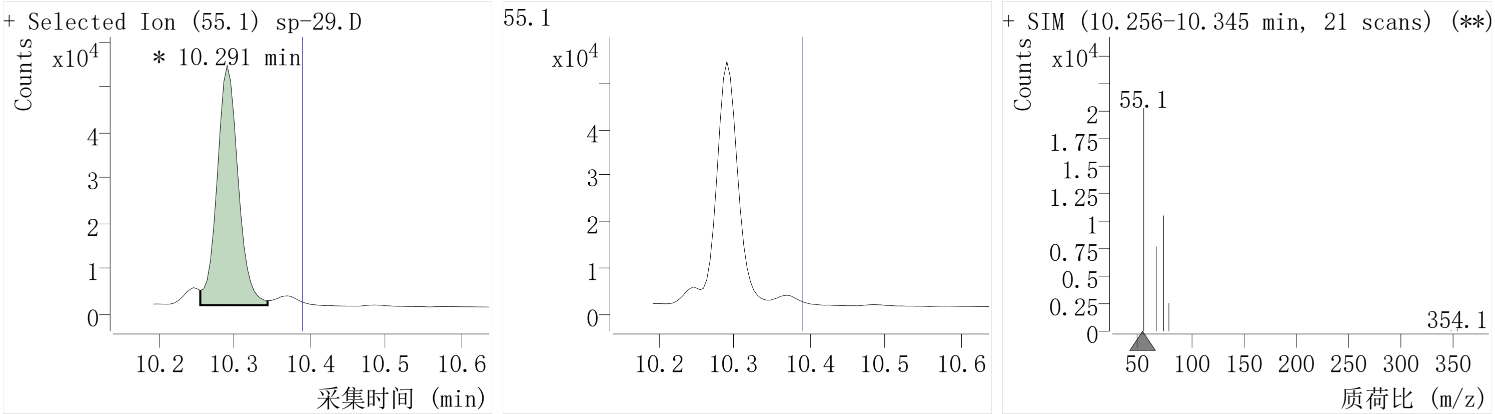

C20:2

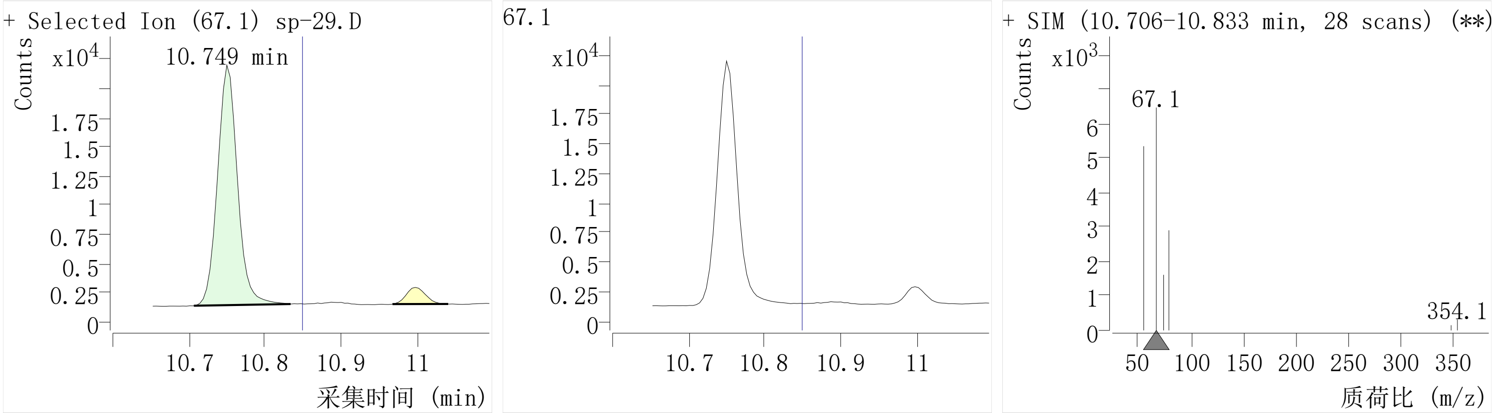

C21:0

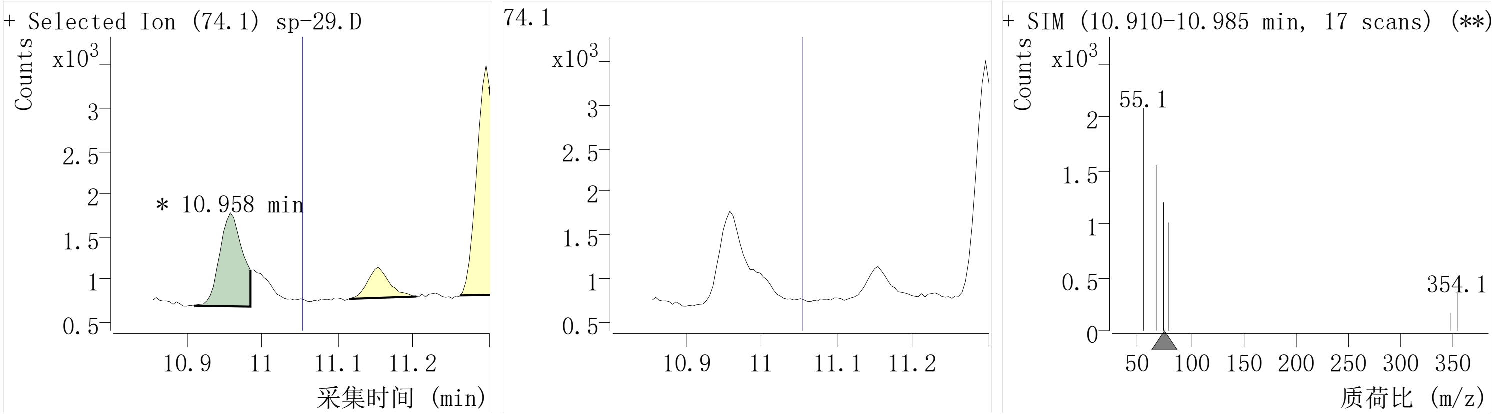

C20:3n6

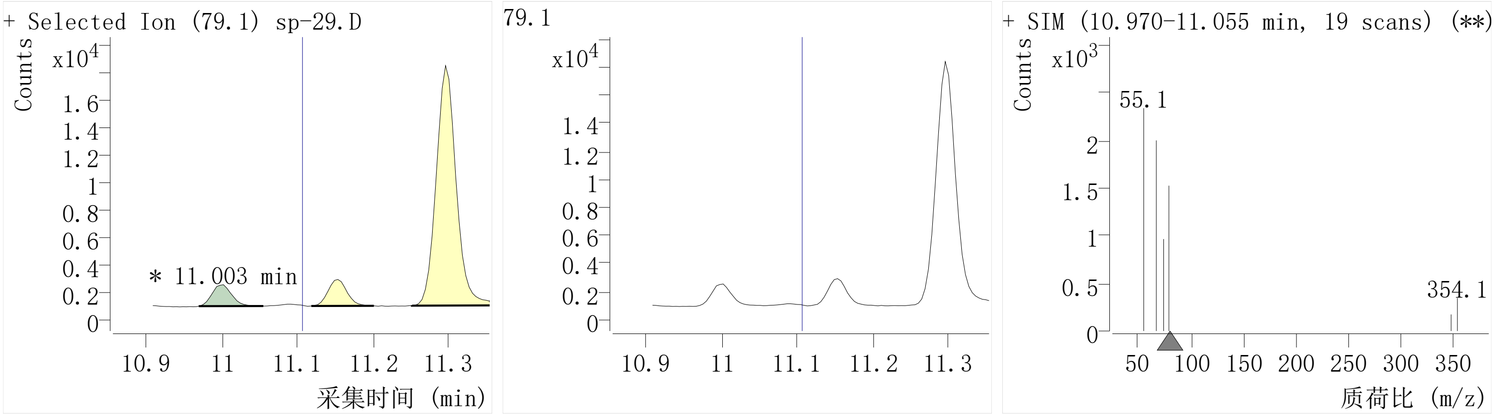

生成时间 2025/5/16 14:52

C20:4n6

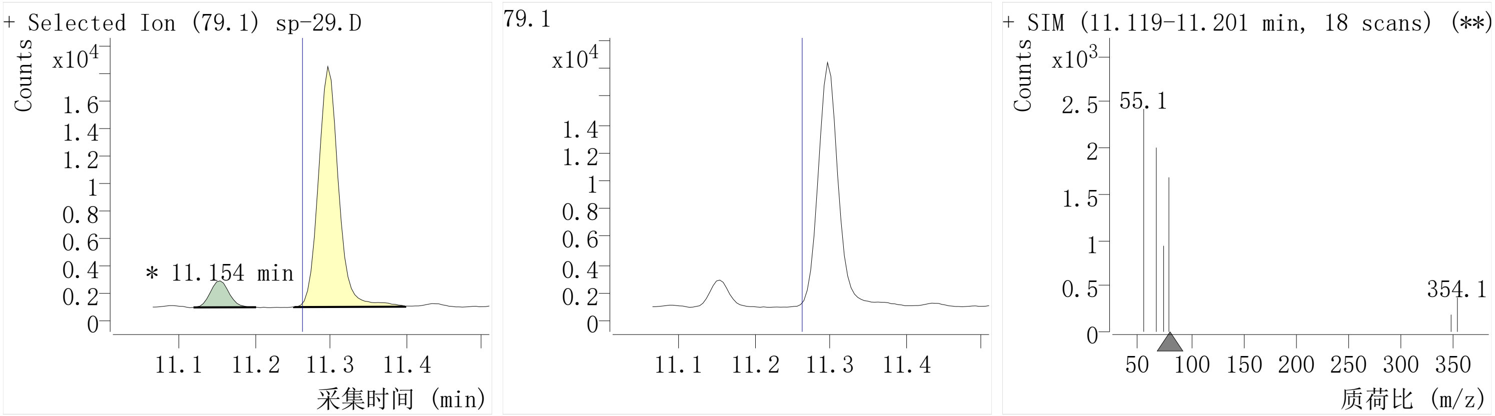

C20:3n3

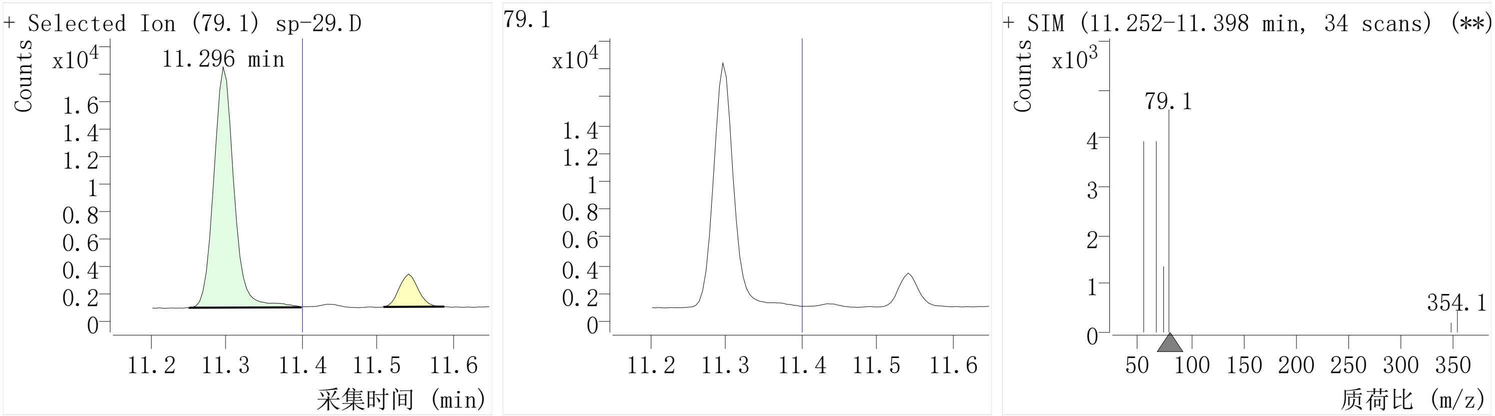

C20:5n3

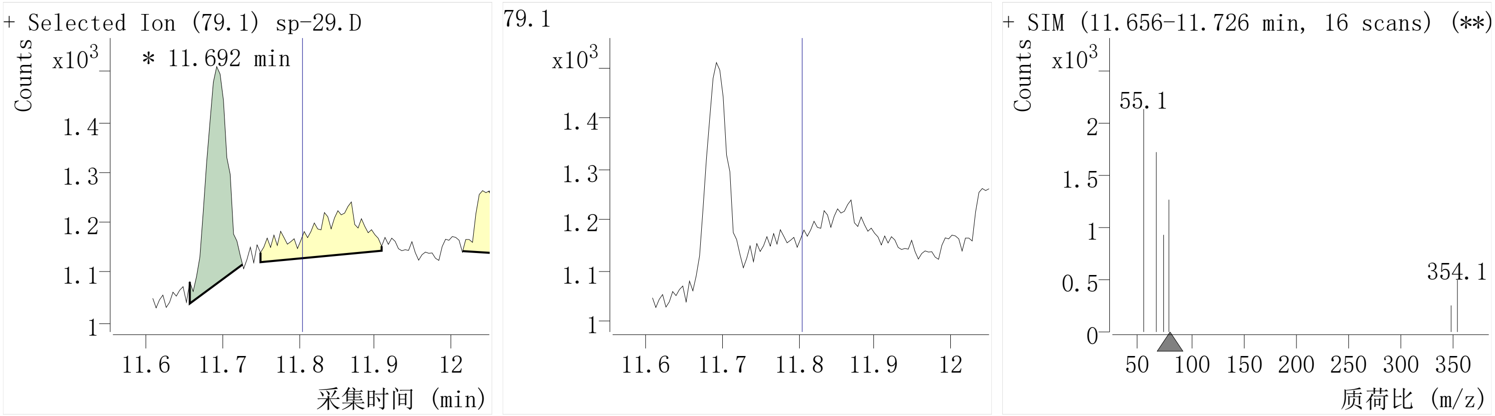

C22:0

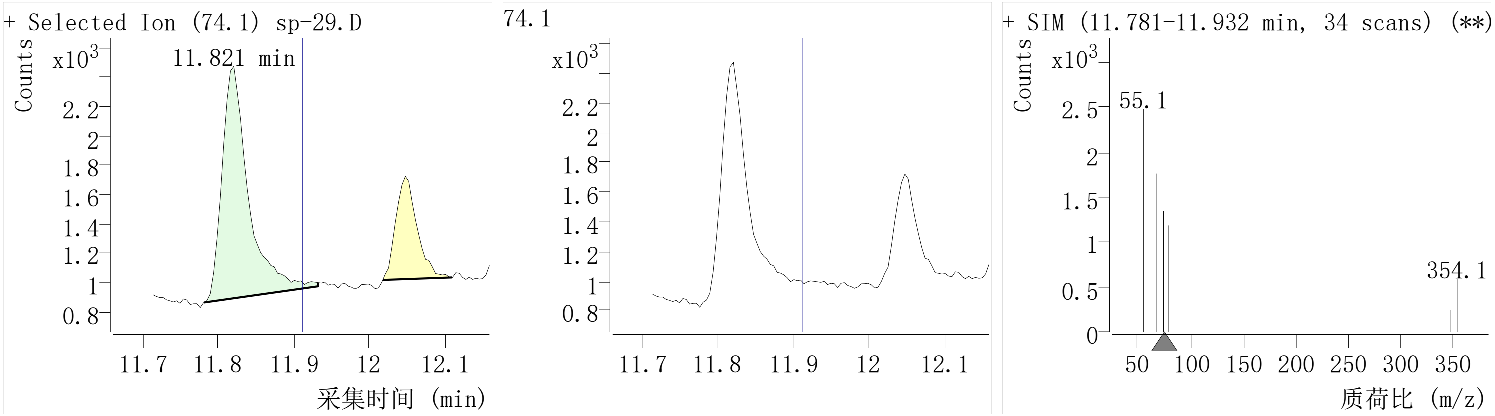

C22:1n9

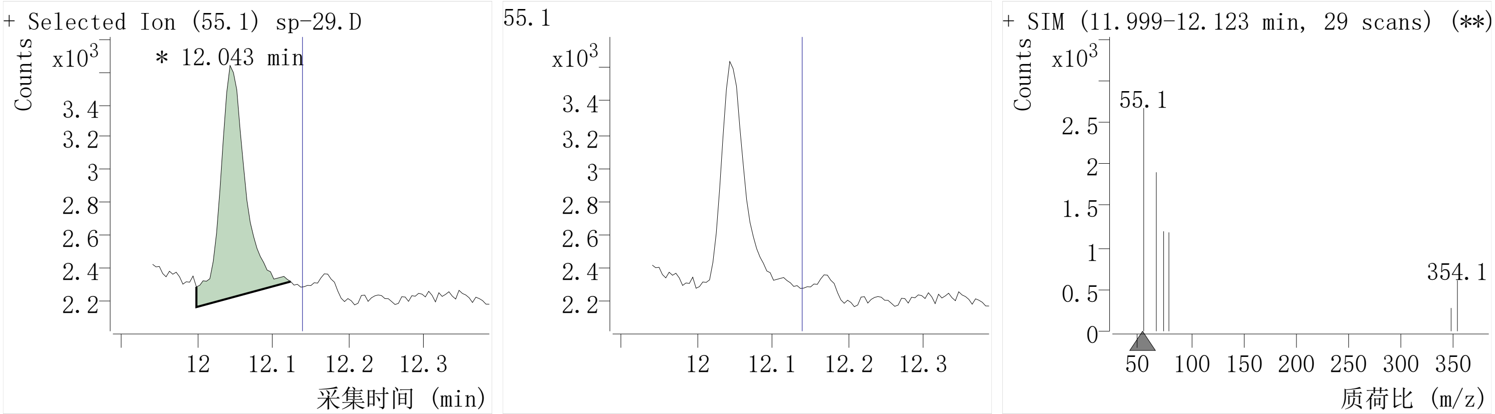

C22:2n6

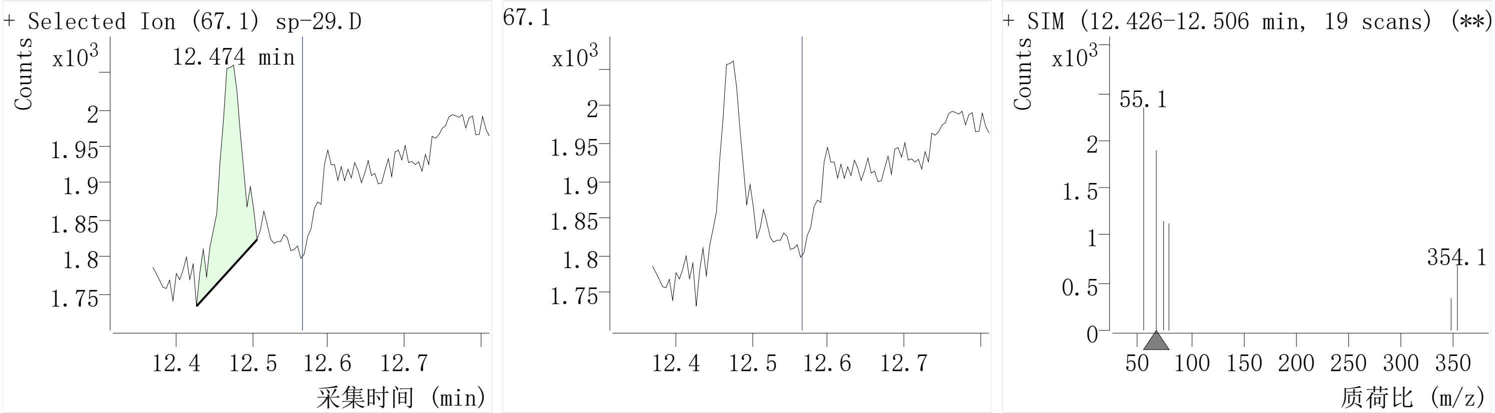

C23:0

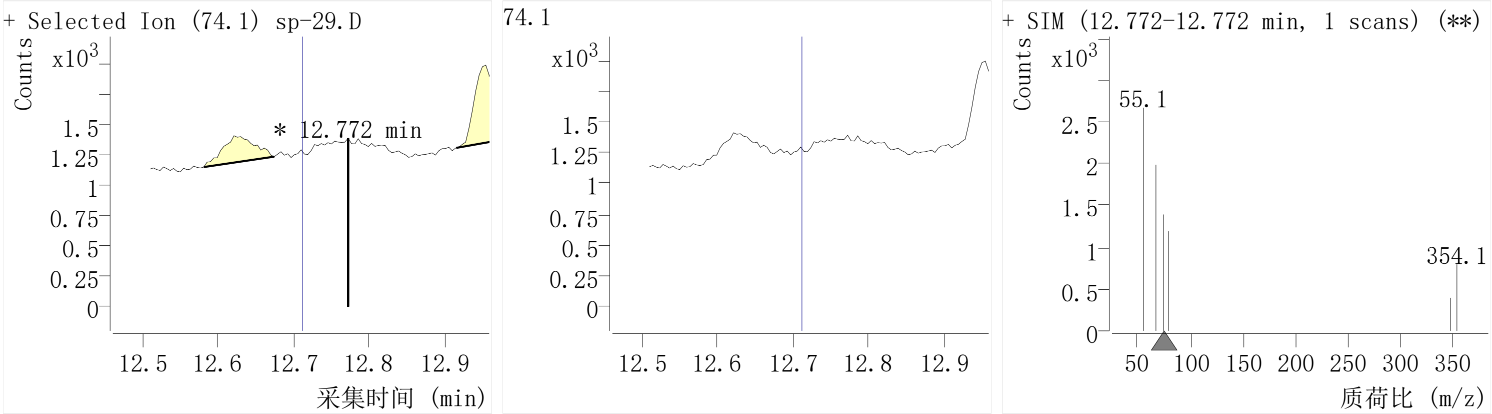

C24:0

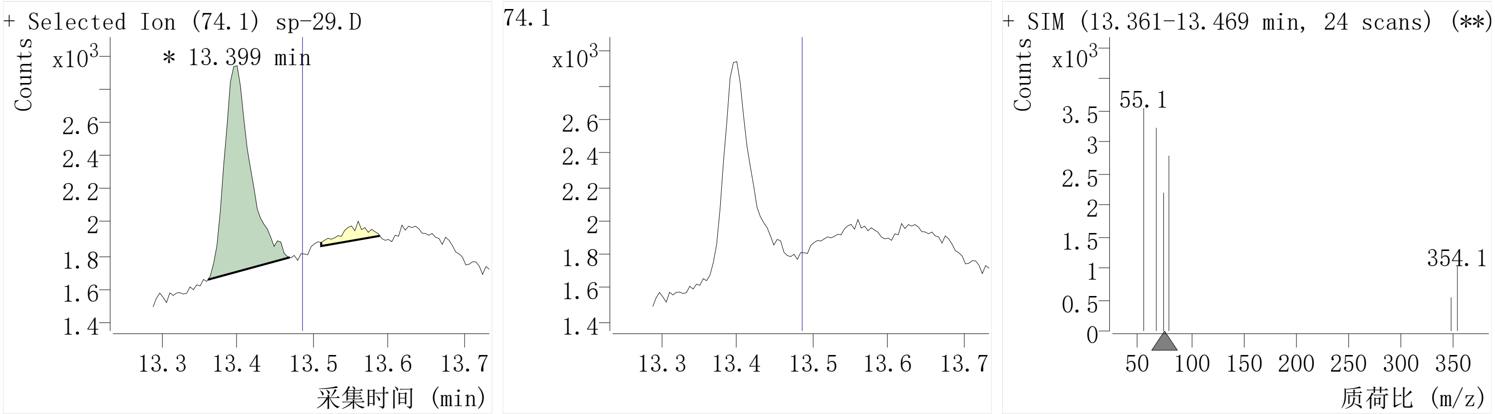

C22:6

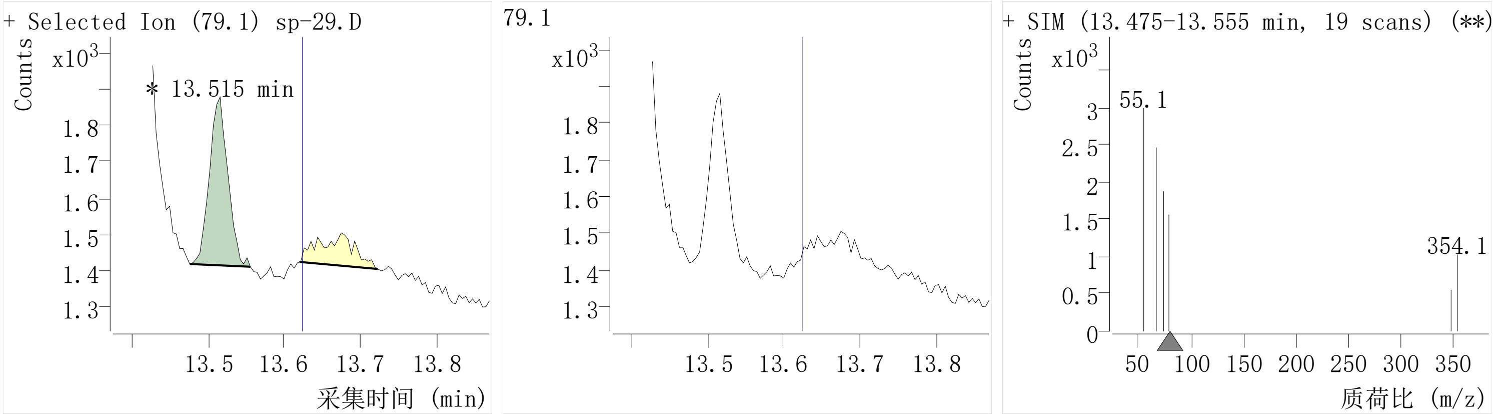
+ Selected Ion (79.1) sp-29.D
Counts
x10^3
* 13.515 min
1.8
1.7
1.6
1.5
1.4
1.3
13.5
13.6
13.7
13.8
采集时间 (min)
79.1
+ SIM (13.475-13.555 min, 19 scans) (**)
55.1
354.1
3
2.5
2
1.5
1
0.5
0
50
100
150
200
250
300
350
质荷比 (m/z)

C24:1

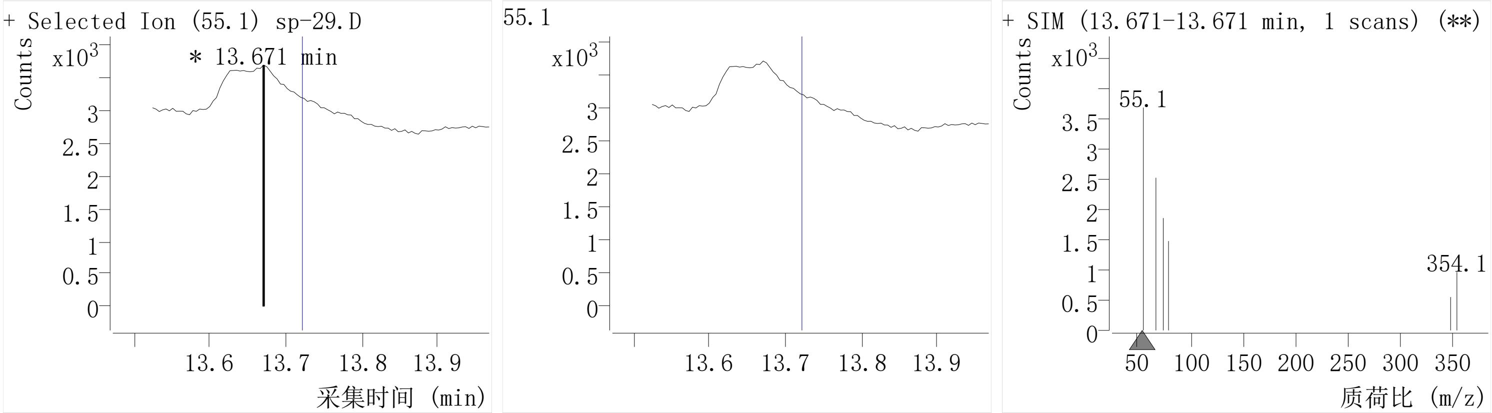
+ Selected Ion (55.1) sp-29.D
Counts
x10^3
* 13.671 min
3
2.5
2
1.5
1
0.5
0
13.6
13.7
13.8
13.9
采集时间 (min)
55.1
+ SIM (13.671-13.671 min, 1 scans) (**)
55.1
354.1
3.5
质荷比 (m/z)

定量分析完成报告

**批处理路径**	G:\GC-MS\HX250430-4-GCMS总脂肪酸靶向检测\HX250430-4\QuantResults\HX250430-4.batch.bin		
**分析时间**	2025/5/14 16:58	**分析员姓名**	DESKTOP-M3A0GPO\omics
**报告时间**	2025/5/16 14:53:35	**报告员姓名**	DESKTOP-M3A0GPO\omics
**最近校正更新**	2025/5/14 16:58	**批处理状态**	已处理
**定量批处理版本**	10.2	**定量报告版本**	10.2

**采集时间**	2025/5/9 8:38	**数据文件**	sp-30.D
**样品类型**	样品	**样品名称**	sp-30
**稀释**	1	**采集方法**	脂肪酸

样品色谱图

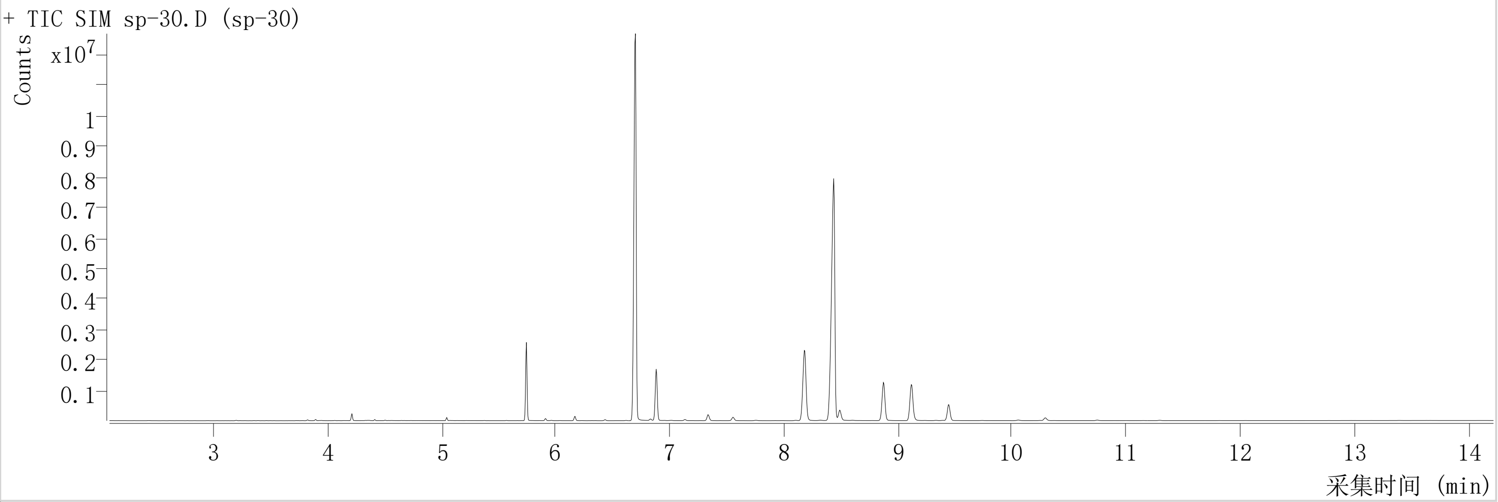

化合物	ISTD	RT	响应	ISTD 响应	响应比	最终浓度	单位
C4:0	C19:0	2.203	186	1720523	0.0001	ND	ug/ml
C6:0	C19:0	2.959	616	1720523	0.0004	0.0038	ug/ml
C8:0	C19:0	3.724	1939	1720523	0.0011	0.0073	ug/ml
C10:0	C19:0	4.413	20693	1720523	0.0120	0.1732	ug/ml
C11:0	C19:0	4.729	962	1720523	0.0006	0.0044	ug/ml
C12:0	C19:0	5.044	59832	1720523	0.0348	0.4942	ug/ml
C13:0	C19:0	5.373	3153	1720523	0.0018	0.0180	ug/ml
C14:0	C19:0	5.742	1749426	1720523	1.0168	21.1619	ug/ml
C14:1	C19:0	5.911	34713	1720523	0.0202	0.9108	ug/ml
C15:0	C19:0	6.169	114566	1720523	0.0666	1.1109	ug/ml
C15:1	C19:0	6.432	0	1720523	0.0000	ND	ug/ml
C16:0	C19:0	6.698	12856234	1720523	7.4723	289.1350	ug/ml
C16:1	C19:0	6.881	990550	1720523	0.5757	35.0949	ug/ml
C17:0	C19:0	7.334	221281	1720523	0.1286	2.6832	ug/ml
C17:1	C19:0	7.552	84984	1720523	0.0494	2.6314	ug/ml
C18:0	C19:0	8.179	3465857	1720523	2.0144	44.2375	ug/ml
C18:1n9t	C19:0	8.321	11567	1720523	0.0067	0.3966	ug/ml
C18:1n9c	C19:0	8.437	7339255	1720523	4.2657	289.4277	ug/ml
C18:2n6t	C19:0	8.873	0	1720523	0.0000	ND	ug/ml
C18:2n6c	C19:0	8.873	1013345	1720523	0.5890	34.4785	ug/ml
C18:3n6	C19:0	9.122	17907	1720523	0.0104	ND	ug/ml
C18:3n3	C19:0	9.446	423696	1720523	0.2463	12.2374	ug/ml
C20:0	C19:0	10.056	34597	1720523	0.0201	0.5412	ug/ml
C20:1	C19:0	10.291	91545	1720523	0.0532	3.3188	ug/ml
C20:2	C19:0	10.749	16295	1720523	0.0095	0.6336	ug/ml
C21:0	C19:0	10.958	2094	1720523	0.0012	0.0337	ug/ml
C20:3n6	C19:0	11.003	1420	1720523	0.0008	0.0963	ug/ml
C20:4n6	C19:0	11.154	2501	1720523	0.0015	0.1259	ug/ml
C20:3n3	C19:0	11.296	10797	1720523	0.0063	0.3791	ug/ml
C20:5n3	C19:0	11.696	433	1720523	0.0003	0.0491	ug/ml

化合物	ISTD	RT	响应	ISTD 响应	响应比	最终浓度	单位
C22:0	C19:0	11.816	4086	1720523	0.0024	0.0972	ug/ml
C22:1n9	C19:0	12.048	2339	1720523	0.0014	0.0859	ug/ml
C22:2n6	C19:0	12.479	318	1720523	0.0002	0.0516	ug/ml
C23:0	C19:0	12.755	0	1720523	0.0000	ND	ug/ml
C24:0	C19:0	13.395	1121	1720523	0.0007	0.0357	ug/ml
C22:6	C19:0	13.506	403	1720523	0.0002	0.0225	ug/ml
C24:1	C19:0	13.671	0	1720523	0.0000	ND	ug/ml

C4:0

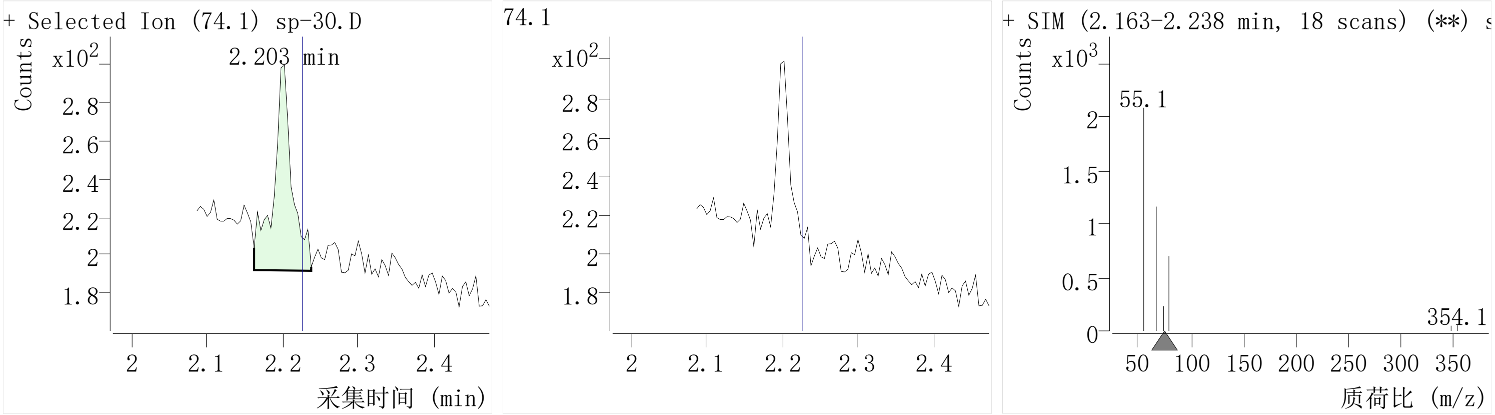

C6:0

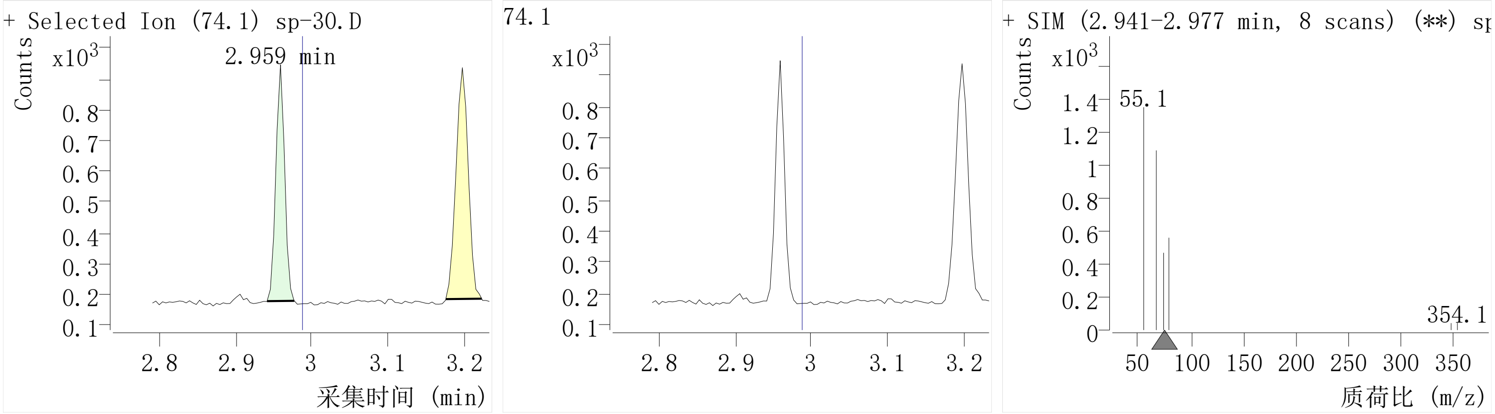

C8:0

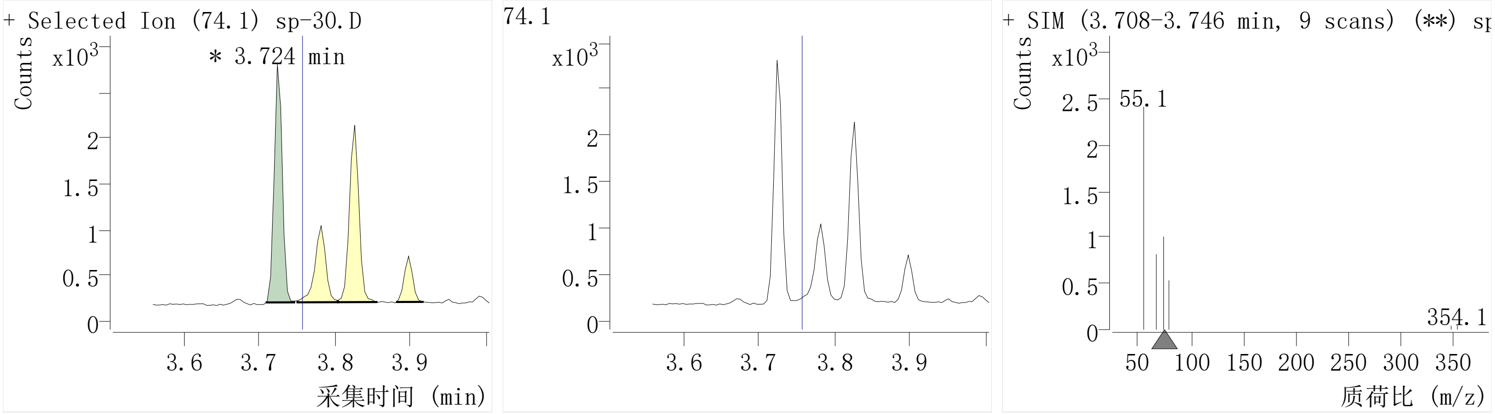

C10:0

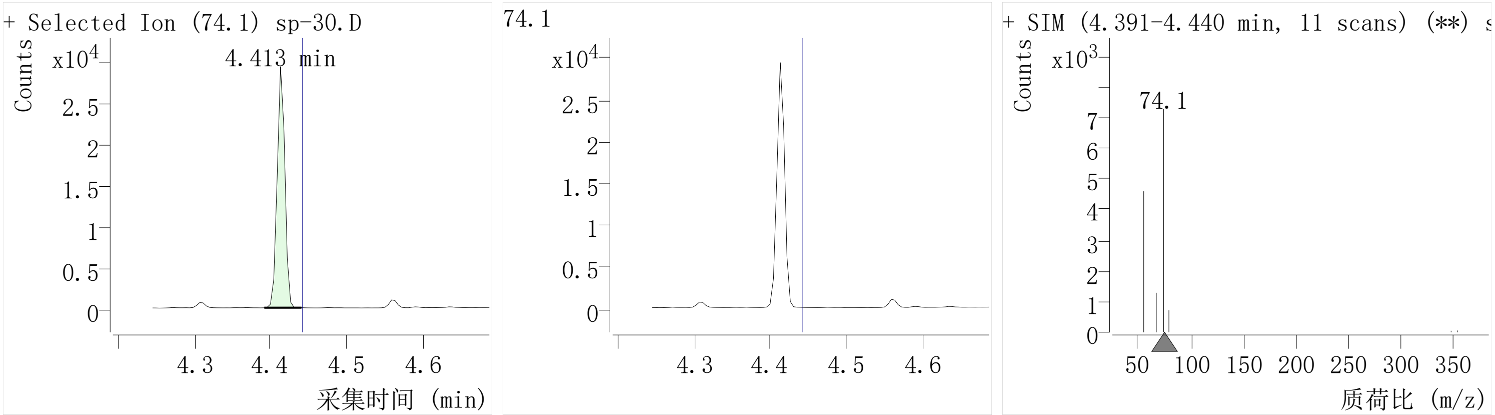

C11:0

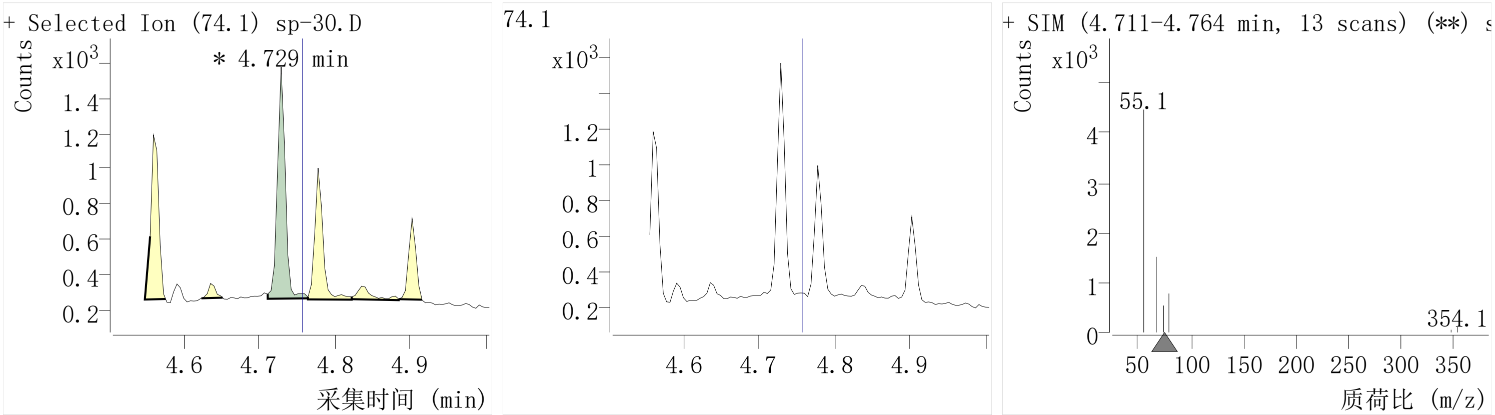

C12:0

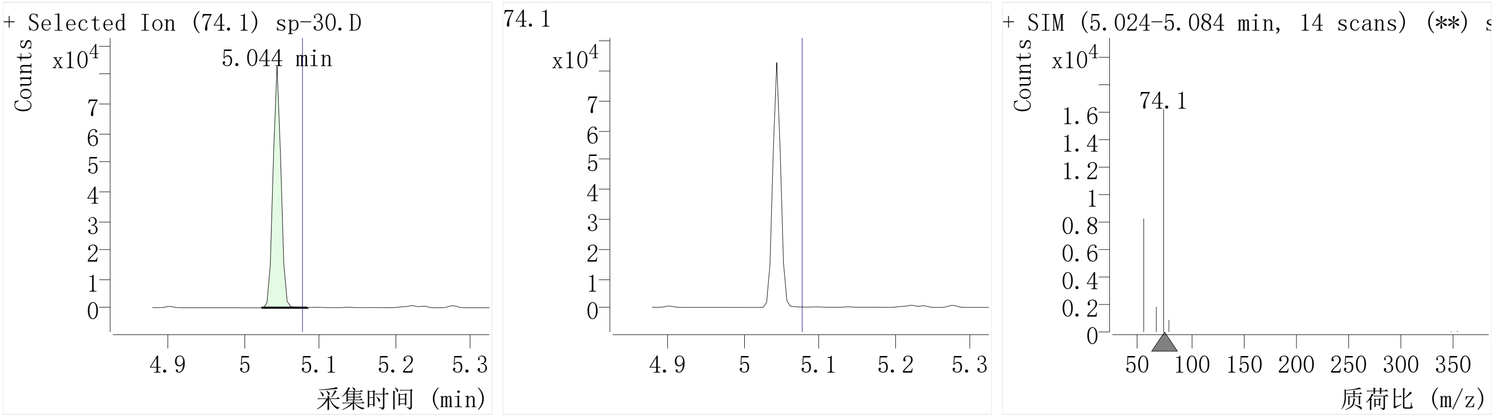

C13:0

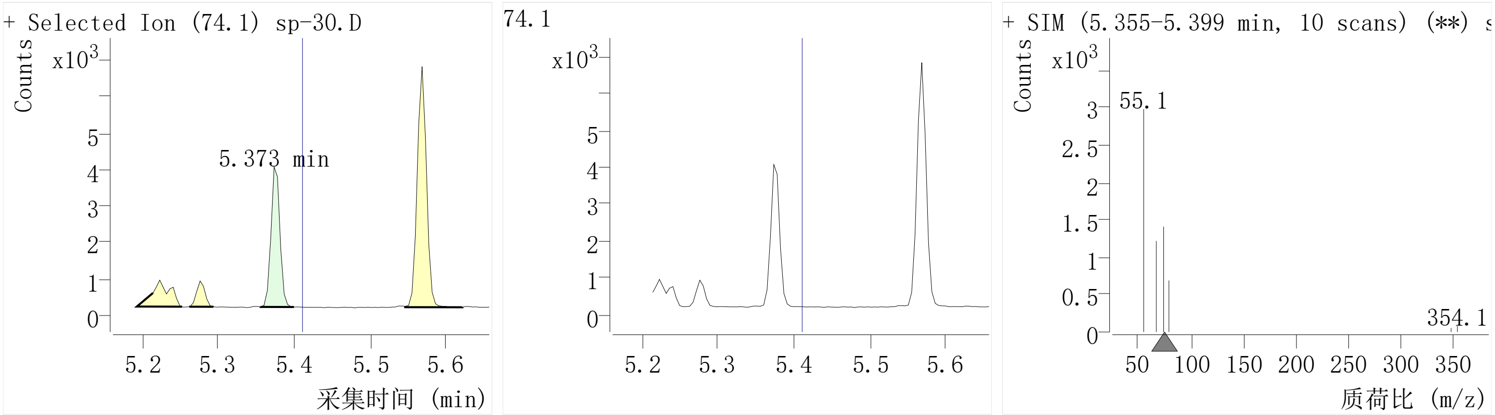

C14:0

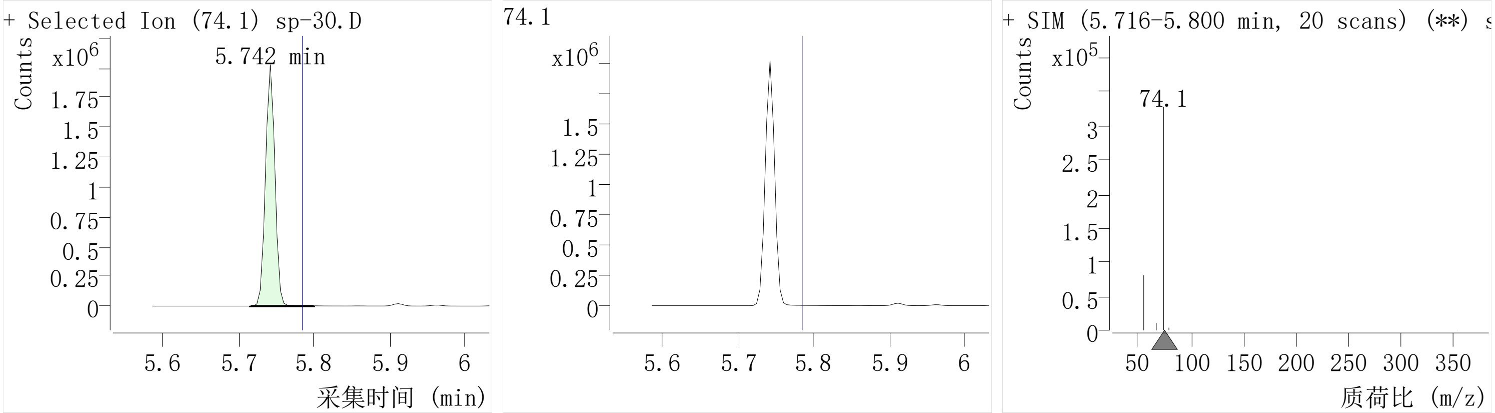

C14:1

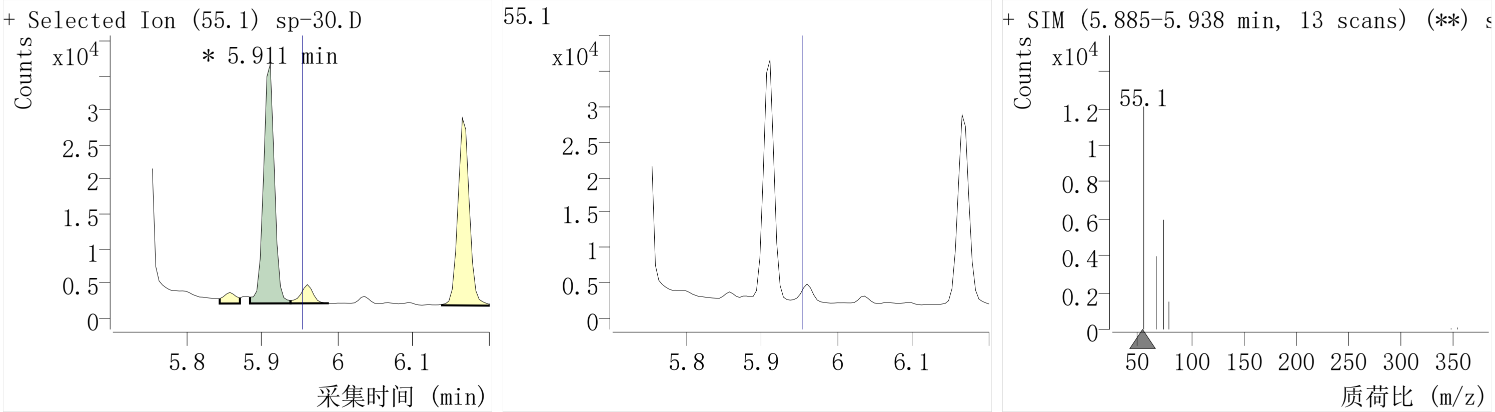

C15:0

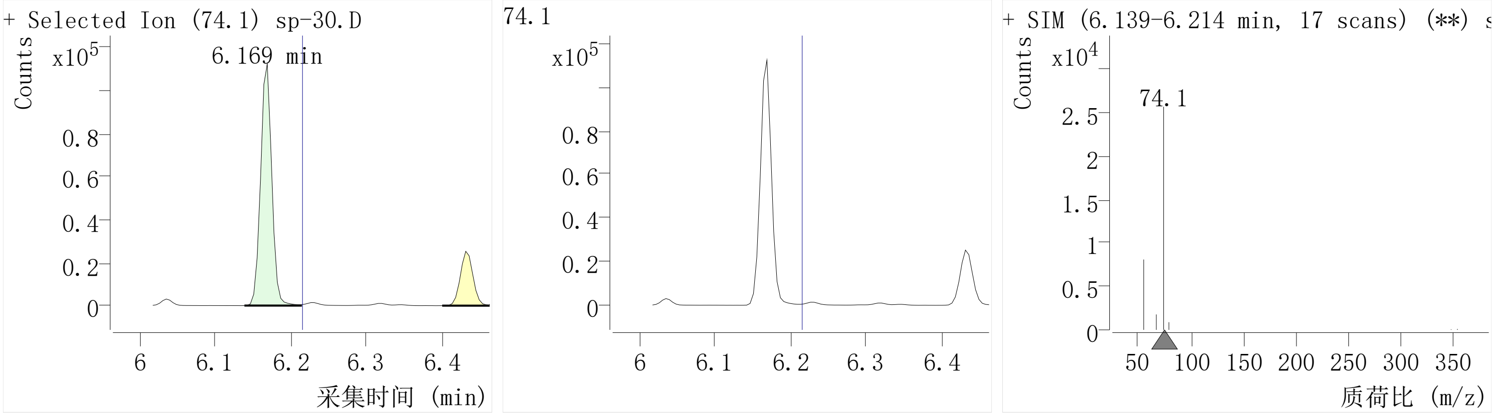

C15:1

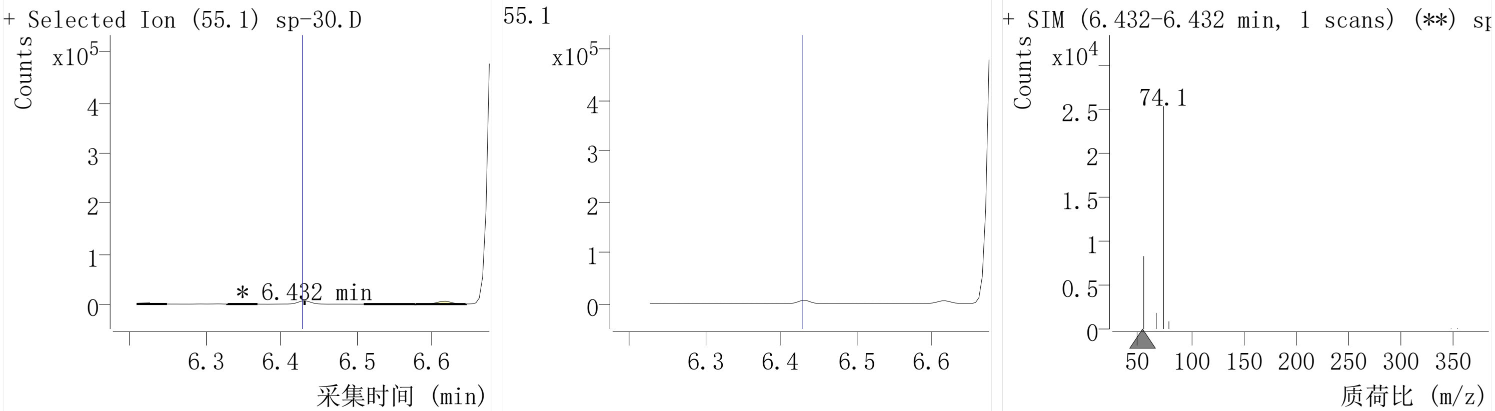

C16:0

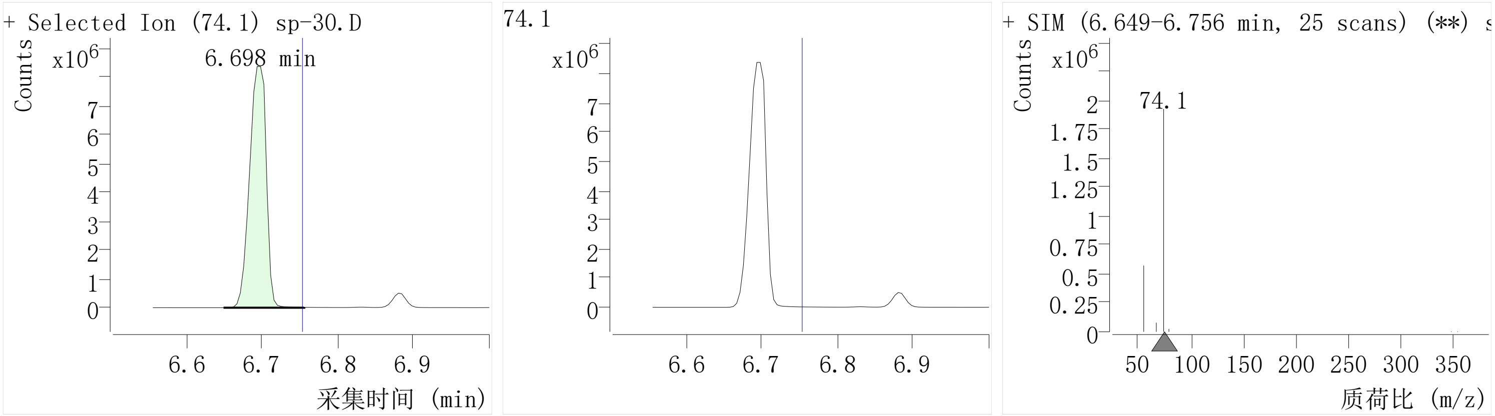

C16:1

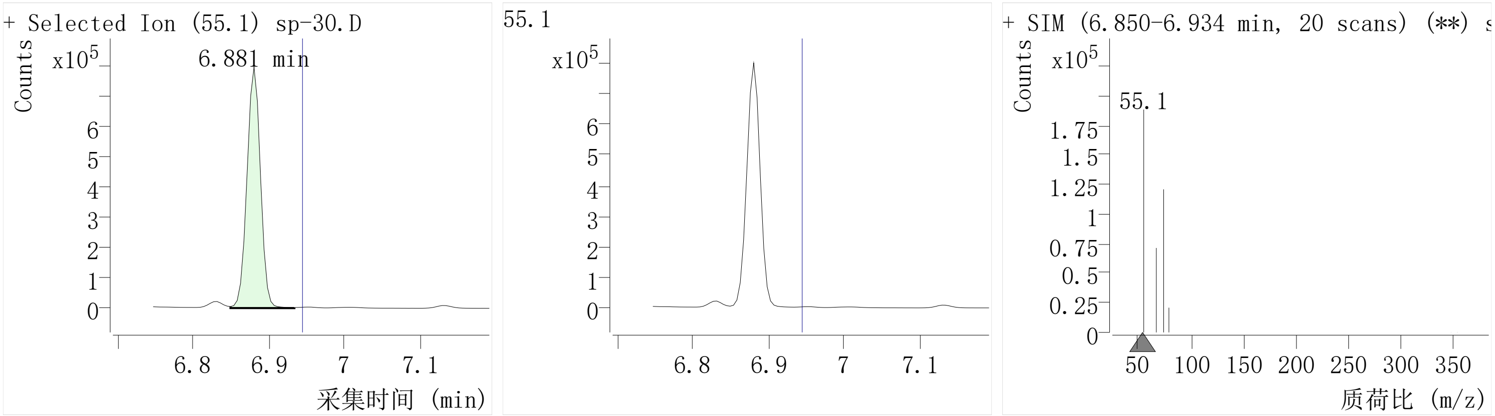

C17:0

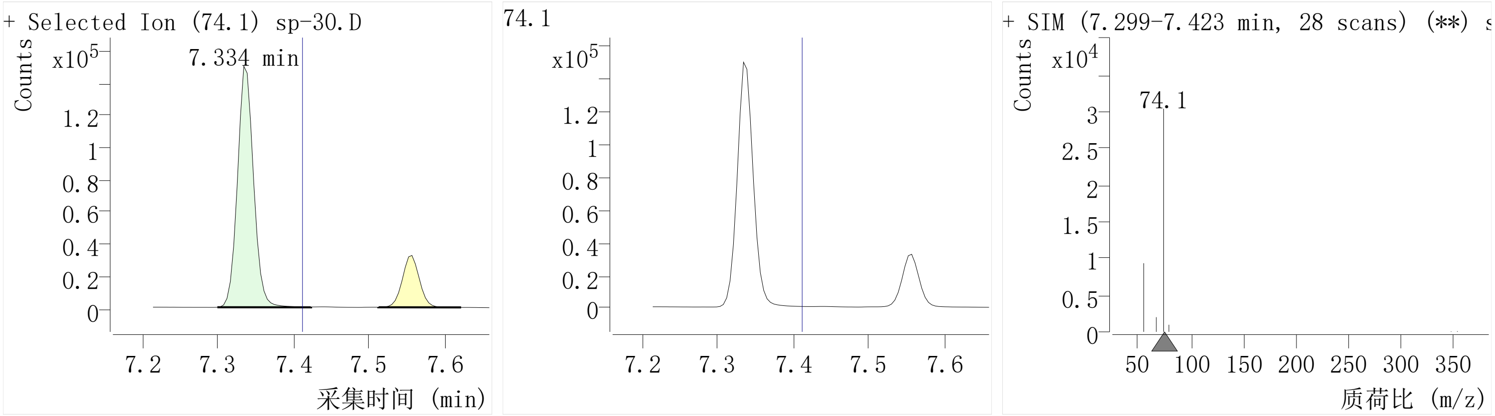

C17:1

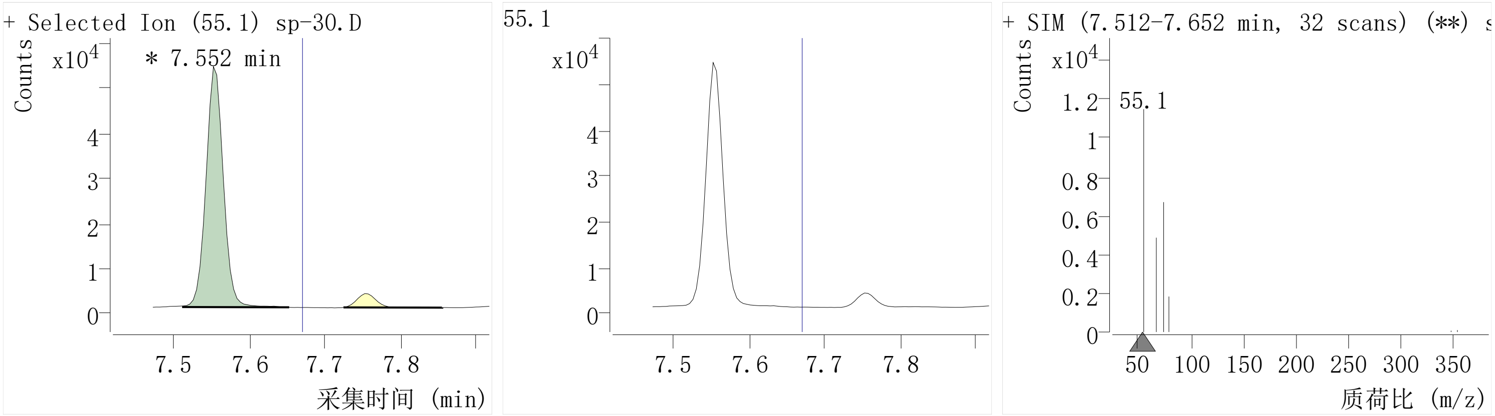

C18:0

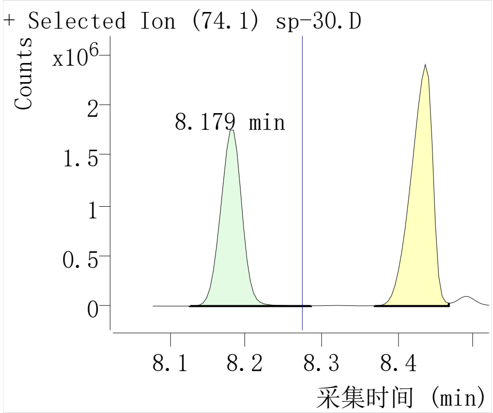

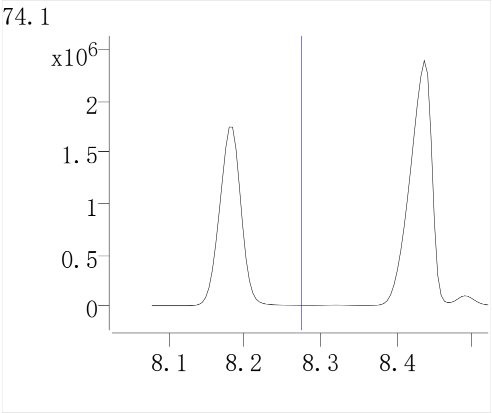

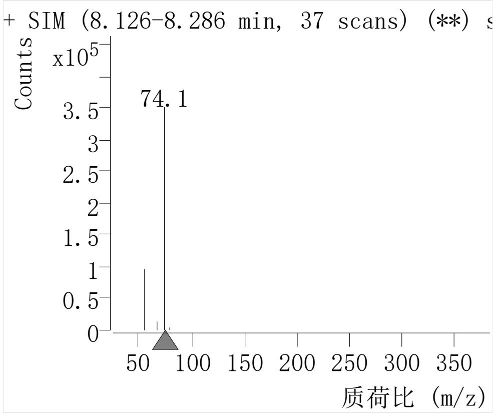

C18:1n9t

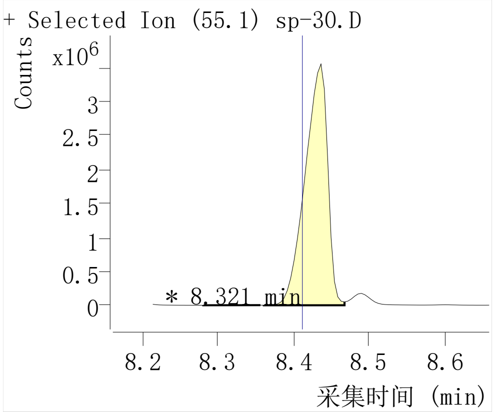

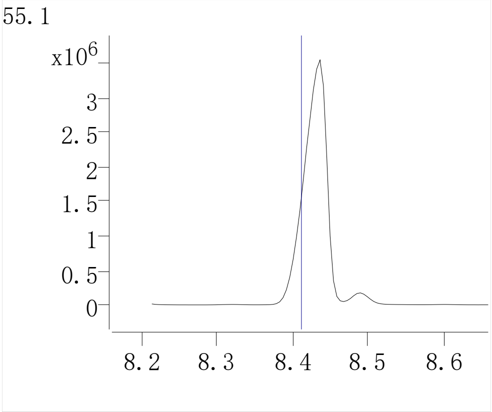

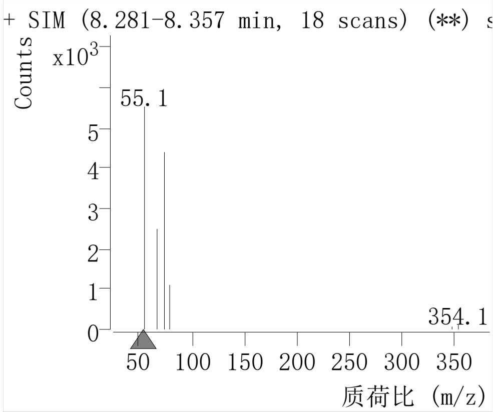

C18:1n9c

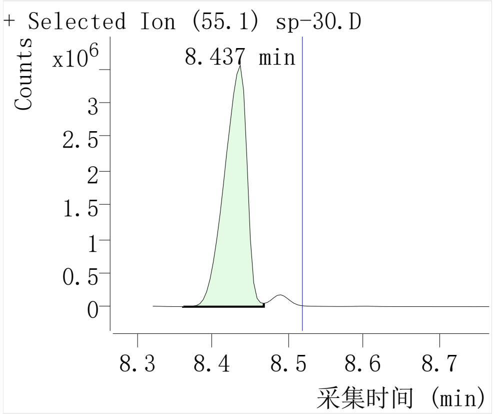

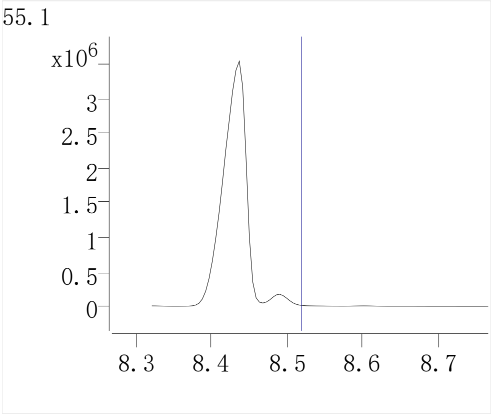

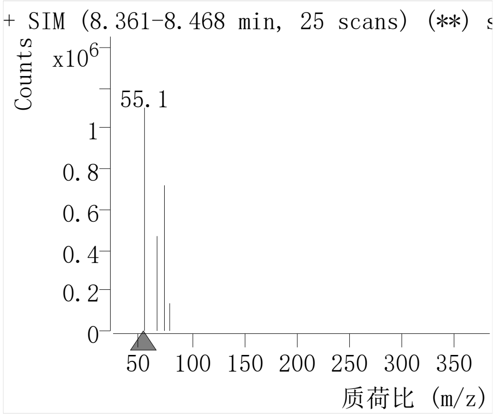

C18:2n6t

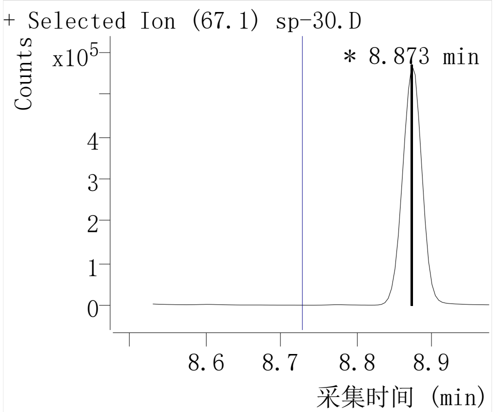

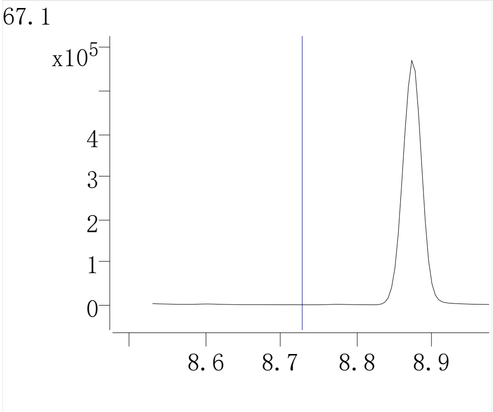

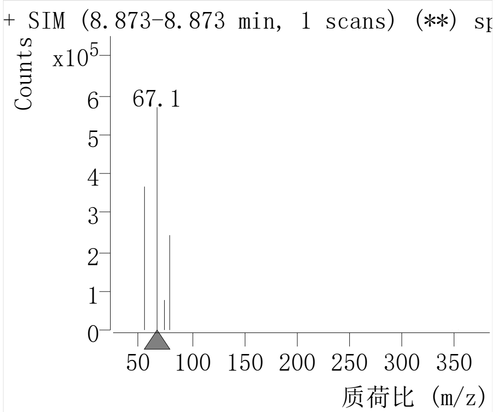

C18:2n6c

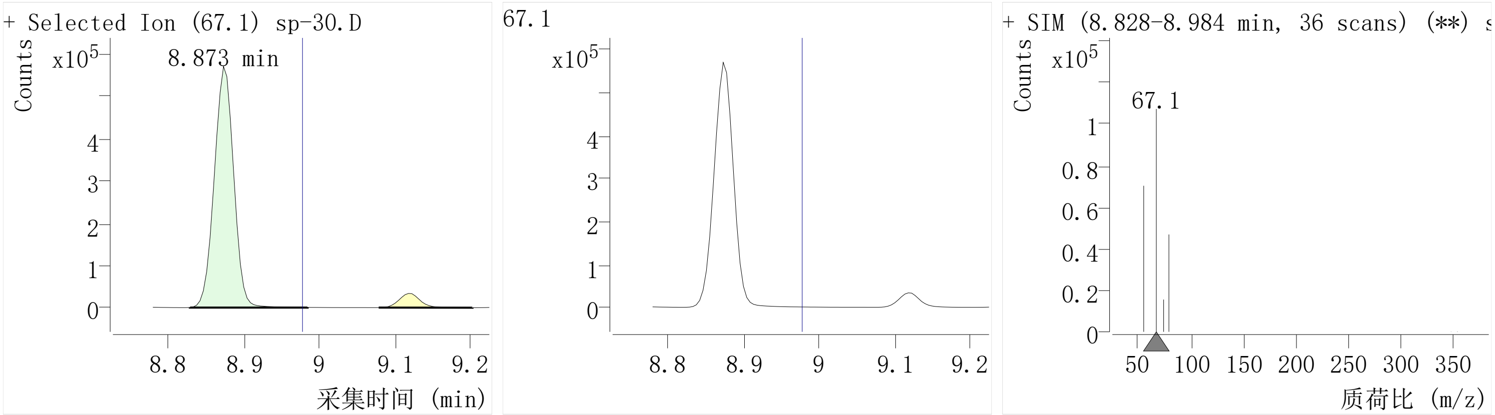

C18:3n6

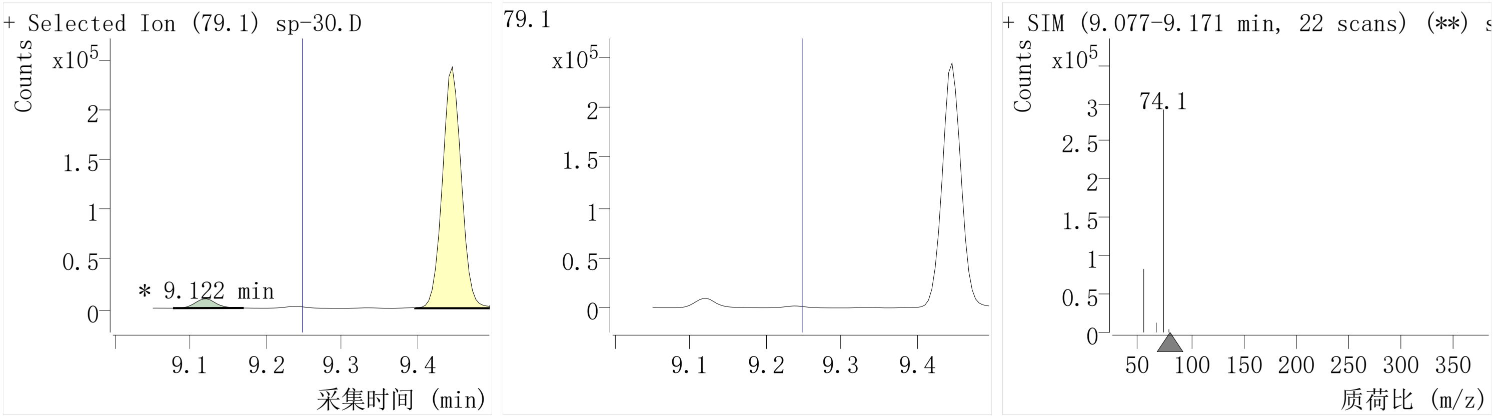

C18:3n3

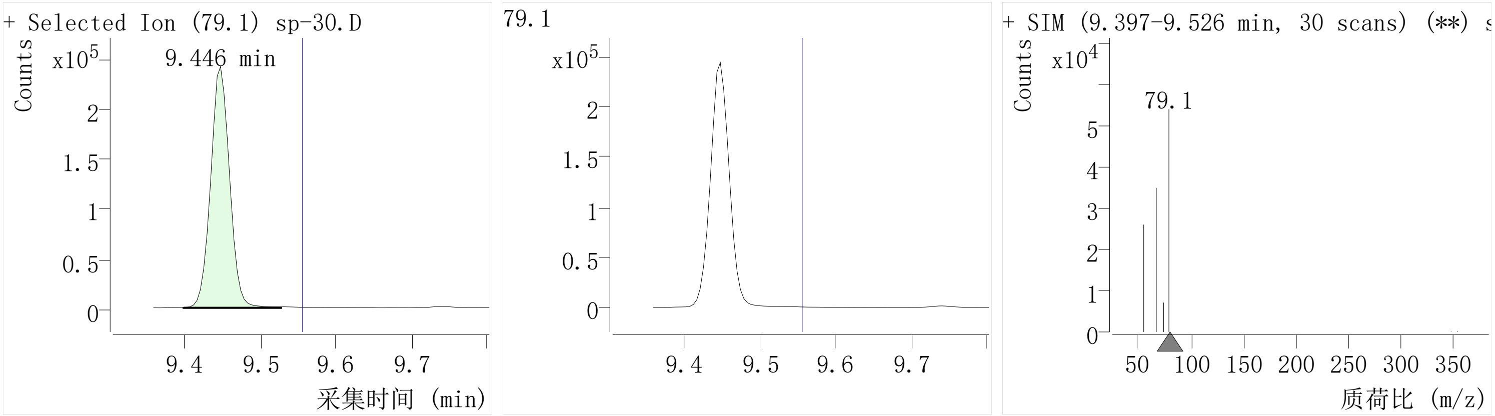

C20:0

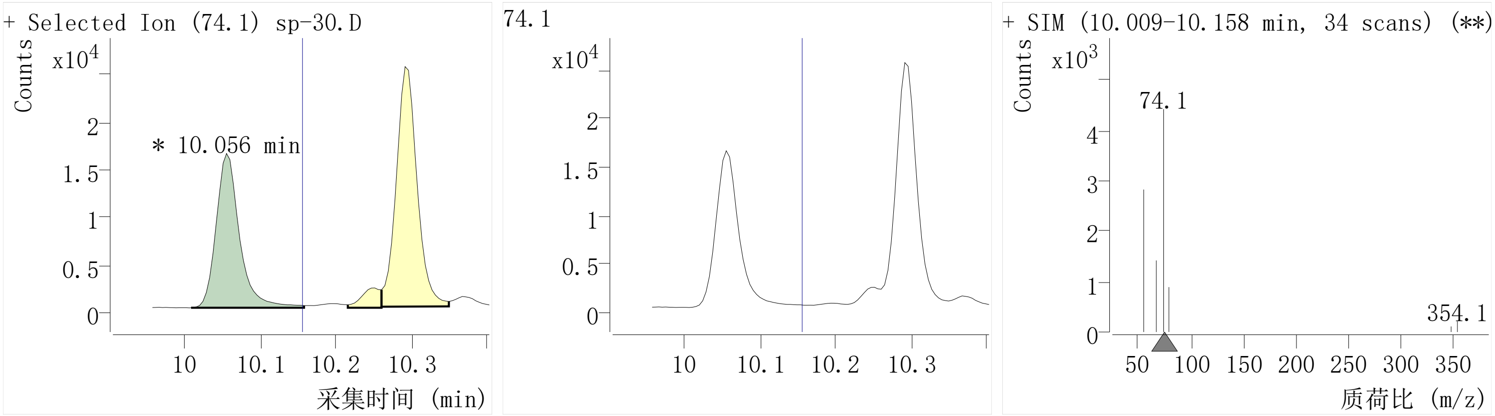

 生成时间 2025/5/16 14:52

C20:1

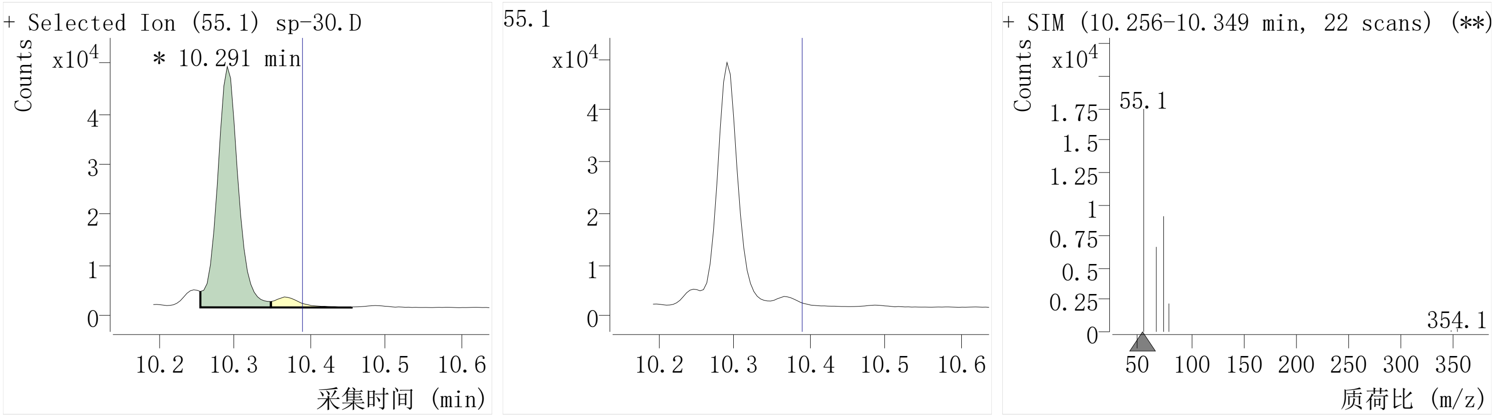

C20:2

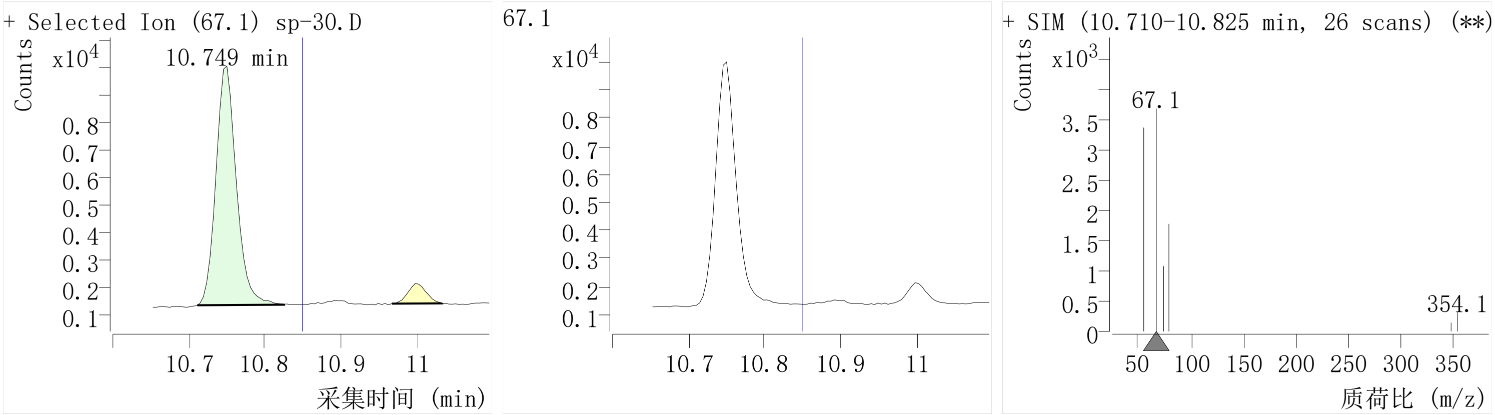

C21:0

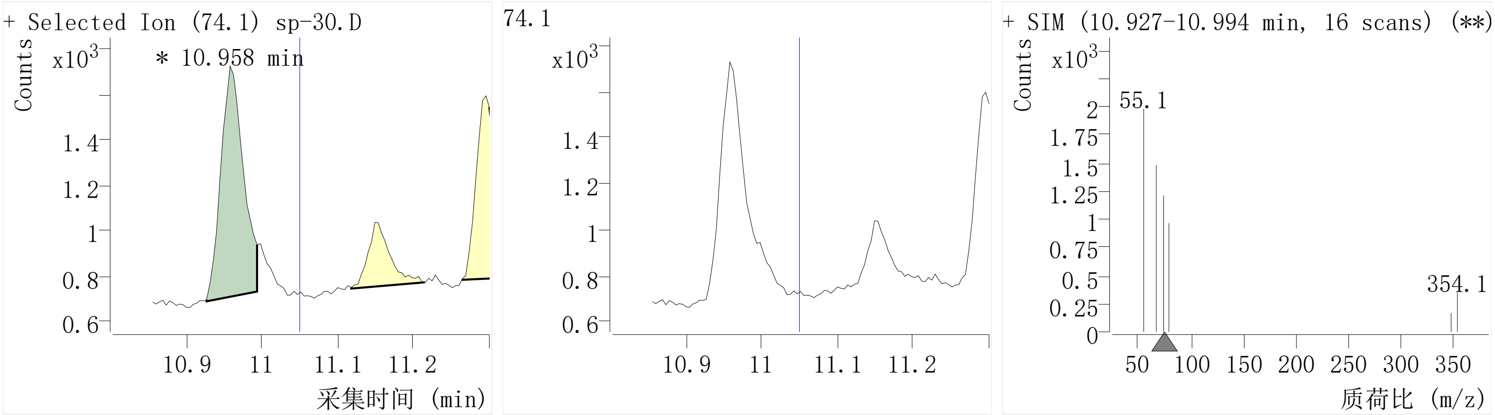

C20:3n6

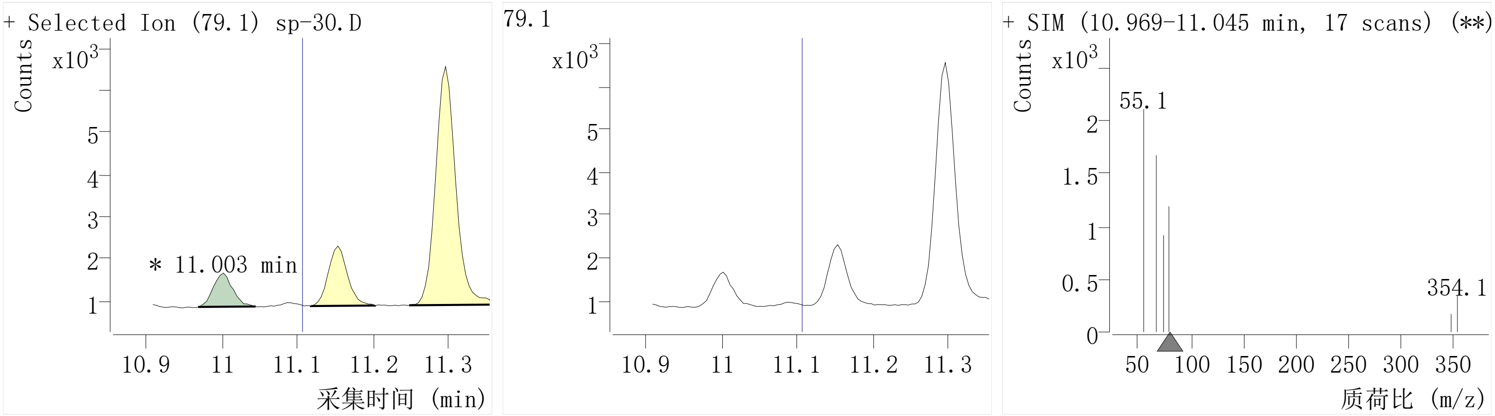

C20:4n6

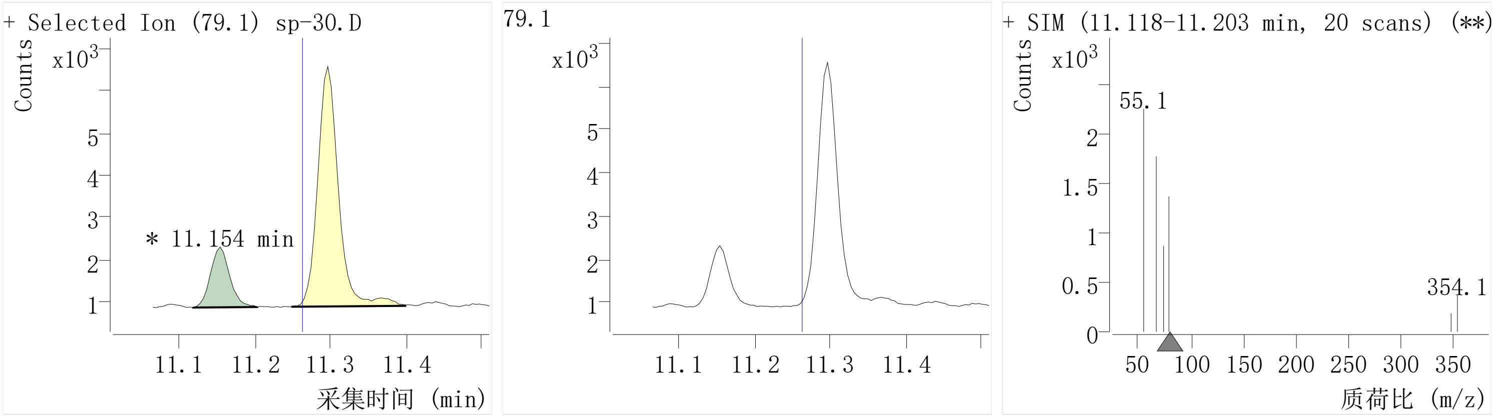

C20:3n3

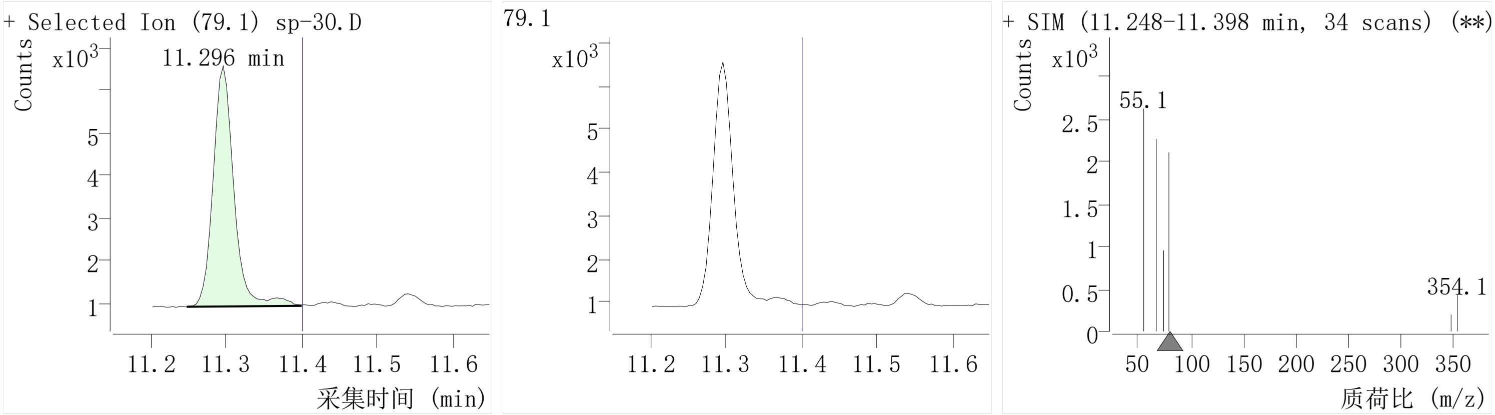

C20:5n3

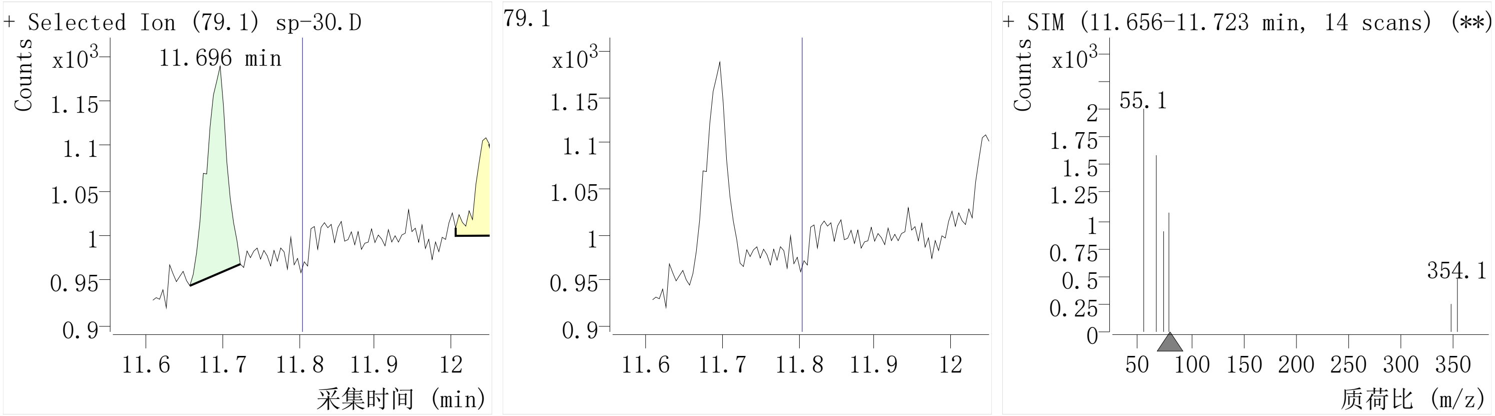

C22:0

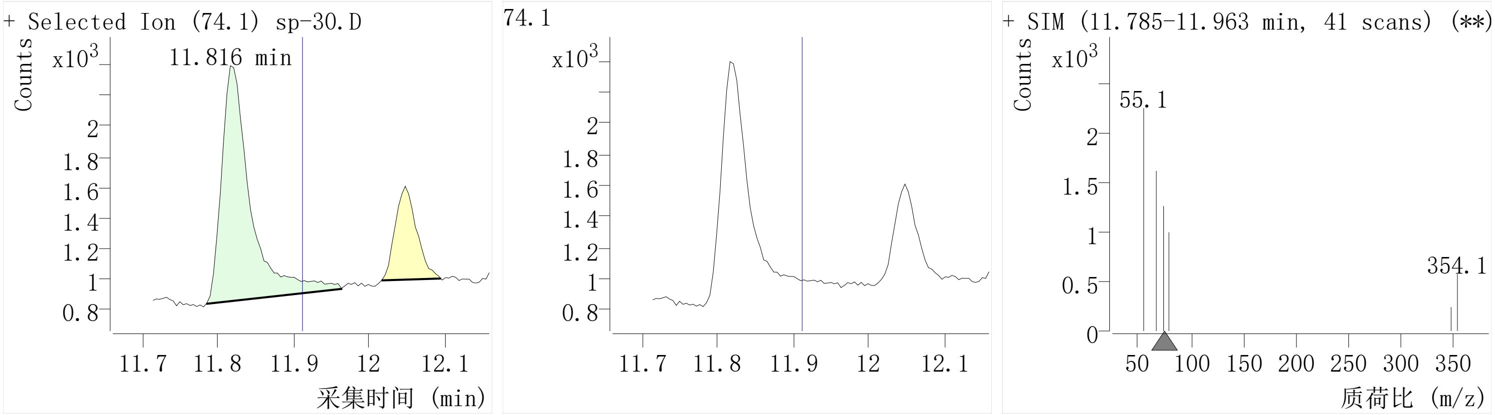

C22:1n9

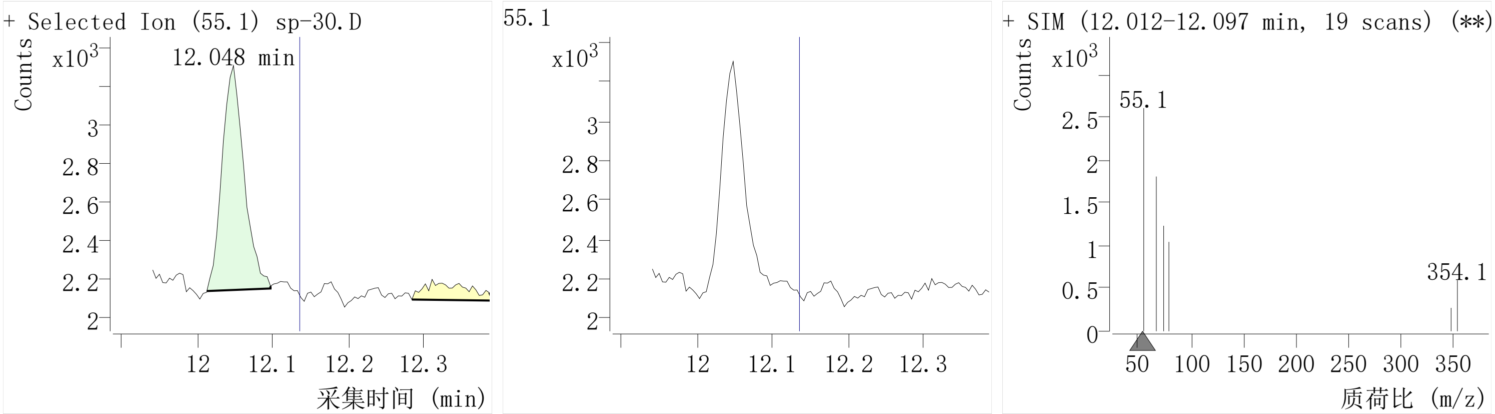

C22:2n6

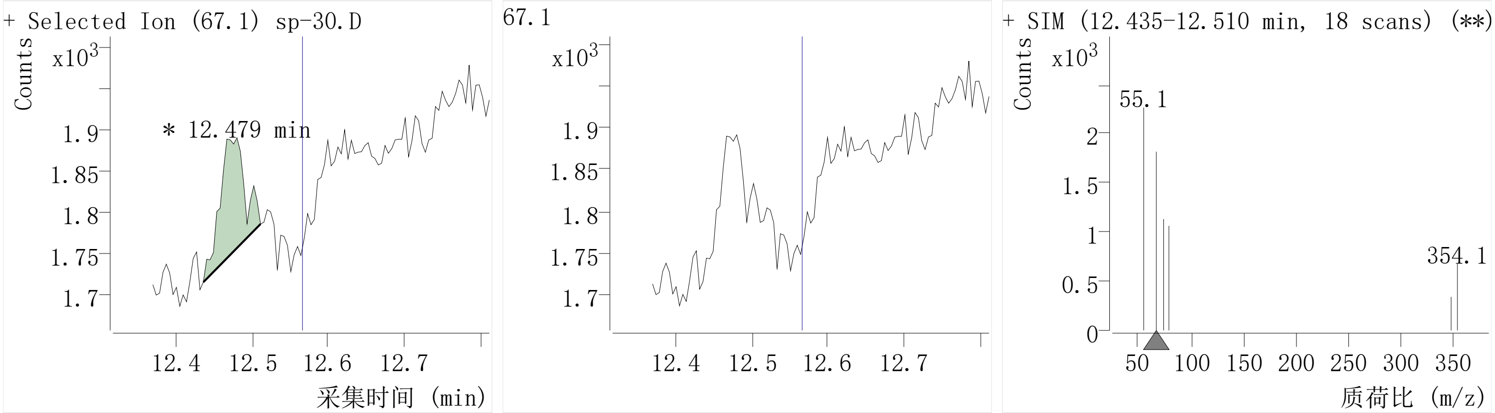

C23:0

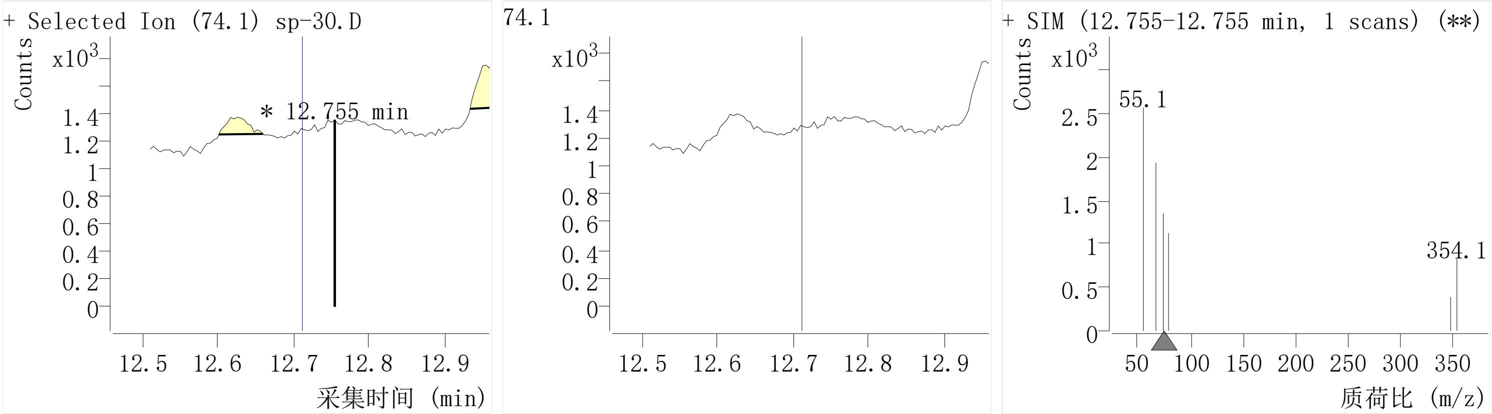

C24:0

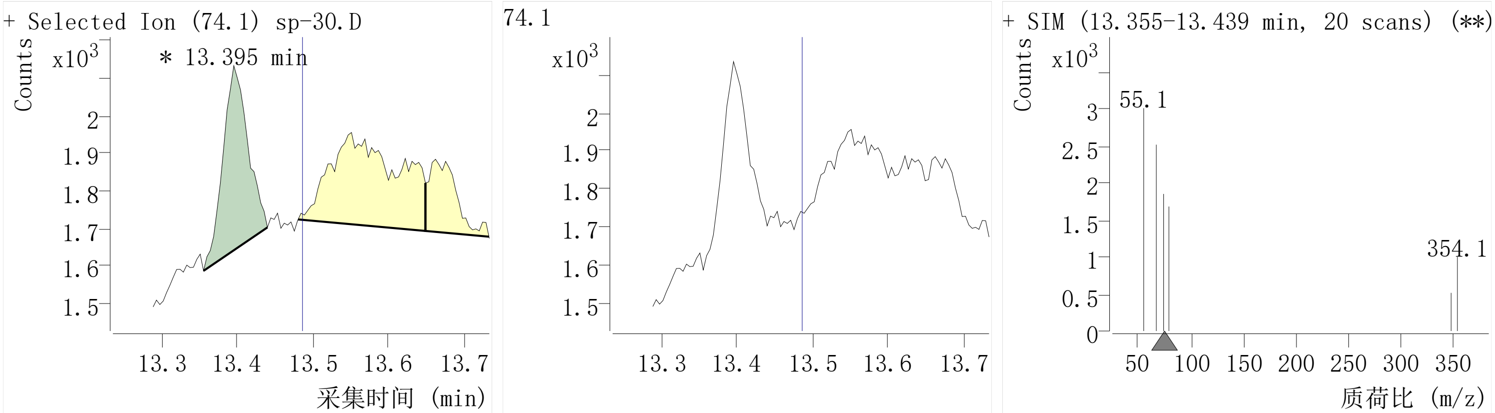

C22:6

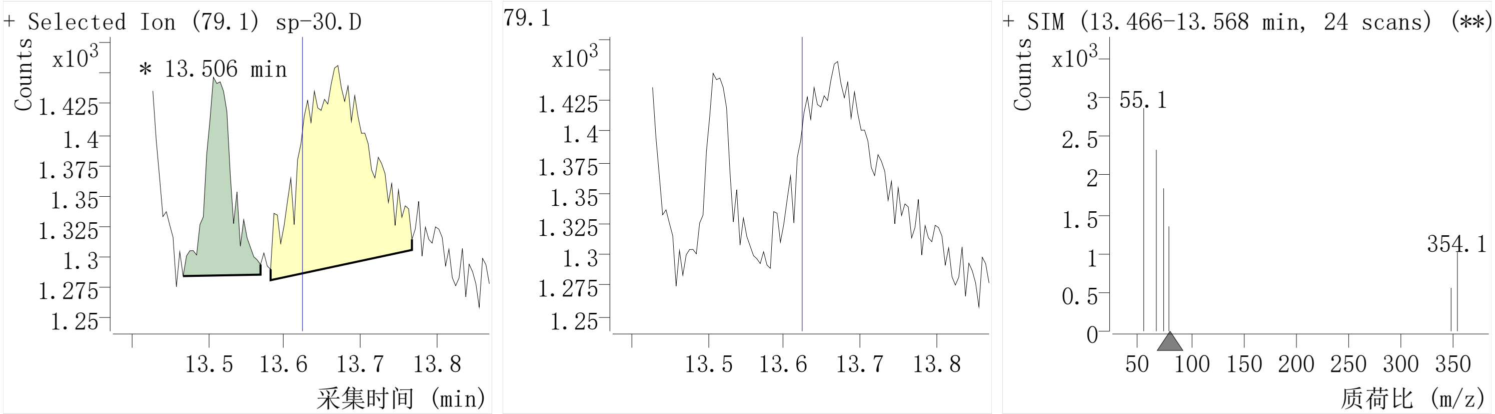
+ Selected Ion (79.1) sp-30.D
Counts
x10^3
* 13.506 min
采集时间 (min)
79.1
+ SIM (13.466-13.568 min, 24 scans) (**)
55.1
354.1
质荷比 (m/z)

C24:1

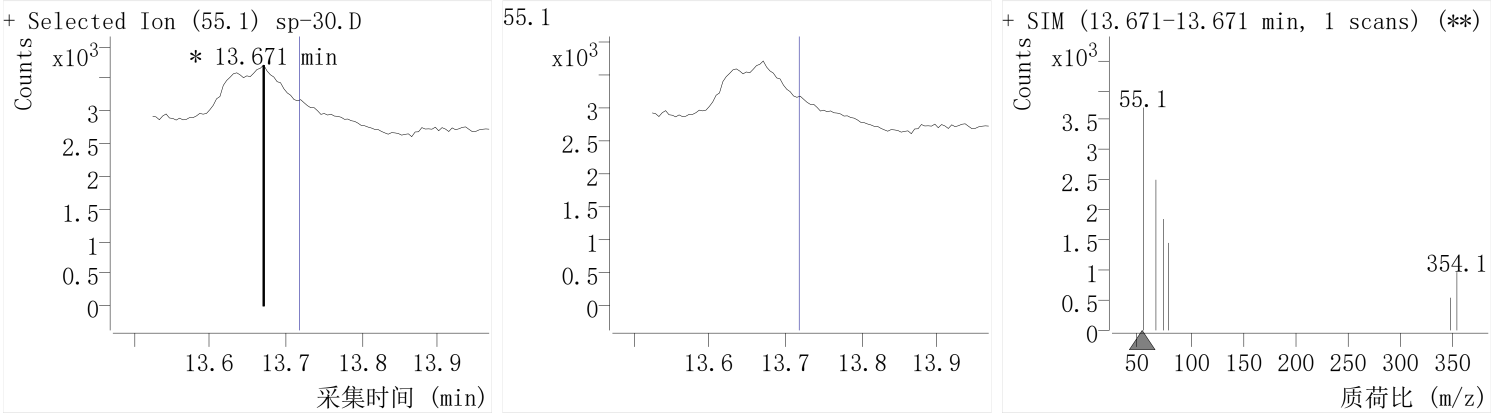
+ Selected Ion (55.1) sp-30.D
Counts
x10^3
* 13.671 min
采集时间 (min)
55.1
+ SIM (13.671-13.671 min, 1 scans) (**)
55.1
354.1
质荷比 (m/z)

**批处理路径**	G:\GC-MS\HX250430-4-GCMS总脂肪酸靶向检测\HX250430-4\QuantResults\HX250430-4.batch.bin		
**分析时间**	2025/5/14 16:58	**分析员姓名**	DESKTOP-M3A0GPO\omics
**报告时间**	2025/5/16 14:53:37	**报告员姓名**	DESKTOP-M3A0GPO\omics
**最近校正更新**	2025/5/14 16:58	**批处理状态**	已处理
**定量批处理版本**	10.2	**定量报告版本**	10.2

**采集时间**	2025/5/9 8:58	**数据文件**	sp-31.D
**样品类型**	样品	**样品名称**	sp-31
**稀释**	1	**采集方法**	脂肪酸

样品色谱图

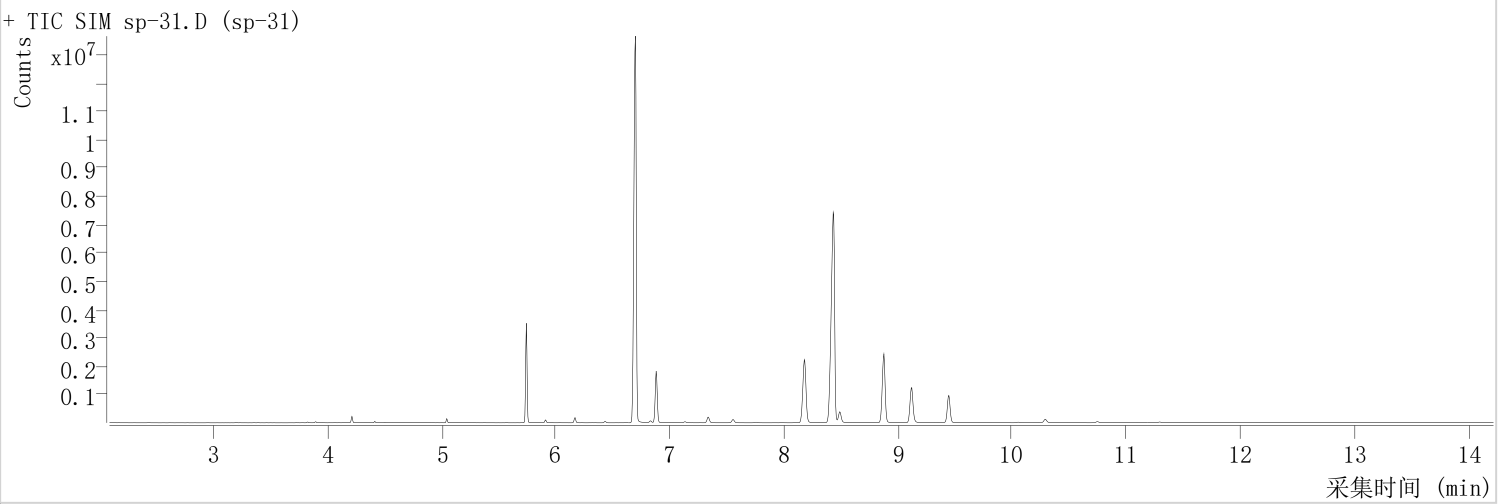

化合物	ISTD	RT	响应	ISTD 响应	响应比	最终浓度	单位
C4:0	C19:0	2.198	208	1756510	0.0001	ND	ug/ml
C6:0	C19:0	2.959	656	1756510	0.0004	0.0040	ug/ml
C8:0	C19:0	3.724	2183	1756510	0.0012	0.0084	ug/ml
C10:0	C19:0	4.413	26880	1756510	0.0153	0.2333	ug/ml
C11:0	C19:0	4.728	1278	1756510	0.0007	0.0060	ug/ml
C12:0	C19:0	5.044	84086	1756510	0.0479	0.7255	ug/ml
C13:0	C19:0	5.373	4051	1756510	0.0023	0.0236	ug/ml
C14:0	C19:0	5.742	2391768	1756510	1.3617	30.1402	ug/ml
C14:1	C19:0	5.911	49955	1756510	0.0284	1.3277	ug/ml
C15:0	C19:0	6.169	145188	1756510	0.0827	1.4137	ug/ml
C15:1	C19:0	6.432	0	1756510	0.0000	ND	ug/ml
C16:0	C19:0	6.696	13954516	1756510	7.9445	314.9363	ug/ml
C16:1	C19:0	6.881	1075159	1756510	0.6121	37.3177	ug/ml
C17:0	C19:0	7.339	230258	1756510	0.1311	2.7349	ug/ml
C17:1	C19:0	7.552	87327	1756510	0.0497	2.6488	ug/ml
C18:0	C19:0	8.179	3315577	1756510	1.8876	41.0973	ug/ml
C18:1n9t	C19:0	8.317	9457	1756510	0.0054	0.3176	ug/ml
C18:1n9c	C19:0	8.433	7021385	1756510	3.9973	270.0769	ug/ml
C18:2n6t	C19:0	8.877	0	1756510	0.0000	ND	ug/ml
C18:2n6c	C19:0	8.877	1922838	1756510	1.0947	76.2156	ug/ml
C18:3n6	C19:0	9.122	17986	1756510	0.0102	ND	ug/ml
C18:3n3	C19:0	9.446	769785	1756510	0.4382	21.7826	ug/ml
C20:0	C19:0	10.056	32793	1756510	0.0187	0.5049	ug/ml
C20:1	C19:0	10.291	124683	1756510	0.0710	4.4142	ug/ml
C20:2	C19:0	10.749	31616	1756510	0.0180	1.1547	ug/ml
C21:0	C19:0	10.958	2076	1756510	0.0012	0.0327	ug/ml
C20:3n6	C19:0	10.998	1995	1756510	0.0011	0.1159	ug/ml
C20:4n6	C19:0	11.154	2934	1756510	0.0017	0.1394	ug/ml
C20:3n3	C19:0	11.296	20985	1756510	0.0119	0.6756	ug/ml
C20:5n3	C19:0	11.696	549	1756510	0.0003	0.0525	ug/ml

化合物	ISTD	RT	响应	ISTD 响应	响应比	最终浓度	单位
C22:0	C19:0	11.816	3858	1756510	0.0022	0.0914	ug/ml
C22:1n9	C19:0	12.043	3908	1756510	0.0022	0.1499	ug/ml
C22:2n6	C19:0	12.470	549	1756510	0.0003	0.0617	ug/ml
C23:0	C19:0	12.777	0	1756510	0.0000	ND	ug/ml
C24:0	C19:0	13.395	2118	1756510	0.0012	0.0607	ug/ml
C22:6	C19:0	13.511	529	1756510	0.0003	0.0275	ug/ml
C24:1	C19:0	13.635	0	1756510	0.0000	ND	ug/ml

C4:0

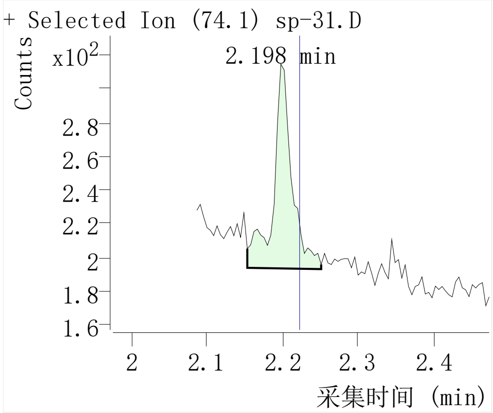

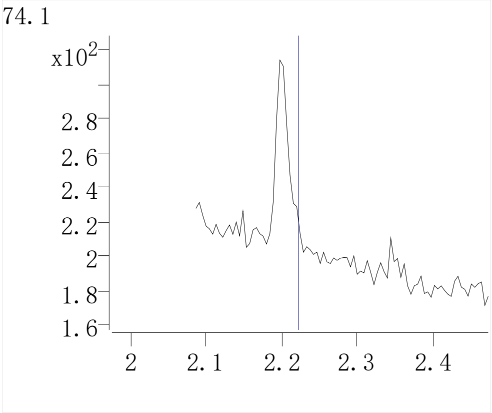

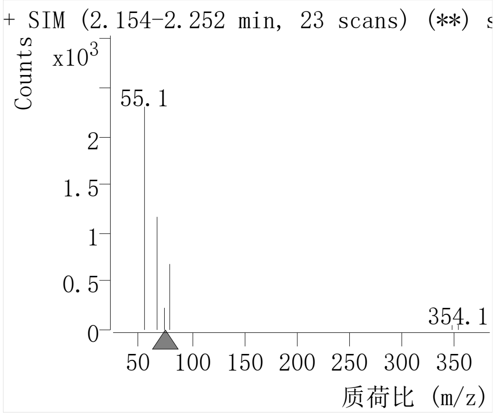

C6:0

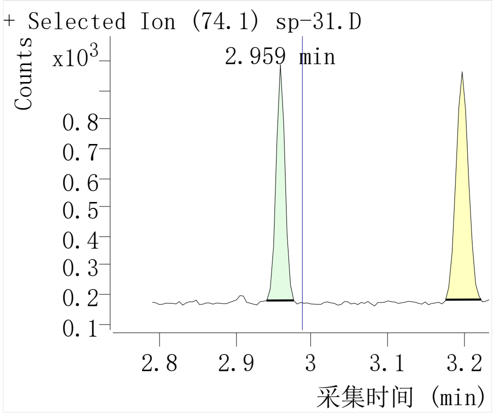

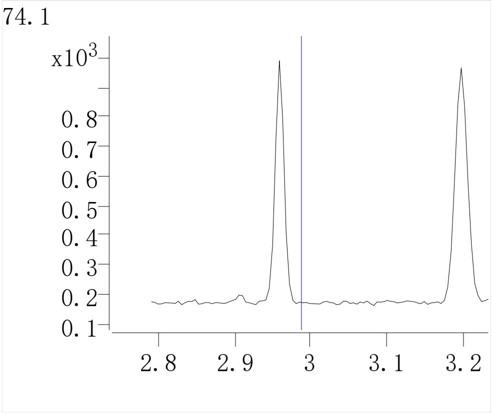

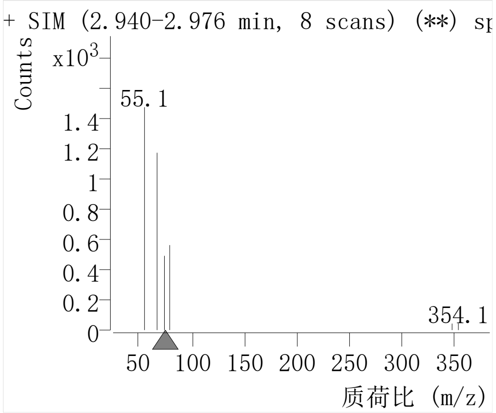

C8:0

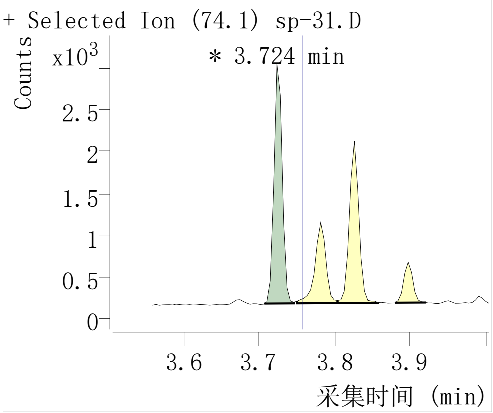

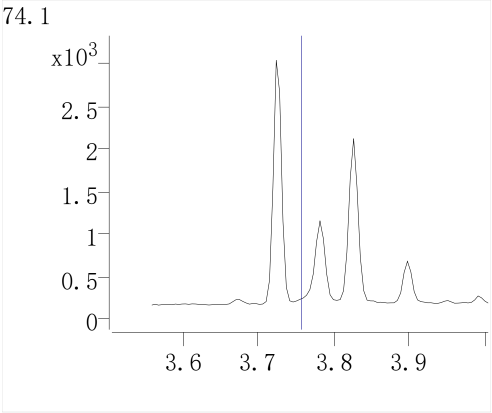

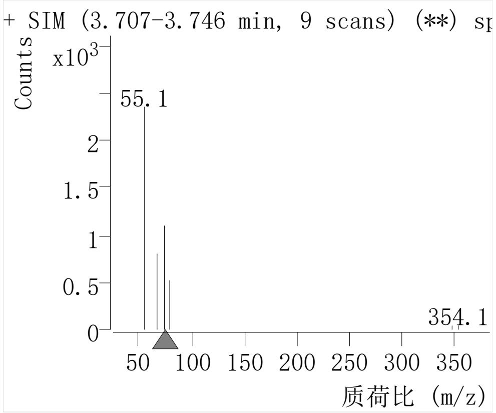

C10:0

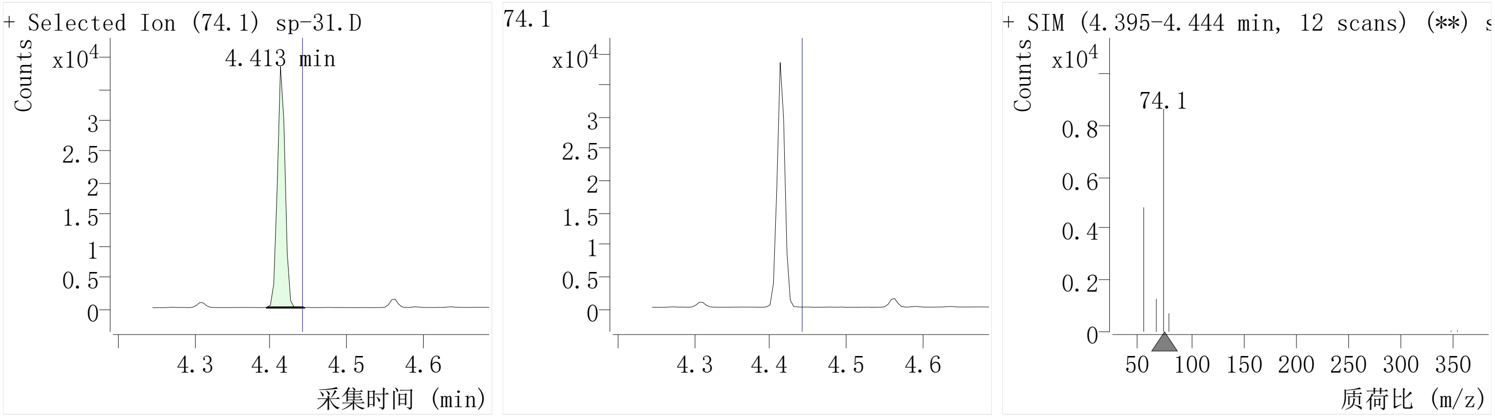

C11:0

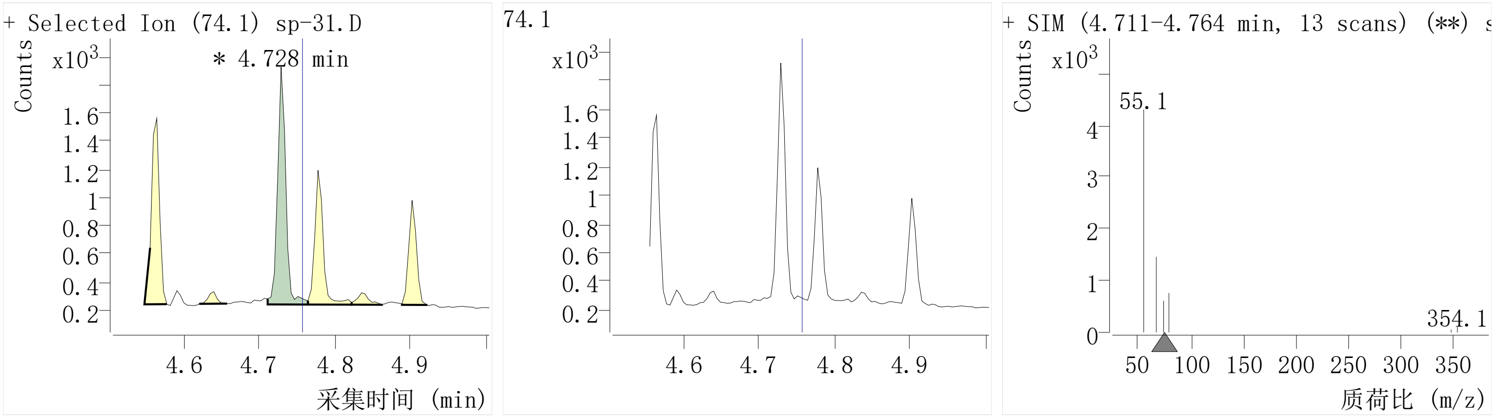

C12:0

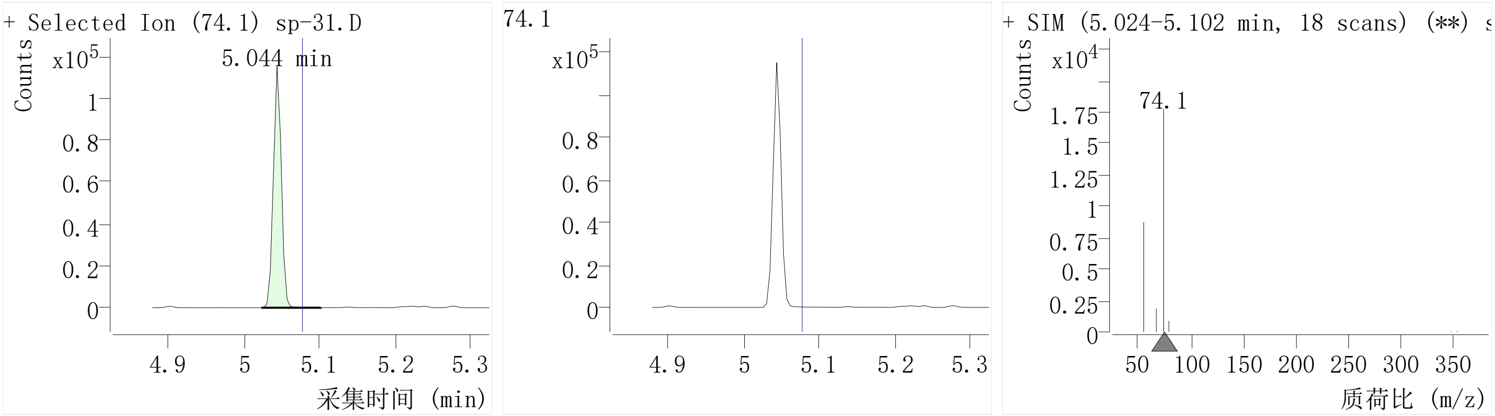

C13:0

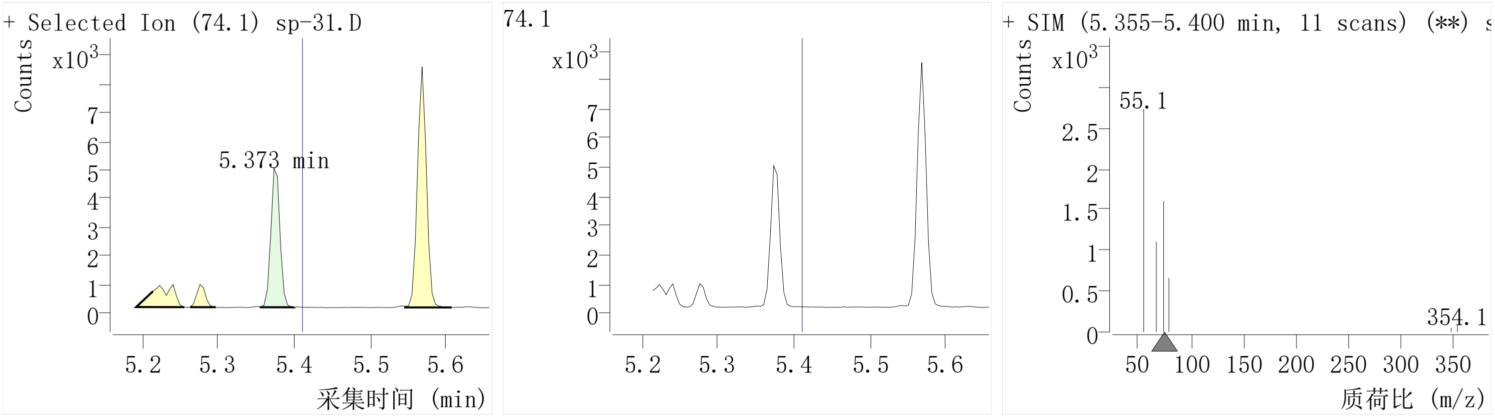

C14:0

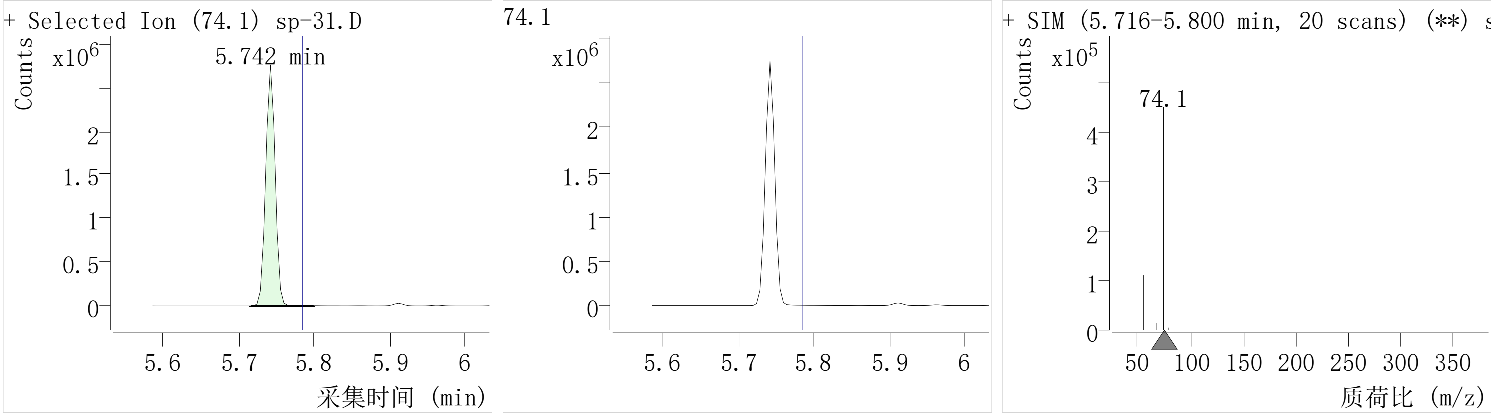

C14:1

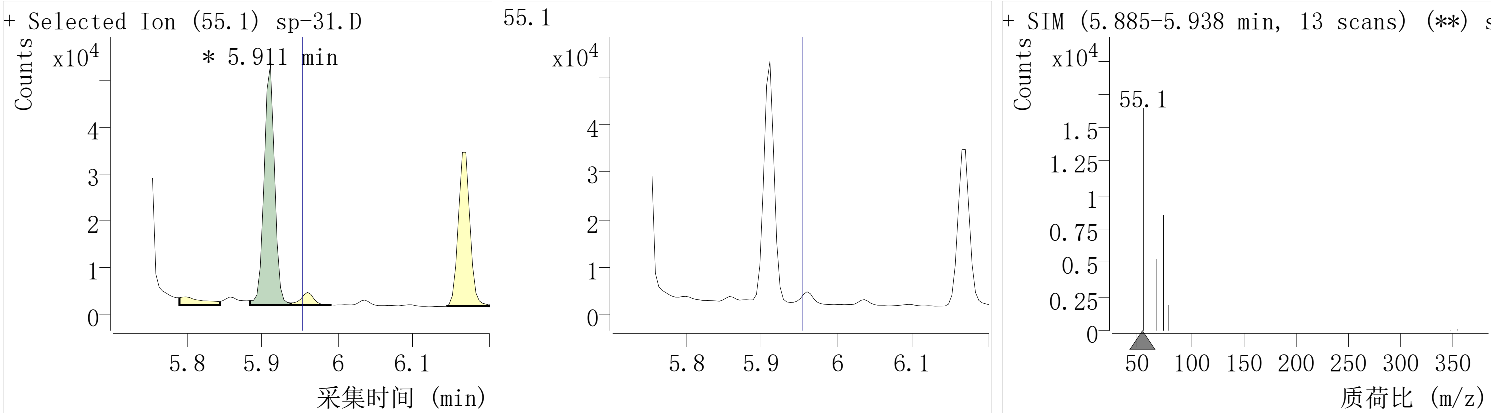

C15:0

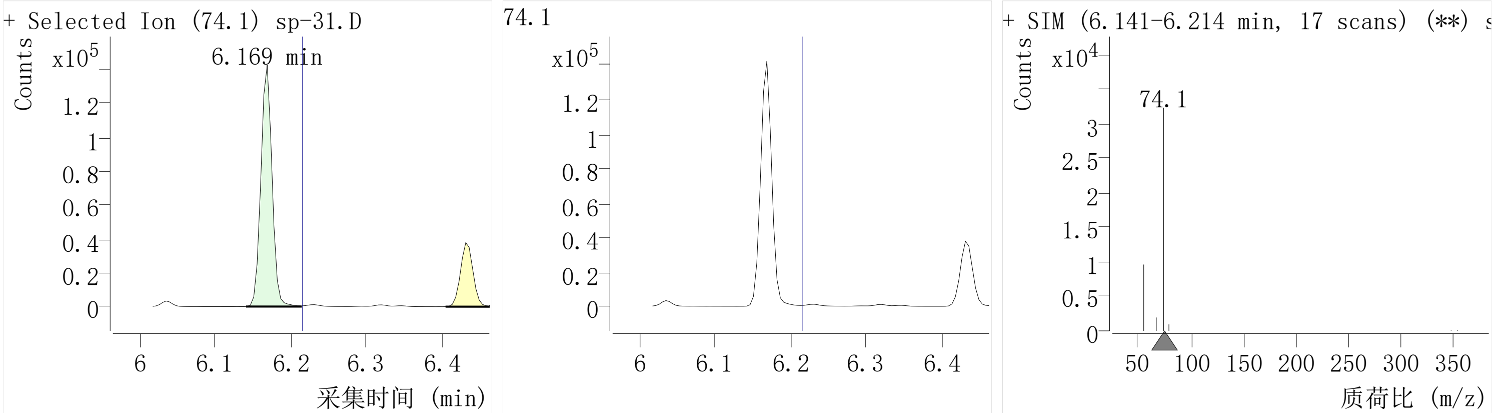

C15:1

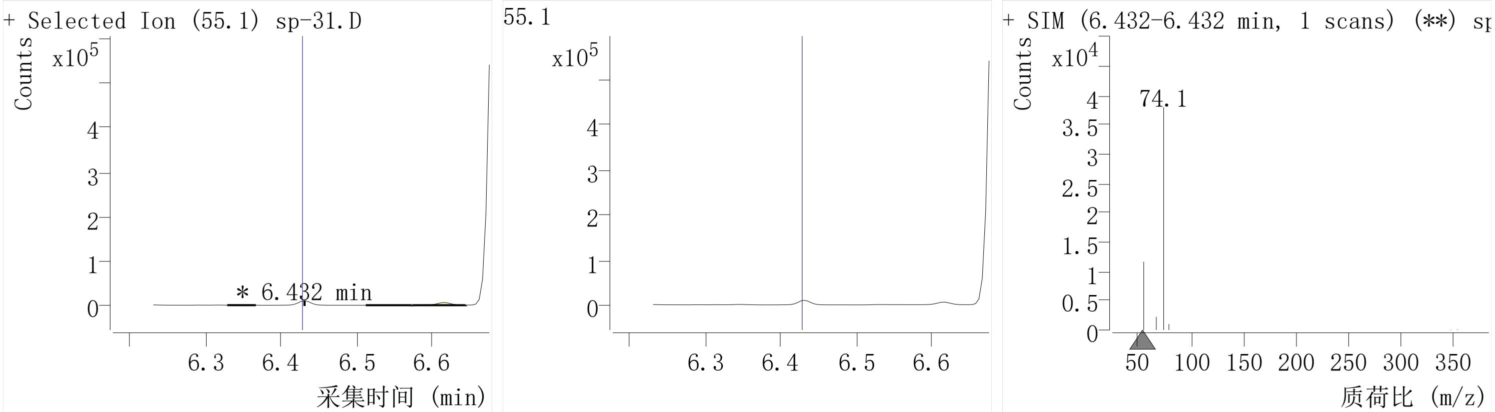

C16:0

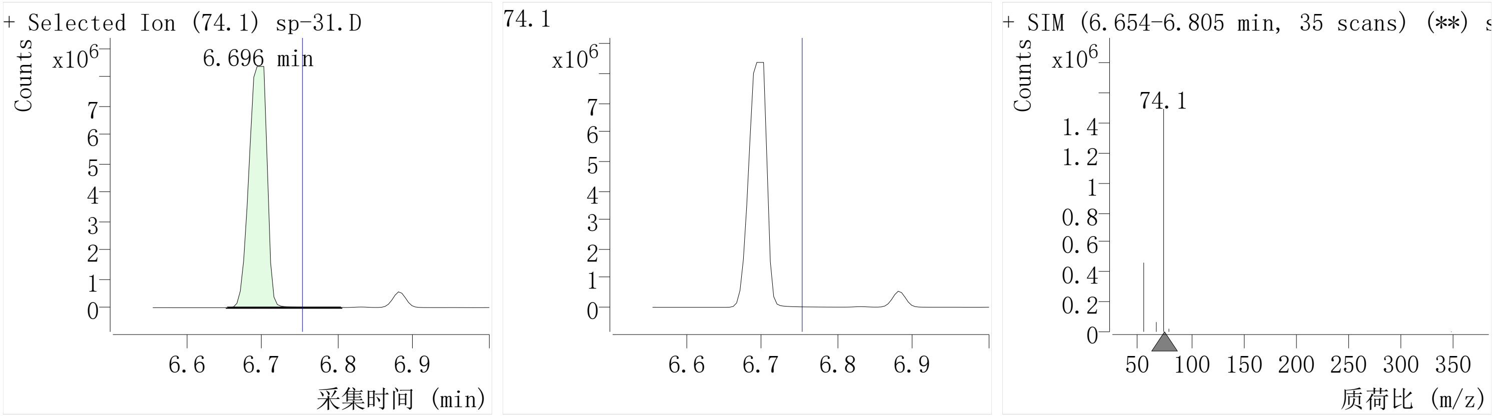

C16:1

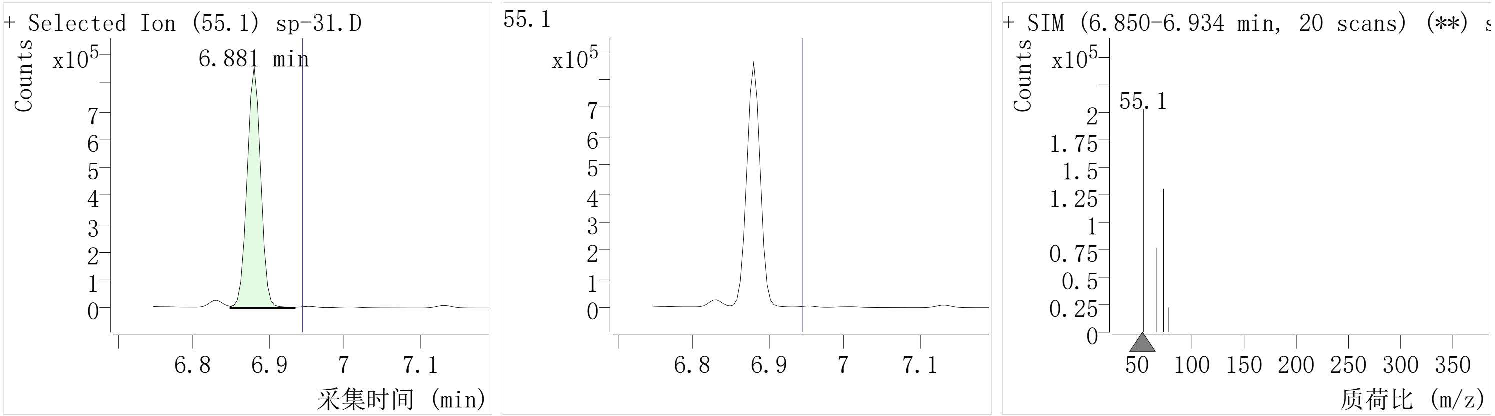

C17:0

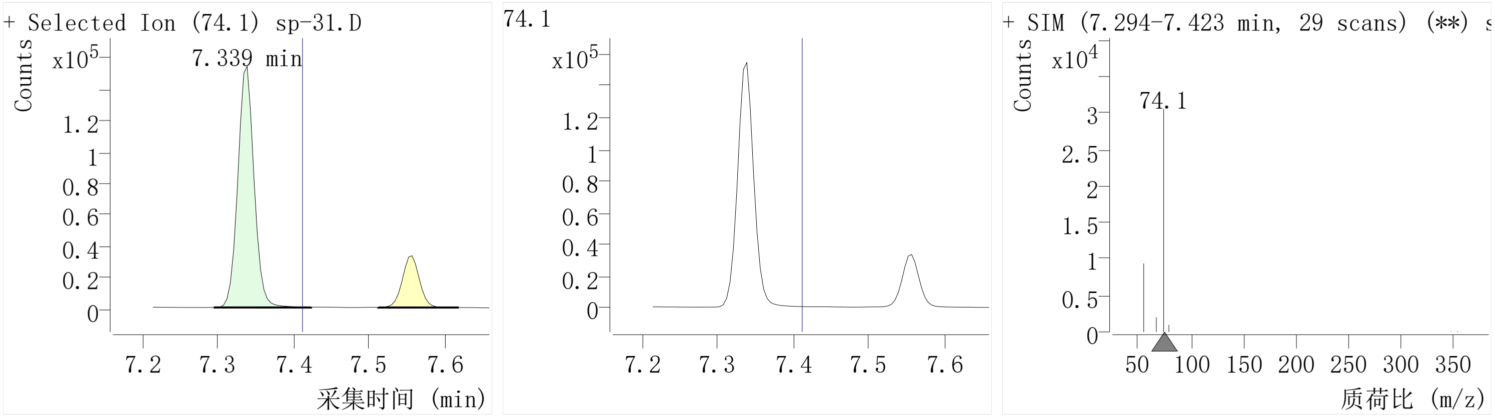

C17:1

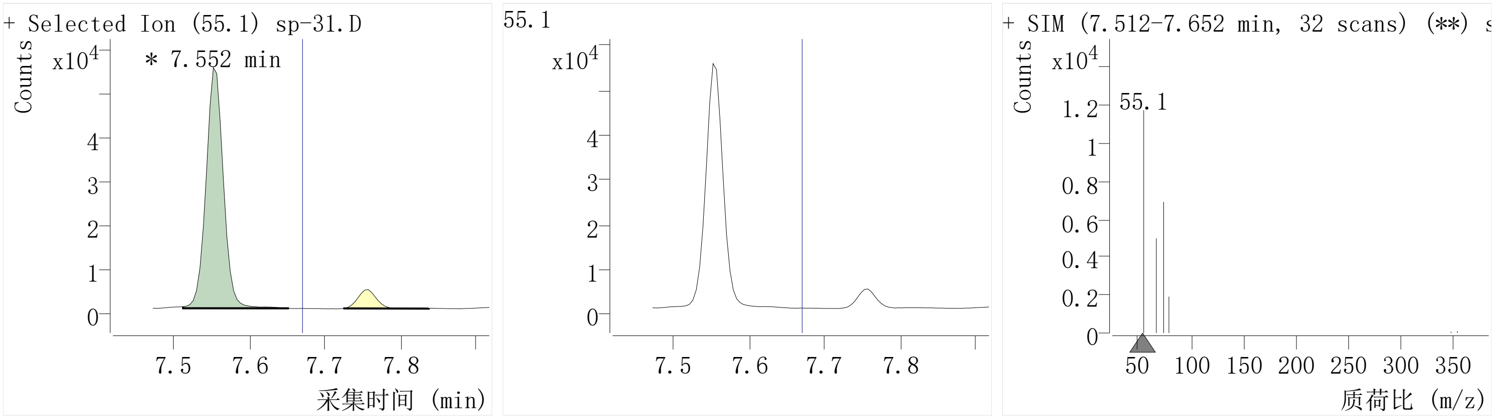

C18:0

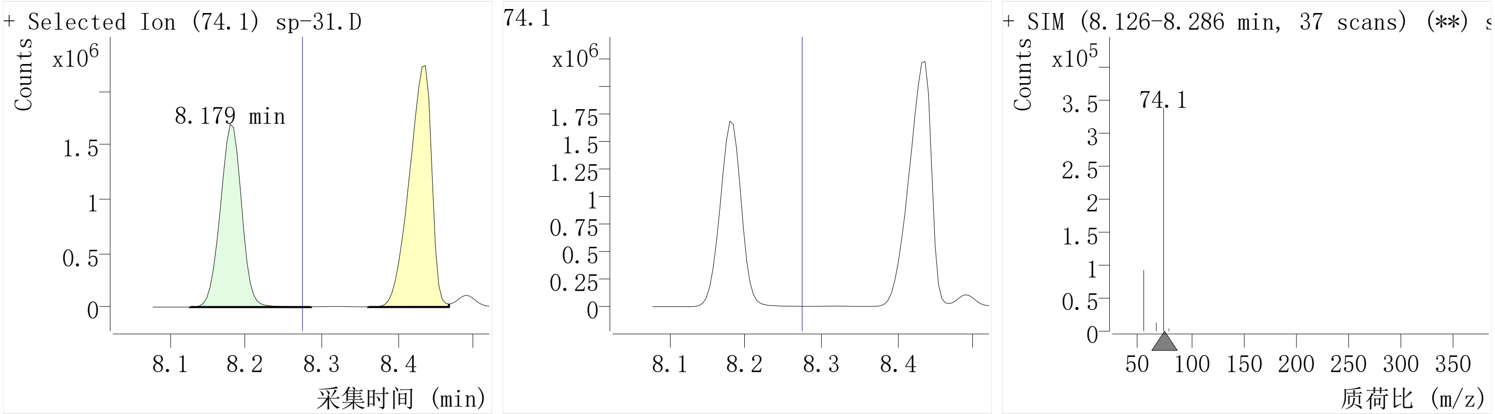

C18:1n9t

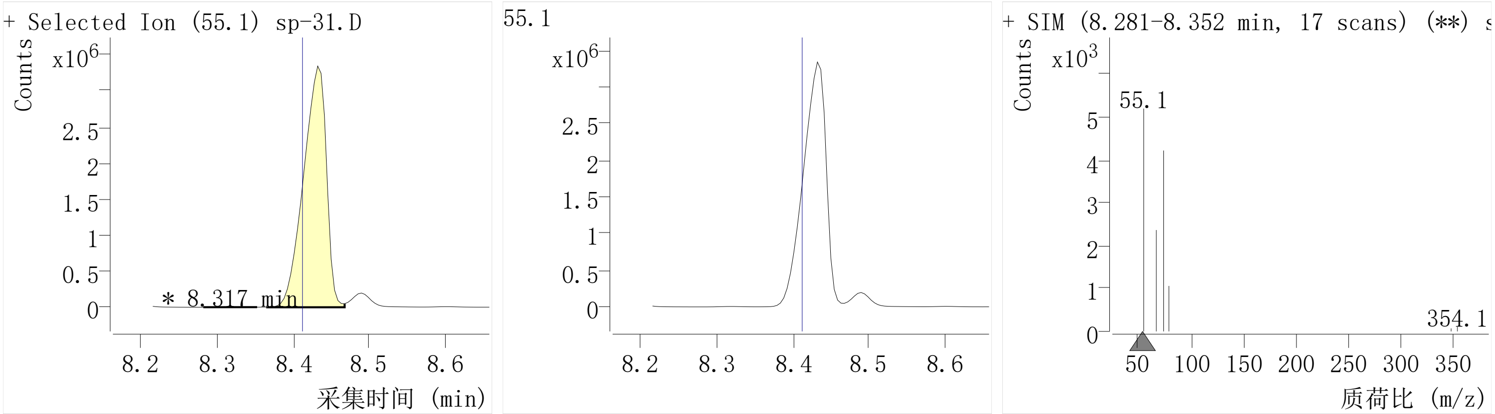

C18:1n9c

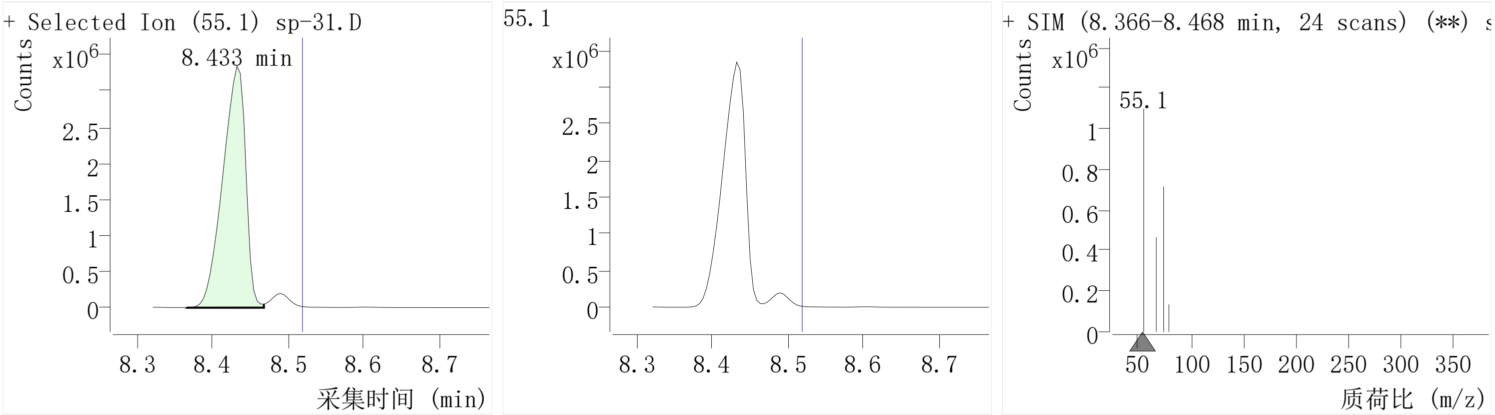

C18:2n6t

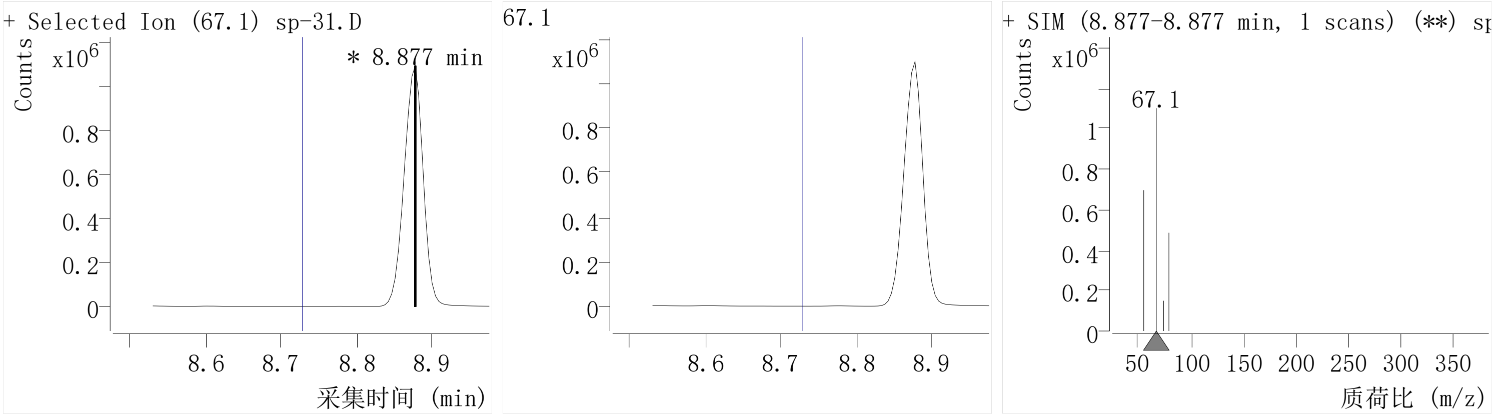

C18:2n6c

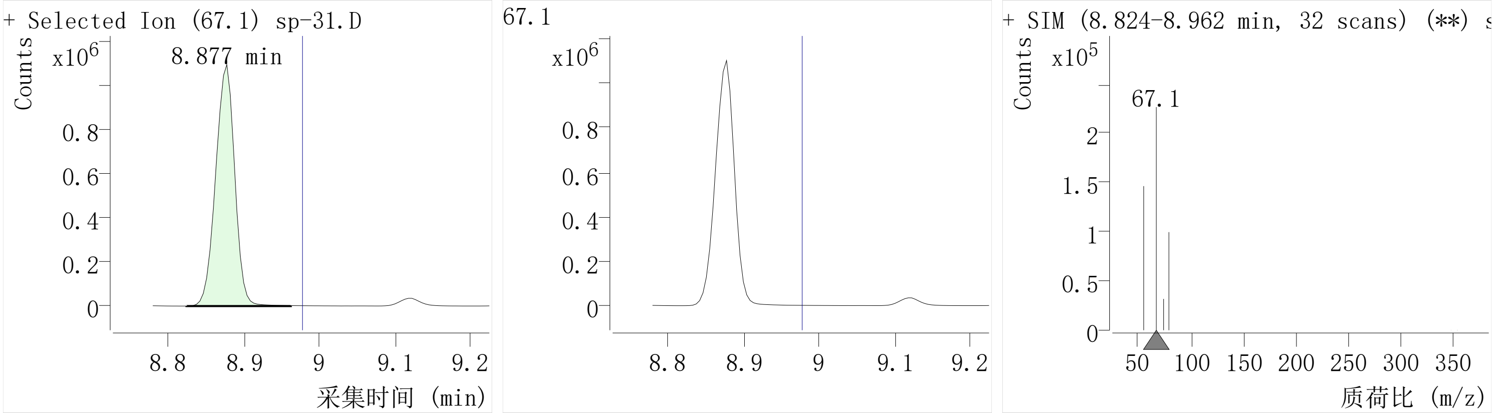

C18:3n6

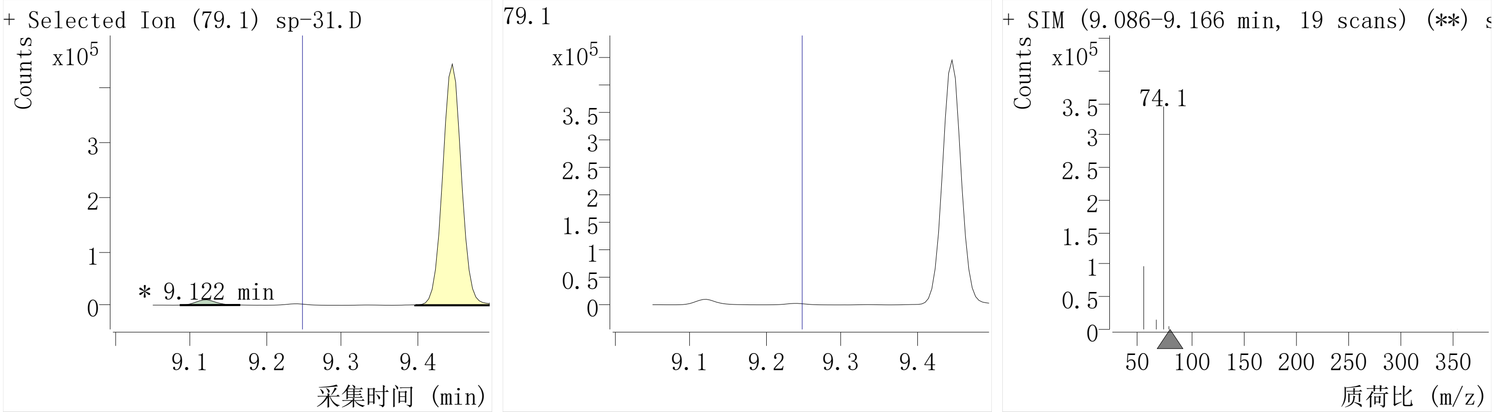

C18:3n3

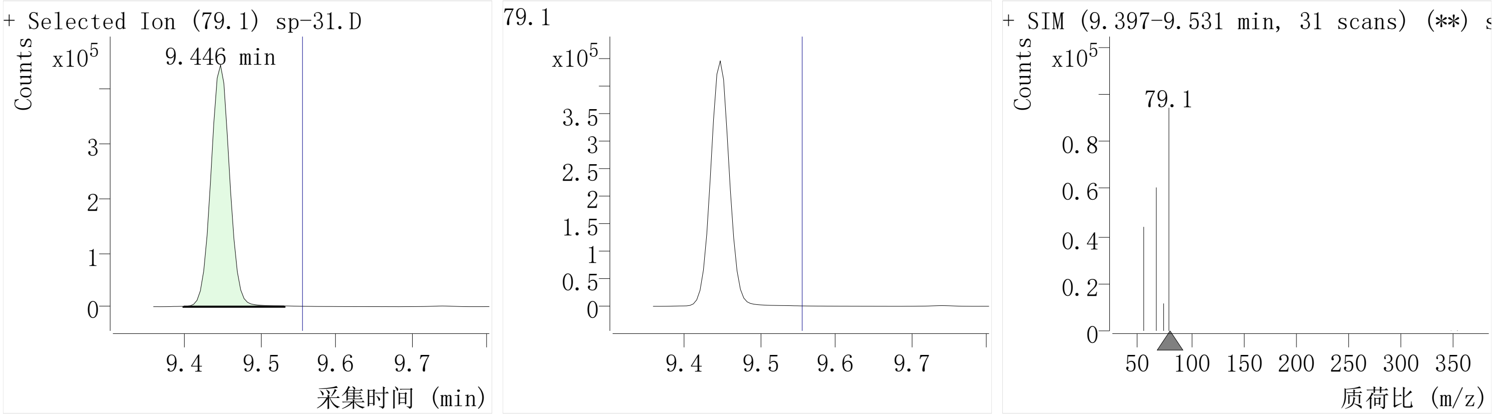

C20:0

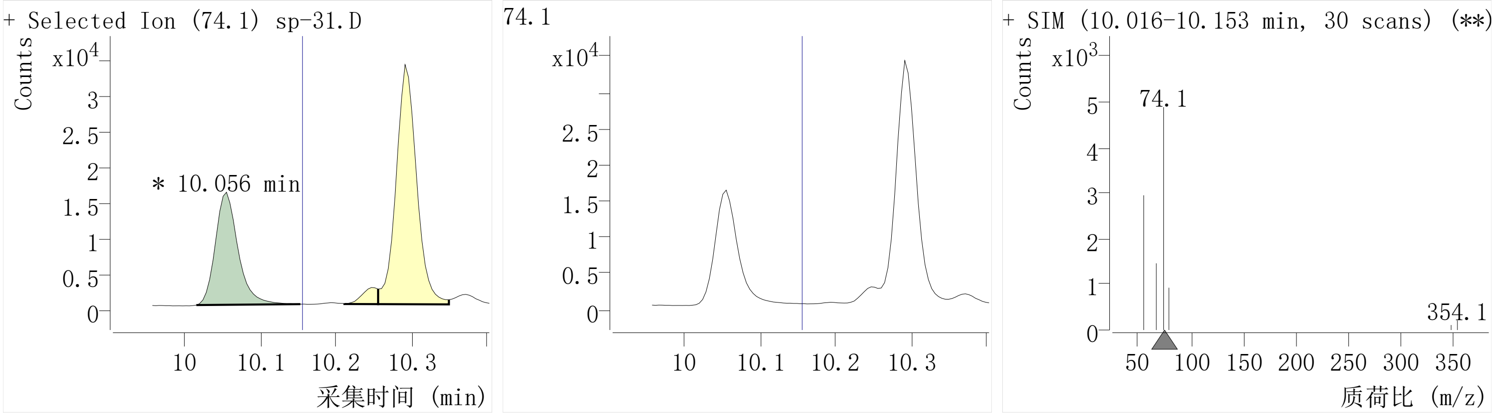

C20:1

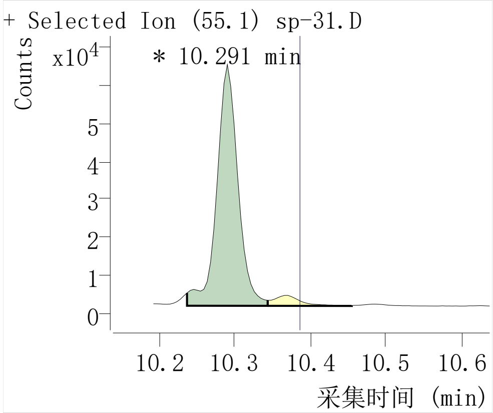

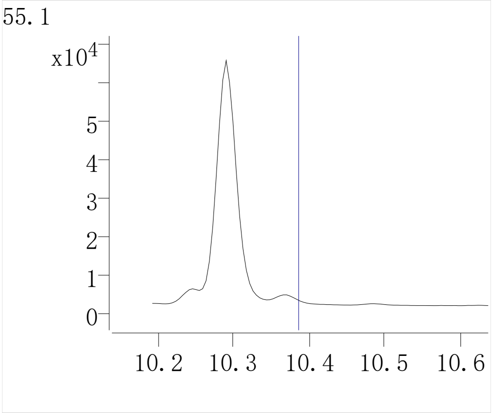

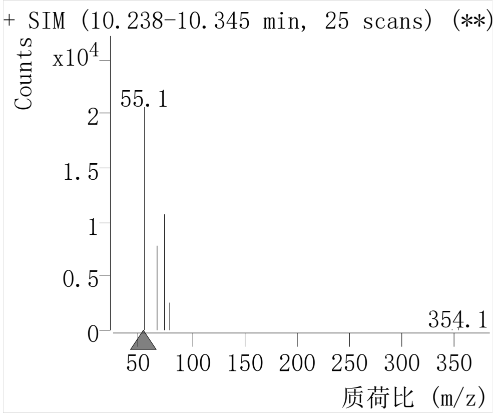

C20:2

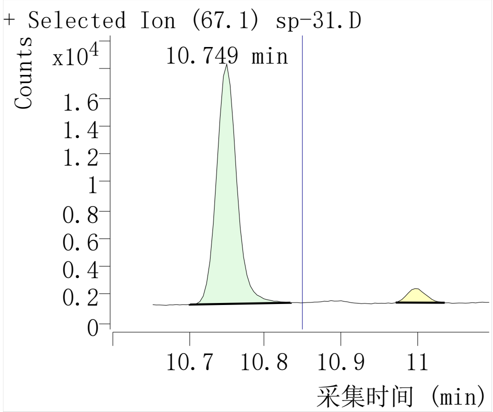

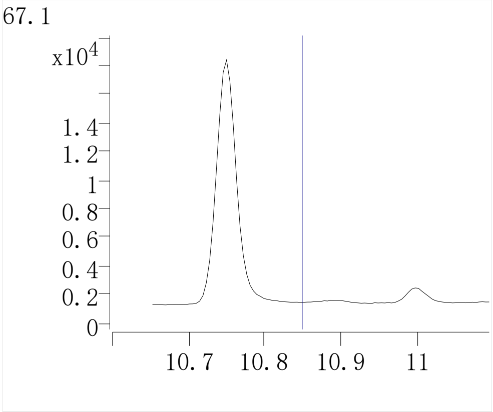

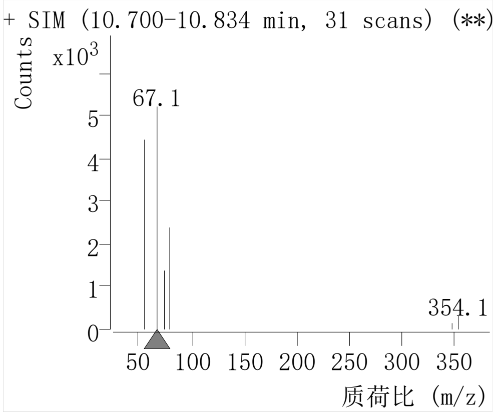

C21:0

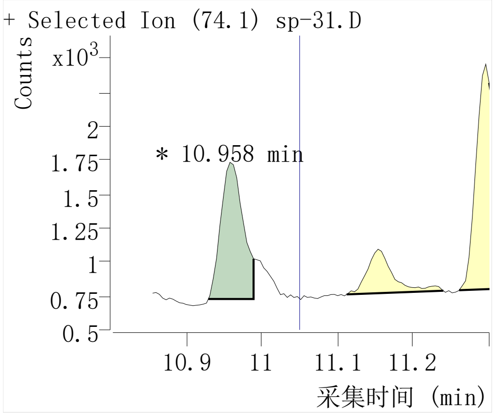

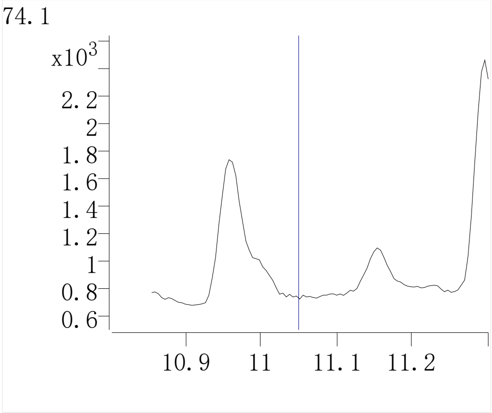

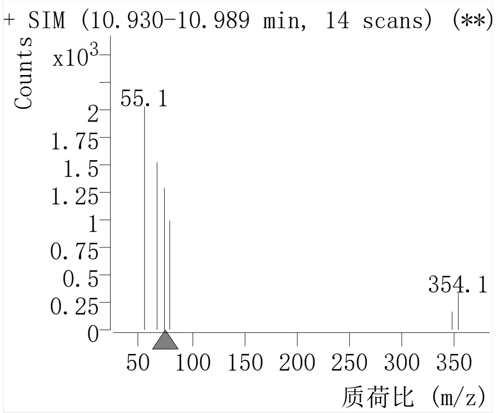

C20:3n6

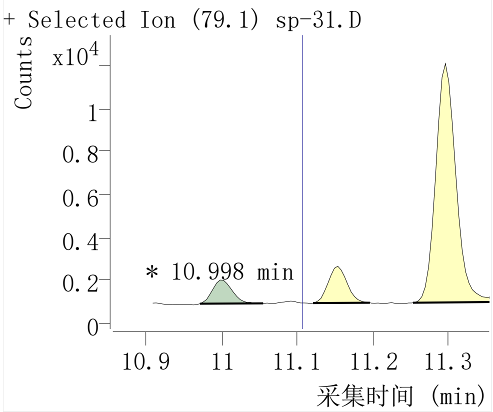

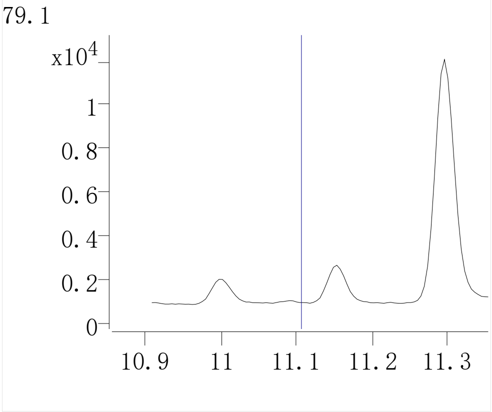

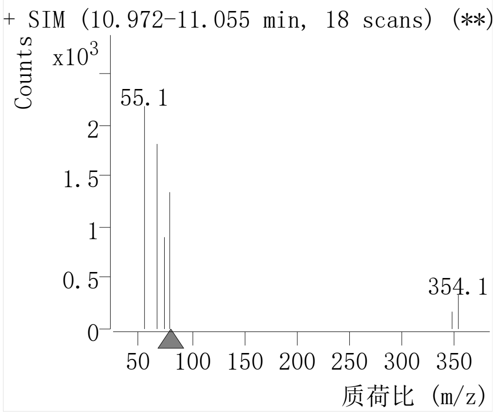

C20:4n6

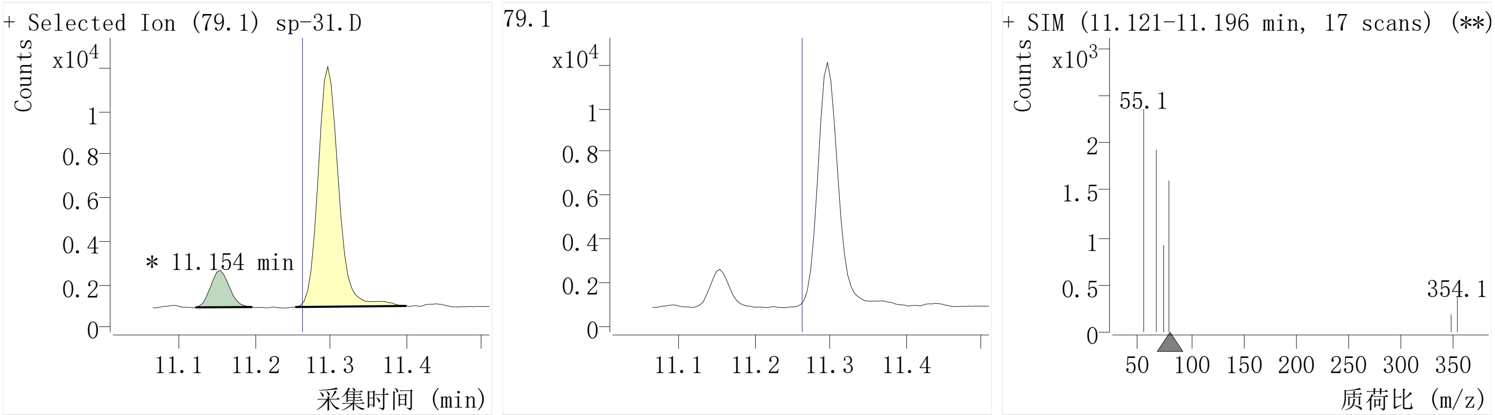

C20:3n3

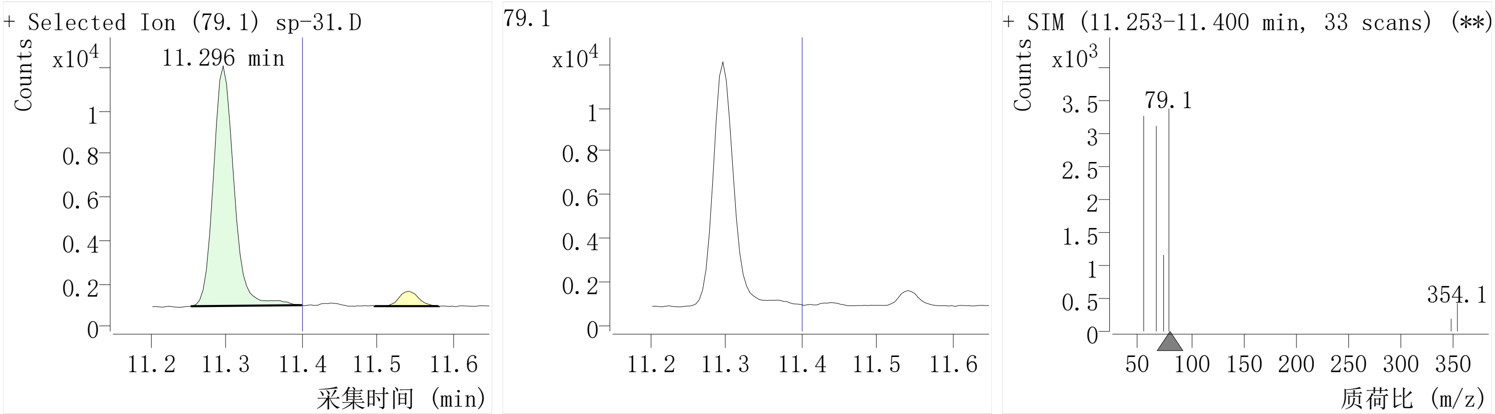

C20:5n3

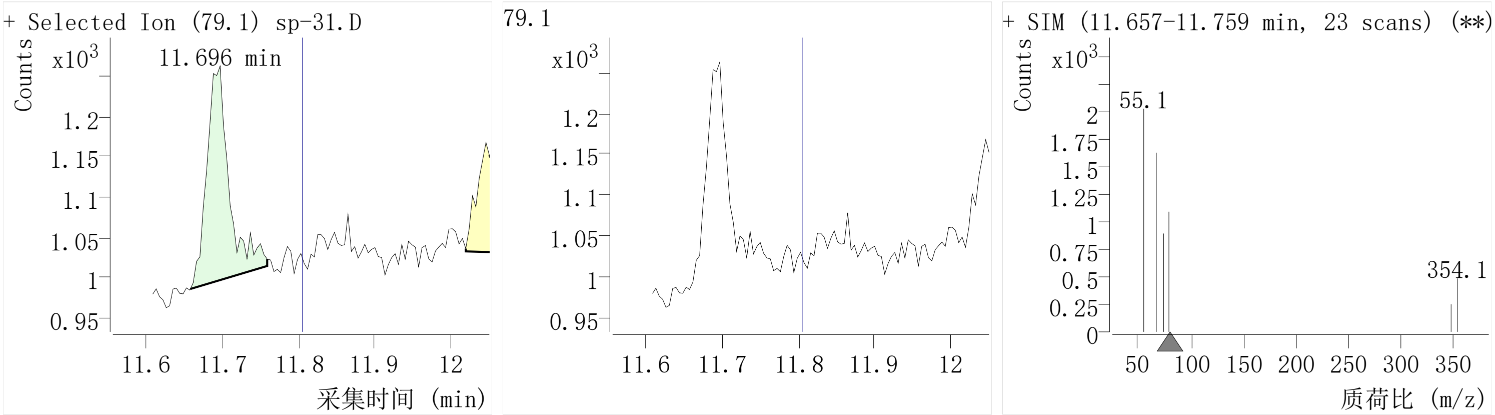

C22:0

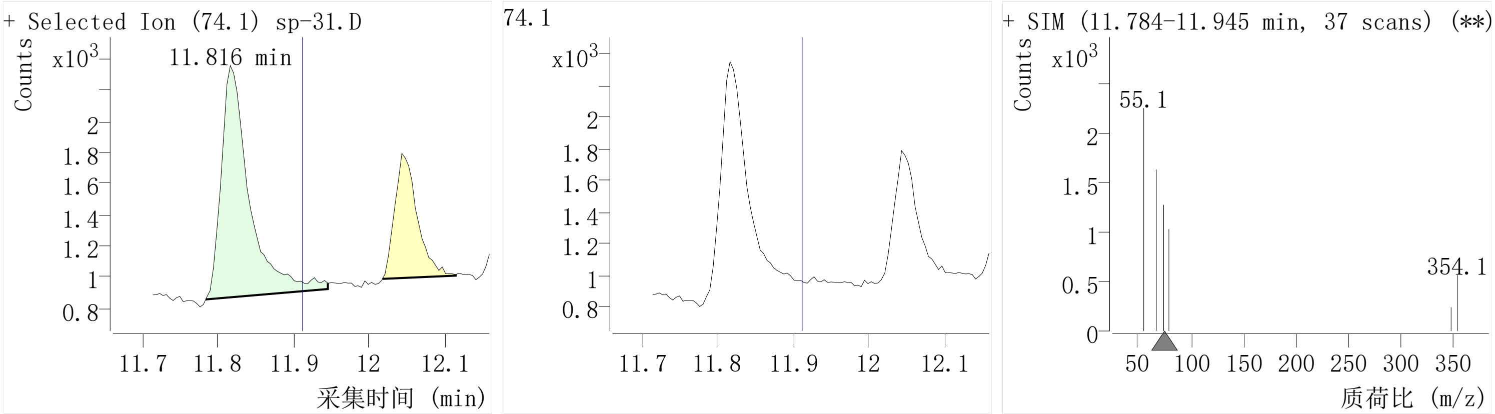

C22:1n9

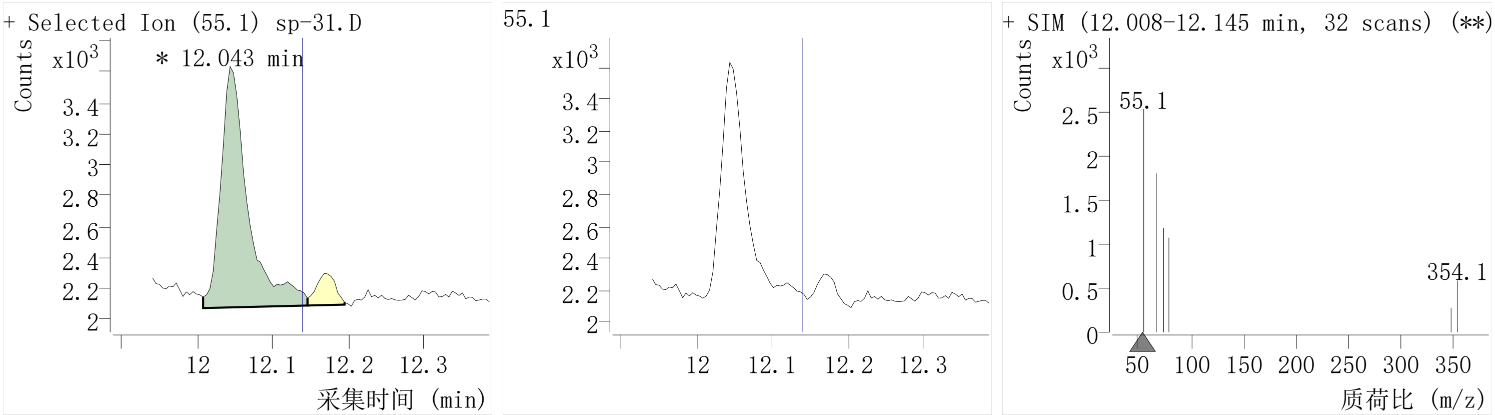

C22:2n6

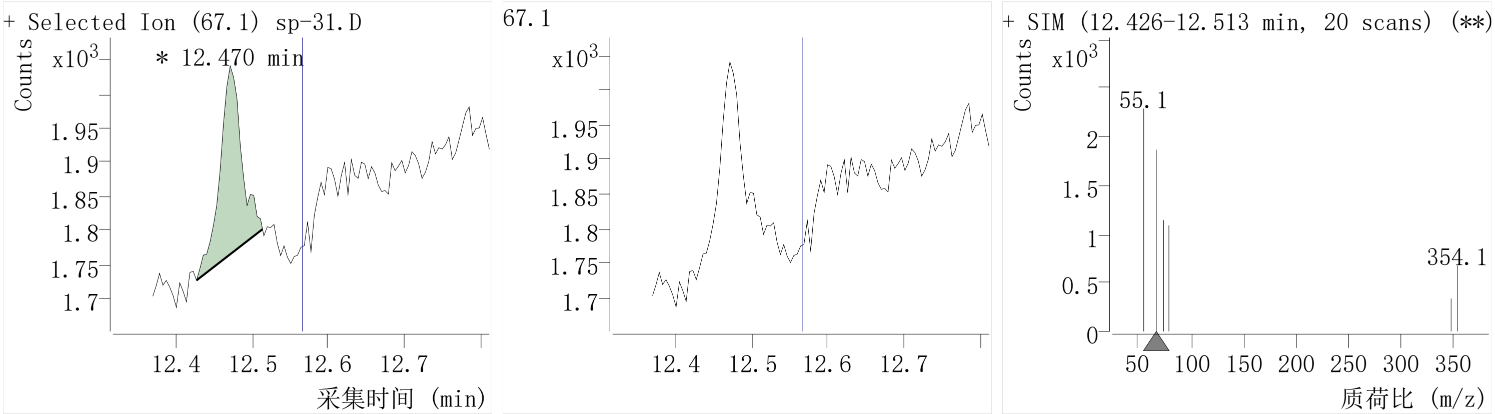

C23:0

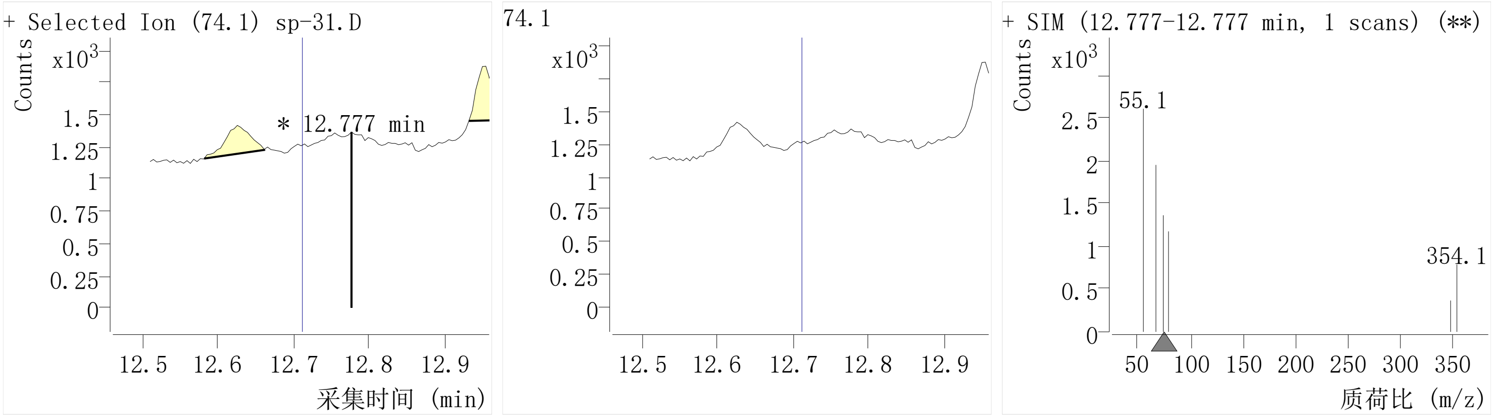

C24:0

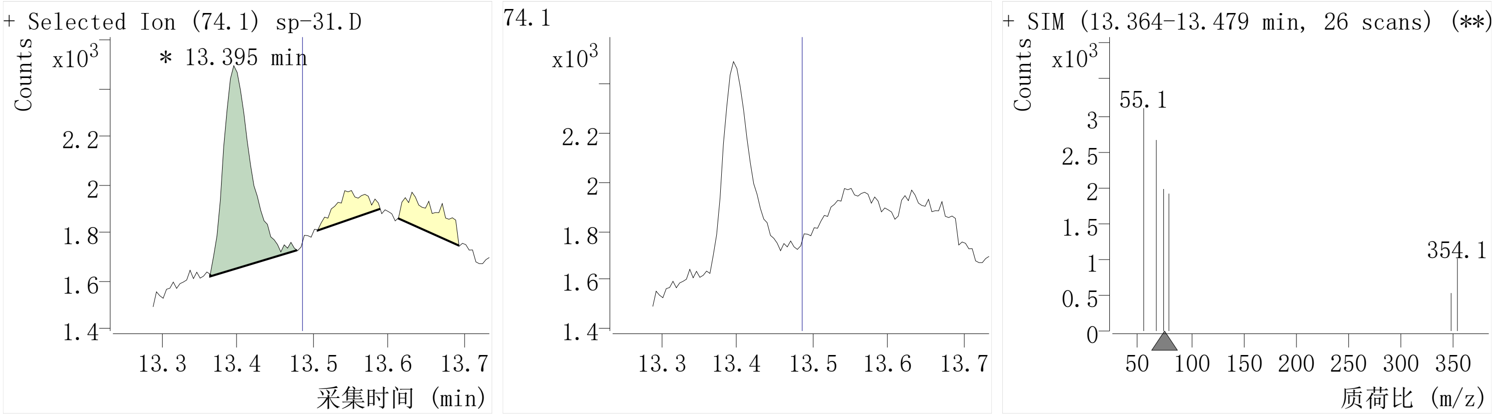

C22:6

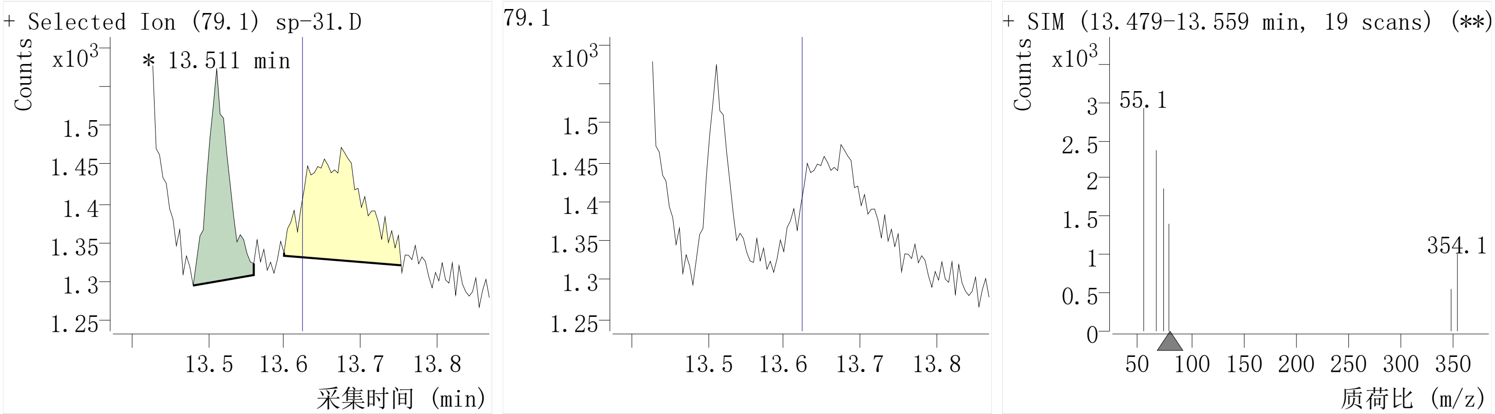
+ Selected Ion (79.1) sp-31.D
Counts x10^3
* 13.511 min
1.5
1.45
1.4
1.35
1.3
1.25
13.5 13.6 13.7 13.8
采集时间 (min)
79.1
x10^3
1.5
1.45
1.4
1.35
1.3
1.25
13.5 13.6 13.7 13.8
+ SIM (13.479-13.559 min, 19 scans) (**)
Counts x10^3
55.1
354.1
3
2.5
2
1.5
1
0.5
0
50 100 150 200 250 300 350
质荷比 (m/z)

C24:1

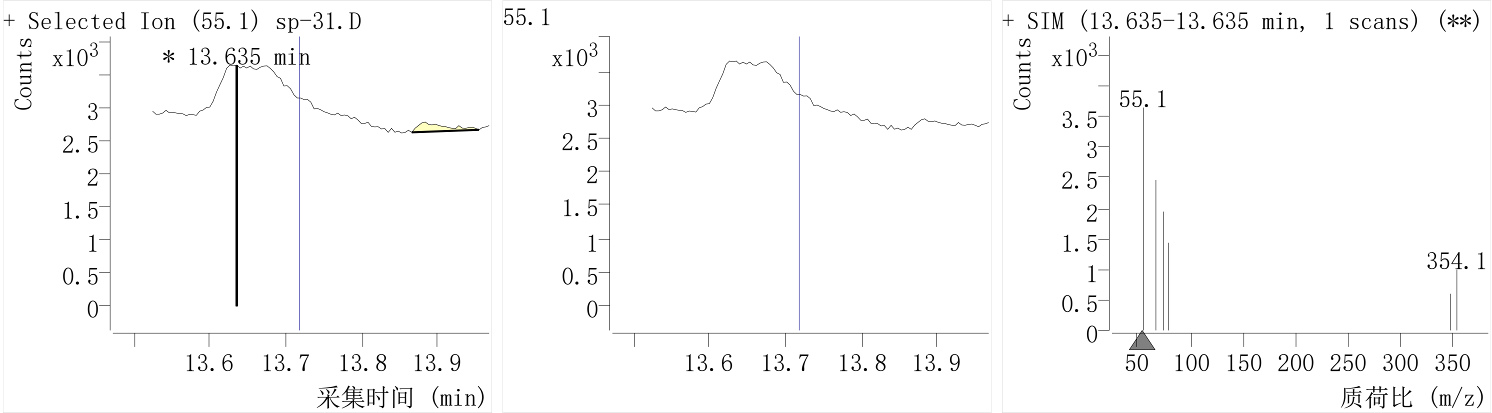
+ Selected Ion (55.1) sp-31.D
Counts x10^3
* 13.635 min
3
2.5
2
1.5
1
0.5
0
13.6 13.7 13.8 13.9
采集时间 (min)
55.1
x10^3
3
2.5
2
1.5
1
0.5
0
13.6 13.7 13.8 13.9
+ SIM (13.635-13.635 min, 1 scans) (**)
Counts x10^3
55.1
354.1
3.5
3
2.5
2
1.5
1
0.5
0
50 100 150 200 250 300 350
质荷比 (m/z)

**批处理路径**	G:\GC-MS\HX250430-4-GCMS总脂肪酸靶向检测\HX250430-4\QuantResults\HX250430-4.batch.bin		
**分析时间**	2025/5/14 16:58	**分析员姓名**	DESKTOP-M3A0GPO\omics
**报告时间**	2025/5/16 14:53:38	**报告员姓名**	DESKTOP-M3A0GPO\omics
**最近校正更新**	2025/5/14 16:58	**批处理状态**	已处理
**定量批处理版本**	10.2	**定量报告版本**	10.2

**采集时间**	2025/5/9 9:18	**数据文件**	sp-32.D
**样品类型**	样品	**样品名称**	sp-32
**稀释**	1	**采集方法**	脂肪酸

样品色谱图

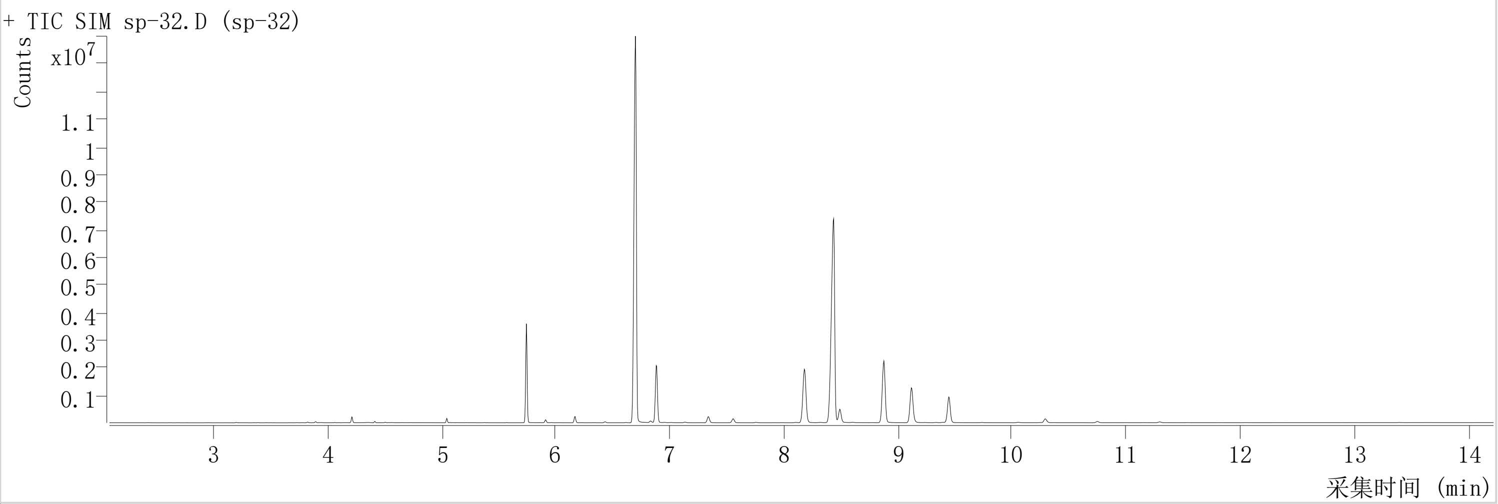

化合物	ISTD	RT	响应	ISTD 响应	响应比	最终浓度	单位
C4:0	C19:0	2.198	270	1813710	0.0001	ND	ug/ml
C6:0	C19:0	2.959	948	1813710	0.0005	0.0062	ug/ml
C8:0	C19:0	3.724	2945	1813710	0.0016	0.0121	ug/ml
C10:0	C19:0	4.413	28357	1813710	0.0156	0.2396	ug/ml
C11:0	C19:0	4.729	1326	1813710	0.0007	0.0060	ug/ml
C12:0	C19:0	5.044	92862	1813710	0.0512	0.7866	ug/ml
C13:0	C19:0	5.378	4826	1813710	0.0027	0.0278	ug/ml
C14:0	C19:0	5.742	2442631	1813710	1.3468	29.7226	ug/ml
C14:1	C19:0	5.911	51265	1813710	0.0283	1.3189	ug/ml
C15:0	C19:0	6.169	183207	1813710	0.1010	1.7680	ug/ml
C15:1	C19:0	6.432	0	1813710	0.0000	ND	ug/ml
C16:0	C19:0	6.697	14115491	1813710	7.7827	306.0251	ug/ml
C16:1	C19:0	6.881	1269982	1813710	0.7002	42.7024	ug/ml
C17:0	C19:0	7.339	254978	1813710	0.1406	2.9330	ug/ml
C17:1	C19:0	7.552	108985	1813710	0.0601	3.2075	ug/ml
C18:0	C19:0	8.179	2872982	1813710	1.5840	33.8219	ug/ml
C18:1n9t	C19:0	8.321	9180	1813710	0.0051	0.2986	ug/ml
C18:1n9c	C19:0	8.433	6843617	1813710	3.7733	253.9834	ug/ml
C18:2n6t	C19:0	8.877	0	1813710	0.0000	ND	ug/ml
C18:2n6c	C19:0	8.877	1825150	1813710	1.0063	68.1068	ug/ml
C18:3n6	C19:0	9.122	18964	1813710	0.0105	ND	ug/ml
C18:3n3	C19:0	9.446	749682	1813710	0.4133	20.5444	ug/ml
C20:0	C19:0	10.056	26946	1813710	0.0149	0.4087	ug/ml
C20:1	C19:0	10.291	136680	1813710	0.0754	4.6839	ug/ml
C20:2	C19:0	10.749	36377	1813710	0.0201	1.2805	ug/ml
C21:0	C19:0	10.958	2060	1813710	0.0011	0.0314	ug/ml
C20:3n6	C19:0	10.998	2806	1813710	0.0015	0.1419	ug/ml
C20:4n6	C19:0	11.154	2977	1813710	0.0016	0.1376	ug/ml
C20:3n3	C19:0	11.296	24690	1813710	0.0136	0.7627	ug/ml
C20:5n3	C19:0	11.692	553	1813710	0.0003	0.0521	ug/ml

定量分析完成报告

化合物	ISTD	RT	响应	ISTD 响应	响应比	最终浓度	单位
C22:0	C19:0	11.816	2472	1813710	0.0014	0.0644	ug/ml
C22:1n9	C19:0	12.048	3618	1813710	0.0020	0.1329	ug/ml
C22:2n6	C19:0	12.470	594	1813710	0.0003	0.0628	ug/ml
C23:0	C19:0	12.781	0	1813710	0.0000	ND	ug/ml
C24:0	C19:0	13.395	1575	1813710	0.0009	0.0455	ug/ml
C22:6	C19:0	13.511	465	1813710	0.0003	0.0242	ug/ml
C24:1	C19:0	13.675	0	1813710	0.0000	ND	ug/ml

C4:0

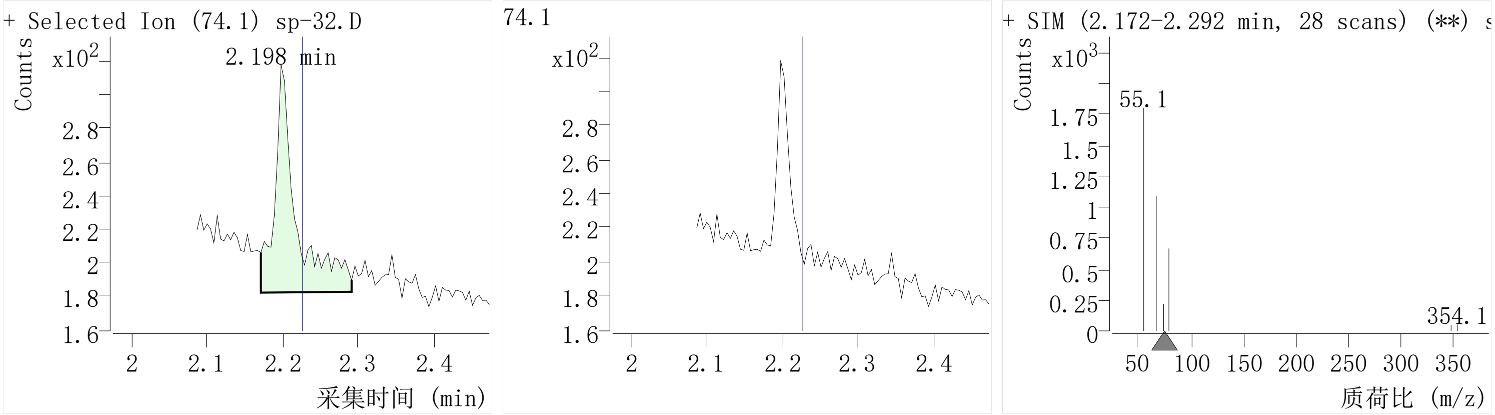

C6:0

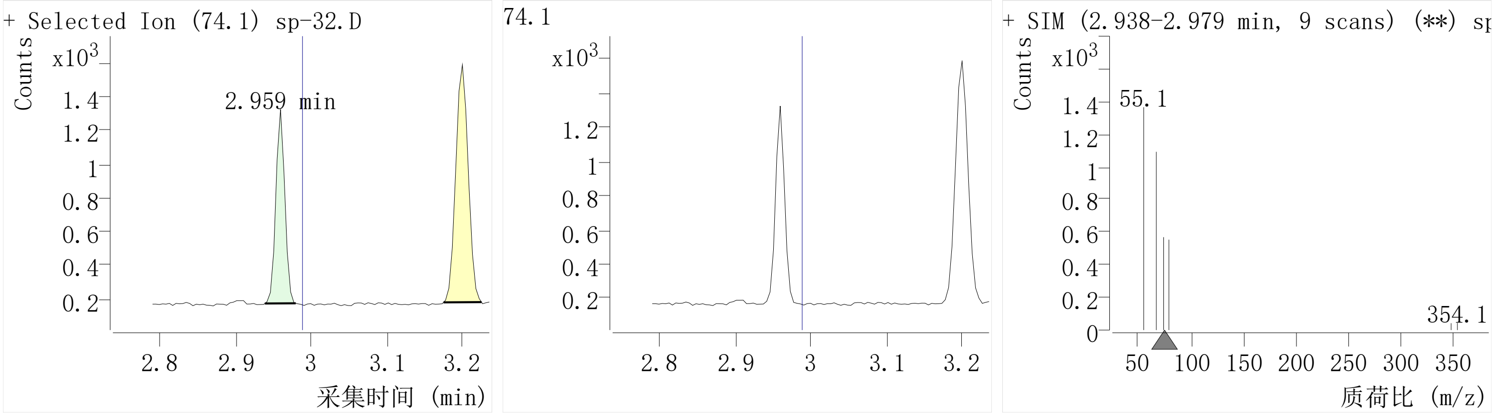

C8:0

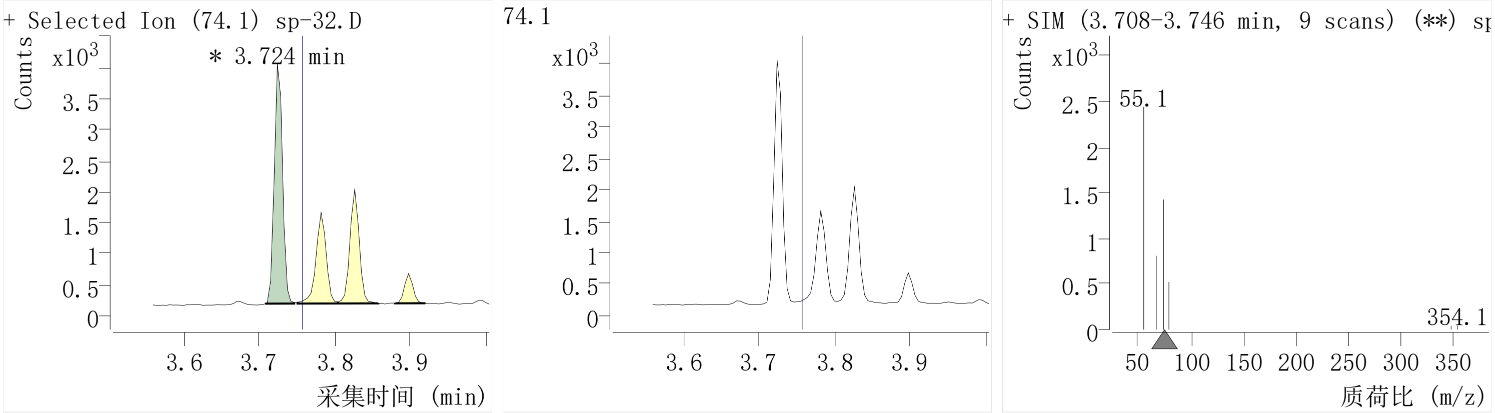

 生成时间 2025/5/16 14:52

C10:0

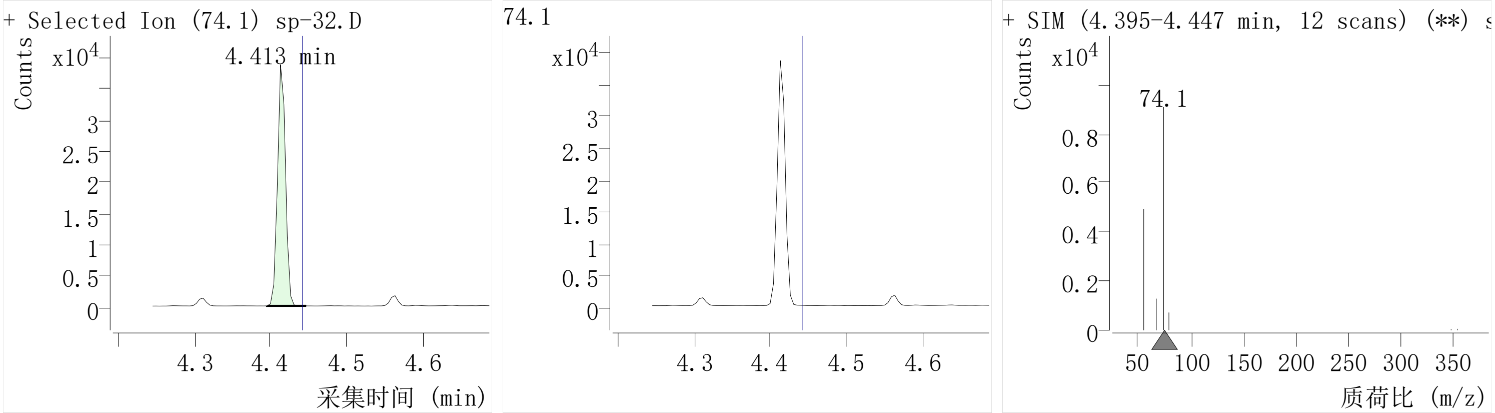

C11:0

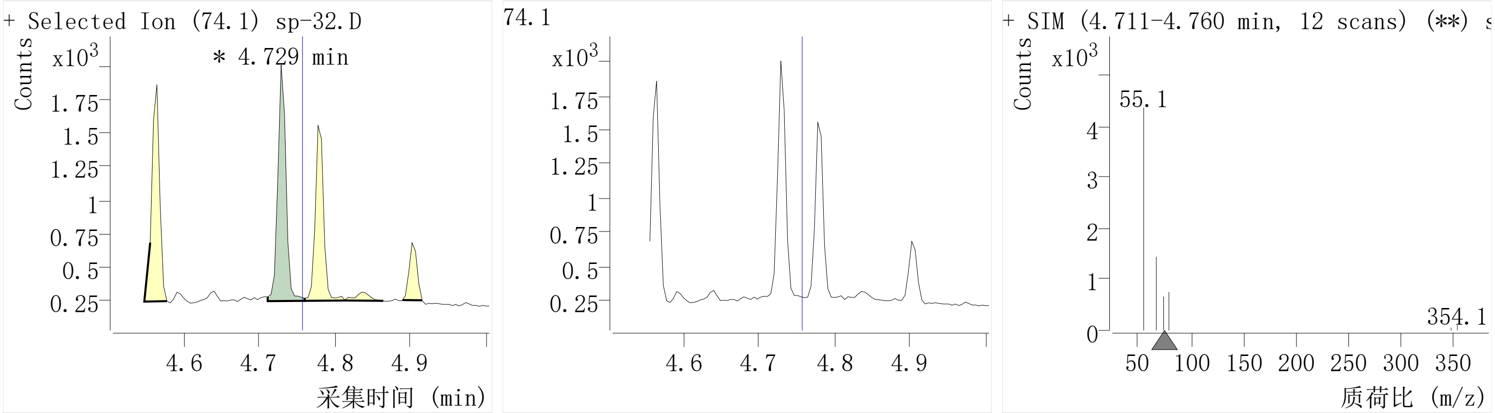

C12:0

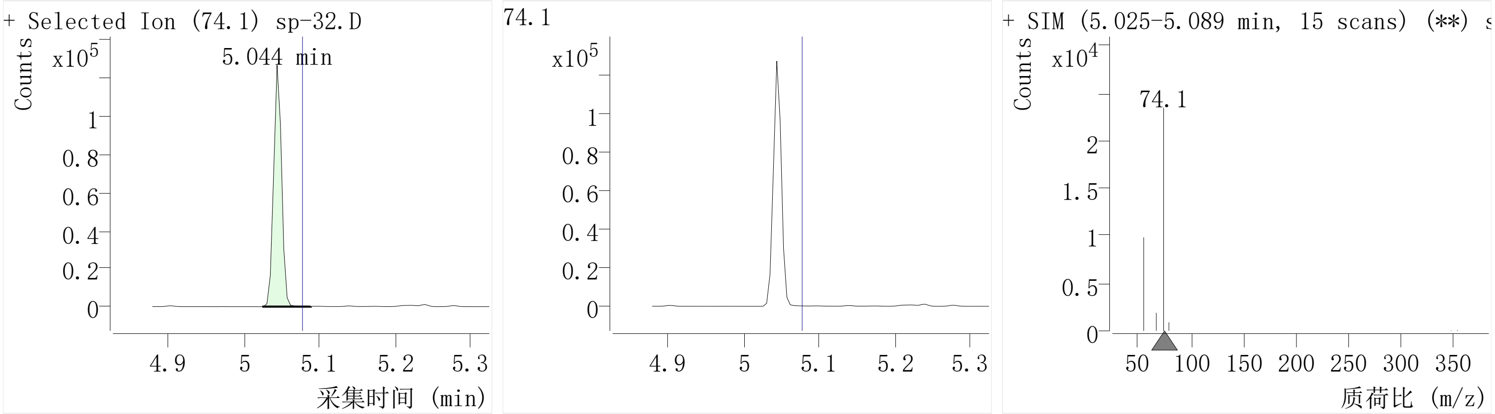

C13:0

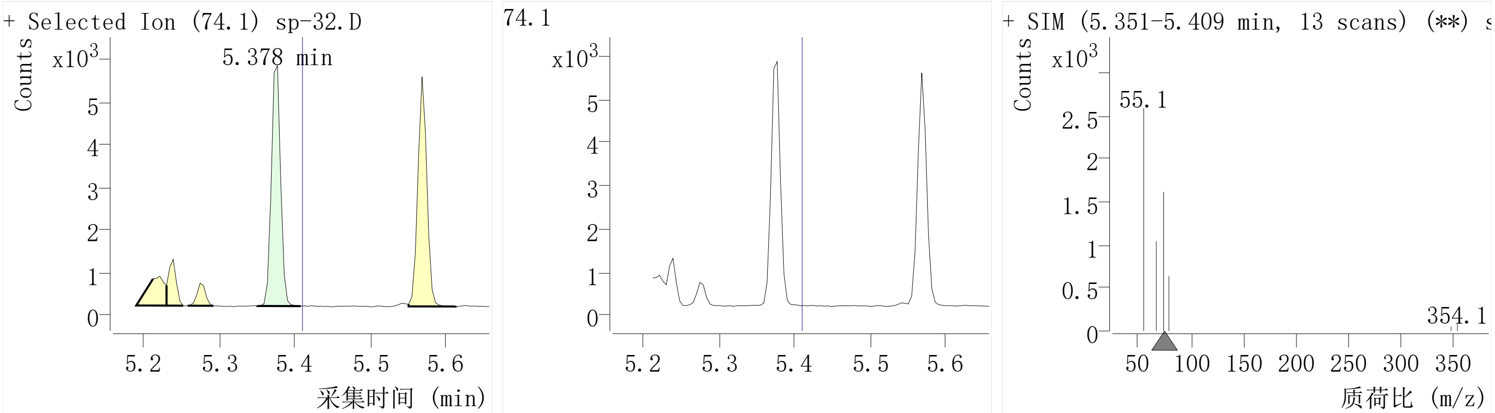

C14:0

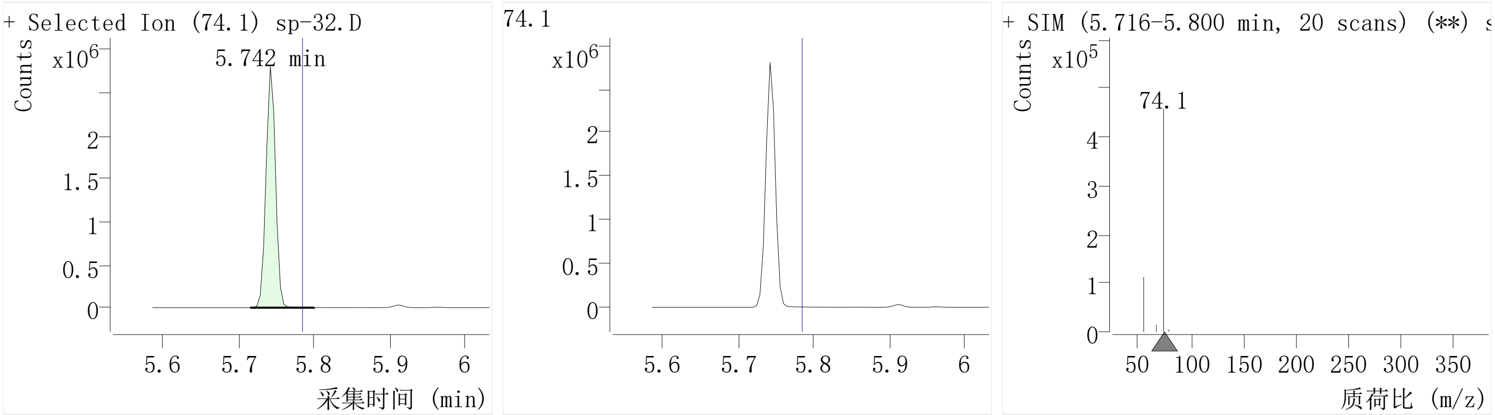

C14:1

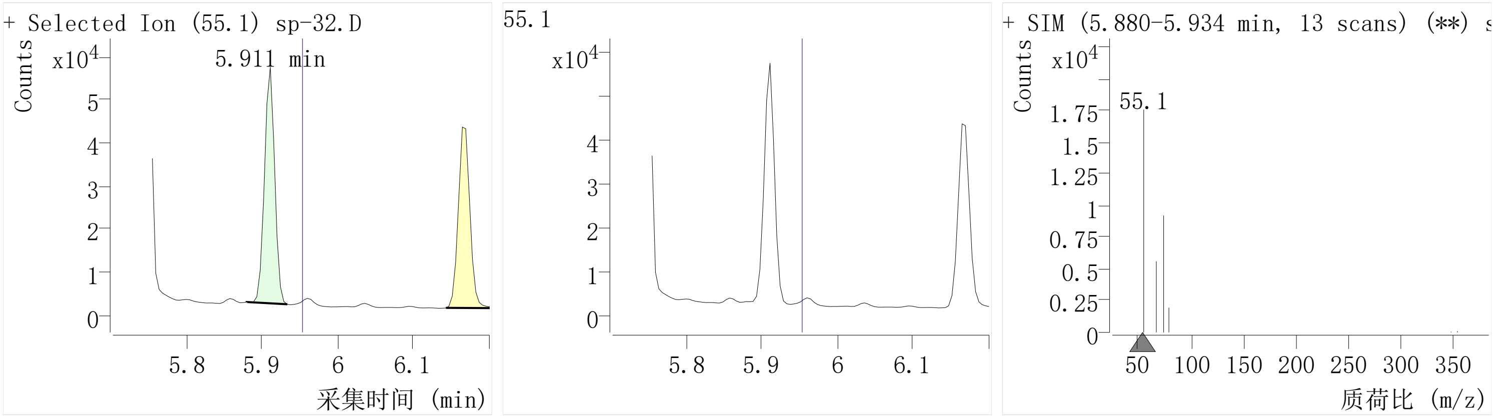

C15:0

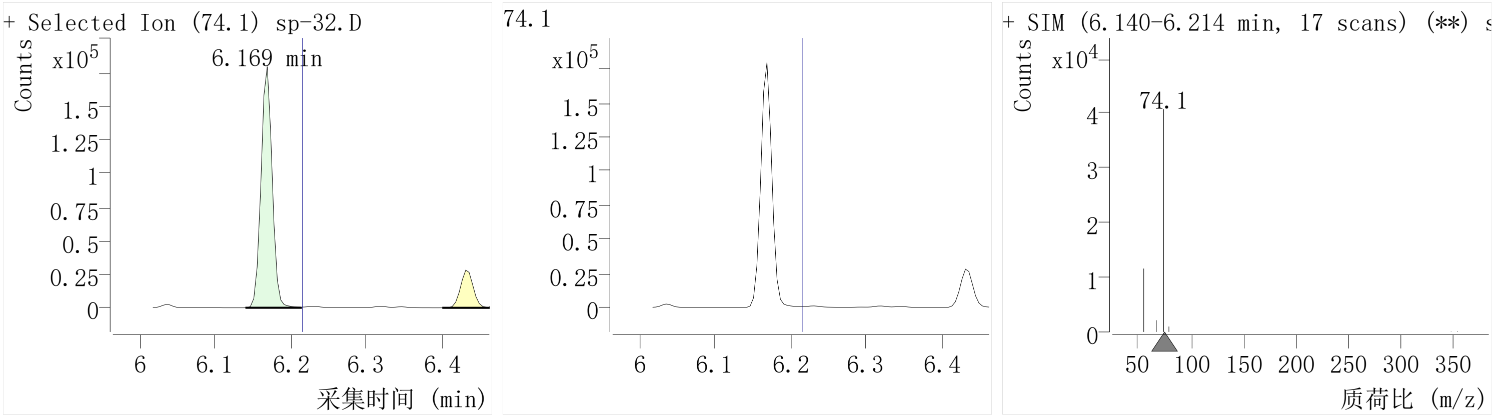

C15:1

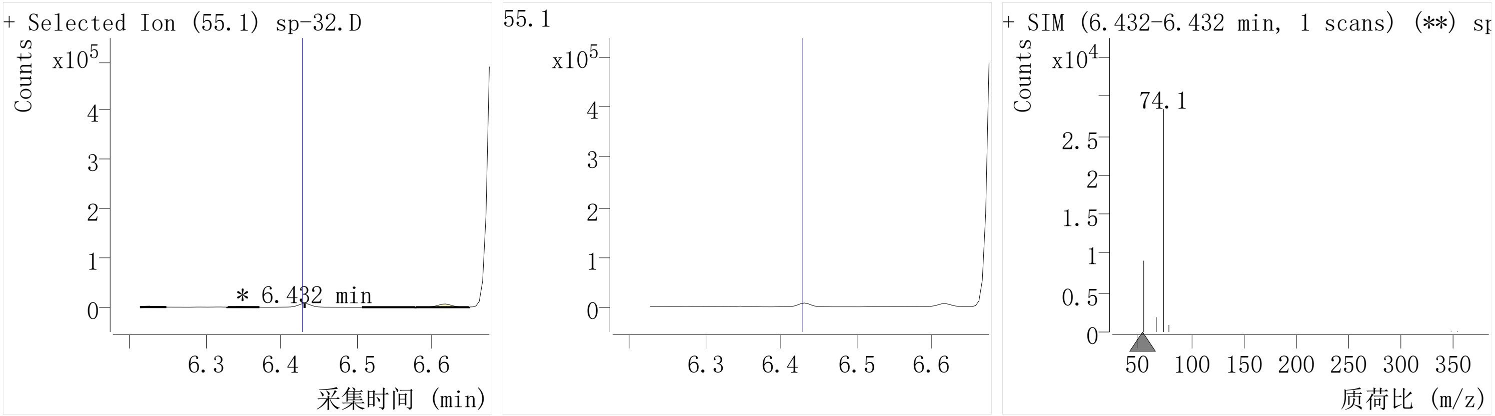

C16:0

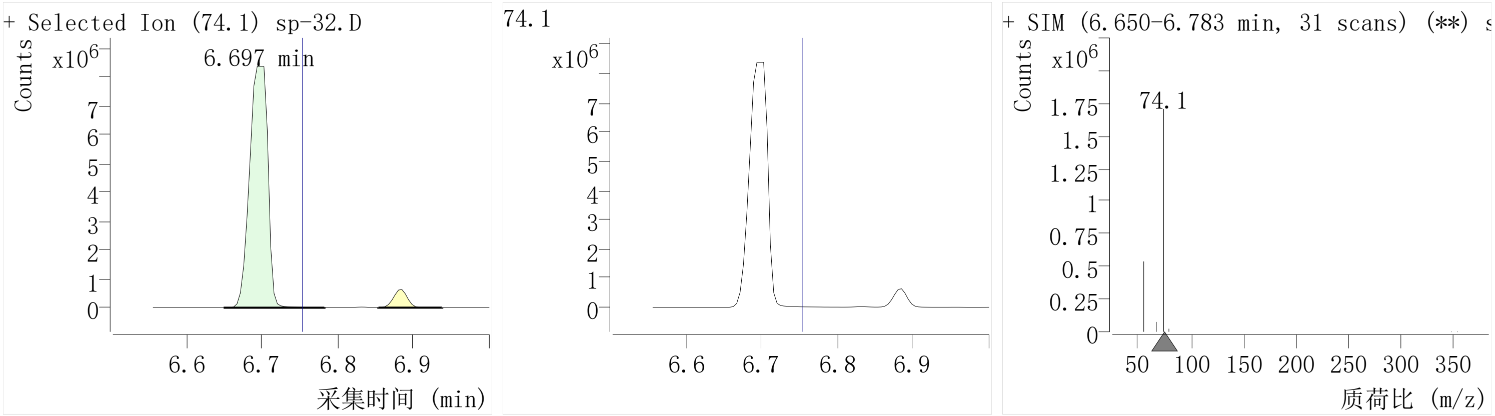

C16:1

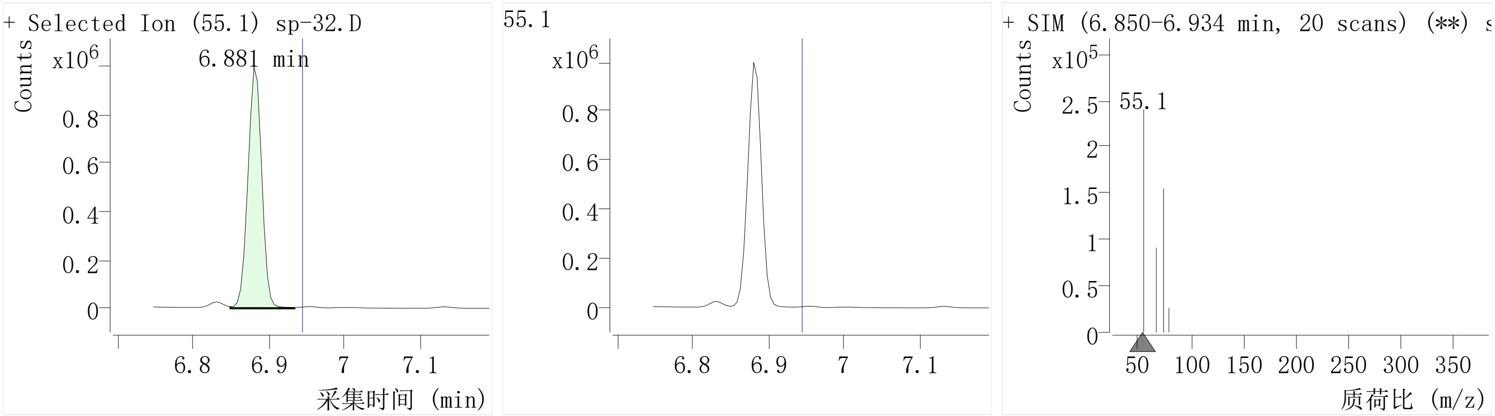

C17:0

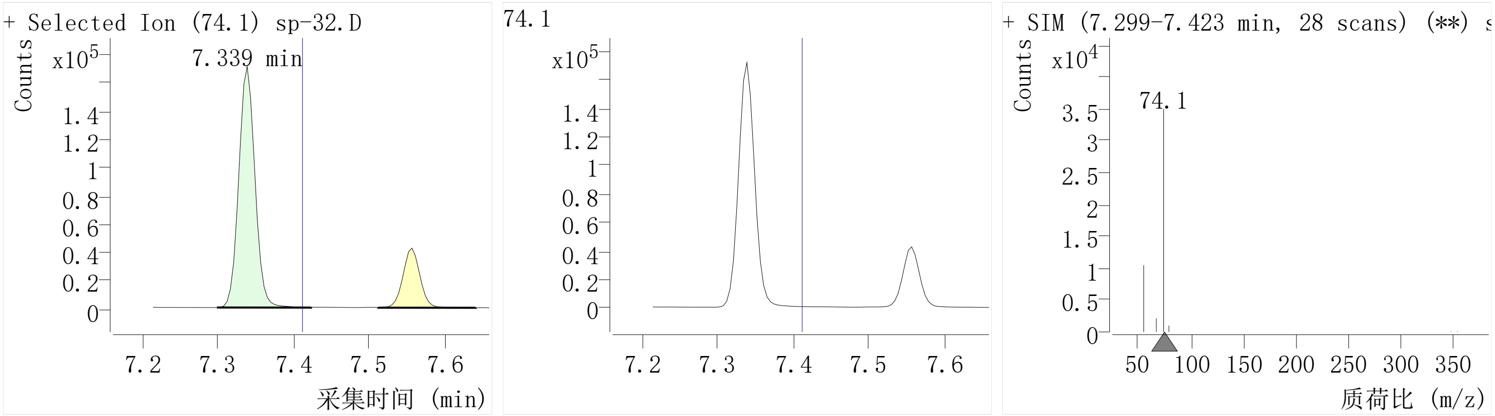

C17:1

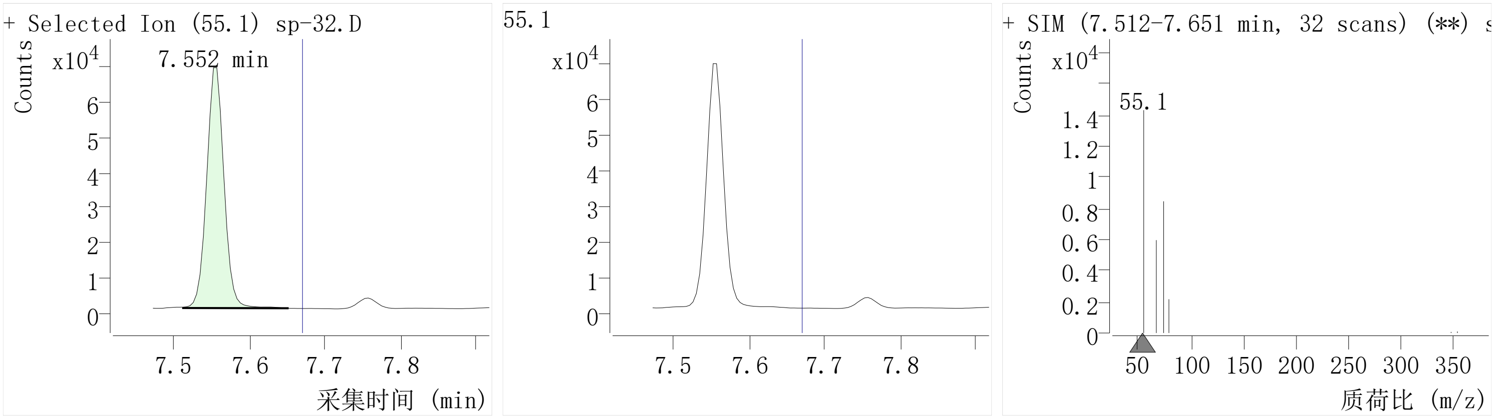

C18:0

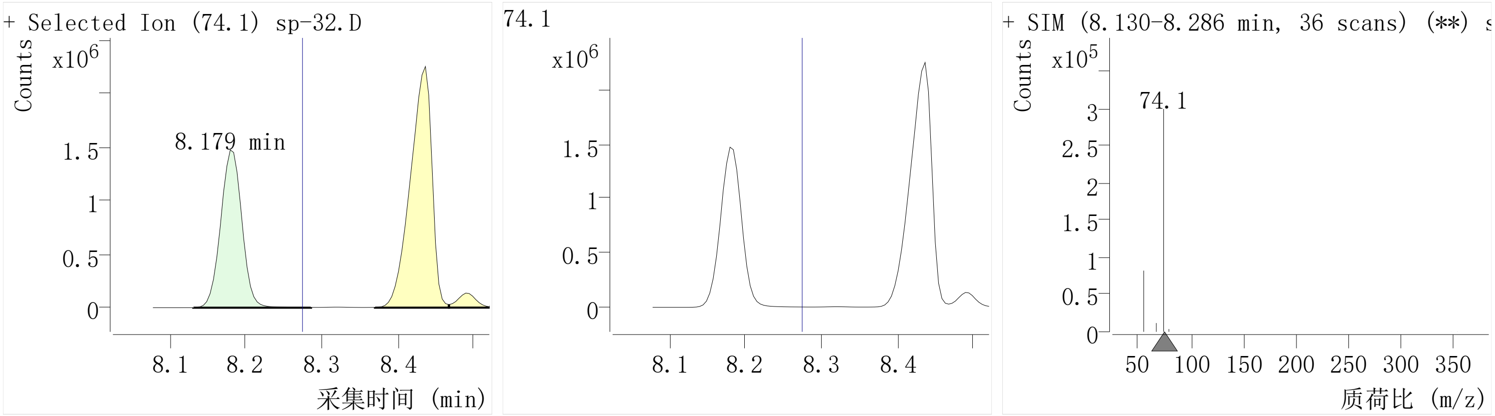

C18:1n9t

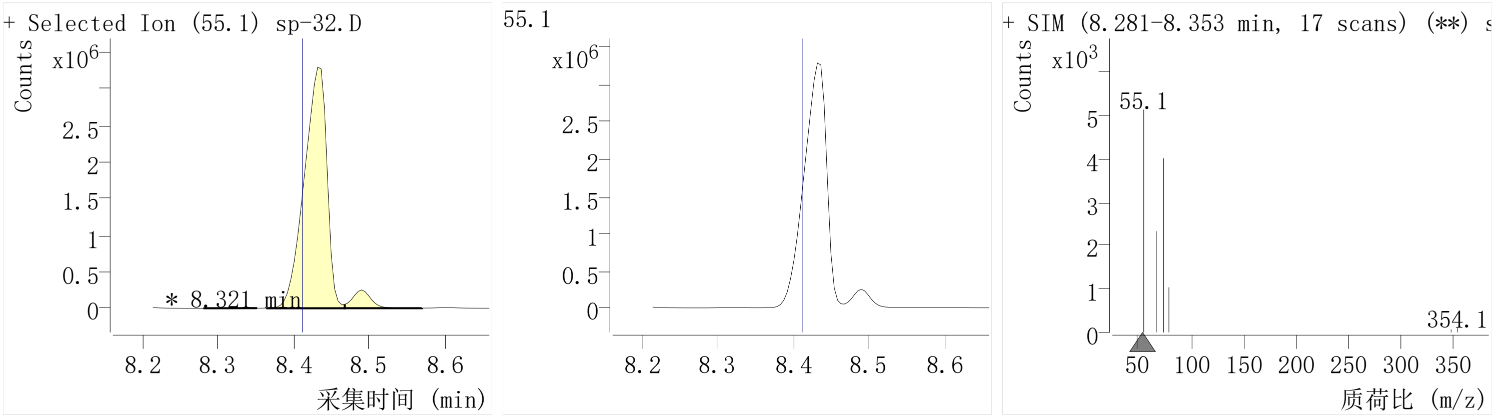

C18:1n9c

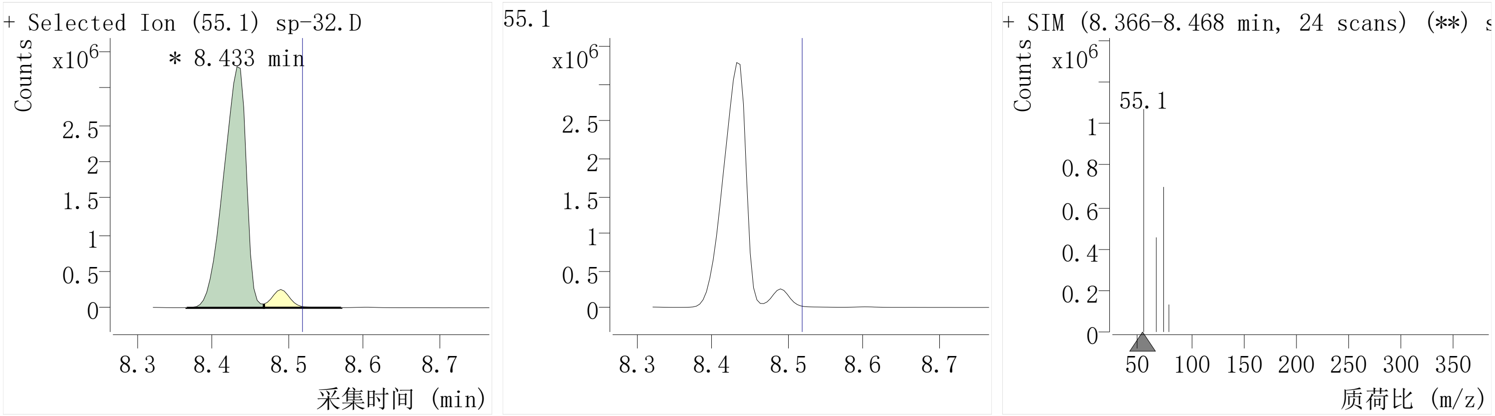

C18:2n6t

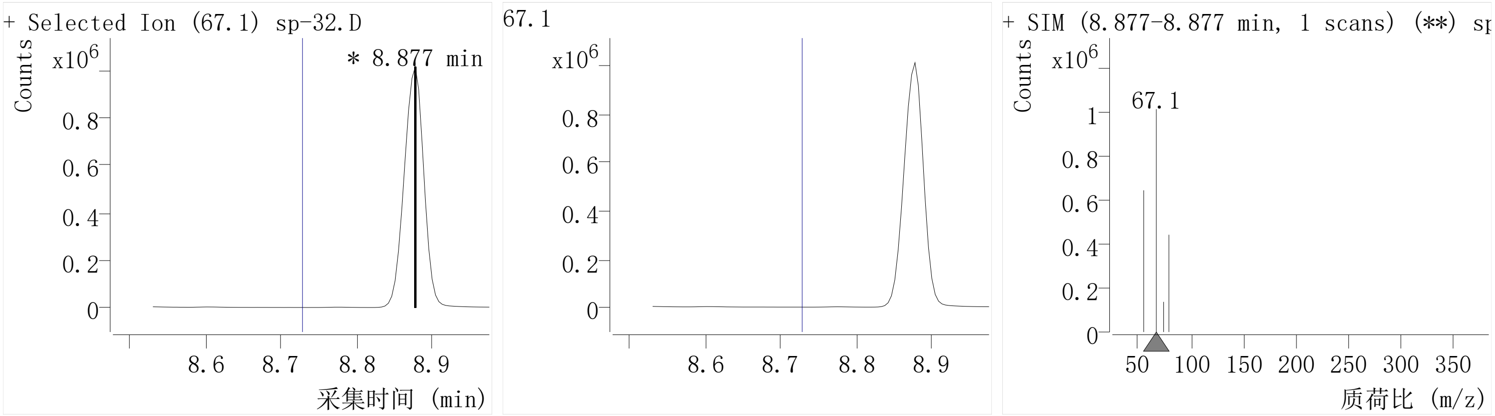

C18:2n6c

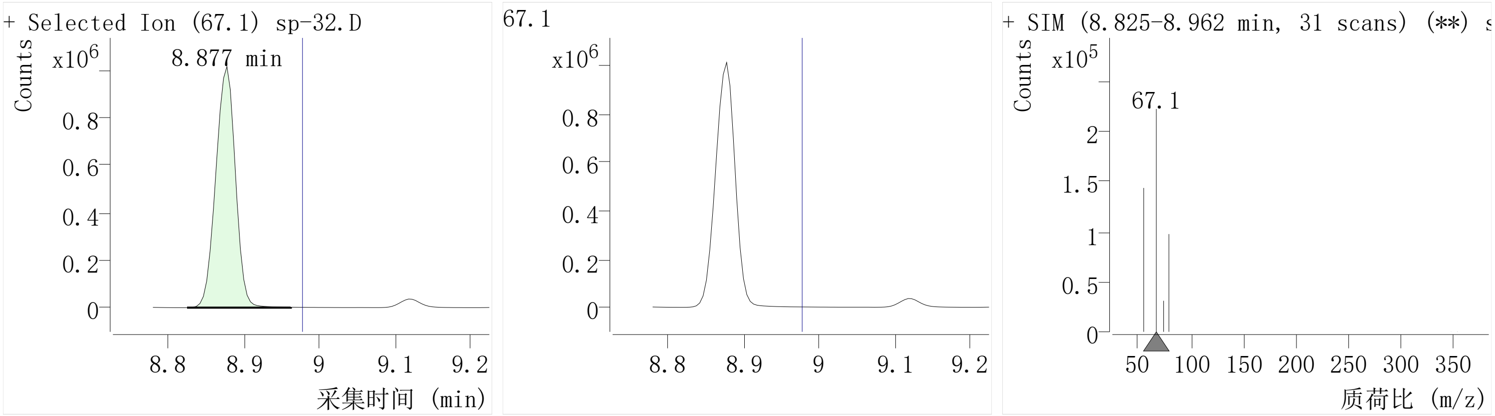

C18:3n6

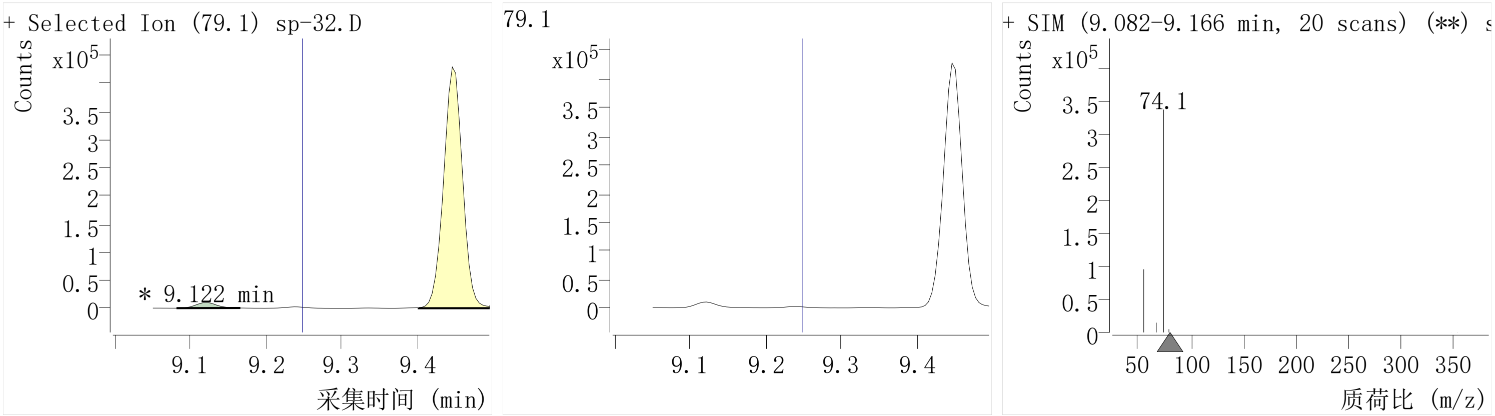

C18:3n3

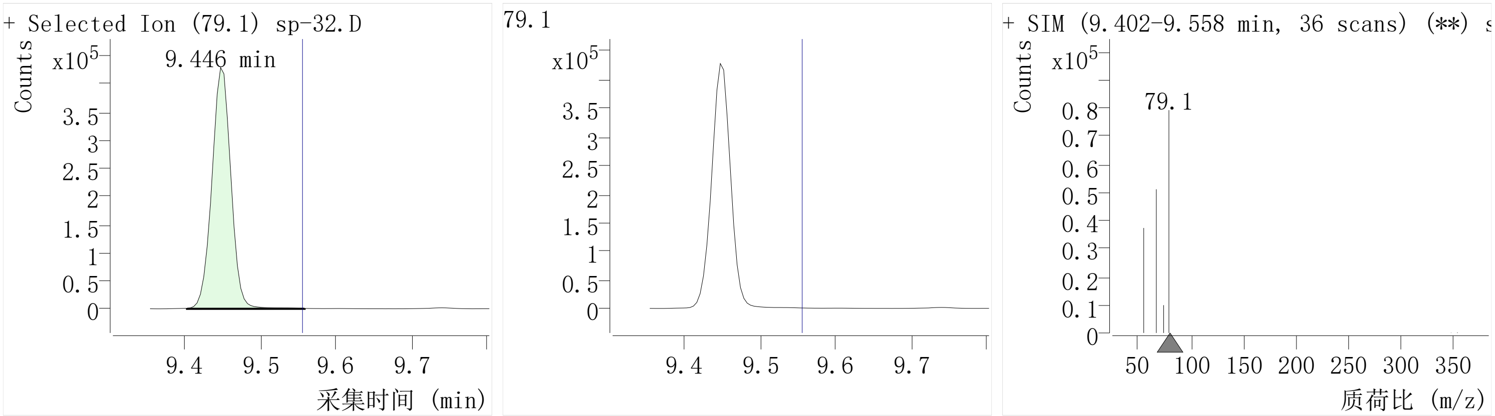

C20:0

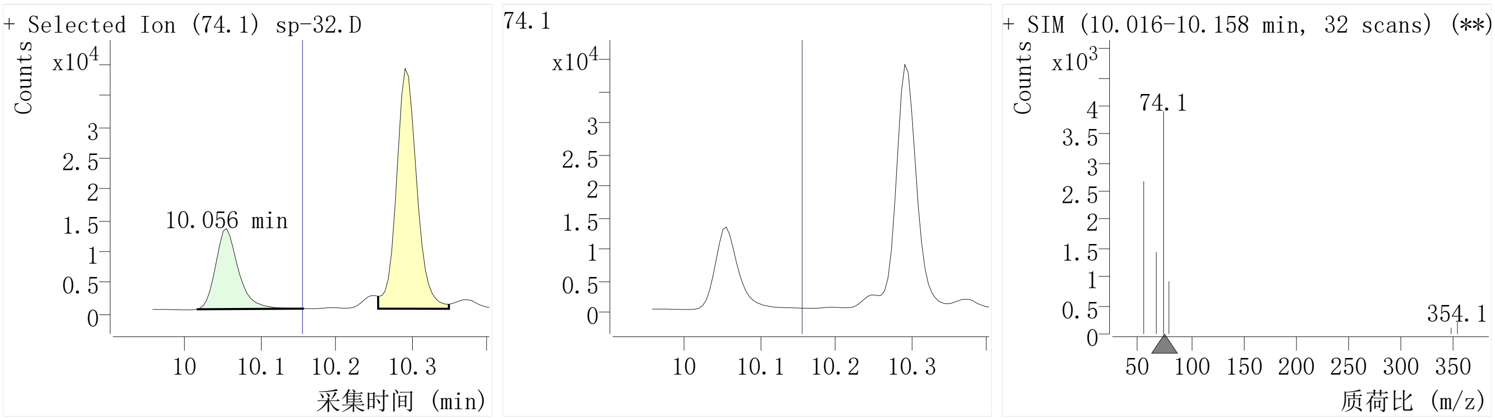

C20:1

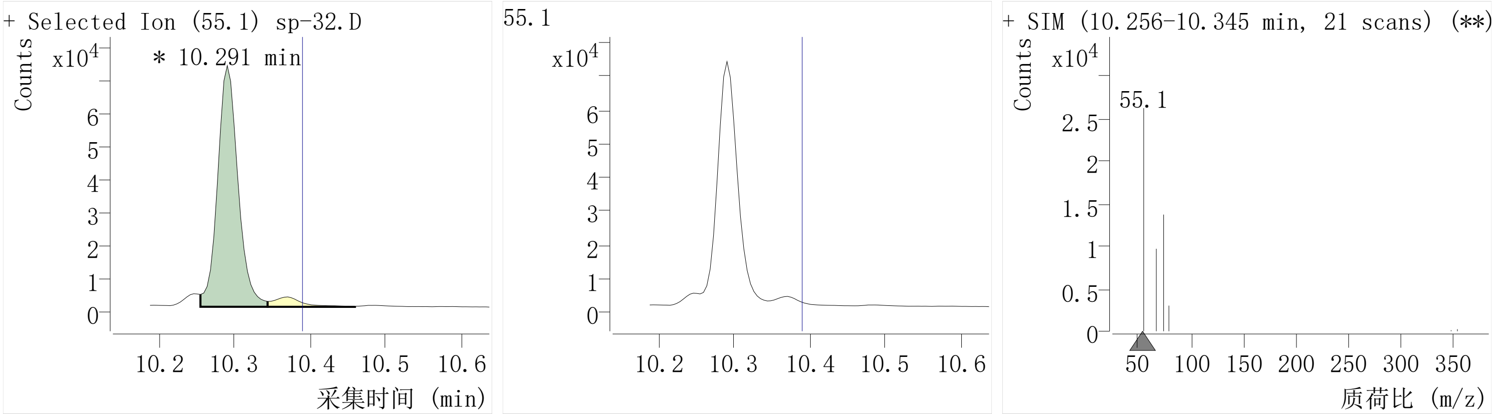

C20:2

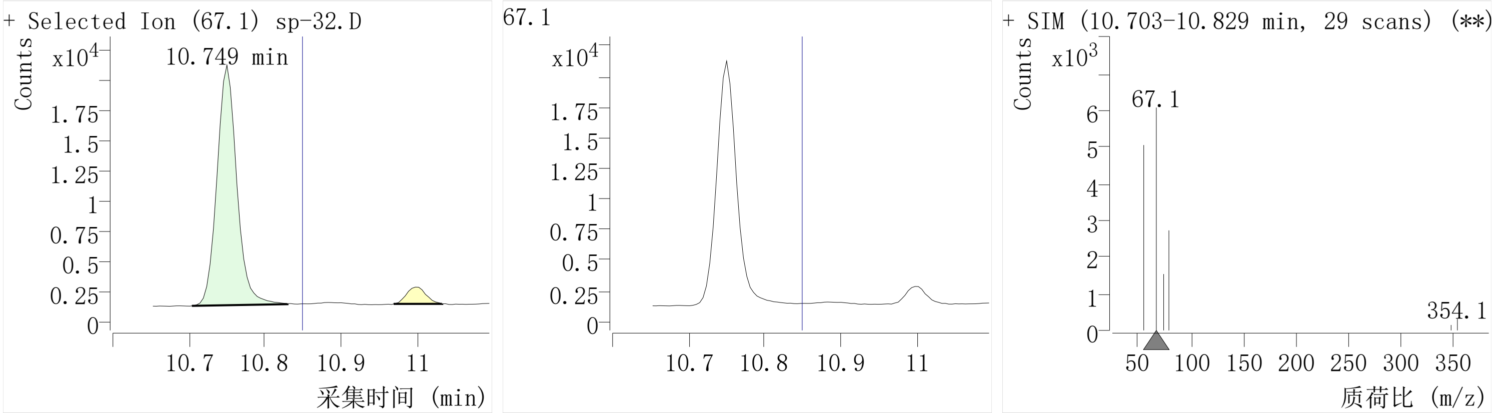

C21:0

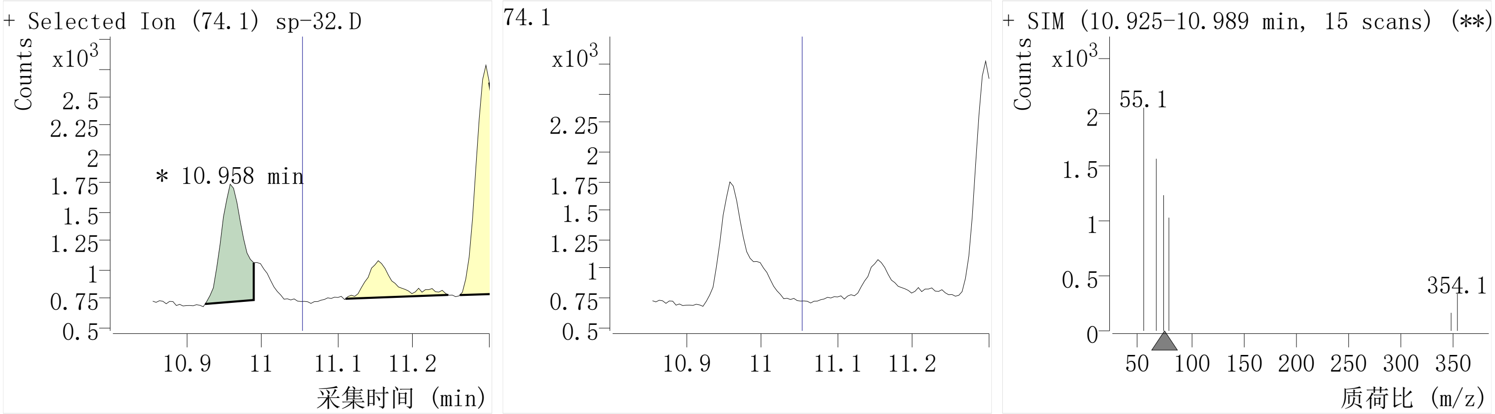

C20:3n6

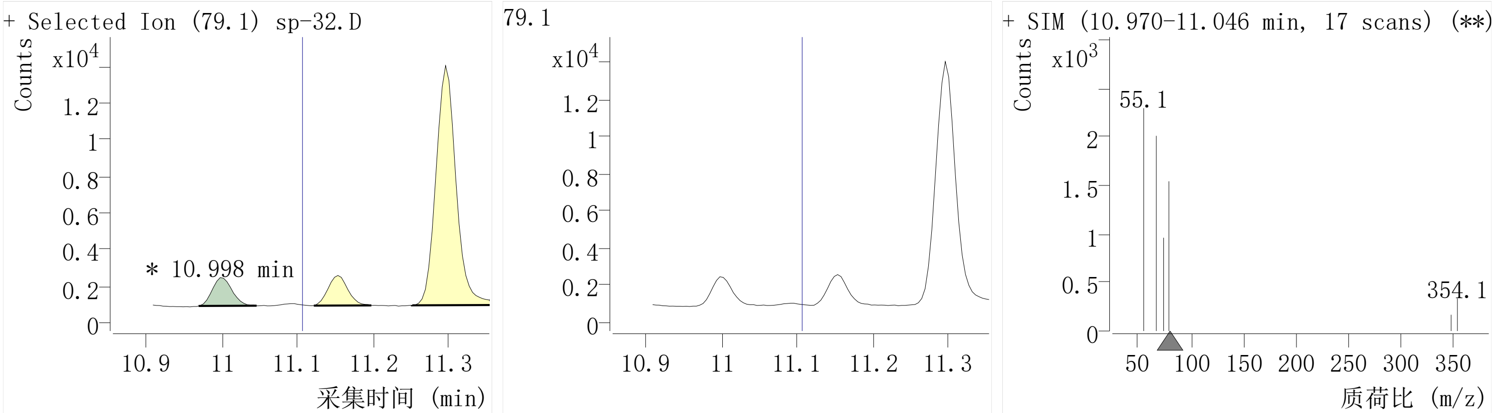

C20:4n6

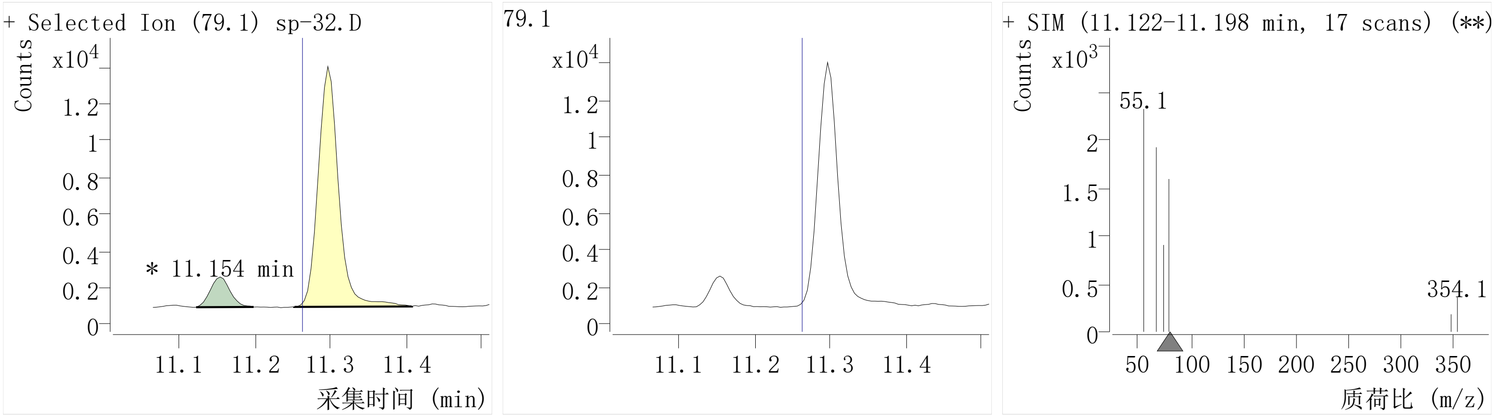

C20:3n3

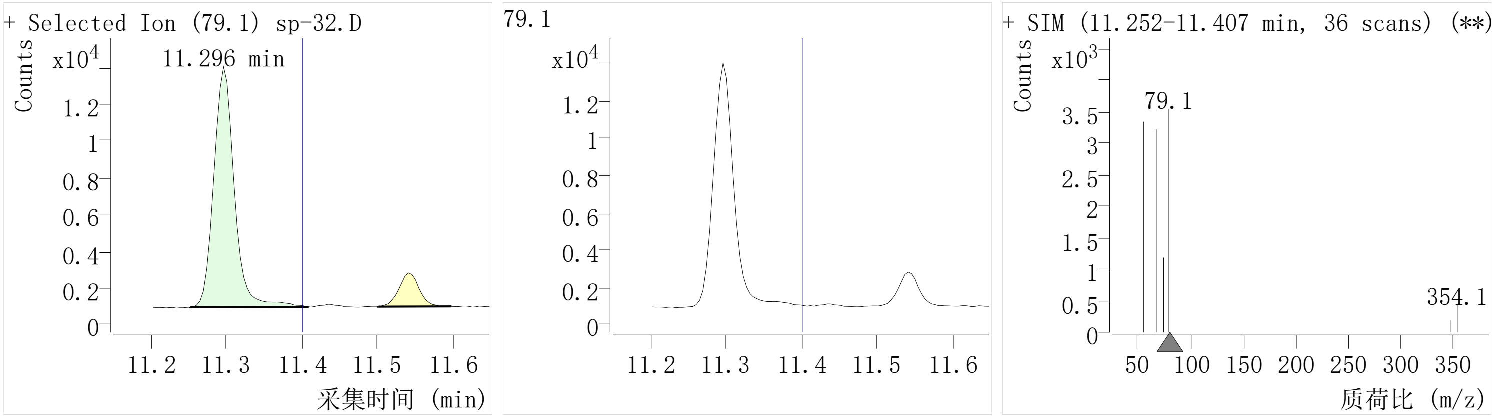

C20:5n3

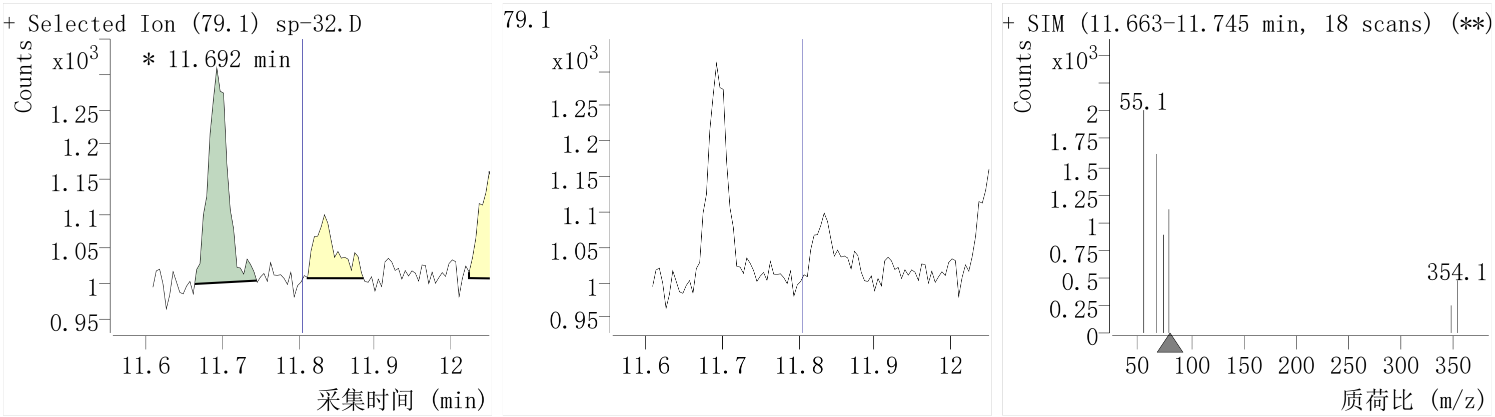

C22:0

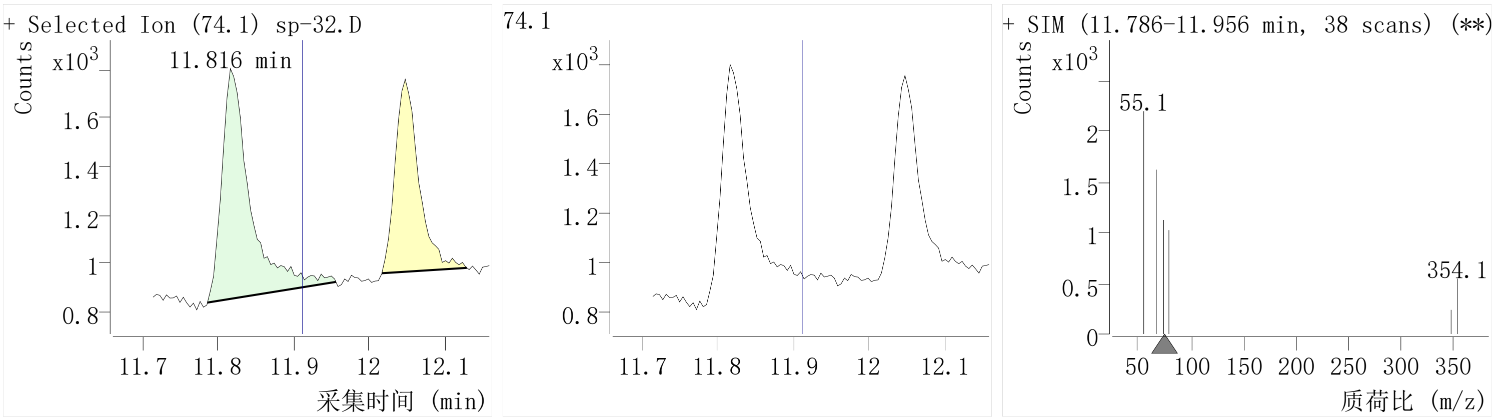

C22:1n9

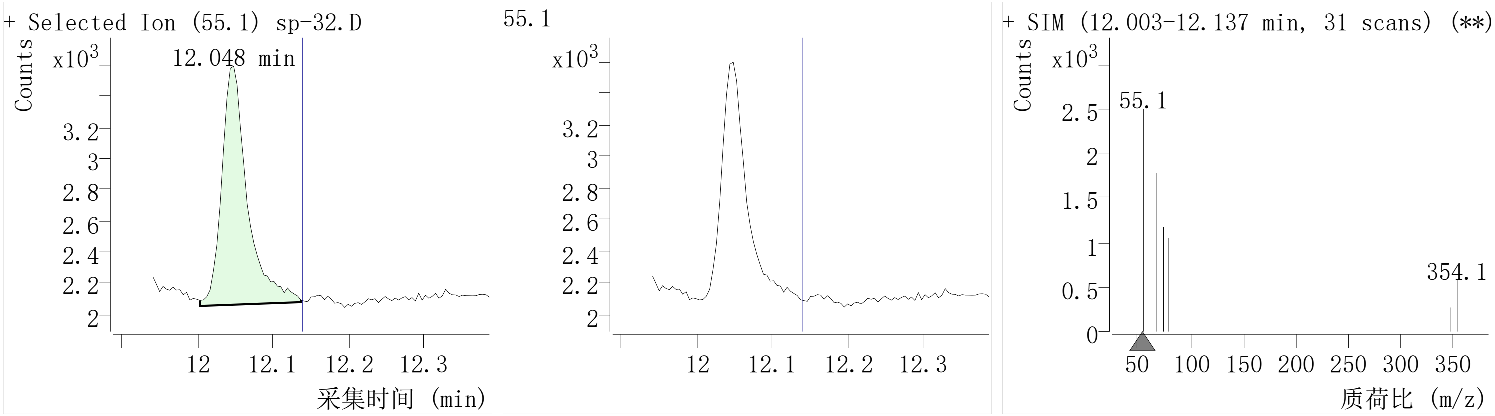

C22:2n6

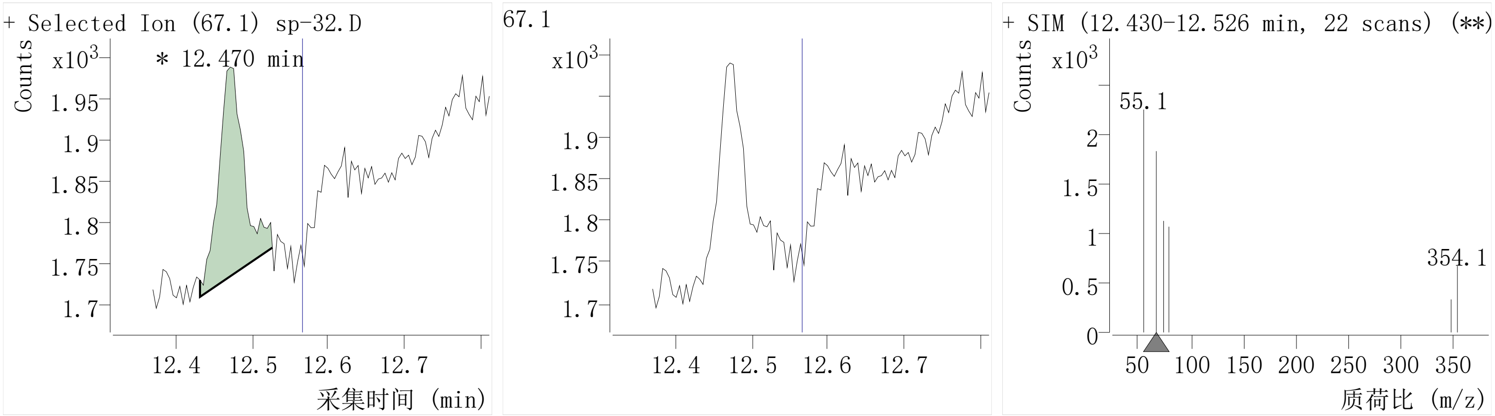

C23:0

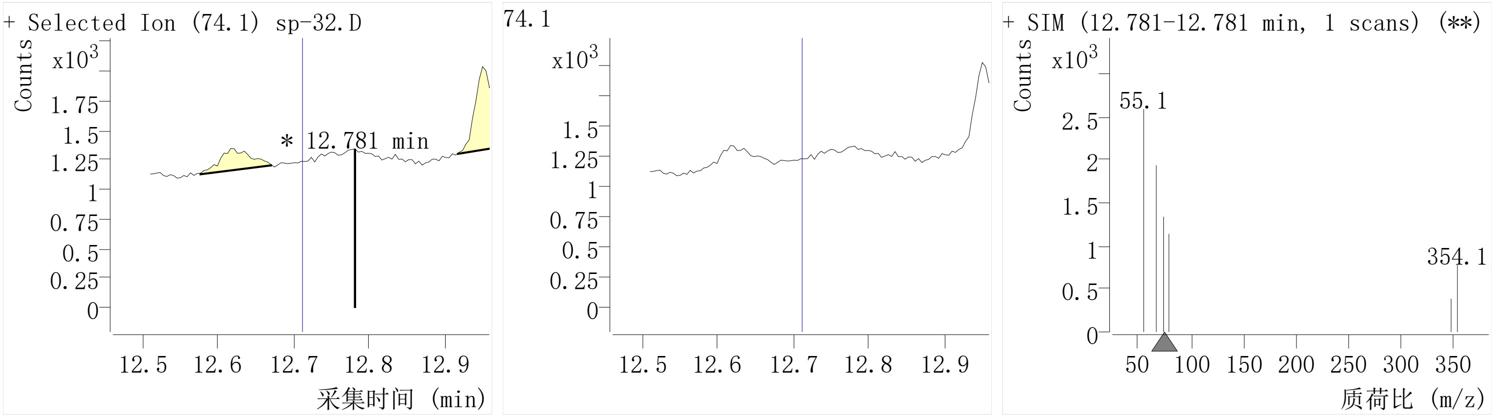

C24:0

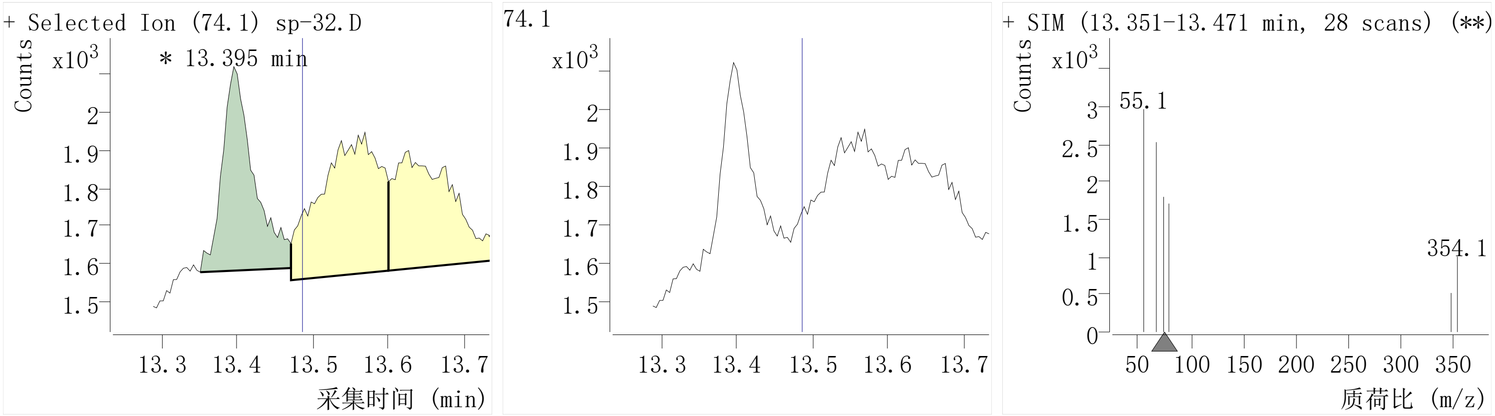

C22:6

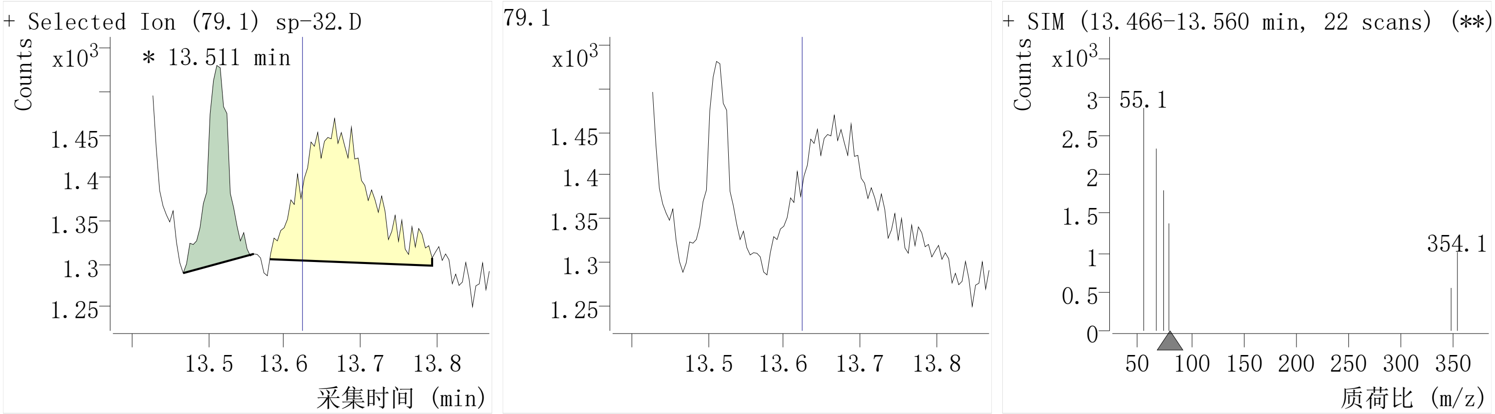
+ Selected Ion (79.1) sp-32.D
Counts
x10^3
* 13.511 min
1.45
1.4
1.35
1.3
1.25
13.5
13.6
13.7
13.8
采集时间 (min)
79.1
+ SIM (13.466-13.560 min, 22 scans) (**)
55.1
354.1
50
100
150
200
250
300
350
质荷比 (m/z)

C24:1

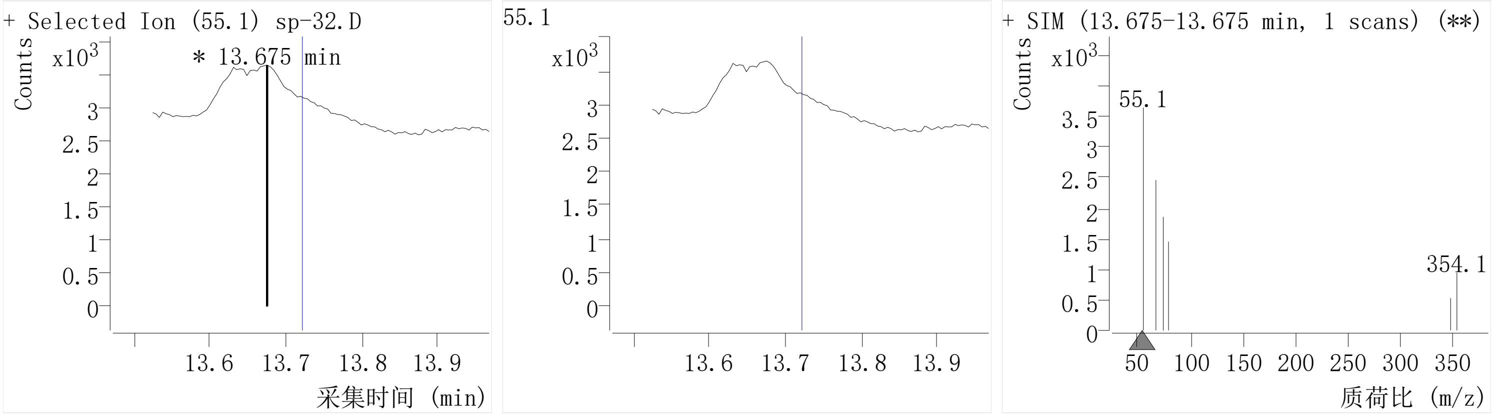
+ Selected Ion (55.1) sp-32.D
Counts
x10^3
* 13.675 min
3
2.5
2
1.5
1
0.5
0
13.6
13.7
13.8
13.9
采集时间 (min)
55.1
+ SIM (13.675-13.675 min, 1 scans) (**)
3.5
55.1
354.1
50
100
150
200
250
300
350
质荷比 (m/z)

定量分析完成报告

**批处理路径**	G:\GC-MS\HX250430-4-GCMS总脂肪酸靶向检测\HX250430-4\QuantResults\HX250430-4.batch.bin		
**分析时间**	2025/5/14 16:58	**分析员姓名**	DESKTOP-M3A0GPO\omics
**报告时间**	2025/5/16 14:53:40	**报告员姓名**	DESKTOP-M3A0GPO\omics
**最近校正更新**	2025/5/14 16:58	**批处理状态**	已处理
**定量批处理版本**	10.2	**定量报告版本**	10.2

**采集时间**	2025/5/9 9:38	**数据文件**	qc-5.D
**样品类型**	样品	**样品名称**	qc-5
**稀释**	1	**采集方法**	脂肪酸

样品色谱图

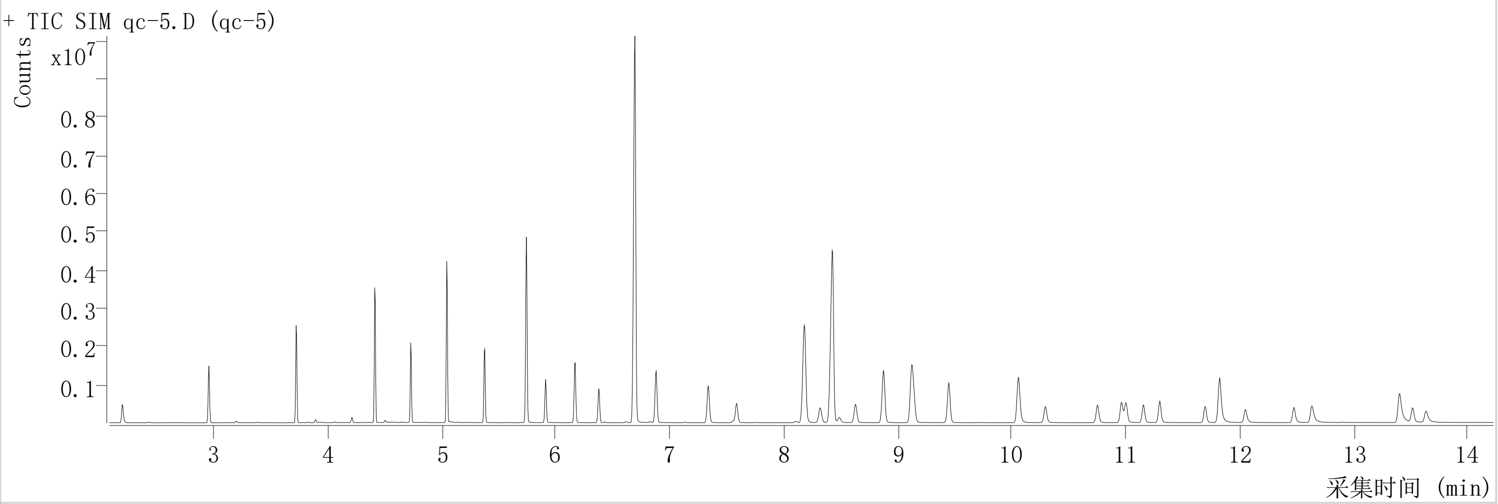

化合物	ISTD	RT	响应	ISTD 响应	响应比	最终浓度	单位
C4:0	C19:0	2.198	470662	2038657	0.2309	45.3041	ug/ml
C6:0	C19:0	2.959	1063853	2038657	0.5218	64.4037	ug/ml
C8:0	C19:0	3.724	1588662	2038657	0.7793	58.3737	ug/ml
C10:0	C19:0	4.413	2117114	2038657	1.0385	42.9392	ug/ml
C11:0	C19:0	4.729	1185016	2038657	0.5813	19.2746	ug/ml
C12:0	C19:0	5.044	2492631	2038657	1.2227	35.5837	ug/ml
C13:0	C19:0	5.378	1266917	2038657	0.6214	15.6621	ug/ml
C14:0	C19:0	5.742	3333821	2038657	1.6353	38.4634	ug/ml
C14:1	C19:0	5.911	533690	2038657	0.2618	13.0980	ug/ml
C15:0	C19:0	6.169	1238365	2038657	0.6074	13.0717	ug/ml
C15:1	C19:0	6.378	511674	2038657	0.2510	12.6660	ug/ml
C16:0	C19:0	6.694	10239313	2038657	5.0226	166.1215	ug/ml
C16:1	C19:0	6.881	817839	2038657	0.4012	24.4273	ug/ml
C17:0	C19:0	7.339	1104091	2038657	0.5416	11.2989	ug/ml
C17:1	C19:0	7.583	421295	2038657	0.2067	11.1011	ug/ml
C18:0	C19:0	8.179	3767970	2038657	1.8483	40.1359	ug/ml
C18:1n9t	C19:0	8.317	348694	2038657	0.1710	10.0896	ug/ml
C18:1n9c	C19:0	8.424	3903978	2038657	1.9150	123.3427	ug/ml
C18:2n6t	C19:0	8.628	387449	2038657	0.1901	10.4373	ug/ml
C18:2n6c	C19:0	8.873	1091184	2038657	0.5352	30.7062	ug/ml
C18:3n6	C19:0	9.140	392643	2038657	0.1926	10.0321	ug/ml
C18:3n3	C19:0	9.446	819004	2038657	0.4017	19.9674	ug/ml
C20:0	C19:0	10.056	1753502	2038657	0.8601	21.7300	ug/ml
C20:1	C19:0	10.291	398394	2038657	0.1954	12.0826	ug/ml
C20:2	C19:0	10.749	357368	2038657	0.1753	10.7664	ug/ml
C21:0	C19:0	10.963	855472	2038657	0.4196	11.5440	ug/ml
C20:3n6	C19:0	11.003	335624	2038657	0.1646	10.4415	ug/ml
C20:4n6	C19:0	11.154	361306	2038657	0.1772	11.0864	ug/ml
C20:3n3	C19:0	11.296	412218	2038657	0.2022	10.6224	ug/ml
C20:5n3	C19:0	11.696	369956	2038657	0.1815	10.0646	ug/ml

定量分析完成报告

化合物	ISTD	RT	响应	ISTD 响应	响应比	最终浓度	单位
C22:0	C19:0	11.821	1627107	2038657	0.7981	25.8617	ug/ml
C22:1n9	C19:0	12.048	343969	2038657	0.1687	12.4761	ug/ml
C22:2n6	C19:0	12.470	315898	2038657	0.1550	12.2824	ug/ml
C23:0	C19:0	12.630	751553	2038657	0.3687	14.2173	ug/ml
C24:0	C19:0	13.400	1368092	2038657	0.6711	30.2665	ug/ml
C22:6	C19:0	13.515	346340	2038657	0.1699	12.5547	ug/ml
C24:1	C19:0	13.631	344696	2038657	0.1691	14.9078	ug/ml

C4:0

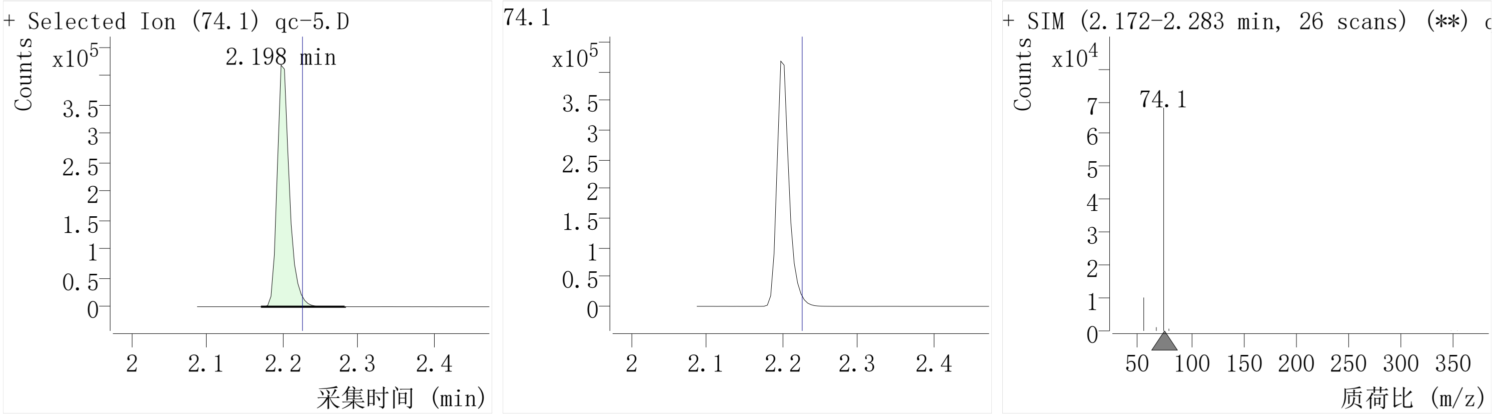

C6:0

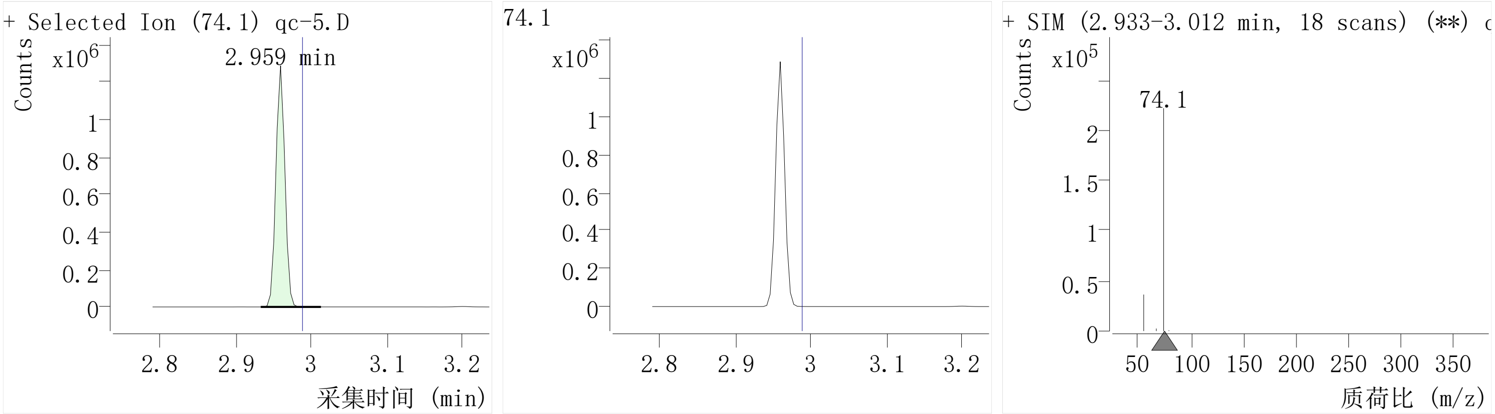

C8:0

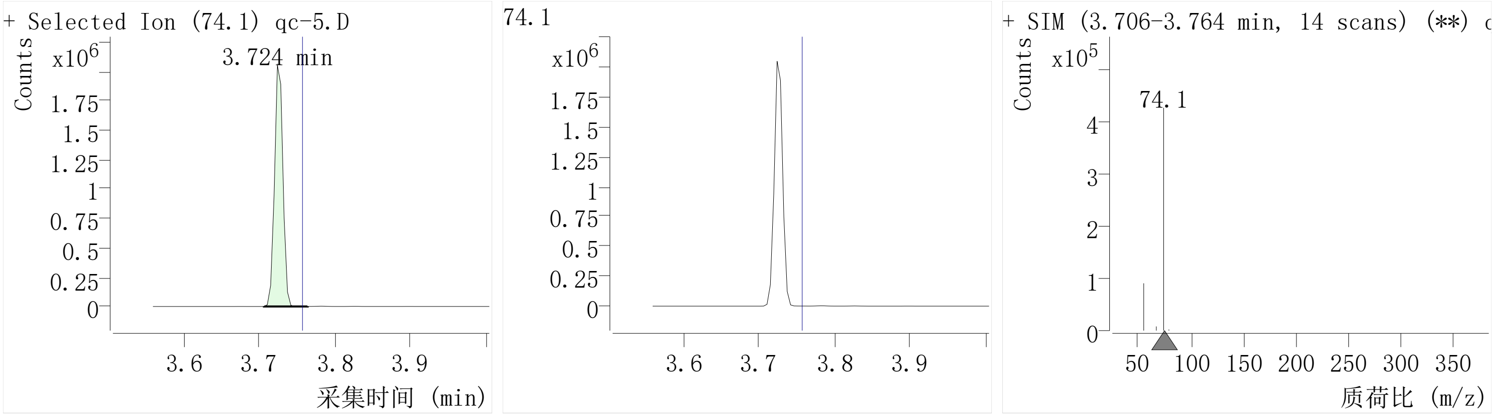

C10:0

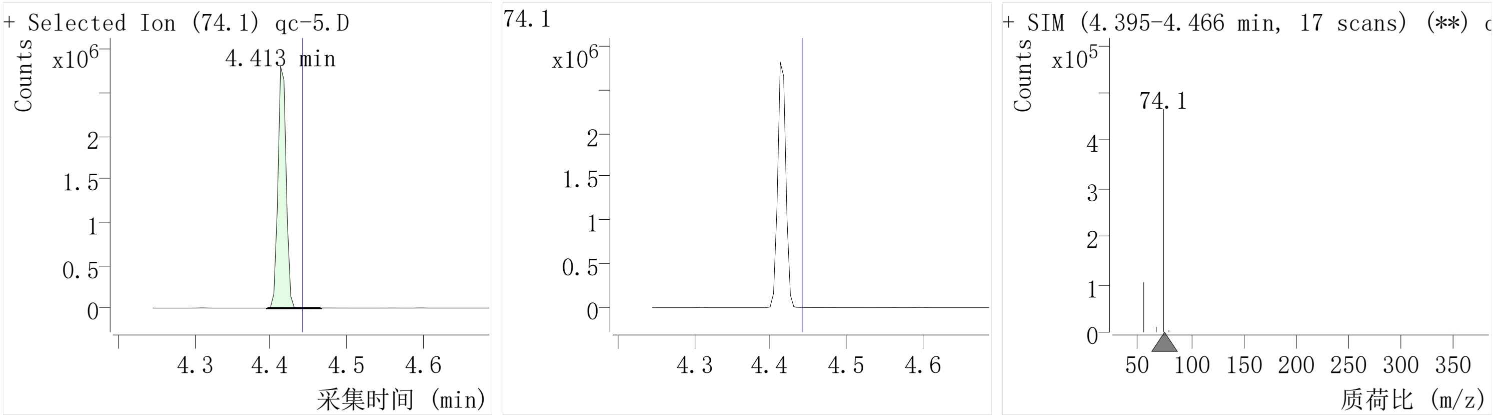

C11:0

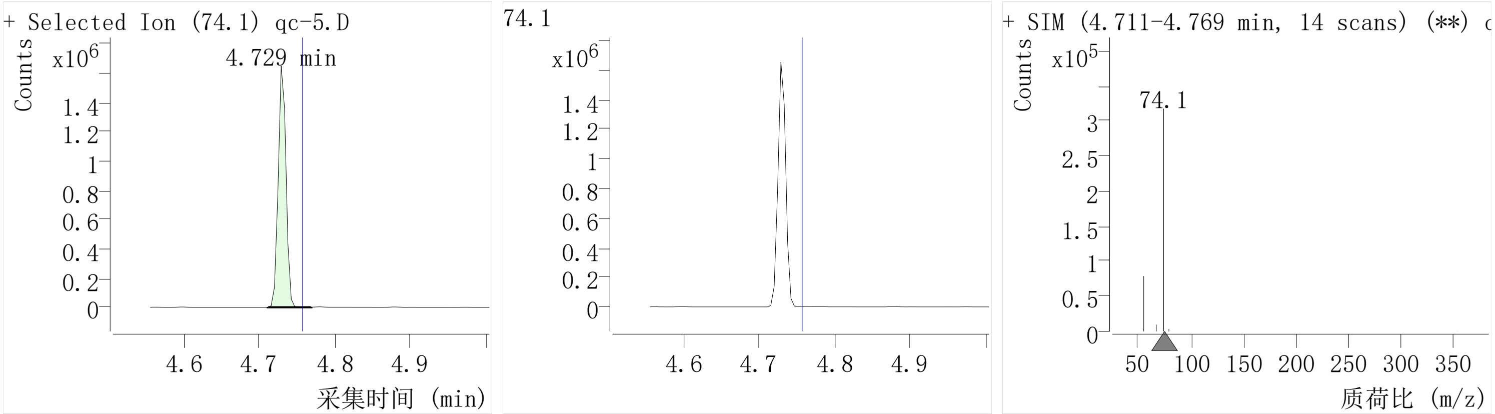

C12:0

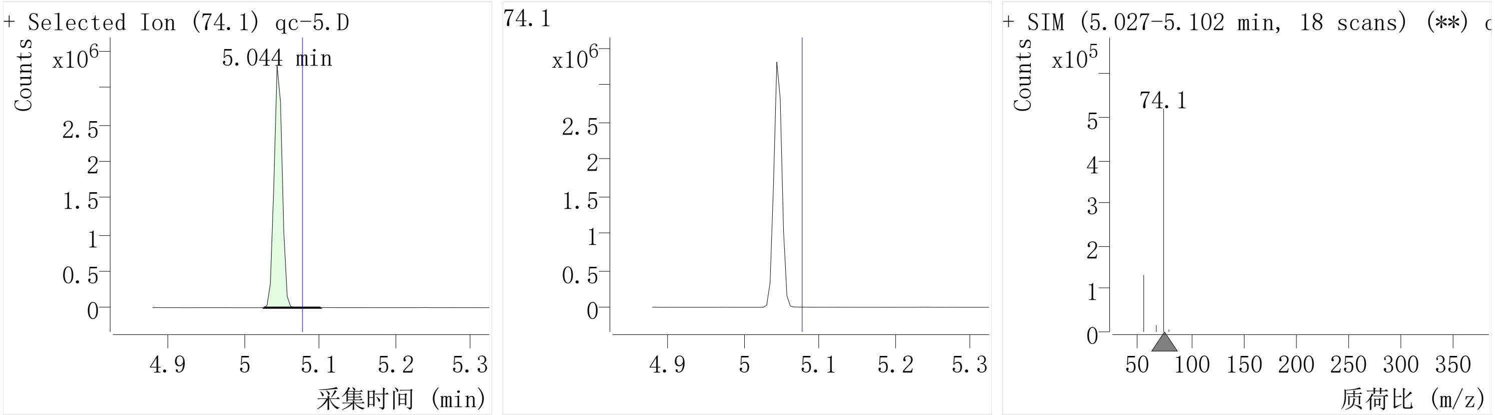

C13:0

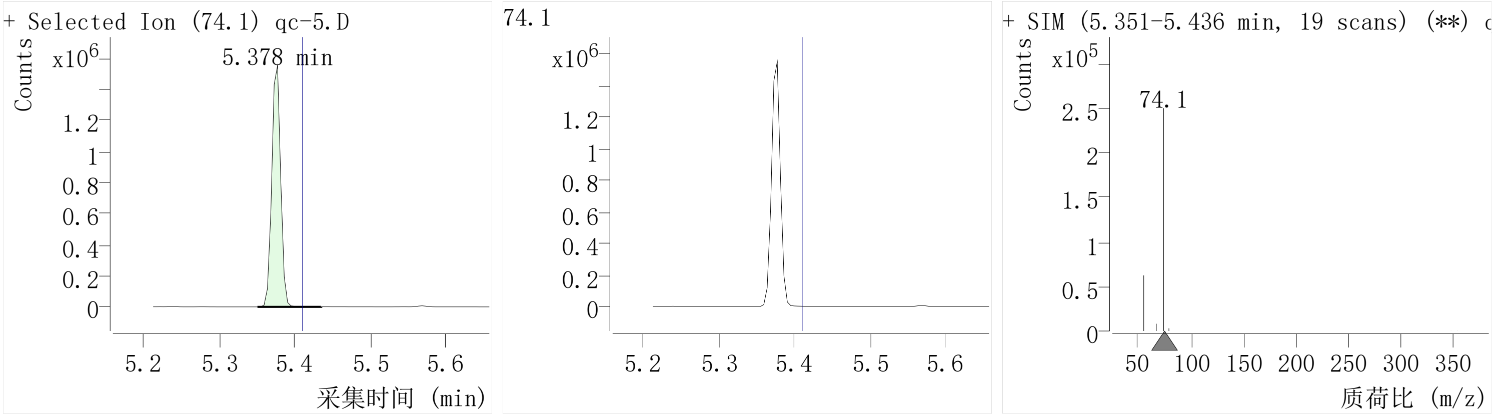

C14:0

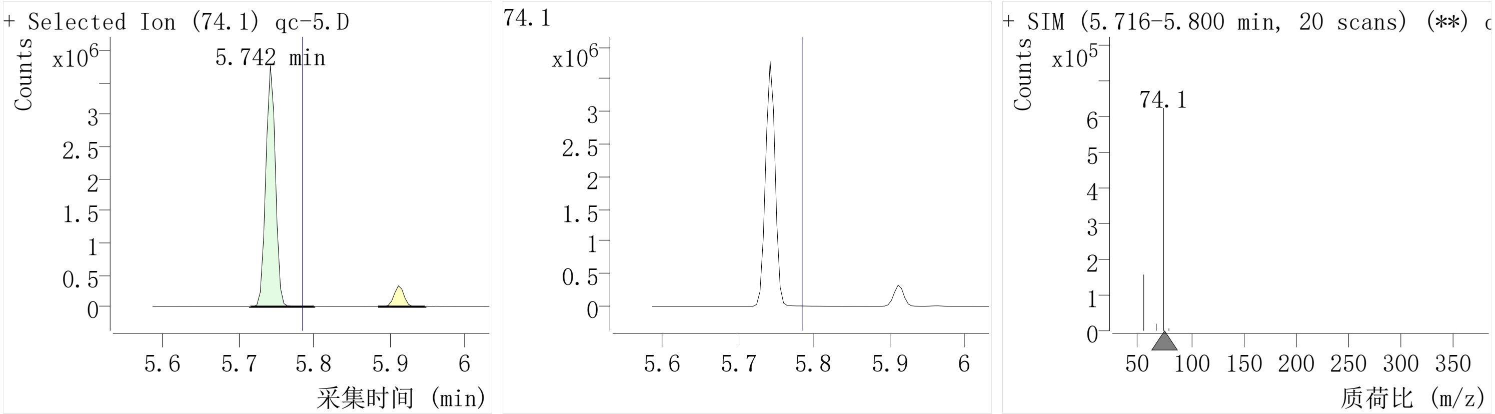

C14:1

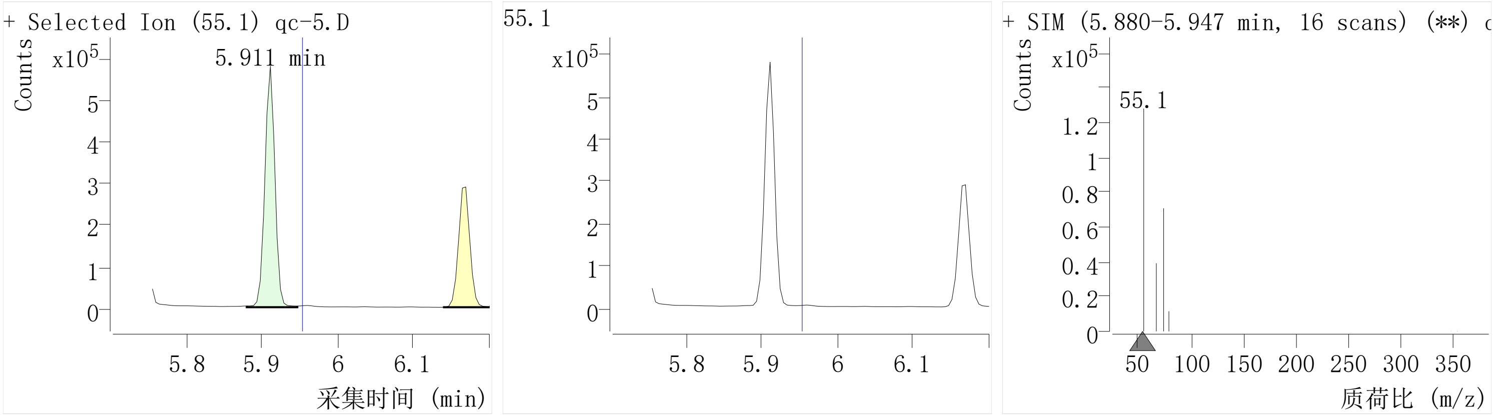

C15:0

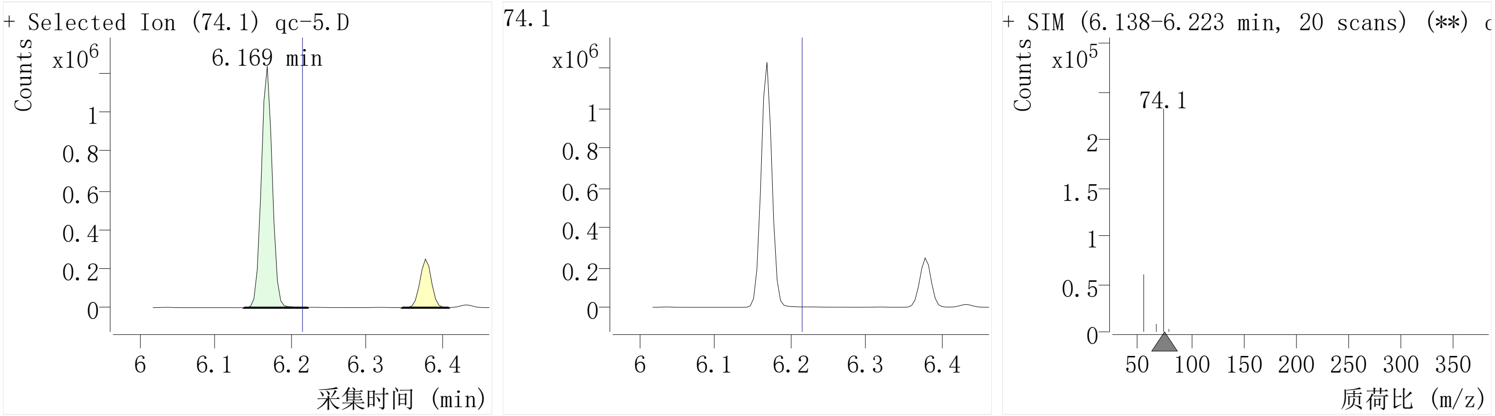

C15:1

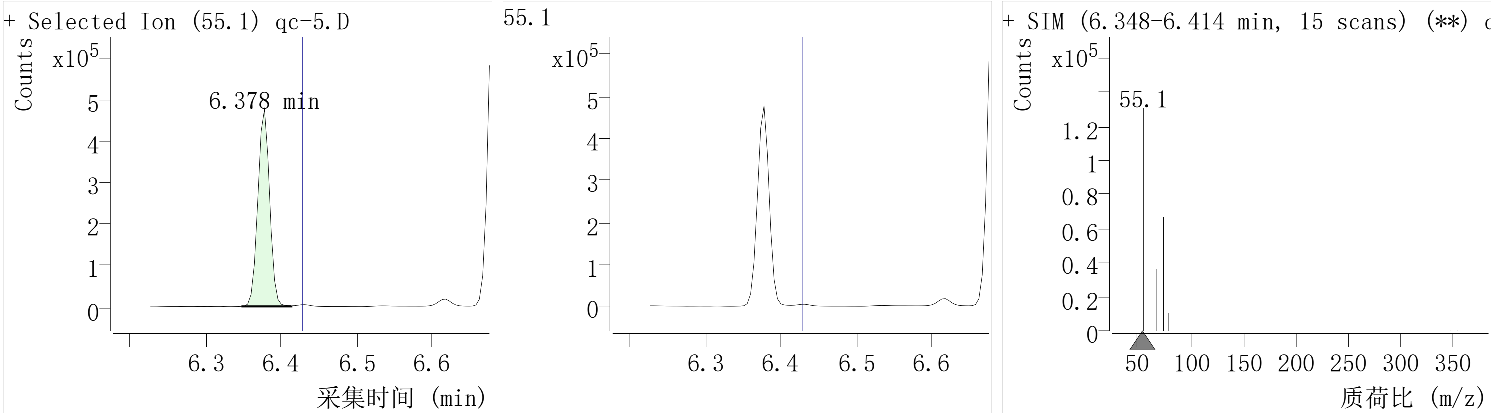

C16:0

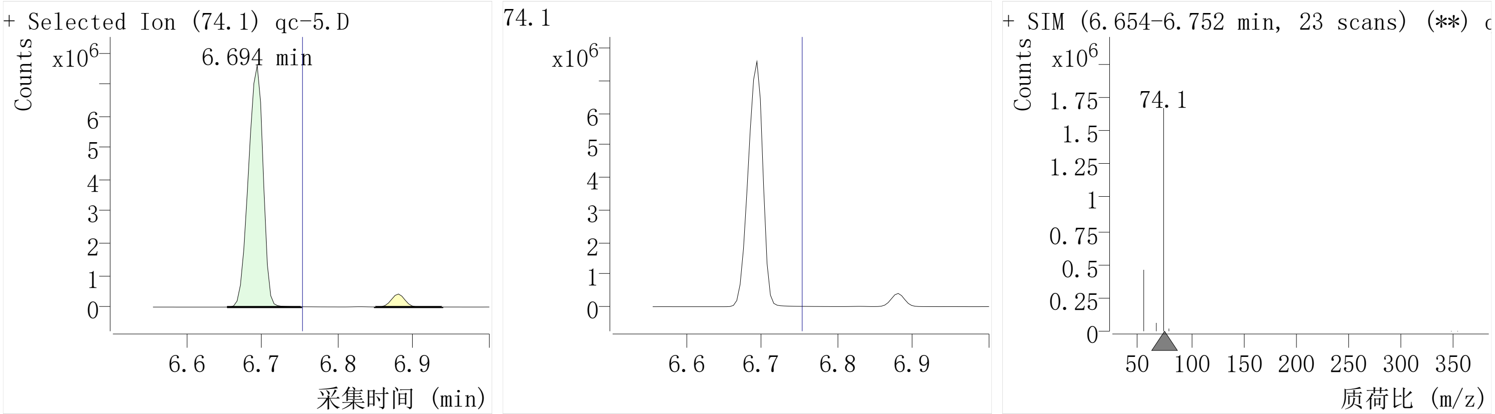

C16:1

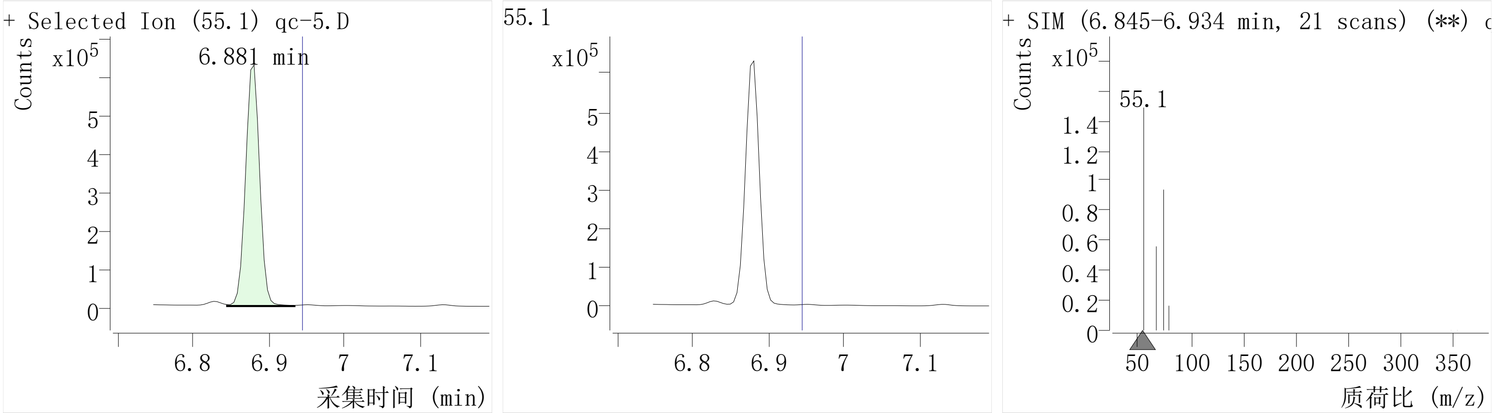

C17:0

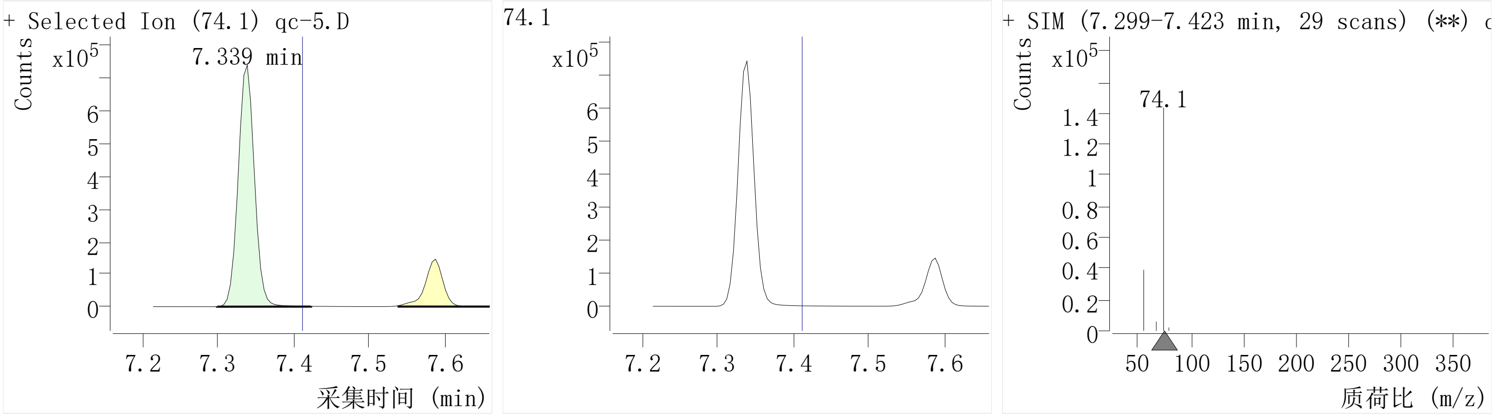

C17:1

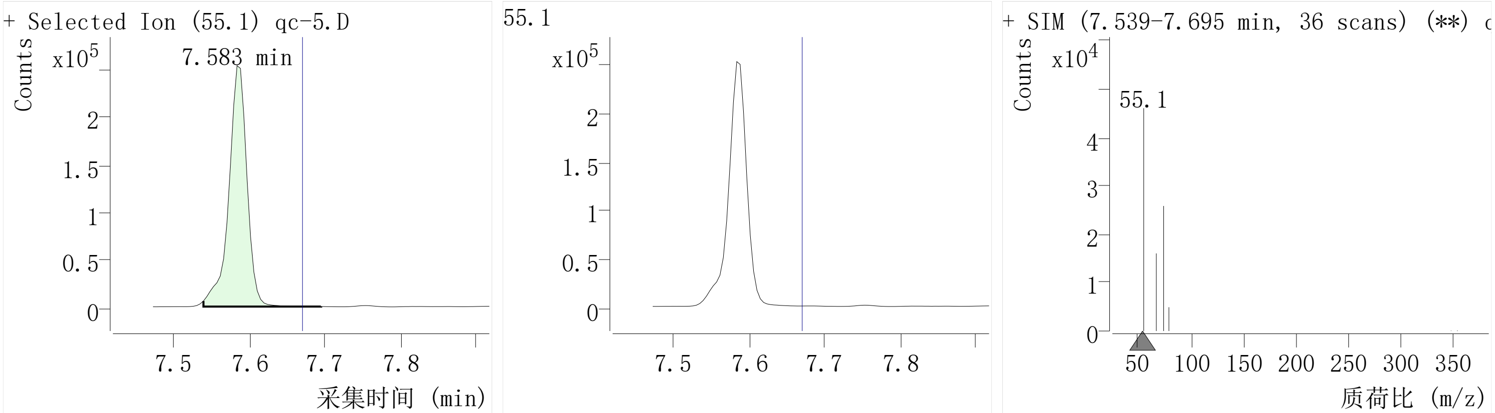

C18:0

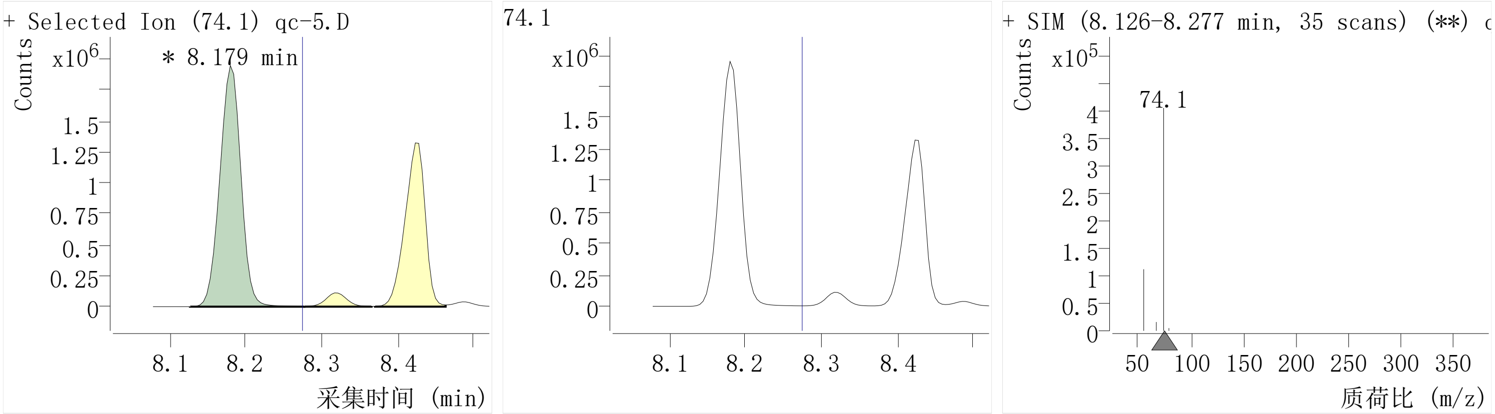

C18:1n9t

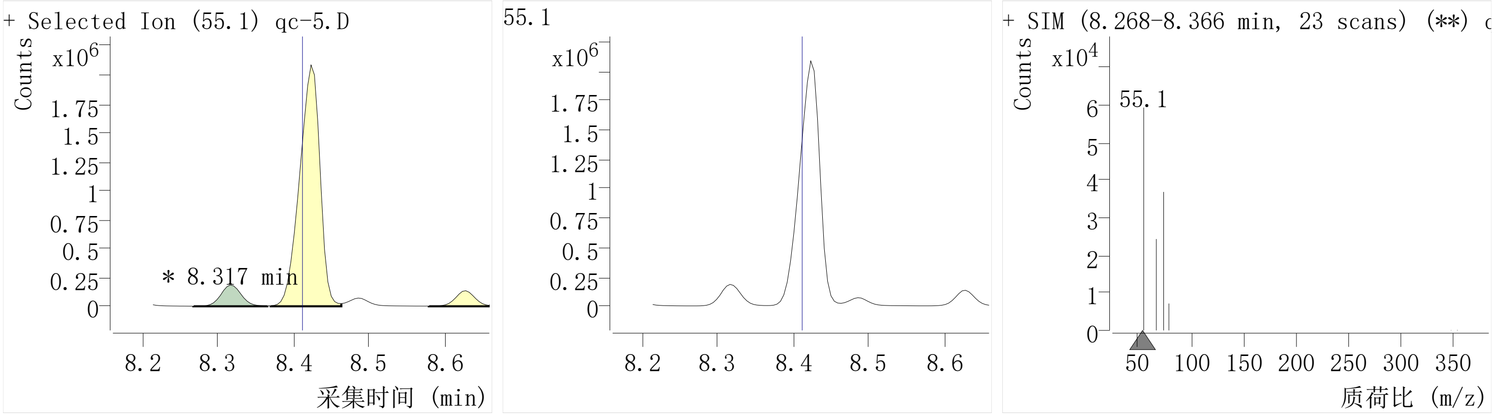

C18:1n9c

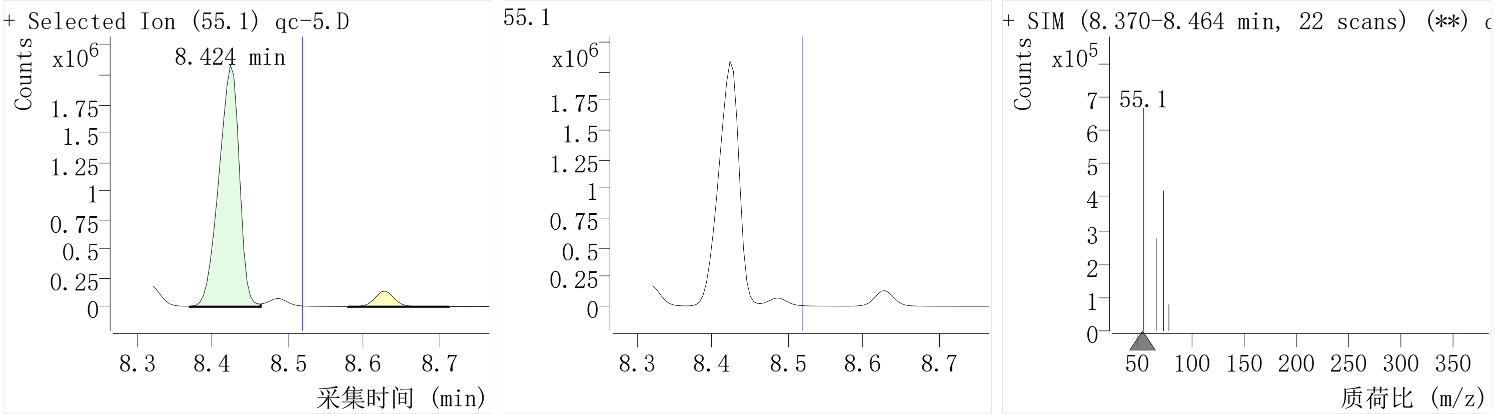

C18:2n6t

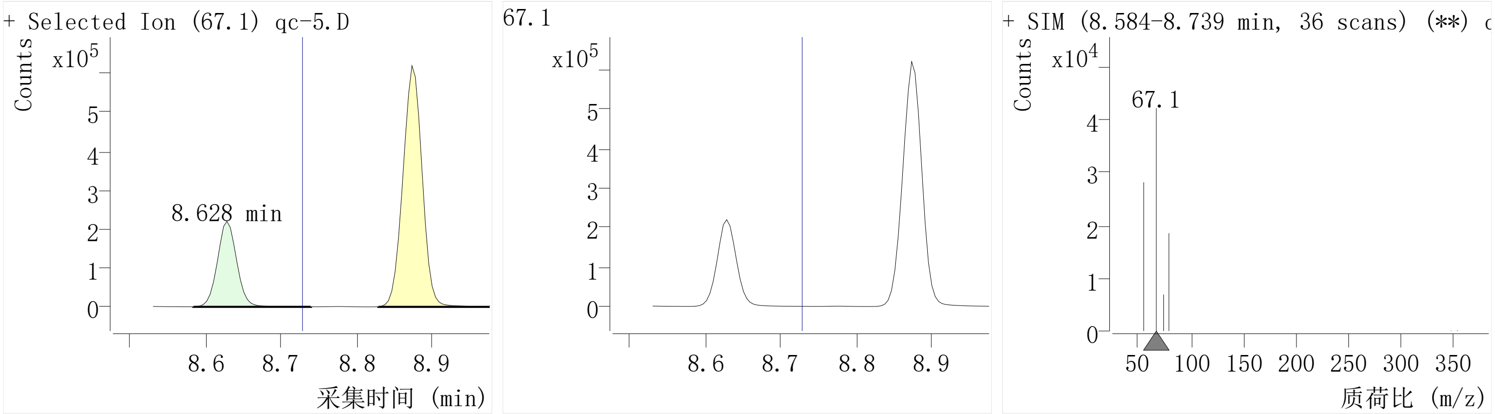

C18:2n6c

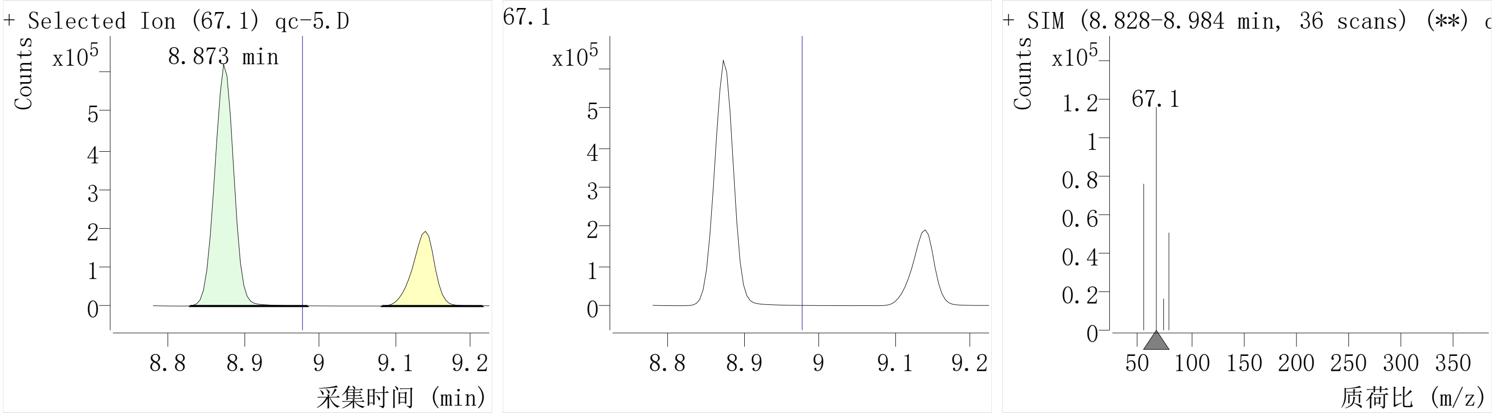

C18:3n6

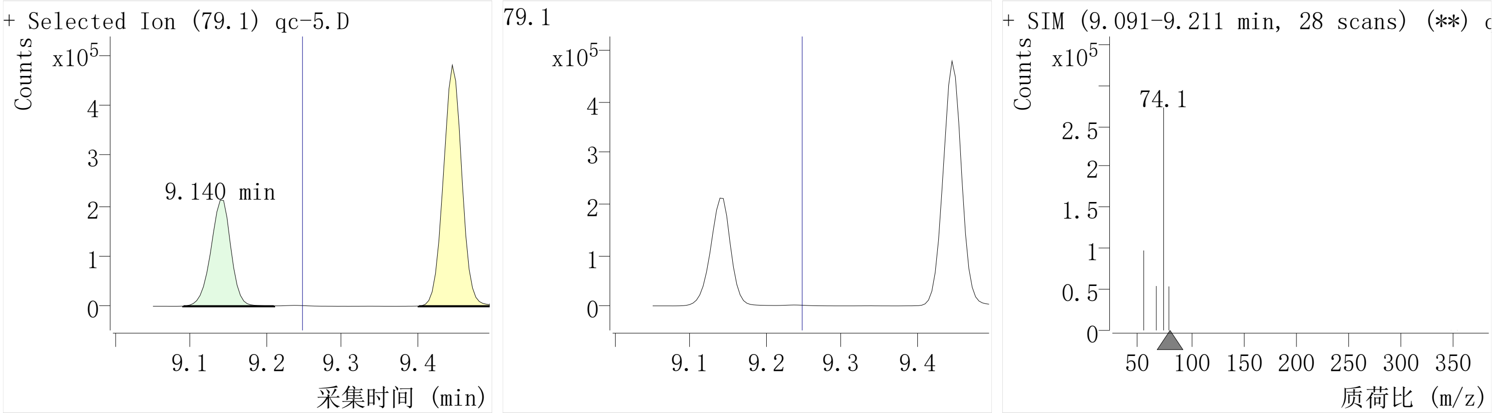

C18:3n3

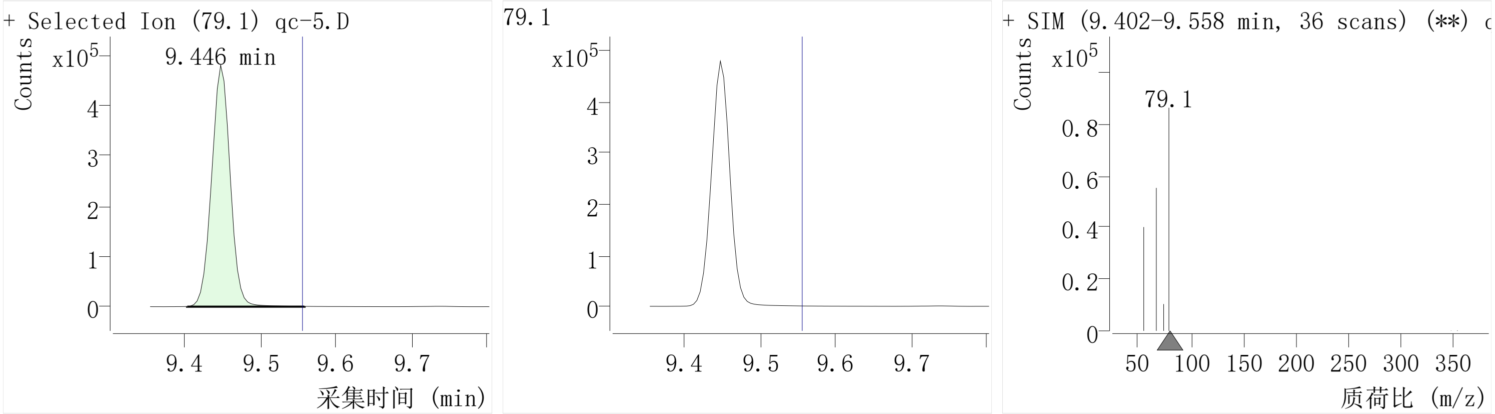

C20:0

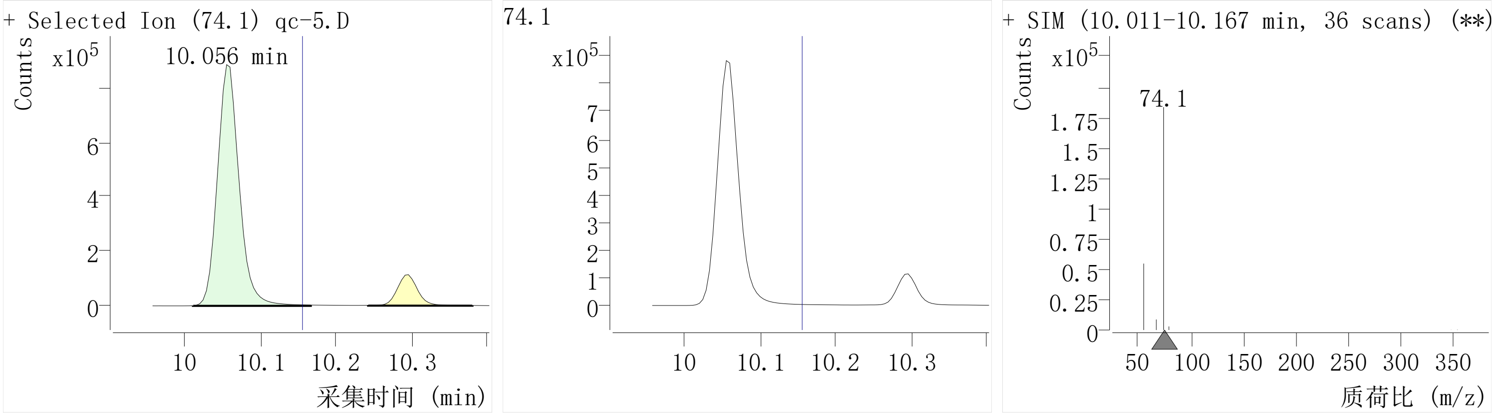

C20:1

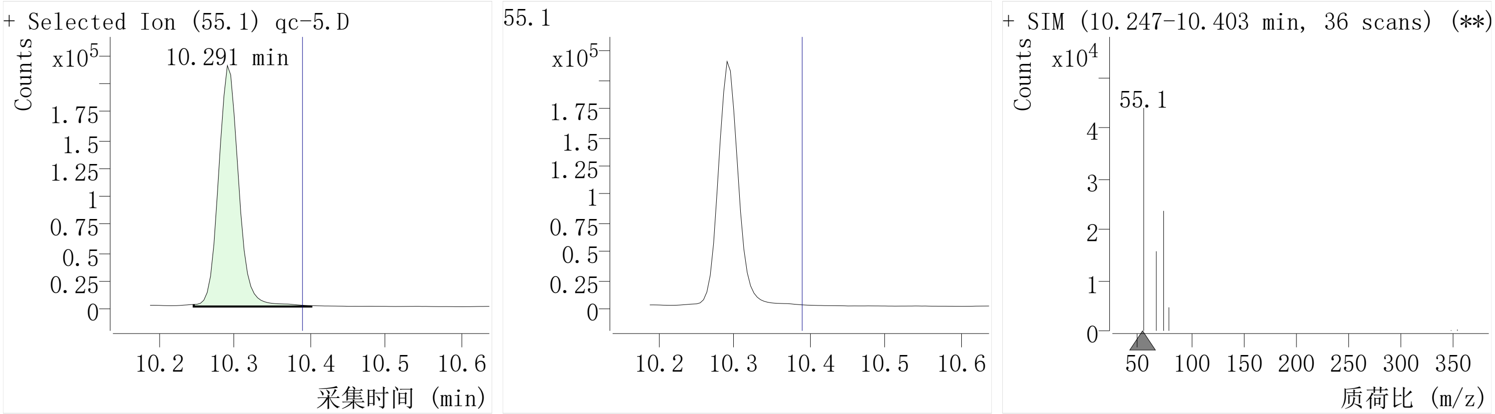

C20:2

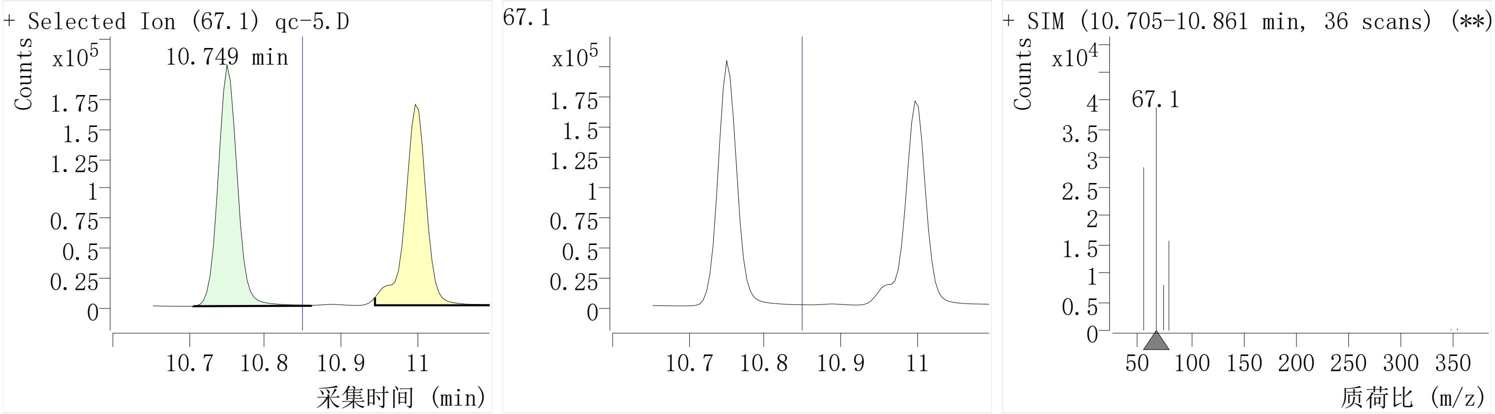

C21:0

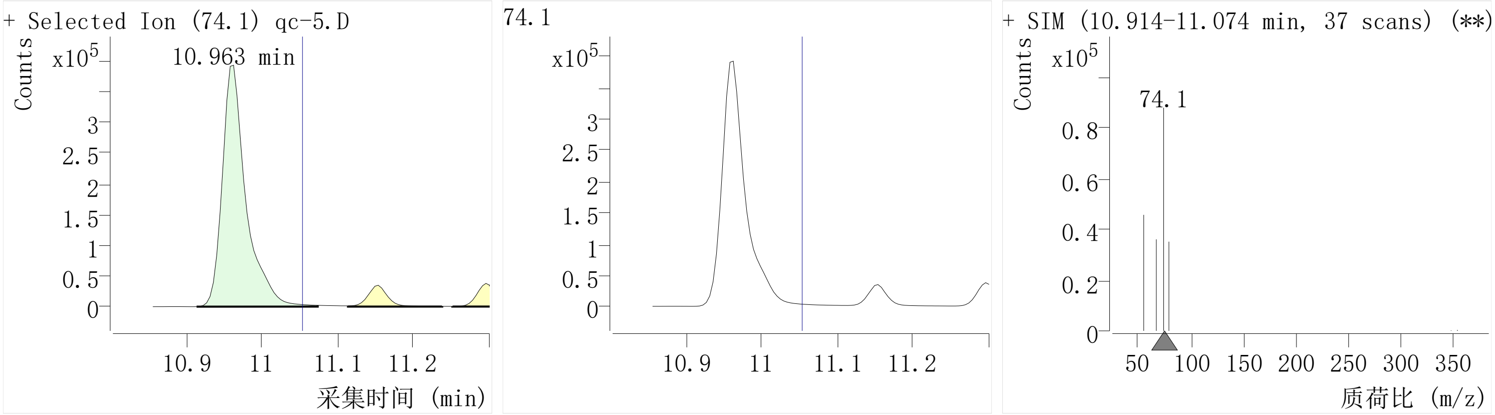

C20:3n6

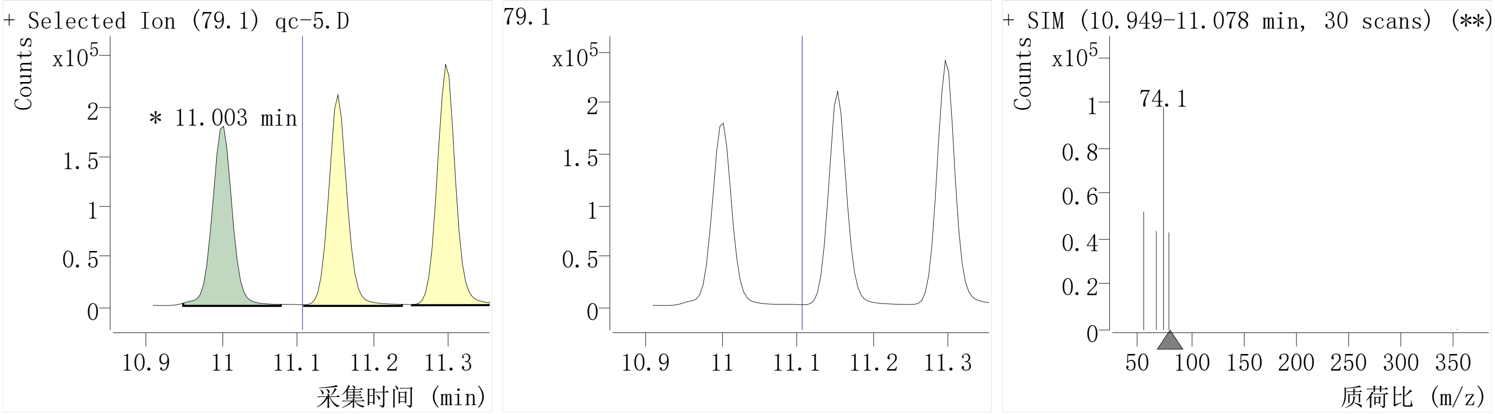

C20:4n6

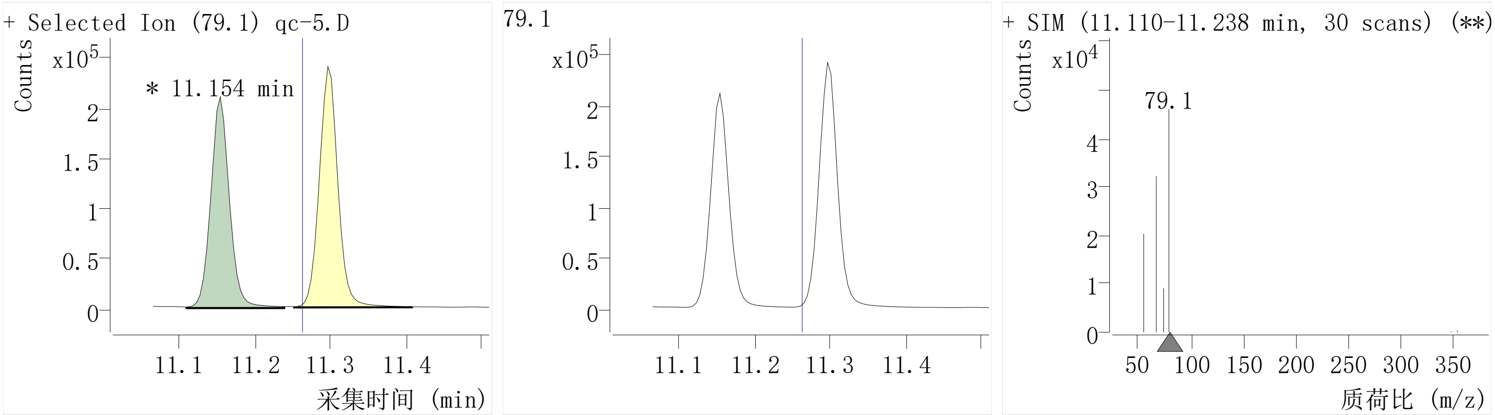

C20:3n3

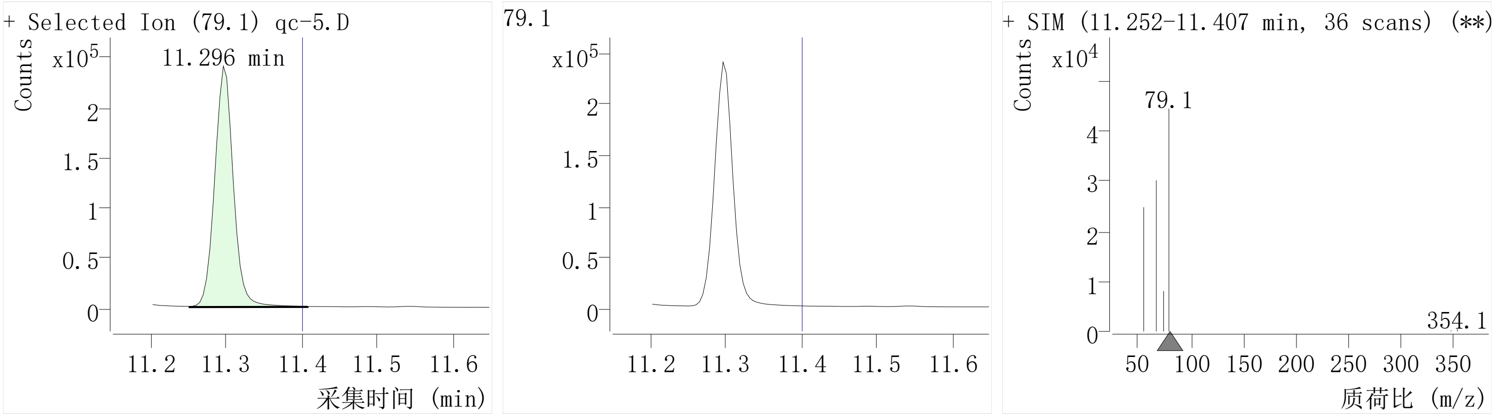

C20:5n3

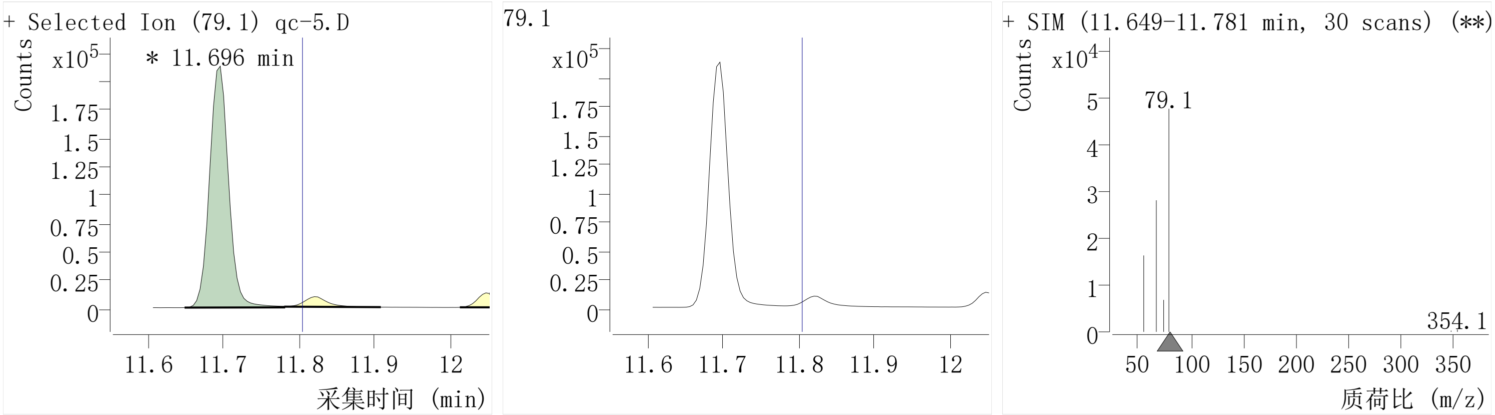

C22:0

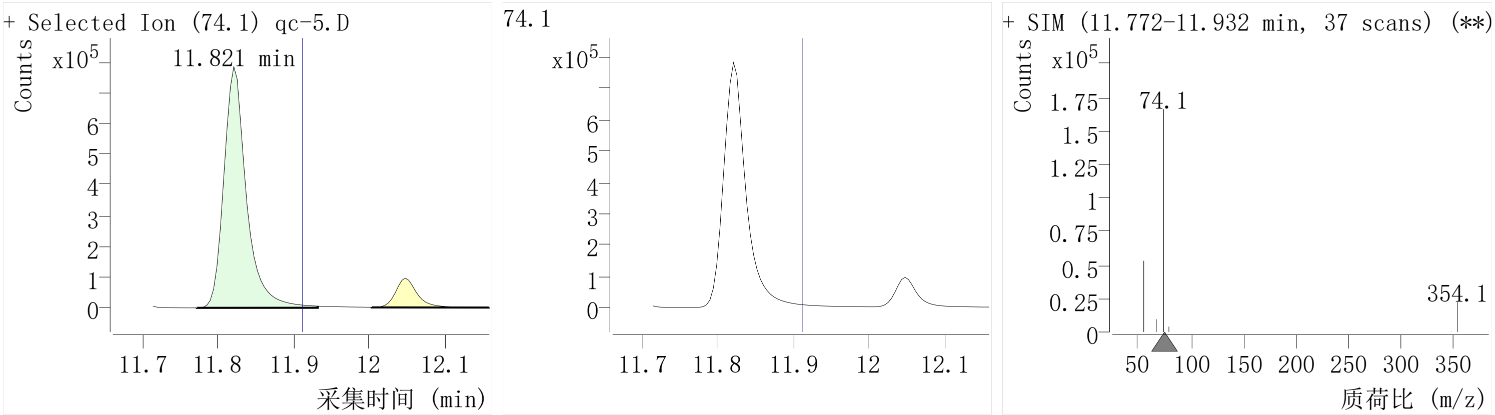

C22:1n9

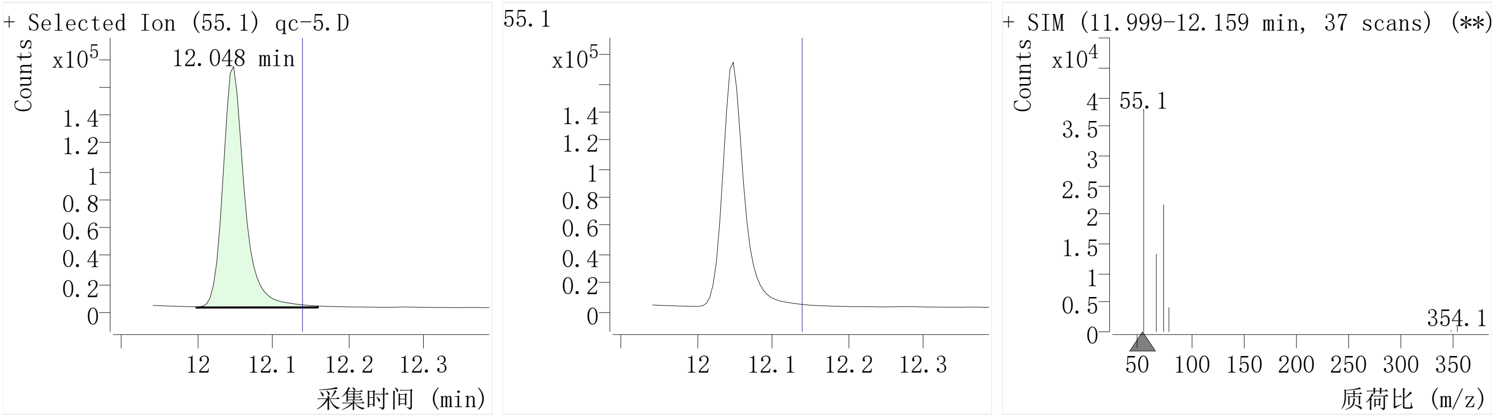

C22:2n6

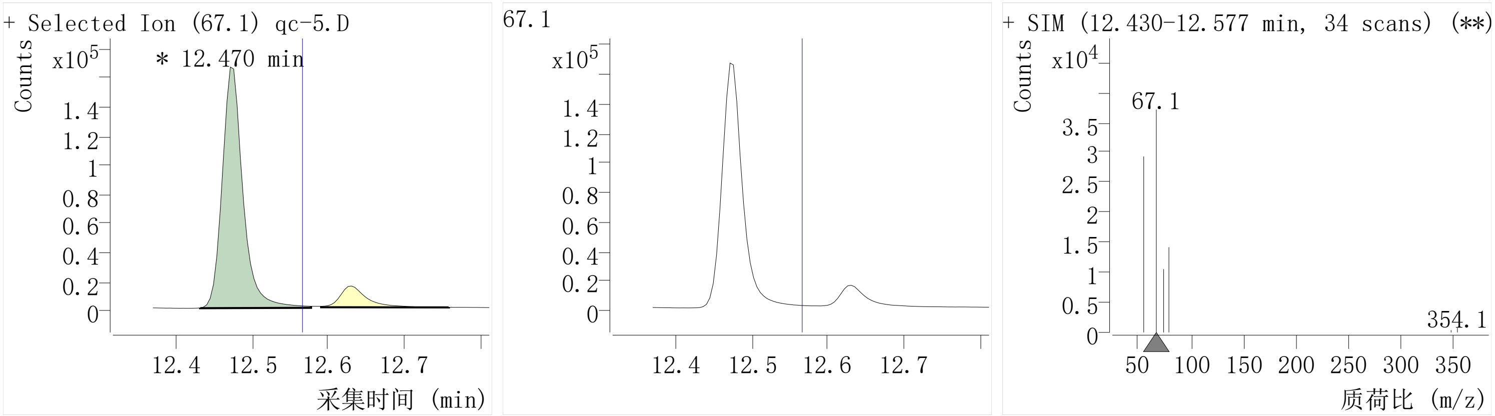

C23:0

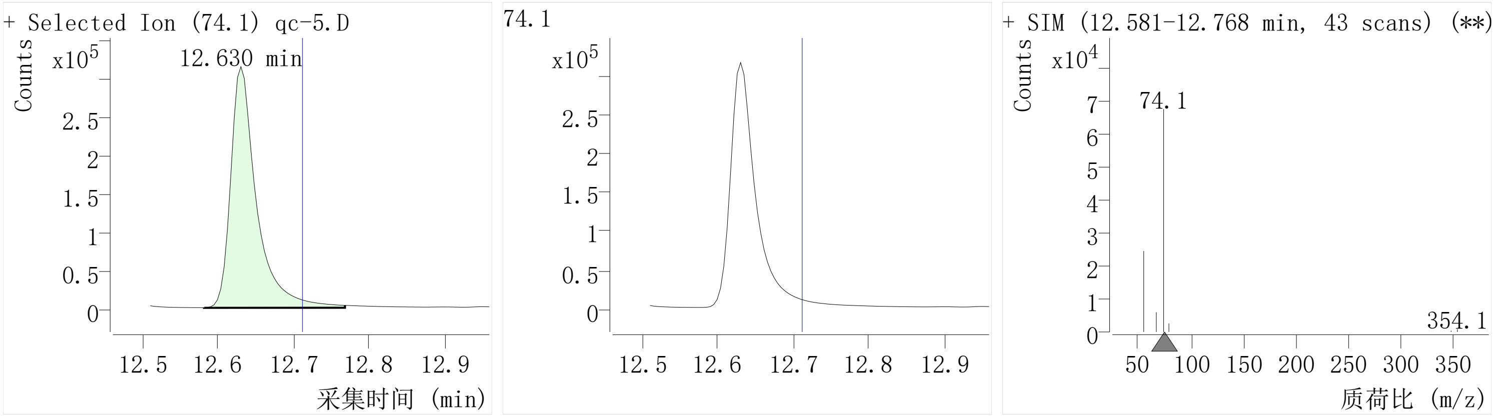

C24:0

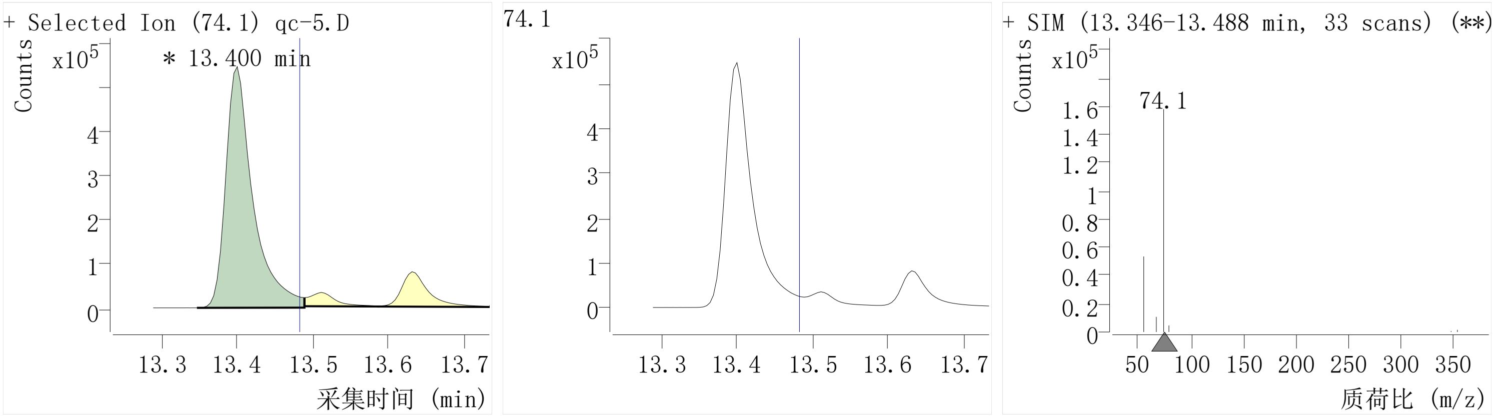

C22:6

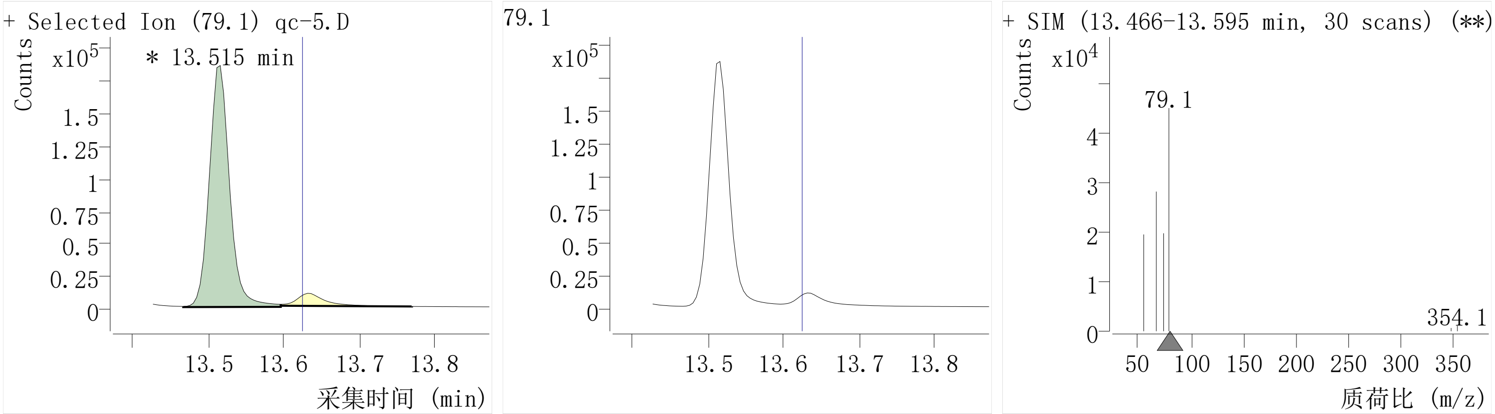
+ Selected Ion (79.1) qc-5.D
Counts
x10^5
* 13.515 min
采集时间 (min)
79.1
+ SIM (13.466-13.595 min, 30 scans) (**)
x10^4
79.1
354.1
质荷比 (m/z)

C24:1

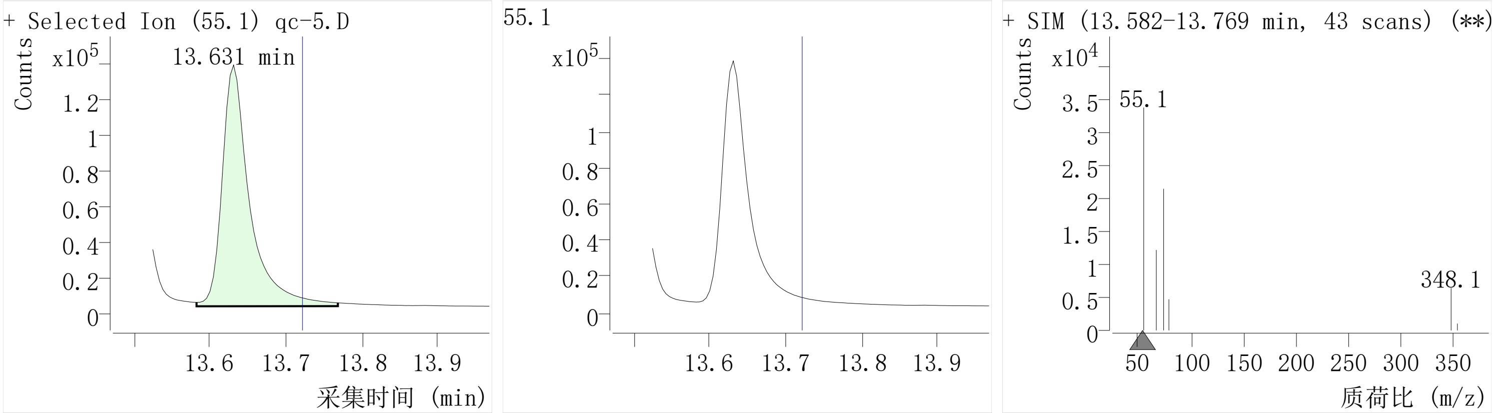
+ Selected Ion (55.1) qc-5.D
Counts
x10^5
13.631 min
采集时间 (min)
55.1
+ SIM (13.582-13.769 min, 43 scans) (**)
x10^4
55.1
348.1
质荷比 (m/z)
