# Supplementary material for: Biomarkers of Adipose Color: A Multi-Omics Analysis Unravels the Molecular Landscape of White and Yellow Fat in Kazakh Horse
Source: Biology (Basel). 2026 Apr 1;15(7):563. doi: 10.3390/biology15070563 (PMC13072367; doi:10.3390/biology15070563)

Sample Name: "S1" Sample ID: "" File: "20250515-50AA.wiff"
Peak Name: "Gly" Mass(es): "246.080/171.010 Da"
Comment: "" Annotation: ""

```
Sample Index:       12
Sample Type:        Standard
Concentration:      12.5    umol/L
Calculated Conc:    12.8    umol/L
Acq. Date:          2025-05-15
Acq. Time:          17:36:17

Modified:           Yes
Proc. Algorithm: Specify Parameters - MQ III
```

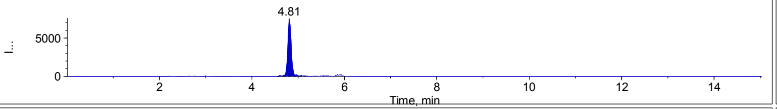

Sample Name: "S1" Sample ID: "" File: "20250515-50AA.wiff"
Peak Name: "Ala" Mass(es): "260.100/171.010 Da"
Comment: "" Annotation: ""

```
Sample Index:       12
Sample Type:        Standard
Concentration:      25.0    umol/L
Calculated Conc:    18.1    umol/L
Acq. Date:          2025-05-15
Acq. Time:          17:36:17

Modified:           Yes
Proc. Algorithm: Specify Parameters - MQ III
```

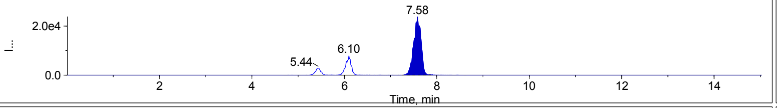

Sample Name: "S1" Sample ID: "" File: "20250515-50AA.wiff"
Peak Name: "Ser" Mass(es): "276.100/171.010 Da"
Comment: "" Annotation: ""

```
Sample Index:       12
Sample Type:        Standard
Concentration:      6.25    umol/L
Calculated Conc:    5.99    umol/L
Acq. Date:          2025-05-15
Acq. Time:          17:36:17

Modified:           Yes
Proc. Algorithm: Specify Parameters - MQ III
```

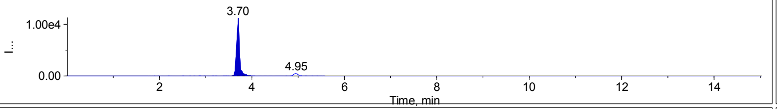

Sample Name: "S1" Sample ID: "" File: "20250515-50AA.wiff"
Peak Name: "Pro" Mass(es): "286.150/171.010 Da"
Comment: "" Annotation: ""

```
Sample Index:       12
Sample Type:        Standard
Concentration:      12.5    umol/L
Calculated Conc:    8.49    umol/L
Acq. Date:          2025-05-15
Acq. Time:          17:36:17

Modified:           Yes
Proc. Algorithm: Specify Parameters - MQ III
```

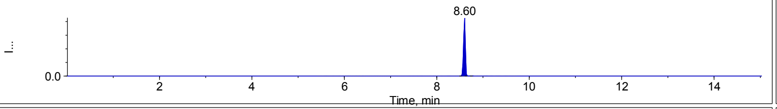

Sample Name: "S1" Sample ID: "" File: "20250515-50AA.wiff"
Peak Name: "Val" Mass(es): "288.150/171.010 Da"
Comment: "" Annotation: ""

```
Sample Index:       12
Sample Type:        Standard
Concentration:      12.5    umol/L
Calculated Conc:    19.6    umol/L
Acq. Date:          2025-05-15
Acq. Time:          17:36:17

Modified:           Yes
Proc. Algorithm: Specify Parameters - MQ III
```

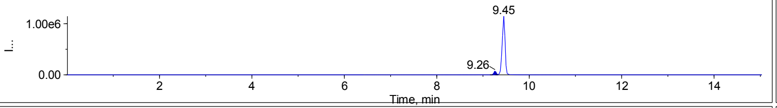

Sample Name: "S1" Sample ID: "" File: "20250515-50AA.wiff"
Peak Name: "Thr" Mass(es): "290.100/171.010 Da"
Comment: "" Annotation: ""

```
Sample Index:       12
Sample Type:        Standard
Concentration:      6.25    umol/L
Calculated Conc:    6.51    umol/L
Acq. Date:          2025-05-15
Acq. Time:          17:36:17

Modified:           Yes
Proc. Algorithm: Specify Parameters - MQ III
```

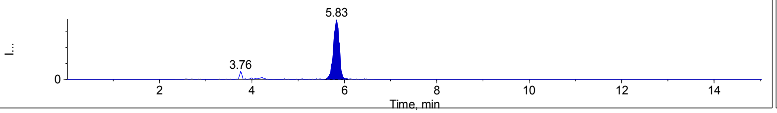

Sample Name: "S1" Sample ID: "" File: "20250515-50AA.wiff"
Peak Name: "MSLAB-Nvl(IS)" Mass(es): "288.150/171.010 Da"
Comment: "" Annotation: ""

```
Sample Index:       12
Sample Type:        Standard
Concentration:      1.00    ng/mL
Calculated Conc:    N/A
Acq. Date:          2025-05-15
Acq. Time:          17:36:17

Modified:           Yes
Proc. Algorithm: Specify Parameters - MQ III
```

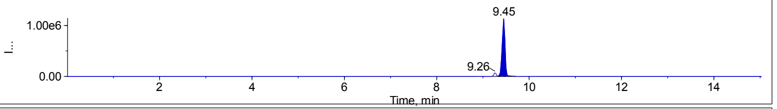

Sample Name: "S1" Sample ID: "" File: "20250515-50AA.wiff"
Peak Name: "MSLAB-Nvl(IS)" Mass(es): "288.150/171.010 Da"
Comment: "" Annotation: ""

Sample Index: 12
Sample Type: Standard
Concentration: 1.00 ng/mL
Calculated Conc: N/A
Acq. Date: 2025-05-15
Acq. Time: 17:36:17

Modified: Yes
Proc. Algorithm: Specify Parameters - MQ III

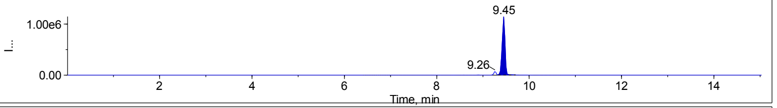

Sample Name: "S1" Sample ID: "" File: "20250515-50AA.wiff"
Peak Name: "MSLAB-Nvl(IS)" Mass(es): "288.150/171.010 Da"
Comment: "" Annotation: ""

Sample Index: 12
Sample Type: Standard
Concentration: 1.00 ng/mL
Calculated Conc: N/A
Acq. Date: 2025-05-15
Acq. Time: 17:36:17

Modified: Yes
Proc. Algorithm: Specify Parameters - MQ III

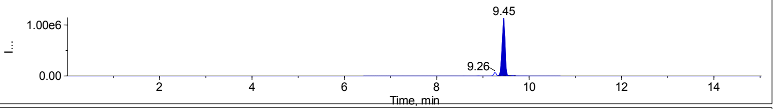

Sample Name: "S1" Sample ID: "" File: "20250515-50AA.wiff"
Peak Name: "MSLAB-Nvl(IS)" Mass(es): "288.150/171.010 Da"
Comment: "" Annotation: ""

Sample Index: 12
Sample Type: Standard
Concentration: 1.00 ng/mL
Calculated Conc: N/A
Acq. Date: 2025-05-15
Acq. Time: 17:36:17

Modified: Yes
Proc. Algorithm: Specify Parameters - MQ III

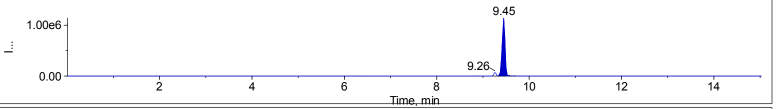

Sample Name: "S1" Sample ID: "" File: "20250515-50AA.wiff"
Peak Name: "MSLAB-Nvl(IS)" Mass(es): "288.150/171.010 Da"
Comment: "" Annotation: ""

Sample Index: 12
Sample Type: Standard
Concentration: 1.00 ng/mL
Calculated Conc: N/A
Acq. Date: 2025-05-15
Acq. Time: 17:36:17

Modified: Yes
Proc. Algorithm: Specify Parameters - MQ III

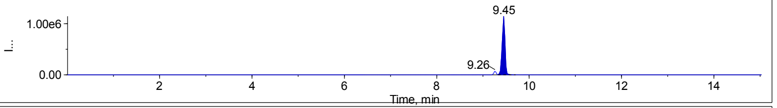

Sample Name: "S1" Sample ID: "" File: "20250515-50AA.wiff"
Peak Name: "MSLAB-Nvl(IS)" Mass(es): "288.150/171.010 Da"
Comment: "" Annotation: ""

Sample Index: 12
Sample Type: Standard
Concentration: 1.00 ng/mL
Calculated Conc: N/A
Acq. Date: 2025-05-15
Acq. Time: 17:36:17

Modified: Yes
Proc. Algorithm: Specify Parameters - MQ III

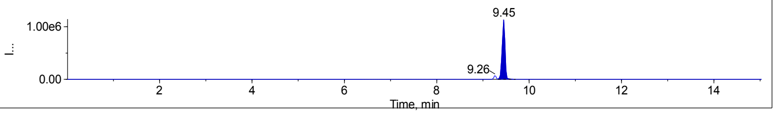

Sample Name: "S1" Sample ID: "" File: "20250515-50AA.wiff"
Peak Name: "Cys1/2" Mass(es): "292.200/171.010 Da"
Comment: "" Annotation: ""

```
Sample Index:       12
Sample Type:      Standard
Concentration:      3.13    umol/L
Calculated Conc:    3.38    umol/L
Acq. Date:        2025-05-15
Acq. Time:        17:36:17

Modified:           Yes
Proc. Algorithm: Specify Parameters - MQ III
```

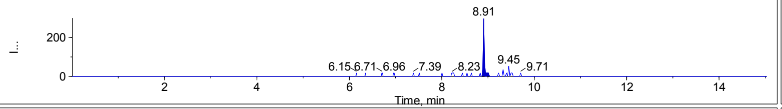

Sample Name: "S1" Sample ID: "" File: "20250515-50AA.wiff"
Peak Name: "MSLAB-Nvl(IS)" Mass(es): "288.150/171.010 Da"
Comment: "" Annotation: ""

```
Sample Index:       12
Sample Type:      Standard
Concentration:      1.00    ng/mL
Calculated Conc:    N/A
Acq. Date:        2025-05-15
Acq. Time:        17:36:17

Modified:           Yes
Proc. Algorithm: Specify Parameters - MQ III
```

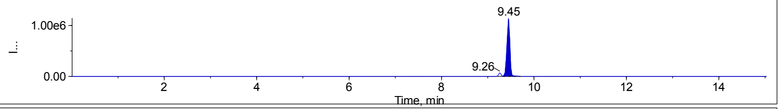

Sample Name: "S1" Sample ID: "" File: "20250515-50AA.wiff"
Peak Name: "Ile" Mass(es): "302.150/171.010 Da"
Comment: "" Annotation: ""

```
Sample Index:       12
Sample Type:      Standard
Concentration:      3.13    umol/L
Calculated Conc:    3.06    umol/L
Acq. Date:        2025-05-15
Acq. Time:        17:36:17

Modified:           Yes
Proc. Algorithm: Specify Parameters - MQ III
```

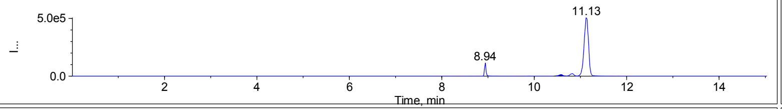

Sample Name: "S1" Sample ID: "" File: "20250515-50AA.wiff"
Peak Name: "MSLAB-Nvl(IS)" Mass(es): "288.150/171.010 Da"
Comment: "" Annotation: ""

```
Sample Index:       12
Sample Type:      Standard
Concentration:      1.00    ng/mL
Calculated Conc:    N/A
Acq. Date:        2025-05-15
Acq. Time:        17:36:17

Modified:           Yes
Proc. Algorithm: Specify Parameters - MQ III
```

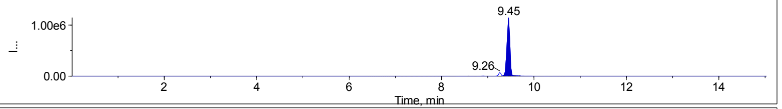

Sample Name: "S1" Sample ID: "" File: "20250515-50AA.wiff"
Peak Name: "Asn" Mass(es): "303.130/171.010 Da"
Comment: "" Annotation: ""

```
Sample Index:       12
Sample Type:      Standard
Concentration:      3.13    umol/L
Calculated Conc:    2.98    umol/L
Acq. Date:        2025-05-15
Acq. Time:        17:36:17

Modified:           Yes
Proc. Algorithm: Specify Parameters - MQ III
```

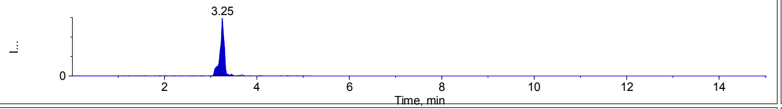

Sample Name: "S1" Sample ID: "" File: "20250515-50AA.wiff"
Peak Name: "MSLAB-Nvl(IS)" Mass(es): "288.150/171.010 Da"
Comment: "" Annotation: ""

```
Sample Index:       12
Sample Type:      Standard
Concentration:      1.00    ng/mL
Calculated Conc:    N/A
Acq. Date:        2025-05-15
Acq. Time:        17:36:17

Modified:           Yes
Proc. Algorithm: Specify Parameters - MQ III
```

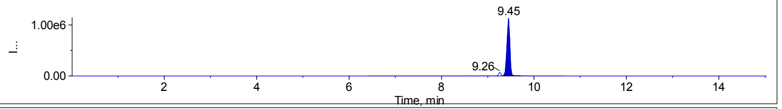

Sample Name: "S1" Sample ID: "" File: "20250515-50AA.wiff"
Peak Name: "Asp" Mass(es): "304.110/171.010 Da"
Comment: "" Annotation: ""

```
Sample Index:       12
Sample Type:      Standard
Concentration:      3.13    umol/L
Calculated Conc:    2.01    umol/L
Acq. Date:        2025-05-15
Acq. Time:        17:36:17

Modified:           Yes
Proc. Algorithm: Specify Parameters - MQ III
```

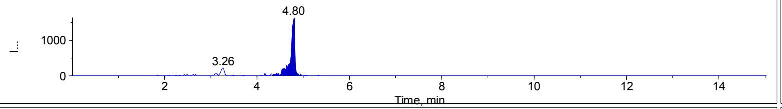

Sample Name: "S1" Sample ID: "" File: "20250515-50AA.wiff"
Peak Name: "MSLAB-Nvl(IS)" Mass(es): "288.150/171.010 Da"
Comment: "" Annotation: ""

```
Sample Index:       12
Sample Type:      Standard
Concentration:      1.00    ng/mL
Calculated Conc:    N/A
Acq. Date:        2025-05-15
Acq. Time:        17:36:17

Modified:           Yes
Proc. Algorithm: Specify Parameters - MQ III
```

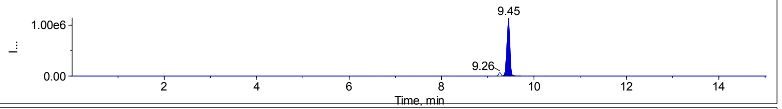

Sample Name: "S1" Sample ID: "" File: "20250515-50AA.wiff"
Peak Name: "Gln" Mass(es): "317.150/171.010 Da"
Comment: "" Annotation: ""

```
Sample Index:       12
Sample Type:      Standard
Concentration:      25.0    umol/L
Calculated Conc:    23.2    umol/L
Acq. Date:        2025-05-15
Acq. Time:        17:36:17

Modified:           Yes
Proc. Algorithm: Specify Parameters - MQ III
```

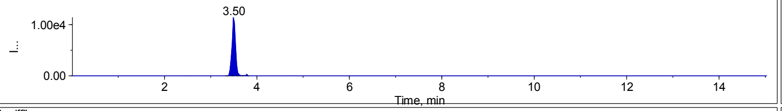

Sample Name: "S1" Sample ID: "" File: "20250515-50AA.wiff"
Peak Name: "MSLAB-Nvl(IS)" Mass(es): "288.150/171.010 Da"
Comment: "" Annotation: ""

```
Sample Index:       12
Sample Type:      Standard
Concentration:      1.00    ng/mL
Calculated Conc:    N/A
Acq. Date:        2025-05-15
Acq. Time:        17:36:17

Modified:           Yes
Proc. Algorithm: Specify Parameters - MQ III
```

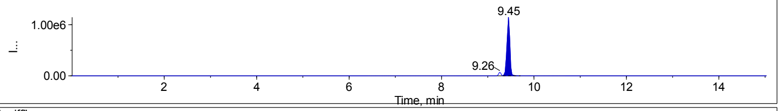

Sample Name: "S1" Sample ID: "" File: "20250515-50AA.wiff"
Peak Name: "Glu" Mass(es): "318.100/171.010 Da"
Comment: "" Annotation: ""

```
Sample Index:       12
Sample Type:      Standard
Concentration:      3.13    umol/L
Calculated Conc:    3.36    umol/L
Acq. Date:        2025-05-15
Acq. Time:        17:36:17

Modified:           Yes
Proc. Algorithm: Specify Parameters - MQ III
```

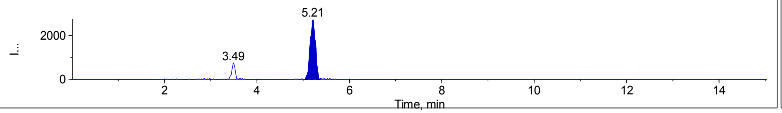

Sample Name: "S1" Sample ID: "" File: "20250515-50AA.wiff"
Peak Name: "MSLAB-Nvl(IS)" Mass(es): "288.150/171.010 Da"
Comment: "" Annotation: ""

```
Sample Index:       12
Sample Type:      Standard
Concentration:      1.00    ng/mL
Calculated Conc:    N/A
Acq. Date:        2025-05-15
Acq. Time:        17:36:17

Modified:           Yes
Proc. Algorithm: Specify Parameters - MQ III
```

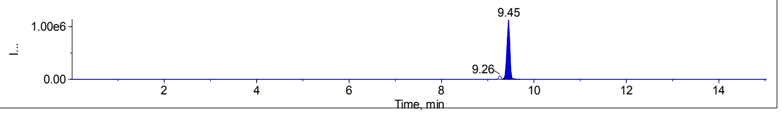

Sample Name: "S1" Sample ID: "" File: "20250515-50AA.wiff"
Peak Name: "Met" Mass(es): "320.210/171.010 Da"
Comment: "" Annotation: ""

```
Sample Index:       12
Sample Type:        Standard
Concentration:      3.13    umol/L
Calculated Conc:    2.85    umol/L
Acq. Date:          2025-05-15
Acq. Time:          17:36:17

Modified:           Yes
Proc. Algorithm: Specify Parameters - MQ III
```

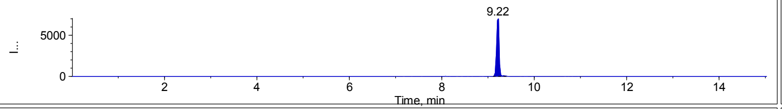

Sample Name: "S1" Sample ID: "" File: "20250515-50AA.wiff"
Peak Name: "His" Mass(es): "326.150/171.010 Da"
Comment: "" Annotation: ""

```
Sample Index:       12
Sample Type:        Standard
Concentration:      3.13    umol/L
Calculated Conc:    3.79    umol/L
Acq. Date:          2025-05-15
Acq. Time:          17:36:17

Modified:           Yes
Proc. Algorithm: Specify Parameters - MQ III
```

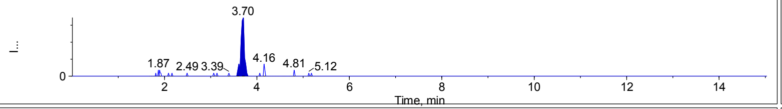

Sample Name: "S1" Sample ID: "" File: "20250515-50AA.wiff"
Peak Name: "Phe" Mass(es): "336.200/171.010 Da"
Comment: "" Annotation: ""

```
Sample Index:       12
Sample Type:        Standard
Concentration:      3.13    umol/L
Calculated Conc:    2.95    umol/L
Acq. Date:          2025-05-15
Acq. Time:          17:36:17

Modified:           Yes
Proc. Algorithm: Specify Parameters - MQ III
```

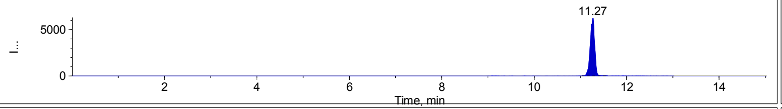

Sample Name: "S1" Sample ID: "" File: "20250515-50AA.wiff"
Peak Name: "Arg" Mass(es): "345.210/171.010 Da"
Comment: "" Annotation: ""

```
Sample Index:       12
Sample Type:        Standard
Concentration:      6.25    umol/L
Calculated Conc:    6.80    umol/L
Acq. Date:          2025-05-15
Acq. Time:          17:36:17

Modified:           Yes
Proc. Algorithm: Specify Parameters - MQ III
```

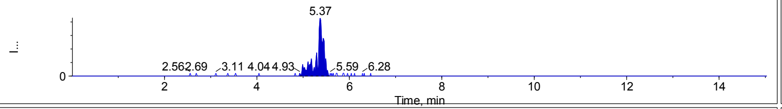

Sample Name: "S1" Sample ID: "" File: "20250515-50AA.wiff"
Peak Name: "Trp" Mass(es): "375.200/171.010 Da"
Comment: "" Annotation: ""

```
Sample Index:       12
Sample Type:        Standard
Concentration:      3.13    umol/L
Calculated Conc:    2.80    umol/L
Acq. Date:          2025-05-15
Acq. Time:          17:36:17

Modified:           Yes
Proc. Algorithm: Specify Parameters - MQ III
```

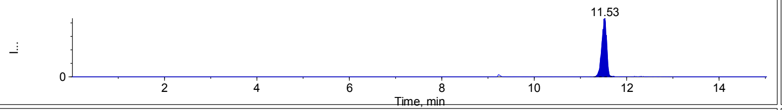

Sample Name: "S1" Sample ID: "" File: "20250515-50AA.wiff"
Peak Name: "Lys" Mass(es): "487.200/171.010 Da"
Comment: "" Annotation: ""

```
Sample Index:       12
Sample Type:        Standard
Concentration:      6.25    umol/L
Calculated Conc:    6.40    umol/L
Acq. Date:          2025-05-15
Acq. Time:          17:36:17

Modified:           Yes
Proc. Algorithm: Specify Parameters - MQ III
```

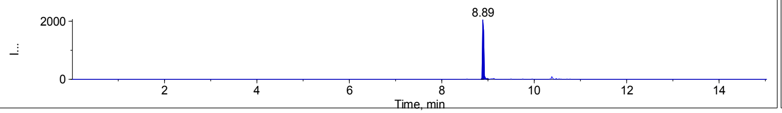

Sample Name: "S1" Sample ID: "" File: "20250515-50AA.wiff"
Peak Name: "MSLAB-Nvl(IS)" Mass(es): "288.150/171.010 Da"
Comment: "" Annotation: ""

```
Sample Index:       12
Sample Type:        Standard
Concentration:      1.00    ng/mL
Calculated Conc:    N/A
Acq. Date:          2025-05-15
Acq. Time:          17:36:17

Modified:           Yes
Proc. Algorithm: Specify Parameters - MQ III
```

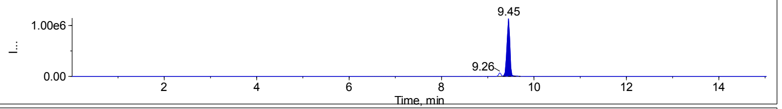

Sample Name: "S1" Sample ID: "" File: "20250515-50AA.wiff"
Peak Name: "MSLAB-Nvl(IS)" Mass(es): "288.150/171.010 Da"
Comment: "" Annotation: ""

```
Sample Index:       12
Sample Type:        Standard
Concentration:      1.00    ng/mL
Calculated Conc:    N/A
Acq. Date:          2025-05-15
Acq. Time:          17:36:17

Modified:           Yes
Proc. Algorithm: Specify Parameters - MQ III
```

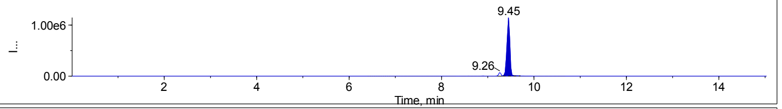

Sample Name: "S1" Sample ID: "" File: "20250515-50AA.wiff"
Peak Name: "MSLAB-Nvl(IS)" Mass(es): "288.150/171.010 Da"
Comment: "" Annotation: ""

```
Sample Index:       12
Sample Type:        Standard
Concentration:      1.00    ng/mL
Calculated Conc:    N/A
Acq. Date:          2025-05-15
Acq. Time:          17:36:17

Modified:           Yes
Proc. Algorithm: Specify Parameters - MQ III
```

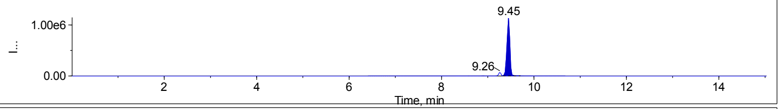

Sample Name: "S1" Sample ID: "" File: "20250515-50AA.wiff"
Peak Name: "MSLAB-Nvl(IS)" Mass(es): "288.150/171.010 Da"
Comment: "" Annotation: ""

```
Sample Index:       12
Sample Type:        Standard
Concentration:      1.00    ng/mL
Calculated Conc:    N/A
Acq. Date:          2025-05-15
Acq. Time:          17:36:17

Modified:           Yes
Proc. Algorithm: Specify Parameters - MQ III
```

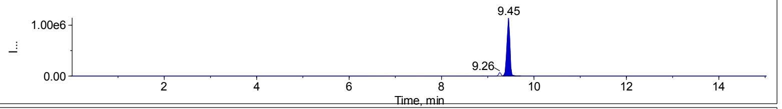

Sample Name: "S1" Sample ID: "" File: "20250515-50AA.wiff"
Peak Name: "MSLAB-Nvl(IS)" Mass(es): "288.150/171.010 Da"
Comment: "" Annotation: ""

```
Sample Index:       12
Sample Type:        Standard
Concentration:      1.00    ng/mL
Calculated Conc:    N/A
Acq. Date:          2025-05-15
Acq. Time:          17:36:17

Modified:           Yes
Proc. Algorithm: Specify Parameters - MQ III
```

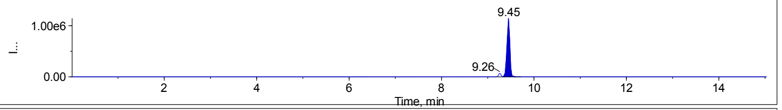

Sample Name: "S1" Sample ID: "" File: "20250515-50AA.wiff"
Peak Name: "MSLAB-Nvl(IS)" Mass(es): "288.150/171.010 Da"
Comment: "" Annotation: ""

```
Sample Index:       12
Sample Type:        Standard
Concentration:      1.00    ng/mL
Calculated Conc:    N/A
Acq. Date:          2025-05-15
Acq. Time:          17:36:17

Modified:           Yes
Proc. Algorithm: Specify Parameters - MQ III
```

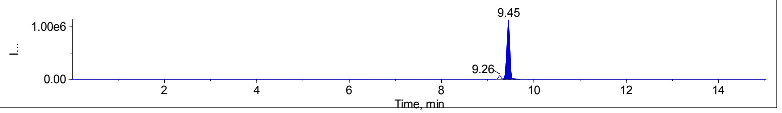

Sample Name: "S1" Sample ID: "" File: "20250515-50AA.wiff"
Peak Name: "tyr" Mass(es): "352.200/171.010 Da"
Comment: "" Annotation: ""

Sample Index: 12
Sample Type: Standard
Concentration: 3.13 umol/L
Calculated Conc: 2.69 umol/L
Acq. Date: 2025-05-15
Acq. Time: 17:36:17

Modified: Yes
Proc. Algorithm: Specify Parameters - MQ III

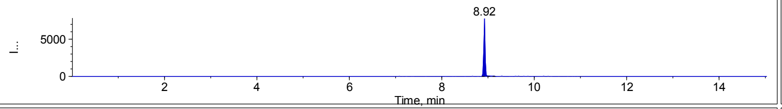

Sample Name: "S1" Sample ID: "" File: "20250515-50AA.wiff"
Peak Name: "Leu" Mass(es): "302.150/171.010 Da"
Comment: "" Annotation: ""

Sample Index: 12
Sample Type: Standard
Concentration: 6.25 umol/L
Calculated Conc: 6.12 umol/L
Acq. Date: 2025-05-15
Acq. Time: 17:36:17

Modified: Yes
Proc. Algorithm: Specify Parameters - MQ III

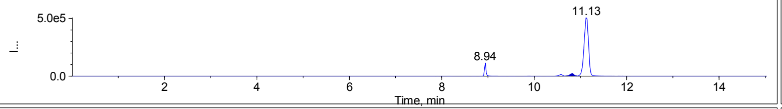

Sample Name: "S2" Sample ID: "" File: "20250515-50AA.wiff"
Peak Name: "Gly" Mass(es): "246.080/171.010 Da"
Comment: "" Annotation: ""

Sample Index: 13
Sample Type: Standard
Concentration: 25.0 umol/L
Calculated Conc: 24.8 umol/L
Acq. Date: 2025-05-15
Acq. Time: 17:51:45

Modified: Yes
Proc. Algorithm: Specify Parameters - MQ III

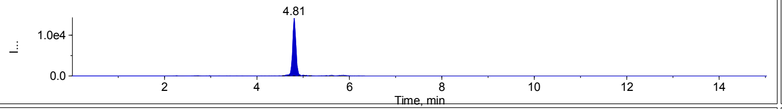

Sample Name: "S2" Sample ID: "" File: "20250515-50AA.wiff"
Peak Name: "Ala" Mass(es): "260.100/171.010 Da"
Comment: "" Annotation: ""

Sample Index: 13
Sample Type: Standard
Concentration: 50.0 umol/L
Calculated Conc: 51.9 umol/L
Acq. Date: 2025-05-15
Acq. Time: 17:51:45

Modified: Yes
Proc. Algorithm: Specify Parameters - MQ III

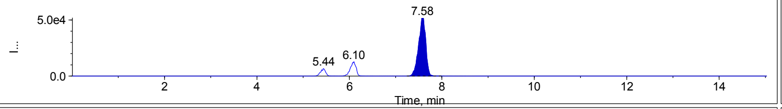

Sample Name: "S2" Sample ID: "" File: "20250515-50AA.wiff"
Peak Name: "Ser" Mass(es): "276.100/171.010 Da"
Comment: "" Annotation: ""

Sample Index: 13
Sample Type: Standard
Concentration: 12.5 umol/L
Calculated Conc: 13.9 umol/L
Acq. Date: 2025-05-15
Acq. Time: 17:51:45

Modified: Yes
Proc. Algorithm: Specify Parameters - MQ III

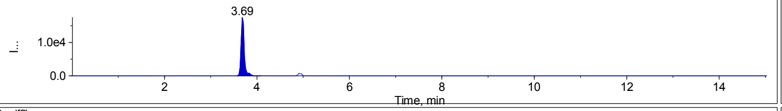

Sample Name: "S2" Sample ID: "" File: "20250515-50AA.wiff"
Peak Name: "Pro" Mass(es): "286.150/171.010 Da"
Comment: "" Annotation: ""

Sample Index: 13
Sample Type: Standard
Concentration: 25.0 umol/L
Calculated Conc: 26.3 umol/L
Acq. Date: 2025-05-15
Acq. Time: 17:51:45

Modified: Yes
Proc. Algorithm: Specify Parameters - MQ III

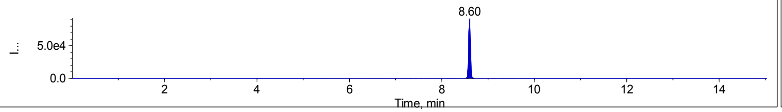

Sample Name: "S1" Sample ID: "" File: "20250515-50AA.wiff"
Peak Name: "MSLAB-Nvl(IS)" Mass(es): "288.150/171.010 Da"
Comment: "" Annotation: ""

Sample Index: 12
Sample Type: Standard
Concentration: 1.00 ng/mL
Calculated Conc: N/A
Acq. Date: 2025-05-15
Acq. Time: 17:36:17

Modified: Yes
Proc. Algorithm: Specify Parameters - MQ III

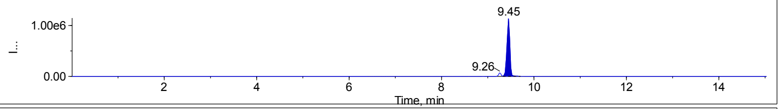

Sample Name: "S1" Sample ID: "" File: "20250515-50AA.wiff"
Peak Name: "MSLAB-Nvl(IS)" Mass(es): "288.150/171.010 Da"
Comment: "" Annotation: ""

Sample Index: 12
Sample Type: Standard
Concentration: 1.00 ng/mL
Calculated Conc: N/A
Acq. Date: 2025-05-15
Acq. Time: 17:36:17

Modified: Yes
Proc. Algorithm: Specify Parameters - MQ III

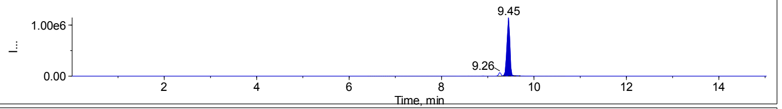

Sample Name: "S2" Sample ID: "" File: "20250515-50AA.wiff"
Peak Name: "MSLAB-Nvl(IS)" Mass(es): "288.150/171.010 Da"
Comment: "" Annotation: ""

Sample Index: 13
Sample Type: Standard
Concentration: 1.00 ng/mL
Calculated Conc: N/A
Acq. Date: 2025-05-15
Acq. Time: 17:51:45

Modified: Yes
Proc. Algorithm: Specify Parameters - MQ III

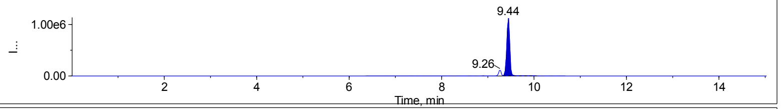

Sample Name: "S2" Sample ID: "" File: "20250515-50AA.wiff"
Peak Name: "MSLAB-Nvl(IS)" Mass(es): "288.150/171.010 Da"
Comment: "" Annotation: ""

Sample Index: 13
Sample Type: Standard
Concentration: 1.00 ng/mL
Calculated Conc: N/A
Acq. Date: 2025-05-15
Acq. Time: 17:51:45

Modified: Yes
Proc. Algorithm: Specify Parameters - MQ III

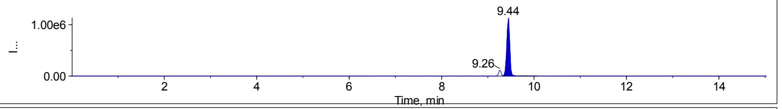

Sample Name: "S2" Sample ID: "" File: "20250515-50AA.wiff"
Peak Name: "MSLAB-Nvl(IS)" Mass(es): "288.150/171.010 Da"
Comment: "" Annotation: ""

Sample Index: 13
Sample Type: Standard
Concentration: 1.00 ng/mL
Calculated Conc: N/A
Acq. Date: 2025-05-15
Acq. Time: 17:51:45

Modified: Yes
Proc. Algorithm: Specify Parameters - MQ III

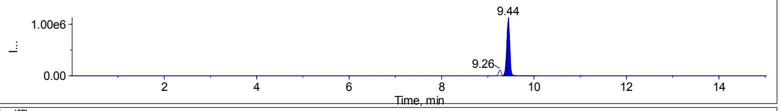

Sample Name: "S2" Sample ID: "" File: "20250515-50AA.wiff"
Peak Name: "MSLAB-Nvl(IS)" Mass(es): "288.150/171.010 Da"
Comment: "" Annotation: ""

Sample Index: 13
Sample Type: Standard
Concentration: 1.00 ng/mL
Calculated Conc: N/A
Acq. Date: 2025-05-15
Acq. Time: 17:51:45

Modified: Yes
Proc. Algorithm: Specify Parameters - MQ III

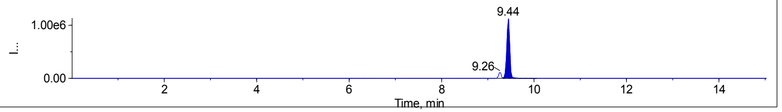

Sample Name: "S2" Sample ID: "" File: "20250515-50AA.wiff"
Peak Name: "Val" Mass(es): "288.150/171.010 Da"
Comment: "" Annotation: ""

Sample Index: 13
Sample Type: Standard
Concentration: 25.0 umol/L
Calculated Conc: 34.1 umol/L
Acq. Date: 2025-05-15
Acq. Time: 17:51:45

Modified: Yes
Proc. Algorithm: Specify Parameters - MQ III

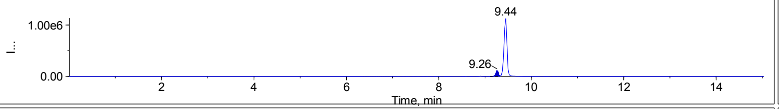

Sample Name: "S2" Sample ID: "" File: "20250515-50AA.wiff"
Peak Name: "Thr" Mass(es): "290.100/171.010 Da"
Comment: "" Annotation: ""

Sample Index: 13
Sample Type: Standard
Concentration: 12.5 umol/L
Calculated Conc: 12.5 umol/L
Acq. Date: 2025-05-15
Acq. Time: 17:51:45

Modified: Yes
Proc. Algorithm: Specify Parameters - MQ III

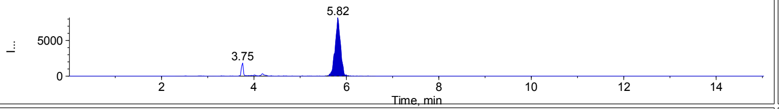

Sample Name: "S2" Sample ID: "" File: "20250515-50AA.wiff"
Peak Name: "Cys1/2" Mass(es): "292.200/171.010 Da"
Comment: "" Annotation: ""

Sample Index: 13
Sample Type: Standard
Concentration: 6.26 umol/L
Calculated Conc: 6.03 umol/L
Acq. Date: 2025-05-15
Acq. Time: 17:51:45

Modified: Yes
Proc. Algorithm: Specify Parameters - MQ III

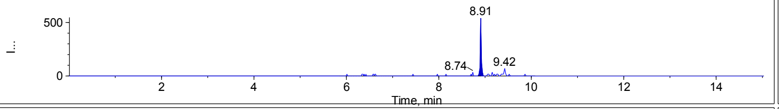

Sample Name: "S2" Sample ID: "" File: "20250515-50AA.wiff"
Peak Name: "Ile" Mass(es): "302.150/171.010 Da"
Comment: "" Annotation: ""

Sample Index: 13
Sample Type: Standard
Concentration: 6.26 umol/L
Calculated Conc: 6.25 umol/L
Acq. Date: 2025-05-15
Acq. Time: 17:51:45

Modified: Yes
Proc. Algorithm: Specify Parameters - MQ III

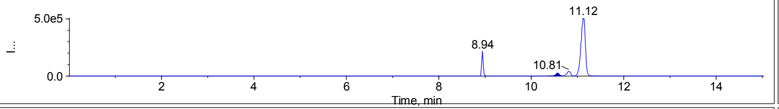

Sample Name: "S2" Sample ID: "" File: "20250515-50AA.wiff"
Peak Name: "Asn" Mass(es): "303.130/171.010 Da"
Comment: "" Annotation: ""

Sample Index: 13
Sample Type: Standard
Concentration: 6.26 umol/L
Calculated Conc: 6.50 umol/L
Acq. Date: 2025-05-15
Acq. Time: 17:51:45

Modified: Yes
Proc. Algorithm: Specify Parameters - MQ III

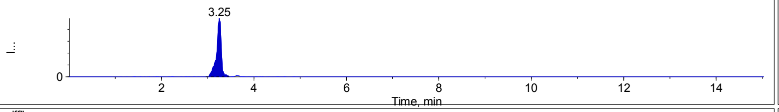

Sample Name: "S2" Sample ID: "" File: "20250515-50AA.wiff"
Peak Name: "Asp" Mass(es): "304.110/171.010 Da"
Comment: "" Annotation: ""

Sample Index: 13
Sample Type: Standard
Concentration: 6.26 umol/L
Calculated Conc: 9.55 umol/L
Acq. Date: 2025-05-15
Acq. Time: 17:51:45

Modified: Yes
Proc. Algorithm: Specify Parameters - MQ III

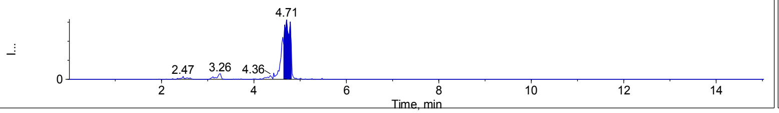

Sample Name: "S2" Sample ID: "" File: "20250515-50AA.wiff"
Peak Name: "MSLAB-Nvl(IS)" Mass(es): "288.150/171.010 Da"
Comment: "" Annotation: ""

Sample Index: 13
Sample Type: Standard
Concentration: 1.00 ng/mL
Calculated Conc: N/A
Acq. Date: 2025-05-15
Acq. Time: 17:51:45

Modified: Yes
Proc. Algorithm: Specify Parameters - MQ III

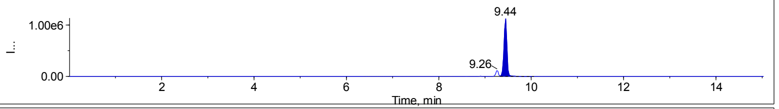

Sample Name: "S2" Sample ID: "" File: "20250515-50AA.wiff"
Peak Name: "MSLAB-Nvl(IS)" Mass(es): "288.150/171.010 Da"
Comment: "" Annotation: ""

Sample Index: 13
Sample Type: Standard
Concentration: 1.00 ng/mL
Calculated Conc: N/A
Acq. Date: 2025-05-15
Acq. Time: 17:51:45

Modified: Yes
Proc. Algorithm: Specify Parameters - MQ III

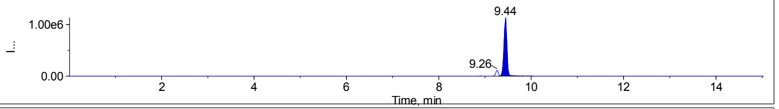

Sample Name: "S2" Sample ID: "" File: "20250515-50AA.wiff"
Peak Name: "MSLAB-Nvl(IS)" Mass(es): "288.150/171.010 Da"
Comment: "" Annotation: ""

Sample Index: 13
Sample Type: Standard
Concentration: 1.00 ng/mL
Calculated Conc: N/A
Acq. Date: 2025-05-15
Acq. Time: 17:51:45

Modified: Yes
Proc. Algorithm: Specify Parameters - MQ III

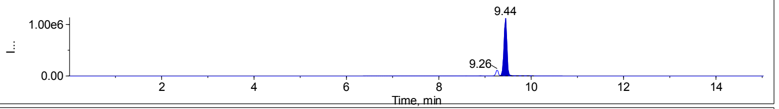

Sample Name: "S2" Sample ID: "" File: "20250515-50AA.wiff"
Peak Name: "MSLAB-Nvl(IS)" Mass(es): "288.150/171.010 Da"
Comment: "" Annotation: ""

Sample Index: 13
Sample Type: Standard
Concentration: 1.00 ng/mL
Calculated Conc: N/A
Acq. Date: 2025-05-15
Acq. Time: 17:51:45

Modified: Yes
Proc. Algorithm: Specify Parameters - MQ III

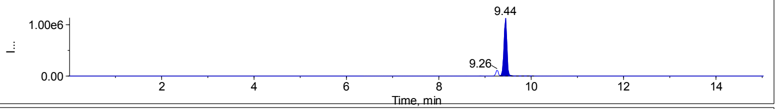

Sample Name: "S2" Sample ID: "" File: "20250515-50AA.wiff"
Peak Name: "MSLAB-Nvl(IS)" Mass(es): "288.150/171.010 Da"
Comment: "" Annotation: ""

Sample Index: 13
Sample Type: Standard
Concentration: 1.00 ng/mL
Calculated Conc: N/A
Acq. Date: 2025-05-15
Acq. Time: 17:51:45

Modified: Yes
Proc. Algorithm: Specify Parameters - MQ III

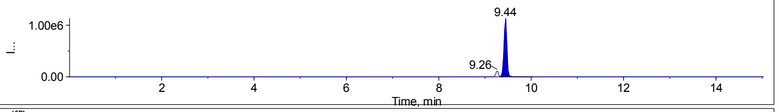

Sample Name: "S2" Sample ID: "" File: "20250515-50AA.wiff"
Peak Name: "MSLAB-Nvl(IS)" Mass(es): "288.150/171.010 Da"
Comment: "" Annotation: ""

Sample Index: 13
Sample Type: Standard
Concentration: 1.00 ng/mL
Calculated Conc: N/A
Acq. Date: 2025-05-15
Acq. Time: 17:51:45

Modified: Yes
Proc. Algorithm: Specify Parameters - MQ III

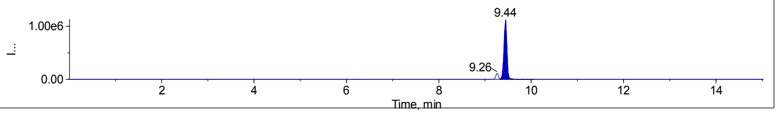

Sample Name: "S2" Sample ID: "" File: "20250515-50AA.wiff"
Peak Name: "Gln" Mass(es): "317.150/171.010 Da"
Comment: "" Annotation: ""
Sample Index: 13
Sample Type: Standard
Concentration: 50.0 umol/L
Calculated Conc: 48.8 umol/L
Acq. Date: 2025-05-15
Acq. Time: 17:51:45
Modified: Yes
Proc. Algorithm: Specify Parameters - MQ III

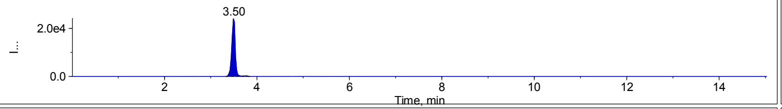

Sample Name: "S2" Sample ID: "" File: "20250515-50AA.wiff"
Peak Name: "Glu" Mass(es): "318.100/171.010 Da"
Comment: "" Annotation: ""
Sample Index: 13
Sample Type: Standard
Concentration: 6.26 umol/L
Calculated Conc: 6.08 umol/L
Acq. Date: 2025-05-15
Acq. Time: 17:51:45
Modified: Yes
Proc. Algorithm: Specify Parameters - MQ III

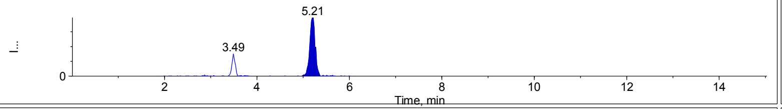

Sample Name: "S2" Sample ID: "" File: "20250515-50AA.wiff"
Peak Name: "Met" Mass(es): "320.210/171.010 Da"
Comment: "" Annotation: ""
Sample Index: 13
Sample Type: Standard
Concentration: 6.26 umol/L
Calculated Conc: 6.19 umol/L
Acq. Date: 2025-05-15
Acq. Time: 17:51:45
Modified: Yes
Proc. Algorithm: Specify Parameters - MQ III

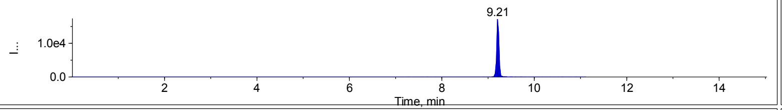

Sample Name: "S2" Sample ID: "" File: "20250515-50AA.wiff"
Peak Name: "His" Mass(es): "326.150/171.010 Da"
Comment: "" Annotation: ""
Sample Index: 13
Sample Type: Standard
Concentration: 6.26 umol/L
Calculated Conc: 5.32 umol/L
Acq. Date: 2025-05-15
Acq. Time: 17:51:45
Modified: Yes
Proc. Algorithm: Specify Parameters - MQ III

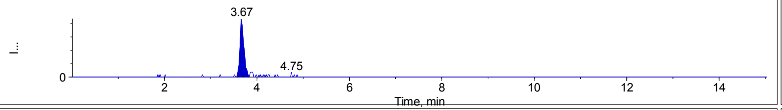

Sample Name: "S2" Sample ID: "" File: "20250515-50AA.wiff"
Peak Name: "Phe" Mass(es): "336.200/171.010 Da"
Comment: "" Annotation: ""
Sample Index: 13
Sample Type: Standard
Concentration: 6.26 umol/L
Calculated Conc: 6.01 umol/L
Acq. Date: 2025-05-15
Acq. Time: 17:51:45
Modified: Yes
Proc. Algorithm: Specify Parameters - MQ III

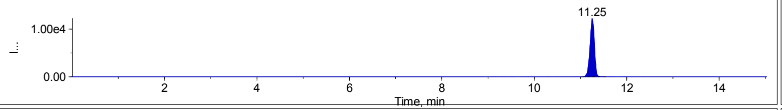

Sample Name: "S2" Sample ID: "" File: "20250515-50AA.wiff"
Peak Name: "Arg" Mass(es): "345.210/171.010 Da"
Comment: "" Annotation: ""
Sample Index: 13
Sample Type: Standard
Concentration: 12.5 umol/L
Calculated Conc: 12.9 umol/L
Acq. Date: 2025-05-15
Acq. Time: 17:51:45
Modified: Yes
Proc. Algorithm: Specify Parameters - MQ III

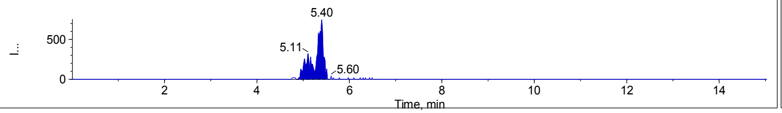

Sample Name: "S2" Sample ID: "" File: "20250515-50AA.wiff"
Peak Name: "MSLAB-Nvl(IS)" Mass(es): "288.150/171.010 Da"
Comment: "" Annotation: ""
Sample Index: 13
Sample Type: Standard
Concentration: 1.00 ng/mL
Calculated Conc: N/A
Acq. Date: 2025-05-15
Acq. Time: 17:51:45
Modified: Yes
Proc. Algorithm: Specify Parameters - MQ III

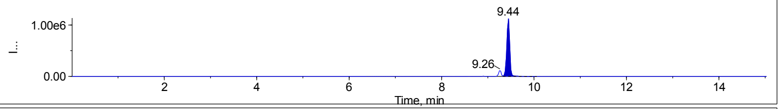

Sample Name: "S2" Sample ID: "" File: "20250515-50AA.wiff"
Peak Name: "MSLAB-Nvl(IS)" Mass(es): "288.150/171.010 Da"
Comment: "" Annotation: ""
Sample Index: 13
Sample Type: Standard
Concentration: 1.00 ng/mL
Calculated Conc: N/A
Acq. Date: 2025-05-15
Acq. Time: 17:51:45
Modified: Yes
Proc. Algorithm: Specify Parameters - MQ III

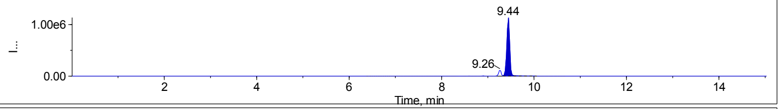

Sample Name: "S2" Sample ID: "" File: "20250515-50AA.wiff"
Peak Name: "MSLAB-Nvl(IS)" Mass(es): "288.150/171.010 Da"
Comment: "" Annotation: ""
Sample Index: 13
Sample Type: Standard
Concentration: 1.00 ng/mL
Calculated Conc: N/A
Acq. Date: 2025-05-15
Acq. Time: 17:51:45
Modified: Yes
Proc. Algorithm: Specify Parameters - MQ III

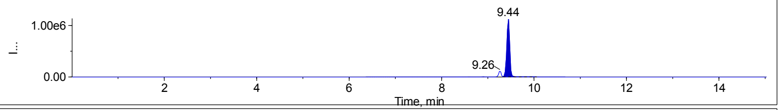

Sample Name: "S2" Sample ID: "" File: "20250515-50AA.wiff"
Peak Name: "MSLAB-Nvl(IS)" Mass(es): "288.150/171.010 Da"
Comment: "" Annotation: ""
Sample Index: 13
Sample Type: Standard
Concentration: 1.00 ng/mL
Calculated Conc: N/A
Acq. Date: 2025-05-15
Acq. Time: 17:51:45
Modified: Yes
Proc. Algorithm: Specify Parameters - MQ III

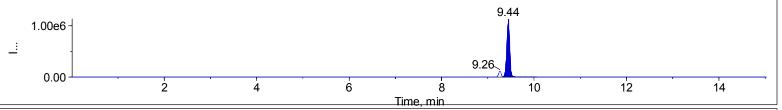

Sample Name: "S2" Sample ID: "" File: "20250515-50AA.wiff"
Peak Name: "MSLAB-Nvl(IS)" Mass(es): "288.150/171.010 Da"
Comment: "" Annotation: ""
Sample Index: 13
Sample Type: Standard
Concentration: 1.00 ng/mL
Calculated Conc: N/A
Acq. Date: 2025-05-15
Acq. Time: 17:51:45
Modified: Yes
Proc. Algorithm: Specify Parameters - MQ III

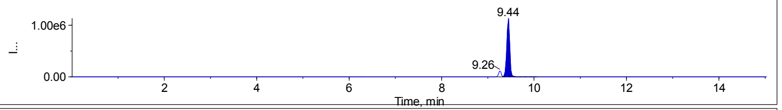

Sample Name: "S2" Sample ID: "" File: "20250515-50AA.wiff"
Peak Name: "MSLAB-Nvl(IS)" Mass(es): "288.150/171.010 Da"
Comment: "" Annotation: ""
Sample Index: 13
Sample Type: Standard
Concentration: 1.00 ng/mL
Calculated Conc: N/A
Acq. Date: 2025-05-15
Acq. Time: 17:51:45
Modified: Yes
Proc. Algorithm: Specify Parameters - MQ III

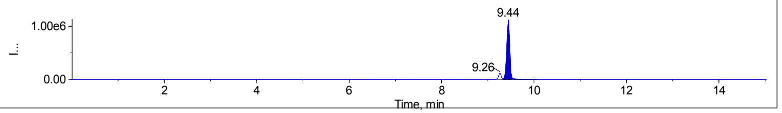

Sample Name: "S2" Sample ID: "" File: "20250515-50AA.wiff"
Peak Name: "Trp" Mass(es): "375.200/171.010 Da"
Comment: "" Annotation: ""
Sample Index: 13
Sample Type: Standard
Concentration: 6.26 umol/L
Calculated Conc: 5.82 umol/L
Acq. Date: 2025-05-15
Acq. Time: 17:51:45
Modified: Yes
Proc. Algorithm: Specify Parameters - MQ III

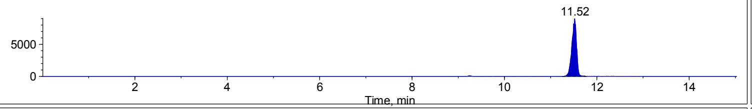

Sample Name: "S2" Sample ID: "" File: "20250515-50AA.wiff"
Peak Name: "MSLAB-Nvl(IS)" Mass(es): "288.150/171.010 Da"
Comment: "" Annotation: ""
Sample Index: 13
Sample Type: Standard
Concentration: 1.00 ng/mL
Calculated Conc: N/A
Acq. Date: 2025-05-15
Acq. Time: 17:51:45
Modified: Yes
Proc. Algorithm: Specify Parameters - MQ III

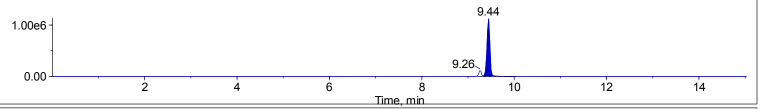

Sample Name: "S2" Sample ID: "" File: "20250515-50AA.wiff"
Peak Name: "Lys" Mass(es): "487.200/171.010 Da"
Comment: "" Annotation: ""
Sample Index: 13
Sample Type: Standard
Concentration: 12.5 umol/L
Calculated Conc: 13.4 umol/L
Acq. Date: 2025-05-15
Acq. Time: 17:51:45
Modified: Yes
Proc. Algorithm: Specify Parameters - MQ III

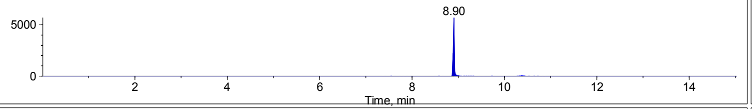

Sample Name: "S2" Sample ID: "" File: "20250515-50AA.wiff"
Peak Name: "MSLAB-Nvl(IS)" Mass(es): "288.150/171.010 Da"
Comment: "" Annotation: ""
Sample Index: 13
Sample Type: Standard
Concentration: 1.00 ng/mL
Calculated Conc: N/A
Acq. Date: 2025-05-15
Acq. Time: 17:51:45
Modified: Yes
Proc. Algorithm: Specify Parameters - MQ III

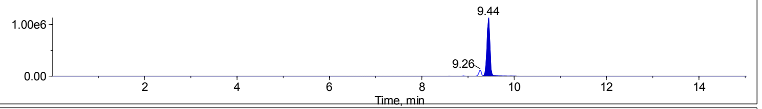

Sample Name: "S2" Sample ID: "" File: "20250515-50AA.wiff"
Peak Name: "tyr" Mass(es): "352.200/171.010 Da"
Comment: "" Annotation: ""
Sample Index: 13
Sample Type: Standard
Concentration: 6.26 umol/L
Calculated Conc: 6.29 umol/L
Acq. Date: 2025-05-15
Acq. Time: 17:51:45
Modified: Yes
Proc. Algorithm: Specify Parameters - MQ III

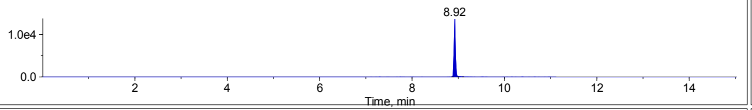

Sample Name: "S2" Sample ID: "" File: "20250515-50AA.wiff"
Peak Name: "MSLAB-Nvl(IS)" Mass(es): "288.150/171.010 Da"
Comment: "" Annotation: ""
Sample Index: 13
Sample Type: Standard
Concentration: 1.00 ng/mL
Calculated Conc: N/A
Acq. Date: 2025-05-15
Acq. Time: 17:51:45
Modified: Yes
Proc. Algorithm: Specify Parameters - MQ III

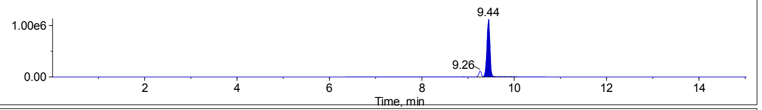

Sample Name: "S2" Sample ID: "" File: "20250515-50AA.wiff"
Peak Name: "Leu" Mass(es): "302.150/171.010 Da"
Comment: "" Annotation: ""
Sample Index: 13
Sample Type: Standard
Concentration: 12.5 umol/L
Calculated Conc: 12.3 umol/L
Acq. Date: 2025-05-15
Acq. Time: 17:51:45
Modified: Yes
Proc. Algorithm: Specify Parameters - MQ III

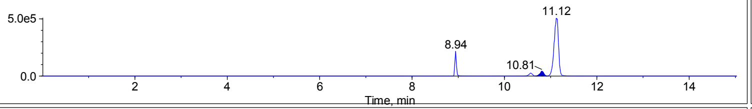

Sample Name: "S2" Sample ID: "" File: "20250515-50AA.wiff"
Peak Name: "MSLAB-Nvl(IS)" Mass(es): "288.150/171.010 Da"
Comment: "" Annotation: ""
Sample Index: 13
Sample Type: Standard
Concentration: 1.00 ng/mL
Calculated Conc: N/A
Acq. Date: 2025-05-15
Acq. Time: 17:51:45
Modified: Yes
Proc. Algorithm: Specify Parameters - MQ III

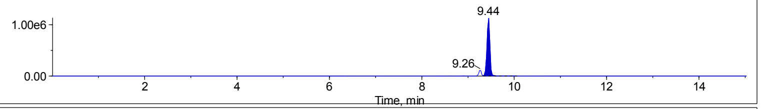

Sample Name: "S3" Sample ID: "" File: "20250515-50AA.wiff"
Peak Name: "Gly" Mass(es): "246.080/171.010 Da"
Comment: "" Annotation: ""
Sample Index: 14
Sample Type: Standard
Concentration: 50.0 umol/L
Calculated Conc: 48.5 umol/L
Acq. Date: 2025-05-15
Acq. Time: 18:07:13
Modified: Yes
Proc. Algorithm: Specify Parameters - MQ III

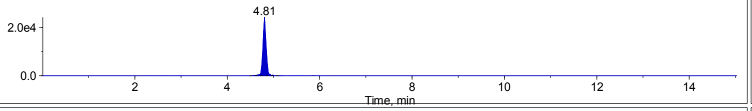

Sample Name: "S3" Sample ID: "" File: "20250515-50AA.wiff"
Peak Name: "MSLAB-Nvl(IS)" Mass(es): "288.150/171.010 Da"
Comment: "" Annotation: ""
Sample Index: 14
Sample Type: Standard
Concentration: 1.00 ng/mL
Calculated Conc: N/A
Acq. Date: 2025-05-15
Acq. Time: 18:07:13
Modified: Yes
Proc. Algorithm: Specify Parameters - MQ III

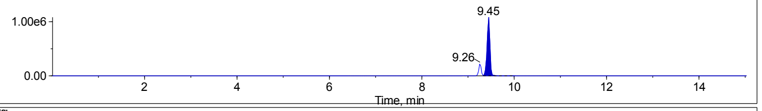

Sample Name: "S3" Sample ID: "" File: "20250515-50AA.wiff"
Peak Name: "Ala" Mass(es): "260.100/171.010 Da"
Comment: "" Annotation: ""
Sample Index: 14
Sample Type: Standard
Concentration: 100. umol/L
Calculated Conc: 111. umol/L
Acq. Date: 2025-05-15
Acq. Time: 18:07:13
Modified: Yes
Proc. Algorithm: Specify Parameters - MQ III

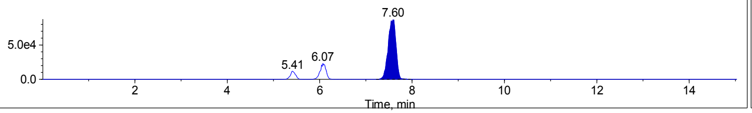

Sample Name: "S3" Sample ID: "" File: "20250515-50AA.wiff"
Peak Name: "MSLAB-Nvl(IS)" Mass(es): "288.150/171.010 Da"
Comment: "" Annotation: ""
Sample Index: 14
Sample Type: Standard
Concentration: 1.00 ng/mL
Calculated Conc: N/A
Acq. Date: 2025-05-15
Acq. Time: 18:07:13
Modified: Yes
Proc. Algorithm: Specify Parameters - MQ III

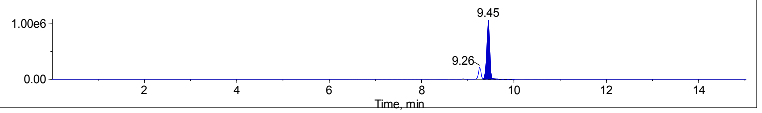

Sample Name: "S3" Sample ID: "" File: "20250515-50AA.wiff"
Peak Name: "Ser" Mass(es): "276.100/171.010 Da"
Comment: "" Annotation: ""
Sample Index: 14
Sample Type: Standard
Concentration: 25.0 umol/L
Calculated Conc: 24.4 umol/L
Acq. Date: 2025-05-15
Acq. Time: 18:07:13
Modified: Yes
Proc. Algorithm: Specify Parameters - MQ III

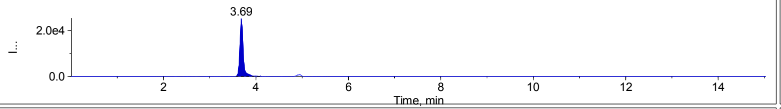

Sample Name: "S3" Sample ID: "" File: "20250515-50AA.wiff"
Peak Name: "Pro" Mass(es): "286.150/171.010 Da"
Comment: "" Annotation: ""
Sample Index: 14
Sample Type: Standard
Concentration: 50.0 umol/L
Calculated Conc: 54.0 umol/L
Acq. Date: 2025-05-15
Acq. Time: 18:07:13
Modified: Yes
Proc. Algorithm: Specify Parameters - MQ III

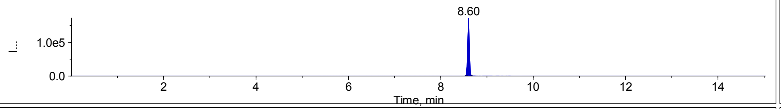

Sample Name: "S3" Sample ID: "" File: "20250515-50AA.wiff"
Peak Name: "Val" Mass(es): "288.150/171.010 Da"
Comment: "" Annotation: ""
Sample Index: 14
Sample Type: Standard
Concentration: 50.0 umol/L
Calculated Conc: 66.9 umol/L
Acq. Date: 2025-05-15
Acq. Time: 18:07:13
Modified: Yes
Proc. Algorithm: Specify Parameters - MQ III

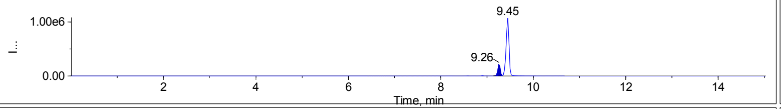

Sample Name: "S3" Sample ID: "" File: "20250515-50AA.wiff"
Peak Name: "Thr" Mass(es): "290.100/171.010 Da"
Comment: "" Annotation: ""
Sample Index: 14
Sample Type: Standard
Concentration: 25.0 umol/L
Calculated Conc: 24.3 umol/L
Acq. Date: 2025-05-15
Acq. Time: 18:07:13
Modified: Yes
Proc. Algorithm: Specify Parameters - MQ III

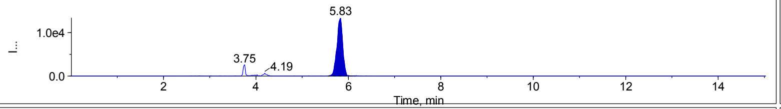

Sample Name: "S3" Sample ID: "" File: "20250515-50AA.wiff"
Peak Name: "Cys1/2" Mass(es): "292.200/171.010 Da"
Comment: "" Annotation: ""
Sample Index: 14
Sample Type: Standard
Concentration: 12.5 umol/L
Calculated Conc: 13.2 umol/L
Acq. Date: 2025-05-15
Acq. Time: 18:07:13
Modified: Yes
Proc. Algorithm: Specify Parameters - MQ III

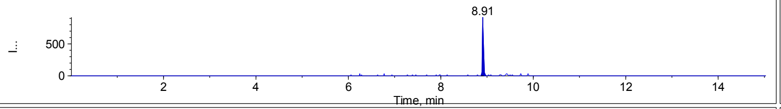

Sample Name: "S3" Sample ID: "" File: "20250515-50AA.wiff"
Peak Name: "Ile" Mass(es): "302.150/171.010 Da"
Comment: "" Annotation: ""
Sample Index: 14
Sample Type: Standard
Concentration: 12.5 umol/L
Calculated Conc: 12.5 umol/L
Acq. Date: 2025-05-15
Acq. Time: 18:07:13
Modified: Yes
Proc. Algorithm: Specify Parameters - MQ III

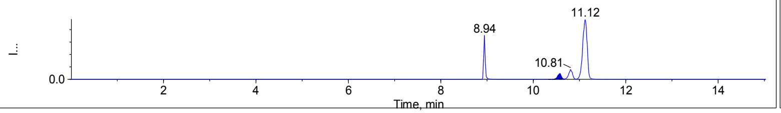

Sample Name: "S3" Sample ID: "" File: "20250515-50AA.wiff"
Peak Name: "MSLAB-Nvl(IS)" Mass(es): "288.150/171.010 Da"
Comment: "" Annotation: ""
Sample Index: 14
Sample Type: Standard
Concentration: 1.00 ng/mL
Calculated Conc: N/A
Acq. Date: 2025-05-15
Acq. Time: 18:07:13
Modified: Yes
Proc. Algorithm: Specify Parameters - MQ III

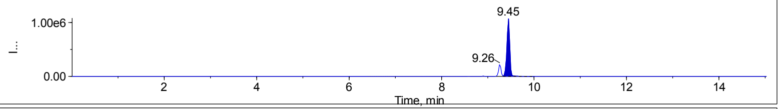

Sample Name: "S3" Sample ID: "" File: "20250515-50AA.wiff"
Peak Name: "MSLAB-Nvl(IS)" Mass(es): "288.150/171.010 Da"
Comment: "" Annotation: ""
Sample Index: 14
Sample Type: Standard
Concentration: 1.00 ng/mL
Calculated Conc: N/A
Acq. Date: 2025-05-15
Acq. Time: 18:07:13
Modified: Yes
Proc. Algorithm: Specify Parameters - MQ III

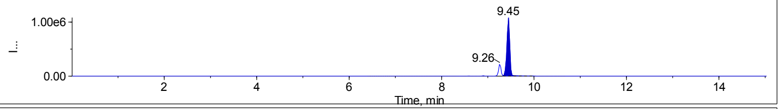

Sample Name: "S3" Sample ID: "" File: "20250515-50AA.wiff"
Peak Name: "MSLAB-Nvl(IS)" Mass(es): "288.150/171.010 Da"
Comment: "" Annotation: ""
Sample Index: 14
Sample Type: Standard
Concentration: 1.00 ng/mL
Calculated Conc: N/A
Acq. Date: 2025-05-15
Acq. Time: 18:07:13
Modified: Yes
Proc. Algorithm: Specify Parameters - MQ III

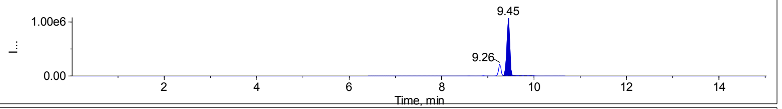

Sample Name: "S3" Sample ID: "" File: "20250515-50AA.wiff"
Peak Name: "MSLAB-Nvl(IS)" Mass(es): "288.150/171.010 Da"
Comment: "" Annotation: ""
Sample Index: 14
Sample Type: Standard
Concentration: 1.00 ng/mL
Calculated Conc: N/A
Acq. Date: 2025-05-15
Acq. Time: 18:07:13
Modified: Yes
Proc. Algorithm: Specify Parameters - MQ III

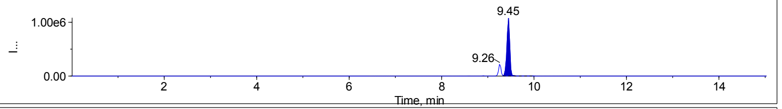

Sample Name: "S3" Sample ID: "" File: "20250515-50AA.wiff"
Peak Name: "MSLAB-Nvl(IS)" Mass(es): "288.150/171.010 Da"
Comment: "" Annotation: ""
Sample Index: 14
Sample Type: Standard
Concentration: 1.00 ng/mL
Calculated Conc: N/A
Acq. Date: 2025-05-15
Acq. Time: 18:07:13
Modified: Yes
Proc. Algorithm: Specify Parameters - MQ III

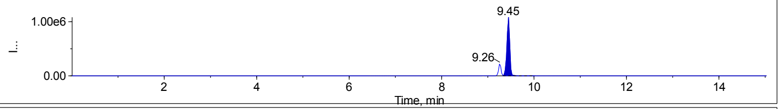

Sample Name: "S3" Sample ID: "" File: "20250515-50AA.wiff"
Peak Name: "MSLAB-Nvl(IS)" Mass(es): "288.150/171.010 Da"
Comment: "" Annotation: ""
Sample Index: 14
Sample Type: Standard
Concentration: 1.00 ng/mL
Calculated Conc: N/A
Acq. Date: 2025-05-15
Acq. Time: 18:07:13
Modified: Yes
Proc. Algorithm: Specify Parameters - MQ III

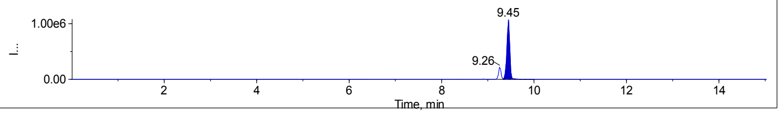

Sample Name: "S3" Sample ID: "" File: "20250515-50AA.wiff"
Peak Name: "Asn" Mass(es): "303.130/171.010 Da"
Comment: "" Annotation: ""

```
Sample Index:       14
Sample Type:        Standard
Concentration:      12.5   umol/L
Calculated Conc:    12.7   umol/L
Acq. Date:          2025-05-15
Acq. Time:          18:07:13

Modified:              Yes
Proc. Algorithm: Specify Parameters - MQ III
```

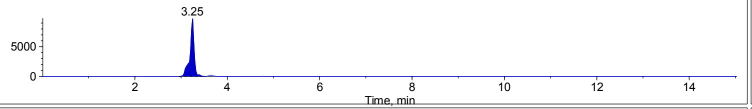

Sample Name: "S3" Sample ID: "" File: "20250515-50AA.wiff"
Peak Name: "MSLAB-Nvl(IS)" Mass(es): "288.150/171.010 Da"
Comment: "" Annotation: ""

```
Sample Index:       14
Sample Type:        Standard
Concentration:      1.00    ng/mL
Calculated Conc:    N/A
Acq. Date:          2025-05-15
Acq. Time:          18:07:13

Modified:              Yes
Proc. Algorithm: Specify Parameters - MQ III
```

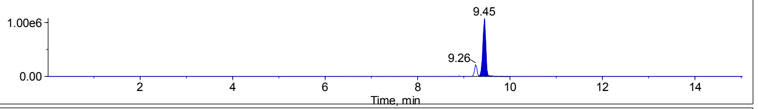

Sample Name: "S3" Sample ID: "" File: "20250515-50AA.wiff"
Peak Name: "Asp" Mass(es): "304.110/171.010 Da"
Comment: "" Annotation: ""

```
Sample Index:       14
Sample Type:        Standard
Concentration:      12.5   umol/L
Calculated Conc:    10.3   umol/L
Acq. Date:          2025-05-15
Acq. Time:          18:07:13

Modified:              Yes
Proc. Algorithm: Specify Parameters - MQ III
```

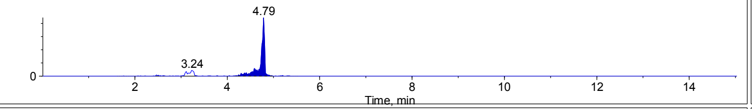

Sample Name: "S3" Sample ID: "" File: "20250515-50AA.wiff"
Peak Name: "MSLAB-Nvl(IS)" Mass(es): "288.150/171.010 Da"
Comment: "" Annotation: ""

```
Sample Index:       14
Sample Type:        Standard
Concentration:      1.00    ng/mL
Calculated Conc:    N/A
Acq. Date:          2025-05-15
Acq. Time:          18:07:13

Modified:              Yes
Proc. Algorithm: Specify Parameters - MQ III
```

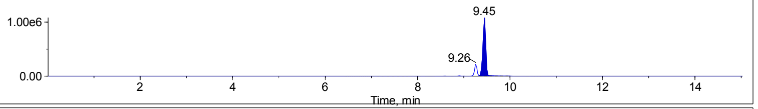

Sample Name: "S3" Sample ID: "" File: "20250515-50AA.wiff"
Peak Name: "Gln" Mass(es): "317.150/171.010 Da"
Comment: "" Annotation: ""

```
Sample Index:       14
Sample Type:        Standard
Concentration:      100.   umol/L
Calculated Conc:    97.8   umol/L
Acq. Date:          2025-05-15
Acq. Time:          18:07:13

Modified:              Yes
Proc. Algorithm: Specify Parameters - MQ III
```

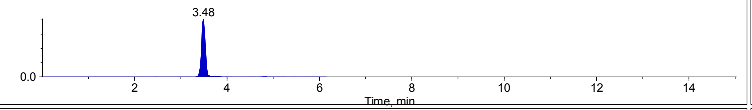

Sample Name: "S3" Sample ID: "" File: "20250515-50AA.wiff"
Peak Name: "MSLAB-Nvl(IS)" Mass(es): "288.150/171.010 Da"
Comment: "" Annotation: ""

```
Sample Index:       14
Sample Type:        Standard
Concentration:      1.00    ng/mL
Calculated Conc:    N/A
Acq. Date:          2025-05-15
Acq. Time:          18:07:13

Modified:              Yes
Proc. Algorithm: Specify Parameters - MQ III
```

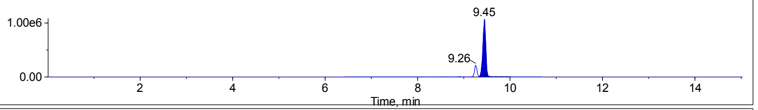

Sample Name: "S3" Sample ID: "" File: "20250515-50AA.wiff"
Peak Name: "Glu" Mass(es): "318.100/171.010 Da"
Comment: "" Annotation: ""

```
Sample Index:       14
Sample Type:        Standard
Concentration:      12.5   umol/L
Calculated Conc:    12.5   umol/L
Acq. Date:          2025-05-15
Acq. Time:          18:07:13

Modified:              Yes
Proc. Algorithm: Specify Parameters - MQ III
```

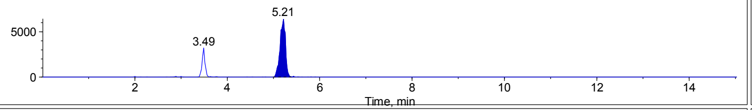

Sample Name: "S3" Sample ID: "" File: "20250515-50AA.wiff"
Peak Name: "MSLAB-Nvl(IS)" Mass(es): "288.150/171.010 Da"
Comment: "" Annotation: ""

```
Sample Index:       14
Sample Type:        Standard
Concentration:      1.00    ng/mL
Calculated Conc:    N/A
Acq. Date:          2025-05-15
Acq. Time:          18:07:13

Modified:              Yes
Proc. Algorithm: Specify Parameters - MQ III
```

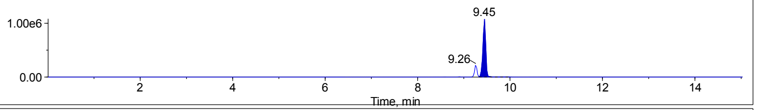

Sample Name: "S3" Sample ID: "" File: "20250515-50AA.wiff"
Peak Name: "Met" Mass(es): "320.210/171.010 Da"
Comment: "" Annotation: ""

```
Sample Index:       14
Sample Type:        Standard
Concentration:      12.5   umol/L
Calculated Conc:    13.1   umol/L
Acq. Date:          2025-05-15
Acq. Time:          18:07:13

Modified:              Yes
Proc. Algorithm: Specify Parameters - MQ III
```

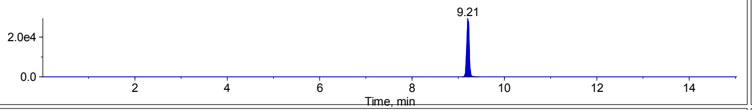

Sample Name: "S3" Sample ID: "" File: "20250515-50AA.wiff"
Peak Name: "MSLAB-Nvl(IS)" Mass(es): "288.150/171.010 Da"
Comment: "" Annotation: ""

```
Sample Index:       14
Sample Type:        Standard
Concentration:      1.00    ng/mL
Calculated Conc:    N/A
Acq. Date:          2025-05-15
Acq. Time:          18:07:13

Modified:              Yes
Proc. Algorithm: Specify Parameters - MQ III
```

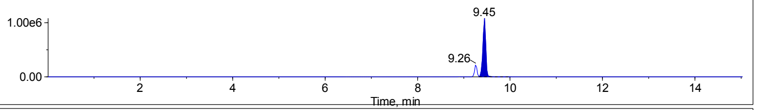

Sample Name: "S3" Sample ID: "" File: "20250515-50AA.wiff"
Peak Name: "His" Mass(es): "326.150/171.010 Da"
Comment: "" Annotation: ""

```
Sample Index:       14
Sample Type:        Standard
Concentration:      12.5   umol/L
Calculated Conc:    11.6   umol/L
Acq. Date:          2025-05-15
Acq. Time:          18:07:13

Modified:              Yes
Proc. Algorithm: Specify Parameters - MQ III
```

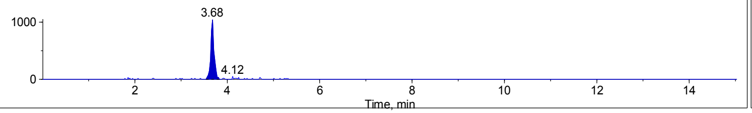

Sample Name: "S3" Sample ID: "" File: "20250515-50AA.wiff"
Peak Name: "MSLAB-Nvl(IS)" Mass(es): "288.150/171.010 Da"
Comment: "" Annotation: ""

```
Sample Index:       14
Sample Type:        Standard
Concentration:      1.00    ng/mL
Calculated Conc:    N/A
Acq. Date:          2025-05-15
Acq. Time:          18:07:13

Modified:              Yes
Proc. Algorithm: Specify Parameters - MQ III
```

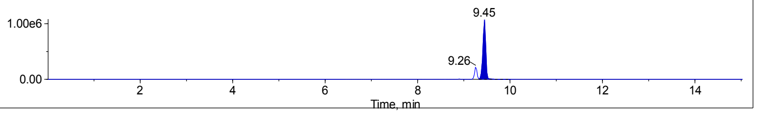

Sample Name: "S3" Sample ID: "" File: "20250515-50AA.wiff"
Peak Name: "Phe" Mass(es): "336.200/171.010 Da"
Comment: "" Annotation: ""
Sample Index: 14
Sample Type: Standard
Concentration: 12.5 umol/L
Calculated Conc: 12.4 umol/L
Acq. Date: 2025-05-15
Acq. Time: 18:07:13
Modified: Yes
Proc. Algorithm: Specify Parameters - MQ III

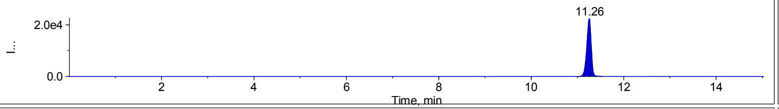

Sample Name: "S3" Sample ID: "" File: "20250515-50AA.wiff"
Peak Name: "MSLAB-Nvl(IS)" Mass(es): "288.150/171.010 Da"
Comment: "" Annotation: ""
Sample Index: 14
Sample Type: Standard
Concentration: 1.00 ng/mL
Calculated Conc: N/A
Acq. Date: 2025-05-15
Acq. Time: 18:07:13
Modified: Yes
Proc. Algorithm: Specify Parameters - MQ III

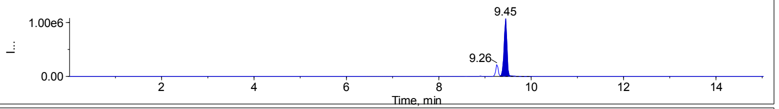

Sample Name: "S3" Sample ID: "" File: "20250515-50AA.wiff"
Peak Name: "Arg" Mass(es): "345.210/171.010 Da"
Comment: "" Annotation: ""
Sample Index: 14
Sample Type: Standard
Concentration: 25.0 umol/L
Calculated Conc: 25.5 umol/L
Acq. Date: 2025-05-15
Acq. Time: 18:07:13
Modified: Yes
Proc. Algorithm: Specify Parameters - MQ III

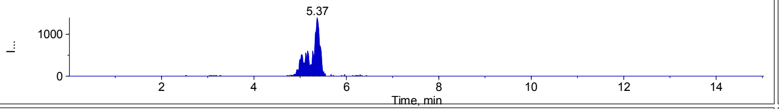

Sample Name: "S3" Sample ID: "" File: "20250515-50AA.wiff"
Peak Name: "MSLAB-Nvl(IS)" Mass(es): "288.150/171.010 Da"
Comment: "" Annotation: ""
Sample Index: 14
Sample Type: Standard
Concentration: 1.00 ng/mL
Calculated Conc: N/A
Acq. Date: 2025-05-15
Acq. Time: 18:07:13
Modified: Yes
Proc. Algorithm: Specify Parameters - MQ III

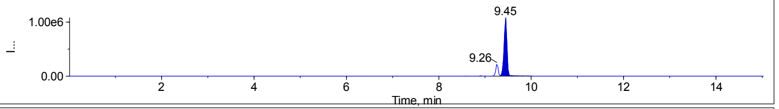

Sample Name: "S3" Sample ID: "" File: "20250515-50AA.wiff"
Peak Name: "Trp" Mass(es): "375.200/171.010 Da"
Comment: "" Annotation: ""
Sample Index: 14
Sample Type: Standard
Concentration: 12.5 umol/L
Calculated Conc: 12.7 umol/L
Acq. Date: 2025-05-15
Acq. Time: 18:07:13
Modified: Yes
Proc. Algorithm: Specify Parameters - MQ III

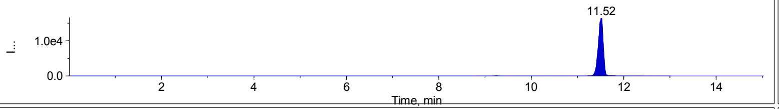

Sample Name: "S3" Sample ID: "" File: "20250515-50AA.wiff"
Peak Name: "MSLAB-Nvl(IS)" Mass(es): "288.150/171.010 Da"
Comment: "" Annotation: ""
Sample Index: 14
Sample Type: Standard
Concentration: 1.00 ng/mL
Calculated Conc: N/A
Acq. Date: 2025-05-15
Acq. Time: 18:07:13
Modified: Yes
Proc. Algorithm: Specify Parameters - MQ III

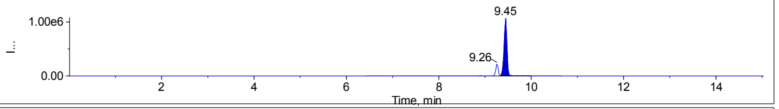

Sample Name: "S3" Sample ID: "" File: "20250515-50AA.wiff"
Peak Name: "Lys" Mass(es): "487.200/171.010 Da"
Comment: "" Annotation: ""
Sample Index: 14
Sample Type: Standard
Concentration: 25.0 umol/L
Calculated Conc: 25.7 umol/L
Acq. Date: 2025-05-15
Acq. Time: 18:07:13
Modified: Yes
Proc. Algorithm: Specify Parameters - MQ III

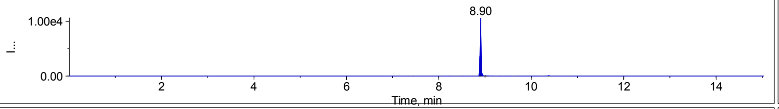

Sample Name: "S3" Sample ID: "" File: "20250515-50AA.wiff"
Peak Name: "MSLAB-Nvl(IS)" Mass(es): "288.150/171.010 Da"
Comment: "" Annotation: ""
Sample Index: 14
Sample Type: Standard
Concentration: 1.00 ng/mL
Calculated Conc: N/A
Acq. Date: 2025-05-15
Acq. Time: 18:07:13
Modified: Yes
Proc. Algorithm: Specify Parameters - MQ III

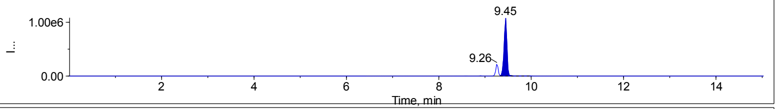

Sample Name: "S3" Sample ID: "" File: "20250515-50AA.wiff"
Peak Name: "tyr" Mass(es): "352.200/171.010 Da"
Comment: "" Annotation: ""
Sample Index: 14
Sample Type: Standard
Concentration: 12.5 umol/L
Calculated Conc: 12.0 umol/L
Acq. Date: 2025-05-15
Acq. Time: 18:07:13
Modified: Yes
Proc. Algorithm: Specify Parameters - MQ III

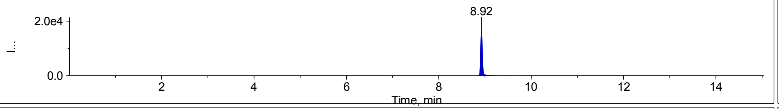

Sample Name: "S3" Sample ID: "" File: "20250515-50AA.wiff"
Peak Name: "MSLAB-Nvl(IS)" Mass(es): "288.150/171.010 Da"
Comment: "" Annotation: ""
Sample Index: 14
Sample Type: Standard
Concentration: 1.00 ng/mL
Calculated Conc: N/A
Acq. Date: 2025-05-15
Acq. Time: 18:07:13
Modified: Yes
Proc. Algorithm: Specify Parameters - MQ III

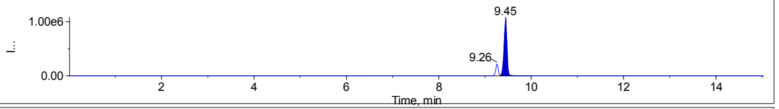

Sample Name: "S3" Sample ID: "" File: "20250515-50AA.wiff"
Peak Name: "Leu" Mass(es): "302.150/171.010 Da"
Comment: "" Annotation: ""
Sample Index: 14
Sample Type: Standard
Concentration: 25.0 umol/L
Calculated Conc: 25.0 umol/L
Acq. Date: 2025-05-15
Acq. Time: 18:07:13
Modified: Yes
Proc. Algorithm: Specify Parameters - MQ III

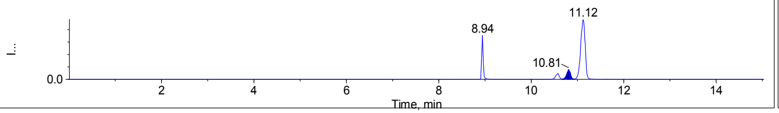

Sample Name: "S3" Sample ID: "" File: "20250515-50AA.wiff"
Peak Name: "MSLAB-Nvl(IS)" Mass(es): "288.150/171.010 Da"
Comment: "" Annotation: ""
Sample Index: 14
Sample Type: Standard
Concentration: 1.00 ng/mL
Calculated Conc: N/A
Acq. Date: 2025-05-15
Acq. Time: 18:07:13
Modified: Yes
Proc. Algorithm: Specify Parameters - MQ III

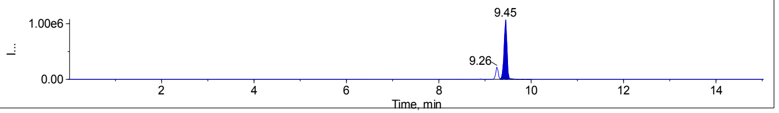

Sample Name: "S4"   Sample ID: ""   File: "20250515-50AA.wiff"
Peak Name: "Gly"   Mass(es): "246.080/171.010 Da"
Comment: ""   Annotation: ""

```
Sample Index:       15
Sample Type:        Standard
Concentration:      100.    umol/L
Calculated Conc:    102.    umol/L
Acq. Date:          2025-05-15
Acq. Time:          18:22:42

Modified:           Yes
Proc. Algorithm: Specify Parameters - MQ III
```

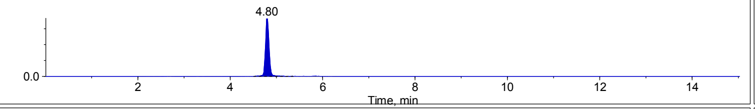

Sample Name: "S4"   Sample ID: ""   File: "20250515-50AA.wiff"
Peak Name: "MSLAB-Nvl(IS)"   Mass(es): "288.150/171.010 Da"
Comment: ""   Annotation: ""

```
Sample Index:       15
Sample Type:        Standard
Concentration:      1.00    ng/mL
Calculated Conc:    N/A
Acq. Date:          2025-05-15
Acq. Time:          18:22:42

Modified:           Yes
Proc. Algorithm: Specify Parameters - MQ III
```

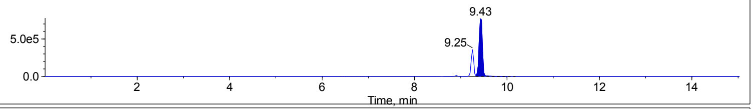

Sample Name: "S4"   Sample ID: ""   File: "20250515-50AA.wiff"
Peak Name: "Ala"   Mass(es): "260.100/171.010 Da"
Comment: ""   Annotation: ""

```
Sample Index:       15
Sample Type:        Standard
Concentration:      200.    umol/L
Calculated Conc:    229.    umol/L
Acq. Date:          2025-05-15
Acq. Time:          18:22:42

Modified:           Yes
Proc. Algorithm: Specify Parameters - MQ III
```

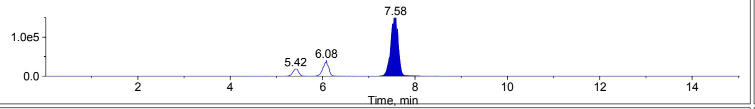

Sample Name: "S4"   Sample ID: ""   File: "20250515-50AA.wiff"
Peak Name: "MSLAB-Nvl(IS)"   Mass(es): "288.150/171.010 Da"
Comment: ""   Annotation: ""

```
Sample Index:       15
Sample Type:        Standard
Concentration:      1.00    ng/mL
Calculated Conc:    N/A
Acq. Date:          2025-05-15
Acq. Time:          18:22:42

Modified:           Yes
Proc. Algorithm: Specify Parameters - MQ III
```

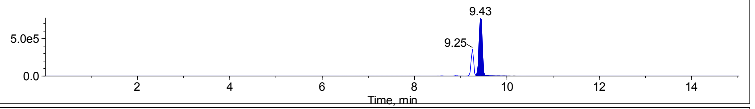

Sample Name: "S4"   Sample ID: ""   File: "20250515-50AA.wiff"
Peak Name: "Ser"   Mass(es): "276.100/171.010 Da"
Comment: ""   Annotation: ""

```
Sample Index:       15
Sample Type:        Standard
Concentration:      50.0    umol/L
Calculated Conc:    47.8    umol/L
Acq. Date:          2025-05-15
Acq. Time:          18:22:42

Modified:           Yes
Proc. Algorithm: Specify Parameters - MQ III
```

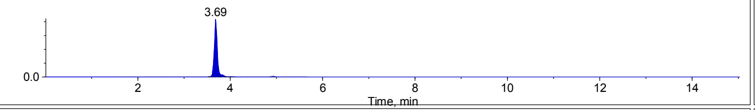

Sample Name: "S4"   Sample ID: ""   File: "20250515-50AA.wiff"
Peak Name: "MSLAB-Nvl(IS)"   Mass(es): "288.150/171.010 Da"
Comment: ""   Annotation: ""

```
Sample Index:       15
Sample Type:        Standard
Concentration:      1.00    ng/mL
Calculated Conc:    N/A
Acq. Date:          2025-05-15
Acq. Time:          18:22:42

Modified:           Yes
Proc. Algorithm: Specify Parameters - MQ III
```

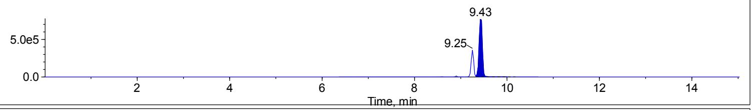

Sample Name: "S4"   Sample ID: ""   File: "20250515-50AA.wiff"
Peak Name: "Pro"   Mass(es): "286.150/171.010 Da"
Comment: ""   Annotation: ""

```
Sample Index:       15
Sample Type:        Standard
Concentration:      100.    umol/L
Calculated Conc:    122.    umol/L
Acq. Date:          2025-05-15
Acq. Time:          18:22:42

Modified:           Yes
Proc. Algorithm: Specify Parameters - MQ III
```

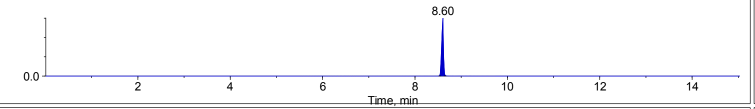

Sample Name: "S4"   Sample ID: ""   File: "20250515-50AA.wiff"
Peak Name: "MSLAB-Nvl(IS)"   Mass(es): "288.150/171.010 Da"
Comment: ""   Annotation: ""

```
Sample Index:       15
Sample Type:        Standard
Concentration:      1.00    ng/mL
Calculated Conc:    N/A
Acq. Date:          2025-05-15
Acq. Time:          18:22:42

Modified:           Yes
Proc. Algorithm: Specify Parameters - MQ III
```

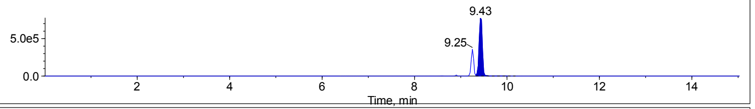

Sample Name: "S4"   Sample ID: ""   File: "20250515-50AA.wiff"
Peak Name: "Val"   Mass(es): "288.150/171.010 Da"
Comment: ""   Annotation: ""

```
Sample Index:       15
Sample Type:        Standard
Concentration:      100.    umol/L
Calculated Conc:    133.    umol/L
Acq. Date:          2025-05-15
Acq. Time:          18:22:42

Modified:           Yes
Proc. Algorithm: Specify Parameters - MQ III
```

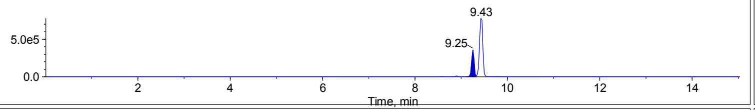

Sample Name: "S4"   Sample ID: ""   File: "20250515-50AA.wiff"
Peak Name: "MSLAB-Nvl(IS)"   Mass(es): "288.150/171.010 Da"
Comment: ""   Annotation: ""

```
Sample Index:       15
Sample Type:        Standard
Concentration:      1.00    ng/mL
Calculated Conc:    N/A
Acq. Date:          2025-05-15
Acq. Time:          18:22:42

Modified:           Yes
Proc. Algorithm: Specify Parameters - MQ III
```

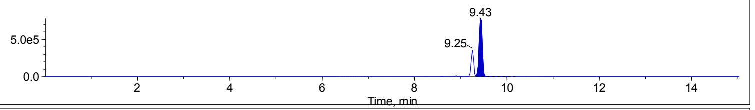

Sample Name: "S4"   Sample ID: ""   File: "20250515-50AA.wiff"
Peak Name: "Thr"   Mass(es): "290.100/171.010 Da"
Comment: ""   Annotation: ""

```
Sample Index:       15
Sample Type:        Standard
Concentration:      50.0    umol/L
Calculated Conc:    50.5    umol/L
Acq. Date:          2025-05-15
Acq. Time:          18:22:42

Modified:           Yes
Proc. Algorithm: Specify Parameters - MQ III
```

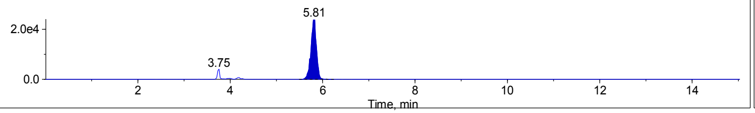

Sample Name: "S4"   Sample ID: ""   File: "20250515-50AA.wiff"
Peak Name: "MSLAB-Nvl(IS)"   Mass(es): "288.150/171.010 Da"
Comment: ""   Annotation: ""

```
Sample Index:       15
Sample Type:        Standard
Concentration:      1.00    ng/mL
Calculated Conc:    N/A
Acq. Date:          2025-05-15
Acq. Time:          18:22:42

Modified:           Yes
Proc. Algorithm: Specify Parameters - MQ III
```

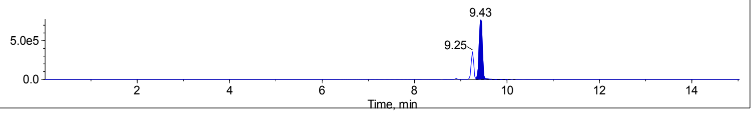

Sample Name: "S4" Sample ID: "" File: "20250515-50AA.wiff"
Peak Name: "Cys1/2" Mass(es): "292.200/171.010 Da"
Comment: "" Annotation: ""

Sample Index: 15
Sample Type: Standard
Concentration: 25.0 umol/L
Calculated Conc: 21.8 umol/L
Acq. Date: 2025-05-15
Acq. Time: 18:22:42

Modified: Yes
Proc. Algorithm: Specify Parameters - MQ III

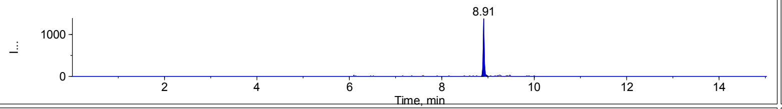

Sample Name: "S4" Sample ID: "" File: "20250515-50AA.wiff"
Peak Name: "MSLAB-Nvl(IS)" Mass(es): "288.150/171.010 Da"
Comment: "" Annotation: ""

Sample Index: 15
Sample Type: Standard
Concentration: 1.00 ng/mL
Calculated Conc: N/A
Acq. Date: 2025-05-15
Acq. Time: 18:22:42

Modified: Yes
Proc. Algorithm: Specify Parameters - MQ III

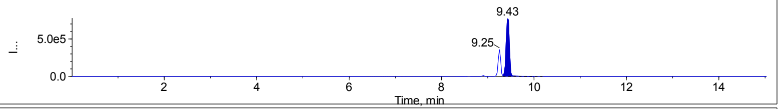

Sample Name: "S4" Sample ID: "" File: "20250515-50AA.wiff"
Peak Name: "Ile" Mass(es): "302.150/171.010 Da"
Comment: "" Annotation: ""

Sample Index: 15
Sample Type: Standard
Concentration: 25.0 umol/L
Calculated Conc: 25.9 umol/L
Acq. Date: 2025-05-15
Acq. Time: 18:22:42

Modified: Yes
Proc. Algorithm: Specify Parameters - MQ III

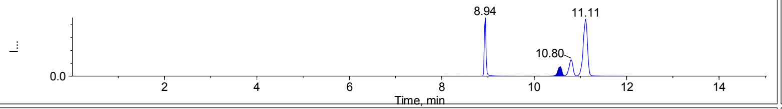

Sample Name: "S4" Sample ID: "" File: "20250515-50AA.wiff"
Peak Name: "MSLAB-Nvl(IS)" Mass(es): "288.150/171.010 Da"
Comment: "" Annotation: ""

Sample Index: 15
Sample Type: Standard
Concentration: 1.00 ng/mL
Calculated Conc: N/A
Acq. Date: 2025-05-15
Acq. Time: 18:22:42

Modified: Yes
Proc. Algorithm: Specify Parameters - MQ III

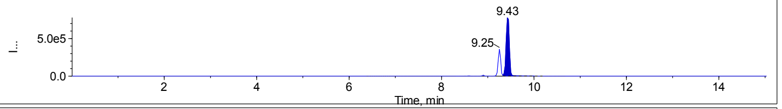

Sample Name: "S4" Sample ID: "" File: "20250515-50AA.wiff"
Peak Name: "Asn" Mass(es): "303.130/171.010 Da"
Comment: "" Annotation: ""

Sample Index: 15
Sample Type: Standard
Concentration: 25.0 umol/L
Calculated Conc: 25.5 umol/L
Acq. Date: 2025-05-15
Acq. Time: 18:22:42

Modified: Yes
Proc. Algorithm: Specify Parameters - MQ III

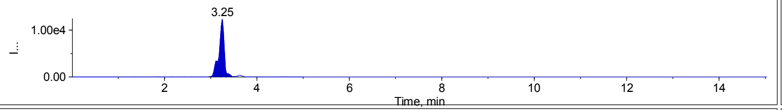

Sample Name: "S4" Sample ID: "" File: "20250515-50AA.wiff"
Peak Name: "MSLAB-Nvl(IS)" Mass(es): "288.150/171.010 Da"
Comment: "" Annotation: ""

Sample Index: 15
Sample Type: Standard
Concentration: 1.00 ng/mL
Calculated Conc: N/A
Acq. Date: 2025-05-15
Acq. Time: 18:22:42

Modified: Yes
Proc. Algorithm: Specify Parameters - MQ III

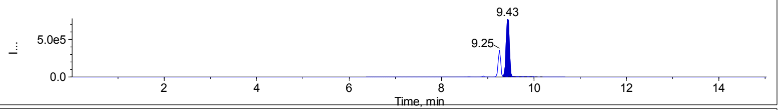

Sample Name: "S4" Sample ID: "" File: "20250515-50AA.wiff"
Peak Name: "Asp" Mass(es): "304.110/171.010 Da"
Comment: "" Annotation: ""

Sample Index: 15
Sample Type: Standard
Concentration: 25.0 umol/L
Calculated Conc: 25.5 umol/L
Acq. Date: 2025-05-15
Acq. Time: 18:22:42

Modified: Yes
Proc. Algorithm: Specify Parameters - MQ III

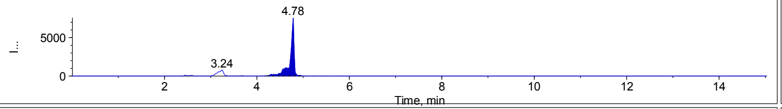

Sample Name: "S4" Sample ID: "" File: "20250515-50AA.wiff"
Peak Name: "MSLAB-Nvl(IS)" Mass(es): "288.150/171.010 Da"
Comment: "" Annotation: ""

Sample Index: 15
Sample Type: Standard
Concentration: 1.00 ng/mL
Calculated Conc: N/A
Acq. Date: 2025-05-15
Acq. Time: 18:22:42

Modified: Yes
Proc. Algorithm: Specify Parameters - MQ III

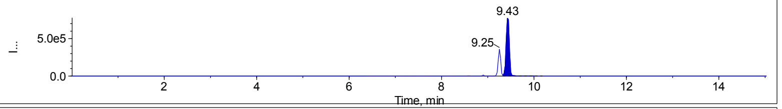

Sample Name: "S4" Sample ID: "" File: "20250515-50AA.wiff"
Peak Name: "Gln" Mass(es): "317.150/171.010 Da"
Comment: "" Annotation: ""

Sample Index: 15
Sample Type: Standard
Concentration: 200. umol/L
Calculated Conc: 202. umol/L
Acq. Date: 2025-05-15
Acq. Time: 18:22:42

Modified: Yes
Proc. Algorithm: Specify Parameters - MQ III

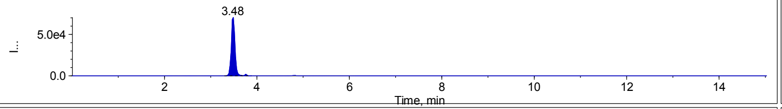

Sample Name: "S4" Sample ID: "" File: "20250515-50AA.wiff"
Peak Name: "MSLAB-Nvl(IS)" Mass(es): "288.150/171.010 Da"
Comment: "" Annotation: ""

Sample Index: 15
Sample Type: Standard
Concentration: 1.00 ng/mL
Calculated Conc: N/A
Acq. Date: 2025-05-15
Acq. Time: 18:22:42

Modified: Yes
Proc. Algorithm: Specify Parameters - MQ III

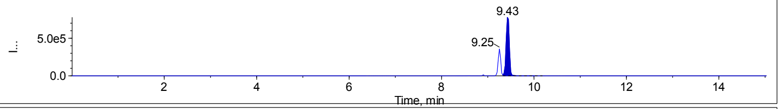

Sample Name: "S4" Sample ID: "" File: "20250515-50AA.wiff"
Peak Name: "Glu" Mass(es): "318.100/171.010 Da"
Comment: "" Annotation: ""

Sample Index: 15
Sample Type: Standard
Concentration: 25.0 umol/L
Calculated Conc: 24.6 umol/L
Acq. Date: 2025-05-15
Acq. Time: 18:22:42

Modified: Yes
Proc. Algorithm: Specify Parameters - MQ III

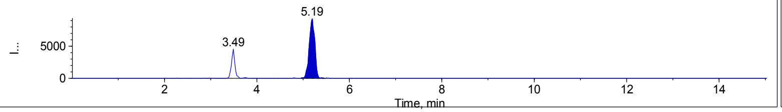

Sample Name: "S4" Sample ID: "" File: "20250515-50AA.wiff"
Peak Name: "MSLAB-Nvl(IS)" Mass(es): "288.150/171.010 Da"
Comment: "" Annotation: ""

Sample Index: 15
Sample Type: Standard
Concentration: 1.00 ng/mL
Calculated Conc: N/A
Acq. Date: 2025-05-15
Acq. Time: 18:22:42

Modified: Yes
Proc. Algorithm: Specify Parameters - MQ III

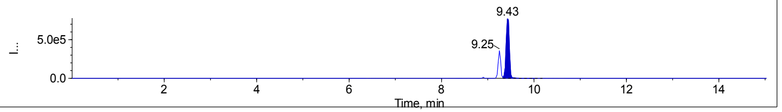

Sample Name: "S4" Sample ID: "" File: "20250515-50AA.wiff"
Peak Name: "Met" Mass(es): "320.210/171.010 Da"
Comment: "" Annotation: ""
Sample Index: 15
Sample Type: Standard
Concentration: 25.0 umol/L
Calculated Conc: 25.8 umol/L
Acq. Date: 2025-05-15
Acq. Time: 18:22:42
Modified: Yes
Proc. Algorithm: Specify Parameters - MQ III

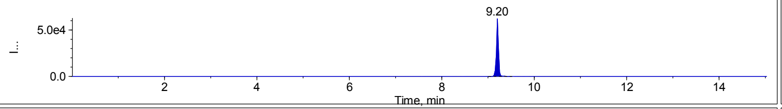

Sample Name: "S4" Sample ID: "" File: "20250515-50AA.wiff"
Peak Name: "MSLAB-Nvl(IS)" Mass(es): "288.150/171.010 Da"
Comment: "" Annotation: ""
Sample Index: 15
Sample Type: Standard
Concentration: 1.00 ng/mL
Calculated Conc: N/A
Acq. Date: 2025-05-15
Acq. Time: 18:22:42
Modified: Yes
Proc. Algorithm: Specify Parameters - MQ III

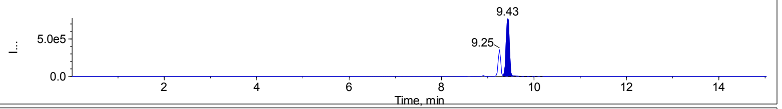

Sample Name: "S4" Sample ID: "" File: "20250515-50AA.wiff"
Peak Name: "His" Mass(es): "326.150/171.010 Da"
Comment: "" Annotation: ""
Sample Index: 15
Sample Type: Standard
Concentration: 25.0 umol/L
Calculated Conc: 25.0 umol/L
Acq. Date: 2025-05-15
Acq. Time: 18:22:42
Modified: Yes
Proc. Algorithm: Specify Parameters - MQ III

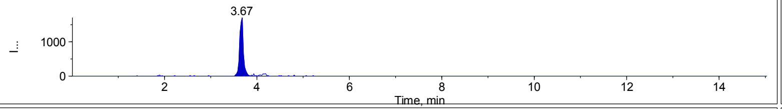

Sample Name: "S4" Sample ID: "" File: "20250515-50AA.wiff"
Peak Name: "MSLAB-Nvl(IS)" Mass(es): "288.150/171.010 Da"
Comment: "" Annotation: ""
Sample Index: 15
Sample Type: Standard
Concentration: 1.00 ng/mL
Calculated Conc: N/A
Acq. Date: 2025-05-15
Acq. Time: 18:22:42
Modified: Yes
Proc. Algorithm: Specify Parameters - MQ III

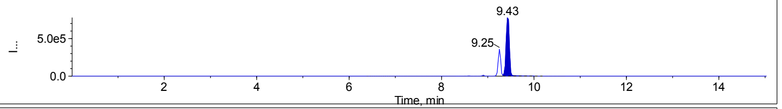

Sample Name: "S4" Sample ID: "" File: "20250515-50AA.wiff"
Peak Name: "Phe" Mass(es): "336.200/171.010 Da"
Comment: "" Annotation: ""
Sample Index: 15
Sample Type: Standard
Concentration: 25.0 umol/L
Calculated Conc: 28.2 umol/L
Acq. Date: 2025-05-15
Acq. Time: 18:22:42
Modified: Yes
Proc. Algorithm: Specify Parameters - MQ III

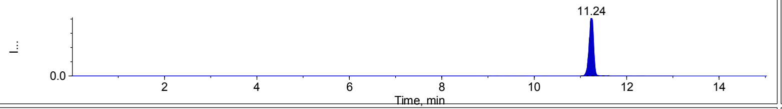

Sample Name: "S4" Sample ID: "" File: "20250515-50AA.wiff"
Peak Name: "MSLAB-Nvl(IS)" Mass(es): "288.150/171.010 Da"
Comment: "" Annotation: ""
Sample Index: 15
Sample Type: Standard
Concentration: 1.00 ng/mL
Calculated Conc: N/A
Acq. Date: 2025-05-15
Acq. Time: 18:22:42
Modified: Yes
Proc. Algorithm: Specify Parameters - MQ III

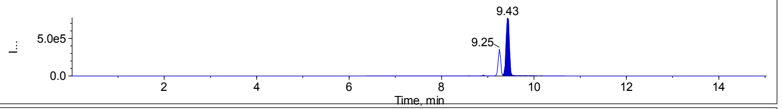

Sample Name: "S4" Sample ID: "" File: "20250515-50AA.wiff"
Peak Name: "Arg" Mass(es): "345.210/171.010 Da"
Comment: "" Annotation: ""
Sample Index: 15
Sample Type: Standard
Concentration: 50.0 umol/L
Calculated Conc: 44.0 umol/L
Acq. Date: 2025-05-15
Acq. Time: 18:22:42
Modified: Yes
Proc. Algorithm: Specify Parameters - MQ III

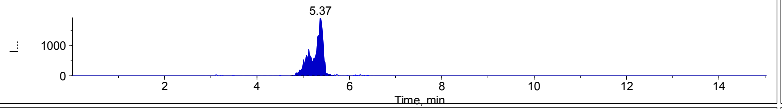

Sample Name: "S4" Sample ID: "" File: "20250515-50AA.wiff"
Peak Name: "MSLAB-Nvl(IS)" Mass(es): "288.150/171.010 Da"
Comment: "" Annotation: ""
Sample Index: 15
Sample Type: Standard
Concentration: 1.00 ng/mL
Calculated Conc: N/A
Acq. Date: 2025-05-15
Acq. Time: 18:22:42
Modified: Yes
Proc. Algorithm: Specify Parameters - MQ III

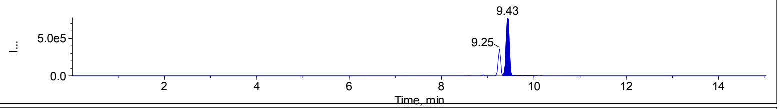

Sample Name: "S4" Sample ID: "" File: "20250515-50AA.wiff"
Peak Name: "Trp" Mass(es): "375.200/171.010 Da"
Comment: "" Annotation: ""
Sample Index: 15
Sample Type: Standard
Concentration: 25.0 umol/L
Calculated Conc: 29.7 umol/L
Acq. Date: 2025-05-15
Acq. Time: 18:22:42
Modified: Yes
Proc. Algorithm: Specify Parameters - MQ III

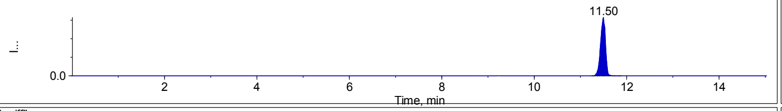

Sample Name: "S4" Sample ID: "" File: "20250515-50AA.wiff"
Peak Name: "MSLAB-Nvl(IS)" Mass(es): "288.150/171.010 Da"
Comment: "" Annotation: ""
Sample Index: 15
Sample Type: Standard
Concentration: 1.00 ng/mL
Calculated Conc: N/A
Acq. Date: 2025-05-15
Acq. Time: 18:22:42
Modified: Yes
Proc. Algorithm: Specify Parameters - MQ III

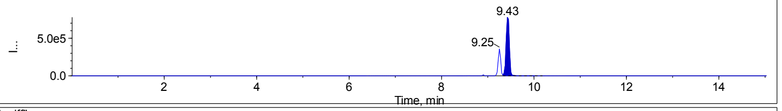

Sample Name: "S4" Sample ID: "" File: "20250515-50AA.wiff"
Peak Name: "Lys" Mass(es): "487.200/171.010 Da"
Comment: "" Annotation: ""
Sample Index: 15
Sample Type: Standard
Concentration: 50.0 umol/L
Calculated Conc: 42.8 umol/L
Acq. Date: 2025-05-15
Acq. Time: 18:22:42
Modified: Yes
Proc. Algorithm: Specify Parameters - MQ III

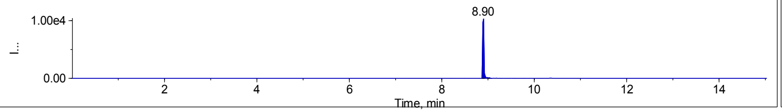

Sample Name: "S4" Sample ID: "" File: "20250515-50AA.wiff"
Peak Name: "MSLAB-Nvl(IS)" Mass(es): "288.150/171.010 Da"
Comment: "" Annotation: ""
Sample Index: 15
Sample Type: Standard
Concentration: 1.00 ng/mL
Calculated Conc: N/A
Acq. Date: 2025-05-15
Acq. Time: 18:22:42
Modified: Yes
Proc. Algorithm: Specify Parameters - MQ III

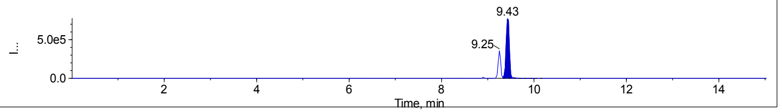

Sample Name: "S4" Sample ID: "" File: "20250515-50AA.wiff"
Peak Name: "tyr" Mass(es): "352.200/171.010 Da"
Comment: "" Annotation: ""
Sample Index: 15
Sample Type: Standard
Concentration: 25.0 umol/L
Calculated Conc: 30.8 umol/L
Acq. Date: 2025-05-15
Acq. Time: 18:22:42
Modified: Yes
Proc. Algorithm: Specify Parameters - MQ III

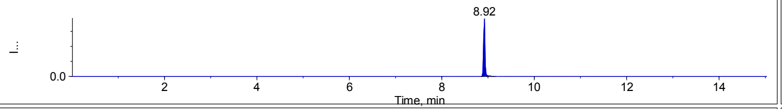

Sample Name: "S4" Sample ID: "" File: "20250515-50AA.wiff"
Peak Name: "MSLAB-Nvl(IS)" Mass(es): "288.150/171.010 Da"
Comment: "" Annotation: ""
Sample Index: 15
Sample Type: Standard
Concentration: 1.00 ng/mL
Calculated Conc: N/A
Acq. Date: 2025-05-15
Acq. Time: 18:22:42
Modified: Yes
Proc. Algorithm: Specify Parameters - MQ III

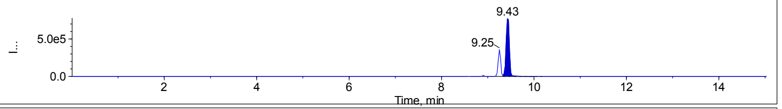

Sample Name: "S4" Sample ID: "" File: "20250515-50AA.wiff"
Peak Name: "Leu" Mass(es): "302.150/171.010 Da"
Comment: "" Annotation: ""
Sample Index: 15
Sample Type: Standard
Concentration: 50.0 umol/L
Calculated Conc: 52.1 umol/L
Acq. Date: 2025-05-15
Acq. Time: 18:22:42
Modified: Yes
Proc. Algorithm: Specify Parameters - MQ III

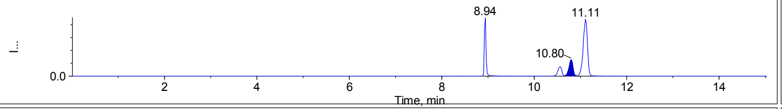

Sample Name: "S4" Sample ID: "" File: "20250515-50AA.wiff"
Peak Name: "MSLAB-Nvl(IS)" Mass(es): "288.150/171.010 Da"
Comment: "" Annotation: ""
Sample Index: 15
Sample Type: Standard
Concentration: 1.00 ng/mL
Calculated Conc: N/A
Acq. Date: 2025-05-15
Acq. Time: 18:22:42
Modified: Yes
Proc. Algorithm: Specify Parameters - MQ III

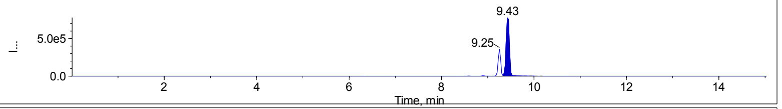

Sample Name: "S5" Sample ID: "" File: "20250515-50AA.wiff"
Peak Name: "Gly" Mass(es): "246.080/171.010 Da"
Comment: "" Annotation: ""
Sample Index: 16
Sample Type: Standard
Concentration: 200. umol/L
Calculated Conc: 199. umol/L
Acq. Date: 2025-05-15
Acq. Time: 18:38:11
Modified: Yes
Proc. Algorithm: Specify Parameters - MQ III

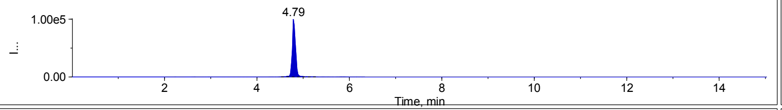

Sample Name: "S5" Sample ID: "" File: "20250515-50AA.wiff"
Peak Name: "MSLAB-Nvl(IS)" Mass(es): "288.150/171.010 Da"
Comment: "" Annotation: ""
Sample Index: 16
Sample Type: Standard
Concentration: 1.00 ng/mL
Calculated Conc: N/A
Acq. Date: 2025-05-15
Acq. Time: 18:38:11
Modified: Yes
Proc. Algorithm: Specify Parameters - MQ III

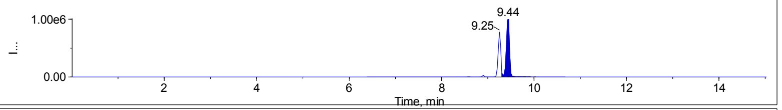

Sample Name: "S5" Sample ID: "" File: "20250515-50AA.wiff"
Peak Name: "Ala" Mass(es): "260.100/171.010 Da"
Comment: "" Annotation: ""
Sample Index: 16
Sample Type: Standard
Concentration: 400. umol/L
Calculated Conc: 420. umol/L
Acq. Date: 2025-05-15
Acq. Time: 18:38:11
Modified: Yes
Proc. Algorithm: Specify Parameters - MQ III

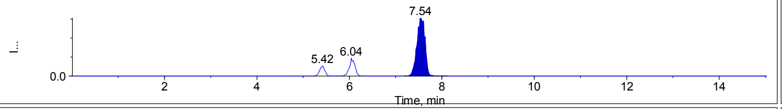

Sample Name: "S5" Sample ID: "" File: "20250515-50AA.wiff"
Peak Name: "MSLAB-Nvl(IS)" Mass(es): "288.150/171.010 Da"
Comment: "" Annotation: ""
Sample Index: 16
Sample Type: Standard
Concentration: 1.00 ng/mL
Calculated Conc: N/A
Acq. Date: 2025-05-15
Acq. Time: 18:38:11
Modified: Yes
Proc. Algorithm: Specify Parameters - MQ III

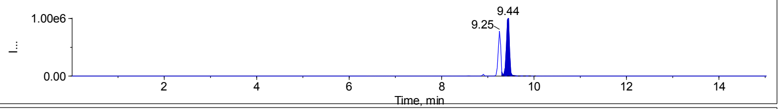

Sample Name: "S5" Sample ID: "" File: "20250515-50AA.wiff"
Peak Name: "Ser" Mass(es): "276.100/171.010 Da"
Comment: "" Annotation: ""
Sample Index: 16
Sample Type: Standard
Concentration: 100. umol/L
Calculated Conc: 97.9 umol/L
Acq. Date: 2025-05-15
Acq. Time: 18:38:11
Modified: Yes
Proc. Algorithm: Specify Parameters - MQ III

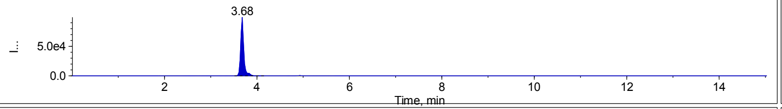

Sample Name: "S5" Sample ID: "" File: "20250515-50AA.wiff"
Peak Name: "MSLAB-Nvl(IS)" Mass(es): "288.150/171.010 Da"
Comment: "" Annotation: ""
Sample Index: 16
Sample Type: Standard
Concentration: 1.00 ng/mL
Calculated Conc: N/A
Acq. Date: 2025-05-15
Acq. Time: 18:38:11
Modified: Yes
Proc. Algorithm: Specify Parameters - MQ III

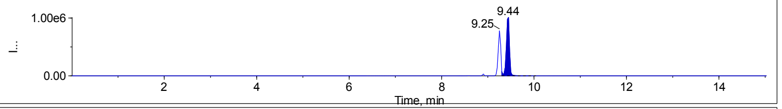

Sample Name: "S5" Sample ID: "" File: "20250515-50AA.wiff"
Peak Name: "Pro" Mass(es): "286.150/171.010 Da"
Comment: "" Annotation: ""
Sample Index: 16
Sample Type: Standard
Concentration: 200. umol/L
Calculated Conc: 210. umol/L
Acq. Date: 2025-05-15
Acq. Time: 18:38:11
Modified: Yes
Proc. Algorithm: Specify Parameters - MQ III

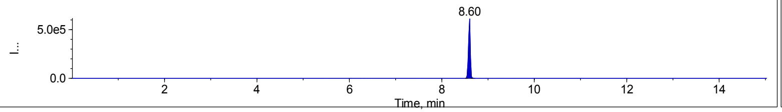

Sample Name: "S5" Sample ID: "" File: "20250515-50AA.wiff"
Peak Name: "MSLAB-Nvl(IS)" Mass(es): "288.150/171.010 Da"
Comment: "" Annotation: ""
Sample Index: 16
Sample Type: Standard
Concentration: 1.00 ng/mL
Calculated Conc: N/A
Acq. Date: 2025-05-15
Acq. Time: 18:38:11
Modified: Yes
Proc. Algorithm: Specify Parameters - MQ III

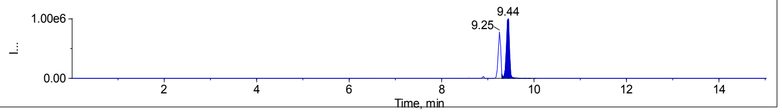

Sample Name: "S5" Sample ID: "" File: "20250515-50AA.wiff"
Peak Name: "Val" Mass(es): "288.150/171.010 Da"
Comment: "" Annotation: ""

Sample Index:	16
Sample Type:	Standard
Concentration:	200. umol/L
Calculated Conc:	239. umol/L
Acq. Date:	2025-05-15
Acq. Time:	18:38:11
Modified:	Yes
Proc. Algorithm:	Specify Parameters - MQ III

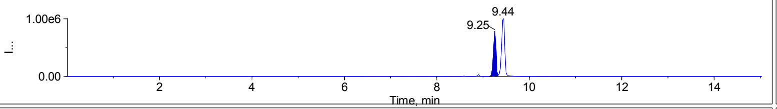

Sample Name: "S5" Sample ID: "" File: "20250515-50AA.wiff"
Peak Name: "MSLAB-Nvl(IS)" Mass(es): "288.150/171.010 Da"
Comment: "" Annotation: ""

Sample Index:	16
Sample Type:	Standard
Concentration:	1.00 ng/mL
Calculated Conc:	N/A
Acq. Date:	2025-05-15
Acq. Time:	18:38:11
Modified:	Yes
Proc. Algorithm:	Specify Parameters - MQ III

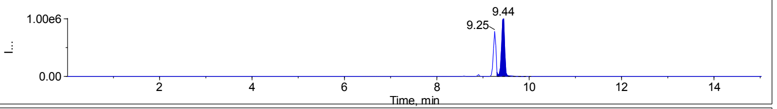

Sample Name: "S5" Sample ID: "" File: "20250515-50AA.wiff"
Peak Name: "Thr" Mass(es): "290.100/171.010 Da"
Comment: "" Annotation: ""

Sample Index:	16
Sample Type:	Standard
Concentration:	100. umol/L
Calculated Conc:	96.0 umol/L
Acq. Date:	2025-05-15
Acq. Time:	18:38:11
Modified:	Yes
Proc. Algorithm:	Specify Parameters - MQ III

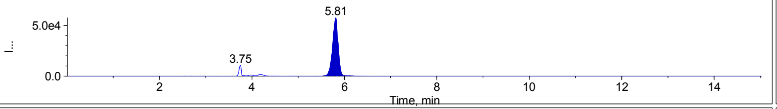

Sample Name: "S5" Sample ID: "" File: "20250515-50AA.wiff"
Peak Name: "MSLAB-Nvl(IS)" Mass(es): "288.150/171.010 Da"
Comment: "" Annotation: ""

Sample Index:	16
Sample Type:	Standard
Concentration:	1.00 ng/mL
Calculated Conc:	N/A
Acq. Date:	2025-05-15
Acq. Time:	18:38:11
Modified:	Yes
Proc. Algorithm:	Specify Parameters - MQ III

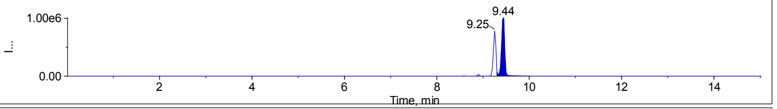

Sample Name: "S5" Sample ID: "" File: "20250515-50AA.wiff"
Peak Name: "Cys1/2" Mass(es): "292.200/171.010 Da"
Comment: "" Annotation: ""

Sample Index:	16
Sample Type:	Standard
Concentration:	50.0 umol/L
Calculated Conc:	50.2 umol/L
Acq. Date:	2025-05-15
Acq. Time:	18:38:11
Modified:	Yes
Proc. Algorithm:	Specify Parameters - MQ III

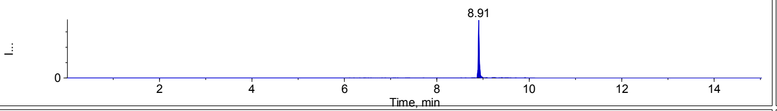

Sample Name: "S5" Sample ID: "" File: "20250515-50AA.wiff"
Peak Name: "MSLAB-Nvl(IS)" Mass(es): "288.150/171.010 Da"
Comment: "" Annotation: ""

Sample Index:	16
Sample Type:	Standard
Concentration:	1.00 ng/mL
Calculated Conc:	N/A
Acq. Date:	2025-05-15
Acq. Time:	18:38:11
Modified:	Yes
Proc. Algorithm:	Specify Parameters - MQ III

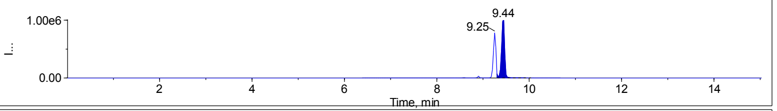

Sample Name: "S5" Sample ID: "" File: "20250515-50AA.wiff"
Peak Name: "Ile" Mass(es): "302.150/171.010 Da"
Comment: "" Annotation: ""

Sample Index:	16
Sample Type:	Standard
Concentration:	50.0 umol/L
Calculated Conc:	49.5 umol/L
Acq. Date:	2025-05-15
Acq. Time:	18:38:11
Modified:	Yes
Proc. Algorithm:	Specify Parameters - MQ III

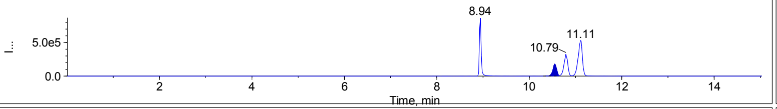

Sample Name: "S5" Sample ID: "" File: "20250515-50AA.wiff"
Peak Name: "MSLAB-Nvl(IS)" Mass(es): "288.150/171.010 Da"
Comment: "" Annotation: ""

Sample Index:	16
Sample Type:	Standard
Concentration:	1.00 ng/mL
Calculated Conc:	N/A
Acq. Date:	2025-05-15
Acq. Time:	18:38:11
Modified:	Yes
Proc. Algorithm:	Specify Parameters - MQ III

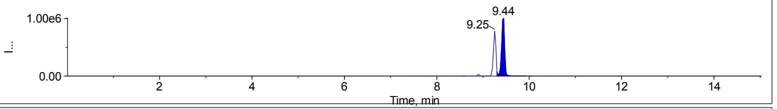

Sample Name: "S5" Sample ID: "" File: "20250515-50AA.wiff"
Peak Name: "Asn" Mass(es): "303.130/171.010 Da"
Comment: "" Annotation: ""

Sample Index:	16
Sample Type:	Standard
Concentration:	50.0 umol/L
Calculated Conc:	48.3 umol/L
Acq. Date:	2025-05-15
Acq. Time:	18:38:11
Modified:	Yes
Proc. Algorithm:	Specify Parameters - MQ III

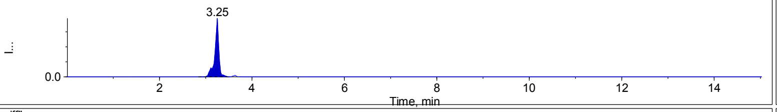

Sample Name: "S5" Sample ID: "" File: "20250515-50AA.wiff"
Peak Name: "MSLAB-Nvl(IS)" Mass(es): "288.150/171.010 Da"
Comment: "" Annotation: ""

Sample Index:	16
Sample Type:	Standard
Concentration:	1.00 ng/mL
Calculated Conc:	N/A
Acq. Date:	2025-05-15
Acq. Time:	18:38:11
Modified:	Yes
Proc. Algorithm:	Specify Parameters - MQ III

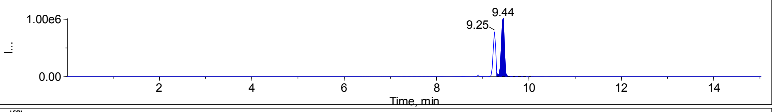

Sample Name: "S5" Sample ID: "" File: "20250515-50AA.wiff"
Peak Name: "Asp" Mass(es): "304.110/171.010 Da"
Comment: "" Annotation: ""

Sample Index:	16
Sample Type:	Standard
Concentration:	50.0 umol/L
Calculated Conc:	49.5 umol/L
Acq. Date:	2025-05-15
Acq. Time:	18:38:11
Modified:	Yes
Proc. Algorithm:	Specify Parameters - MQ III

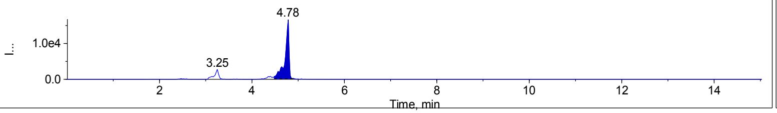

Sample Name: "S5" Sample ID: "" File: "20250515-50AA.wiff"
Peak Name: "MSLAB-Nvl(IS)" Mass(es): "288.150/171.010 Da"
Comment: "" Annotation: ""

Sample Index:	16
Sample Type:	Standard
Concentration:	1.00 ng/mL
Calculated Conc:	N/A
Acq. Date:	2025-05-15
Acq. Time:	18:38:11
Modified:	Yes
Proc. Algorithm:	Specify Parameters - MQ III

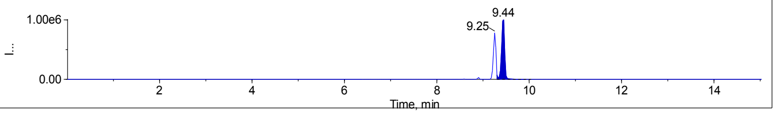

Sample Name: "S5"   Sample ID: ""   File: "20250515-50AA.wiff"
Peak Name: "Gln"   Mass(es): "317.150/171.010 Da"
Comment: ""   Annotation: ""
Sample Index: 16
Sample Type: Standard
Concentration: 400.   umol/L
Calculated Conc: 407.   umol/L
Acq. Date: 2025-05-15
Acq. Time: 18:38:11
Modified: Yes
Proc. Algorithm: Specify Parameters - MQ III

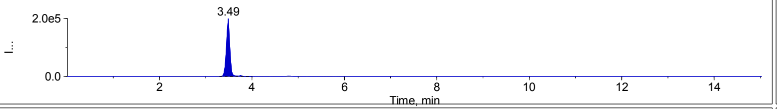

Sample Name: "S5"   Sample ID: ""   File: "20250515-50AA.wiff"
Peak Name: "MSLAB-Nvl(IS)"   Mass(es): "288.150/171.010 Da"
Comment: ""   Annotation: ""
Sample Index: 16
Sample Type: Standard
Concentration: 1.00   ng/mL
Calculated Conc: N/A
Acq. Date: 2025-05-15
Acq. Time: 18:38:11
Modified: Yes
Proc. Algorithm: Specify Parameters - MQ III

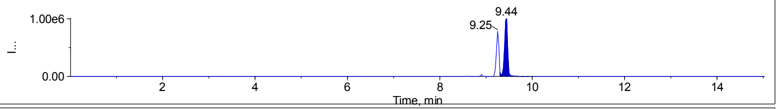

Sample Name: "S5"   Sample ID: ""   File: "20250515-50AA.wiff"
Peak Name: "Glu"   Mass(es): "318.100/171.010 Da"
Comment: ""   Annotation: ""
Sample Index: 16
Sample Type: Standard
Concentration: 50.0   umol/L
Calculated Conc: 47.0   umol/L
Acq. Date: 2025-05-15
Acq. Time: 18:38:11
Modified: Yes
Proc. Algorithm: Specify Parameters - MQ III

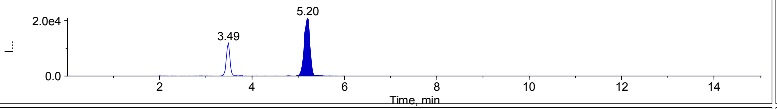

Sample Name: "S5"   Sample ID: ""   File: "20250515-50AA.wiff"
Peak Name: "MSLAB-Nvl(IS)"   Mass(es): "288.150/171.010 Da"
Comment: ""   Annotation: ""
Sample Index: 16
Sample Type: Standard
Concentration: 1.00   ng/mL
Calculated Conc: N/A
Acq. Date: 2025-05-15
Acq. Time: 18:38:11
Modified: Yes
Proc. Algorithm: Specify Parameters - MQ III

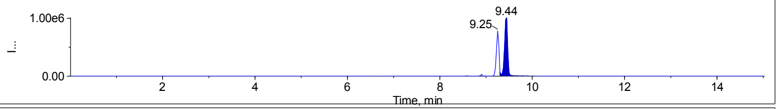

Sample Name: "S5"   Sample ID: ""   File: "20250515-50AA.wiff"
Peak Name: "Met"   Mass(es): "320.210/171.010 Da"
Comment: ""   Annotation: ""
Sample Index: 16
Sample Type: Standard
Concentration: 50.0   umol/L
Calculated Conc: 53.1   umol/L
Acq. Date: 2025-05-15
Acq. Time: 18:38:11
Modified: Yes
Proc. Algorithm: Specify Parameters - MQ III

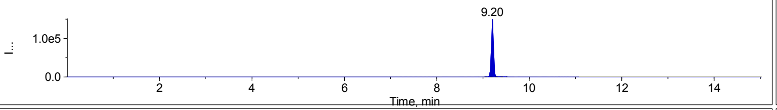

Sample Name: "S5"   Sample ID: ""   File: "20250515-50AA.wiff"
Peak Name: "MSLAB-Nvl(IS)"   Mass(es): "288.150/171.010 Da"
Comment: ""   Annotation: ""
Sample Index: 16
Sample Type: Standard
Concentration: 1.00   ng/mL
Calculated Conc: N/A
Acq. Date: 2025-05-15
Acq. Time: 18:38:11
Modified: Yes
Proc. Algorithm: Specify Parameters - MQ III

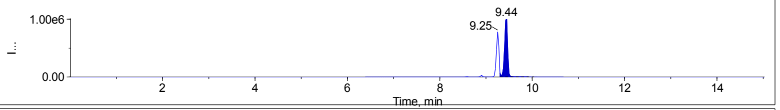

Sample Name: "S5"   Sample ID: ""   File: "20250515-50AA.wiff"
Peak Name: "His"   Mass(es): "326.150/171.010 Da"
Comment: ""   Annotation: ""
Sample Index: 16
Sample Type: Standard
Concentration: 50.0   umol/L
Calculated Conc: 50.1   umol/L
Acq. Date: 2025-05-15
Acq. Time: 18:38:11
Modified: Yes
Proc. Algorithm: Specify Parameters - MQ III

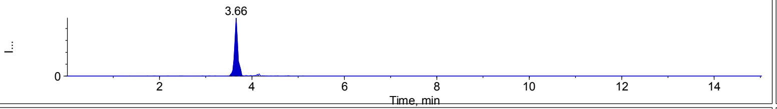

Sample Name: "S5"   Sample ID: ""   File: "20250515-50AA.wiff"
Peak Name: "MSLAB-Nvl(IS)"   Mass(es): "288.150/171.010 Da"
Comment: ""   Annotation: ""
Sample Index: 16
Sample Type: Standard
Concentration: 1.00   ng/mL
Calculated Conc: N/A
Acq. Date: 2025-05-15
Acq. Time: 18:38:11
Modified: Yes
Proc. Algorithm: Specify Parameters - MQ III

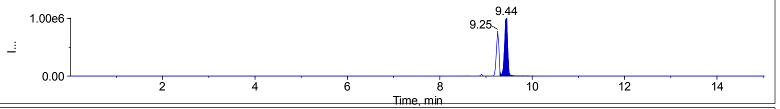

Sample Name: "S5"   Sample ID: ""   File: "20250515-50AA.wiff"
Peak Name: "Phe"   Mass(es): "336.200/171.010 Da"
Comment: ""   Annotation: ""
Sample Index: 16
Sample Type: Standard
Concentration: 50.0   umol/L
Calculated Conc: 50.4   umol/L
Acq. Date: 2025-05-15
Acq. Time: 18:38:11
Modified: Yes
Proc. Algorithm: Specify Parameters - MQ III

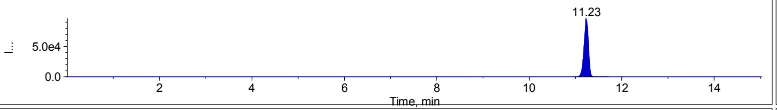

Sample Name: "S5"   Sample ID: ""   File: "20250515-50AA.wiff"
Peak Name: "MSLAB-Nvl(IS)"   Mass(es): "288.150/171.010 Da"
Comment: ""   Annotation: ""
Sample Index: 16
Sample Type: Standard
Concentration: 1.00   ng/mL
Calculated Conc: N/A
Acq. Date: 2025-05-15
Acq. Time: 18:38:11
Modified: Yes
Proc. Algorithm: Specify Parameters - MQ III

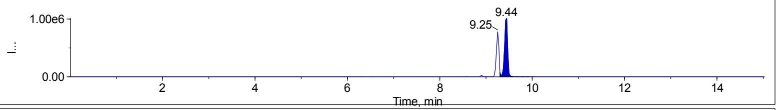

Sample Name: "S5"   Sample ID: ""   File: "20250515-50AA.wiff"
Peak Name: "Arg"   Mass(es): "345.210/171.010 Da"
Comment: ""   Annotation: ""
Sample Index: 16
Sample Type: Standard
Concentration: 100.   umol/L
Calculated Conc: 92.2   umol/L
Acq. Date: 2025-05-15
Acq. Time: 18:38:11
Modified: Yes
Proc. Algorithm: Specify Parameters - MQ III

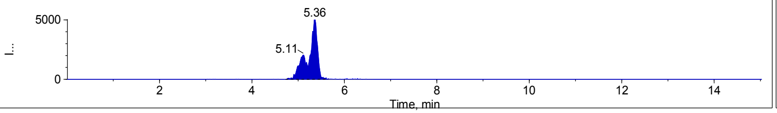

Sample Name: "S5"   Sample ID: ""   File: "20250515-50AA.wiff"
Peak Name: "MSLAB-Nvl(IS)"   Mass(es): "288.150/171.010 Da"
Comment: ""   Annotation: ""
Sample Index: 16
Sample Type: Standard
Concentration: 1.00   ng/mL
Calculated Conc: N/A
Acq. Date: 2025-05-15
Acq. Time: 18:38:11
Modified: Yes
Proc. Algorithm: Specify Parameters - MQ III

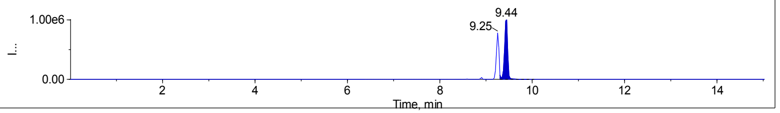

Sample Name: "S5" Sample ID: "" File: "20250515-50AA.wiff"
Peak Name: "Trp" Mass(es): "375.200/171.010 Da"
Comment: "" Annotation: ""

Sample Index: 16
Sample Type: Standard
Concentration: 50.0 umol/L
Calculated Conc: 51.4 umol/L
Acq. Date: 2025-05-15
Acq. Time: 18:38:11

Modified: Yes
Proc. Algorithm: Specify Parameters - MQ III

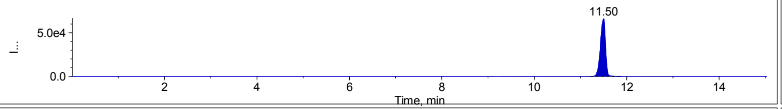

Sample Name: "S5" Sample ID: "" File: "20250515-50AA.wiff"
Peak Name: "MSLAB-Nvl(IS)" Mass(es): "288.150/171.010 Da"
Comment: "" Annotation: ""

Sample Index: 16
Sample Type: Standard
Concentration: 1.00 ng/mL
Calculated Conc: N/A
Acq. Date: 2025-05-15
Acq. Time: 18:38:11

Modified: Yes
Proc. Algorithm: Specify Parameters - MQ III

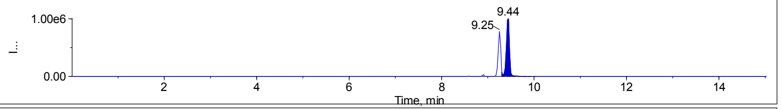

Sample Name: "S5" Sample ID: "" File: "20250515-50AA.wiff"
Peak Name: "Lys" Mass(es): "487.200/171.010 Da"
Comment: "" Annotation: ""

Sample Index: 16
Sample Type: Standard
Concentration: 100. umol/L
Calculated Conc: 99.2 umol/L
Acq. Date: 2025-05-15
Acq. Time: 18:38:11

Modified: Yes
Proc. Algorithm: Specify Parameters - MQ III

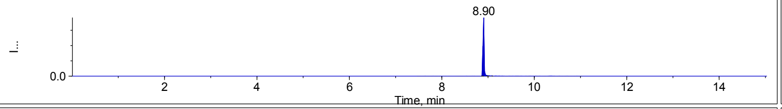

Sample Name: "S5" Sample ID: "" File: "20250515-50AA.wiff"
Peak Name: "MSLAB-Nvl(IS)" Mass(es): "288.150/171.010 Da"
Comment: "" Annotation: ""

Sample Index: 16
Sample Type: Standard
Concentration: 1.00 ng/mL
Calculated Conc: N/A
Acq. Date: 2025-05-15
Acq. Time: 18:38:11

Modified: Yes
Proc. Algorithm: Specify Parameters - MQ III

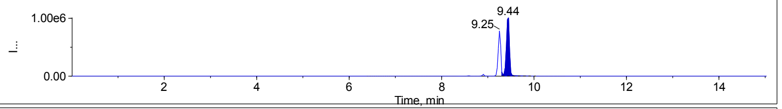

Sample Name: "S5" Sample ID: "" File: "20250515-50AA.wiff"
Peak Name: "tyr" Mass(es): "352.200/171.010 Da"
Comment: "" Annotation: ""

Sample Index: 16
Sample Type: Standard
Concentration: 50.0 umol/L
Calculated Conc: 48.8 umol/L
Acq. Date: 2025-05-15
Acq. Time: 18:38:11

Modified: Yes
Proc. Algorithm: Specify Parameters - MQ III

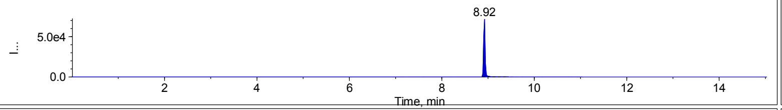

Sample Name: "S5" Sample ID: "" File: "20250515-50AA.wiff"
Peak Name: "MSLAB-Nvl(IS)" Mass(es): "288.150/171.010 Da"
Comment: "" Annotation: ""

Sample Index: 16
Sample Type: Standard
Concentration: 1.00 ng/mL
Calculated Conc: N/A
Acq. Date: 2025-05-15
Acq. Time: 18:38:11

Modified: Yes
Proc. Algorithm: Specify Parameters - MQ III

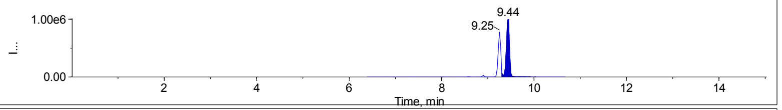

Sample Name: "S5" Sample ID: "" File: "20250515-50AA.wiff"
Peak Name: "Leu" Mass(es): "302.150/171.010 Da"
Comment: "" Annotation: ""

Sample Index: 16
Sample Type: Standard
Concentration: 100. umol/L
Calculated Conc: 101. umol/L
Acq. Date: 2025-05-15
Acq. Time: 18:38:11

Modified: Yes
Proc. Algorithm: Specify Parameters - MQ III

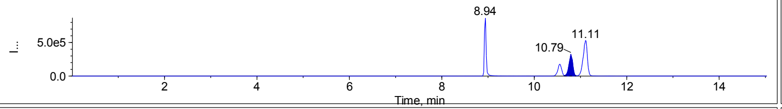

Sample Name: "S5" Sample ID: "" File: "20250515-50AA.wiff"
Peak Name: "MSLAB-Nvl(IS)" Mass(es): "288.150/171.010 Da"
Comment: "" Annotation: ""

Sample Index: 16
Sample Type: Standard
Concentration: 1.00 ng/mL
Calculated Conc: N/A
Acq. Date: 2025-05-15
Acq. Time: 18:38:11

Modified: Yes
Proc. Algorithm: Specify Parameters - MQ III

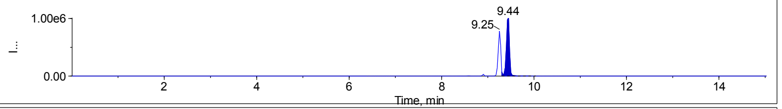

Sample Name: "S6" Sample ID: "" File: "20250515-50AA.wiff"
Peak Name: "Gly" Mass(es): "246.080/171.010 Da"
Comment: "" Annotation: ""

Sample Index: 17
Sample Type: Standard
Concentration: 400. umol/L
Calculated Conc: 401. umol/L
Acq. Date: 2025-05-15
Acq. Time: 18:53:37

Modified: Yes
Proc. Algorithm: Specify Parameters - MQ III

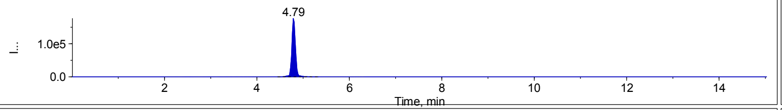

Sample Name: "S6" Sample ID: "" File: "20250515-50AA.wiff"
Peak Name: "MSLAB-Nvl(IS)" Mass(es): "288.150/171.010 Da"
Comment: "" Annotation: ""

Sample Index: 17
Sample Type: Standard
Concentration: 1.00 ng/mL
Calculated Conc: N/A
Acq. Date: 2025-05-15
Acq. Time: 18:53:37

Modified: Yes
Proc. Algorithm: Specify Parameters - MQ III

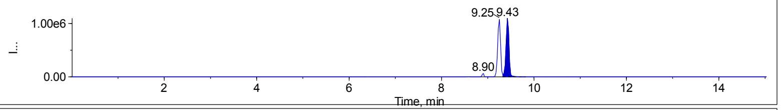

Sample Name: "S6" Sample ID: "" File: "20250515-50AA.wiff"
Peak Name: "Ala" Mass(es): "260.100/171.010 Da"
Comment: "" Annotation: ""

Sample Index: 17
Sample Type: Standard
Concentration: 800. umol/L
Calculated Conc: 745. umol/L
Acq. Date: 2025-05-15
Acq. Time: 18:53:37

Modified: Yes
Proc. Algorithm: Specify Parameters - MQ III

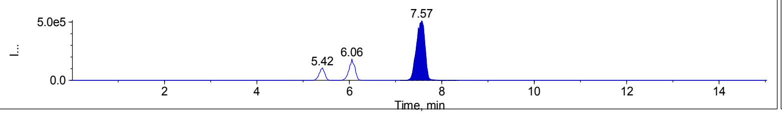

Sample Name: "S6" Sample ID: "" File: "20250515-50AA.wiff"
Peak Name: "MSLAB-Nvl(IS)" Mass(es): "288.150/171.010 Da"
Comment: "" Annotation: ""

Sample Index: 17
Sample Type: Standard
Concentration: 1.00 ng/mL
Calculated Conc: N/A
Acq. Date: 2025-05-15
Acq. Time: 18:53:37

Modified: Yes
Proc. Algorithm: Specify Parameters - MQ III

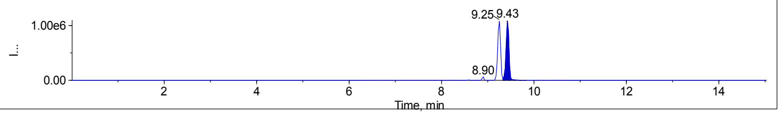

Sample Name: "S6" Sample ID: "" File: "20250515-50AA.wiff"
Peak Name: "Ser" Mass(es): "276.100/171.010 Da"
Comment: "" Annotation: ""

Sample Index: 17
Sample Type: Standard
Concentration: 200. umol/L
Calculated Conc: 204. umol/L
Acq. Date: 2025-05-15
Acq. Time: 18:53:37

Modified: Yes
Proc. Algorithm: Specify Parameters - MQ III

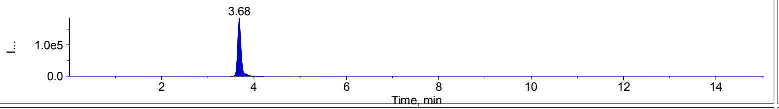

Sample Name: "S6" Sample ID: "" File: "20250515-50AA.wiff"
Peak Name: "MSLAB-Nvl(IS)" Mass(es): "288.150/171.010 Da"
Comment: "" Annotation: ""

Sample Index: 17
Sample Type: Standard
Concentration: 1.00 ng/mL
Calculated Conc: N/A
Acq. Date: 2025-05-15
Acq. Time: 18:53:37

Modified: Yes
Proc. Algorithm: Specify Parameters - MQ III

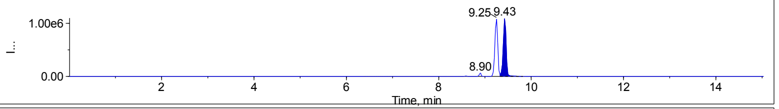

Sample Name: "S6" Sample ID: "" File: "20250515-50AA.wiff"
Peak Name: "Pro" Mass(es): "286.150/171.010 Da"
Comment: "" Annotation: ""

Sample Index: 17
Sample Type: Standard
Concentration: 400. umol/L
Calculated Conc: 366. umol/L
Acq. Date: 2025-05-15
Acq. Time: 18:53:37

Modified: Yes
Proc. Algorithm: Specify Parameters - MQ III

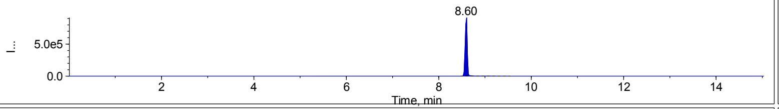

Sample Name: "S6" Sample ID: "" File: "20250515-50AA.wiff"
Peak Name: "MSLAB-Nvl(IS)" Mass(es): "288.150/171.010 Da"
Comment: "" Annotation: ""

Sample Index: 17
Sample Type: Standard
Concentration: 1.00 ng/mL
Calculated Conc: N/A
Acq. Date: 2025-05-15
Acq. Time: 18:53:37

Modified: Yes
Proc. Algorithm: Specify Parameters - MQ III

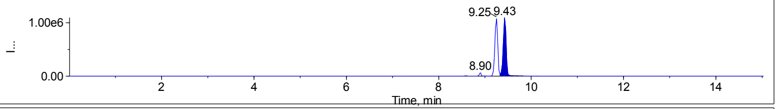

Sample Name: "S6" Sample ID: "" File: "20250515-50AA.wiff"
Peak Name: "Val" Mass(es): "288.150/171.010 Da"
Comment: "" Annotation: ""

Sample Index: 17
Sample Type: Standard
Concentration: 400. umol/L
Calculated Conc: 369. umol/L
Acq. Date: 2025-05-15
Acq. Time: 18:53:37

Modified: Yes
Proc. Algorithm: Specify Parameters - MQ III

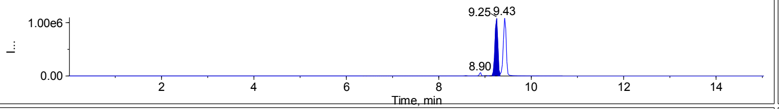

Sample Name: "S6" Sample ID: "" File: "20250515-50AA.wiff"
Peak Name: "MSLAB-Nvl(IS)" Mass(es): "288.150/171.010 Da"
Comment: "" Annotation: ""

Sample Index: 17
Sample Type: Standard
Concentration: 1.00 ng/mL
Calculated Conc: N/A
Acq. Date: 2025-05-15
Acq. Time: 18:53:37

Modified: Yes
Proc. Algorithm: Specify Parameters - MQ III

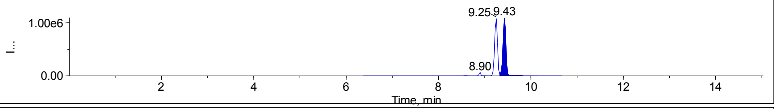

Sample Name: "S6" Sample ID: "" File: "20250515-50AA.wiff"
Peak Name: "Thr" Mass(es): "290.100/171.010 Da"
Comment: "" Annotation: ""

Sample Index: 17
Sample Type: Standard
Concentration: 200. umol/L
Calculated Conc: 204. umol/L
Acq. Date: 2025-05-15
Acq. Time: 18:53:37

Modified: Yes
Proc. Algorithm: Specify Parameters - MQ III

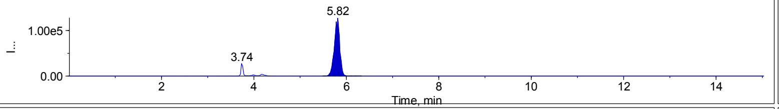

Sample Name: "S6" Sample ID: "" File: "20250515-50AA.wiff"
Peak Name: "MSLAB-Nvl(IS)" Mass(es): "288.150/171.010 Da"
Comment: "" Annotation: ""

Sample Index: 17
Sample Type: Standard
Concentration: 1.00 ng/mL
Calculated Conc: N/A
Acq. Date: 2025-05-15
Acq. Time: 18:53:37

Modified: Yes
Proc. Algorithm: Specify Parameters - MQ III

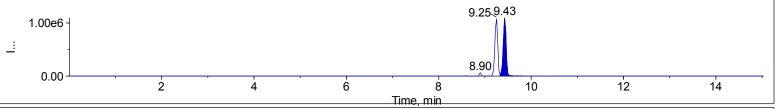

Sample Name: "S6" Sample ID: "" File: "20250515-50AA.wiff"
Peak Name: "Cys1/2" Mass(es): "292.200/171.010 Da"
Comment: "" Annotation: ""

Sample Index: 17
Sample Type: Standard
Concentration: 100. umol/L
Calculated Conc: 102. umol/L
Acq. Date: 2025-05-15
Acq. Time: 18:53:37

Modified: Yes
Proc. Algorithm: Specify Parameters - MQ III

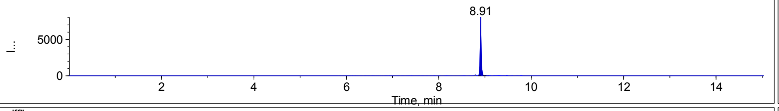

Sample Name: "S6" Sample ID: "" File: "20250515-50AA.wiff"
Peak Name: "MSLAB-Nvl(IS)" Mass(es): "288.150/171.010 Da"
Comment: "" Annotation: ""

Sample Index: 17
Sample Type: Standard
Concentration: 1.00 ng/mL
Calculated Conc: N/A
Acq. Date: 2025-05-15
Acq. Time: 18:53:37

Modified: Yes
Proc. Algorithm: Specify Parameters - MQ III

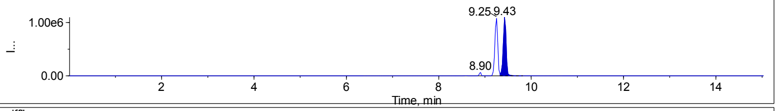

Sample Name: "S6" Sample ID: "" File: "20250515-50AA.wiff"
Peak Name: "Ile" Mass(es): "302.150/171.010 Da"
Comment: "" Annotation: ""

Sample Index: 17
Sample Type: Standard
Concentration: 100. umol/L
Calculated Conc: 99.7 umol/L
Acq. Date: 2025-05-15
Acq. Time: 18:53:37

Modified: Yes
Proc. Algorithm: Specify Parameters - MQ III

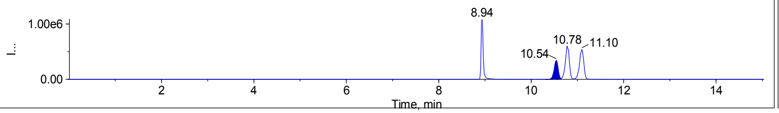

Sample Name: "S6" Sample ID: "" File: "20250515-50AA.wiff"
Peak Name: "MSLAB-Nvl(IS)" Mass(es): "288.150/171.010 Da"
Comment: "" Annotation: ""

Sample Index: 17
Sample Type: Standard
Concentration: 1.00 ng/mL
Calculated Conc: N/A
Acq. Date: 2025-05-15
Acq. Time: 18:53:37

Modified: Yes
Proc. Algorithm: Specify Parameters - MQ III

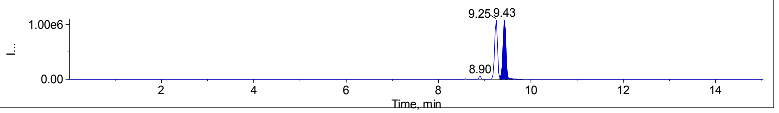

Sample Name: "S6" Sample ID: "" File: "20250515-50AA.wiff"
Peak Name: "Asn" Mass(es): "303.130/171.010 Da"
Comment: "" Annotation: ""

Sample Index: 17
Sample Type: Standard
Concentration: 100. umol/L
Calculated Conc: 101. umol/L
Acq. Date: 2025-05-15
Acq. Time: 18:53:37

Modified: Yes
Proc. Algorithm: Specify Parameters - MQ III

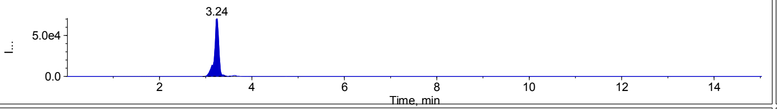

Sample Name: "S6" Sample ID: "" File: "20250515-50AA.wiff"
Peak Name: "Asp" Mass(es): "304.110/171.010 Da"
Comment: "" Annotation: ""

Sample Index: 17
Sample Type: Standard
Concentration: 100. umol/L
Calculated Conc: 100. umol/L
Acq. Date: 2025-05-15
Acq. Time: 18:53:37

Modified: Yes
Proc. Algorithm: Specify Parameters - MQ III

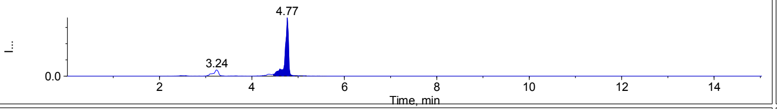

Sample Name: "S6" Sample ID: "" File: "20250515-50AA.wiff"
Peak Name: "Gln" Mass(es): "317.150/171.010 Da"
Comment: "" Annotation: ""

Sample Index: 17
Sample Type: Standard
Concentration: 800. umol/L
Calculated Conc: 796. umol/L
Acq. Date: 2025-05-15
Acq. Time: 18:53:37

Modified: Yes
Proc. Algorithm: Specify Parameters - MQ III

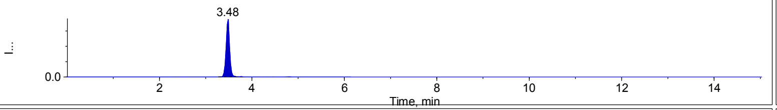

Sample Name: "S6" Sample ID: "" File: "20250515-50AA.wiff"
Peak Name: "Glu" Mass(es): "318.100/171.010 Da"
Comment: "" Annotation: ""

Sample Index: 17
Sample Type: Standard
Concentration: 100. umol/L
Calculated Conc: 103. umol/L
Acq. Date: 2025-05-15
Acq. Time: 18:53:37

Modified: Yes
Proc. Algorithm: Specify Parameters - MQ III

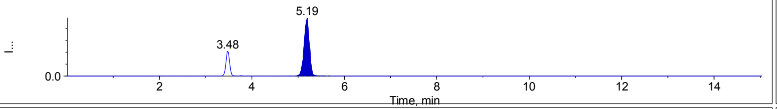

Sample Name: "S6" Sample ID: "" File: "20250515-50AA.wiff"
Peak Name: "Met" Mass(es): "320.210/171.010 Da"
Comment: "" Annotation: ""

Sample Index: 17
Sample Type: Standard
Concentration: 100. umol/L
Calculated Conc: 95.8 umol/L
Acq. Date: 2025-05-15
Acq. Time: 18:53:37

Modified: Yes
Proc. Algorithm: Specify Parameters - MQ III

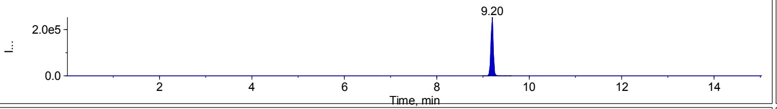

Sample Name: "S6" Sample ID: "" File: "20250515-50AA.wiff"
Peak Name: "His" Mass(es): "326.150/171.010 Da"
Comment: "" Annotation: ""

Sample Index: 17
Sample Type: Standard
Concentration: 100. umol/L
Calculated Conc: 101. umol/L
Acq. Date: 2025-05-15
Acq. Time: 18:53:37

Modified: Yes
Proc. Algorithm: Specify Parameters - MQ III

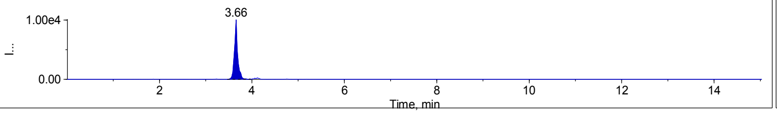

Sample Name: "S6" Sample ID: "" File: "20250515-50AA.wiff"
Peak Name: "MSLAB-Nvl(IS)" Mass(es): "288.150/171.010 Da"
Comment: "" Annotation: ""

Sample Index: 17
Sample Type: Standard
Concentration: 1.00 ng/mL
Calculated Conc: N/A
Acq. Date: 2025-05-15
Acq. Time: 18:53:37

Modified: Yes
Proc. Algorithm: Specify Parameters - MQ III

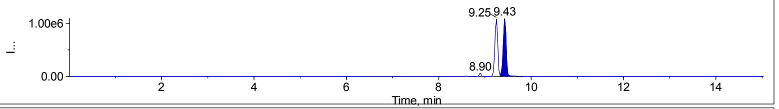

Sample Name: "S6" Sample ID: "" File: "20250515-50AA.wiff"
Peak Name: "MSLAB-Nvl(IS)" Mass(es): "288.150/171.010 Da"
Comment: "" Annotation: ""

Sample Index: 17
Sample Type: Standard
Concentration: 1.00 ng/mL
Calculated Conc: N/A
Acq. Date: 2025-05-15
Acq. Time: 18:53:37

Modified: Yes
Proc. Algorithm: Specify Parameters - MQ III

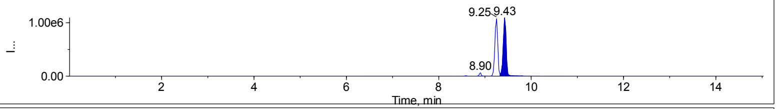

Sample Name: "S6" Sample ID: "" File: "20250515-50AA.wiff"
Peak Name: "MSLAB-Nvl(IS)" Mass(es): "288.150/171.010 Da"
Comment: "" Annotation: ""

Sample Index: 17
Sample Type: Standard
Concentration: 1.00 ng/mL
Calculated Conc: N/A
Acq. Date: 2025-05-15
Acq. Time: 18:53:37

Modified: Yes
Proc. Algorithm: Specify Parameters - MQ III

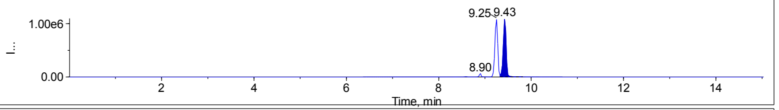

Sample Name: "S6" Sample ID: "" File: "20250515-50AA.wiff"
Peak Name: "MSLAB-Nvl(IS)" Mass(es): "288.150/171.010 Da"
Comment: "" Annotation: ""

Sample Index: 17
Sample Type: Standard
Concentration: 1.00 ng/mL
Calculated Conc: N/A
Acq. Date: 2025-05-15
Acq. Time: 18:53:37

Modified: Yes
Proc. Algorithm: Specify Parameters - MQ III

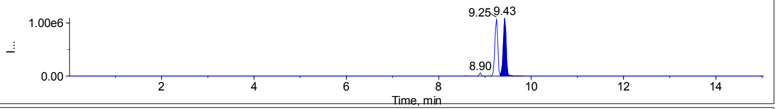

Sample Name: "S6" Sample ID: "" File: "20250515-50AA.wiff"
Peak Name: "MSLAB-Nvl(IS)" Mass(es): "288.150/171.010 Da"
Comment: "" Annotation: ""

Sample Index: 17
Sample Type: Standard
Concentration: 1.00 ng/mL
Calculated Conc: N/A
Acq. Date: 2025-05-15
Acq. Time: 18:53:37

Modified: Yes
Proc. Algorithm: Specify Parameters - MQ III

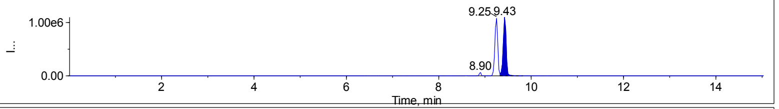

Sample Name: "S6" Sample ID: "" File: "20250515-50AA.wiff"
Peak Name: "MSLAB-Nvl(IS)" Mass(es): "288.150/171.010 Da"
Comment: "" Annotation: ""

Sample Index: 17
Sample Type: Standard
Concentration: 1.00 ng/mL
Calculated Conc: N/A
Acq. Date: 2025-05-15
Acq. Time: 18:53:37

Modified: Yes
Proc. Algorithm: Specify Parameters - MQ III

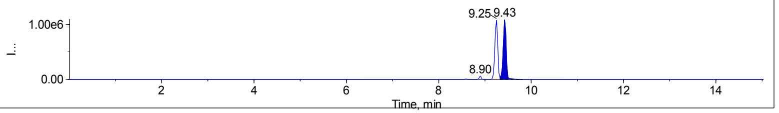

Sample Name: "S6"   Sample ID: ""   File: "20250515-50AA.wiff"
Peak Name: "Phe"   Mass(es): "336.200/171.010 Da"
Comment: ""   Annotation: ""

```
Sample Index:      17
Sample Type:       Standard
Concentration:     100.    umol/L
Calculated Conc:   96.9    umol/L
Acq. Date:         2025-05-15
Acq. Time:         18:53:37
Modified:          Yes
Proc. Algorithm: Specify Parameters - MQ III
```

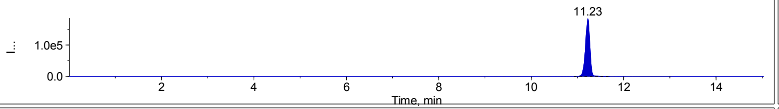

Sample Name: "S6"   Sample ID: ""   File: "20250515-50AA.wiff"
Peak Name: "MSLAB-Nvl(IS)"   Mass(es): "288.150/171.010 Da"
Comment: ""   Annotation: ""

```
Sample Index:      17
Sample Type:       Standard
Concentration:     1.00    ng/mL
Calculated Conc:   N/A
Acq. Date:         2025-05-15
Acq. Time:         18:53:37
Modified:          Yes
Proc. Algorithm: Specify Parameters - MQ III
```

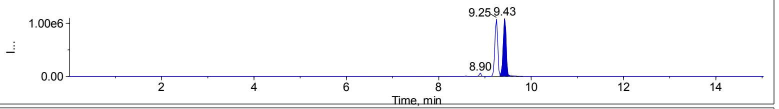

Sample Name: "S6"   Sample ID: ""   File: "20250515-50AA.wiff"
Peak Name: "Arg"   Mass(es): "345.210/171.010 Da"
Comment: ""   Annotation: ""

```
Sample Index:      17
Sample Type:       Standard
Concentration:     200.    umol/L
Calculated Conc:   212.    umol/L
Acq. Date:         2025-05-15
Acq. Time:         18:53:37
Modified:          Yes
Proc. Algorithm: Specify Parameters - MQ III
```

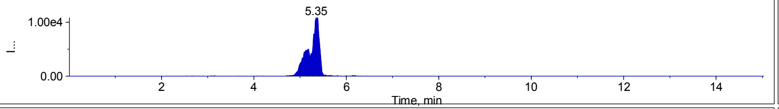

Sample Name: "S6"   Sample ID: ""   File: "20250515-50AA.wiff"
Peak Name: "MSLAB-Nvl(IS)"   Mass(es): "288.150/171.010 Da"
Comment: ""   Annotation: ""

```
Sample Index:      17
Sample Type:       Standard
Concentration:     1.00    ng/mL
Calculated Conc:   N/A
Acq. Date:         2025-05-15
Acq. Time:         18:53:37
Modified:          Yes
Proc. Algorithm: Specify Parameters - MQ III
```

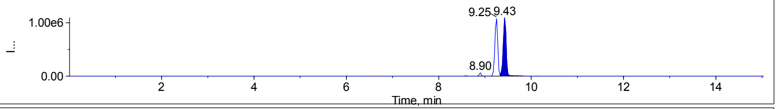

Sample Name: "S6"   Sample ID: ""   File: "20250515-50AA.wiff"
Peak Name: "Trp"   Mass(es): "375.200/171.010 Da"
Comment: ""   Annotation: ""

```
Sample Index:      17
Sample Type:       Standard
Concentration:     100.    umol/L
Calculated Conc:   94.4    umol/L
Acq. Date:         2025-05-15
Acq. Time:         18:53:37
Modified:          Yes
Proc. Algorithm: Specify Parameters - MQ III
```

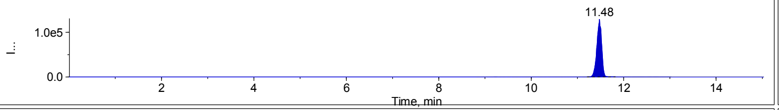

Sample Name: "S6"   Sample ID: ""   File: "20250515-50AA.wiff"
Peak Name: "MSLAB-Nvl(IS)"   Mass(es): "288.150/171.010 Da"
Comment: ""   Annotation: ""

```
Sample Index:      17
Sample Type:       Standard
Concentration:     1.00    ng/mL
Calculated Conc:   N/A
Acq. Date:         2025-05-15
Acq. Time:         18:53:37
Modified:          Yes
Proc. Algorithm: Specify Parameters - MQ III
```

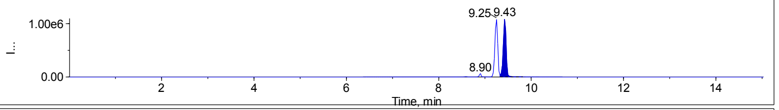

Sample Name: "S6"   Sample ID: ""   File: "20250515-50AA.wiff"
Peak Name: "Lys"   Mass(es): "487.200/171.010 Da"
Comment: ""   Annotation: ""

```
Sample Index:      17
Sample Type:       Standard
Concentration:     200.    umol/L
Calculated Conc:   206.    umol/L
Acq. Date:         2025-05-15
Acq. Time:         18:53:37
Modified:          Yes
Proc. Algorithm: Specify Parameters - MQ III
```

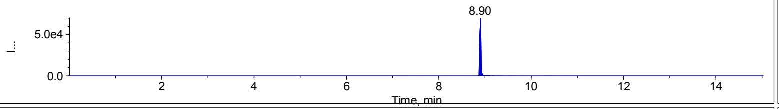

Sample Name: "S6"   Sample ID: ""   File: "20250515-50AA.wiff"
Peak Name: "MSLAB-Nvl(IS)"   Mass(es): "288.150/171.010 Da"
Comment: ""   Annotation: ""

```
Sample Index:      17
Sample Type:       Standard
Concentration:     1.00    ng/mL
Calculated Conc:   N/A
Acq. Date:         2025-05-15
Acq. Time:         18:53:37
Modified:          Yes
Proc. Algorithm: Specify Parameters - MQ III
```

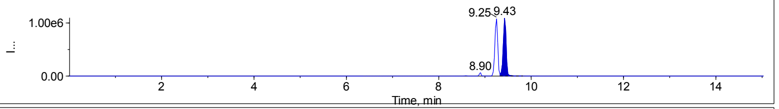

Sample Name: "S6"   Sample ID: ""   File: "20250515-50AA.wiff"
Peak Name: "tyr"   Mass(es): "352.200/171.010 Da"
Comment: ""   Annotation: ""

```
Sample Index:      17
Sample Type:       Standard
Concentration:     100.    umol/L
Calculated Conc:   96.3    umol/L
Acq. Date:         2025-05-15
Acq. Time:         18:53:37
Modified:          Yes
Proc. Algorithm: Specify Parameters - MQ III
```

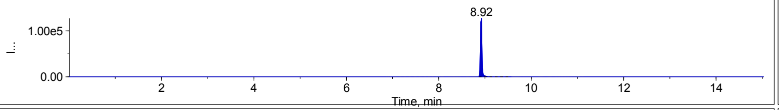

Sample Name: "S6"   Sample ID: ""   File: "20250515-50AA.wiff"
Peak Name: "MSLAB-Nvl(IS)"   Mass(es): "288.150/171.010 Da"
Comment: ""   Annotation: ""

```
Sample Index:      17
Sample Type:       Standard
Concentration:     1.00    ng/mL
Calculated Conc:   N/A
Acq. Date:         2025-05-15
Acq. Time:         18:53:37
Modified:          Yes
Proc. Algorithm: Specify Parameters - MQ III
```

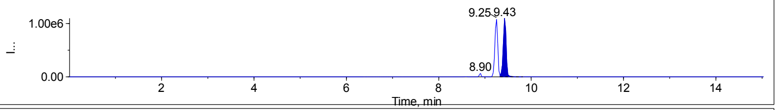

Sample Name: "S6"   Sample ID: ""   File: "20250515-50AA.wiff"
Peak Name: "Leu"   Mass(es): "302.150/171.010 Da"
Comment: ""   Annotation: ""

```
Sample Index:      17
Sample Type:       Standard
Concentration:     200.    umol/L
Calculated Conc:   197.    umol/L
Acq. Date:         2025-05-15
Acq. Time:         18:53:37
Modified:          Yes
Proc. Algorithm: Specify Parameters - MQ III
```

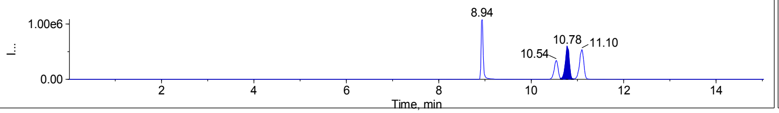

Sample Name: "S6"   Sample ID: ""   File: "20250515-50AA.wiff"
Peak Name: "MSLAB-Nvl(IS)"   Mass(es): "288.150/171.010 Da"
Comment: ""   Annotation: ""

```
Sample Index:      17
Sample Type:       Standard
Concentration:     1.00    ng/mL
Calculated Conc:   N/A
Acq. Date:         2025-05-15
Acq. Time:         18:53:37
Modified:          Yes
Proc. Algorithm: Specify Parameters - MQ III
```

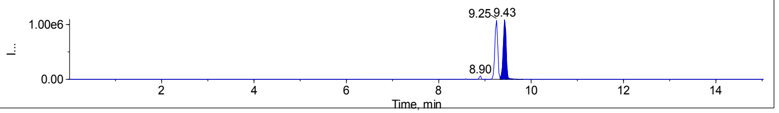

Sample Name: "ZW-1" Sample ID: "" File: "20250515-50AA.wiff"
Peak Name: "Gly" Mass(es): "246.080/171.010 Da"
Comment: "" Annotation: ""
Sample Index: 36
Sample Type: Unknown
Concentration: N/A
Calculated Conc: 9.18 umol/L
Acq. Date: 2025-05-15
Acq. Time: 23:48:08
Modified: Yes
Proc. Algorithm: Specify Parameters - MQ III

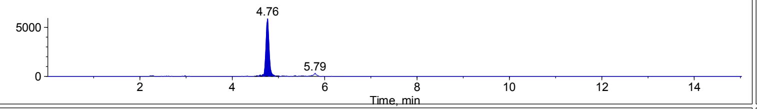

Sample Name: "ZW-1" Sample ID: "" File: "20250515-50AA.wiff"
Peak Name: "MSLAB-Nvl(IS)" Mass(es): "288.150/171.010 Da"
Comment: "" Annotation: ""
Sample Index: 36
Sample Type: Unknown
Concentration: 1.00 ng/mL
Calculated Conc: N/A
Acq. Date: 2025-05-15
Acq. Time: 23:48:08
Modified: Yes
Proc. Algorithm: Specify Parameters - MQ III

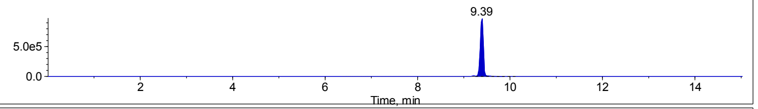

Sample Name: "ZW-1" Sample ID: "" File: "20250515-50AA.wiff"
Peak Name: "Ala" Mass(es): "260.100/171.010 Da"
Comment: "" Annotation: ""
Sample Index: 36
Sample Type: Unknown
Concentration: N/A
Calculated Conc: No Intercept
Acq. Date: 2025-05-15
Acq. Time: 23:48:08
Modified: Yes
Proc. Algorithm: Specify Parameters - MQ III

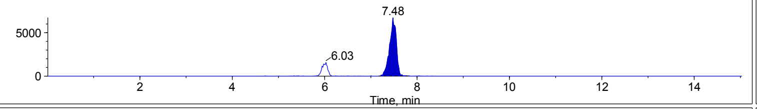

Sample Name: "ZW-1" Sample ID: "" File: "20250515-50AA.wiff"
Peak Name: "MSLAB-Nvl(IS)" Mass(es): "288.150/171.010 Da"
Comment: "" Annotation: ""
Sample Index: 36
Sample Type: Unknown
Concentration: 1.00 ng/mL
Calculated Conc: N/A
Acq. Date: 2025-05-15
Acq. Time: 23:48:08
Modified: Yes
Proc. Algorithm: Specify Parameters - MQ III

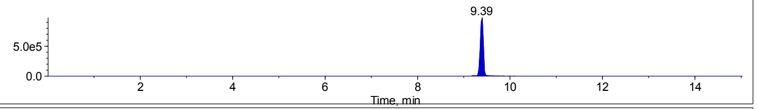

Sample Name: "ZW-1" Sample ID: "" File: "20250515-50AA.wiff"
Peak Name: "Ser" Mass(es): "276.100/171.010 Da"
Comment: "" Annotation: ""
Sample Index: 36
Sample Type: Unknown
Concentration: N/A
Calculated Conc: 0.336 umol/L
Acq. Date: 2025-05-15
Acq. Time: 23:48:08
Modified: Yes
Proc. Algorithm: Specify Parameters - MQ III

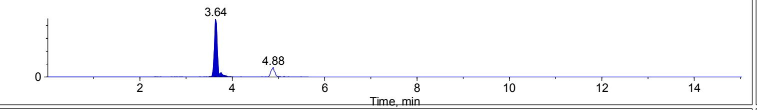

Sample Name: "ZW-1" Sample ID: "" File: "20250515-50AA.wiff"
Peak Name: "MSLAB-Nvl(IS)" Mass(es): "288.150/171.010 Da"
Comment: "" Annotation: ""
Sample Index: 36
Sample Type: Unknown
Concentration: 1.00 ng/mL
Calculated Conc: N/A
Acq. Date: 2025-05-15
Acq. Time: 23:48:08
Modified: Yes
Proc. Algorithm: Specify Parameters - MQ III

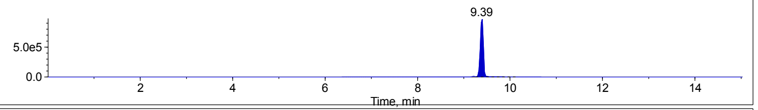

Sample Name: "ZW-1" Sample ID: "" File: "20250515-50AA.wiff"
Peak Name: "Pro" Mass(es): "286.150/171.010 Da"
Comment: "" Annotation: ""
Sample Index: 36
Sample Type: Unknown
Concentration: N/A
Calculated Conc: 0.416 umol/L
Acq. Date: 2025-05-15
Acq. Time: 23:48:08
Modified: Yes
Proc. Algorithm: Specify Parameters - MQ III

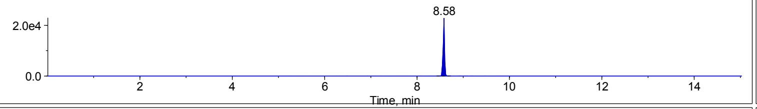

Sample Name: "ZW-1" Sample ID: "" File: "20250515-50AA.wiff"
Peak Name: "MSLAB-Nvl(IS)" Mass(es): "288.150/171.010 Da"
Comment: "" Annotation: ""
Sample Index: 36
Sample Type: Unknown
Concentration: 1.00 ng/mL
Calculated Conc: N/A
Acq. Date: 2025-05-15
Acq. Time: 23:48:08
Modified: Yes
Proc. Algorithm: Specify Parameters - MQ III

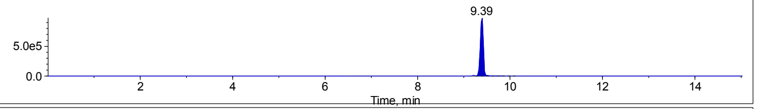

Sample Name: "ZW-1" Sample ID: "" File: "20250515-50AA.wiff"
Peak Name: "Val" Mass(es): "288.150/171.010 Da"
Comment: "" Annotation: ""
Sample Index: 36
Sample Type: Unknown
Concentration: N/A
Calculated Conc: 4.29 umol/L
Acq. Date: 2025-05-15
Acq. Time: 23:48:08
Modified: Yes
Proc. Algorithm: Specify Parameters - MQ III

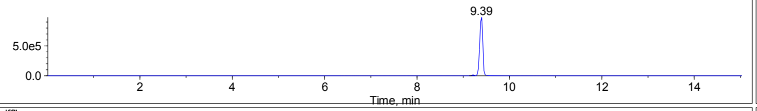

Sample Name: "ZW-1" Sample ID: "" File: "20250515-50AA.wiff"
Peak Name: "MSLAB-Nvl(IS)" Mass(es): "288.150/171.010 Da"
Comment: "" Annotation: ""
Sample Index: 36
Sample Type: Unknown
Concentration: 1.00 ng/mL
Calculated Conc: N/A
Acq. Date: 2025-05-15
Acq. Time: 23:48:08
Modified: Yes
Proc. Algorithm: Specify Parameters - MQ III

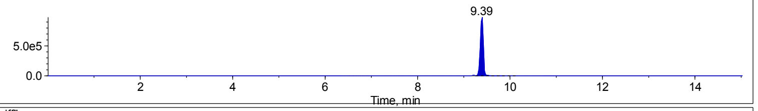

Sample Name: "ZW-1" Sample ID: "" File: "20250515-50AA.wiff"
Peak Name: "Thr" Mass(es): "290.100/171.010 Da"
Comment: "" Annotation: ""
Sample Index: 36
Sample Type: Unknown
Concentration: N/A
Calculated Conc: 2.69 umol/L
Acq. Date: 2025-05-15
Acq. Time: 23:48:08
Modified: Yes
Proc. Algorithm: Specify Parameters - MQ III

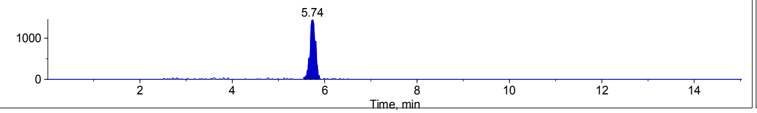

Sample Name: "ZW-1" Sample ID: "" File: "20250515-50AA.wiff"
Peak Name: "MSLAB-Nvl(IS)" Mass(es): "288.150/171.010 Da"
Comment: "" Annotation: ""
Sample Index: 36
Sample Type: Unknown
Concentration: 1.00 ng/mL
Calculated Conc: N/A
Acq. Date: 2025-05-15
Acq. Time: 23:48:08
Modified: Yes
Proc. Algorithm: Specify Parameters - MQ III

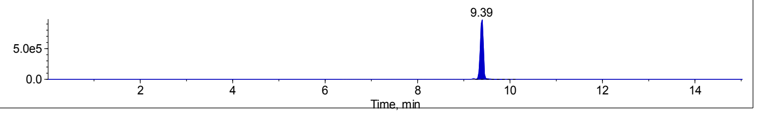

Sample Name: "ZW-1" Sample ID: "" File: "20250515-50AA.wiff"
Peak Name: "Cys1/2" Mass(es): "292.200/171.010 Da"
Comment: "" Annotation: ""

Sample Index: 36
Sample Type: Unknown
Concentration: N/A
Calculated Conc: 0.398 umol/L
Acq. Date: 2025-05-15
Acq. Time: 23:48:08
Modified: Yes
Proc. Algorithm: Specify Parameters - MQ III

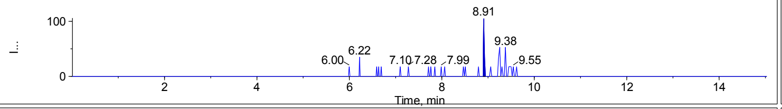

Sample Name: "ZW-1" Sample ID: "" File: "20250515-50AA.wiff"
Peak Name: "MSLAB-Nvl(IS)" Mass(es): "288.150/171.010 Da"
Comment: "" Annotation: ""

Sample Index: 36
Sample Type: Unknown
Concentration: 1.00 ng/mL
Calculated Conc: N/A
Acq. Date: 2025-05-15
Acq. Time: 23:48:08
Modified: Yes
Proc. Algorithm: Specify Parameters - MQ III

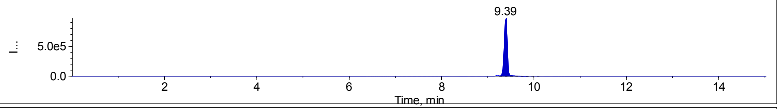

Sample Name: "ZW-1" Sample ID: "" File: "20250515-50AA.wiff"
Peak Name: "Ile" Mass(es): "302.150/171.010 Da"
Comment: "" Annotation: ""

Sample Index: 36
Sample Type: Unknown
Concentration: N/A
Calculated Conc: 1.47 umol/L
Acq. Date: 2025-05-15
Acq. Time: 23:48:08
Modified: Yes
Proc. Algorithm: Specify Parameters - MQ III

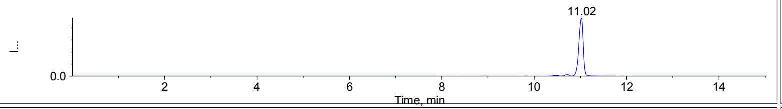

Sample Name: "ZW-1" Sample ID: "" File: "20250515-50AA.wiff"
Peak Name: "MSLAB-Nvl(IS)" Mass(es): "288.150/171.010 Da"
Comment: "" Annotation: ""

Sample Index: 36
Sample Type: Unknown
Concentration: 1.00 ng/mL
Calculated Conc: N/A
Acq. Date: 2025-05-15
Acq. Time: 23:48:08
Modified: Yes
Proc. Algorithm: Specify Parameters - MQ III

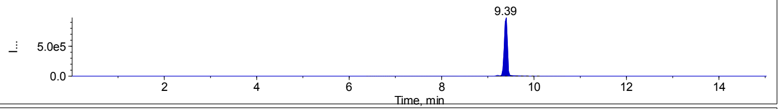

Sample Name: "ZW-1" Sample ID: "" File: "20250515-50AA.wiff"
Peak Name: "Asn" Mass(es): "303.130/171.010 Da"
Comment: "" Annotation: ""

Sample Index: 36
Sample Type: Unknown
Concentration: N/A
Calculated Conc: 0.00 umol/L
Acq. Date: 2025-05-15
Acq. Time: 23:48:08
Modified: Yes

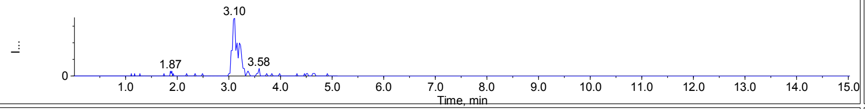

Sample Name: "ZW-1" Sample ID: "" File: "20250515-50AA.wiff"
Peak Name: "MSLAB-Nvl(IS)" Mass(es): "288.150/171.010 Da"
Comment: "" Annotation: ""

Sample Index: 36
Sample Type: Unknown
Concentration: 1.00 ng/mL
Calculated Conc: N/A
Acq. Date: 2025-05-15
Acq. Time: 23:48:08
Modified: Yes
Proc. Algorithm: Specify Parameters - MQ III

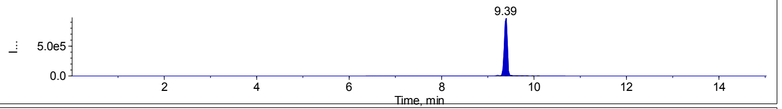

Sample Name: "ZW-1" Sample ID: "" File: "20250515-50AA.wiff"
Peak Name: "Asp" Mass(es): "304.110/171.010 Da"
Comment: "" Annotation: ""

Sample Index: 36
Sample Type: Unknown
Concentration: N/A
Calculated Conc: 1.54 umol/L
Acq. Date: 2025-05-15
Acq. Time: 23:48:08
Modified: Yes
Proc. Algorithm: Specify Parameters - MQ III

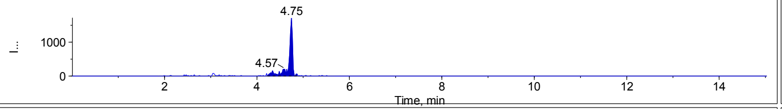

Sample Name: "ZW-1" Sample ID: "" File: "20250515-50AA.wiff"
Peak Name: "MSLAB-Nvl(IS)" Mass(es): "288.150/171.010 Da"
Comment: "" Annotation: ""

Sample Index: 36
Sample Type: Unknown
Concentration: 1.00 ng/mL
Calculated Conc: N/A
Acq. Date: 2025-05-15
Acq. Time: 23:48:08
Modified: Yes
Proc. Algorithm: Specify Parameters - MQ III

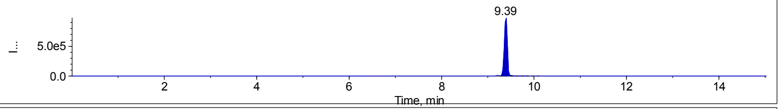

Sample Name: "ZW-1" Sample ID: "" File: "20250515-50AA.wiff"
Peak Name: "Gln" Mass(es): "317.150/171.010 Da"
Comment: "" Annotation: ""

Sample Index: 36
Sample Type: Unknown
Concentration: N/A
Calculated Conc: No Intercept
Acq. Date: 2025-05-15
Acq. Time: 23:48:08
Modified: Yes
Proc. Algorithm: Specify Parameters - MQ III

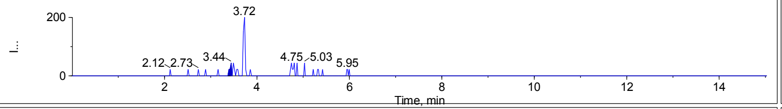

Sample Name: "ZW-1" Sample ID: "" File: "20250515-50AA.wiff"
Peak Name: "MSLAB-Nvl(IS)" Mass(es): "288.150/171.010 Da"
Comment: "" Annotation: ""

Sample Index: 36
Sample Type: Unknown
Concentration: 1.00 ng/mL
Calculated Conc: N/A
Acq. Date: 2025-05-15
Acq. Time: 23:48:08
Modified: Yes
Proc. Algorithm: Specify Parameters - MQ III

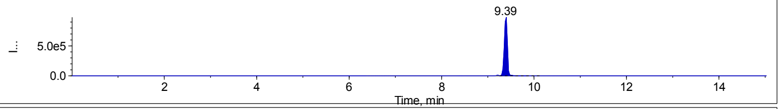

Sample Name: "ZW-1" Sample ID: "" File: "20250515-50AA.wiff"
Peak Name: "Glu" Mass(es): "318.100/171.010 Da"
Comment: "" Annotation: ""

Sample Index: 36
Sample Type: Unknown
Concentration: N/A
Calculated Conc: 4.84 umol/L
Acq. Date: 2025-05-15
Acq. Time: 23:48:08
Modified: Yes
Proc. Algorithm: Specify Parameters - MQ III

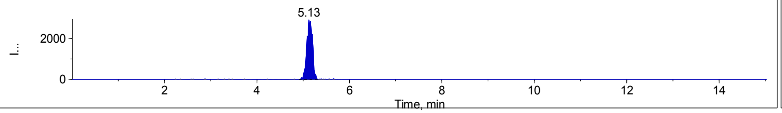

Sample Name: "ZW-1" Sample ID: "" File: "20250515-50AA.wiff"
Peak Name: "MSLAB-Nvl(IS)" Mass(es): "288.150/171.010 Da"
Comment: "" Annotation: ""

Sample Index: 36
Sample Type: Unknown
Concentration: 1.00 ng/mL
Calculated Conc: N/A
Acq. Date: 2025-05-15
Acq. Time: 23:48:08
Modified: Yes
Proc. Algorithm: Specify Parameters - MQ III

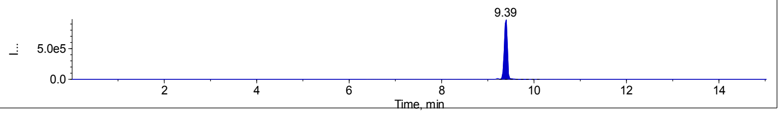

Sample Name: "ZW-1" Sample ID: "" File: "20250515-50AA.wiff"
Peak Name: "Met" Mass(es): "320.210/171.010 Da"
Comment: "" Annotation: ""

Sample Index: 36
Sample Type: Unknown
Concentration: N/A
Calculated Conc: < 0
Acq. Date: 2025-05-15
Acq. Time: 23:48:08

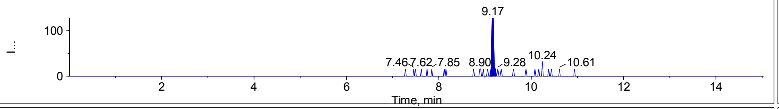

Modified: Yes
Proc. Algorithm: Specify Parameters - MQ III

Sample Name: "ZW-1" Sample ID: "" File: "20250515-50AA.wiff"
Peak Name: "His" Mass(es): "326.150/171.010 Da"
Comment: "" Annotation: ""

Sample Index: 36
Sample Type: Unknown
Concentration: N/A
Calculated Conc: 1.90 umol/L
Acq. Date: 2025-05-15
Acq. Time: 23:48:08

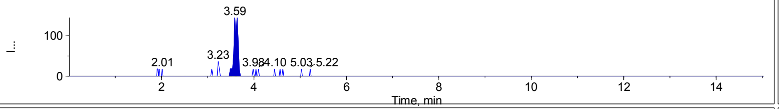

Modified: Yes
Proc. Algorithm: Specify Parameters - MQ III

Sample Name: "ZW-1" Sample ID: "" File: "20250515-50AA.wiff"
Peak Name: "Phe" Mass(es): "336.200/171.010 Da"
Comment: "" Annotation: ""

Sample Index: 36
Sample Type: Unknown
Concentration: N/A
Calculated Conc: 1.66 umol/L
Acq. Date: 2025-05-15
Acq. Time: 23:48:08

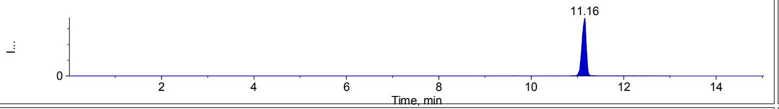

Modified: Yes
Proc. Algorithm: Specify Parameters - MQ III

Sample Name: "ZW-1" Sample ID: "" File: "20250515-50AA.wiff"
Peak Name: "Arg" Mass(es): "345.210/171.010 Da"
Comment: "" Annotation: ""

Sample Index: 36
Sample Type: Unknown
Concentration: N/A
Calculated Conc: 3.85 umol/L
Acq. Date: 2025-05-15
Acq. Time: 23:48:08

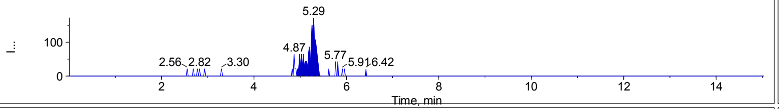

Modified: Yes
Proc. Algorithm: Specify Parameters - MQ III

Sample Name: "ZW-1" Sample ID: "" File: "20250515-50AA.wiff"
Peak Name: "Trp" Mass(es): "375.200/171.010 Da"
Comment: "" Annotation: ""

Sample Index: 36
Sample Type: Unknown
Concentration: N/A
Calculated Conc: < 0
Acq. Date: 2025-05-15
Acq. Time: 23:48:08

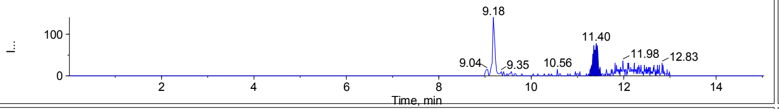

Modified: Yes
Proc. Algorithm: Specify Parameters - MQ III

Sample Name: "ZW-1" Sample ID: "" File: "20250515-50AA.wiff"
Peak Name: "Lys" Mass(es): "487.200/171.010 Da"
Comment: "" Annotation: ""

Sample Index: 36
Sample Type: Unknown
Concentration: N/A
Calculated Conc: 4.36 umol/L
Acq. Date: 2025-05-15
Acq. Time: 23:48:08

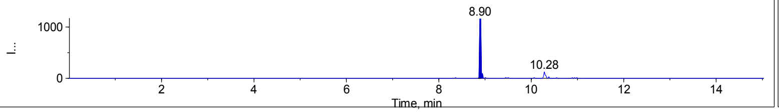

Modified: Yes
Proc. Algorithm: Specify Parameters - MQ III

Sample Name: "ZW-1" Sample ID: "" File: "20250515-50AA.wiff"
Peak Name: "MSLAB-Nvl(IS)" Mass(es): "288.150/171.010 Da"
Comment: "" Annotation: ""

Sample Index: 36
Sample Type: Unknown
Concentration: 1.00 ng/mL
Calculated Conc: N/A
Acq. Date: 2025-05-15
Acq. Time: 23:48:08

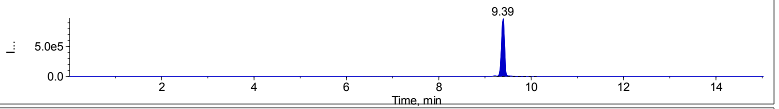

Modified: Yes
Proc. Algorithm: Specify Parameters - MQ III

Sample Name: "ZW-1" Sample ID: "" File: "20250515-50AA.wiff"
Peak Name: "MSLAB-Nvl(IS)" Mass(es): "288.150/171.010 Da"
Comment: "" Annotation: ""

Sample Index: 36
Sample Type: Unknown
Concentration: 1.00 ng/mL
Calculated Conc: N/A
Acq. Date: 2025-05-15
Acq. Time: 23:48:08

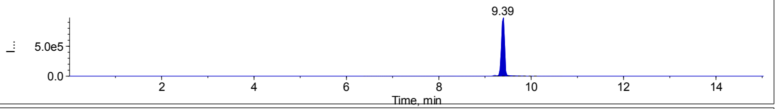

Modified: Yes
Proc. Algorithm: Specify Parameters - MQ III

Sample Name: "ZW-1" Sample ID: "" File: "20250515-50AA.wiff"
Peak Name: "MSLAB-Nvl(IS)" Mass(es): "288.150/171.010 Da"
Comment: "" Annotation: ""

Sample Index: 36
Sample Type: Unknown
Concentration: 1.00 ng/mL
Calculated Conc: N/A
Acq. Date: 2025-05-15
Acq. Time: 23:48:08

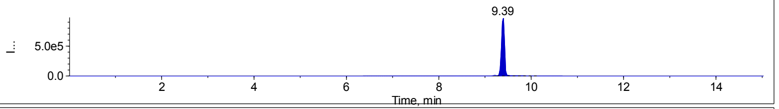

Modified: Yes
Proc. Algorithm: Specify Parameters - MQ III

Sample Name: "ZW-1" Sample ID: "" File: "20250515-50AA.wiff"
Peak Name: "MSLAB-Nvl(IS)" Mass(es): "288.150/171.010 Da"
Comment: "" Annotation: ""

Sample Index: 36
Sample Type: Unknown
Concentration: 1.00 ng/mL
Calculated Conc: N/A
Acq. Date: 2025-05-15
Acq. Time: 23:48:08

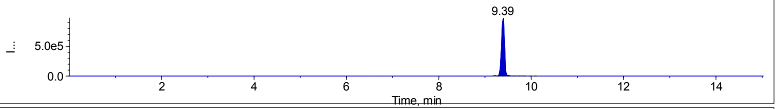

Modified: Yes
Proc. Algorithm: Specify Parameters - MQ III

Sample Name: "ZW-1" Sample ID: "" File: "20250515-50AA.wiff"
Peak Name: "MSLAB-Nvl(IS)" Mass(es): "288.150/171.010 Da"
Comment: "" Annotation: ""

Sample Index: 36
Sample Type: Unknown
Concentration: 1.00 ng/mL
Calculated Conc: N/A
Acq. Date: 2025-05-15
Acq. Time: 23:48:08

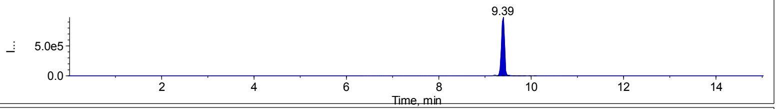

Modified: Yes
Proc. Algorithm: Specify Parameters - MQ III

Sample Name: "ZW-1" Sample ID: "" File: "20250515-50AA.wiff"
Peak Name: "MSLAB-Nvl(IS)" Mass(es): "288.150/171.010 Da"
Comment: "" Annotation: ""

Sample Index: 36
Sample Type: Unknown
Concentration: 1.00 ng/mL
Calculated Conc: N/A
Acq. Date: 2025-05-15
Acq. Time: 23:48:08

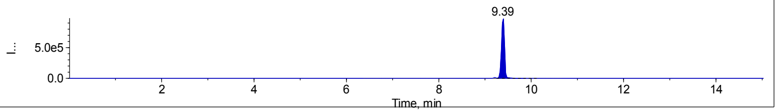

Modified: Yes
Proc. Algorithm: Specify Parameters - MQ III

Sample Name: "ZW-1" Sample ID: "" File: "20250515-50AA.wiff"
Peak Name: "tyr" Mass(es): "352.200/171.010 Da"
Comment: "" Annotation: ""
Sample Index: 36
Sample Type: Unknown
Concentration: N/A
Calculated Conc: < 0
Acq. Date: 2025-05-15
Acq. Time: 23:48:08
Modified: Yes
Proc. Algorithm: Specify Parameters - MQ III

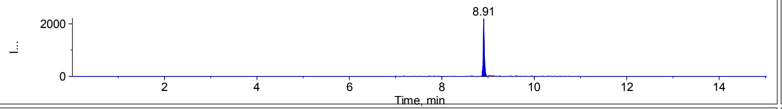

Sample Name: "ZW-1" Sample ID: "" File: "20250515-50AA.wiff"
Peak Name: "MSLAB-Nvl(IS)" Mass(es): "288.150/171.010 Da"
Comment: "" Annotation: ""
Sample Index: 36
Sample Type: Unknown
Concentration: 1.00 ng/mL
Calculated Conc: N/A
Acq. Date: 2025-05-15
Acq. Time: 23:48:08
Modified: Yes
Proc. Algorithm: Specify Parameters - MQ III

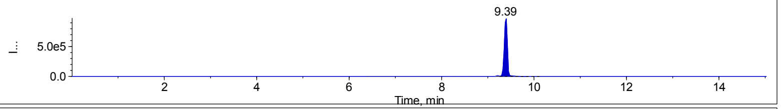

Sample Name: "ZW-1" Sample ID: "" File: "20250515-50AA.wiff"
Peak Name: "Leu" Mass(es): "302.150/171.010 Da"
Comment: "" Annotation: ""
Sample Index: 36
Sample Type: Unknown
Concentration: N/A
Calculated Conc: 3.70 umol/L
Acq. Date: 2025-05-15
Acq. Time: 23:48:08
Modified: Yes
Proc. Algorithm: Specify Parameters - MQ III

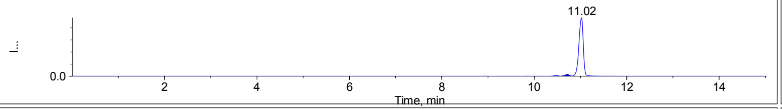

Sample Name: "ZW-1" Sample ID: "" File: "20250515-50AA.wiff"
Peak Name: "MSLAB-Nvl(IS)" Mass(es): "288.150/171.010 Da"
Comment: "" Annotation: ""
Sample Index: 36
Sample Type: Unknown
Concentration: 1.00 ng/mL
Calculated Conc: N/A
Acq. Date: 2025-05-15
Acq. Time: 23:48:08
Modified: Yes
Proc. Algorithm: Specify Parameters - MQ III

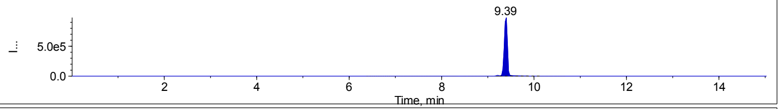

Sample Name: "ZW-2" Sample ID: "" File: "20250515-50AA.wiff"
Peak Name: "Gly" Mass(es): "246.080/171.010 Da"
Comment: "" Annotation: ""
Sample Index: 37
Sample Type: Unknown
Concentration: N/A
Calculated Conc: 2.51 umol/L
Acq. Date: 2025-05-16
Acq. Time: 00:03:38
Modified: Yes
Proc. Algorithm: Specify Parameters - MQ III

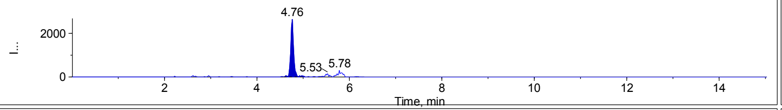

Sample Name: "ZW-2" Sample ID: "" File: "20250515-50AA.wiff"
Peak Name: "MSLAB-Nvl(IS)" Mass(es): "288.150/171.010 Da"
Comment: "" Annotation: ""
Sample Index: 37
Sample Type: Unknown
Concentration: 1.00 ng/mL
Calculated Conc: N/A
Acq. Date: 2025-05-16
Acq. Time: 00:03:38
Modified: Yes
Proc. Algorithm: Specify Parameters - MQ III

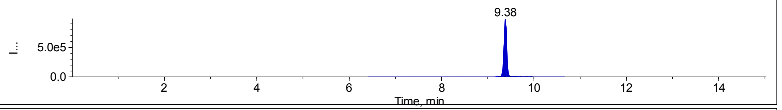

Sample Name: "ZW-2" Sample ID: "" File: "20250515-50AA.wiff"
Peak Name: "Ala" Mass(es): "260.100/171.010 Da"
Comment: "" Annotation: ""
Sample Index: 37
Sample Type: Unknown
Concentration: N/A
Calculated Conc: No Intercept
Acq. Date: 2025-05-16
Acq. Time: 00:03:38
Modified: Yes
Proc. Algorithm: Specify Parameters - MQ III

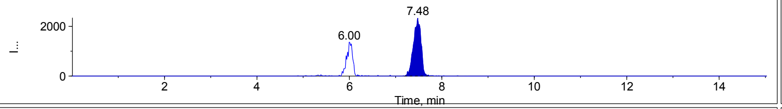

Sample Name: "ZW-2" Sample ID: "" File: "20250515-50AA.wiff"
Peak Name: "MSLAB-Nvl(IS)" Mass(es): "288.150/171.010 Da"
Comment: "" Annotation: ""
Sample Index: 37
Sample Type: Unknown
Concentration: 1.00 ng/mL
Calculated Conc: N/A
Acq. Date: 2025-05-16
Acq. Time: 00:03:38
Modified: Yes
Proc. Algorithm: Specify Parameters - MQ III

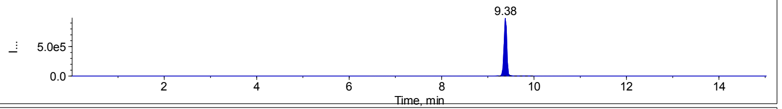

Sample Name: "ZW-2" Sample ID: "" File: "20250515-50AA.wiff"
Peak Name: "Ser" Mass(es): "276.100/171.010 Da"
Comment: "" Annotation: ""
Sample Index: 37
Sample Type: Unknown
Concentration: N/A
Calculated Conc: 1.46 umol/L
Acq. Date: 2025-05-16
Acq. Time: 00:03:38
Modified: Yes
Proc. Algorithm: Specify Parameters - MQ III

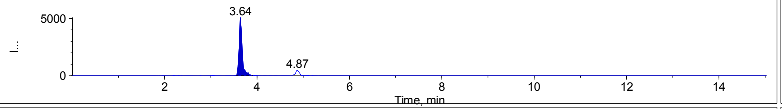

Sample Name: "ZW-2" Sample ID: "" File: "20250515-50AA.wiff"
Peak Name: "MSLAB-Nvl(IS)" Mass(es): "288.150/171.010 Da"
Comment: "" Annotation: ""
Sample Index: 37
Sample Type: Unknown
Concentration: 1.00 ng/mL
Calculated Conc: N/A
Acq. Date: 2025-05-16
Acq. Time: 00:03:38
Modified: Yes
Proc. Algorithm: Specify Parameters - MQ III

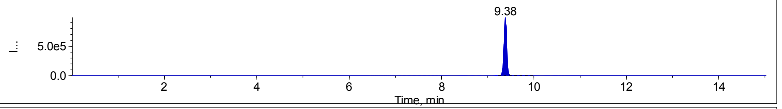

Sample Name: "ZW-2" Sample ID: "" File: "20250515-50AA.wiff"
Peak Name: "Pro" Mass(es): "286.150/171.010 Da"
Comment: "" Annotation: ""
Sample Index: 37
Sample Type: Unknown
Concentration: N/A
Calculated Conc: No Intercept
Acq. Date: 2025-05-16
Acq. Time: 00:03:38
Modified: Yes
Proc. Algorithm: Specify Parameters - MQ III

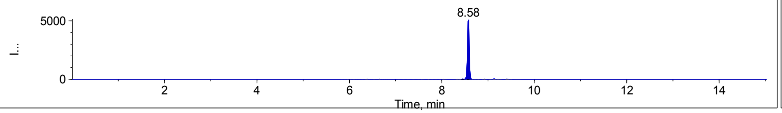

Sample Name: "ZW-2" Sample ID: "" File: "20250515-50AA.wiff"
Peak Name: "MSLAB-Nvl(IS)" Mass(es): "288.150/171.010 Da"
Comment: "" Annotation: ""
Sample Index: 37
Sample Type: Unknown
Concentration: 1.00 ng/mL
Calculated Conc: N/A
Acq. Date: 2025-05-16
Acq. Time: 00:03:38
Modified: Yes
Proc. Algorithm: Specify Parameters - MQ III

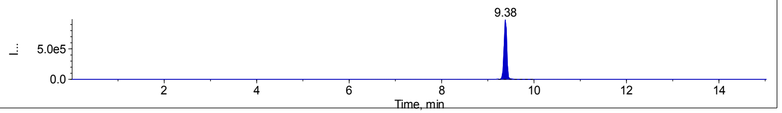

Sample Name: "ZW-2" Sample ID: "" File: "20250515-50AA.wiff"
Peak Name: "Val" Mass(es): "288.150/171.010 Da"
Comment: "" Annotation: ""

```
Sample Index:        37
Sample Type:         Unknown
Concentration:       N/A
Calculated Conc:     1.95    umol/L
Acq. Date:           2025-05-16
Acq. Time:           00:03:38
Modified:            Yes
Proc. Algorithm: Specify Parameters - MQ III
```

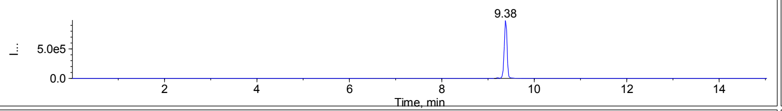

Sample Name: "ZW-2" Sample ID: "" File: "20250515-50AA.wiff"
Peak Name: "Thr" Mass(es): "290.100/171.010 Da"
Comment: "" Annotation: ""

```
Sample Index:        37
Sample Type:         Unknown
Concentration:       N/A
Calculated Conc:     1.46    umol/L
Acq. Date:           2025-05-16
Acq. Time:           00:03:38
Modified:            Yes
Proc. Algorithm: Specify Parameters - MQ III
```

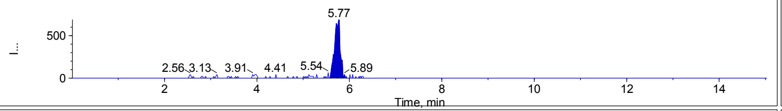

Sample Name: "ZW-2" Sample ID: "" File: "20250515-50AA.wiff"
Peak Name: "Cys1/2" Mass(es): "292.200/171.010 Da"
Comment: "" Annotation: ""

```
Sample Index:        37
Sample Type:         Unknown
Concentration:       N/A
Calculated Conc:     0.157   umol/L
Acq. Date:           2025-05-16
Acq. Time:           00:03:38
Modified:            Yes
Proc. Algorithm: Specify Parameters - MQ III
```

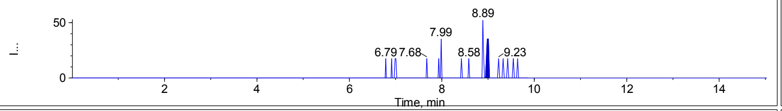

Sample Name: "ZW-2" Sample ID: "" File: "20250515-50AA.wiff"
Peak Name: "Ile" Mass(es): "302.150/171.010 Da"
Comment: "" Annotation: ""

```
Sample Index:        37
Sample Type:         Unknown
Concentration:       N/A
Calculated Conc:     0.335   umol/L
Acq. Date:           2025-05-16
Acq. Time:           00:03:38
Modified:            Yes
Proc. Algorithm: Specify Parameters - MQ III
```

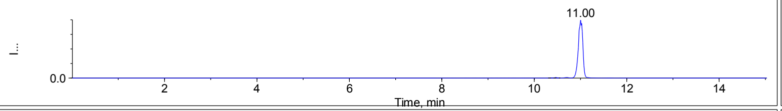

Sample Name: "ZW-2" Sample ID: "" File: "20250515-50AA.wiff"
Peak Name: "Asn" Mass(es): "303.130/171.010 Da"
Comment: "" Annotation: ""

```
Sample Index:        37
Sample Type:         Unknown
Concentration:       N/A
Calculated Conc:     < 0
Acq. Date:           2025-05-16
Acq. Time:           00:03:38
Modified:            Yes
Proc. Algorithm: Specify Parameters - MQ III
```

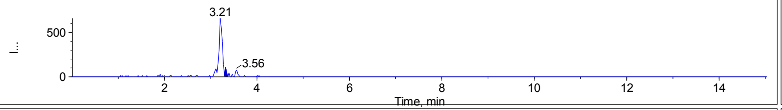

Sample Name: "ZW-2" Sample ID: "" File: "20250515-50AA.wiff"
Peak Name: "Asp" Mass(es): "304.110/171.010 Da"
Comment: "" Annotation: ""

```
Sample Index:        37
Sample Type:         Unknown
Concentration:       N/A
Calculated Conc:     < 0
Acq. Date:           2025-05-16
Acq. Time:           00:03:38
Modified:            Yes
Proc. Algorithm: Specify Parameters - MQ III
```

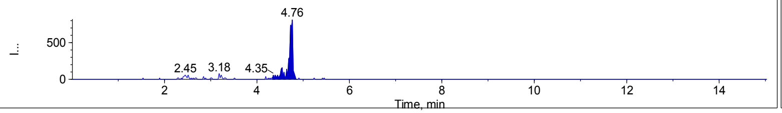

Sample Name: "ZW-2" Sample ID: "" File: "20250515-50AA.wiff"
Peak Name: "MSLAB-Nvl(IS)" Mass(es): "288.150/171.010 Da"
Comment: "" Annotation: ""

```
Sample Index:        37
Sample Type:         Unknown
Concentration:       1.00    ng/mL
Calculated Conc:     N/A
Acq. Date:           2025-05-16
Acq. Time:           00:03:38
Modified:            Yes
Proc. Algorithm: Specify Parameters - MQ III
```

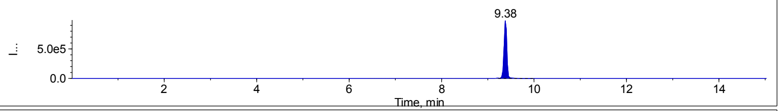

Sample Name: "ZW-2" Sample ID: "" File: "20250515-50AA.wiff"
Peak Name: "MSLAB-Nvl(IS)" Mass(es): "288.150/171.010 Da"
Comment: "" Annotation: ""

Sample Index: 37
Sample Type: Unknown
Concentration: 1.00 ng/mL
Calculated Conc: N/A
Acq. Date: 2025-05-16
Acq. Time: 00:03:38
Modified: Yes
Proc. Algorithm: Specify Parameters - MQ III

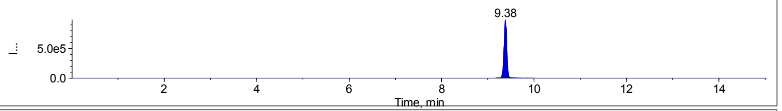

Sample Name: "ZW-2" Sample ID: "" File: "20250515-50AA.wiff"
Peak Name: "MSLAB-Nvl(IS)" Mass(es): "288.150/171.010 Da"
Comment: "" Annotation: ""
Sample Index: 37
Sample Type: Unknown
Concentration: 1.00 ng/mL
Calculated Conc: N/A
Acq. Date: 2025-05-16
Acq. Time: 00:03:38
Modified: Yes
Proc. Algorithm: Specify Parameters - MQ III

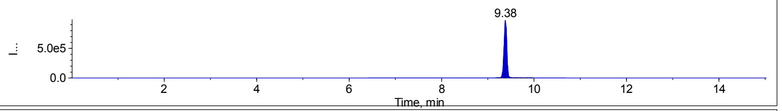

Sample Name: "ZW-2" Sample ID: "" File: "20250515-50AA.wiff"
Peak Name: "MSLAB-Nvl(IS)" Mass(es): "288.150/171.010 Da"
Comment: "" Annotation: ""
Sample Index: 37
Sample Type: Unknown
Concentration: 1.00 ng/mL
Calculated Conc: N/A
Acq. Date: 2025-05-16
Acq. Time: 00:03:38
Modified: Yes
Proc. Algorithm: Specify Parameters - MQ III

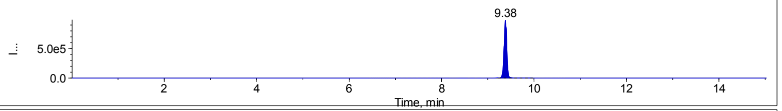

Sample Name: "ZW-2" Sample ID: "" File: "20250515-50AA.wiff"
Peak Name: "MSLAB-Nvl(IS)" Mass(es): "288.150/171.010 Da"
Comment: "" Annotation: ""
Sample Index: 37
Sample Type: Unknown
Concentration: 1.00 ng/mL
Calculated Conc: N/A
Acq. Date: 2025-05-16
Acq. Time: 00:03:38
Modified: Yes
Proc. Algorithm: Specify Parameters - MQ III

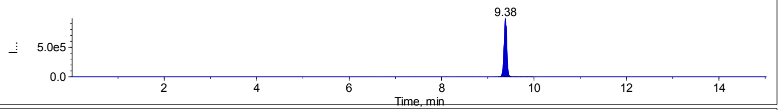

Sample Name: "ZW-2" Sample ID: "" File: "20250515-50AA.wiff"
Peak Name: "MSLAB-Nvl(IS)" Mass(es): "288.150/171.010 Da"
Comment: "" Annotation: ""
Sample Index: 37
Sample Type: Unknown
Concentration: 1.00 ng/mL
Calculated Conc: N/A
Acq. Date: 2025-05-16
Acq. Time: 00:03:38
Modified: Yes
Proc. Algorithm: Specify Parameters - MQ III

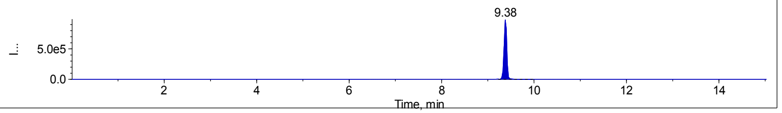

Sample Name: "ZW-2" Sample ID: "" File: "20250515-50AA.wiff"
Peak Name: "Gln" Mass(es): "317.150/171.010 Da"
Comment: "" Annotation: ""

```
Sample Index:      37
Sample Type:       Unknown
Concentration:        N/A
Calculated Conc: No Intercept
Acq. Date:         2025-05-16
Acq. Time:         00:03:38

Modified:               Yes
Proc. Algorithm: Specify Parameters - MQ III
```

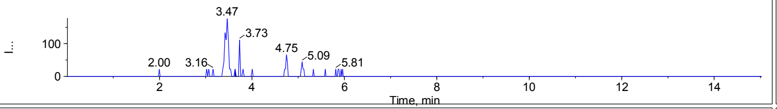

Sample Name: "ZW-2" Sample ID: "" File: "20250515-50AA.wiff"
Peak Name: "MSLAB-Nvl(IS)" Mass(es): "288.150/171.010 Da"
Comment: "" Annotation: ""

```
Sample Index:      37
Sample Type:       Unknown
Concentration:        1.00    ng/mL
Calculated Conc:      N/A
Acq. Date:         2025-05-16
Acq. Time:         00:03:38

Modified:               Yes
Proc. Algorithm: Specify Parameters - MQ III
```

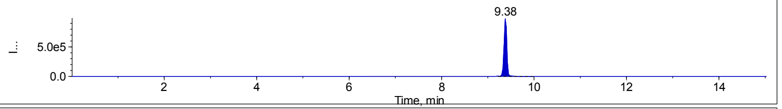

Sample Name: "ZW-2" Sample ID: "" File: "20250515-50AA.wiff"
Peak Name: "Glu" Mass(es): "318.100/171.010 Da"
Comment: "" Annotation: ""

```
Sample Index:      37
Sample Type:       Unknown
Concentration:        N/A
Calculated Conc:      1.22    umol/L
Acq. Date:         2025-05-16
Acq. Time:         00:03:38

Modified:               Yes
Proc. Algorithm: Specify Parameters - MQ III
```

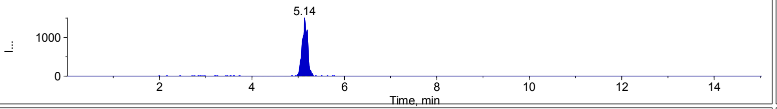

Sample Name: "ZW-2" Sample ID: "" File: "20250515-50AA.wiff"
Peak Name: "MSLAB-Nvl(IS)" Mass(es): "288.150/171.010 Da"
Comment: "" Annotation: ""

```
Sample Index:      37
Sample Type:       Unknown
Concentration:        1.00    ng/mL
Calculated Conc:      N/A
Acq. Date:         2025-05-16
Acq. Time:         00:03:38

Modified:               Yes
Proc. Algorithm: Specify Parameters - MQ III
```

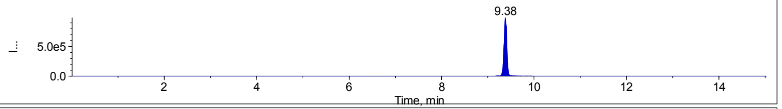

Sample Name: "ZW-2" Sample ID: "" File: "20250515-50AA.wiff"
Peak Name: "Met" Mass(es): "320.210/171.010 Da"
Comment: "" Annotation: ""

```
Sample Index:      37
Sample Type:       Unknown
Concentration:        N/A
Calculated Conc:       < 0
Acq. Date:         2025-05-16
Acq. Time:         00:03:38

Modified:               Yes
Proc. Algorithm: Specify Parameters - MQ III
```

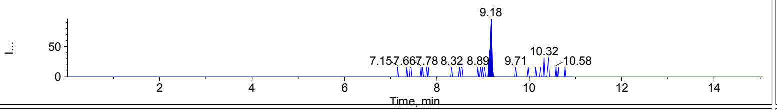

Sample Name: "ZW-2" Sample ID: "" File: "20250515-50AA.wiff"
Peak Name: "MSLAB-Nvl(IS)" Mass(es): "288.150/171.010 Da"
Comment: "" Annotation: ""

```
Sample Index:      37
Sample Type:       Unknown
Concentration:        1.00    ng/mL
Calculated Conc:      N/A
Acq. Date:         2025-05-16
Acq. Time:         00:03:38

Modified:               Yes
Proc. Algorithm: Specify Parameters - MQ III
```

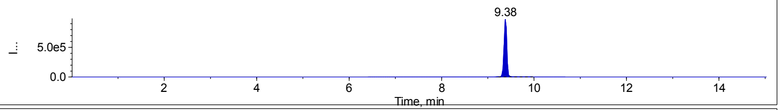

Sample Name: "ZW-2" Sample ID: "" File: "20250515-50AA.wiff"
Peak Name: "His" Mass(es): "326.150/171.010 Da"
Comment: "" Annotation: ""

```
Sample Index:      37
Sample Type:       Unknown
Concentration:        N/A
Calculated Conc:      1.33    umol/L
Acq. Date:         2025-05-16
Acq. Time:         00:03:38

Modified:               Yes
Proc. Algorithm: Specify Parameters - MQ III
```

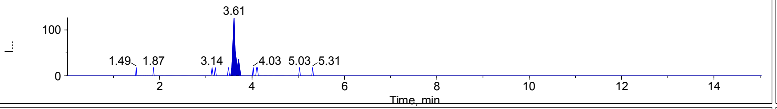

Sample Name: "ZW-2" Sample ID: "" File: "20250515-50AA.wiff"
Peak Name: "MSLAB-Nvl(IS)" Mass(es): "288.150/171.010 Da"
Comment: "" Annotation: ""

```
Sample Index:      37
Sample Type:       Unknown
Concentration:        1.00    ng/mL
Calculated Conc:      N/A
Acq. Date:         2025-05-16
Acq. Time:         00:03:38

Modified:               Yes
Proc. Algorithm: Specify Parameters - MQ III
```

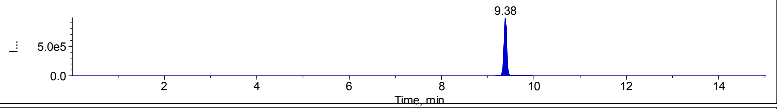

Sample Name: "ZW-2" Sample ID: "" File: "20250515-50AA.wiff"
Peak Name: "Phe" Mass(es): "336.200/171.010 Da"
Comment: "" Annotation: ""

```
Sample Index:      37
Sample Type:       Unknown
Concentration:        N/A
Calculated Conc:      0.459   umol/L
Acq. Date:         2025-05-16
Acq. Time:         00:03:38

Modified:               Yes
Proc. Algorithm: Specify Parameters - MQ III
```

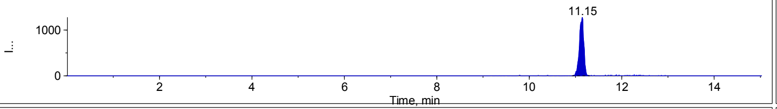

Sample Name: "ZW-2" Sample ID: "" File: "20250515-50AA.wiff"
Peak Name: "MSLAB-Nvl(IS)" Mass(es): "288.150/171.010 Da"
Comment: "" Annotation: ""

```
Sample Index:      37
Sample Type:       Unknown
Concentration:        1.00    ng/mL
Calculated Conc:      N/A
Acq. Date:         2025-05-16
Acq. Time:         00:03:38

Modified:               Yes
Proc. Algorithm: Specify Parameters - MQ III
```

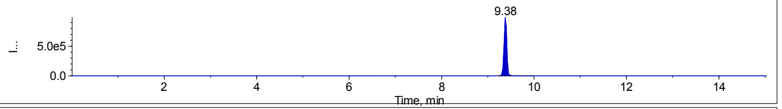

Sample Name: "ZW-2" Sample ID: "" File: "20250515-50AA.wiff"
Peak Name: "Arg" Mass(es): "345.210/171.010 Da"
Comment: "" Annotation: ""

```
Sample Index:      37
Sample Type:       Unknown
Concentration:        N/A
Calculated Conc:      2.15    umol/L
Acq. Date:         2025-05-16
Acq. Time:         00:03:38

Modified:               Yes
Proc. Algorithm: Specify Parameters - MQ III
```

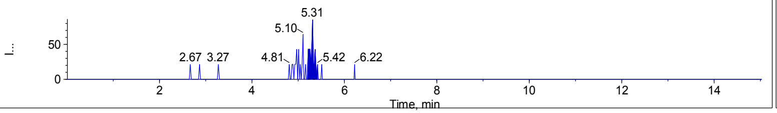

Sample Name: "ZW-2" Sample ID: "" File: "20250515-50AA.wiff"
Peak Name: "MSLAB-Nvl(IS)" Mass(es): "288.150/171.010 Da"
Comment: "" Annotation: ""

```
Sample Index:      37
Sample Type:       Unknown
Concentration:        1.00    ng/mL
Calculated Conc:      N/A
Acq. Date:         2025-05-16
Acq. Time:         00:03:38

Modified:               Yes
Proc. Algorithm: Specify Parameters - MQ III
```

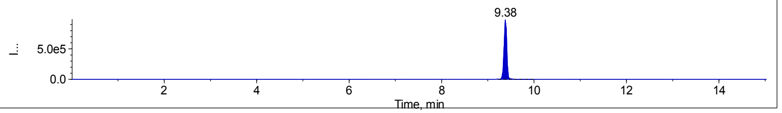

Sample Name: "ZW-2" Sample ID: "" File: "20250515-50AA.wiff"
Peak Name: "Trp" Mass(es): "375.200/171.010 Da"
Comment: "" Annotation: ""

Sample Index: 37
Sample Type: Unknown
Concentration: N/A
Calculated Conc: < 0
Acq. Date: 2025-05-16
Acq. Time: 00:03:38
Modified: Yes
Proc. Algorithm: Specify Parameters - MQ III

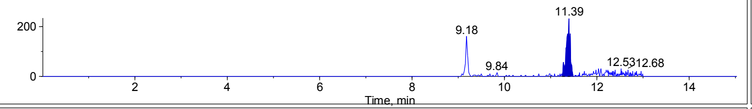

Sample Name: "ZW-2" Sample ID: "" File: "20250515-50AA.wiff"
Peak Name: "MSLAB-Nvl(IS)" Mass(es): "288.150/171.010 Da"
Comment: "" Annotation: ""

Sample Index: 37
Sample Type: Unknown
Concentration: 1.00 ng/mL
Calculated Conc: N/A
Acq. Date: 2025-05-16
Acq. Time: 00:03:38
Modified: Yes
Proc. Algorithm: Specify Parameters - MQ III

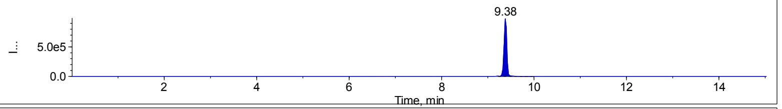

Sample Name: "ZW-2" Sample ID: "" File: "20250515-50AA.wiff"
Peak Name: "Lys" Mass(es): "487.200/171.010 Da"
Comment: "" Annotation: ""

Sample Index: 37
Sample Type: Unknown
Concentration: N/A
Calculated Conc: 1.37 umol/L
Acq. Date: 2025-05-16
Acq. Time: 00:03:38
Modified: Yes
Proc. Algorithm: Specify Parameters - MQ III

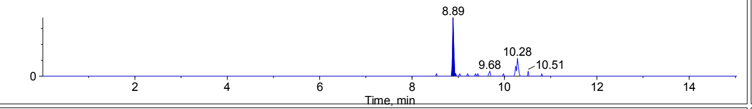

Sample Name: "ZW-2" Sample ID: "" File: "20250515-50AA.wiff"
Peak Name: "MSLAB-Nvl(IS)" Mass(es): "288.150/171.010 Da"
Comment: "" Annotation: ""

Sample Index: 37
Sample Type: Unknown
Concentration: 1.00 ng/mL
Calculated Conc: N/A
Acq. Date: 2025-05-16
Acq. Time: 00:03:38
Modified: Yes
Proc. Algorithm: Specify Parameters - MQ III

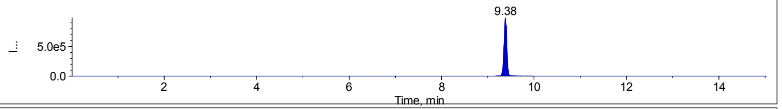

Sample Name: "ZW-2" Sample ID: "" File: "20250515-50AA.wiff"
Peak Name: "tyr" Mass(es): "352.200/171.010 Da"
Comment: "" Annotation: ""

Sample Index: 37
Sample Type: Unknown
Concentration: N/A
Calculated Conc: No Intercept
Acq. Date: 2025-05-16
Acq. Time: 00:03:38
Modified: Yes
Proc. Algorithm: Specify Parameters - MQ III

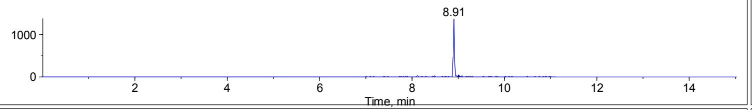

Sample Name: "ZW-2" Sample ID: "" File: "20250515-50AA.wiff"
Peak Name: "MSLAB-Nvl(IS)" Mass(es): "288.150/171.010 Da"
Comment: "" Annotation: ""

Sample Index: 37
Sample Type: Unknown
Concentration: 1.00 ng/mL
Calculated Conc: N/A
Acq. Date: 2025-05-16
Acq. Time: 00:03:38
Modified: Yes
Proc. Algorithm: Specify Parameters - MQ III

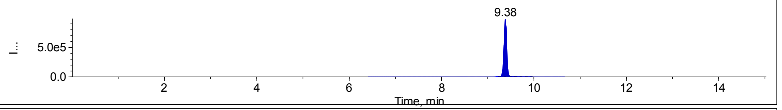

Sample Name: "ZW-2" Sample ID: "" File: "20250515-50AA.wiff"
Peak Name: "Leu" Mass(es): "302.150/171.010 Da"
Comment: "" Annotation: ""

Sample Index: 37
Sample Type: Unknown
Concentration: N/A
Calculated Conc: 0.691 umol/L
Acq. Date: 2025-05-16
Acq. Time: 00:03:38
Modified: Yes
Proc. Algorithm: Specify Parameters - MQ III

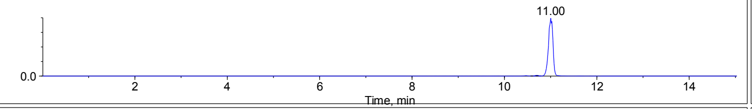

Sample Name: "ZW-2" Sample ID: "" File: "20250515-50AA.wiff"
Peak Name: "MSLAB-Nvl(IS)" Mass(es): "288.150/171.010 Da"
Comment: "" Annotation: ""

Sample Index: 37
Sample Type: Unknown
Concentration: 1.00 ng/mL
Calculated Conc: N/A
Acq. Date: 2025-05-16
Acq. Time: 00:03:38
Modified: Yes
Proc. Algorithm: Specify Parameters - MQ III

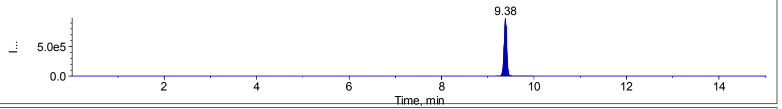

Sample Name: "ZW-3" Sample ID: "" File: "20250515-50AA.wiff"
Peak Name: "Gly" Mass(es): "246.080/171.010 Da"
Comment: "" Annotation: ""

Sample Index: 38
Sample Type: Unknown
Concentration: N/A
Calculated Conc: 2.45 umol/L
Acq. Date: 2025-05-16
Acq. Time: 00:19:08
Modified: Yes
Proc. Algorithm: Specify Parameters - MQ III

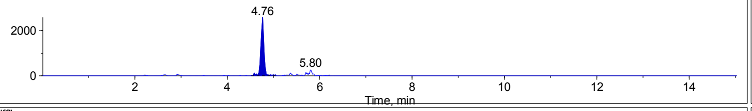

Sample Name: "ZW-3" Sample ID: "" File: "20250515-50AA.wiff"
Peak Name: "MSLAB-Nvl(IS)" Mass(es): "288.150/171.010 Da"
Comment: "" Annotation: ""

Sample Index: 38
Sample Type: Unknown
Concentration: 1.00 ng/mL
Calculated Conc: N/A
Acq. Date: 2025-05-16
Acq. Time: 00:19:08
Modified: Yes
Proc. Algorithm: Specify Parameters - MQ III

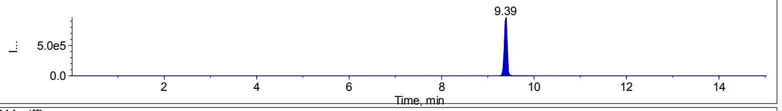

Sample Name: "ZW-3" Sample ID: "" File: "20250515-50AA.wiff"
Peak Name: "Ala" Mass(es): "260.100/171.010 Da"
Comment: "" Annotation: ""

Sample Index: 38
Sample Type: Unknown
Concentration: N/A
Calculated Conc: No Intercept
Acq. Date: 2025-05-16
Acq. Time: 00:19:08
Modified: Yes
Proc. Algorithm: Specify Parameters - MQ III

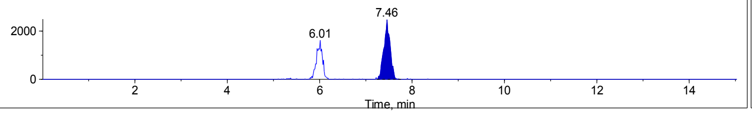

Sample Name: "ZW-3" Sample ID: "" File: "20250515-50AA.wiff"
Peak Name: "MSLAB-Nvl(IS)" Mass(es): "288.150/171.010 Da"
Comment: "" Annotation: ""

Sample Index: 38
Sample Type: Unknown
Concentration: 1.00 ng/mL
Calculated Conc: N/A
Acq. Date: 2025-05-16
Acq. Time: 00:19:08
Modified: Yes
Proc. Algorithm: Specify Parameters - MQ III

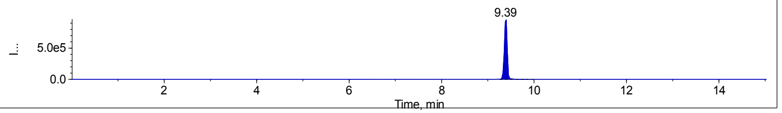

Sample Name: "ZW-3" Sample ID: "" File: "20250515-50AA.wiff"
Peak Name: "Ser" Mass(es): "276.100/171.010 Da"
Comment: "" Annotation: ""

Sample Index: 38
Sample Type: Unknown
Concentration: N/A
Calculated Conc: No Intercept
Acq. Date: 2025-05-16
Acq. Time: 00:19:08

Modified: Yes
Proc. Algorithm: Specify Parameters - MQ III

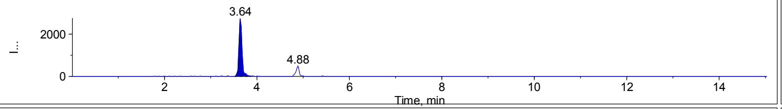

Sample Name: "ZW-3" Sample ID: "" File: "20250515-50AA.wiff"
Peak Name: "MSLAB-Nvl(IS)" Mass(es): "288.150/171.010 Da"
Comment: "" Annotation: ""

Sample Index: 38
Sample Type: Unknown
Concentration: 1.00 ng/mL
Calculated Conc: N/A
Acq. Date: 2025-05-16
Acq. Time: 00:19:08

Modified: Yes
Proc. Algorithm: Specify Parameters - MQ III

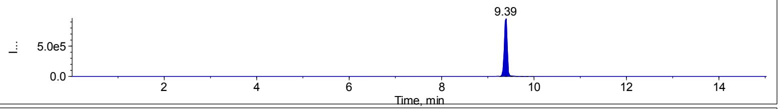

Sample Name: "ZW-3" Sample ID: "" File: "20250515-50AA.wiff"
Peak Name: "Pro" Mass(es): "286.150/171.010 Da"
Comment: "" Annotation: ""

Sample Index: 38
Sample Type: Unknown
Concentration: N/A
Calculated Conc: No Intercept
Acq. Date: 2025-05-16
Acq. Time: 00:19:08

Modified: Yes
Proc. Algorithm: Specify Parameters - MQ III

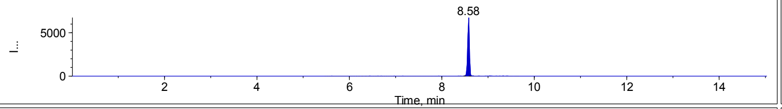

Sample Name: "ZW-3" Sample ID: "" File: "20250515-50AA.wiff"
Peak Name: "MSLAB-Nvl(IS)" Mass(es): "288.150/171.010 Da"
Comment: "" Annotation: ""

Sample Index: 38
Sample Type: Unknown
Concentration: 1.00 ng/mL
Calculated Conc: N/A
Acq. Date: 2025-05-16
Acq. Time: 00:19:08

Modified: Yes
Proc. Algorithm: Specify Parameters - MQ III

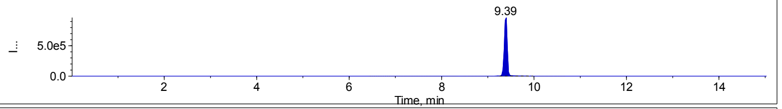

Sample Name: "ZW-3" Sample ID: "" File: "20250515-50AA.wiff"
Peak Name: "Val" Mass(es): "288.150/171.010 Da"
Comment: "" Annotation: ""

Sample Index: 38
Sample Type: Unknown
Concentration: N/A
Calculated Conc: 1.51 umol/L
Acq. Date: 2025-05-16
Acq. Time: 00:19:08

Modified: Yes
Proc. Algorithm: Specify Parameters - MQ III

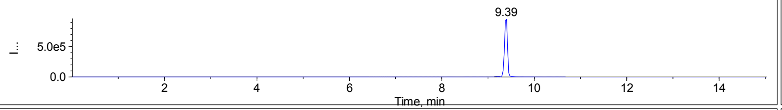

Sample Name: "ZW-3" Sample ID: "" File: "20250515-50AA.wiff"
Peak Name: "MSLAB-Nvl(IS)" Mass(es): "288.150/171.010 Da"
Comment: "" Annotation: ""

Sample Index: 38
Sample Type: Unknown
Concentration: 1.00 ng/mL
Calculated Conc: N/A
Acq. Date: 2025-05-16
Acq. Time: 00:19:08

Modified: Yes
Proc. Algorithm: Specify Parameters - MQ III

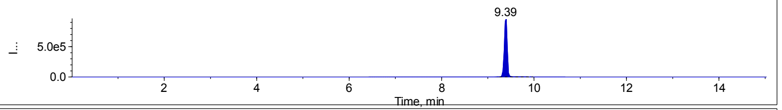

Sample Name: "ZW-3" Sample ID: "" File: "20250515-50AA.wiff"
Peak Name: "Thr" Mass(es): "290.100/171.010 Da"
Comment: "" Annotation: ""

Sample Index: 38
Sample Type: Unknown
Concentration: N/A
Calculated Conc: 1.02 umol/L
Acq. Date: 2025-05-16
Acq. Time: 00:19:08

Modified: Yes
Proc. Algorithm: Specify Parameters - MQ III

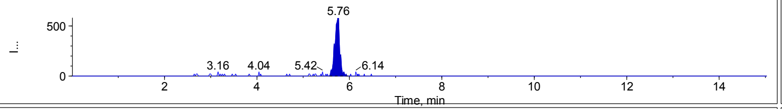

Sample Name: "ZW-3" Sample ID: "" File: "20250515-50AA.wiff"
Peak Name: "MSLAB-Nvl(IS)" Mass(es): "288.150/171.010 Da"
Comment: "" Annotation: ""

Sample Index: 38
Sample Type: Unknown
Concentration: 1.00 ng/mL
Calculated Conc: N/A
Acq. Date: 2025-05-16
Acq. Time: 00:19:08

Modified: Yes
Proc. Algorithm: Specify Parameters - MQ III

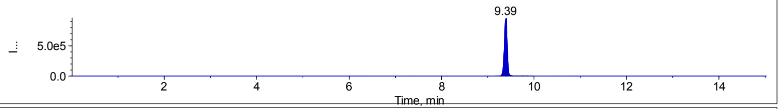

Sample Name: "ZW-3" Sample ID: "" File: "20250515-50AA.wiff"
Peak Name: "Cys1/2" Mass(es): "292.200/171.010 Da"
Comment: "" Annotation: ""

Sample Index: 38
Sample Type: Unknown
Concentration: N/A
Calculated Conc: < 0
Acq. Date: 2025-05-16
Acq. Time: 00:19:08

Modified: Yes
Proc. Algorithm: Specify Parameters - MQ III

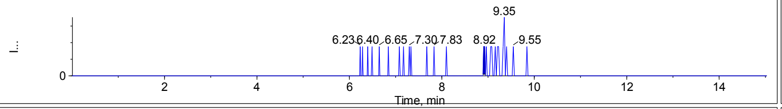

Sample Name: "ZW-3" Sample ID: "" File: "20250515-50AA.wiff"
Peak Name: "MSLAB-Nvl(IS)" Mass(es): "288.150/171.010 Da"
Comment: "" Annotation: ""

Sample Index: 38
Sample Type: Unknown
Concentration: 1.00 ng/mL
Calculated Conc: N/A
Acq. Date: 2025-05-16
Acq. Time: 00:19:08

Modified: Yes
Proc. Algorithm: Specify Parameters - MQ III

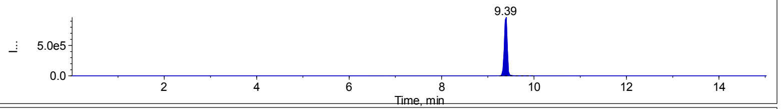

Sample Name: "ZW-3" Sample ID: "" File: "20250515-50AA.wiff"
Peak Name: "Ile" Mass(es): "302.150/171.010 Da"
Comment: "" Annotation: ""

Sample Index: 38
Sample Type: Unknown
Concentration: N/A
Calculated Conc: 0.168 umol/L
Acq. Date: 2025-05-16
Acq. Time: 00:19:08

Modified: Yes
Proc. Algorithm: Specify Parameters - MQ III

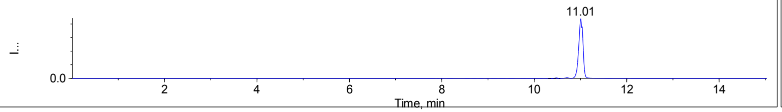

Sample Name: "ZW-3" Sample ID: "" File: "20250515-50AA.wiff"
Peak Name: "MSLAB-Nvl(IS)" Mass(es): "288.150/171.010 Da"
Comment: "" Annotation: ""

Sample Index: 38
Sample Type: Unknown
Concentration: 1.00 ng/mL
Calculated Conc: N/A
Acq. Date: 2025-05-16
Acq. Time: 00:19:08

Modified: Yes
Proc. Algorithm: Specify Parameters - MQ III

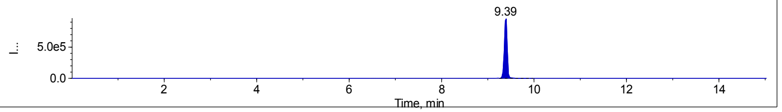

Sample Name: "ZW-3" Sample ID: "" File: "20250515-50AA.wiff"
Peak Name: "Asn" Mass(es): "303.130/171.010 Da"
Comment: "" Annotation: ""

Sample Index: 38
Sample Type: Unknown
Concentration: N/A
Calculated Conc: < 0
Acq. Date: 2025-05-16
Acq. Time: 00:19:08

Modified: Yes
Proc. Algorithm: Specify Parameters - MQ III

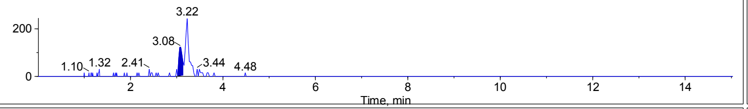

Sample Name: "ZW-3" Sample ID: "" File: "20250515-50AA.wiff"
Peak Name: "MSLAB-Nvl(IS)" Mass(es): "288.150/171.010 Da"
Comment: "" Annotation: ""

Sample Index: 38
Sample Type: Unknown
Concentration: 1.00 ng/mL
Calculated Conc: N/A
Acq. Date: 2025-05-16
Acq. Time: 00:19:08

Modified: Yes
Proc. Algorithm: Specify Parameters - MQ III

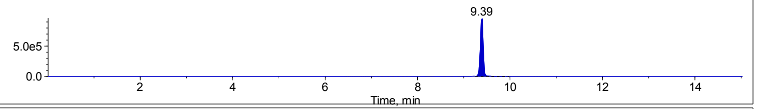

Sample Name: "ZW-3" Sample ID: "" File: "20250515-50AA.wiff"
Peak Name: "Asp" Mass(es): "304.110/171.010 Da"
Comment: "" Annotation: ""

Sample Index: 38
Sample Type: Unknown
Concentration: N/A
Calculated Conc: No Intercept
Acq. Date: 2025-05-16
Acq. Time: 00:19:08

Modified: Yes
Proc. Algorithm: Specify Parameters - MQ III

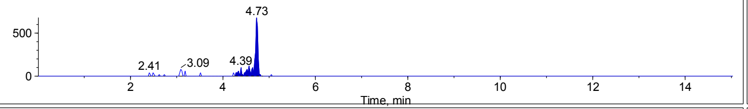

Sample Name: "ZW-3" Sample ID: "" File: "20250515-50AA.wiff"
Peak Name: "MSLAB-Nvl(IS)" Mass(es): "288.150/171.010 Da"
Comment: "" Annotation: ""

Sample Index: 38
Sample Type: Unknown
Concentration: 1.00 ng/mL
Calculated Conc: N/A
Acq. Date: 2025-05-16
Acq. Time: 00:19:08

Modified: Yes
Proc. Algorithm: Specify Parameters - MQ III

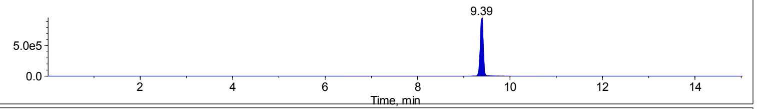

Sample Name: "ZW-3" Sample ID: "" File: "20250515-50AA.wiff"
Peak Name: "Gln" Mass(es): "317.150/171.010 Da"
Comment: "" Annotation: ""

Sample Index: 38
Sample Type: Unknown
Concentration: N/A
Calculated Conc: No Intercept
Acq. Date: 2025-05-16
Acq. Time: 00:19:08

Modified: Yes
Proc. Algorithm: Specify Parameters - MQ III

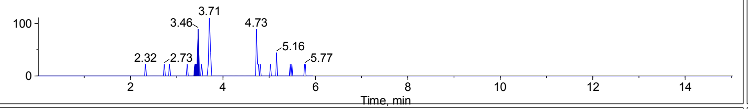

Sample Name: "ZW-3" Sample ID: "" File: "20250515-50AA.wiff"
Peak Name: "MSLAB-Nvl(IS)" Mass(es): "288.150/171.010 Da"
Comment: "" Annotation: ""

Sample Index: 38
Sample Type: Unknown
Concentration: 1.00 ng/mL
Calculated Conc: N/A
Acq. Date: 2025-05-16
Acq. Time: 00:19:08

Modified: Yes
Proc. Algorithm: Specify Parameters - MQ III

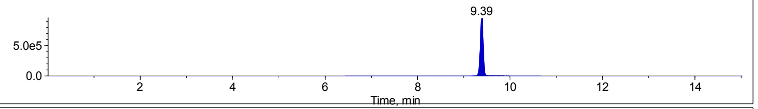

Sample Name: "ZW-3" Sample ID: "" File: "20250515-50AA.wiff"
Peak Name: "Glu" Mass(es): "318.100/171.010 Da"
Comment: "" Annotation: ""

Sample Index: 38
Sample Type: Unknown
Concentration: N/A
Calculated Conc: 1.29 umol/L
Acq. Date: 2025-05-16
Acq. Time: 00:19:08

Modified: Yes
Proc. Algorithm: Specify Parameters - MQ III

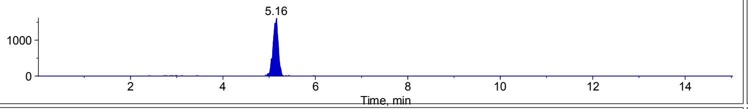

Sample Name: "ZW-3" Sample ID: "" File: "20250515-50AA.wiff"
Peak Name: "MSLAB-Nvl(IS)" Mass(es): "288.150/171.010 Da"
Comment: "" Annotation: ""

Sample Index: 38
Sample Type: Unknown
Concentration: 1.00 ng/mL
Calculated Conc: N/A
Acq. Date: 2025-05-16
Acq. Time: 00:19:08

Modified: Yes
Proc. Algorithm: Specify Parameters - MQ III

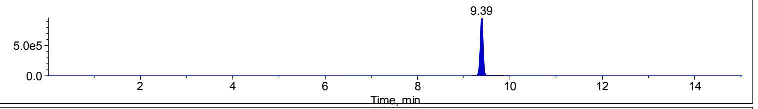

Sample Name: "ZW-3" Sample ID: "" File: "20250515-50AA.wiff"
Peak Name: "Met" Mass(es): "320.210/171.010 Da"
Comment: "" Annotation: ""

Sample Index: 38
Sample Type: Unknown
Concentration: N/A
Calculated Conc: < 0
Acq. Date: 2025-05-16
Acq. Time: 00:19:08

Modified: Yes
Proc. Algorithm: Specify Parameters - MQ III

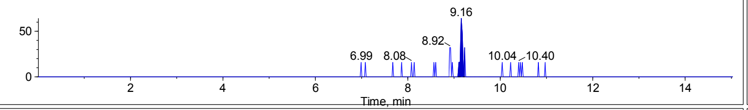

Sample Name: "ZW-3" Sample ID: "" File: "20250515-50AA.wiff"
Peak Name: "MSLAB-Nvl(IS)" Mass(es): "288.150/171.010 Da"
Comment: "" Annotation: ""

Sample Index: 38
Sample Type: Unknown
Concentration: 1.00 ng/mL
Calculated Conc: N/A
Acq. Date: 2025-05-16
Acq. Time: 00:19:08

Modified: Yes
Proc. Algorithm: Specify Parameters - MQ III

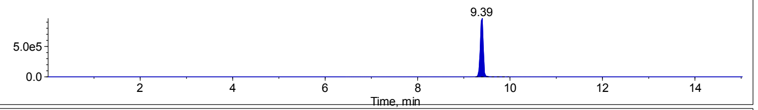

Sample Name: "ZW-3" Sample ID: "" File: "20250515-50AA.wiff"
Peak Name: "His" Mass(es): "326.150/171.010 Da"
Comment: "" Annotation: ""

Sample Index: 38
Sample Type: Unknown
Concentration: N/A
Calculated Conc: 0.400 umol/L
Acq. Date: 2025-05-16
Acq. Time: 00:19:08

Modified: Yes
Proc. Algorithm: Specify Parameters - MQ III

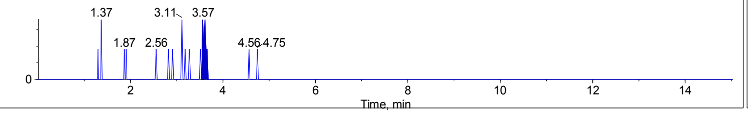

Sample Name: "ZW-3" Sample ID: "" File: "20250515-50AA.wiff"
Peak Name: "MSLAB-Nvl(IS)" Mass(es): "288.150/171.010 Da"
Comment: "" Annotation: ""

Sample Index: 38
Sample Type: Unknown
Concentration: 1.00 ng/mL
Calculated Conc: N/A
Acq. Date: 2025-05-16
Acq. Time: 00:19:08

Modified: Yes
Proc. Algorithm: Specify Parameters - MQ III

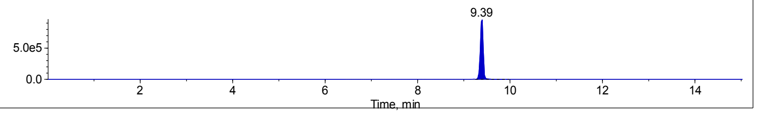

Sample Name: "ZW-3" Sample ID: "" File: "20250515-50AA.wiff"
Peak Name: "Phe" Mass(es): "336.200/171.010 Da"
Comment: "" Annotation: ""

```
Sample Index:       38
Sample Type:      Unknown
Concentration:       N/A
Calculated Conc:    0.402   umol/L
Acq. Date:        2025-05-16
Acq. Time:        00:19:08

Modified:            Yes
Proc. Algorithm: Specify Parameters - MQ III
```

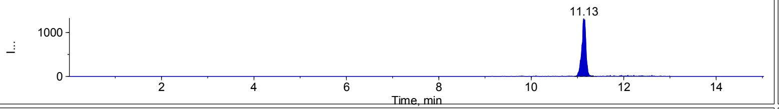

Sample Name: "ZW-3" Sample ID: "" File: "20250515-50AA.wiff"
Peak Name: "Arg" Mass(es): "345.210/171.010 Da"
Comment: "" Annotation: ""

```
Sample Index:       38
Sample Type:      Unknown
Concentration:       N/A
Calculated Conc:    1.88    umol/L
Acq. Date:        2025-05-16
Acq. Time:        00:19:08

Modified:            Yes
Proc. Algorithm: Specify Parameters - MQ III
```

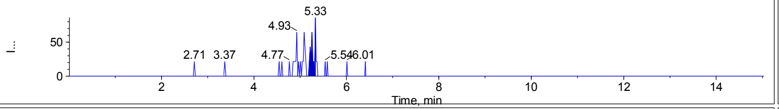

Sample Name: "ZW-3" Sample ID: "" File: "20250515-50AA.wiff"
Peak Name: "Trp" Mass(es): "375.200/171.010 Da"
Comment: "" Annotation: ""

```
Sample Index:       38
Sample Type:      Unknown
Concentration:       N/A
Calculated Conc:      < 0
Acq. Date:        2025-05-16
Acq. Time:        00:19:08

Modified:            Yes
Proc. Algorithm: Specify Parameters - MQ III
```

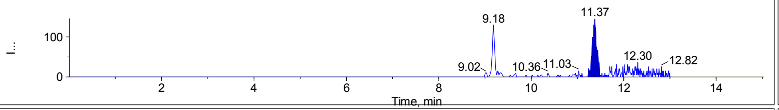

Sample Name: "ZW-3" Sample ID: "" File: "20250515-50AA.wiff"
Peak Name: "Lys" Mass(es): "487.200/171.010 Da"
Comment: "" Annotation: ""

```
Sample Index:       38
Sample Type:      Unknown
Concentration:       N/A
Calculated Conc:    1.69    umol/L
Acq. Date:        2025-05-16
Acq. Time:        00:19:08

Modified:            Yes
Proc. Algorithm: Specify Parameters - MQ III
```

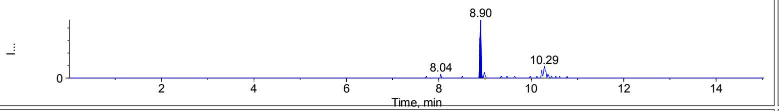

Sample Name: "ZW-3" Sample ID: "" File: "20250515-50AA.wiff"
Peak Name: "tyr" Mass(es): "352.200/171.010 Da"
Comment: "" Annotation: ""

```
Sample Index:       38
Sample Type:      Unknown
Concentration:       N/A
Calculated Conc: No Intercept
Acq. Date:        2025-05-16
Acq. Time:        00:19:08

Modified:            Yes
Proc. Algorithm: Specify Parameters - MQ III
```

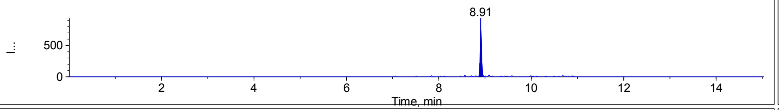

Sample Name: "ZW-3" Sample ID: "" File: "20250515-50AA.wiff"
Peak Name: "Leu" Mass(es): "302.150/171.010 Da"
Comment: "" Annotation: ""

```
Sample Index:       38
Sample Type:      Unknown
Concentration:       N/A
Calculated Conc:    0.577   umol/L
Acq. Date:        2025-05-16
Acq. Time:        00:19:08

Modified:            Yes
Proc. Algorithm: Specify Parameters - MQ III
```

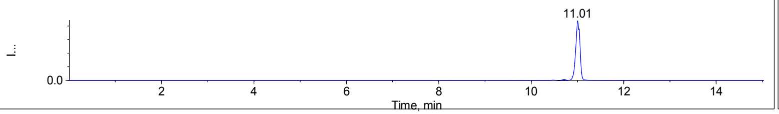

Sample Name: "ZW-3" Sample ID: "" File: "20250515-50AA.wiff"
Peak Name: "MSLAB-Nvl(IS)" Mass(es): "288.150/171.010 Da"
Comment: "" Annotation: ""

```
Sample Index:       38
Sample Type:      Unknown
Concentration:      1.00    ng/mL
Calculated Conc:     N/A
Acq. Date:        2025-05-16
Acq. Time:        00:19:08

Modified:            Yes
Proc. Algorithm: Specify Parameters - MQ III
```

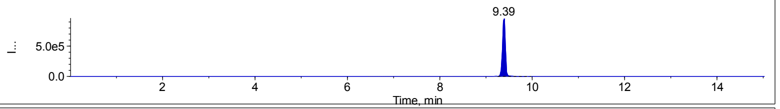

Sample Name: "ZW-3" Sample ID: "" File: "20250515-50AA.wiff"
Peak Name: "MSLAB-Nvl(IS)" Mass(es): "288.150/171.010 Da"
Comment: "" Annotation: ""

```
Sample Index:       38
Sample Type:      Unknown
Concentration:      1.00    ng/mL
Calculated Conc:     N/A
Acq. Date:        2025-05-16
Acq. Time:        00:19:08

Modified:            Yes
Proc. Algorithm: Specify Parameters - MQ III
```

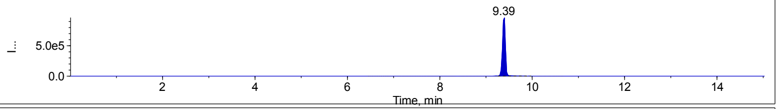

Sample Name: "ZW-3" Sample ID: "" File: "20250515-50AA.wiff"
Peak Name: "MSLAB-Nvl(IS)" Mass(es): "288.150/171.010 Da"
Comment: "" Annotation: ""

```
Sample Index:       38
Sample Type:      Unknown
Concentration:      1.00    ng/mL
Calculated Conc:     N/A
Acq. Date:        2025-05-16
Acq. Time:        00:19:08

Modified:            Yes
Proc. Algorithm: Specify Parameters - MQ III
```

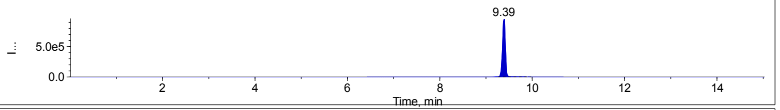

Sample Name: "ZW-3" Sample ID: "" File: "20250515-50AA.wiff"
Peak Name: "MSLAB-Nvl(IS)" Mass(es): "288.150/171.010 Da"
Comment: "" Annotation: ""

```
Sample Index:       38
Sample Type:      Unknown
Concentration:      1.00    ng/mL
Calculated Conc:     N/A
Acq. Date:        2025-05-16
Acq. Time:        00:19:08

Modified:            Yes
Proc. Algorithm: Specify Parameters - MQ III
```

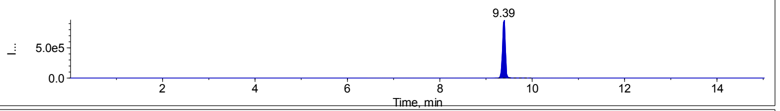

Sample Name: "ZW-3" Sample ID: "" File: "20250515-50AA.wiff"
Peak Name: "MSLAB-Nvl(IS)" Mass(es): "288.150/171.010 Da"
Comment: "" Annotation: ""

```
Sample Index:       38
Sample Type:      Unknown
Concentration:      1.00    ng/mL
Calculated Conc:     N/A
Acq. Date:        2025-05-16
Acq. Time:        00:19:08

Modified:            Yes
Proc. Algorithm: Specify Parameters - MQ III
```

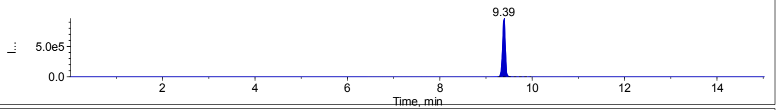

Sample Name: "ZW-3" Sample ID: "" File: "20250515-50AA.wiff"
Peak Name: "MSLAB-Nvl(IS)" Mass(es): "288.150/171.010 Da"
Comment: "" Annotation: ""

```
Sample Index:       38
Sample Type:      Unknown
Concentration:      1.00    ng/mL
Calculated Conc:     N/A
Acq. Date:        2025-05-16
Acq. Time:        00:19:08

Modified:            Yes
Proc. Algorithm: Specify Parameters - MQ III
```

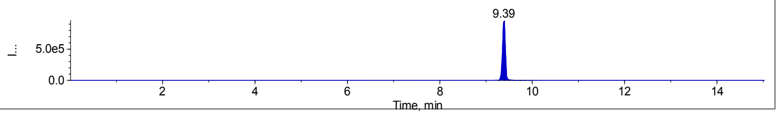

Sample Name: "ZW-9" Sample ID: "" File: "20250515-50AA.wiff"
Peak Name: "Gly" Mass(es): "246.080/171.010 Da"
Comment: "" Annotation: ""

```
Sample Index:       39
Sample Type:        Unknown
Concentration:      N/A
Calculated Conc:    1.91   umol/L
Acq. Date:          2025-05-16
Acq. Time:          00:34:37

Modified:           Yes
Proc. Algorithm: Specify Parameters - MQ III
```

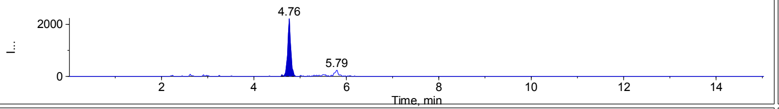

Sample Name: "ZW-9" Sample ID: "" File: "20250515-50AA.wiff"
Peak Name: "Ala" Mass(es): "260.100/171.010 Da"
Comment: "" Annotation: ""

```
Sample Index:       39
Sample Type:        Unknown
Concentration:      N/A
Calculated Conc: No Intercept
Acq. Date:          2025-05-16
Acq. Time:          00:34:37

Modified:           Yes
Proc. Algorithm: Specify Parameters - MQ III
```

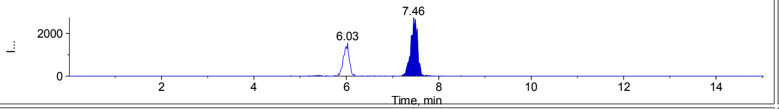

Sample Name: "ZW-9" Sample ID: "" File: "20250515-50AA.wiff"
Peak Name: "Ser" Mass(es): "276.100/171.010 Da"
Comment: "" Annotation: ""

```
Sample Index:       39
Sample Type:        Unknown
Concentration:      N/A
Calculated Conc:      < 0
Acq. Date:          2025-05-16
Acq. Time:          00:34:37

Modified:           Yes
Proc. Algorithm: Specify Parameters - MQ III
```

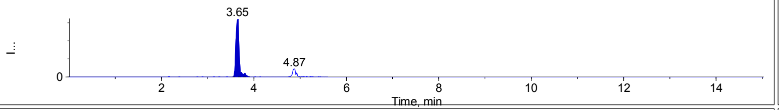

Sample Name: "ZW-9" Sample ID: "" File: "20250515-50AA.wiff"
Peak Name: "Pro" Mass(es): "286.150/171.010 Da"
Comment: "" Annotation: ""

```
Sample Index:       39
Sample Type:        Unknown
Concentration:      N/A
Calculated Conc: No Intercept
Acq. Date:          2025-05-16
Acq. Time:          00:34:37

Modified:           Yes
Proc. Algorithm: Specify Parameters - MQ III
```

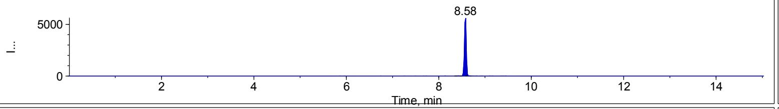

Sample Name: "ZW-9" Sample ID: "" File: "20250515-50AA.wiff"
Peak Name: "Val" Mass(es): "288.150/171.010 Da"
Comment: "" Annotation: ""

```
Sample Index:       39
Sample Type:        Unknown
Concentration:      N/A
Calculated Conc:    2.30   umol/L
Acq. Date:          2025-05-16
Acq. Time:          00:34:37

Modified:           Yes
Proc. Algorithm: Specify Parameters - MQ III
```

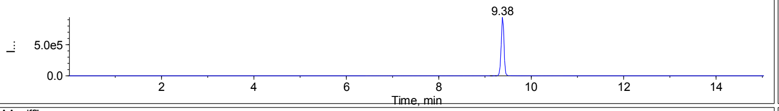

Sample Name: "ZW-9" Sample ID: "" File: "20250515-50AA.wiff"
Peak Name: "Thr" Mass(es): "290.100/171.010 Da"
Comment: "" Annotation: ""

```
Sample Index:       39
Sample Type:        Unknown
Concentration:      N/A
Calculated Conc:    1.50   umol/L
Acq. Date:          2025-05-16
Acq. Time:          00:34:37

Modified:           Yes
Proc. Algorithm: Specify Parameters - MQ III
```

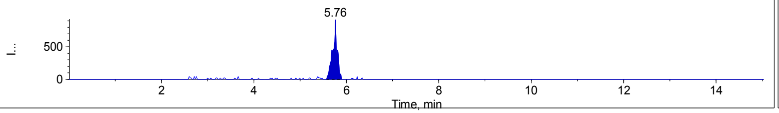

Sample Name: "ZW-9" Sample ID: "" File: "20250515-50AA.wiff"
Peak Name: "MSLAB-Nvl(IS)" Mass(es): "288.150/171.010 Da"
Comment: "" Annotation: ""

```
Sample Index:       39
Sample Type:        Unknown
Concentration:      1.00    ng/mL
Calculated Conc:    N/A
Acq. Date:          2025-05-16
Acq. Time:          00:34:37

Modified:           Yes
Proc. Algorithm: Specify Parameters - MQ III
```

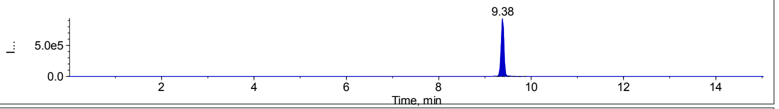

Sample Name: "ZW-9" Sample ID: "" File: "20250515-50AA.wiff"
Peak Name: "MSLAB-Nvl(IS)" Mass(es): "288.150/171.010 Da"
Comment: "" Annotation: ""

```
Sample Index:       39
Sample Type:        Unknown
Concentration:      1.00    ng/mL
Calculated Conc:    N/A
Acq. Date:          2025-05-16
Acq. Time:          00:34:37

Modified:           Yes
Proc. Algorithm: Specify Parameters - MQ III
```

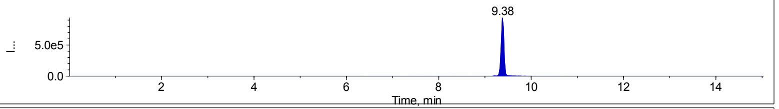

Sample Name: "ZW-9" Sample ID: "" File: "20250515-50AA.wiff"
Peak Name: "MSLAB-Nvl(IS)" Mass(es): "288.150/171.010 Da"
Comment: "" Annotation: ""

```
Sample Index:       39
Sample Type:        Unknown
Concentration:      1.00    ng/mL
Calculated Conc:    N/A
Acq. Date:          2025-05-16
Acq. Time:          00:34:37

Modified:           Yes
Proc. Algorithm: Specify Parameters - MQ III
```

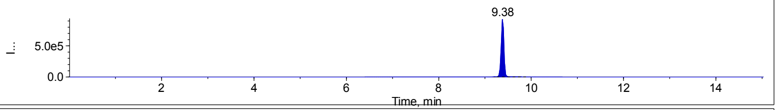

Sample Name: "ZW-9" Sample ID: "" File: "20250515-50AA.wiff"
Peak Name: "MSLAB-Nvl(IS)" Mass(es): "288.150/171.010 Da"
Comment: "" Annotation: ""

```
Sample Index:       39
Sample Type:        Unknown
Concentration:      1.00    ng/mL
Calculated Conc:    N/A
Acq. Date:          2025-05-16
Acq. Time:          00:34:37

Modified:           Yes
Proc. Algorithm: Specify Parameters - MQ III
```

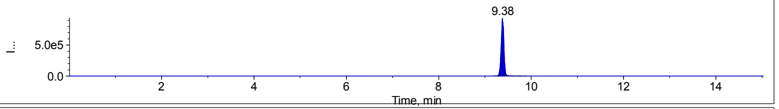

Sample Name: "ZW-9" Sample ID: "" File: "20250515-50AA.wiff"
Peak Name: "MSLAB-Nvl(IS)" Mass(es): "288.150/171.010 Da"
Comment: "" Annotation: ""

```
Sample Index:       39
Sample Type:        Unknown
Concentration:      1.00    ng/mL
Calculated Conc:    N/A
Acq. Date:          2025-05-16
Acq. Time:          00:34:37

Modified:           Yes
Proc. Algorithm: Specify Parameters - MQ III
```

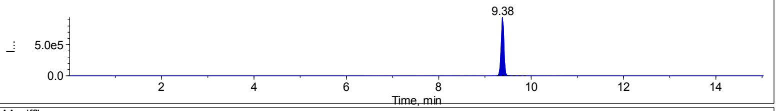

Sample Name: "ZW-9" Sample ID: "" File: "20250515-50AA.wiff"
Peak Name: "MSLAB-Nvl(IS)" Mass(es): "288.150/171.010 Da"
Comment: "" Annotation: ""

```
Sample Index:       39
Sample Type:        Unknown
Concentration:      1.00    ng/mL
Calculated Conc:    N/A
Acq. Date:          2025-05-16
Acq. Time:          00:34:37

Modified:           Yes
Proc. Algorithm: Specify Parameters - MQ III
```

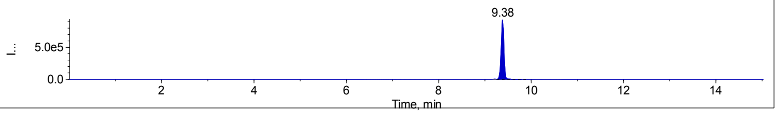

Sample Name: "ZW-9" Sample ID: "" File: "20250515-50AA.wiff"
Peak Name: "Cys1/2" Mass(es): "292.200/171.010 Da"
Comment: "" Annotation: ""

Sample Index: 39
Sample Type: Unknown
Concentration: N/A
Calculated Conc: 0.220 umol/L
Acq. Date: 2025-05-16
Acq. Time: 00:34:37

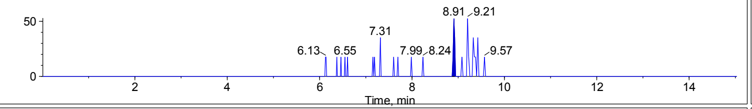

Modified: Yes
Proc. Algorithm: Specify Parameters - MQ III

Sample Name: "ZW-9" Sample ID: "" File: "20250515-50AA.wiff"
Peak Name: "Ile" Mass(es): "302.150/171.010 Da"
Comment: "" Annotation: ""

Sample Index: 39
Sample Type: Unknown
Concentration: N/A
Calculated Conc: 0.447 umol/L
Acq. Date: 2025-05-16
Acq. Time: 00:34:37

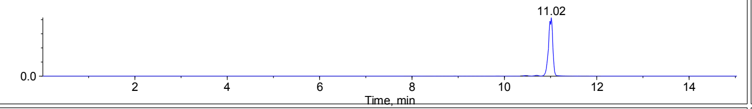

Modified: Yes
Proc. Algorithm: Specify Parameters - MQ III

Sample Name: "ZW-9" Sample ID: "" File: "20250515-50AA.wiff"
Peak Name: "Asn" Mass(es): "303.130/171.010 Da"
Comment: "" Annotation: ""

Sample Index: 39
Sample Type: Unknown
Concentration: N/A
Calculated Conc: < 0
Acq. Date: 2025-05-16
Acq. Time: 00:34:37

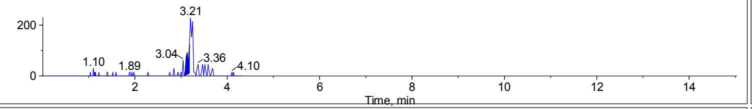

Modified: Yes
Proc. Algorithm: Specify Parameters - MQ III

Sample Name: "ZW-9" Sample ID: "" File: "20250515-50AA.wiff"
Peak Name: "Asp" Mass(es): "304.110/171.010 Da"
Comment: "" Annotation: ""

Sample Index: 39
Sample Type: Unknown
Concentration: N/A
Calculated Conc: No Intercept
Acq. Date: 2025-05-16
Acq. Time: 00:34:37

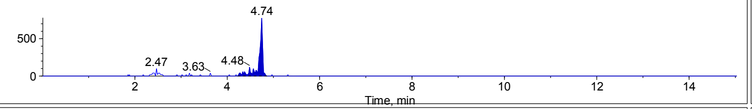

Modified: Yes
Proc. Algorithm: Specify Parameters - MQ III

Sample Name: "ZW-9" Sample ID: "" File: "20250515-50AA.wiff"
Peak Name: "Gln" Mass(es): "317.150/171.010 Da"
Comment: "" Annotation: ""

Sample Index: 39
Sample Type: Unknown
Concentration: N/A
Calculated Conc: No Intercept
Acq. Date: 2025-05-16
Acq. Time: 00:34:37

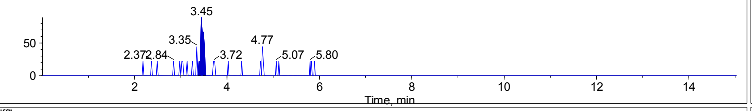

Modified: Yes
Proc. Algorithm: Specify Parameters - MQ III

Sample Name: "ZW-9" Sample ID: "" File: "20250515-50AA.wiff"
Peak Name: "Glu" Mass(es): "318.100/171.010 Da"
Comment: "" Annotation: ""

Sample Index: 39
Sample Type: Unknown
Concentration: N/A
Calculated Conc: 2.19 umol/L
Acq. Date: 2025-05-16
Acq. Time: 00:34:37

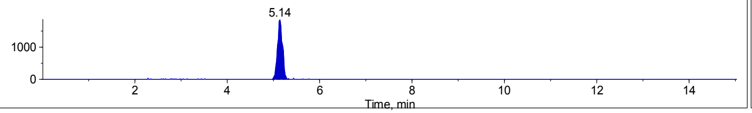

Modified: Yes
Proc. Algorithm: Specify Parameters - MQ III

Sample Name: "ZW-9" Sample ID: "" File: "20250515-50AA.wiff"
Peak Name: "MSLAB-Nvl(IS)" Mass(es): "288.150/171.010 Da"
Comment: "" Annotation: ""

Sample Index: 39
Sample Type: Unknown
Concentration: 1.00 ng/mL
Calculated Conc: N/A
Acq. Date: 2025-05-16
Acq. Time: 00:34:37

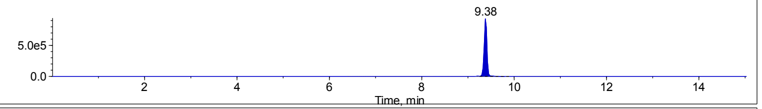

Modified: Yes
Proc. Algorithm: Specify Parameters - MQ III

Sample Name: "ZW-9" Sample ID: "" File: "20250515-50AA.wiff"
Peak Name: "MSLAB-Nvl(IS)" Mass(es): "288.150/171.010 Da"
Comment: "" Annotation: ""

Sample Index: 39
Sample Type: Unknown
Concentration: 1.00 ng/mL
Calculated Conc: N/A
Acq. Date: 2025-05-16
Acq. Time: 00:34:37

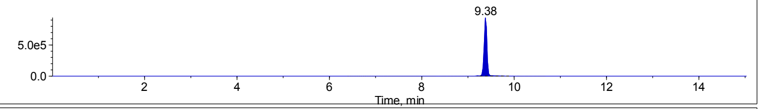

Modified: Yes
Proc. Algorithm: Specify Parameters - MQ III

Sample Name: "ZW-9" Sample ID: "" File: "20250515-50AA.wiff"
Peak Name: "MSLAB-Nvl(IS)" Mass(es): "288.150/171.010 Da"
Comment: "" Annotation: ""

Sample Index: 39
Sample Type: Unknown
Concentration: 1.00 ng/mL
Calculated Conc: N/A
Acq. Date: 2025-05-16
Acq. Time: 00:34:37

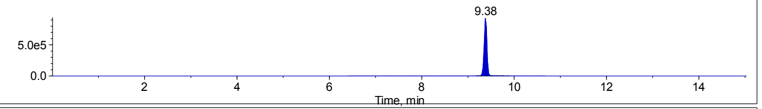

Modified: Yes
Proc. Algorithm: Specify Parameters - MQ III

Sample Name: "ZW-9" Sample ID: "" File: "20250515-50AA.wiff"
Peak Name: "MSLAB-Nvl(IS)" Mass(es): "288.150/171.010 Da"
Comment: "" Annotation: ""

Sample Index: 39
Sample Type: Unknown
Concentration: 1.00 ng/mL
Calculated Conc: N/A
Acq. Date: 2025-05-16
Acq. Time: 00:34:37

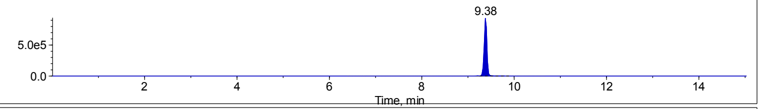

Modified: Yes
Proc. Algorithm: Specify Parameters - MQ III

Sample Name: "ZW-9" Sample ID: "" File: "20250515-50AA.wiff"
Peak Name: "MSLAB-Nvl(IS)" Mass(es): "288.150/171.010 Da"
Comment: "" Annotation: ""

Sample Index: 39
Sample Type: Unknown
Concentration: 1.00 ng/mL
Calculated Conc: N/A
Acq. Date: 2025-05-16
Acq. Time: 00:34:37

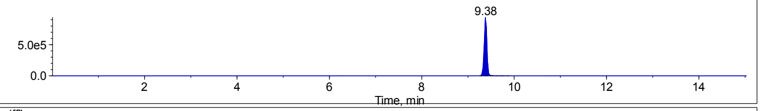

Modified: Yes
Proc. Algorithm: Specify Parameters - MQ III

Sample Name: "ZW-9" Sample ID: "" File: "20250515-50AA.wiff"
Peak Name: "MSLAB-Nvl(IS)" Mass(es): "288.150/171.010 Da"
Comment: "" Annotation: ""

Sample Index: 39
Sample Type: Unknown
Concentration: 1.00 ng/mL
Calculated Conc: N/A
Acq. Date: 2025-05-16
Acq. Time: 00:34:37

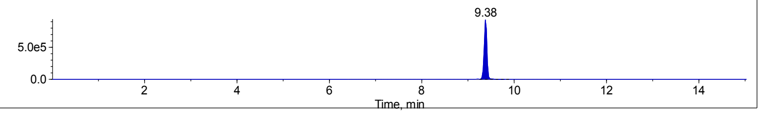

Modified: Yes
Proc. Algorithm: Specify Parameters - MQ III

Sample Name: "ZW-9" Sample ID: "" File: "20250515-50AA.wiff"
Peak Name: "Met" Mass(es): "320.210/171.010 Da"
Comment: "" Annotation: ""

```
Sample Index:        39
Sample Type:         Unknown
Concentration:       N/A
Calculated Conc:     < 0
Acq. Date:           2025-05-16
Acq. Time:           00:34:37
```

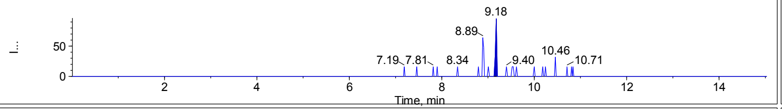


```
Modified:            Yes
Proc. Algorithm: Specify Parameters - MQ III
```

Sample Name: "ZW-9" Sample ID: "" File: "20250515-50AA.wiff"
Peak Name: "MSLAB-Nvl(IS)" Mass(es): "288.150/171.010 Da"
Comment: "" Annotation: ""

```
Sample Index:        39
Sample Type:         Unknown
Concentration:       1.00    ng/mL
Calculated Conc:     N/A
Acq. Date:           2025-05-16
Acq. Time:           00:34:37
```

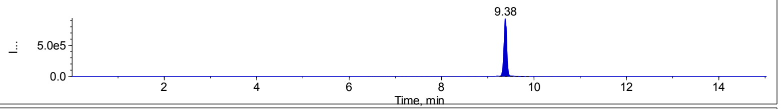


```
Modified:            Yes
Proc. Algorithm: Specify Parameters - MQ III
```

Sample Name: "ZW-9" Sample ID: "" File: "20250515-50AA.wiff"
Peak Name: "His" Mass(es): "326.150/171.010 Da"
Comment: "" Annotation: ""

```
Sample Index:        39
Sample Type:         Unknown
Concentration:       N/A
Calculated Conc:     0.645   umol/L
Acq. Date:           2025-05-16
Acq. Time:           00:34:37
```

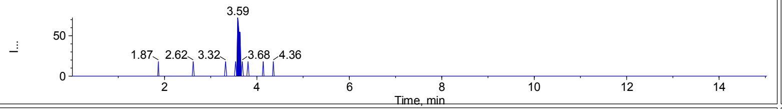


```
Modified:            Yes
Proc. Algorithm: Specify Parameters - MQ III
```

Sample Name: "ZW-9" Sample ID: "" File: "20250515-50AA.wiff"
Peak Name: "MSLAB-Nvl(IS)" Mass(es): "288.150/171.010 Da"
Comment: "" Annotation: ""

```
Sample Index:        39
Sample Type:         Unknown
Concentration:       1.00    ng/mL
Calculated Conc:     N/A
Acq. Date:           2025-05-16
Acq. Time:           00:34:37
```

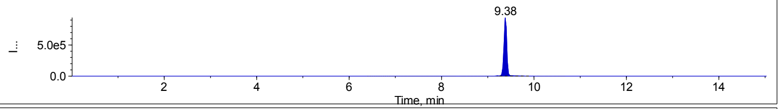


```
Modified:            Yes
Proc. Algorithm: Specify Parameters - MQ III
```

Sample Name: "ZW-9" Sample ID: "" File: "20250515-50AA.wiff"
Peak Name: "Phe" Mass(es): "336.200/171.010 Da"
Comment: "" Annotation: ""

```
Sample Index:        39
Sample Type:         Unknown
Concentration:       N/A
Calculated Conc:     0.670   umol/L
Acq. Date:           2025-05-16
Acq. Time:           00:34:37
```

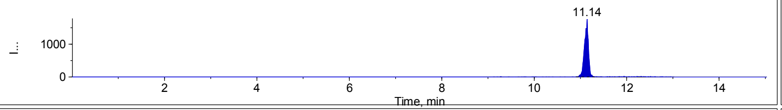


```
Modified:            Yes
Proc. Algorithm: Specify Parameters - MQ III
```

Sample Name: "ZW-9" Sample ID: "" File: "20250515-50AA.wiff"
Peak Name: "MSLAB-Nvl(IS)" Mass(es): "288.150/171.010 Da"
Comment: "" Annotation: ""

```
Sample Index:        39
Sample Type:         Unknown
Concentration:       1.00    ng/mL
Calculated Conc:     N/A
Acq. Date:           2025-05-16
Acq. Time:           00:34:37
```

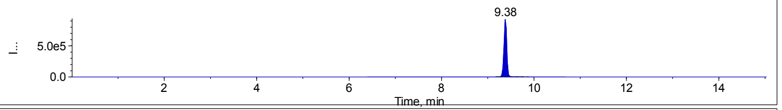


```
Modified:            Yes
Proc. Algorithm: Specify Parameters - MQ III
```

Sample Name: "ZW-9" Sample ID: "" File: "20250515-50AA.wiff"
Peak Name: "Arg" Mass(es): "345.210/171.010 Da"
Comment: "" Annotation: ""

```
Sample Index:        39
Sample Type:         Unknown
Concentration:       N/A
Calculated Conc:     2.16    umol/L
Acq. Date:           2025-05-16
Acq. Time:           00:34:37
```

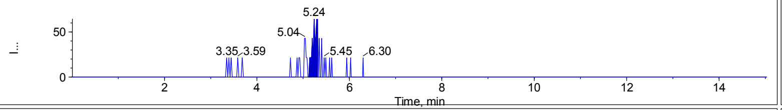


```
Modified:            Yes
Proc. Algorithm: Specify Parameters - MQ III
```

Sample Name: "ZW-9" Sample ID: "" File: "20250515-50AA.wiff"
Peak Name: "MSLAB-Nvl(IS)" Mass(es): "288.150/171.010 Da"
Comment: "" Annotation: ""

```
Sample Index:        39
Sample Type:         Unknown
Concentration:       1.00    ng/mL
Calculated Conc:     N/A
Acq. Date:           2025-05-16
Acq. Time:           00:34:37
```

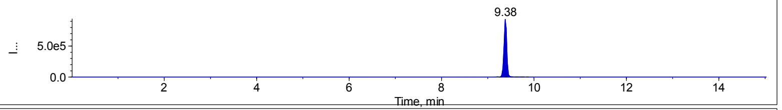


```
Modified:            Yes
Proc. Algorithm: Specify Parameters - MQ III
```

Sample Name: "ZW-9" Sample ID: "" File: "20250515-50AA.wiff"
Peak Name: "Trp" Mass(es): "375.200/171.010 Da"
Comment: "" Annotation: ""

```
Sample Index:        39
Sample Type:         Unknown
Concentration:       N/A
Calculated Conc:     < 0
Acq. Date:           2025-05-16
Acq. Time:           00:34:37
```

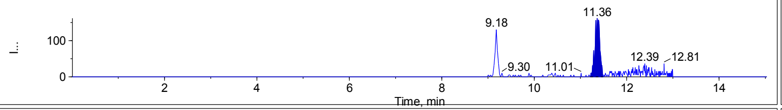


```
Modified:            Yes
Proc. Algorithm: Specify Parameters - MQ III
```

Sample Name: "ZW-9" Sample ID: "" File: "20250515-50AA.wiff"
Peak Name: "MSLAB-Nvl(IS)" Mass(es): "288.150/171.010 Da"
Comment: "" Annotation: ""

```
Sample Index:        39
Sample Type:         Unknown
Concentration:       1.00    ng/mL
Calculated Conc:     N/A
Acq. Date:           2025-05-16
Acq. Time:           00:34:37
```

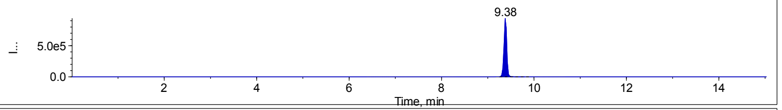


```
Modified:            Yes
Proc. Algorithm: Specify Parameters - MQ III
```

Sample Name: "ZW-9" Sample ID: "" File: "20250515-50AA.wiff"
Peak Name: "Lys" Mass(es): "487.200/171.010 Da"
Comment: "" Annotation: ""

```
Sample Index:        39
Sample Type:         Unknown
Concentration:       N/A
Calculated Conc:     1.89    umol/L
Acq. Date:           2025-05-16
Acq. Time:           00:34:37
```

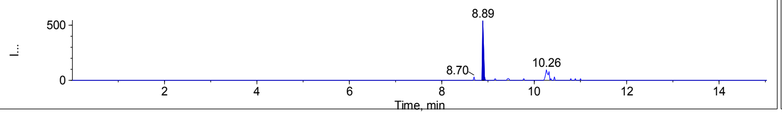


```
Modified:            Yes
Proc. Algorithm: Specify Parameters - MQ III
```

Sample Name: "ZW-9" Sample ID: "" File: "20250515-50AA.wiff"
Peak Name: "MSLAB-Nvl(IS)" Mass(es): "288.150/171.010 Da"
Comment: "" Annotation: ""

```
Sample Index:        39
Sample Type:         Unknown
Concentration:       1.00    ng/mL
Calculated Conc:     N/A
Acq. Date:           2025-05-16
Acq. Time:           00:34:37
```

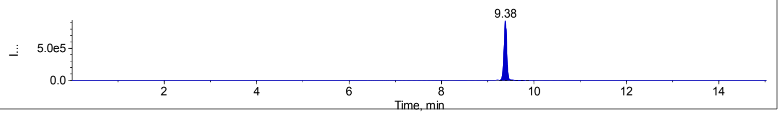


```
Modified:            Yes
Proc. Algorithm: Specify Parameters - MQ III
```

Sample Name: "ZW-9" Sample ID: "" File: "20250515-50AA.wiff"
Peak Name: "tyr" Mass(es): "352.200/171.010 Da"
Comment: "" Annotation: ""
Sample Index: 39
Sample Type: Unknown
Concentration: N/A
Calculated Conc: No Intercept
Acq. Date: 2025-05-16
Acq. Time: 00:34:37
Modified: Yes
Proc. Algorithm: Specify Parameters - MQ III

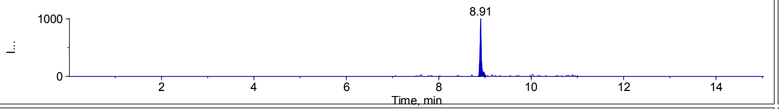

Sample Name: "ZW-9" Sample ID: "" File: "20250515-50AA.wiff"
Peak Name: "MSLAB-Nvl(IS)" Mass(es): "288.150/171.010 Da"
Comment: "" Annotation: ""
Sample Index: 39
Sample Type: Unknown
Concentration: 1.00 ng/mL
Calculated Conc: N/A
Acq. Date: 2025-05-16
Acq. Time: 00:34:37
Modified: Yes
Proc. Algorithm: Specify Parameters - MQ III

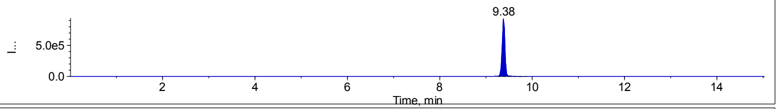

Sample Name: "ZW-9" Sample ID: "" File: "20250515-50AA.wiff"
Peak Name: "Leu" Mass(es): "302.150/171.010 Da"
Comment: "" Annotation: ""
Sample Index: 39
Sample Type: Unknown
Concentration: N/A
Calculated Conc: 1.36 umol/L
Acq. Date: 2025-05-16
Acq. Time: 00:34:37
Modified: Yes
Proc. Algorithm: Specify Parameters - MQ III

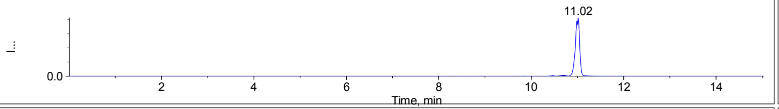

Sample Name: "ZW-9" Sample ID: "" File: "20250515-50AA.wiff"
Peak Name: "MSLAB-Nvl(IS)" Mass(es): "288.150/171.010 Da"
Comment: "" Annotation: ""
Sample Index: 39
Sample Type: Unknown
Concentration: 1.00 ng/mL
Calculated Conc: N/A
Acq. Date: 2025-05-16
Acq. Time: 00:34:37
Modified: Yes
Proc. Algorithm: Specify Parameters - MQ III

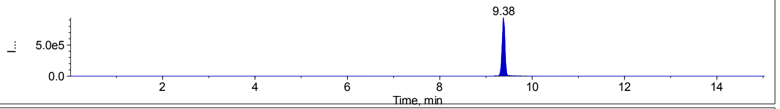

Sample Name: "ZW-10" Sample ID: "" File: "20250515-50AA.wiff"
Peak Name: "Gly" Mass(es): "246.080/171.010 Da"
Comment: "" Annotation: ""
Sample Index: 40
Sample Type: Unknown
Concentration: N/A
Calculated Conc: 11.3 umol/L
Acq. Date: 2025-05-16
Acq. Time: 00:50:06
Modified: Yes
Proc. Algorithm: Specify Parameters - MQ III

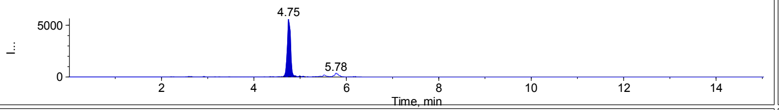

Sample Name: "ZW-10" Sample ID: "" File: "20250515-50AA.wiff"
Peak Name: "MSLAB-Nvl(IS)" Mass(es): "288.150/171.010 Da"
Comment: "" Annotation: ""
Sample Index: 40
Sample Type: Unknown
Concentration: 1.00 ng/mL
Calculated Conc: N/A
Acq. Date: 2025-05-16
Acq. Time: 00:50:06
Modified: Yes
Proc. Algorithm: Specify Parameters - MQ III

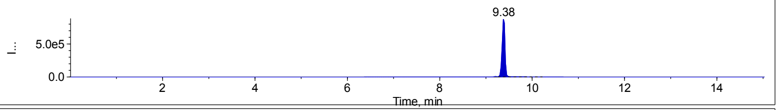

Sample Name: "ZW-10" Sample ID: "" File: "20250515-50AA.wiff"
Peak Name: "Ala" Mass(es): "260.100/171.010 Da"
Comment: "" Annotation: ""
Sample Index: 40
Sample Type: Unknown
Concentration: N/A
Calculated Conc: No Intercept
Acq. Date: 2025-05-16
Acq. Time: 00:50:06
Modified: Yes
Proc. Algorithm: Specify Parameters - MQ III

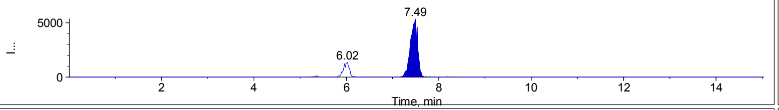

Sample Name: "ZW-10" Sample ID: "" File: "20250515-50AA.wiff"
Peak Name: "MSLAB-Nvl(IS)" Mass(es): "288.150/171.010 Da"
Comment: "" Annotation: ""
Sample Index: 40
Sample Type: Unknown
Concentration: 1.00 ng/mL
Calculated Conc: N/A
Acq. Date: 2025-05-16
Acq. Time: 00:50:06
Modified: Yes
Proc. Algorithm: Specify Parameters - MQ III

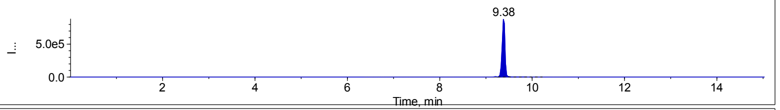

Sample Name: "ZW-10" Sample ID: "" File: "20250515-50AA.wiff"
Peak Name: "Ser" Mass(es): "276.100/171.010 Da"
Comment: "" Annotation: ""
Sample Index: 40
Sample Type: Unknown
Concentration: N/A
Calculated Conc: 4.44 umol/L
Acq. Date: 2025-05-16
Acq. Time: 00:50:06
Modified: Yes
Proc. Algorithm: Specify Parameters - MQ III

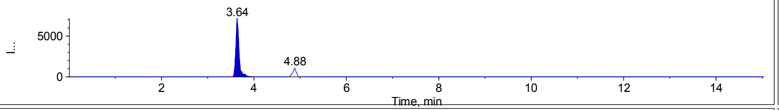

Sample Name: "ZW-10" Sample ID: "" File: "20250515-50AA.wiff"
Peak Name: "MSLAB-Nvl(IS)" Mass(es): "288.150/171.010 Da"
Comment: "" Annotation: ""
Sample Index: 40
Sample Type: Unknown
Concentration: 1.00 ng/mL
Calculated Conc: N/A
Acq. Date: 2025-05-16
Acq. Time: 00:50:06
Modified: Yes
Proc. Algorithm: Specify Parameters - MQ III

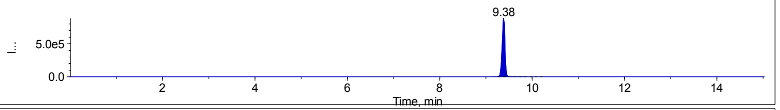

Sample Name: "ZW-10" Sample ID: "" File: "20250515-50AA.wiff"
Peak Name: "Pro" Mass(es): "286.150/171.010 Da"
Comment: "" Annotation: ""
Sample Index: 40
Sample Type: Unknown
Concentration: N/A
Calculated Conc: < 0
Acq. Date: 2025-05-16
Acq. Time: 00:50:06
Modified: Yes
Proc. Algorithm: Specify Parameters - MQ III

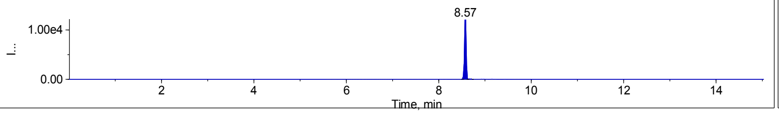

Sample Name: "ZW-10" Sample ID: "" File: "20250515-50AA.wiff"
Peak Name: "MSLAB-Nvl(IS)" Mass(es): "288.150/171.010 Da"
Comment: "" Annotation: ""
Sample Index: 40
Sample Type: Unknown
Concentration: 1.00 ng/mL
Calculated Conc: N/A
Acq. Date: 2025-05-16
Acq. Time: 00:50:06
Modified: Yes
Proc. Algorithm: Specify Parameters - MQ III

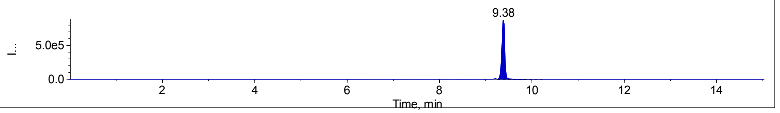

Sample Name: "ZW-10" Sample ID: "" File: "20250515-50AA.wiff"
Peak Name: "Val" Mass(es): "288.150/171.010 Da"
Comment: "" Annotation: ""
Sample Index: 40
Sample Type: Unknown
Concentration: N/A
Calculated Conc: 3.54 umol/L
Acq. Date: 2025-05-16
Acq. Time: 00:50:06
Modified: Yes
Proc. Algorithm: Specify Parameters - MQ III

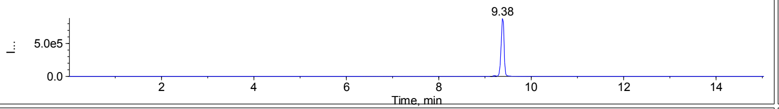

Sample Name: "ZW-10" Sample ID: "" File: "20250515-50AA.wiff"
Peak Name: "MSLAB-Nvl(IS)" Mass(es): "288.150/171.010 Da"
Comment: "" Annotation: ""
Sample Index: 40
Sample Type: Unknown
Concentration: 1.00 ng/mL
Calculated Conc: N/A
Acq. Date: 2025-05-16
Acq. Time: 00:50:06
Modified: Yes
Proc. Algorithm: Specify Parameters - MQ III

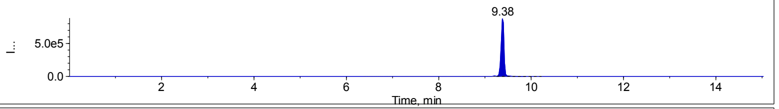

Sample Name: "ZW-10" Sample ID: "" File: "20250515-50AA.wiff"
Peak Name: "Thr" Mass(es): "290.100/171.010 Da"
Comment: "" Annotation: ""
Sample Index: 40
Sample Type: Unknown
Concentration: N/A
Calculated Conc: 2.45 umol/L
Acq. Date: 2025-05-16
Acq. Time: 00:50:06
Modified: Yes
Proc. Algorithm: Specify Parameters - MQ III

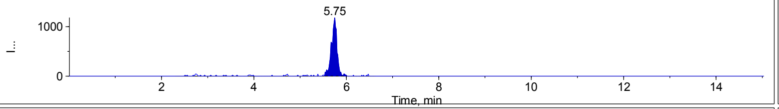

Sample Name: "ZW-10" Sample ID: "" File: "20250515-50AA.wiff"
Peak Name: "MSLAB-Nvl(IS)" Mass(es): "288.150/171.010 Da"
Comment: "" Annotation: ""
Sample Index: 40
Sample Type: Unknown
Concentration: 1.00 ng/mL
Calculated Conc: N/A
Acq. Date: 2025-05-16
Acq. Time: 00:50:06
Modified: Yes
Proc. Algorithm: Specify Parameters - MQ III

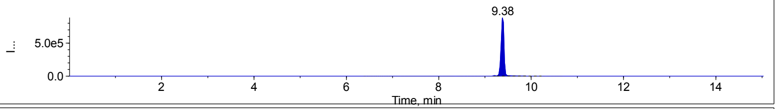

Sample Name: "ZW-10" Sample ID: "" File: "20250515-50AA.wiff"
Peak Name: "Cys1/2" Mass(es): "292.200/171.010 Da"
Comment: "" Annotation: ""
Sample Index: 40
Sample Type: Unknown
Concentration: N/A
Calculated Conc: 0.429 umol/L
Acq. Date: 2025-05-16
Acq. Time: 00:50:06
Modified: Yes
Proc. Algorithm: Specify Parameters - MQ III

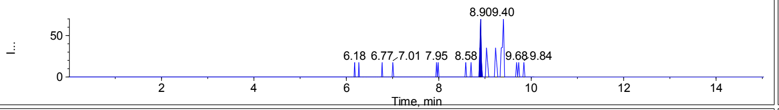

Sample Name: "ZW-10" Sample ID: "" File: "20250515-50AA.wiff"
Peak Name: "MSLAB-Nvl(IS)" Mass(es): "288.150/171.010 Da"
Comment: "" Annotation: ""
Sample Index: 40
Sample Type: Unknown
Concentration: 1.00 ng/mL
Calculated Conc: N/A
Acq. Date: 2025-05-16
Acq. Time: 00:50:06
Modified: Yes
Proc. Algorithm: Specify Parameters - MQ III

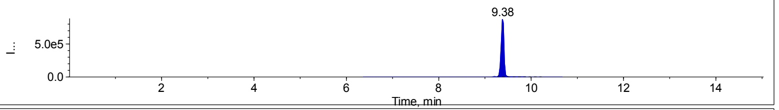

Sample Name: "ZW-10" Sample ID: "" File: "20250515-50AA.wiff"
Peak Name: "Ile" Mass(es): "302.150/171.010 Da"
Comment: "" Annotation: ""
Sample Index: 40
Sample Type: Unknown
Concentration: N/A
Calculated Conc: 1.05 umol/L
Acq. Date: 2025-05-16
Acq. Time: 00:50:06
Modified: Yes
Proc. Algorithm: Specify Parameters - MQ III

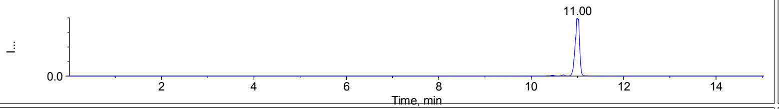

Sample Name: "ZW-10" Sample ID: "" File: "20250515-50AA.wiff"
Peak Name: "MSLAB-Nvl(IS)" Mass(es): "288.150/171.010 Da"
Comment: "" Annotation: ""
Sample Index: 40
Sample Type: Unknown
Concentration: 1.00 ng/mL
Calculated Conc: N/A
Acq. Date: 2025-05-16
Acq. Time: 00:50:06
Modified: Yes
Proc. Algorithm: Specify Parameters - MQ III

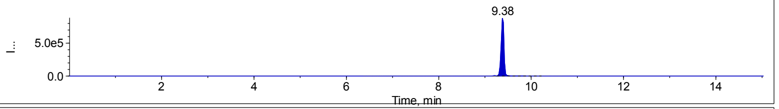

Sample Name: "ZW-10" Sample ID: "" File: "20250515-50AA.wiff"
Peak Name: "Asn" Mass(es): "303.130/171.010 Da"
Comment: "" Annotation: ""
Sample Index: 40
Sample Type: Unknown
Concentration: N/A
Calculated Conc: < 0
Acq. Date: 2025-05-16
Acq. Time: 00:50:06
Modified: Yes
Proc. Algorithm: Specify Parameters - MQ III

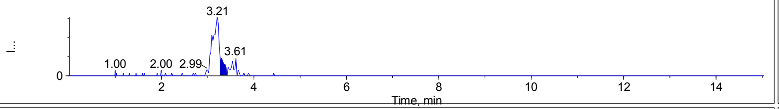

Sample Name: "ZW-10" Sample ID: "" File: "20250515-50AA.wiff"
Peak Name: "MSLAB-Nvl(IS)" Mass(es): "288.150/171.010 Da"
Comment: "" Annotation: ""
Sample Index: 40
Sample Type: Unknown
Concentration: 1.00 ng/mL
Calculated Conc: N/A
Acq. Date: 2025-05-16
Acq. Time: 00:50:06
Modified: Yes
Proc. Algorithm: Specify Parameters - MQ III

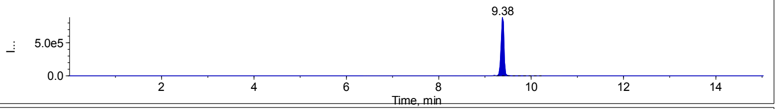

Sample Name: "ZW-10" Sample ID: "" File: "20250515-50AA.wiff"
Peak Name: "Asp" Mass(es): "304.110/171.010 Da"
Comment: "" Annotation: ""
Sample Index: 40
Sample Type: Unknown
Concentration: N/A
Calculated Conc: 0.990 umol/L
Acq. Date: 2025-05-16
Acq. Time: 00:50:06
Modified: Yes
Proc. Algorithm: Specify Parameters - MQ III

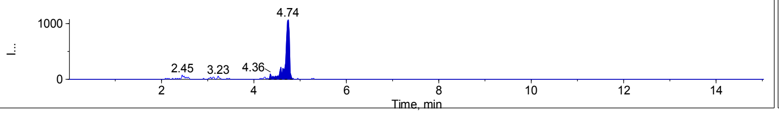

Sample Name: "ZW-10" Sample ID: "" File: "20250515-50AA.wiff"
Peak Name: "MSLAB-Nvl(IS)" Mass(es): "288.150/171.010 Da"
Comment: "" Annotation: ""
Sample Index: 40
Sample Type: Unknown
Concentration: 1.00 ng/mL
Calculated Conc: N/A
Acq. Date: 2025-05-16
Acq. Time: 00:50:06
Modified: Yes
Proc. Algorithm: Specify Parameters - MQ III

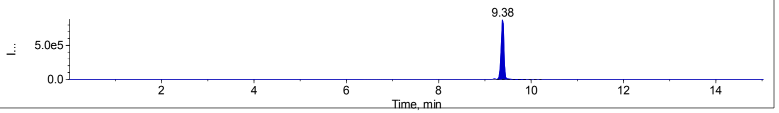

Sample Name: "ZW-10" Sample ID: "" File: "20250515-50AA.wiff"
Peak Name: "Gln" Mass(es): "317.150/171.010 Da"
Comment: "" Annotation: ""

Sample Index: 40
Sample Type: Unknown
Concentration: N/A
Calculated Conc: < 0
Acq. Date: 2025-05-16
Acq. Time: 00:50:06

Modified: Yes
Proc. Algorithm: Specify Parameters - MQ III

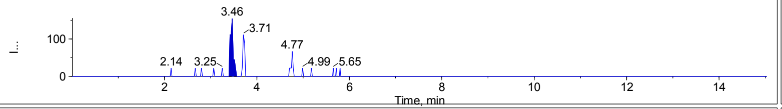

Sample Name: "ZW-10" Sample ID: "" File: "20250515-50AA.wiff"
Peak Name: "Glu" Mass(es): "318.100/171.010 Da"
Comment: "" Annotation: ""

Sample Index: 40
Sample Type: Unknown
Concentration: N/A
Calculated Conc: 2.78 umol/L
Acq. Date: 2025-05-16
Acq. Time: 00:50:06

Modified: Yes
Proc. Algorithm: Specify Parameters - MQ III

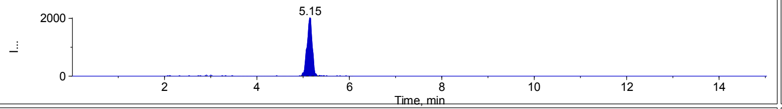

Sample Name: "ZW-10" Sample ID: "" File: "20250515-50AA.wiff"
Peak Name: "Met" Mass(es): "320.210/171.010 Da"
Comment: "" Annotation: ""

Sample Index: 40
Sample Type: Unknown
Concentration: N/A
Calculated Conc: < 0
Acq. Date: 2025-05-16
Acq. Time: 00:50:06

Modified: Yes
Proc. Algorithm: Specify Parameters - MQ III

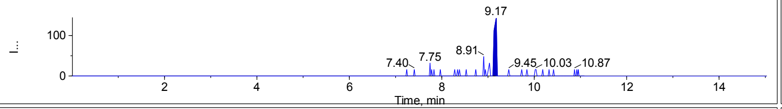

Sample Name: "ZW-10" Sample ID: "" File: "20250515-50AA.wiff"
Peak Name: "His" Mass(es): "326.150/171.010 Da"
Comment: "" Annotation: ""

Sample Index: 40
Sample Type: Unknown
Concentration: N/A
Calculated Conc: 1.48 umol/L
Acq. Date: 2025-05-16
Acq. Time: 00:50:06

Modified: Yes
Proc. Algorithm: Specify Parameters - MQ III

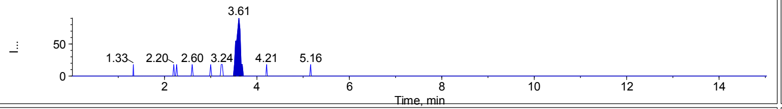

Sample Name: "ZW-10" Sample ID: "" File: "20250515-50AA.wiff"
Peak Name: "Phe" Mass(es): "336.200/171.010 Da"
Comment: "" Annotation: ""

Sample Index: 40
Sample Type: Unknown
Concentration: N/A
Calculated Conc: 1.12 umol/L
Acq. Date: 2025-05-16
Acq. Time: 00:50:06

Modified: Yes
Proc. Algorithm: Specify Parameters - MQ III

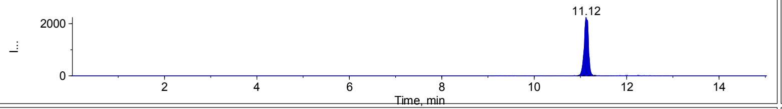

Sample Name: "ZW-10" Sample ID: "" File: "20250515-50AA.wiff"
Peak Name: "Arg" Mass(es): "345.210/171.010 Da"
Comment: "" Annotation: ""

Sample Index: 40
Sample Type: Unknown
Concentration: N/A
Calculated Conc: 3.50 umol/L
Acq. Date: 2025-05-16
Acq. Time: 00:50:06

Modified: Yes
Proc. Algorithm: Specify Parameters - MQ III

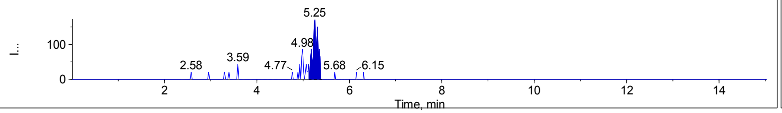

Sample Name: "ZW-10" Sample ID: "" File: "20250515-50AA.wiff"
Peak Name: "MSLAB-Nvl(IS)" Mass(es): "288.150/171.010 Da"
Comment: "" Annotation: ""

Sample Index: 40
Sample Type: Unknown
Concentration: 1.00 ng/mL
Calculated Conc: N/A
Acq. Date: 2025-05-16
Acq. Time: 00:50:06

Modified: Yes
Proc. Algorithm: Specify Parameters - MQ III

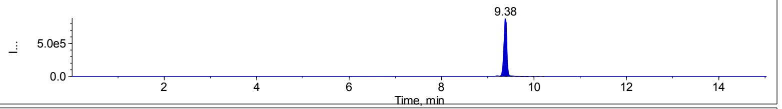

Sample Name: "ZW-10" Sample ID: "" File: "20250515-50AA.wiff"
Peak Name: "MSLAB-Nvl(IS)" Mass(es): "288.150/171.010 Da"
Comment: "" Annotation: ""

Sample Index: 40
Sample Type: Unknown
Concentration: 1.00 ng/mL
Calculated Conc: N/A
Acq. Date: 2025-05-16
Acq. Time: 00:50:06

Modified: Yes
Proc. Algorithm: Specify Parameters - MQ III

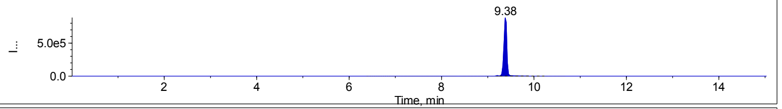

Sample Name: "ZW-10" Sample ID: "" File: "20250515-50AA.wiff"
Peak Name: "MSLAB-Nvl(IS)" Mass(es): "288.150/171.010 Da"
Comment: "" Annotation: ""

Sample Index: 40
Sample Type: Unknown
Concentration: 1.00 ng/mL
Calculated Conc: N/A
Acq. Date: 2025-05-16
Acq. Time: 00:50:06

Modified: Yes
Proc. Algorithm: Specify Parameters - MQ III

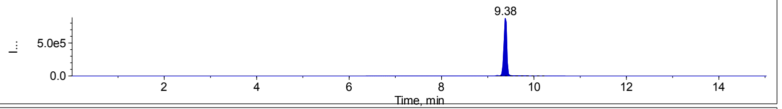

Sample Name: "ZW-10" Sample ID: "" File: "20250515-50AA.wiff"
Peak Name: "MSLAB-Nvl(IS)" Mass(es): "288.150/171.010 Da"
Comment: "" Annotation: ""

Sample Index: 40
Sample Type: Unknown
Concentration: 1.00 ng/mL
Calculated Conc: N/A
Acq. Date: 2025-05-16
Acq. Time: 00:50:06

Modified: Yes
Proc. Algorithm: Specify Parameters - MQ III

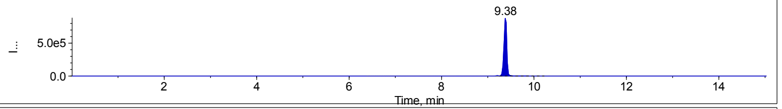

Sample Name: "ZW-10" Sample ID: "" File: "20250515-50AA.wiff"
Peak Name: "MSLAB-Nvl(IS)" Mass(es): "288.150/171.010 Da"
Comment: "" Annotation: ""

Sample Index: 40
Sample Type: Unknown
Concentration: 1.00 ng/mL
Calculated Conc: N/A
Acq. Date: 2025-05-16
Acq. Time: 00:50:06

Modified: Yes
Proc. Algorithm: Specify Parameters - MQ III

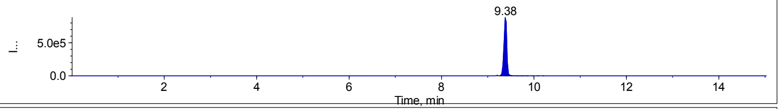

Sample Name: "ZW-10" Sample ID: "" File: "20250515-50AA.wiff"
Peak Name: "MSLAB-Nvl(IS)" Mass(es): "288.150/171.010 Da"
Comment: "" Annotation: ""

Sample Index: 40
Sample Type: Unknown
Concentration: 1.00 ng/mL
Calculated Conc: N/A
Acq. Date: 2025-05-16
Acq. Time: 00:50:06

Modified: Yes
Proc. Algorithm: Specify Parameters - MQ III

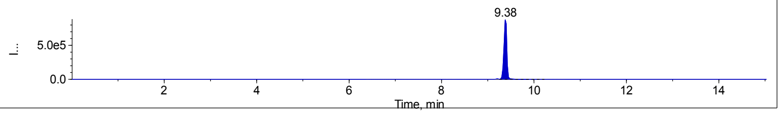

Sample Name: "ZW-10" Sample ID: "" File: "20250515-50AA.wiff"
Peak Name: "Trp" Mass(es): "375.200/171.010 Da"
Comment: "" Annotation: ""
Sample Index: 40
Sample Type: Unknown
Concentration: N/A
Calculated Conc: < 0
Acq. Date: 2025-05-16
Acq. Time: 00:50:06
Modified: Yes
Proc. Algorithm: Specify Parameters - MQ III

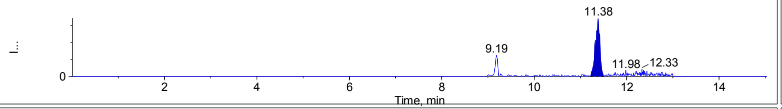

Sample Name: "ZW-10" Sample ID: "" File: "20250515-50AA.wiff"
Peak Name: "MSLAB-Nvl(IS)" Mass(es): "288.150/171.010 Da"
Comment: "" Annotation: ""
Sample Index: 40
Sample Type: Unknown
Concentration: 1.00 ng/mL
Calculated Conc: N/A
Acq. Date: 2025-05-16
Acq. Time: 00:50:06
Modified: Yes
Proc. Algorithm: Specify Parameters - MQ III

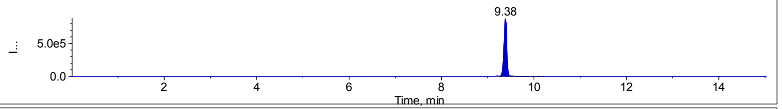

Sample Name: "ZW-10" Sample ID: "" File: "20250515-50AA.wiff"
Peak Name: "Lys" Mass(es): "487.200/171.010 Da"
Comment: "" Annotation: ""
Sample Index: 40
Sample Type: Unknown
Concentration: N/A
Calculated Conc: 3.13 umol/L
Acq. Date: 2025-05-16
Acq. Time: 00:50:06
Modified: Yes
Proc. Algorithm: Specify Parameters - MQ III

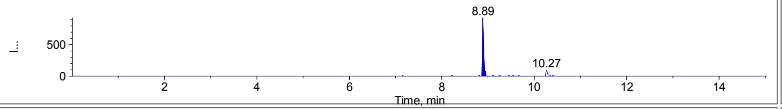

Sample Name: "ZW-10" Sample ID: "" File: "20250515-50AA.wiff"
Peak Name: "MSLAB-Nvl(IS)" Mass(es): "288.150/171.010 Da"
Comment: "" Annotation: ""
Sample Index: 40
Sample Type: Unknown
Concentration: 1.00 ng/mL
Calculated Conc: N/A
Acq. Date: 2025-05-16
Acq. Time: 00:50:06
Modified: Yes
Proc. Algorithm: Specify Parameters - MQ III

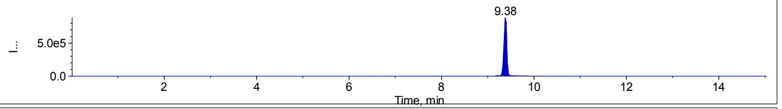

Sample Name: "ZW-10" Sample ID: "" File: "20250515-50AA.wiff"
Peak Name: "tyr" Mass(es): "352.200/171.010 Da"
Comment: "" Annotation: ""
Sample Index: 40
Sample Type: Unknown
Concentration: N/A
Calculated Conc: < 0
Acq. Date: 2025-05-16
Acq. Time: 00:50:06
Modified: Yes
Proc. Algorithm: Specify Parameters - MQ III

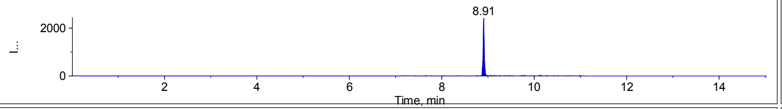

Sample Name: "ZW-10" Sample ID: "" File: "20250515-50AA.wiff"
Peak Name: "MSLAB-Nvl(IS)" Mass(es): "288.150/171.010 Da"
Comment: "" Annotation: ""
Sample Index: 40
Sample Type: Unknown
Concentration: 1.00 ng/mL
Calculated Conc: N/A
Acq. Date: 2025-05-16
Acq. Time: 00:50:06
Modified: Yes
Proc. Algorithm: Specify Parameters - MQ III

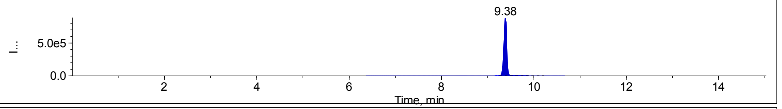

Sample Name: "ZW-10" Sample ID: "" File: "20250515-50AA.wiff"
Peak Name: "Leu" Mass(es): "302.150/171.010 Da"
Comment: "" Annotation: ""
Sample Index: 40
Sample Type: Unknown
Concentration: N/A
Calculated Conc: 2.12 umol/L
Acq. Date: 2025-05-16
Acq. Time: 00:50:06
Modified: Yes
Proc. Algorithm: Specify Parameters - MQ III

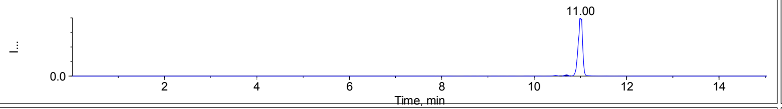

Sample Name: "ZW-10" Sample ID: "" File: "20250515-50AA.wiff"
Peak Name: "MSLAB-Nvl(IS)" Mass(es): "288.150/171.010 Da"
Comment: "" Annotation: ""
Sample Index: 40
Sample Type: Unknown
Concentration: 1.00 ng/mL
Calculated Conc: N/A
Acq. Date: 2025-05-16
Acq. Time: 00:50:06
Modified: Yes
Proc. Algorithm: Specify Parameters - MQ III

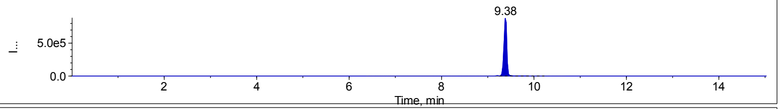

Sample Name: "ZW-11" Sample ID: "" File: "20250515-50AA.wiff"
Peak Name: "Gly" Mass(es): "246.080/171.010 Da"
Comment: "" Annotation: ""
Sample Index: 41
Sample Type: Unknown
Concentration: N/A
Calculated Conc: 2.01 umol/L
Acq. Date: 2025-05-16
Acq. Time: 01:05:35
Modified: Yes
Proc. Algorithm: Specify Parameters - MQ III

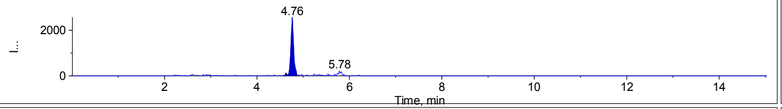

Sample Name: "ZW-11" Sample ID: "" File: "20250515-50AA.wiff"
Peak Name: "MSLAB-Nvl(IS)" Mass(es): "288.150/171.010 Da"
Comment: "" Annotation: ""
Sample Index: 41
Sample Type: Unknown
Concentration: 1.00 ng/mL
Calculated Conc: N/A
Acq. Date: 2025-05-16
Acq. Time: 01:05:35
Modified: Yes
Proc. Algorithm: Specify Parameters - MQ III

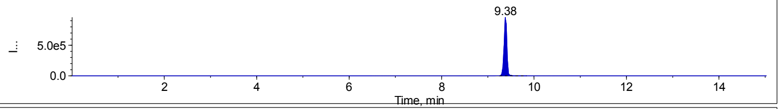

Sample Name: "ZW-11" Sample ID: "" File: "20250515-50AA.wiff"
Peak Name: "Ala" Mass(es): "260.100/171.010 Da"
Comment: "" Annotation: ""
Sample Index: 41
Sample Type: Unknown
Concentration: N/A
Calculated Conc: No Intercept
Acq. Date: 2025-05-16
Acq. Time: 01:05:35
Modified: Yes
Proc. Algorithm: Specify Parameters - MQ III

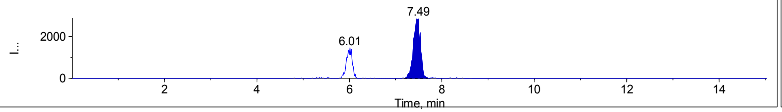

Sample Name: "ZW-11" Sample ID: "" File: "20250515-50AA.wiff"
Peak Name: "MSLAB-Nvl(IS)" Mass(es): "288.150/171.010 Da"
Comment: "" Annotation: ""
Sample Index: 41
Sample Type: Unknown
Concentration: 1.00 ng/mL
Calculated Conc: N/A
Acq. Date: 2025-05-16
Acq. Time: 01:05:35
Modified: Yes
Proc. Algorithm: Specify Parameters - MQ III

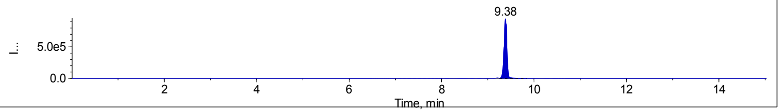

Sample Name: "ZW-11"   Sample ID: ""   File: "20250515-50AA.wiff"
Peak Name: "Ser"   Mass(es): "276.100/171.010 Da"
Comment: ""   Annotation: ""

```
Sample Index:      41
Sample Type:      Unknown
Concentration:       N/A
Calculated Conc: No Intercept
Acq. Date:        2025-05-16
Acq. Time:        01:05:35

Modified:            Yes
Proc. Algorithm: Specify Parameters - MQ III
```

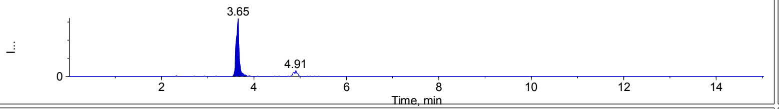

Sample Name: "ZW-11"   Sample ID: ""   File: "20250515-50AA.wiff"
Peak Name: "MSLAB-Nvl(IS)"   Mass(es): "288.150/171.010 Da"
Comment: ""   Annotation: ""

```
Sample Index:      41
Sample Type:      Unknown
Concentration:       1.00    ng/mL
Calculated Conc:     N/A
Acq. Date:        2025-05-16
Acq. Time:        01:05:35

Modified:            Yes
Proc. Algorithm: Specify Parameters - MQ III
```

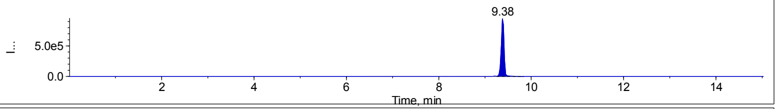

Sample Name: "ZW-11"   Sample ID: ""   File: "20250515-50AA.wiff"
Peak Name: "Pro"   Mass(es): "286.150/171.010 Da"
Comment: ""   Annotation: ""

```
Sample Index:      41
Sample Type:      Unknown
Concentration:       N/A
Calculated Conc: No Intercept
Acq. Date:        2025-05-16
Acq. Time:        01:05:35

Modified:            Yes
Proc. Algorithm: Specify Parameters - MQ III
```

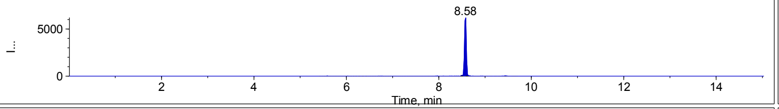

Sample Name: "ZW-11"   Sample ID: ""   File: "20250515-50AA.wiff"
Peak Name: "MSLAB-Nvl(IS)"   Mass(es): "288.150/171.010 Da"
Comment: ""   Annotation: ""

```
Sample Index:      41
Sample Type:      Unknown
Concentration:       1.00    ng/mL
Calculated Conc:     N/A
Acq. Date:        2025-05-16
Acq. Time:        01:05:35

Modified:            Yes
Proc. Algorithm: Specify Parameters - MQ III
```

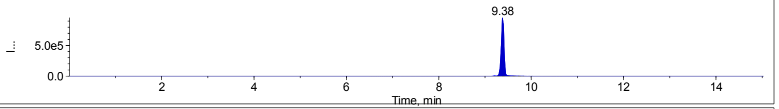

Sample Name: "ZW-11"   Sample ID: ""   File: "20250515-50AA.wiff"
Peak Name: "Val"   Mass(es): "288.150/171.010 Da"
Comment: ""   Annotation: ""

```
Sample Index:      41
Sample Type:      Unknown
Concentration:       N/A
Calculated Conc:     2.15    umol/L
Acq. Date:        2025-05-16
Acq. Time:        01:05:35

Modified:            Yes
Proc. Algorithm: Specify Parameters - MQ III
```

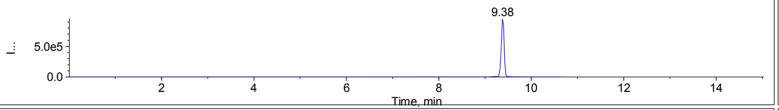

Sample Name: "ZW-11"   Sample ID: ""   File: "20250515-50AA.wiff"
Peak Name: "MSLAB-Nvl(IS)"   Mass(es): "288.150/171.010 Da"
Comment: ""   Annotation: ""

```
Sample Index:      41
Sample Type:      Unknown
Concentration:       1.00    ng/mL
Calculated Conc:     N/A
Acq. Date:        2025-05-16
Acq. Time:        01:05:35

Modified:            Yes
Proc. Algorithm: Specify Parameters - MQ III
```

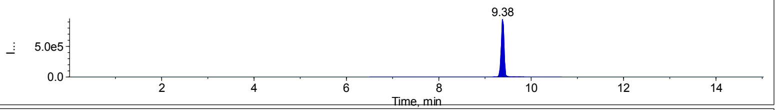

Sample Name: "ZW-11"   Sample ID: ""   File: "20250515-50AA.wiff"
Peak Name: "Thr"   Mass(es): "290.100/171.010 Da"
Comment: ""   Annotation: ""

```
Sample Index:      41
Sample Type:      Unknown
Concentration:       N/A
Calculated Conc:     1.64    umol/L
Acq. Date:        2025-05-16
Acq. Time:        01:05:35

Modified:            Yes
Proc. Algorithm: Specify Parameters - MQ III
```

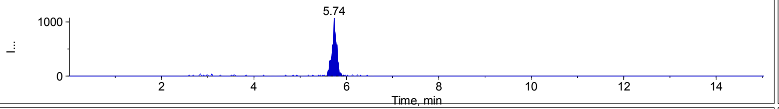

Sample Name: "ZW-11"   Sample ID: ""   File: "20250515-50AA.wiff"
Peak Name: "MSLAB-Nvl(IS)"   Mass(es): "288.150/171.010 Da"
Comment: ""   Annotation: ""

```
Sample Index:      41
Sample Type:      Unknown
Concentration:       1.00    ng/mL
Calculated Conc:     N/A
Acq. Date:        2025-05-16
Acq. Time:        01:05:35

Modified:            Yes
Proc. Algorithm: Specify Parameters - MQ III
```

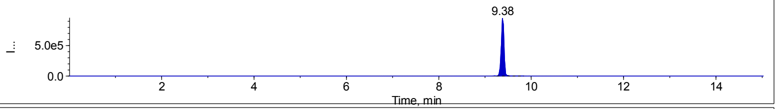

Sample Name: "ZW-11"   Sample ID: ""   File: "20250515-50AA.wiff"
Peak Name: "Cys1/2"   Mass(es): "292.200/171.010 Da"
Comment: ""   Annotation: ""

```
Sample Index:      41
Sample Type:      Unknown
Concentration:       N/A
Calculated Conc:     0.360    umol/L
Acq. Date:        2025-05-16
Acq. Time:        01:05:35

Modified:            Yes
Proc. Algorithm: Specify Parameters - MQ III
```

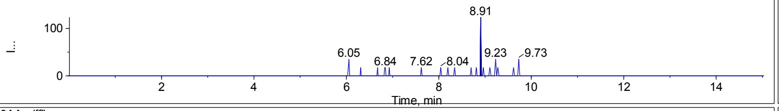

Sample Name: "ZW-11"   Sample ID: ""   File: "20250515-50AA.wiff"
Peak Name: "MSLAB-Nvl(IS)"   Mass(es): "288.150/171.010 Da"
Comment: ""   Annotation: ""

```
Sample Index:      41
Sample Type:      Unknown
Concentration:       1.00    ng/mL
Calculated Conc:     N/A
Acq. Date:        2025-05-16
Acq. Time:        01:05:35

Modified:            Yes
Proc. Algorithm: Specify Parameters - MQ III
```

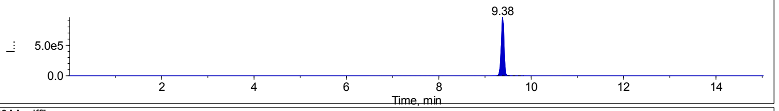

Sample Name: "ZW-11"   Sample ID: ""   File: "20250515-50AA.wiff"
Peak Name: "Ile"   Mass(es): "302.150/171.010 Da"
Comment: ""   Annotation: ""

```
Sample Index:      41
Sample Type:      Unknown
Concentration:       N/A
Calculated Conc:     0.535    umol/L
Acq. Date:        2025-05-16
Acq. Time:        01:05:35

Modified:            Yes
Proc. Algorithm: Specify Parameters - MQ III
```

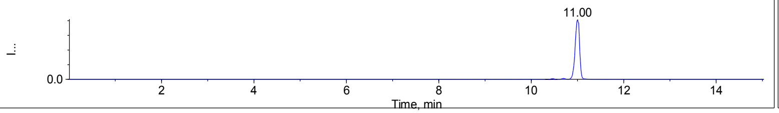

Sample Name: "ZW-11"   Sample ID: ""   File: "20250515-50AA.wiff"
Peak Name: "MSLAB-Nvl(IS)"   Mass(es): "288.150/171.010 Da"
Comment: ""   Annotation: ""

```
Sample Index:      41
Sample Type:      Unknown
Concentration:       1.00    ng/mL
Calculated Conc:     N/A
Acq. Date:        2025-05-16
Acq. Time:        01:05:35

Modified:            Yes
Proc. Algorithm: Specify Parameters - MQ III
```

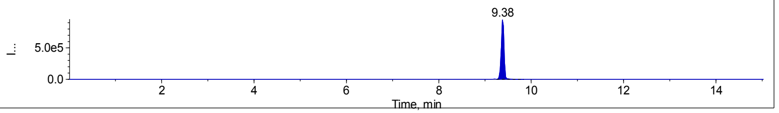

Sample Name: "ZW-11" Sample ID: "" File: "20250515-50AA.wiff"
Peak Name: "Asn" Mass(es): "303.130/171.010 Da"
Comment: "" Annotation: ""

Sample Index: 41
Sample Type: Unknown
Concentration: N/A
Calculated Conc: 0.00 umol/L
Acq. Date: 2025-05-16
Acq. Time: 01:05:35

Modified: Yes

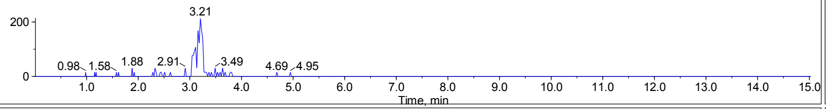

Sample Name: "ZW-11" Sample ID: "" File: "20250515-50AA.wiff"
Peak Name: "MSLAB-Nvl(IS)" Mass(es): "288.150/171.010 Da"
Comment: "" Annotation: ""

Sample Index: 41
Sample Type: Unknown
Concentration: 1.00 ng/mL
Calculated Conc: N/A
Acq. Date: 2025-05-16
Acq. Time: 01:05:35

Modified: Yes
Proc. Algorithm: Specify Parameters - MQ III

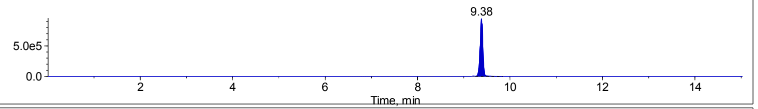

Sample Name: "ZW-11" Sample ID: "" File: "20250515-50AA.wiff"
Peak Name: "Asp" Mass(es): "304.110/171.010 Da"
Comment: "" Annotation: ""

Sample Index: 41
Sample Type: Unknown
Concentration: N/A
Calculated Conc: < 0
Acq. Date: 2025-05-16
Acq. Time: 01:05:35

Modified: Yes
Proc. Algorithm: Specify Parameters - MQ III

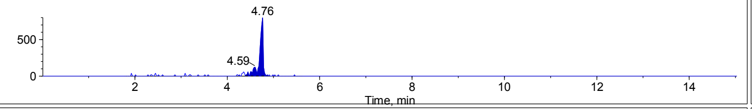

Sample Name: "ZW-11" Sample ID: "" File: "20250515-50AA.wiff"
Peak Name: "MSLAB-Nvl(IS)" Mass(es): "288.150/171.010 Da"
Comment: "" Annotation: ""

Sample Index: 41
Sample Type: Unknown
Concentration: 1.00 ng/mL
Calculated Conc: N/A
Acq. Date: 2025-05-16
Acq. Time: 01:05:35

Modified: Yes
Proc. Algorithm: Specify Parameters - MQ III

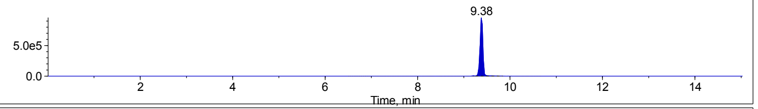

Sample Name: "ZW-11" Sample ID: "" File: "20250515-50AA.wiff"
Peak Name: "Gln" Mass(es): "317.150/171.010 Da"
Comment: "" Annotation: ""

Sample Index: 41
Sample Type: Unknown
Concentration: N/A
Calculated Conc: No Intercept
Acq. Date: 2025-05-16
Acq. Time: 01:05:35

Modified: Yes
Proc. Algorithm: Specify Parameters - MQ III

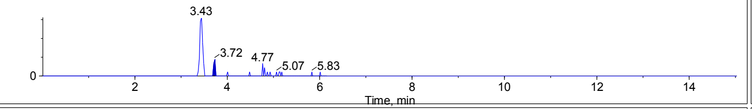

Sample Name: "ZW-11" Sample ID: "" File: "20250515-50AA.wiff"
Peak Name: "MSLAB-Nvl(IS)" Mass(es): "288.150/171.010 Da"
Comment: "" Annotation: ""

Sample Index: 41
Sample Type: Unknown
Concentration: 1.00 ng/mL
Calculated Conc: N/A
Acq. Date: 2025-05-16
Acq. Time: 01:05:35

Modified: Yes
Proc. Algorithm: Specify Parameters - MQ III

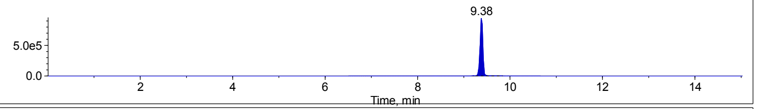

Sample Name: "ZW-11" Sample ID: "" File: "20250515-50AA.wiff"
Peak Name: "Glu" Mass(es): "318.100/171.010 Da"
Comment: "" Annotation: ""

Sample Index: 41
Sample Type: Unknown
Concentration: N/A
Calculated Conc: 1.72 umol/L
Acq. Date: 2025-05-16
Acq. Time: 01:05:35

Modified: Yes
Proc. Algorithm: Specify Parameters - MQ III

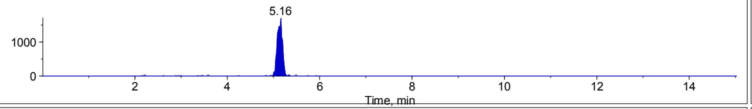

Sample Name: "ZW-11" Sample ID: "" File: "20250515-50AA.wiff"
Peak Name: "MSLAB-Nvl(IS)" Mass(es): "288.150/171.010 Da"
Comment: "" Annotation: ""

Sample Index: 41
Sample Type: Unknown
Concentration: 1.00 ng/mL
Calculated Conc: N/A
Acq. Date: 2025-05-16
Acq. Time: 01:05:35

Modified: Yes
Proc. Algorithm: Specify Parameters - MQ III

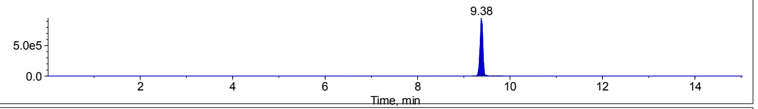

Sample Name: "ZW-11" Sample ID: "" File: "20250515-50AA.wiff"
Peak Name: "Met" Mass(es): "320.210/171.010 Da"
Comment: "" Annotation: ""

Sample Index: 41
Sample Type: Unknown
Concentration: N/A
Calculated Conc: < 0
Acq. Date: 2025-05-16
Acq. Time: 01:05:35

Modified: Yes
Proc. Algorithm: Specify Parameters - MQ III

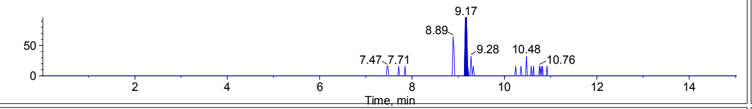

Sample Name: "ZW-11" Sample ID: "" File: "20250515-50AA.wiff"
Peak Name: "MSLAB-Nvl(IS)" Mass(es): "288.150/171.010 Da"
Comment: "" Annotation: ""

Sample Index: 41
Sample Type: Unknown
Concentration: 1.00 ng/mL
Calculated Conc: N/A
Acq. Date: 2025-05-16
Acq. Time: 01:05:35

Modified: Yes
Proc. Algorithm: Specify Parameters - MQ III

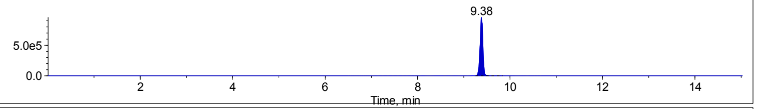

Sample Name: "ZW-11" Sample ID: "" File: "20250515-50AA.wiff"
Peak Name: "His" Mass(es): "326.150/171.010 Da"
Comment: "" Annotation: ""

Sample Index: 41
Sample Type: Unknown
Concentration: N/A
Calculated Conc: 0.618 umol/L
Acq. Date: 2025-05-16
Acq. Time: 01:05:35

Modified: Yes
Proc. Algorithm: Specify Parameters - MQ III

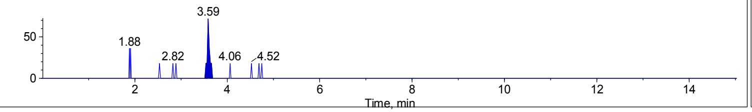

Sample Name: "ZW-11" Sample ID: "" File: "20250515-50AA.wiff"
Peak Name: "MSLAB-Nvl(IS)" Mass(es): "288.150/171.010 Da"
Comment: "" Annotation: ""

Sample Index: 41
Sample Type: Unknown
Concentration: 1.00 ng/mL
Calculated Conc: N/A
Acq. Date: 2025-05-16
Acq. Time: 01:05:35

Modified: Yes
Proc. Algorithm: Specify Parameters - MQ III

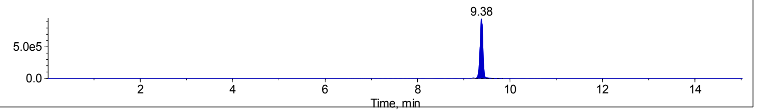

Sample Name: "ZW-11" Sample ID: "" File: "20250515-50AA.wiff"
Peak Name: "Phe" Mass(es): "336.200/171.010 Da"
Comment: "" Annotation: ""
Sample Index: 41
Sample Type: Unknown
Concentration: N/A
Calculated Conc: 0.783 umol/L
Acq. Date: 2025-05-16
Acq. Time: 01:05:35
Modified: Yes
Proc. Algorithm: Specify Parameters - MQ III

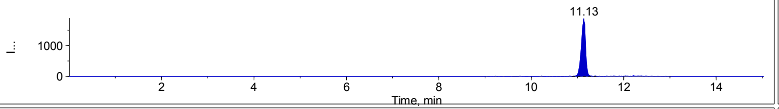

Sample Name: "ZW-11" Sample ID: "" File: "20250515-50AA.wiff"
Peak Name: "MSLAB-Nvl(IS)" Mass(es): "288.150/171.010 Da"
Comment: "" Annotation: ""
Sample Index: 41
Sample Type: Unknown
Concentration: 1.00 ng/mL
Calculated Conc: N/A
Acq. Date: 2025-05-16
Acq. Time: 01:05:35
Modified: Yes
Proc. Algorithm: Specify Parameters - MQ III

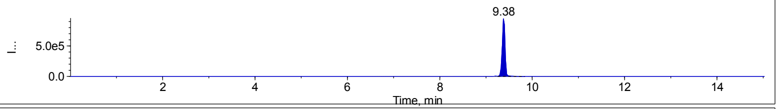

Sample Name: "ZW-11" Sample ID: "" File: "20250515-50AA.wiff"
Peak Name: "Arg" Mass(es): "345.210/171.010 Da"
Comment: "" Annotation: ""
Sample Index: 41
Sample Type: Unknown
Concentration: N/A
Calculated Conc: 2.14 umol/L
Acq. Date: 2025-05-16
Acq. Time: 01:05:35
Modified: Yes
Proc. Algorithm: Specify Parameters - MQ III

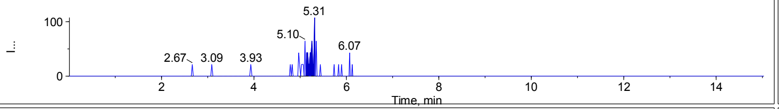

Sample Name: "ZW-11" Sample ID: "" File: "20250515-50AA.wiff"
Peak Name: "MSLAB-Nvl(IS)" Mass(es): "288.150/171.010 Da"
Comment: "" Annotation: ""
Sample Index: 41
Sample Type: Unknown
Concentration: 1.00 ng/mL
Calculated Conc: N/A
Acq. Date: 2025-05-16
Acq. Time: 01:05:35
Modified: Yes
Proc. Algorithm: Specify Parameters - MQ III

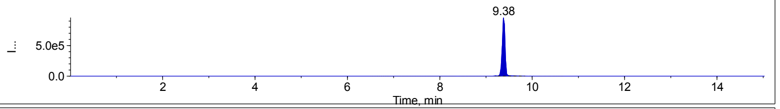

Sample Name: "ZW-11" Sample ID: "" File: "20250515-50AA.wiff"
Peak Name: "Trp" Mass(es): "375.200/171.010 Da"
Comment: "" Annotation: ""
Sample Index: 41
Sample Type: Unknown
Concentration: N/A
Calculated Conc: < 0
Acq. Date: 2025-05-16
Acq. Time: 01:05:35
Modified: Yes
Proc. Algorithm: Specify Parameters - MQ III

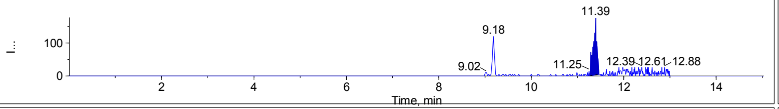

Sample Name: "ZW-11" Sample ID: "" File: "20250515-50AA.wiff"
Peak Name: "MSLAB-Nvl(IS)" Mass(es): "288.150/171.010 Da"
Comment: "" Annotation: ""
Sample Index: 41
Sample Type: Unknown
Concentration: 1.00 ng/mL
Calculated Conc: N/A
Acq. Date: 2025-05-16
Acq. Time: 01:05:35
Modified: Yes
Proc. Algorithm: Specify Parameters - MQ III

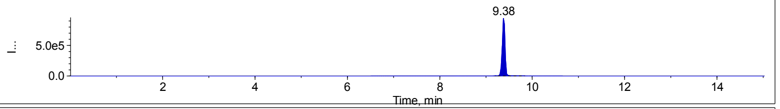

Sample Name: "ZW-11" Sample ID: "" File: "20250515-50AA.wiff"
Peak Name: "Lys" Mass(es): "487.200/171.010 Da"
Comment: "" Annotation: ""
Sample Index: 41
Sample Type: Unknown
Concentration: N/A
Calculated Conc: 2.14 umol/L
Acq. Date: 2025-05-16
Acq. Time: 01:05:35
Modified: Yes
Proc. Algorithm: Specify Parameters - MQ III

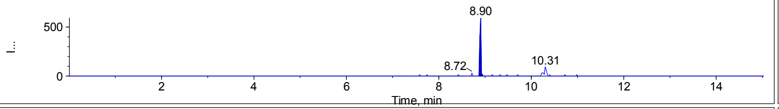

Sample Name: "ZW-11" Sample ID: "" File: "20250515-50AA.wiff"
Peak Name: "MSLAB-Nvl(IS)" Mass(es): "288.150/171.010 Da"
Comment: "" Annotation: ""
Sample Index: 41
Sample Type: Unknown
Concentration: 1.00 ng/mL
Calculated Conc: N/A
Acq. Date: 2025-05-16
Acq. Time: 01:05:35
Modified: Yes
Proc. Algorithm: Specify Parameters - MQ III

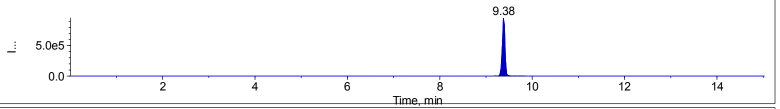

Sample Name: "ZW-11" Sample ID: "" File: "20250515-50AA.wiff"
Peak Name: "tyr" Mass(es): "352.200/171.010 Da"
Comment: "" Annotation: ""
Sample Index: 41
Sample Type: Unknown
Concentration: N/A
Calculated Conc: < 0
Acq. Date: 2025-05-16
Acq. Time: 01:05:35
Modified: Yes
Proc. Algorithm: Specify Parameters - MQ III

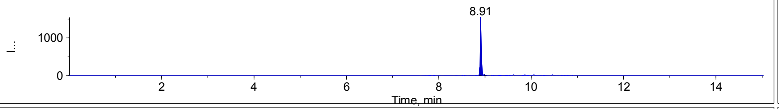

Sample Name: "ZW-11" Sample ID: "" File: "20250515-50AA.wiff"
Peak Name: "MSLAB-Nvl(IS)" Mass(es): "288.150/171.010 Da"
Comment: "" Annotation: ""
Sample Index: 41
Sample Type: Unknown
Concentration: 1.00 ng/mL
Calculated Conc: N/A
Acq. Date: 2025-05-16
Acq. Time: 01:05:35
Modified: Yes
Proc. Algorithm: Specify Parameters - MQ III

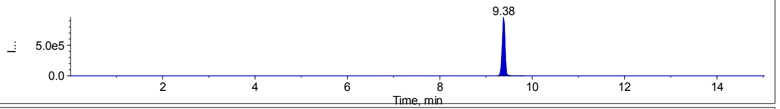

Sample Name: "ZW-11" Sample ID: "" File: "20250515-50AA.wiff"
Peak Name: "Leu" Mass(es): "302.150/171.010 Da"
Comment: "" Annotation: ""
Sample Index: 41
Sample Type: Unknown
Concentration: N/A
Calculated Conc: 1.38 umol/L
Acq. Date: 2025-05-16
Acq. Time: 01:05:35
Modified: Yes
Proc. Algorithm: Specify Parameters - MQ III

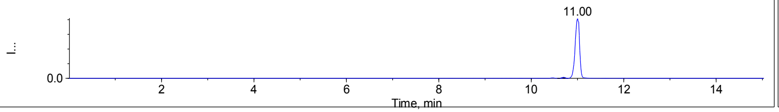

Sample Name: "ZW-11" Sample ID: "" File: "20250515-50AA.wiff"
Peak Name: "MSLAB-Nvl(IS)" Mass(es): "288.150/171.010 Da"
Comment: "" Annotation: ""
Sample Index: 41
Sample Type: Unknown
Concentration: 1.00 ng/mL
Calculated Conc: N/A
Acq. Date: 2025-05-16
Acq. Time: 01:05:35
Modified: Yes
Proc. Algorithm: Specify Parameters - MQ III

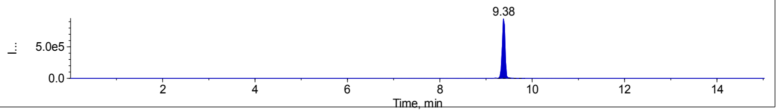

Sample Name: "ZW-12" Sample ID: "" File: "20250515-50AA.wiff"
Peak Name: "Gly" Mass(es): "246.080/171.010 Da"
Comment: "" Annotation: ""

Sample Index: 42
Sample Type: Unknown
Concentration: N/A
Calculated Conc: 1.37 umol/L
Acq. Date: 2025-05-16
Acq. Time: 01:21:04

Modified: Yes
Proc. Algorithm: Specify Parameters - MQ III

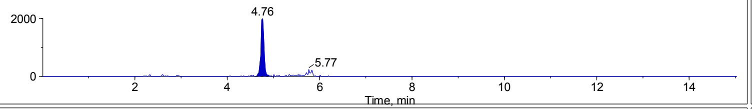

Sample Name: "ZW-12" Sample ID: "" File: "20250515-50AA.wiff"
Peak Name: "Ala" Mass(es): "260.100/171.010 Da"
Comment: "" Annotation: ""

Sample Index: 42
Sample Type: Unknown
Concentration: N/A
Calculated Conc: No Intercept
Acq. Date: 2025-05-16
Acq. Time: 01:21:04

Modified: Yes
Proc. Algorithm: Specify Parameters - MQ III

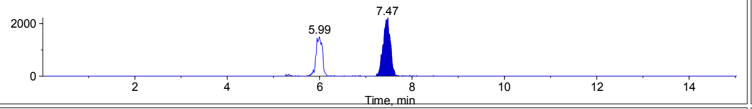

Sample Name: "ZW-12" Sample ID: "" File: "20250515-50AA.wiff"
Peak Name: "Ser" Mass(es): "276.100/171.010 Da"
Comment: "" Annotation: ""

Sample Index: 42
Sample Type: Unknown
Concentration: N/A
Calculated Conc: 0.528 umol/L
Acq. Date: 2025-05-16
Acq. Time: 01:21:04

Modified: Yes
Proc. Algorithm: Specify Parameters - MQ III

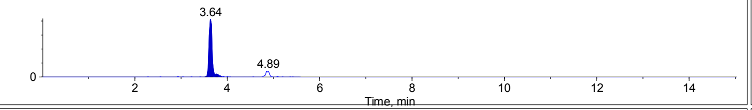

Sample Name: "ZW-12" Sample ID: "" File: "20250515-50AA.wiff"
Peak Name: "Pro" Mass(es): "286.150/171.010 Da"
Comment: "" Annotation: ""

Sample Index: 42
Sample Type: Unknown
Concentration: N/A
Calculated Conc: No Intercept
Acq. Date: 2025-05-16
Acq. Time: 01:21:04

Modified: Yes
Proc. Algorithm: Specify Parameters - MQ III

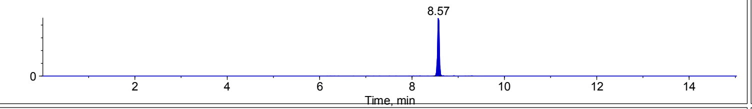

Sample Name: "ZW-12" Sample ID: "" File: "20250515-50AA.wiff"
Peak Name: "Val" Mass(es): "288.150/171.010 Da"
Comment: "" Annotation: ""

Sample Index: 42
Sample Type: Unknown
Concentration: N/A
Calculated Conc: 1.69 umol/L
Acq. Date: 2025-05-16
Acq. Time: 01:21:04

Modified: Yes
Proc. Algorithm: Specify Parameters - MQ III

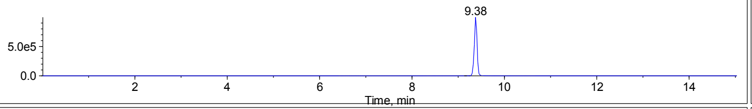

Sample Name: "ZW-12" Sample ID: "" File: "20250515-50AA.wiff"
Peak Name: "Thr" Mass(es): "290.100/171.010 Da"
Comment: "" Annotation: ""

Sample Index: 42
Sample Type: Unknown
Concentration: N/A
Calculated Conc: 1.40 umol/L
Acq. Date: 2025-05-16
Acq. Time: 01:21:04

Modified: Yes
Proc. Algorithm: Specify Parameters - MQ III

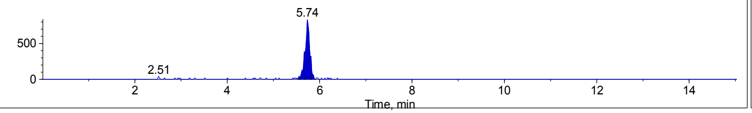

Sample Name: "ZW-12" Sample ID: "" File: "20250515-50AA.wiff"
Peak Name: "MSLAB-Nvl(IS)" Mass(es): "288.150/171.010 Da"
Comment: "" Annotation: ""

Sample Index: 42
Sample Type: Unknown
Concentration: 1.00 ng/mL
Calculated Conc: N/A
Acq. Date: 2025-05-16
Acq. Time: 01:21:04

Modified: Yes
Proc. Algorithm: Specify Parameters - MQ III

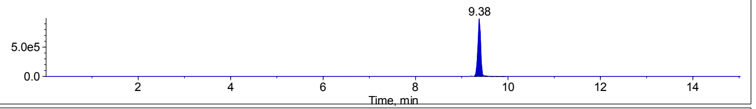

Sample Name: "ZW-12" Sample ID: "" File: "20250515-50AA.wiff"
Peak Name: "MSLAB-Nvl(IS)" Mass(es): "288.150/171.010 Da"
Comment: "" Annotation: ""

Sample Index: 42
Sample Type: Unknown
Concentration: 1.00 ng/mL
Calculated Conc: N/A
Acq. Date: 2025-05-16
Acq. Time: 01:21:04

Modified: Yes
Proc. Algorithm: Specify Parameters - MQ III

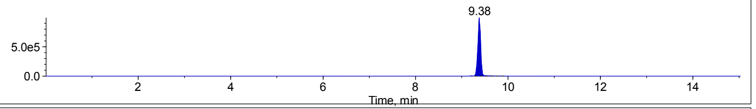

Sample Name: "ZW-12" Sample ID: "" File: "20250515-50AA.wiff"
Peak Name: "MSLAB-Nvl(IS)" Mass(es): "288.150/171.010 Da"
Comment: "" Annotation: ""

Sample Index: 42
Sample Type: Unknown
Concentration: 1.00 ng/mL
Calculated Conc: N/A
Acq. Date: 2025-05-16
Acq. Time: 01:21:04

Modified: Yes
Proc. Algorithm: Specify Parameters - MQ III

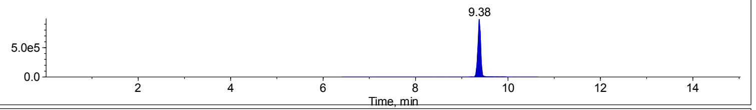

Sample Name: "ZW-12" Sample ID: "" File: "20250515-50AA.wiff"
Peak Name: "MSLAB-Nvl(IS)" Mass(es): "288.150/171.010 Da"
Comment: "" Annotation: ""

Sample Index: 42
Sample Type: Unknown
Concentration: 1.00 ng/mL
Calculated Conc: N/A
Acq. Date: 2025-05-16
Acq. Time: 01:21:04

Modified: Yes
Proc. Algorithm: Specify Parameters - MQ III

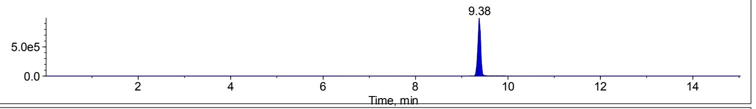

Sample Name: "ZW-12" Sample ID: "" File: "20250515-50AA.wiff"
Peak Name: "MSLAB-Nvl(IS)" Mass(es): "288.150/171.010 Da"
Comment: "" Annotation: ""

Sample Index: 42
Sample Type: Unknown
Concentration: 1.00 ng/mL
Calculated Conc: N/A
Acq. Date: 2025-05-16
Acq. Time: 01:21:04

Modified: Yes
Proc. Algorithm: Specify Parameters - MQ III

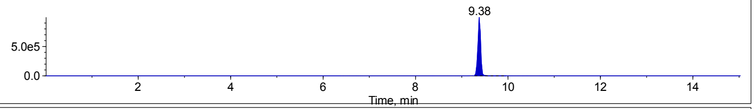

Sample Name: "ZW-12" Sample ID: "" File: "20250515-50AA.wiff"
Peak Name: "MSLAB-Nvl(IS)" Mass(es): "288.150/171.010 Da"
Comment: "" Annotation: ""

Sample Index: 42
Sample Type: Unknown
Concentration: 1.00 ng/mL
Calculated Conc: N/A
Acq. Date: 2025-05-16
Acq. Time: 01:21:04

Modified: Yes
Proc. Algorithm: Specify Parameters - MQ III

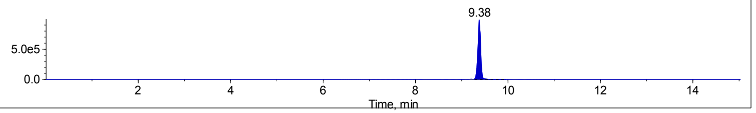

Sample Name: "ZW-12" Sample ID: "" File: "20250515-50AA.wiff"
Peak Name: "Cys1/2" Mass(es): "292.200/171.010 Da"
Comment: "" Annotation: ""

Sample Index: 42
Sample Type: Unknown
Concentration: N/A
Calculated Conc: < 0
Acq. Date: 2025-05-16
Acq. Time: 01:21:04

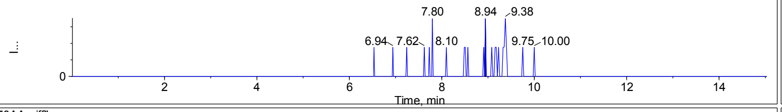

Modified: Yes
Proc. Algorithm: Specify Parameters - MQ III

Sample Name: "ZW-12" Sample ID: "" File: "20250515-50AA.wiff"
Peak Name: "MSLAB-Nvl(IS)" Mass(es): "288.150/171.010 Da"
Comment: "" Annotation: ""

Sample Index: 42
Sample Type: Unknown
Concentration: 1.00 ng/mL
Calculated Conc: N/A
Acq. Date: 2025-05-16
Acq. Time: 01:21:04

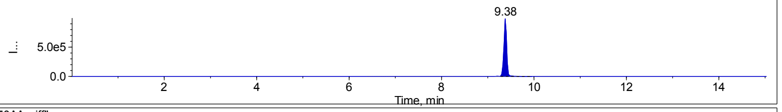

Modified: Yes
Proc. Algorithm: Specify Parameters - MQ III

Sample Name: "ZW-12" Sample ID: "" File: "20250515-50AA.wiff"
Peak Name: "Ile" Mass(es): "302.150/171.010 Da"
Comment: "" Annotation: ""

Sample Index: 42
Sample Type: Unknown
Concentration: N/A
Calculated Conc: 0.302 umol/L
Acq. Date: 2025-05-16
Acq. Time: 01:21:04

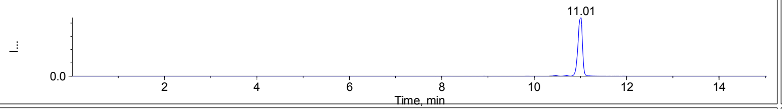

Modified: Yes
Proc. Algorithm: Specify Parameters - MQ III

Sample Name: "ZW-12" Sample ID: "" File: "20250515-50AA.wiff"
Peak Name: "MSLAB-Nvl(IS)" Mass(es): "288.150/171.010 Da"
Comment: "" Annotation: ""

Sample Index: 42
Sample Type: Unknown
Concentration: 1.00 ng/mL
Calculated Conc: N/A
Acq. Date: 2025-05-16
Acq. Time: 01:21:04

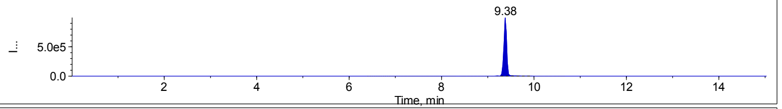

Modified: Yes
Proc. Algorithm: Specify Parameters - MQ III

Sample Name: "ZW-12" Sample ID: "" File: "20250515-50AA.wiff"
Peak Name: "Asn" Mass(es): "303.130/171.010 Da"
Comment: "" Annotation: ""

Sample Index: 42
Sample Type: Unknown
Concentration: N/A
Calculated Conc: < 0
Acq. Date: 2025-05-16
Acq. Time: 01:21:04

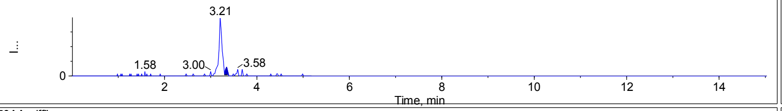

Modified: Yes
Proc. Algorithm: Specify Parameters - MQ III

Sample Name: "ZW-12" Sample ID: "" File: "20250515-50AA.wiff"
Peak Name: "MSLAB-Nvl(IS)" Mass(es): "288.150/171.010 Da"
Comment: "" Annotation: ""

Sample Index: 42
Sample Type: Unknown
Concentration: 1.00 ng/mL
Calculated Conc: N/A
Acq. Date: 2025-05-16
Acq. Time: 01:21:04

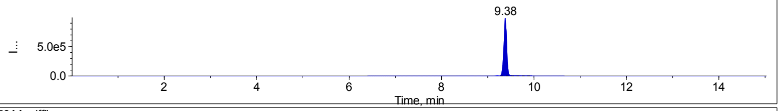

Modified: Yes
Proc. Algorithm: Specify Parameters - MQ III

Sample Name: "ZW-12" Sample ID: "" File: "20250515-50AA.wiff"
Peak Name: "Asp" Mass(es): "304.110/171.010 Da"
Comment: "" Annotation: ""

Sample Index: 42
Sample Type: Unknown
Concentration: N/A
Calculated Conc: No Intercept
Acq. Date: 2025-05-16
Acq. Time: 01:21:04

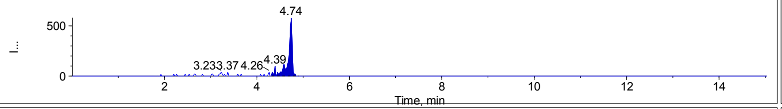

Modified: Yes
Proc. Algorithm: Specify Parameters - MQ III

Sample Name: "ZW-12" Sample ID: "" File: "20250515-50AA.wiff"
Peak Name: "MSLAB-Nvl(IS)" Mass(es): "288.150/171.010 Da"
Comment: "" Annotation: ""

Sample Index: 42
Sample Type: Unknown
Concentration: 1.00 ng/mL
Calculated Conc: N/A
Acq. Date: 2025-05-16
Acq. Time: 01:21:04

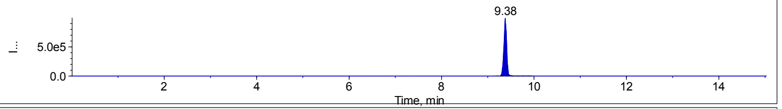

Modified: Yes
Proc. Algorithm: Specify Parameters - MQ III

Sample Name: "ZW-12" Sample ID: "" File: "20250515-50AA.wiff"
Peak Name: "Gln" Mass(es): "317.150/171.010 Da"
Comment: "" Annotation: ""

Sample Index: 42
Sample Type: Unknown
Concentration: N/A
Calculated Conc: < 0
Acq. Date: 2025-05-16
Acq. Time: 01:21:04

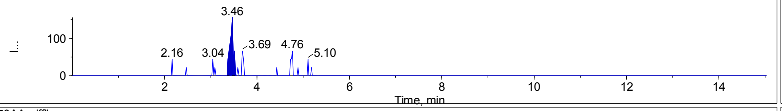

Modified: Yes
Proc. Algorithm: Specify Parameters - MQ III

Sample Name: "ZW-12" Sample ID: "" File: "20250515-50AA.wiff"
Peak Name: "MSLAB-Nvl(IS)" Mass(es): "288.150/171.010 Da"
Comment: "" Annotation: ""

Sample Index: 42
Sample Type: Unknown
Concentration: 1.00 ng/mL
Calculated Conc: N/A
Acq. Date: 2025-05-16
Acq. Time: 01:21:04

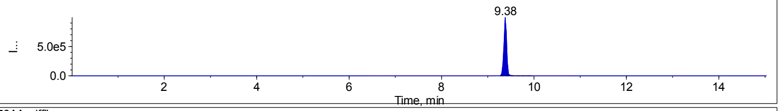

Modified: Yes
Proc. Algorithm: Specify Parameters - MQ III

Sample Name: "ZW-12" Sample ID: "" File: "20250515-50AA.wiff"
Peak Name: "Glu" Mass(es): "318.100/171.010 Da"
Comment: "" Annotation: ""

Sample Index: 42
Sample Type: Unknown
Concentration: N/A
Calculated Conc: 1.72 umol/L
Acq. Date: 2025-05-16
Acq. Time: 01:21:04

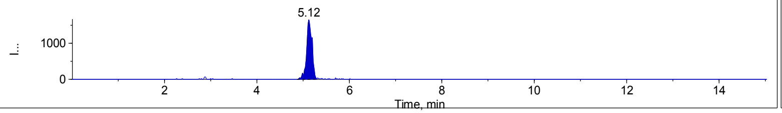

Modified: Yes
Proc. Algorithm: Specify Parameters - MQ III

Sample Name: "ZW-12" Sample ID: "" File: "20250515-50AA.wiff"
Peak Name: "MSLAB-Nvl(IS)" Mass(es): "288.150/171.010 Da"
Comment: "" Annotation: ""

Sample Index: 42
Sample Type: Unknown
Concentration: 1.00 ng/mL
Calculated Conc: N/A
Acq. Date: 2025-05-16
Acq. Time: 01:21:04

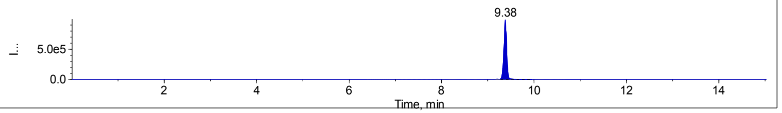

Modified: Yes
Proc. Algorithm: Specify Parameters - MQ III

Sample Name: "ZW-12" Sample ID: "" File: "20250515-50AA.wiff"
Peak Name: "Met" Mass(es): "320.210/171.010 Da"
Comment: "" Annotation: ""

Sample Index: 42
Sample Type: Unknown
Concentration: N/A
Calculated Conc: < 0
Acq. Date: 2025-05-16
Acq. Time: 01:21:04
Modified: Yes
Proc. Algorithm: Specify Parameters - MQ III

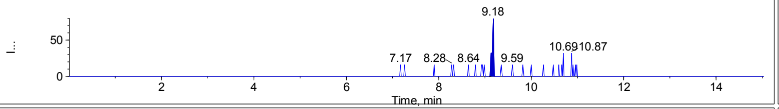

Sample Name: "ZW-12" Sample ID: "" File: "20250515-50AA.wiff"
Peak Name: "His" Mass(es): "326.150/171.010 Da"
Comment: "" Annotation: ""

Sample Index: 42
Sample Type: Unknown
Concentration: N/A
Calculated Conc: 0.549 umol/L
Acq. Date: 2025-05-16
Acq. Time: 01:21:04
Modified: Yes
Proc. Algorithm: Specify Parameters - MQ III

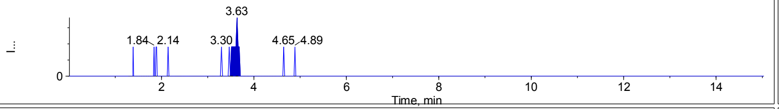

Sample Name: "ZW-12" Sample ID: "" File: "20250515-50AA.wiff"
Peak Name: "Phe" Mass(es): "336.200/171.010 Da"
Comment: "" Annotation: ""

Sample Index: 42
Sample Type: Unknown
Concentration: N/A
Calculated Conc: 0.461 umol/L
Acq. Date: 2025-05-16
Acq. Time: 01:21:04
Modified: Yes
Proc. Algorithm: Specify Parameters - MQ III

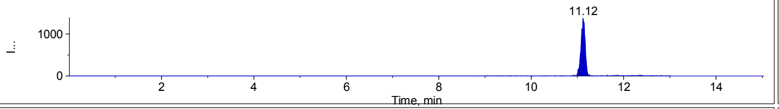

Sample Name: "ZW-12" Sample ID: "" File: "20250515-50AA.wiff"
Peak Name: "Arg" Mass(es): "345.210/171.010 Da"
Comment: "" Annotation: ""

Sample Index: 42
Sample Type: Unknown
Concentration: N/A
Calculated Conc: 2.40 umol/L
Acq. Date: 2025-05-16
Acq. Time: 01:21:04
Modified: Yes
Proc. Algorithm: Specify Parameters - MQ III

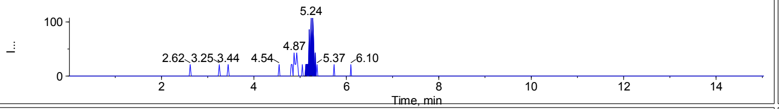

Sample Name: "ZW-12" Sample ID: "" File: "20250515-50AA.wiff"
Peak Name: "Trp" Mass(es): "375.200/171.010 Da"
Comment: "" Annotation: ""

Sample Index: 42
Sample Type: Unknown
Concentration: N/A
Calculated Conc: < 0
Acq. Date: 2025-05-16
Acq. Time: 01:21:04
Modified: Yes
Proc. Algorithm: Specify Parameters - MQ III

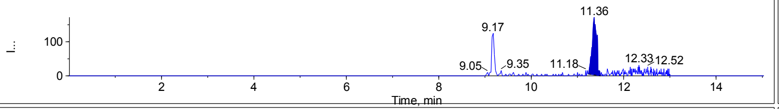

Sample Name: "ZW-12" Sample ID: "" File: "20250515-50AA.wiff"
Peak Name: "Lys" Mass(es): "487.200/171.010 Da"
Comment: "" Annotation: ""

Sample Index: 42
Sample Type: Unknown
Concentration: N/A
Calculated Conc: 1.64 umol/L
Acq. Date: 2025-05-16
Acq. Time: 01:21:04
Modified: Yes
Proc. Algorithm: Specify Parameters - MQ III

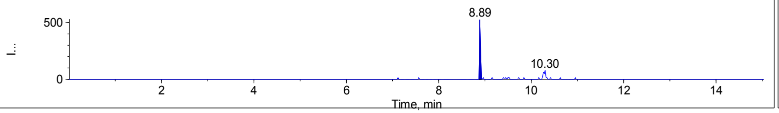

Sample Name: "ZW-12" Sample ID: "" File: "20250515-50AA.wiff"
Peak Name: "MSLAB-Nvl(IS)" Mass(es): "288.150/171.010 Da"
Comment: "" Annotation: ""

Sample Index: 42
Sample Type: Unknown
Concentration: 1.00 ng/mL
Calculated Conc: N/A
Acq. Date: 2025-05-16
Acq. Time: 01:21:04
Modified: Yes
Proc. Algorithm: Specify Parameters - MQ III

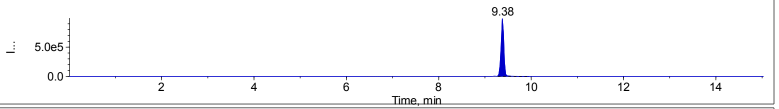

Sample Name: "ZW-12" Sample ID: "" File: "20250515-50AA.wiff"
Peak Name: "MSLAB-Nvl(IS)" Mass(es): "288.150/171.010 Da"
Comment: "" Annotation: ""

Sample Index: 42
Sample Type: Unknown
Concentration: 1.00 ng/mL
Calculated Conc: N/A
Acq. Date: 2025-05-16
Acq. Time: 01:21:04
Modified: Yes
Proc. Algorithm: Specify Parameters - MQ III

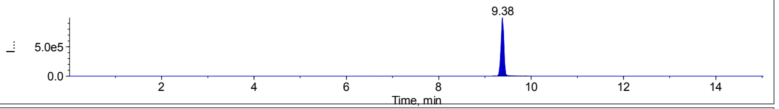

Sample Name: "ZW-12" Sample ID: "" File: "20250515-50AA.wiff"
Peak Name: "MSLAB-Nvl(IS)" Mass(es): "288.150/171.010 Da"
Comment: "" Annotation: ""

Sample Index: 42
Sample Type: Unknown
Concentration: 1.00 ng/mL
Calculated Conc: N/A
Acq. Date: 2025-05-16
Acq. Time: 01:21:04
Modified: Yes
Proc. Algorithm: Specify Parameters - MQ III

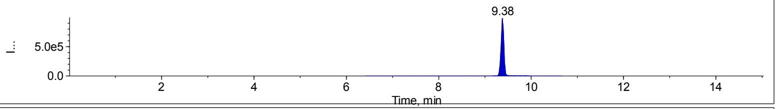

Sample Name: "ZW-12" Sample ID: "" File: "20250515-50AA.wiff"
Peak Name: "MSLAB-Nvl(IS)" Mass(es): "288.150/171.010 Da"
Comment: "" Annotation: ""

Sample Index: 42
Sample Type: Unknown
Concentration: 1.00 ng/mL
Calculated Conc: N/A
Acq. Date: 2025-05-16
Acq. Time: 01:21:04
Modified: Yes
Proc. Algorithm: Specify Parameters - MQ III

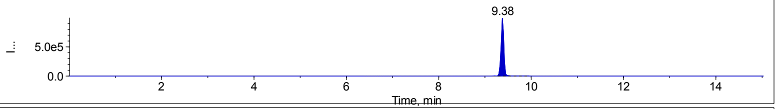

Sample Name: "ZW-12" Sample ID: "" File: "20250515-50AA.wiff"
Peak Name: "MSLAB-Nvl(IS)" Mass(es): "288.150/171.010 Da"
Comment: "" Annotation: ""

Sample Index: 42
Sample Type: Unknown
Concentration: 1.00 ng/mL
Calculated Conc: N/A
Acq. Date: 2025-05-16
Acq. Time: 01:21:04
Modified: Yes
Proc. Algorithm: Specify Parameters - MQ III

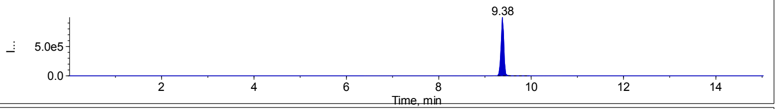

Sample Name: "ZW-12" Sample ID: "" File: "20250515-50AA.wiff"
Peak Name: "MSLAB-Nvl(IS)" Mass(es): "288.150/171.010 Da"
Comment: "" Annotation: ""

Sample Index: 42
Sample Type: Unknown
Concentration: 1.00 ng/mL
Calculated Conc: N/A
Acq. Date: 2025-05-16
Acq. Time: 01:21:04
Modified: Yes
Proc. Algorithm: Specify Parameters - MQ III

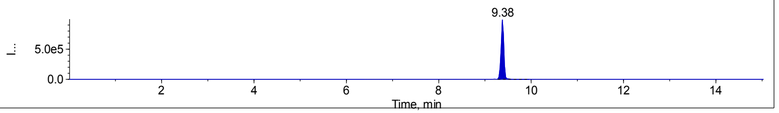

Sample Name: "ZW-12" Sample ID: "" File: "20250515-50AA.wiff"
Peak Name: "tyr" Mass(es): "352.200/171.010 Da"
Comment: "" Annotation: ""
Sample Index: 42
Sample Type: Unknown
Concentration: N/A
Calculated Conc: No Intercept
Acq. Date: 2025-05-16
Acq. Time: 01:21:04
Modified: Yes
Proc. Algorithm: Specify Parameters - MQ III

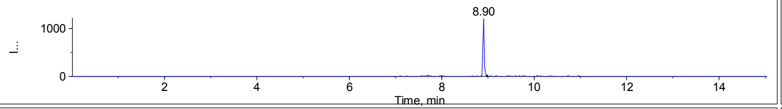

Sample Name: "ZW-12" Sample ID: "" File: "20250515-50AA.wiff"
Peak Name: "MSLAB-Nvl(IS)" Mass(es): "288.150/171.010 Da"
Comment: "" Annotation: ""
Sample Index: 42
Sample Type: Unknown
Concentration: 1.00 ng/mL
Calculated Conc: N/A
Acq. Date: 2025-05-16
Acq. Time: 01:21:04
Modified: Yes
Proc. Algorithm: Specify Parameters - MQ III

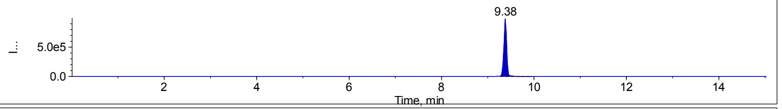

Sample Name: "ZW-12" Sample ID: "" File: "20250515-50AA.wiff"
Peak Name: "Leu" Mass(es): "302.150/171.010 Da"
Comment: "" Annotation: ""
Sample Index: 42
Sample Type: Unknown
Concentration: N/A
Calculated Conc: 0.755 umol/L
Acq. Date: 2025-05-16
Acq. Time: 01:21:04
Modified: Yes
Proc. Algorithm: Specify Parameters - MQ III

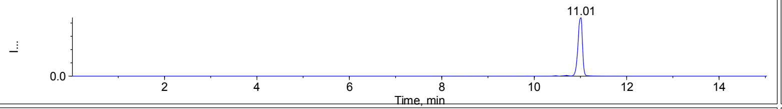

Sample Name: "ZW-12" Sample ID: "" File: "20250515-50AA.wiff"
Peak Name: "MSLAB-Nvl(IS)" Mass(es): "288.150/171.010 Da"
Comment: "" Annotation: ""
Sample Index: 42
Sample Type: Unknown
Concentration: 1.00 ng/mL
Calculated Conc: N/A
Acq. Date: 2025-05-16
Acq. Time: 01:21:04
Modified: Yes
Proc. Algorithm: Specify Parameters - MQ III

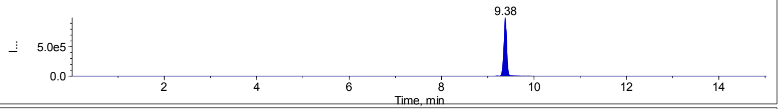

Sample Name: "ZW-13" Sample ID: "" File: "20250515-50AA.wiff"
Peak Name: "Gly" Mass(es): "246.080/171.010 Da"
Comment: "" Annotation: ""
Sample Index: 43
Sample Type: Unknown
Concentration: N/A
Calculated Conc: 1.28 umol/L
Acq. Date: 2025-05-16
Acq. Time: 01:36:31
Modified: Yes
Proc. Algorithm: Specify Parameters - MQ III

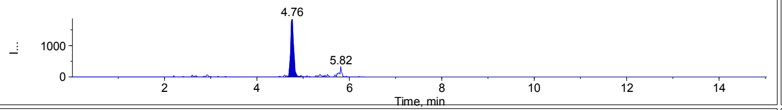

Sample Name: "ZW-13" Sample ID: "" File: "20250515-50AA.wiff"
Peak Name: "MSLAB-Nvl(IS)" Mass(es): "288.150/171.010 Da"
Comment: "" Annotation: ""
Sample Index: 43
Sample Type: Unknown
Concentration: 1.00 ng/mL
Calculated Conc: N/A
Acq. Date: 2025-05-16
Acq. Time: 01:36:31
Modified: Yes
Proc. Algorithm: Specify Parameters - MQ III

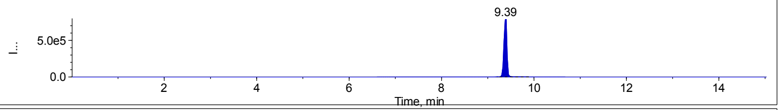

Sample Name: "ZW-13" Sample ID: "" File: "20250515-50AA.wiff"
Peak Name: "Ala" Mass(es): "260.100/171.010 Da"
Comment: "" Annotation: ""
Sample Index: 43
Sample Type: Unknown
Concentration: N/A
Calculated Conc: No Intercept
Acq. Date: 2025-05-16
Acq. Time: 01:36:31
Modified: Yes
Proc. Algorithm: Specify Parameters - MQ III

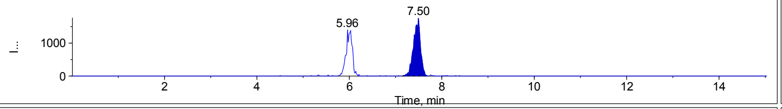

Sample Name: "ZW-13" Sample ID: "" File: "20250515-50AA.wiff"
Peak Name: "MSLAB-Nvl(IS)" Mass(es): "288.150/171.010 Da"
Comment: "" Annotation: ""
Sample Index: 43
Sample Type: Unknown
Concentration: 1.00 ng/mL
Calculated Conc: N/A
Acq. Date: 2025-05-16
Acq. Time: 01:36:31
Modified: Yes
Proc. Algorithm: Specify Parameters - MQ III

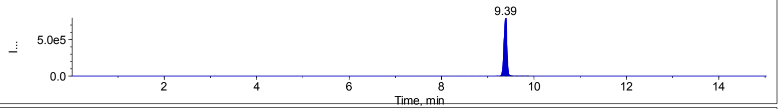

Sample Name: "ZW-13" Sample ID: "" File: "20250515-50AA.wiff"
Peak Name: "Ser" Mass(es): "276.100/171.010 Da"
Comment: "" Annotation: ""
Sample Index: 43
Sample Type: Unknown
Concentration: N/A
Calculated Conc: No Intercept
Acq. Date: 2025-05-16
Acq. Time: 01:36:31
Modified: Yes
Proc. Algorithm: Specify Parameters - MQ III

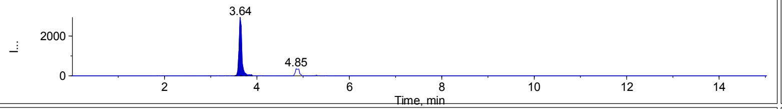

Sample Name: "ZW-13" Sample ID: "" File: "20250515-50AA.wiff"
Peak Name: "MSLAB-Nvl(IS)" Mass(es): "288.150/171.010 Da"
Comment: "" Annotation: ""
Sample Index: 43
Sample Type: Unknown
Concentration: 1.00 ng/mL
Calculated Conc: N/A
Acq. Date: 2025-05-16
Acq. Time: 01:36:31
Modified: Yes
Proc. Algorithm: Specify Parameters - MQ III

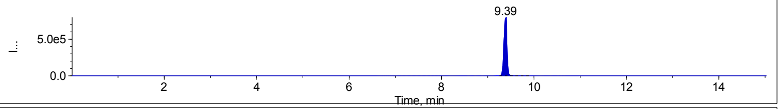

Sample Name: "ZW-13" Sample ID: "" File: "20250515-50AA.wiff"
Peak Name: "Pro" Mass(es): "286.150/171.010 Da"
Comment: "" Annotation: ""
Sample Index: 43
Sample Type: Unknown
Concentration: N/A
Calculated Conc: No Intercept
Acq. Date: 2025-05-16
Acq. Time: 01:36:31
Modified: Yes
Proc. Algorithm: Specify Parameters - MQ III

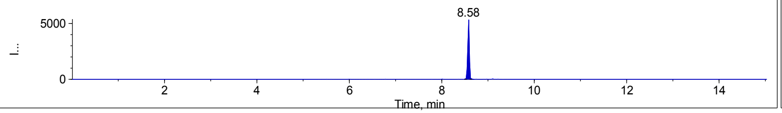

Sample Name: "ZW-13" Sample ID: "" File: "20250515-50AA.wiff"
Peak Name: "MSLAB-Nvl(IS)" Mass(es): "288.150/171.010 Da"
Comment: "" Annotation: ""
Sample Index: 43
Sample Type: Unknown
Concentration: 1.00 ng/mL
Calculated Conc: N/A
Acq. Date: 2025-05-16
Acq. Time: 01:36:31
Modified: Yes
Proc. Algorithm: Specify Parameters - MQ III

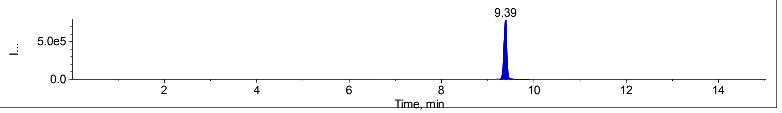

Sample Name: "ZW-13"   Sample ID: ""   File: "20250515-50AA.wiff"
Peak Name: "Val"   Mass(es): "288.150/171.010 Da"
Comment: ""   Annotation: ""

```
Sample Index:       43
Sample Type:      Unknown
Concentration:       N/A
Calculated Conc:     1.60    umol/L
Acq. Date:         2025-05-16
Acq. Time:         01:36:31

Modified:            Yes
Proc. Algorithm: Specify Parameters - MQ III
```

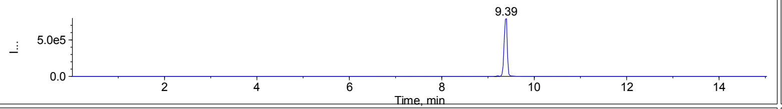

Sample Name: "ZW-13"   Sample ID: ""   File: "20250515-50AA.wiff"
Peak Name: "MSLAB-Nvl(IS)"   Mass(es): "288.150/171.010 Da"
Comment: ""   Annotation: ""

```
Sample Index:       43
Sample Type:      Unknown
Concentration:       1.00    ng/mL
Calculated Conc:     N/A
Acq. Date:         2025-05-16
Acq. Time:         01:36:31

Modified:            Yes
Proc. Algorithm: Specify Parameters - MQ III
```

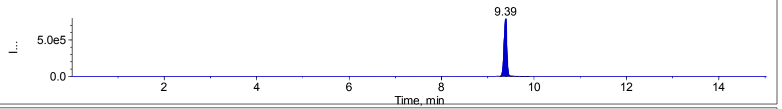

Sample Name: "ZW-13"   Sample ID: ""   File: "20250515-50AA.wiff"
Peak Name: "Thr"   Mass(es): "290.100/171.010 Da"
Comment: ""   Annotation: ""

```
Sample Index:       43
Sample Type:      Unknown
Concentration:       N/A
Calculated Conc:     1.18    umol/L
Acq. Date:         2025-05-16
Acq. Time:         01:36:31

Modified:            Yes
Proc. Algorithm: Specify Parameters - MQ III
```

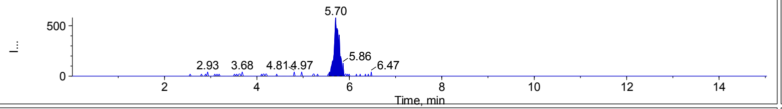

Sample Name: "ZW-13"   Sample ID: ""   File: "20250515-50AA.wiff"
Peak Name: "MSLAB-Nvl(IS)"   Mass(es): "288.150/171.010 Da"
Comment: ""   Annotation: ""

```
Sample Index:       43
Sample Type:      Unknown
Concentration:       1.00    ng/mL
Calculated Conc:     N/A
Acq. Date:         2025-05-16
Acq. Time:         01:36:31

Modified:            Yes
Proc. Algorithm: Specify Parameters - MQ III
```

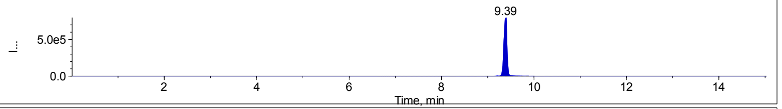

Sample Name: "ZW-13"   Sample ID: ""   File: "20250515-50AA.wiff"
Peak Name: "Cys1/2"   Mass(es): "292.200/171.010 Da"
Comment: ""   Annotation: ""

```
Sample Index:       43
Sample Type:      Unknown
Concentration:       N/A
Calculated Conc:      < 0
Acq. Date:         2025-05-16
Acq. Time:         01:36:31

Modified:            Yes
Proc. Algorithm: Specify Parameters - MQ III
```

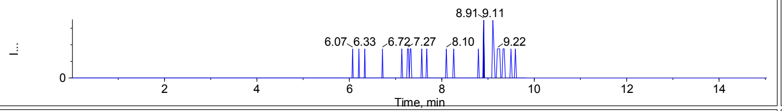

Sample Name: "ZW-13"   Sample ID: ""   File: "20250515-50AA.wiff"
Peak Name: "MSLAB-Nvl(IS)"   Mass(es): "288.150/171.010 Da"
Comment: ""   Annotation: ""

```
Sample Index:       43
Sample Type:      Unknown
Concentration:       1.00    ng/mL
Calculated Conc:     N/A
Acq. Date:         2025-05-16
Acq. Time:         01:36:31

Modified:            Yes
Proc. Algorithm: Specify Parameters - MQ III
```

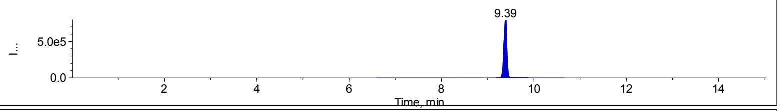

Sample Name: "ZW-13"   Sample ID: ""   File: "20250515-50AA.wiff"
Peak Name: "Ile"   Mass(es): "302.150/171.010 Da"
Comment: ""   Annotation: ""

```
Sample Index:       43
Sample Type:      Unknown
Concentration:       N/A
Calculated Conc:     0.213    umol/L
Acq. Date:         2025-05-16
Acq. Time:         01:36:31

Modified:            Yes
Proc. Algorithm: Specify Parameters - MQ III
```

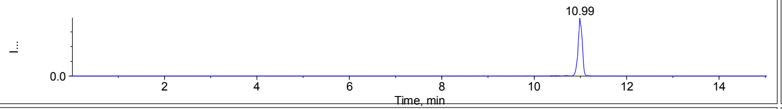

Sample Name: "ZW-13"   Sample ID: ""   File: "20250515-50AA.wiff"
Peak Name: "MSLAB-Nvl(IS)"   Mass(es): "288.150/171.010 Da"
Comment: ""   Annotation: ""

```
Sample Index:       43
Sample Type:      Unknown
Concentration:       1.00    ng/mL
Calculated Conc:     N/A
Acq. Date:         2025-05-16
Acq. Time:         01:36:31

Modified:            Yes
Proc. Algorithm: Specify Parameters - MQ III
```

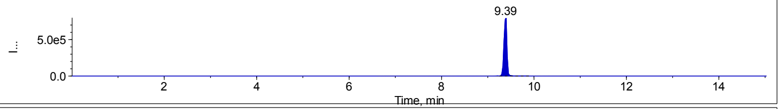

Sample Name: "ZW-13"   Sample ID: ""   File: "20250515-50AA.wiff"
Peak Name: "Asn"   Mass(es): "303.130/171.010 Da"
Comment: ""   Annotation: ""

```
Sample Index:       43
Sample Type:      Unknown
Concentration:       N/A
Calculated Conc:      < 0
Acq. Date:         2025-05-16
Acq. Time:         01:36:31

Modified:            Yes
Proc. Algorithm: Specify Parameters - MQ III
```

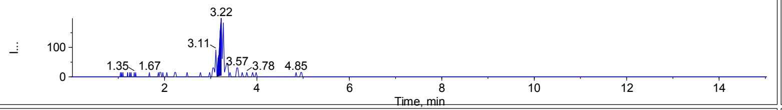

Sample Name: "ZW-13"   Sample ID: ""   File: "20250515-50AA.wiff"
Peak Name: "MSLAB-Nvl(IS)"   Mass(es): "288.150/171.010 Da"
Comment: ""   Annotation: ""

```
Sample Index:       43
Sample Type:      Unknown
Concentration:       1.00    ng/mL
Calculated Conc:     N/A
Acq. Date:         2025-05-16
Acq. Time:         01:36:31

Modified:            Yes
Proc. Algorithm: Specify Parameters - MQ III
```

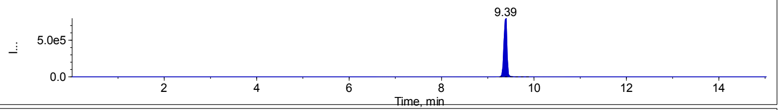

Sample Name: "ZW-13"   Sample ID: ""   File: "20250515-50AA.wiff"
Peak Name: "Asp"   Mass(es): "304.110/171.010 Da"
Comment: ""   Annotation: ""

```
Sample Index:       43
Sample Type:      Unknown
Concentration:       N/A
Calculated Conc:     1.34    umol/L
Acq. Date:         2025-05-16
Acq. Time:         01:36:31

Modified:            Yes
Proc. Algorithm: Specify Parameters - MQ III
```

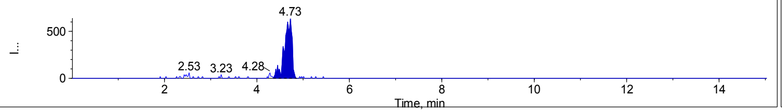

Sample Name: "ZW-13"   Sample ID: ""   File: "20250515-50AA.wiff"
Peak Name: "MSLAB-Nvl(IS)"   Mass(es): "288.150/171.010 Da"
Comment: ""   Annotation: ""

```
Sample Index:       43
Sample Type:      Unknown
Concentration:       1.00    ng/mL
Calculated Conc:     N/A
Acq. Date:         2025-05-16
Acq. Time:         01:36:31

Modified:            Yes
Proc. Algorithm: Specify Parameters - MQ III
```

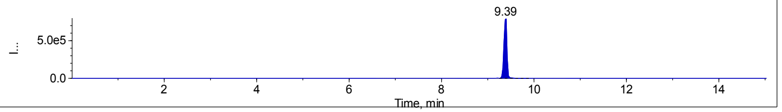

Sample Name: "ZW-13" Sample ID: "" File: "20250515-50AA.wiff"
Peak Name: "Gln" Mass(es): "317.150/171.010 Da"
Comment: "" Annotation: ""
Sample Index: 43
Sample Type: Unknown
Concentration: N/A
Calculated Conc: < 0
Acq. Date: 2025-05-16
Acq. Time: 01:36:31
Modified: Yes
Proc. Algorithm: Specify Parameters - MQ III

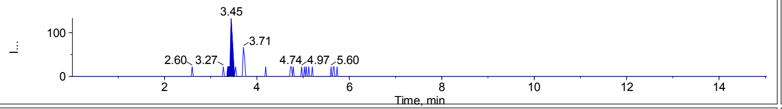

Sample Name: "ZW-13" Sample ID: "" File: "20250515-50AA.wiff"
Peak Name: "MSLAB-Nvl(IS)" Mass(es): "288.150/171.010 Da"
Comment: "" Annotation: ""
Sample Index: 43
Sample Type: Unknown
Concentration: 1.00 ng/mL
Calculated Conc: N/A
Acq. Date: 2025-05-16
Acq. Time: 01:36:31
Modified: Yes
Proc. Algorithm: Specify Parameters - MQ III

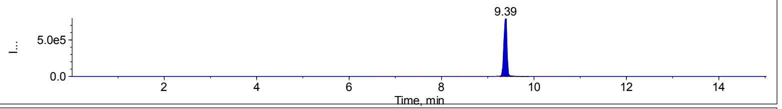

Sample Name: "ZW-13" Sample ID: "" File: "20250515-50AA.wiff"
Peak Name: "Glu" Mass(es): "318.100/171.010 Da"
Comment: "" Annotation: ""
Sample Index: 43
Sample Type: Unknown
Concentration: N/A
Calculated Conc: 1.69 umol/L
Acq. Date: 2025-05-16
Acq. Time: 01:36:31
Modified: Yes
Proc. Algorithm: Specify Parameters - MQ III

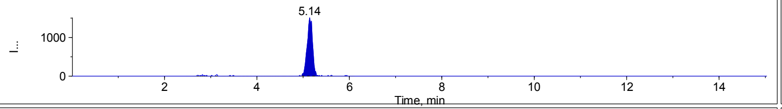

Sample Name: "ZW-13" Sample ID: "" File: "20250515-50AA.wiff"
Peak Name: "MSLAB-Nvl(IS)" Mass(es): "288.150/171.010 Da"
Comment: "" Annotation: ""
Sample Index: 43
Sample Type: Unknown
Concentration: 1.00 ng/mL
Calculated Conc: N/A
Acq. Date: 2025-05-16
Acq. Time: 01:36:31
Modified: Yes
Proc. Algorithm: Specify Parameters - MQ III

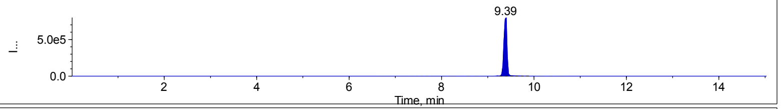

Sample Name: "ZW-13" Sample ID: "" File: "20250515-50AA.wiff"
Peak Name: "Met" Mass(es): "320.210/171.010 Da"
Comment: "" Annotation: ""
Sample Index: 43
Sample Type: Unknown
Concentration: N/A
Calculated Conc: < 0
Acq. Date: 2025-05-16
Acq. Time: 01:36:31
Modified: Yes
Proc. Algorithm: Specify Parameters - MQ III

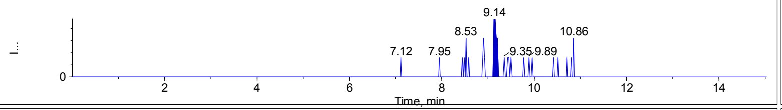

Sample Name: "ZW-13" Sample ID: "" File: "20250515-50AA.wiff"
Peak Name: "MSLAB-Nvl(IS)" Mass(es): "288.150/171.010 Da"
Comment: "" Annotation: ""
Sample Index: 43
Sample Type: Unknown
Concentration: 1.00 ng/mL
Calculated Conc: N/A
Acq. Date: 2025-05-16
Acq. Time: 01:36:31
Modified: Yes
Proc. Algorithm: Specify Parameters - MQ III

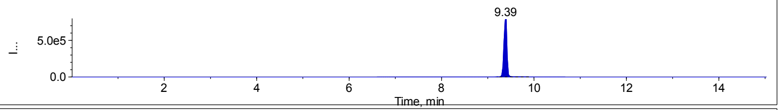

Sample Name: "ZW-13" Sample ID: "" File: "20250515-50AA.wiff"
Peak Name: "His" Mass(es): "326.150/171.010 Da"
Comment: "" Annotation: ""
Sample Index: 43
Sample Type: Unknown
Concentration: N/A
Calculated Conc: 0.466 umol/L
Acq. Date: 2025-05-16
Acq. Time: 01:36:31
Modified: Yes
Proc. Algorithm: Specify Parameters - MQ III

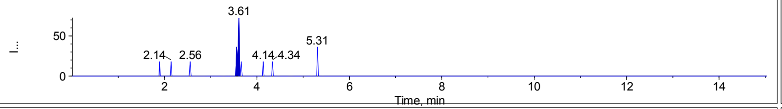

Sample Name: "ZW-13" Sample ID: "" File: "20250515-50AA.wiff"
Peak Name: "MSLAB-Nvl(IS)" Mass(es): "288.150/171.010 Da"
Comment: "" Annotation: ""
Sample Index: 43
Sample Type: Unknown
Concentration: 1.00 ng/mL
Calculated Conc: N/A
Acq. Date: 2025-05-16
Acq. Time: 01:36:31
Modified: Yes
Proc. Algorithm: Specify Parameters - MQ III

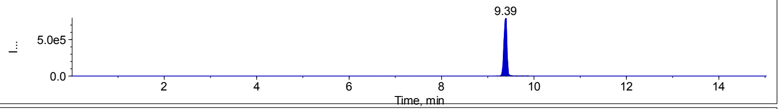

Sample Name: "ZW-13" Sample ID: "" File: "20250515-50AA.wiff"
Peak Name: "Phe" Mass(es): "336.200/171.010 Da"
Comment: "" Annotation: ""
Sample Index: 43
Sample Type: Unknown
Concentration: N/A
Calculated Conc: 0.424 umol/L
Acq. Date: 2025-05-16
Acq. Time: 01:36:31
Modified: Yes
Proc. Algorithm: Specify Parameters - MQ III

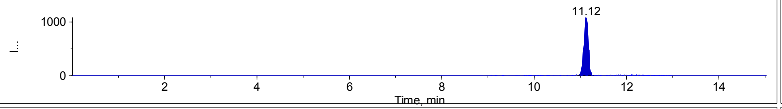

Sample Name: "ZW-13" Sample ID: "" File: "20250515-50AA.wiff"
Peak Name: "MSLAB-Nvl(IS)" Mass(es): "288.150/171.010 Da"
Comment: "" Annotation: ""
Sample Index: 43
Sample Type: Unknown
Concentration: 1.00 ng/mL
Calculated Conc: N/A
Acq. Date: 2025-05-16
Acq. Time: 01:36:31
Modified: Yes
Proc. Algorithm: Specify Parameters - MQ III

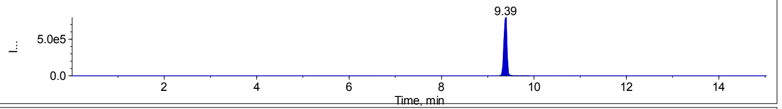

Sample Name: "ZW-13" Sample ID: "" File: "20250515-50AA.wiff"
Peak Name: "Arg" Mass(es): "345.210/171.010 Da"
Comment: "" Annotation: ""
Sample Index: 43
Sample Type: Unknown
Concentration: N/A
Calculated Conc: 2.13 umol/L
Acq. Date: 2025-05-16
Acq. Time: 01:36:31
Modified: Yes
Proc. Algorithm: Specify Parameters - MQ III

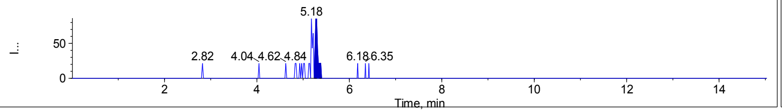

Sample Name: "ZW-13" Sample ID: "" File: "20250515-50AA.wiff"
Peak Name: "MSLAB-Nvl(IS)" Mass(es): "288.150/171.010 Da"
Comment: "" Annotation: ""
Sample Index: 43
Sample Type: Unknown
Concentration: 1.00 ng/mL
Calculated Conc: N/A
Acq. Date: 2025-05-16
Acq. Time: 01:36:31
Modified: Yes
Proc. Algorithm: Specify Parameters - MQ III

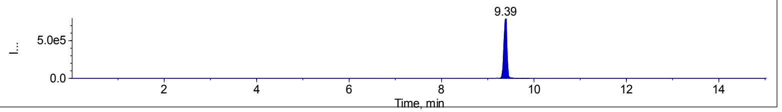

Sample Name: "ZW-13" Sample ID: "" File: "20250515-50AA.wiff"
Peak Name: "Trp" Mass(es): "375.200/171.010 Da"
Comment: "" Annotation: ""

Sample Index: 43
Sample Type: Unknown
Concentration: N/A
Calculated Conc: < 0
Acq. Date: 2025-05-16
Acq. Time: 01:36:31
Modified: Yes
Proc. Algorithm: Specify Parameters - MQ III

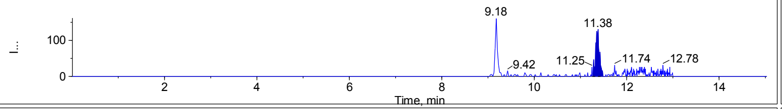

Sample Name: "ZW-13" Sample ID: "" File: "20250515-50AA.wiff"
Peak Name: "MSLAB-Nvl(IS)" Mass(es): "288.150/171.010 Da"
Comment: "" Annotation: ""

Sample Index: 43
Sample Type: Unknown
Concentration: 1.00 ng/mL
Calculated Conc: N/A
Acq. Date: 2025-05-16
Acq. Time: 01:36:31
Modified: Yes
Proc. Algorithm: Specify Parameters - MQ III

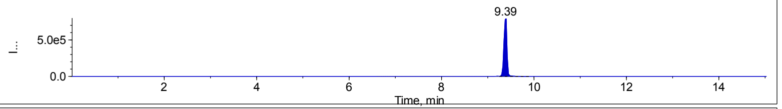

Sample Name: "ZW-13" Sample ID: "" File: "20250515-50AA.wiff"
Peak Name: "Lys" Mass(es): "487.200/171.010 Da"
Comment: "" Annotation: ""

Sample Index: 43
Sample Type: Unknown
Concentration: N/A
Calculated Conc: 1.94 umol/L
Acq. Date: 2025-05-16
Acq. Time: 01:36:31
Modified: Yes
Proc. Algorithm: Specify Parameters - MQ III

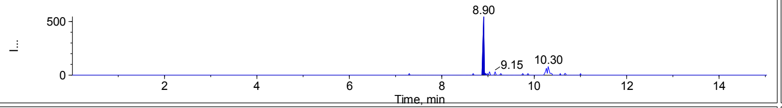

Sample Name: "ZW-13" Sample ID: "" File: "20250515-50AA.wiff"
Peak Name: "MSLAB-Nvl(IS)" Mass(es): "288.150/171.010 Da"
Comment: "" Annotation: ""

Sample Index: 43
Sample Type: Unknown
Concentration: 1.00 ng/mL
Calculated Conc: N/A
Acq. Date: 2025-05-16
Acq. Time: 01:36:31
Modified: Yes
Proc. Algorithm: Specify Parameters - MQ III

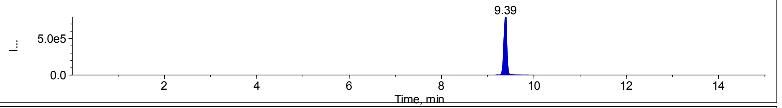

Sample Name: "ZW-13" Sample ID: "" File: "20250515-50AA.wiff"
Peak Name: "tyr" Mass(es): "352.200/171.010 Da"
Comment: "" Annotation: ""

Sample Index: 43
Sample Type: Unknown
Concentration: N/A
Calculated Conc: No Intercept
Acq. Date: 2025-05-16
Acq. Time: 01:36:31
Modified: Yes
Proc. Algorithm: Specify Parameters - MQ III

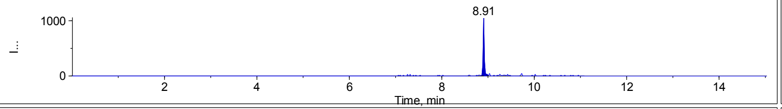

Sample Name: "ZW-13" Sample ID: "" File: "20250515-50AA.wiff"
Peak Name: "MSLAB-Nvl(IS)" Mass(es): "288.150/171.010 Da"
Comment: "" Annotation: ""

Sample Index: 43
Sample Type: Unknown
Concentration: 1.00 ng/mL
Calculated Conc: N/A
Acq. Date: 2025-05-16
Acq. Time: 01:36:31
Modified: Yes
Proc. Algorithm: Specify Parameters - MQ III

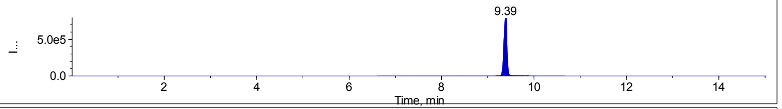

Sample Name: "ZW-13" Sample ID: "" File: "20250515-50AA.wiff"
Peak Name: "Leu" Mass(es): "302.150/171.010 Da"
Comment: "" Annotation: ""

Sample Index: 43
Sample Type: Unknown
Concentration: N/A
Calculated Conc: 0.747 umol/L
Acq. Date: 2025-05-16
Acq. Time: 01:36:31
Modified: Yes
Proc. Algorithm: Specify Parameters - MQ III

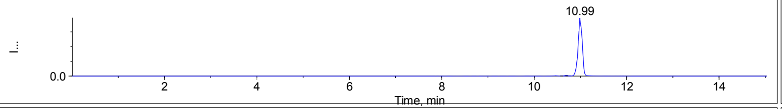

Sample Name: "ZW-13" Sample ID: "" File: "20250515-50AA.wiff"
Peak Name: "MSLAB-Nvl(IS)" Mass(es): "288.150/171.010 Da"
Comment: "" Annotation: ""

Sample Index: 43
Sample Type: Unknown
Concentration: 1.00 ng/mL
Calculated Conc: N/A
Acq. Date: 2025-05-16
Acq. Time: 01:36:31
Modified: Yes
Proc. Algorithm: Specify Parameters - MQ III

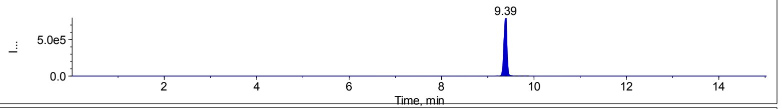

Sample Name: "ZY-3" Sample ID: "" File: "20250515-50AA.wiff"
Peak Name: "Gly" Mass(es): "246.080/171.010 Da"
Comment: "" Annotation: ""

Sample Index: 44
Sample Type: Unknown
Concentration: N/A
Calculated Conc: 6.03 umol/L
Acq. Date: 2025-05-16
Acq. Time: 01:52:01
Modified: Yes
Proc. Algorithm: Specify Parameters - MQ III

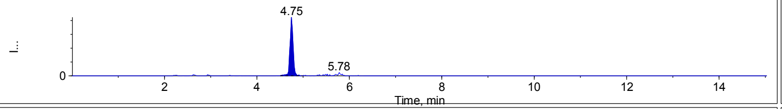

Sample Name: "ZY-3" Sample ID: "" File: "20250515-50AA.wiff"
Peak Name: "MSLAB-Nvl(IS)" Mass(es): "288.150/171.010 Da"
Comment: "" Annotation: ""

Sample Index: 44
Sample Type: Unknown
Concentration: 1.00 ng/mL
Calculated Conc: N/A
Acq. Date: 2025-05-16
Acq. Time: 01:52:01
Modified: Yes
Proc. Algorithm: Specify Parameters - MQ III

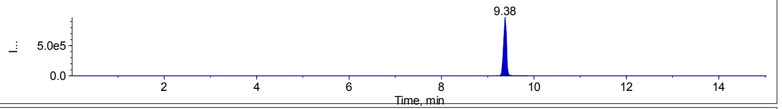

Sample Name: "ZY-3" Sample ID: "" File: "20250515-50AA.wiff"
Peak Name: "Ala" Mass(es): "260.100/171.010 Da"
Comment: "" Annotation: ""

Sample Index: 44
Sample Type: Unknown
Concentration: N/A
Calculated Conc: No Intercept
Acq. Date: 2025-05-16
Acq. Time: 01:52:01
Modified: Yes
Proc. Algorithm: Specify Parameters - MQ III

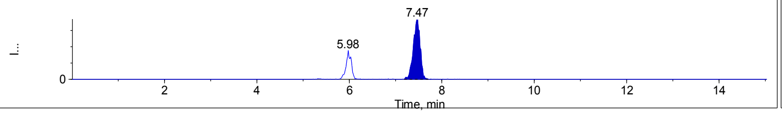

Sample Name: "ZY-3" Sample ID: "" File: "20250515-50AA.wiff"
Peak Name: "MSLAB-Nvl(IS)" Mass(es): "288.150/171.010 Da"
Comment: "" Annotation: ""

Sample Index: 44
Sample Type: Unknown
Concentration: 1.00 ng/mL
Calculated Conc: N/A
Acq. Date: 2025-05-16
Acq. Time: 01:52:01
Modified: Yes
Proc. Algorithm: Specify Parameters - MQ III

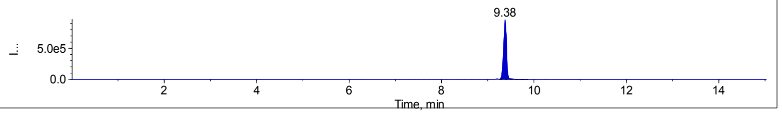

Sample Name: "ZY-3" Sample ID: "" File: "20250515-50AA.wiff"
Peak Name: "Ser" Mass(es): "276.100/171.010 Da"
Comment: "" Annotation: ""
Sample Index: 44
Sample Type: Unknown
Concentration: N/A
Calculated Conc: 1.37 umol/L
Acq. Date: 2025-05-16
Acq. Time: 01:52:01
Modified: Yes
Proc. Algorithm: Specify Parameters - MQ III

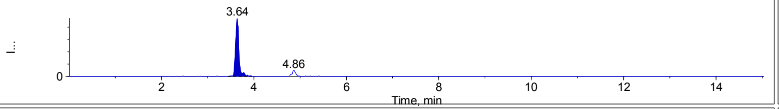

Sample Name: "ZY-3" Sample ID: "" File: "20250515-50AA.wiff"
Peak Name: "MSLAB-Nvl(IS)" Mass(es): "288.150/171.010 Da"
Comment: "" Annotation: ""
Sample Index: 44
Sample Type: Unknown
Concentration: 1.00 ng/mL
Calculated Conc: N/A
Acq. Date: 2025-05-16
Acq. Time: 01:52:01
Modified: Yes
Proc. Algorithm: Specify Parameters - MQ III

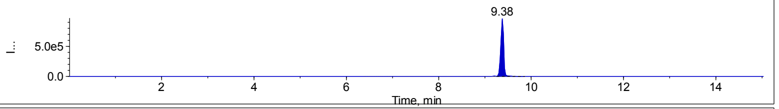

Sample Name: "ZY-3" Sample ID: "" File: "20250515-50AA.wiff"
Peak Name: "Pro" Mass(es): "286.150/171.010 Da"
Comment: "" Annotation: ""
Sample Index: 44
Sample Type: Unknown
Concentration: N/A
Calculated Conc: No Intercept
Acq. Date: 2025-05-16
Acq. Time: 01:52:01
Modified: Yes
Proc. Algorithm: Specify Parameters - MQ III

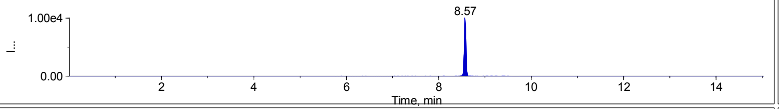

Sample Name: "ZY-3" Sample ID: "" File: "20250515-50AA.wiff"
Peak Name: "MSLAB-Nvl(IS)" Mass(es): "288.150/171.010 Da"
Comment: "" Annotation: ""
Sample Index: 44
Sample Type: Unknown
Concentration: 1.00 ng/mL
Calculated Conc: N/A
Acq. Date: 2025-05-16
Acq. Time: 01:52:01
Modified: Yes
Proc. Algorithm: Specify Parameters - MQ III

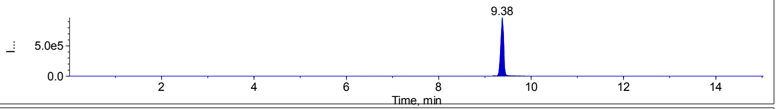

Sample Name: "ZY-3" Sample ID: "" File: "20250515-50AA.wiff"
Peak Name: "Val" Mass(es): "288.150/171.010 Da"
Comment: "" Annotation: ""
Sample Index: 44
Sample Type: Unknown
Concentration: N/A
Calculated Conc: 2.83 umol/L
Acq. Date: 2025-05-16
Acq. Time: 01:52:01
Modified: Yes
Proc. Algorithm: Specify Parameters - MQ III

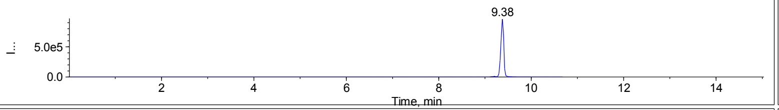

Sample Name: "ZY-3" Sample ID: "" File: "20250515-50AA.wiff"
Peak Name: "MSLAB-Nvl(IS)" Mass(es): "288.150/171.010 Da"
Comment: "" Annotation: ""
Sample Index: 44
Sample Type: Unknown
Concentration: 1.00 ng/mL
Calculated Conc: N/A
Acq. Date: 2025-05-16
Acq. Time: 01:52:01
Modified: Yes
Proc. Algorithm: Specify Parameters - MQ III

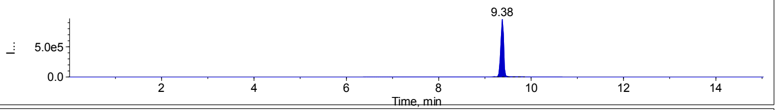

Sample Name: "ZY-3" Sample ID: "" File: "20250515-50AA.wiff"
Peak Name: "Thr" Mass(es): "290.100/171.010 Da"
Comment: "" Annotation: ""
Sample Index: 44
Sample Type: Unknown
Concentration: N/A
Calculated Conc: 2.03 umol/L
Acq. Date: 2025-05-16
Acq. Time: 01:52:01
Modified: Yes
Proc. Algorithm: Specify Parameters - MQ III

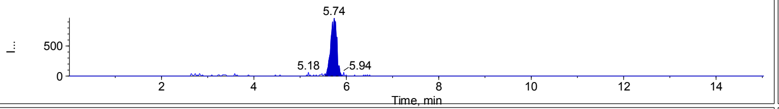

Sample Name: "ZY-3" Sample ID: "" File: "20250515-50AA.wiff"
Peak Name: "MSLAB-Nvl(IS)" Mass(es): "288.150/171.010 Da"
Comment: "" Annotation: ""
Sample Index: 44
Sample Type: Unknown
Concentration: 1.00 ng/mL
Calculated Conc: N/A
Acq. Date: 2025-05-16
Acq. Time: 01:52:01
Modified: Yes
Proc. Algorithm: Specify Parameters - MQ III

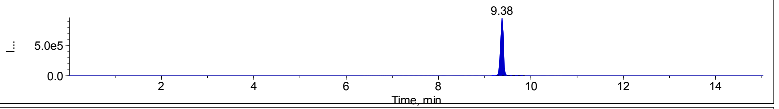

Sample Name: "ZY-3" Sample ID: "" File: "20250515-50AA.wiff"
Peak Name: "Cys1/2" Mass(es): "292.200/171.010 Da"
Comment: "" Annotation: ""
Sample Index: 44
Sample Type: Unknown
Concentration: N/A
Calculated Conc: 0.337 umol/L
Acq. Date: 2025-05-16
Acq. Time: 01:52:01
Modified: Yes
Proc. Algorithm: Specify Parameters - MQ III

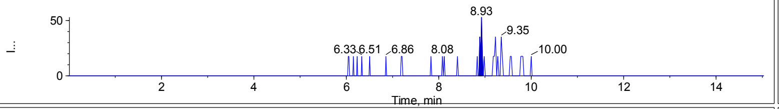

Sample Name: "ZY-3" Sample ID: "" File: "20250515-50AA.wiff"
Peak Name: "MSLAB-Nvl(IS)" Mass(es): "288.150/171.010 Da"
Comment: "" Annotation: ""
Sample Index: 44
Sample Type: Unknown
Concentration: 1.00 ng/mL
Calculated Conc: N/A
Acq. Date: 2025-05-16
Acq. Time: 01:52:01
Modified: Yes
Proc. Algorithm: Specify Parameters - MQ III

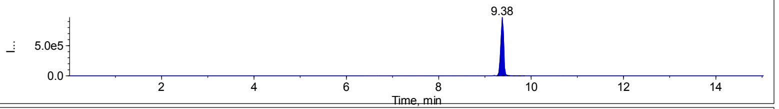

Sample Name: "ZY-3" Sample ID: "" File: "20250515-50AA.wiff"
Peak Name: "Ile" Mass(es): "302.150/171.010 Da"
Comment: "" Annotation: ""
Sample Index: 44
Sample Type: Unknown
Concentration: N/A
Calculated Conc: 0.862 umol/L
Acq. Date: 2025-05-16
Acq. Time: 01:52:01
Modified: Yes
Proc. Algorithm: Specify Parameters - MQ III

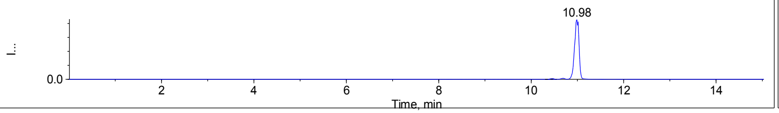

Sample Name: "ZY-3" Sample ID: "" File: "20250515-50AA.wiff"
Peak Name: "MSLAB-Nvl(IS)" Mass(es): "288.150/171.010 Da"
Comment: "" Annotation: ""
Sample Index: 44
Sample Type: Unknown
Concentration: 1.00 ng/mL
Calculated Conc: N/A
Acq. Date: 2025-05-16
Acq. Time: 01:52:01
Modified: Yes
Proc. Algorithm: Specify Parameters - MQ III

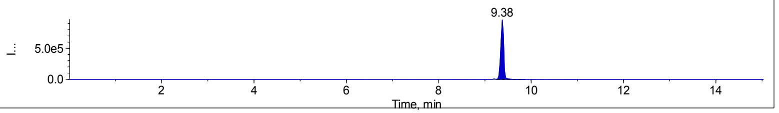

Sample Name: "ZY-3" Sample ID: "" File: "20250515-50AA.wiff"
Peak Name: "Asn" Mass(es): "303.130/171.010 Da"
Comment: "" Annotation: ""

Sample Index: 44
Sample Type: Unknown
Concentration: N/A
Calculated Conc: 0.00 umol/L
Acq. Date: 2025-05-16
Acq. Time: 01:52:01

Modified: Yes

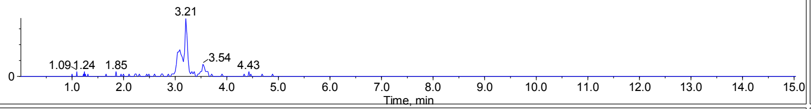

Sample Name: "ZY-3" Sample ID: "" File: "20250515-50AA.wiff"
Peak Name: "MSLAB-Nvl(IS)" Mass(es): "288.150/171.010 Da"
Comment: "" Annotation: ""

Sample Index: 44
Sample Type: Unknown
Concentration: 1.00 ng/mL
Calculated Conc: N/A
Acq. Date: 2025-05-16
Acq. Time: 01:52:01

Modified: Yes
Proc. Algorithm: Specify Parameters - MQ III

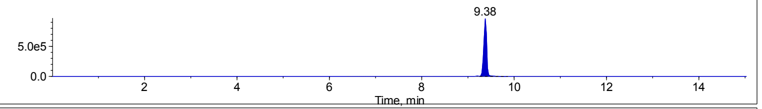

Sample Name: "ZY-3" Sample ID: "" File: "20250515-50AA.wiff"
Peak Name: "Asp" Mass(es): "304.110/171.010 Da"
Comment: "" Annotation: ""

Sample Index: 44
Sample Type: Unknown
Concentration: N/A
Calculated Conc: < 0
Acq. Date: 2025-05-16
Acq. Time: 01:52:01

Modified: Yes
Proc. Algorithm: Specify Parameters - MQ III

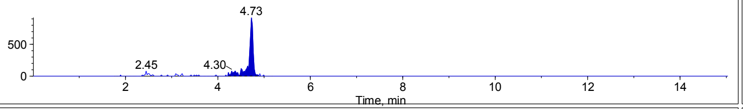

Sample Name: "ZY-3" Sample ID: "" File: "20250515-50AA.wiff"
Peak Name: "MSLAB-Nvl(IS)" Mass(es): "288.150/171.010 Da"
Comment: "" Annotation: ""

Sample Index: 44
Sample Type: Unknown
Concentration: 1.00 ng/mL
Calculated Conc: N/A
Acq. Date: 2025-05-16
Acq. Time: 01:52:01

Modified: Yes
Proc. Algorithm: Specify Parameters - MQ III

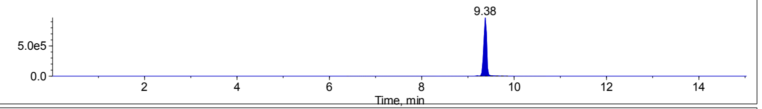

Sample Name: "ZY-3" Sample ID: "" File: "20250515-50AA.wiff"
Peak Name: "Gln" Mass(es): "317.150/171.010 Da"
Comment: "" Annotation: ""

Sample Index: 44
Sample Type: Unknown
Concentration: N/A
Calculated Conc: < 0
Acq. Date: 2025-05-16
Acq. Time: 01:52:01

Modified: Yes
Proc. Algorithm: Specify Parameters - MQ III

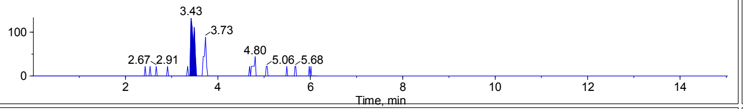

Sample Name: "ZY-3" Sample ID: "" File: "20250515-50AA.wiff"
Peak Name: "MSLAB-Nvl(IS)" Mass(es): "288.150/171.010 Da"
Comment: "" Annotation: ""

Sample Index: 44
Sample Type: Unknown
Concentration: 1.00 ng/mL
Calculated Conc: N/A
Acq. Date: 2025-05-16
Acq. Time: 01:52:01

Modified: Yes
Proc. Algorithm: Specify Parameters - MQ III

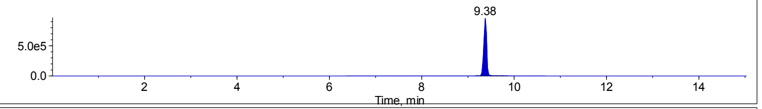

Sample Name: "ZY-3" Sample ID: "" File: "20250515-50AA.wiff"
Peak Name: "Glu" Mass(es): "318.100/171.010 Da"
Comment: "" Annotation: ""

Sample Index: 44
Sample Type: Unknown
Concentration: N/A
Calculated Conc: 3.44 umol/L
Acq. Date: 2025-05-16
Acq. Time: 01:52:01

Modified: Yes
Proc. Algorithm: Specify Parameters - MQ III

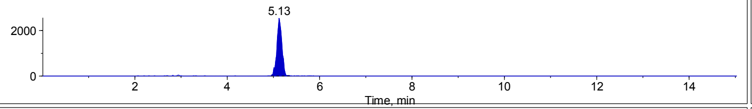

Sample Name: "ZY-3" Sample ID: "" File: "20250515-50AA.wiff"
Peak Name: "MSLAB-Nvl(IS)" Mass(es): "288.150/171.010 Da"
Comment: "" Annotation: ""

Sample Index: 44
Sample Type: Unknown
Concentration: 1.00 ng/mL
Calculated Conc: N/A
Acq. Date: 2025-05-16
Acq. Time: 01:52:01

Modified: Yes
Proc. Algorithm: Specify Parameters - MQ III

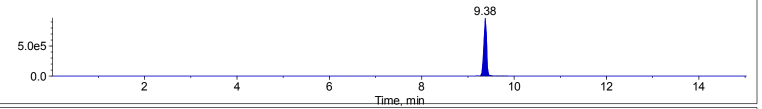

Sample Name: "ZY-3" Sample ID: "" File: "20250515-50AA.wiff"
Peak Name: "Met" Mass(es): "320.210/171.010 Da"
Comment: "" Annotation: ""

Sample Index: 44
Sample Type: Unknown
Concentration: N/A
Calculated Conc: < 0
Acq. Date: 2025-05-16
Acq. Time: 01:52:01

Modified: Yes
Proc. Algorithm: Specify Parameters - MQ III

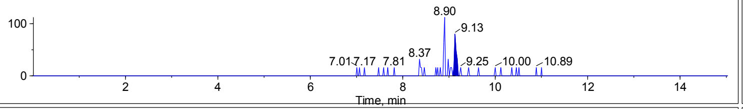

Sample Name: "ZY-3" Sample ID: "" File: "20250515-50AA.wiff"
Peak Name: "MSLAB-Nvl(IS)" Mass(es): "288.150/171.010 Da"
Comment: "" Annotation: ""

Sample Index: 44
Sample Type: Unknown
Concentration: 1.00 ng/mL
Calculated Conc: N/A
Acq. Date: 2025-05-16
Acq. Time: 01:52:01

Modified: Yes
Proc. Algorithm: Specify Parameters - MQ III

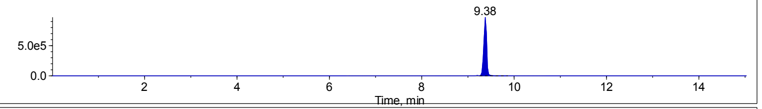

Sample Name: "ZY-3" Sample ID: "" File: "20250515-50AA.wiff"
Peak Name: "His" Mass(es): "326.150/171.010 Da"
Comment: "" Annotation: ""

Sample Index: 44
Sample Type: Unknown
Concentration: N/A
Calculated Conc: 1.44 umol/L
Acq. Date: 2025-05-16
Acq. Time: 01:52:01

Modified: Yes
Proc. Algorithm: Specify Parameters - MQ III

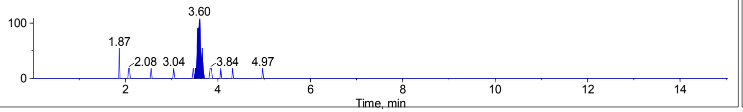

Sample Name: "ZY-3" Sample ID: "" File: "20250515-50AA.wiff"
Peak Name: "MSLAB-Nvl(IS)" Mass(es): "288.150/171.010 Da"
Comment: "" Annotation: ""

Sample Index: 44
Sample Type: Unknown
Concentration: 1.00 ng/mL
Calculated Conc: N/A
Acq. Date: 2025-05-16
Acq. Time: 01:52:01

Modified: Yes
Proc. Algorithm: Specify Parameters - MQ III

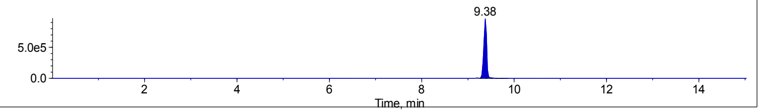

Sample Name: "ZY-3" Sample ID: "" File: "20250515-50AA.wiff"
Peak Name: "Phe" Mass(es): "336.200/171.010 Da"
Comment: "" Annotation: ""

Sample Index: 44
Sample Type: Unknown
Concentration: N/A
Calculated Conc: 0.982 umol/L
Acq. Date: 2025-05-16
Acq. Time: 01:52:01

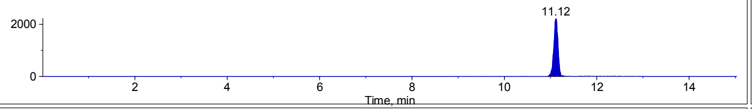

Modified: Yes
Proc. Algorithm: Specify Parameters - MQ III

Sample Name: "ZY-3" Sample ID: "" File: "20250515-50AA.wiff"
Peak Name: "Arg" Mass(es): "345.210/171.010 Da"
Comment: "" Annotation: ""

Sample Index: 44
Sample Type: Unknown
Concentration: N/A
Calculated Conc: 2.44 umol/L
Acq. Date: 2025-05-16
Acq. Time: 01:52:01

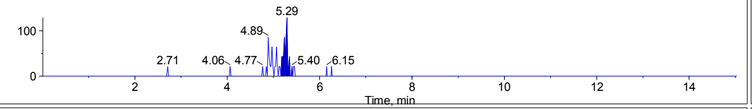

Modified: Yes
Proc. Algorithm: Specify Parameters - MQ III

Sample Name: "ZY-3" Sample ID: "" File: "20250515-50AA.wiff"
Peak Name: "Trp" Mass(es): "375.200/171.010 Da"
Comment: "" Annotation: ""

Sample Index: 44
Sample Type: Unknown
Concentration: N/A
Calculated Conc: < 0
Acq. Date: 2025-05-16
Acq. Time: 01:52:01

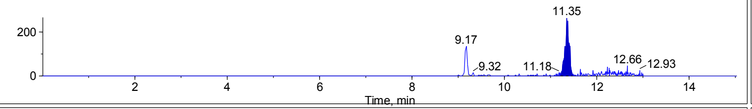

Modified: Yes
Proc. Algorithm: Specify Parameters - MQ III

Sample Name: "ZY-3" Sample ID: "" File: "20250515-50AA.wiff"
Peak Name: "Lys" Mass(es): "487.200/171.010 Da"
Comment: "" Annotation: ""

Sample Index: 44
Sample Type: Unknown
Concentration: N/A
Calculated Conc: 2.82 umol/L
Acq. Date: 2025-05-16
Acq. Time: 01:52:01

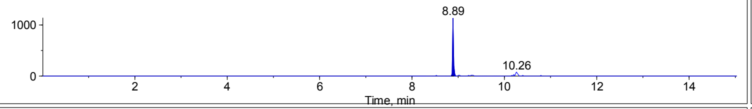

Modified: Yes
Proc. Algorithm: Specify Parameters - MQ III

Sample Name: "ZY-3" Sample ID: "" File: "20250515-50AA.wiff"
Peak Name: "tyr" Mass(es): "352.200/171.010 Da"
Comment: "" Annotation: ""

Sample Index: 44
Sample Type: Unknown
Concentration: N/A
Calculated Conc: No Intercept
Acq. Date: 2025-05-16
Acq. Time: 01:52:01

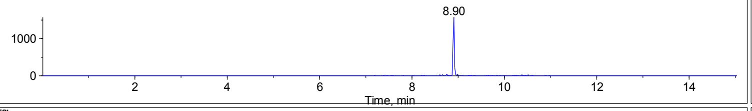

Modified: Yes
Proc. Algorithm: Specify Parameters - MQ III

Sample Name: "ZY-3" Sample ID: "" File: "20250515-50AA.wiff"
Peak Name: "Leu" Mass(es): "302.150/171.010 Da"
Comment: "" Annotation: ""

Sample Index: 44
Sample Type: Unknown
Concentration: N/A
Calculated Conc: 1.90 umol/L
Acq. Date: 2025-05-16
Acq. Time: 01:52:01

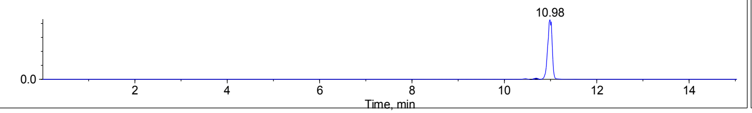

Modified: Yes
Proc. Algorithm: Specify Parameters - MQ III

Sample Name: "ZY-3" Sample ID: "" File: "20250515-50AA.wiff"
Peak Name: "MSLAB-Nvl(IS)" Mass(es): "288.150/171.010 Da"
Comment: "" Annotation: ""

Sample Index: 44
Sample Type: Unknown
Concentration: 1.00 ng/mL
Calculated Conc: N/A
Acq. Date: 2025-05-16
Acq. Time: 01:52:01

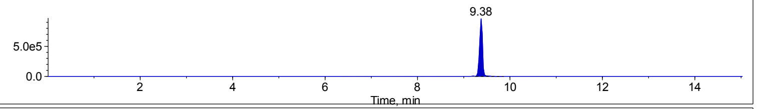

Modified: Yes
Proc. Algorithm: Specify Parameters - MQ III

Sample Name: "ZY-3" Sample ID: "" File: "20250515-50AA.wiff"
Peak Name: "MSLAB-Nvl(IS)" Mass(es): "288.150/171.010 Da"
Comment: "" Annotation: ""

Sample Index: 44
Sample Type: Unknown
Concentration: 1.00 ng/mL
Calculated Conc: N/A
Acq. Date: 2025-05-16
Acq. Time: 01:52:01

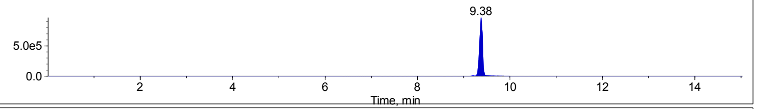

Modified: Yes
Proc. Algorithm: Specify Parameters - MQ III

Sample Name: "ZY-3" Sample ID: "" File: "20250515-50AA.wiff"
Peak Name: "MSLAB-Nvl(IS)" Mass(es): "288.150/171.010 Da"
Comment: "" Annotation: ""

Sample Index: 44
Sample Type: Unknown
Concentration: 1.00 ng/mL
Calculated Conc: N/A
Acq. Date: 2025-05-16
Acq. Time: 01:52:01

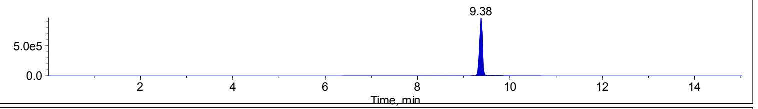

Modified: Yes
Proc. Algorithm: Specify Parameters - MQ III

Sample Name: "ZY-3" Sample ID: "" File: "20250515-50AA.wiff"
Peak Name: "MSLAB-Nvl(IS)" Mass(es): "288.150/171.010 Da"
Comment: "" Annotation: ""

Sample Index: 44
Sample Type: Unknown
Concentration: 1.00 ng/mL
Calculated Conc: N/A
Acq. Date: 2025-05-16
Acq. Time: 01:52:01

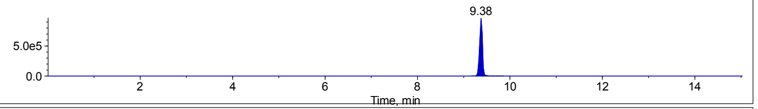

Modified: Yes
Proc. Algorithm: Specify Parameters - MQ III

Sample Name: "ZY-3" Sample ID: "" File: "20250515-50AA.wiff"
Peak Name: "MSLAB-Nvl(IS)" Mass(es): "288.150/171.010 Da"
Comment: "" Annotation: ""

Sample Index: 44
Sample Type: Unknown
Concentration: 1.00 ng/mL
Calculated Conc: N/A
Acq. Date: 2025-05-16
Acq. Time: 01:52:01

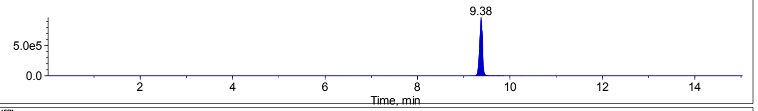

Modified: Yes
Proc. Algorithm: Specify Parameters - MQ III

Sample Name: "ZY-3" Sample ID: "" File: "20250515-50AA.wiff"
Peak Name: "MSLAB-Nvl(IS)" Mass(es): "288.150/171.010 Da"
Comment: "" Annotation: ""

Sample Index: 44
Sample Type: Unknown
Concentration: 1.00 ng/mL
Calculated Conc: N/A
Acq. Date: 2025-05-16
Acq. Time: 01:52:01

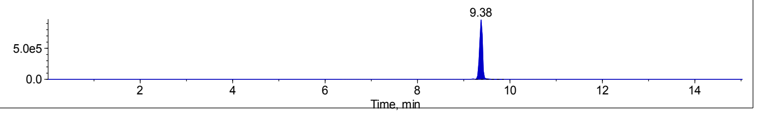

Modified: Yes
Proc. Algorithm: Specify Parameters - MQ III

Sample Name: "ZY-20" Sample ID: "" File: "20250515-50AA.wiff"
Peak Name: "Gly" Mass(es): "246.080/171.010 Da"
Comment: "" Annotation: ""
Sample Index: 45
Sample Type: Unknown
Concentration: N/A
Calculated Conc: 1.94 umol/L
Acq. Date: 2025-05-16
Acq. Time: 02:07:29
Modified: Yes
Proc. Algorithm: Specify Parameters - MQ III

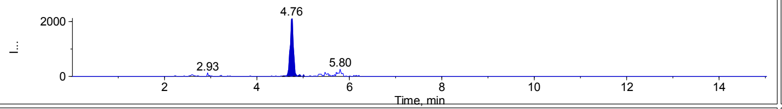

Sample Name: "ZY-20" Sample ID: "" File: "20250515-50AA.wiff"
Peak Name: "MSLAB-Nvl(IS)" Mass(es): "288.150/171.010 Da"
Comment: "" Annotation: ""
Sample Index: 45
Sample Type: Unknown
Concentration: 1.00 ng/mL
Calculated Conc: N/A
Acq. Date: 2025-05-16
Acq. Time: 02:07:29
Modified: Yes
Proc. Algorithm: Specify Parameters - MQ III

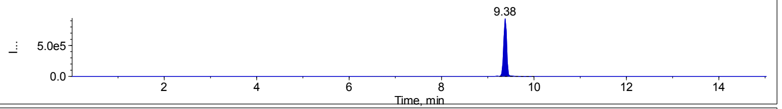

Sample Name: "ZY-20" Sample ID: "" File: "20250515-50AA.wiff"
Peak Name: "Ala" Mass(es): "260.100/171.010 Da"
Comment: "" Annotation: ""
Sample Index: 45
Sample Type: Unknown
Concentration: N/A
Calculated Conc: No Intercept
Acq. Date: 2025-05-16
Acq. Time: 02:07:29
Modified: Yes
Proc. Algorithm: Specify Parameters - MQ III

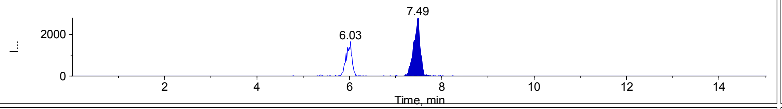

Sample Name: "ZY-20" Sample ID: "" File: "20250515-50AA.wiff"
Peak Name: "MSLAB-Nvl(IS)" Mass(es): "288.150/171.010 Da"
Comment: "" Annotation: ""
Sample Index: 45
Sample Type: Unknown
Concentration: 1.00 ng/mL
Calculated Conc: N/A
Acq. Date: 2025-05-16
Acq. Time: 02:07:29
Modified: Yes
Proc. Algorithm: Specify Parameters - MQ III

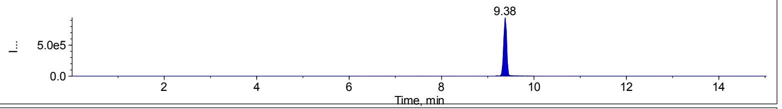

Sample Name: "ZY-20" Sample ID: "" File: "20250515-50AA.wiff"
Peak Name: "Ser" Mass(es): "276.100/171.010 Da"
Comment: "" Annotation: ""
Sample Index: 45
Sample Type: Unknown
Concentration: N/A
Calculated Conc: 0.0837 umol/L
Acq. Date: 2025-05-16
Acq. Time: 02:07:29
Modified: Yes
Proc. Algorithm: Specify Parameters - MQ III

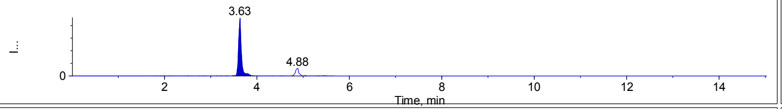

Sample Name: "ZY-20" Sample ID: "" File: "20250515-50AA.wiff"
Peak Name: "MSLAB-Nvl(IS)" Mass(es): "288.150/171.010 Da"
Comment: "" Annotation: ""
Sample Index: 45
Sample Type: Unknown
Concentration: 1.00 ng/mL
Calculated Conc: N/A
Acq. Date: 2025-05-16
Acq. Time: 02:07:29
Modified: Yes
Proc. Algorithm: Specify Parameters - MQ III

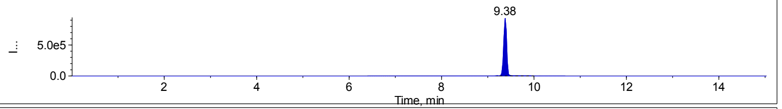

Sample Name: "ZY-20" Sample ID: "" File: "20250515-50AA.wiff"
Peak Name: "Pro" Mass(es): "286.150/171.010 Da"
Comment: "" Annotation: ""
Sample Index: 45
Sample Type: Unknown
Concentration: N/A
Calculated Conc: No Intercept
Acq. Date: 2025-05-16
Acq. Time: 02:07:29
Modified: Yes
Proc. Algorithm: Specify Parameters - MQ III

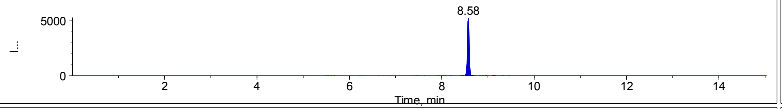

Sample Name: "ZY-20" Sample ID: "" File: "20250515-50AA.wiff"
Peak Name: "MSLAB-Nvl(IS)" Mass(es): "288.150/171.010 Da"
Comment: "" Annotation: ""
Sample Index: 45
Sample Type: Unknown
Concentration: 1.00 ng/mL
Calculated Conc: N/A
Acq. Date: 2025-05-16
Acq. Time: 02:07:29
Modified: Yes
Proc. Algorithm: Specify Parameters - MQ III

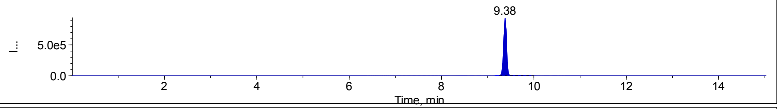

Sample Name: "ZY-20" Sample ID: "" File: "20250515-50AA.wiff"
Peak Name: "Val" Mass(es): "288.150/171.010 Da"
Comment: "" Annotation: ""
Sample Index: 45
Sample Type: Unknown
Concentration: N/A
Calculated Conc: 2.47 umol/L
Acq. Date: 2025-05-16
Acq. Time: 02:07:29
Modified: Yes
Proc. Algorithm: Specify Parameters - MQ III

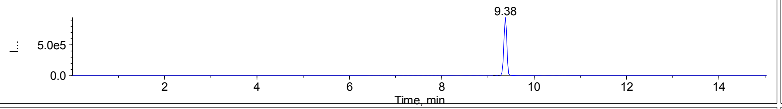

Sample Name: "ZY-20" Sample ID: "" File: "20250515-50AA.wiff"
Peak Name: "MSLAB-Nvl(IS)" Mass(es): "288.150/171.010 Da"
Comment: "" Annotation: ""
Sample Index: 45
Sample Type: Unknown
Concentration: 1.00 ng/mL
Calculated Conc: N/A
Acq. Date: 2025-05-16
Acq. Time: 02:07:29
Modified: Yes
Proc. Algorithm: Specify Parameters - MQ III

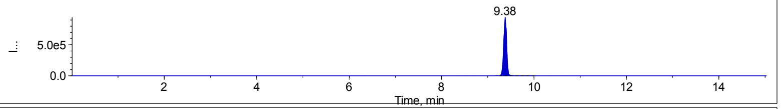

Sample Name: "ZY-20" Sample ID: "" File: "20250515-50AA.wiff"
Peak Name: "Thr" Mass(es): "290.100/171.010 Da"
Comment: "" Annotation: ""
Sample Index: 45
Sample Type: Unknown
Concentration: N/A
Calculated Conc: 1.56 umol/L
Acq. Date: 2025-05-16
Acq. Time: 02:07:29
Modified: Yes
Proc. Algorithm: Specify Parameters - MQ III

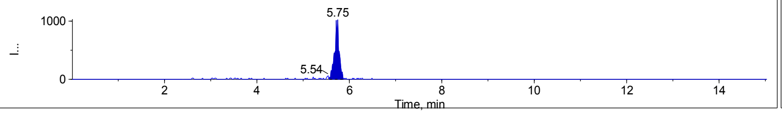

Sample Name: "ZY-20" Sample ID: "" File: "20250515-50AA.wiff"
Peak Name: "MSLAB-Nvl(IS)" Mass(es): "288.150/171.010 Da"
Comment: "" Annotation: ""
Sample Index: 45
Sample Type: Unknown
Concentration: 1.00 ng/mL
Calculated Conc: N/A
Acq. Date: 2025-05-16
Acq. Time: 02:07:29
Modified: Yes
Proc. Algorithm: Specify Parameters - MQ III

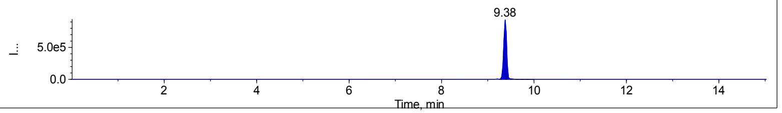

Sample Name: "ZY-20" Sample ID: "" File: "20250515-50AA.wiff"
Peak Name: "Cys1/2" Mass(es): "292.200/171.010 Da"
Comment: "" Annotation: ""
Sample Index: 45
Sample Type: Unknown
Concentration: N/A
Calculated Conc: < 0
Acq. Date: 2025-05-16
Acq. Time: 02:07:29
Modified: Yes
Proc. Algorithm: Specify Parameters - MQ III

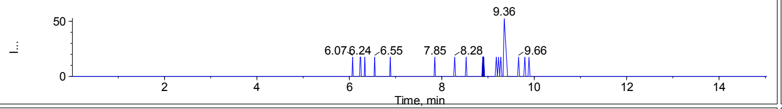

Sample Name: "ZY-20" Sample ID: "" File: "20250515-50AA.wiff"
Peak Name: "MSLAB-Nvl(IS)" Mass(es): "288.150/171.010 Da"
Comment: "" Annotation: ""
Sample Index: 45
Sample Type: Unknown
Concentration: 1.00 ng/mL
Calculated Conc: N/A
Acq. Date: 2025-05-16
Acq. Time: 02:07:29
Modified: Yes
Proc. Algorithm: Specify Parameters - MQ III

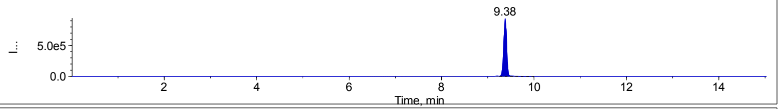

Sample Name: "ZY-20" Sample ID: "" File: "20250515-50AA.wiff"
Peak Name: "Ile" Mass(es): "302.150/171.010 Da"
Comment: "" Annotation: ""
Sample Index: 45
Sample Type: Unknown
Concentration: N/A
Calculated Conc: 0.460 umol/L
Acq. Date: 2025-05-16
Acq. Time: 02:07:29
Modified: Yes
Proc. Algorithm: Specify Parameters - MQ III

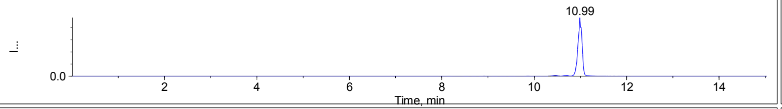

Sample Name: "ZY-20" Sample ID: "" File: "20250515-50AA.wiff"
Peak Name: "MSLAB-Nvl(IS)" Mass(es): "288.150/171.010 Da"
Comment: "" Annotation: ""
Sample Index: 45
Sample Type: Unknown
Concentration: 1.00 ng/mL
Calculated Conc: N/A
Acq. Date: 2025-05-16
Acq. Time: 02:07:29
Modified: Yes
Proc. Algorithm: Specify Parameters - MQ III

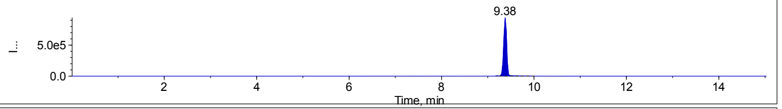

Sample Name: "ZY-20" Sample ID: "" File: "20250515-50AA.wiff"
Peak Name: "Asn" Mass(es): "303.130/171.010 Da"
Comment: "" Annotation: ""
Sample Index: 45
Sample Type: Unknown
Concentration: N/A
Calculated Conc: < 0
Acq. Date: 2025-05-16
Acq. Time: 02:07:29
Modified: Yes
Proc. Algorithm: Specify Parameters - MQ III

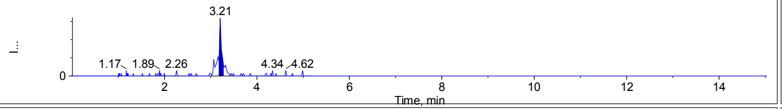

Sample Name: "ZY-20" Sample ID: "" File: "20250515-50AA.wiff"
Peak Name: "MSLAB-Nvl(IS)" Mass(es): "288.150/171.010 Da"
Comment: "" Annotation: ""
Sample Index: 45
Sample Type: Unknown
Concentration: 1.00 ng/mL
Calculated Conc: N/A
Acq. Date: 2025-05-16
Acq. Time: 02:07:29
Modified: Yes
Proc. Algorithm: Specify Parameters - MQ III

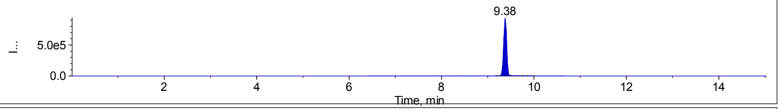

Sample Name: "ZY-20" Sample ID: "" File: "20250515-50AA.wiff"
Peak Name: "Asp" Mass(es): "304.110/171.010 Da"
Comment: "" Annotation: ""
Sample Index: 45
Sample Type: Unknown
Concentration: N/A
Calculated Conc: 0.567 umol/L
Acq. Date: 2025-05-16
Acq. Time: 02:07:29
Modified: Yes
Proc. Algorithm: Specify Parameters - MQ III

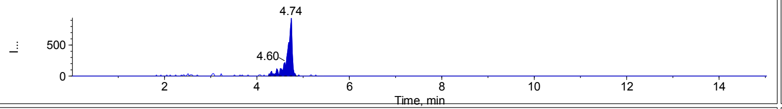

Sample Name: "ZY-20" Sample ID: "" File: "20250515-50AA.wiff"
Peak Name: "MSLAB-Nvl(IS)" Mass(es): "288.150/171.010 Da"
Comment: "" Annotation: ""
Sample Index: 45
Sample Type: Unknown
Concentration: 1.00 ng/mL
Calculated Conc: N/A
Acq. Date: 2025-05-16
Acq. Time: 02:07:29
Modified: Yes
Proc. Algorithm: Specify Parameters - MQ III

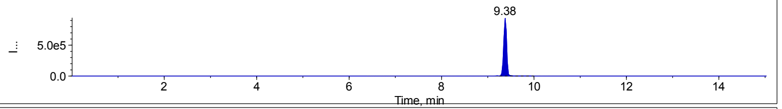

Sample Name: "ZY-20" Sample ID: "" File: "20250515-50AA.wiff"
Peak Name: "Gln" Mass(es): "317.150/171.010 Da"
Comment: "" Annotation: ""
Sample Index: 45
Sample Type: Unknown
Concentration: N/A
Calculated Conc: No Intercept
Acq. Date: 2025-05-16
Acq. Time: 02:07:29
Modified: Yes
Proc. Algorithm: Specify Parameters - MQ III

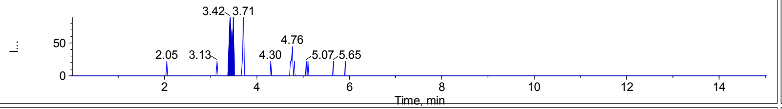

Sample Name: "ZY-20" Sample ID: "" File: "20250515-50AA.wiff"
Peak Name: "MSLAB-Nvl(IS)" Mass(es): "288.150/171.010 Da"
Comment: "" Annotation: ""
Sample Index: 45
Sample Type: Unknown
Concentration: 1.00 ng/mL
Calculated Conc: N/A
Acq. Date: 2025-05-16
Acq. Time: 02:07:29
Modified: Yes
Proc. Algorithm: Specify Parameters - MQ III

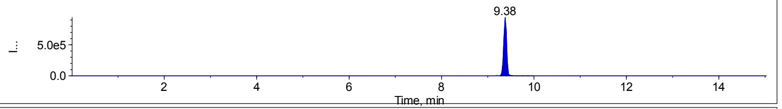

Sample Name: "ZY-20" Sample ID: "" File: "20250515-50AA.wiff"
Peak Name: "Glu" Mass(es): "318.100/171.010 Da"
Comment: "" Annotation: ""
Sample Index: 45
Sample Type: Unknown
Concentration: N/A
Calculated Conc: 2.14 umol/L
Acq. Date: 2025-05-16
Acq. Time: 02:07:29
Modified: Yes
Proc. Algorithm: Specify Parameters - MQ III

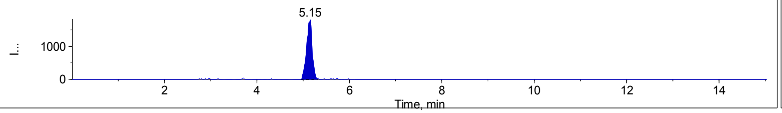

Sample Name: "ZY-20" Sample ID: "" File: "20250515-50AA.wiff"
Peak Name: "MSLAB-Nvl(IS)" Mass(es): "288.150/171.010 Da"
Comment: "" Annotation: ""
Sample Index: 45
Sample Type: Unknown
Concentration: 1.00 ng/mL
Calculated Conc: N/A
Acq. Date: 2025-05-16
Acq. Time: 02:07:29
Modified: Yes
Proc. Algorithm: Specify Parameters - MQ III

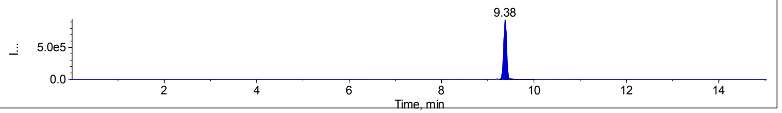

Sample Name: "ZY-20" Sample ID: "" File: "20250515-50AA.wiff"
Peak Name: "Met" Mass(es): "320.210/171.010 Da"
Comment: "" Annotation: ""

```
Sample Index:       45
Sample Type:        Unknown
Concentration:         N/A
Calculated Conc:        < 0
Acq. Date:          2025-05-16
Acq. Time:          02:07:29
Modified:              Yes
Proc. Algorithm: Specify Parameters - MQ III
```

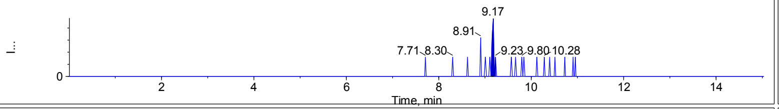

Sample Name: "ZY-20" Sample ID: "" File: "20250515-50AA.wiff"
Peak Name: "MSLAB-Nvl(IS)" Mass(es): "288.150/171.010 Da"
Comment: "" Annotation: ""

```
Sample Index:       45
Sample Type:        Unknown
Concentration:         1.00    ng/mL
Calculated Conc:       N/A
Acq. Date:          2025-05-16
Acq. Time:          02:07:29
Modified:              Yes
Proc. Algorithm: Specify Parameters - MQ III
```

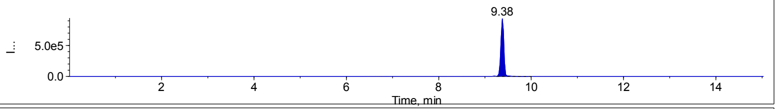

Sample Name: "ZY-20" Sample ID: "" File: "20250515-50AA.wiff"
Peak Name: "His" Mass(es): "326.150/171.010 Da"
Comment: "" Annotation: ""

```
Sample Index:       45
Sample Type:        Unknown
Concentration:         N/A
Calculated Conc:       1.18    umol/L
Acq. Date:          2025-05-16
Acq. Time:          02:07:29
Modified:              Yes
Proc. Algorithm: Specify Parameters - MQ III
```

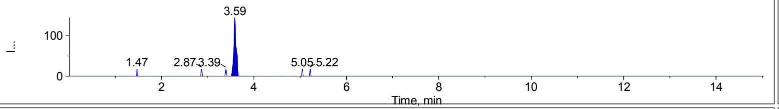

Sample Name: "ZY-20" Sample ID: "" File: "20250515-50AA.wiff"
Peak Name: "MSLAB-Nvl(IS)" Mass(es): "288.150/171.010 Da"
Comment: "" Annotation: ""

```
Sample Index:       45
Sample Type:        Unknown
Concentration:         1.00    ng/mL
Calculated Conc:       N/A
Acq. Date:          2025-05-16
Acq. Time:          02:07:29
Modified:              Yes
Proc. Algorithm: Specify Parameters - MQ III
```

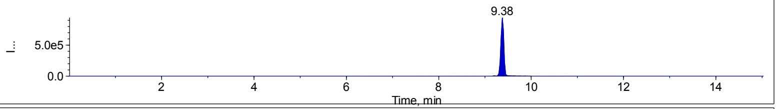

Sample Name: "ZY-20" Sample ID: "" File: "20250515-50AA.wiff"
Peak Name: "Phe" Mass(es): "336.200/171.010 Da"
Comment: "" Annotation: ""

```
Sample Index:       45
Sample Type:        Unknown
Concentration:         N/A
Calculated Conc:       0.700   umol/L
Acq. Date:          2025-05-16
Acq. Time:          02:07:29
Modified:              Yes
Proc. Algorithm: Specify Parameters - MQ III
```

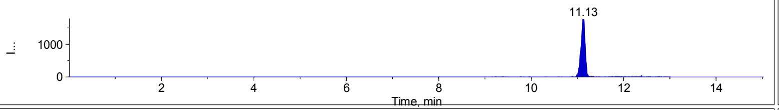

Sample Name: "ZY-20" Sample ID: "" File: "20250515-50AA.wiff"
Peak Name: "MSLAB-Nvl(IS)" Mass(es): "288.150/171.010 Da"
Comment: "" Annotation: ""

```
Sample Index:       45
Sample Type:        Unknown
Concentration:         1.00    ng/mL
Calculated Conc:       N/A
Acq. Date:          2025-05-16
Acq. Time:          02:07:29
Modified:              Yes
Proc. Algorithm: Specify Parameters - MQ III
```

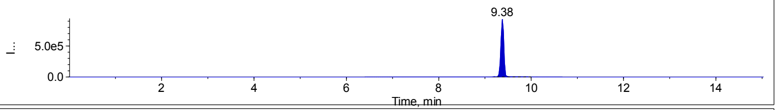

Sample Name: "ZY-20" Sample ID: "" File: "20250515-50AA.wiff"
Peak Name: "Arg" Mass(es): "345.210/171.010 Da"
Comment: "" Annotation: ""

```
Sample Index:       45
Sample Type:        Unknown
Concentration:         N/A
Calculated Conc:       2.32    umol/L
Acq. Date:          2025-05-16
Acq. Time:          02:07:29
Modified:              Yes
Proc. Algorithm: Specify Parameters - MQ III
```

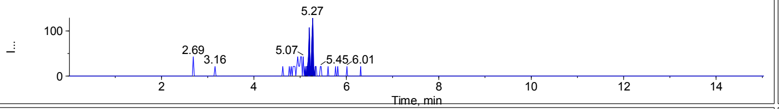

Sample Name: "ZY-20" Sample ID: "" File: "20250515-50AA.wiff"
Peak Name: "MSLAB-Nvl(IS)" Mass(es): "288.150/171.010 Da"
Comment: "" Annotation: ""

```
Sample Index:       45
Sample Type:        Unknown
Concentration:         1.00    ng/mL
Calculated Conc:       N/A
Acq. Date:          2025-05-16
Acq. Time:          02:07:29
Modified:              Yes
Proc. Algorithm: Specify Parameters - MQ III
```

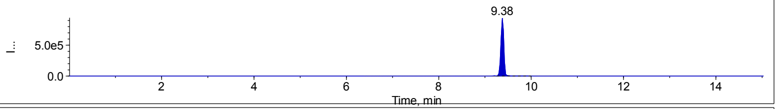

Sample Name: "ZY-20" Sample ID: "" File: "20250515-50AA.wiff"
Peak Name: "Trp" Mass(es): "375.200/171.010 Da"
Comment: "" Annotation: ""

```
Sample Index:       45
Sample Type:        Unknown
Concentration:         N/A
Calculated Conc:        < 0
Acq. Date:          2025-05-16
Acq. Time:          02:07:29
Modified:              Yes
Proc. Algorithm: Specify Parameters - MQ III
```

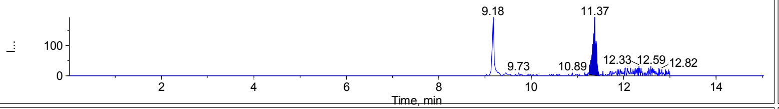

Sample Name: "ZY-20" Sample ID: "" File: "20250515-50AA.wiff"
Peak Name: "MSLAB-Nvl(IS)" Mass(es): "288.150/171.010 Da"
Comment: "" Annotation: ""

```
Sample Index:       45
Sample Type:        Unknown
Concentration:         1.00    ng/mL
Calculated Conc:       N/A
Acq. Date:          2025-05-16
Acq. Time:          02:07:29
Modified:              Yes
Proc. Algorithm: Specify Parameters - MQ III
```

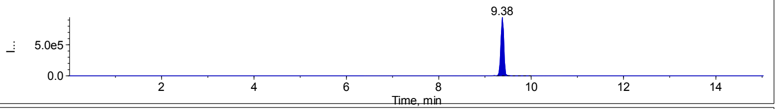

Sample Name: "ZY-20" Sample ID: "" File: "20250515-50AA.wiff"
Peak Name: "Lys" Mass(es): "487.200/171.010 Da"
Comment: "" Annotation: ""

```
Sample Index:       45
Sample Type:        Unknown
Concentration:         N/A
Calculated Conc:       1.90    umol/L
Acq. Date:          2025-05-16
Acq. Time:          02:07:29
Modified:              Yes
Proc. Algorithm: Specify Parameters - MQ III
```

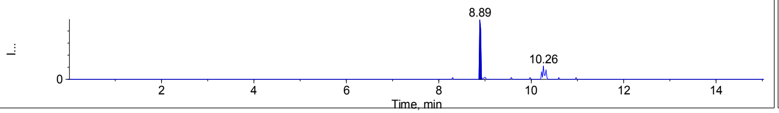

Sample Name: "ZY-20" Sample ID: "" File: "20250515-50AA.wiff"
Peak Name: "MSLAB-Nvl(IS)" Mass(es): "288.150/171.010 Da"
Comment: "" Annotation: ""

```
Sample Index:       45
Sample Type:        Unknown
Concentration:         1.00    ng/mL
Calculated Conc:       N/A
Acq. Date:          2025-05-16
Acq. Time:          02:07:29
Modified:              Yes
Proc. Algorithm: Specify Parameters - MQ III
```

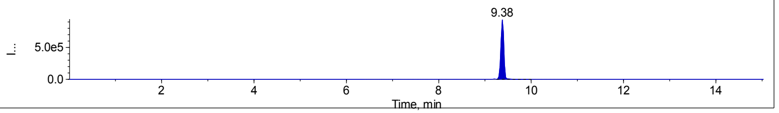

Sample Name: "ZY-20" Sample ID: "" File: "20250515-50AA.wiff"
Peak Name: "tyr" Mass(es): "352.200/171.010 Da"
Comment: "" Annotation: ""
Sample Index: 45
Sample Type: Unknown
Concentration: N/A
Calculated Conc: < 0
Acq. Date: 2025-05-16
Acq. Time: 02:07:29
Modified: Yes
Proc. Algorithm: Specify Parameters - MQ III

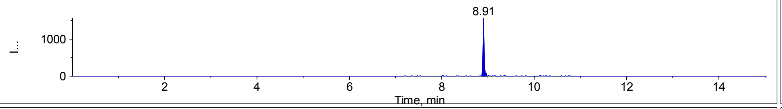

Sample Name: "ZY-20" Sample ID: "" File: "20250515-50AA.wiff"
Peak Name: "MSLAB-Nvl(IS)" Mass(es): "288.150/171.010 Da"
Comment: "" Annotation: ""
Sample Index: 45
Sample Type: Unknown
Concentration: 1.00 ng/mL
Calculated Conc: N/A
Acq. Date: 2025-05-16
Acq. Time: 02:07:29
Modified: Yes
Proc. Algorithm: Specify Parameters - MQ III

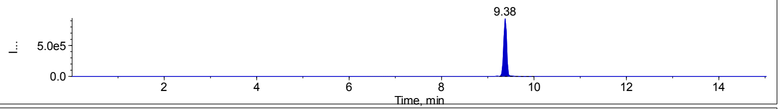

Sample Name: "ZY-20" Sample ID: "" File: "20250515-50AA.wiff"
Peak Name: "Leu" Mass(es): "302.150/171.010 Da"
Comment: "" Annotation: ""
Sample Index: 45
Sample Type: Unknown
Concentration: N/A
Calculated Conc: 1.32 umol/L
Acq. Date: 2025-05-16
Acq. Time: 02:07:29
Modified: Yes
Proc. Algorithm: Specify Parameters - MQ III

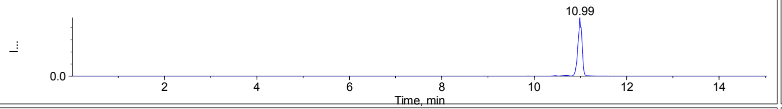

Sample Name: "ZY-20" Sample ID: "" File: "20250515-50AA.wiff"
Peak Name: "MSLAB-Nvl(IS)" Mass(es): "288.150/171.010 Da"
Comment: "" Annotation: ""
Sample Index: 45
Sample Type: Unknown
Concentration: 1.00 ng/mL
Calculated Conc: N/A
Acq. Date: 2025-05-16
Acq. Time: 02:07:29
Modified: Yes
Proc. Algorithm: Specify Parameters - MQ III

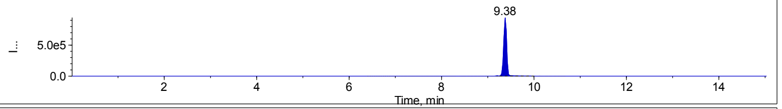

Sample Name: "ZY-21" Sample ID: "" File: "20250515-50AA.wiff"
Peak Name: "Gly" Mass(es): "246.080/171.010 Da"
Comment: "" Annotation: ""
Sample Index: 46
Sample Type: Unknown
Concentration: N/A
Calculated Conc: 0.290 umol/L
Acq. Date: 2025-05-16
Acq. Time: 02:22:57
Modified: Yes
Proc. Algorithm: Specify Parameters - MQ III

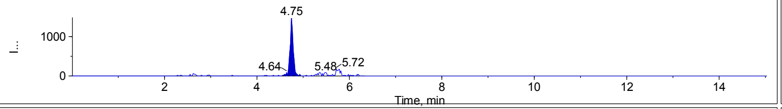

Sample Name: "ZY-21" Sample ID: "" File: "20250515-50AA.wiff"
Peak Name: "MSLAB-Nvl(IS)" Mass(es): "288.150/171.010 Da"
Comment: "" Annotation: ""
Sample Index: 46
Sample Type: Unknown
Concentration: 1.00 ng/mL
Calculated Conc: N/A
Acq. Date: 2025-05-16
Acq. Time: 02:22:57
Modified: Yes
Proc. Algorithm: Specify Parameters - MQ III

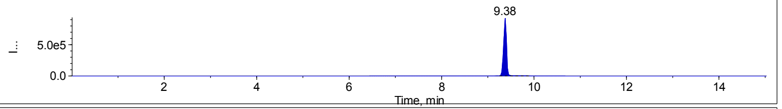

Sample Name: "ZY-21" Sample ID: "" File: "20250515-50AA.wiff"
Peak Name: "Ala" Mass(es): "260.100/171.010 Da"
Comment: "" Annotation: ""
Sample Index: 46
Sample Type: Unknown
Concentration: N/A
Calculated Conc: No Intercept
Acq. Date: 2025-05-16
Acq. Time: 02:22:57
Modified: Yes
Proc. Algorithm: Specify Parameters - MQ III

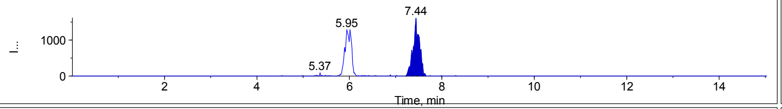

Sample Name: "ZY-21" Sample ID: "" File: "20250515-50AA.wiff"
Peak Name: "MSLAB-Nvl(IS)" Mass(es): "288.150/171.010 Da"
Comment: "" Annotation: ""
Sample Index: 46
Sample Type: Unknown
Concentration: 1.00 ng/mL
Calculated Conc: N/A
Acq. Date: 2025-05-16
Acq. Time: 02:22:57
Modified: Yes
Proc. Algorithm: Specify Parameters - MQ III

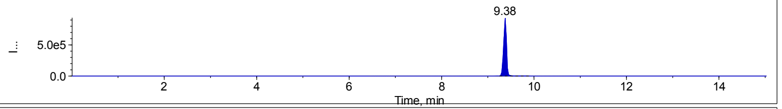

Sample Name: "ZY-21" Sample ID: "" File: "20250515-50AA.wiff"
Peak Name: "Ser" Mass(es): "276.100/171.010 Da"
Comment: "" Annotation: ""
Sample Index: 46
Sample Type: Unknown
Concentration: N/A
Calculated Conc: < 0
Acq. Date: 2025-05-16
Acq. Time: 02:22:57
Modified: Yes
Proc. Algorithm: Specify Parameters - MQ III

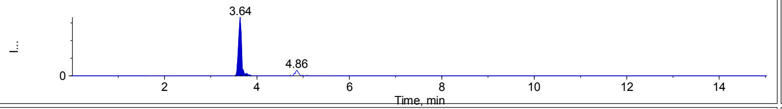

Sample Name: "ZY-21" Sample ID: "" File: "20250515-50AA.wiff"
Peak Name: "MSLAB-Nvl(IS)" Mass(es): "288.150/171.010 Da"
Comment: "" Annotation: ""
Sample Index: 46
Sample Type: Unknown
Concentration: 1.00 ng/mL
Calculated Conc: N/A
Acq. Date: 2025-05-16
Acq. Time: 02:22:57
Modified: Yes
Proc. Algorithm: Specify Parameters - MQ III

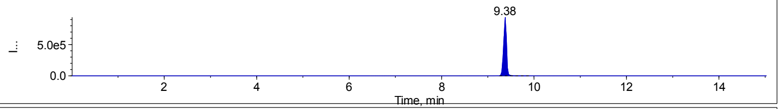

Sample Name: "ZY-21" Sample ID: "" File: "20250515-50AA.wiff"
Peak Name: "Pro" Mass(es): "286.150/171.010 Da"
Comment: "" Annotation: ""
Sample Index: 46
Sample Type: Unknown
Concentration: N/A
Calculated Conc: No Intercept
Acq. Date: 2025-05-16
Acq. Time: 02:22:57
Modified: Yes
Proc. Algorithm: Specify Parameters - MQ III

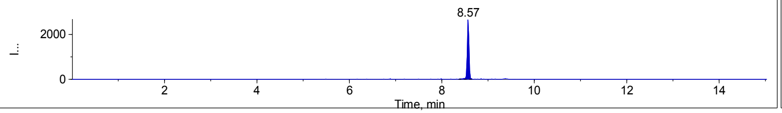

Sample Name: "ZY-21" Sample ID: "" File: "20250515-50AA.wiff"
Peak Name: "MSLAB-Nvl(IS)" Mass(es): "288.150/171.010 Da"
Comment: "" Annotation: ""
Sample Index: 46
Sample Type: Unknown
Concentration: 1.00 ng/mL
Calculated Conc: N/A
Acq. Date: 2025-05-16
Acq. Time: 02:22:57
Modified: Yes
Proc. Algorithm: Specify Parameters - MQ III

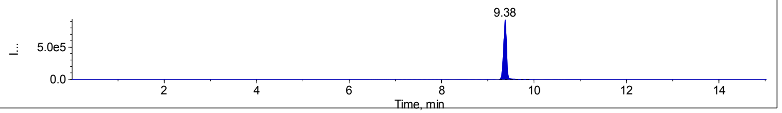

Sample Name: "ZY-21" Sample ID: "" File: "20250515-50AA.wiff"
Peak Name: "Val" Mass(es): "288.150/171.010 Da"
Comment: "" Annotation: ""

Sample Index: 46
Sample Type: Unknown
Concentration: N/A
Calculated Conc: 1.16 umol/L
Acq. Date: 2025-05-16
Acq. Time: 02:22:57

Modified: Yes
Proc. Algorithm: Specify Parameters - MQ III

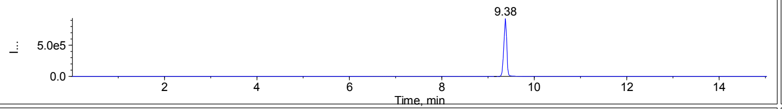

Sample Name: "ZY-21" Sample ID: "" File: "20250515-50AA.wiff"
Peak Name: "MSLAB-Nvl(IS)" Mass(es): "288.150/171.010 Da"
Comment: "" Annotation: ""

Sample Index: 46
Sample Type: Unknown
Concentration: 1.00 ng/mL
Calculated Conc: N/A
Acq. Date: 2025-05-16
Acq. Time: 02:22:57

Modified: Yes
Proc. Algorithm: Specify Parameters - MQ III

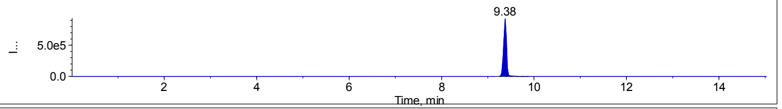

Sample Name: "ZY-21" Sample ID: "" File: "20250515-50AA.wiff"
Peak Name: "Thr" Mass(es): "290.100/171.010 Da"
Comment: "" Annotation: ""

Sample Index: 46
Sample Type: Unknown
Concentration: N/A
Calculated Conc: 0.850 umol/L
Acq. Date: 2025-05-16
Acq. Time: 02:22:57

Modified: Yes
Proc. Algorithm: Specify Parameters - MQ III

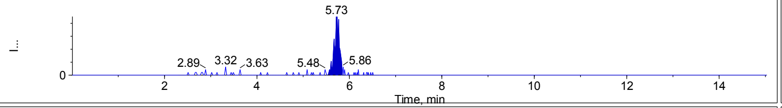

Sample Name: "ZY-21" Sample ID: "" File: "20250515-50AA.wiff"
Peak Name: "MSLAB-Nvl(IS)" Mass(es): "288.150/171.010 Da"
Comment: "" Annotation: ""

Sample Index: 46
Sample Type: Unknown
Concentration: 1.00 ng/mL
Calculated Conc: N/A
Acq. Date: 2025-05-16
Acq. Time: 02:22:57

Modified: Yes
Proc. Algorithm: Specify Parameters - MQ III

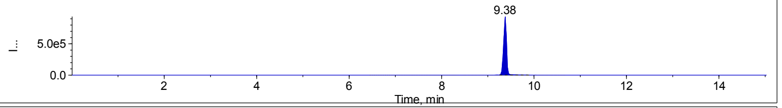

Sample Name: "ZY-21" Sample ID: "" File: "20250515-50AA.wiff"
Peak Name: "Cys1/2" Mass(es): "292.200/171.010 Da"
Comment: "" Annotation: ""

Sample Index: 46
Sample Type: Unknown
Concentration: N/A
Calculated Conc: < 0
Acq. Date: 2025-05-16
Acq. Time: 02:22:57

Modified: Yes
Proc. Algorithm: Specify Parameters - MQ III

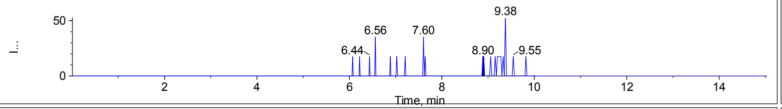

Sample Name: "ZY-21" Sample ID: "" File: "20250515-50AA.wiff"
Peak Name: "MSLAB-Nvl(IS)" Mass(es): "288.150/171.010 Da"
Comment: "" Annotation: ""

Sample Index: 46
Sample Type: Unknown
Concentration: 1.00 ng/mL
Calculated Conc: N/A
Acq. Date: 2025-05-16
Acq. Time: 02:22:57

Modified: Yes
Proc. Algorithm: Specify Parameters - MQ III

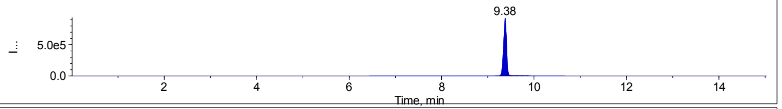

Sample Name: "ZY-21" Sample ID: "" File: "20250515-50AA.wiff"
Peak Name: "Ile" Mass(es): "302.150/171.010 Da"
Comment: "" Annotation: ""

Sample Index: 46
Sample Type: Unknown
Concentration: N/A
Calculated Conc: < 0
Acq. Date: 2025-05-16
Acq. Time: 02:22:57

Modified: Yes
Proc. Algorithm: Specify Parameters - MQ III

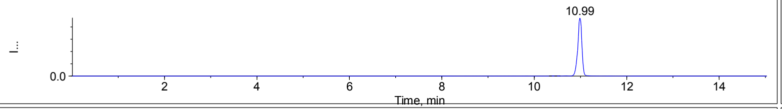

Sample Name: "ZY-21" Sample ID: "" File: "20250515-50AA.wiff"
Peak Name: "MSLAB-Nvl(IS)" Mass(es): "288.150/171.010 Da"
Comment: "" Annotation: ""

Sample Index: 46
Sample Type: Unknown
Concentration: 1.00 ng/mL
Calculated Conc: N/A
Acq. Date: 2025-05-16
Acq. Time: 02:22:57

Modified: Yes
Proc. Algorithm: Specify Parameters - MQ III

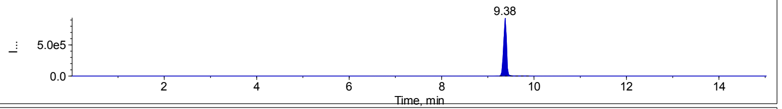

Sample Name: "ZY-21" Sample ID: "" File: "20250515-50AA.wiff"
Peak Name: "Asn" Mass(es): "303.130/171.010 Da"
Comment: "" Annotation: ""

Sample Index: 46
Sample Type: Unknown
Concentration: N/A
Calculated Conc: < 0
Acq. Date: 2025-05-16
Acq. Time: 02:22:57

Modified: Yes
Proc. Algorithm: Specify Parameters - MQ III

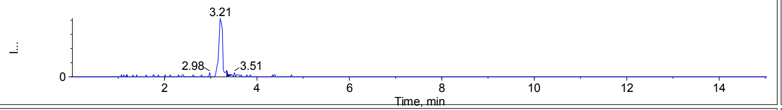

Sample Name: "ZY-21" Sample ID: "" File: "20250515-50AA.wiff"
Peak Name: "MSLAB-Nvl(IS)" Mass(es): "288.150/171.010 Da"
Comment: "" Annotation: ""

Sample Index: 46
Sample Type: Unknown
Concentration: 1.00 ng/mL
Calculated Conc: N/A
Acq. Date: 2025-05-16
Acq. Time: 02:22:57

Modified: Yes
Proc. Algorithm: Specify Parameters - MQ III

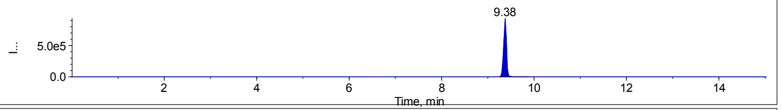

Sample Name: "ZY-21" Sample ID: "" File: "20250515-50AA.wiff"
Peak Name: "Asp" Mass(es): "304.110/171.010 Da"
Comment: "" Annotation: ""

Sample Index: 46
Sample Type: Unknown
Concentration: N/A
Calculated Conc: No Intercept
Acq. Date: 2025-05-16
Acq. Time: 02:22:57

Modified: Yes
Proc. Algorithm: Specify Parameters - MQ III

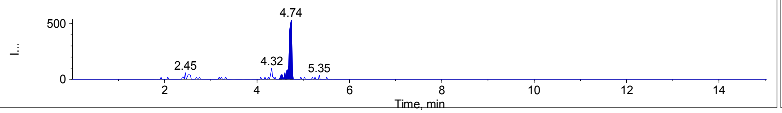

Sample Name: "ZY-21" Sample ID: "" File: "20250515-50AA.wiff"
Peak Name: "MSLAB-Nvl(IS)" Mass(es): "288.150/171.010 Da"
Comment: "" Annotation: ""

Sample Index: 46
Sample Type: Unknown
Concentration: 1.00 ng/mL
Calculated Conc: N/A
Acq. Date: 2025-05-16
Acq. Time: 02:22:57

Modified: Yes
Proc. Algorithm: Specify Parameters - MQ III

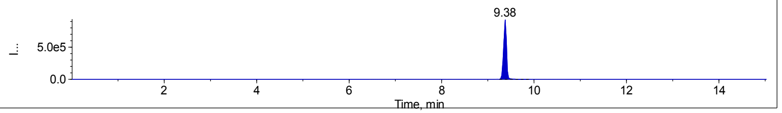

Sample Name: "ZY-21" Sample ID: "" File: "20250515-50AA.wiff"
Peak Name: "Gln" Mass(es): "317.150/171.010 Da"
Comment: "" Annotation: ""

```
Sample Index:      46
Sample Type:       Unknown
Concentration:       N/A
Calculated Conc:      < 0
Acq. Date:         2025-05-16
Acq. Time:         02:22:57

Modified:           Yes
Proc. Algorithm: Specify Parameters - MQ III
```

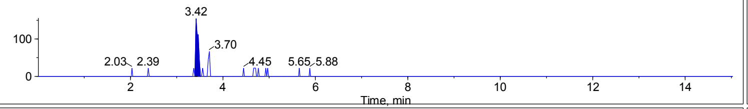

Sample Name: "ZY-21" Sample ID: "" File: "20250515-50AA.wiff"
Peak Name: "MSLAB-Nvl(IS)" Mass(es): "288.150/171.010 Da"
Comment: "" Annotation: ""

```
Sample Index:      46
Sample Type:       Unknown
Concentration:     1.00    ng/mL
Calculated Conc:   N/A
Acq. Date:         2025-05-16
Acq. Time:         02:22:57

Modified:          Yes
Proc. Algorithm: Specify Parameters - MQ III
```

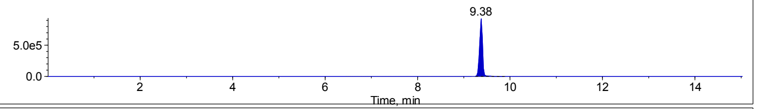

Sample Name: "ZY-21" Sample ID: "" File: "20250515-50AA.wiff"
Peak Name: "Glu" Mass(es): "318.100/171.010 Da"
Comment: "" Annotation: ""

```
Sample Index:      46
Sample Type:       Unknown
Concentration:       N/A
Calculated Conc:   0.845   umol/L
Acq. Date:         2025-05-16
Acq. Time:         02:22:57

Modified:           Yes
Proc. Algorithm: Specify Parameters - MQ III
```

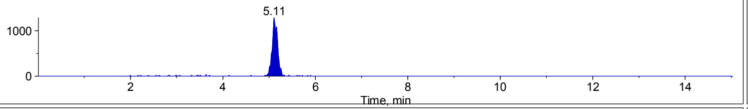

Sample Name: "ZY-21" Sample ID: "" File: "20250515-50AA.wiff"
Peak Name: "MSLAB-Nvl(IS)" Mass(es): "288.150/171.010 Da"
Comment: "" Annotation: ""

```
Sample Index:      46
Sample Type:       Unknown
Concentration:     1.00    ng/mL
Calculated Conc:   N/A
Acq. Date:         2025-05-16
Acq. Time:         02:22:57

Modified:          Yes
Proc. Algorithm: Specify Parameters - MQ III
```

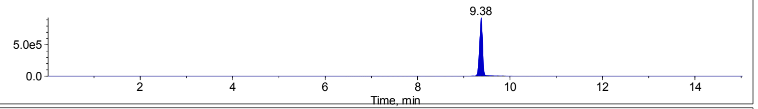

Sample Name: "ZY-21" Sample ID: "" File: "20250515-50AA.wiff"
Peak Name: "Met" Mass(es): "320.210/171.010 Da"
Comment: "" Annotation: ""

```
Sample Index:      46
Sample Type:       Unknown
Concentration:       N/A
Calculated Conc:      < 0
Acq. Date:         2025-05-16
Acq. Time:         02:22:57

Modified:           Yes
Proc. Algorithm: Specify Parameters - MQ III
```

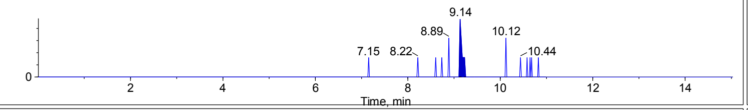

Sample Name: "ZY-21" Sample ID: "" File: "20250515-50AA.wiff"
Peak Name: "MSLAB-Nvl(IS)" Mass(es): "288.150/171.010 Da"
Comment: "" Annotation: ""

```
Sample Index:      46
Sample Type:       Unknown
Concentration:     1.00    ng/mL
Calculated Conc:   N/A
Acq. Date:         2025-05-16
Acq. Time:         02:22:57

Modified:          Yes
Proc. Algorithm: Specify Parameters - MQ III
```

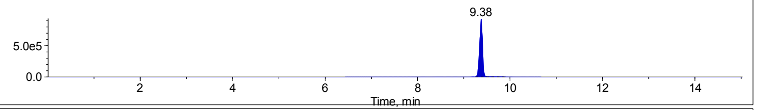

Sample Name: "ZY-21" Sample ID: "" File: "20250515-50AA.wiff"
Peak Name: "His" Mass(es): "326.150/171.010 Da"
Comment: "" Annotation: ""

```
Sample Index:      46
Sample Type:       Unknown
Concentration:       N/A
Calculated Conc:   1.04    umol/L
Acq. Date:         2025-05-16
Acq. Time:         02:22:57

Modified:           Yes
Proc. Algorithm: Specify Parameters - MQ III
```

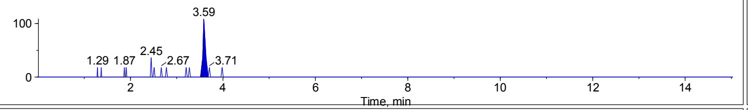

Sample Name: "ZY-21" Sample ID: "" File: "20250515-50AA.wiff"
Peak Name: "MSLAB-Nvl(IS)" Mass(es): "288.150/171.010 Da"
Comment: "" Annotation: ""

```
Sample Index:      46
Sample Type:       Unknown
Concentration:     1.00    ng/mL
Calculated Conc:   N/A
Acq. Date:         2025-05-16
Acq. Time:         02:22:57

Modified:          Yes
Proc. Algorithm: Specify Parameters - MQ III
```

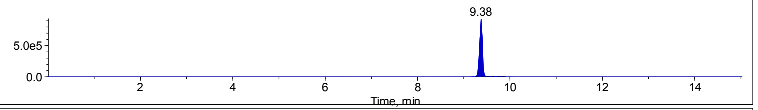

Sample Name: "ZY-21" Sample ID: "" File: "20250515-50AA.wiff"
Peak Name: "Phe" Mass(es): "336.200/171.010 Da"
Comment: "" Annotation: ""

```
Sample Index:      46
Sample Type:       Unknown
Concentration:       N/A
Calculated Conc:   0.0585  umol/L
Acq. Date:         2025-05-16
Acq. Time:         02:22:57

Modified:           Yes
Proc. Algorithm: Specify Parameters - MQ III
```

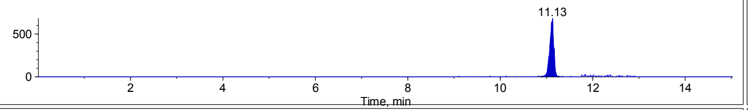

Sample Name: "ZY-21" Sample ID: "" File: "20250515-50AA.wiff"
Peak Name: "MSLAB-Nvl(IS)" Mass(es): "288.150/171.010 Da"
Comment: "" Annotation: ""

```
Sample Index:      46
Sample Type:       Unknown
Concentration:     1.00    ng/mL
Calculated Conc:   N/A
Acq. Date:         2025-05-16
Acq. Time:         02:22:57

Modified:          Yes
Proc. Algorithm: Specify Parameters - MQ III
```

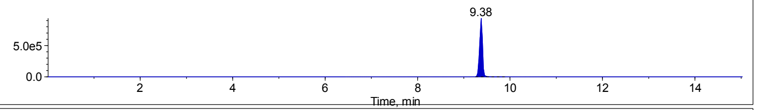

Sample Name: "ZY-21" Sample ID: "" File: "20250515-50AA.wiff"
Peak Name: "Arg" Mass(es): "345.210/171.010 Da"
Comment: "" Annotation: ""

```
Sample Index:      46
Sample Type:       Unknown
Concentration:       N/A
Calculated Conc:   1.66    umol/L
Acq. Date:         2025-05-16
Acq. Time:         02:22:57

Modified:           Yes
Proc. Algorithm: Specify Parameters - MQ III
```

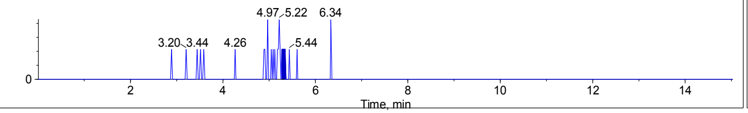

Sample Name: "ZY-21" Sample ID: "" File: "20250515-50AA.wiff"
Peak Name: "MSLAB-Nvl(IS)" Mass(es): "288.150/171.010 Da"
Comment: "" Annotation: ""

```
Sample Index:      46
Sample Type:       Unknown
Concentration:     1.00    ng/mL
Calculated Conc:   N/A
Acq. Date:         2025-05-16
Acq. Time:         02:22:57

Modified:          Yes
Proc. Algorithm: Specify Parameters - MQ III
```

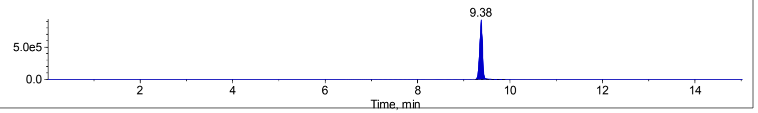

Sample Name: "ZY-21"   Sample ID: ""   File: "20250515-50AA.wiff"
Peak Name: "Trp"   Mass(es): "375.200/171.010 Da"
Comment: ""   Annotation: ""

Sample Index: 46
Sample Type: Unknown
Concentration: N/A
Calculated Conc: < 0
Acq. Date: 2025-05-16
Acq. Time: 02:22:57
Modified: Yes
Proc. Algorithm: Specify Parameters - MQ III

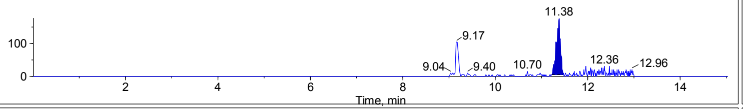

Sample Name: "ZY-21"   Sample ID: ""   File: "20250515-50AA.wiff"
Peak Name: "MSLAB-Nvl(IS)"   Mass(es): "288.150/171.010 Da"
Comment: ""   Annotation: ""

Sample Index: 46
Sample Type: Unknown
Concentration: 1.00 ng/mL
Calculated Conc: N/A
Acq. Date: 2025-05-16
Acq. Time: 02:22:57
Modified: Yes
Proc. Algorithm: Specify Parameters - MQ III

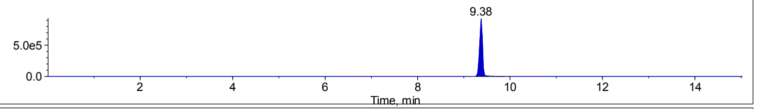

Sample Name: "ZY-21"   Sample ID: ""   File: "20250515-50AA.wiff"
Peak Name: "Lys"   Mass(es): "487.200/171.010 Da"
Comment: ""   Annotation: ""

Sample Index: 46
Sample Type: Unknown
Concentration: N/A
Calculated Conc: 0.818 umol/L
Acq. Date: 2025-05-16
Acq. Time: 02:22:57
Modified: Yes
Proc. Algorithm: Specify Parameters - MQ III

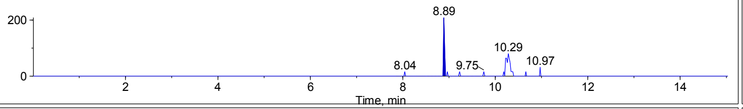

Sample Name: "ZY-21"   Sample ID: ""   File: "20250515-50AA.wiff"
Peak Name: "MSLAB-Nvl(IS)"   Mass(es): "288.150/171.010 Da"
Comment: ""   Annotation: ""

Sample Index: 46
Sample Type: Unknown
Concentration: 1.00 ng/mL
Calculated Conc: N/A
Acq. Date: 2025-05-16
Acq. Time: 02:22:57
Modified: Yes
Proc. Algorithm: Specify Parameters - MQ III

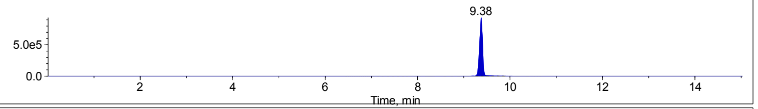

Sample Name: "ZY-21"   Sample ID: ""   File: "20250515-50AA.wiff"
Peak Name: "tyr"   Mass(es): "352.200/171.010 Da"
Comment: ""   Annotation: ""

Sample Index: 46
Sample Type: Unknown
Concentration: N/A
Calculated Conc: No Intercept
Acq. Date: 2025-05-16
Acq. Time: 02:22:57
Modified: Yes
Proc. Algorithm: Specify Parameters - MQ III

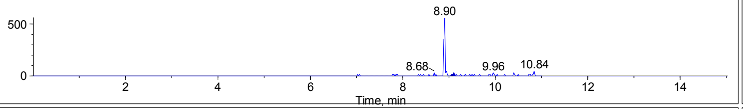

Sample Name: "ZY-21"   Sample ID: ""   File: "20250515-50AA.wiff"
Peak Name: "MSLAB-Nvl(IS)"   Mass(es): "288.150/171.010 Da"
Comment: ""   Annotation: ""

Sample Index: 46
Sample Type: Unknown
Concentration: 1.00 ng/mL
Calculated Conc: N/A
Acq. Date: 2025-05-16
Acq. Time: 02:22:57
Modified: Yes
Proc. Algorithm: Specify Parameters - MQ III

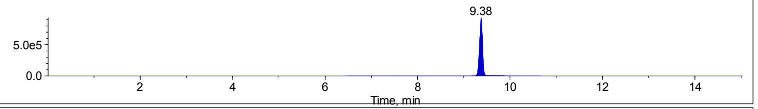

Sample Name: "ZY-21"   Sample ID: ""   File: "20250515-50AA.wiff"
Peak Name: "Leu"   Mass(es): "302.150/171.010 Da"
Comment: ""   Annotation: ""

Sample Index: 46
Sample Type: Unknown
Concentration: N/A
Calculated Conc: < 0
Acq. Date: 2025-05-16
Acq. Time: 02:22:57
Modified: Yes
Proc. Algorithm: Specify Parameters - MQ III

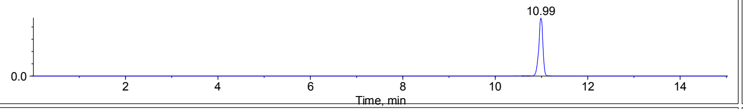

Sample Name: "ZY-21"   Sample ID: ""   File: "20250515-50AA.wiff"
Peak Name: "MSLAB-Nvl(IS)"   Mass(es): "288.150/171.010 Da"
Comment: ""   Annotation: ""

Sample Index: 46
Sample Type: Unknown
Concentration: 1.00 ng/mL
Calculated Conc: N/A
Acq. Date: 2025-05-16
Acq. Time: 02:22:57
Modified: Yes
Proc. Algorithm: Specify Parameters - MQ III

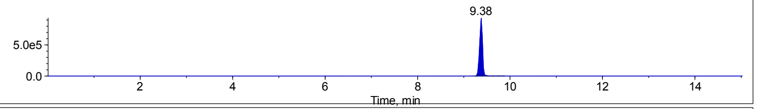

Sample Name: "ZY-22"   Sample ID: ""   File: "20250515-50AA.wiff"
Peak Name: "Gly"   Mass(es): "246.080/171.010 Da"
Comment: ""   Annotation: ""

Sample Index: 47
Sample Type: Unknown
Concentration: N/A
Calculated Conc: 5.68 umol/L
Acq. Date: 2025-05-16
Acq. Time: 02:38:22
Modified: Yes
Proc. Algorithm: Specify Parameters - MQ III

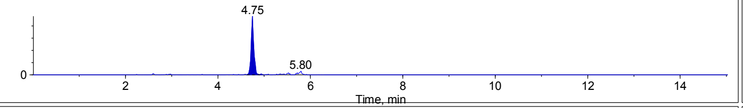

Sample Name: "ZY-22"   Sample ID: ""   File: "20250515-50AA.wiff"
Peak Name: "MSLAB-Nvl(IS)"   Mass(es): "288.150/171.010 Da"
Comment: ""   Annotation: ""

Sample Index: 47
Sample Type: Unknown
Concentration: 1.00 ng/mL
Calculated Conc: N/A
Acq. Date: 2025-05-16
Acq. Time: 02:38:22
Modified: Yes
Proc. Algorithm: Specify Parameters - MQ III

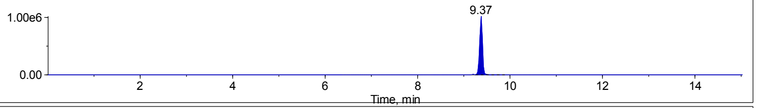

Sample Name: "ZY-22"   Sample ID: ""   File: "20250515-50AA.wiff"
Peak Name: "Ala"   Mass(es): "260.100/171.010 Da"
Comment: ""   Annotation: ""

Sample Index: 47
Sample Type: Unknown
Concentration: N/A
Calculated Conc: No Intercept
Acq. Date: 2025-05-16
Acq. Time: 02:38:22
Modified: Yes
Proc. Algorithm: Specify Parameters - MQ III

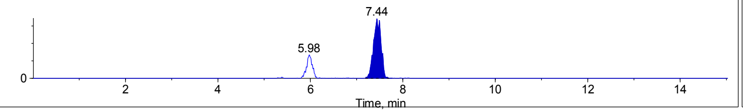

Sample Name: "ZY-22"   Sample ID: ""   File: "20250515-50AA.wiff"
Peak Name: "MSLAB-Nvl(IS)"   Mass(es): "288.150/171.010 Da"
Comment: ""   Annotation: ""

Sample Index: 47
Sample Type: Unknown
Concentration: 1.00 ng/mL
Calculated Conc: N/A
Acq. Date: 2025-05-16
Acq. Time: 02:38:22
Modified: Yes
Proc. Algorithm: Specify Parameters - MQ III

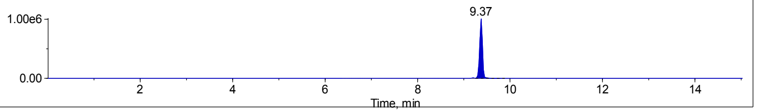

Sample Name: "ZY-22" Sample ID: "" File: "20250515-50AA.wiff"
Peak Name: "Ser" Mass(es): "276.100/171.010 Da"
Comment: "" Annotation: ""
Sample Index: 47
Sample Type: Unknown
Concentration: N/A
Calculated Conc: 2.51 umol/L
Acq. Date: 2025-05-16
Acq. Time: 02:38:22
Modified: Yes
Proc. Algorithm: Specify Parameters - MQ III

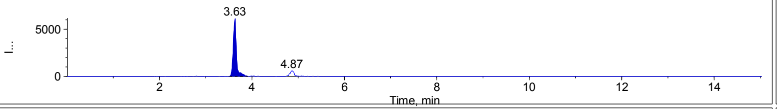

Sample Name: "ZY-22" Sample ID: "" File: "20250515-50AA.wiff"
Peak Name: "MSLAB-Nvl(IS)" Mass(es): "288.150/171.010 Da"
Comment: "" Annotation: ""
Sample Index: 47
Sample Type: Unknown
Concentration: 1.00 ng/mL
Calculated Conc: N/A
Acq. Date: 2025-05-16
Acq. Time: 02:38:22
Modified: Yes
Proc. Algorithm: Specify Parameters - MQ III

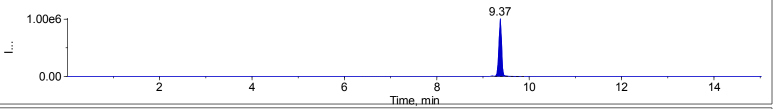

Sample Name: "ZY-22" Sample ID: "" File: "20250515-50AA.wiff"
Peak Name: "Pro" Mass(es): "286.150/171.010 Da"
Comment: "" Annotation: ""
Sample Index: 47
Sample Type: Unknown
Concentration: N/A
Calculated Conc: No Intercept
Acq. Date: 2025-05-16
Acq. Time: 02:38:22
Modified: Yes
Proc. Algorithm: Specify Parameters - MQ III

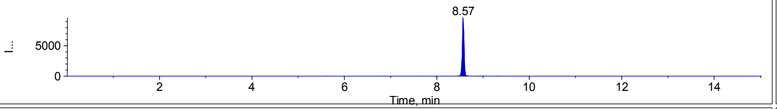

Sample Name: "ZY-22" Sample ID: "" File: "20250515-50AA.wiff"
Peak Name: "MSLAB-Nvl(IS)" Mass(es): "288.150/171.010 Da"
Comment: "" Annotation: ""
Sample Index: 47
Sample Type: Unknown
Concentration: 1.00 ng/mL
Calculated Conc: N/A
Acq. Date: 2025-05-16
Acq. Time: 02:38:22
Modified: Yes
Proc. Algorithm: Specify Parameters - MQ III

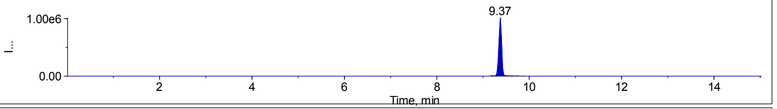

Sample Name: "ZY-22" Sample ID: "" File: "20250515-50AA.wiff"
Peak Name: "Val" Mass(es): "288.150/171.010 Da"
Comment: "" Annotation: ""
Sample Index: 47
Sample Type: Unknown
Concentration: N/A
Calculated Conc: 3.29 umol/L
Acq. Date: 2025-05-16
Acq. Time: 02:38:22
Modified: Yes
Proc. Algorithm: Specify Parameters - MQ III

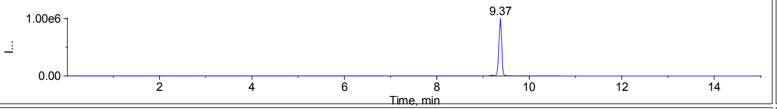

Sample Name: "ZY-22" Sample ID: "" File: "20250515-50AA.wiff"
Peak Name: "MSLAB-Nvl(IS)" Mass(es): "288.150/171.010 Da"
Comment: "" Annotation: ""
Sample Index: 47
Sample Type: Unknown
Concentration: 1.00 ng/mL
Calculated Conc: N/A
Acq. Date: 2025-05-16
Acq. Time: 02:38:22
Modified: Yes
Proc. Algorithm: Specify Parameters - MQ III

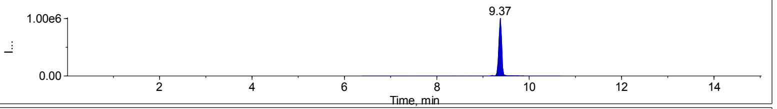

Sample Name: "ZY-22" Sample ID: "" File: "20250515-50AA.wiff"
Peak Name: "Thr" Mass(es): "290.100/171.010 Da"
Comment: "" Annotation: ""
Sample Index: 47
Sample Type: Unknown
Concentration: N/A
Calculated Conc: 2.94 umol/L
Acq. Date: 2025-05-16
Acq. Time: 02:38:22
Modified: Yes
Proc. Algorithm: Specify Parameters - MQ III

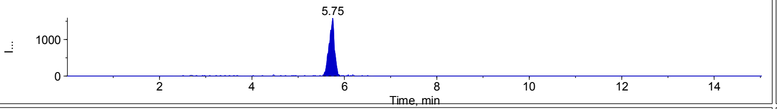

Sample Name: "ZY-22" Sample ID: "" File: "20250515-50AA.wiff"
Peak Name: "MSLAB-Nvl(IS)" Mass(es): "288.150/171.010 Da"
Comment: "" Annotation: ""
Sample Index: 47
Sample Type: Unknown
Concentration: 1.00 ng/mL
Calculated Conc: N/A
Acq. Date: 2025-05-16
Acq. Time: 02:38:22
Modified: Yes
Proc. Algorithm: Specify Parameters - MQ III

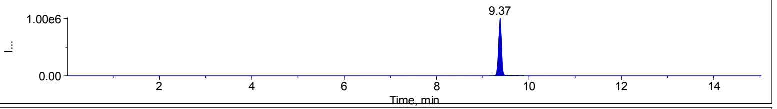

Sample Name: "ZY-22" Sample ID: "" File: "20250515-50AA.wiff"
Peak Name: "Cys1/2" Mass(es): "292.200/171.010 Da"
Comment: "" Annotation: ""
Sample Index: 47
Sample Type: Unknown
Concentration: N/A
Calculated Conc: 0.684 umol/L
Acq. Date: 2025-05-16
Acq. Time: 02:38:22
Modified: Yes
Proc. Algorithm: Specify Parameters - MQ III

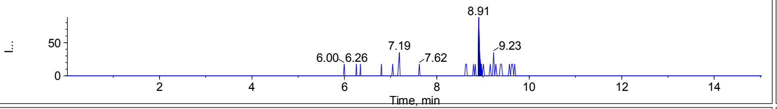

Sample Name: "ZY-22" Sample ID: "" File: "20250515-50AA.wiff"
Peak Name: "MSLAB-Nvl(IS)" Mass(es): "288.150/171.010 Da"
Comment: "" Annotation: ""
Sample Index: 47
Sample Type: Unknown
Concentration: 1.00 ng/mL
Calculated Conc: N/A
Acq. Date: 2025-05-16
Acq. Time: 02:38:22
Modified: Yes
Proc. Algorithm: Specify Parameters - MQ III

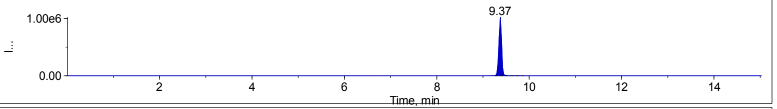

Sample Name: "ZY-22" Sample ID: "" File: "20250515-50AA.wiff"
Peak Name: "Ile" Mass(es): "302.150/171.010 Da"
Comment: "" Annotation: ""
Sample Index: 47
Sample Type: Unknown
Concentration: N/A
Calculated Conc: 0.962 umol/L
Acq. Date: 2025-05-16
Acq. Time: 02:38:22
Modified: Yes
Proc. Algorithm: Specify Parameters - MQ III

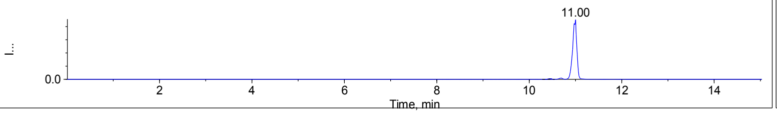

Sample Name: "ZY-22" Sample ID: "" File: "20250515-50AA.wiff"
Peak Name: "MSLAB-Nvl(IS)" Mass(es): "288.150/171.010 Da"
Comment: "" Annotation: ""
Sample Index: 47
Sample Type: Unknown
Concentration: 1.00 ng/mL
Calculated Conc: N/A
Acq. Date: 2025-05-16
Acq. Time: 02:38:22
Modified: Yes
Proc. Algorithm: Specify Parameters - MQ III

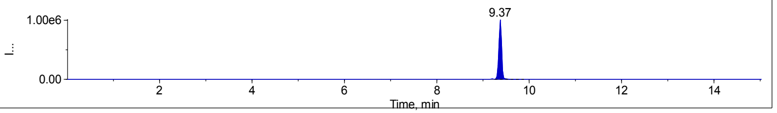

Sample Name: "ZY-22" Sample ID: "" File: "20250515-50AA.wiff"
Peak Name: "Asn" Mass(es): "303.130/171.010 Da"
Comment: "" Annotation: ""

```
Sample Index:      47
Sample Type:      Unknown
Concentration:      N/A
Calculated Conc:     < 0
Acq. Date:        2025-05-16
Acq. Time:        02:38:22

Modified:          Yes
Proc. Algorithm: Specify Parameters - MQ III
```

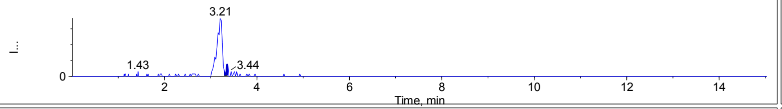

Sample Name: "ZY-22" Sample ID: "" File: "20250515-50AA.wiff"
Peak Name: "MSLAB-Nvl(IS)" Mass(es): "288.150/171.010 Da"
Comment: "" Annotation: ""

```
Sample Index:      47
Sample Type:      Unknown
Concentration:      1.00    ng/mL
Calculated Conc:    N/A
Acq. Date:        2025-05-16
Acq. Time:        02:38:22

Modified:          Yes
Proc. Algorithm: Specify Parameters - MQ III
```

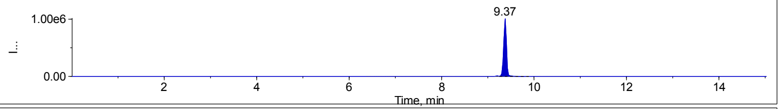

Sample Name: "ZY-22" Sample ID: "" File: "20250515-50AA.wiff"
Peak Name: "Asp" Mass(es): "304.110/171.010 Da"
Comment: "" Annotation: ""

```
Sample Index:      47
Sample Type:      Unknown
Concentration:      N/A
Calculated Conc:    0.267    umol/L
Acq. Date:        2025-05-16
Acq. Time:        02:38:22

Modified:          Yes
Proc. Algorithm: Specify Parameters - MQ III
```

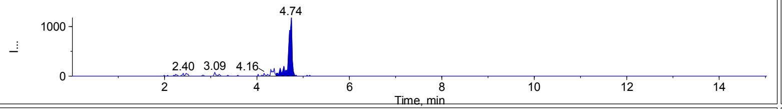

Sample Name: "ZY-22" Sample ID: "" File: "20250515-50AA.wiff"
Peak Name: "MSLAB-Nvl(IS)" Mass(es): "288.150/171.010 Da"
Comment: "" Annotation: ""

```
Sample Index:      47
Sample Type:      Unknown
Concentration:      1.00    ng/mL
Calculated Conc:    N/A
Acq. Date:        2025-05-16
Acq. Time:        02:38:22

Modified:          Yes
Proc. Algorithm: Specify Parameters - MQ III
```

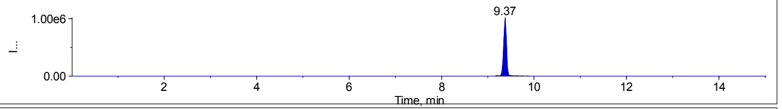

Sample Name: "ZY-22" Sample ID: "" File: "20250515-50AA.wiff"
Peak Name: "Gln" Mass(es): "317.150/171.010 Da"
Comment: "" Annotation: ""

```
Sample Index:      47
Sample Type:      Unknown
Concentration:      N/A
Calculated Conc: No Intercept
Acq. Date:        2025-05-16
Acq. Time:        02:38:22

Modified:          Yes
Proc. Algorithm: Specify Parameters - MQ III
```

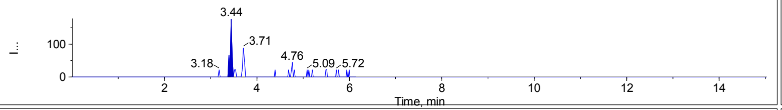

Sample Name: "ZY-22" Sample ID: "" File: "20250515-50AA.wiff"
Peak Name: "MSLAB-Nvl(IS)" Mass(es): "288.150/171.010 Da"
Comment: "" Annotation: ""

```
Sample Index:      47
Sample Type:      Unknown
Concentration:      1.00    ng/mL
Calculated Conc:    N/A
Acq. Date:        2025-05-16
Acq. Time:        02:38:22

Modified:          Yes
Proc. Algorithm: Specify Parameters - MQ III
```

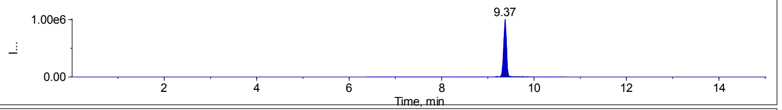

Sample Name: "ZY-22" Sample ID: "" File: "20250515-50AA.wiff"
Peak Name: "Glu" Mass(es): "318.100/171.010 Da"
Comment: "" Annotation: ""

```
Sample Index:      47
Sample Type:      Unknown
Concentration:      N/A
Calculated Conc:    2.88    umol/L
Acq. Date:        2025-05-16
Acq. Time:        02:38:22

Modified:          Yes
Proc. Algorithm: Specify Parameters - MQ III
```

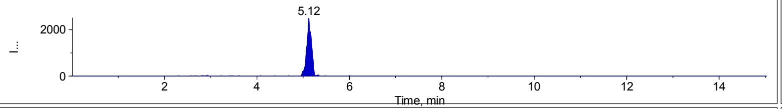

Sample Name: "ZY-22" Sample ID: "" File: "20250515-50AA.wiff"
Peak Name: "MSLAB-Nvl(IS)" Mass(es): "288.150/171.010 Da"
Comment: "" Annotation: ""

```
Sample Index:      47
Sample Type:      Unknown
Concentration:      1.00    ng/mL
Calculated Conc:    N/A
Acq. Date:        2025-05-16
Acq. Time:        02:38:22

Modified:          Yes
Proc. Algorithm: Specify Parameters - MQ III
```

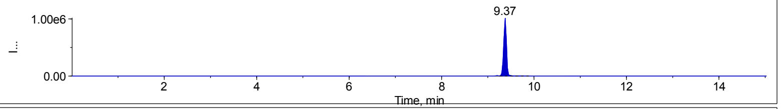

Sample Name: "ZY-22" Sample ID: "" File: "20250515-50AA.wiff"
Peak Name: "Met" Mass(es): "320.210/171.010 Da"
Comment: "" Annotation: ""

```
Sample Index:      47
Sample Type:      Unknown
Concentration:      N/A
Calculated Conc:     < 0
Acq. Date:        2025-05-16
Acq. Time:        02:38:22

Modified:          Yes
Proc. Algorithm: Specify Parameters - MQ III
```

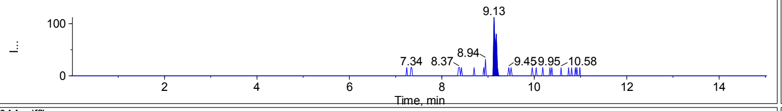

Sample Name: "ZY-22" Sample ID: "" File: "20250515-50AA.wiff"
Peak Name: "MSLAB-Nvl(IS)" Mass(es): "288.150/171.010 Da"
Comment: "" Annotation: ""

```
Sample Index:      47
Sample Type:      Unknown
Concentration:      1.00    ng/mL
Calculated Conc:    N/A
Acq. Date:        2025-05-16
Acq. Time:        02:38:22

Modified:          Yes
Proc. Algorithm: Specify Parameters - MQ III
```

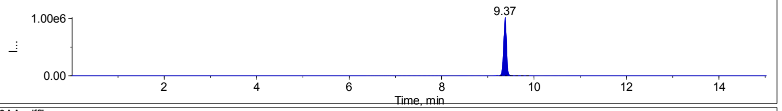

Sample Name: "ZY-22" Sample ID: "" File: "20250515-50AA.wiff"
Peak Name: "His" Mass(es): "326.150/171.010 Da"
Comment: "" Annotation: ""

```
Sample Index:      47
Sample Type:      Unknown
Concentration:      N/A
Calculated Conc:    1.48    umol/L
Acq. Date:        2025-05-16
Acq. Time:        02:38:22

Modified:          Yes
Proc. Algorithm: Specify Parameters - MQ III
```

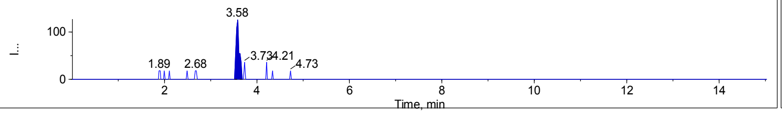

Sample Name: "ZY-22" Sample ID: "" File: "20250515-50AA.wiff"
Peak Name: "MSLAB-Nvl(IS)" Mass(es): "288.150/171.010 Da"
Comment: "" Annotation: ""

```
Sample Index:      47
Sample Type:      Unknown
Concentration:      1.00    ng/mL
Calculated Conc:    N/A
Acq. Date:        2025-05-16
Acq. Time:        02:38:22

Modified:          Yes
Proc. Algorithm: Specify Parameters - MQ III
```

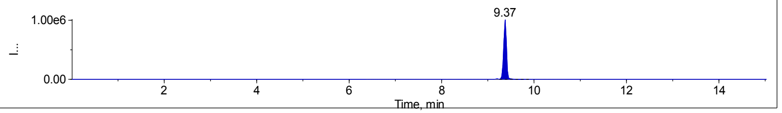

Sample Name: "ZY-22" Sample ID: "" File: "20250515-50AA.wiff"
Peak Name: "Phe" Mass(es): "336.200/171.010 Da"
Comment: "" Annotation: ""

```
Sample Index:       47
Sample Type:      Unknown
Concentration:       N/A
Calculated Conc:     1.24   umol/L
Acq. Date:        2025-05-16
Acq. Time:        02:38:22

Modified:            Yes
Proc. Algorithm: Specify Parameters - MQ III
```

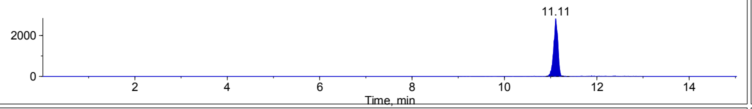

Sample Name: "ZY-22" Sample ID: "" File: "20250515-50AA.wiff"
Peak Name: "Arg" Mass(es): "345.210/171.010 Da"
Comment: "" Annotation: ""

```
Sample Index:       47
Sample Type:      Unknown
Concentration:       N/A
Calculated Conc:     2.52   umol/L
Acq. Date:        2025-05-16
Acq. Time:        02:38:22

Modified:            Yes
Proc. Algorithm: Specify Parameters - MQ III
```

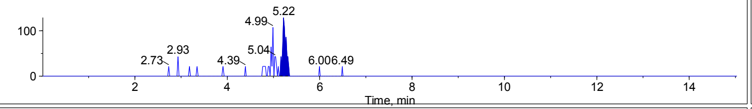

Sample Name: "ZY-22" Sample ID: "" File: "20250515-50AA.wiff"
Peak Name: "Trp" Mass(es): "375.200/171.010 Da"
Comment: "" Annotation: ""

```
Sample Index:       47
Sample Type:      Unknown
Concentration:       N/A
Calculated Conc:      < 0
Acq. Date:        2025-05-16
Acq. Time:        02:38:22

Modified:            Yes
Proc. Algorithm: Specify Parameters - MQ III
```

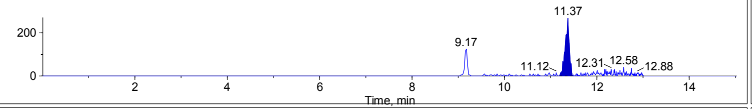

Sample Name: "ZY-22" Sample ID: "" File: "20250515-50AA.wiff"
Peak Name: "Lys" Mass(es): "487.200/171.010 Da"
Comment: "" Annotation: ""

```
Sample Index:       47
Sample Type:      Unknown
Concentration:       N/A
Calculated Conc:     2.65   umol/L
Acq. Date:        2025-05-16
Acq. Time:        02:38:22

Modified:            Yes
Proc. Algorithm: Specify Parameters - MQ III
```

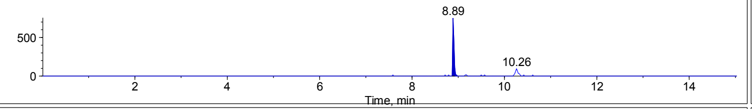

Sample Name: "ZY-22" Sample ID: "" File: "20250515-50AA.wiff"
Peak Name: "tyr" Mass(es): "352.200/171.010 Da"
Comment: "" Annotation: ""

```
Sample Index:       47
Sample Type:      Unknown
Concentration:       N/A
Calculated Conc:      < 0
Acq. Date:        2025-05-16
Acq. Time:        02:38:22

Modified:            Yes
Proc. Algorithm: Specify Parameters - MQ III
```

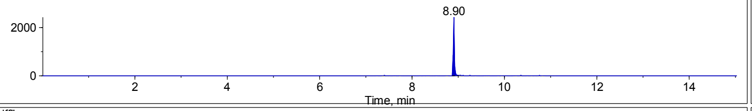

Sample Name: "ZY-22" Sample ID: "" File: "20250515-50AA.wiff"
Peak Name: "Leu" Mass(es): "302.150/171.010 Da"
Comment: "" Annotation: ""

```
Sample Index:       47
Sample Type:      Unknown
Concentration:       N/A
Calculated Conc:     2.50   umol/L
Acq. Date:        2025-05-16
Acq. Time:        02:38:22

Modified:            Yes
Proc. Algorithm: Specify Parameters - MQ III
```

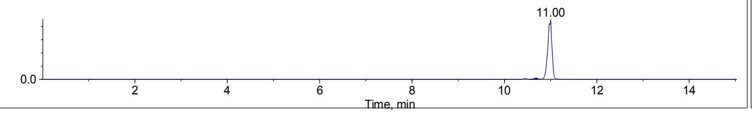

Sample Name: "ZY-22" Sample ID: "" File: "20250515-50AA.wiff"
Peak Name: "MSLAB-Nvl(IS)" Mass(es): "288.150/171.010 Da"
Comment: "" Annotation: ""

```
Sample Index:       47
Sample Type:      Unknown
Concentration:      1.00    ng/mL
Calculated Conc:     N/A
Acq. Date:        2025-05-16
Acq. Time:        02:38:22

Modified:            Yes
Proc. Algorithm: Specify Parameters - MQ III
```

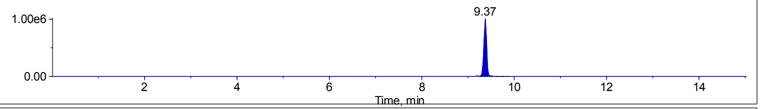

Sample Name: "ZY-22" Sample ID: "" File: "20250515-50AA.wiff"
Peak Name: "MSLAB-Nvl(IS)" Mass(es): "288.150/171.010 Da"
Comment: "" Annotation: ""

```
Sample Index:       47
Sample Type:      Unknown
Concentration:      1.00    ng/mL
Calculated Conc:     N/A
Acq. Date:        2025-05-16
Acq. Time:        02:38:22

Modified:            Yes
Proc. Algorithm: Specify Parameters - MQ III
```

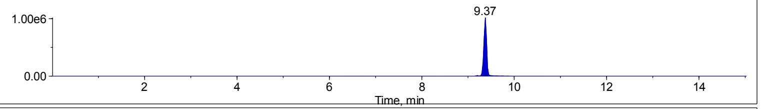

Sample Name: "ZY-22" Sample ID: "" File: "20250515-50AA.wiff"
Peak Name: "MSLAB-Nvl(IS)" Mass(es): "288.150/171.010 Da"
Comment: "" Annotation: ""

```
Sample Index:       47
Sample Type:      Unknown
Concentration:      1.00    ng/mL
Calculated Conc:     N/A
Acq. Date:        2025-05-16
Acq. Time:        02:38:22

Modified:            Yes
Proc. Algorithm: Specify Parameters - MQ III
```

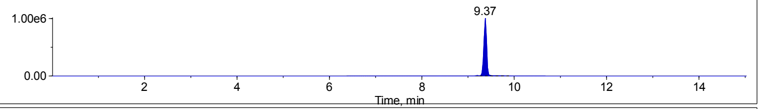

Sample Name: "ZY-22" Sample ID: "" File: "20250515-50AA.wiff"
Peak Name: "MSLAB-Nvl(IS)" Mass(es): "288.150/171.010 Da"
Comment: "" Annotation: ""

```
Sample Index:       47
Sample Type:      Unknown
Concentration:      1.00    ng/mL
Calculated Conc:     N/A
Acq. Date:        2025-05-16
Acq. Time:        02:38:22

Modified:            Yes
Proc. Algorithm: Specify Parameters - MQ III
```

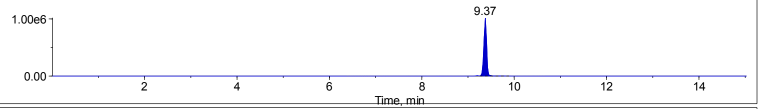

Sample Name: "ZY-22" Sample ID: "" File: "20250515-50AA.wiff"
Peak Name: "MSLAB-Nvl(IS)" Mass(es): "288.150/171.010 Da"
Comment: "" Annotation: ""

```
Sample Index:       47
Sample Type:      Unknown
Concentration:      1.00    ng/mL
Calculated Conc:     N/A
Acq. Date:        2025-05-16
Acq. Time:        02:38:22

Modified:            Yes
Proc. Algorithm: Specify Parameters - MQ III
```

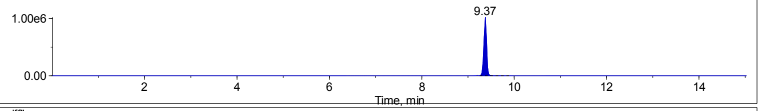

Sample Name: "ZY-22" Sample ID: "" File: "20250515-50AA.wiff"
Peak Name: "MSLAB-Nvl(IS)" Mass(es): "288.150/171.010 Da"
Comment: "" Annotation: ""

```
Sample Index:       47
Sample Type:      Unknown
Concentration:      1.00    ng/mL
Calculated Conc:     N/A
Acq. Date:        2025-05-16
Acq. Time:        02:38:22

Modified:            Yes
Proc. Algorithm: Specify Parameters - MQ III
```

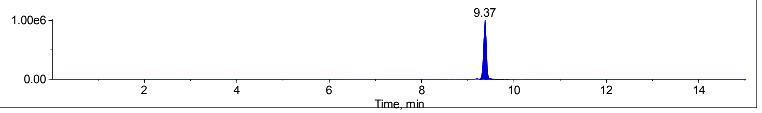

Sample Name: "ZY-23" Sample ID: "" File: "20250515-50AA.wiff"
Peak Name: "Gly" Mass(es): "246.080/171.010 Da"
Comment: "" Annotation: ""
Sample Index: 48
Sample Type: Unknown
Concentration: N/A
Calculated Conc: 2.85 umol/L
Acq. Date: 2025-05-16
Acq. Time: 02:53:51
Modified: Yes
Proc. Algorithm: Specify Parameters - MQ III

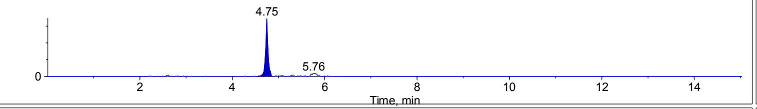

Sample Name: "ZY-23" Sample ID: "" File: "20250515-50AA.wiff"
Peak Name: "MSLAB-Nvl(IS)" Mass(es): "288.150/171.010 Da"
Comment: "" Annotation: ""
Sample Index: 48
Sample Type: Unknown
Concentration: 1.00 ng/mL
Calculated Conc: N/A
Acq. Date: 2025-05-16
Acq. Time: 02:53:51
Modified: Yes
Proc. Algorithm: Specify Parameters - MQ III

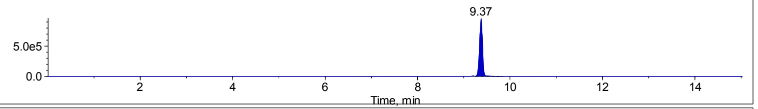

Sample Name: "ZY-23" Sample ID: "" File: "20250515-50AA.wiff"
Peak Name: "Ala" Mass(es): "260.100/171.010 Da"
Comment: "" Annotation: ""
Sample Index: 48
Sample Type: Unknown
Concentration: N/A
Calculated Conc: No Intercept
Acq. Date: 2025-05-16
Acq. Time: 02:53:51
Modified: Yes
Proc. Algorithm: Specify Parameters - MQ III

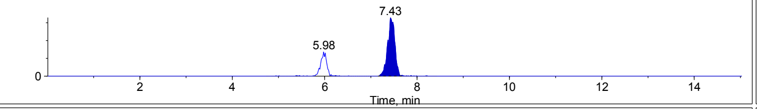

Sample Name: "ZY-23" Sample ID: "" File: "20250515-50AA.wiff"
Peak Name: "MSLAB-Nvl(IS)" Mass(es): "288.150/171.010 Da"
Comment: "" Annotation: ""
Sample Index: 48
Sample Type: Unknown
Concentration: 1.00 ng/mL
Calculated Conc: N/A
Acq. Date: 2025-05-16
Acq. Time: 02:53:51
Modified: Yes
Proc. Algorithm: Specify Parameters - MQ III

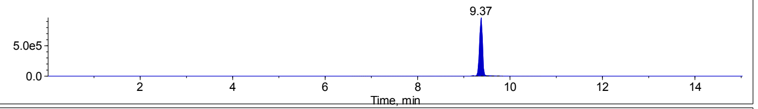

Sample Name: "ZY-23" Sample ID: "" File: "20250515-50AA.wiff"
Peak Name: "Ser" Mass(es): "276.100/171.010 Da"
Comment: "" Annotation: ""
Sample Index: 48
Sample Type: Unknown
Concentration: N/A
Calculated Conc: < 0
Acq. Date: 2025-05-16
Acq. Time: 02:53:51
Modified: Yes
Proc. Algorithm: Specify Parameters - MQ III

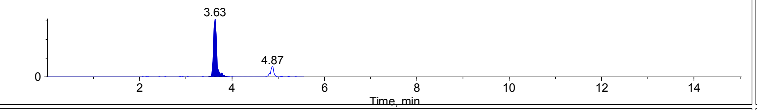

Sample Name: "ZY-23" Sample ID: "" File: "20250515-50AA.wiff"
Peak Name: "MSLAB-Nvl(IS)" Mass(es): "288.150/171.010 Da"
Comment: "" Annotation: ""
Sample Index: 48
Sample Type: Unknown
Concentration: 1.00 ng/mL
Calculated Conc: N/A
Acq. Date: 2025-05-16
Acq. Time: 02:53:51
Modified: Yes
Proc. Algorithm: Specify Parameters - MQ III

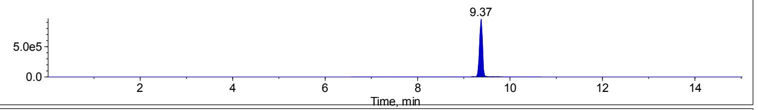

Sample Name: "ZY-23" Sample ID: "" File: "20250515-50AA.wiff"
Peak Name: "Pro" Mass(es): "286.150/171.010 Da"
Comment: "" Annotation: ""
Sample Index: 48
Sample Type: Unknown
Concentration: N/A
Calculated Conc: No Intercept
Acq. Date: 2025-05-16
Acq. Time: 02:53:51
Modified: Yes
Proc. Algorithm: Specify Parameters - MQ III

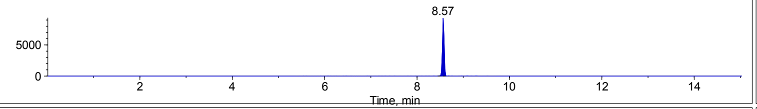

Sample Name: "ZY-23" Sample ID: "" File: "20250515-50AA.wiff"
Peak Name: "MSLAB-Nvl(IS)" Mass(es): "288.150/171.010 Da"
Comment: "" Annotation: ""
Sample Index: 48
Sample Type: Unknown
Concentration: 1.00 ng/mL
Calculated Conc: N/A
Acq. Date: 2025-05-16
Acq. Time: 02:53:51
Modified: Yes
Proc. Algorithm: Specify Parameters - MQ III

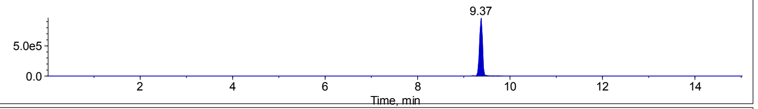

Sample Name: "ZY-23" Sample ID: "" File: "20250515-50AA.wiff"
Peak Name: "Val" Mass(es): "288.150/171.010 Da"
Comment: "" Annotation: ""
Sample Index: 48
Sample Type: Unknown
Concentration: N/A
Calculated Conc: 3.42 umol/L
Acq. Date: 2025-05-16
Acq. Time: 02:53:51
Modified: Yes
Proc. Algorithm: Specify Parameters - MQ III

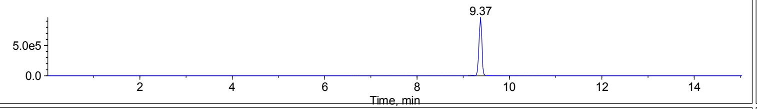

Sample Name: "ZY-23" Sample ID: "" File: "20250515-50AA.wiff"
Peak Name: "MSLAB-Nvl(IS)" Mass(es): "288.150/171.010 Da"
Comment: "" Annotation: ""
Sample Index: 48
Sample Type: Unknown
Concentration: 1.00 ng/mL
Calculated Conc: N/A
Acq. Date: 2025-05-16
Acq. Time: 02:53:51
Modified: Yes
Proc. Algorithm: Specify Parameters - MQ III

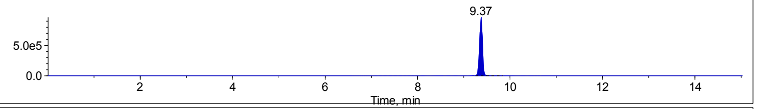

Sample Name: "ZY-23" Sample ID: "" File: "20250515-50AA.wiff"
Peak Name: "Thr" Mass(es): "290.100/171.010 Da"
Comment: "" Annotation: ""
Sample Index: 48
Sample Type: Unknown
Concentration: N/A
Calculated Conc: 2.07 umol/L
Acq. Date: 2025-05-16
Acq. Time: 02:53:51
Modified: Yes
Proc. Algorithm: Specify Parameters - MQ III

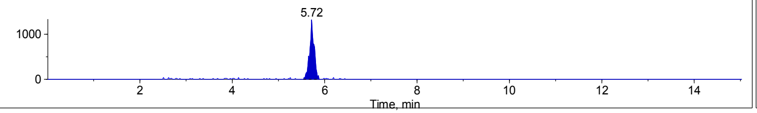

Sample Name: "ZY-23" Sample ID: "" File: "20250515-50AA.wiff"
Peak Name: "MSLAB-Nvl(IS)" Mass(es): "288.150/171.010 Da"
Comment: "" Annotation: ""
Sample Index: 48
Sample Type: Unknown
Concentration: 1.00 ng/mL
Calculated Conc: N/A
Acq. Date: 2025-05-16
Acq. Time: 02:53:51
Modified: Yes
Proc. Algorithm: Specify Parameters - MQ III

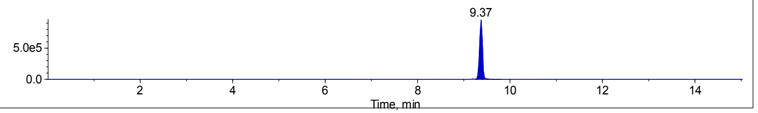

Sample Name: "ZY-23" Sample ID: "" File: "20250515-50AA.wiff"
Peak Name: "Cys1/2" Mass(es): "292.200/171.010 Da"
Comment: "" Annotation: ""

```
Sample Index:       48
Sample Type:      Unknown
Concentration:      N/A
Calculated Conc:    1.10    umol/L
Acq. Date:        2025-05-16
Acq. Time:        02:53:51

Modified:           Yes
Proc. Algorithm: Specify Parameters - MQ III
```

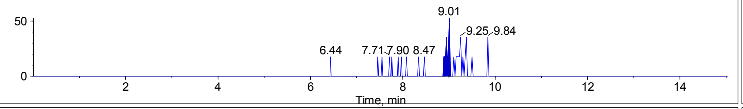

Sample Name: "ZY-23" Sample ID: "" File: "20250515-50AA.wiff"
Peak Name: "MSLAB-Nvl(IS)" Mass(es): "288.150/171.010 Da"
Comment: "" Annotation: ""

```
Sample Index:       48
Sample Type:      Unknown
Concentration:      1.00    ng/mL
Calculated Conc:    N/A
Acq. Date:        2025-05-16
Acq. Time:        02:53:51

Modified:           Yes
Proc. Algorithm: Specify Parameters - MQ III
```

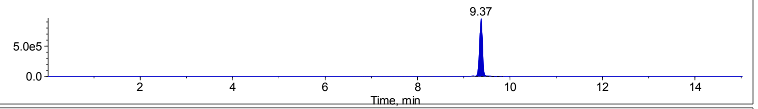

Sample Name: "ZY-23" Sample ID: "" File: "20250515-50AA.wiff"
Peak Name: "Ile" Mass(es): "302.150/171.010 Da"
Comment: "" Annotation: ""

```
Sample Index:       48
Sample Type:      Unknown
Concentration:      N/A
Calculated Conc:    0.946   umol/L
Acq. Date:        2025-05-16
Acq. Time:        02:53:51

Modified:           Yes
Proc. Algorithm: Specify Parameters - MQ III
```

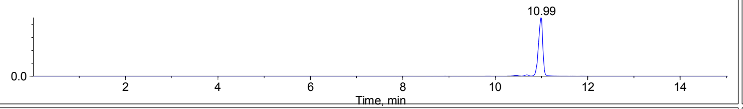

Sample Name: "ZY-23" Sample ID: "" File: "20250515-50AA.wiff"
Peak Name: "MSLAB-Nvl(IS)" Mass(es): "288.150/171.010 Da"
Comment: "" Annotation: ""

```
Sample Index:       48
Sample Type:      Unknown
Concentration:      1.00    ng/mL
Calculated Conc:    N/A
Acq. Date:        2025-05-16
Acq. Time:        02:53:51

Modified:           Yes
Proc. Algorithm: Specify Parameters - MQ III
```

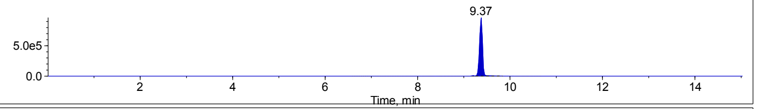

Sample Name: "ZY-23" Sample ID: "" File: "20250515-50AA.wiff"
Peak Name: "Asn" Mass(es): "303.130/171.010 Da"
Comment: "" Annotation: ""

```
Sample Index:       48
Sample Type:      Unknown
Concentration:      N/A
Calculated Conc:      < 0
Acq. Date:        2025-05-16
Acq. Time:        02:53:51

Modified:           Yes
Proc. Algorithm: Specify Parameters - MQ III
```

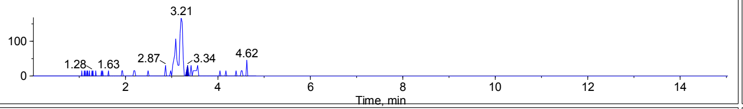

Sample Name: "ZY-23" Sample ID: "" File: "20250515-50AA.wiff"
Peak Name: "MSLAB-Nvl(IS)" Mass(es): "288.150/171.010 Da"
Comment: "" Annotation: ""

```
Sample Index:       48
Sample Type:      Unknown
Concentration:      1.00    ng/mL
Calculated Conc:    N/A
Acq. Date:        2025-05-16
Acq. Time:        02:53:51

Modified:           Yes
Proc. Algorithm: Specify Parameters - MQ III
```

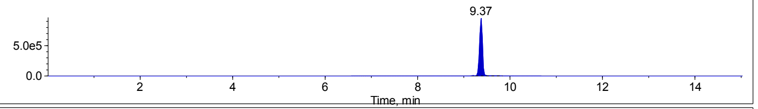

Sample Name: "ZY-23" Sample ID: "" File: "20250515-50AA.wiff"
Peak Name: "Asp" Mass(es): "304.110/171.010 Da"
Comment: "" Annotation: ""

```
Sample Index:       48
Sample Type:      Unknown
Concentration:      N/A
Calculated Conc:    0.246   umol/L
Acq. Date:        2025-05-16
Acq. Time:        02:53:51

Modified:           Yes
Proc. Algorithm: Specify Parameters - MQ III
```

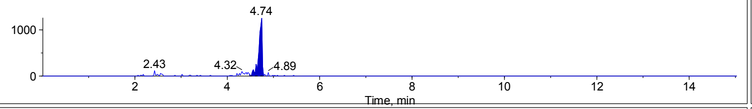

Sample Name: "ZY-23" Sample ID: "" File: "20250515-50AA.wiff"
Peak Name: "MSLAB-Nvl(IS)" Mass(es): "288.150/171.010 Da"
Comment: "" Annotation: ""

```
Sample Index:       48
Sample Type:      Unknown
Concentration:      1.00    ng/mL
Calculated Conc:    N/A
Acq. Date:        2025-05-16
Acq. Time:        02:53:51

Modified:           Yes
Proc. Algorithm: Specify Parameters - MQ III
```

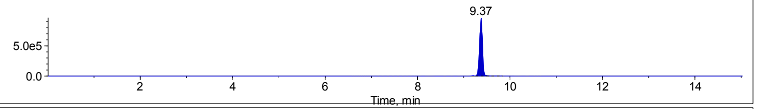

Sample Name: "ZY-23" Sample ID: "" File: "20250515-50AA.wiff"
Peak Name: "Gln" Mass(es): "317.150/171.010 Da"
Comment: "" Annotation: ""

```
Sample Index:       48
Sample Type:      Unknown
Concentration:      N/A
Calculated Conc: No Intercept
Acq. Date:        2025-05-16
Acq. Time:        02:53:51

Modified:           Yes
Proc. Algorithm: Specify Parameters - MQ III
```

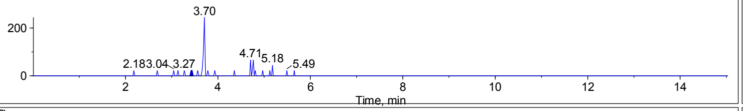

Sample Name: "ZY-23" Sample ID: "" File: "20250515-50AA.wiff"
Peak Name: "MSLAB-Nvl(IS)" Mass(es): "288.150/171.010 Da"
Comment: "" Annotation: ""

```
Sample Index:       48
Sample Type:      Unknown
Concentration:      1.00    ng/mL
Calculated Conc:    N/A
Acq. Date:        2025-05-16
Acq. Time:        02:53:51

Modified:           Yes
Proc. Algorithm: Specify Parameters - MQ III
```

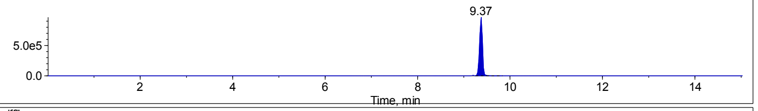

Sample Name: "ZY-23" Sample ID: "" File: "20250515-50AA.wiff"
Peak Name: "Glu" Mass(es): "318.100/171.010 Da"
Comment: "" Annotation: ""

```
Sample Index:       48
Sample Type:      Unknown
Concentration:      N/A
Calculated Conc:    3.75    umol/L
Acq. Date:        2025-05-16
Acq. Time:        02:53:51

Modified:           Yes
Proc. Algorithm: Specify Parameters - MQ III
```

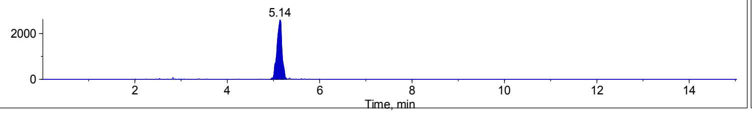

Sample Name: "ZY-23" Sample ID: "" File: "20250515-50AA.wiff"
Peak Name: "MSLAB-Nvl(IS)" Mass(es): "288.150/171.010 Da"
Comment: "" Annotation: ""

```
Sample Index:       48
Sample Type:      Unknown
Concentration:      1.00    ng/mL
Calculated Conc:    N/A
Acq. Date:        2025-05-16
Acq. Time:        02:53:51

Modified:           Yes
Proc. Algorithm: Specify Parameters - MQ III
```

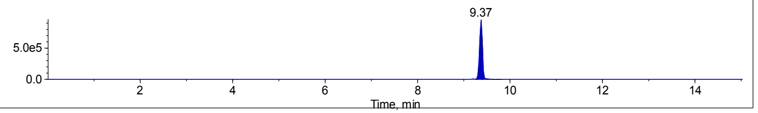

Sample Name: "ZY-23" Sample ID: "" File: "20250515-50AA.wiff"
Peak Name: "Met" Mass(es): "320.210/171.010 Da"
Comment: "" Annotation: ""

Sample Index: 48
Sample Type: Unknown
Concentration: N/A
Calculated Conc: < 0
Acq. Date: 2025-05-16
Acq. Time: 02:53:51

Modified: Yes
Proc. Algorithm: Specify Parameters - MQ III

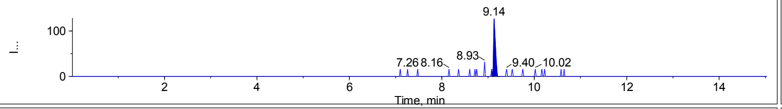

Sample Name: "ZY-23" Sample ID: "" File: "20250515-50AA.wiff"
Peak Name: "MSLAB-Nvl(IS)" Mass(es): "288.150/171.010 Da"
Comment: "" Annotation: ""

Sample Index: 48
Sample Type: Unknown
Concentration: 1.00 ng/mL
Calculated Conc: N/A
Acq. Date: 2025-05-16
Acq. Time: 02:53:51

Modified: Yes
Proc. Algorithm: Specify Parameters - MQ III

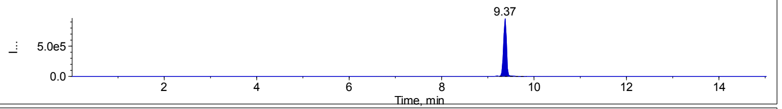

Sample Name: "ZY-23" Sample ID: "" File: "20250515-50AA.wiff"
Peak Name: "His" Mass(es): "326.150/171.010 Da"
Comment: "" Annotation: ""

Sample Index: 48
Sample Type: Unknown
Concentration: N/A
Calculated Conc: 1.12 umol/L
Acq. Date: 2025-05-16
Acq. Time: 02:53:51

Modified: Yes
Proc. Algorithm: Specify Parameters - MQ III

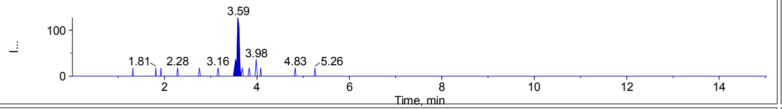

Sample Name: "ZY-23" Sample ID: "" File: "20250515-50AA.wiff"
Peak Name: "MSLAB-Nvl(IS)" Mass(es): "288.150/171.010 Da"
Comment: "" Annotation: ""

Sample Index: 48
Sample Type: Unknown
Concentration: 1.00 ng/mL
Calculated Conc: N/A
Acq. Date: 2025-05-16
Acq. Time: 02:53:51

Modified: Yes
Proc. Algorithm: Specify Parameters - MQ III

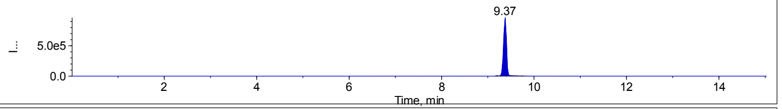

Sample Name: "ZY-23" Sample ID: "" File: "20250515-50AA.wiff"
Peak Name: "Phe" Mass(es): "336.200/171.010 Da"
Comment: "" Annotation: ""

Sample Index: 48
Sample Type: Unknown
Concentration: N/A
Calculated Conc: 1.17 umol/L
Acq. Date: 2025-05-16
Acq. Time: 02:53:51

Modified: Yes
Proc. Algorithm: Specify Parameters - MQ III

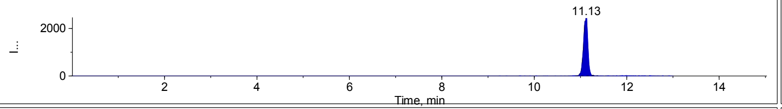

Sample Name: "ZY-23" Sample ID: "" File: "20250515-50AA.wiff"
Peak Name: "MSLAB-Nvl(IS)" Mass(es): "288.150/171.010 Da"
Comment: "" Annotation: ""

Sample Index: 48
Sample Type: Unknown
Concentration: 1.00 ng/mL
Calculated Conc: N/A
Acq. Date: 2025-05-16
Acq. Time: 02:53:51

Modified: Yes
Proc. Algorithm: Specify Parameters - MQ III

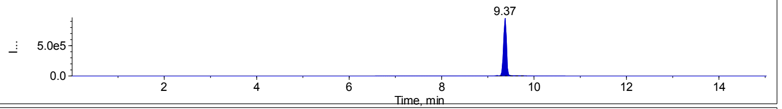

Sample Name: "ZY-23" Sample ID: "" File: "20250515-50AA.wiff"
Peak Name: "Arg" Mass(es): "345.210/171.010 Da"
Comment: "" Annotation: ""

Sample Index: 48
Sample Type: Unknown
Concentration: N/A
Calculated Conc: 3.39 umol/L
Acq. Date: 2025-05-16
Acq. Time: 02:53:51

Modified: Yes
Proc. Algorithm: Specify Parameters - MQ III

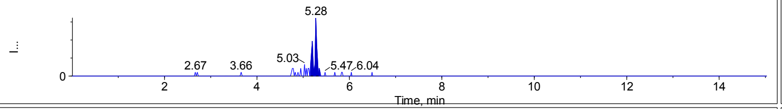

Sample Name: "ZY-23" Sample ID: "" File: "20250515-50AA.wiff"
Peak Name: "MSLAB-Nvl(IS)" Mass(es): "288.150/171.010 Da"
Comment: "" Annotation: ""

Sample Index: 48
Sample Type: Unknown
Concentration: 1.00 ng/mL
Calculated Conc: N/A
Acq. Date: 2025-05-16
Acq. Time: 02:53:51

Modified: Yes
Proc. Algorithm: Specify Parameters - MQ III

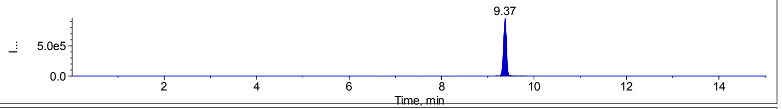

Sample Name: "ZY-23" Sample ID: "" File: "20250515-50AA.wiff"
Peak Name: "Trp" Mass(es): "375.200/171.010 Da"
Comment: "" Annotation: ""

Sample Index: 48
Sample Type: Unknown
Concentration: N/A
Calculated Conc: < 0
Acq. Date: 2025-05-16
Acq. Time: 02:53:51

Modified: Yes
Proc. Algorithm: Specify Parameters - MQ III

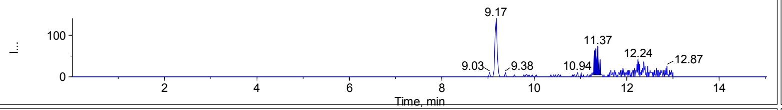

Sample Name: "ZY-23" Sample ID: "" File: "20250515-50AA.wiff"
Peak Name: "MSLAB-Nvl(IS)" Mass(es): "288.150/171.010 Da"
Comment: "" Annotation: ""

Sample Index: 48
Sample Type: Unknown
Concentration: 1.00 ng/mL
Calculated Conc: N/A
Acq. Date: 2025-05-16
Acq. Time: 02:53:51

Modified: Yes
Proc. Algorithm: Specify Parameters - MQ III

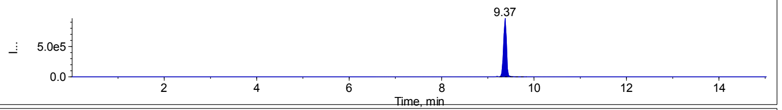

Sample Name: "ZY-23" Sample ID: "" File: "20250515-50AA.wiff"
Peak Name: "Lys" Mass(es): "487.200/171.010 Da"
Comment: "" Annotation: ""

Sample Index: 48
Sample Type: Unknown
Concentration: N/A
Calculated Conc: 2.95 umol/L
Acq. Date: 2025-05-16
Acq. Time: 02:53:51

Modified: Yes
Proc. Algorithm: Specify Parameters - MQ III

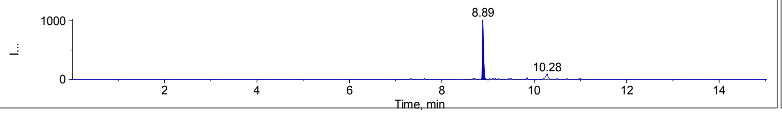

Sample Name: "ZY-23" Sample ID: "" File: "20250515-50AA.wiff"
Peak Name: "MSLAB-Nvl(IS)" Mass(es): "288.150/171.010 Da"
Comment: "" Annotation: ""

Sample Index: 48
Sample Type: Unknown
Concentration: 1.00 ng/mL
Calculated Conc: N/A
Acq. Date: 2025-05-16
Acq. Time: 02:53:51

Modified: Yes
Proc. Algorithm: Specify Parameters - MQ III

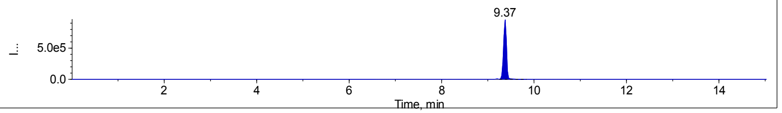

Sample Name: "ZY-23" Sample ID: "" File: "20250515-50AA.wiff"
Peak Name: "tyr" Mass(es): "352.200/171.010 Da"
Comment: "" Annotation: ""

Sample Index: 48
Sample Type: Unknown
Concentration: N/A
Calculated Conc: No Intercept
Acq. Date: 2025-05-16
Acq. Time: 02:53:51

Modified: Yes
Proc. Algorithm: Specify Parameters - MQ III

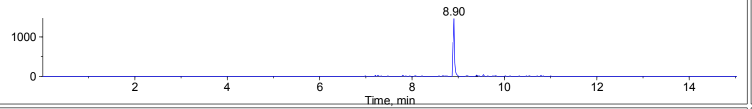

Sample Name: "ZY-23" Sample ID: "" File: "20250515-50AA.wiff"
Peak Name: "MSLAB-Nvl(IS)" Mass(es): "288.150/171.010 Da"
Comment: "" Annotation: ""

Sample Index: 48
Sample Type: Unknown
Concentration: 1.00 ng/mL
Calculated Conc: N/A
Acq. Date: 2025-05-16
Acq. Time: 02:53:51

Modified: Yes
Proc. Algorithm: Specify Parameters - MQ III

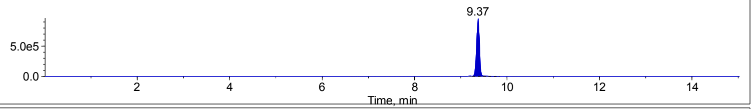

Sample Name: "ZY-23" Sample ID: "" File: "20250515-50AA.wiff"
Peak Name: "Leu" Mass(es): "302.150/171.010 Da"
Comment: "" Annotation: ""

Sample Index: 48
Sample Type: Unknown
Concentration: N/A
Calculated Conc: 2.68 umol/L
Acq. Date: 2025-05-16
Acq. Time: 02:53:51

Modified: Yes
Proc. Algorithm: Specify Parameters - MQ III

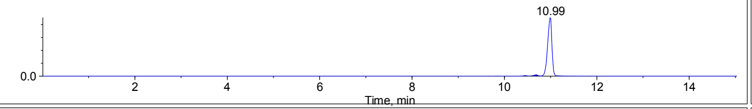

Sample Name: "ZY-23" Sample ID: "" File: "20250515-50AA.wiff"
Peak Name: "MSLAB-Nvl(IS)" Mass(es): "288.150/171.010 Da"
Comment: "" Annotation: ""

Sample Index: 48
Sample Type: Unknown
Concentration: 1.00 ng/mL
Calculated Conc: N/A
Acq. Date: 2025-05-16
Acq. Time: 02:53:51

Modified: Yes
Proc. Algorithm: Specify Parameters - MQ III

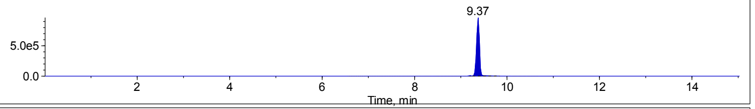

Sample Name: "ZY-24" Sample ID: "" File: "20250515-50AA.wiff"
Peak Name: "Gly" Mass(es): "246.080/171.010 Da"
Comment: "" Annotation: ""

Sample Index: 49
Sample Type: Unknown
Concentration: N/A
Calculated Conc: 0.0964 umol/L
Acq. Date: 2025-05-16
Acq. Time: 03:09:20

Modified: Yes
Proc. Algorithm: Specify Parameters - MQ III

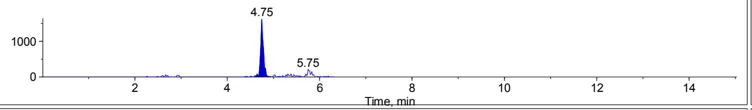

Sample Name: "ZY-24" Sample ID: "" File: "20250515-50AA.wiff"
Peak Name: "MSLAB-Nvl(IS)" Mass(es): "288.150/171.010 Da"
Comment: "" Annotation: ""

Sample Index: 49
Sample Type: Unknown
Concentration: 1.00 ng/mL
Calculated Conc: N/A
Acq. Date: 2025-05-16
Acq. Time: 03:09:20

Modified: Yes
Proc. Algorithm: Specify Parameters - MQ III

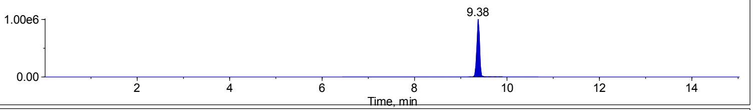

Sample Name: "ZY-24" Sample ID: "" File: "20250515-50AA.wiff"
Peak Name: "Ala" Mass(es): "260.100/171.010 Da"
Comment: "" Annotation: ""

Sample Index: 49
Sample Type: Unknown
Concentration: N/A
Calculated Conc: No Intercept
Acq. Date: 2025-05-16
Acq. Time: 03:09:20

Modified: Yes
Proc. Algorithm: Specify Parameters - MQ III

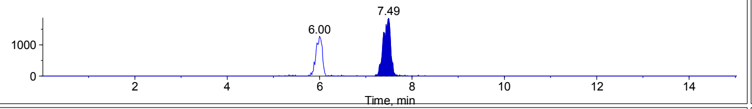

Sample Name: "ZY-24" Sample ID: "" File: "20250515-50AA.wiff"
Peak Name: "MSLAB-Nvl(IS)" Mass(es): "288.150/171.010 Da"
Comment: "" Annotation: ""

Sample Index: 49
Sample Type: Unknown
Concentration: 1.00 ng/mL
Calculated Conc: N/A
Acq. Date: 2025-05-16
Acq. Time: 03:09:20

Modified: Yes
Proc. Algorithm: Specify Parameters - MQ III

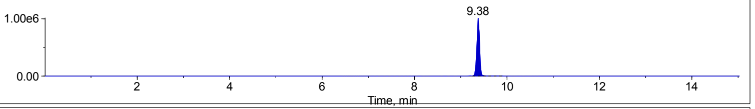

Sample Name: "ZY-24" Sample ID: "" File: "20250515-50AA.wiff"
Peak Name: "Ser" Mass(es): "276.100/171.010 Da"
Comment: "" Annotation: ""

Sample Index: 49
Sample Type: Unknown
Concentration: N/A
Calculated Conc: No Intercept
Acq. Date: 2025-05-16
Acq. Time: 03:09:20

Modified: Yes
Proc. Algorithm: Specify Parameters - MQ III

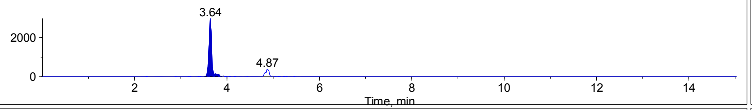

Sample Name: "ZY-24" Sample ID: "" File: "20250515-50AA.wiff"
Peak Name: "MSLAB-Nvl(IS)" Mass(es): "288.150/171.010 Da"
Comment: "" Annotation: ""

Sample Index: 49
Sample Type: Unknown
Concentration: 1.00 ng/mL
Calculated Conc: N/A
Acq. Date: 2025-05-16
Acq. Time: 03:09:20

Modified: Yes
Proc. Algorithm: Specify Parameters - MQ III

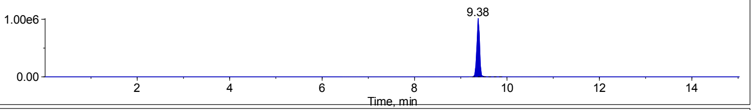

Sample Name: "ZY-24" Sample ID: "" File: "20250515-50AA.wiff"
Peak Name: "Pro" Mass(es): "286.150/171.010 Da"
Comment: "" Annotation: ""

Sample Index: 49
Sample Type: Unknown
Concentration: N/A
Calculated Conc: No Intercept
Acq. Date: 2025-05-16
Acq. Time: 03:09:20

Modified: Yes
Proc. Algorithm: Specify Parameters - MQ III

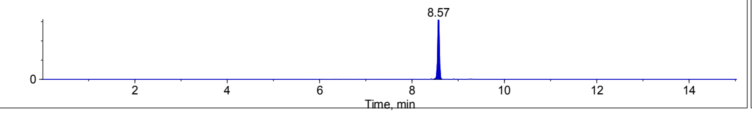

Sample Name: "ZY-24" Sample ID: "" File: "20250515-50AA.wiff"
Peak Name: "MSLAB-Nvl(IS)" Mass(es): "288.150/171.010 Da"
Comment: "" Annotation: ""

Sample Index: 49
Sample Type: Unknown
Concentration: 1.00 ng/mL
Calculated Conc: N/A
Acq. Date: 2025-05-16
Acq. Time: 03:09:20

Modified: Yes
Proc. Algorithm: Specify Parameters - MQ III

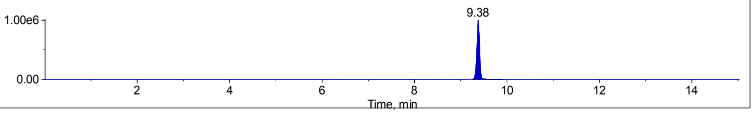

Sample Name: "ZY-24" Sample ID: "" File: "20250515-50AA.wiff"
Peak Name: "Val" Mass(es): "288.150/171.010 Da"
Comment: "" Annotation: ""

Sample Index: 49
Sample Type: Unknown
Concentration: N/A
Calculated Conc: 1.97 umol/L
Acq. Date: 2025-05-16
Acq. Time: 03:09:20
Modified: Yes
Proc. Algorithm: Specify Parameters - MQ III

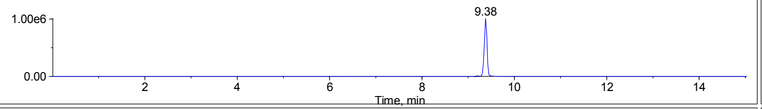

Sample Name: "ZY-24" Sample ID: "" File: "20250515-50AA.wiff"
Peak Name: "MSLAB-Nvl(IS)" Mass(es): "288.150/171.010 Da"
Comment: "" Annotation: ""

Sample Index: 49
Sample Type: Unknown
Concentration: 1.00 ng/mL
Calculated Conc: N/A
Acq. Date: 2025-05-16
Acq. Time: 03:09:20
Modified: Yes
Proc. Algorithm: Specify Parameters - MQ III

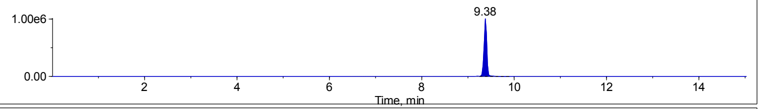

Sample Name: "ZY-24" Sample ID: "" File: "20250515-50AA.wiff"
Peak Name: "Thr" Mass(es): "290.100/171.010 Da"
Comment: "" Annotation: ""

Sample Index: 49
Sample Type: Unknown
Concentration: N/A
Calculated Conc: 1.24 umol/L
Acq. Date: 2025-05-16
Acq. Time: 03:09:20
Modified: Yes
Proc. Algorithm: Specify Parameters - MQ III

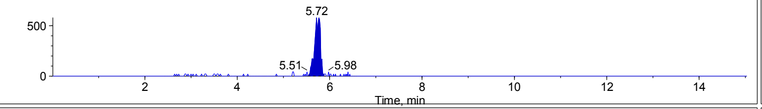

Sample Name: "ZY-24" Sample ID: "" File: "20250515-50AA.wiff"
Peak Name: "MSLAB-Nvl(IS)" Mass(es): "288.150/171.010 Da"
Comment: "" Annotation: ""

Sample Index: 49
Sample Type: Unknown
Concentration: 1.00 ng/mL
Calculated Conc: N/A
Acq. Date: 2025-05-16
Acq. Time: 03:09:20
Modified: Yes
Proc. Algorithm: Specify Parameters - MQ III

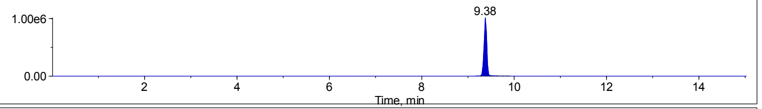

Sample Name: "ZY-24" Sample ID: "" File: "20250515-50AA.wiff"
Peak Name: "Cys1/2" Mass(es): "292.200/171.010 Da"
Comment: "" Annotation: ""

Sample Index: 49
Sample Type: Unknown
Concentration: N/A
Calculated Conc: 0.347 umol/L
Acq. Date: 2025-05-16
Acq. Time: 03:09:20
Modified: Yes
Proc. Algorithm: Specify Parameters - MQ III

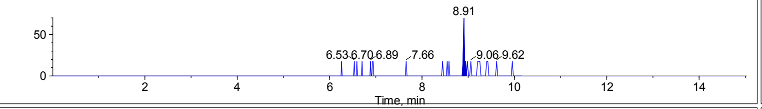

Sample Name: "ZY-24" Sample ID: "" File: "20250515-50AA.wiff"
Peak Name: "MSLAB-Nvl(IS)" Mass(es): "288.150/171.010 Da"
Comment: "" Annotation: ""

Sample Index: 49
Sample Type: Unknown
Concentration: 1.00 ng/mL
Calculated Conc: N/A
Acq. Date: 2025-05-16
Acq. Time: 03:09:20
Modified: Yes
Proc. Algorithm: Specify Parameters - MQ III

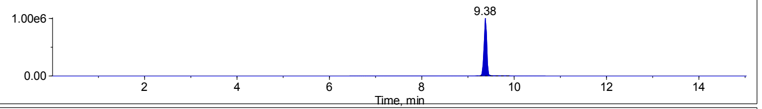

Sample Name: "ZY-24" Sample ID: "" File: "20250515-50AA.wiff"
Peak Name: "Ile" Mass(es): "302.150/171.010 Da"
Comment: "" Annotation: ""

Sample Index: 49
Sample Type: Unknown
Concentration: N/A
Calculated Conc: 0.267 umol/L
Acq. Date: 2025-05-16
Acq. Time: 03:09:20
Modified: Yes
Proc. Algorithm: Specify Parameters - MQ III

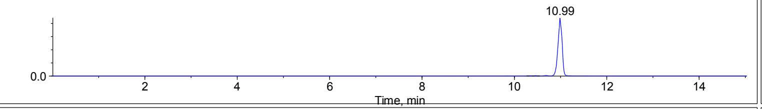

Sample Name: "ZY-24" Sample ID: "" File: "20250515-50AA.wiff"
Peak Name: "MSLAB-Nvl(IS)" Mass(es): "288.150/171.010 Da"
Comment: "" Annotation: ""

Sample Index: 49
Sample Type: Unknown
Concentration: 1.00 ng/mL
Calculated Conc: N/A
Acq. Date: 2025-05-16
Acq. Time: 03:09:20
Modified: Yes
Proc. Algorithm: Specify Parameters - MQ III

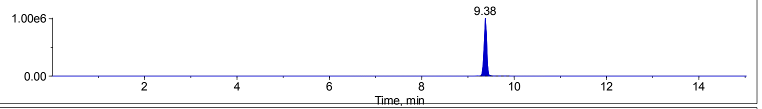

Sample Name: "ZY-24" Sample ID: "" File: "20250515-50AA.wiff"
Peak Name: "Asn" Mass(es): "303.130/171.010 Da"
Comment: "" Annotation: ""

Sample Index: 49
Sample Type: Unknown
Concentration: N/A
Calculated Conc: < 0
Acq. Date: 2025-05-16
Acq. Time: 03:09:20
Modified: Yes
Proc. Algorithm: Specify Parameters - MQ III

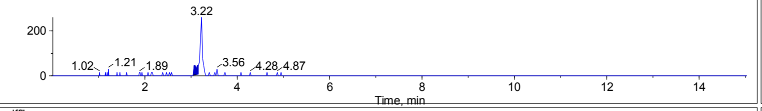

Sample Name: "ZY-24" Sample ID: "" File: "20250515-50AA.wiff"
Peak Name: "MSLAB-Nvl(IS)" Mass(es): "288.150/171.010 Da"
Comment: "" Annotation: ""

Sample Index: 49
Sample Type: Unknown
Concentration: 1.00 ng/mL
Calculated Conc: N/A
Acq. Date: 2025-05-16
Acq. Time: 03:09:20
Modified: Yes
Proc. Algorithm: Specify Parameters - MQ III

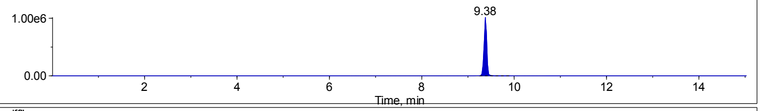

Sample Name: "ZY-24" Sample ID: "" File: "20250515-50AA.wiff"
Peak Name: "Asp" Mass(es): "304.110/171.010 Da"
Comment: "" Annotation: ""

Sample Index: 49
Sample Type: Unknown
Concentration: N/A
Calculated Conc: No Intercept
Acq. Date: 2025-05-16
Acq. Time: 03:09:20
Modified: Yes
Proc. Algorithm: Specify Parameters - MQ III

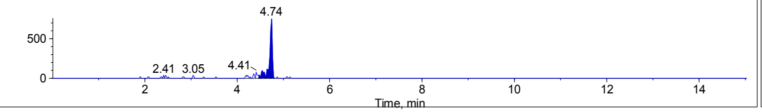

Sample Name: "ZY-24" Sample ID: "" File: "20250515-50AA.wiff"
Peak Name: "MSLAB-Nvl(IS)" Mass(es): "288.150/171.010 Da"
Comment: "" Annotation: ""

Sample Index: 49
Sample Type: Unknown
Concentration: 1.00 ng/mL
Calculated Conc: N/A
Acq. Date: 2025-05-16
Acq. Time: 03:09:20
Modified: Yes
Proc. Algorithm: Specify Parameters - MQ III

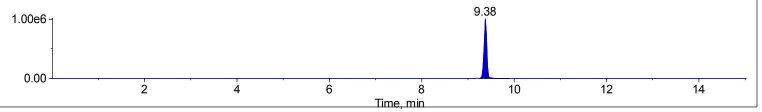

Sample Name: "ZY-24" Sample ID: "" File: "20250515-50AA.wiff"
Peak Name: "Gln" Mass(es): "317.150/171.010 Da"
Comment: "" Annotation: ""

```
Sample Index:      49
Sample Type:       Unknown
Concentration:        N/A
Calculated Conc: No Intercept
Acq. Date:         2025-05-16
Acq. Time:         03:09:20

Modified:              Yes
Proc. Algorithm: Specify Parameters - MQ III
```

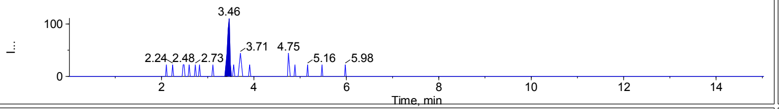

Sample Name: "ZY-24" Sample ID: "" File: "20250515-50AA.wiff"
Peak Name: "MSLAB-Nvl(IS)" Mass(es): "288.150/171.010 Da"
Comment: "" Annotation: ""

```
Sample Index:      49
Sample Type:       Unknown
Concentration:       1.00    ng/mL
Calculated Conc:      N/A
Acq. Date:         2025-05-16
Acq. Time:         03:09:20

Modified:              Yes
Proc. Algorithm: Specify Parameters - MQ III
```

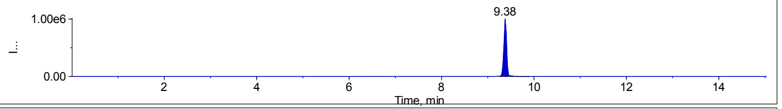

Sample Name: "ZY-24" Sample ID: "" File: "20250515-50AA.wiff"
Peak Name: "Glu" Mass(es): "318.100/171.010 Da"
Comment: "" Annotation: ""

```
Sample Index:      49
Sample Type:       Unknown
Concentration:        N/A
Calculated Conc:      1.59    umol/L
Acq. Date:         2025-05-16
Acq. Time:         03:09:20

Modified:              Yes
Proc. Algorithm: Specify Parameters - MQ III
```

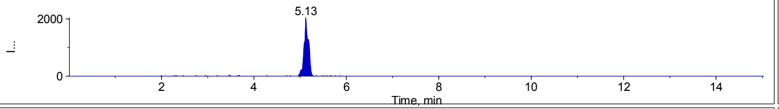

Sample Name: "ZY-24" Sample ID: "" File: "20250515-50AA.wiff"
Peak Name: "MSLAB-Nvl(IS)" Mass(es): "288.150/171.010 Da"
Comment: "" Annotation: ""

```
Sample Index:      49
Sample Type:       Unknown
Concentration:       1.00    ng/mL
Calculated Conc:      N/A
Acq. Date:         2025-05-16
Acq. Time:         03:09:20

Modified:              Yes
Proc. Algorithm: Specify Parameters - MQ III
```

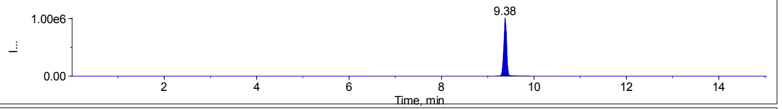

Sample Name: "ZY-24" Sample ID: "" File: "20250515-50AA.wiff"
Peak Name: "Met" Mass(es): "320.210/171.010 Da"
Comment: "" Annotation: ""

```
Sample Index:      49
Sample Type:       Unknown
Concentration:        N/A
Calculated Conc:       < 0
Acq. Date:         2025-05-16
Acq. Time:         03:09:20

Modified:              Yes
Proc. Algorithm: Specify Parameters - MQ III
```

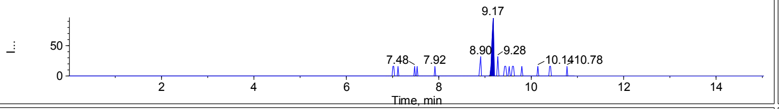

Sample Name: "ZY-24" Sample ID: "" File: "20250515-50AA.wiff"
Peak Name: "MSLAB-Nvl(IS)" Mass(es): "288.150/171.010 Da"
Comment: "" Annotation: ""

```
Sample Index:      49
Sample Type:       Unknown
Concentration:       1.00    ng/mL
Calculated Conc:      N/A
Acq. Date:         2025-05-16
Acq. Time:         03:09:20

Modified:              Yes
Proc. Algorithm: Specify Parameters - MQ III
```

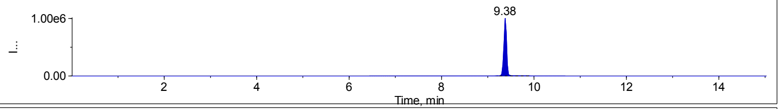

Sample Name: "ZY-24" Sample ID: "" File: "20250515-50AA.wiff"
Peak Name: "His" Mass(es): "326.150/171.010 Da"
Comment: "" Annotation: ""

```
Sample Index:      49
Sample Type:       Unknown
Concentration:        N/A
Calculated Conc:     0.998    umol/L
Acq. Date:         2025-05-16
Acq. Time:         03:09:20

Modified:              Yes
Proc. Algorithm: Specify Parameters - MQ III
```

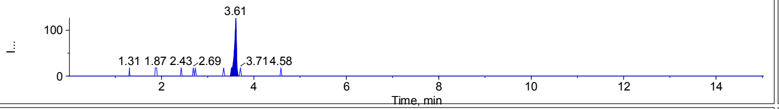

Sample Name: "ZY-24" Sample ID: "" File: "20250515-50AA.wiff"
Peak Name: "MSLAB-Nvl(IS)" Mass(es): "288.150/171.010 Da"
Comment: "" Annotation: ""

```
Sample Index:      49
Sample Type:       Unknown
Concentration:       1.00    ng/mL
Calculated Conc:      N/A
Acq. Date:         2025-05-16
Acq. Time:         03:09:20

Modified:              Yes
Proc. Algorithm: Specify Parameters - MQ III
```

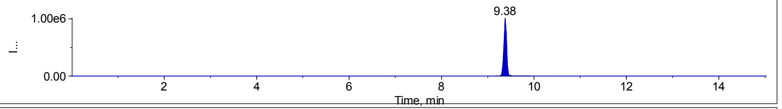

Sample Name: "ZY-24" Sample ID: "" File: "20250515-50AA.wiff"
Peak Name: "Phe" Mass(es): "336.200/171.010 Da"
Comment: "" Annotation: ""

```
Sample Index:      49
Sample Type:       Unknown
Concentration:        N/A
Calculated Conc:     0.446    umol/L
Acq. Date:         2025-05-16
Acq. Time:         03:09:20

Modified:              Yes
Proc. Algorithm: Specify Parameters - MQ III
```

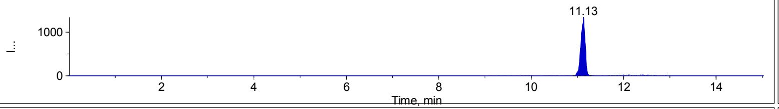

Sample Name: "ZY-24" Sample ID: "" File: "20250515-50AA.wiff"
Peak Name: "MSLAB-Nvl(IS)" Mass(es): "288.150/171.010 Da"
Comment: "" Annotation: ""

```
Sample Index:      49
Sample Type:       Unknown
Concentration:       1.00    ng/mL
Calculated Conc:      N/A
Acq. Date:         2025-05-16
Acq. Time:         03:09:20

Modified:              Yes
Proc. Algorithm: Specify Parameters - MQ III
```

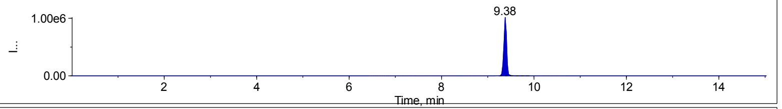

Sample Name: "ZY-24" Sample ID: "" File: "20250515-50AA.wiff"
Peak Name: "Arg" Mass(es): "345.210/171.010 Da"
Comment: "" Annotation: ""

```
Sample Index:      49
Sample Type:       Unknown
Concentration:        N/A
Calculated Conc:      2.13    umol/L
Acq. Date:         2025-05-16
Acq. Time:         03:09:20

Modified:              Yes
Proc. Algorithm: Specify Parameters - MQ III
```

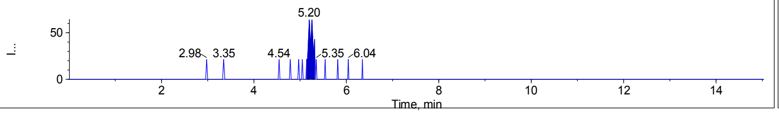

Sample Name: "ZY-24" Sample ID: "" File: "20250515-50AA.wiff"
Peak Name: "MSLAB-Nvl(IS)" Mass(es): "288.150/171.010 Da"
Comment: "" Annotation: ""

```
Sample Index:      49
Sample Type:       Unknown
Concentration:       1.00    ng/mL
Calculated Conc:      N/A
Acq. Date:         2025-05-16
Acq. Time:         03:09:20

Modified:              Yes
Proc. Algorithm: Specify Parameters - MQ III
```

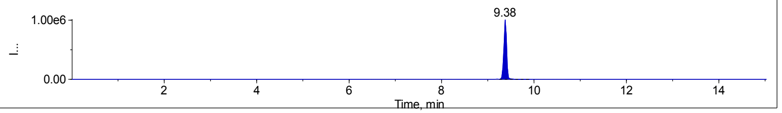

Sample Name: "ZY-24" Sample ID: "" File: "20250515-50AA.wiff"
Peak Name: "Trp" Mass(es): "375.200/171.010 Da"
Comment: "" Annotation: ""

Sample Index: 49
Sample Type: Unknown
Concentration: N/A
Calculated Conc: < 0
Acq. Date: 2025-05-16
Acq. Time: 03:09:20

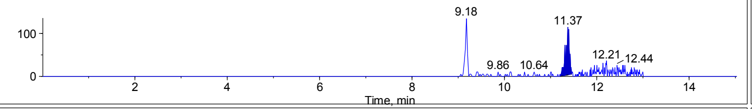

Modified: Yes
Proc. Algorithm: Specify Parameters - MQ III

Sample Name: "ZY-24" Sample ID: "" File: "20250515-50AA.wiff"
Peak Name: "Lys" Mass(es): "487.200/171.010 Da"
Comment: "" Annotation: ""

Sample Index: 49
Sample Type: Unknown
Concentration: N/A
Calculated Conc: 1.46 umol/L
Acq. Date: 2025-05-16
Acq. Time: 03:09:20

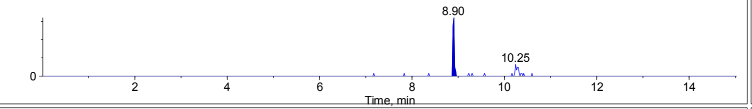

Modified: Yes
Proc. Algorithm: Specify Parameters - MQ III

Sample Name: "ZY-24" Sample ID: "" File: "20250515-50AA.wiff"
Peak Name: "tyr" Mass(es): "352.200/171.010 Da"
Comment: "" Annotation: ""

Sample Index: 49
Sample Type: Unknown
Concentration: N/A
Calculated Conc: No Intercept
Acq. Date: 2025-05-16
Acq. Time: 03:09:20

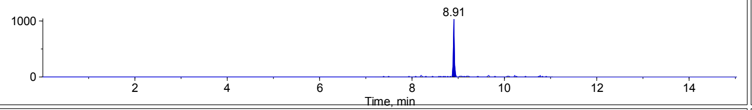

Modified: Yes
Proc. Algorithm: Specify Parameters - MQ III

Sample Name: "ZY-24" Sample ID: "" File: "20250515-50AA.wiff"
Peak Name: "Leu" Mass(es): "302.150/171.010 Da"
Comment: "" Annotation: ""

Sample Index: 49
Sample Type: Unknown
Concentration: N/A
Calculated Conc: 0.795 umol/L
Acq. Date: 2025-05-16
Acq. Time: 03:09:20

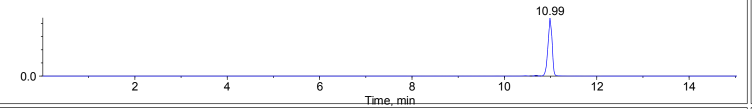

Modified: Yes
Proc. Algorithm: Specify Parameters - MQ III

Sample Name: "ZY-25" Sample ID: "" File: "20250515-50AA.wiff"
Peak Name: "Gly" Mass(es): "246.080/171.010 Da"
Comment: "" Annotation: ""

Sample Index: 50
Sample Type: Unknown
Concentration: N/A
Calculated Conc: 2.53 umol/L
Acq. Date: 2025-05-16
Acq. Time: 03:24:48

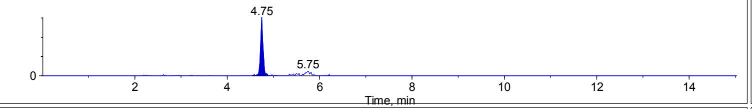

Modified: Yes
Proc. Algorithm: Specify Parameters - MQ III

Sample Name: "ZY-25" Sample ID: "" File: "20250515-50AA.wiff"
Peak Name: "Ala" Mass(es): "260.100/171.010 Da"
Comment: "" Annotation: ""

Sample Index: 50
Sample Type: Unknown
Concentration: N/A
Calculated Conc: No Intercept
Acq. Date: 2025-05-16
Acq. Time: 03:24:48

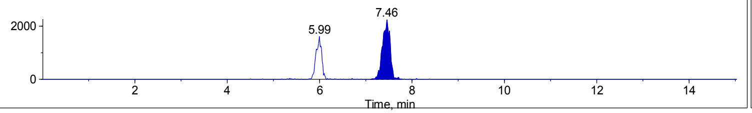

Modified: Yes
Proc. Algorithm: Specify Parameters - MQ III

Sample Name: "ZY-24" Sample ID: "" File: "20250515-50AA.wiff"
Peak Name: "MSLAB-Nvl(IS)" Mass(es): "288.150/171.010 Da"
Comment: "" Annotation: ""

Sample Index: 49
Sample Type: Unknown
Concentration: 1.00 ng/mL
Calculated Conc: N/A
Acq. Date: 2025-05-16
Acq. Time: 03:09:20

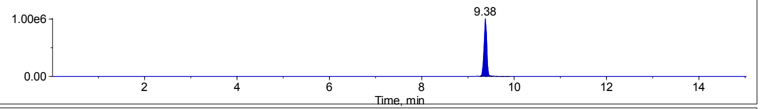

Modified: Yes
Proc. Algorithm: Specify Parameters - MQ III

Sample Name: "ZY-24" Sample ID: "" File: "20250515-50AA.wiff"
Peak Name: "MSLAB-Nvl(IS)" Mass(es): "288.150/171.010 Da"
Comment: "" Annotation: ""

Sample Index: 49
Sample Type: Unknown
Concentration: 1.00 ng/mL
Calculated Conc: N/A
Acq. Date: 2025-05-16
Acq. Time: 03:09:20

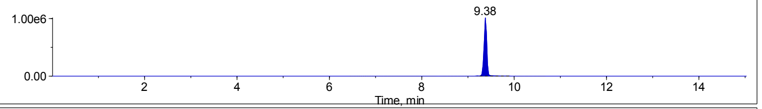

Modified: Yes
Proc. Algorithm: Specify Parameters - MQ III

Sample Name: "ZY-24" Sample ID: "" File: "20250515-50AA.wiff"
Peak Name: "MSLAB-Nvl(IS)" Mass(es): "288.150/171.010 Da"
Comment: "" Annotation: ""

Sample Index: 49
Sample Type: Unknown
Concentration: 1.00 ng/mL
Calculated Conc: N/A
Acq. Date: 2025-05-16
Acq. Time: 03:09:20

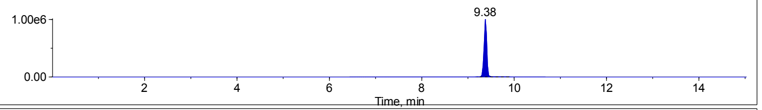

Modified: Yes
Proc. Algorithm: Specify Parameters - MQ III

Sample Name: "ZY-24" Sample ID: "" File: "20250515-50AA.wiff"
Peak Name: "MSLAB-Nvl(IS)" Mass(es): "288.150/171.010 Da"
Comment: "" Annotation: ""

Sample Index: 49
Sample Type: Unknown
Concentration: 1.00 ng/mL
Calculated Conc: N/A
Acq. Date: 2025-05-16
Acq. Time: 03:09:20

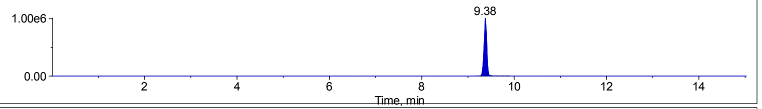

Modified: Yes
Proc. Algorithm: Specify Parameters - MQ III

Sample Name: "ZY-25" Sample ID: "" File: "20250515-50AA.wiff"
Peak Name: "MSLAB-Nvl(IS)" Mass(es): "288.150/171.010 Da"
Comment: "" Annotation: ""

Sample Index: 50
Sample Type: Unknown
Concentration: 1.00 ng/mL
Calculated Conc: N/A
Acq. Date: 2025-05-16
Acq. Time: 03:24:48

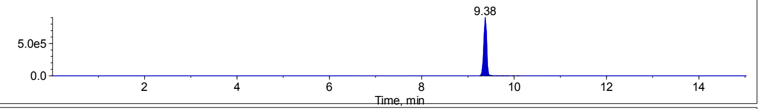

Modified: Yes
Proc. Algorithm: Specify Parameters - MQ III

Sample Name: "ZY-25" Sample ID: "" File: "20250515-50AA.wiff"
Peak Name: "MSLAB-Nvl(IS)" Mass(es): "288.150/171.010 Da"
Comment: "" Annotation: ""

Sample Index: 50
Sample Type: Unknown
Concentration: 1.00 ng/mL
Calculated Conc: N/A
Acq. Date: 2025-05-16
Acq. Time: 03:24:48

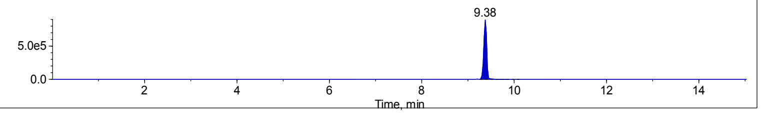

Modified: Yes
Proc. Algorithm: Specify Parameters - MQ III

Sample Name: "ZY-25" Sample ID: "" File: "20250515-50AA.wiff"
Peak Name: "Ser" Mass(es): "276.100/171.010 Da"
Comment: "" Annotation: ""
Sample Index: 50
Sample Type: Unknown
Concentration: N/A
Calculated Conc: 1.17 umol/L
Acq. Date: 2025-05-16
Acq. Time: 03:24:48
Modified: Yes
Proc. Algorithm: Specify Parameters - MQ III

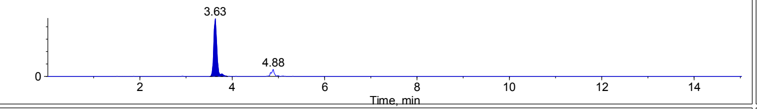

Sample Name: "ZY-25" Sample ID: "" File: "20250515-50AA.wiff"
Peak Name: "MSLAB-Nvl(IS)" Mass(es): "288.150/171.010 Da"
Comment: "" Annotation: ""
Sample Index: 50
Sample Type: Unknown
Concentration: 1.00 ng/mL
Calculated Conc: N/A
Acq. Date: 2025-05-16
Acq. Time: 03:24:48
Modified: Yes
Proc. Algorithm: Specify Parameters - MQ III

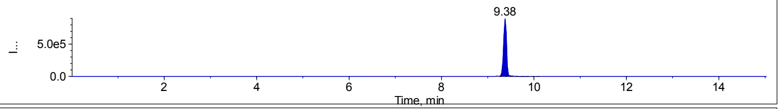

Sample Name: "ZY-25" Sample ID: "" File: "20250515-50AA.wiff"
Peak Name: "Pro" Mass(es): "286.150/171.010 Da"
Comment: "" Annotation: ""
Sample Index: 50
Sample Type: Unknown
Concentration: N/A
Calculated Conc: No Intercept
Acq. Date: 2025-05-16
Acq. Time: 03:24:48
Modified: Yes
Proc. Algorithm: Specify Parameters - MQ III

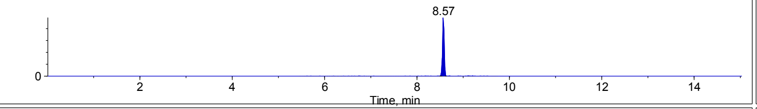

Sample Name: "ZY-25" Sample ID: "" File: "20250515-50AA.wiff"
Peak Name: "MSLAB-Nvl(IS)" Mass(es): "288.150/171.010 Da"
Comment: "" Annotation: ""
Sample Index: 50
Sample Type: Unknown
Concentration: 1.00 ng/mL
Calculated Conc: N/A
Acq. Date: 2025-05-16
Acq. Time: 03:24:48
Modified: Yes
Proc. Algorithm: Specify Parameters - MQ III

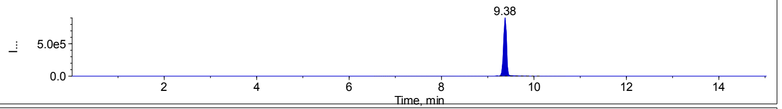

Sample Name: "ZY-25" Sample ID: "" File: "20250515-50AA.wiff"
Peak Name: "Val" Mass(es): "288.150/171.010 Da"
Comment: "" Annotation: ""
Sample Index: 50
Sample Type: Unknown
Concentration: N/A
Calculated Conc: 2.07 umol/L
Acq. Date: 2025-05-16
Acq. Time: 03:24:48
Modified: Yes
Proc. Algorithm: Specify Parameters - MQ III

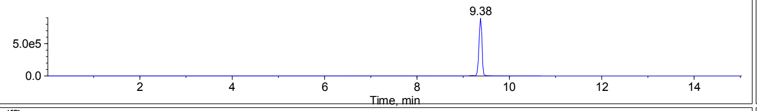

Sample Name: "ZY-25" Sample ID: "" File: "20250515-50AA.wiff"
Peak Name: "MSLAB-Nvl(IS)" Mass(es): "288.150/171.010 Da"
Comment: "" Annotation: ""
Sample Index: 50
Sample Type: Unknown
Concentration: 1.00 ng/mL
Calculated Conc: N/A
Acq. Date: 2025-05-16
Acq. Time: 03:24:48
Modified: Yes
Proc. Algorithm: Specify Parameters - MQ III

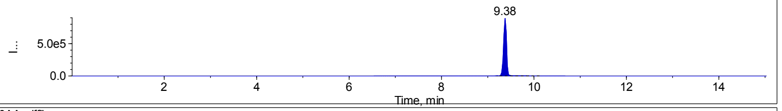

Sample Name: "ZY-25" Sample ID: "" File: "20250515-50AA.wiff"
Peak Name: "Thr" Mass(es): "290.100/171.010 Da"
Comment: "" Annotation: ""
Sample Index: 50
Sample Type: Unknown
Concentration: N/A
Calculated Conc: 1.34 umol/L
Acq. Date: 2025-05-16
Acq. Time: 03:24:48
Modified: Yes
Proc. Algorithm: Specify Parameters - MQ III

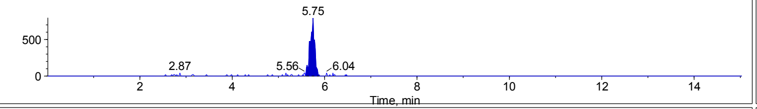

Sample Name: "ZY-25" Sample ID: "" File: "20250515-50AA.wiff"
Peak Name: "MSLAB-Nvl(IS)" Mass(es): "288.150/171.010 Da"
Comment: "" Annotation: ""
Sample Index: 50
Sample Type: Unknown
Concentration: 1.00 ng/mL
Calculated Conc: N/A
Acq. Date: 2025-05-16
Acq. Time: 03:24:48
Modified: Yes
Proc. Algorithm: Specify Parameters - MQ III

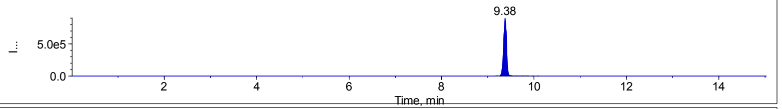

Sample Name: "ZY-25" Sample ID: "" File: "20250515-50AA.wiff"
Peak Name: "Cys1/2" Mass(es): "292.200/171.010 Da"
Comment: "" Annotation: ""
Sample Index: 50
Sample Type: Unknown
Concentration: N/A
Calculated Conc: 0.00989 umol/L
Acq. Date: 2025-05-16
Acq. Time: 03:24:48
Modified: Yes
Proc. Algorithm: Specify Parameters - MQ III

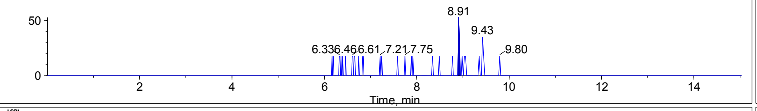

Sample Name: "ZY-25" Sample ID: "" File: "20250515-50AA.wiff"
Peak Name: "MSLAB-Nvl(IS)" Mass(es): "288.150/171.010 Da"
Comment: "" Annotation: ""
Sample Index: 50
Sample Type: Unknown
Concentration: 1.00 ng/mL
Calculated Conc: N/A
Acq. Date: 2025-05-16
Acq. Time: 03:24:48
Modified: Yes
Proc. Algorithm: Specify Parameters - MQ III

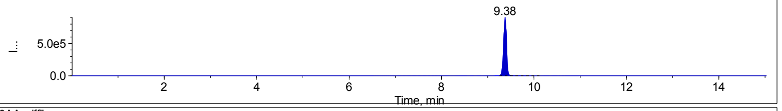

Sample Name: "ZY-25" Sample ID: "" File: "20250515-50AA.wiff"
Peak Name: "Ile" Mass(es): "302.150/171.010 Da"
Comment: "" Annotation: ""
Sample Index: 50
Sample Type: Unknown
Concentration: N/A
Calculated Conc: 0.353 umol/L
Acq. Date: 2025-05-16
Acq. Time: 03:24:48
Modified: Yes
Proc. Algorithm: Specify Parameters - MQ III

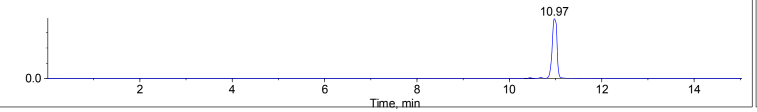

Sample Name: "ZY-25" Sample ID: "" File: "20250515-50AA.wiff"
Peak Name: "MSLAB-Nvl(IS)" Mass(es): "288.150/171.010 Da"
Comment: "" Annotation: ""
Sample Index: 50
Sample Type: Unknown
Concentration: 1.00 ng/mL
Calculated Conc: N/A
Acq. Date: 2025-05-16
Acq. Time: 03:24:48
Modified: Yes
Proc. Algorithm: Specify Parameters - MQ III

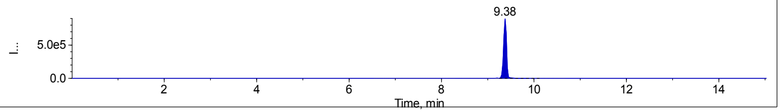

Sample Name: "ZY-25" Sample ID: "" File: "20250515-50AA.wiff"
Peak Name: "Asn" Mass(es): "303.130/171.010 Da"
Comment: "" Annotation: ""

```
Sample Index:       50
Sample Type:        Unknown
Concentration:      N/A
Calculated Conc:    < 0
Acq. Date:          2025-05-16
Acq. Time:          03:24:48

Modified:           Yes
Proc. Algorithm: Specify Parameters - MQ III
```

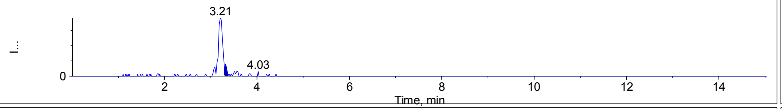

Sample Name: "ZY-25" Sample ID: "" File: "20250515-50AA.wiff"
Peak Name: "MSLAB-Nvl(IS)" Mass(es): "288.150/171.010 Da"
Comment: "" Annotation: ""

```
Sample Index:       50
Sample Type:        Unknown
Concentration:      1.00    ng/mL
Calculated Conc:    N/A
Acq. Date:          2025-05-16
Acq. Time:          03:24:48

Modified:           Yes
Proc. Algorithm: Specify Parameters - MQ III
```

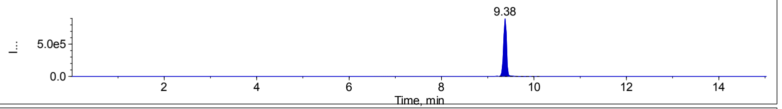

Sample Name: "ZY-25" Sample ID: "" File: "20250515-50AA.wiff"
Peak Name: "Asp" Mass(es): "304.110/171.010 Da"
Comment: "" Annotation: ""

```
Sample Index:       50
Sample Type:        Unknown
Concentration:      N/A
Calculated Conc: No Intercept
Acq. Date:          2025-05-16
Acq. Time:          03:24:48

Modified:           Yes
Proc. Algorithm: Specify Parameters - MQ III
```

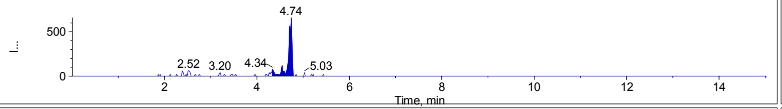

Sample Name: "ZY-25" Sample ID: "" File: "20250515-50AA.wiff"
Peak Name: "MSLAB-Nvl(IS)" Mass(es): "288.150/171.010 Da"
Comment: "" Annotation: ""

```
Sample Index:       50
Sample Type:        Unknown
Concentration:      1.00    ng/mL
Calculated Conc:    N/A
Acq. Date:          2025-05-16
Acq. Time:          03:24:48

Modified:           Yes
Proc. Algorithm: Specify Parameters - MQ III
```

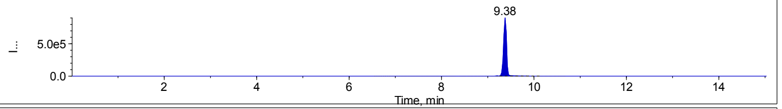

Sample Name: "ZY-25" Sample ID: "" File: "20250515-50AA.wiff"
Peak Name: "Gln" Mass(es): "317.150/171.010 Da"
Comment: "" Annotation: ""

```
Sample Index:       50
Sample Type:        Unknown
Concentration:      N/A
Calculated Conc:    < 0
Acq. Date:          2025-05-16
Acq. Time:          03:24:48

Modified:           Yes
Proc. Algorithm: Specify Parameters - MQ III
```

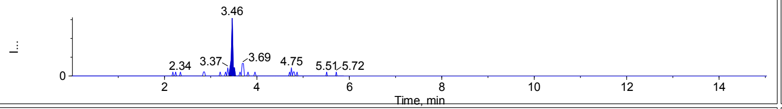

Sample Name: "ZY-25" Sample ID: "" File: "20250515-50AA.wiff"
Peak Name: "MSLAB-Nvl(IS)" Mass(es): "288.150/171.010 Da"
Comment: "" Annotation: ""

```
Sample Index:       50
Sample Type:        Unknown
Concentration:      1.00    ng/mL
Calculated Conc:    N/A
Acq. Date:          2025-05-16
Acq. Time:          03:24:48

Modified:           Yes
Proc. Algorithm: Specify Parameters - MQ III
```

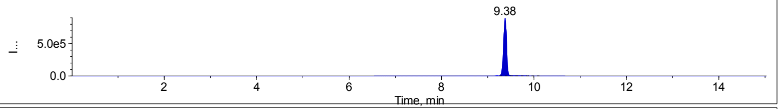

Sample Name: "ZY-25" Sample ID: "" File: "20250515-50AA.wiff"
Peak Name: "Glu" Mass(es): "318.100/171.010 Da"
Comment: "" Annotation: ""

```
Sample Index:       50
Sample Type:        Unknown
Concentration:      N/A
Calculated Conc:    1.71    umol/L
Acq. Date:          2025-05-16
Acq. Time:          03:24:48

Modified:           Yes
Proc. Algorithm: Specify Parameters - MQ III
```

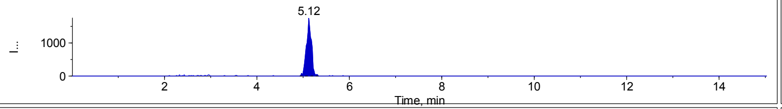

Sample Name: "ZY-25" Sample ID: "" File: "20250515-50AA.wiff"
Peak Name: "MSLAB-Nvl(IS)" Mass(es): "288.150/171.010 Da"
Comment: "" Annotation: ""

```
Sample Index:       50
Sample Type:        Unknown
Concentration:      1.00    ng/mL
Calculated Conc:    N/A
Acq. Date:          2025-05-16
Acq. Time:          03:24:48

Modified:           Yes
Proc. Algorithm: Specify Parameters - MQ III
```

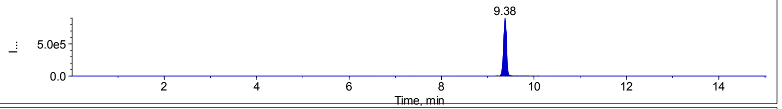

Sample Name: "ZY-25" Sample ID: "" File: "20250515-50AA.wiff"
Peak Name: "Met" Mass(es): "320.210/171.010 Da"
Comment: "" Annotation: ""

```
Sample Index:       50
Sample Type:        Unknown
Concentration:      N/A
Calculated Conc:    < 0
Acq. Date:          2025-05-16
Acq. Time:          03:24:48

Modified:           Yes
Proc. Algorithm: Specify Parameters - MQ III
```

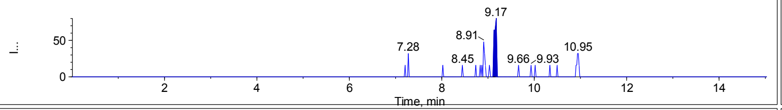

Sample Name: "ZY-25" Sample ID: "" File: "20250515-50AA.wiff"
Peak Name: "MSLAB-Nvl(IS)" Mass(es): "288.150/171.010 Da"
Comment: "" Annotation: ""

```
Sample Index:       50
Sample Type:        Unknown
Concentration:      1.00    ng/mL
Calculated Conc:    N/A
Acq. Date:          2025-05-16
Acq. Time:          03:24:48

Modified:           Yes
Proc. Algorithm: Specify Parameters - MQ III
```

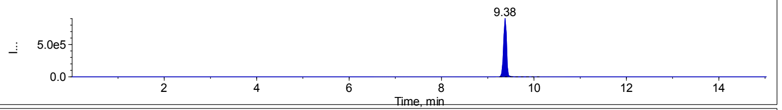

Sample Name: "ZY-25" Sample ID: "" File: "20250515-50AA.wiff"
Peak Name: "His" Mass(es): "326.150/171.010 Da"
Comment: "" Annotation: ""

```
Sample Index:       50
Sample Type:        Unknown
Concentration:      N/A
Calculated Conc:    0.700   umol/L
Acq. Date:          2025-05-16
Acq. Time:          03:24:48

Modified:           Yes
Proc. Algorithm: Specify Parameters - MQ III
```

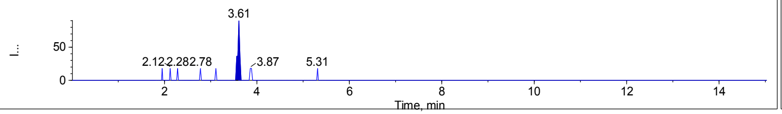

Sample Name: "ZY-25" Sample ID: "" File: "20250515-50AA.wiff"
Peak Name: "MSLAB-Nvl(IS)" Mass(es): "288.150/171.010 Da"
Comment: "" Annotation: ""

```
Sample Index:       50
Sample Type:        Unknown
Concentration:      1.00    ng/mL
Calculated Conc:    N/A
Acq. Date:          2025-05-16
Acq. Time:          03:24:48

Modified:           Yes
Proc. Algorithm: Specify Parameters - MQ III
```

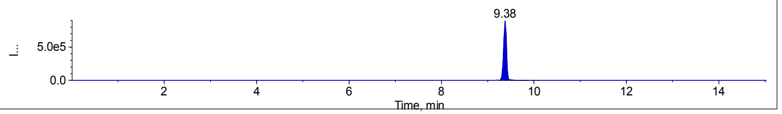

Sample Name: "ZY-25" Sample ID: "" File: "20250515-50AA.wiff"
Peak Name: "Phe" Mass(es): "336.200/171.010 Da"
Comment: "" Annotation: ""
Sample Index: 50
Sample Type: Unknown
Concentration: N/A
Calculated Conc: 0.484 umol/L
Acq. Date: 2025-05-16
Acq. Time: 03:24:48
Modified: Yes
Proc. Algorithm: Specify Parameters - MQ III

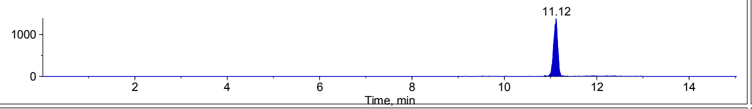

Sample Name: "ZY-25" Sample ID: "" File: "20250515-50AA.wiff"
Peak Name: "MSLAB-Nvl(IS)" Mass(es): "288.150/171.010 Da"
Comment: "" Annotation: ""
Sample Index: 50
Sample Type: Unknown
Concentration: 1.00 ng/mL
Calculated Conc: N/A
Acq. Date: 2025-05-16
Acq. Time: 03:24:48
Modified: Yes
Proc. Algorithm: Specify Parameters - MQ III

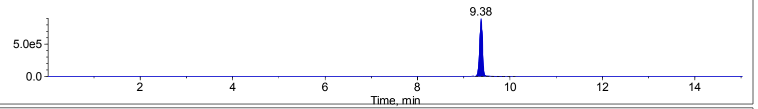

Sample Name: "ZY-25" Sample ID: "" File: "20250515-50AA.wiff"
Peak Name: "Arg" Mass(es): "345.210/171.010 Da"
Comment: "" Annotation: ""
Sample Index: 50
Sample Type: Unknown
Concentration: N/A
Calculated Conc: 2.02 umol/L
Acq. Date: 2025-05-16
Acq. Time: 03:24:48
Modified: Yes
Proc. Algorithm: Specify Parameters - MQ III

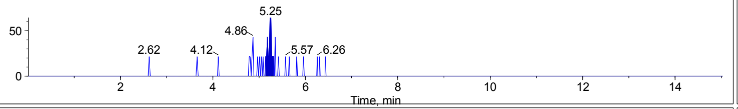

Sample Name: "ZY-25" Sample ID: "" File: "20250515-50AA.wiff"
Peak Name: "MSLAB-Nvl(IS)" Mass(es): "288.150/171.010 Da"
Comment: "" Annotation: ""
Sample Index: 50
Sample Type: Unknown
Concentration: 1.00 ng/mL
Calculated Conc: N/A
Acq. Date: 2025-05-16
Acq. Time: 03:24:48
Modified: Yes
Proc. Algorithm: Specify Parameters - MQ III

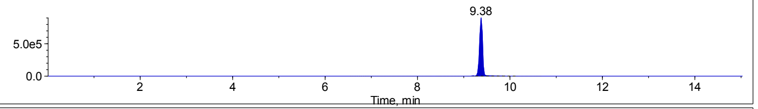

Sample Name: "ZY-25" Sample ID: "" File: "20250515-50AA.wiff"
Peak Name: "Trp" Mass(es): "375.200/171.010 Da"
Comment: "" Annotation: ""
Sample Index: 50
Sample Type: Unknown
Concentration: N/A
Calculated Conc: < 0
Acq. Date: 2025-05-16
Acq. Time: 03:24:48
Modified: Yes
Proc. Algorithm: Specify Parameters - MQ III

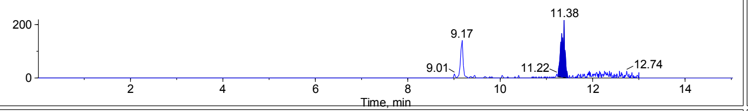

Sample Name: "ZY-25" Sample ID: "" File: "20250515-50AA.wiff"
Peak Name: "MSLAB-Nvl(IS)" Mass(es): "288.150/171.010 Da"
Comment: "" Annotation: ""
Sample Index: 50
Sample Type: Unknown
Concentration: 1.00 ng/mL
Calculated Conc: N/A
Acq. Date: 2025-05-16
Acq. Time: 03:24:48
Modified: Yes
Proc. Algorithm: Specify Parameters - MQ III

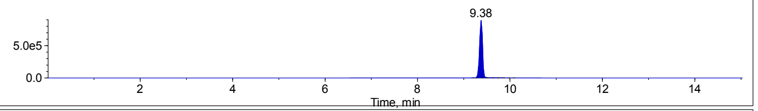

Sample Name: "ZY-25" Sample ID: "" File: "20250515-50AA.wiff"
Peak Name: "Lys" Mass(es): "487.200/171.010 Da"
Comment: "" Annotation: ""
Sample Index: 50
Sample Type: Unknown
Concentration: N/A
Calculated Conc: 1.71 umol/L
Acq. Date: 2025-05-16
Acq. Time: 03:24:48
Modified: Yes
Proc. Algorithm: Specify Parameters - MQ III

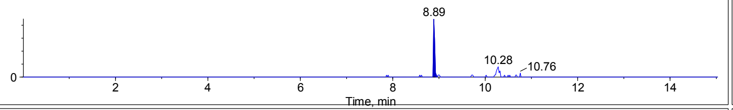

Sample Name: "ZY-25" Sample ID: "" File: "20250515-50AA.wiff"
Peak Name: "MSLAB-Nvl(IS)" Mass(es): "288.150/171.010 Da"
Comment: "" Annotation: ""
Sample Index: 50
Sample Type: Unknown
Concentration: 1.00 ng/mL
Calculated Conc: N/A
Acq. Date: 2025-05-16
Acq. Time: 03:24:48
Modified: Yes
Proc. Algorithm: Specify Parameters - MQ III

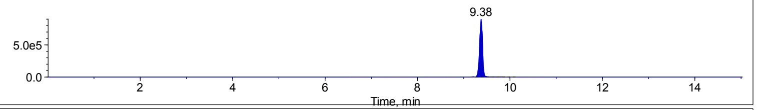

Sample Name: "ZY-25" Sample ID: "" File: "20250515-50AA.wiff"
Peak Name: "tyr" Mass(es): "352.200/171.010 Da"
Comment: "" Annotation: ""
Sample Index: 50
Sample Type: Unknown
Concentration: N/A
Calculated Conc: No Intercept
Acq. Date: 2025-05-16
Acq. Time: 03:24:48
Modified: Yes
Proc. Algorithm: Specify Parameters - MQ III

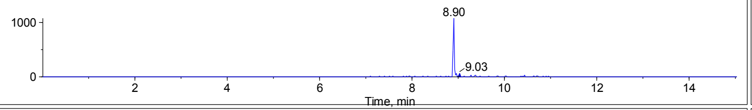

Sample Name: "ZY-25" Sample ID: "" File: "20250515-50AA.wiff"
Peak Name: "MSLAB-Nvl(IS)" Mass(es): "288.150/171.010 Da"
Comment: "" Annotation: ""
Sample Index: 50
Sample Type: Unknown
Concentration: 1.00 ng/mL
Calculated Conc: N/A
Acq. Date: 2025-05-16
Acq. Time: 03:24:48
Modified: Yes
Proc. Algorithm: Specify Parameters - MQ III

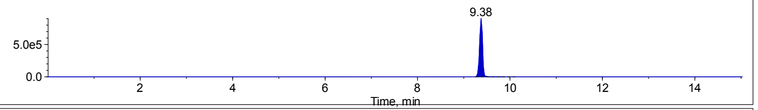

Sample Name: "ZY-25" Sample ID: "" File: "20250515-50AA.wiff"
Peak Name: "Leu" Mass(es): "302.150/171.010 Da"
Comment: "" Annotation: ""
Sample Index: 50
Sample Type: Unknown
Concentration: N/A
Calculated Conc: 0.805 umol/L
Acq. Date: 2025-05-16
Acq. Time: 03:24:48
Modified: Yes
Proc. Algorithm: Specify Parameters - MQ III

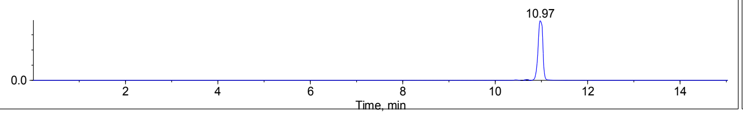

Sample Name: "ZY-25" Sample ID: "" File: "20250515-50AA.wiff"
Peak Name: "MSLAB-Nvl(IS)" Mass(es): "288.150/171.010 Da"
Comment: "" Annotation: ""
Sample Index: 50
Sample Type: Unknown
Concentration: 1.00 ng/mL
Calculated Conc: N/A
Acq. Date: 2025-05-16
Acq. Time: 03:24:48
Modified: Yes
Proc. Algorithm: Specify Parameters - MQ III

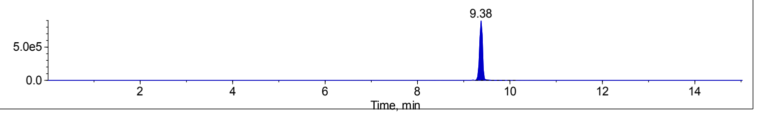

Sample Name: "ZY-26" Sample ID: "" File: "20250515-50AA.wiff"
Peak Name: "Gly" Mass(es): "246.080/171.010 Da"
Comment: "" Annotation: ""
Sample Index: 51
Sample Type: Unknown
Concentration: N/A
Calculated Conc: 1.57 umol/L
Acq. Date: 2025-05-16
Acq. Time: 03:40:16
Modified: Yes
Proc. Algorithm: Specify Parameters - MQ III

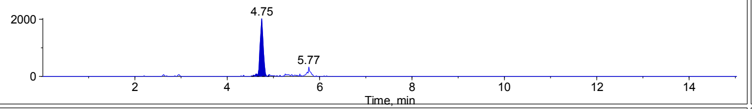

Sample Name: "ZY-26" Sample ID: "" File: "20250515-50AA.wiff"
Peak Name: "Ala" Mass(es): "260.100/171.010 Da"
Comment: "" Annotation: ""
Sample Index: 51
Sample Type: Unknown
Concentration: N/A
Calculated Conc: No Intercept
Acq. Date: 2025-05-16
Acq. Time: 03:40:16
Modified: Yes
Proc. Algorithm: Specify Parameters - MQ III

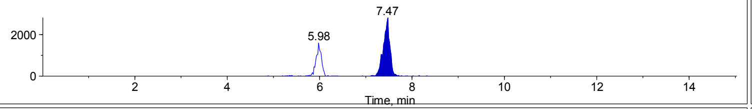

Sample Name: "ZY-26" Sample ID: "" File: "20250515-50AA.wiff"
Peak Name: "Ser" Mass(es): "276.100/171.010 Da"
Comment: "" Annotation: ""
Sample Index: 51
Sample Type: Unknown
Concentration: N/A
Calculated Conc: 0.0983 umol/L
Acq. Date: 2025-05-16
Acq. Time: 03:40:16
Modified: Yes
Proc. Algorithm: Specify Parameters - MQ III

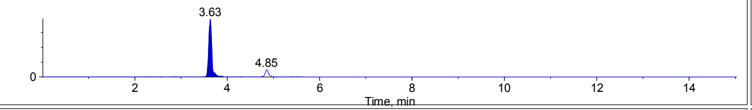

Sample Name: "ZY-26" Sample ID: "" File: "20250515-50AA.wiff"
Peak Name: "Pro" Mass(es): "286.150/171.010 Da"
Comment: "" Annotation: ""
Sample Index: 51
Sample Type: Unknown
Concentration: N/A
Calculated Conc: No Intercept
Acq. Date: 2025-05-16
Acq. Time: 03:40:16
Modified: Yes
Proc. Algorithm: Specify Parameters - MQ III

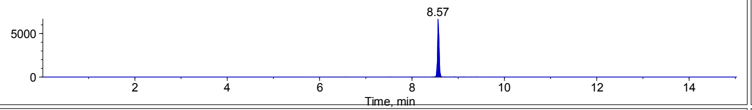

Sample Name: "ZY-26" Sample ID: "" File: "20250515-50AA.wiff"
Peak Name: "Val" Mass(es): "288.150/171.010 Da"
Comment: "" Annotation: ""
Sample Index: 51
Sample Type: Unknown
Concentration: N/A
Calculated Conc: 2.54 umol/L
Acq. Date: 2025-05-16
Acq. Time: 03:40:16
Modified: Yes
Proc. Algorithm: Specify Parameters - MQ III

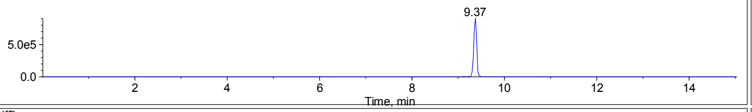

Sample Name: "ZY-26" Sample ID: "" File: "20250515-50AA.wiff"
Peak Name: "Thr" Mass(es): "290.100/171.010 Da"
Comment: "" Annotation: ""
Sample Index: 51
Sample Type: Unknown
Concentration: N/A
Calculated Conc: 1.64 umol/L
Acq. Date: 2025-05-16
Acq. Time: 03:40:16
Modified: Yes
Proc. Algorithm: Specify Parameters - MQ III

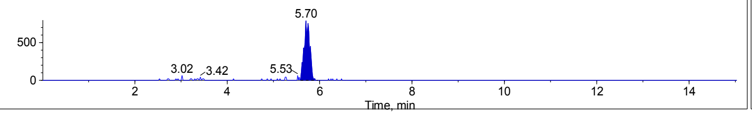

Sample Name: "ZY-26" Sample ID: "" File: "20250515-50AA.wiff"
Peak Name: "MSLAB-Nvl(IS)" Mass(es): "288.150/171.010 Da"
Comment: "" Annotation: ""
Sample Index: 51
Sample Type: Unknown
Concentration: 1.00 ng/mL
Calculated Conc: N/A
Acq. Date: 2025-05-16
Acq. Time: 03:40:16
Modified: Yes
Proc. Algorithm: Specify Parameters - MQ III

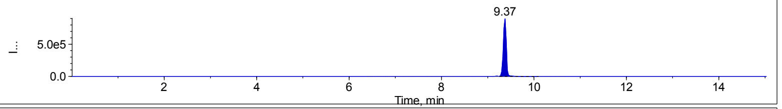

Sample Name: "ZY-26" Sample ID: "" File: "20250515-50AA.wiff"
Peak Name: "MSLAB-Nvl(IS)" Mass(es): "288.150/171.010 Da"
Comment: "" Annotation: ""
Sample Index: 51
Sample Type: Unknown
Concentration: 1.00 ng/mL
Calculated Conc: N/A
Acq. Date: 2025-05-16
Acq. Time: 03:40:16
Modified: Yes
Proc. Algorithm: Specify Parameters - MQ III

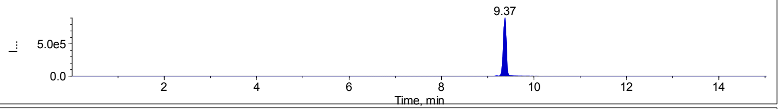

Sample Name: "ZY-26" Sample ID: "" File: "20250515-50AA.wiff"
Peak Name: "MSLAB-Nvl(IS)" Mass(es): "288.150/171.010 Da"
Comment: "" Annotation: ""
Sample Index: 51
Sample Type: Unknown
Concentration: 1.00 ng/mL
Calculated Conc: N/A
Acq. Date: 2025-05-16
Acq. Time: 03:40:16
Modified: Yes
Proc. Algorithm: Specify Parameters - MQ III

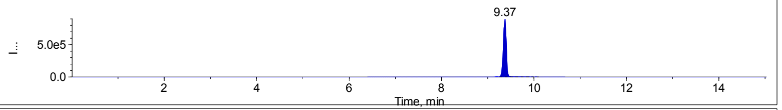

Sample Name: "ZY-26" Sample ID: "" File: "20250515-50AA.wiff"
Peak Name: "MSLAB-Nvl(IS)" Mass(es): "288.150/171.010 Da"
Comment: "" Annotation: ""
Sample Index: 51
Sample Type: Unknown
Concentration: 1.00 ng/mL
Calculated Conc: N/A
Acq. Date: 2025-05-16
Acq. Time: 03:40:16
Modified: Yes
Proc. Algorithm: Specify Parameters - MQ III

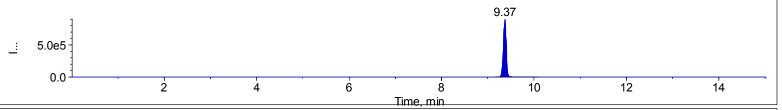

Sample Name: "ZY-26" Sample ID: "" File: "20250515-50AA.wiff"
Peak Name: "MSLAB-Nvl(IS)" Mass(es): "288.150/171.010 Da"
Comment: "" Annotation: ""
Sample Index: 51
Sample Type: Unknown
Concentration: 1.00 ng/mL
Calculated Conc: N/A
Acq. Date: 2025-05-16
Acq. Time: 03:40:16
Modified: Yes
Proc. Algorithm: Specify Parameters - MQ III

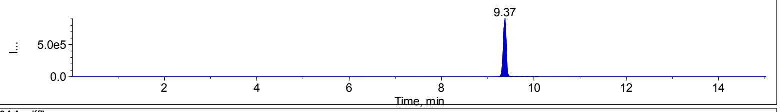

Sample Name: "ZY-26" Sample ID: "" File: "20250515-50AA.wiff"
Peak Name: "MSLAB-Nvl(IS)" Mass(es): "288.150/171.010 Da"
Comment: "" Annotation: ""
Sample Index: 51
Sample Type: Unknown
Concentration: 1.00 ng/mL
Calculated Conc: N/A
Acq. Date: 2025-05-16
Acq. Time: 03:40:16
Modified: Yes
Proc. Algorithm: Specify Parameters - MQ III

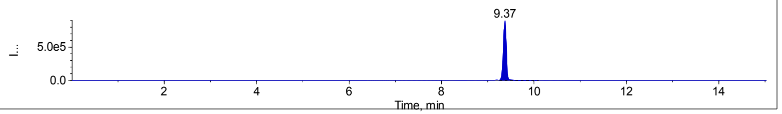

Sample Name: "ZY-26" Sample ID: "" File: "20250515-50AA.wiff"
Peak Name: "Cys1/2" Mass(es): "292.200/171.010 Da"
Comment: "" Annotation: ""

```
Sample Index:       51
Sample Type:        Unknown
Concentration:         N/A
Calculated Conc:     0.385    umol/L
Acq. Date:          2025-05-16
Acq. Time:          03:40:16

Modified:               Yes
Proc. Algorithm: Specify Parameters - MQ III
```

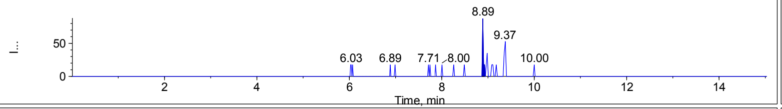

Sample Name: "ZY-26" Sample ID: "" File: "20250515-50AA.wiff"
Peak Name: "MSLAB-Nvl(IS)" Mass(es): "288.150/171.010 Da"
Comment: "" Annotation: ""

```
Sample Index:       51
Sample Type:        Unknown
Concentration:       1.00     ng/mL
Calculated Conc:     N/A
Acq. Date:          2025-05-16
Acq. Time:          03:40:16

Modified:               Yes
Proc. Algorithm: Specify Parameters - MQ III
```

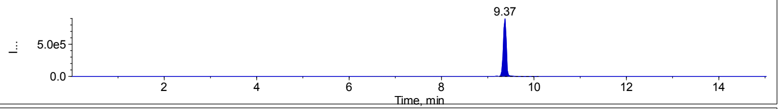

Sample Name: "ZY-26" Sample ID: "" File: "20250515-50AA.wiff"
Peak Name: "Ile" Mass(es): "302.150/171.010 Da"
Comment: "" Annotation: ""

```
Sample Index:       51
Sample Type:        Unknown
Concentration:         N/A
Calculated Conc:     0.640    umol/L
Acq. Date:          2025-05-16
Acq. Time:          03:40:16

Modified:               Yes
Proc. Algorithm: Specify Parameters - MQ III
```

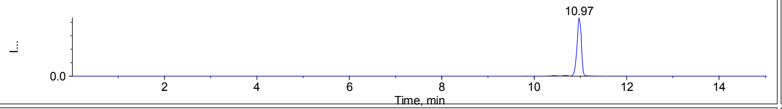

Sample Name: "ZY-26" Sample ID: "" File: "20250515-50AA.wiff"
Peak Name: "MSLAB-Nvl(IS)" Mass(es): "288.150/171.010 Da"
Comment: "" Annotation: ""

```
Sample Index:       51
Sample Type:        Unknown
Concentration:       1.00     ng/mL
Calculated Conc:     N/A
Acq. Date:          2025-05-16
Acq. Time:          03:40:16

Modified:               Yes
Proc. Algorithm: Specify Parameters - MQ III
```

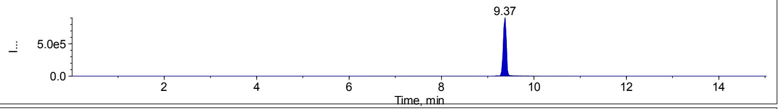

Sample Name: "ZY-26" Sample ID: "" File: "20250515-50AA.wiff"
Peak Name: "Asn" Mass(es): "303.130/171.010 Da"
Comment: "" Annotation: ""

```
Sample Index:       51
Sample Type:        Unknown
Concentration:         N/A
Calculated Conc:       < 0
Acq. Date:          2025-05-16
Acq. Time:          03:40:16

Modified:               Yes
Proc. Algorithm: Specify Parameters - MQ III
```

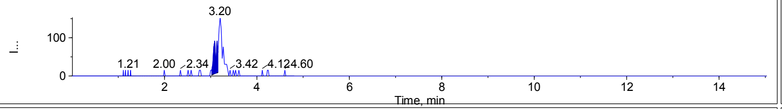

Sample Name: "ZY-26" Sample ID: "" File: "20250515-50AA.wiff"
Peak Name: "MSLAB-Nvl(IS)" Mass(es): "288.150/171.010 Da"
Comment: "" Annotation: ""

```
Sample Index:       51
Sample Type:        Unknown
Concentration:       1.00     ng/mL
Calculated Conc:     N/A
Acq. Date:          2025-05-16
Acq. Time:          03:40:16

Modified:               Yes
Proc. Algorithm: Specify Parameters - MQ III
```

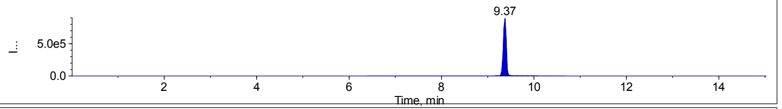

Sample Name: "ZY-26" Sample ID: "" File: "20250515-50AA.wiff"
Peak Name: "Asp" Mass(es): "304.110/171.010 Da"
Comment: "" Annotation: ""

```
Sample Index:       51
Sample Type:        Unknown
Concentration:         N/A
Calculated Conc:       < 0
Acq. Date:          2025-05-16
Acq. Time:          03:40:16

Modified:               Yes
Proc. Algorithm: Specify Parameters - MQ III
```

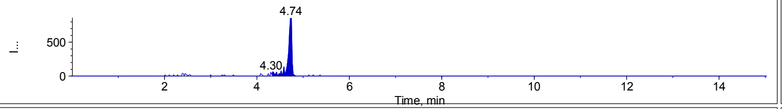

Sample Name: "ZY-26" Sample ID: "" File: "20250515-50AA.wiff"
Peak Name: "MSLAB-Nvl(IS)" Mass(es): "288.150/171.010 Da"
Comment: "" Annotation: ""

```
Sample Index:       51
Sample Type:        Unknown
Concentration:       1.00     ng/mL
Calculated Conc:     N/A
Acq. Date:          2025-05-16
Acq. Time:          03:40:16

Modified:               Yes
Proc. Algorithm: Specify Parameters - MQ III
```

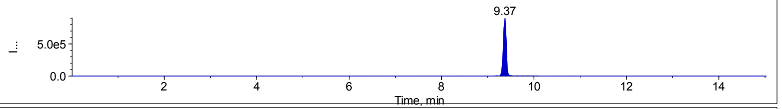

Sample Name: "ZY-26" Sample ID: "" File: "20250515-50AA.wiff"
Peak Name: "Gln" Mass(es): "317.150/171.010 Da"
Comment: "" Annotation: ""

```
Sample Index:       51
Sample Type:        Unknown
Concentration:         N/A
Calculated Conc: No Intercept
Acq. Date:          2025-05-16
Acq. Time:          03:40:16

Modified:               Yes
Proc. Algorithm: Specify Parameters - MQ III
```

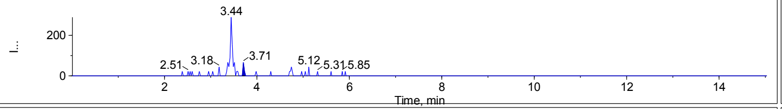

Sample Name: "ZY-26" Sample ID: "" File: "20250515-50AA.wiff"
Peak Name: "MSLAB-Nvl(IS)" Mass(es): "288.150/171.010 Da"
Comment: "" Annotation: ""

```
Sample Index:       51
Sample Type:        Unknown
Concentration:       1.00     ng/mL
Calculated Conc:     N/A
Acq. Date:          2025-05-16
Acq. Time:          03:40:16

Modified:               Yes
Proc. Algorithm: Specify Parameters - MQ III
```

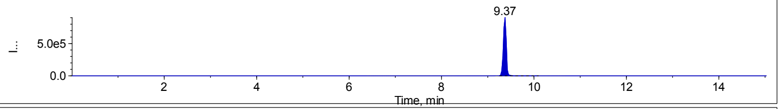

Sample Name: "ZY-26" Sample ID: "" File: "20250515-50AA.wiff"
Peak Name: "Glu" Mass(es): "318.100/171.010 Da"
Comment: "" Annotation: ""

```
Sample Index:       51
Sample Type:        Unknown
Concentration:         N/A
Calculated Conc:     2.98     umol/L
Acq. Date:          2025-05-16
Acq. Time:          03:40:16

Modified:               Yes
Proc. Algorithm: Specify Parameters - MQ III
```

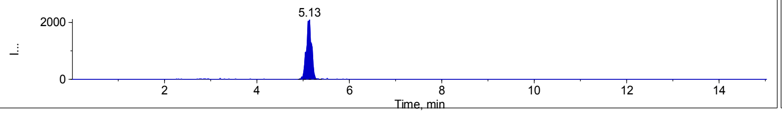

Sample Name: "ZY-26" Sample ID: "" File: "20250515-50AA.wiff"
Peak Name: "MSLAB-Nvl(IS)" Mass(es): "288.150/171.010 Da"
Comment: "" Annotation: ""

```
Sample Index:       51
Sample Type:        Unknown
Concentration:       1.00     ng/mL
Calculated Conc:     N/A
Acq. Date:          2025-05-16
Acq. Time:          03:40:16

Modified:               Yes
Proc. Algorithm: Specify Parameters - MQ III
```

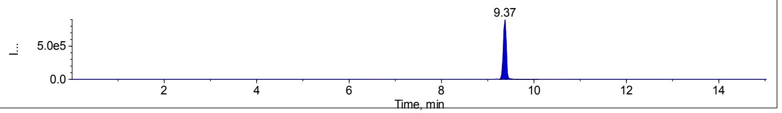

Sample Name: "ZY-26" Sample ID: "" File: "20250515-50AA.wiff"
Peak Name: "Met" Mass(es): "320.210/171.010 Da"
Comment: "" Annotation: ""

Sample Index: 51
Sample Type: Unknown
Concentration: N/A
Calculated Conc: < 0
Acq. Date: 2025-05-16
Acq. Time: 03:40:16
Modified: Yes
Proc. Algorithm: Specify Parameters - MQ III

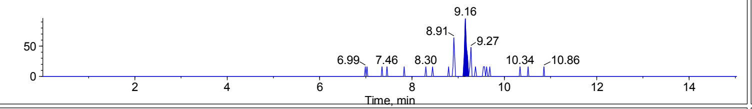

Sample Name: "ZY-26" Sample ID: "" File: "20250515-50AA.wiff"
Peak Name: "MSLAB-Nvl(IS)" Mass(es): "288.150/171.010 Da"
Comment: "" Annotation: ""

Sample Index: 51
Sample Type: Unknown
Concentration: 1.00 ng/mL
Calculated Conc: N/A
Acq. Date: 2025-05-16
Acq. Time: 03:40:16
Modified: Yes
Proc. Algorithm: Specify Parameters - MQ III

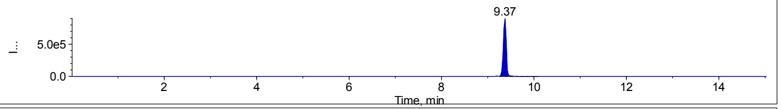

Sample Name: "ZY-26" Sample ID: "" File: "20250515-50AA.wiff"
Peak Name: "His" Mass(es): "326.150/171.010 Da"
Comment: "" Annotation: ""

Sample Index: 51
Sample Type: Unknown
Concentration: N/A
Calculated Conc: 0.564 umol/L
Acq. Date: 2025-05-16
Acq. Time: 03:40:16
Modified: Yes
Proc. Algorithm: Specify Parameters - MQ III

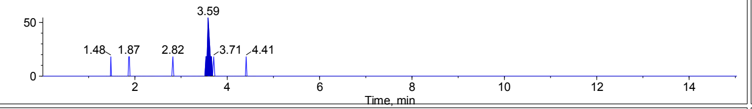

Sample Name: "ZY-26" Sample ID: "" File: "20250515-50AA.wiff"
Peak Name: "MSLAB-Nvl(IS)" Mass(es): "288.150/171.010 Da"
Comment: "" Annotation: ""

Sample Index: 51
Sample Type: Unknown
Concentration: 1.00 ng/mL
Calculated Conc: N/A
Acq. Date: 2025-05-16
Acq. Time: 03:40:16
Modified: Yes
Proc. Algorithm: Specify Parameters - MQ III

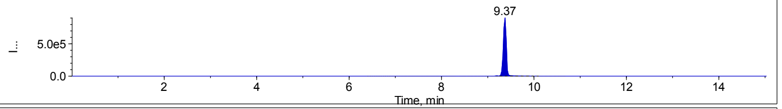

Sample Name: "ZY-26" Sample ID: "" File: "20250515-50AA.wiff"
Peak Name: "Phe" Mass(es): "336.200/171.010 Da"
Comment: "" Annotation: ""

Sample Index: 51
Sample Type: Unknown
Concentration: N/A
Calculated Conc: 0.758 umol/L
Acq. Date: 2025-05-16
Acq. Time: 03:40:16
Modified: Yes
Proc. Algorithm: Specify Parameters - MQ III

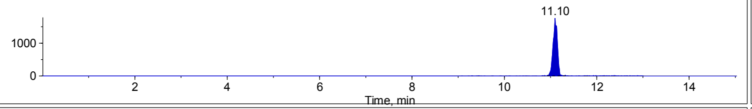

Sample Name: "ZY-26" Sample ID: "" File: "20250515-50AA.wiff"
Peak Name: "MSLAB-Nvl(IS)" Mass(es): "288.150/171.010 Da"
Comment: "" Annotation: ""

Sample Index: 51
Sample Type: Unknown
Concentration: 1.00 ng/mL
Calculated Conc: N/A
Acq. Date: 2025-05-16
Acq. Time: 03:40:16
Modified: Yes
Proc. Algorithm: Specify Parameters - MQ III

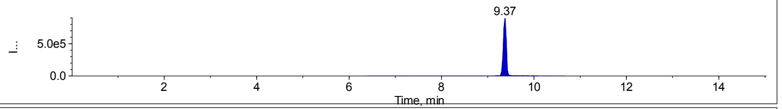

Sample Name: "ZY-26" Sample ID: "" File: "20250515-50AA.wiff"
Peak Name: "Arg" Mass(es): "345.210/171.010 Da"
Comment: "" Annotation: ""

Sample Index: 51
Sample Type: Unknown
Concentration: N/A
Calculated Conc: 2.26 umol/L
Acq. Date: 2025-05-16
Acq. Time: 03:40:16
Modified: Yes
Proc. Algorithm: Specify Parameters - MQ III

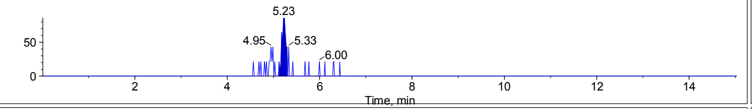

Sample Name: "ZY-26" Sample ID: "" File: "20250515-50AA.wiff"
Peak Name: "MSLAB-Nvl(IS)" Mass(es): "288.150/171.010 Da"
Comment: "" Annotation: ""

Sample Index: 51
Sample Type: Unknown
Concentration: 1.00 ng/mL
Calculated Conc: N/A
Acq. Date: 2025-05-16
Acq. Time: 03:40:16
Modified: Yes
Proc. Algorithm: Specify Parameters - MQ III

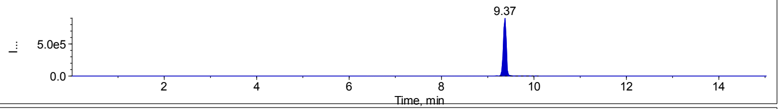

Sample Name: "ZY-26" Sample ID: "" File: "20250515-50AA.wiff"
Peak Name: "Trp" Mass(es): "375.200/171.010 Da"
Comment: "" Annotation: ""

Sample Index: 51
Sample Type: Unknown
Concentration: N/A
Calculated Conc: < 0
Acq. Date: 2025-05-16
Acq. Time: 03:40:16
Modified: Yes
Proc. Algorithm: Specify Parameters - MQ III

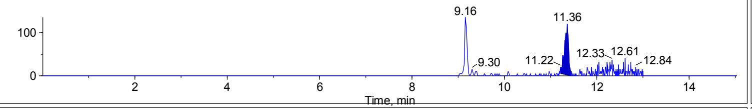

Sample Name: "ZY-26" Sample ID: "" File: "20250515-50AA.wiff"
Peak Name: "MSLAB-Nvl(IS)" Mass(es): "288.150/171.010 Da"
Comment: "" Annotation: ""

Sample Index: 51
Sample Type: Unknown
Concentration: 1.00 ng/mL
Calculated Conc: N/A
Acq. Date: 2025-05-16
Acq. Time: 03:40:16
Modified: Yes
Proc. Algorithm: Specify Parameters - MQ III

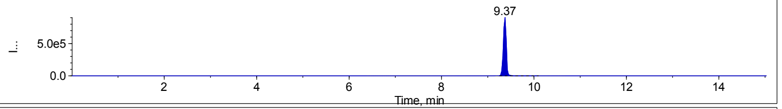

Sample Name: "ZY-26" Sample ID: "" File: "20250515-50AA.wiff"
Peak Name: "Lys" Mass(es): "487.200/171.010 Da"
Comment: "" Annotation: ""

Sample Index: 51
Sample Type: Unknown
Concentration: N/A
Calculated Conc: 2.24 umol/L
Acq. Date: 2025-05-16
Acq. Time: 03:40:16
Modified: Yes
Proc. Algorithm: Specify Parameters - MQ III

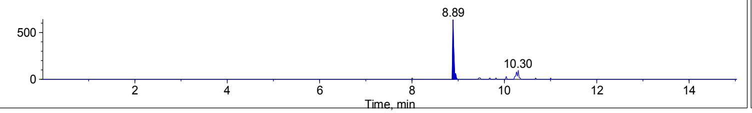

Sample Name: "ZY-26" Sample ID: "" File: "20250515-50AA.wiff"
Peak Name: "MSLAB-Nvl(IS)" Mass(es): "288.150/171.010 Da"
Comment: "" Annotation: ""

Sample Index: 51
Sample Type: Unknown
Concentration: 1.00 ng/mL
Calculated Conc: N/A
Acq. Date: 2025-05-16
Acq. Time: 03:40:16
Modified: Yes
Proc. Algorithm: Specify Parameters - MQ III

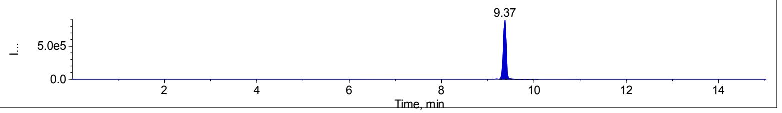

Sample Name: "ZY-26" Sample ID: "" File: "20250515-50AA.wiff"
Peak Name: "tyr" Mass(es): "352.200/171.010 Da"
Comment: "" Annotation: ""

```
Sample Index:       51
Sample Type:        Unknown
Concentration:      N/A
Calculated Conc: No Intercept
Acq. Date:          2025-05-16
Acq. Time:          03:40:16

Modified:           Yes
Proc. Algorithm: Specify Parameters - MQ III
```

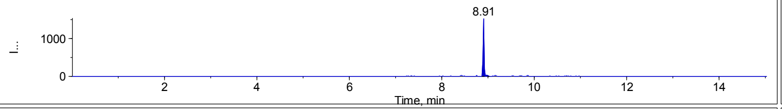

Sample Name: "ZY-26" Sample ID: "" File: "20250515-50AA.wiff"
Peak Name: "MSLAB-Nvl(IS)" Mass(es): "288.150/171.010 Da"
Comment: "" Annotation: ""

```
Sample Index:       51
Sample Type:        Unknown
Concentration:      1.00    ng/mL
Calculated Conc:    N/A
Acq. Date:          2025-05-16
Acq. Time:          03:40:16

Modified:           Yes
Proc. Algorithm: Specify Parameters - MQ III
```

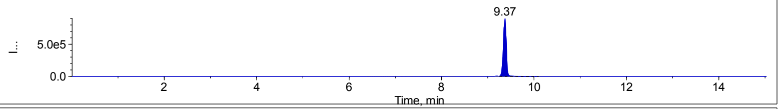

Sample Name: "ZY-26" Sample ID: "" File: "20250515-50AA.wiff"
Peak Name: "Leu" Mass(es): "302.150/171.010 Da"
Comment: "" Annotation: ""

```
Sample Index:       51
Sample Type:        Unknown
Concentration:      N/A
Calculated Conc:    1.73   umol/L
Acq. Date:          2025-05-16
Acq. Time:          03:40:16

Modified:           Yes
Proc. Algorithm: Specify Parameters - MQ III
```

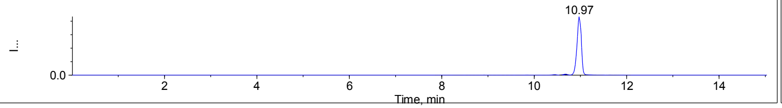

Sample Name: "ZY-26" Sample ID: "" File: "20250515-50AA.wiff"
Peak Name: "MSLAB-Nvl(IS)" Mass(es): "288.150/171.010 Da"
Comment: "" Annotation: ""

```
Sample Index:       51
Sample Type:        Unknown
Concentration:      1.00    ng/mL
Calculated Conc:    N/A
Acq. Date:          2025-05-16
Acq. Time:          03:40:16

Modified:           Yes
Proc. Algorithm: Specify Parameters - MQ III
```

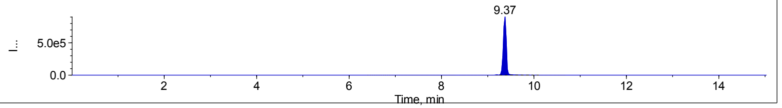

Supplement: Supplementary file 1 [file biology-15-00563-s001.zip › File S2.pdf]
